# Global, regional, and national trends in under-5 mortality between 1990 and 2019 with scenario-based projections until 2030: a systematic analysis by the UN Inter-agency Group for Child Mortality Estimation

**DOI:** 10.1016/S2214-109X(21)00515-5

**Published:** 2022-01-18

**Authors:** David Sharrow, Lucia Hug, Danzhen You, Leontine Alkema, Robert Black, Simon Cousens, Trevor Croft, Victor Gaigbe-Togbe, Patrick Gerland, Michel Guillot, Kenneth Hill, Bruno Masquelier, Colin Mathers, Jon Pedersen, Kathleen L Strong, Emi Suzuki, Jon Wakefield, Neff Walker, UN Inter-agency Group for Child Mortality Estimation and its Technical Advisory Group

**Affiliations:** a Division of Data, Analytics, Planning and Monitoring, UNICEF, New York, NY, USA; b Department of Biostatistics and Epidemiology, University of Massachusetts Amherst, Amherst, MA, USA; c Department of International Health, Johns Hopkins University, Baltimore, MD, USA; d London School of Hygiene & Tropical Medicine, London, UK; e The Demographic and Health Surveys Program, ICF, Rockville, MD, USA; f United Nations Population Division, New York, NY, USA; g Population Studies Center, University of Pennsylvania, Philadelphia, PA, USA; h French Institute for Demographic Studies, Paris, France; i Stanton-Hill Research, Moultonborough, NH, USA; j Catholic University of Louvain, Louvain-la-Neuve, Belgium; k University of Edinburgh, Edinburgh, UK; l Mikro, Oslo, Norway; m Department of Maternal, Newborn, Child and Adolescent Health, World Health Organization, Geneva, Switzerland; n The World Bank, Washington, DC, USA; o University of Washington, Seattle, WA, USA

## Abstract

**Background:**

The Sustainable Development Goals (SDGs), set in 2015 by the UN General Assembly, call for all countries to reach an under-5 mortality rate (U5MR) of at least as low as 25 deaths per 1000 livebirths and a neonatal mortality rate (NMR) of at least as low as 12 deaths per 1000 livebirths by 2030. We estimated levels and trends in under-5 mortality for 195 countries from 1990 to 2019, and conducted scenario-based projections of the U5MR and NMR from 2020 to 2030 to assess country progress in, and potential for, reaching SDG targets on child survival and the potential under-5 and neonatal deaths over the next decade.

**Methods:**

Levels and trends in under-5 mortality are based on the UN Inter-agency Group for Child Mortality Estimation (UN IGME) database on under-5 mortality, which contains around 18 000 country-year datapoints for 195 countries—nearly 10 000 of those datapoints since 1990. The database includes nationally representative mortality data from vital registration systems, sample registration systems, population censuses, and household surveys. As with previous sets of national UN IGME estimates, a Bayesian B-spline bias-reduction model (B3) that considers the systematic biases associated with the different data source types was fitted to these data to generate estimates of under-5 (age 0–4 years) mortality with uncertainty intervals for 1990–2019 for all countries. Levels and trends in the neonatal mortality rate (0–27 days) are modelled separately as the log ratio of the neonatal mortality rate to the under-5 mortality rate using a Bayesian model. Estimated mortality rates are combined with livebirths data to calculate the number of under-5 and neonatal deaths. To assess the regional and global burden of under-5 deaths in the present decade and progress towards SDG targets, we constructed several scenario-based projections of under-5 mortality from 2020 to 2030 and estimated national, regional, and global under-5 mortality trends up to 2030 for each scenario.

**Findings:**

The global U5MR decreased by 59% (90% uncertainty interval [UI] 56–61) from 93·0 (91·7–94·5) deaths per 1000 livebirths in 1990 to 37·7 (36·1–40·8) in 2019, while the annual number of global under-5 deaths declined from 12·5 (12·3–12·7) million in 1990 to 5·2 (5·0–5·6) million in 2019—a 58% (55–60) reduction. The global NMR decreased by 52% (90% UI 48–55) from 36·6 (35·6–37·8) deaths per 1000 livebirths in 1990, to 17·5 (16·6–19·0) in 2019, and the annual number of global neonatal deaths declined from 5·0 (4·9–5·2) million in 1990, to 2·4 (2·3–2·7) million in 2019, a 51% (47–54) reduction. As of 2019, 122 of 195 countries have achieved the SDG U5MR target, and 20 countries are on track to achieve the target by 2030, while 53 will need to accelerate progress to meet the target by 2030. 116 countries have reached the SDG NMR target with 16 on track, leaving 63 at risk of missing the target. If current trends continue, 48·1 million under-5 deaths are projected to occur between 2020 and 2030, almost half of them projected to occur during the neonatal period. If all countries met the SDG target on under-5 mortality, 11 million under-5 deaths could be averted between 2020 and 2030.

**Interpretation:**

As a result of effective global health initiatives, millions of child deaths have been prevented since 1990. However, the task of ending all preventable child deaths is not done and millions more deaths could be averted by meeting international targets. Geographical and economic variation demonstrate the possibility of even lower mortality rates for children under age 5 years and point to the regions and countries with highest mortality rates and in greatest need of resources and action.

**Funding:**

Bill & Melinda Gates Foundation, US Agency for International Development.

**Research in context**

**Evidence before this study**

Data from a well functioning vital registration system that covers the total population and delivers high-quality, timely data on child mortality rates at regular intervals are the gold standard for monitoring under-5 mortality. However, only about a third of the 195 countries under study here have such a system in place that can be relied upon as the sole source of information on child survival. For the remaining countries, child mortality can be estimated from household survey and census data, along with sample registration system data in a small number of countries. Adding to the overall challenge of mortality estimation, these data are not always timely and might have sampling and non-sampling errors. In accordance with its aims to produce timely, accurate, and transparent estimates of child mortality for monitoring progress towards child survival goals, the UN Inter-agency Group for Child Mortality Estimation (UN IGME) regularly compiles and assesses the quality of all publicly available data on under-5, infant, and neonatal mortality. The UN IGME under-5 mortality database used in this study contains over 18 000 country-year datapoints from more than 1600 data series across 195 countries with empirical data spanning from the 1950s (or earlier) up to 2019. The UN IGME updates its estimates of under-5, infant, and neonatal mortality annually based on these data. Other organisations also produce child mortality estimates, such as those from the Global Burden of Disease Study 2019 (GBD 2019); however these estimates produced by the UN IGME use more recent available underlying data in the estimation process and other estimates do not have the benefit of annual consultation with countries on the data and methods used to produce such estimates.

**Added value of this study**

This study estimated levels and trends in under-5 mortality and deaths at the global, regional, and country level from 1990 to 2019, provides an assessment of progress towards child survival goals, and produces scenario-based projections of under-5 mortality and deaths between 2020 and 2030 to assess the global mortality and deaths burden impact of varying levels of under-5 mortality over the next decade. Comparable estimates of under-5 mortality with uncertainty are produced using a single model that can accommodate the various data sources on child mortality and account for biases by data source type. Since the 2015 update of these estimates, the UN IGME has expanded the global database on under-5 mortality by adding or updating over 10 000 country-year datapoints. Other inputs to the estimates have also been updated such as HIV/AIDS estimates from UNAIDS and revised livebirths and population estimates from the UN Population Division's World Population Prospects. The country-level estimates are used to produce other indicators, such as rates of change, and global and regional aggregates. Compared with other child mortality estimates, these estimates have the added value of being produced annually in consultation with countries and using the most up-to-date and expansive underlying data on child mortality globally.

**Implications of all the available evidence**

The global decline in mortality over the past three decades means millions more children enjoy a better chance at survival to adolescence and adulthood today than in 1990. Even with that impressive progress, however, more than 5 million children died before reaching age 5 years in 2019 alone, primarily of preventable or treatable causes. Meanwhile, the Sustainable Development Goals (SDG), among other international initiatives, have called for an end to preventable child deaths. On current trends, more than 48 million under-5 deaths are likely to occur between 2020 and 2030. Almost 11 million of those deaths could be averted if all countries met the SDG target for under-5 mortality by 2030, but that will take intensified investments and coordinated action in countries that are at risk of missing the target, and continued, or even strengthened efforts to maintain large declines in countries that have achieved such gains.

## Introduction

More than two decades ago, the global community agreed upon numerous development goals, known as the Millennium Development Goals (MDGs), to combat disease, poverty, hunger, and environmental degradation, among other problems. These goals included a child mortality target, which called for a two-thirds reduction in the under-5 mortality rate (U5MR) between 1990 and 2015 (MDG 4).^1^ Although more than 60 countries managed to achieve MDG 4, globally the target was missed, with U5MR declining by roughly half in the 25-year MDG period.^2^ With the understanding that maintaining progress would require intensified mobilisation of resources and monitoring, child survival goals and calls to collect appropriate data to assess progress have been included in several post-MDG era global initiatives, including the UN Global Strategy for Women's, Children's and Adolescent's Health (2016–2030),^3^ which builds on the Sustainable Development Goals (SDGs). The SDGs, established in 2015 by the UN General Assembly to address unfinished business from the MDG era with new, ambitious goals for 2030, call for an end to preventable deaths of newborn babies and children younger than 5 years. The SDG child survival targets aim for all countries to achieve a U5MR of 25 or fewer deaths per 1000 livebirths by 2030, (SDG target 3.2.1) and a neonatal mortality rate (NMR) of 12 or fewer deaths per 1000 livebirths by 2030 (SDG target 3.2.2).^4^ Roughly one-third of the way into the SDG era, it is time to assess country progress thus far and look ahead over the new decade to evaluate where greater acceleration is needed to achieve the SDG targets on under-5 mortality.

In the absence of accurate and continually collected vital registration data—the preferred source of data for monitoring vital events—evidence-based estimation of child mortality remains indispensable for tracking progress towards child survival goals, developing national health strategies, and planning policies and interventions around child health.

The UN Inter-agency Group for Child Mortality Estimation (UN IGME), led by UNICEF and including members from WHO, the World Bank Group, and the UN Population Division, was established in 2004, to advance the work on monitoring progress towards the achievement of child survival goals and to augment country capacity to collect high-quality data on, and produce timely estimates of, child mortality. The UN IGME technical advisory group, comprised of academic scholars and independent experts in demography and biostatistics, provides guidance on estimation methods, technical issues, and strategies for data analysis and data quality assessment. UN IGME updates its estimates of neonatal, infant, and under-5 mortality annually after reviewing newly available data and assessing their quality. UN IGME estimates are developed in consultation with countries. The annual country consultation process gives each country's Ministry of Health, National Statistics Office, or relevant agency the opportunity to review and provide feedback on all data inputs, the estimation method, and the draft estimates of child mortality.

In this Article, we estimate levels and trends in under-5 mortality at the global, regional, and country level. We also provide an assessment of current progress towards the SDG targets. To derive child mortality estimates for all countries, we use a single model that can accommodate the varying data sources on child mortality, account for biases by data source type, and produce comparable trend lines of U5MR over time with uncertainty up to a common reference year (2019). We also present projections of the U5MR and under-5 deaths from 2020 to 2030, under several scenarios, to demonstrate potential impact of varying rates of change in the coming years.

## Methods

### Estimation process overview

The estimation process begins with compiling and assessing the quality of all available nationally representative data relevant to the estimation of child mortality. Under-5 mortality data are harmonised through recalculation, adding standard errors and calendar-year reference dates. A statistical model is fit to these data to generate a smooth trend curve considering the possibly disparate estimates from different data sources for a country. During each annual estimation cycle, draft estimates are prepared as above and shared with countries for consultation. The statistical model is fit again after country consultation incorporating any newly available data or relevant feedback to produce the final estimates.

### Under-5 mortality database

The underlying data consist of nationally representative data of under-5 mortality derived from several sources, including vital registration systems, population censuses, and household surveys. Demographic surveillance sites and facility (eg, hospital) data are excluded as they are not nationally representative. A civil registration system that records births and deaths on a continuous, complete, and timely basis is the preferred source of data on under-5 mortality; however, these systems are not well developed in many low-income and middle-income countries. In the absence of high-quality vital registration systems, household surveys, such as the UNICEF-supported Multiple Indicator Cluster Surveys, the US Agency for International Development-supported Demographic and Health Surveys (DHS), and periodic population censuses, have become the primary sources of data on mortality among children younger than 5 years. Estimates from these sources are derived from the birth history module, which can provide either direct estimates that take into account the age at death collected in the full birth history, or indirect estimates based on the proportions of children who have died collected in the summary birth history. These sources can suffer from sampling and non-sampling errors (eg, recall errors, misreporting of age, or under-reporting of deaths). 89 countries, containing 45% of the 2019 under-5 population, have no vital registration or sample vital registration data since 2000 included in the estimation model and rely solely on household survey and census data to estimate child mortality (58 countries have no vital registration data since 2000, and 31 countries have vital registration data that are not included in the estimation model due to incompleteness of death reporting). Of these 89 countries, 86 are low-income or middle-income countries.

In this update (the last one was in September, 2019), a substantial amount of newly available data has been added to the underlying database for under-5 mortality. Data from 44 new surveys or censuses were added for 39 countries and data from vital registration systems or sample vital registration systems were updated for 135 countries. In total, more than 6400 country-year datapoints from 179 series were added or updated. The U5MR database, as of Aug 25, 2020, contains more than 18 000 country-year datapoints from more than 1600 series across 195 countries. The full set of empirical data used to model U5MR and NMR in this analysis is publicly available from the UN IGME website.

### Modelling under-5 mortality

Estimation of U5MR was done using the Bayesian B-splines bias-adjusted model, referred to as the B3 model.^5^ The B3 model has been used to produce previous sets of UN IGME child mortality estimates since 2014.^2^, ^6^, ^7^, ^8^, ^9^, ^10^ In the B3 model, the logarithm of U5MR is estimated with a flexible spline regression model. The spline regression model is fitted to all U5MR observations in the country. An observed value for U5MR is considered to be the true value for this parameter multiplied by an error multiplier (ie, observed U5MR = true U5MR × error multiplier or on the logarithmic scale log[observed U5MR]=log[true U5MR] + log[error multiplier]). The error multiplier refers to the relative difference between an observation and the true value, with the error multiplier equal to 1 (or log[error multiplier] equal to zero) if there is no error.

While estimating the true U5MR, properties of the errors that provide information about the quality of the observation—ie, the extent of error that we expect—are considered and summarised in the so-called data model. These properties include the standard error of the observation, its source type (eg, DHS *vs* census), and whether the observation is part of a data series from a specific survey programme, as well as how far the data series diverges from other series with overlapping observation periods. When estimating the trend in U5MR, the data model adjusts for predictable errors in observations, including the average systematic biases associated with different types of data sources, using information on data quality for different source types from all countries.

The NMR is defined as the probability of dying between birth and exact age 28 days, expressed per 1000 livebirths. We used a similar Bayesian estimation method to estimate levels and trends in NMR that captures data driven trends in countries and over time for all countries.^11^ For NMR, we modelled the ratio R(c,t), which refers to the ratio of NMR to the difference between U5MR and NMR in country c and year t. For each country-year, the ratio is given by R(c,t)=W(c,t) × P(c,t), where W(c,t) refers to the expected ratio for that country-year based on a global relation between observed ratios and the UN IGME-estimated U5MR for that country-year and P(c,t), modelled with a B-splines regression model, is a country multiplier representing country-specific trends in the ratio over time that differ from the expected level. Modelling the ratio instead of NMR directly allows us to use the global relationship between NMR and U5MR (ie, as the level of U5MR decreases, the proportion of the under-5 deaths occurring in the neonatal period increases) to estimate NMR in countries with sparse or no data on NMR and restricts the NMR estimate to be lower than the U5MR for any given year. Moreover, the NMR database contains fewer datapoints than the U5MR database since NMR estimates cannot easily be derived using indirect methods with summary birth histories. Further details on modelling NMR can be found in the appendix (pp 10–11).

Given the high uncertainty associated with child mortality estimates, we report 90% uncertainty intervals (UIs)—ie, there is a 90% chance the interval contains the true value. Although there is a greater chance of excluding the true value with this narrower interval relative to the more conventional 95% CIs, with such a highly uncertain indicator, the wider intervals can lose their utility to meaningfully summarise the range of possible outcomes.

If the most recent underlying empirical data series for a country ends before the final year of the period for which the estimates are reported, in this instance mid-2019, we extrapolate the estimates to the common end year based on a combination of the past trend in a country and the global trend. The average extrapolation period in the 2020 round of estimation was 4·6 years for U5MR, with half of the countries having datapoints within the past 3·0 years (appendix p 9). For more than a third of all countries, the latest available child mortality datapoint was more than 5 years old.

Estimates are adjusted for conflict and other major environmental disasters (eg, floods, cyclones, and earthquakes) and for the impact of HIV/AIDS in a select group of countries (appendix pp 9–11).

A birth-week cohort method is used to calculate the absolute number of deaths among neonates and children under age 5 years. First, for each country, each annual birth cohort is divided into 52 equal birth-week cohorts. Then each birth-week cohort is exposed throughout the first 5 years of life to the appropriate calendar-year-specific and age-specific mortality rates depending on cohort age. All deaths from birth-week cohorts occurring as a result of exposure to the mortality rate for a given calendar year are allocated to that year and are summed by age group at death to get the total number of deaths for a given year and age group. Under-5 deaths in each calendar year are calculated by summing up all the deaths under age 5 years across all age group cohorts in that year. The annual number of livebirths in each country is derived from the World Population Prospects 2019 revision.^12^ The 90% UIs for the deaths are based on the uncertainty in the mortality estimates and do not account for other sources of uncertainty in its inputs such as the annual number of livebirths.

The B3 model code in R is available from the UN IGME upon request.

### Scenarios

We project U5MR and under-5 deaths at the country, regional, and global level under four scenarios: (1) constant 2019; (2) current trends; (3) achieving SDG target; and (4) achieving high-income country (HIC) U5MR.

In the constant 2019 scenario, the U5MR for each country was held constant between 2020 and 2030 at the 2019 level for that country.

In the current trends scenario, for each country, the annual rate of reduction (ARR) for the period 2010–19 was used to project the U5MR at the country level from 2020 to 2030. The ARR was calculated as ln(U5MR_t2_/U5MR_t1_)/(t1 – t2), where t1 and t2 refer to different years with t1 smaller than t2. If a country had a negative ARR (ie, an increase in mortality between 2010 and 2019), the rate was held constant at the 2019 level. If a country reached the current lowest observed U5MR (ie, 1·7 deaths per 1000 livebirths) during the projection period, the mortality rate was held constant at that lowest observed level for the remainder of the projection period because we assumed that there will not be zero mortality but cannot be certain as to what the lowest possible rate will be.

In the achieving SDG target scenario, each country was projected to reach the SDG target by 2030. For each country that has not already reached the SDG target, U5MR in 2030 is equal to 25 deaths per 1000 livebirths and mortality rates between 2020 and 2030 are projected based on the required ARR to achieve the target, ie, the ARR calculated for the country's level of mortality in 2019 and the SDG target (appendix p 82). For countries that have already achieved the target or are on track to reach the target by 2030, the projections from the current trends scenario were used.

In the achieving HIC U5MR scenario, each country is set to achieve a U5MR in 2030 equal to the average U5MR of HICs in 2019 (5·0 deaths per 1000 livebirths). For countries that have already achieved the HIC U5MR for 2019 or are on track to achieve that rate by 2030, the projections from the current trends scenario were used.

We also projected the NMR and neonatal deaths to assess the age-specificity of the under-5 deaths burden under the four scenarios. For the current trends scenario, we used the country-level ARRs for NMR 2010–19 and the lowest observed NMR, 0·85 deaths per 1000 livebirths. We also constrained the ratio of NMR to U5MR to ensure that it did not exceed the highest observed ratio in a country with good vital registration data (Denmark, 0·79) during the projection period. For the achieving SDG target scenarios, we used the SDG NMR target of 12·0 deaths per 1000 livebirths. For the achieving HIC NMR scenario, we used the HIC NMR for 2019 of 2·9 deaths per 1000 livebirths.

The number of projected deaths was calculated with the birth-week cohort method using the projected rates in each of the scenarios and the projected annual number of livebirths between 2020 and 2030 from the World Population Prospects 2019 (the median variant projection).^13^ The annual number of livebirths is the same in each scenario, thus the number of deaths that could be averted under different mortality levels and paces of decline can be assessed. Changes in mortality can impact the number of livebirths through behavioural changes, but these effects were not considered to isolate the effect of changing mortality levels over the projection period. These scenarios also do not consider potential changes to intervention coverage or other factors that might affect the level of child mortality or its pace of decline. Crisis mortality was removed from the estimates for the calculation of the ARR between 2010 and 2019. Regional and global aggregates were calculated based on the projected country level estimates. Furthermore, the scenario projections do not include any projected impact of the COVID-19 pandemic on child survival for 2020 or beyond because of a lack of information to guide such adjustments.

Regional and income classifications are shown in the appendix (pp 13–15).

### Role of the funding source

The funders of the study had no role in study design, data collection, data analysis, data interpretation, or writing of the report.

## Results

The global U5MR in 2019 was 37·7 (90% UI 36·1–40·8) deaths per 1000 livebirths, and the estimated number of under-5 deaths was 5·2 million (5·0–5·6 million) (table 1), with 257·2 million (252·2–263·0 million) under-5 deaths occurring between 1990 and 2019. Regionally, sub-Saharan Africa continues to have the highest rate of under-5 mortality in the world—estimated at 75·8 (70·2–85·9) deaths per 1000 livebirths in 2019 (table 1), equivalent to one in 13 children dying before reaching age 5 years. Within sub-Saharan Africa, the highest regional U5MR was west and central Africa at 94·7 (83·9–111·3) deaths per 1000 livebirths in 2019 (table 1, appendix p 81)—19-times higher than the average 2019 U5MR in HICs. Two regions accounted for the vast majority of under-5 deaths in 2019: sub-Saharan Africa with 2·8 (2·6–3·1) million or 55% (53–57) of global under-5 deaths, and south Asia with 1·4 (1·3–1·5) million or 26% (26–27) of the total (table 1). Although they accounted for more than 80% of the global under-5 deaths in 2019, these two regions only accounted for 51% of the global under-5 population.

**Table 1:** Global and regional under-5 mortality rates and number of under-5 deaths

		**Under-5 mortality rate (deaths per 1000 livebirths; 90% UI)**					**Number of under-5 deaths (thousands; 90% UI)**					
		1990	2000	2010	2019	Decrease 1990–2019 (%)	1990	2000	2010	2019	Decrease 1990–2019 (%)	Share of global deaths 2019 (%)
World		93·0 (91·7–94·5)	75·8 (74·8–76·9)	51·2 (50·3–52·5)	37·7 (36·1–40·8)	59% (56–61)	12494 (12 320–12 698)	9749 (9624–9897)	6950 (6820–7112)	5189 (4970–5600)	58% (55 to 60)	100% (100–100)
By region												
	Sub-Saharan Africa	178·5 (175·0–182·5)	150·5 (147·7–153·9)	101·2 (98·0–105·2)	75·8 (70·2–85·9)	57% (52–61)	3826 (3752–3910)	3987 (3911–4075)	3303 (3200–3432)	2844 (2636–3218)	26% (16 to 31)	55% (53–57)
	West and central Africa	196·2 (190·1–202·7)	168·0 (163·1–173·5)	120·6 (115·1–126·9)	94·7 (83·9–111·3)	51% (43–57)	2020 (1957–2086)	2190 (2126–2261)	1989 (1897–2091)	1836 (1627–2155)	9% (−7 to 20)	35% (33–38)
	Eastern and southern Africa	161·9 (158·1–166·3)	133·4 (130·3–137·3)	81·2 (77·9–85·9)	55·4 (51·0–64·2)	65% (60–69)	1806 (1765–1854)	1796 (1755–1848)	1314 (1261–1388)	1009 (929–1166)	44% (36 to 49)	19% (19–21)
	Middle East and north Africa	64·9 (63·3–66·7)	42·1 (40·9–43·5)	27·1 (25·8–28·5)	21·8 (19·0–26·1)	66% (60–71)	545 (532–561)	324 (315–335)	246 (235–260)	219 (191–261)	60% (52 to 65)	4% (4–5)
	South Asia	129·7 (126·5–133·1)	93·4 (90·8–96·1)	61·9 (59·9–64·2)	40·2 (37·2–43·4)	69% (66–71)	4748 (4632–4872)	3548 (3449–3651)	2276 (2199–2359)	1406 (1300–1518)	70% (68 to 73)	27% (26–27)
	East Asia and Pacific	56·7 (54·0–60·0)	39·4 (38·2–40·9)	21·9 (21·1–22·7)	14·3 (13·2–15·8)	75% (72–77)	2301 (2190–2432)	1257 (1216–1303)	695 (671–720)	435 (404–480)	81% (79 to 83)	8% (8–9)
	Latin America and the Caribbean	54·6 (53·1–56·3)	33·1 (32·2–34·1)	24·7 (24·1–25·6)	16·3 (15·4–17·6)	70% (68–72)	641 (623–661)	381 (371–392)	265 (258–275)	169 (160–183)	74% (71 to 75)	3% (3–3)
	Europe and central Asia	30·7 (29·9–31·6)	21·4 (20·7–22·1)	12·0 (11·6–12·5)	8·1 (7·6–8·7)	74% (72–75)	386 (376–397)	217 (211–225)	132 (128–138)	88 (83–95)	77% (75 to 78)	2% (2–2)
	Eastern Europe and central Asia	46·3 (44·9–47·9)	35·4 (34·2–36·9)	18·5 (17·7–19·4)	11·5 (10·7–12·7)	75% (73–77)	328 (318–339)	187 (180–195)	110 (105–115)	70 (65–77)	79% (77 to 80)	1% (1–1)
	Western Europe	10·5 (10·4–10·6)	6·2 (6·2–6·2)	4·5 (4·4–4·5)	3·8 (3·8–3·9)	64% (63–64)	58 (57–58)	30 (30–31)	23 (23–23)	19 (18–19)	68% (67 to 68)	0
	North America	11·0 (10·8–11·2)	8·3 (8·1–8·4)	7·2 (7·1–7·3)	6·3 (6·0–6·7)	42% (39–46)	47 (46–48)	35 (35–36)	32 (32–33)	27 (26–29)	42% (39 to 46)	1% (1–1)
By income group												
	Low income	182·5 (178·7–187·0)	143·6 (140·5–147·4)	95·6 (92·1–100·0)	67·6 (62·3–77·7)	62% (57–66)	2359 (2311–2416)	2326 (2276–2387)	1872 (1804–1956)	1509 (1391–1730)	36% (27 to 41)	29% (28–31)
	Lower-middle income	124·3 (122·0–126·8)	97·2 (95·3–99·3)	66·8 (64·9–68·9)	48·9 (45·5–54·0)	60% (57–63)	7151 (7021–7291)	5804 (5689–5927)	4153 (4041–4280)	3074 (2866–3391)	57% (53 to 60)	59% (58–61)
	Upper-middle income	54·7 (52·5–57·2)	36·9 (35·9–38·0)	20·5 (20·0–21·1)	13·3 (12·7–14·3)	75% (73–77)	2804 (2692–2935)	1514 (1474–1560)	844 (823–869)	542 (517–582)	81% (79 to 82)	10% (10–10)
	High income	13·0 (12·7–13·4)	8·2 (8·0–8·3)	6·1 (6·0–6·3)	5·0 (4·9–5·2)	61% (59–63)	179 (175–185)	105 (103–107)	81 (80–83)	63 (61–66)	65% (63 to 66)	1% (1–1)

Regional and income classifications can be found in the appendix pp 13–15. UI=uncertainty interval.

**Figure 1:**
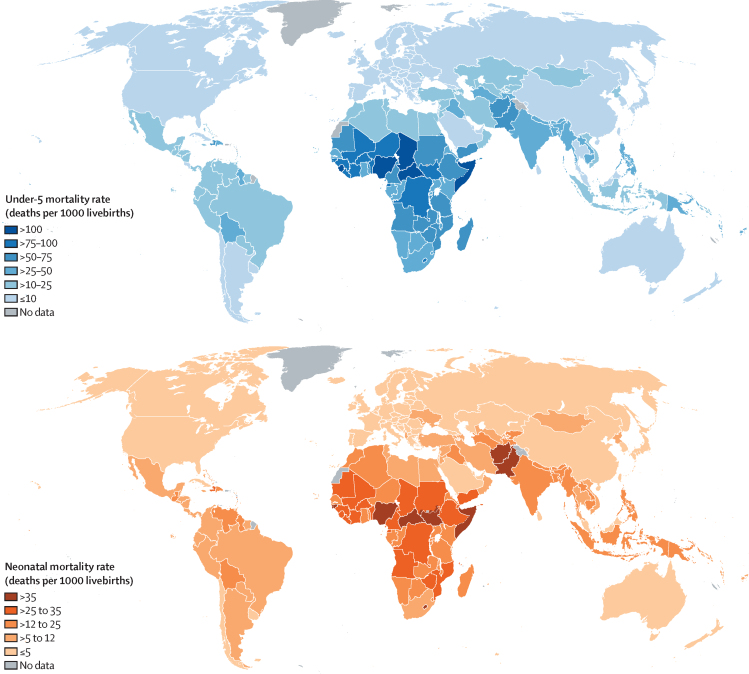
Under-5 mortality rate (top panel) and neonatal mortality rate (bottom panel) in 2019, by country

At the country level, U5MR in 2019 ranged from 117·2 (90% UI 92·2 -152·5) deaths per 1000 livebirths in Nigeria to 2·1 (1·8–2·4) deaths per 1000 livebirths in Slovenia (among countries with at least 10 000 livebirths in 2019; figure 1). Just five countries, all in sub-Saharan Africa, had a U5MR in 2019 greater than 100 deaths per 1000 livebirths based on point estimates: Central African Republic, Chad, Nigeria, Sierra Leone, and Somalia; in 1990, more than 50 countries had rates greater than 100. The under-5 deaths were also concentrated in a small number of countries—49% of all under-5 deaths in 2019 occurred in just five countries: Nigeria, India, Pakistan, Democratic Republic of the Congo, and Ethiopia; Nigeria and India alone accounted for almost a third of all under-5 deaths (appendix pp 16–37).

**Figure 2:**
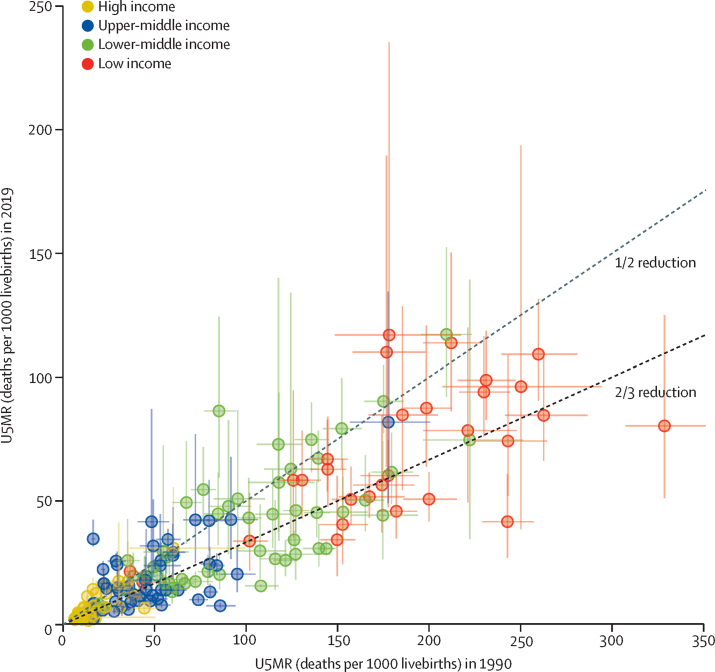
U5MR in 1990 and 2019 Estimated rates are shown with 90% UI (vertical lines are 90% UI in 2019, and horizontal lines 90% UI in 1990), by country and income group (income group classification can be found in the appendix, pp 13–15). U5MR=under-5 mortality rate. UI=uncertainty interval.

The global U5MR decreased by 59% (90% UI 56–61) from 93·0 (91·7–94·5) deaths per 1000 livebirths in 1990 to 37·7 (36·1–40·8) deaths per 1000 livebirths in 2019, and the global number of under-5 deaths declined by 58% (55–60) from 12·5 (12·3–12·7) million in 1990 to 5·2 (5·0–5·6) million in 2019 (table 1). All regions, except North America, had U5MR decline by at least 50% since 1990. The highest decreases occurred in east Asia and Pacific (75% [72–77]) and eastern Europe and Central Asia (75% [73–77]). West and central Africa, where U5MR fell by 51% (43–57), had the second smallest decrease in U5MR since 1990, after North America. At the country level, 85 (43–134) countries, including 34 (12–58) low-income and lower-middle-income countries, decreased their U5MR by at least two-thirds since 1990 (figure 2).

**Figure 3:**
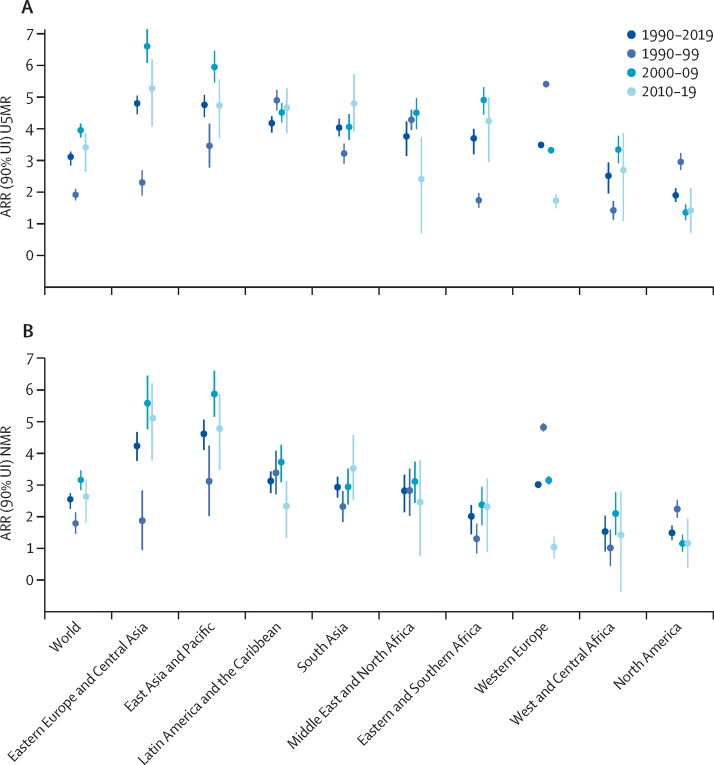
ARR in U5MR (A) and NMR (B) Estimated data are shown with 90% UI, worldwide and by region. The formula for ARR gives an instantaneous rate of reduction, which we refer to as annual given the yearly nature of the estimates. ARR=annual rate of reduction. NMR=neonatal mortality rate. U5MR=under-5 mortality rate. UI=uncertainty interval.

Globally, the pace of decline in U5MR over the 30-year period of 1990–2019 was 3·1% (90% UI 2·8–3·3) annually (figure 3). All regions made progress in reducing U5MR since 1990, with ARRs for 1990–2019 by region ranging from 1·9% (1·7–2·1) in North America up to 4·8% (4·5–5·1) in eastern Europe and central Asia and 4·8% (4·4–5·1) in east Asia and Pacific.

The pace of decline in global U5MR quickened in the 2000s compared with the 1990s, then had a possible slowdown in the most recent decade, based on point estimates. The ARR in under-5 mortality doubled from 1·9% (1·7–2·1) in 1990–99 to 4·0% (3·7–4·2) in 2000–09, before possibly slowing to 3·4% (2·6–3·9) in 2010–19 (figure 3). Notably, for the world and nearly all regions, the UIs for ARR 1990–99 and ARR 2000–09 do not overlap, whereas the UI for ARR 2010–19 overlaps with that from the preceding decade due to wider uncertainty for the most recent period reflecting fewer data for more recent years. Regionally, sub-Saharan Africa (west and central Africa and eastern and southern Africa), eastern Europe and central Asia, east Asia and Pacific, and the Middle East and north Africa followed the global pattern in U5MR decline, with the fastest pace of decline between 2000 and 2009, followed by a possible slowdown for 2010–19. In comparison, in North America and western Europe, with relatively low mortality rates at the start of this period, in 1990, the pace of decline slowed after 2000. South Asia was the only region to see acceleration in the decline of U5MR in each decade starting in 1990.

In 2019, global NMR was 17·5 (90% UI 16·6–19·0) deaths per 1000 livebirths, down by just over half from 36·6 (35·6–37·8) deaths in 1990 (table 2 and appendix p 81). Neonatal deaths also dropped by a little over half from 5·0 (4·9–5·2) million in 1990, to 2·4 (2·3–2·7) million in 2019. Globally, as the level of under-5 mortality has declined, a greater share of under-5 mortality is concentrated in the neonatal period—47% (45–49) of all under-5 deaths occurred during the neonatal period in 2019, an increase from 40% (39–41) in 1990—ie, the decline in mortality among children aged 1–59 months has outpaced the decline in mortality among neonates. While the decline in global NMR was not as quick as that for U5MR, with just a 2·5% (2·3–2·7) annual reduction in NMR between 1990 and 2019, the global pattern in decline in NMR followed that of U5MR, with a quickening in the pace of decline in the 2000s and possible slowdown in the most recent decade (figure 3).

**Table 2:** Global and regional neonatal mortality rates and numbers of neonatal deaths

		**Neonatal mortality rate (deaths per 1000 livebirths; 90% UI)**					**Number of neonatal deaths (thousands; 90% UI)**					
		1990	2000	2010	2019	Decrease 1990–2019 (%)	1990	2000	2010	2019	Decrease 1990–2019 (%)	Share of global deaths 2019 (%)
World		36·6 (35·6–37·8)	30·3 (29·6–31·2)	22·2 (21·6–22·8)	17·5 (16·6–19·0)	52% (48–55)	5014 (4875–5175)	3996 (3900–4105)	3062 (2984–3156)	2440 (2322–2651)	51% (47–54)	100% (100–100)
By region												
	Sub-Saharan Africa	45·3 (43·5–47·2)	40·1 (38·6–41·8)	32·2 (30·9–34·0)	27·5 (25·2–31·7)	39% (30–45)	1016 (977–1060)	1111 (1069–1159)	1091 (1046–1151)	1059 (970–1222)	−4% (−20 to 5)	43% (42–46)
	West and central Africa	48·0 (45·4–50·9)	43·0 (40·6–45·5)	35·1 (32·8–37·6)	30·9 (26·9–36·9)	36% (23–44)	519 (490–550)	589 (556–624)	601 (564–645)	616 (536–737)	−19% (−42 to −3)	25% (23–28)
	Eastern and southern Africa	42·7 (40·9–44·8)	37·2 (35·7–39·0)	29·4 (28·0–31·4)	23·8 (21·7–28·0)	44% (34–50)	497 (477–522)	522 (501–547)	490 (466–523)	443 (404–521)	11% (−5 to 20)	18% (17–20)
	Middle East and North Africa	27·8 (26·0–29·4)	20·9 (20·1–21·9)	15·3 (14·5–16·3)	12·3 (10·7–14·7)	56% (46–62)	235 (220–249)	162 (155–170)	143 (136–152)	123 (108–148)	47% (36–55)	5% (5–6)
	South Asia	58·7 (56·4–61·3)	46·3 (44·4–48·3)	34·5 (32·9–36·0)	25·1 (23·0–27·3)	57% (53–61)	2193 (2105–2287)	1782 (1710–1856)	1265 (1209–1322)	882 (810–960)	60% (56–63)	36% (35–36)
	East Asia and Pacific	27·5 (25·4–30·0)	19·9 (18·9–21·0)	11·1 (10·5–11·6)	7·2 (6·5–8·1)	74% (70–77)	1106 (1019–1204)	633 (600–669)	354 (337–372)	218 (198–246)	80% (77–83)	9% (9–9)
	Latin America and the Caribbean	22·5 (21·3–23·6)	16·0 (15·1–16·8)	11·2 (10·9–11·6)	9·1 (8·4–10·0)	60% (55–63)	266 (253–280)	184 (174–193)	120 (117–124)	94 (88–104)	65% (60–67)	4% (4–4)
	Europe and central Asia	14·0 (13·3–14·8)	10·3 (9·8–10·8)	6·3 (5·9–6·7)	4·4 (4·0–4·8)	69% (65–72)	174 (166–185)	104 (99–110)	71 (66–75)	47 (44–52)	73% (70–75)	2% (2–2)
	Eastern Europe and central Asia	20·5 (19·3–22·0)	16·6 (15·7–17·7)	9·5 (8·8–10·3)	6·0 (5·4–6·8)	71% (66–74)	144 (136–154)	87 (83–93)	58 (53–62)	36 (33–41)	75% (71–78)	1% (1–2)
	Western Europe	5·6 (5·5–5·7)	3·5 (3·4–3·5)	2·5 (2·5–2·6)	2·3 (2·2–2·4)	58% (57–60)	30 (30–31)	17 (17–17)	13 (13–13)	11 (11–12)	63% (62–64)	0
	North America	5·6 (5·5–5·8)	4·6 (4·5–4·7)	4·1 (4·0–4·2)	3·7 (3·4–3·9)	35% (30–39)	24 (24–25)	20 (19–20)	18 (18–19)	16 (15–17)	35% (31–40)	1% (1–1)
By income group												
	Low-income	48·2 (46·3–50·5)	41·2 (39·6–43·1)	32·6 (31·1–34·6)	26·6 (23·9–31·4)	45% (35–51)	656 (630–687)	699 (670–730)	659 (628–700)	609 (547–718)	7% (−9 to 17)	25% (24–27)
	Lower-middle-income	49·8 (48·1–51·5)	40·6 (39·3–41·9)	30·5 (29·5–31·7)	23·8 (22·2–26·0)	52% (48–56)	2929 (2834–3033)	2474 (2395–2557)	1926 (1860–1998)	1516 (1418–1658)	48% (43–52)	62% (61–63)
	Upper-middle-income	26·4 (24·6–28·4)	18·7 (17·9–19·6)	10·4 (10·0–10·8)	6·9 (6·5–7·6)	74% (70–76)	1338 (1249–1439)	767 (733–804)	432 (416–451)	280 (263–307)	79% (76–81)	11% (11–12)
	High-income	6·6 (6·3–6·9)	4·4 (4·2–4·6)	3·3 (3·2–3·5)	2·9 (2·8–3·1)	56% (53–59)	91 (87–96)	56 (54–59)	44 (43–46)	36 (35–38)	60% (58–62)	1% (1–1)

Regional and income classifications can be found in the appendix pp 13–15. UI=uncertainty interval.

As with U5MR, neonatal mortality is regionally concentrated, with sub-Saharan Africa having the highest NMR in the world in 2019, at 27·5 deaths (25·2–31·7) per 1000 livebirths, a 39% reduction since 1990 (table 2, figure 1). Despite that decline, neonatal deaths have stagnated at around 1 million per year in sub-Saharan Africa, about 43% (42–46) of global under-5 deaths in 2019, due to the increase in births. Another 36% (35–36) of global under-5 deaths occurred in south Asia in 2019 (table 2).

Of 195 countries analysed, 122 have already met the SDG target for under-5 mortality, and 20 countries are expected to do so by 2030, if current trends continue. Of the remaining 53 countries, almost three-quarters of which are in sub-Saharan Africa, 23 will need to at least triple and 12 more will need to at least double their current rate of progress to meet the U5MR SDG target on time (appendix p 82). Based on current trends, 25 countries would not achieve the U5MR 2030 target until after 2050, and eight would not achieve the target until after 2099. Even more countries are at risk of missing the neonatal mortality target—63 countries need to accelerate progress if they are to meet the NMR SDG target by 2030. Among these 63 countries, 38 need to more than triple and another 14 need to at least double their current ARR for neonatal mortality to meet the SDG target on time (appendix p 82).

Scenario-based projections of the global U5MR and number of under-5 deaths between 2020 and 2030 are shown in table 3 and the appendix (p 83). In the current trends scenario, the global U5MR would be 27·6 deaths per 1000 livebirths and about 3·8 million under-5 deaths would occur in 2030. In this scenario, 48·1 million under-5 deaths would occur in the 2020–30 period, with 49% of those deaths (23·6 million) occurring in the neonatal period. More than half (59%) of these deaths would occur in sub-Saharan Africa (28·4 million) and 24% in south Asia (11·6 million).

**Table 3:** Projected global under-5 mortality rate and neonatal mortality rate with projected deaths under different scenarios

	**Under-5 mortality**					**Neonatal mortality**				
	Projected under-5 mortality rate (deaths per 1000 livebirths)		Projected under-5 deaths (millions)		Projected total under-5 deaths (millions)	Projected neonatal mortality rate (deaths per 1000 livebirths)		Projected neonatal deaths (millions)		Projected total neonatal deaths (millions)
	2025	2030	2025	2030	2020–30	2025	2030	2025	2030	2020–30
Constant 2019	39·4	40·7	5·4	5·6	59·4	18·0	18·4	2·5	2·6	27·5
Current trends	31·7	27·6	4·3	3·8	48·1	15·4	13·9	2·1	1·9	23·6
Achieve SDG target	23·9	16·7	3·3	2·3	37·3	12·1	8·9	1·7	1·2	18·8
Achieve HIC average	11·5	4·7	1·6	0·7	21·1	6·1	2·7	0·8	0·4	10·8

HIC=high-income country. SDG=Sustainable Development Goal.

Under the achieving SDG target scenario, the number of under-5 deaths in the 2020–30 period would be 37·3 million—almost 11 million fewer deaths compared with the current trends scenario. In this scenario, global U5MR in 2030 would be 16·7 deaths per 1000 livebirths, with 2·3 million deaths.

Even more deaths could be averted (more than 27 million from 2020 to 2030) compared with the current trends scenario if all countries achieved the average under-5 mortality level in HICs by 2030. Global U5MR would be lowest in 2030 under this scenario—4·7 deaths per 1000 livebirths—with 700 000 under-5 deaths and just under 400 000 neonatal deaths in 2030.

In the constant 2019 scenario, where decline has stagnated, 5·6 million under-5 deaths would occur in 2030 alone (nearly half a million more deaths than in 2019), and more than 59 million under-5 deaths would occur in the 2020–30 period, with 46% of those deaths occurring in the neonatal period (27·5 million).

## Discussion

Undeniably, the intensive investments and targeted actions of the global health community to combat the main causes of child mortality with high-impact interventions, such as immunisations, access to nutrition and micronutrients, skilled attendants around birth and postnatal care, and expanded access to safe water, sanitation, and hygiene, have paid off with substantial reductions in under-5 mortality since 1990. However, a large number of deaths remains, and children face widespread geographical and income disparities in their chances of survival.

The global U5MR declined by 59% since 1990, and all regions had under-5 mortality decline since 1990. These declines were aided by an acceleration in the pace of decline globally and in most regions in the 2000s compared with the 1990s, but the pace of decline might have slowed slightly in the most recent decade. This global pattern was found in relatively high mortality regions such as sub-Saharan Africa, where, in a subset of countries, the higher ARR for 2000–09 partially reflects rapid decline in HIV/AIDS-related child mortality. Nevertheless, the point estimate ARRs for 2010–19 remain above those for the 1990s in those regions.

Despite this progress, about 5·2 million children died before reaching age 5 years in 2019 alone—most of preventable or treatable causes—and more than 257 million under-5 deaths occurred in the past three decades. Based on current trends (ie, no acceleration in under-5 mortality decline), 48·1 million under-5 deaths are projected to occur between 2020 and 2030. That number would be reduced by almost 11 million deaths if all countries met the SDG target on under-5 mortality. However, a substantial number of under-5 deaths—5·6 million—are projected to occur in that time period among countries that have already achieved the SDG target, if their current trends continue. If decline in under-5 mortality was to stagnate and mortality levels remained as they were in 2019, there would be nearly half a million more deaths in 2030 than in 2019 as a result of growing under-5 populations and the shift of population towards high-mortality regions, such as west and central Africa, over this decade.

Further reducing global under-5 mortality will require overcoming the widely different chances of survival children face across countries, regions, and income groups. Close to 75% of countries at risk of missing the SDG target on under-5 mortality are in sub-Saharan Africa, and more than 80% of under-5 deaths in 2019 occurred in just two regions: sub-Saharan Africa and south Asia. If current trends continue, 28·4 million under-5 deaths are projected in sub-Saharan Africa, 59% of the global total under-5 deaths over that time (sub-Saharan Africa accounts for about 30% of projected livebirths 2020–30). The region is projected to see 462 million births from 2020 to 2030, and the under-5 population is expected to increase to roughly 199 million by 2030, from 168 million in 2019.^12^ Without a quickening in the pace of mortality decline, the increase in births could lead to stagnation or increase in the number of deaths. Moreover, children in low-income and lower-middle-income countries continue to face far higher mortality rates than children in HICs—if all countries reached the average 2019 U5MR of high-income countries by 2030, 27 million under-5 deaths could be averted between 2020 and 2030, compared with the current trends scenario.

The decline in mortality among children aged 1–59 months has outpaced the decline in mortality among neonates since the main causes of death differ for the two age groups and respond differently to various health interventions. The slower reduction in neonatal mortality compared with overall under-5 mortality and the fact that more countries are at risk of missing the SDG NMR target than U5MR target demonstrate the need for intensified focus on the neonatal period. Further reductions in neonatal mortality will require greater investment in building stronger health systems and services; improving coverage, quality, and equity of care in the antenatal period and at birth; and expanding coverage of high-quality care in the first week of life and for small and sick newborns, which not only saves neonates lives, but also prevents disability.^14^

The estimates presented here are up to the year 2019, with scenario-based projections for 2020–30, but these projections do not include the possible impact of the COVID-19 pandemic on child survival since we do not yet know the full extent of that impact. Although the direct mortality effect of COVID-19 on children and youths appears to be limited, experience with past epidemics^15^, ^16^, ^17^, ^18^ has shown that indirect effects of an outbreak—eg, medical and food supply chain disruption, declining use and provision of health-care services, and health-care resource and personnel reallocation—can be severe, sometimes outpacing the direct impact. Although the long-term implications of the SARS-CoV-2 pandemic on child health outcomes remain unknown, if hard won gains in child survival and progress towards SDG targets are to be maintained, timely, accurate, and widespread monitoring of child health and survival must be intensified, and commitments and investments from governments and donors must be scaled up^19^ to maintain and expand coverage of life-saving interventions.

To our knowledge, the UN IGME estimates are the most comprehensive, transparent, and up-to-date information on child survival, providing the basis for assessing progress towards survival goals and for evidence-based policy. However, despite expansion in data availability and advances in analytical methods, the lack of timely, high-quality data on child mortality for many countries is one of the limitations of this study. Only about a third of the countries in this study have well functioning vital registration systems than can be relied upon as the sole source of information on child survival, and these countries tend to be high-income. Household survey data and periodic censuses continue to be the primary data sources on child mortality in most low-income and middle-income countries. In addition to various sampling and non-sampling errors and biases, these data are not timely—for about a third of all countries, the latest available child mortality datapoint was for 2014 or earlier. The timeliness of these data is a limitation of this study since estimates in recent periods with no data rely on extrapolation based on a combination of country and global trends, and recent changes in mortality or in the pace of decline might not be picked up by older data and, thus, not reflected in these estimates. The lack of recent data in countries with ongoing conflicts or emergencies (eg, Yemen) is especially problematic as we might be underestimating mortality without sufficient data to inform on the crisis. Likewise, although we do adjust for crisis where applicable, the lack of data on the mortality situation of children in ongoing conflicts or emergencies hinders making informative long-term adjustments. Furthermore, these estimates are only as accurate as the quality of the underlying data. Although the B3 model can accommodate disparate data sources for a country and more data from vital registration systems and full birth histories are included in the UN IGME databases than ever before, it remains a critically important challenge to collect and assess the quality of all relevant data for child mortality estimation.

Finally, although this study aims to provide an exhaustive description of the global, regional, and country levels and trends in under-5 mortality along with projections of possible mortality scenarios, it does not directly address the underlying causes of child mortality nor specific interventions that might be directly associated with child deaths since these topics are at least partially addressed by other groups and models—see for example the cause of death estimation work of WHO and the Maternal and Child Epidemiology Estimation group^20^ and the Lives Saved Tool,^21^ which links intervention coverage to changes in mortality. Notably, the estimated under-5 deaths envelope is used by both previously mentioned studies.

Notwithstanding dramatic reductions in child mortality over the past 30 years, the global number of child deaths remains unacceptably large, and children continue to face widespread inequality in their chances of survival. Countries and the international community must take immediate and targeted action to support further decline in child mortality where it is most needed. If these actions are absent, millions more children will die preventable deaths in the next decade.

## Data sharing

All underlying input data from civil registration systems, surveys, and censuses, along with the country level estimates and regional aggregation, are publicly available at childmortality.org, where links are provided to additional explanatory notes. The B3 model code is available upon request.

## Declaration of interests

We declare no competing interests.
