# Supplementary material for: Global, regional, and national trends in under-5 mortality between 1990 and 2019 with scenario-based projections until 2030: a systematic analysis by the UN Inter-agency Group for Child Mortality Estimation
Source: Lancet Glob Health. 2022 Jan 18;10(2):e195–206. doi: 10.1016/S2214-109X(21)00515-5 (PMC8789561; doi:10.1016/S2214-109X(21)00515-5)
Supplement: Supplementary appendix [file mmc1.pdf]

THE LANCET
Global Health

Supplementary appendix

This appendix formed part of the original submission and has been peer reviewed. We post it as supplied by the authors.

Supplement to: Sharrow D, Hug L, You D, et al. Global, regional, and national trends in under-5 mortality between 1990 and 2019 with scenario-based projections until 2030: a systematic analysis by the UN Inter-agency Group for Child Mortality Estimation. *Lancet Glob Health* 2022; **10:** e195–206.

Supplementary appendix to Global, regional and national trends in under-5 mortality 1990-2019 with scenario-based projections to 2030: a systematic analysis by the United Nations Inter-agency Group for Child Mortality Estimation

Contents

1. Methods 2
1.1. Overview 2
1.2. Data sources 2
Civil registration data 3
Survey Data 4
Adjustment for missing mothers in high-HIV-prevalence settings 5
Systematic and random measurement error 6
1.3. Estimating levels and trends in under-5 mortality rate 6
Summary 6
Splines regression 7
Adjustment for rapidly changing child mortality driven by HIV/AIDS 9
1.4. Estimating levels and trends in neonatal mortality rate 10
1.5. Calculating number of deaths 11
1.6. Child mortality due to conflict and environmental disasters 11
2. Regional Classifications 13
2.1. UNICEF classifications 13
2.2. World Bank classifications 14
3. Supplementary tables 16
3.1. Table A.1 Levels and trends in U5MR, by country, 1990-2019 16
3.2. Table A.2 Levels and trends in NMR, by country, 1990-2019 23
3.3. Table A.3 Number of under-5 and neonatal deaths, by country, 1990-2019 30
3.4. Table A.4 Series in U5MR database 38
4. Supplementary figures 81
4.1. Figure A.1 Trends in U5MR, NMR, and number of under-5 deaths, 1990-2019, with 90% uncertainty intervals, by region 81
4.2. Figure A.2 Annual rate of reduction 2010-2019 and required annual rate of reduction to achieve SDG target 82

4.3. Figure A.3 Projected global under-5 mortality rate and the number of under-5 deaths 2020-2030 83

4.4. Figure A.4 Under-five mortality estimates with underlying data, by country 84

4.5. Figure A.5 Neonatal mortality estimates with underlying data, by country 280

1. Methods

1.1. Overview

UN IGME follows the following broad strategy to arrive at annual estimates of child mortality:

1. Compile and assess the quality of all available nationally representative data relevant to the estimation of child mortality, including data from vital registration systems, population censuses, household surveys and sample registration systems;
2. Assess data quality, recalculate data inputs and make adjustments as needed by applying standard methods;
3. Fit a statistical model to these data to generate a smooth trend curve that averages possibly disparate estimates from the different data sources for a country;
4. Extrapolate the model to a target year (in this case, mid-2019).

To increase the transparency of the estimation process, UN IGME has developed a child mortality web portal, Child Mortality Estimation (CME) Info, available at <www.childmortality. org>. It includes all available data and shows estimates for each country as well as which data are currently officially used by UN IGME. Once new estimates are finalized, CME Info is updated accordingly.

UN IGME applies a common methodology across countries and uses original empirical data from each country. However, it does not report figures produced by individual countries using other methods, which would not be comparable to other country estimates. Applying a consistent methodology allows for comparisons between countries, despite the varied number and types of data sources.

UN IGME estimates are based on nationally available data from censuses, surveys, vital registration systems or sample registration systems. UN IGME does not use covariates to derive its estimates but rather, applies a curve-fitting method to empirical data after data quality assessment. Countries often use a single source for their official estimates or apply methods different to those used by UN IGME. The differences between UN IGME and national official estimates are usually not large if the empirical data are of good quality. UN IGME aims to minimize errors for each estimate, harmonize trends over time, and produce up to date and properly evaluated estimates of child mortality. Because errors are inevitable in data, there will always be uncertainty around data and estimates, both nationally and internationally. To allow for added comparability, UN IGME generates all child mortality estimates with uncertainty bounds.

1.2. Data sources

The first step in the process of arriving at estimates of levels and recent trends of child mortality is to compile all newly available data and add the data to the UN IGME database (newly available may include newer, recently released data and occasionally, results from older censuses or surveys not previously available). Nationally representative estimates of under-five mortality can be derived from several

different sources, including civil registration and sample surveys. Demographic surveillance sites and hospital data are excluded as they are rarely nationally representative. The preferred source of data is a civil registration system that records births and deaths on a continuous basis. If registration is complete and this system functions efficiently, the resulting estimates will be accurate and timely. However, many low- and middle-income countries do not have well-functioning vital registration (VR) systems. Therefore, household surveys such as the UNICEF-supported Multiple Indicator Cluster Surveys, the USAID-supported Demographic and Health Surveys, and periodic population censuses have become the primary sources of data on mortality among children under age 5. These surveys ask women about the survival of their children and it is these reports (or microdata upon availability) that provide the basis of child mortality estimates for a majority of low- and middle-income countries.

Whatever the method used to derive the estimates, data quality is critical. UN IGME assesses data quality and does not include data sources with substantial non-sampling errors or omissions as underlying empirical data in its statistical model.

The full set of empirical data used in this analysis is publicly available from the UN IGME web portal, CME Info <www.childmortality.org>. The web portal also includes data that have been excluded and indicates the fact.

Civil registration data

Data from civil registration systems are the preferred data source for child mortality estimation. For data from civil registration, the calculation of U5MR and IMR is derived from a standard period abridged life table. The inputs are number of deaths for age group <1 year (noted D_0) and for the age group 1-4 years ($D_{1\text{-}4}$), as well as the mid-year population for the same age groups (P_0 and $P_{1\text{-}4}$).

The formulae are as follows:

Given that:

${}_nq_x$ is the probability of dying between age x and age $x+n$,

${}_1M_0 = D_0/P_0$, death rate for age <1,

${}_4M_1 = D_{1\text{-}4}/P_{1\text{-}4}$, death rate for age group 1-4,

Then:

$${}_1q_0 = {}_1M_0 \;/\; [1+(1-{}_1a_0)*{}_1M_0]$$

where ${}_1a_0$ is the fraction of year lived by an infant who died

${}_1a_0$= 0.1 for low mortality country and ${}_1a_0$= 0.3 for high mortality country

$${}_5q_0 = 1-(1-{}_1q_0)*(1-{}_4q_1)$$

where ${}_4q_1 = 4*{}_4M_1 \;/[1+ (4-{}_4a_1) * {}_4M_1]$

where ${}_4a_1$ is the fraction of years lived by a child aged 1-4 years who died

${}_4a_1$= 1.6

Finally: IMR = ${}_1q_0$ *1000 and U5MR= ${}_5q_0$*1000

For NMR, the number of deaths under 28 days of age and live births are used to calculate the neonatal mortality rate.

For civil registration data (with available data on the number of deaths and mid-year populations), annual observations were initially constructed for all observation years in a country. For country-years in which the coefficient of variation exceeded 10 per cent for children under 5 years, deaths and mid-year populations were pooled over longer periods. Starting from more recent years, deaths and population were combined with one or more previous years to reduce spurious fluctuations in countries where small numbers of births and deaths were observed. The coefficient of variation is defined to be the stochastic standard error of the 5q0 (5q0=U5MR/1,000) observation divided by the value of the 5q0 observation. The stochastic standard error of the observation is calculated using a Poisson approximation using live birth numbers, given by sqrt(5q0/lb), where lb is the number of live births in the year of the observation. After this recalculation of the civil registration data, the standard errors are set to a minimum of 2.5 per cent for input into the model following the original model specification[1]. A similar approach was used for neonatal mortality. Figure 1 illustrates the recalculation of civil registration data for Iceland.

Figure 1 Illustration of the recalculation of civil registration data for Iceland. Black dots refer to annual observations while red dots refer to the recalculated observations with longer observation periods, with 95% observation-specific uncertainty intervals

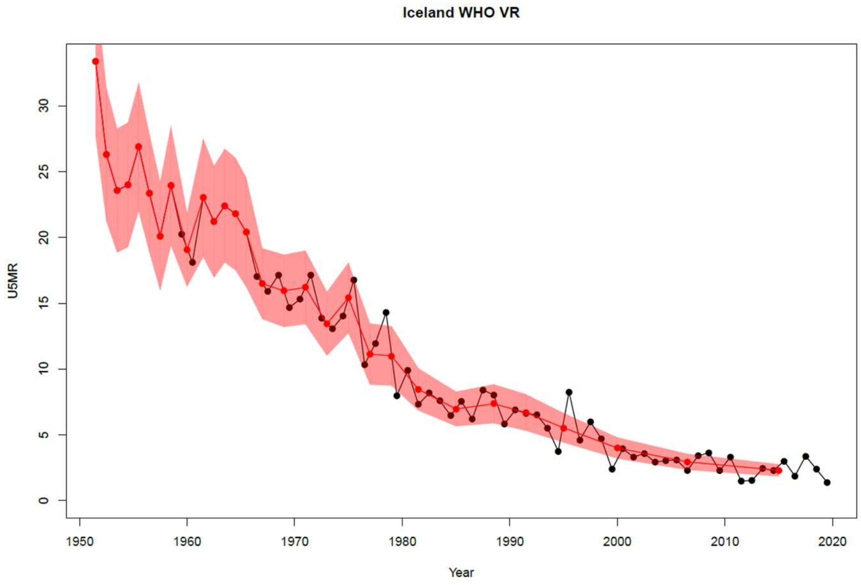

Survey Data

The majority of survey data on under-five mortality is collected in one of two ways: the full birth history (FBH), whereby women are asked for the date of birth of each of their children, whether the child is still alive, and if not, the age at death; and the summary birth history (SBH), whereby women are asked only about the number of their children ever born and the number that have died (or equivalently the number still alive).

---

[1] Alkema L, New JR. Global estimation of child mortality using a Bayesian B-spline bias-reduction method. The Annals of Applied Statistics. 2014; 8: 2122–49.

FBH data, collected by all Demographic and Health Surveys (DHS) and increasingly also by Multiple Indicator Cluster Surveys (MICS), allow the calculation of child mortality indicators for specific time periods in the past. DHS and MICS usually publish under-five child mortality estimates for three 5-year periods before the survey, that is, 0 to 4, 5 to 9, and 10 to 14 years before the survey.[2, 3, 4] UN IGME has re-calculated estimates for calendar year periods, using single calendar years for periods shortly before the survey, and then gradually increasing the number of years for periods further in the past, whenever microdata from the survey are available. The cut-off points for a given survey for shifting from estimates for single calendar years to two years, or two years to three, etc., are based on the coefficients of variation (a measure of sampling uncertainty) of the estimates[5].

In general, SBH data, collected by censuses and many household surveys, use the age of the woman as an indicator of the exposure time of her children to the risk of death and use models to estimate under-five mortality indicators for periods in the past for women aged 25 to 29 through 45 to 49. This method is well known but has several shortcomings. Starting with the 2014 round of estimation, the UN IGME changed the method of estimation for summary birth histories to one based on classification of women by the time that has passed since their first birth. This newer method has several benefits over the previous one: First, it generally has lower sampling errors. Second, it avoids the problematic assumption that the estimates derived for each age group adequately represent the mortality of the whole population. As a result, it has less susceptibility to the selection effect of young women who give birth early, since all women who give birth necessarily must have a first birth and therefore are not selected for. Third, the method tends to show less fluctuation across time, in particular in countries with relatively low fertility and mortality. The IGME considers the improvements in the estimates based on time since first birth worthwhile when compared to the estimates derived from the classification by age of mother, hence in cases where the microdata is available, the UN IGME has reanalysed the data using the new method. Child mortality estimates from SBH data were not included if estimates from FBH data in the same survey were available [6].

SBH data are not used to derive neonatal mortality.

Adjustment for missing mothers in high-HIV-prevalence settings

In populations severely affected by HIV/AIDS, HIV-positive (HIV+) children will be more likely to die than other children and will also be less likely to be reported since their mothers will have been more likely to

---

[2] http://mics.unicef.org/tools

[3] Croft, Trevor N., Aileen M. J. Marshall, Courtney K. Allen, et al. Guide to DHS Statistics. Rockville, Maryland, USA: ICF. 2018

[4] Hill, K. Chapter 15, Child Mortality in Moultrie TA, RE Dorrington, AG Hill, K Hill, IM Timæus and B Zaba (eds). 2013. Tools for Demographic Estimation. Paris: International Union for the Scientific Study of Population. demographicestimation.iussp.org

[5] Pedersen J, Liu J. Child Mortality Estimation: Appropriate Time Periods for Child Mortality Estimates from Full Birth Histories. Plos Medicine. 2012;9(8).

[6] Silva R. Child Mortality Estimation: Consistency of Under-Five Mortality Rate Estimates Using Full Birth Histories and Summary Birth Histories. Plos Medicine. 2012;9(8).

die also. Child mortality estimates will thus be biased downwards. The magnitude of the bias will depend on the extent to which the elevated under-five mortality of HIV+ children is not reported because of the deaths of their mothers. A method was developed to adjust HIV/AIDS related mortality for each survey data observation from FBH during HIV/AIDS epidemics (1980-present), by adopting a set of simplified but reasonable assumptions about the distribution of births to HIV+ women, primarily relating to the duration of their infection, vertical transmission rates, and survival times of both mothers and children from the time of the birth[7] . This method was applied to all DHS and MICS surveys with FBH. The model was improved to incorporate the impact of antiretroviral therapies (ART) and prevention of mother to child transmission (PMTCT)[8].

Systematic and random measurement error

Data from these different sources require different calculation methods and may suffer from different errors, for example random errors in sample surveys or systematic errors due to misreporting. As a result, different surveys often yield widely different estimates of U5MR or other mortality indicators for a given time period. In order to reconcile these differences and take better account of the systematic biases associated with the various types of data inputs, Alkema and New (2014) developed an estimation method to fit a smoothed trend curve to a set of observations and to extrapolate that trend to a defined time point, in this case 2019. This method is described in the following section.

1.3. Estimating levels and trends in under-5 mortality rate

Summary

Estimation and extrapolation of under-5 mortality rates (U5MR) was undertaken using the Bayesian B-splines bias-adjusted model, referred to as the B3 model. This model was developed, validated, and used to produce the previous round of UN IGME child mortality estimates.

In the B3 model, log(U5MR) is estimated with a flexible spline regression model. The spline regression model is fitted to all U5MR observations in the country. An observed value for U5MR is considered to be the true value for U5MR multiplied by an error factor, i.e. observed U5MR = true U5MR * error, or on the log-scale, log(observed u5mr) = log(true U5MR) + log(error), where error refers to the relative difference between an observation and the truth. While estimating the true U5MR, properties of the errors that provide information about the quality of the observation, or in other words, the extent of error that we expect, are taken into account. These properties include the standard error of the observation, its source type (e.g. DHS versus census) and if the observation is part of a data series from a specific survey (and how far the data series is from other series with overlapping observation periods). These properties are summarized in the so-called data model. When estimating the U5MR, the data model adjusts for the errors in the observations, including the average systematic biases associated with different types of data sources, using information on data quality for different source types from all countries in the world.

---

[7] Walker N, Hill K, Zhao FM. Child Mortality Estimation: Methods Used to Adjust for Bias due to AIDS in Estimating Trends in Under-Five Mortality. Plos Medicine. 2012;9(8).

[8] Johnson P, Mizoguchi N, Pantazis A. Improved Method for Adjusting for Bias due to HIV Mortality in Estimates of Child Mortality. Paper prepared for Population Association of America Annual Meeting April 22-25, 2020. Washington, DC (forthcoming).

Figure 2 displays plots of the U5MR over time for Senegal, used here for illustrative purposes. The B3 estimates are in red. Ninety per cent uncertainty intervals for the U5MR are given by the pink bands. All data available for the country are shown as coloured points, with observations from the same data series joined by lines. Solid points and lines represent data series/observations that were included for curve-fitting. Grey bands represent the standard errors of the observations where available.

Figure 2 Illustration of the B3 model for Senegal. Left: Plot of the U5MR over time for Senegal, with the B3 estimates in red. Right: Zoomed in version of the plot on the left.

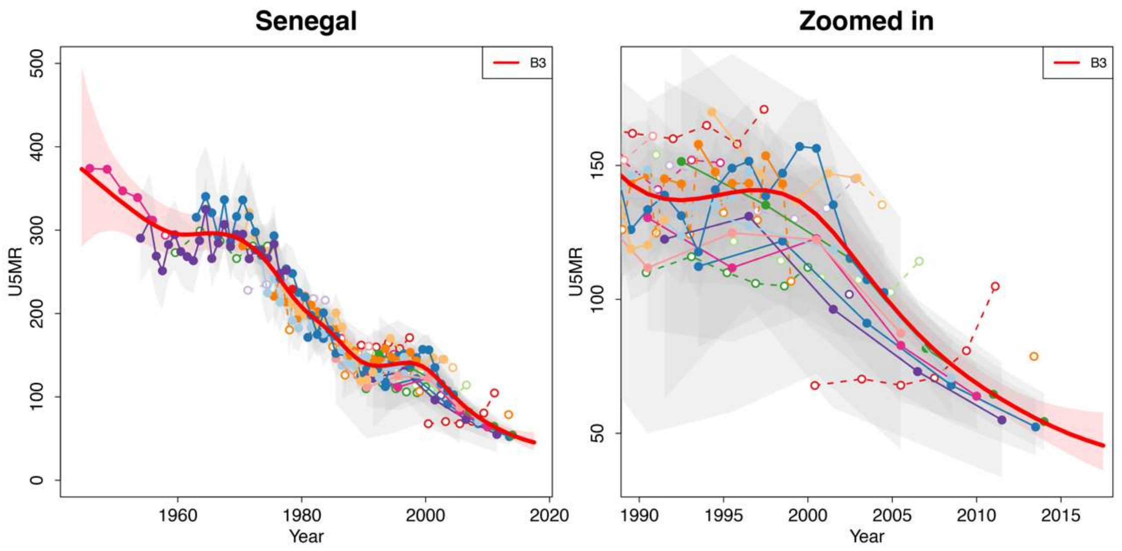

The B3 method was developed and implemented for the UN IGME by Leontine Alkema and Jin Rou New from the National University of Singapore with guidance and review by the TAG of the UN IGME. A more complete technical description of the B3 model is available elsewhere[9].

Splines regression

The splines regression fitting method for Norway is illustrated in Figure 3. Splines are smooth curves, placed 2.5 years apart, that add up to 1 at any point in time. For any year, the estimated log(U5MR) is the sum of the non-zero splines in that year multiplied by the corresponding spline coefficients (displayed by dots). For example, log(U5MR) in 1980 in Norway is given by the sum of the yellow and grey splines to the left of black line (at the year 1980) and the black and red splines to the right, multiplied by their respective spline coefficients in the same colour.

The spline coefficients determine what the resulting fitted curve looks like. When estimating the spline coefficients, we obtain a flexible yet reasonably smooth U5MR curve by assuming that the difference between two adjacent coefficients (for example for years 1981 and 1983.5) is given by the difference between the previous two coefficients (for years 1978.5 and 1981) with an estimated data-driven "distortion term" added to it. For example, in Norway during the early 1980s, these distortion terms are estimated to be around zero when U5MR did not change much, but they are negative in the late 1980s

---

[9] Alkema L, New JR. Global estimation of child mortality using a Bayesian B-spline bias-reduction method. The Annals of Applied Statistics. 2014; 8: 2122–49.

when the U5MR started to decline again. The resulting fit in Norway illustrates that the spline fit is able to follow the observed changes in the data closely.

Figure 3 Illustration of the B-splines regression model for Norway. Top row: B-splines and the estimated spline coefficients. Bottom row: Observed log(U5MR) and U5MR (black dots) plotted against time, together with the spline estimates (red line).

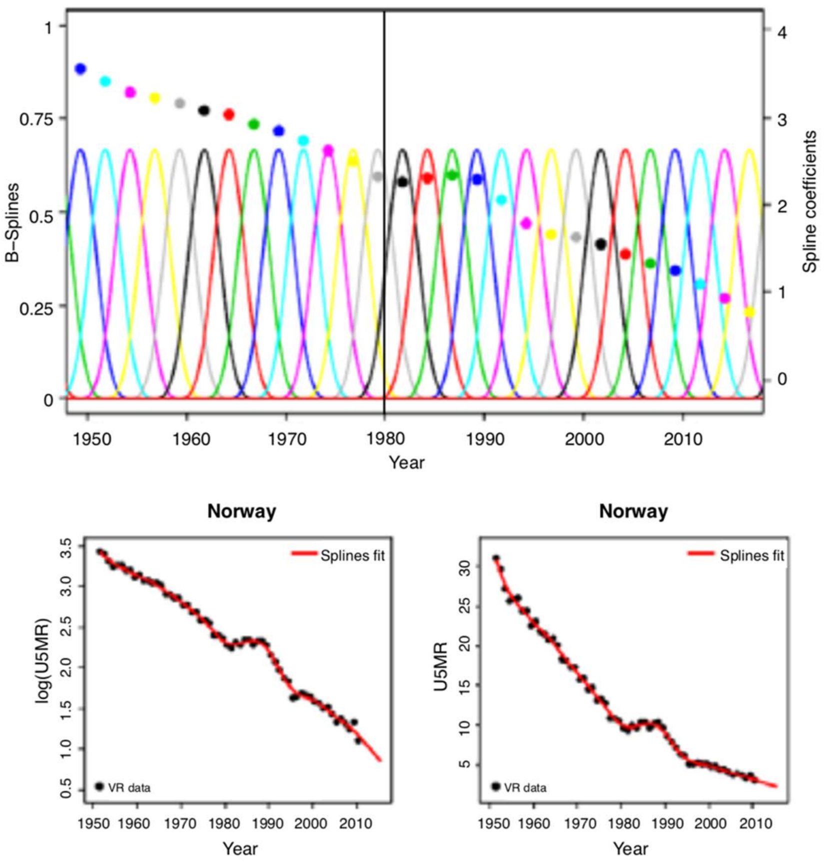

The variance of the distortion terms determines the smoothness of the fit during the observation period; large fluctuations in these distortion terms imply that the trend can vary greatly from one period to the next. The amount of smoothing is country-specific for the majority of countries. An average global level of smoothing is used for countries with a small number of live births, countries with both vital registration (VR) and non-VR data included in the fitting and countries with a gap of more than five years in their VR data.

Due to the nature of the data in such countries, a large variance for the distortion terms tends to be estimated, so a global level of smoothing helps to reduce fluctuations in the trend.

After the most recent observation period ends, country-specific U5MR is extrapolated to the target year through the estimation of “future spline coefficients”, or equivalently, by ‘projecting’ the differences between adjacent spline coefficients for the extrapolation period. The mean projected difference in spline coefficients is given by the estimated difference in the two most recent adjacent spline coefficients, and the uncertainty therein is based on the variability in the observed distortions in the

country's past. Based on out-of-sample validation exercises, this approach is shown to work well for the majority of countries but leads to unnecessarily wide uncertainty intervals (or extreme extrapolations) for a subset of countries where the most recent change in spline coefficients is very uncertain (or an extreme value). We avoid such uncertain and extreme U5MR extrapolations in longer extrapolation periods by combining the country-specific projected differences in spline coefficients with a global distribution of observed differences in the past. This final step results in the removal of very extreme country-specific U5MR extrapolations.

Figure 4 Extrapolation period (i.e. the difference in years between the most recent included data point and the common end year, 2019.5) for the UN IGME 2020 estimation round

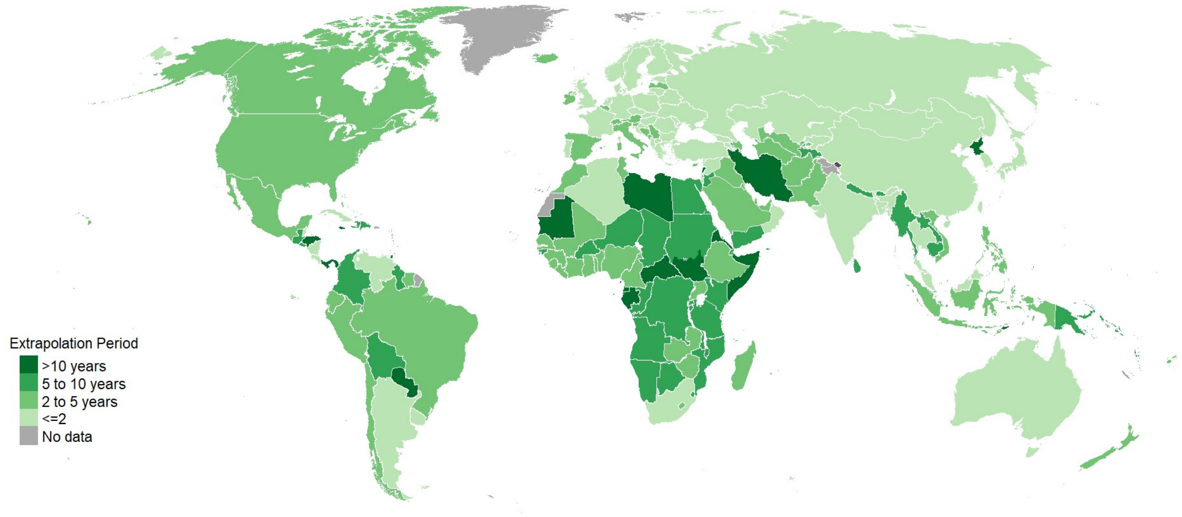

Note: This map does not reflect a position by UN IGME agencies or those of the institutions to which the authors are affiliated on the legal status of any country or territory or the delimitation of any frontiers.

Adjustment for rapidly changing child mortality driven by HIV/AIDS

To capture the extraordinarily rapid changes in child mortality driven by HIV/AIDS over the epidemic period in some countries, the regression model was fitted to data points for the U5MR from all causes other than HIV/AIDS, and then UNAIDS estimates of HIV/AIDS under-five mortality[10] were added to the estimates from the regression model. This method was used for 17 countries where the HIV prevalence rate exceeded 5 per cent at any point in time since 1980. Steps were as follows:

1. Compile and assess the quality of all newly available nationally representative data relevant to the estimation of child mortality.
2. Use UNAIDS estimates of HIV/AIDS child mortality[10] to adjust the data points from 1980 onwards to exclude HIV deaths (see section 1.2.
3. Fit the standard statistical model to the observations to HIV-free data points.
4. Extrapolate the model to the target year, in this case 2019.
5. Add back estimates of deaths due to HIV/AIDS (from UNAIDS)

[10] UNAIDS 1990–2018 HIV and AIDS estimates, 2019

6. For the epidemic period, a non-HIV curve of IMR is derived from U5MR using model life tables (see Section 4) and then the UNAIDS estimates of HIV/AIDS deaths for children under age 1 are added to generate the final IMR estimates.

1.4. Estimating levels and trends in neonatal mortality rate

The neonatal mortality rate (NMR) is defined as the probability of dying between birth and exact age 28 days per 1000 live births. In 2015 the UN IGME method for estimating NMR was updated. The new Bayesian methodology is similar to that used to estimate U5MR and derive estimates by sex. It has the advantage that, compared to the previous model, it can capture data-driven trends in NMR within countries and over time for all countries. A more complete technical description of the model is available elsewhere[11].

We model the ratio R(c,t), which refers to the ratio of NMR to the difference of U5MR and NMR in country c and year t, i.e. R(c,t) = NMR/(U5MR - NMR). For each country-year, we assume that the ratio is given by:

R(c,t) = W(c,t) * P(c,t),

where

W(c,t) refers to the expected ratio for that country-year, and

Country multiplier P(c,t) represents country-specific trends in the ratio over time that differ from the expected level.

As U5MR decreases, the proportional share of mortality in the first month of life tends to increase. The W(c,t) term accounts for this relationship; it is the expected ratio for the country-year based on the UN IGME-estimated U5MR for that country-year. It is modelled as a linear function of U5MR with a changing slope:

$$W(c,t) = \beta 0 \qquad \text{if } U5MR(c,t) < Ucut$$

$$W(c,t) = \beta 0 + \beta 1 * U5MR(c,t) \qquad \text{if } U5MR(c,t) \geq Ucut$$

Ucut is an estimated constant that represents the level of U5MR after which as U5MR increases, the ratio NMR/(U5MR - NMR) decreases. The parameters of this model are estimated based on all available data such that W(c,t) represents a ‘global relation’ between the ratio and U5MR.

The country multiplier P(c,t) is modelled with a B-splines regression model. The P(c,t) represents a country-specific intercept, which is modelled hierarchically, and fluctuations around that intercept over time. For any particular country, the ratio can overall be higher- or lower-than-expected given the level of U5MR in that country, but the fluctuations allow this relationship to change over time within a country. A degree of smoothness is imposed on the fluctuations to ensure relatively smooth trajectories for any given country through time. We model the ratio of NMR/(U5MR - NMR); estimates of NMR are obtained by recombining the estimates of the ratio with UN IGME-estimated U5MR.

[11] Alexander, M. & Alkema, L. Global Estimation of Neonatal Mortality using a Bayesian Hierarchical Splines Regression Model Demographic Research, 2018, 38, 335-372.

For neonatal mortality in HIV-affected and crisis-affected populations, the ratio is estimated initially for non-AIDS and non-crisis mortality. After estimation, crisis neonatal deaths are added back on to the neonatal deaths to compute the total estimated neonatal mortality rate. No AIDS deaths are added back to the NMR, thereby assuming that HIV/AIDS-related deaths only affect child mortality after the first month of life.

1.5. Calculating number of deaths

A birth-week cohort method is used to calculate the absolute number of deaths among neonates, infants, and children under age 5. First, each annual birth cohort is divided into 52 equal birth-week cohorts. Then, each birth-week cohort is exposed throughout the first five years of life to the appropriate calendar year- and age-specific mortality rates depending on cohort age, assuming that mortality rates are constant over the age intervals. For example, the 20th birth week cohort of the year 2000 will be exposed to the infant mortality rates in both 2000 and 2001. All deaths from birth-week cohorts occurring as a result of exposure to the mortality rate for a given calendar year are allocated to that year and are summed by age group at death to get the total number of deaths for a given year and age group. Continuing with the above example, deaths from the 20th birth-week cohort of the year 2000 would contribute to infant deaths in year 2000 and 2001. Any deaths occurring among the 20th birth-week cohort of year 2000 after the 20th week in 2001 would contribute to under-5 deaths for year 2001 and so forth. Under-five deaths in each calendar year are calculated by summing up all the deaths under age five across all age group cohorts in that year. The annual estimate of the number of live births in each country from the World Population Prospects: the 2019 revision[12] are used to calculate the numbers of deaths.

1.6. Child mortality due to conflict and environmental disasters

Estimated deaths for major crises including conflicts, environmental disasters (e.g. floods, earthquakes, cyclones), and epidemics were derived from various data sources from 1990 to present. Data on all-age deaths from environmental disasters were obtained from the CRED International Disaster Database[13] and the proportions of deaths occurring under age 5 and under age 1 were estimated using disaster-type specific age patterns described elsewhere[14]. Conflict deaths were obtained from the Uppsala Conflict Data Program/Peace Research Institute Oslo datasets as well as reports prepared by the UN and other organizations on specific conflicts, again using age patterns to estimate the proportions of under-five and under-one deaths. We calculate a crisis-U5MR and crisis-IMR from these age-specific crisis deaths.

---

[12] United Nations Department of Economic and Social Affairs Population Division. World Population Prospects - the 2019 revision. New York: United Nations, 2019.

[13] CRED. EM-DAT: The CRED International Disaster Database. Belgium: Université Catholique de Louvain. (available from: http://www.emdat.be/)

[14] World Health Organization. WHO methods and data sources for country-level causes of death 2000-2017. Global Health Estimates Technical Paper WHO/HMM/IER/GHE/2018.4. Geneva: WHO, 2018. (available from: <www.who.int/healthinfo/global_burden_disease/childcod_methods_2000_2017.pdf>).

Estimated child deaths due to major crises were used to adjust mortality rates in specific country-years if the crisis met the following criteria:

1. The crisis was isolated to a few years
2. Under-five crisis deaths were more than 10% of under-five non-crisis deaths in the given year
3. Crisis U5MR was more than 0.2 per 1,000 in the given year
4. Number of under-five crisis deaths was greater than 10.

These criteria resulted in crisis adjustments being incorporated into the UN IGME under-five mortality estimates for 22 countries. Crisis deaths were incorporated into the U5MR estimates by first excluding any data points from crisis years, fitting the B3 model to the remaining data, and then adding the crisis-specific death rate to the fitted crisis-free B3 curve. For countries where IMR is derived from U5MR, the crisis-free U5MR trajectories are used to derive a crisis-free IMR to which infant crisis adjustments are applied. The procedure produces a smooth crisis-free fit in all non-crisis years, while also including acute spikes related to specific crises.

Crisis death estimates are uncertain but presently no uncertainty around crisis deaths is included in the U5MR uncertainty intervals. We assume the relative uncertainty in the adjusted U5MR is equal to the relative uncertainty in the non-adjusted U5MR; this assumption will be revisited in the near future. The UN IGME has assessed recent, ongoing crises, and based on the lack of currently available data and the difficulties of estimating the broader impact of these crises on health systems, UN IGME holds the mortality rate estimates constant from the start of the crisis while increasing the uncertainty over the crisis time for three countries: South Sudan, Venezuela (Bolivarian Republic of) and Yemen. Where applicable, direct crisis deaths have been added to the constant trend estimate. UN IGME will review new data, if available, in the next estimation round and revise estimates accordingly.

2. Regional Classifications

Estimates at the regional level in the paper were based on the following classifications. The paper makes use of both UNICEF regional classifications and World Bank income classifications.

2.1. UNICEF classifications

East Asia and Pacific

Australia; Brunei Darussalam; Cambodia; China; Cook Islands; Democratic People's Republic of Korea; Fiji; Indonesia; Japan; Kiribati; Lao People's Democratic Republic; Malaysia; Marshall Islands; Micronesia (Federated States of); Mongolia; Myanmar; Nauru; New Zealand; Niue; Palau; Papua New Guinea; Philippines; Republic of Korea; Samoa; Singapore; Solomon Islands; Thailand; Timor-Leste; Tonga; Tuvalu; Vanuatu; Viet Nam

Europe and Central Asia

Eastern Europe and Central Asia; Western Europe

Eastern Europe and Central Asia

Albania; Armenia; Azerbaijan; Belarus; Bosnia and Herzegovina; Bulgaria; Croatia; Georgia; Kazakhstan; Kyrgyzstan; Montenegro; Republic of Moldova; Northern Macedonia; Romania; Russian Federation; Serbia; Tajikistan; Turkey; Turkmenistan; Ukraine; Uzbekistan

Western Europe

Andorra; Austria; Belgium; Cyprus; Czechia; Denmark; Estonia; Finland; France; Germany; Greece; Hungary; Iceland; Ireland; Italy; Latvia; Lithuania; Luxembourg; Malta; Monaco; Netherlands; Norway; Poland; Portugal; San Marino; Slovakia; Slovenia; Spain; Sweden; Switzerland; United Kingdom

Latin America and Caribbean

Antigua and Barbuda; Argentina; Bahamas; Barbados; Belize; Bolivia (Plurinational State of); Brazil; Chile; Colombia; Costa Rica; Cuba; Dominica; Dominican Republic; Ecuador; El Salvador; Grenada; Guatemala; Guyana; Haiti; Honduras; Jamaica; Mexico; Nicaragua; Panama; Paraguay; Peru; Saint Kitts and Nevis; Saint Lucia; Saint Vincent and the Grenadines; Suriname; Trinidad and Tobago; Uruguay; Venezuela (Bolivarian Republic of)

Middle East and North Africa

Algeria; Bahrain; Egypt; Iran (Islamic Republic of); Iraq; Israel; Jordan; Kuwait; Lebanon; Libya; Morocco; Oman; Qatar; Saudi Arabia; State of Palestine; Syrian Arab Republic; Tunisia; United Arab Emirates; Yemen

North America

Canada; United States of America

South Asia

Afghanistan; Bangladesh; Bhutan; India; Maldives; Nepal; Pakistan; Sri Lanka

Sub-Saharan Africa

Eastern and Southern Africa; West and Central Africa

Eastern and Southern Africa

Angola; Botswana; Burundi; Comoros; Djibouti; Eritrea; Eswatini; Ethiopia; Kenya; Lesotho; Madagascar; Malawi; Mauritius; Mozambique; Namibia; Rwanda; Seychelles; Somalia; South Africa; South Sudan; Sudan; Uganda; United Republic of Tanzania; Zambia; Zimbabwe

West and Central Africa

Benin; Burkina Faso; Cabo Verde; Cameroon; Central African Republic; Chad; Congo; Côte d'Ivoire; Democratic Republic of the Congo; Equatorial Guinea; Gabon; Gambia; Ghana; Guinea; Guinea-Bissau; Liberia; Mali; Mauritania; Niger; Nigeria; Sao Tome and Principe; Senegal; Sierra Leone; Togo

Map A1: UNICEF regional classifications.

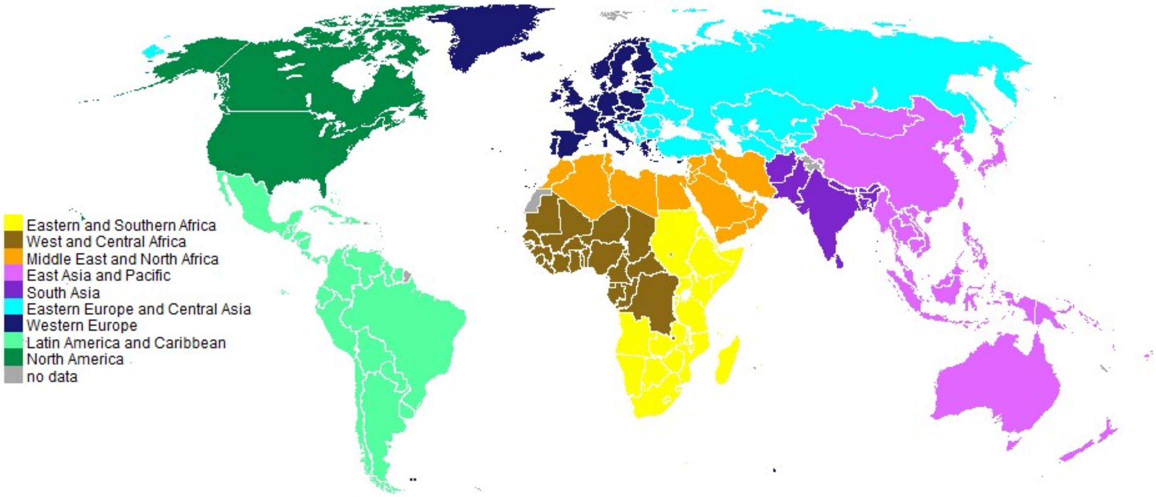

Note: This map does not reflect a position by UN IGME agencies or those of the institutions to which the authors are affiliated on the legal status of any country or territory or the delimitation of any frontiers.

2.2. World Bank classifications

Low income

Afghanistan; Burkina Faso; Burundi; Central African Republic; Chad; Democratic People's Republic of Korea; Democratic Republic of the Congo; Eritrea; Ethiopia; Gambia; Guinea; Guinea-Bissau; Haiti; Liberia; Madagascar; Malawi; Mali; Mozambique; Niger; Rwanda; Sierra Leone; Somalia; South Sudan; Sudan; Syrian Arab Republic; Tajikistan; Togo; Uganda; Yemen

Lower middle income

Algeria; Angola; Bangladesh; Benin; Bhutan; Bolivia (Plurinational State of); Cabo Verde; Cambodia; Cameroon; Comoros; Congo; Cote d'Ivoire; Djibouti; Egypt; El Salvador; Eswatini; Ghana; Honduras; India; Kenya; Kiribati; Kyrgyzstan; Lao People's Democratic Republic; Lesotho; Mauritania; Micronesia (Federated States of); Mongolia; Morocco; Myanmar; Nepal; Nicaragua; Nigeria; Pakistan; Papua New Guinea; Philippines; Republic of Moldova; Sao Tome and Principe; Senegal; Solomon Islands; Sri Lanka; State of Palestine; Timor-Leste; Tunisia; Ukraine; United Republic of Tanzania; Uzbekistan; Vanuatu; Viet Nam; Zambia; Zimbabwe

Upper middle income

Albania; Argentina; Armenia; Azerbaijan; Belarus; Belize; Bosnia and Herzegovina; Botswana; Brazil; Bulgaria; China; Colombia; Costa Rica; Cuba; Dominica; Dominican Republic; Ecuador; Equatorial Guinea; Fiji; Gabon; Georgia; Grenada; Guatemala; Guyana; Indonesia; Iran (Islamic Republic of); Iraq; Jamaica; Jordan; Kazakhstan; Lebanon; Libya; Malaysia; Maldives; Marshall Islands; Mexico; Montenegro; Namibia; Paraguay; Peru; Republic of North Macedonia; Russian Federation; Saint Lucia; Saint Vincent and the Grenadines; Samoa; Serbia; South Africa; Suriname; Thailand; Tonga; Turkey; Turkmenistan; Tuvalu; Venezuela (Bolivarian Republic of)

High income

Andorra; Antigua and Barbuda; Australia; Austria; Bahamas; Bahrain; Barbados; Belgium; Brunei Darussalam; Canada; Chile; Croatia; Cyprus; Czechia; Denmark; Estonia; Finland; France; Germany; Greece; Hungary; Iceland; Ireland; Israel; Italy; Japan; Kuwait; Latvia; Liechtenstein; Lithuania; Luxembourg; Malta; Mauritius; Monaco; Nauru; Netherlands; New Zealand; Norway; Oman; Palau; Panama; Poland; Portugal; Qatar; Republic of Korea; Romania; Saint Kitts and Nevis; San Marino; Saudi Arabia; Seychelles; Singapore; Slovakia; Slovenia; Spain; Sweden; Switzerland; Trinidad and Tobago; United Arab Emirates; United Kingdom; United States of America; Uruguay

3. Supplementary tables

3.1. Table A.1 Levels and trends in U5MR, by country, 1990-2019

Note: Numbers in brackets are 90% uncertainty intervals.

	Under-five mortality rate (per 1,000 live births)				Average annual rate of reduction in under-five mortality rate (%)		
	**1990**	**2000**	**2010**	**2019**	**1990-1999**	**2000-2009**	**2010-2019**
Afghanistan	177.7 (162.5-194.3)	128.7 (119.6-138.9)	87.6 (80.3-95.8)	60.3 (47.4-74.6)	3.2 (2.4-4)	3.8 (3-4.6)	4.2 (2.2-6.4)
Albania	41 (36.4-46.1)	27.2 (24.8-29.9)	13.2 (12.8-13.7)	9.7 (9.1-10.3)	4 (2.7-5.4)	7 (5.9-8.2)	3.5 (2.6-4.3)
Algeria	49.5 (46.1-52.9)	39.7 (38.8-40.6)	27.4 (26.9-28)	23.3 (22.5-24)	2.3 (1.5-3.1)	3.7 (3.4-4)	1.8 (1.4-2.3)
Andorra	13 (3.3-50.4)	7.7 (3.8-15.3)	4.6 (2.2-9.6)	3 (0.8-11)	5.3 (-4.1-14.8)	5.1 (-3.6-13.4)	4.9 (-3.4-12.8)
Angola	222.2 (196.8-251.3)	203.9 (178.6-233.7)	120.3 (83.3-166.8)	74.7 (34.6-139.3)	0.7 (-0.6-1.9)	5.1 (2-8.7)	5.3 (0.9-11)
Antigua and Barbuda	14 (12.4-15.8)	15.5 (13.9-17.1)	10 (8.8-11.4)	6.6 (4.6-9.3)	-1.3 (-2.9-0.3)	4.2 (2.6-5.8)	4.6 (1-8.4)
Argentina	28.6 (28.1-29.2)	19.6 (19.3-20)	14.4 (14.2-14.7)	9.3 (8.8-9.8)	3.8 (3.5-4.1)	3 (2.7-3.3)	4.9 (4.3-5.5)
Armenia	48.9 (44.4-53.9)	30.7 (27.6-33.8)	18.5 (15.6-21.2)	11.8 (8.8-15.2)	4.6 (3.9-5.5)	5 (3.8-6.5)	5 (2.9-7.3)
Australia	9.2 (9-9.4)	6.2 (6.1-6.3)	4.8 (4.7-4.9)	3.6 (3.4-3.8)	4.2 (3.9-4.5)	2.4 (2.1-2.7)	3.1 (2.5-3.8)
Austria	9.5 (9.3-9.8)	5.5 (5.3-5.7)	4.3 (4.2-4.5)	3.5 (3-4)	5.9 (5.4-6.4)	2.4 (1.8-3)	2.5 (0.7-4.2)
Azerbaijan	95.2 (86.2-105.1)	74.6 (66.5-83.7)	37.4 (30.9-45.3)	20.4 (13.1-31.9)	2 (0.7-3.1)	6.9 (4.9-9)	6.7 (2.1-11.4)
Bahamas	23.5 (22.3-24.7)	16.3 (15.3-17.3)	15.6 (14.6-16.6)	12.6 (9.8-16)	4 (3.2-4.9)	0.3 (-0.7-1.2)	2.3 (-0.4-5.2)
Bahrain	23 (22.2-23.8)	12.5 (11.9-13.1)	8.5 (8.1-9)	6.9 (5.5-8.8)	6.2 (5.5-6.8)	3.8 (3-4.5)	2.3 (-0.5-5)
Bangladesh	143.8 (139.6-148.1)	86.5 (83.7-89.3)	48.7 (46.8-50.6)	30.8 (27.9-33.8)	5 (4.6-5.4)	5.8 (5.3-6.2)	5.1 (4-6.2)
Barbados	18.1 (17-19.3)	15.1 (14.1-16.3)	15 (13.8-16.3)	12.7 (9-17.6)	2.1 (1.1-3.1)	0 (-1.2-1.2)	1.9 (-1.6-5.5)
Belarus	15.2 (14.9-15.5)	12.7 (12.5-13)	5.5 (5.3-5.7)	3.2 (2.9-3.6)	1.2 (0.8-1.5)	8.4 (8-8.8)	6 (4.8-7.1)
Belgium	10 (9.8-10.2)	5.8 (5.6-6)	4.5 (4.3-4.6)	3.4 (2.9-4)	5.6 (5.2-6)	2.7 (2.2-3.2)	3 (1.3-4.8)
Belize	38.3 (33.7-43.5)	23.6 (22.1-25.1)	18.9 (18-19.9)	12.3 (10.7-14.3)	5 (3.7-6.2)	2.2 (1.2-3.1)	4.8 (3-6.5)
Benin	175.1 (164.8-186.4)	138.8 (130.4-148.1)	110.8 (102.2-120.4)	90.3 (77.4-104.8)	2.3 (1.9-2.8)	2.3 (1.7-2.9)	2.3 (1-3.6)
Bhutan	127 (112.4-146.1)	77.1 (69.6-86.1)	42.2 (34.9-50.3)	28.5 (18.2-43.7)	4.9 (3.8-6.1)	6 (4.4-8)	4.4 (0.4-8.4)
Bolivia (Plurinational State of)	121.5 (115-128.4)	75.5 (70.9-80.5)	41.3 (36.7-46.4)	26 (19.6-34.1)	4.6 (4.1-5.2)	6 (5-7.1)	5.2 (2.7-7.7)
Bosnia and Herzegovina	18.1 (17.7-18.6)	9.9 (9.6-10.3)	7.2 (6.9-7.5)	5.9 (4.9-6.9)	6.2 (5.8-6.7)	3 (2.4-3.6)	2.3 (0.3-4.3)
Botswana	48.4 (40.7-57.9)	69.3 (55.8-95.9)	35.1 (20.3-58.9)	41.6 (15.1-87.2)	-4.2 (-7.6–1.6)	7.4 (2.3-13.2)	-1.9 (-9.9-8.4)
Brazil	62.9 (58.6-67.4)	34.6 (32.4-37.1)	18.7 (18.2-19.2)	13.9 (11.7-16.5)	5.9 (5.1-6.7)	6.3 (5.6-7.1)	3.3 (1.3-5.3)
Brunei Darussalam	13.3 (12.5-14.1)	10.3 (9.7-11)	9.7 (9-10.4)	11.4 (10.1-12.9)	2.4 (1.5-3.4)	0.8 (-0.2-1.9)	-1.8 (-3.5–0.2)
Bulgaria	18.4 (18-18.9)	17.4 (16.9-17.9)	10.9 (10.5-11.2)	6.7 (6.3-7.2)	0.1 (-0.3-0.5)	5 (4.5-5.4)	5.3 (4.5-6.1)

	Under-five mortality rate (per 1,000 live births)				Average annual rate of reduction in under-five mortality rate (%)		
	1990	2000	2010	2019	1990-1999	2000-2009	2010-2019
Burkina Faso	198.5 (185.9-212.1)	179.2 (166.9-192)	121.7 (109.4-134.9)	87.5 (63.9-120.9)	0.9 (0.3-1.5)	3.8 (3-4.6)	3.7 (0.4-6.7)
Burundi	174.3 (158.7-191.9)	155.2 (141.3-170.9)	90.6 (78.6-104)	56.5 (37.3-84.8)	0.9 (-0.2-2)	5.3 (3.8-6.7)	5.3 (1.1-9.4)
Cabo Verde	60.5 (58.3-62.7)	38.1 (37-39.3)	26.3 (25.2-27.3)	14.9 (12.3-18)	4 (3.4-4.5)	3.8 (3.3-4.4)	6.3 (4.1-8.5)
Cambodia	115.9 (107.4-125.4)	106.3 (97.6-115.9)	44 (37-52.4)	26.6 (14-50.3)	0.2 (-0.6-1)	8.9 (7.2-10.7)	5.6 (-1-12.3)
Cameroon	135.7 (126.5-145.5)	143.4 (133.1-154.6)	110.1 (100.3-120.7)	74.8 (61.6-89.9)	-0.8 (-1.6-0)	2.5 (1.7-3.4)	4.3 (2.4-6.3)
Canada	8.2 (8.1-8.4)	6.2 (6.1-6.3)	5.6 (5.5-5.7)	4.9 (4.5-5.2)	3 (2.8-3.4)	0.9 (0.6-1.2)	1.7 (0.9-2.5)
Central African Republic	176.8 (158.2-198.1)	169.8 (151-190.4)	147.6 (114.7-192.5)	110.1 (63.9-189.4)	0.4 (-0.8-1.6)	1.3 (-1.1-3.4)	3.3 (-1.3-8)
Chad	212.1 (196.7-228.8)	185.1 (171.5-200.6)	148.6 (133.7-165.4)	113.8 (86.4-150.4)	1.3 (0.6-1.9)	2.1 (1.3-3.1)	3 (0.3-5.6)
Chile	19.1 (18.7-19.5)	10.9 (10.7-11.1)	8.7 (8.5-8.9)	7 (5.3-9)	5.6 (5.2-6)	2.3 (2-2.7)	2.5 (-0.4-5.4)
China	53.8 (49.4-58.6)	36.8 (34.7-39.1)	15.8 (14.9-16.7)	7.9 (6.9-9.1)	3.5 (2.4-4.7)	8.5 (7.6-9.5)	7.7 (5.9-9.4)
Colombia	35.1 (32.6-37.6)	24.9 (22.9-26.9)	18.4 (16.1-21)	13.8 (9.7-19.4)	3.4 (2.7-4.2)	3 (1.7-4.2)	3.2 (0-6.5)
Comoros	124.4 (109.9-139.3)	100.4 (80.8-118.4)	84.4 (53.3-138.2)	62.9 (30.6-134.2)	2.3 (0.5-4.4)	1.6 (-3.3-5.1)	3.3 (-1.9-8.4)
Congo	90.4 (79.3-102.7)	114 (102.5-126.7)	62.2 (53.6-71.9)	47.8 (28.5-82.7)	-2.7 (-4.1–1.3)	6.3 (4.6-7.9)	2.9 (-2.8-8.1)
Cook Islands	24 (21.7-26.7)	18 (16-20.1)	10.7 (8.3-13.8)	7.6 (4.5-12.9)	2.7 (1.4-4)	5.2 (3-7.4)	3.8 (-0.8-8.3)
Costa Rica	16.9 (16.5-17.2)	13.1 (12.8-13.4)	10.5 (10.2-10.8)	8.6 (8.2-9.1)	2.4 (2.1-2.7)	2.2 (1.8-2.6)	2.2 (1.6-2.8)
Cote d'Ivoire	152.3 (142-163.2)	142.3 (132.2-153.3)	106.4 (96.2-117.3)	79.3 (63.1-99.6)	0.5 (-0.2-1.3)	2.9 (2-3.7)	3.3 (1.1-5.4)
Croatia	12.8 (12.4-13.2)	8.3 (8.1-8.6)	5.5 (5.2-5.7)	4.8 (4.3-5.3)	4.4 (3.9-4.9)	4.3 (3.7-4.9)	1.5 (0-2.7)
Cuba	13.5 (12.4-14.6)	8.8 (8-9.5)	6.3 (5.8-6.9)	5.1 (4.4-6.1)	4.4 (3.5-5.3)	3.4 (2.4-4.3)	2.3 (0.3-3.9)
Cyprus	11.2 (10.6-12)	6.6 (6.1-7.1)	3.5 (3.2-4)	2.3 (1.6-3.4)	5.2 (4.2-6.4)	6.2 (4.7-7.8)	4.8 (0.2-8.8)
Czechia	12.2 (11.9-12.4)	5.5 (5.3-5.7)	3.4 (3.3-3.5)	3.2 (3-3.4)	8.1 (7.7-8.6)	4.9 (4.3-5.4)	0.7 (-0.1-1.6)
Democratic People's Republic of Korea	43.4 (34-55.6)	60 (47-76.9)	29.5 (23.1-37.8)	17.3 (13.6-22.2)	-4.9 (-4.9–4.9)	7.3 (7.4-7.4)	5.9 (5.9-5.9)
Democratic Republic of the Congo	185.4 (167-204.7)	159.5 (144.7-176.8)	114.8 (95.1-136.1)	84.8 (54.3-128.7)	1.4 (0.3-2.4)	3.3 (1.7-5.1)	3.4 (-0.6-7.3)
Denmark	9 (8.7-9.3)	5.7 (5.5-5.9)	4.1 (3.9-4.3)	3.8 (3.5-4.1)	4.9 (4.3-5.4)	3.4 (2.7-4.1)	1 (-0.1-2)
Djibouti	117.9 (101.1-137.4)	101.1 (86.6-119.4)	76.4 (59.6-97.5)	57.5 (33.9-93.8)	1.5 (-0.7-3.1)	2.8 (0.5-5.5)	3.2 (-1.1-7.5)
Dominica	16.5 (15.4-17.6)	17 (15.8-18.2)	24.5 (22.5-26.7)	34.7 (31.6-42.4)	-0.1 (-0.6-0.4)	-3.5 (-4.2–2.8)	-3.9 (-5.6–3.4)
Dominican Republic	60 (56.3-64)	40.7 (37.5-44)	34.1 (29.4-39.6)	28 (19.4-40.7)	4 (3.2-4.8)	1.8 (0.3-3.3)	2.2 (-1.2-5.5)
Ecuador	53.6 (48.5-59.2)	28.9 (25.7-32.5)	18.3 (16.1-20.8)	14 (12.7-15.3)	6.2 (5-7.3)	4.6 (3.3-5.9)	3 (0.9-5.1)
Egypt	85.5 (81.5-89.9)	46.6 (43.6-49.6)	28.8 (25.6-32.5)	20.3 (14.3-29.1)	6 (5.4-6.7)	4.9 (3.7-6)	3.9 (0.4-7.4)
El Salvador	59.6 (54.5-65)	32.8 (29.5-36.3)	19.2 (15.5-23.6)	13.3 (8.5-20.8)	6 (5-7.1)	5.4 (3.5-7.2)	4.1 (0.3-7.8)

	Under-five mortality rate (per 1,000 live births)				Average annual rate of reduction in under-five mortality rate (%)		
	1990	2000	2010	2019	1990-1999	2000-2009	2010-2019
Equatorial Guinea	177.7 (156.7-200.6)	156.3 (139.7-178.3)	111.7 (88.1-139.2)	81.8 (49.1-134.6)	1.1 (-0.6-2.6)	3.3 (1.3-5.8)	3.5 (-1-8)
Eritrea	152.7 (140.3-166.6)	85.4 (78.1-93.1)	55.2 (46.3-65.8)	40.5 (24.5-67)	5.9 (5.1-6.7)	4.4 (3-5.8)	3.5 (-1.3-8.2)
Estonia	17.7 (17-18.3)	10.9 (10.4-11.5)	4.6 (4.3-4.9)	2.4 (2-2.8)	4.4 (3.7-5.1)	8.7 (7.8-9.7)	7.2 (5.1-9.1)
Eswatini	67.4 (59.7-75.6)	109.7 (99.2-121.2)	86.6 (73.9-101.4)	49.4 (32.7-74.2)	-5 (-6.2–3.8)	1.4 (-0.2-2.9)	6.3 (2.5-10.1)
Ethiopia	199.9 (185.9-215.2)	140.3 (130.9-150.7)	82 (75.4-89.2)	50.7 (41.6-61.7)	3.5 (2.9-4)	5.4 (4.6-6.2)	5.3 (3.3-7.3)
Fiji	28.9 (24.5-34.1)	22.7 (21.6-23.8)	23.7 (22.8-24.6)	25.7 (23-28.8)	2.5 (0.9-4.1)	-0.5 (-1.2-0.2)	-0.9 (-2.3-0.4)
Finland	6.7 (6.5-7)	4.3 (4.1-4.5)	3 (2.9-3.2)	2.4 (2.2-2.6)	4.7 (4.1-5.4)	3.4 (2.7-4.1)	2.6 (1.3-3.8)
France	9 (8.8-9.2)	5.4 (5.3-5.5)	4.3 (4.2-4.4)	4.5 (4.2-4.7)	5.5 (5.1-5.8)	2.5 (2.1-2.8)	-0.5 (-1.1-0.1)
Gabon	91.7 (78.7-106.5)	84 (72.5-98.8)	61.6 (50.2-74.4)	42.5 (26.6-67.8)	0.8 (-0.7-2)	3 (1.1-5.4)	4.1 (-0.2-8.3)
Gambia	167.4 (150.7-186.4)	112.5 (102.4-123.7)	72.9 (65-81.4)	51.7 (43.3-61.4)	3.9 (3-4.9)	4.4 (3.4-5.5)	3.8 (2.2-5.4)
Georgia	47.7 (42.9-53.3)	36.6 (32.3-42.1)	14.2 (13.3-15.2)	9.6 (8.2-11.3)	2.3 (0.8-3.7)	9.5 (7.9-11.2)	4.3 (2.3-6.4)
Germany	8.5 (8.4-8.7)	5.4 (5.3-5.5)	4.2 (4.1-4.2)	3.8 (3.6-4)	4.8 (4.6-5.1)	2.5 (2.3-2.8)	1 (0.5-1.6)
Ghana	127.2 (120.3-134.4)	99.4 (93.7-105)	69.1 (64.2-74.3)	46.2 (37.1-57)	2.3 (1.6-3)	3.5 (2.8-4.3)	4.5 (2.2-6.9)
Greece	10.4 (10.1-10.7)	6.4 (6.2-6.6)	3.9 (3.8-4.1)	3.8 (3.3-4.2)	4.6 (4.1-5.1)	5.4 (4.9-6)	0.4 (-1-1.9)
Grenada	22.4 (20.8-24.1)	15.5 (14.1-16.9)	15.1 (13.7-16.6)	16.5 (13.5-20.2)	3.9 (2.7-5.1)	0.4 (-0.9-1.8)	-1 (-3.5-1.5)
Guatemala	80.1 (75.3-85.1)	51.9 (48.2-55.9)	34.7 (31.1-38.7)	24.5 (19-31.4)	4.4 (3.7-5.1)	4 (3.2-4.8)	3.8 (1.6-6.1)
Guinea	231.2 (215.8-247.3)	164.3 (153.1-176)	118.1 (108.2-129.3)	98.8 (82.7-118.7)	3.3 (2.8-3.9)	3.4 (2.7-4.2)	2 (0.2-3.6)
Guinea-Bissau	221.2 (196.9-248.1)	173 (156-192.7)	113.1 (93.7-137)	78.5 (49.4-120)	2.4 (1.3-3.5)	4.2 (2.5-6)	4.1 (0.4-8.1)
Guyana	60.1 (54.4-66.7)	46.3 (41.8-51.5)	37.3 (29.8-46.7)	29.3 (18.2-47.5)	2.6 (1.5-3.8)	2.2 (0.1-4.3)	2.7 (-1.2-6.6)
Haiti	144.6 (135.2-154.8)	103.7 (95.5-111.9)	207 (186.2-231.2)	62.8 (47.6-84.1)	3.3 (2.7-4.1)	2.7 (1.6-3.6)	13.3 (10.5-15.8)
Honduras	58 (53.5-62.8)	37 (33.9-40.5)	23.3 (20.3-26.9)	16.8 (11.2-25.2)	4.5 (3.8-5.1)	4.6 (3.6-5.8)	3.6 (-0.4-7.7)
Hungary	17.2 (16.8-17.6)	10.1 (9.9-10.4)	6 (5.8-6.2)	3.7 (3.2-4.2)	5.3 (4.8-5.7)	5.5 (4.9-6)	5.5 (4-6.9)
Iceland	6.5 (5.9-7.1)	4 (3.6-4.5)	2.6 (2.3-3)	2 (1.5-2.6)	4.7 (3.3-6.1)	4.4 (2.8-6)	3.2 (0-6.4)
India	126.2 (122-130.8)	91.8 (88.3-95.3)	58.2 (55.5-61.2)	34.3 (30.7-37.9)	3.1 (2.7-3.5)	4.5 (3.9-5)	5.9 (4.7-7.3)
Indonesia	84 (80.1-88.2)	52.2 (49.6-55)	33.9 (31.4-36.7)	23.9 (20.1-28.9)	4.8 (4.3-5.3)	4.3 (3.7-4.9)	3.9 (2.1-5.5)
Iran (Islamic Republic of)	56.1 (51.8-61)	34.3 (31.1-38.1)	19.7 (16.7-23.3)	13.9 (8.6-22.7)	4.8 (3.8-5.8)	5.6 (3.8-7.5)	3.9 (-0.7-8.4)
Iraq	53.5 (48.9-58.4)	44 (40.2-48.2)	34.6 (30.6-39.3)	25.9 (20-33.2)	1.9 (1.1-2.7)	2.3 (1.3-3.3)	3.2 (1-5.7)
Ireland	9.2 (8.8-9.5)	7.1 (6.9-7.5)	4.2 (4-4.3)	3.3 (2.8-3.8)	2.5 (2-3.1)	5.7 (5.1-6.3)	2.7 (0.9-4.6)
Israel	11.6 (11.4-11.9)	6.9 (6.7-7.1)	4.6 (4.4-4.7)	3.7 (3.5-3.9)	5.4 (5-5.8)	4.2 (3.8-4.6)	2.4 (1.7-3.1)
Italy	9.7	5.6	4	3.1	5.5	3.5	2.6

	Under-five mortality rate (per 1,000 live births)				Average annual rate of reduction in under-five mortality rate (%)		
	1990	2000	2010	2019	1990-1999	2000-2009	2010-2019
	(9.5-9.9)	(5.5-5.7)	(3.9-4)	(2.9-3.4)	(5.2-5.8)	(3.2-3.8)	(1.7-3.5)
Jamaica	30.3	22	18.1	13.9	3.2	1.9	3
	(25.9-35.9)	(19-25.5)	(13.9-24)	(8-24.2)	(1.8-5)	(-0.6-4)	(-1.7-7.8)
Japan	6.3	4.5	3.2	2.5	3.2	3.4	2.9
	(6.2-6.4)	(4.4-4.6)	(3.1-3.3)	(2.3-2.6)	(2.9-3.5)	(3.1-3.7)	(2.3-3.5)
Jordan	36	26.9	20.2	15.6	2.9	2.8	2.9
	(33.6-38.4)	(24.9-29.1)	(17.8-23)	(11.7-20.9)	(2.1-3.7)	(1.7-4)	(0.4-5.3)
Kazakhstan	51.8	42.2	20.4	10.5	1.6	7	7.4
	(47-57)	(38.5-46.1)	(19.9-20.9)	(10.1-10.8)	(0.7-2.5)	(6-8)	(7-7.9)
Kenya	101.5	98.5	57.9	43.2	-0.1	5.5	3.3
	(95.2-108.3)	(91.6-106.3)	(52.2-64.2)	(31.9-59.3)	(-0.9-0.7)	(4.5-6.5)	(-0.1-6.3)
Kiribati	95.5	71	64.5	50.9	3	0.9	2.6
	(82.4-110.3)	(62.6-80.2)	(50.8-81.9)	(29.6-86.8)	(1.4-4.5)	(-1.2-3.1)	(-2-7.4)
Kuwait	17.5	12.4	10.4	7.9	3.6	1.6	3
	(16.9-18.1)	(12-12.7)	(10.1-10.7)	(7.2-8.8)	(3.1-4.1)	(1.1-2.1)	(1.8-4.2)
Kyrgyzstan	65	49.7	29.5	18.3	2.4	5	5.3
	(57.6-73.4)	(44.3-54.3)	(28.7-30.3)	(17.5-19.4)	(1.1-3.8)	(3.6-6.1)	(4.6-5.9)
Lao People's Democratic Republic	153	106.4	68	45.5	3.6	4.4	4.5
	(140.6-166.4)	(97.8-116.3)	(59.7-77.2)	(33.7-59.7)	(2.8-4.4)	(3.4-5.6)	(2.1-7.1)
Latvia	17	14.2	7.8	3.6	1.1	5.9	8.6
	(16.5-17.6)	(13.5-14.9)	(7.4-8.3)	(2.9-4.6)	(0.5-1.8)	(5.1-6.8)	(5.9-11.2)
Lebanon	32.1	19.8	10.3	7.2	4.8	6.7	3.9
	(28.7-35.9)	(16.7-23.5)	(6.5-15.4)	(3.6-13.9)	(3-6.5)	(3.4-11.1)	(-0.8-8.9)
Lesotho	85.2	106.8	97.9	86.4	-2.2	0.3	1.4
	(77.2-94.6)	(97.9-116.3)	(86.9-110.2)	(59.4-124.3)	(-3.3–1)	(-1-1.6)	(-2.2-5.3)
Liberia	262.6	188	100.8	84.6	3	6.6	1.9
	(241.5-286.6)	(174.4-203.3)	(91.7-110.8)	(66.3-108.4)	(2.1-3.8)	(5.6-7.6)	(-0.8-4.6)
Libya	41.7	28.1	16.6	11.5	4.1	5.2	4.1
	(35.6-48.8)	(26.5-29.8)	(14.8-18.7)	(7.2-18.4)	(2.5-5.6)	(4.1-6.3)	(-0.5-8.7)
Lithuania	15	10.7	6.1	3.7	3.3	5.3	5.6
	(14.4-15.5)	(10.2-11.3)	(5.7-6.5)	(3.2-4.2)	(2.6-3.9)	(4.5-6.1)	(4-7.3)
Luxembourg	8.7	4.7	3	2.8	6.3	4.6	1
	(8-9.4)	(4.2-5.2)	(2.7-3.4)	(2.1-3.6)	(5-7.6)	(3.2-6.1)	(-1.9-3.9)
Madagascar	157.3	106.5	68.5	50.6	3.8	4.5	3.4
	(146.9-168.8)	(98.7-115.1)	(61.1-76.4)	(40.3-63.9)	(3-4.6)	(3.5-5.6)	(1.1-5.5)
Malawi	242.8	172.6	84.9	41.6	3.1	7.3	7.9
	(229-257.1)	(162.6-183.5)	(77.4-93.4)	(27-60.9)	(2.6-3.6)	(6.4-8.1)	(3.6-12.9)
Malaysia	16.6	10.2	8.1	8.6	4.6	2.6	-0.7
	(16.1-17)	(10-10.4)	(7.9-8.2)	(8.1-9)	(4.2-4.9)	(2.3-2.9)	(-1.3–0.1)
Maldives	85.8	38.9	13.9	7.6	7.6	10.7	6.7
	(78.4-94.3)	(35.3-42.8)	(13.1-14.7)	(6.7-8.7)	(6.6-8.6)	(9.5-11.8)	(5-8.4)
Mali	230	187.2	130.3	94	1.9	3.6	3.6
	(216.4-244.2)	(176-199.6)	(120.2-141.7)	(76-116.5)	(1.4-2.5)	(2.9-4.4)	(1.5-5.7)
Malta	11.4	7.6	6.8	7	4.1	1.2	-0.3
	(10.6-12.2)	(6.9-8.3)	(6.2-7.5)	(5.6-8.8)	(2.9-5.2)	(-0.1-2.5)	(-3-2.4)
Marshall Islands	49.3	40.9	38.7	31.8	2.1	0.5	2.2
	(42.1-57.5)	(35.3-47.6)	(31.8-46.9)	(19.8-50.6)	(0.3-3.8)	(-1.7-2.8)	(-2-6.4)
Mauritania	117.7	112.7	96.6	72.9	0.4	1.4	3.1
	(105.9-130.5)	(100.1-127.1)	(66.3-140.4)	(37.5-140)	(-1-1.8)	(-1.9-4.8)	(-1.9-8.2)
Mauritius	23	18.8	14.5	16	1.4	2.8	-1.1
	(22-24.1)	(17.8-19.8)	(13.6-15.5)	(14.5-17.8)	(0.6-2.1)	(1.9-3.7)	(-2.5-0.2)
Mexico	45.3	28.2	19.1	14.2	4.7	3.9	3.3
	(42.2-48.7)	(26.2-30.3)	(18.1-20.3)	(12.9-15.6)	(4.1-5.5)	(3.3-4.6)	(2.1-4.5)
Micronesia (Federated States of)	54.8	52.6	39.4	29.4	0.3	2.9	3.3
	(44-68.5)	(35.9-77.4)	(20.2-76.3)	(12-72.5)	(-2.6-3)	(-2-7.7)	(-2-8.4)
Monaco	7.8	5.2	4	3.1	4.2	2.6	2.8
	(7-8.6)	(4.9-5.5)	(3.7-4.3)	(2.1-4.5)	(2.8-5.5)	(1.6-3.6)	(-1.1-6.6)
Mongolia	108.1	65.5	30.2	15.6	4.8	7.8	7.3

	Under-five mortality rate (per 1,000 live births)				Average annual rate of reduction in under-five mortality rate (%)		
	1990	2000	2010	2019	1990-1999	2000-2009	2010-2019
	(99.6-117.6)	(60-71.2)	(29.3-31.1)	(15.1-16.2)	(3.8-5.8)	(6.8-8.7)	(6.8-7.8)
Montenegro	16.5	14.2	6.6	2.3	1.4	7.4	11.5
	(15.7-17.3)	(13.5-15)	(6.1-7.1)	(1.9-2.9)	(0.5-2.2)	(6.4-8.4)	(9.1-14)
Morocco	79.1	49.4	31.8	21.4	4.7	4.4	4.4
	(73.7-84.9)	(44.9-54.2)	(28-35.9)	(16.1-28.2)	(3.9-5.6)	(3.2-5.6)	(1.9-6.9)
Mozambique	243.1	169.7	104.9	74.2	3.4	5.1	3.8
	(224.4-264.4)	(156.9-183.7)	(95-116)	(52.2-108.9)	(2.7-4)	(4.2-5.9)	(0-7.3)
Myanmar	114.6	89	63.4	44.7	2.5	3.3	3.9
	(105.1-125.6)	(82.1-96.6)	(55-72.5)	(31.1-62.5)	(1.6-3.4)	(2.2-4.6)	(0.8-7.2)
Namibia	72.3	75.4	49.7	42.4	-0.5	4.2	1.8
	(65.8-79.7)	(67.9-84.4)	(40.4-62)	(24.8-77)	(-1.8-0.9)	(2-6.3)	(-3.5-6.5)
Nauru	60.3	41.5	38.9	30.9	3.8	0.6	2.6
	(36.2-99.2)	(35.5-48.5)	(28-52.4)	(17-55.6)	(-1-8.7)	(-2.4-4)	(-2.2-7.2)
Nepal	139.8	81	46.6	30.8	5.4	5.5	4.6
	(131.1-148.9)	(75.8-86.8)	(42.2-51.6)	(23.6-40.7)	(4.8-6)	(4.7-6.4)	(1.9-7.2)
Netherlands	8.3	6.2	4.4	4	3.1	3.4	1
	(8.2-8.5)	(6.1-6.3)	(4.3-4.5)	(3.9-4.2)	(2.8-3.4)	(3-3.8)	(0.4-1.6)
New Zealand	11.2	7.4	6.1	4.7	4.3	1.9	2.9
	(10.9-11.5)	(7.2-7.6)	(6-6.4)	(4.1-5.4)	(3.8-4.7)	(1.4-2.4)	(1.3-4.7)
Nicaragua	66.5	38.5	23.9	16.6	5.5	4.8	4
	(61.7-71.5)	(34.8-42.3)	(21.3-26.1)	(14.5-19.5)	(4.5-6.5)	(3.8-6)	(1.8-5.9)
Niger	328.6	224.8	122.2	80.4	3.8	6	4.7
	(307.5-351.2)	(208.6-242.5)	(104.9-141.1)	(51.2-125)	(3.1-4.4)	(4.7-7.5)	(0.4-8.9)
Nigeria	209.5	183.1	136	117.2	1.2	3.1	1.7
	(195.7-223.4)	(172.1-194.6)	(124.8-147.9)	(92.2-152.5)	(0.6-1.7)	(2.3-3.8)	(-1.1-4.1)
Niue	13.4	23.7	29.9	23.2	-5.6	-2.8	2.8
	(8.4-21.5)	(15.8-35.8)	(15.3-60.1)	(9.6-57.7)	(-11–0.5)	(-7.7-2.1)	(-2.3-7.9)
Norway	8.7	4.9	3.3	2.4	6.1	3.9	3.2
	(8.4-9)	(4.7-5.1)	(3.1-3.4)	(2.2-2.7)	(5.6-6.7)	(3.2-4.6)	(2-4.4)
Oman	38.7	16.4	11.7	11.4	8.7	3.6	0.3
	(33.9-44.2)	(14.4-18.8)	(11.4-12.1)	(10.3-12.7)	(7.4-10.1)	(2.2-5.1)	(-0.9-1.5)
Pakistan	139.5	107.5	87.1	67.2	2.6	2.1	2.9
	(134.4-144.9)	(102.8-112.2)	(81.8-92.7)	(57.6-78.4)	(2.1-3.1)	(1.4-2.7)	(1.4-4.4)
Palau	35.4	28.8	23	17.3	2	2.2	3.1
	(30.3-41.4)	(24.6-33.8)	(15.3-34.8)	(8.8-33.6)	(-0.2-4.3)	(-1.7-6)	(-1.7-8)
Panama	30.7	25.9	19.9	14.9	1.7	2.6	3.2
	(27-34.4)	(22.3-29.8)	(14.3-27.6)	(8.1-27.2)	(0.1-3.2)	(0-5.2)	(-1.7-8.1)
Papua New Guinea	84.7	70.6	57.3	44.8	1.8	2	2.7
	(77.1-92.7)	(63.5-78.8)	(48.8-67.4)	(32.2-61.5)	(0.8-2.7)	(0.7-3.3)	(0-5.6)
Paraguay	45.3	33.7	26	19.4	3	2.5	3.2
	(40.8-50.4)	(28.2-40.4)	(17.6-39.6)	(10.3-38.5)	(1.5-4.5)	(-0.9-5.5)	(-1.7-8.1)
Peru	80.3	38.5	19.9	13.2	7.3	6.7	4.6
	(76.3-84.6)	(36.3-40.8)	(18.6-21.3)	(10.7-16.5)	(6.6-7.9)	(5.9-7.5)	(2.2-6.7)
Philippines	56.6	37.7	31.7	27.3	4.3	1.8	1.7
	(52.8-60.7)	(35.1-40.6)	(28.8-35)	(20.7-36.1)	(3.5-5.1)	(0.7-2.8)	(-1.2-4.6)
Poland	17.4	9.3	6	4.4	6.2	4.3	3.4
	(17-17.8)	(9.1-9.5)	(5.8-6.1)	(4.2-4.5)	(5.9-6.5)	(4-4.6)	(2.9-3.9)
Portugal	14.7	7.2	3.8	3.7	7.2	6.8	0.4
	(14.4-15.1)	(7-7.4)	(3.7-4)	(3.4-3.9)	(6.8-7.6)	(6.3-7.3)	(-0.5-1.3)
Qatar	20.8	12.5	9.1	6.5	5.3	3.2	3.7
	(19.5-22.2)	(11.9-13.1)	(8.7-9.5)	(5.7-7.4)	(4.3-6.2)	(2.5-4)	(2.1-5.3)
Republic of Korea	15.4	7.5	4.1	3.2	7.3	6.2	2.8
	(14.6-16.2)	(7.3-7.8)	(4-4.2)	(3-3.4)	(6.6-8)	(5.8-6.6)	(2.2-3.5)
Republic of Moldova	33.2	31.4	17.1	14.4	-0.3	6.6	1.9
	(28.5-38.5)	(26-37.3)	(14.9-20.5)	(10.6-19.2)	(-2.3-1.6)	(4-8.6)	(-1.7-5.8)
Republic of North Macedonia	35.7	15.8	10.1	6.1	8.3	4.3	5.5
	(34.6-36.9)	(15.1-16.6)	(9.5-10.7)	(5.4-6.9)	(7.7-8.9)	(3.5-5.1)	(4-7.1)
Romania	30.9	21.4	12.4	7	3.3	5.5	6.4
	(30.1-31.7)	(20.8-22)	(12-12.7)	(6.7-7.3)	(2.9-3.7)	(5.1-5.9)	(5.8-6.9)

	Under-five mortality rate (per 1,000 live births)				Average annual rate of reduction in under-five mortality rate (%)		
	1990	2000	2010	2019	1990-1999	2000-2009	2010-2019
Russian Federation	21.6 (21.2-22)	19.3 (18.9-19.7)	10.4 (10.2-10.6)	5.8 (5.6-6)	0.8 (0.5-1.1)	6.5 (6.2-6.8)	6.5 (6.1-6.9)
Rwanda	149.8 (140.6-159.8)	178.7 (165.1-193.9)	63.7 (55.8-72.6)	34.3 (19.7-60.2)	-3 (-4–2)	10.3 (9.1-11.5)	6.9 (1.2-12.5)
Saint Kitts and Nevis	30.5 (27.8-33.4)	23.8 (21.3-26.6)	17.5 (15.3-20)	15.4 (11-21.6)	2.3 (0.9-3.7)	3.3 (1.7-4.9)	1.5 (-2.1-5)
Saint Lucia	21.8 (20.8-22.9)	18.5 (17.5-19.6)	19.5 (18.2-20.8)	22.3 (19.5-25.7)	1.8 (1.2-2.4)	-0.4 (-1.1-0.3)	-1.5 (-2.9–0.1)
Saint Vincent and the Grenadines	23.8 (22.2-25.4)	22.2 (20.5-24)	19.8 (18.1-21.6)	14.6 (11.5-18.4)	0.7 (-0.4-1.8)	1 (-0.2-2.2)	3.4 (0.7-6.1)
Samoa	29.8 (26.1-34.1)	20.7 (18-23.5)	18.4 (15.5-21.4)	15 (10.3-21.8)	3.6 (2.2-5.1)	-1.6 (-3.3-0)	2.3 (-1.2-5.5)
San Marino	13.8 (10.6-17.9)	6 (4.5-8.2)	2.7 (1.8-4.2)	1.7 (0.9-3.3)	8.3 (4.9-11.7)	8 (4.3-11.6)	5.3 (0.8-9.8)
Sao Tome and Principe	107.6 (95.1-121.9)	84 (73.1-96.5)	45 (35.5-56.4)	29.8 (18.4-48.6)	2.2 (0.6-3.7)	6.3 (4-8.5)	4.6 (0.5-8.7)
Saudi Arabia	44.4 (36.9-53)	22 (19.6-24.6)	11.9 (10.2-15.1)	6.6 (5-9)	7.1 (5.4-9.1)	6 (3.3-7.6)	6.5 (3.6-10.5)
Senegal	138.6 (131.7-145.8)	129.1 (121.6-137.5)	66.3 (61.3-71.7)	45.3 (35.2-58.2)	0.4 (-0.3-1)	6.8 (5.9-7.8)	4.2 (1.4-7.1)
Serbia	27.9 (27.2-28.6)	12.7 (12.3-13.1)	7.6 (7.3-7.9)	5.3 (4.3-6.6)	8.3 (7.9-8.8)	5.4 (4.9-6)	4 (1.6-6.4)
Seychelles	16.3 (14.8-17.9)	13.8 (12.4-15.2)	14.1 (12.7-15.6)	14.2 (10.7-18.9)	1.9 (0.5-3.2)	-0.2 (-1.6-1.2)	-0.1 (-3.2-3)
Sierra Leone	259.8 (239.6-281)	227.7 (212.2-244.1)	160.5 (147.9-173.8)	109.2 (90.5-131.6)	1.2 (0.4-2)	3.4 (2.7-4.1)	4.3 (2.4-6.1)
Singapore	7.7 (7.4-8)	3.9 (3.6-4.1)	2.8 (2.6-3)	2.5 (2.2-2.9)	6.9 (6.1-7.7)	3.4 (2.4-4.4)	1.2 (-0.4-2.8)
Slovakia	14.7 (14.5-15)	9.8 (9.5-10)	7 (6.8-7.2)	5.8 (5.5-6.1)	4.1 (3.8-4.5)	3.4 (3-3.8)	2.2 (1.4-2.9)
Slovenia	10.4 (10-10.7)	5.5 (5.2-5.8)	3.2 (3-3.4)	2.1 (1.8-2.4)	6.4 (5.8-7.1)	5.4 (4.6-6.1)	4.8 (3.1-6.3)
Solomon Islands	38.4 (33.5-43.8)	30.4 (27.2-34)	25.9 (22-30.5)	19.7 (13-29.1)	2.4 (1.1-3.7)	1.5 (-0.1-3.1)	3.1 (-0.5-7)
Somalia	178.2 (148.4-217.5)	171.1 (135.4-221.2)	156.1 (109.8-227.3)	117 (59.8-235.3)	0.4 (-0.8-1.6)	0.7 (-1-2.3)	3.2 (-2-8.4)
South Africa	57.3 (51.1-64.1)	71.1 (66.6-76)	51.2 (48.5-53.9)	34.5 (30.5-38.8)	-2.1 (-3.3–1)	1.9 (1-2.9)	4.4 (2.9-5.9)
South Sudan	250.2 (207.3-294.3)	181.2 (154-212)	106.8 (75.9-142.7)	96.2 (38.5-193.6)	3.1 (1.3-4.7)	5.3 (2.5-8.7)	1.2 (-4.1-8.3)
Spain	9.1 (9-9.3)	5.4 (5.3-5.5)	3.8 (3.8-3.9)	3.1 (2.9-3.4)	5.4 (5.1-5.7)	3.4 (3.1-3.7)	2.4 (1.4-3.3)
Sri Lanka	21.9 (21.5-22.3)	16.5 (16.2-16.9)	11.6 (11.3-11.9)	7.1 (5.8-8.6)	2.7 (2.4-3)	3.6 (3.3-3.9)	5.5 (3.2-7.7)
State of Palestine	44.5 (41-48.2)	30 (27.6-32.5)	23.2 (19.9-27.1)	19.4 (13.9-27.8)	4 (3.2-4.9)	2.6 (1.2-4)	1.9 (-1.1-4.7)
Sudan	130.6 (121.2-140.9)	103.5 (95.2-112.5)	75.4 (66.4-85.5)	58.4 (42.7-78.4)	2.2 (1.5-3)	3.2 (2.2-4.2)	2.8 (0.1-5.7)
Suriname	45 (38.3-52.9)	31.2 (26.4-36.4)	23.1 (18.5-28.7)	18 (12.2-27.5)	3.7 (2.1-5.5)	3.1 (1-4.9)	2.8 (-0.9-5.7)
Sweden	7 (6.8-7.2)	4.1 (4-4.3)	3.1 (2.9-3.2)	2.6 (2.4-2.8)	5.8 (5.3-6.4)	3 (2.4-3.6)	1.9 (1-2.8)
Switzerland	8.2 (8-8.4)	5.6 (5.5-5.8)	4.6 (4.4-4.7)	4 (3.6-4.5)	4 (3.5-4.5)	2.2 (1.7-2.8)	1.4 (0-2.8)
Syrian Arab Republic	36.6 (33.1-40.3)	23.1 (20.7-25.7)	19 (15.7-22.2)	21.5 (12.3-28.3)	4.7 (3.6-5.8)	2.2 (0.5-4.1)	-1.4 (-4.4-3.2)
Tajikistan	101.9 (92.9-111.8)	83.6 (74.7-93.3)	42.9 (37.3-49.5)	33.8 (21.9-53.3)	1.4 (0.3-2.5)	7 (5.4-8.6)	2.7 (-2.6-7.6)

	Under-five mortality rate (per 1,000 live births)				Average annual rate of reduction in under-five mortality rate (%)		
	1990	2000	2010	2019	1990-1999	2000-2009	2010-2019
Thailand	36.9<br>(34.6-39.2)	22<br>(19.7-24)	13.6<br>(12-15.3)	9<br>(7.7-11.5)	5.2<br>(4.4-6.3)	4.8<br>(3.7-5.8)	4.6<br>(2.3-5.9)
Timor-Leste	174.7<br>(157.4-194)	108.1<br>(98.1-119.2)	61.8<br>(49.7-76.2)	44.2<br>(26.4-74.3)	4.8<br>(3.7-5.8)	5.7<br>(4-7.6)	3.7<br>(-1-8.4)
Togo	144.5<br>(134.2-155.6)	117.9<br>(109.5-126.9)	88<br>(79.8-96.8)	66.9<br>(53.8-82.7)	2<br>(1.2-2.6)	2.9<br>(2.2-3.8)	3<br>(1.1-5)
Tonga	22.2<br>(18.7-26.5)	18<br>(15.3-21.2)	16.8<br>(14.1-20)	16.6<br>(12.2-22.3)	2.2<br>(0.5-3.8)	0.7<br>(-1.1-2.7)	0.1<br>(-2.9-3.2)
Trinidad and Tobago	30.4<br>(25.3-35.6)	28.2<br>(21.2-38.9)	22.9<br>(13.2-44.1)	17.5<br>(7.7-41.2)	0.8<br>(-2.6-3.2)	1.9<br>(-2.5-5.8)	3<br>(-1.9-8.4)
Tunisia	55.3<br>(48.6-62.7)	29.9<br>(26.1-34.1)	18.4<br>(17.6-19.2)	16.9<br>(15.5-18.3)	6<br>(4.6-7.4)	5.1<br>(3.5-6.7)	1<br>(-0.2-2.1)
Turkey	73.9<br>(69-79.1)	38.6<br>(35.6-42)	18.2<br>(16.7-19.6)	10<br>(8.9-11.4)	6.4<br>(5.5-7.2)	7.5<br>(6.7-8.6)	6.6<br>(4.9-8.1)
Turkmenistan	79.8<br>(69.8-91.1)	69.8<br>(60.6-80.6)	42.8<br>(33.5-54.1)	42<br>(30-58.5)	0.9<br>(-0.6-2.3)	5.2<br>(2.7-8)	0.2<br>(-4.1-4.2)
Tuvalu	53.1<br>(44.1-64.2)	41.1<br>(36.9-45.5)	31.8<br>(23.2-43.7)	23.9<br>(13.2-44.6)	2.5<br>(0.4-4.7)	2.5<br>(-0.6-5.6)	3.2<br>(-1.8-8)
Uganda	182.1<br>(171.5-193.3)	146.3<br>(137.6-155.4)	77<br>(71.2-83)	45.8<br>(34.8-60)	2<br>(1.4-2.6)	6.4<br>(5.6-7.2)	5.8<br>(2.8-8.7)
Ukraine	19.4<br>(17.5-22.1)	18.3<br>(16.8-20.5)	11.7<br>(11.4-12.1)	8.4<br>(8-8.8)	0.2<br>(-0.9-1.2)	4.4<br>(3.4-5.7)	3.7<br>(3-4.4)
United Arab Emirates	16.6<br>(14-19.4)	11.2<br>(10.6-11.9)	8.5<br>(8.2-8.7)	7.5<br>(6.4-8.7)	4<br>(2.5-5.6)	2.8<br>(2.1-3.6)	1.4<br>(-0.4-3.2)
United Kingdom	9.3<br>(9.1-9.5)	6.5<br>(6.4-6.7)	5.2<br>(5.1-5.3)	4.3<br>(4.1-4.5)	3.7<br>(3.4-4)	2.2<br>(1.9-2.5)	2.1<br>(1.5-2.7)
United Republic of Tanzania	165.1<br>(155.6-175.5)	128.8<br>(120.9-137.5)	71.9<br>(65.4-79.4)	50.3<br>(37.8-68.6)	2.1<br>(1.4-2.8)	5.9<br>(5-6.9)	4<br>(0.8-7)
United States of America	11.2<br>(11-11.4)	8.4<br>(8.3-8.6)	7.3<br>(7.2-7.5)	6.5<br>(6.1-6.9)	3<br>(2.7-3.3)	1.4<br>(1.1-1.6)	1.4<br>(0.6-2.1)
Uruguay	22.9<br>(22.5-23.4)	17<br>(16.6-17.4)	10.7<br>(10.3-11)	7.1<br>(6.6-7.5)	3<br>(2.6-3.3)	4.6<br>(4.1-5)	4.6<br>(3.8-5.4)
Uzbekistan	72.3<br>(63.8-81.9)	62<br>(54-71.8)	33.3<br>(27.1-40.4)	17.4<br>(14.2-21.4)	1.3<br>(-0.3-2.8)	6.1<br>(4.2-8.3)	7.2<br>(4-10.3)
Vanuatu	35.3<br>(29.5-42.1)	28.3<br>(24.1-33.4)	29.6<br>(23.4-37.7)	25.9<br>(15.7-42.7)	2.4<br>(0.9-3.9)	-0.4<br>(-2.6-1.8)	1.5<br>(-2.7-5.7)
Venezuela (Bolivarian Republic of)	29.5<br>(28.7-30.4)	21.6<br>(21.1-22)	17.1<br>(16.7-17.4)	24.2<br>(20-29.6)	0.8<br>(0.4-1.1)	2.6<br>(2.3-2.9)	-3.9<br>(-6.1–1.7)
Viet Nam	51.3<br>(47.3-55.8)	29.7<br>(25.1-34.6)	22.9<br>(20.7-25)	19.9<br>(16.1-25.4)	5.5<br>(3.8-7.4)	2.8<br>(1.3-4)	1.5<br>(-1.7-4.1)
Yemen	125.8<br>(118-134.5)	94.8<br>(87.1-103.1)	56<br>(48.2-64.8)	58.4<br>(35-94.7)	2.6<br>(1.9-3.4)	5.5<br>(4.1-6.9)	-0.5<br>(-4.9-4.4)
Zambia	179.5<br>(168.5-191.2)	152.1<br>(142.2-163)	79.6<br>(73.2-86.4)	61.7<br>(48.8-77.6)	1.4<br>(0.8-2)	6.6<br>(5.8-7.4)	2.8<br>(0.3-5.4)
Zimbabwe	76.6<br>(69.9-83.5)	92.7<br>(84.1-101.6)	87.6<br>(80.3-95.7)	54.6<br>(41.1-71.7)	-2.3<br>(-3.5–1.1)	0.1<br>(-1.2-1.2)	5.3<br>(2.2-8.4)

3.2. Table A.2 Levels and trends in NMR, by country, 1990-2019

Note: Numbers in brackets are 90% uncertainty intervals.

	Neonatal mortality rate (per 1,000 live births)				Average annual rate of reduction in neonatal mortality rate (%)		
	**1990**	**2000**	**2010**	**2019**	**1990-1999**	**2000-2009**	**2010-2019**
Afghanistan	74.3 (66.4-83.1)	60.6 (55.3-66.7)	47.2 (42.6-52.3)	35.9 (27.8-45.2)	2 (0.8-3.3)	2.4 (1.3-3.6)	3.1 (0.8-5.6)
Albania	12.6 (9.8-15.8)	12.2 (9.9-14.8)	6.7 (5.9-7.4)	7.5 (6.9-8.2)	0.1 (-3.2-3.3)	5.6 (3.2-7.9)	-1.3 (-2.7-0)
Algeria	23.2 (20.6-26)	21 (19-23.1)	17.5 (16.9-18.2)	16.3 (15.6-17)	1.1 (-0.6-2.7)	1.6 (0.4-2.7)	0.8 (0.3-1.3)
Andorra	7 (1.7-27.5)	3.9 (1.9-7.9)	2.2 (1-4.8)	1.4 (0.4-5.5)	5.8 (-4.5-16.2)	5.9 (-3.4-15.9)	4.8 (-4.8-14)
Angola	53.3 (43.7-65.8)	50.3 (41.9-60.4)	36.2 (24.6-51.4)	27.6 (12.2-53.7)	0.5 (-2.2-3.2)	3.2 (-0.4-7.2)	3 (-2.1-9.2)
Antigua and Barbuda	8.1 (6.9-9.4)	9.6 (8.5-10.9)	5.9 (5-6.8)	3.6 (2.5-5.3)	-2.1 (-4.1-0)	4.7 (2.7-6.7)	5.3 (1.3-9.5)
Argentina	14.9 (14.4-15.4)	10.9 (10.6-11.3)	7.7 (7.4-7.9)	6.1 (5.7-6.5)	3.5 (3.1-3.8)	3.8 (3.4-4.1)	2.6 (1.8-3.3)
Armenia	22.5 (19.5-26.2)	16.4 (14.3-18.8)	10.1 (8-12.5)	6.4 (4.3-9.1)	3.1 (1.4-4.9)	4.9 (2.7-7.2)	5.1 (2.3-8.2)
Australia	4.6 (4.5-4.8)	3.5 (3.4-3.6)	2.7 (2.6-2.8)	2.3 (2.1-2.4)	3 (2.5-3.4)	2.4 (2-2.8)	2 (1.2-2.8)
Austria	4.6 (4.4-4.8)	3.1 (2.9-3.3)	2.5 (2.4-2.7)	2.1 (1.7-2.4)	4.4 (3.7-5.1)	2.1 (1.3-2.9)	2.2 (0.2-4.1)
Azerbaijan	32.9 (27.3-39.1)	33.5 (28.3-40.1)	19.9 (16-24.8)	10.9 (6.6-17.5)	-0.6 (-3.2-1.7)	5.1 (2.4-7.9)	6.7 (1.6-12.1)
Bahamas	13.8 (12.7-15)	8.1 (7.3-8.9)	8.1 (7.4-8.9)	6.7 (5-8.9)	6 (4.6-7.3)	-0.3 (-1.8-1.1)	2.1 (-1.2-5.4)
Bahrain	15.1 (14.1-16.1)	4.8 (4.3-5.2)	3.2 (2.8-3.5)	2.9 (2.2-3.9)	11.7 (10.5-12.9)	4.5 (3.1-5.9)	0.8 (-2.6-4.1)
Bangladesh	64.2 (60.5-68.2)	42.8 (40.2-45.4)	28.3 (26.7-30)	19.1 (17-21.3)	4.1 (3.3-4.8)	4.1 (3.3-4.8)	4.4 (3.1-5.8)
Barbados	11.8 (10.8-12.7)	9.3 (8.4-10.2)	10 (9-11)	8.4 (5.8-11.9)	2.7 (1.4-4)	-0.9 (-2.4-0.6)	1.9 (-1.8-5.7)
Belarus	8.5 (6.5-10.8)	6 (5.2-6.8)	1.8 (1.5-2.2)	1.2 (1-1.5)	2.8 (-1-6.6)	12.5 (10.9-14.2)	4.6 (2.1-7.2)
Belgium	4.6 (4.4-4.8)	2.9 (2.8-3.1)	2.3 (2.2-2.5)	2 (1.6-2.4)	4.4 (3.8-5)	2.3 (1.6-3)	1.7 (-0.5-4)
Belize	18.1 (15.2-21.4)	12 (10.8-13.3)	9.3 (8.4-10.3)	8.1 (6.9-9.6)	4.1 (2.2-6)	3.5 (2-5)	1.5 (-0.6-3.7)
Benin	45.7 (41.5-50.3)	39.6 (35.8-43.5)	34.5 (30.8-38.5)	30.6 (25.3-36.5)	1.4 (0.5-2.4)	1.4 (0.4-2.4)	1.3 (-0.3-3.1)
Bhutan	42.1 (30.9-53.9)	31.7 (26.1-37.8)	22.2 (17.5-28.1)	16.6 (9.8-27)	2.8 (-1.1-6.3)	3.5 (0.5-6.4)	3.3 (-1.2-8.1)
Bolivia (Plurinational State of)	40.9 (37.1-44.8)	29.1 (25.3-32.4)	21.2 (18.4-24.3)	14.6 (10.6-19.7)	3.4 (2.2-4.8)	3.1 (0.9-4.7)	4.1 (1.4-7.3)
Bosnia and Herzegovina	11.3 (10.8-11.7)	6.9 (6.5-7.2)	5.1 (4.8-5.4)	4.2 (3.5-5.1)	4.9 (4.1-5.6)	3.1 (2.3-3.9)	2.1 (-0.1-4.4)
Botswana	16.8 (13.2-21.1)	5.8 (4.1-8.5)	10.5 (5.3-19.1)	17.9 (5.7-40.6)	11.1 (7.3-14.4)	-5.3 (-11-0.9)	-6 (-14.1-4.6)
Brazil	25.3 (22.2-28.4)	18 (15.5-20.3)	11.2 (10.6-11.8)	7.9 (6.4-9.5)	3.4 (1.7-5.2)	4.8 (3.2-6.2)	3.9 (1.8-6.1)
Brunei Darussalam	5.6 (5-6.3)	5 (4.5-5.5)	4.8 (4.4-5.3)	6 (5.2-7)	1.4 (-0.4-3.2)	0.3 (-1.3-1.8)	-2.4 (-4.5–0.3)
Bulgaria	8.1 (7.7-8.4)	7.8 (7.5-8.2)	5.1 (4.9-5.4)	3.3 (3.1-3.6)	-0.1 (-0.6-0.4)	4.6 (4-5.2)	4.8 (3.8-5.8)
Burkina Faso	45.6 (40.4-51.1)	40.9 (36.1-45.9)	30.9 (25.3-37.1)	25.9 (16.6-38.8)	1 (-0.4-2.4)	2.9 (1-4.9)	1.9 (-1.9-5.9)
Burundi	39.9	36.9	27.8	21	0.7	2.7	3.1

	Neonatal mortality rate (per 1,000 live births)				Average annual rate of reduction in neonatal mortality rate (%)		
	1990	2000	2010	2019	1990-1999	2000-2009	2010-2019
	(34.4-45.9)	(31.8-42.3)	(23.4-32.9)	(13.6-32.3)	(-1.1-2.5)	(0.7-4.7)	(-1.3-7.5)
Cabo Verde	19.9	17.6	15.3	9	0.9	1.3	5.9
	(17.4-22.6)	(15.5-19.9)	(14-16.7)	(7.2-11.3)	(-1.2-2.9)	(-0.4-3)	(3.3-8.7)
Cambodia	40.1	35.3	21.2	14.5	1	5.1	4.2
	(36-44.9)	(31.2-39.7)	(17.5-25.6)	(7.5-27.9)	(-0.4-2.4)	(2.9-7.3)	(-2.7-11)
Cameroon	40.2	35.2	31.2	26.1	1.2	1.2	2
	(36-44.5)	(31.1-39.4)	(27.4-35.2)	(20.4-33)	(-0.1-2.5)	(-0.3-2.7)	(-0.5-4.6)
Canada	4.4	3.7	3.7	3.3	1.7	-0.1	1.3
	(4.2-4.5)	(3.6-3.8)	(3.6-3.8)	(3.1-3.6)	(1.3-2.1)	(-0.4-0.3)	(0.4-2.3)
Central African Republic	52	49.4	46.8	39.7	0.5	0.5	1.8
	(45-60.4)	(40.1-60.8)	(33.4-65.4)	(22.1-72.8)	(-1.5-2.6)	(-2.2-3.1)	(-3-6.7)
Chad	51.9	44.4	38	33.3	1.5	1.6	1.5
	(46.1-58.5)	(39.1-50.1)	(32.7-44.2)	(23.4-46.6)	(0.1-3)	(0-3.3)	(-1.8-4.9)
Chile	8.6	5.7	5.3	4.6	3.9	0.8	1.7
	(8.2-9)	(5.5-5.9)	(5.1-5.5)	(3.4-6.1)	(3.5-4.4)	(0.4-1.3)	(-1.5-4.9)
China	29.6	21	8.4	3.9	3.3	9.1	8.6
	(26.3-33.5)	(19.2-22.8)	(7.6-9.2)	(3.2-4.6)	(1.6-5)	(7.8-10.5)	(6.4-10.9)
Colombia	17.7	13.5	10	7.5	2.6	2.9	3.2
	(16.2-19.3)	(12.3-14.9)	(8.6-11.7)	(5.2-10.7)	(1.6-3.7)	(1.4-4.4)	(-0.1-6.7)
Comoros	49.6	40.6	36.2	29.8	2.2	1	2.2
	(41.2-59)	(25.8-55.7)	(17.3-67.4)	(11.1-70.4)	(-1.4-6.8)	(-4.8-6.4)	(-3.8-8.3)
Congo	27.1	30.7	22.3	19.3	-1.4	3.3	1.6
	(23.1-31.7)	(26.5-35.4)	(18.8-26.5)	(11.1-34.1)	(-3.4-0.5)	(1.1-5.4)	(-4.3-7.1)
Cook Islands	13.3	10	5.9	4.1	2.6	5.3	3.9
	(9.9-16.6)	(6.9-12.9)	(3.7-8.6)	(2.1-7.7)	(0.6-4.9)	(2.5-8.2)	(-1-8.8)
Costa Rica	9	7.7	6.7	6.2	1.5	1.5	0.9
	(8.5-9.4)	(7.3-8)	(6.4-7)	(5.8-6.5)	(1-2.1)	(0.9-2.1)	(0.1-1.7)
Cote d'Ivoire	48.7	45.1	38.8	33	0.7	1.5	1.8
	(43.6-53.9)	(40.3-50.5)	(33.4-44.3)	(25.2-42.7)	(-0.7-2)	(0.1-3)	(-0.6-4.2)
Croatia	8.1	5.6	3.6	2.9	3.9	4.6	2.2
	(7.7-8.5)	(5.3-5.9)	(3.3-3.8)	(2.5-3.4)	(3.1-4.6)	(3.7-5.5)	(0.4-4.2)
Cuba	6.8	4.3	2.9	2.2	5	4	3.1
	(6.3-7.5)	(3.9-4.7)	(2.6-3.2)	(1.9-2.6)	(4-6)	(2.9-5.1)	(0.9-4.8)
Cyprus	6.2	3.7	2	1.3	5.2	6	4.7
	(5.2-7.2)	(3.1-4.1)	(1.7-2.4)	(0.9-2)	(2.8-7.5)	(3.6-8.3)	(-0.3-9.3)
Czechia	7.3	2.7	1.7	1.6	10.1	4.8	0.3
	(7-7.6)	(2.5-2.9)	(1.6-1.8)	(1.5-1.8)	(9.5-10.8)	(3.9-5.6)	(-0.9-1.6)
Democratic People's Republic of Korea	21.3	26.3	16.2	9.5	-3.2	5	5.9
	(14.5-30)	(16.3-39.5)	(10-23.8)	(5.6-14.2)	(-5.3–0.6)	(3.1-6.9)	(4.3-7.9)
Democratic Republic of the Congo	41.7	38.5	32.5	27.4	0.8	1.6	1.9
	(35.6-48.4)	(32.9-44.7)	(25.7-40)	(16.5-43.7)	(-1.3-2.9)	(-0.7-4.2)	(-2.5-6.4)
Denmark	4.4	3.5	2.9	3	2	2.4	-0.5
	(4.1-4.6)	(3.3-3.7)	(2.7-3)	(2.5-3.4)	(1-2.9)	(1.5-3.3)	(-2.2-1.7)
Djibouti	49.2	43.9	37.3	30.5	1.1	1.6	2.2
	(40.8-59.2)	(35.9-53.4)	(28.4-49)	(17.5-52)	(-1.5-3.6)	(-1.4-4.7)	(-2.3-7.1)
Dominica	10.4	12.9	19.5	28.1	-2	-4.1	-4
	(9.4-11.4)	(11.8-14)	(17.7-21.5)	(23.6-33.1)	(-3.1–0.9)	(-5.1–3.1)	(-5.5–2.6)
Dominican Republic	24.4	23.4	22.8	19.4	0.4	0.3	1.8
	(22.1-26.9)	(21.2-25.7)	(19.3-26.7)	(13.2-28.6)	(-0.8-1.7)	(-1.5-2)	(-1.8-5.4)
Ecuador	22.2	14.5	9.2	7.1	4.2	4.6	3
	(19-25.5)	(12.5-16.7)	(7.9-10.8)	(6.2-7.9)	(2.4-5.9)	(2.8-6.3)	(0.6-5.4)
Egypt	33.3	22.3	15.9	11.1	4	3.3	4
	(30.9-35.8)	(20.5-24.2)	(14-18.1)	(7.7-16.2)	(3.1-5)	(1.9-4.7)	(0.3-7.6)
El Salvador	22.4	14.8	9.4	6.6	4.2	4.6	4
	(19.8-25.8)	(12.8-16.8)	(7.5-11.9)	(4-10.7)	(2.5-6.2)	(2.1-6.8)	(-0.3-8.1)
Equatorial Guinea	47.9	44.4	34.9	29	0.7	2.4	2.1
	(37.1-61.8)	(36-55.3)	(24.7-47.2)	(14.9-52.5)	(-3.2-4.5)	(-0.9-6.2)	(-3.3-7.6)
Eritrea	34.7	26.5	21.7	17.8	2.8	2	2.2
	(29.9-39.6)	(23.3-30)	(17.7-26.8)	(10.3-30.7)	(1.2-4.2)	(-0.2-3.9)	(-2.9-7.3)

	Neonatal mortality rate (per 1,000 live births)				Average annual rate of reduction in neonatal mortality rate (%)		
	1990	2000	2010	2019	1990-1999	2000-2009	2010-2019
Estonia	9.9<br>(9.2-10.5)	5.3<br>(4.8-5.8)	2.2<br>(1.9-2.4)	1.1<br>(0.9-1.3)	5.9<br>(4.8-7)	9.1<br>(7.6-10.5)	7.8<br>(5.1-10.6)
Eswatini	21.5<br>(18.1-25.3)	21.6<br>(18.7-24.8)	19.8<br>(16.2-24.3)	18.4<br>(11.4-29)	0<br>(-2.1-2.1)	1<br>(-1.4-3.4)	0.8<br>(-3.5-5.3)
Ethiopia	58.9<br>(52.7-65.5)	48.2<br>(43.3-53.3)	38<br>(34.1-42.3)	27.6<br>(22.4-34.2)	2<br>(0.9-3.2)	2.3<br>(0.9-3.6)	3.5<br>(1.3-5.8)
Fiji	12.2<br>(9.6-15.4)	9.1<br>(8.1-10.1)	9.6<br>(8.5-10.8)	10.8<br>(9-13)	3.1<br>(0.3-5.9)	-1<br>(-2.4-0.6)	-1.3<br>(-3.4-0.8)
Finland	3.9<br>(3.7-4.1)	2.5<br>(2.3-2.6)	1.7<br>(1.6-1.8)	1.4<br>(1.2-1.6)	4.5<br>(3.7-5.4)	3.7<br>(2.7-4.6)	2<br>(0.4-3.6)
France	3.6<br>(3.5-3.8)	2.7<br>(2.6-2.9)	2.3<br>(2.2-2.4)	2.7<br>(2.5-2.9)	2.9<br>(2.4-3.3)	1.8<br>(1.4-2.3)	-1.7<br>(-2.7–0.8)
Gabon	31.1<br>(25.6-37.5)	28.5<br>(23.6-34.6)	24.7<br>(19.4-31.1)	20.2<br>(12.2-33.2)	0.9<br>(-1.5-3.1)	1.4<br>(-1.3-4.2)	2.2<br>(-2.3-6.7)
Gambia	45.8<br>(35.2-56.5)	37.6<br>(31.2-43.7)	32.9<br>(28-38.7)	27.1<br>(21.7-33.8)	2<br>(-1.2-5.1)	1.3<br>(-1.2-3.4)	2.1<br>(-0.1-4.4)
Georgia	24.8<br>(21.7-28.3)	22.4<br>(19.4-26.2)	10.1<br>(9.3-10.9)	4.9<br>(4.1-5.9)	0.8<br>(-1.2-2.6)	7.9<br>(6.1-9.8)	8.1<br>(5.8-10.3)
Germany	3.4<br>(3.2-3.5)	2.8<br>(2.7-2.9)	2.3<br>(2.2-2.4)	2.3<br>(2.1-2.4)	2<br>(1.6-2.4)	1.8<br>(1.4-2.2)	0.1<br>(-0.6-0.9)
Ghana	42.3<br>(38.7-46)	36.1<br>(33-39.1)	29.8<br>(27.1-32.7)	23.1<br>(18.3-29)	1.5<br>(0.4-2.6)	1.9<br>(0.7-2.9)	2.8<br>(0.3-5.4)
Greece	6.5<br>(6.2-6.7)	3.9<br>(3.8-4.1)	2.1<br>(2-2.3)	2.3<br>(2-2.6)	4.6<br>(4-5.2)	6.9<br>(6.1-7.7)	-0.6<br>(-2.3-1.1)
Grenada	12.3<br>(11-13.7)	8<br>(7-9.1)	8.9<br>(7.9-10)	11<br>(8.8-13.8)	4.7<br>(3-6.6)	-0.7<br>(-2.6-1.1)	-2.3<br>(-5.1-0.5)
Guatemala	28.1<br>(25.4-30.9)	21.2<br>(19.1-23.4)	16.2<br>(14.2-18.4)	12.2<br>(9.2-15.9)	2.8<br>(1.6-4)	2.7<br>(1.4-4)	3.2<br>(0.6-5.8)
Guinea	61<br>(54.2-68.3)	45.5<br>(40.4-51.1)	34.8<br>(30.3-39.7)	30.4<br>(23-39.6)	2.6<br>(1.3-4)	2.8<br>(1.3-4.4)	1.5<br>(-1.4-4.5)
Guinea-Bissau	63.3<br>(53.8-73.8)	54.7<br>(47-63.3)	44.2<br>(35.8-55)	35.1<br>(21.2-55.8)	1.4<br>(-0.6-3.4)	2<br>(-0.3-4.3)	2.6<br>(-1.5-7.1)
Guyana	30.5<br>(26.4-34.8)	26.7<br>(23.3-30.6)	22.5<br>(17.6-28.8)	18.6<br>(11.3-30.9)	1.2<br>(-0.6-2.9)	1.7<br>(-0.7-4.1)	2.1<br>(-2-6.3)
Haiti	38.7<br>(33.8-44)	29.7<br>(25.9-33.9)	30.8<br>(26.8-35.4)	25.3<br>(17.5-36.1)	2.7<br>(1.2-4.3)	0.3<br>(-1.4-2.1)	2.2<br>(-1.4-5.9)
Honduras	21.9<br>(19.1-24.6)	17.4<br>(15.5-19.4)	12.8<br>(10.7-15.3)	9.2<br>(5.8-14.4)	2.3<br>(0.9-3.6)	3<br>(1.2-4.6)	3.7<br>(-0.6-8.1)
Hungary	11.1<br>(10.8-11.5)	5.7<br>(5.5-6)	3.4<br>(3.2-3.5)	2<br>(1.7-2.3)	6.7<br>(6.2-7.3)	5.5<br>(4.9-6.2)	5.8<br>(4.1-7.6)
Iceland	3.5<br>(3.1-3.9)	2.1<br>(1.8-2.4)	1.3<br>(1.1-1.5)	1<br>(0.7-1.4)	5.2<br>(3.3-7.1)	4.7<br>(2.6-6.9)	3<br>(-0.7-6.6)
India	57.4<br>(54.5-60.6)	45<br>(42.6-47.5)	32<br>(30-34)	21.7<br>(19.2-24.1)	2.4<br>(1.7-3)	3.4<br>(2.6-4.2)	4.3<br>(3-5.8)
Indonesia	30.6<br>(28.4-32.9)	22.8<br>(21.1-24.5)	17.4<br>(15.9-19)	12.4<br>(10.2-15.2)	2.9<br>(2.1-3.7)	2.7<br>(1.7-3.5)	3.7<br>(1.8-5.6)
Iran (Islamic Republic of)	25.4<br>(18.1-31.3)	18.8<br>(15.7-22.1)	12.2<br>(9.6-15.7)	8.6<br>(4.8-15)	3<br>(-1.1-6.2)	4.2<br>(1.1-7)	4<br>(-1.2-9.4)
Iraq	26.1<br>(23.2-29)	23.6<br>(21.3-26.3)	19.7<br>(17.1-22.7)	15.3<br>(11.7-20)	1<br>(-0.4-2.2)	1.7<br>(0.4-3.1)	2.8<br>(0.4-5.5)
Ireland	4.7<br>(4.5-5)	4<br>(3.8-4.2)	2.5<br>(2.3-2.6)	2.1<br>(1.8-2.5)	1.7<br>(0.8-2.5)	5.4<br>(4.5-6.3)	1.9<br>(-0.2-4)
Israel	6.3<br>(6-6.5)	3.6<br>(3.4-3.7)	2.4<br>(2.3-2.5)	1.9<br>(1.8-2.1)	5.9<br>(5.3-6.4)	4.4<br>(3.8-5)	2.3<br>(1.4-3.3)
Italy	6.4<br>(6.3-6.6)	3.5<br>(3.4-3.6)	2.4<br>(2.3-2.5)	1.9<br>(1.7-2.1)	6.2<br>(5.8-6.5)	3.8<br>(3.5-4.2)	2.5<br>(1.3-3.7)
Jamaica	20.1<br>(15.8-24.8)	16.7<br>(14.1-19.8)	13<br>(9.6-17.8)	9.8<br>(5.4-18)	1.8<br>(-1-4.1)	2.4<br>(-0.4-5.3)	3.2<br>(-1.9-8.3)
Japan	2.5	1.8	1.1	0.8	3.7	4.7	2.9

	Neonatal mortality rate (per 1,000 live births)				Average annual rate of reduction in neonatal mortality rate (%)		
	**1990**	**2000**	**2010**	**2019**	**1990-1999**	**2000-2009**	**2010-2019**
	(2.5-2.6)	(1.7-1.8)	(1.1-1.1)	(0.8-0.9)	(3.3-4.1)	(4.3-5.2)	(2.1-3.8)
Jordan	19.7	16.1	12	9.2	2	3	2.9
	(17.9-21.6)	(14.6-17.8)	(10.2-13.9)	(6.6-12.7)	(0.7-3.1)	(1.5-4.6)	(0.1-5.8)
Kazakhstan	22.5	22.7	11.8	4.7	-0.4	5.7	10.3
	(19.6-25.8)	(19.9-25.9)	(11.1-12.4)	(4.3-5)	(-2.2-1.3)	(4.2-7.3)	(9.6-11.1)
Kenya	27.6	28.5	24.5	21	-0.4	1.5	1.7
	(24.6-30.7)	(25.6-31.6)	(21.4-27.8)	(15-29.7)	(-1.7-0.9)	(0-3)	(-1.9-5.1)
Kiribati	35.2	28.7	25.7	22.1	2	1.1	1.7
	(27.5-45.8)	(23.3-35.4)	(16.9-35.8)	(10.3-41.1)	(-1.5-5.7)	(-2.4-5.6)	(-3.7-7.7)
Kuwait	10.4	6.5	5.5	4.5	4.8	1.7	2.2
	(9.7-11.3)	(6.2-6.9)	(5.2-5.8)	(4-5.1)	(3.9-5.9)	(1-2.5)	(0.6-3.7)
Kyrgyzstan	24.3	20.5	17.2	12.3	1.5	1.6	3.7
	(20.1-28.8)	(17.1-23.9)	(15.6-18.7)	(10.3-14.3)	(-0.9-4.1)	(-0.6-3.6)	(1.6-5.9)
Lao People's Democratic Republic	47.2	38	28.6	22	2.2	2.8	2.9
	(42.1-53.1)	(34.1-42.7)	(24.7-33.2)	(15.9-29.6)	(0.8-3.4)	(1.3-4.4)	(0.2-5.9)
Latvia	8.4	7.2	4.2	1.9	0.7	5.1	9.1
	(7.8-8.9)	(6.6-7.7)	(3.9-4.6)	(1.4-2.4)	(-0.2-1.7)	(3.9-6.3)	(5.8-12.3)
Lebanon	20.5	11.7	6	4.2	5.6	6.9	4
	(17.7-23.5)	(9-14.5)	(3.3-9.5)	(1.7-8.6)	(3.1-8.8)	(2.7-12.1)	(-1.2-9.8)
Lesotho	38.5	36.9	40.5	42.8	0.5	-0.9	-0.6
	(33.7-43.9)	(33.1-40.9)	(35.1-46.7)	(28.4-63.6)	(-1-2.1)	(-2.6-0.8)	(-4.6-3.7)
Liberia	59.2	45.7	33	32.4	2.4	3.5	0.2
	(51-68.3)	(39.9-52)	(28.7-37.5)	(24.5-43)	(0.7-4.1)	(1.7-5.3)	(-3.2-3.3)
Libya	20.9	15.9	9.4	6.5	2.7	5.2	4.1
	(17.5-25)	(13.5-18.9)	(7.2-12)	(3.7-11)	(0.5-5)	(3.5-7.1)	(-0.7-9.1)
Lithuania	7.9	4.5	2.9	2	5.6	4.3	3.8
	(7.4-8.4)	(4.1-5)	(2.6-3.2)	(1.7-2.4)	(4.6-6.6)	(3-5.6)	(1.7-6)
Luxembourg	4.1	2.4	1.6	1.5	5.7	4.5	0.7
	(3.7-4.7)	(2.1-2.7)	(1.3-1.8)	(1.1-2)	(3.7-7.5)	(2.4-6.5)	(-2.8-4.3)
Madagascar	39	30.9	24	20.1	2.3	2.5	2
	(35-43.6)	(27.7-34.3)	(21-27.6)	(15.6-26)	(1-3.6)	(1.1-4)	(-0.6-4.5)
Malawi	49.5	38.7	28.2	19.8	2.2	3.3	3.9
	(44.4-54.8)	(34.6-42.8)	(25.1-31.8)	(12.7-29.8)	(1.1-3.3)	(1.8-4.7)	(-0.6-9.1)
Malaysia	7.6	4.9	4.2	4.6	4.1	1.9	-1.1
	(6.6-8.8)	(4.4-5.6)	(3.8-4.5)	(4.2-5.1)	(2.3-5.8)	(0.3-3.6)	(-2.2–0.1)
Maldives	39.4	21.8	7.6	4.9	5.4	10.7	4.8
	(31.3-47.5)	(18.6-25.2)	(6.7-8.6)	(4.1-5.8)	(2.3-8.2)	(8.8-12.6)	(2.5-7.2)
Mali	66.5	50.7	38.8	32.1	2.6	2.7	2.1
	(60-73.9)	(45.6-56.4)	(34.3-43.8)	(24.5-41.6)	(1.5-3.8)	(1.3-4.1)	(-0.6-4.9)
Malta	7.6	5.1	4.6	4.7	4.1	1.2	-0.3
	(7-8.2)	(4.6-5.6)	(4.1-5.1)	(3.7-6)	(2.8-5.4)	(-0.3-2.7)	(-3.2-2.6)
Marshall Islands	19.4	18.3	17.6	15.3	0.7	0.4	1.6
	(15.5-24.3)	(14.9-22.4)	(12.7-23.7)	(8.8-26.2)	(-2.5-3.8)	(-3.1-4)	(-3.1-6.2)
Mauritania	46.4	42.9	38.3	32	0.8	1	2
	(40.3-53.7)	(36.2-50.5)	(24.8-59)	(15.5-64.5)	(-1.2-2.9)	(-2.8-5)	(-3.3-7.5)
Mauritius	14.6	12.4	9.3	10.2	0.7	3	-1
	(13.8-15.5)	(11.6-13.2)	(8.6-10)	(9.1-11.5)	(-0.1-1.6)	(2-4)	(-2.5-0.5)
Mexico	22.2	14.2	9	8.6	4.2	4.9	0.5
	(19.9-24.6)	(12.8-15.7)	(8.4-9.7)	(7.7-9.5)	(2.9-5.6)	(3.9-6)	(-0.8-1.8)
Micronesia (Federated States of)	24.7	24	19.5	15.9	0.2	2.1	2.3
	(16.9-34.6)	(13.8-39.6)	(8.8-41.9)	(5.8-41.7)	(-3.3-3.8)	(-3.3-7.3)	(-3.2-7.9)
Monaco	4.3	2.9	2.2	1.7	4.1	2.6	3
	(3.2-5.4)	(2-3.7)	(1.4-3)	(0.9-2.8)	(2.1-6.5)	(0.7-4.8)	(-1.4-7.1)
Mongolia	29.8	23.5	11.6	8.1	2	7	4
	(25.9-34.1)	(20.5-26.7)	(10.8-12.5)	(7.6-8.7)	(0-4.1)	(5.3-8.7)	(3.1-4.9)
Montenegro	10.8	8.7	4.3	1.3	2.1	6.6	13.3
	(9.8-11.8)	(7.8-9.5)	(3.8-4.9)	(1-1.7)	(0.6-4.1)	(4.7-8.2)	(10-17)
Morocco	35.9	26.9	20	13.6	2.9	2.9	4.3
	(32.6-39.5)	(24-30)	(17.3-23)	(10.1-18.2)	(1.7-4.1)	(1.3-4.4)	(1.7-7)

	Neonatal mortality rate (per 1,000 live births)				Average annual rate of reduction in neonatal mortality rate (%)		
	1990	2000	2010	2019	1990-1999	2000-2009	2010-2019
Mozambique	61.9 (55.6-69.2)	46.1 (41.6-51.1)	33.7 (29.4-38.4)	28.5 (19.7-42.3)	2.7 (1.6-3.9)	3.3 (1.9-4.7)	1.8 (-2.1-5.4)
Myanmar	47.4 (41-55.6)	37.3 (32.8-42.1)	28.2 (23.4-33.3)	22.4 (14.8-32.8)	2.4 (0.5-4.4)	2.8 (0.9-4.9)	2.5 (-1.1-6.4)
Namibia	27.8 (24.4-31.6)	22.6 (20.1-25.7)	20.1 (15.9-25.4)	19.1 (10.8-35.8)	2.1 (0.2-3.8)	1.4 (-1-4)	0.5 (-5.1-5.7)
Nauru	29.1 (16.2-49.6)	24.5 (20.1-30.1)	23.9 (16.1-35.1)	19.9 (10.1-37.6)	1.7 (-4.7-7.4)	0.2 (-3.9-4.5)	2 (-3.1-7.5)
Nepal	58.4 (53.5-63.6)	40.4 (37.1-44)	27.3 (24.4-30.5)	19.8 (15-26.4)	3.7 (2.8-4.6)	3.9 (2.8-5)	3.6 (0.8-6.3)
Netherlands	4.6 (4.4-4.8)	3.8 (3.7-4)	2.8 (2.7-2.9)	2.6 (2.4-2.7)	2.1 (1.7-2.6)	3.2 (2.7-3.7)	0.8 (0.1-1.6)
New Zealand	4.3 (4.1-4.6)	3.5 (3.3-3.7)	3.2 (3.1-3.4)	2.6 (2.1-3.2)	2.4 (1.6-3.2)	1.4 (0.5-2.2)	2.3 (0.1-4.9)
Nicaragua	22.8 (20.3-25.6)	16.5 (14.3-18.7)	13.6 (11.8-15.5)	10.2 (8.8-12.1)	3.2 (1.6-4.9)	2 (0-3.8)	3.1 (0.7-5.4)
Niger	54.5 (48.6-61.1)	43.1 (38-48.6)	30.5 (24.9-36.8)	24.3 (14.9-39)	2.3 (1-3.6)	3.5 (1.5-5.5)	2.5 (-2-7.1)
Nigeria	49.7 (44.5-55.1)	46.3 (41.7-51)	38 (33.7-42.6)	35.9 (27.5-47.7)	0.5 (-0.6-1.7)	2.1 (0.9-3.4)	0.6 (-2.4-3.4)
Niue	7.4 (4.3-12.4)	13 (7.7-21.1)	16.1 (7.5-34.4)	12.5 (4.7-33.2)	-5.7 (-11.3–0.1)	-2.5 (-7.9-2.7)	2.8 (-2.6-8.2)
Norway	4 (3.7-4.2)	2.7 (2.5-2.8)	1.9 (1.7-2)	1.4 (1.2-1.6)	4.2 (3.3-5)	3.6 (2.6-4.5)	3.2 (1.7-4.7)
Oman	16.8 (13.9-19.8)	7.6 (5.8-9.5)	5.4 (4-6.8)	5.3 (3.6-7)	8.1 (5.7-10.8)	3.6 (1.5-5.9)	0.3 (-1.7-2.5)
Pakistan	64.1 (59.5-68.7)	56.9 (53.2-60.7)	49.9 (46.1-53.8)	41.2 (34.6-48.8)	1.1 (0.2-2)	1.3 (0.5-2.2)	2.1 (0.4-4)
Palau	18.6 (13.4-24.3)	15.9 (10.9-21.3)	12.5 (7.2-20.9)	9.4 (4.2-19.8)	1.5 (-1.2-4.4)	2.3 (-2-6.5)	3.2 (-1.9-8.5)
Panama	17.7 (14-21.1)	15.2 (10.9-19.1)	11.5 (7.1-17.3)	8.6 (4.1-16.7)	1.5 (-0.7-3.9)	2.7 (-0.4-5.8)	3.2 (-1.8-8.5)
Papua New Guinea	33.6 (29.4-38)	30.7 (26.8-35.2)	26.1 (21.7-31.3)	21.9 (15.1-31)	0.8 (-1-2.3)	1.6 (-0.1-3.4)	1.9 (-1.2-5.3)
Paraguay	21.8 (19-25)	18.1 (14.8-22.3)	14.6 (9.5-22.9)	10.9 (5.5-22.2)	1.9 (-0.2-3.9)	2.1 (-1.6-5.5)	3.3 (-1.8-8.3)
Peru	27.9 (25.8-30.2)	15.9 (14.5-17.3)	9.6 (8.7-10.5)	6.4 (5-8.1)	5.6 (4.5-6.6)	5.1 (3.9-6.3)	4.5 (2-7)
Philippines	19.3 (17.4-21.3)	16.3 (14.8-18)	15 (13.3-16.9)	13.3 (9.9-17.8)	1.8 (0.5-3)	0.9 (-0.5-2.2)	1.3 (-1.8-4.4)
Poland	11.1 (10.8-11.5)	5.8 (5.6-5.9)	3.6 (3.5-3.7)	2.7 (2.6-2.8)	6.5 (6.1-6.8)	4.6 (4.2-5)	3.1 (2.5-3.7)
Portugal	7.3 (6.9-7.6)	3.4 (3.2-3.5)	2.1 (2-2.3)	2 (1.8-2.2)	7.9 (7.3-8.6)	5.2 (4.3-6)	0.9 (-0.4-2.2)
Qatar	11.3 (10.1-12.9)	6.6 (6-7.2)	4.6 (4.2-5)	3.4 (2.9-4)	5.5 (3.9-7.3)	3.7 (2.5-4.9)	3.4 (1.1-5.4)
Republic of Korea	7.3 (6.4-8.5)	3.4 (3.1-3.7)	1.8 (1.6-1.9)	1.5 (1.4-1.7)	7.8 (6-9.6)	7.1 (6.1-8.1)	1.5 (0.3-2.6)
Republic of Moldova	18.7 (15.8-22.1)	20.7 (17-24.9)	12.7 (10.8-15.3)	10.7 (7.7-14.6)	-1.8 (-4-0.5)	5.4 (2.7-7.6)	1.9 (-1.9-6)
Republic of North Macedonia	17.2 (16.1-18.3)	9.1 (8.5-9.8)	6.6 (6.1-7.1)	3.9 (3.4-4.5)	6.3 (5.4-7.2)	2.8 (1.8-3.9)	5.8 (4-7.7)
Romania	15.8 (12.4-20.1)	10.5 (8.7-12.3)	5.4 (4.4-6.4)	3.4 (2.9-4)	3.7 (0.5-7.3)	6.6 (4-9.4)	5.1 (3.4-6.7)
Russian Federation	10.9 (7.9-14.3)	9 (7.7-10.3)	4.4 (3.7-5.1)	2.6 (2.2-3)	1.9 (-2.6-6.4)	7.5 (6.1-8.9)	5.8 (4.3-7.3)
Rwanda	40.8 (36.7-45.1)	40.9 (36.2-45.9)	23.1 (19.7-27)	15.9 (8.9-28)	-0.4 (-1.8-1)	5.7 (4-7.3)	4.2 (-1.6-10)
Saint Kitts and Nevis	19.3	16.6	11.7	10.3	1.3	3.7	1.5

	Neonatal mortality rate (per 1,000 live births)				Average annual rate of reduction in neonatal mortality rate (%)		
	1990	2000	2010	2019	1990-1999	2000-2009	2010-2019
	(17-21.7)	(14.6-18.9)	(10-13.7)	(7.2-14.8)	(-0.5-3.1)	(1.8-5.6)	(-2.5-5.2)
Saint Lucia	12.1	11.6	13	12.7	0.6	-1.1	0.2
	(11.1-13.1)	(10.7-12.6)	(11.9-14.1)	(10.6-15.2)	(-0.5-1.8)	(-2.3-0)	(-1.8-2.2)
Saint Vincent and the Grenadines	12.8	13.2	12.5	9.1	-0.3	0.4	3.5
	(11.5-14.2)	(11.9-14.5)	(11.2-14)	(7-11.7)	(-1.8-1.2)	(-1.2-2)	(0.6-6.6)
Samoa	16.4	11.5	10.2	8.2	3.6	1.1	2.4
	(12.1-21.1)	(7.9-15.2)	(6.4-14)	(4.6-13.4)	(1.5-6)	(-1.2-3.7)	(-1.5-6.1)
San Marino	7.4	3	1.3	0.8	8.9	8.9	5.4
	(5.2-10.8)	(2.1-4.4)	(0.6-2.2)	(0.3-1.7)	(4-14.5)	(3.5-16.4)	(-0.5-12.3)
Sao Tome and Principe	26.4	22.6	17.9	14.1	1.6	2.4	2.7
	(20.4-33.1)	(17-28)	(13.3-23.8)	(7.9-24.9)	(-1.7-5.5)	(-1.7-5.9)	(-2.4-7.8)
Saudi Arabia	21.6	12.2	6.7	3.7	5.8	6	6.6
	(15.2-29.1)	(8.5-15.9)	(4.2-9.4)	(2.1-5.7)	(3.3-8.6)	(2.9-8.6)	(3.2-10.9)
Senegal	40	37.9	26.8	22	0.4	3.5	2.2
	(36.9-43.3)	(34.3-41.5)	(24.3-29.5)	(16.9-28.8)	(-0.6-1.4)	(2.2-4.7)	(-0.9-5.2)
Serbia	17.2	7.8	4.8	3.2	8.3	5.2	4.4
	(15.5-18.7)	(7.3-8.2)	(4.5-5.1)	(2.5-4.1)	(6.9-9.6)	(4.5-6)	(1.8-7)
Seychelles	10.9	8.7	8.7	8.6	2.5	0	0.1
	(9.7-12.3)	(7.6-9.8)	(7.6-9.9)	(6.2-11.7)	(0.8-4.5)	(-1.7-1.9)	(-3.4-3.8)
Sierra Leone	52.4	49.2	39.4	31.2	0.6	2.1	2.6
	(45.4-60.7)	(43.3-56.3)	(34.6-44.7)	(24.2-39.5)	(-1.4-2.4)	(0.5-3.9)	(0.1-5.3)
Singapore	4.1	1.6	1.1	0.9	9.3	3.8	2.3
	(3.8-4.3)	(1.5-1.8)	(1-1.2)	(0.8-1.1)	(8.2-10.4)	(2.3-5.4)	(0-4.6)
Slovakia	8.7	5.1	3.4	2.9	5.5	4.5	1.6
	(8.3-9)	(4.8-5.3)	(3.2-3.5)	(2.7-3.1)	(4.9-6.1)	(3.8-5.3)	(0.5-2.7)
Slovenia	5.7	3.3	1.8	1.2	5.8	6	4.7
	(5.4-6.1)	(3-3.5)	(1.6-1.9)	(1-1.4)	(4.8-6.9)	(4.8-7.3)	(2.5-6.8)
Solomon Islands	14.6	12.9	10.9	8.2	1.3	1.5	3.2
	(11.8-18)	(11-15.3)	(8.7-13.5)	(4.9-13.1)	(-1.5-3.9)	(-0.9-4.4)	(-1.3-8.1)
Somalia	44.8	44	43.2	36.9	0.3	0	1.8
	(34.2-58.2)	(32.7-60.8)	(26.3-71.1)	(16.2-80.7)	(-3-3.5)	(-4-4)	(-4.1-8)
South Africa	19.6	14.9	11.2	11.5	2.7	2.9	-0.3
	(16.5-23.2)	(12.7-18.1)	(9.3-12.9)	(9.2-14.3)	(-0.1-5.1)	(0.8-5.9)	(-3.7-2.7)
South Sudan	64.1	56	42	38.6	1.3	2.8	0.9
	(44.8-86.1)	(41.5-73.3)	(26.2-64.5)	(13.6-88.8)	(-3.7-5.7)	(-2.3-8.2)	(-5.2-8.9)
Spain	4.9	2.8	2.1	1.8	6	3.2	1.8
	(4.7-5.2)	(2.7-3)	(2-2.2)	(1.6-2.1)	(5.6-6.5)	(2.7-3.7)	(0.3-3.4)
Sri Lanka	13.4	9.6	6.4	4.3	2.7	3.9	4.5
	(12.8-14.1)	(9.2-10)	(6.1-6.7)	(3.4-5.3)	(2.1-3.2)	(3.4-4.4)	(1.9-7)
State of Palestine	21.7	16.5	12.8	10.7	2.8	2.6	2
	(19.3-24.3)	(14.8-18.3)	(10.8-15.2)	(7.3-15.7)	(1.4-4.3)	(0.8-4.4)	(-1.4-5.2)
Sudan	42.4	36.9	31.7	27.2	1.4	1.6	1.7
	(37.9-47.3)	(32.6-41.2)	(27.3-36.8)	(19.5-37.5)	(0.1-2.8)	(-0.1-3)	(-1.4-4.8)
Suriname	20.6	17.2	14.8	11.2	1.9	1.4	3.1
	(13.1-27.3)	(13.5-21.2)	(11.3-19.3)	(6.9-18)	(-3.3-5.8)	(-2.2-4.4)	(-1.3-7.5)
Sweden	3.5	2.3	1.6	1.4	4.5	3.7	1.8
	(3.3-3.7)	(2.2-2.5)	(1.5-1.7)	(1.3-1.5)	(3.7-5.2)	(2.9-4.6)	(0.6-2.9)
Switzerland	3.9	3.5	3.1	2.8	1.3	1	1.4
	(3.7-4.1)	(3.3-3.6)	(3-3.3)	(2.4-3.1)	(0.6-2.1)	(0.3-1.7)	(-0.2-3)
Syrian Arab Republic	16.2	12.2	10.1	10.8	2.8	2	-0.7
	(14.1-18.5)	(10.6-14)	(8.2-12.2)	(6.1-14.8)	(1.1-4.6)	(-0.1-4.3)	(-4.2-4.1)
Tajikistan	30.7	28.4	20.2	15	0.5	3.4	3.3
	(25.7-37)	(24.1-33.6)	(17-24.3)	(8.5-24.7)	(-2-3.1)	(0.9-5.6)	(-2.6-10.1)
Thailand	20.5	12.8	8	5.3	4.6	4.8	4.5
	(16.1-23.4)	(9.6-15.6)	(5.6-10.1)	(3.6-7.3)	(3-6.4)	(3.1-6.6)	(2-6.6)
Timor-Leste	55.3	36.8	24.5	19.6	4	4.2	2.5
	(46.8-65.8)	(31.5-42.5)	(17.2-32.4)	(10.3-35.5)	(1.9-6.3)	(1.4-7.8)	(-2.9-7.8)
Togo	43	36.2	29.2	24.8	1.7	2.2	1.8
	(38.4-48.1)	(32.3-40.4)	(25.5-33.3)	(19.1-31.9)	(0.4-3)	(0.8-3.6)	(-0.5-4.3)

	Neonatal mortality rate (per 1,000 live births)				Average annual rate of reduction in neonatal mortality rate (%)		
	**1990**	**2000**	**2010**	**2019**	**1990-1999**	**2000-2009**	**2010-2019**
Tonga	10.3<br>(8-14)	7.9<br>(6.2-9.8)	7.3<br>(5.5-9.2)	7.4<br>(5-10.8)	2.7<br>(-0.2-6.7)	1<br>(-2-4.1)	-0.2<br>(-4.4-3.8)
Trinidad and Tobago	20.2<br>(16.3-24.2)	18.8<br>(13.4-26.6)	15.3<br>(8.4-29.7)	11.5<br>(4.9-27.9)	0.7<br>(-2.8-3.6)	2<br>(-2.6-6.1)	3.2<br>(-2.1-8.6)
Tunisia	27.6<br>(23.6-32.1)	18.8<br>(16-21.9)	12.5<br>(11.5-13.4)	11.9<br>(10.7-13.3)	3.7<br>(1.8-5.6)	4.4<br>(2.5-6.3)	0.5<br>(-1.1-2.1)
Turkey	33<br>(29.7-36.6)	18.7<br>(16.6-20.9)	9.1<br>(8-10.2)	5.3<br>(4.6-6)	5.7<br>(4.2-7.2)	7.1<br>(5.6-8.8)	6<br>(4-7.8)
Turkmenistan	26.8<br>(22.4-32)	29.7<br>(24.9-35.4)	22.7<br>(17.6-29.1)	23.6<br>(16.4-33.2)	-1.4<br>(-3.6-0.8)	2.8<br>(-0.1-5.9)	-0.4<br>(-5-3.9)
Tuvalu	27.7<br>(21.8-34.9)	24.2<br>(20.9-27.8)	21.1<br>(14.7-30)	16<br>(8.5-30.9)	1.4<br>(-1.6-4.3)	1.2<br>(-2.4-4.9)	3.1<br>(-2.1-8.1)
Uganda	38.8<br>(34.7-43.1)	32.1<br>(28.6-35.7)	25.3<br>(22.5-28.4)	20<br>(14.6-27.1)	1.8<br>(0.5-3.2)	2.4<br>(0.8-3.9)	2.6<br>(-0.8-6)
Ukraine	12<br>(10.2-14.3)	11.2<br>(9.4-13.1)	7<br>(5.3-8.3)	5<br>(3.7-6.2)	0.3<br>(-1.9-2.7)	4.6<br>(2.2-7.7)	3.7<br>(2.3-5.3)
United Arab Emirates	8.3<br>(6.9-10)	5.9<br>(5.2-6.9)	4.5<br>(4.1-5.2)	4<br>(3.3-4.8)	3.5<br>(1-5.7)	2.7<br>(0.9-4.5)	1.5<br>(-1-4.2)
United Kingdom	4.5<br>(4.3-4.6)	3.8<br>(3.7-3.9)	3<br>(3-3.1)	2.8<br>(2.6-2.9)	1.7<br>(1.3-2.1)	2.1<br>(1.7-2.5)	1<br>(0.3-1.7)
United Republic of Tanzania	39.9<br>(36.5-43.7)	33.6<br>(30.7-36.8)	24.7<br>(22-28)	20.3<br>(15-28.1)	1.6<br>(0.5-2.7)	3.1<br>(1.8-4.5)	2.2<br>(-1.1-5.3)
United States of America	5.8<br>(5.6-5.9)	4.6<br>(4.5-4.8)	4.1<br>(4-4.2)	3.7<br>(3.4-4)	2.3<br>(2-2.6)	1.2<br>(0.9-1.5)	1.1<br>(0.3-2)
Uruguay	12<br>(10.4-13.6)	8.4<br>(7.8-9.1)	4.8<br>(4.4-5.2)	4.2<br>(3.8-4.6)	3.8<br>(1.8-5.5)	5.7<br>(4.7-6.6)	1.5<br>(0.1-2.8)
Uzbekistan	30.9<br>(25.8-36.6)	28.1<br>(23.3-33.9)	18.4<br>(13-24.4)	9.9<br>(6.8-13.5)	0.8<br>(-1.9-3.4)	4.1<br>(1.4-7.5)	6.9<br>(3.1-10.7)
Vanuatu	15.9<br>(12.2-21.8)	12.5<br>(9.3-15.7)	13<br>(9.2-17.6)	11.4<br>(5.8-20.8)	2.5<br>(-0.8-7.5)	-0.4<br>(-4.3-3.3)	1.5<br>(-4-7.5)
Venezuela (Bolivarian Republic of)	12.8<br>(12.1-13.6)	11.1<br>(10.5-11.6)	10.5<br>(10.1-10.9)	14.6<br>(11.2-18.5)	1.4<br>(0.8-2)	0.9<br>(0.4-1.4)	-3.6<br>(-6.3–0.7)
Viet Nam	23.4<br>(20.6-26.9)	15.4<br>(12.7-18.3)	12<br>(10.2-13.8)	10.5<br>(7.7-14.3)	4.2<br>(2-6.8)	2.7<br>(0.5-4.7)	1.5<br>(-2.2-4.8)
Yemen	43.3<br>(39.5-47.5)	36.8<br>(33.1-40.9)	26.8<br>(22.8-31.5)	26.7<br>(15.8-43.8)	1.5<br>(0.4-2.6)	3.3<br>(1.6-5)	0.1<br>(-4.5-5)
Zambia	36<br>(32.6-39.6)	33.9<br>(30.8-37.4)	25.7<br>(23.1-28.6)	23.3<br>(18-30.1)	0.5<br>(-0.7-1.6)	2.9<br>(1.7-4.2)	1.1<br>(-1.7-4)
Zimbabwe	24.6<br>(21.9-27.5)	26.4<br>(23.7-29.3)	31.7<br>(28.4-35.5)	25.9<br>(18.8-34.9)	-0.7<br>(-2.2-0.7)	-2.2<br>(-3.7–0.8)	2.3<br>(-1.2-5.8)

3.3. Table A.3 Number of under-5 and neonatal deaths, by country, 1990-2019

Note: Numbers in brackets are 90% uncertainty intervals.

	Number of under-five deaths				Number of neonatal deaths			
	**1990**	**2000**	**2010**	**2019**	**1990**	**2000**	**2010**	**2019**
Afghanistan	110011 (100845-120093)	125079 (116331-134873)	101508 (93034-110889)	72186 (56868-89342)	48398 (43221-54133)	62013 (56556-68197)	55545 (50093-61526)	43424 (33631-54696)
Albania	3257 (2897-3659)	1448 (1319-1589)	472 (457-488)	327 (306-349)	1000 (775-1256)	625 (505-756)	237 (211-262)	251 (231-273)
Algeria	41074 (38276-43936)	24415 (23872-24963)	23338 (22888-23793)	23598 (22845-24379)	19240 (17087-21539)	12772 (11524-14000)	15477 (14906-16056)	16484 (15790-17185)
Andorra	9 (2-34)	5 (3-11)	4 (2-8)	2 (0-6)	5 (1-19)	3 (1-6)	2 (1-4)	1 (0-3)
Angola	124961 (110909-141161)	152254 (133577-174328)	122120 (84842-169230)	92690 (43228-172073)	31778 (26046-39242)	39789 (33129-47772)	38656 (26246-54820)	35358 (15603-68835)
Antigua and Barbuda	16 (14-18)	23 (21-25)	15 (13-17)	10 (7-14)	10 (8-11)	14 (13-16)	9 (7-10)	5 (4-8)
Argentina	20533 (20153-20915)	14077 (13819-14345)	10679 (10477-10881)	6977 (6633-7346)	10796 (10458-11140)	7865 (7625-8110)	5689 (5512-5871)	4583 (4299-4885)
Armenia	3732 (3389-4116)	1241 (1116-1368)	818 (692-938)	482 (358-621)	1684 (1463-1962)	651 (565-744)	449 (356-554)	258 (175-366)
Australia	2307 (2264-2352)	1532 (1503-1561)	1421 (1393-1450)	1146 (1084-1213)	1176 (1137-1216)	870 (842-897)	825 (798-852)	726 (679-777)
Austria	841 (818-865)	449 (433-465)	338 (325-352)	306 (265-356)	410 (390-431)	248 (235-261)	197 (186-208)	184 (155-217)
Azerbaijan	19421 (17608-21430)	10767 (9587-12085)	5919 (4903-7155)	3370 (2164-5255)	6790 (5644-8068)	4716 (3989-5655)	3268 (2619-4064)	1765 (1062-2838)
Bahamas	143 (136-150)	88 (83-94)	82 (77-87)	68 (53-86)	85 (78-92)	43 (39-47)	43 (39-47)	36 (27-48)
Bahrain	329 (318-340)	182 (174-191)	164 (157-173)	151 (119-193)	217 (203-231)	70 (64-77)	62 (56-69)	65 (48-87)
Bangladesh	512896 (498112-528230)	301684 (291812-311531)	152809 (147000-158756)	89796 (81574-98812)	232185 (218701-246586)	150029 (141166-159148)	88248 (83315-93477)	55542 (49459-62101)
Barbados	76 (71-81)	57 (53-62)	50 (46-54)	39 (27-54)	49 (45-53)	35 (32-39)	33 (30-36)	26 (18-36)
Belarus	2256 (2207-2303)	1151 (1124-1177)	588 (570-606)	360 (327-396)	1210 (928-1534)	533 (461-603)	202 (168-237)	133 (105-164)
Belgium	1194 (1167-1220)	663 (645-682)	563 (546-581)	424 (363-494)	550 (524-578)	336 (318-354)	297 (281-313)	249 (205-300)
Belize	252 (222-286)	171 (160-182)	142 (135-149)	98 (85-113)	121 (102-143)	88 (80-98)	71 (64-78)	65 (55-77)
Benin	38307 (36083-40734)	38840 (36523-41409)	38636 (35646-41952)	37100 (31832-43035)	10531 (9565-11596)	11533 (10452-12689)	12477 (11155-13923)	12931 (10696-15455)
Bhutan	2488 (2205-2854)	1250 (1128-1395)	569 (470-679)	369 (236-565)	832 (610-1065)	505 (417-603)	297 (233-374)	215 (127-350)
Bolivia (Plurinational State of)	28796 (27273-30398)	19029 (17875-20291)	10354 (9198-11615)	6399 (4831-8404)	9937 (9005-10885)	7427 (6469-8268)	5318 (4618-6114)	3598 (2612-4857)
Bosnia and Herzegovina	1198 (1169-1229)	424 (409-440)	246 (235-257)	158 (133-187)	725 (694-756)	283 (270-298)	173 (164-183)	111 (92-133)
Botswana	2136 (1801-2557)	3248 (2614-4504)	1902 (1101-3181)	2320 (845-4862)	749 (589-943)	278 (196-408)	578 (294-1054)	999 (320-2261)
Brazil	232778 (217103-249453)	122065 (114257-130622)	56301 (54738-57883)	40429 (33847-47989)	93557 (82038-105268)	63185 (54586-71233)	33515 (31751-35362)	22736 (18638-27579)
Brunei Darussalam	99 (94-105)	78 (74-84)	65 (61-70)	73 (64-82)	43 (38-48)	38 (34-42)	33 (30-36)	38 (32-44)
Bulgaria	2011 (1963-2059)	1155 (1124-1186)	804 (779-830)	422 (395-450)	846 (812-881)	511 (488-535)	377 (359-396)	206 (190-223)
Burkina Faso	79061	91457	77477	64744	19054	21982	20438	19767

	Number of under-five deaths				Number of neonatal deaths			
	1990	2000	2010	2019	1990	2000	2010	2019
	(74061-84404)	(85252-97962)	(69746-85798)	(47418-89259)	(16914-21366)	(19391-24679)	(16729-24511)	(12652-29582)
Burundi	45577 (41522-50131)	42075 (38333-46286)	32091 (27888-36813)	24412 (16185-36590)	10770 (9276-12387)	10270 (8861-11775)	10354 (8704-12269)	9288 (6015-14296)
Cabo Verde	834 (804-864)	455 (442-469)	290 (278-301)	157 (130-190)	279 (243-315)	208 (183-236)	169 (155-185)	94 (75-118)
Cambodia	44081 (40860-47671)	36125 (33197-39397)	15904 (13374-18916)	9647 (5090-18201)	15342 (13776-17162)	11980 (10612-13507)	7748 (6417-9367)	5257 (2730-10125)
Cameroon	68727 (64112-73634)	88571 (82298-95493)	85244 (77717-93409)	66071 (54455-79419)	21190 (18995-23501)	22647 (20010-25356)	25004 (21995-28280)	23557 (18451-29830)
Canada	3152 (3099-3210)	2089 (2051-2126)	2118 (2081-2155)	1873 (1748-2002)	1678 (1626-1731)	1240 (1203-1278)	1420 (1383-1458)	1285 (1184-1391)
Central African Republic	20246 (18140-22644)	24446 (21785-27386)	24497 (19037-31983)	18095 (10540-31113)	6185 (5350-7186)	7426 (6033-9135)	7896 (5640-11024)	6646 (3694-12177)
Chad	60310 (56012-64993)	74268 (68892-80409)	79352 (71492-88297)	73024 (55545-96532)	15776 (14002-17796)	18979 (16730-21454)	21245 (18259-24678)	22123 (15599-30991)
Chile	5597 (5477-5718)	2814 (2754-2875)	2159 (2114-2208)	1612 (1236-2090)	2544 (2437-2653)	1450 (1393-1507)	1324 (1279-1371)	1044 (779-1398)
China	1378848 (1268159-1504390)	652138 (615720-693869)	274277 (258490-290978)	132256 (115661-151885)	744617 (660009-841243)	368525 (337973-400917)	147011 (133862-160300)	63895 (53379-75611)
Colombia	31152 (29011-33393)	22041 (20313-23849)	14068 (12305-16029)	10091 (7093-14230)	15899 (14585-17332)	11900 (10817-13120)	7600 (6496-8879)	5482 (3779-7869)
Comoros	2117 (1873-2367)	1983 (1599-2337)	1997 (1266-3258)	1656 (808-3510)	869 (722-1033)	826 (525-1132)	881 (420-1639)	797 (297-1880)
Congo	7821 (6864-8879)	13102 (11789-14555)	9487 (8194-10954)	8204 (4901-14177)	2428 (2070-2843)	3698 (3197-4269)	3523 (2960-4177)	3369 (1943-5945)
Cook Islands	14 (12-15)	8 (7-9)	4 (3-5)	2 (1-3)	8 (6-10)	4 (3-6)	2 (1-3)	1 (1-2)
Costa Rica	1433 (1405-1464)	1011 (988-1035)	753 (734-773)	599 (571-630)	769 (730-807)	587 (559-614)	480 (457-502)	425 (401-452)
Cote d’Ivoire	75180 (70199-80539)	91945 (85481-98958)	79631 (72000-87712)	70330 (56077-88277)	25151 (22533-27879)	30334 (27086-33921)	29892 (25721-34110)	30156 (22996-39007)
Croatia	710 (690-729)	382 (370-396)	234 (224-244)	176 (159-196)	438 (417-459)	251 (238-265)	152 (143-162)	105 (90-121)
Cuba	2374 (2182-2569)	1295 (1186-1406)	792 (727-861)	591 (506-704)	1191 (1091-1296)	624 (568-682)	361 (329-396)	246 (209-297)
Cyprus	164 (154-175)	83 (77-90)	45 (40-50)	29 (20-42)	91 (76-105)	45 (39-52)	26 (22-30)	16 (11-24)
Czechia	1555 (1523-1590)	489 (471-507)	378 (364-393)	350 (328-374)	921 (883-961)	239 (224-254)	189 (177-202)	179 (163-195)
Democratic People’s Republic of Korea	17956 (14078-22986)	24921 (19566-31847)	10226 (8028-13102)	6116 (4797-7834)	8970 (6100-12626)	10858 (6710-16284)	5575 (3451-8194)	3376 (1991-5034)
Democratic Republic of the Congo	282491 (254988-311342)	331074 (300983-366445)	319024 (264964-377367)	290859 (187364-439624)	67152 (57342-77959)	83892 (71721-97268)	94325 (74603-115942)	96760 (58228-154141)
Denmark	543 (527-563)	376 (363-391)	255 (243-267)	232 (214-251)	272 (256-289)	230 (217-244)	175 (165-186)	187 (155-214)
Djibouti	2516 (2162-2922)	2254 (1933-2661)	1623 (1266-2070)	1173 (693-1910)	1103 (914-1327)	986 (805-1197)	796 (605-1045)	623 (357-1063)
Dominica	26 (24-27)	21 (20-23)	22 (20-24)	33 (30-41)	16 (15-18)	16 (14-17)	17 (16-19)	27 (23-32)
Dominican Republic	12502 (11733-13321)	8518 (7851-9212)	7051 (6073-8172)	5768 (3998-8387)	5155 (4665-5669)	4908 (4459-5393)	4721 (4003-5539)	3989 (2709-5884)
Ecuador	16173	9293	5854	4696	6796	4675	2978	2379

	Number of under-five deaths				Number of neonatal deaths			
	1990	2000	2010	2019	1990	2000	2010	2019
	(14642-17849)	(8258-10460)	(5155-6655)	(4278-5141)	(5829-7816)	(4026-5399)	(2543-3469)	(2102-2676)
Egypt	158216 (150901-166200)	82642 (77348-87895)	62792 (55903-70720)	52291 (36884-75075)	61843 (57404-66578)	39847 (36661-43248)	36190 (31730-41222)	28710 (19792-41788)
El Salvador	9753 (8917-10634)	4893 (4401-5424)	2344 (1899-2886)	1552 (992-2425)	3697 (3257-4252)	2166 (1874-2464)	1142 (908-1443)	765 (472-1252)
Equatorial Guinea	2973 (2629-3350)	3685 (3300-4200)	3782 (2988-4709)	3519 (2121-5766)	848 (656-1093)	1111 (901-1383)	1242 (879-1677)	1289 (660-2333)
Eritrea	13887 (12766-15131)	6371 (5835-6943)	6172 (5185-7350)	4247 (2569-7032)	3172 (2735-3623)	2047 (1794-2315)	2465 (2016-3045)	1863 (1074-3205)
Estonia	386 (372-400)	140 (133-147)	69 (64-74)	33 (28-39)	206 (192-219)	67 (61-73)	32 (29-36)	14 (12-18)
Eswatini	2125 (1884-2382)	3524 (3186-3895)	2810 (2396-3290)	1468 (974-2206)	686 (577-808)	695 (602-800)	641 (523-785)	548 (339-863)
Ethiopia	432253 (402465-464882)	392688 (366419-421366)	252415 (232170-274505)	177849 (145985-215961)	134342 (120398-149406)	139801 (125597-154390)	119835 (107380-133501)	98795 (80029-122189)
Fiji	616 (523-727)	455 (433-477)	448 (432-466)	482 (431-540)	260 (204-328)	182 (162-202)	183 (162-205)	202 (168-242)
Finland	428 (413-444)	249 (239-259)	180 (171-188)	122 (111-135)	247 (234-260)	142 (134-151)	100 (93-106)	70 (62-79)
France	6773 (6634-6914)	4029 (3948-4115)	3402 (3336-3473)	3251 (3082-3446)	2705 (2598-2818)	2076 (2000-2154)	1814 (1748-1880)	1920 (1773-2087)
Gabon	3150 (2708-3651)	3359 (2906-3951)	3184 (2602-3836)	2807 (1762-4469)	1108 (912-1336)	1166 (967-1419)	1346 (1057-1693)	1359 (821-2230)
Gambia	6874 (6207-7632)	6080 (5540-6675)	5130 (4582-5716)	4504 (3778-5341)	2036 (1563-2511)	2121 (1759-2467)	2414 (2057-2841)	2427 (1943-3027)
Georgia	4432 (3983-4949)	2035 (1794-2345)	794 (743-850)	513 (441-604)	2269 (1984-2593)	1204 (1040-1408)	574 (530-621)	258 (216-310)
Germany	7130 (7005-7264)	4053 (3982-4125)	2821 (2770-2871)	2959 (2829-3100)	2786 (2679-2894)	2074 (2004-2143)	1559 (1506-1611)	1788 (1677-1905)
Ghana	72554 (68666-76648)	66595 (62857-70380)	53890 (50056-57919)	40168 (32278-49574)	25012 (22890-27249)	24858 (22698-26923)	23883 (21751-26229)	20399 (16164-25615)
Greece	1126 (1097-1156)	686 (664-709)	419 (403-436)	302 (266-341)	692 (666-718)	421 (402-440)	226 (213-239)	178 (154-204)
Grenada	65 (60-69)	30 (27-33)	28 (25-31)	30 (25-37)	34 (31-38)	15 (13-17)	17 (15-19)	20 (16-25)
Guatemala	28205 (26553-29968)	21123 (19607-22758)	14005 (12563-15644)	10364 (8046-13247)	10169 (9202-11167)	8745 (7868-9664)	6575 (5744-7476)	5180 (3904-6782)
Guinea	66821 (62435-71392)	57583 (53718-61642)	46599 (42716-51007)	44114 (36976-53009)	18587 (16516-20800)	16520 (14644-18552)	14072 (12274-16075)	13960 (10566-18211)
Guinea-Bissau	9752 (8702-10905)	8454 (7629-9407)	6514 (5398-7888)	5111 (3224-7800)	2886 (2450-3363)	2759 (2370-3189)	2638 (2134-3279)	2329 (1410-3704)
Guyana	1282 (1161-1423)	908 (818-1008)	595 (476-745)	453 (281-733)	647 (561-740)	516 (450-591)	358 (280-459)	287 (175-477)
Haiti	37390 (34966-39977)	27498 (25343-29650)	58767 (52566-66029)	16890 (12803-22616)	10148 (8855-11530)	8000 (6964-9120)	8474 (7370-9737)	6827 (4729-9740)
Honduras	10811 (9986-11695)	8145 (7454-8916)	4877 (4233-5619)	3480 (2309-5207)	4176 (3656-4690)	3857 (3447-4318)	2654 (2227-3173)	1907 (1217-2995)
Hungary	2139 (2090-2189)	990 (961-1018)	557 (537-578)	335 (294-381)	1384 (1340-1430)	552 (529-576)	311 (295-328)	182 (157-211)
Iceland	28 (26-31)	17 (15-19)	12 (10-14)	8 (6-11)	15 (14-17)	9 (7-10)	6 (5-7)	4 (3-6)
India	3419688 (3306718-3541320)	2532713 (2436908-2630588)	1527855 (1456287-1606111)	824448 (737541-912680)	1579257 (1498204-1666710)	1254908 (1187315-1324747)	832286 (781864-884340)	522249 (463296-581137)
Indonesia	392965	236672	169428	114994	143435	104919	87474	59591

	Number of under-five deaths				Number of neonatal deaths			
	1990	2000	2010	2019	1990	2000	2010	2019
	(375095-412522)	(224865-249469)	(157161-183262)	(96549-138932)	(132970-154309)	(97174-112746)	(80052-95630)	(49081-72909)
Iran (Islamic Republic of)	103324 (95498-112402)	40392 (36542-44849)	25904 (21941-30598)	21248 (13160-34592)	45514 (32417-55935)	21810 (18191-25680)	16408 (12822-21090)	13075 (7351-22922)
Iraq	34488 (31550-37647)	36067 (32977-39478)	34217 (30223-38755)	28801 (22213-36957)	17260 (15360-19215)	19821 (17873-22142)	19950 (17367-22973)	17203 (13124-22438)
Ireland	493 (475-512)	387 (372-403)	298 (286-311)	203 (172-236)	246 (232-260)	222 (210-235)	179 (169-190)	128 (107-151)
Israel	1138 (1112-1166)	849 (828-871)	696 (677-715)	624 (591-659)	623 (598-649)	452 (432-472)	373 (356-391)	329 (306-354)
Italy	5429 (5326-5534)	2964 (2909-3020)	2226 (2184-2270)	1432 (1321-1553)	3594 (3501-3691)	1847 (1796-1900)	1342 (1302-1383)	858 (774-950)
Jamaica	1871 (1601-2213)	1258 (1086-1458)	910 (700-1208)	652 (376-1138)	1235 (972-1528)	946 (798-1122)	652 (478-889)	461 (251-843)
Japan	8358 (8207-8512)	5295 (5196-5395)	3547 (3476-3619)	2343 (2232-2461)	3281 (3168-3393)	2061 (1989-2134)	1211 (1163-1261)	782 (726-842)
Jordan	4465 (4169-4762)	4305 (3990-4655)	4142 (3641-4712)	3355 (2512-4494)	2538 (2298-2782)	2622 (2376-2903)	2513 (2151-2924)	1974 (1419-2729)
Kazakhstan	19971 (18128-21978)	10050 (9174-10983)	7300 (7138-7466)	3976 (3836-4124)	8536 (7410-9755)	5372 (4707-6110)	4343 (4102-4578)	1757 (1624-1894)
Kenya	100018 (93856-106737)	120720 (112277-130204)	84323 (76109-93469)	63623 (47100-87435)	27830 (24827-30887)	36541 (32827-40504)	36195 (31667-41068)	31343 (22353-44268)
Kiribati	244 (211-282)	182 (161-206)	190 (150-241)	164 (95-279)	93 (72-120)	74 (60-92)	78 (51-108)	72 (33-133)
Kuwait	825 (799-852)	540 (523-556)	596 (580-614)	448 (406-496)	468 (437-506)	290 (273-307)	322 (305-341)	251 (221-286)
Kyrgyzstan	8821 (7822-9952)	5327 (4743-5821)	4119 (3997-4230)	2824 (2693-2985)	3315 (2744-3939)	2166 (1810-2523)	2505 (2271-2732)	1880 (1569-2183)
Lao People's Democratic Republic	26757 (24639-29059)	18262 (16790-19949)	11310 (9938-12843)	7520 (5580-9864)	8632 (7697-9698)	6470 (5796-7267)	4802 (4137-5570)	3637 (2629-4903)
Latvia	640 (619-663)	278 (264-292)	174 (163-185)	74 (58-93)	301 (281-321)	138 (127-149)	93 (85-101)	37 (29-49)
Lebanon	2497 (2236-2791)	1675 (1417-1986)	849 (540-1274)	839 (419-1617)	1634 (1415-1878)	980 (751-1218)	514 (288-821)	485 (203-1003)
Lesotho	5054 (4582-5604)	6523 (5981-7102)	5691 (5049-6402)	4831 (3326-6954)	2304 (2015-2628)	2291 (2056-2540)	2358 (2042-2717)	2401 (1594-3567)
Liberia	24731 (22735-27005)	20532 (19072-22166)	14009 (12752-15390)	13365 (10474-17124)	5548 (4782-6400)	5343 (4663-6073)	4714 (4107-5367)	5239 (3959-6947)
Libya	5447 (4653-6378)	3258 (3076-3450)	2215 (1970-2491)	1448 (899-2303)	2730 (2286-3261)	1844 (1566-2198)	1266 (978-1616)	803 (456-1365)
Lithuania	832 (801-862)	375 (357-395)	191 (179-203)	106 (93-120)	429 (402-458)	151 (138-165)	91 (82-101)	58 (49-68)
Luxembourg	40 (37-44)	25 (23-28)	17 (15-19)	18 (14-23)	20 (17-22)	13 (11-15)	9 (8-10)	9 (7-13)
Madagascar	76971 (71902-82551)	67044 (62133-72381)	49789 (44462-55524)	43110 (34379-54400)	20099 (18027-22452)	20116 (18022-22357)	17910 (15623-20528)	17607 (13636-22750)
Malawi	100072 (94410-105943)	80433 (75810-85464)	48615 (44347-53500)	25712 (16713-37565)	21395 (19167-23676)	18821 (16847-20794)	16536 (14699-18664)	12469 (7995-18795)
Malaysia	8266 (8052-8481)	5253 (5155-5352)	3878 (3807-3951)	4513 (4296-4747)	3862 (3318-4463)	2501 (2214-2823)	2022 (1847-2191)	2448 (2208-2696)
Maldives	783 (716-859)	243 (220-267)	99 (94-105)	54 (47-61)	360 (287-435)	132 (112-152)	56 (49-63)	34 (29-41)
Mali	91308 (86010-96891)	94723 (89104-100885)	87037 (80406-94560)	73632 (59576-91137)	27654 (24967-30707)	27196 (24460-30217)	27030 (23891-30475)	25958 (19762-33629)
Malta	62 (58-66)	34 (31-37)	27 (24-29)	30 (24-38)	41 (38-45)	22 (20-24)	18 (16-20)	20 (16-26)
Marshall Islands	98 (84-115)	76 (66-89)	65 (54-79)	44 (27-70)	39 (31-49)	34 (28-42)	29 (21-39)	21 (12-36)

	Number of under-five deaths				Number of neonatal deaths			
	1990	2000	2010	2019	1990	2000	2010	2019
Mauritania	9295 (8377-10295)	10903 (9689-12285)	11849 (8168-17196)	10699 (5541-20412)	3795 (3297-4389)	4318 (3637-5079)	4895 (3172-7541)	4820 (2337-9706)
Mauritius	505 (483-529)	381 (361-401)	224 (210-239)	206 (186-228)	325 (308-344)	249 (234-265)	140 (129-151)	130 (117-147)
Mexico	108864 (101435-116897)	66468 (61706-71458)	43652 (41386-46247)	31368 (28454-34503)	53599 (48097-59371)	33505 (30106-37108)	20569 (19162-22119)	18906 (16951-21068)
Micronesia (Federated States of)	179 (144-223)	172 (117-253)	100 (51-194)	75 (31-185)	81 (56-114)	77 (44-128)	49 (22-105)	41 (15-108)
Monaco	3 (3-3)	2 (2-2)	2 (2-2)	2 (1-2)	2 (1-2)	1 (1-1)	1 (1-1)	1 (0-1)
Mongolia	7607 (7010-8277)	3094 (2835-3367)	1937 (1883-1994)	1183 (1142-1226)	2070 (1799-2369)	1089 (948-1239)	784 (728-844)	611 (571-651)
Montenegro	170 (161-178)	121 (114-127)	52 (48-56)	17 (14-21)	110 (100-120)	74 (66-80)	34 (30-38)	10 (7-12)
Morocco	57460 (53532-61683)	31607 (28737-34676)	21494 (18932-24280)	14511 (10954-19117)	26097 (23682-28676)	17206 (15398-19242)	13741 (11909-15827)	9187 (6831-12280)
Mozambique	140673 (130058-152811)	127995 (118489-138312)	97698 (88497-107972)	81507 (57433-119361)	37238 (33437-41652)	36753 (33153-40728)	32337 (28234-36810)	32259 (22260-47831)
Myanmar	130235 (119555-142698)	100470 (92778-108964)	64480 (55915-73747)	41896 (29188-58635)	53719 (46418-62998)	42752 (37656-48276)	28284 (23500-33384)	21134 (13909-30840)
Namibia	3769 (3433-4154)	4181 (3767-4679)	3104 (2526-3866)	2954 (1731-5369)	1494 (1312-1700)	1248 (1109-1422)	1295 (1023-1638)	1343 (758-2514)
Nauru	17 (11-28)	14 (12-16)	12 (9-16)	9 (5-17)	9 (5-15)	8 (7-10)	8 (5-11)	6 (3-11)
Nepal	98952 (92915-105331)	61174 (57222-65507)	28667 (25928-31686)	17296 (13258-22837)	42695 (39093-46464)	30395 (27954-33119)	16603 (14849-18538)	11128 (8447-14827)
Netherlands	1582 (1554-1611)	1217 (1194-1243)	811 (792-830)	696 (667-728)	884 (853-915)	756 (730-782)	506 (487-525)	446 (422-471)
New Zealand	631 (615-648)	413 (400-426)	387 (375-401)	283 (242-325)	250 (235-265)	195 (184-207)	205 (193-217)	157 (127-189)
Nicaragua	9944 (9229-10695)	5286 (4787-5816)	3264 (2911-3572)	2212 (1927-2590)	3430 (3044-3843)	2257 (1955-2563)	1860 (1616-2121)	1352 (1160-1595)
Niger	137305 (128558-146618)	127077 (118014-136923)	94589 (81292-109051)	81635 (52383-126519)	24379 (21723-27322)	26194 (23099-29578)	25077 (20487-30262)	25861 (15886-41560)
Nigeria	847558 (792474-903271)	921271 (866360-977885)	857975 (787673-933600)	857899 (674948-1117668)	209193 (187632-232226)	244605 (220477-269484)	249215 (221108-279805)	269897 (206717-359081)
Niue	1 (1-1)	1 (1-1)	1 (0-2)	1 (0-2)	0 (0-1)	0 (0-1)	0 (0-1)	0 (0-1)
Norway	496 (480-513)	283 (271-295)	196 (186-205)	145 (132-159)	231 (218-245)	155 (146-165)	112 (105-120)	83 (74-93)
Oman	2710 (2377-3092)	932 (819-1068)	772 (750-796)	1034 (931-1147)	1176 (977-1390)	421 (324-526)	371 (275-464)	477 (330-635)
Pakistan	594968 (573624-618001)	520226 (497821-542755)	460168 (432339-489531)	399418 (342642-465183)	284059 (263626-304280)	280411 (262210-299105)	269460 (249030-290251)	248342 (208335-294149)
Palau	12 (10-14)	9 (8-11)	6 (4-9)	4 (2-8)	7 (5-9)	5 (3-7)	3 (2-5)	2 (1-5)
Panama	1977 (1736-2209)	1826 (1573-2095)	1514 (1087-2098)	1175 (644-2146)	1151 (908-1371)	1080 (772-1357)	883 (544-1329)	680 (328-1326)
Papua New Guinea	13272 (12089-14515)	13656 (12283-15219)	12368 (10529-14535)	10393 (7491-14257)	5403 (4726-6108)	6091 (5320-6979)	5716 (4749-6851)	5160 (3555-7287)
Paraguay	6311 (5677-7017)	4840 (4050-5800)	3645 (2463-5550)	2774 (1467-5488)	3085 (2684-3534)	2602 (2116-3196)	2048 (1338-3218)	1555 (783-3185)
Peru	53823 (51162-56653)	23759 (22394-25189)	12031 (11234-12890)	7563 (6135-9418)	18905 (17452-20464)	9793 (8945-10668)	5728 (5234-6267)	3653 (2882-4667)

	Number of under-five deaths				Number of neonatal deaths			
	1990	2000	2010	2019	1990	2000	2010	2019
Philippines	113076 (105522-121107)	85573 (79683-92156)	74512 (67728-82217)	59751 (45268-79169)	39410 (35523-43507)	37714 (34235-41560)	35325 (31431-39752)	28992 (21602-38848)
Poland	10054 (9835-10274)	3621 (3539-3703)	2351 (2298-2405)	1624 (1568-1681)	6301 (6123-6480)	2179 (2111-2245)	1417 (1371-1463)	997 (954-1041)
Portugal	1703 (1667-1742)	808 (786-833)	375 (361-391)	295 (275-316)	821 (785-859)	379 (358-401)	204 (192-217)	156 (141-172)
Qatar	218 (205-233)	143 (137-151)	176 (168-184)	172 (151-196)	119 (106-135)	78 (71-84)	94 (85-102)	90 (76-107)
Republic of Korea	10152 (9602-10708)	4448 (4310-4596)	1880 (1846-1916)	1200 (1134-1269)	4839 (4220-5610)	1938 (1776-2109)	798 (727-869)	565 (505-628)
Republic of Moldova	2770 (2380-3218)	1567 (1296-1866)	768 (669-923)	584 (433-781)	1508 (1273-1782)	991 (812-1191)	574 (491-695)	428 (309-588)
Republic of North Macedonia	1275 (1234-1317)	424 (404-444)	232 (218-247)	137 (121-155)	606 (568-647)	240 (224-257)	152 (140-165)	87 (74-100)
Romania	10420 (10155-10701)	4675 (4555-4802)	2694 (2624-2768)	1317 (1259-1374)	5113 (4012-6488)	2268 (1875-2671)	1176 (958-1385)	644 (545-751)
Russian Federation	45302 (44419-46185)	25391 (24895-25898)	17911 (17570-18258)	10556 (10217-10897)	21771 (15893-28576)	11934 (10202-13676)	7857 (6619-9082)	4751 (4017-5483)
Rwanda	47944 (45011-51165)	51321 (47503-55495)	21667 (18994-24686)	13310 (7648-23261)	12951 (11648-14314)	12241 (10851-13740)	8023 (6820-9347)	6261 (3511-11062)
Saint Kitts and Nevis	26 (24-29)	19 (17-21)	13 (11-15)	11 (8-15)	16 (14-18)	13 (11-15)	9 (7-10)	7 (5-10)
Saint Lucia	85 (82-90)	56 (53-59)	44 (41-47)	48 (42-56)	47 (43-51)	34 (31-37)	29 (27-32)	28 (23-33)
Saint Vincent and the Grenadines	64 (60-68)	49 (45-53)	35 (32-39)	23 (18-29)	34 (31-38)	28 (26-31)	22 (20-25)	14 (11-18)
Samoa	162 (142-185)	111 (97-125)	96 (81-112)	72 (49-104)	89 (66-114)	61 (42-81)	53 (34-73)	39 (22-64)
San Marino	4 (3-5)	2 (1-2)	1 (1-1)	0 (0-1)	2 (1-3)	1 (1-1)	0 (0-1)	0 (0-0)
Sao Tome and Principe	495 (438-561)	460 (401-528)	287 (226-358)	198 (122-323)	125 (97-157)	128 (96-158)	115 (86-154)	94 (53-167)
Saudi Arabia	25226 (20986-30084)	12073 (10787-13518)	7206 (6175-9125)	3964 (2972-5378)	12496 (8768-16830)	6700 (4671-8779)	4059 (2586-5744)	2170 (1247-3346)
Senegal	42929 (40806-45141)	47921 (45140-51036)	31047 (28693-33530)	24651 (19201-31643)	12869 (11883-13927)	14583 (13216-15967)	13045 (11853-14339)	12156 (9356-15921)
Serbia	4019 (3919-4123)	1519 (1475-1566)	720 (693-748)	441 (356-546)	2447 (2216-2669)	918 (863-971)	443 (415-471)	265 (208-333)
Seychelles	27 (25-30)	21 (19-23)	24 (21-26)	23 (17-30)	18 (16-21)	13 (12-15)	15 (13-17)	14 (10-19)
Sierra Leone	49148 (45390-53101)	45600 (42543-48839)	38814 (35805-41990)	27580 (22873-33217)	10268 (8900-11891)	10231 (9003-11713)	9779 (8578-11103)	8013 (6227-10140)
Singapore	409 (392-427)	200 (188-212)	138 (128-147)	128 (113-145)	222 (208-236)	81 (74-89)	55 (49-61)	46 (38-54)
Slovakia	1200 (1179-1222)	539 (526-552)	394 (382-405)	325 (309-344)	693 (667-720)	273 (259-286)	190 (179-201)	164 (151-177)
Slovenia	241 (233-250)	97 (92-101)	67 (64-71)	42 (37-48)	129 (121-137)	57 (52-61)	38 (35-41)	23 (19-27)
Solomon Islands	470 (411-535)	438 (392-490)	456 (387-537)	416 (275-614)	183 (148-225)	190 (161-225)	197 (158-243)	176 (105-279)
Somalia	58841 (49095-71747)	71078 (56368-91556)	79997 (56301-116810)	72126 (37180-144567)	15464 (11814-20087)	19072 (14163-26330)	22984 (13968-37780)	23723 (10435-51887)
South Africa	64220 (57235-71842)	72667 (68120-77677)	59647 (56585-62777)	40631 (35967-45708)	21966 (18452-25966)	15288 (13028-18605)	13396 (11122-15409)	13489 (10816-16840)

	Number of under-five deaths				Number of neonatal deaths			
	1990	2000	2010	2019	1990	2000	2010	2019
South Sudan	62176 (51678-72833)	45908 (39185-53518)	36498 (25973-48685)	36916 (14811-74426)	16214 (11331-21793)	14829 (11008-19415)	15028 (9379-23084)	14976 (5279-34421)
Spain	3759 (3688-3831)	2119 (2079-2158)	1908 (1873-1944)	1221 (1130-1326)	1994 (1910-2077)	1123 (1072-1173)	1042 (997-1086)	698 (607-797)
Sri Lanka	7892 (7737-8042)	5714 (5607-5825)	4202 (4097-4309)	2378 (1945-2883)	4801 (4579-5021)	3329 (3186-3470)	2305 (2197-2414)	1416 (1133-1766)
State of Palestine	4138 (3819-4478)	3579 (3292-3880)	3096 (2666-3617)	2771 (1985-3963)	2113 (1879-2372)	1985 (1776-2204)	1739 (1468-2069)	1528 (1053-2244)
Sudan	105625 (98107-113855)	108266 (99668-117536)	90839 (79995-102883)	78028 (57099-104633)	36076 (32222-40233)	39942 (35336-44626)	38951 (33574-45222)	37126 (26558-51113)
Suriname	503 (429-590)	348 (294-406)	250 (200-311)	191 (130-292)	233 (148-308)	193 (151-238)	160 (122-209)	119 (74-191)
Sweden	799 (776-825)	379 (365-393)	341 (328-354)	305 (285-327)	408 (388-429)	213 (201-225)	183 (172-194)	165 (151-180)
Switzerland	650 (632-670)	435 (421-449)	358 (345-371)	353 (314-395)	314 (299-330)	264 (252-277)	251 (239-262)	244 (214-278)
Syrian Arab Republic	16210 (14690-17843)	11637 (10461-12941)	10851 (8982-12723)	9195 (5261-12114)	7232 (6312-8278)	6257 (5450-7166)	5725 (4645-6918)	4545 (2589-6261)
Tajikistan	21209 (19349-23254)	15889 (14210-17728)	9919 (8635-11433)	9377 (6082-14771)	6503 (5442-7844)	5417 (4600-6398)	4820 (4048-5810)	4183 (2361-6905)
Thailand	40239 (37741-42712)	20327 (18256-22172)	10830 (9543-12216)	6444 (5538-8197)	22168 (17460-25365)	11716 (8744-14276)	6284 (4443-7964)	3759 (2522-5173)
Timor-Leste	5161 (4662-5708)	3763 (3419-4144)	2075 (1670-2560)	1645 (987-2752)	1724 (1461-2050)	1312 (1124-1515)	822 (578-1087)	742 (389-1345)
Togo	21883 (20347-23539)	21938 (20407-23588)	20480 (18588-22532)	17331 (13953-21420)	6697 (5983-7498)	7073 (6322-7894)	6982 (6099-7951)	6563 (5044-8445)
Tonga	66 (56-79)	50 (42-59)	48 (40-57)	42 (31-56)	31 (24-42)	22 (17-27)	20 (15-26)	19 (13-27)
Trinidad and Tobago	794 (663-932)	527 (397-728)	462 (266-885)	312 (137-733)	511 (413-612)	352 (250-498)	310 (169-601)	202 (86-489)
Tunisia	12010 (10547-13620)	5068 (4425-5783)	3453 (3296-3610)	3398 (3129-3695)	5908 (5055-6866)	3155 (2685-3667)	2405 (2214-2591)	2381 (2138-2650)
Turkey	103389 (96637-110649)	52806 (48685-57400)	23676 (21774-25556)	13149 (11651-14925)	45994 (41418-51031)	25558 (22693-28674)	11800 (10395-13225)	6905 (6067-7897)
Turkmenistan	10254 (8980-11690)	7483 (6497-8636)	5356 (4204-6751)	5793 (4141-8067)	3513 (2938-4197)	3171 (2661-3777)	2959 (2286-3789)	3233 (2251-4544)
Tuvalu	14 (12-17)	10 (9-11)	9 (6-12)	7 (4-13)	7 (6-9)	6 (5-7)	6 (4-8)	5 (2-9)
Uganda	147695 (139217-156611)	160536 (151140-170395)	107995 (100014-116448)	74053 (56345-96855)	33549 (29961-37289)	36959 (32921-41213)	36708 (32768-41252)	32914 (24097-44724)
Ukraine	12850 (11577-14682)	7572 (6928-8466)	5729 (5554-5904)	3514 (3340-3697)	7747 (6553-9246)	4507 (3799-5281)	3477 (2639-4124)	2047 (1490-2519)
United Arab Emirates	777 (659-908)	579 (547-613)	767 (746-790)	750 (643-872)	397 (328-479)	313 (277-364)	427 (384-485)	398 (328-480)
United Kingdom	7223 (7084-7370)	4612 (4527-4700)	4080 (4004-4159)	3313 (3148-3487)	3490 (3380-3605)	2636 (2561-2714)	2430 (2363-2502)	2142 (2015-2275)
United Republic of Tanzania	175465 (165418-186338)	172198 (161805-183603)	124464 (113265-137204)	103222 (77641-140529)	44425 (40682-48694)	47036 (42994-51573)	44220 (39362-50097)	42814 (31593-59216)
United States of America	43948 (43185-44759)	33310 (32759-33854)	30021 (29532-30516)	25352 (23785-27005)	22777 (22153-23442)	18384 (17913-18870)	16696 (16265-17136)	14546 (13500-15658)
Uruguay	1295 (1268-1321)	921 (899-943)	516 (499-533)	337 (315-359)	686 (592-774)	455 (423-488)	232 (210-252)	200 (182-220)
Uzbekistan	50580	35606	21186	12045	22012	15846	11970	6827

	Number of under-five deaths				Number of neonatal deaths			
	1990	2000	2010	2019	1990	2000	2010	2019
	(44678-57178)	(30992-41245)	(17231-25685)	(9807-14796)	(18366-26044)	(13100-19110)	(8485-15881)	(4649-9317)
Vanuatu	184 (154-219)	173 (147-204)	222 (175-282)	224 (136-368)	85 (65-116)	78 (58-97)	100 (71-135)	100 (51-182)
Venezuela (Bolivarian Republic of)	16484 (16024-16942)	12361 (12108-12624)	10088 (9888-10291)	12620 (10422-15417)	7214 (6802-7646)	6372 (6056-6693)	6226 (5970-6485)	7520 (5770-9511)
Viet Nam	98618 (91061-107317)	42632 (35985-49625)	34215 (30962-37426)	31689 (25637-40419)	45456 (40149-52315)	21505 (17644-25552)	18238 (15500-21047)	16587 (12143-22593)
Yemen	74760 (70188-79853)	64423 (59222-69999)	43746 (37653-50578)	50312 (30260-81616)	27149 (24732-29777)	25454 (22874-28288)	21467 (18229-25226)	23220 (13792-38118)
Zambia	62826 (59004-66916)	68180 (63726-73020)	43540 (40100-47286)	38460 (30438-48353)	13191 (11919-14508)	15977 (14516-17614)	14498 (13038-16097)	14902 (11528-19262)
Zimbabwe	28408 (25928-30950)	34362 (31142-37661)	39169 (35938-42786)	24166 (18143-31818)	9220 (8210-10296)	9945 (8930-11017)	14781 (13234-16547)	11283 (8219-15216)

3.4. Table A.4 Series in U5MR database

Note: The series name refers to a specific data collection source and estimation method. There are several sources – including VR, censuses and sample surveys – where:

- VR (direct) series are based on birth and death registration data
- Survey (direct) series are based on data on births and deaths from full birth histories
- Census (direct) series are based on household deaths in the previous 12 months
- Census or survey (indirect) series are based on summary birth histories (i.e., mean number of children ever born and children surviving by age of mother or time since first birth)

	Data series	Inclusion
Afghanistan	National Demographic and Family Guidance Survey 1972 (Household Deaths)	0
	National Demographic and Family Guidance Survey 1972 (Indirect)	1
	Census 1979 (Household Deaths)	1
	Multiple Indicator Cluster Survey 1997 (Indirect)	1
	Multiple Indicator Cluster Survey 2000 (Indirect)	0
	Multiple Indicator Cluster Survey 2003 (Direct)	0
	Multiple Indicator Cluster Survey 2003 (Indirect)	0
	Afghanistan Health Survey 2006-2007 (Indirect)	1
	National Risk and Vulnerability Assessment Survey 2007-2008 (Indirect)	1
	Afghanistan Mortality Survey (AMS) Excluding South Zone 2010 (Direct)	0
	Afghanistan Mortality Survey (AMS) Excluding South Zone (Household data (5 year)) 2010 (Household Deaths)	1
	Afghanistan Mortality Survey (AMS) Excluding South Zone (NN adjusted) 2010 (Direct)	1
	Multiple Indicator Cluster Survey 2010-2011 (Indirect)	1
	Multiple Indicator Cluster Survey Excluding South Zone 2010-2011 (Indirect)	0
	Afghanistan Living Conditions Survey 2011-2012 (Indirect)	0
	Demographic and Health Survey 2015 (Direct)	0
	Demographic and Health Survey (NN adjusted) 2015 (Direct)	1
	Afghanistan Health Survey 2015 2015 (Direct)	0
	Afghanistan Health Survey 2018 (Direct)	0
	Afghanistan Health Survey (NN adjusted) 2018 (Direct)	1
Albania	Multiple Indicator Cluster Survey 2000 (Indirect)	1
	Reproductive Health Survey 2002 (Direct)	1
	Multiple Indicator Cluster Survey 2005 (Indirect)	1
	Demographic and Health Survey 2008-2009 (Direct)	1
	Demographic and Health Survey 2008-2009 (Indirect)	0
	Census 2011 (Indirect)	0
	Demographic and Health Survey 2017-2018 (Direct)	0
	UNPD Demographic Yearbook Data 2020 version 2020 (VR)	0
	VR Submitted to WHO/UNIGME 2020 version 2020 (VR)	1
Algeria	Life Tables (Life Table)	1
	Algeria Fertility Survey 1970 (Indirect)	1
	Demographic Survey 1970 (Direct)	1
	National Fertility Survey 1986 (Direct)	1
	PAPCHILD Maternal and Child Health Survey 1992 (Direct)	1
	Multiple Indicator Cluster Survey 1995 (Direct)	1
	Multiple Indicator Cluster Survey 2000 (Indirect)	1
	PAPFAM Family Health Survey 2002 (Direct)	1
	Multiple Indicator Cluster Survey 2006 (Indirect)	1
	Multiple Indicator Cluster Survey 2012-2013 (Direct)	0
	VR from Demographie Algerienne 2012 2011 (VR)	0

	Data series	Inclusion
	VR from Demographie Algerienne 2013 2012 (VR)	0
	VR from Demographie Algerienne 2014 2013 (VR)	0
	VR from Demographie Algerienne 2014 2014 (VR)	0
	VR from Demographie Algerienne 2015 2015 (VR)	0
	VR from Demographie Algerienne 2016 2016 (VR)	0
	VR from Demographie Algerienne 2017 2017 (VR)	0
	VR from Demographie Algerienne 2018 2018 (VR)	0
	VR Submitted to WHO/UNIGME 2020 version 2020 (VR)	1
Andorra	Recalculated VR Submitted to WHO/UNIGME 2020 version 2020 (VR)	1
	VR Submitted to WHO/UNIGME 2020 version 2020 (VR)	0
Angola	Census 1940 (Indirect)	0
	Multiple Indicator Cluster Survey 1996 (Indirect)	1
	Multiple Indicator Cluster Survey 2001 (Indirect)	1
	Malaria Indicator Survey 2006-2007 (Indirect)	1
	Household Incomes and Expenditures Survey IBEP 2008-2009 (Indirect)	1
	Malaria Indicator Survey 2011 (Direct)	0
	Census 2014 (Indirect)	0
	Census 2014 (Household Deaths)	0
	Demographic and Health Survey 2015-2016 (Direct)	1
Antigua and Barbuda	Recalculated VR Submitted to WHO/UNIGME 2020 version 2020 (VR)	1
	UNPD Demographic Yearbook Data 2020 version 2020 (VR)	1
	VR Submitted to WHO/UNIGME 2020 version 2020 (VR)	0
Argentina	TABLAS DE VIDA NACIONALES (Life Table)	0
	Census 1970 (Indirect)	0
	Census 1980 (Indirect)	0
	Census 1991 (Indirect)	0
	Census 2001 (Indirect)	0
	Census 2010 (Indirect)	0
	VR Submitted to WHO/UNIGME 2020 version 2020 (VR)	1
Armenia	Census 1989 (Indirect)	0
	Demographic and Health Survey 2000 (Direct)	1
	Demographic and Health Survey 2000 (Indirect)	0
	Census 2001 (Indirect)	1
	Demographic and Health Survey 2005 (Direct)	1
	Demographic and Health Survey 2005 (Indirect)	0
	Demographic and Health Survey 2010 (Direct)	1
	Census 2011 (Household Deaths)	0
	Census 2011 (Indirect)	0
	Demographic and Health Survey 2015-2016 (Direct)	0
	Transmonee Vital Registration Data 2008 version 2008 (VR)	0
	Vital Registration data from The Demographic Handbook of Armenia 2011 2011 (VR)	0
	Vital Registration data from The Demographic Handbook of Armenia 2014 2014 (VR)	0
	Vital Registration data from The Demographic Handbook of Armenia 2016 2016 (VR)	0
	VR Submitted to WHO/UNIGME 2020 version 2020 (VR)	1
Australia	VR Submitted to WHO/UNIGME 2020 version 2020 (VR)	1
Austria	UNPD Demographic Yearbook Data 2020 version 2020 (VR)	1
	VR Submitted to WHO/UNIGME 2020 version 2020 (VR)	1
Azerbaijan	Census 1989 (Indirect)	0
	Census 1999 (Indirect)	0
	Multiple Indicator Cluster Survey 2000 (Indirect)	1
	Reproductive Health Survey 2001 (Direct)	1
	Demographic and Health Survey 2006 (Direct)	1
	Demographic and Health Survey 2006 (Indirect)	0
	Census 2009 (Indirect)	0

	Data series	Inclusion
	Azerbaijan National DHS 2011 (Direct)	1
	Transmonee Vital Registration Data 2008 version 2008 (VR)	0
	VR from Children in Azerbaijan Report 2013 2013 (VR)	0
	VR from Children in Azerbaijan Report 2016 2016 (VR)	0
	Adjusted National Vital Registration Data 2018 (VR)	1
	VR Submitted to WHO/UNIGME 2020 version 2020 (VR)	1
Bahamas	Recalculated VR Submitted to WHO/UNIGME 2020 version 2020 (VR)	1
	VR Submitted to WHO/UNIGME 2020 version 2020 (VR)	0
Bahrain	Census 1965 (Indirect)	0
	Census 1971 (Indirect)	1
	Census 1981 (Indirect)	1
	Child Health Survey 1989 (Direct)	1
	Child Health Survey 1989 (Indirect)	0
	Census 1991 (Indirect)	1
	Gulf Family Health Survey 1995 (Direct)	1
	Gulf Family Health Survey 1995 (Indirect)	0
	Census 2001 (Indirect)	1
	VR Submitted to WHO/UNIGME 2020 version 2020 (VR)	1
Bangladesh	Population Growth Estimation Experiment 1962-1965 (Household Deaths)	0
	Population Growth Estimation Experiment 1965 (Direct)	1
	Retrospective Fertility and Mortality Survey (UN SA) 1974 (Indirect)	1
	World Fertility Survey 1975-1976 (Direct)	1
	World Fertility Survey 1975-1976 (Indirect)	0
	Contraceptive Prevalence Survey 1979-1980 (Indirect)	0
	Contraceptive Prevalence Survey 1981 (Indirect)	1
	Contraceptive Prevalence Survey 1983-1984 (Indirect)	1
	Contraceptive Prevalence Survey 1985-1986 (Indirect)	1
	National Life Tables 1987 (Life Table)	1
	Fertility Survey 1988-1989 (Direct)	1
	Demographic and Health Survey 1993-1994 (Direct)	1
	Demographic and Health Survey 1993-1994 (Indirect)	0
	Health and Demographic Survey 1994 (Indirect)	1
	Demographic and Health Survey 1996-1997 (Direct)	1
	Demographic and Health Survey 1996-1997 (Indirect)	0
	Demographic and Health Survey 1999-2000 (Direct)	1
	Demographic and Health Survey 1999-2000 (Indirect)	0
	Maternal Health Services and Maternal Mortality Survey 2001 (Direct)	1
	Demographic and Health Survey 2004 (Direct)	1
	Demographic and Health Survey 2004 (Indirect)	0
	Demographic and Health Survey 2007 (Direct)	1
	Demographic and Health Survey 2007 (Indirect)	0
	Multiple Indicator Cluster Survey 2009 (Indirect)	1
	Demographic and Health Survey 2011 (Direct)	1
	Census 2011 (Household Deaths)	1
	Multiple Indicator Cluster Survey 2012-2013 (Indirect)	1
	Demographic and Health Survey 2014 (Direct)	1
	Demographic and Health Survey 2017-2018 (Direct)	1
	Multiple Indicator Cluster Survey 2019 (Direct)	1
	Bangladesh Demographic Survey and Vital Registration 1988 (VR)	1
	Report on Sample Vital Registration System 2012 (VR)	1
	Report on Sample Vital Registration System 2018 (VR)	1
Barbados	Recalculated VR Submitted to WHO/UNIGME 2020 version 2020 (VR)	1
	UNPD Demographic Yearbook Data 2020 version 2020 (VR)	1
	VR Submitted to WHO/UNIGME 2020 version 2020 (VR)	0

	Data series	Inclusion
Belarus	Census 1989 (Indirect)	0
	Census 1999 (Indirect)	0
	Multiple Indicator Cluster Survey 2005 (Indirect)	0
	Transmonee Vital Registration Data 2008 version 2008 (VR)	0
	Demographic Yearbook of the Republic of Belarus 2012 (VR)	0
	VR Submitted to WHO/UNIGME 2020 version 2020 (VR)	1
Belgium	UNPD Demographic Yearbook Data 2020 version 2020 (VR)	1
	VR Submitted to WHO/UNIGME 2020 version 2020 (VR)	1
Belize	Family Health Survey 1991 (Direct)	1
	Family Health Survey 1999 (Indirect)	1
	Census 2000 (Indirect)	1
	Multiple Indicator Cluster Survey 2006 (Indirect)	1
	Multiple Indicator Cluster Survey 2011 (Indirect)	1
	Multiple Indicator Cluster Survey 2015-2016 (Direct)	0
	Vital Registration Data from MOH (Abstract of Statistics 2008) 2008 (VR)	0
	UNPD Demographic Yearbook Data 2020 version 2020 (VR)	0
	VR Submitted to WHO/UNIGME 2020 version 2020 (VR)	1
Benin	Survey 1961 (Indirect)	1
	Benin Fertility Survey 1981-1982 (Indirect)	0
	World Fertility Survey 1981-1982 (Direct)	1
	Enquete a passages repetes 1981-1983 (Household Deaths)	1
	Census 1992 (Indirect)	1
	Demographic and Health Survey 1996 (Direct)	1
	Demographic and Health Survey 1996 (Indirect)	0
	Demographic and Health Survey 2001 (Direct)	1
	Demographic and Health Survey 2001 (Indirect)	0
	Demographic and Health Survey 2006 (Direct)	1
	Demographic and Health Survey 2006 (Indirect)	0
	Demographic and Health Survey 2011-2012 (Direct)	0
	Multiple Indicator Cluster Survey 2014 (Direct)	1
	Demographic and Health Survey 2017-2018 (Direct)	1
Bhutan	Demographic Sample Survey 1984 (Direct)	1
	Demographic Sample Survey 1984 (Indirect)	1
	National Health Survey 1994 (Household Deaths)	1
	National Health Survey 2000 (Direct)	1
	Census 2005 (Household Deaths)	1
	Census 2005 (Indirect)	1
	Multiple Indicator Cluster Survey 2010 (Indirect)	1
	National Health Survey 2012 (Direct)	1
	Census 2017 (Household Deaths)	0
Bolivia (Plurinational State of)	EDEN 1975 (Indirect)	1
	Census 1976 (Indirect)	1
	National Demographic Survey 1980 (Household Deaths)	1
	National Demographic Survey 1980 (Indirect)	1
	ENPV 1988 (Indirect)	1
	Demographic and Health Survey 1989 (Direct)	1
	Demographic and Health Survey 1989 (Indirect)	0
	Census 1992 (Household Deaths)	0
	Census 1992 (Indirect)	0
	Demographic and Health Survey 1993-1994 (Direct)	1
	Demographic and Health Survey 1993-1994 (Indirect)	0
	Demographic and Health Survey 1998 (Direct)	1
	Demographic and Health Survey 1998 (Indirect)	0
	Multiple Indicator Cluster Survey 2000 (Indirect)	1

	Data series	Inclusion
	Census 2001 (Household Deaths)	0
	Census 2001 (Indirect)	1
	Demographic and Health Survey 2003 (Direct)	1
	Demographic and Health Survey 2003 (Indirect)	0
	Demographic and Health Survey 2008 (Direct)	1
	Census 2012 (Indirect)	1
	Census 2012 (Household Deaths)	1
	Demographic and Health Survey 2016 (Direct)	1
Bosnia and Herzegovina	VR Submitted to WHO/UNIGME 2020 version 2020 (VR)	1
Botswana	Census 1971 (Indirect)	1
	Census 1981 (Household Deaths)	0
	Census 1981 (Indirect)	1
	Family Health Survey 1984 (Indirect)	1
	Family Health Survey 1988 (Direct)	0
	Family Health Survey 1988 (Indirect)	0
	Family Health Survey (MM adjusted) 1988 (Direct)	1
	Census 1991 (Household Deaths)	0
	Census 1991 (Indirect)	1
	Family Health Survey 1996 (Direct)	0
	Family Health Survey (MM adjusted) 1996 (Direct)	0
	Demographic Survey 1998 (Household Deaths)	0
	Multiple Indicator Cluster Survey 2000 (Indirect)	1
	Census 2001 (Household Deaths)	0
	Census 2001 (Indirect)	1
	Demographic Survey 2006 (Household Deaths)	0
	Demographic Survey 2006 (Indirect)	1
	Family Health Survey 2007-2008 (Direct)	0
	Family Health Survey (MM adjusted) 2007-2008 (Direct)	1
	Census 2011 (Household Deaths)	0
	Census 2011 (Indirect)	0
	Demographic Survey 2017 (Indirect)	1
	Vital Statistics Report from Statistics Botswana 2020 (VR)	0
Brazil	Census 1970 (Indirect)	1
	National Health Survey 1972 (Indirect)	1
	National Health Survey 1973 (Indirect)	1
	National Health Survey 1976 (Indirect)	1
	National Health Survey 1977 (Indirect)	1
	National Health Survey 1978 (Indirect)	1
	Census 1980 (Indirect)	1
	National Health Survey 1984 (Indirect)	1
	Demographic and Health Survey 1986 (Direct)	1
	Demographic and Health Survey 1986 (Indirect)	0
	National Health Survey 1986 (Indirect)	1
	Pesquisa Nacional por Amostra de Domicilios 1986 (Direct)	1
	Census 1991 (Indirect)	1
	Demographic and Health Survey 1996 (Direct)	1
	Demographic and Health Survey 1996 (Indirect)	0
	Census 2000 (Indirect)	1
	Pesquisa Nacional por Amostra de Domicilios 2002 (Indirect)	1
	Pesquisa Nacional por Amostra de Domicilios 2003 (Indirect)	1
	Pesquisa Nacional por Amostra de Domicilios 2004 (Indirect)	1
	Pesquisa Nacional por Amostra de Domicilios 2005 (Indirect)	1

	Data series	Inclusion
	Pesquisa Nacional por Amostra de Domicilios 2006 (Indirect)	1
	Pesquisa Nacional por Amostra de Domicilios 2007 (Indirect)	1
	Pesquisa Nacional por Amostra de Domicilios 2008 (Indirect)	1
	Pesquisa Nacional por Amostra de Domicilios 2009 (Indirect)	1
	Census 2010 (Indirect)	0
	Census 2010 (Household Deaths)	0
	Pesquisa Nacional por Amostra de Domicilios 2011 (Indirect)	1
	Pesquisa Nacional por Amostra de Domicilios 2012 (Indirect)	1
	Pesquisa Nacional por Amostra de Domicilios 2013 (Indirect)	1
	Pesquisa Nacional por Amostra de Domicilios 2014 (Indirect)	1
	Pesquisa Nacional por Amostra de Domicilios 2015 (Indirect)	1
	Data from Information System of Ministry of Health (Busca Ativa) 2018 (VR)	1
	Data from Information System of Ministry of Health (Direct) 2018 (VR)	0
	VR Submitted to WHO/UNIGME 2020 version 2020 (VR)	0
Brunei Darussalam	Census 1960 (Indirect)	1
	Recalculated VR Submitted to WHO/UNIGME 2020 version 2020 (VR)	1
	UNPD Demographic Yearbook Data 2020 version 2020 (VR)	1
	VR Submitted to WHO/UNIGME 2020 version 2020 (VR)	0
Bulgaria	UNPD Demographic Yearbook Data 2020 version 2020 (VR)	1
	VR Submitted to WHO/UNIGME 2020 version 2020 (VR)	1
Burkina Faso	Survey 1960-1961 (Indirect)	1
	Post-Enumeration Survey 1976 (Indirect)	1
	Census 1985 (Household Deaths)	0
	Census 1985 (Indirect)	1
	Survey 1991 (Indirect)	1
	Demographic and Health Survey 1993 (Direct)	1
	Demographic and Health Survey 1993 (Indirect)	0
	Demographic and Health Survey 1998-1999 (Direct)	1
	Demographic and Health Survey 1998-1999 (Indirect)	0
	Demographic and Health Survey 2003 (Direct)	1
	Demographic and Health Survey 2003 (Indirect)	0
	Census 2006 (Household Deaths)	0
	Census 2006 (Indirect)	0
	Multiple Indicator Cluster Survey 2006 (Indirect)	1
	Recensement General de la Population et de l'Habitat (RGPH) 2006 (Household Deaths)	0
	Recensement General de la Population et de l'Habitat (RGPH) 2006 (Indirect)	0
	Global Fund Evaluation Survey 2008 (Household Deaths)	0
	Demographic and Health Survey 2010 (Direct)	1
	Malaria Indicator Survey 2014 (Indirect)	1
	L Enquete sur le Module Demographie et Sante 2015 (Direct)	0
	Malaria Indicator Survey 2017-2018 (Indirect)	1
Burundi	Population Survey 1965 (Household Deaths)	0
	Demographic Survey 1970 (Direct)	1
	Demographic Survey 1970 (Indirect)	0
	Post Census Survey 1979 (Indirect)	1
	Demographic and Health Survey 1987 (Direct)	1
	Demographic and Health Survey 1987 (Indirect)	0
	Census 1990 (Indirect)	1
	Multiple Indicator Cluster Survey 2000 (Indirect)	1
	Multiple Indicator Cluster Survey 2005 (Indirect)	1
	Census 2008 (Indirect)	1
	Census 2008 (Household Deaths)	0
	Demographic and Health Survey 2010-2011 (Direct)	1
	Malaria Indicator Survey 2012 (Indirect)	1

	Data series	Inclusion
	Enquete menages pour le suivi et l'evaluation de l'impact de l'appui au systeme de remboursement du Paquet Minimum des Services de sante 2012 (Indirect)	1
	Demographic and Health Survey 2016-2017 (Direct)	1
Cabo Verde	Census 1960 (Indirect)	1
	Census 1970 (Direct)	1
	Census 1980 (Direct)	1
	Demographic and Reproductive Health Survey 1998 (Direct)	1
	Census 2000 (Direct)	1
	Census 2000 (Household Deaths)	0
	Demographic and Health Survey 2005 (Direct)	1
	Demographic and Health Survey 2005 (Indirect)	0
	Census 2010 (Indirect)	1
	Vital Registration Data from Relatorio Estatistico 2007 2009 Ministry of Health 2007-2009 (VR)	0
	UNPD Vital Registration Data 2010 version 2010 (VR)	0
	VR from Relatorio Estatistico 2012 (VR)	0
	VR Submitted to WHO/UNIGME 2020 version 2020 (VR)	1
Cambodia	Survey 1959 (Household Deaths)	0
	Census extracted from IPUMS 1962 (Indirect)	0
	Census 1998 (Indirect)	1
	National Health Survey 1998 (Direct)	0
	National Health Survey 1998 (Indirect)	1
	Demographic and Health Survey 2000 (Direct)	1
	Demographic and Health Survey 2000 (Indirect)	0
	Inter-censal Population Survey 2004 (Indirect)	1
	Demographic and Health Survey 2005 (Direct)	1
	Demographic and Health Survey 2005 (Indirect)	0
	Census 2008 (Indirect)	0
	Census extracted from IPUMS 2008 (Household Deaths)	0
	Demographic and Health Survey 2010 (Direct)	1
	Demographic and Health Survey 2014 (Direct)	1
Cameroon	Census 1976 (Household Deaths)	0
	World Fertility Survey 1978 (Direct)	1
	World Fertility Survey 1978 (Indirect)	0
	Census 1987 (Household Deaths)	0
	Demographic and Health Survey 1991 (Direct)	0
	Demographic and Health Survey 1991 (Indirect)	0
	Demographic and Health Survey (MM adjusted) 1991 (Direct)	1
	Demographic and Health Survey 1998 (Direct)	0
	Demographic and Health Survey 1998 (Indirect)	0
	Demographic and Health Survey (MM adjusted) 1998 (Direct)	1
	Multiple Indicator Cluster Survey 2000 (Indirect)	1
	Demographic and Health Survey 2004 (Direct)	0
	Demographic and Health Survey 2004 (Indirect)	0
	Demographic and Health Survey (MM adjusted) 2004 (Direct)	1
	Census 2005 (Indirect)	0
	Census 2005 (Household Deaths)	0
	Demographic and Health Survey 2011 (Direct)	0
	Demographic and Health Survey (MM adjusted) 2011 (Direct)	1
	Multiple Indicator Cluster Survey 2014 (Direct)	0
	Multiple Indicator Cluster Survey (MM adjusted) 2014 (Direct)	1
	Demographic and Health Survey 2018 (Direct)	0
	Demographic and Health Survey (MM adjusted) 2018 (Direct)	1
Canada	VR Submitted to WHO/UNIGME 2020 version 2020 (VR)	1
	Survey 1959-1960 (Household Deaths)	0

	Data series	Inclusion
Central African Republic	Census 1975 (Indirect)	1
	Census 1988 (Household Deaths)	0
	Census 1988 (Indirect)	1
	Demographic and Health Survey 1994-1995 (Direct)	0
	Demographic and Health Survey 1994-1995 (Indirect)	0
	Demographic and Health Survey (MM adjusted) 1994-1995 (Direct)	1
	Multiple Indicator Cluster Survey 2000 (Indirect)	1
	Census 2003 (Household Deaths)	0
	Multiple Indicator Cluster Survey 2006 (Indirect)	1
	Multiple Indicator Cluster Survey 2010 (Indirect)	1
	Enquete Nationale Sur la Situation Nutritionnelle et la Mortalite 2012 (Household Deaths)	0
Chad	Census 1993 (Indirect)	0
	Demographic and Health Survey 1996-1997 (Direct)	1
	Demographic and Health Survey 1996-1997 (Indirect)	0
	Multiple Indicator Cluster Survey 2000 (Indirect)	1
	Demographic and Health Survey 2004 (Direct)	1
	Demographic and Health Survey 2004 (Indirect)	0
	Census 2009 (Indirect)	0
	Census 2009 (Household Deaths)	0
	Multiple Indicator Cluster Survey 2010 (Indirect)	1
	Demographic and Health Survey 2014-2015 (Direct)	1
Chile	TABLAS DE VIDA NACIONALES (Life Table)	0
	Census 1970 (Indirect)	0
	Census 1982 (Indirect)	0
	Census 1992 (Indirect)	0
	Census 2002 (Indirect)	0
	Census 2017 (Indirect)	0
	Vital Registration from Ministerio de Salud. Departamento de Estadisticas de Salud.Anexo Salud1 (VR)	0
	VR Submitted to WHO/UNIGME 2020 version 2020 (VR)	1
China	Adjusted Census Deaths (Household Deaths)	1
	National Life Tables (Life Table)	0
	Census 1982 (Indirect)	1
	Population Sample Survey 1982 (Indirect)	1
	Intercensal Population Sample Survey of One-Percent 1987 (Household Deaths)	0
	Intercensal Population Sample Survey of One-Percent 1987 (Indirect)	1
	National Survey on Fertility and Birth Control 1988 (Direct)	1
	Census 1990 (Indirect)	0
	Fertility Sampling Survey 1992 (Direct)	1
	Intercensal Population Sample Survey of One-Percent 1995 (Indirect)	0
	Census 2000 (Indirect)	0
	National Survey on Causes of Death 2004-2005 (Household Deaths)	0
	Intercensal Population Sample Survey of One-Percent 2005 (Direct)	0
	Intercensal Population Sample Survey of One-Percent 2005 (Indirect)	0
	Census 2010 (Household Deaths)	0
	Census 2010 (Indirect)	0
	Intercensal Population Sample Survey of One-Percent 2015 (Indirect)	0
	NHFPC, Death Registration Data 2019 (VR)	1
Colombia	Census 1973 (Indirect)	1
	World Fertility Survey 1976 (Direct)	1
	World Fertility Survey 1976 (Indirect)	0
	Contraceptive Prevalence Survey 1978 (Indirect)	1
	Household Survey (HOG) 1978 (Indirect)	1
	Household Survey (HOG) 1980 (Indirect)	1
	Census 1985 (Indirect)	1

	Data series	Inclusion
	Demographic and Health Survey 1986 (Direct)	1
	Demographic and Health Survey 1986 (Indirect)	0
	Demographic and Health Survey 1990 (Direct)	1
	Demographic and Health Survey 1990 (Indirect)	0
	Demographic and Health Survey 1995 (Direct)	1
	Demographic and Health Survey 1995 (Indirect)	0
	Demographic and Health Survey 2000 (Direct)	1
	Demographic and Health Survey 2000 (Indirect)	0
	Demographic and Health Survey 2005 (Direct)	1
	Demographic and Health Survey 2005 (Indirect)	0
	Census 2005 (Household Deaths)	0
	Census 2005 (Indirect)	0
	Demographic and Health Survey 2010 (Direct)	1
	Demographic and Health Survey 2010 (Indirect)	0
	Demographic and Health Survey 2015 (Direct)	1
	Vital Registration (Mexico March 2009) 2009 (VR)	0
	UNPD Demographic Yearbook Data 2020 version 2020 (VR)	0
	VR Submitted to WHO/UNIGME 2020 version 2020 (VR)	0
Comoros	Census 1958 (Household Deaths)	0
	Census 1980 (Household Deaths)	1
	Census 1980 (Indirect)	0
	Census 1991 (Household Deaths)	1
	Demographic and Health Survey 1996 (Direct)	1
	Demographic and Health Survey 1996 (Indirect)	0
	Multiple Indicator Cluster Survey 2000 (Indirect)	0
	Census 2003 (Household Deaths)	1
	Demographic and Health Survey 2012 (Direct)	0
Congo	Census 1974 (Household Deaths)	0
	Census 1974 (Indirect)	1
	Census 1984 (Household Deaths)	0
	Demographic and Health Survey 2005 (Direct)	1
	Demographic and Health Survey 2005 (Indirect)	0
	AIDS Indicator Survey 2009 (Indirect)	1
	Demographic and Health Survey 2011-2012 (Direct)	1
	Multiple Indicator Cluster Survey 2014-2015 (Direct)	1
Cook Islands	Census 1966 (Indirect)	1
	Census 1976 (Indirect)	0
	Census 1981 (Indirect)	1
	Census 1996 (Indirect)	1
	Census 2001 (Indirect)	1
	Census 2006 (Indirect)	1
	Census 2011 (Indirect)	1
	Vital Statistics Report 2009-2013 (VR)	0
	Recalculated VR Submitted to WHO/UNIGME 2020 version 2020 (VR)	1
	VR Submitted to WHO/UNIGME 2020 version 2020 (VR)	0
Costa Rica	TABLAS DE VIDA NACIONALES (Life Table)	0
	Census 1973 (Indirect)	1
	National Fertility Survey 1976 (Indirect)	0
	World Fertility Survey 1976 (Direct)	1
	Contraceptive Prevalence Survey 1978 (Indirect)	0
	Contraceptive Prevalence Survey 1981 (Indirect)	0
	Census 1984 (Indirect)	1
	National Survey of Fertility and Health 1986 (Indirect)	0
	Census 2000 (Indirect)	0

	Data series	Inclusion
	Census 2011 (Indirect)	0
	Vital Registration (Mexico March 26, Roberto) (VR)	0
	UNPD Demographic Yearbook Data 2020 version 2020 (VR)	1
	VR Submitted to WHO/UNIGME 2020 version 2020 (VR)	1
Cote d’Ivoire	Survey 1957-1958 (Indirect)	0
	Demographic Survey Repeated Passages 1978-1979 (Household Deaths)	0
	Demographic Survey Repeated Passages 1978-1979 (Direct)	1
	Demographic Survey Repeated Passages 1978-1979 (Indirect)	0
	World Fertility Survey 1980-1981 (Direct)	1
	World Fertility Survey 1980-1981 (Indirect)	0
	Census 1988 (Indirect)	0
	Demographic and Health Survey 1994 (Direct)	0
	Demographic and Health Survey 1994 (Indirect)	0
	Demographic and Health Survey (MM adjusted) 1994 (Direct)	1
	Census 1998 (Household Deaths)	0
	Demographic and Health Survey 1998-1999 (Direct)	0
	Demographic and Health Survey 1998-1999 (Indirect)	0
	Demographic and Health Survey (MM adjusted) 1998-1999 (Direct)	1
	AIDS Indicator Survey 2005 (Direct)	0
	AIDS Indicator Survey 2005 (Indirect)	0
	AIDS Indicator Survey (MM adjusted) 2005 (Direct)	1
	AIDS Indicator Survey 2005 (Household Deaths)	0
	Demographic and Health Survey 2011-2012 (Direct)	0
	Demographic and Health Survey (MM adjusted) 2011-2012 (Direct)	1
	Multiple Indicator Cluster Survey 2016 (Direct)	0
	Multiple Indicator Cluster Survey (MM adjusted) 2016 (Direct)	1
Croatia	Census 2011 (Household Deaths)	0
	VR Submitted to WHO/UNIGME 2020 version 2020 (VR)	1
Cuba	National Life Tables (Life Table)	0
	TABLAS DE VIDA NACIONALES (Life Table)	0
	National Population Survey on Income and Expenditures 1974 (Indirect)	0
	National Demographic Survey 1979 (Indirect)	0
	Census 1981 (Indirect)	0
	National Fertility Survey 1987 (Indirect)	0
	Multiple Indicator Cluster Survey 2010-2011 (Indirect)	0
	Vital Registration 2013 (VR)	0
	Vital Registration 2016 (VR)	0
	UNPD Demographic Yearbook Data 2020 version 2020 (VR)	1
	VR Submitted to WHO/UNIGME 2020 version 2020 (VR)	1
Cyprus	Recalculated VR Submitted to WHO/UNIGME 2020 version 2020 (VR)	1
	UNPD Demographic Yearbook Data 2020 version 2020 (VR)	1
	VR Submitted to WHO/UNIGME 2020 version 2020 (VR)	0
Czechia	VR Submitted to WHO/UNIGME 2020 version 2020 (VR)	1
Democratic People’s Republic of Korea	Census (adjusted fro 1q0 and thus 5q0 based on 4q0 and south model life table) 1993 (Household Deaths)	1
	Census (adjusted 1q0 and thus 5q0 based on 4q1 and north model life table) 2008 (Household Deaths)	1
	Socio-economic, Demographic and Health Survey 2014 (Life Table)	0
	Multiple Indicator Cluster Survey 2017 (Direct)	0
	Civil Registration data from Central Bureau of Statistics 2000 (VR)	1
Democratic Republic of the Congo	Census 1984 (Indirect)	1
	Multiple Indicator Cluster Survey 1995 (Indirect)	1
	Multiple Indicator Cluster Survey 2001 (Indirect)	1
	Demographic and Health Survey 2007 (Direct)	1
	Demographic and Health Survey 2007 (Indirect)	0
	Multiple Indicator Cluster Survey 2010 (Indirect)	1

	Data series	Inclusion
	Demographic and Health Survey 2013-2014 (Direct)	1
	Multiple Indicator Cluster Survey 2017-2018 (Direct)	0
Denmark	VR Submitted to WHO/UNIGME 2020 version 2020 (VR)	1
Djibouti	Demographic Survey 1991 (Indirect)	1
	PAPFAM Family Health Survey 2002 (Direct)	1
	Multiple Indicator Cluster Survey 2006 (Indirect)	1
	PAPFAM Family Health Survey 2012 (Direct)	1
Dominica	Recalculated VR Submitted to WHO/UNIGME 2020 version 2020 (VR)	1
	VR Submitted to WHO/UNIGME 2020 version 2020 (VR)	0
Dominican Republic	Census 1970 (Indirect)	1
	World Fertility Survey 1975 (Direct)	1
	World Fertility Survey 1975 (Indirect)	0
	World Fertility Survey 1980 (Direct)	1
	World Fertility Survey 1980 (Indirect)	0
	Census 1981 (Indirect)	1
	Contraceptive Prevalence Survey 1983 (Indirect)	1
	Demographic and Health Survey 1986 (Direct)	1
	Demographic and Health Survey 1986 (Indirect)	0
	Demographic and Health Survey 1991 (Direct)	1
	Demographic and Health Survey 1991 (Indirect)	0
	Demographic and Health Survey 1996 (Direct)	1
	Demographic and Health Survey 1996 (Indirect)	0
	Demographic and Health Survey 1999 (Direct)	0
	Multiple Indicator Cluster Survey 2000 (Indirect)	1
	Demographic and Health Survey 2002 (Direct)	1
	Demographic and Health Survey 2002 (Indirect)	0
	Census 2002 (Indirect)	0
	Multiple Indicator Cluster Survey 2006 (Direct)	1
	Demographic and Health Survey 2007 (Direct)	1
	Demographic and Health Survey 2007 (Indirect)	0
	Census 2010 (Indirect)	0
	Census 2010 (Household Deaths)	0
	Demographic and Health Survey 2013 (Direct)	1
	Multiple Indicator Cluster Survey 2014 (Direct)	1
	VR Submitted to WHO/UNIGME 2020 version 2020 (VR)	0
Ecuador	Census 1974 (Indirect)	1
	National Fertility Survey 1979-1980 (Indirect)	0
	World Fertility Survey 1979-1980 (Direct)	1
	Census 1982 (Indirect)	1
	ESMID 1982 (Indirect)	1
	Demographic and Family Health Survey 1987 (Direct)	1
	Demographic and Family Health Survey 1987 (Indirect)	0
	Demographic and Maternal and Child Health Survey 1989 (Direct)	1
	Census 1990 (Indirect)	1
	Demographic and Maternal and Child Health Survey 1994 (Direct)	0
	Demographic and Maternal and Child Health Survey 1999 (Direct)	1
	Census 2001 (Indirect)	1
	Demographic and Maternal and Child Health Survey 2004 (Direct)	1
	Census 2010 (Indirect)	1
	National Health and Nutrition Survey 2012 (Direct)	0
	National Health and Nutrition Survey, Imputed birth 2012 (Direct)	0
	National Health and Nutrition Survey, Imputed birth and deaths 2012 (Direct)	1
	National Health and Nutrition Survey 2018 (Direct)	0
	National Health and Nutrition Survey 2018 (Indirect)	0

	Data series	Inclusion
	Adjusted Vital Registration (Live Births) 2018 (VR)	1
	Vital Registration (INEC Population projection) 2018 (VR)	0
	Vital Registration (Live Births) 2018 (VR)	0
	UNPD Demographic Yearbook Data 2020 version 2020 (VR)	0
	VR Submitted to WHO/UNIGME 2020 version 2020 (VR)	0
Egypt	Census 1960 (Household Deaths)	0
	Census 1976 (Indirect)	0
	World Fertility Survey 1980 (Direct)	1
	World Fertility Survey 1980 (Indirect)	0
	Contraceptive Prevalence Survey 1984 (Indirect)	1
	Census 1986 (Indirect)	0
	Demographic and Health Survey 1988 (Direct)	1
	Demographic and Health Survey 1988 (Indirect)	0
	PAPCHILD Maternal and Child Health Survey 1991 (Direct)	1
	Demographic and Health Survey 1992 (Direct)	1
	Demographic and Health Survey 1992 (Indirect)	0
	Demographic and Health Survey 1995 (Direct)	1
	Demographic and Health Survey 1995 (Indirect)	0
	Demographic and Health Survey 1997 (Direct)	1
	Demographic and Health Survey 1998 (Direct)	1
	Demographic and Health Survey 2000 (Direct)	1
	Demographic and Health Survey 2000 (Indirect)	0
	Demographic and Health Survey 2003 (Direct)	1
	Demographic and Health Survey 2003 (Indirect)	0
	Demographic and Health Survey 2005 (Direct)	1
	Demographic and Health Survey 2005 (Indirect)	0
	Demographic and Health Survey 2008 (Direct)	1
	Demographic and Health Survey 2014 (Direct)	1
	Health Indicator Survey 2015 (Indirect)	1
	UNPD Demographic Yearbook Data 2020 version 2020 (VR)	0
	VR Submitted to WHO/UNIGME 2020 version 2020 (VR)	0
El Salvador	Census 1971 (Indirect)	1
	National Fertility Survey 1973 (Indirect)	1
	National Family Health Survey 1985 (Direct)	0
	National Family Health Survey 1985 (Indirect)	1
	National Family Health Survey 1988 (Direct)	1
	Census 1992 (Household Deaths)	0
	Census 1992 (Indirect)	1
	Household Survey of Multiple Purposes 1992 (Indirect)	1
	Household Survey of Multiple Purposes 1993 (Indirect)	1
	National Family Health Survey 1993 (Direct)	1
	National Family Health Survey 1993 (Indirect)	0
	National Family Health Survey 1998 (Direct)	1
	National Family Health Survey 1998 (Indirect)	0
	National Family Health Survey 2002-2003 (Direct)	1
	Census 2007 (Household Deaths)	0
	Census 2007 (Indirect)	1
	National Family Health Survey 2008 (Direct)	1
	National Health Survey 2014 (Direct)	1
	Vital Registration data from Informe de Labores Ministerio de Salud 2010-2011 (VR)	0
	Vital Registration data from Informe de Labores Ministerio de Salud 2013-2014 2013-2014 (VR)	0
	Vital Registration data from Informe de Labores Ministerio de Salud 2015-2016 2015-2016 (VR)	0
	UNPD Demographic Yearbook Data 2020 version 2020 (VR)	0
	VR Submitted to WHO/UNIGME 2020 version 2020 (VR)	0

	Data series	Inclusion
Equatorial Guinea	Census 1983 (Household Deaths)	1
	Census 1994 (Household Deaths)	1
	Multiple Indicator Cluster Survey 2000 (Indirect)	1
	Census 2001 (Household Deaths)	1
	Demographic and Health Survey 2011 (Direct)	1
Eritrea	Demographic and Health Survey 1995-1996 (Direct)	1
	Demographic and Health Survey 1995-1996 (Indirect)	0
	Demographic and Health Survey 2002 (Direct)	1
	Demographic and Health Survey 2002 (Indirect)	0
	Population and Health Survey 2010 (Direct)	1
Estonia	Census 2011-2012 (Household Deaths)	0
	Recalculated VR Submitted to WHO/UNIGME 2020 version 2020 (VR)	1
	VR Submitted to WHO/UNIGME 2020 version 2020 (VR)	0
Eswatini	Census 1966 (Indirect)	1
	Census 1976 (Indirect)	1
	Census 1986 (Indirect)	1
	Census 1997 (Household Deaths)	0
	Census 1997 (Indirect)	1
	Multiple Indicator Cluster Survey 1999-2000 (Indirect)	1
	Demographic and Health Survey 2006-2007 (Household Deaths)	0
	Demographic and Health Survey 2006-2007 (Direct)	0
	Demographic and Health Survey 2006-2007 (Indirect)	0
	Demographic and Health Survey (MM adjusted) 2006-2007 (Direct)	1
	Census 2007 (Household Deaths)	0
	Census 2007 (Indirect)	1
	Multiple Indicator Cluster Survey 2010 (Direct)	0
	Multiple Indicator Cluster Survey (MM adjusted) 2010 (Direct)	1
	Multiple Indicator Cluster Survey 2014 (Direct)	0
	Multiple Indicator Cluster Survey (MM adjusted) 2014 (Direct)	1
Ethiopia	Demographic Survey 1981 (Household Deaths)	0
	Demographic Survey 1981 (Indirect)	1
	Census 1984 (Household Deaths)	0
	Census 1984 (Indirect)	0
	National Family and Fertility Survey 1990 (Direct)	1
	National Family and Fertility Survey 1990 (Indirect)	0
	Census 1994 (Indirect)	0
	Demographic and Health Survey 2000 (Direct)	1
	Demographic and Health Survey 2000 (Indirect)	0
	Demographic and Health Survey 2005 (Direct)	1
	Demographic and Health Survey 2005 (Indirect)	0
	Census 2007 (Household Deaths)	0
	Census 2007 (Indirect)	0
	Demographic and Health Survey 2011 (Direct)	1
	Demographic and Health Survey 2016 (Direct)	1
	Mini Demographic and Health Survey 2019 (Direct)	1
Fiji	Census 1966 (Indirect)	1
	World Fertility Survey 1974 (Direct)	1
	Census 1986 (Indirect)	1
	Census 1996 (Indirect)	1
	VR Submitted to WHO/UNIGME 2020 version 2020 (VR)	1
Finland	UNPD Demographic Yearbook Data 2020 version 2020 (VR)	1
	VR Submitted to WHO/UNIGME 2020 version 2020 (VR)	1
France	VR Submitted to WHO/UNIGME 2020 version 2020 (VR)	1
Gabon	Census 1960-1961 (Household Deaths)	0

	Data series	Inclusion
	Demographic and Health Survey 2000 (Direct)	0
	Demographic and Health Survey 2000 (Indirect)	0
	Demographic and Health Survey (MM adjusted) 2000 (Direct)	1
	Demographic and Health Survey 2012 (Direct)	0
	Demographic and Health Survey (MM adjusted) 2012 (Direct)	1
Gambia	Census 1973 (Indirect)	1
	Census 1983 (Indirect)	1
	Census 1993 (Indirect)	1
	Multiple Indicator Cluster Survey 2000 (Indirect)	1
	Census 2003 (Indirect)	1
	Multiple Indicator Cluster Survey 2005-2006 (Indirect)	1
	Multiple Indicator Cluster Survey 2010 (Indirect)	1
	Demographic and Health Survey 2013 (Direct)	0
	Census 2013 (Indirect)	0
	Multiple Indicator Cluster Survey 2018 (Direct)	1
	Demographic and Health Survey (Preliminary) 2019-2020 (Direct)	1
Georgia	Census 1989 (Indirect)	0
	Reproductive Health Survey 1999-2000 (Direct)	1
	Census 2002 (Indirect)	1
	Multiple Indicator Cluster Survey 2005 (Indirect)	1
	Reproductive Health Survey 2005 (Direct)	1
	Reproductive Health Survey 2010 (Direct)	1
	Census 2014 (Indirect)	0
	Multiple Indicator Cluster Survey 2018 (Indirect)	0
	Vital Statistics of the National Center for Disease Control and Public Health 2016 (VR)	1
	Vital Statistics of the National Center for Disease Control and Public Health 2018 (VR)	1
	VR Submitted to WHO/UNIGME 2020 version 2020 (VR)	0
Germany	VR Submitted to WHO/UNIGME 2020 version 2020 (VR)	1
Ghana	Census 1948 (Indirect)	1
	Census 1960 (Indirect)	1
	Census 1971 (Indirect)	1
	Ghana Fertility Survey 1979-1980 (Indirect)	0
	World Fertility Survey 1979-1980 (Direct)	0
	Demographic and Health Survey 1988 (Direct)	1
	Demographic and Health Survey 1988 (Indirect)	0
	Demographic and Health Survey 1993-1994 (Direct)	1
	Demographic and Health Survey 1993-1994 (Indirect)	0
	Demographic and Health Survey 1998-1999 (Direct)	1
	Demographic and Health Survey 1998-1999 (Indirect)	0
	Census 2000 (Indirect)	0
	Demographic and Health Survey 2003 (Direct)	1
	Demographic and Health Survey 2003 (Indirect)	0
	Multiple Indicator Cluster Survey 2006 (Indirect)	1
	Maternal Health Survey 2007 (Direct)	1
	Demographic and Health Survey 2008 (Direct)	1
	Demographic and Health Survey 2008 (Indirect)	0
	Census 2010 (Household Deaths)	0
	Census 2010 (Indirect)	1
	Multiple Indicator Cluster Survey 2011 (Direct)	1
	Demographic and Health Survey 2014 (Direct)	1
	Malaria Indicator Survey 2016 (Indirect)	1
	Maternal Health Survey 2017 (Direct)	1
	Multiple Indicator Cluster Survey 2017-2018 (Direct)	1
	Dual Registration 1969 (VR)	1

	Data series	Inclusion
	CCP Registration 1975 (VR)	1
Greece	UNPD Demographic Yearbook Data 2020 version 2020 (VR)	1
	VR Submitted to WHO/UNIGME 2020 version 2020 (VR)	1
Grenada	Recalculated VR Submitted to WHO/UNIGME 2020 version 2020 (VR)	1
	UNPD Demographic Yearbook Data 2020 version 2020 (VR)	1
	VR Submitted to WHO/UNIGME 2020 version 2020 (VR)	0
Guatemala	Census 1973 (Indirect)	1
	Encuesta Nacional de Fecundidad, Planificacion Familiar y Comunicacion 1978-1979 (Indirect)	1
	Census 1981 (Indirect)	1
	Demographic and Health Survey 1987 (Direct)	1
	Demographic and Health Survey 1987 (Indirect)	0
	Enc. Nacional SocioDemografica 1987 (Indirect)	1
	Enc. Nacional Sociodemografica 1989 (Indirect)	1
	Census 1994 (Indirect)	1
	Demographic and Health Survey 1995 (Direct)	1
	Demographic and Health Survey 1995 (Indirect)	0
	Demographic and Health Survey 1998-1999 (Direct)	1
	Demographic and Health Survey 1998-1999 (Indirect)	0
	Census 2002 (Indirect)	1
	Encuesta Nacional de Salud Materno Infantil (ENSMI) 2002 (Direct)	1
	Encuesta Nacional de Salud Materno Infantil (ENSMI) 2002 (Indirect)	0
	Encuesta Nacional de Salud Materno Infantil (ENSMI) 2008-2009 (Direct)	1
	Demographic and Health Survey 2014-2015 (Direct)	1
	Census 2018 (Indirect)	1
	UNPD Demographic Yearbook Data 2020 version 2020 (VR)	0
	VR Submitted to WHO/UNIGME 2020 version 2020 (VR)	0
Guinea	Survey 1954-1955 (Household Deaths)	1
	Survey 1954-1955 (Indirect)	1
	Census 1983 (Household Deaths)	0
	Demographic and Health Survey 1992 (Direct)	1
	Demographic and Health Survey 1992 (Indirect)	0
	Census 1996 (Household Deaths)	0
	Census 1996 (Indirect)	1
	Demographic and Health Survey 1999 (Direct)	1
	Demographic and Health Survey 1999 (Indirect)	0
	Multiple Indicator Cluster Survey 2003 (Indirect)	1
	Demographic and Health Survey 2005 (Direct)	1
	Demographic and Health Survey 2005 (Indirect)	0
	Demographic and Health Survey 2012 (Direct)	1
	Census 2014 (Household Deaths)	0
	Census 2014 (Indirect)	1
	Multiple Indicator Cluster Survey 2016 (Direct)	0
	Demographic and Health Survey 2018 (Direct)	1
Guinea-Bissau	Census 1950 (Indirect)	0
	Multiple Indicator Cluster Survey 2000 (Indirect)	1
	Multiple Indicator Cluster Survey 2006 (Indirect)	1
	Multiple Indicator Cluster Survey 2010 (Direct)	1
	Multiple Indicator Cluster Survey 2010 (Indirect)	0
	Multiple Indicator Cluster Survey 2014 (Direct)	1
Guyana	World Fertility Survey 1975 (Direct)	1
	World Fertility Survey 1975 (Indirect)	0
	Multiple Indicator Cluster Survey 2000-2001 (Indirect)	1
	Census 2002 (Indirect)	1
	AIDS Indicator Survey 2005 (Direct)	1

	Data series	Inclusion
	AIDS Indicator Survey 2005 (Indirect)	0
	Multiple Indicator Cluster Survey 2006 (Indirect)	1
	Demographic and Health Survey 2009 (Direct)	1
	Census 2012 (Household Deaths)	0
	Multiple Indicator Cluster Survey 2014 (Direct)	1
	VR Submitted to WHO/UNIGME 2020 version 2020 (VR)	0
Haiti	Census 1971 (Direct)	0
	Census 1971 (Household Deaths)	0
	World Fertility Survey 1977 (Direct)	1
	World Fertility Survey 1977 (Indirect)	0
	Census 1982 (Indirect)	1
	Contraceptive Prevalence Survey 1983 (Indirect)	1
	Mortality, morbidity and service utilization survey 1987 (Direct)	1
	Mortality, morbidity and service utilization survey 1987 (Indirect)	0
	Demographic and Health Survey 1994-1995 (Direct)	1
	Demographic and Health Survey 1994-1995 (Indirect)	0
	Demographic and Health Survey 2000 (Direct)	1
	Demographic and Health Survey 2000 (Indirect)	0
	Census 2003 (Household Deaths)	0
	Census 2003 (Indirect)	0
	Demographic and Health Survey 2005-2006 (Direct)	1
	Demographic and Health Survey 2005-2006 (Indirect)	0
	Demographic and Health Survey 2012 (Direct)	1
	Demographic and Health Survey 2016-2017 (Direct)	1
	VR Submitted to WHO/UNIGME 2020 version 2020 (VR)	0
Honduras	National Demographic Survey 1970-1972 (Household Deaths)	0
	National Demographic Survey 1972 (Direct)	1
	National Demographic Survey 1972 (Indirect)	1
	Census 1974 (Indirect)	1
	National Demographic Survey 1983 (Indirect)	1
	National Survey of Maternal and Child Health 1984 (Indirect)	1
	National Survey of Epidemiology and Family Health 1987 (Direct)	1
	National Survey of Epidemiology and Family Health 1987 (Indirect)	0
	Census 1988 (Indirect)	1
	National Survey of Epidemiology and Family Health 1991-1992 (Direct)	1
	National Survey of Epidemiology and Family Health 1991-1992 (Indirect)	0
	National Survey of Epidemiology and Family Health 1996 (Direct)	1
	National Survey of Epidemiology and Family Health 1996 (Indirect)	0
	Census 2001 (Household Deaths)	0
	Census 2001 (Indirect)	1
	National Survey of Epidemiology and Family Health 2001 (Direct)	0
	Demographic and Health Survey 2005-2006 (Direct)	1
	Demographic and Health Survey 2005-2006 (Indirect)	0
	Demographic and Health Survey 2011-2012 (Direct)	1
	Census 2013 (Household Deaths)	0
	Census 2013 (Indirect)	1
	VR Submitted to WHO/UNIGME 2020 version 2020 (VR)	0
Hungary	UNPD Demographic Yearbook Data 2020 version 2020 (VR)	1
	VR Submitted to WHO/UNIGME 2020 version 2020 (VR)	1
Iceland	Recalculated VR Submitted to WHO/UNIGME 2020 version 2020 (VR)	1
	UNPD Demographic Yearbook Data 2020 version 2020 (VR)	1
	VR Submitted to WHO/UNIGME 2020 version 2020 (VR)	0
India	National Sample Survey 1966 (Indirect)	1
	National Family Planning Survey 1970 (Indirect)	1

	Data series	Inclusion
	National Fertility Survey 1972 (Indirect)	1
	Survey on Infant and Child Mortality 1979 (Indirect)	0
	National Family Planning Survey 1980 (Indirect)	1
	Census 1981 (Indirect)	0
	Second All-India Family Planning Survey 1981 (Indirect)	1
	Census 1991 (Indirect)	0
	National Family Health Survey 1992-1993 (Direct)	1
	National Family Health Survey 1992-1993 (Indirect)	0
	National Family Health Survey 1998-1999 (Direct)	1
	National Family Health Survey 1998-1999 (Indirect)	0
	District Level Household Survey 1999 (Indirect)	1
	Census 2001 (Indirect)	0
	District Level Household Survey 2004 (Indirect)	1
	National Family Health Survey 2005-2006 (Direct)	1
	National Family Health Survey 2005-2006 (Indirect)	0
	District Level Household Survey 2008 (Indirect)	1
	Census 2011 (Indirect)	1
	National Family Health Survey 2015-2016 (Direct)	0
	Provincial Annual Sanitary-Health Reports 1947 (VR)	0
	Sample Registration System 2017 (VR)	1
	Sample Registration System 2018 (VR)	1
Indonesia	National Socio-economic Survey 1964-1965 (Household Deaths)	0
	Census 1971 (Indirect)	1
	World Fertility Survey 1976 (Direct)	1
	World Fertility Survey 1976 (Indirect)	0
	Census 1980 (Indirect)	1
	Demographic and Health Survey 1987 (Direct)	1
	Demographic and Health Survey 1987 (Indirect)	0
	Census 1990 (Indirect)	1
	Demographic and Health Survey 1991 (Direct)	1
	Demographic and Health Survey 1991 (Indirect)	0
	Demographic and Health Survey 1994 (Direct)	1
	Demographic and Health Survey 1994 (Indirect)	0
	Intercensal Survey 1995 (Indirect)	0
	Demographic and Health Survey 1997 (Direct)	1
	Demographic and Health Survey 1997 (Indirect)	0
	Census 2000 (Indirect)	1
	Demographic and Health Survey 2002-2003 (Direct)	1
	Demographic and Health Survey 2002-2003 (Indirect)	0
	National Socio-Economic Survey (SUSENAS) 2003 (Indirect)	1
	National Socio-Economic Survey (SUSENAS) 2004 (Indirect)	1
	National Socio-Economic Survey (SUSENAS) 2005 (Indirect)	1
	National Socio-Economic Survey (SUSENAS) 2006 (Indirect)	1
	Demographic and Health Survey 2007 (Direct)	1
	Demographic and Health Survey 2007 (Indirect)	0
	National Socio-Economic Survey (SUSENAS) 2007 (Indirect)	1
	National Socio-Economic Survey (SUSENAS) 2008 (Indirect)	1
	National Socio-Economic Survey (SUSENAS) 2009 (Indirect)	1
	Census 2010 (Household Deaths)	0
	Census 2010 (Indirect)	1
	National Socio-Economic Survey (SUSENAS) 2010 (Indirect)	1
	Demographic and Health Survey 2012 (Direct)	1
	Demographic and Health Survey 2017 (Direct)	1
	Report on Sample Vital Registration System 2014 2014 (VR)	0

	Data series	Inclusion
Iran (Islamic Republic of)	Population Growth Survey 1973-1976 (Direct)	1
	Population Growth Survey 1973-1976 (Indirect)	0
	Population Health Survey 1974 (Direct)	1
	Iran National MICS 1985 (Household Deaths)	1
	Census 1986 (Indirect)	1
	Infant and Child Mortality Survey 1989 (Direct)	1
	Infant and Child Mortality Survey 1989 (Indirect)	0
	Intercensal Population Survey 1991 (Indirect)	1
	Iran National MICS 1995 (Household Deaths)	1
	Census 1996 (Indirect)	1
	Demographic Survey 1998 (Indirect)	1
	Iran National MICS 1998 (Household Deaths)	1
	Demographic and Health Survey 2000 (Direct)	1
	Demographic and Health Survey 2000 (Indirect)	0
	Census 2006 (Indirect)	1
	National Multiple-Indicator Demographic and Health Survey 2010 (Direct)	1
	Census 2016 (Indirect)	0
	Iran National Child Mortality Surveillance System 2008 (VR)	1
	VR Submitted to WHO/UNIGME 2020 version 2020 (VR)	0
Iraq	Census 1957 (Indirect)	1
	Demographic Sample Survey and Sample Registration System 1973 (Direct)	1
	Fertility Survey 1974 (Indirect)	1
	Census 1987 (Indirect)	1
	Gulf Child Health Survey 1989 (Direct)	0
	Gulf Child Health Survey 1989 (Indirect)	1
	Immunization, Diarrhoeal Disease, Maternal and Child Mortality Survey 1990 (Indirect)	1
	Infant and Child Mortality and Nutrition Survey 1991 (Direct)	0
	Census 1997 (Indirect)	0
	Child and Maternal Mortality Survey 1999 (Direct)	0
	Living Conditions Survey 2004 (Direct)	1
	Iraq Family Health Survey 2006 (Indirect)	1
	Multiple Indicator Cluster Survey 2006 (Direct)	1
	Multiple Indicator Cluster Survey 2006 (Indirect)	0
	Multiple Indicator Cluster Survey 2011 (Direct)	1
	Multiple Indicator Cluster Survey 2018 (Direct)	1
	VR Submitted to WHO/UNIGME 2020 version 2020 (VR)	0
Ireland	Census 2011 (Household Deaths)	0
	VR Submitted to WHO/UNIGME 2020 version 2020 (VR)	1
Israel	National Life table 1969-1988 (Life Table)	0
	UNPD Demographic Yearbook Data 2020 version 2020 (VR)	1
	VR Submitted to WHO/UNIGME 2020 version 2020 (VR)	1
Italy	VR Submitted to WHO/UNIGME 2020 version 2020 (VR)	1
Jamaica	National Life Tables (Life Table)	1
	Jamaica Fertility Survey 1975-1976 (Indirect)	0
	World Fertility Survey 1975-1976 (Direct)	1
	Census 1982 (Indirect)	1
	Contraceptive Prevalence Survey 1989 (Direct)	0
	Multiple Indicator Cluster Survey 2000 (Indirect)	0
	Census 2001 (Indirect)	0
	Multiple Indicator Cluster Survey 2005 (Indirect)	1
	Reproductive Health Survey 2008-2009 (Direct)	1
	Census 2011 (Indirect)	1
	UNPD Demographic Yearbook Data 2020 version 2020 (VR)	0
	VR Submitted to WHO/UNIGME 2020 version 2020 (VR)	0

	Data series	Inclusion
Japan	VR Submitted to WHO/UNIGME 2020 version 2020 (VR)	1
Jordan	Census 1961 (Indirect)	1
	Jordan Fertility Survey 1972 (Indirect)	1
	World Fertility Survey 1976 (Direct)	1
	World Fertility Survey 1976 (Indirect)	0
	Census 1979 (Indirect)	1
	Jordan Demographic Survey 1981 (Indirect)	1
	EPI/CDD and Child Mortality Survey 1988 (Indirect)	1
	Demographic and Health Survey 1990 (Direct)	1
	Demographic and Health Survey 1990 (Indirect)	0
	EPI/CDD and Child Mortality Survey 1990 (Indirect)	1
	Demographic and Health Survey 1997 (Direct)	1
	Demographic and Health Survey 1997 (Indirect)	0
	Annual Fertility Survey 1999 (Indirect)	1
	Demographic and Health Survey 2002 (Direct)	1
	Demographic and Health Survey 2002 (Indirect)	0
	Demographic and Health Survey 2007 (Direct)	1
	Demographic and Health Survey 2007 (Indirect)	0
	Demographic and Health Survey 2009 (Direct)	1
	Demographic and Health Survey 2009 (Indirect)	0
	Demographic and Health Survey 2012 (Direct)	1
	Census 2015 (Household Deaths)	0
	Demographic and Health Survey 2017 (Direct)	1
	VR Submitted to WHO/UNIGME 2020 version 2020 (VR)	0
Kazakhstan	Census 1989 (Indirect)	1
	Demographic and Health Survey 1995 (Direct)	1
	Demographic and Health Survey 1995 (Indirect)	0
	lsms 1996 (Indirect)	1
	Demographic and Health Survey 1999 (Direct)	1
	Demographic and Health Survey 1999 (Indirect)	0
	Census 1999 (Indirect)	0
	Multiple Indicator Cluster Survey 2006 (Indirect)	1
	Census 2009 (Indirect)	1
	Multiple Indicator Cluster Survey 2010-2011 (Indirect)	1
	Multiple Indicator Cluster Survey 2015 (Indirect)	1
	Vital Registration Data from The Agency of Statistics of Kazakhstan (VR)	0
	Transmonee Vital Registration Data 2008 version 2008 (VR)	0
	VR Submitted to WHO/UNIGME 2020 version 2020 (VR)	1
Kenya	Census 1969 (Indirect)	1
	Demographic Survey 1977 (Indirect)	1
	World Fertility Survey 1977-1978 (Direct)	1
	World Fertility Survey 1977-1978 (Indirect)	0
	Census 1979 (Indirect)	1
	Demographic Survey 1983 (Indirect)	1
	Demographic and Health Survey 1989 (Direct)	0
	Demographic and Health Survey 1989 (Indirect)	0
	Demographic and Health Survey (MM adjusted) 1989 (Direct)	1
	Census 1989 (Indirect)	1
	Demographic and Health Survey 1993 (Direct)	0
	Demographic and Health Survey 1993 (Indirect)	0
	Demographic and Health Survey (MM adjusted) 1993 (Direct)	1
	Demographic and Health Survey 1998 (Direct)	0
	Demographic and Health Survey 1998 (Indirect)	0
	Demographic and Health Survey (MM adjusted) 1998 (Direct)	1

	Data series	Inclusion
	Census 1999 (Indirect)	1
	Multiple Indicator Cluster Survey 2000 (Indirect)	1
	Demographic and Health Survey 2003 (Direct)	0
	Demographic and Health Survey 2003 (Indirect)	0
	Demographic and Health Survey (MM adjusted) 2003 (Direct)	1
	Demographic and Health Survey 2008-2009 (Direct)	0
	Demographic and Health Survey 2008-2009 (Indirect)	0
	Demographic and Health Survey (MM adjusted) 2008-2009 (Direct)	1
	Census 2009 (Household Deaths)	0
	Demographic and Health Survey 2014 (Direct)	0
	Demographic and Health Survey (MM adjusted) 2014 (Direct)	1
	Malaria Indicator Survey 2015 (Indirect)	1
Kiribati	Census 1978 (Indirect)	1
	Census 1990 (Indirect)	1
	Census 1995 (Indirect)	1
	Census 2005 (Indirect)	1
	SPC Demographic and Health Survey 2009 (Direct)	1
	Census 2010 (Indirect)	1
	Census 2010 (Household Deaths)	0
	Multiple Indicator Cluster Survey 2018-2019 (Direct)	0
	VR Submitted to WHO/UNIGME 2020 version 2020 (VR)	0
Kuwait	National Life Tables (Life Table)	1
	Census 1975 (Indirect)	1
	Census 1980 (Indirect)	1
	Child Health Survey 1987 (Indirect)	1
	UNPD Demographic Yearbook Data 2020 version 2020 (VR)	0
	VR Submitted to WHO/UNIGME 2020 version 2020 (VR)	1
Kyrgyzstan	Census 1989 (Indirect)	0
	Demographic and Health Survey 1997 (Direct)	1
	Demographic and Health Survey 1997 (Indirect)	0
	Census 1999 (Indirect)	0
	Multiple Indicator Cluster Survey 2005-2006 (Indirect)	1
	Census 2009 (Indirect)	0
	Demographic and Health Survey 2012 (Direct)	1
	Multiple Indicator Cluster Survey 2014 (Direct)	1
	Multiple Indicator Cluster Survey 2018 (Direct)	1
	VR Submitted to WHO/UNIGME 2020 version 2020 (VR)	1
Lao People's Democratic Republic	Social Indicator Survey 1993 (Indirect)	1
	Fertility and Birth Spacing Survey 1994 (Direct)	1
	Census 1995 (Indirect)	1
	Reproductive Health Survey 2000 (Direct)	1
	Reproductive Health Survey 2005 (Direct)	1
	Census 2005 (Indirect)	1
	Reproductive Health Survey 2005 (Indirect)	0
	Lao Social Indicator Survey (combined MICS4/DHS) 2012 (Direct)	1
	Census 2015 (Direct)	0
	Lao Social Indicator Survey 2017 (Direct)	1
Latvia	Recalculated VR Submitted to WHO/UNIGME 2020 version 2020 (VR)	1
	VR Submitted to WHO/UNIGME 2020 version 2020 (VR)	0
Lebanon	National Fertility and Family Planning Survey 1971 (Indirect)	1
	National EPI CDD IMR Survey 1990 (Indirect)	1
	Population and Housing Survey 1996 (Indirect)	1
	Maternal and Child Health Survey 1996 (Indirect)	1
	PAPCHILD Maternal and Child Health Survey 1996 (Direct)	1

	Data series	Inclusion
	Multiple Indicator Cluster Survey 2000 (Indirect)	1
	PAPFAM Family Health Survey 2004 (Direct)	1
	Multiple Indicator Cluster Survey 2009 (Indirect)	1
	VR Submitted to WHO/UNIGME 2020 version 2020 (VR)	0
Lesotho	ces 1968 (Indirect)	1
	Demographic Survey 1971-1973 (Household Deaths)	0
	Demographic Survey 1971-1973 (Indirect)	1
	Census 1976 (Indirect)	1
	World Fertility Survey 1977 (Direct)	1
	World Fertility Survey 1977 (Indirect)	0
	World Fertility Survey 1977 (Household Deaths)	0
	Census 1986 (Household Deaths)	0
	Census 1986 (Indirect)	1
	Census 1996 (Household Deaths)	0
	Census 1996 (Indirect)	1
	Multiple Indicator Cluster Survey 2000 (Indirect)	1
	Demographic Survey 2001 (Household Deaths)	0
	Demographic Survey 2001 (Indirect)	1
	Demographic and Health Survey 2004 (Direct)	0
	Demographic and Health Survey 2004 (Indirect)	0
	Demographic and Health Survey (MM adjusted) 2004 (Direct)	1
	Census 2006 (Household Deaths)	0
	Census 2006 (Indirect)	1
	Demographic and Health Survey 2009 (Direct)	0
	Demographic and Health Survey 2009 (Indirect)	0
	Demographic and Health Survey (MM adjusted) 2009 (Direct)	1
	Demographic and Health Survey 2014 (Direct)	0
	Demographic and Health Survey (MM adjusted) 2014 (Direct)	1
	Multiple Indicator Cluster Survey 2018 (Direct)	0
	Multiple Indicator Cluster Survey (MM adjusted) 2018 (Direct)	1
Liberia	Population Growth Survey 1969-1970 (Direct)	0
	Population Growth Survey 1969-1970 (Indirect)	0
	Population Growth Survey 1970-1971 (Direct)	1
	Population Growth Survey 1970-1971 (Indirect)	1
	Census 1974 (Indirect)	0
	Demographic and Health Survey 1986 (Direct)	1
	Demographic and Health Survey 1986 (Indirect)	0
	LDHS 1999-2000 (Direct)	0
	Demographic and Health Survey 2006-2007 (Direct)	1
	Demographic and Health Survey 2006-2007 (Indirect)	0
	Population and Housing Census 2008 (Household Deaths)	0
	Population and Housing Census 2008 (Indirect)	0
	Malaria Indicator Survey 2008-2009 (Direct)	1
	Malaria Indicator Survey 2008-2009 (Indirect)	0
	Malaria Indicator Survey 2011 (Indirect)	1
	Demographic and Health Survey 2013 (Direct)	1
	Malaria Indicator Survey 2016 (Indirect)	1
	Demographic and Health Survey (Preliminary) 2019-2020 (Direct)	1
Libya	Census 1973 (Indirect)	1
	PAPCHILD Maternal and Child Health Survey 1995 (Direct)	1
	Multiple Indicator Cluster Survey (Preliminary) 2003 (Indirect)	1
	PAPFAM Family Health Survey 2007 (Direct)	1
	PAPFAM Family Health Survey 2014 (Direct)	0
	VR Data from Health and Environment Report 2009 2009 (VR)	1

	Data series	Inclusion
Lithuania	Census 2011 (Household Deaths)	0
	VR Submitted to WHO/UNIGME 2020 version 2020 (VR)	1
Luxembourg	Recalculated VR Submitted to WHO/UNIGME 2020 version 2020 (VR)	1
	UNPD Demographic Yearbook Data 2020 version 2020 (VR)	1
	VR Submitted to WHO/UNIGME 2020 version 2020 (VR)	0
Madagascar	Demographic Survey 1966 (Household Deaths)	0
	Madagascar Demographic Survey 1966 (Indirect)	0
	Demographic and Health Survey 1992 (Direct)	1
	Demographic and Health Survey 1992 (Indirect)	0
	Census 1993 (Household Deaths)	0
	Multiple Indicator Cluster Survey 1995 (Indirect)	1
	Demographic and Health Survey 1997 (Direct)	1
	Demographic and Health Survey 1997 (Indirect)	0
	Multiple Indicator Cluster Survey 2000 (Indirect)	1
	Demographic and Health Survey 2003-2004 (Direct)	1
	Demographic and Health Survey 2003-2004 (Indirect)	0
	Demographic and Health Survey 2008-2009 (Direct)	1
	Demographic and Health Survey 2008-2009 (Indirect)	0
	Malaria Indicator Survey 2011 (Indirect)	0
	National Survey on Monitoring the MDGs (ENSOMD) 2012-2013 (Direct)	0
	Malaria Indicator Survey 2013 (Indirect)	1
	Malaria Indicator Survey 2016 (Indirect)	1
	Multiple Indicator Cluster Survey 2018 (Direct)	1
Malawi	Population Change Survey 1970-1972 (Household Deaths)	0
	Population Change Survey 1970-1972 (Direct)	1
	Population Change Survey 1970-1972 (Indirect)	0
	Census 1977 (Household Deaths)	0
	Census 1977 (Indirect)	1
	Demographic Survey 1982 (Indirect)	1
	Family Formation Survey 1984 (Direct)	1
	Family Formation Survey 1984 (Household Deaths)	0
	Family Formation Survey 1984 (Indirect)	0
	Census 1987 (Household Deaths)	0
	Census 1987 (Indirect)	1
	Demographic and Health Survey 1992 (Direct)	0
	Demographic and Health Survey 1992 (Indirect)	0
	Demographic and Health Survey (MM adjusted) 1992 (Direct)	1
	Census 1998 (Household Deaths)	0
	Census 1998 (Indirect)	1
	Demographic and Health Survey 2000 (Direct)	0
	Demographic and Health Survey 2000 (Indirect)	0
	Demographic and Health Survey (MM adjusted) 2000 (Direct)	1
	Demographic and Health Survey 2004 (Direct)	0
	Demographic and Health Survey 2004 (Indirect)	0
	Demographic and Health Survey (MM adjusted) 2004 (Direct)	1
	Second Integrated Household Survey 2004-2005 (Household Deaths)	0
	Multiple Indicator Cluster Survey 2006 (Direct)	0
	Multiple Indicator Cluster Survey 2006 (Indirect)	0
	Multiple Indicator Cluster Survey (MM adjusted) 2006 (Direct)	1
	Census 2008 (Household Deaths)	0
	Census 2008 (Indirect)	1
	Demographic and Health Survey 2010 (Direct)	0
	Demographic and Health Survey (MM adjusted) 2010 (Direct)	1
	Demographic and Health Survey 2010 (Household Deaths)	0

	Data series	Inclusion
	Third Integrated Household Survey 2010-2011 (Household Deaths)	0
	Malaria Indicator Survey 2012 (Indirect)	0
	MDG Endline Survey 2013-2014 (Direct)	0
	MDG Endline Survey (MM adjusted) 2013-2014 (Direct)	1
	Malaria Indicator Survey 2014 (Indirect)	1
	Demographic and Health Survey 2015-2016 (Direct)	0
	Demographic and Health Survey (MM adjusted) 2015-2016 (Direct)	1
	Malaria Indicator Survey 2017 (Indirect)	1
	Census 2018 (Indirect)	1
Malaysia	National Life Table (Life Table)	1
	Census 1970 (Indirect)	1
	World Fertility Survey 1974 (Direct)	1
	Vital Registration Data from Department of Statistics 2017 (VR)	0
	Vital Registration Data from Department of Statistics 2019 (VR)	1
	VR Submitted to WHO/UNIGME 2020 version 2020 (VR)	1
Maldives	Census 1977 (Indirect)	1
	Census 1985 (Indirect)	1
	Census 1990 (Indirect)	1
	Census 1995 (Indirect)	1
	Poverty and Vulnerability Survey 1997 (Indirect)	1
	Census 2000 (Indirect)	1
	Vulnerability and Poverty Assessment 2004 (Indirect)	1
	Census 2006 (Indirect)	1
	Demographic and Health Survey 2009 (Direct)	1
	Demographic and Health Survey 2009 (Indirect)	0
	Demographic and Health Survey 2016-2017 (Direct)	1
	Vital Registration data from Statistical Yearbook of Maldives 2005 version 2005 (VR)	0
	Vital Statistics 2012 (VR)	0
	Recalculated VR Submitted to WHO/UNIGME 2020 version 2020 (VR)	1
	VR Submitted to WHO/UNIGME 2020 version 2020 (VR)	0
Mali	Survey 1957 (Indirect)	0
	Survey 1960.5 (Indirect)	0
	Census 1976 (Household Deaths)	0
	Demographic and Health Survey 1987 (Direct)	1
	Demographic and Health Survey 1987 (Indirect)	0
	Census 1987 (Household Deaths)	0
	Census 1987 (Indirect)	0
	Demographic and Health Survey 1995-1996 (Direct)	1
	Demographic and Health Survey 1995-1996 (Indirect)	0
	Census 1998 (Household Deaths)	0
	Census 1998 (Indirect)	0
	Demographic and Health Survey 2001 (Direct)	1
	Demographic and Health Survey 2001 (Indirect)	0
	Demographic and Health Survey 2006 (Direct)	1
	Demographic and Health Survey 2006 (Indirect)	0
	Census 2009 (Indirect)	1
	Census 2009 (Household Deaths)	0
	Demographic and Health Survey 2012-2013 (Direct)	1
	Multiple Indicator Cluster Survey 2015 (Direct)	1
	Malaria Indicator Survey 2015 (Indirect)	1
	Demographic and Health Survey 2018 (Direct)	1
Malta	Recalculated VR Submitted to WHO/UNIGME 2020 version 2020 (VR)	1
	UNPD Demographic Yearbook Data 2020 version 2020 (VR)	1
	VR Submitted to WHO/UNIGME 2020 version 2020 (VR)	0

	Data series	Inclusion
Marshall Islands	Census 1973 (Indirect)	1
	Census 1980 (Indirect)	1
	Women's Health Survey 1985 (Indirect)	1
	Census 1988 (Indirect)	1
	Census 1999 (Indirect)	1
	Demographic and Health Survey 2007 (Direct)	1
	Demographic and Health Survey 2007 (Indirect)	0
	Census 2011 (Household Deaths)	1
	Census 2011 (Indirect)	0
	VR Submitted to WHO/UNIGME 2020 version 2020 (VR)	0
Mauritania	Fouta-Toro-Survey 1957 (Indirect)	1
	Survey 1964-1965 (Indirect)	0
	Census 1977 (Indirect)	1
	World Fertility Survey 1981-1982 (Direct)	1
	World Fertility Survey 1981-1982 (Indirect)	0
	Census 1988 (Household Deaths)	0
	Census 1988 (Indirect)	1
	Maternal and Child Health Survey 1990 (Direct)	1
	Maternal and Child Health Survey 1990 (Indirect)	0
	PAPCHILD Maternal and Child Health Survey 1990 (Direct)	1
	Multiple Indicator Cluster Survey 1995 (Indirect)	0
	Demographic and Health Survey 2000-2001 (Direct)	1
	Demographic and Health Survey 2000-2001 (Indirect)	0
	EMIP survey 2003-2004 (Direct)	1
	EMIP survey 2003-2004 (Indirect)	0
	Multiple Indicator Cluster Survey 2007 (Indirect)	1
	Multiple Indicator Cluster Survey 2011 (Direct)	0
	Census 2013 (Indirect)	0
	Census 2013 (Household Deaths)	0
	Multiple Indicator Cluster Survey 2015 (Direct)	0
Mauritius	UNPD Demographic Yearbook Data 2020 version 2020 (VR)	1
	VR Submitted to WHO/UNIGME 2020 version 2020 (VR)	1
Mexico	World Fertility Survey 1976 (Direct)	1
	World Fertility Survey 1976 (Indirect)	0
	Contraceptive Prevalence Survey 1979 (Indirect)	1
	Census 1980 (Indirect)	1
	Demographic And Health Survey 1987 (Indirect)	0
	Demographic and Health Survey 1987 (Direct)	1
	Census 1990 (Indirect)	1
	Encuesta Nacional de la Dinamica Demografica (ENADID) 1992 (Direct)	1
	Encuesta Nacional de la Dinamica Demografica (ENADID) 1992 (Indirect)	0
	Encuesta Nacional de la Dinamica Demografica (ENADID) 1997 (Direct)	1
	Census 2000 (Indirect)	1
	Conteo 2005 (Indirect)	1
	Encuesta Nacional de la Dinamica Demografica (ENADID) 2006 (Indirect)	1
	Encuesta Nacional de la Dinamica Demografica (ENADID) 2009 (Direct)	0
	Census 2010 (Indirect)	1
	Encuesta Nacional de la Dinamica Demografica (ENADID) 2014 (Direct)	1
	Encuesta Nacional de la Dinamica Demografica (ENADID) 2014 (Indirect)	0
	Encuesta Intercensal 2015 (Indirect)	1
	Encuesta Nacional de la Dinamica Demografica (ENADID) 2018 (Direct)	1
	Vital Registration data from Mexico Ministry of Health 2018 (VR)	1
	VR Submitted to WHO/UNIGME 2020 version 2020 (VR)	0
	Census 1994 (Indirect)	1

	Data series	Inclusion
Micronesia (Federated States of)	Census 2000 (Indirect)	1
Monaco	VR Submitted to WHO/UNIGME 2020 version 2020 (VR)	1
Mongolia	Census 1989 (Indirect)	0
	Demographic Survey 1994 (Indirect)	1
	Demographic Survey 1996 (Direct)	1
	Reproductive Health Survey 1998 (Direct)	1
	Reproductive Health Survey 1998 (Indirect)	0
	Multiple Indicator Cluster Survey 2000 (Indirect)	1
	Reproductive Health Survey 2003 (Direct)	0
	Reproductive Health Survey 2003 (Indirect)	1
	Multiple Indicator Cluster Survey 2005 (Indirect)	1
	Reproductive Health Survey 2008 (Direct)	0
	Reproductive Health Survey 2008 (Indirect)	1
	Multiple Indicator Cluster Survey 2010 (Indirect)	1
	Social Indicator Sample Survey (SISS) 2013-2014 (Direct)	1
	Multiple Indicator Cluster Survey 2018 (Direct)	0
	VR Submitted to WHO/UNIGME 2020 version 2020 (VR)	1
Montenegro	Vital Registration from Statistical Yearbook 2012 (VR)	0
	Recalculated VR Submitted to WHO/UNIGME 2020 version 2020 (VR)	1
	VR Submitted to WHO/UNIGME 2020 version 2020 (VR)	0
Morocco	World Fertility Survey 1980 (Direct)	1
	World Fertility Survey 1980 (Indirect)	0
	Census 1982 (Indirect)	1
	Contraceptive Prevalence Survey 1983 (Indirect)	1
	Demographic and Health Survey 1987 (Direct)	1
	Demographic and Health Survey 1987 (Indirect)	0
	Demographic and Health Survey 1992 (Direct)	1
	Demographic and Health Survey 1992 (Indirect)	0
	Census 1994 (Indirect)	1
	Demographic and Health Survey 1995 (Direct)	1
	Demographic and Health Survey 1995 (Indirect)	0
	PAPG ENSME grand-echantillon 1996 1996 (Direct)	0
	PAPCHILD Maternal and Child Health Survey 1997 (Direct)	1
	Demographic and Health Survey 2003-2004 (Direct)	1
	Demographic and Health Survey 2003-2004 (Indirect)	0
	Census 2004 (Indirect)	1
	National Demographic Survey with repeated passages 2009-2010 (Household Deaths)	1
	National Survey on Population and Family Health 2011 (Direct)	1
	National Survey on Population and Family Health 2018 (Direct)	1
	VR Submitted to WHO/UNIGME 2020 version 2020 (VR)	0
Mozambique	Census 1940 (Indirect)	0
	Census 1950 (Indirect)	0
	Census 1970 (Indirect)	0
	Census 1980 (Indirect)	1
	Mozambique National Demographic Survey 1991 (Indirect)	1
	Multiple Indicator Cluster Survey 1995 (Indirect)	1
	Census 1997 (Household Deaths)	0
	Demographic and Health Survey 1997 (Direct)	0
	Demographic and Health Survey 1997 (Indirect)	0
	Demographic and Health Survey (MM adjusted) 1997 (Direct)	0
	Census 1997 (Indirect)	1
	Demographic and Health Survey 2003-2004 (Direct)	0

	Data series	Inclusion
	Demographic and Health Survey 2003-2004 (Indirect)	0
	Demographic and Health Survey (MM adjusted) 2003-2004 (Direct)	1
	Census 2007 (Household Deaths)	0
	Census 2007 (Indirect)	1
	Post-census Mortality Survey 2007-2008 (Direct)	0
	Multiple Indicator Cluster Survey 2008 (Direct)	0
	Multiple Indicator Cluster Survey (MM adjusted) 2008 (Direct)	1
	AIDS Indicator Survey 2009 (Indirect)	1
	Demographic and Health Survey 2011 (Direct)	0
	Demographic and Health Survey (MM adjusted) 2011 (Direct)	1
	AIDS Indicator Survey 2015 (Indirect)	1
	Census 2017 (Household Deaths)	0
	Census 2017 (Indirect)	1
	Malaria Indicator Survey 2018 (Indirect)	1
Myanmar	Census 1983 (Indirect)	1
	Population Change and Fertility Survey 1991 (Direct)	1
	Fertility and Reproductive Health Survey 1997 (Direct)	1
	Fertility and Reproductive Health Survey 1997 (Indirect)	0
	National Mortality Survey 1999 (Household Deaths)	1
	Fertility and Reproductive Health Survey 2001 (Direct)	1
	Fertility and Reproductive Health Survey 2001 (Indirect)	0
	Under-Five Mortality Survey 2002 (Direct)	1
	Fertility and Reproductive Health Survey 2007 (Direct)	1
	Fertility and Reproductive Health Survey 2007 (Indirect)	0
	Multiple Indicator Cluster Survey 2009-2010 (Direct)	1
	Multiple Indicator Cluster Survey 2009-2010 (Indirect)	0
	Census 2014 (Indirect)	1
	Demographic and Health Survey 2015-2016 (Direct)	1
	VR from Central Statistical Organization 2011 (VR)	0
Namibia	Census 1991 (Indirect)	1
	Demographic and Health Survey 1992 (Direct)	0
	Demographic and Health Survey 1992 (Indirect)	0
	Demographic and Health Survey (MM adjusted) 1992 (Direct)	1
	Demographic and Health Survey 2000 (Direct)	0
	Demographic and Health Survey 2000 (Indirect)	0
	Demographic and Health Survey (MM adjusted) 2000 (Direct)	1
	Census 2001 (Household Deaths)	1
	Demographic and Health Survey 2006-2007 (Household Deaths)	0
	Demographic and Health Survey 2006-2007 (Direct)	0
	Demographic and Health Survey 2006-2007 (Indirect)	0
	Demographic and Health Survey (MM adjusted) 2006-2007 (Direct)	1
	Census 2011 (Indirect)	1
	Census 2011 (Household Deaths)	0
	Demographic and Health Survey 2013 (Direct)	0
	Demographic and Health Survey (MM adjusted) 2013 (Direct)	1
	Inter-censal Demographic Survey 2016 (Household Deaths)	0
	Inter-censal Demographic Survey 2016 (Indirect)	0
Nauru	Demographic and Health Survey 2007 (Direct)	1
	Census 2011 (Indirect)	1
	Vital Registration Data and Census 2000 (VR)	1
	Vital Statistics Report 2008-2013 (VR)	0
	Vital Statistics Report (preliminary) 2015-2017 (VR)	0
	VR Submitted to WHO/UNIGME 2020 version 2020 (VR)	0
Nepal	Census 1971 (Indirect)	0

	Data series	Inclusion
	World Fertility Survey 1976 (Direct)	1
	World Fertility Survey 1976 (Indirect)	0
	Census 1981 (Indirect)	0
	Contraceptive Prevalence Survey 1981 (Indirect)	0
	Fertility and Family Planning Survey 1986 (Indirect)	0
	Census 1991 (Indirect)	0
	Fertility and Family Planning Survey 1991 (Direct)	1
	Fertility and Family Planning Survey 1991 (Indirect)	0
	Demographic and Health Survey 1996 (Direct)	1
	Demographic and Health Survey 1996 (Indirect)	0
	Demographic and Health Survey 2001 (Direct)	1
	Demographic and Health Survey 2001 (Indirect)	0
	Census 2001 (Household Deaths)	0
	Census 2001 (Indirect)	0
	Demographic and Health Survey 2006 (Direct)	1
	Demographic and Health Survey 2006 (Indirect)	0
	Demographic and Health Survey 2011 (Direct)	1
	Multiple Indicator Cluster Survey 2014 (Direct)	1
	Demographic and Health Survey 2016 (Direct)	1
Netherlands	VR Submitted to WHO/UNIGME 2020 version 2020 (VR)	1
New Zealand	Census 2006 (Household Deaths)	0
	VR Submitted to WHO/UNIGME 2020 version 2020 (VR)	1
Nicaragua	Census 1971 (Indirect)	1
	Enc.Demografica Nacional 1978 (Indirect)	1
	Enc.Socio-Demografica Nicaraguense 1985 (Indirect)	1
	Family Health Survey 1992-1993 (Direct)	1
	Family Health Survey 1992-1993 (Indirect)	0
	Census 1995 (Household Deaths)	0
	Census 1995 (Indirect)	1
	Demographic and Health Survey 1998 (Direct)	1
	Demographic and Health Survey 1998 (Indirect)	0
	Demographic and Health Survey 2001 (Direct)	1
	Demographic and Health Survey 2001 (Indirect)	0
	Living Standards Measurement Survey 2001 (Household Deaths)	0
	Census 2005 (Household Deaths)	0
	Census 2005 (Indirect)	1
	Encuesta Nicaraguense de Demografia y Salud 2006-2007 (Direct)	1
	Encuesta Nicaraguense de Demografia y Salud 2011-2012 (Direct)	1
	UNPD Demographic Yearbook Data 2020 version 2020 (VR)	0
	VR Submitted to WHO/UNIGME 2020 version 2020 (VR)	1
Niger	Survey 1960 (Indirect)	0
	Census 1988 (Indirect)	0
	Demographic and Health Survey 1992 (Direct)	1
	Demographic and Health Survey 1992 (Indirect)	0
	Multiple Indicator Cluster Survey 1996 (Direct)	1
	Multiple Indicator Cluster Survey 1996 (Indirect)	0
	Demographic and Health Survey 1998 (Direct)	1
	Demographic and Health Survey 1998 (Indirect)	0
	Multiple Indicator Cluster Survey 2000 (Indirect)	1
	Census 2001 (Household Deaths)	0
	Census 2001 (Indirect)	0
	Demographic and Health Survey 2006 (Direct)	1
	Demographic and Health Survey 2006 (Indirect)	0

	Data series	Inclusion
	Child Survival and Mortality Survey New 2010 (Direct)	1
	Demographic and Health Survey 2012 (Direct)	1
	Etude Nationale d'Evaluation des Indicateurs Socioeconomiques et Demographiques (Preliminary) 2015 (Direct)	0
Nigeria	Malumfashi DSS 1962-1977 (Household Deaths)	0
	Rural Demographic Sample Survey 1965-1966 (Household Deaths)	1
	Survey 1971-1973 (Indirect)	0
	World Fertility Survey 1981-1982 (Direct)	0
	World Fertility Survey 1981-1982 (Indirect)	0
	Demographic and Health Survey 1986 (Direct)	0
	Demographic and Health Survey 1990 (Direct)	1
	Demographic and Health Survey 1990 (Indirect)	0
	Multiple Indicator Cluster Survey 1995 (Indirect)	0
	Demographic and Health Survey 1999 (Direct)	0
	Demographic and Health Survey 1999 (Indirect)	0
	Multiple Indicator Cluster Survey 1999 (Indirect)	0
	Demographic and Health Survey 2003 (Direct)	1
	Demographic and Health Survey 2003 (Indirect)	0
	Multiple Indicator Cluster Survey 2007 (Indirect)	0
	Demographic and Health Survey 2008 (Household Deaths)	0
	Demographic and Health Survey 2008 (Direct)	1
	Demographic and Health Survey 2008 (Indirect)	0
	GHS Panel Survey 2010 (Household Deaths)	0
	Malaria Indicator Survey 2010 (Direct)	1
	Multiple Indicator Cluster Survey 2011 (Indirect)	1
	GHS Panel Survey 2012-2013 (Household Deaths)	0
	Demographic and Health Survey 2013 (Household Deaths)	0
	Demographic and Health Survey 2013 (Direct)	1
	Malaria Indicator Survey 2015 (Indirect)	0
	Multiple Indicator Cluster Survey 2016-2017 (Direct)	0
	Demographic and Health Survey (Preliminary) 2018 (Direct)	1
Niue	Vital Statistics Report 2012-2016 (VR)	0
	Recalculated VR Submitted to WHO/UNIGME 2020 version 2020 (VR)	1
	VR Submitted to WHO/UNIGME 2020 version 2020 (VR)	0
Norway	VR Submitted to WHO/UNIGME 2020 version 2020 (VR)	1
Oman	Socio-Demographic Survey in 5 towns 1975 (Indirect)	1
	Socio-Demographic Survey 1977-1979 (Indirect)	1
	Child Health Survey 1988 (Direct)	1
	Child Health Survey 1988 (Indirect)	0
	Census 1993 (Indirect)	1
	Family Health Survey 1995 (Direct)	1
	Family Health Survey 1995 (Indirect)	0
	Comprehensive Health Survey for Evaluation and Reproductive Health 2000 (Indirect)	1
	Census 2003 (Household Deaths)	1
	Census 2003 (Indirect)	1
	Multiple Indicator Cluster Survey 2014 (Direct)	0
	Data from Ministry of Health Annual Health Report 2012 (VR)	0
	Data from Ministry of Health Annual Health Report 2013 (VR)	1
	Data from Ministry of Health Annual Health Report 2014 (VR)	1
	Data from Ministry of Health Annual Health Report 2015 (VR)	1
	Data from Ministry of Health Annual Health Report 2016 (VR)	1
	Data from Ministry of Health Annual Health Report 2017 (VR)	1
	Data from Ministry of Health Annual Health Report 2018 (VR)	1
	VR Submitted to WHO/UNIGME 2020 version 2020 (VR)	0

	Data series	Inclusion
Pakistan	Population Growth Estimation Experiment 1962-1965 (Household Deaths)	0
	Population Growth Estimation Experiment 1962-1965 (Direct)	1
	Population Growth Survey I 1971 (Direct)	1
	World Fertility Survey 1975 (Direct)	1
	Population Growth Survey II 1976-1978 (Direct)	1
	Labour Force and Migration Survey 1980 (Direct)	1
	Census 1981 (Indirect)	1
	Contraceptive Prevalence Survey 1984 (Indirect)	1
	Demographic Survey 1984 (Indirect)	0
	Demographic Survey 1988 (Direct)	1
	Demographic Survey 1988 (Indirect)	0
	Demographic and Health Survey 1990-1991 (Direct)	1
	Demographic and Health Survey 1990-1991 (Indirect)	0
	Living Standards Survey 1991 (Direct)	1
	Living Standards Survey 1991 (Indirect)	0
	Contraceptive Prevalence Survey 1993 (Indirect)	0
	Contraceptive Prevalence Survey 1994-1995 (Indirect)	1
	Integrated Household Survey 1996 (Indirect)	1
	Pakistan Fertility and Family Planning Survey 1996 (Direct)	1
	Pakistan Fertility and Family Planning Survey 1996 (Indirect)	0
	Census 1998 (Indirect)	1
	Integrated Household Survey 1998 (Direct)	1
	Integrated Household Survey 1998 (Indirect)	0
	Reproductive Health and Family Planning Survey 2000-2001 (Direct)	1
	Reproductive Health and Family Planning Survey 2000-2001 (Indirect)	0
	Pakistan Demographic Survey 2000-2007 (Household Deaths)	1
	Integrated Household Survey 2001 (Direct)	1
	Integrated Household Survey 2001 (Indirect)	0
	Demographic and Health Survey 2006-2007 (Direct)	1
	Demographic and Health Survey 2006-2007 (Indirect)	0
	Demographic and Health Survey 2012-2013 (Direct)	1
	Social & Living Standards Measurement Survey 2013-2014 (Direct)	1
	Demographic and Health Survey 2017-2018 (Direct)	1
	Social & Living Standards Measurement Survey 2018-2019 (Direct)	1
	Pakistan Demographic Survey 1984-1999 (VR)	1
Palau	Census 1995 (Indirect)	0
	Census 2000 (Indirect)	0
	Census 2005 (Indirect)	0
	Recalculated VR Submitted to WHO/UNIGME 2020 version 2020 (VR)	1
	VR Submitted to WHO/UNIGME 2020 version 2020 (VR)	0
Panama	TABLAS DE VIDA NACIONALES (Life Table)	1
	World Fertility Survey 1975-1976 (Direct)	1
	Encuesta Demografica Nacional 1975-1977 (Direct)	0
	Encuesta Demografica Nacional 1975-1977 (Indirect)	0
	Census 1980 (Indirect)	1
	Census 1990 (Indirect)	1
	Census 2000 (Household Deaths)	0
	Census 2000 (Indirect)	1
	Census 2010 (Indirect)	1
	Vital Registration and Census (VR)	0
	Vital Registration from National Statistical Institute (VR)	0
	UNPD Demographic Yearbook Data 2020 version 2020 (VR)	0
	VR Submitted to WHO/UNIGME 2020 version 2020 (VR)	0
	Census 1971 (Indirect)	1

	Data series	Inclusion
Papua New Guinea	Census 1980 (Indirect)	0
	Demographic and Health Survey 1991 (Indirect)	1
	Demographic and Health Survey 1996 (Direct)	1
	Demographic and Health Survey 1996 (Indirect)	0
	Census 2000 (Indirect)	1
	Demographic and Health Survey 2006 (Direct)	1
	Demographic and Health Survey 2016-2018 (Direct)	1
	VR Submitted to WHO/UNIGME 2020 version 2020 (VR)	0
Paraguay	Census 1972 (Indirect)	1
	National Survey of Contraceptive Use 1977 (Indirect)	1
	National Fertility Survey 1979 (Indirect)	0
	World Fertility Survey 1979 (Direct)	1
	Census 1982 (Indirect)	1
	Demographic and Health Survey 1990 (Direct)	1
	Demographic and Health Survey 1990 (Indirect)	0
	Census 1992 (Indirect)	1
	National Survey of Demography and Reproductive Health 1995-1996 (Direct)	1
	National Survey of Demography and Reproductive Health 1995-1996 (Indirect)	0
	Census 2002 (Household Deaths)	0
	Census 2002 (Indirect)	1
	Reproductive Health Survey 2004 (Direct)	1
	Encuesta Nacional de Demografia y Salud Sexual y Reproductiva 2008 (Direct)	1
	Multiple Indicator Cluster Survey 2016 (Direct)	0
	VR Ministerio de Salud Pulbica y Bienestar Social 2016 (VR)	0
	VR Submitted to WHO/UNIGME 2020 version 2020 (VR)	0
Peru	Census 1972 (Indirect)	1
	Demographic Survey 1974-1976 (Direct)	1
	Demographic Survey 1974-1976 (Indirect)	0
	World Fertility Survey 1977-1978 (Direct)	1
	World Fertility Survey 1977-1978 (Indirect)	0
	Census 1981 (Indirect)	1
	Contraceptive Prevalence Survey 1981 (Indirect)	1
	Demographic and Health Survey 1986 (Direct)	1
	Demographic and Health Survey 1986 (Indirect)	0
	Demographic and Health Survey 1991-1992 (Direct)	1
	Demographic and Health Survey 1991-1992 (Indirect)	0
	Census 1993 (Indirect)	1
	Demographic and Health Survey 1996 (Direct)	1
	Demographic and Health Survey 1996 (Indirect)	0
	Demographic and Health Survey 2000 (Direct)	1
	Demographic and Health Survey 2000 (Indirect)	0
	Demographic and Health Survey 2004-2008 (Direct)	1
	Demographic and Health Survey 2004-2008 (Indirect)	0
	Census 2007 (Indirect)	1
	Demographic and Health Survey 2009 (Direct)	1
	Demographic and Health Survey 2010 (Direct)	1
	Demographic and Health Survey 2011 (Direct)	1
	Demographic and Health Survey 2012 (Direct)	1
	Demographic and Health Survey 2013 (Direct)	1
	National Demographic and Health Survey 2014 (Direct)	1
	National Demographic and Health Survey 2017 (Direct)	1
	Census 2017 (Indirect)	1
	National Demographic and Health Survey 2018 (Direct)	1
	Encuesta Demografica y de Salud Familiar 2019 (Direct)	1

	Data series	Inclusion
	Vital Registration (26 March 2009) 2009 (VR)	0
	VR Submitted to WHO/UNIGME 2020 version 2020 (VR)	0
Philippines	Census 1970 (Indirect)	0
	World Fertility Survey 1978 (Direct)	1
	Census 1980 (Indirect)	0
	Demographic Survey 1988 (Indirect)	1
	Demographic and Health Survey 1993 (Direct)	1
	Demographic and Health Survey 1993 (Indirect)	0
	Demographic and Health Survey 1998 (Direct)	1
	Demographic and Health Survey 1998 (Indirect)	0
	Demographic and Health Survey 2003 (Direct)	1
	Demographic and Health Survey 2003 (Indirect)	0
	Family Planning Survey 2006 (Direct)	0
	Family Planning Survey 2006 (Indirect)	1
	Demographic and Health Survey 2008 (Direct)	1
	Demographic and Health Survey 2008 (Indirect)	0
	Family Health Survey 2011 (Direct)	1
	National Demographic and Health Survey 2013 (Direct)	1
	Demographic and Health Survey 2017 (Direct)	1
	VR Submitted to WHO/UNIGME 2020 version 2020 (VR)	0
Poland	Census 2011 (Household Deaths)	0
	UNPD Demographic Yearbook Data 2020 version 2020 (VR)	1
	VR Submitted to WHO/UNIGME 2020 version 2020 (VR)	1
Portugal	World Fertility Survey 1979-1980 (Direct)	0
	Census 2011 (Household Deaths)	0
	UNPD Demographic Yearbook Data 2020 version 2020 (VR)	1
	VR Submitted to WHO/UNIGME 2020 version 2020 (VR)	1
Qatar	Child Health Survey 1987 (Direct)	1
	Child Health Survey 1987 (Indirect)	0
	Gulf Family Health Survey 1998 (Direct)	0
	Gulf Family Health Survey 1998 (Indirect)	0
	Census 2004 (Indirect)	0
	Statistics Authority, publication of vital statistics 2012 (VR)	0
	Vital Statistics, Annual Bulletin 2015 (VR)	0
	VR Submitted to WHO/UNIGME 2020 version 2020 (VR)	1
Republic of Korea	National Life Table (Life Table)	0
	National Life Table 1970-2015 (Life Table)	1
	Census 1970 (Indirect)	1
	Korea Fertility Survey 1974 (Indirect)	0
	World Fertility Survey 1974 (Direct)	1
	Census 1975 (Indirect)	1
	Census 1980 (Indirect)	1
	Census 1985 (Indirect)	1
	Census 1990 (Indirect)	1
	VR Submitted to WHO/UNIGME 2020 version 2020 (VR)	1
Republic of Moldova	Census 1989 (Indirect)	1
	Demographic and Health Survey 2005 (Direct)	1
	Demographic and Health Survey 2005 (Indirect)	0
	Multiple Indicator Cluster Survey 2012 (Direct)	1
	VR Submitted to WHO/UNIGME 2020 version 2020 (VR)	1
Republic of North Macedonia	Multiple Indicator Cluster Survey 2005 (Indirect)	0
	Multiple Indicator Cluster Survey 2018-2019 (Direct)	0
	VR Submitted to WHO/UNIGME 2020 version 2020 (VR)	1
Romania	Reproductive Health Survey 1999 (Direct)	0

	Data series	Inclusion
	UNPD Demographic Yearbook Data 2020 version 2020 (VR)	1
	VR Submitted to WHO/UNIGME 2020 version 2020 (VR)	1
Russian Federation	Census 1989 (Indirect)	1
	Trans MONEE Vital Registration (VR)	0
	VR Submitted to WHO/UNIGME 2020 version 2020 (VR)	1
Rwanda	Demographic Survey 1970 (Indirect)	1
	Census 1978 (Indirect)	1
	World Fertility Survey 1983 (Direct)	1
	World Fertility Survey 1983 (Indirect)	0
	Census 1991 (Indirect)	1
	Demographic and Health Survey 1992 (Direct)	0
	Demographic and Health Survey 1992 (Indirect)	0
	Demographic and Health Survey (MM adjusted) 1992 (Direct)	1
	Socio-demographic Survey 1996 (Indirect)	0
	Demographic and Health Survey 2000 (Direct)	0
	Demographic and Health Survey 2000 (Indirect)	0
	Demographic and Health Survey (MM adjusted) 2000 (Direct)	1
	Census 2002 (Household Deaths)	0
	Census 2002 (Indirect)	0
	Demographic and Health Survey 2005 (Direct)	0
	Demographic and Health Survey 2005 (Indirect)	0
	Demographic and Health Survey (MM adjusted) 2005 (Direct)	1
	Demographic and Health Survey Adjusted by adding 20 points 2007-2008 (Indirect)	0
	Interim Demographic and Health Survey 2007-2008 (Direct)	0
	Interim Demographic and Health Survey (MM adjusted) 2007-2008 (Direct)	1
	Demographic and Health Survey 2010 (Direct)	0
	Demographic and Health Survey 2010 (Indirect)	0
	Demographic and Health Survey (MM adjusted) 2010 (Direct)	1
	Census 2012 (Indirect)	1
	Census 2012 (Household Deaths)	0
	Malaria Indicator Survey 2013 (Indirect)	1
	Demographic and Health Survey 2014-2015 (Direct)	0
	Demographic and Health Survey (MM adjusted) 2014-2015 (Direct)	1
Saint Kitts and Nevis	Recalculated VR Submitted to WHO/UNIGME 2020 version 2020 (VR)	1
	VR Submitted to WHO/UNIGME 2020 version 2020 (VR)	0
Saint Lucia	Recalculated VR Submitted to WHO/UNIGME 2020 version 2020 (VR)	1
	UNPD Demographic Yearbook Data 2020 version 2020 (VR)	1
	VR Submitted to WHO/UNIGME 2020 version 2020 (VR)	0
Saint Vincent and the Grenadines	Recalculated VR Submitted to WHO/UNIGME 2020 version 2020 (VR)	1
	UNPD Demographic Yearbook Data 2020 version 2020 (VR)	1
	VR Submitted to WHO/UNIGME 2020 version 2020 (VR)	0
Samoa	Census 1956 (Indirect)	1
	Census 1961 (Indirect)	1
	Census 1966 (Indirect)	1
	Census 1971 (Indirect)	0
	Census 1976 (Indirect)	1
	Demographic and Health Survey 1999 (Household Deaths)	1
	Demographic and Health Survey 1999 (Indirect)	1
	Demographic and Vital Statistics Survey 2000 (Indirect)	1
	Population and Housing Census 2001 (Indirect)	1
	Population and Housing Census 2006 (Household Deaths)	0
	Population and Housing Census 2006 (Indirect)	1
	Demographic and Health Survey 2009 (Direct)	0
	Demographic and Health Survey 2009 (Indirect)	0

	Data series	Inclusion
	Population and Housing Census 2011 (Household Deaths)	1
	Demographic and Health Survey 2014 (Direct)	0
	Population and Housing Census 2016 (Household Deaths)	0
	Population and Housing Census 2016 (Indirect)	1
	VR Submitted to WHO/UNIGME 2020 version 2020 (VR)	0
San Marino	Recalculated VR Submitted to WHO/UNIGME 2020 version 2020 (VR)	1
	UNPD Demographic Yearbook Data 2020 version 2020 (VR)	0
	VR Submitted to WHO/UNIGME 2020 version 2020 (VR)	0
Sao Tome and Principe	Census 1980 (Indirect)	1
	Census 1991 (Indirect)	1
	Multiple Indicator Cluster Survey 2000 (Indirect)	1
	Multiple Indicator Cluster Survey 2006 (Indirect)	0
	Demographic and Health Survey 2008-2009 (Direct)	1
	Demographic and Health Survey 2008-2009 (Indirect)	0
	Census 2012 (Household Deaths)	0
	Multiple Indicator Cluster Survey 2014 (Direct)	1
	VR Submitted to WHO/UNIGME 2020 version 2020 (VR)	0
Saudi Arabia	Child Health Survey 1987 (Indirect)	1
	Family Health Survey 1996 (Indirect)	1
	Gulf Family Health Survey 1996 (Direct)	0
	Demographic Survey 1999 (Household Deaths)	1
	Demographic Survey 2000 (Household Deaths)	1
	Census 2004 (Household Deaths)	1
	Demographic Survey 2007 (Household Deaths)	1
	Demographic Survey 2016 (Household Deaths)	1
	VR Submitted to WHO/UNIGME 2020 version 2020 (VR)	0
Senegal	Survey 1960-1961 (Indirect)	1
	Multiround Survey 1970-1971 (Household Deaths)	1
	World Fertility Survey 1978 (Direct)	1
	World Fertility Survey 1978 (Indirect)	0
	Multiround Survey 1978-1979 (Household Deaths)	1
	Demographic and Health Survey 1986 (Direct)	1
	Demographic and Health Survey 1986 (Indirect)	0
	Demographic and Health Survey 1992-1993 (Direct)	1
	Demographic and Health Survey 1992-1993 (Indirect)	0
	Multiple Indicator Cluster Survey 1996 (Indirect)	0
	Demographic and Health Survey 1997 (Direct)	1
	Demographic and Health Survey 1997 (Indirect)	0
	Demographic and Health Survey 1999-2000 (Direct)	1
	Demographic and Health Survey 1999-2000 (Indirect)	0
	Census 2002 (Household Deaths)	0
	Census 2002 (Indirect)	0
	Demographic and Health Survey 2005 (Direct)	1
	Demographic and Health Survey 2005 (Indirect)	0
	Malaria Indicator Survey 2006 (Indirect)	1
	Malaria Indicator Survey 2008-2009 (Direct)	1
	Malaria Indicator Survey 2008-2009 (Indirect)	0
	Demographic and Health Survey 2010-2011 (Direct)	1
	Demographic and Health Survey 2012-2013 (Direct)	1
	Census 2013 (Household Deaths)	0
	Census 2013 (Indirect)	0
	Demographic and Health Survey 2014 (Direct)	1
	Demographic and Health Survey 2015 (Direct)	1
	Demographic and Health Survey 2016 (Direct)	1

	Data series	Inclusion
	Demographic and Health Survey 2017 (Direct)	1
	Demographic and Health Survey 2018 (Direct)	1
Serbia	Multiple Indicator Cluster Survey 2010 (Indirect)	0
	Vital Registration data from Statistical Office of the Republic of Serbia and Institute of Public Health of Serbia (VR)	0
	VR Submitted to WHO/UNIGME 2020 version 2020 (VR)	1
Seychelles	Census 1960 (Household Deaths)	0
	Census 1960 (Indirect)	1
	Census 1971 (Indirect)	1
	Recalculated VR Submitted to WHO/UNIGME 2020 version 2020 (VR)	1
	VR Submitted to WHO/UNIGME 2020 version 2020 (VR)	0
Sierra Leone	National Fertility Survey 1969 (Indirect)	0
	Pilot Census 1973 (Indirect)	1
	Census 1974 (Indirect)	1
	National Survey 1977 (Household Deaths)	1
	Census 1985 (Indirect)	1
	DSMS 1992 (Household Deaths)	1
	DSMS 1992 (Indirect)	1
	Multiple Indicator Cluster Survey 2000 (Indirect)	1
	Pilot Census 2003 (Indirect)	0
	Census 2004 (Household Deaths)	1
	Census 2004 (Indirect)	1
	Multiple Indicator Cluster Survey 2005-2006 (Indirect)	1
	Demographic and Health Survey 2008 (Direct)	0
	Demographic and Health Survey 2008 (Indirect)	0
	Multiple Indicator Cluster Survey 2010 (Indirect)	1
	Demographic and Health Survey 2013 (Direct)	1
	Census 2015 (Household Deaths)	0
	Census 2015 (Indirect)	1
	Malaria Indicator Survey 2016 (Indirect)	1
	Multiple Indicator Cluster Survey 2017 (Direct)	0
	Demographic and Health Survey 2019 (Direct)	1
	VR from Health Management Information System 2012 (VR)	0
Singapore	UNPD Demographic Yearbook Data 2020 version 2020 (VR)	1
	VR Submitted to WHO/UNIGME 2020 version 2020 (VR)	1
Slovakia	VR Submitted to WHO/UNIGME 2020 version 2020 (VR)	1
Slovenia	Recalculated VR Submitted to WHO/UNIGME 2020 version 2020 (VR)	1
	VR Submitted to WHO/UNIGME 2020 version 2020 (VR)	0
Solomon Islands	Census 1976 (Indirect)	1
	Census 1986 (Indirect)	1
	Census 1999 (Indirect)	1
	Demographic and Health Survey 2007 (Direct)	1
	Demographic and Health Survey 2007 (Indirect)	0
	Census 2009 (Indirect)	1
	Census 2009 (Household Deaths)	0
	Demographic and Health Survey 2015 (Direct)	1
Somalia	Multiple Indicator Cluster Survey 1999 (Indirect)	1
	Multiple Indicator Cluster Survey 2006 (Direct)	1
	Multiple Indicator Cluster Survey 2006 (Indirect)	0
South Africa	Demographic and Health Survey 1987-1989 (Direct)	0
	HSRC 1990 (Direct)	1
	Household Survey 1993 (Household Deaths)	0
	Household Survey 1995 (Household Deaths)	0
	Household Survey 1996 (Household Deaths)	0

	Data series	Inclusion
	Household Survey 1997 (Household Deaths)	0
	Demographic and Health Survey 1998 (Direct)	0
	Demographic and Health Survey 1998 (Indirect)	0
	Demographic and Health Survey (MM adjusted) 1998 (Direct)	1
	Household Survey 1998 (Household Deaths)	0
	Census 2001 (Household Deaths)	1
	Census 2001 (Indirect)	0
	Demographic and Health Survey 2004 (Direct)	0
	Demographic and Health Survey (MM adjusted) 2004 (Direct)	0
	Community Survey 2007 (Household Deaths)	1
	Census 2011 (Household Deaths)	0
	Demographic and Health Survey 2016 (Direct)	0
	Demographic and Health Survey (MM adjusted) 2016 (Direct)	1
	Rapid Mortality Surveillance (Preliminary) 2019 (VR)	1
	VR Submitted to WHO/UNIGME 2020 version 2020 (VR)	0
South Sudan	Census 1955-1956 (Household Deaths)	1
	Census 1973 (Indirect)	0
	Census 1983 (Indirect)	0
	UNICEF Survey 1985 (Indirect)	0
	UNICEF Survey (Urban) 1985 (Indirect)	0
	Census 1993 (Household Deaths)	1
	Census 1993 (Indirect)	1
	Demography of Forced Migration Project DFMP survey 1999 (Indirect)	0
	Sudan Household Health Survey 2006 (Household Deaths)	1
	Sudan Household Health Survey 2006 (Indirect)	0
	Census 2008 (Household Deaths)	0
	Census 2008 (Indirect)	1
	Household Health Survey 2010 (Direct)	0
Spain	VR Submitted to WHO/UNIGME 2020 version 2020 (VR)	1
Sri Lanka	National life table (Life Table)	1
	Census 1971 (Indirect)	1
	World Fertility Survey 1975 (Direct)	1
	World Fertility Survey 1975 (Indirect)	0
	Demographic and Health Survey 1987 (Direct)	1
	Demographic and Health Survey 1987 (Indirect)	0
	Demographic and Health Survey 1993 (Direct)	1
	Demographic and Health Survey 2000 (Direct)	1
	Demographic and Health Survey 2006 (Direct)	1
	Demographic and Health Survey 2006 (Indirect)	0
	Census 2012 (Indirect)	1
	Demographic and Health Survey 2016 (Direct)	1
	VR Submitted to WHO/UNIGME 2020 version 2020 (VR)	1
State of Palestine	FALCOT-survey 1992 (Indirect)	1
	Demographic Survey 1995 (Direct)	1
	Demographic Survey 1995 (Indirect)	0
	Census 1997 (Indirect)	1
	Health Survey 2000 (Direct)	1
	Health Survey 2000 (Indirect)	0
	Demographic and Health Survey 2004 (Direct)	1
	Demographic and Health Survey 2004 (Indirect)	0
	PAPFAM Family Health Survey 2006 (Direct)	1
	PAPFAM Family Health Survey 2006 (Indirect)	0
	Census 2007 (Indirect)	1
	Multiple Indicator Cluster Survey-Family Health Survey 2010 (Direct)	1

	Data series	Inclusion
	Multiple Indicator Cluster Survey 2014 (Direct)	1
	Census 2017 (Indirect)	1
	VR Submitted to WHO/UNIGME 2020 version 2020 (VR)	0
Sudan	Census 1955-1956 (Household Deaths)	1
	Census 1973 (Indirect)	1
	World Fertility Survey 1978-1979 (Direct)	1
	World Fertility Survey (Household Survey) 1978-1979 (Indirect)	0
	World Fertility Survey (Individual Survey) 1978-1979 (Indirect)	0
	Census 1983 (Indirect)	1
	Demographic and Health Survey 1989-1990 (Direct)	1
	Demographic and Health Survey 1989-1990 (Indirect)	0
	PAPCHILD Maternal and Child Health Survey 1992 (Direct)	1
	Census 1993 (Household Deaths)	1
	Census 1993 (Indirect)	1
	Sudanese Maternal and Child Health Survey SMCHS 1993 (Indirect)	1
	Safe Motherhood Survey 1999 (Direct)	1
	Safe Motherhood Survey 1999 (Indirect)	0
	Sudan Household Health Survey 2006 (Household Deaths)	1
	Sudan Household Health Survey 2006 (Indirect)	1
	Census 2008 (Household Deaths)	0
	Census 2008 (Indirect)	1
	Multiple Indicator Cluster Survey 2010 (Direct)	1
	Multiple Indicator Cluster Survey 2014 (Direct)	1
Suriname	Multiple Indicator Cluster Survey 1999-2000 (Indirect)	1
	Census 2004 (Indirect)	0
	Multiple Indicator Cluster Survey 2006 (Indirect)	1
	Multiple Indicator Cluster Survey 2018 (Direct)	1
	VR Submitted to WHO/UNIGME 2020 version 2020 (VR)	0
Sweden	VR Submitted to WHO/UNIGME 2020 version 2020 (VR)	1
Switzerland	VR Submitted to WHO/UNIGME 2020 version 2020 (VR)	1
Syrian Arab Republic	Census 1970 (Indirect)	1
	Census 1976 (Indirect)	1
	World Fertility Survey 1978 (Direct)	1
	World Fertility Survey 1978 (Indirect)	0
	Census 1981 (Indirect)	1
	EPI/CDD and Child Mortality Survey 1990 (Indirect)	1
	PAPCHILD Maternal and Child Health Survey 1993 (Direct)	1
	Census 1994 (Indirect)	1
	PAPFAM Family Health Survey 2001 (Direct)	1
	Population and Housing Census 2004 (Household Deaths)	1
	Multiple Indicator Cluster Survey 2006 (Indirect)	1
	Health survey for causes of child deaths 2007-2008 (Household Deaths)	1
	PAPFAM Family Health Survey 2009 (Direct)	1
	Health survey for causes of child deaths 2017-2018 (Household Deaths)	0
	Vital Registration/hospitals/funeral offices Data 2009 (VR)	0
	VR Submitted to WHO/UNIGME 2020 version 2020 (VR)	0
Tajikistan	Census 1989 (Indirect)	1
	Tajikistan Living Standards Survey 1999 (Direct)	1
	Tajikistan Living Standards Survey 1999 (Indirect)	0
	Multiple Indicator Cluster Survey 2000 (Indirect)	1
	Demographic Survey 2002 (Direct)	1
	Demographic Survey 2002 (Indirect)	0
	Tajikistan Living Standards Survey 2003 (Direct)	0
	Tajikistan Living Standards Survey 2003 (Indirect)	0

	Data series	Inclusion
	Multiple Indicator Cluster Survey 2005 (Indirect)	1
	Tajikistan Living Standards Survey 2007 (Direct)	0
	Tajikistan Living Standards Survey 2007 (Indirect)	0
	Census 2010 (Indirect)	0
	Survey on Infant, Child and Maternal Mortality 2010 (Direct)	0
	Survey on Infant, Child and Maternal Mortality 2010 (Indirect)	0
	Demographic and Health Survey 2012 (Direct)	1
	Demographic and Health Survey 2017 (Direct)	1
	Vital Registration data from Statistical Yearbook Health Status and Performance of Health Facilities 2010 2010 (VR)	0
	Vital Registration data from State Agency for Statistics 2012 2012 (VR)	0
	VR Submitted to WHO/UNIGME 2020 version 2020 (VR)	1
Thailand	Life Table by Hill 1980-1990 Intercensal Death (Life Table)	1
	Life Table by Hill 1990-2000 Intercensal Death (Life Table)	1
	Life Table in 2002 by Hill (Life Table)	1
	National Life Tables (Life Table)	1
	Census 1970 (Indirect)	1
	Survey of Population Change 1974-1975 (Direct)	1
	Survey of Population Change 1974-1975 (Indirect)	0
	World Fertility Survey 1975 (Direct)	1
	World Fertility Survey 1975 (Indirect)	0
	National Fertility Survey 1979 (Indirect)	1
	Census 1980 (Indirect)	1
	Contraceptive Prevalence Survey 1981 (Indirect)	1
	Contraceptive Prevalence Survey 1984 (Indirect)	1
	Survey of Population Change 1985-1986 (Direct)	1
	Survey of Population Change 1985-1986 (Indirect)	0
	Demographic and Health Survey 1987 (Direct)	1
	Demographic and Health Survey 1987 (Indirect)	0
	Survey of Population Change 1989 (Direct)	1
	Survey of Population Change 1989 (Indirect)	0
	Census 1990 (Indirect)	0
	Survey of Population Change 1995-1996 (Direct)	1
	Survey of Population Change 1995-1996 (Indirect)	0
	Contraceptive Prevalence Survey 1996 (Indirect)	1
	Census 2000 (Indirect)	1
	Multiple Indicator Cluster Survey 2005-2006 (Indirect)	1
	Survey of Population Change 2005-2006 (Direct)	1
	Survey of Population Change 2005-2006 (Indirect)	0
	Census 2010 (Indirect)	0
	Multiple Indicator Cluster Survey 2012-2013 (Indirect)	1
	Survey of Population Change 2015-2016 (Indirect)	0
	Multiple Indicator Cluster Survey 2015-2016 (Indirect)	0
	Vital Registration (VR)	0
	Vital Registration from Ministry of Public Health 2015 (VR)	0
	VR Submitted to WHO/UNIGME 2020 version 2020 (VR)	1
Timor-Leste	Census 1990 (Indirect)	0
	ICS 1995 (Indirect)	0
	Living Standards Survey 2001 (Indirect)	1
	Multiple Indicator Cluster Survey 2002 (Indirect)	1
	Demographic and Health Survey 2003 (Direct)	1
	Census 2004 (Indirect)	1
	Demographic and Health Survey 2009-2010 (Direct)	1
	Census 2010 (Indirect)	1

	Data series	Inclusion
	Demographic and Health Survey 2016 (Direct)	0
Togo	Demographic survey 1961 (Household Deaths)	0
	Demographic survey 1961 (Indirect)	1
	Demographic Survey 1971 (Indirect)	1
	Census 1981 (Household Deaths)	0
	Demographic and Health Survey 1988 (Direct)	1
	Demographic and Health Survey 1988 (Indirect)	0
	Demographic and Health Survey 1998 (Direct)	1
	Demographic and Health Survey 1998 (Indirect)	0
	Multiple Indicator Cluster Survey 2006 (Indirect)	1
	Census 2010 (Household Deaths)	0
	Census 2010 (Indirect)	1
	Multiple Indicator Cluster Survey 2010 (Indirect)	1
	Demographic and Health Survey 2013-2014 (Direct)	1
	Multiple Indicator Cluster Survey 2017 (Direct)	1
Tonga	Census 1976 (Indirect)	1
	Census 1986 (Indirect)	1
	Census 1996 (Indirect)	1
	Census 2006 (Household Deaths)	0
	Census 2006 (Indirect)	1
	National Demographic and Health Survey 2012 (Direct)	1
	Multiple Indicator Cluster Survey (Preliminary) 2019 (Direct)	1
	Recalculated VR Submitted to WHO/UNIGME 2020 version 2020 (VR)	0
	VR Submitted to WHO/UNIGME 2020 version 2020 (VR)	0
Trinidad and Tobago	National Life Tables (Life Table)	0
	World Fertility Survey 1977 (Direct)	1
	World Fertility Survey 1977 (Indirect)	0
	Demographic and Health Survey 1987 (Direct)	1
	Demographic and Health Survey 1987 (Indirect)	0
	Multiple Indicator Cluster Survey 2000 (Indirect)	1
	Multiple Indicator Cluster Survey 2006 (Indirect)	1
	Population and Housing Census 2011 (Indirect)	0
	Multiple Indicator Cluster Survey 2011 (Indirect)	0
	Preliminary MoH Vital Registration Data 2019 (VR)	0
	UNPD Demographic Yearbook Data 2020 version 2020 (VR)	0
	VR Submitted to WHO/UNIGME 2020 version 2020 (VR)	0
Tunisia	National Demographic Survey 1968-1969 (Household Deaths)	1
	Census 1975 (Indirect)	1
	World Fertility Survey 1978 (Direct)	0
	World Fertility Survey 1978 (Indirect)	0
	Contraceptive Prevalence Survey 1983 (Indirect)	1
	Census 1984 (Indirect)	1
	Demographic and Health Survey 1988 (Direct)	1
	Demographic and Health Survey 1988 (Indirect)	0
	PAPCHILD Maternal and Child Health Survey 1994 (Direct)	1
	PAPFAM Family Health Survey 2001 (Direct)	1
	Census 2004 (Indirect)	1
	Multiple Indicator Cluster Survey 2011-2012 (Direct)	1
	Census 2014 (Indirect)	0
	Multiple Indicator Cluster Survey 2018 (Direct)	0
	Vital Registration from Institut National de la Statistique (VR)	1
	VR Submitted to WHO/UNIGME 2020 version 2020 (VR)	1
Turkey	Turkey Demographic Survey 1967 (Direct)	1
	Turkey Demographic Survey 1967 (Indirect)	0

	Data series	Inclusion
	Census 1970 (Indirect)	1
	Census 1975 (Indirect)	1
	Turkey Fertility Survey 1978 (Indirect)	0
	World Fertility Survey 1978 (Direct)	1
	Census 1980 (Indirect)	1
	Turkey Population and Health Survey 1983 (Indirect)	1
	Census 1985 (Indirect)	1
	Turkey Population and Health Survey 1988 (Direct)	1
	Turkey Population and Health Survey 1988 (Indirect)	0
	Demographic Survey 1989 (Indirect)	1
	Census 1990 (Indirect)	1
	Demographic and Health Survey 1993 (Direct)	1
	Demographic and Health Survey 1993 (Indirect)	0
	Demographic and Health Survey 1998 (Direct)	1
	Demographic and Health Survey 1998 (Indirect)	0
	National Verbal Autopsy Survey 2003 (Household Deaths)	0
	Demographic and Health Survey 2003-2004 (Direct)	1
	Demographic and Health Survey 2003-2004 (Indirect)	0
	Demographic and Health Survey 2008 (Direct)	1
	Turkey Child Mortality Survey 2010 (Direct)	0
	Turkey Child Mortality Survey 2012 (Direct)	0
	Demographic and Health Survey 2013 (Direct)	1
	Demographic and Health Survey 2018 (Indirect)	0
	VR Submitted to WHO/UNIGME 2020 version 2020 (VR)	1
Turkmenistan	Demographic and Health Survey 2000 (Direct)	1
	Demographic and Health Survey 2000 (Indirect)	0
	Multiple Indicator Cluster Survey 2006 (Indirect)	1
	Multiple Indicator Cluster Survey 2015-2016 (Direct)	0
	Multiple Indicator Cluster Survey 2019 (Direct)	1
	Vital Registration (VR)	0
	Transmonee Vital Registration Data 2008 version 2008 (VR)	0
	Vital Registration Data (Live Births) 2020 (VR)	0
	VR Submitted to WHO/UNIGME 2020 version 2020 (VR)	1
Tuvalu	Census 1991 (Indirect)	1
	Census 2002 (Direct)	1
	Demographic and Health Survey 2007 (Direct)	1
	Recalculated VR Submitted to WHO/UNIGME 2020 version 2020 (VR)	1
	VR Submitted to WHO/UNIGME 2020 version 2020 (VR)	0
Uganda	Census 1969 (Indirect)	1
	Demographic and Health Survey 1988-1989 (Direct)	0
	Demographic and Health Survey 1988-1989 (Indirect)	0
	Demographic and Health Survey (MM adjusted) 1988-1989 (Direct)	1
	Census 1991 (Direct)	1
	Demographic and Health Survey 1995 (Direct)	0
	Demographic and Health Survey 1995 (Indirect)	0
	Demographic and Health Survey (MM adjusted) 1995 (Direct)	1
	Demographic and Health Survey 2000-2001 (Direct)	0
	Demographic and Health Survey 2000-2001 (Indirect)	0
	Demographic and Health Survey (MM adjusted) 2000-2001 (Direct)	1
	Census 2002 (Household Deaths)	0
	Census 2002 (Direct)	1
	Demographic and Health Survey 2006 (Household Deaths)	0
	Demographic and Health Survey 2006 (Direct)	0
	Demographic and Health Survey 2006 (Indirect)	0

	Data series	Inclusion
	Demographic and Health Survey (MM adjusted) 2006 (Direct)	1
	Malaria Indicator Survey 2009-2010 (Direct)	0
	Malaria Indicator Survey 2009-2010 (Indirect)	0
	Malaria Indicator Survey (MM adjusted) 2009-2010 (Direct)	1
	Demographic and Health Survey 2011 (Direct)	0
	Demographic and Health Survey (MM adjusted) 2011 (Direct)	1
	AIDS Indicator Survey 2011 (Household Deaths)	0
	Census 2014 (Indirect)	1
	Malaria Indicator Survey 2014-2015 (Indirect)	1
	Demographic and Health Survey 2016 (Direct)	0
	Demographic and Health Survey (MM adjusted) 2016 (Direct)	1
	Malaria Indicator Survey 2018-2019 (Indirect)	1
Ukraine	Census 1989 (Indirect)	1
	Reproductive Health Survey 1999 (Direct)	1
	Census 2001 (Indirect)	1
	Multiple Indicator Cluster Survey 2005 (Indirect)	0
	Demographic and Health Survey 2007 (Direct)	1
	Demographic and Health Survey 2007 (Indirect)	0
	Multiple Indicator Cluster Survey 2012 (Direct)	0
	Multiple Indicator Cluster Survey 2012 (Indirect)	0
	Vital Registration Data from Human Mortality Database (VR)	0
	Vital Registration Data from State Statistics Committee of Ukraine (VR)	0
	VR Submitted to WHO/UNIGME 2020 version 2020 (VR)	1
United Arab Emirates	Census 1975 (Indirect)	1
	Census 1980 (Indirect)	1
	Child Health Survey 1987 (Indirect)	1
	Family Health Survey 1995 (Direct)	1
	Family Health Survey 1995 (Indirect)	0
	Vital Registration by Federal Competitiveness and Statistics Authority 2016 (VR)	1
	Vital Registration by Federal Competitiveness and Statistics Authority 2018 (VR)	1
	VR Submitted to WHO/UNIGME 2020 version 2020 (VR)	0
United Kingdom	VR Submitted to WHO/UNIGME 2020 version 2020 (VR)	1
United Republic of Tanzania	Census 1967 (Household Deaths)	0
	Census 1967 (Indirect)	1
	National Demographic Survey 1973 (Household Deaths)	0
	Census 1978 (Indirect)	1
	Census 1988 (Household Deaths)	0
	Census 1988 (Indirect)	1
	Demographic and Health Survey 1991-1992 (Direct)	0
	Demographic and Health Survey 1991-1992 (Indirect)	0
	Demographic and Health Survey (MM adjusted) 1991-1992 (Direct)	1
	Demographic and Health Survey 1996 (Direct)	0
	Demographic and Health Survey 1996 (Indirect)	0
	Demographic and Health Survey (MM adjusted) 1996 (Direct)	1
	Demographic and Health Survey 1999 (Direct)	0
	Demographic and Health Survey 1999 (Indirect)	0
	Demographic and Health Survey (MM adjusted) 1999 (Direct)	1
	Census 2002 (Household Deaths)	0
	Census 2002 (Indirect)	1
	Demographic and Health Survey 2004-2005 (Direct)	0
	Demographic and Health Survey 2004-2005 (Indirect)	0
	Demographic and Health Survey (MM adjusted) 2004-2005 (Direct)	1
	AIDS Indicator Survey 2007-2008 (Direct)	0

	Data series	Inclusion
	AIDS Indicator Survey 2007-2008 (Indirect)	0
	AIDS Indicator Survey (MM adjusted) 2007-2008 (Direct)	1
	AIDS Indicator Survey 2007-2008 (Household Deaths)	0
	National Panel Survey 2008-2009 (Household Deaths)	0
	Demographic and Health Survey 2010 (Direct)	0
	Demographic and Health Survey (MM adjusted) 2010 (Direct)	1
	National Panel Survey 2010-2011 (Household Deaths)	0
	Census 2012 (Indirect)	0
	National Panel Survey 2012-2013 (Household Deaths)	0
	Demographic and Health Survey 2015-2016 (Direct)	0
	Demographic and Health Survey (MM adjusted) 2015-2016 (Direct)	1
	Malaria Indicator Survey 2017 (Indirect)	1
United States of America	VR Submitted to WHO/UNIGME 2020 version 2020 (VR)	1
Uruguay	TABLAS DE VIDA NACIONALES (Life Table)	0
	Census 1975 (Indirect)	0
	Census 1985 (Indirect)	0
	Census 1996 (Indirect)	0
	Census 2010 (Indirect)	1
	UNPD Demographic Yearbook Data 2020 version 2020 (VR)	1
	VR Submitted to WHO/UNIGME 2020 version 2020 (VR)	1
Uzbekistan	Demographic and Health Survey 1996 (Direct)	0
	Demographic and Health Survey 1996 (Indirect)	0
	Multiple Indicator Cluster Survey 2000 (Indirect)	1
	Demographic and Health Survey 2002 (Direct)	1
	Demographic and Health Survey 2002 (Indirect)	0
	Multiple Indicator Cluster Survey 2006 (Indirect)	1
	Vital registration from National Statistical Office 2017 (VR)	0
	VR Submitted to WHO/UNIGME 2020 version 2020 (VR)	1
Vanuatu	Census 1967 (Indirect)	1
	Census 1989 (Indirect)	1
	Census 1999 (Indirect)	1
	Multiple Indicator Cluster Survey 2007-2008 (Indirect)	0
	Census 2009 (Household Deaths)	0
	Census 2009 (Indirect)	1
	Demographic and Health Survey 2013 (Direct)	1
Venezuela (Bolivarian Republic of)	TABLAS DE VIDA NACIONALES (Life Table)	1
	World Fertility Survey 1977 (Direct)	1
	World Fertility Survey 1977 (Indirect)	0
	Census 1981 (Indirect)	1
	Census 1990 (Indirect)	1
	National Population and Family Survey 1998 (Direct)	1
	Census 2001 (Indirect)	1
	Census 2011 (Indirect)	1
	Vital Registration Data from Ministerio del Poder Popular para la Planificacion y Desarrollo (May 2009) 2009 (VR)	0
	Vital Registration data from Instituto Nacional de Estadistica 2014 (VR)	0
	Vital Registration Data from Ministerio del Poder Popular para la Salud 2017 (VR)	0
	Provisional adjusted data from Ministerio del Poder Popular para la Salud and Instituto Nacional de Estadistica 2019 (VR)	0
	VR submitted to UN IGME in 2018 2019 (VR)	0
	UNPD Demographic Yearbook Data 2020 version 2020 (VR)	0
	VR Submitted to WHO/UNIGME 2020 version 2020 (VR)	1

	Data series	Inclusion
Viet Nam	Demographic and Health Survey 1988 (Direct)	1
	Demographic and Health Survey 1988 (Indirect)	0
	Census 1989 (Indirect)	1
	Intercensal Demographic Survey 1994 (Direct)	1
	Intercensal Demographic Survey 1994 (Indirect)	0
	Demographic and Health Survey 1997 (Direct)	1
	Demographic and Health Survey 1997 (Indirect)	0
	Vietnam Longitudinal Survey (VLSS) 1998 (Direct)	1
	Census 1999 (Household Deaths)	0
	Census 1999 (Indirect)	0
	Multiple Indicator Cluster Survey 2000 (Indirect)	1
	Demographic and Health Survey 2002 (Direct)	1
	Demographic and Health Survey 2002 (Indirect)	0
	Multiple Indicator Cluster Survey 2006 (Indirect)	0
	Population Change and Family Planning Survey 2006 (Indirect)	0
	Population Change and Family Planning Survey 2007 (Household Deaths)	0
	Population Change and Family Planning Survey 2007 (Indirect)	0
	Population Change and Family Planning Survey 2008 (Indirect)	0
	Census 2009 (Indirect)	0
	Population Change and Family Planning Survey 2010 (Indirect)	0
	Multiple Indicator Cluster Survey 2010-2011 (Indirect)	0
	Population Change and Family Planning Survey 2011 (Household Deaths)	1
	Population Change and Family Planning Survey 2011 (Indirect)	0
	Population Change and Family Planning Survey 2012 (Household Deaths)	1
	Population Change and Family Planning Survey 2012 (Indirect)	0
	Population Change and Family Planning Survey 2013 (Household Deaths)	1
	Population Change and Family Planning Survey 2013 (Indirect)	0
	Multiple Indicator Cluster Survey 2013-2014 (Direct)	1
	Intercensal Population and Housing Survey 2014 (Household Deaths)	1
	Population Change and Family Planning Survey 2015 (Household Deaths)	1
	Population Change and Family Planning Survey 2015 (Indirect)	0
	Population Change and Family Planning Survey 2016 (Household Deaths)	1
	Population Change and Family Planning Survey 2016 (Indirect)	0
Yemen	WFS-Household survey 1979 (Indirect)	0
	WFS-Individual survey 1979 (Indirect)	0
	World Fertility Survey 1979 (Direct)	1
	Demographic and Health Survey 1991-1992 (Direct)	1
	Demographic and Health Survey 1991-1992 (Indirect)	0
	Census 1994 (Indirect)	1
	Demographic and Health Survey 1997 (Direct)	1
	Demographic and Health Survey 1997 (Indirect)	0
	PAPFAM Family Health Survey 2003 (Direct)	1
	Census 2004 (Household Deaths)	1
	Census 2004 (Indirect)	1
	Multiple Indicator Cluster Survey 2006 (Direct)	1
	Multiple Indicator Cluster Survey 2006 (Indirect)	0
	Demographic and Health Survey 2013 (Direct)	1
Zambia	Census 1969 (Indirect)	1
	Sample Census of Population 1974 (Indirect)	1
	Census 1980 (Indirect)	1
	Census 1990 (Indirect)	1
	Demographic and Health Survey 1992 (Direct)	0
	Demographic and Health Survey 1992 (Indirect)	0
	Demographic and Health Survey (MM adjusted) 1992 (Direct)	1

	Data series	Inclusion
	Demographic and Health Survey 1996-1997 (Direct)	0
	Demographic and Health Survey 1996-1997 (Indirect)	0
	Demographic and Health Survey (MM adjusted) 1996-1997 (Direct)	1
	Census 2000 (Indirect)	1
	Demographic and Health Survey 2001-2002 (Direct)	0
	Demographic and Health Survey 2001-2002 (Indirect)	0
	Demographic and Health Survey (MM adjusted) 2001-2002 (Direct)	1
	Demographic and Health Survey 2007 (Household Deaths)	0
	Demographic and Health Survey 2007 (Direct)	0
	Demographic and Health Survey (MM adjusted) 2007 (Direct)	1
	Demographic Sample Survey 2007 (Indirect)	1
	Global Fund Evaluation Survey 2008 (Household Deaths)	0
	Census 2010 (Indirect)	1
	Census 2010 (Household Deaths)	0
	Demographic and Health Survey 2013-2014 (Direct)	0
	Demographic and Health Survey (MM adjusted) 2013-2014 (Direct)	1
	Demographic and Health Survey 2018 (Direct)	0
	Demographic and Health Survey (MM adjusted) 2018 (Direct)	1
Zimbabwe	Census 1969 (Indirect)	1
	Census 1982 (Indirect)	1
	Reproductive Health Survey 1984 (Indirect)	1
	Inter-censal Demographic Survey 1987 (Indirect)	1
	Demographic and Health Survey 1988-1989 (Direct)	0
	Demographic and Health Survey 1988-1989 (Indirect)	0
	Demographic and Health Survey (MM adjusted) 1988-1989 (Direct)	1
	Census 1992 (Household Deaths)	0
	Census 1992 (Indirect)	1
	Demographic and Health Survey 1994 (Direct)	0
	Demographic and Health Survey 1994 (Indirect)	0
	Demographic and Health Survey (MM adjusted) 1994 (Direct)	1
	Inter-censal Demographic Survey 1997 (Household Deaths)	0
	Inter-censal Demographic Survey 1997 (Indirect)	1
	Demographic and Health Survey 1999 (Direct)	0
	Demographic and Health Survey 1999 (Indirect)	0
	Demographic and Health Survey (MM adjusted) 1999 (Direct)	1
	Census 2002 (Household Deaths)	0
	Census 2002 (Indirect)	1
	Demographic and Health Survey 2005-2006 (Household Deaths)	0
	Demographic and Health Survey 2005-2006 (Direct)	0
	Demographic and Health Survey 2005-2006 (Indirect)	0
	Demographic and Health Survey (MM adjusted) 2005-2006 (Direct)	0
	Multiple Indicator Cluster Survey 2009 (Direct)	0
	Multiple Indicator Cluster Survey (MM adjusted) 2009 (Direct)	1
	Demographic and Health Survey 2010-2011 (Direct)	0
	Demographic and Health Survey (MM adjusted) 2010-2011 (Direct)	1
	Census 2012 (Household Deaths)	0
	Multiple Indicator Cluster Survey 2014 (Direct)	0
	Multiple Indicator Cluster Survey (MM adjusted) 2014 (Direct)	1
	Demographic and Health Survey 2015 (Direct)	0
	Demographic and Health Survey (MM adjusted) 2015 (Direct)	1
	Multiple Indicator Cluster Survey 2019 (Direct)	0
	Multiple Indicator Cluster Survey (MM adjusted) 2019 (Direct)	1

4. Supplementary figures

4.1. Figure A.1 Trends in U5MR, NMR, and number of under-5 deaths, 1990-2019, with 90% uncertainty intervals, by region

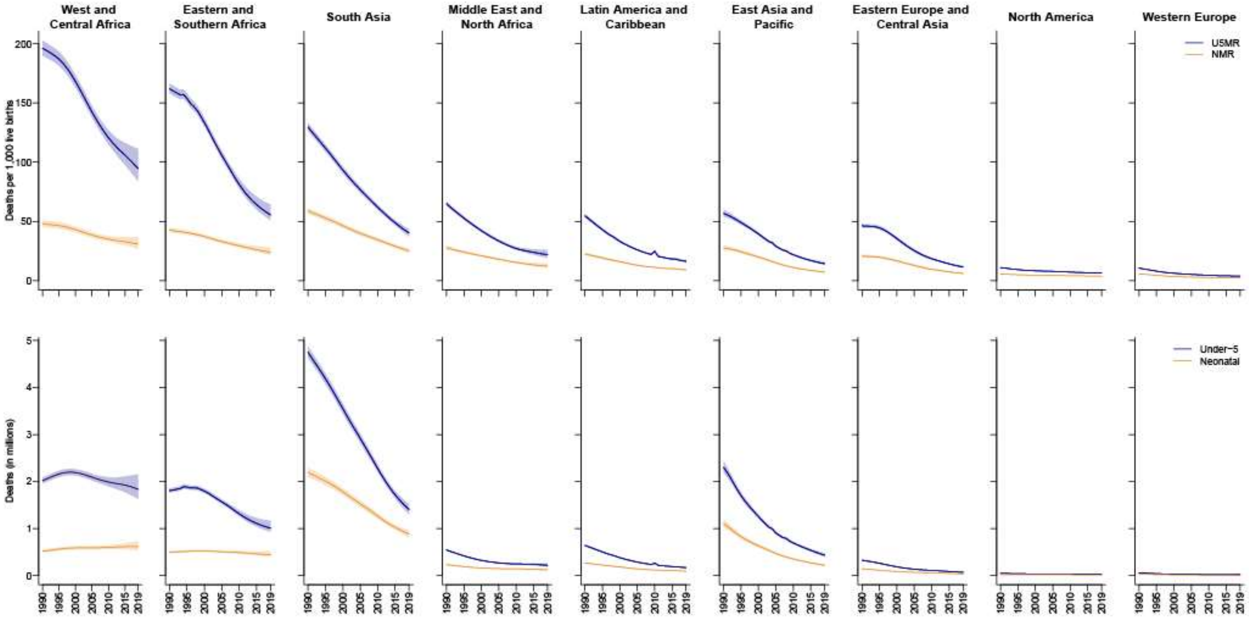

4.2. Figure A.2 Annual rate of reduction 2010-2019 and required annual rate of reduction to achieve SDG target

Under-five mortality rate (SDG 3.2.1)

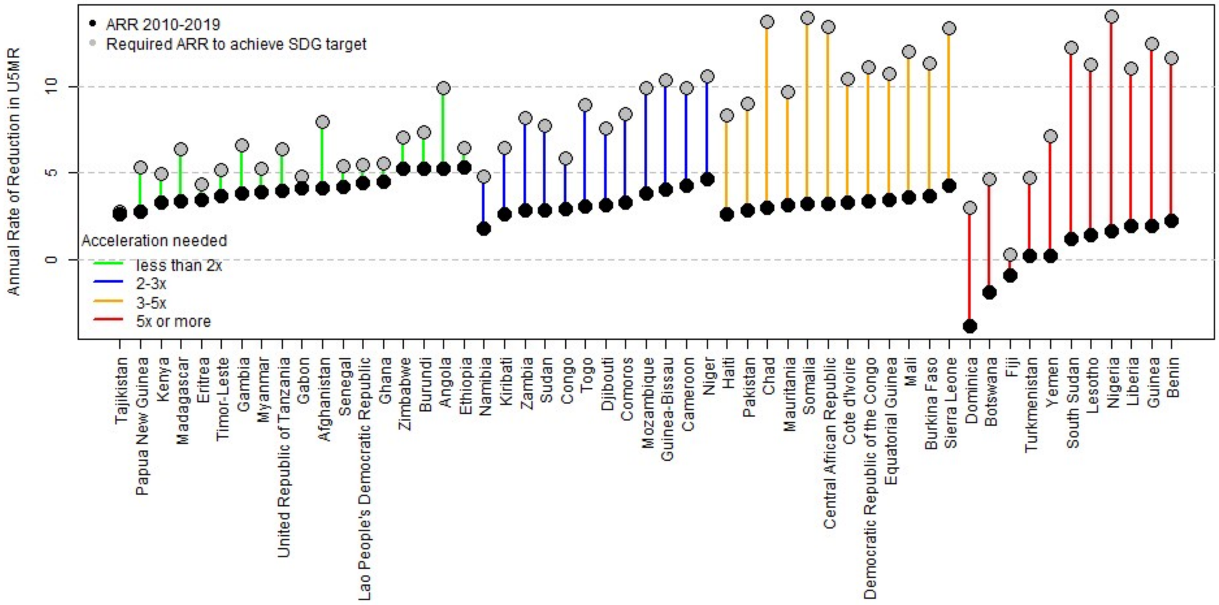

Neonatal mortality rate (SDG 3.2.2)

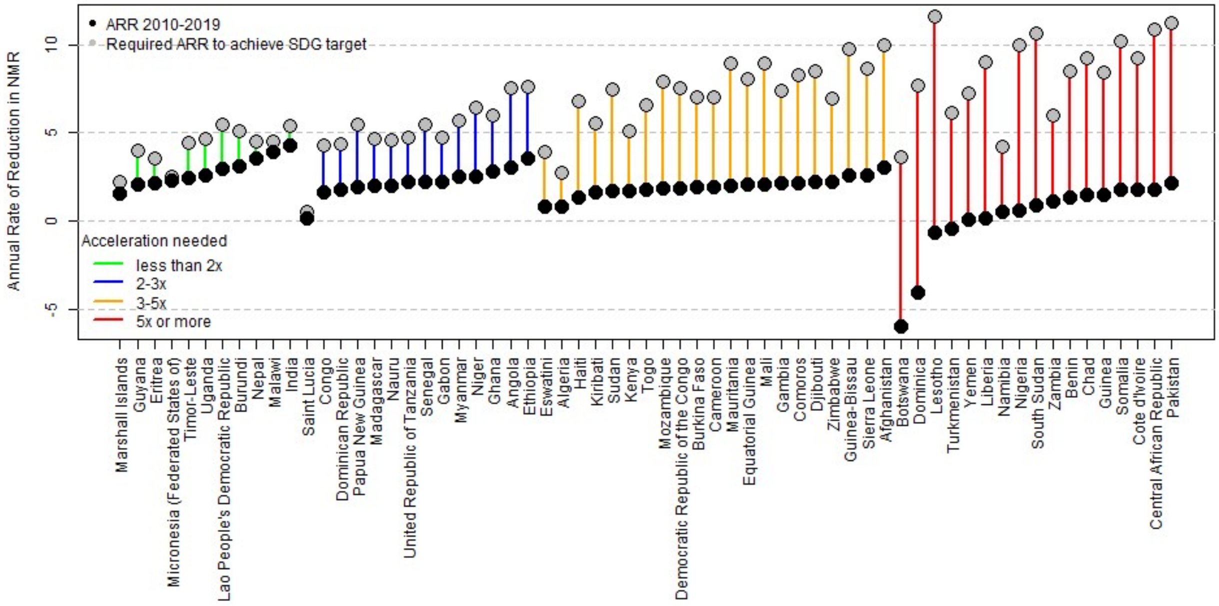

4.3. Figure A.3 Projected global under-5 mortality rate and the number of under-5 deaths 2020-2030

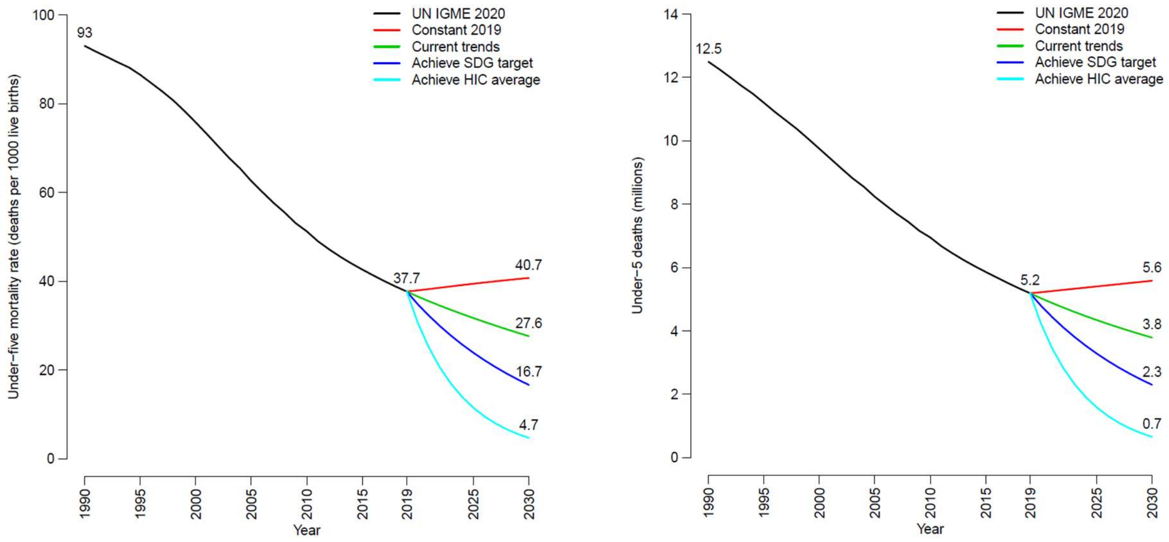

4.4. Figure A.4 Under-five mortality estimates with underlying data, by country

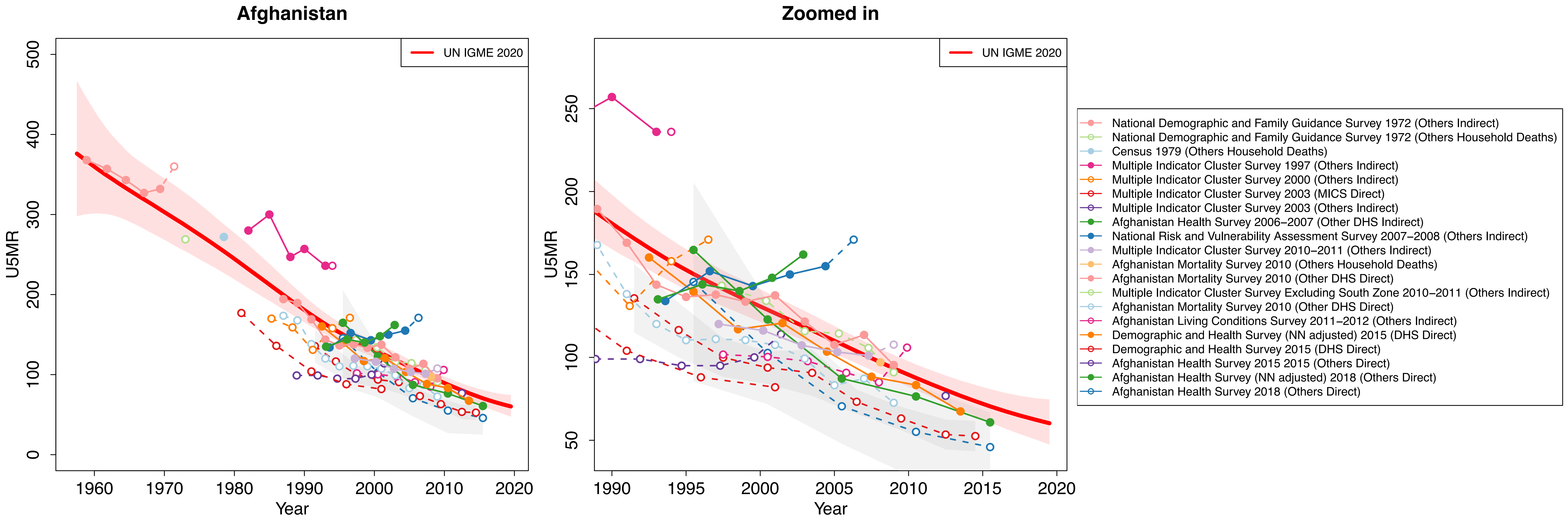

Afghanistan
Zoomed in
UN IGME 2020
U5MR
Year
National Demographic and Family Guidance Survey 1972 (Others Indirect)
National Demographic and Family Guidance Survey 1972 (Others Household Deaths)
Census 1979 (Others Household Deaths)
Multiple Indicator Cluster Survey 1997 (Others Indirect)
Multiple Indicator Cluster Survey 2000 (Others Indirect)
Multiple Indicator Cluster Survey 2003 (MICS Direct)
Multiple Indicator Cluster Survey 2003 (Others Indirect)
Afghanistan Health Survey 2006–2007 (Other DHS Indirect)
National Risk and Vulnerability Assessment Survey 2007–2008 (Others Indirect)
Multiple Indicator Cluster Survey 2010–2011 (Others Indirect)
Afghanistan Mortality Survey 2010 (Others Household Deaths)
Afghanistan Mortality Survey 2010 (Other DHS Direct)
Multiple Indicator Cluster Survey Excluding South Zone 2010–2011 (Others Indirect)
Afghanistan Mortality Survey 2010 (Other DHS Direct)
Afghanistan Living Conditions Survey 2011–2012 (Others Indirect)
Demographic and Health Survey (NN adjusted) 2015 (DHS Direct)
Demographic and Health Survey 2015 (DHS Direct)
Afghanistan Health Survey 2015 2015 (Others Direct)
Afghanistan Health Survey (NN adjusted) 2018 (Others Direct)
Afghanistan Health Survey 2018 (Others Direct)

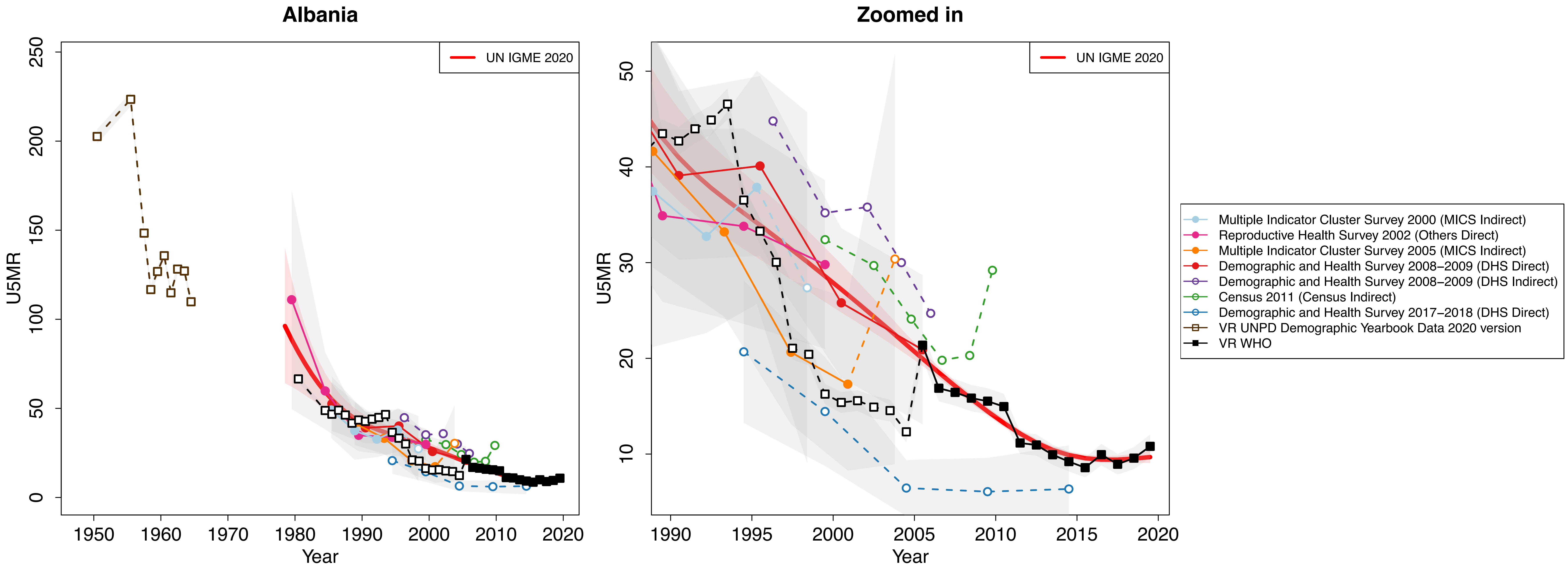

Albania
Zoomed in
UN IGME 2020
U5MR
Year
Multiple Indicator Cluster Survey 2000 (MICS Indirect)
Reproductive Health Survey 2002 (Others Direct)
Multiple Indicator Cluster Survey 2005 (MICS Indirect)
Demographic and Health Survey 2008–2009 (DHS Direct)
Demographic and Health Survey 2008–2009 (DHS Indirect)
Census 2011 (Census Indirect)
Demographic and Health Survey 2017–2018 (DHS Direct)
VR UNPD Demographic Yearbook Data 2020 version
VR WHO

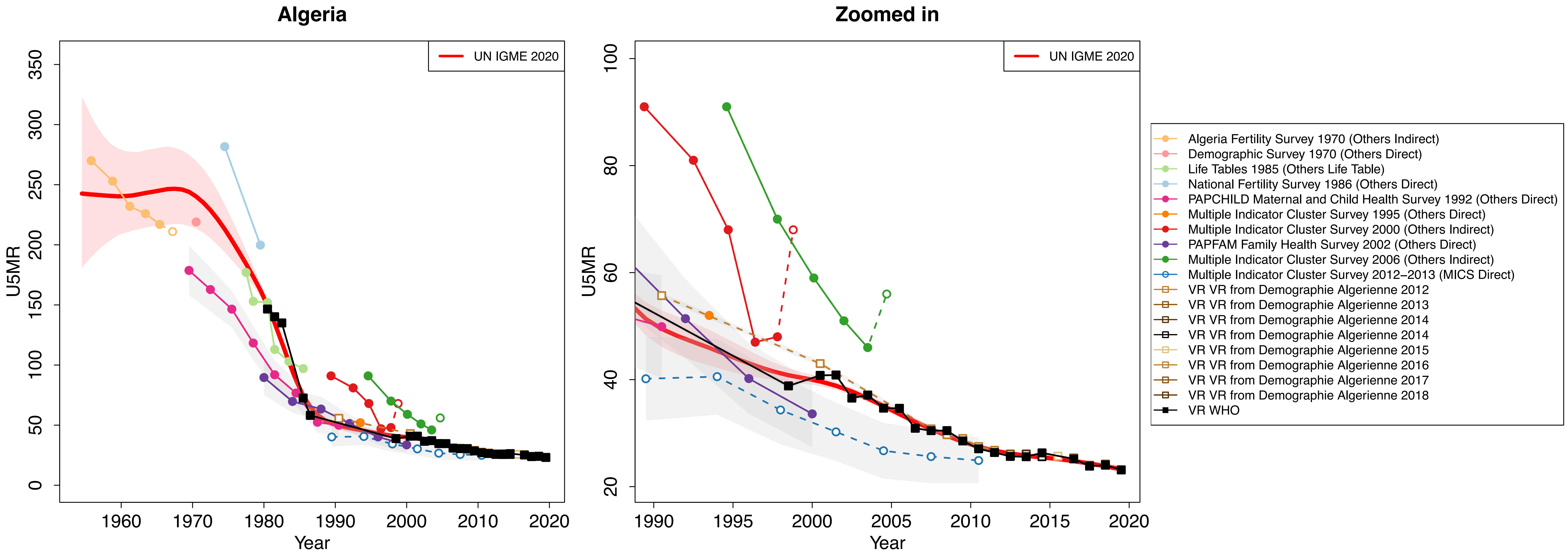

Algeria
Zoomed in
UN IGME 2020
U5MR
Year
Algeria Fertility Survey 1970 (Others Indirect)
Demographic Survey 1970 (Others Direct)
Life Tables 1985 (Others Life Table)
National Fertility Survey 1986 (Others Direct)
PAPCHILD Maternal and Child Health Survey 1992 (Others Direct)
Multiple Indicator Cluster Survey 1995 (Others Direct)
Multiple Indicator Cluster Survey 2000 (Others Indirect)
PAPFAM Family Health Survey 2002 (Others Direct)
Multiple Indicator Cluster Survey 2006 (Others Indirect)
Multiple Indicator Cluster Survey 2012–2013 (MICS Direct)
VR VR from Demographie Algerienne 2012
VR VR from Demographie Algerienne 2013
VR VR from Demographie Algerienne 2014
VR VR from Demographie Algerienne 2014
VR VR from Demographie Algerienne 2015
VR VR from Demographie Algerienne 2016
VR VR from Demographie Algerienne 2017
VR VR from Demographie Algerienne 2018
VR WHO

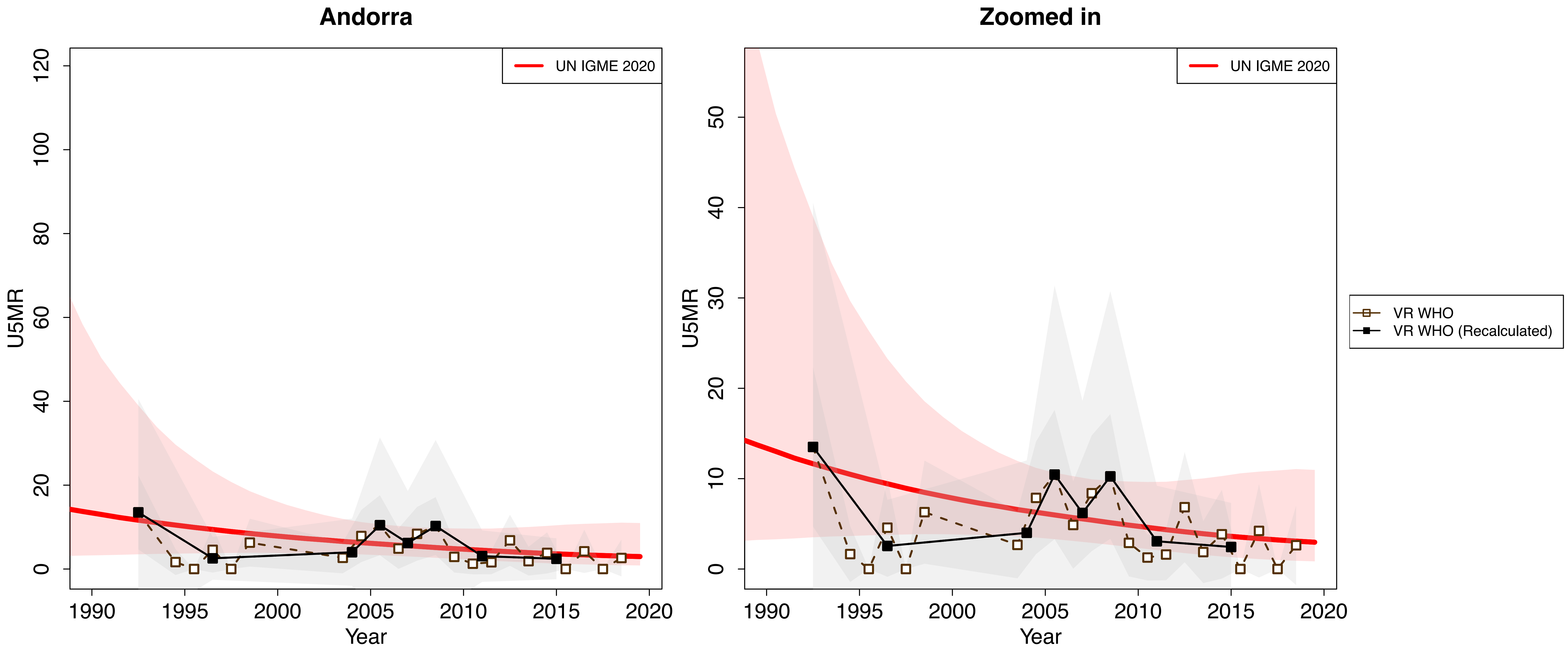

Andorra
Zoomed in
UN IGME 2020
UN IGME 2020
VR WHO
VR WHO (Recalculated)
U5MR
U5MR
Year
Year
0
20
40
60
80
100
120
0
10
20
30
40
50
1990
1995
2000
2005
2010
2015
2020

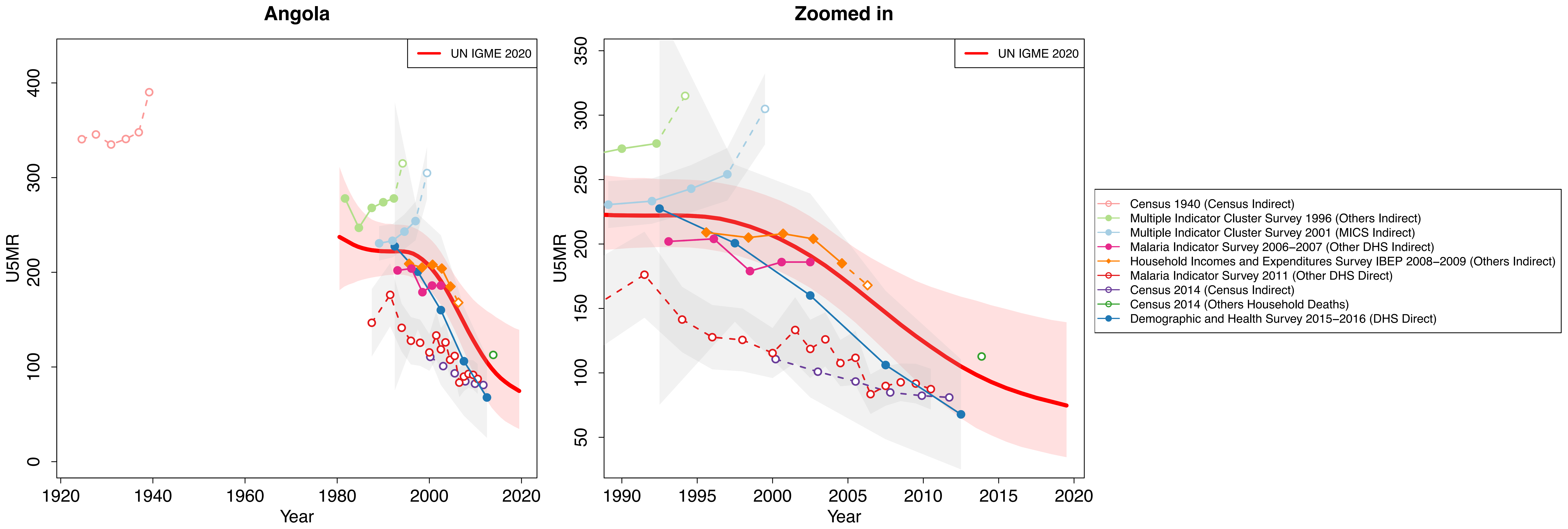

Angola
Zoomed in
UN IGME 2020
U5MR
Year
Census 1940 (Census Indirect)
Multiple Indicator Cluster Survey 1996 (Others Indirect)
Multiple Indicator Cluster Survey 2001 (MICS Indirect)
Malaria Indicator Survey 2006–2007 (Other DHS Indirect)
Household Incomes and Expenditures Survey IBEP 2008–2009 (Others Indirect)
Malaria Indicator Survey 2011 (Other DHS Direct)
Census 2014 (Census Indirect)
Census 2014 (Others Household Deaths)
Demographic and Health Survey 2015–2016 (DHS Direct)

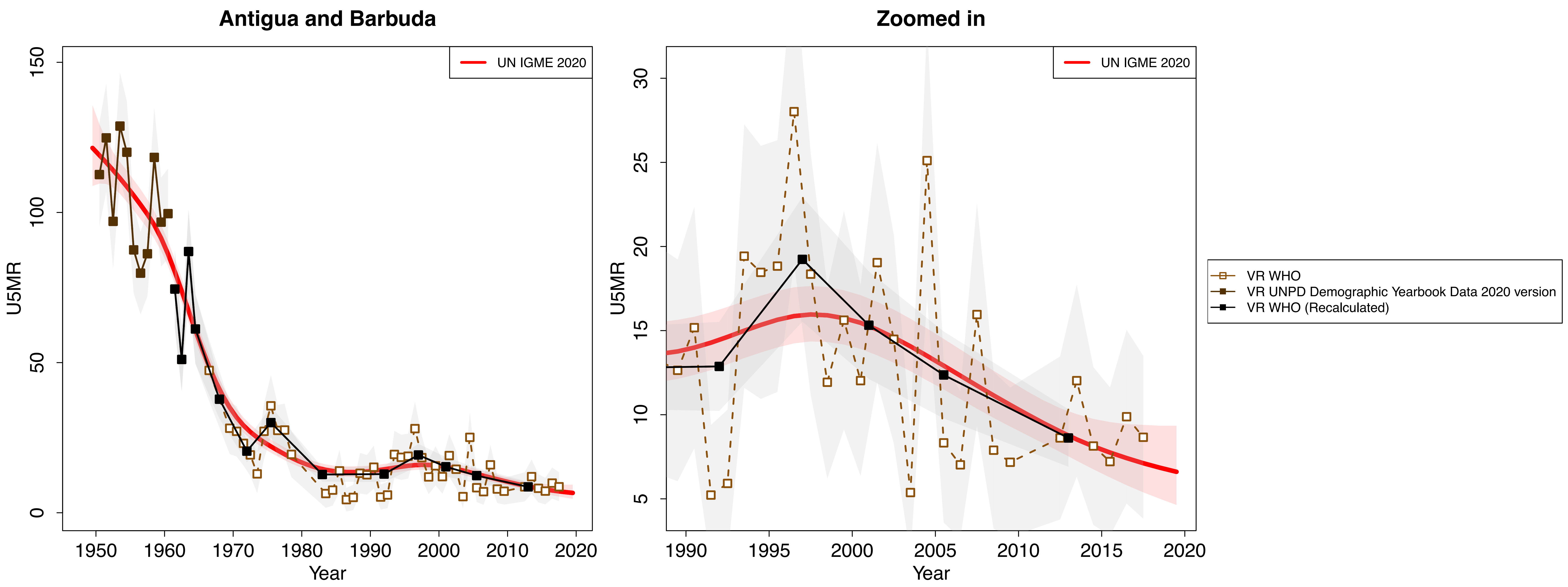

Antigua and Barbuda
Zoomed in
UN IGME 2020
U5MR
Year
VR WHO
VR UNPD Demographic Yearbook Data 2020 version
VR WHO (Recalculated)

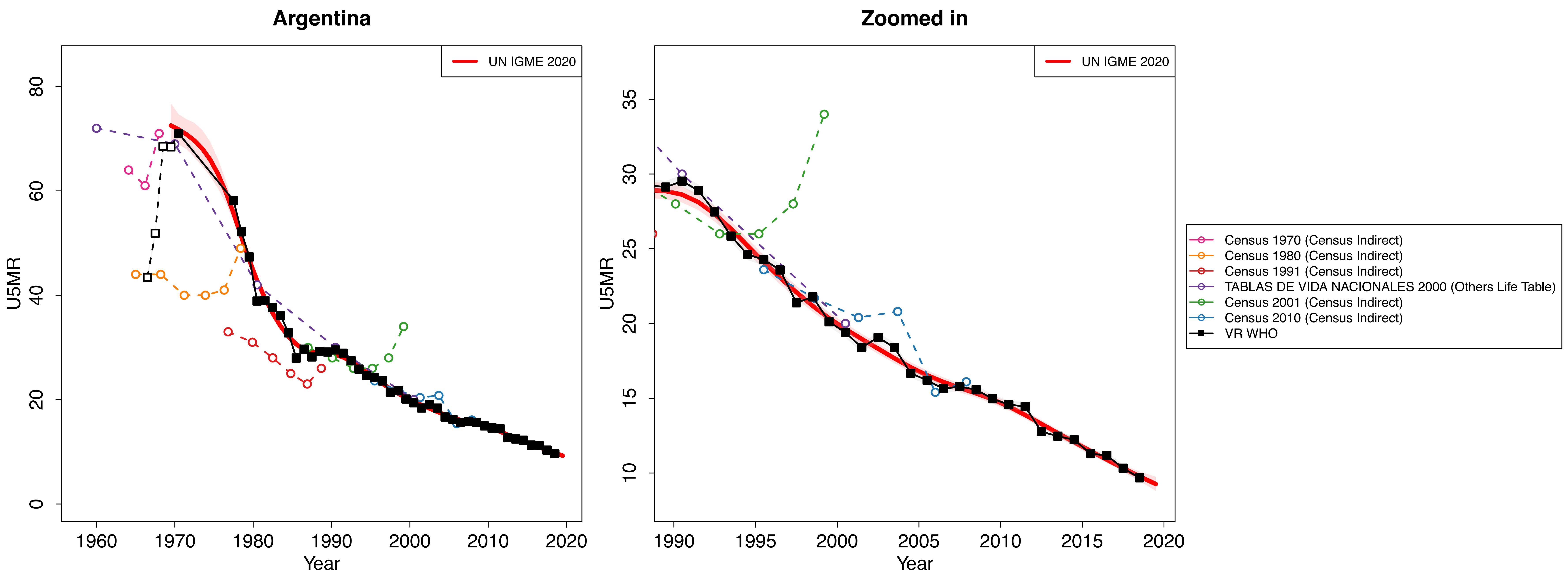
Argentina
Zoomed in
UN IGME 2020
U5MR
Year
Census 1970 (Census Indirect)
Census 1980 (Census Indirect)
Census 1991 (Census Indirect)
TABLAS DE VIDA NACIONALES 2000 (Others Life Table)
Census 2001 (Census Indirect)
Census 2010 (Census Indirect)
VR WHO

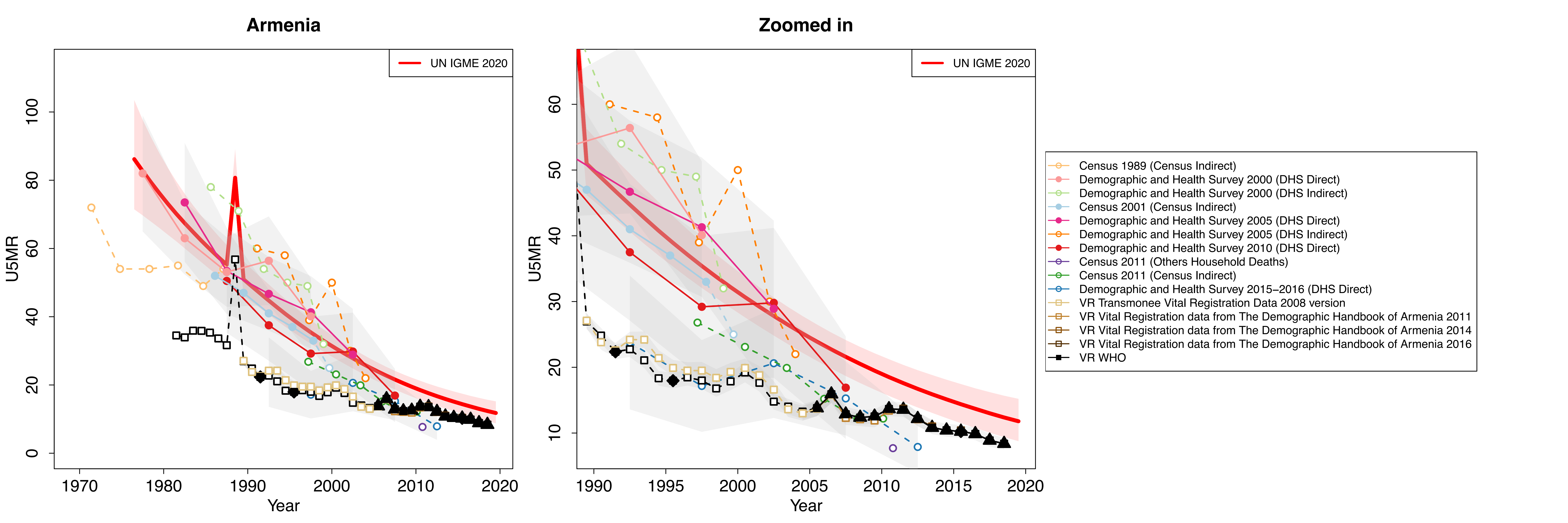

Armenia
Zoomed in
UN IGME 2020
U5MR
Year
Census 1989 (Census Indirect)
Demographic and Health Survey 2000 (DHS Direct)
Demographic and Health Survey 2000 (DHS Indirect)
Census 2001 (Census Indirect)
Demographic and Health Survey 2005 (DHS Direct)
Demographic and Health Survey 2005 (DHS Indirect)
Demographic and Health Survey 2010 (DHS Direct)
Census 2011 (Others Household Deaths)
Census 2011 (Census Indirect)
Demographic and Health Survey 2015–2016 (DHS Direct)
VR Transmonee Vital Registration Data 2008 version
VR Vital Registration data from The Demographic Handbook of Armenia 2011
VR Vital Registration data from The Demographic Handbook of Armenia 2014
VR Vital Registration data from The Demographic Handbook of Armenia 2016
VR WHO

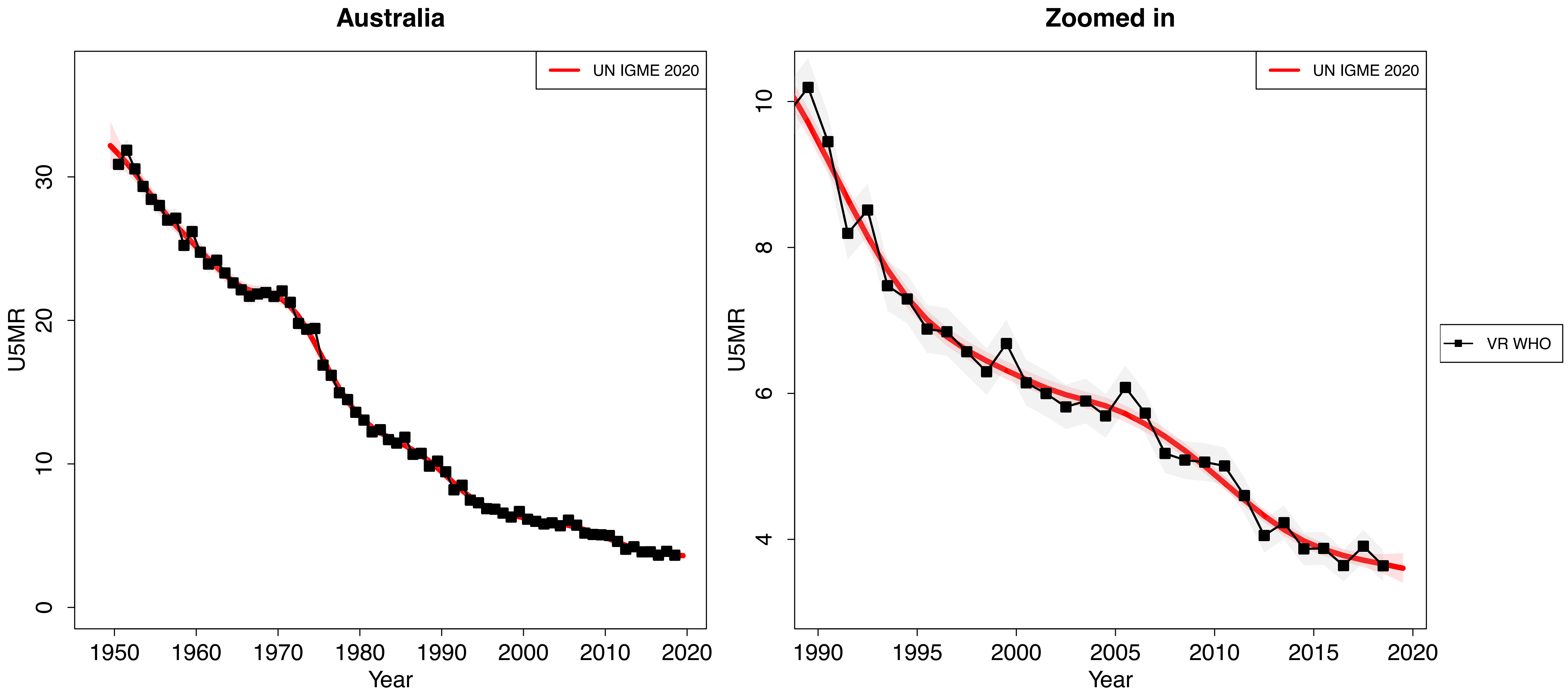

Australia
Zoomed in
UN IGME 2020
UN IGME 2020
VR WHO
U5MR
U5MR
Year
Year
0
10
20
30
4
6
8
10
1950
1960
1970
1980
1990
2000
2010
2020
1990
1995
2000
2005
2010
2015
2020

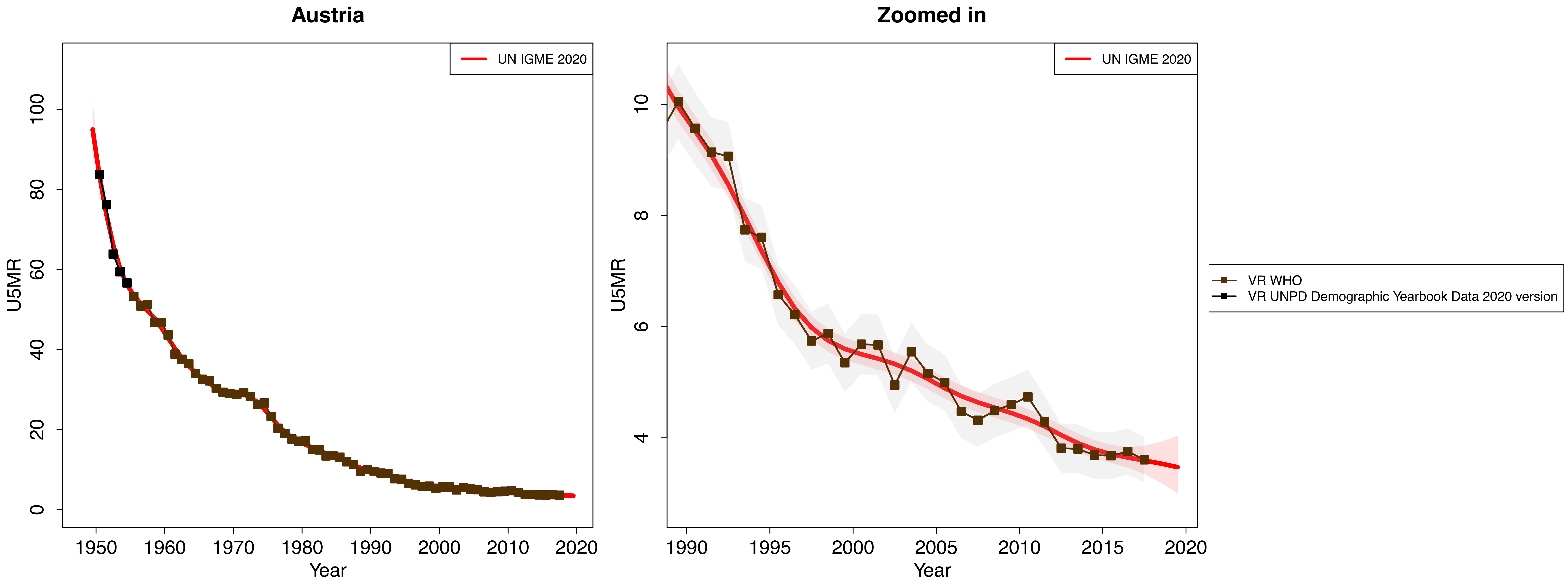
Austria
Zoomed in
UN IGME 2020
UN IGME 2020
U5MR
U5MR
Year
Year
0
20
40
60
80
100
4
6
8
10
1950
1960
1970
1980
1990
2000
2010
2020
1990
1995
2000
2005
2010
2015
2020
VR WHO
VR UNPD Demographic Yearbook Data 2020 version

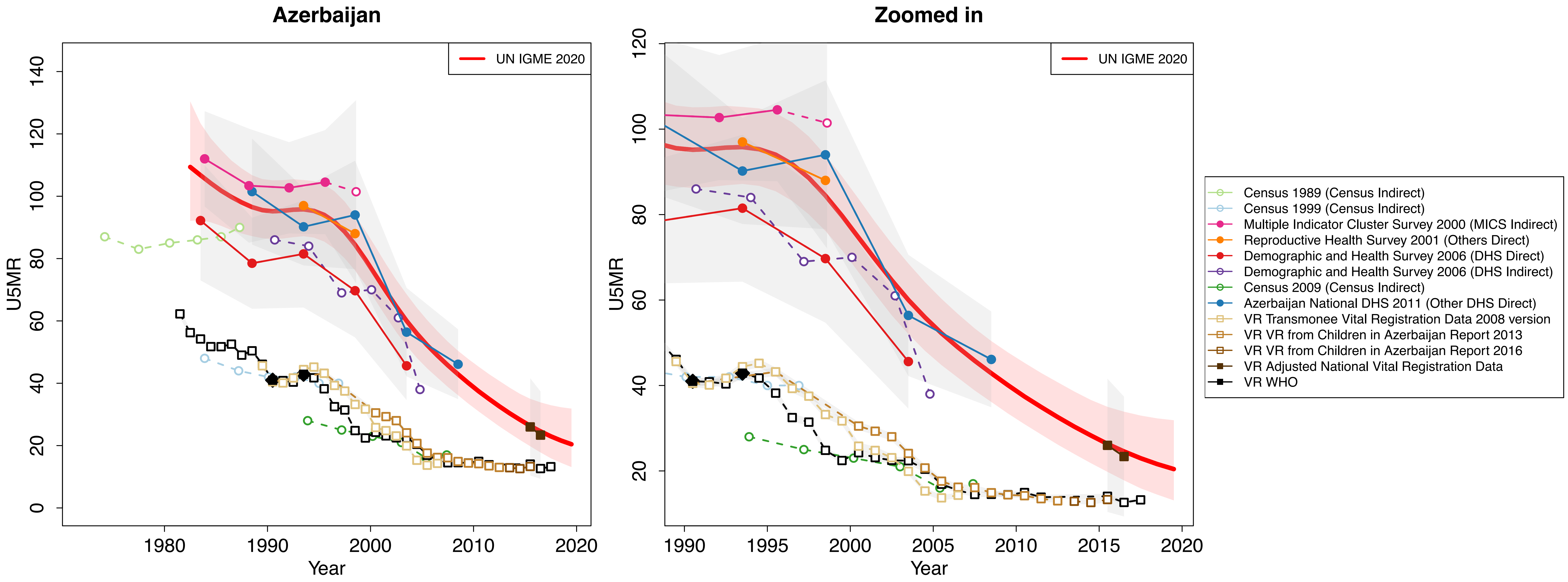

Azerbaijan
Zoomed in
UN IGME 2020
U5MR
Year
Census 1989 (Census Indirect)
Census 1999 (Census Indirect)
Multiple Indicator Cluster Survey 2000 (MICS Indirect)
Reproductive Health Survey 2001 (Others Direct)
Demographic and Health Survey 2006 (DHS Direct)
Demographic and Health Survey 2006 (DHS Indirect)
Census 2009 (Census Indirect)
Azerbaijan National DHS 2011 (Other DHS Direct)
VR Transmonee Vital Registration Data 2008 version
VR VR from Children in Azerbaijan Report 2013
VR VR from Children in Azerbaijan Report 2016
VR Adjusted National Vital Registration Data
VR WHO

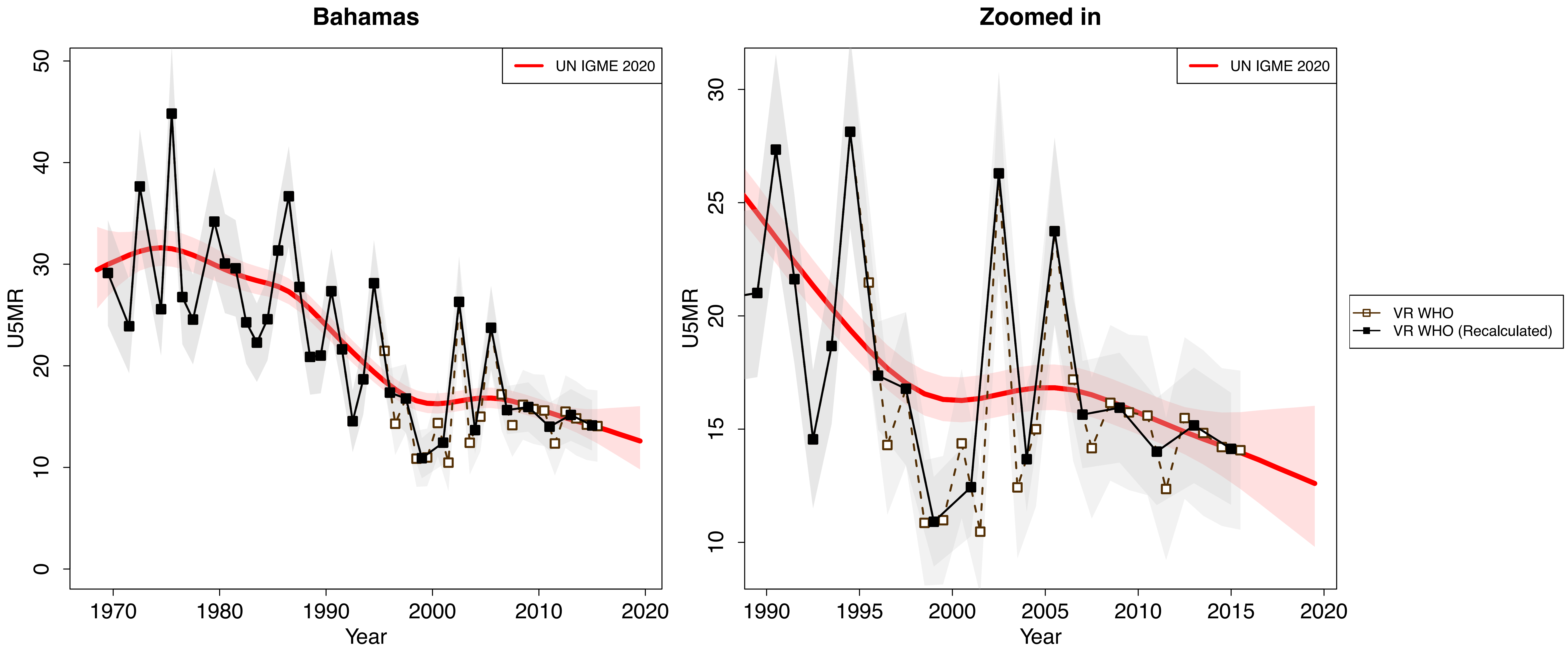

Bahamas
Zoomed in
UN IGME 2020
U5MR
Year
VR WHO
VR WHO (Recalculated)

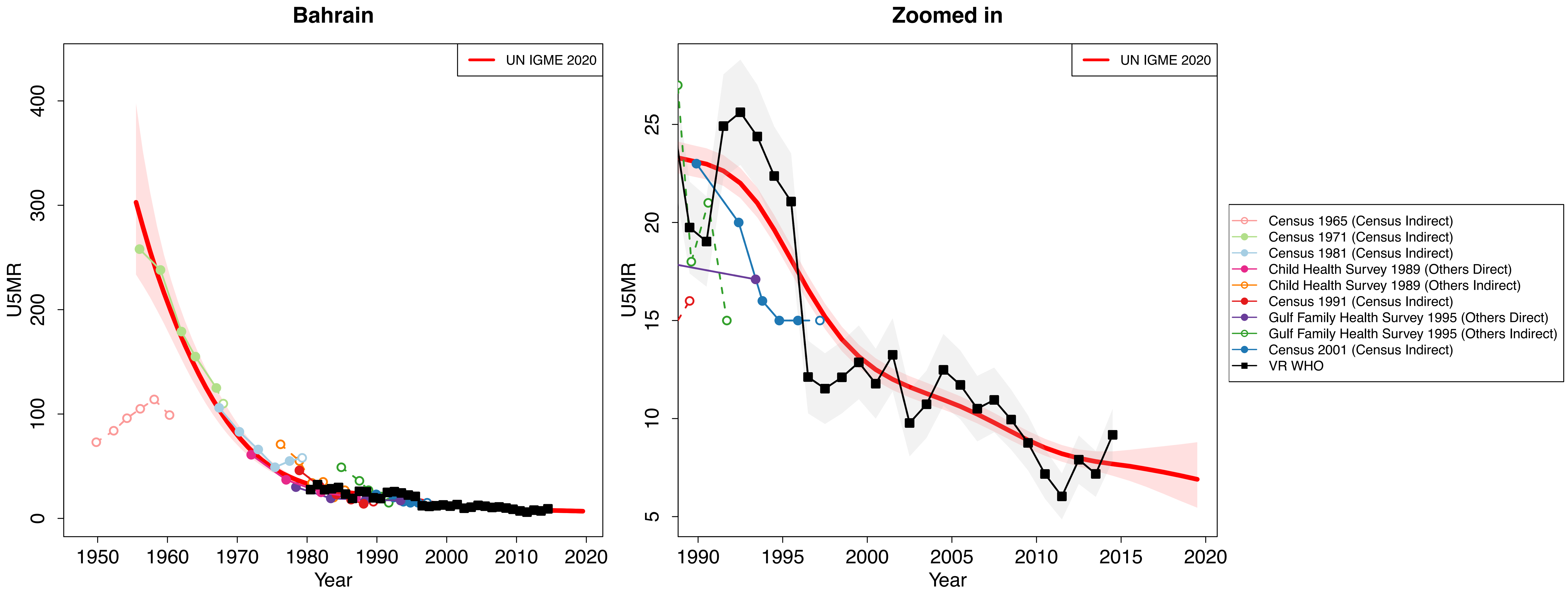

Bahrain
UN IGME 2020
U5MR
Year
Zoomed in
Census 1965 (Census Indirect)
Census 1971 (Census Indirect)
Census 1981 (Census Indirect)
Child Health Survey 1989 (Others Direct)
Child Health Survey 1989 (Others Indirect)
Census 1991 (Census Indirect)
Gulf Family Health Survey 1995 (Others Direct)
Gulf Family Health Survey 1995 (Others Indirect)
Census 2001 (Census Indirect)
VR WHO

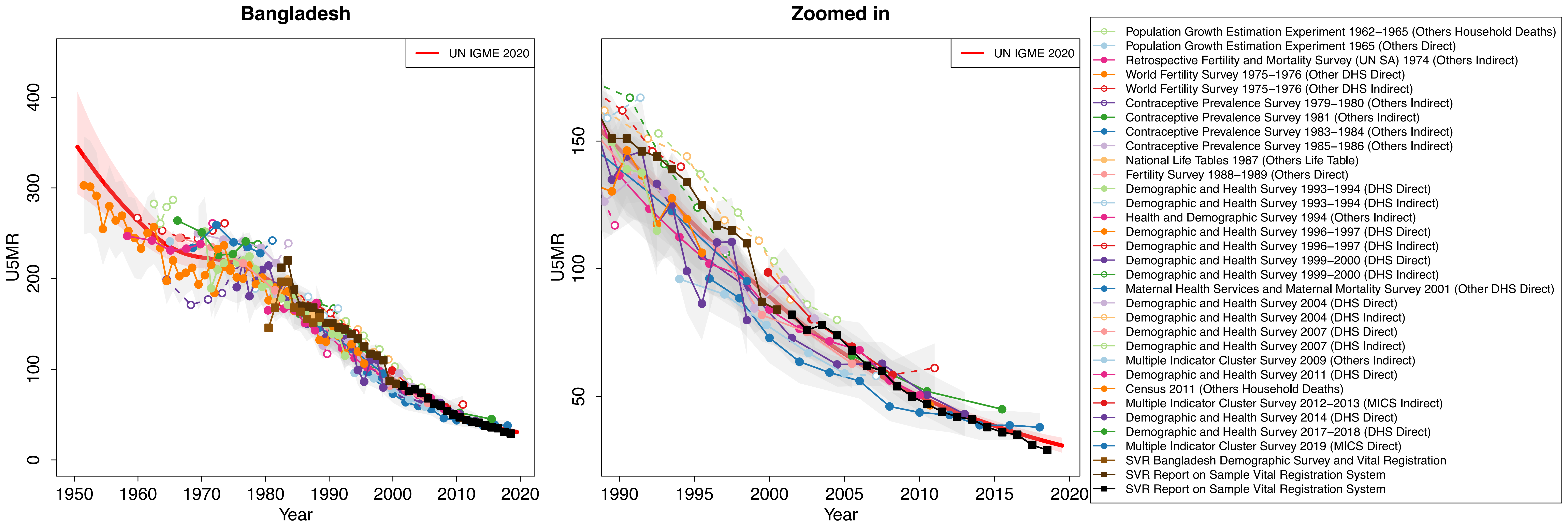

Bangladesh
Zoomed in
UN IGME 2020
U5MR
Year
Population Growth Estimation Experiment 1962–1965 (Others Household Deaths)
Population Growth Estimation Experiment 1965 (Others Direct)
Retrospective Fertility and Mortality Survey (UN SA) 1974 (Others Indirect)
World Fertility Survey 1975–1976 (Other DHS Direct)
World Fertility Survey 1975–1976 (Other DHS Indirect)
Contraceptive Prevalence Survey 1979–1980 (Others Indirect)
Contraceptive Prevalence Survey 1981 (Others Indirect)
Contraceptive Prevalence Survey 1983–1984 (Others Indirect)
Contraceptive Prevalence Survey 1985–1986 (Others Indirect)
National Life Tables 1987 (Others Life Table)
Fertility Survey 1988–1989 (Others Direct)
Demographic and Health Survey 1993–1994 (DHS Direct)
Demographic and Health Survey 1993–1994 (DHS Indirect)
Health and Demographic Survey 1994 (Others Indirect)
Demographic and Health Survey 1996–1997 (DHS Direct)
Demographic and Health Survey 1996–1997 (DHS Indirect)
Demographic and Health Survey 1999–2000 (DHS Direct)
Demographic and Health Survey 1999–2000 (DHS Indirect)
Maternal Health Services and Maternal Mortality Survey 2001 (Other DHS Direct)
Demographic and Health Survey 2004 (DHS Direct)
Demographic and Health Survey 2004 (DHS Indirect)
Demographic and Health Survey 2007 (DHS Direct)
Demographic and Health Survey 2007 (DHS Indirect)
Multiple Indicator Cluster Survey 2009 (Others Indirect)
Demographic and Health Survey 2011 (DHS Direct)
Census 2011 (Others Household Deaths)
Multiple Indicator Cluster Survey 2012–2013 (MICS Indirect)
Demographic and Health Survey 2014 (DHS Direct)
Demographic and Health Survey 2017–2018 (DHS Direct)
Multiple Indicator Cluster Survey 2019 (MICS Direct)
SVR Bangladesh Demographic Survey and Vital Registration
SVR Report on Sample Vital Registration System
SVR Report on Sample Vital Registration System

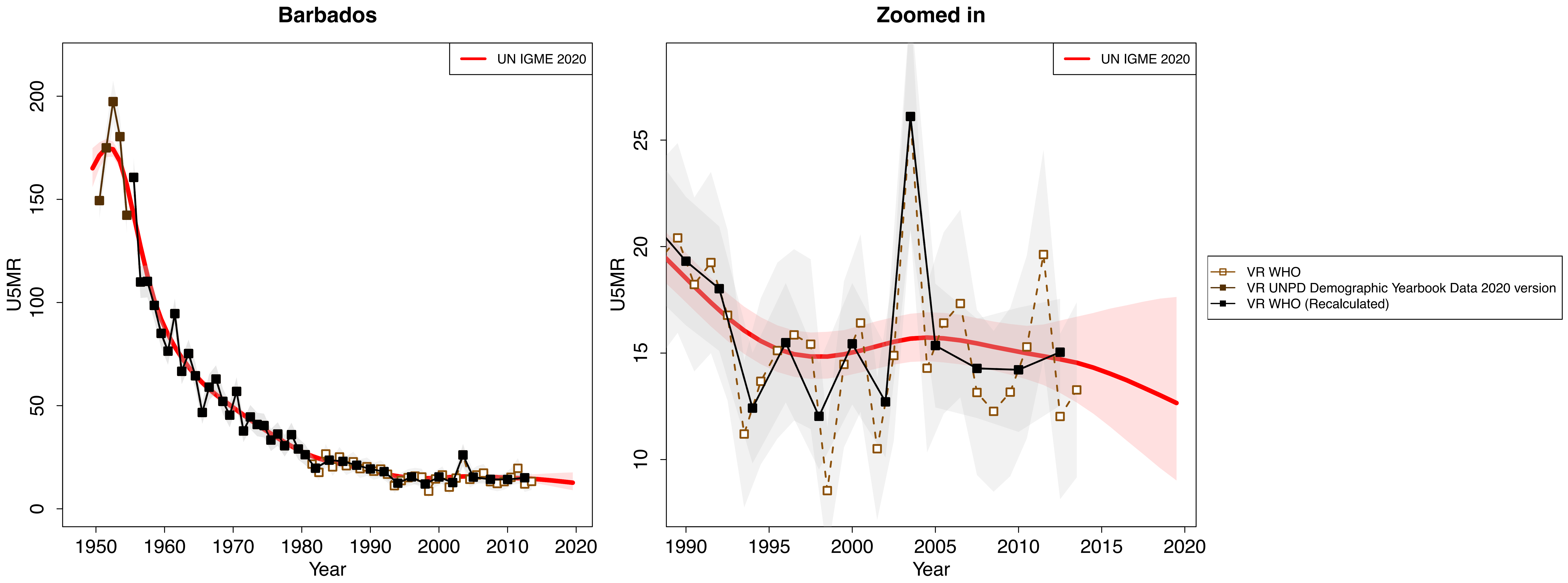
Barbados
Zoomed in
UN IGME 2020
U5MR
Year
VR WHO
VR UNPD Demographic Yearbook Data 2020 version
VR WHO (Recalculated)

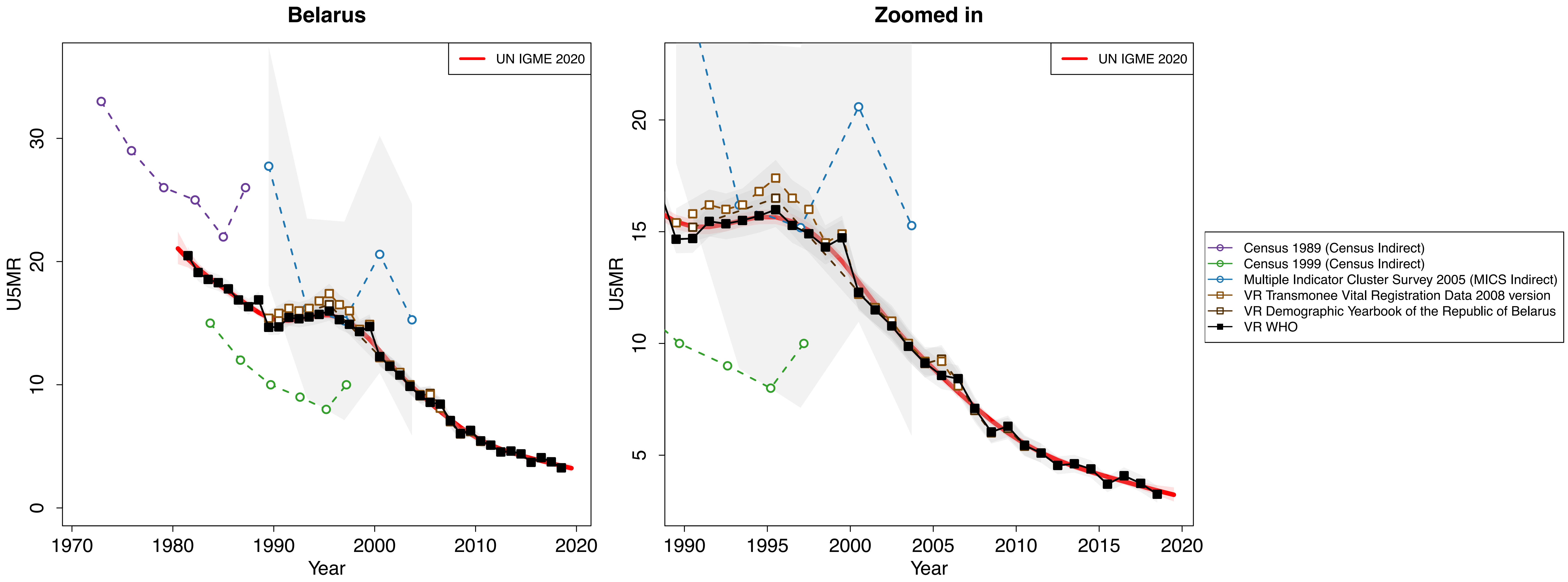

Belarus
Zoomed in
UN IGME 2020
U5MR
Year
Census 1989 (Census Indirect)
Census 1999 (Census Indirect)
Multiple Indicator Cluster Survey 2005 (MICS Indirect)
VR Transmonee Vital Registration Data 2008 version
VR Demographic Yearbook of the Republic of Belarus
VR WHO

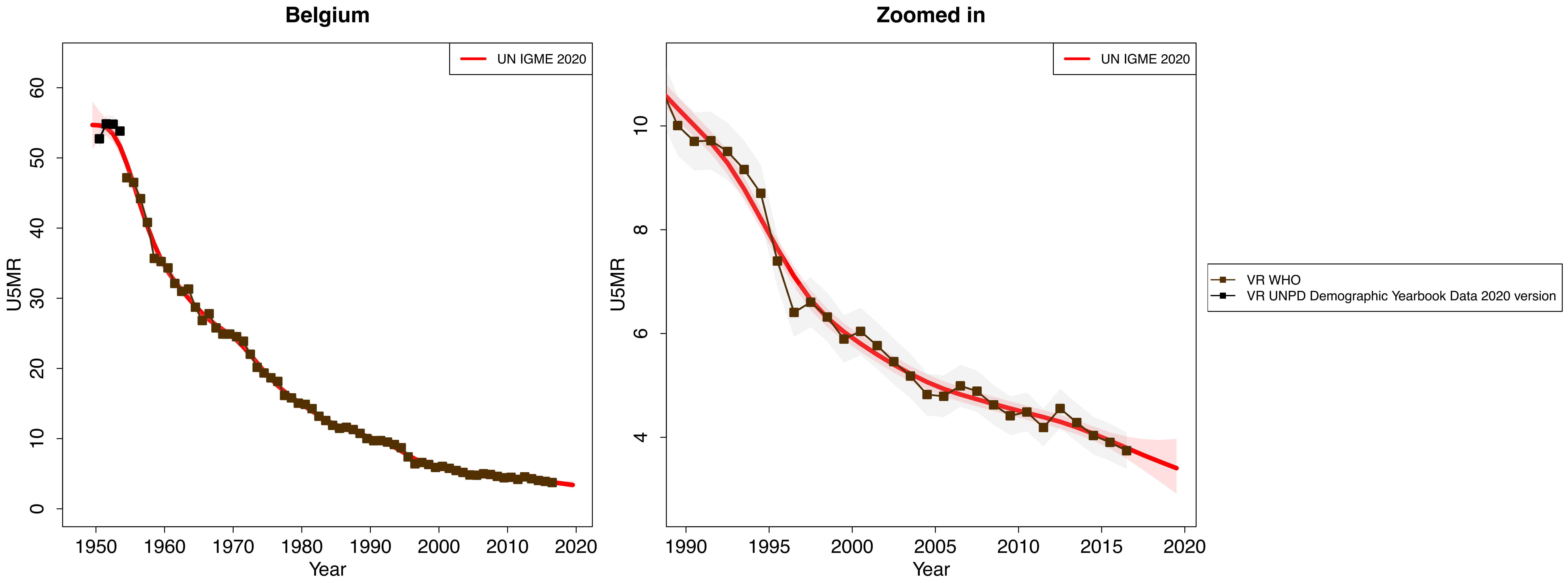
Belgium
Zoomed in
UN IGME 2020
UN IGME 2020
U5MR
Year
VR WHO
VR UNPD Demographic Yearbook Data 2020 version
1950
1960
1970
1980
1990
2000
2010
2020
1990
1995
2000
2005
2010
2015
2020
0
10
20
30
40
50
60
4
6
8
10

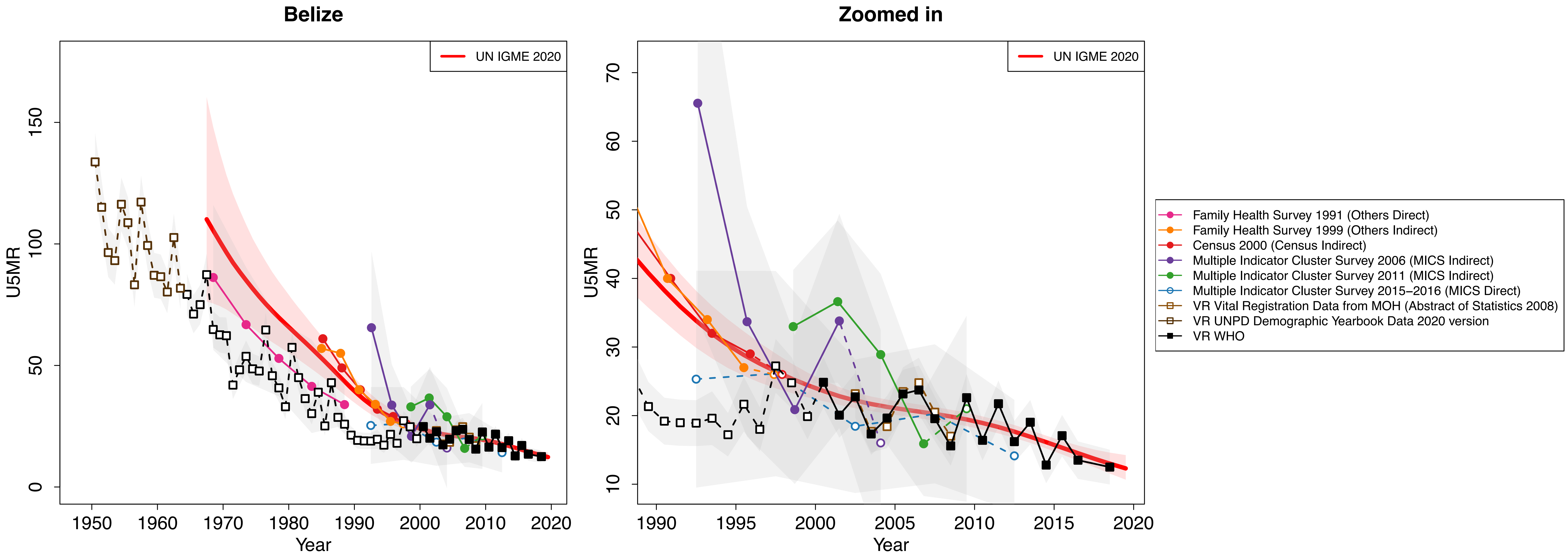
Belize
Zoomed in
UN IGME 2020
U5MR
Year
Family Health Survey 1991 (Others Direct)
Family Health Survey 1999 (Others Indirect)
Census 2000 (Census Indirect)
Multiple Indicator Cluster Survey 2006 (MICS Indirect)
Multiple Indicator Cluster Survey 2011 (MICS Indirect)
Multiple Indicator Cluster Survey 2015–2016 (MICS Direct)
VR Vital Registration Data from MOH (Abstract of Statistics 2008)
VR UNPD Demographic Yearbook Data 2020 version
VR WHO

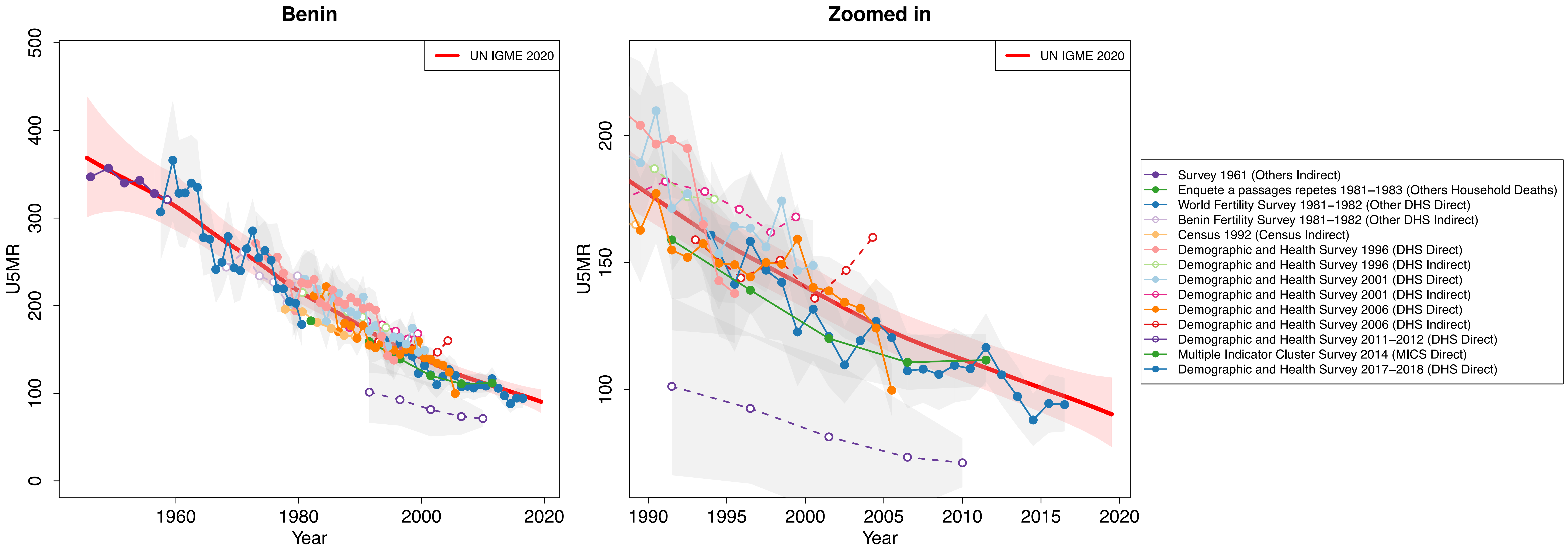
Benin
Zoomed in
UN IGME 2020
U5MR
Year
Survey 1961 (Others Indirect)
Enquete a passages repetes 1981–1983 (Others Household Deaths)
World Fertility Survey 1981–1982 (Other DHS Direct)
Benin Fertility Survey 1981–1982 (Other DHS Indirect)
Census 1992 (Census Indirect)
Demographic and Health Survey 1996 (DHS Direct)
Demographic and Health Survey 1996 (DHS Indirect)
Demographic and Health Survey 2001 (DHS Direct)
Demographic and Health Survey 2001 (DHS Indirect)
Demographic and Health Survey 2006 (DHS Direct)
Demographic and Health Survey 2006 (DHS Indirect)
Demographic and Health Survey 2011–2012 (DHS Direct)
Multiple Indicator Cluster Survey 2014 (MICS Direct)
Demographic and Health Survey 2017–2018 (DHS Direct)

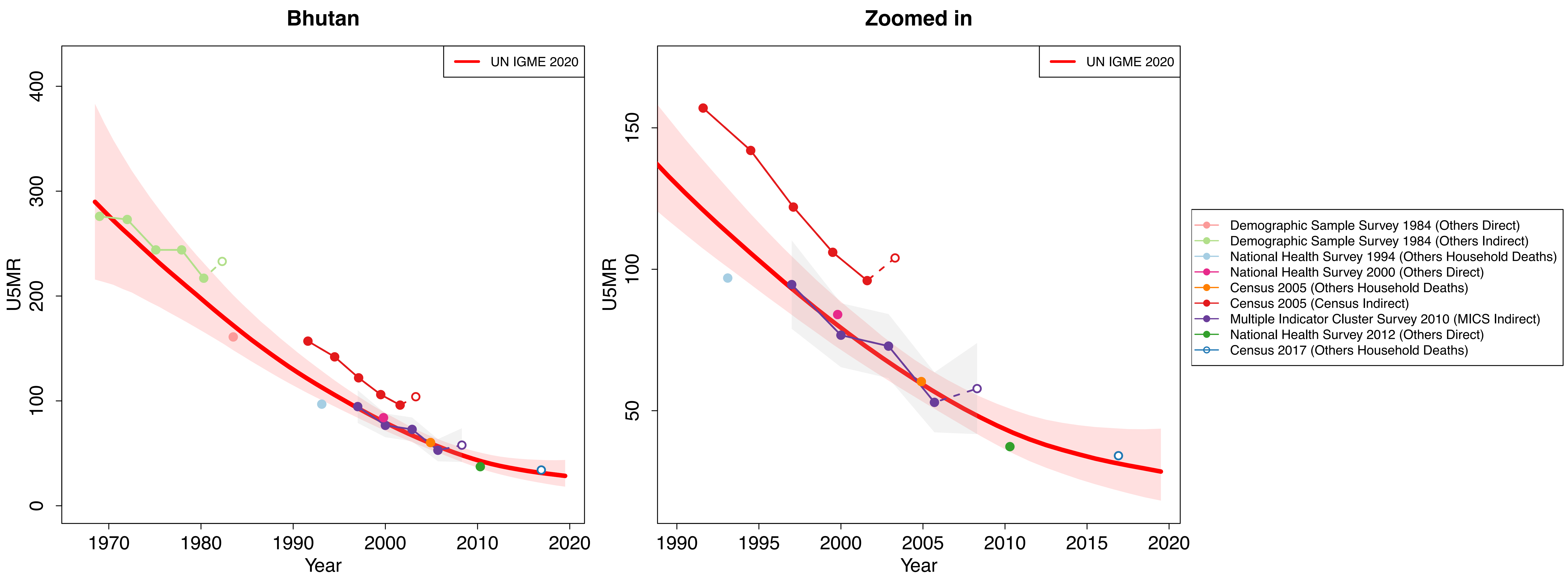

Bhutan
Zoomed in
UN IGME 2020
UN IGME 2020
U5MR
U5MR
Year
Year
0
100
200
300
400
50
100
150
1970
1980
1990
2000
2010
2020
1990
1995
2000
2005
2010
2015
2020
Demographic Sample Survey 1984 (Others Direct)
Demographic Sample Survey 1984 (Others Indirect)
National Health Survey 1994 (Others Household Deaths)
National Health Survey 2000 (Others Direct)
Census 2005 (Others Household Deaths)
Census 2005 (Census Indirect)
Multiple Indicator Cluster Survey 2010 (MICS Indirect)
National Health Survey 2012 (Others Direct)
Census 2017 (Others Household Deaths)

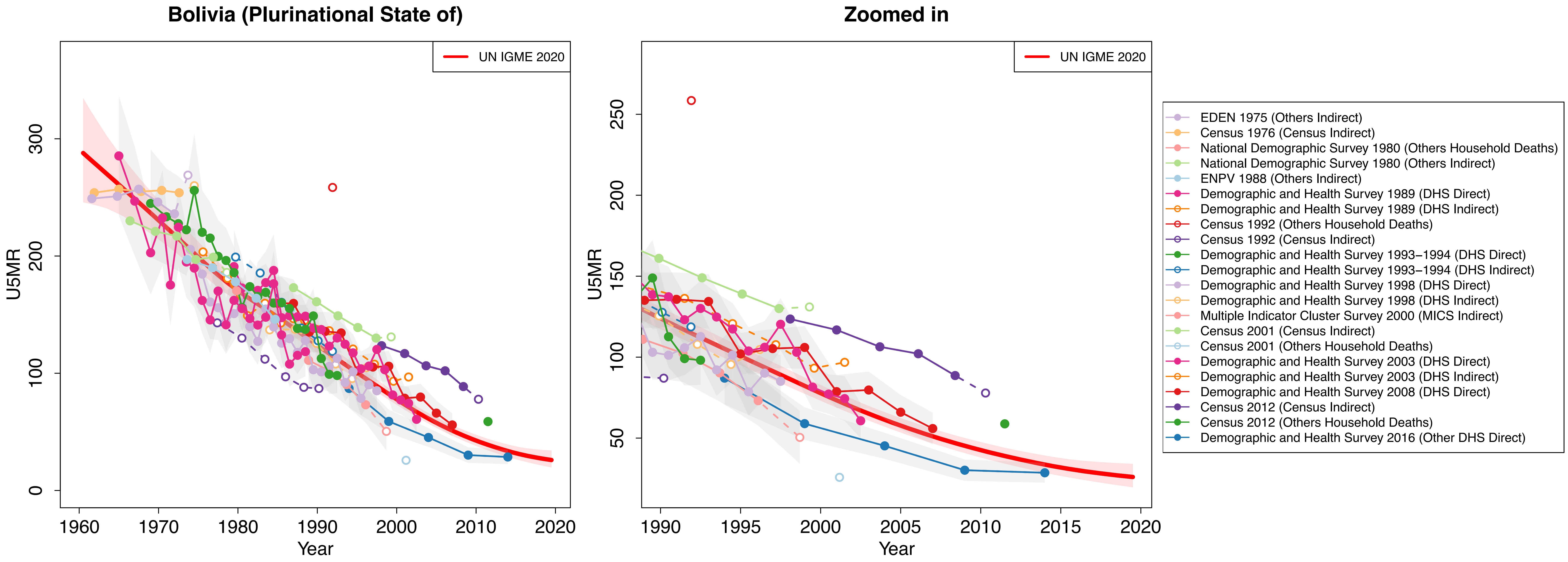
Bolivia (Plurinational State of)
Zoomed in
UN IGME 2020
U5MR
Year
0
100
200
300
50
150
250
1960
1970
1980
1990
2000
2010
2020
1995
2005
2015
EDEN 1975 (Others Indirect)
Census 1976 (Census Indirect)
National Demographic Survey 1980 (Others Household Deaths)
National Demographic Survey 1980 (Others Indirect)
ENPV 1988 (Others Indirect)
Demographic and Health Survey 1989 (DHS Direct)
Demographic and Health Survey 1989 (DHS Indirect)
Census 1992 (Others Household Deaths)
Census 1992 (Census Indirect)
Demographic and Health Survey 1993–1994 (DHS Direct)
Demographic and Health Survey 1993–1994 (DHS Indirect)
Demographic and Health Survey 1998 (DHS Direct)
Demographic and Health Survey 1998 (DHS Indirect)
Multiple Indicator Cluster Survey 2000 (MICS Indirect)
Census 2001 (Census Indirect)
Census 2001 (Others Household Deaths)
Demographic and Health Survey 2003 (DHS Direct)
Demographic and Health Survey 2003 (DHS Indirect)
Demographic and Health Survey 2008 (DHS Direct)
Census 2012 (Census Indirect)
Census 2012 (Others Household Deaths)
Demographic and Health Survey 2016 (Other DHS Direct)

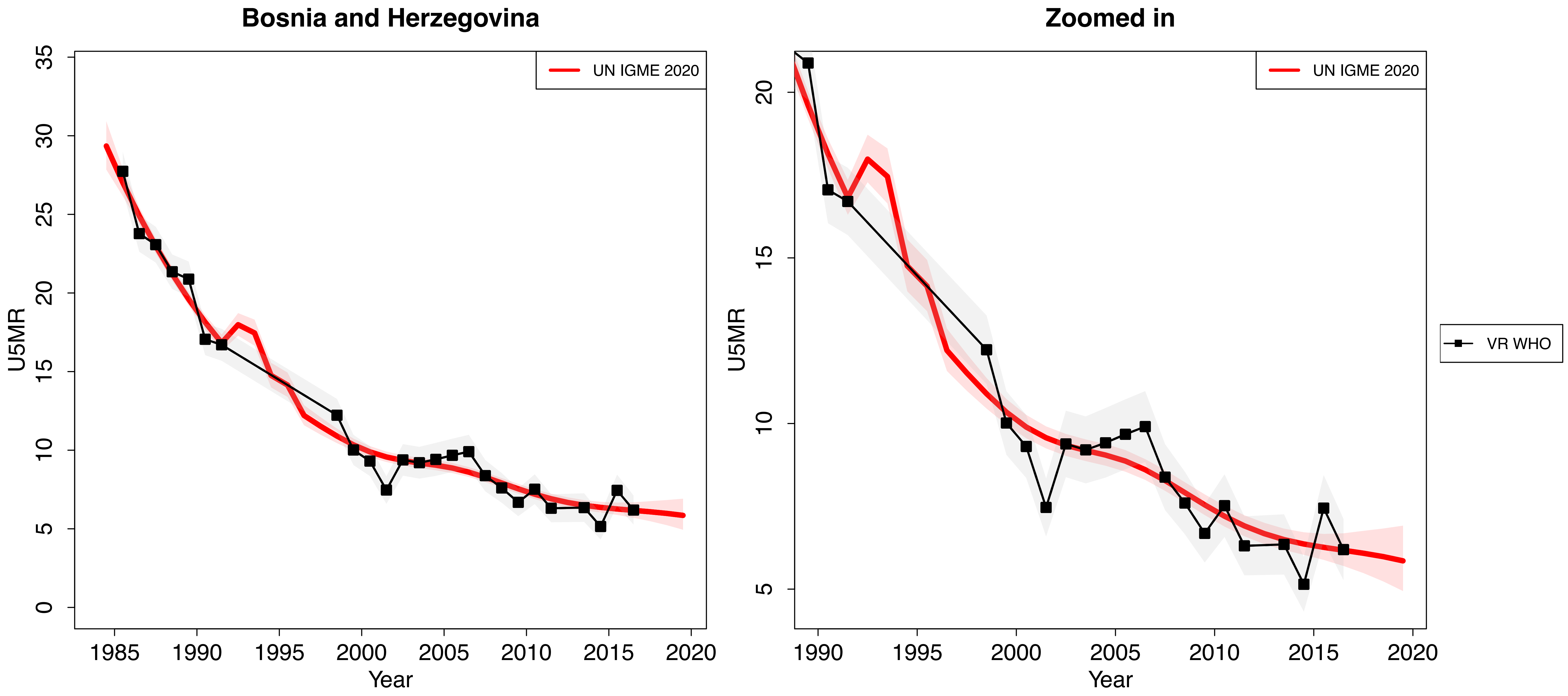

Bosnia and Herzegovina
Zoomed in
UN IGME 2020
VR WHO
U5MR
Year
0
5
10
15
20
25
30
35
1985
1990
1995
2000
2005
2010
2015
2020

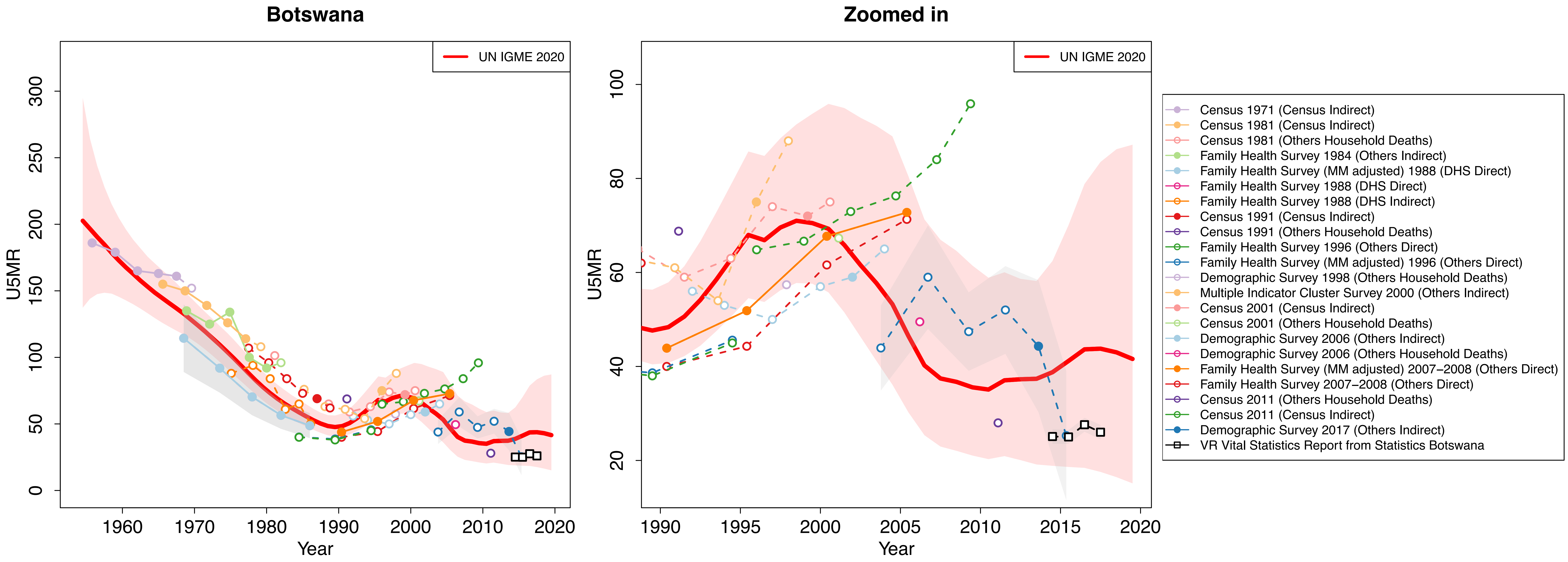

Botswana
Zoomed in
UN IGME 2020
U5MR
Year
0
50
100
150
200
250
300
1960
1970
1980
1990
2000
2010
2020
20
40
60
80
100
1995
2005
2015
Census 1971 (Census Indirect)
Census 1981 (Census Indirect)
Census 1981 (Others Household Deaths)
Family Health Survey 1984 (Others Indirect)
Family Health Survey (MM adjusted) 1988 (DHS Direct)
Family Health Survey 1988 (DHS Direct)
Family Health Survey 1988 (DHS Indirect)
Census 1991 (Census Indirect)
Census 1991 (Others Household Deaths)
Family Health Survey 1996 (Others Direct)
Family Health Survey (MM adjusted) 1996 (Others Direct)
Demographic Survey 1998 (Others Household Deaths)
Multiple Indicator Cluster Survey 2000 (Others Indirect)
Census 2001 (Census Indirect)
Census 2001 (Others Household Deaths)
Demographic Survey 2006 (Others Indirect)
Demographic Survey 2006 (Others Household Deaths)
Family Health Survey (MM adjusted) 2007–2008 (Others Direct)
Family Health Survey 2007–2008 (Others Direct)
Census 2011 (Others Household Deaths)
Census 2011 (Census Indirect)
Demographic Survey 2017 (Others Indirect)
VR Vital Statistics Report from Statistics Botswana

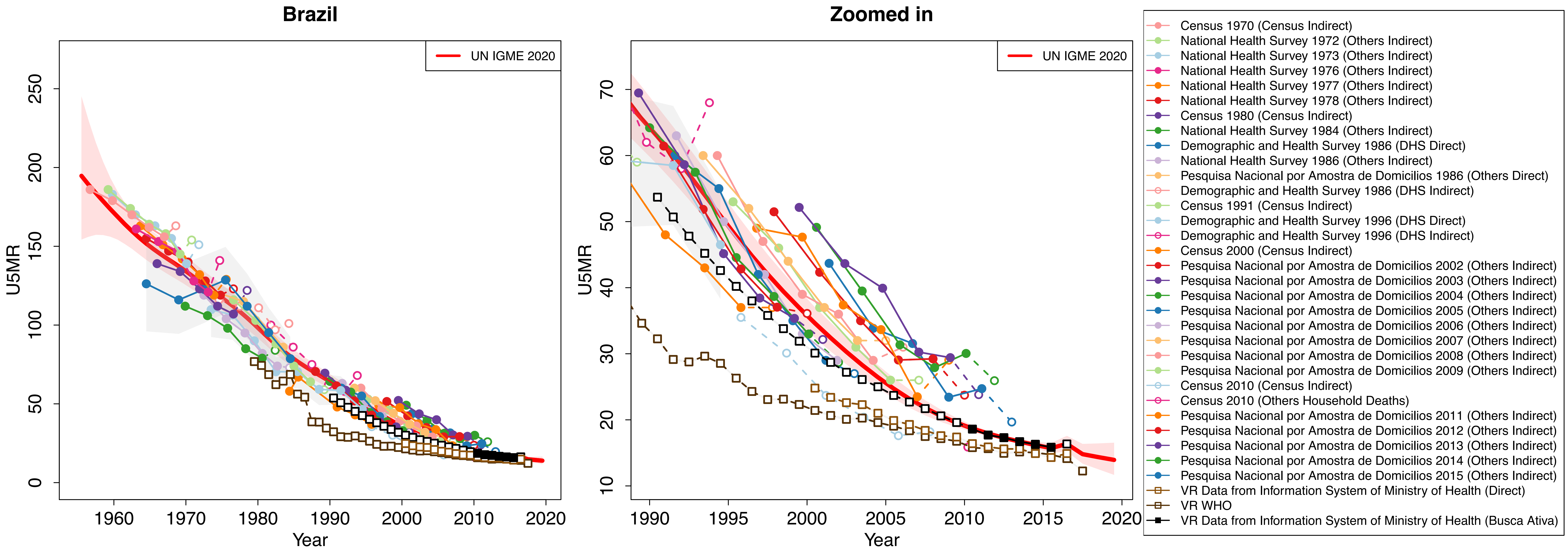
Brazil
Zoomed in
UN IGME 2020
U5MR
Year
Census 1970 (Census Indirect)
National Health Survey 1972 (Others Indirect)
National Health Survey 1973 (Others Indirect)
National Health Survey 1976 (Others Indirect)
National Health Survey 1977 (Others Indirect)
National Health Survey 1978 (Others Indirect)
Census 1980 (Census Indirect)
National Health Survey 1984 (Others Indirect)
Demographic and Health Survey 1986 (DHS Direct)
National Health Survey 1986 (Others Indirect)
Pesquisa Nacional por Amostra de Domicilios 1986 (Others Direct)
Demographic and Health Survey 1986 (DHS Indirect)
Census 1991 (Census Indirect)
Demographic and Health Survey 1996 (DHS Direct)
Demographic and Health Survey 1996 (DHS Indirect)
Census 2000 (Census Indirect)
Pesquisa Nacional por Amostra de Domicilios 2002 (Others Indirect)
Pesquisa Nacional por Amostra de Domicilios 2003 (Others Indirect)
Pesquisa Nacional por Amostra de Domicilios 2004 (Others Indirect)
Pesquisa Nacional por Amostra de Domicilios 2005 (Others Indirect)
Pesquisa Nacional por Amostra de Domicilios 2006 (Others Indirect)
Pesquisa Nacional por Amostra de Domicilios 2007 (Others Indirect)
Pesquisa Nacional por Amostra de Domicilios 2008 (Others Indirect)
Pesquisa Nacional por Amostra de Domicilios 2009 (Others Indirect)
Census 2010 (Census Indirect)
Census 2010 (Others Household Deaths)
Pesquisa Nacional por Amostra de Domicilios 2011 (Others Indirect)
Pesquisa Nacional por Amostra de Domicilios 2012 (Others Indirect)
Pesquisa Nacional por Amostra de Domicilios 2013 (Others Indirect)
Pesquisa Nacional por Amostra de Domicilios 2014 (Others Indirect)
Pesquisa Nacional por Amostra de Domicilios 2015 (Others Indirect)
VR Data from Information System of Ministry of Health (Direct)
VR WHO
VR Data from Information System of Ministry of Health (Busca Ativa)

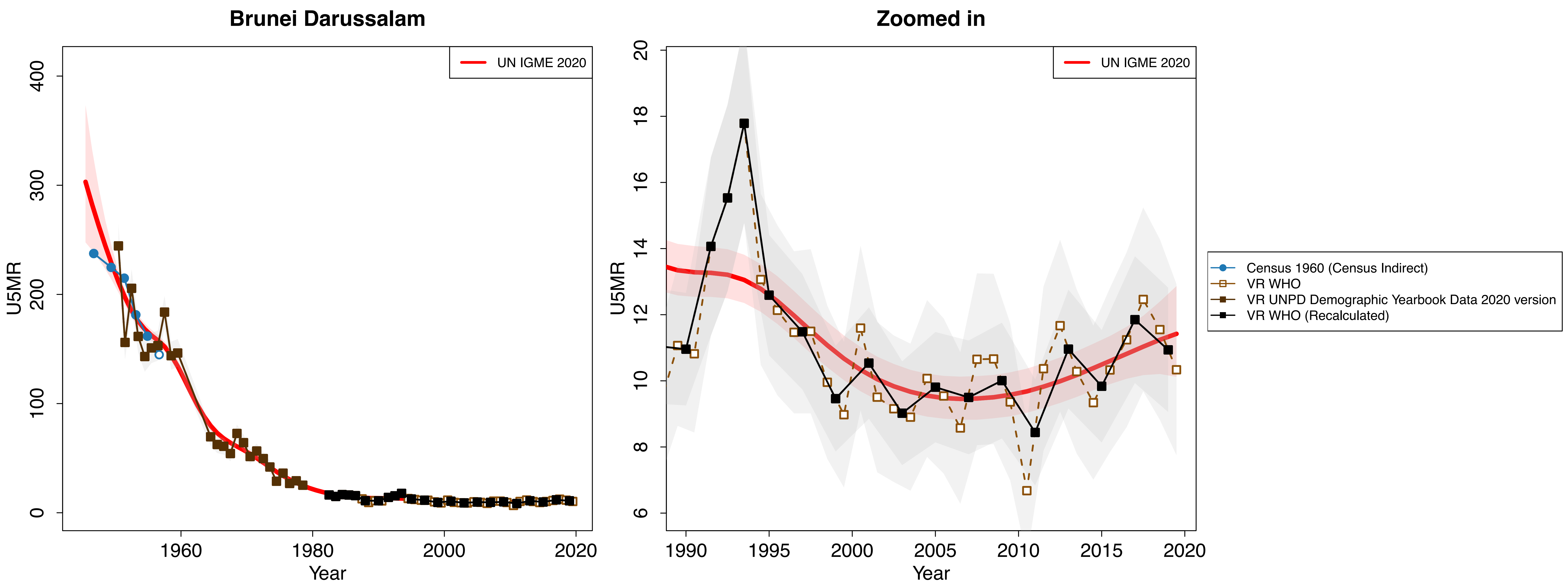

Brunei Darussalam
Zoomed in
UN IGME 2020
U5MR
Year
Census 1960 (Census Indirect)
VR WHO
VR UNPD Demographic Yearbook Data 2020 version
VR WHO (Recalculated)

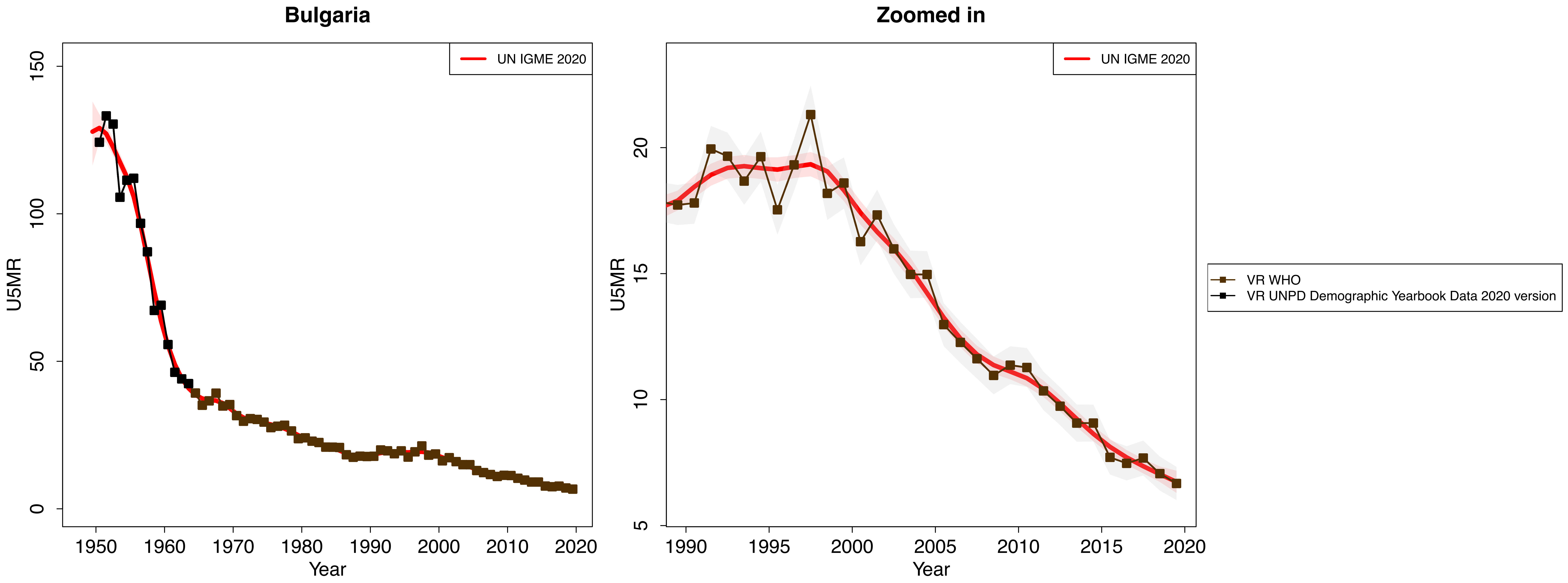

Bulgaria
Zoomed in
UN IGME 2020
UN IGME 2020
U5MR
U5MR
Year
Year
0
50
100
150
5
10
15
20
1950
1960
1970
1980
1990
2000
2010
2020
1990
1995
2000
2005
2010
2015
2020
VR WHO
VR UNPD Demographic Yearbook Data 2020 version

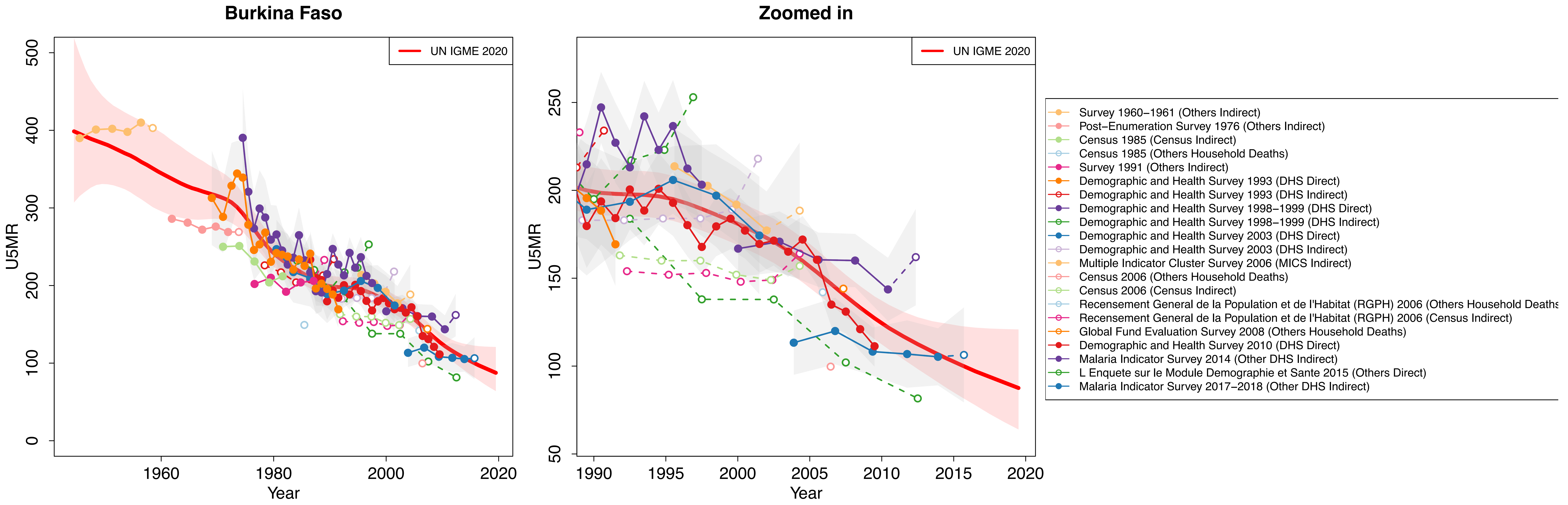

Burkina Faso
Zoomed in
UN IGME 2020
U5MR
Year
Survey 1960−1961 (Others Indirect)
Post−Enumeration Survey 1976 (Others Indirect)
Census 1985 (Census Indirect)
Census 1985 (Others Household Deaths)
Survey 1991 (Others Indirect)
Demographic and Health Survey 1993 (DHS Direct)
Demographic and Health Survey 1993 (DHS Indirect)
Demographic and Health Survey 1998−1999 (DHS Direct)
Demographic and Health Survey 1998−1999 (DHS Indirect)
Demographic and Health Survey 2003 (DHS Direct)
Demographic and Health Survey 2003 (DHS Indirect)
Multiple Indicator Cluster Survey 2006 (MICS Indirect)
Census 2006 (Others Household Deaths)
Census 2006 (Census Indirect)
Recensement General de la Population et de l'Habitat (RGPH) 2006 (Others Household Deaths
Recensement General de la Population et de l'Habitat (RGPH) 2006 (Census Indirect)
Global Fund Evaluation Survey 2008 (Others Household Deaths)
Demographic and Health Survey 2010 (DHS Direct)
Malaria Indicator Survey 2014 (Other DHS Indirect)
L Enquete sur le Module Demographie et Sante 2015 (Others Direct)
Malaria Indicator Survey 2017−2018 (Other DHS Indirect)

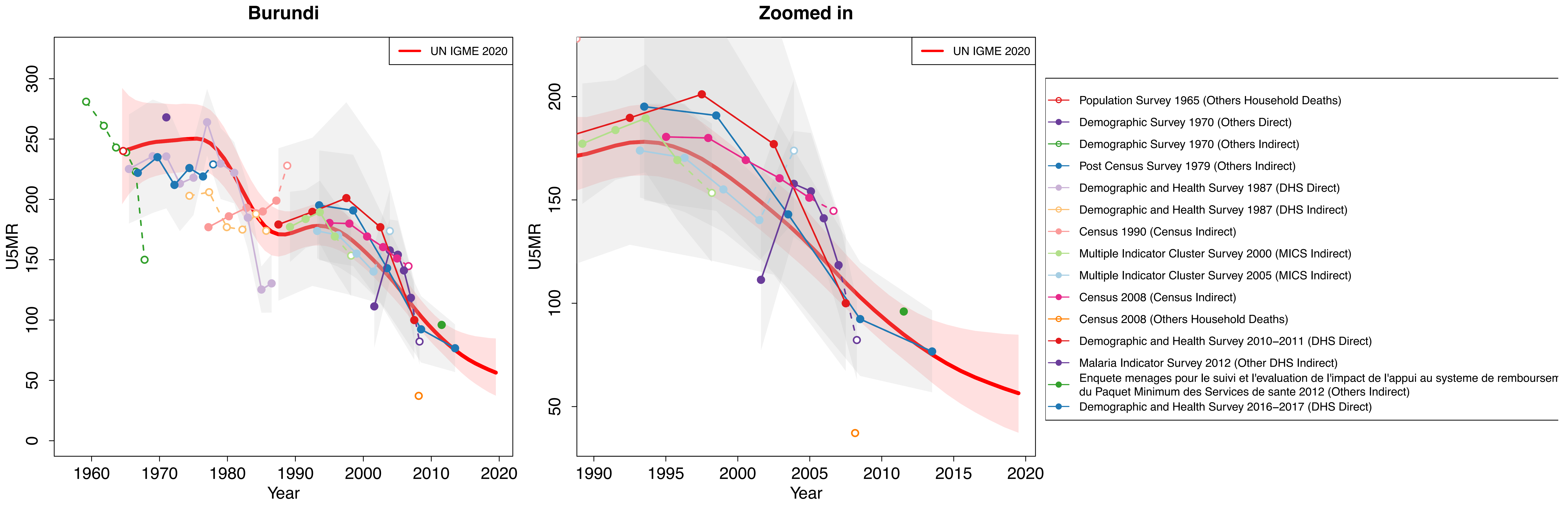

Burundi
Zoomed in
UN IGME 2020
U5MR
Year
Population Survey 1965 (Others Household Deaths)
Demographic Survey 1970 (Others Direct)
Demographic Survey 1970 (Others Indirect)
Post Census Survey 1979 (Others Indirect)
Demographic and Health Survey 1987 (DHS Direct)
Demographic and Health Survey 1987 (DHS Indirect)
Census 1990 (Census Indirect)
Multiple Indicator Cluster Survey 2000 (MICS Indirect)
Multiple Indicator Cluster Survey 2005 (MICS Indirect)
Census 2008 (Census Indirect)
Census 2008 (Others Household Deaths)
Demographic and Health Survey 2010–2011 (DHS Direct)
Malaria Indicator Survey 2012 (Other DHS Indirect)
Enquete menages pour le suivi et l'evaluation de l'impact de l'appui au systeme de rembourser
du Paquet Minimum des Services de sante 2012 (Others Indirect)
Demographic and Health Survey 2016–2017 (DHS Direct)

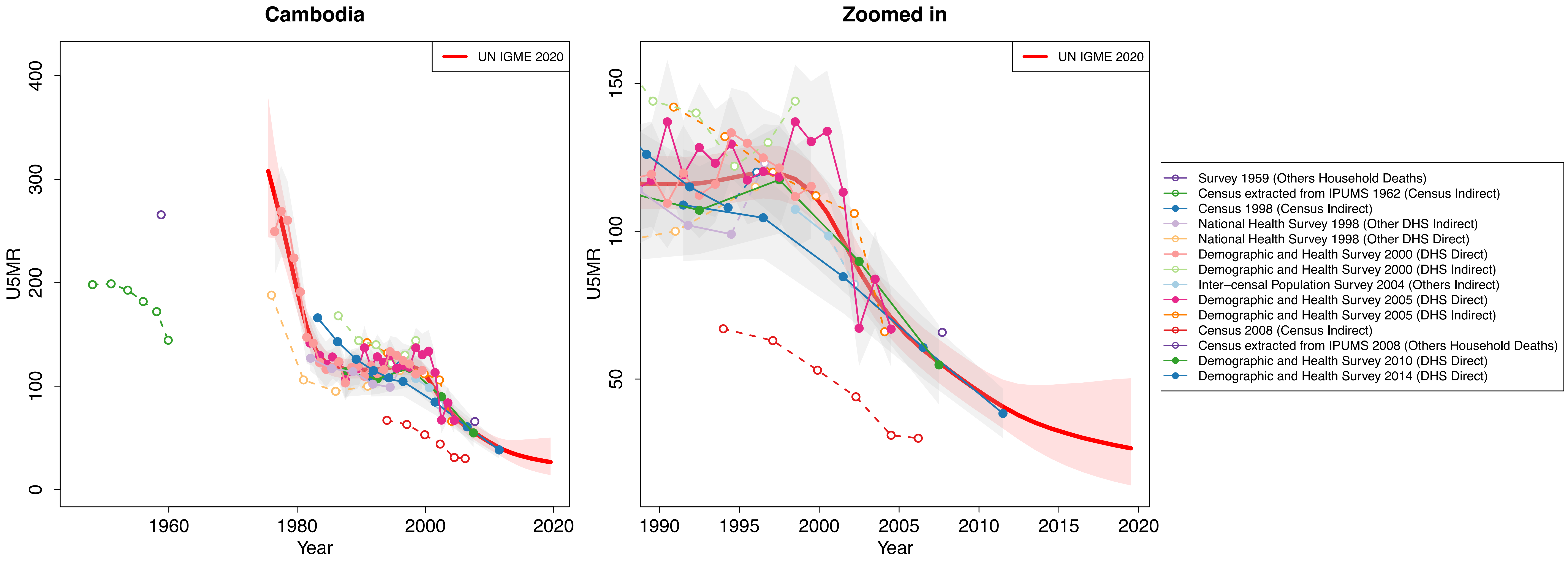

Cambodia
Zoomed in
UN IGME 2020
U5MR
Year
Survey 1959 (Others Household Deaths)
Census extracted from IPUMS 1962 (Census Indirect)
Census 1998 (Census Indirect)
National Health Survey 1998 (Other DHS Indirect)
National Health Survey 1998 (Other DHS Direct)
Demographic and Health Survey 2000 (DHS Direct)
Demographic and Health Survey 2000 (DHS Indirect)
Inter−censal Population Survey 2004 (Others Indirect)
Demographic and Health Survey 2005 (DHS Direct)
Demographic and Health Survey 2005 (DHS Indirect)
Census 2008 (Census Indirect)
Census extracted from IPUMS 2008 (Others Household Deaths)
Demographic and Health Survey 2010 (DHS Direct)
Demographic and Health Survey 2014 (DHS Direct)

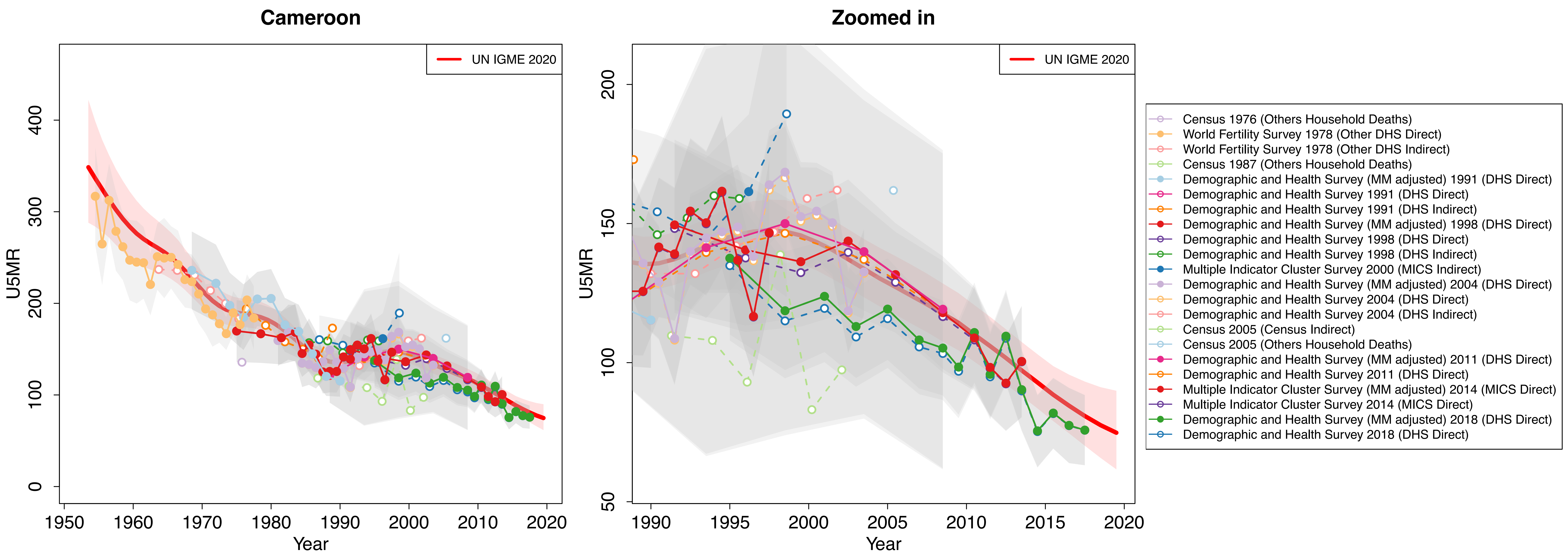

Cameroon
Zoomed in
UN IGME 2020
U5MR
Year
0
100
200
300
400
1950
1960
1970
1980
1990
2000
2010
2020
50
100
150
200
1995
2005
2015
Census 1976 (Others Household Deaths)
World Fertility Survey 1978 (Other DHS Direct)
World Fertility Survey 1978 (Other DHS Indirect)
Census 1987 (Others Household Deaths)
Demographic and Health Survey (MM adjusted) 1991 (DHS Direct)
Demographic and Health Survey 1991 (DHS Direct)
Demographic and Health Survey 1991 (DHS Indirect)
Demographic and Health Survey (MM adjusted) 1998 (DHS Direct)
Demographic and Health Survey 1998 (DHS Direct)
Demographic and Health Survey 1998 (DHS Indirect)
Multiple Indicator Cluster Survey 2000 (MICS Indirect)
Demographic and Health Survey (MM adjusted) 2004 (DHS Direct)
Demographic and Health Survey 2004 (DHS Direct)
Demographic and Health Survey 2004 (DHS Indirect)
Census 2005 (Census Indirect)
Census 2005 (Others Household Deaths)
Demographic and Health Survey (MM adjusted) 2011 (DHS Direct)
Demographic and Health Survey 2011 (DHS Direct)
Multiple Indicator Cluster Survey (MM adjusted) 2014 (MICS Direct)
Multiple Indicator Cluster Survey 2014 (MICS Direct)
Demographic and Health Survey (MM adjusted) 2018 (DHS Direct)
Demographic and Health Survey 2018 (DHS Direct)

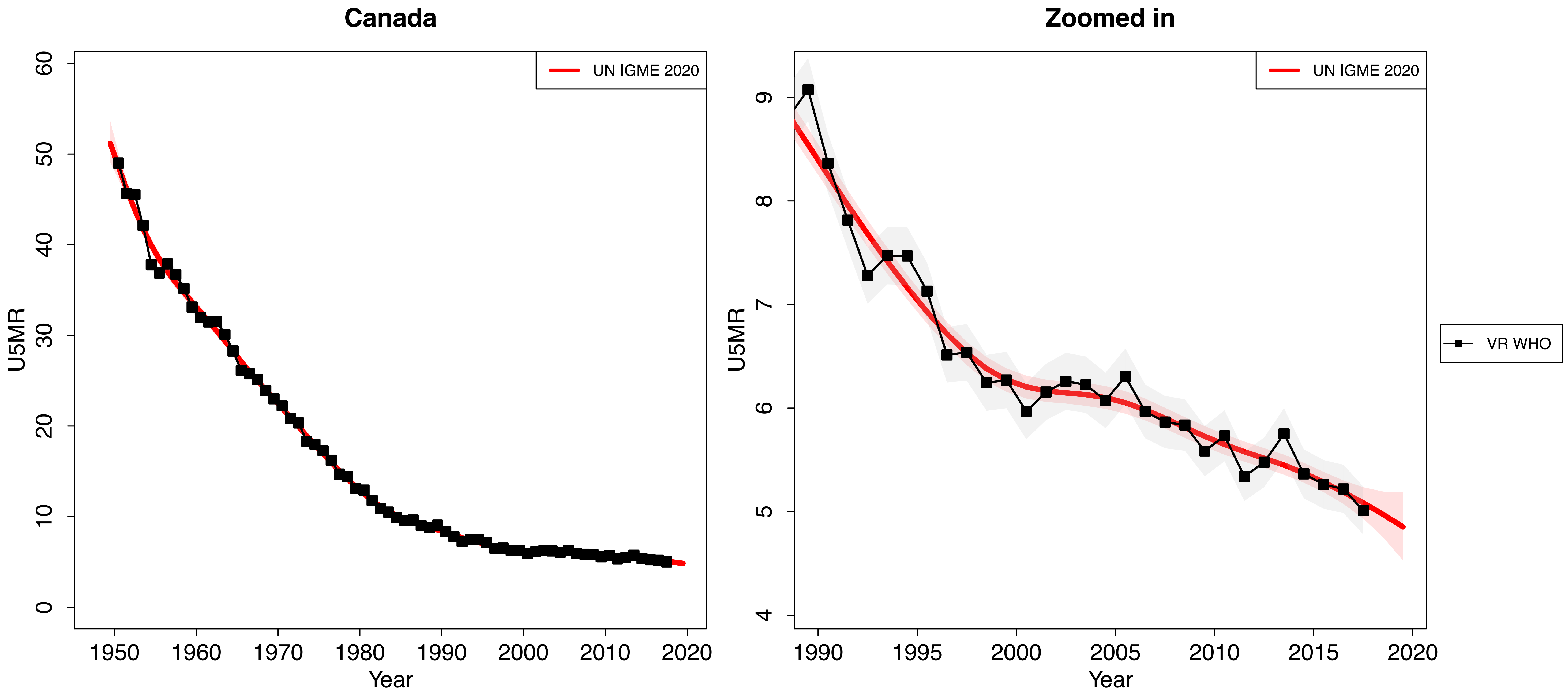
Canada
Zoomed in
UN IGME 2020
VR WHO
U5MR
Year
0
10
20
30
40
50
60
1950
1960
1970
1980
1990
2000
2010
2020
4
5
6
7
8
9
1995
2005
2015

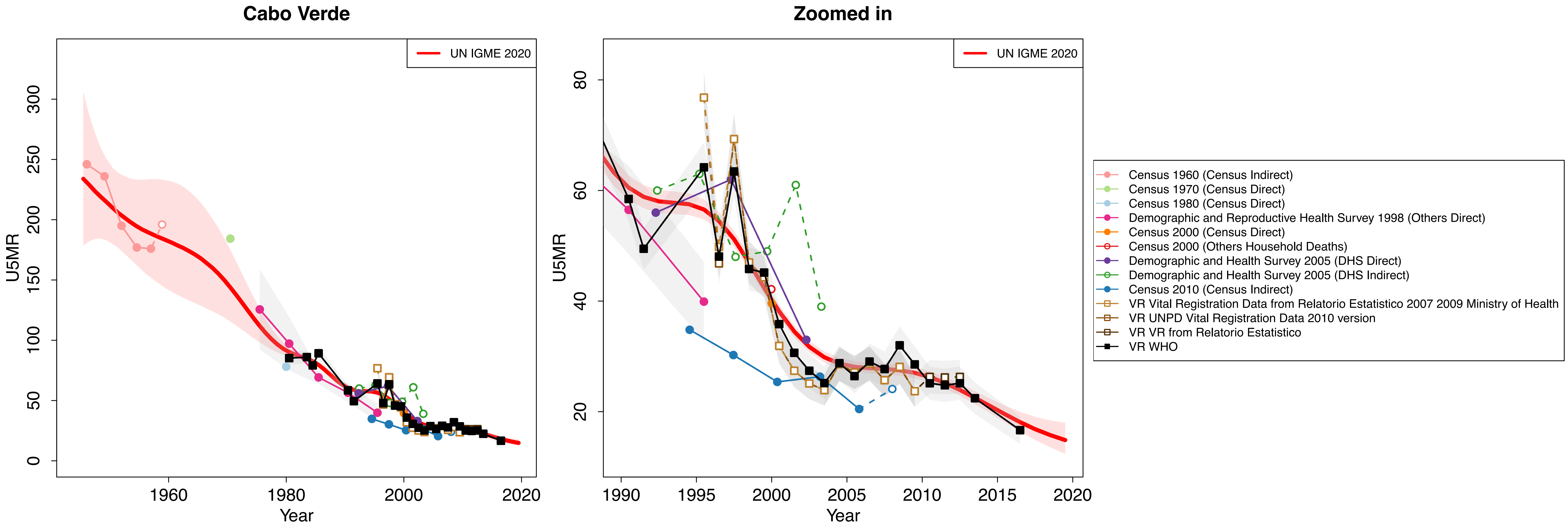

Cabo Verde
Zoomed in
UN IGME 2020
UN IGME 2020
U5MR
U5MR
Year
Year
0
50
100
150
200
250
300
1960
1980
2000
2020
20
40
60
80
1990
1995
2000
2005
2010
2015
2020
Census 1960 (Census Indirect)
Census 1970 (Census Direct)
Census 1980 (Census Direct)
Demographic and Reproductive Health Survey 1998 (Others Direct)
Census 2000 (Census Direct)
Census 2000 (Others Household Deaths)
Demographic and Health Survey 2005 (DHS Direct)
Demographic and Health Survey 2005 (DHS Indirect)
Census 2010 (Census Indirect)
VR Vital Registration Data from Relatorio Estatistico 2007 2009 Ministry of Health
VR UNPD Vital Registration Data 2010 version
VR VR from Relatorio Estatistico
VR WHO

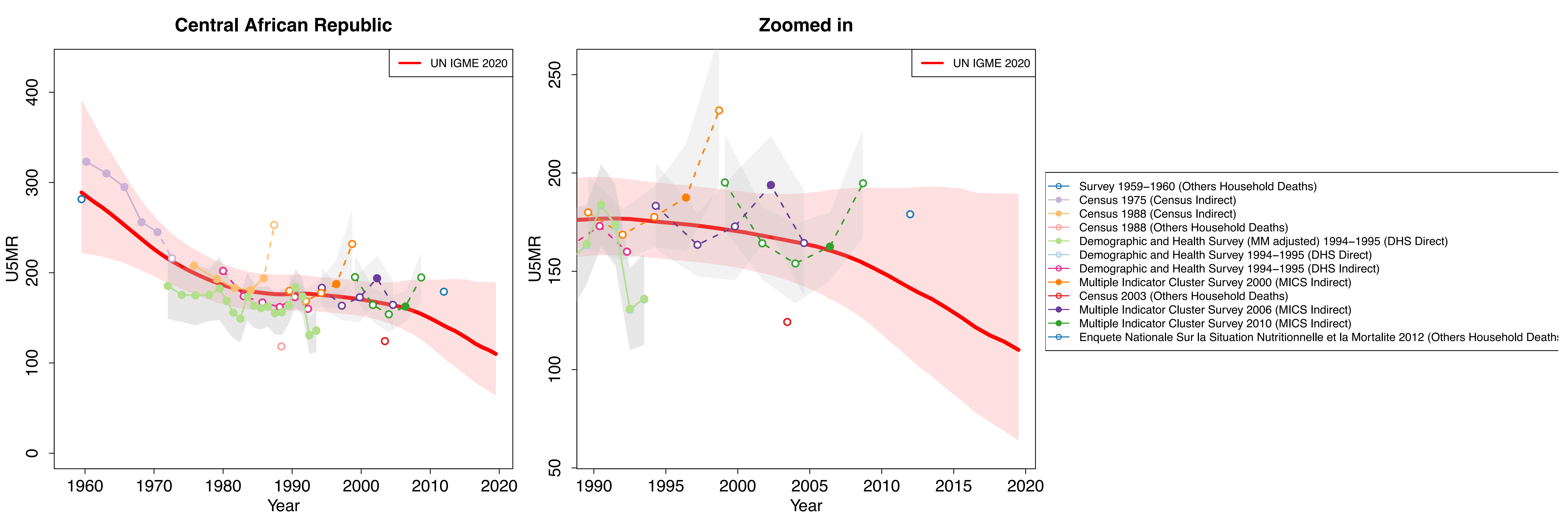
Central African Republic
Zoomed in
UN IGME 2020
U5MR
Year
Survey 1959–1960 (Others Household Deaths)
Census 1975 (Census Indirect)
Census 1988 (Census Indirect)
Census 1988 (Others Household Deaths)
Demographic and Health Survey (MM adjusted) 1994–1995 (DHS Direct)
Demographic and Health Survey 1994–1995 (DHS Direct)
Demographic and Health Survey 1994–1995 (DHS Indirect)
Multiple Indicator Cluster Survey 2000 (MICS Indirect)
Census 2003 (Others Household Deaths)
Multiple Indicator Cluster Survey 2006 (MICS Indirect)
Multiple Indicator Cluster Survey 2010 (MICS Indirect)
Enquete Nationale Sur la Situation Nutritionnelle et la Mortalite 2012 (Others Household Deaths

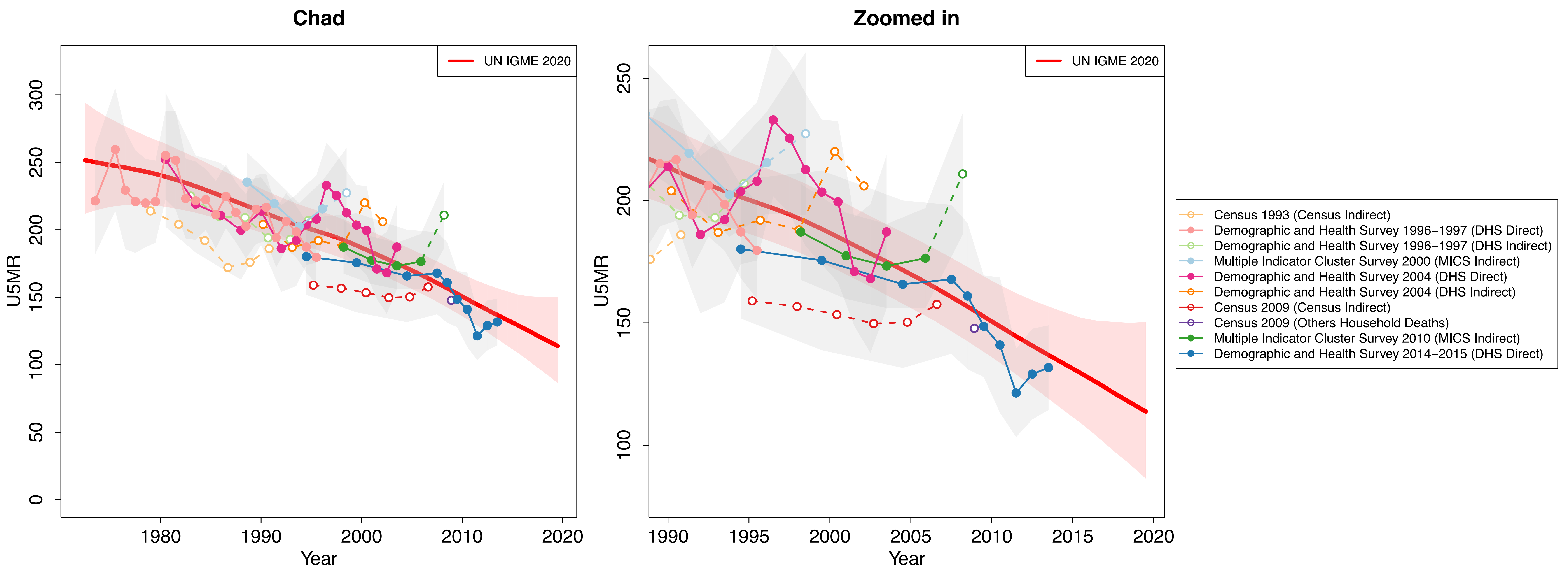

Chad
Zoomed in
UN IGME 2020
U5MR
Year
0
50
100
150
200
250
300
1980
1990
2000
2010
2020
1995
2005
2015
Census 1993 (Census Indirect)
Demographic and Health Survey 1996–1997 (DHS Direct)
Demographic and Health Survey 1996–1997 (DHS Indirect)
Multiple Indicator Cluster Survey 2000 (MICS Indirect)
Demographic and Health Survey 2004 (DHS Direct)
Demographic and Health Survey 2004 (DHS Indirect)
Census 2009 (Census Indirect)
Census 2009 (Others Household Deaths)
Multiple Indicator Cluster Survey 2010 (MICS Indirect)
Demographic and Health Survey 2014–2015 (DHS Direct)

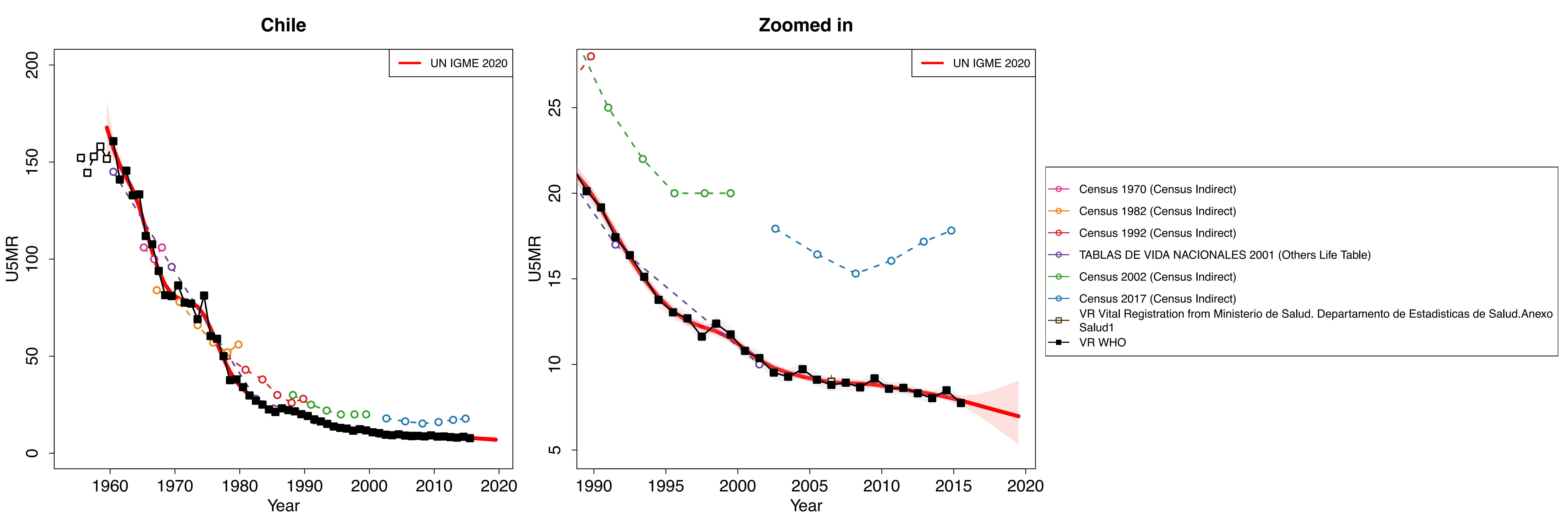
Chile
Zoomed in
UN IGME 2020
U5MR
Year
Census 1970 (Census Indirect)
Census 1982 (Census Indirect)
Census 1992 (Census Indirect)
TABLAS DE VIDA NACIONALES 2001 (Others Life Table)
Census 2002 (Census Indirect)
Census 2017 (Census Indirect)
VR Vital Registration from Ministerio de Salud. Departamento de Estadisticas de Salud.Anexo Salud1
VR WHO

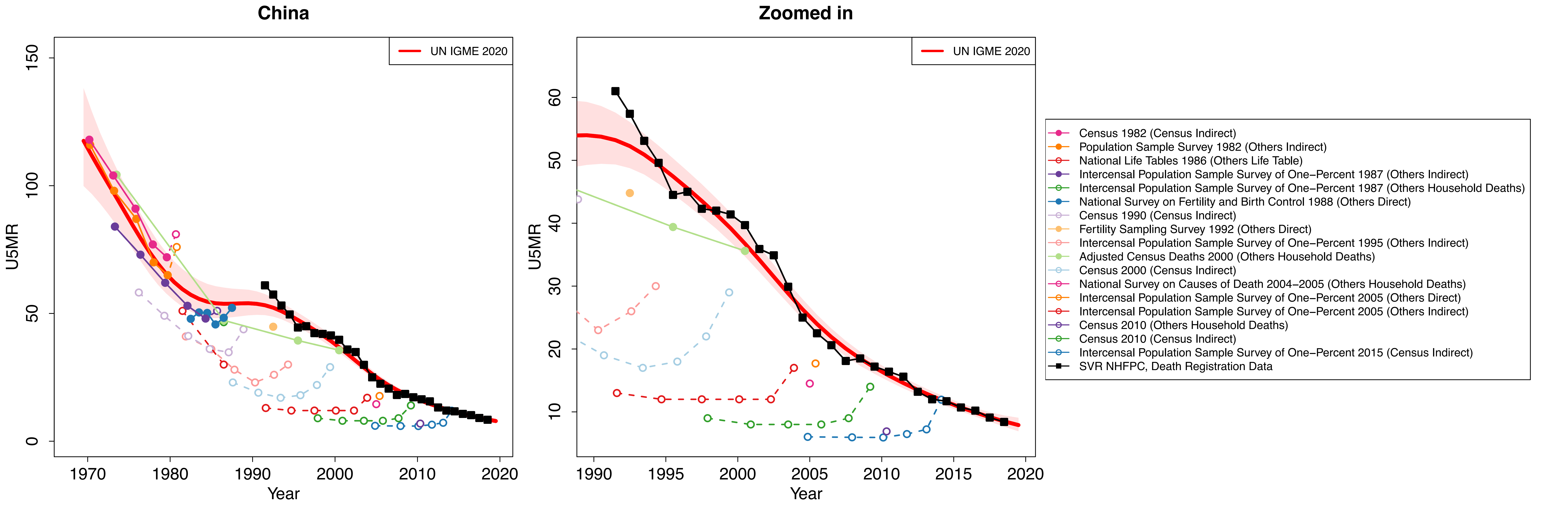
China
Zoomed in
UN IGME 2020
U5MR
Year
Census 1982 (Census Indirect)
Population Sample Survey 1982 (Others Indirect)
National Life Tables 1986 (Others Life Table)
Intercensal Population Sample Survey of One−Percent 1987 (Others Indirect)
Intercensal Population Sample Survey of One−Percent 1987 (Others Household Deaths)
National Survey on Fertility and Birth Control 1988 (Others Direct)
Census 1990 (Census Indirect)
Fertility Sampling Survey 1992 (Others Direct)
Intercensal Population Sample Survey of One−Percent 1995 (Others Indirect)
Adjusted Census Deaths 2000 (Others Household Deaths)
Census 2000 (Census Indirect)
National Survey on Causes of Death 2004−2005 (Others Household Deaths)
Intercensal Population Sample Survey of One−Percent 2005 (Others Direct)
Intercensal Population Sample Survey of One−Percent 2005 (Others Indirect)
Census 2010 (Others Household Deaths)
Census 2010 (Census Indirect)
Intercensal Population Sample Survey of One−Percent 2015 (Census Indirect)
SVR NHFPC, Death Registration Data

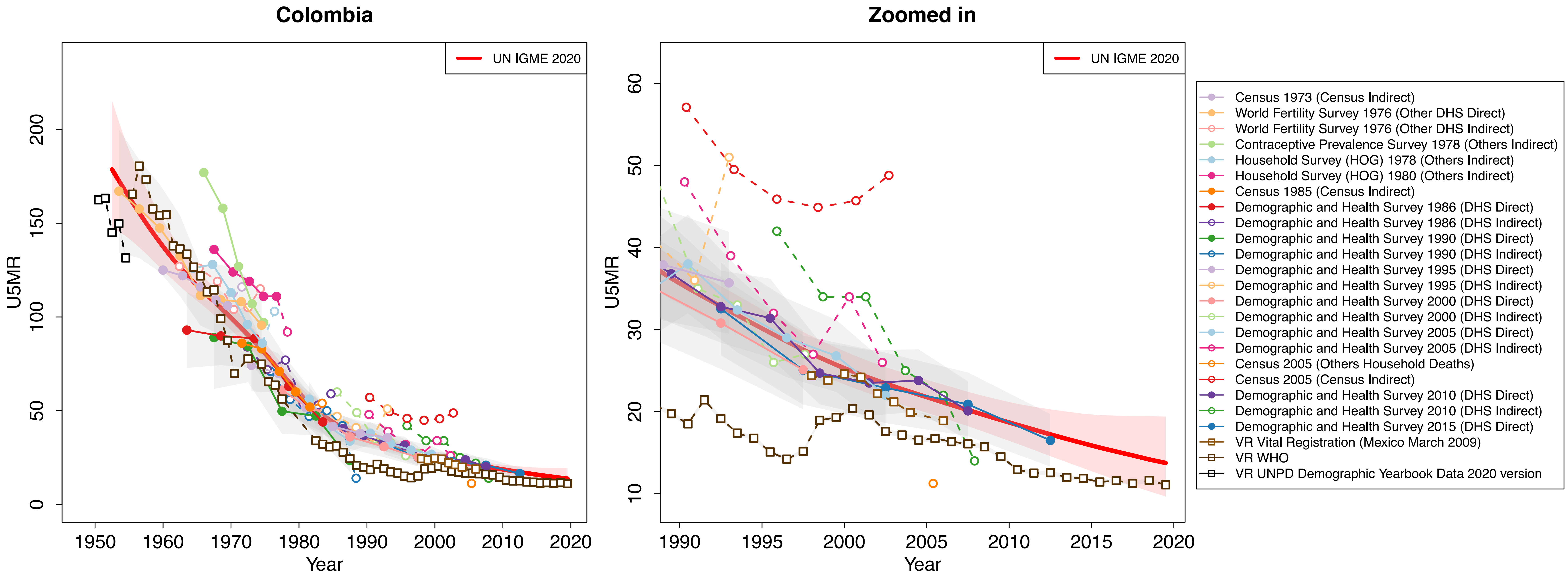
Colombia
Zoomed in
UN IGME 2020
U5MR
Year
Census 1973 (Census Indirect)
World Fertility Survey 1976 (Other DHS Direct)
World Fertility Survey 1976 (Other DHS Indirect)
Contraceptive Prevalence Survey 1978 (Others Indirect)
Household Survey (HOG) 1978 (Others Indirect)
Household Survey (HOG) 1980 (Others Indirect)
Census 1985 (Census Indirect)
Demographic and Health Survey 1986 (DHS Direct)
Demographic and Health Survey 1986 (DHS Indirect)
Demographic and Health Survey 1990 (DHS Direct)
Demographic and Health Survey 1990 (DHS Indirect)
Demographic and Health Survey 1995 (DHS Direct)
Demographic and Health Survey 1995 (DHS Indirect)
Demographic and Health Survey 2000 (DHS Direct)
Demographic and Health Survey 2000 (DHS Indirect)
Demographic and Health Survey 2005 (DHS Direct)
Demographic and Health Survey 2005 (DHS Indirect)
Census 2005 (Others Household Deaths)
Census 2005 (Census Indirect)
Demographic and Health Survey 2010 (DHS Direct)
Demographic and Health Survey 2010 (DHS Indirect)
Demographic and Health Survey 2015 (DHS Direct)
VR Vital Registration (Mexico March 2009)
VR WHO
VR UNPD Demographic Yearbook Data 2020 version

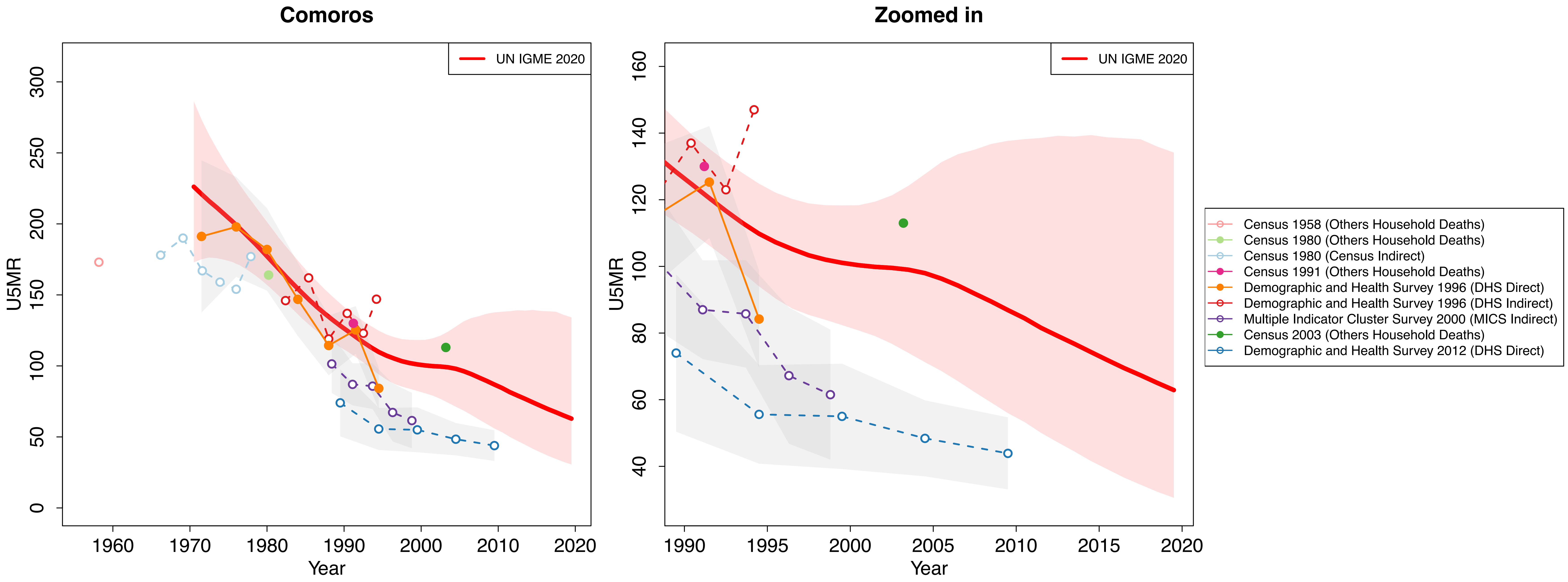

Comoros
Zoomed in
UN IGME 2020
UN IGME 2020
U5MR
Year
Census 1958 (Others Household Deaths)
Census 1980 (Others Household Deaths)
Census 1980 (Census Indirect)
Census 1991 (Others Household Deaths)
Demographic and Health Survey 1996 (DHS Direct)
Demographic and Health Survey 1996 (DHS Indirect)
Multiple Indicator Cluster Survey 2000 (MICS Indirect)
Census 2003 (Others Household Deaths)
Demographic and Health Survey 2012 (DHS Direct)

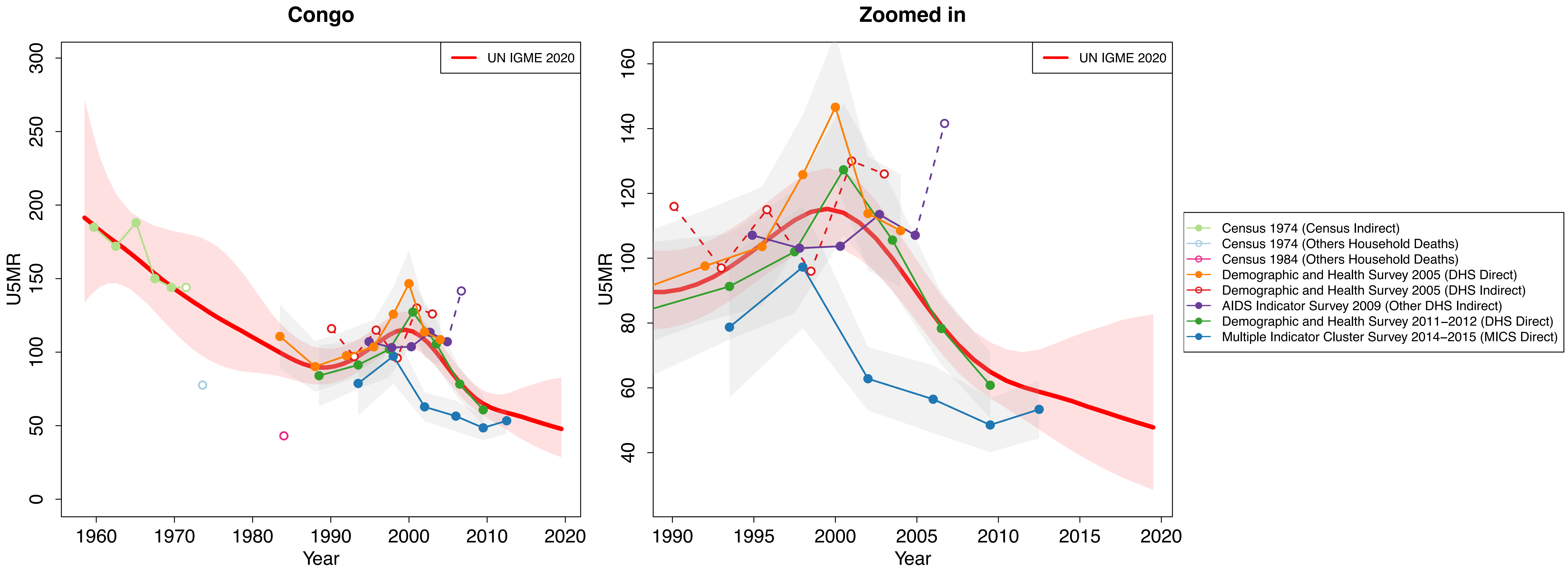
Congo
Zoomed in
UN IGME 2020
UN IGME 2020
U5MR
U5MR
Year
Year
0
50
100
150
200
250
300
40
60
80
100
120
140
160
1960
1970
1980
1990
2000
2010
2020
1990
1995
2000
2005
2010
2015
2020
Census 1974 (Census Indirect)
Census 1974 (Others Household Deaths)
Census 1984 (Others Household Deaths)
Demographic and Health Survey 2005 (DHS Direct)
Demographic and Health Survey 2005 (DHS Indirect)
AIDS Indicator Survey 2009 (Other DHS Indirect)
Demographic and Health Survey 2011–2012 (DHS Direct)
Multiple Indicator Cluster Survey 2014–2015 (MICS Direct)

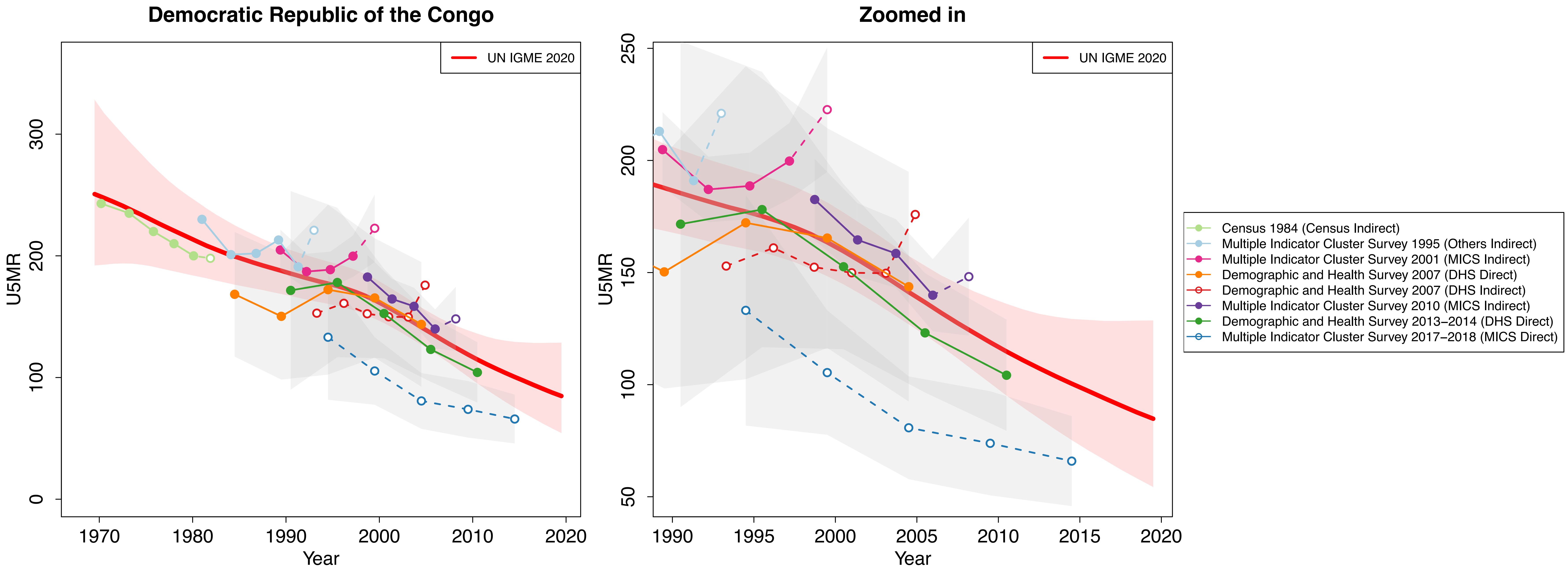

Democratic Republic of the Congo
Zoomed in
UN IGME 2020
U5MR
Year
0
100
200
300
1970
1980
1990
2000
2010
2020
50
100
150
200
250
1995
2005
2015
Census 1984 (Census Indirect)
Multiple Indicator Cluster Survey 1995 (Others Indirect)
Multiple Indicator Cluster Survey 2001 (MICS Indirect)
Demographic and Health Survey 2007 (DHS Direct)
Demographic and Health Survey 2007 (DHS Indirect)
Multiple Indicator Cluster Survey 2010 (MICS Indirect)
Demographic and Health Survey 2013–2014 (DHS Direct)
Multiple Indicator Cluster Survey 2017–2018 (MICS Direct)

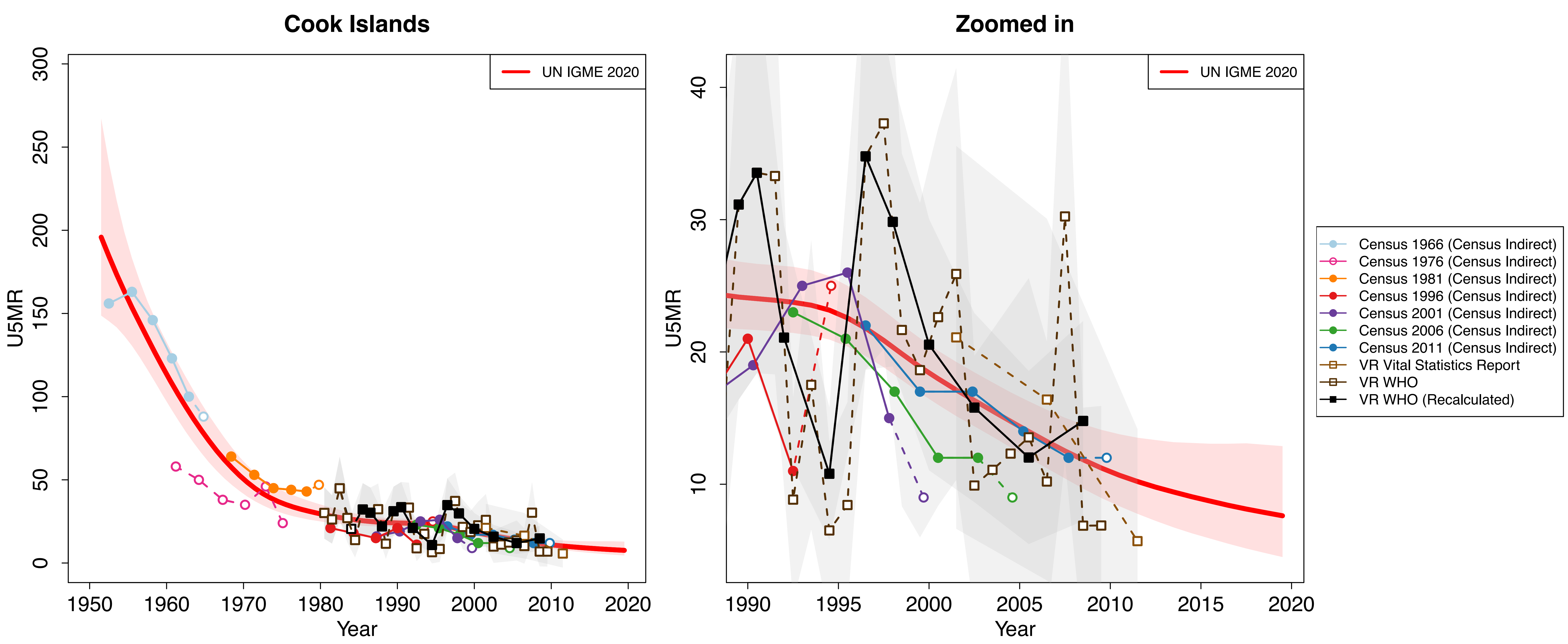

Cook Islands
Zoomed in
UN IGME 2020
U5MR
Year
0
50
100
150
200
250
300
1950
1960
1970
1980
1990
2000
2010
2020
10
20
30
40
1995
2005
2015
Census 1966 (Census Indirect)
Census 1976 (Census Indirect)
Census 1981 (Census Indirect)
Census 1996 (Census Indirect)
Census 2001 (Census Indirect)
Census 2006 (Census Indirect)
Census 2011 (Census Indirect)
VR Vital Statistics Report
VR WHO
VR WHO (Recalculated)

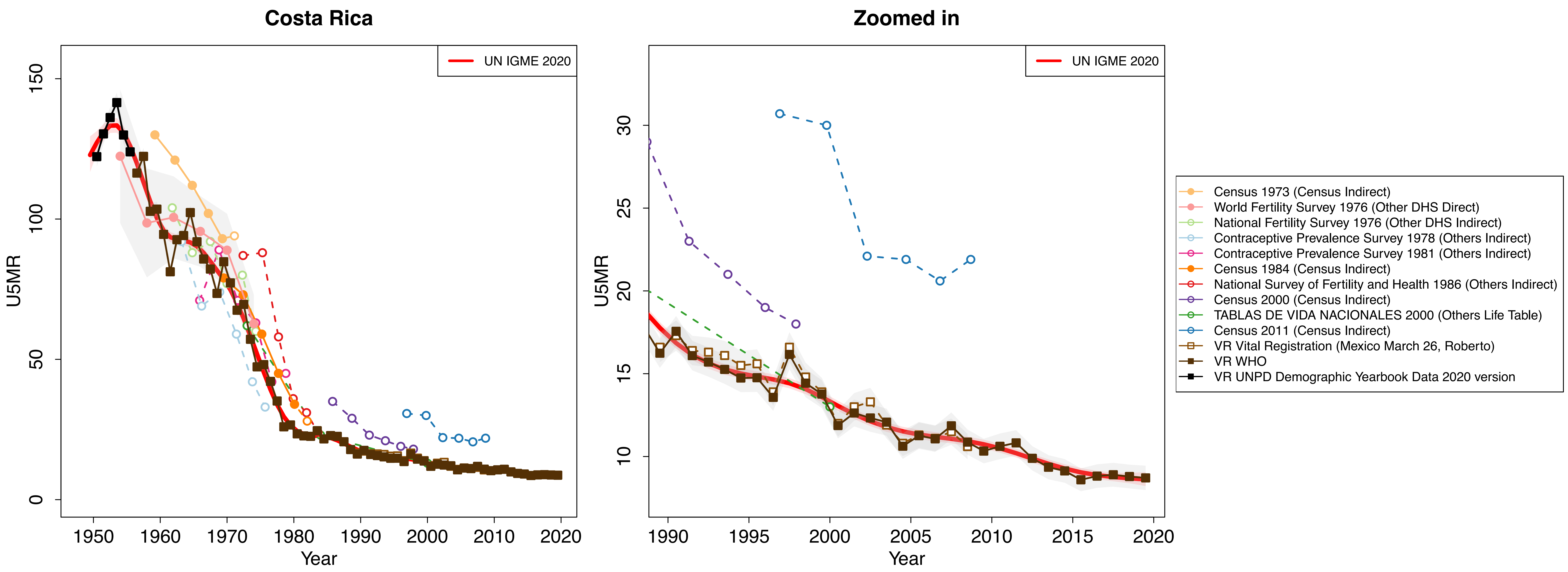
Costa Rica
Zoomed in
UN IGME 2020
UN IGME 2020
U5MR
U5MR
Year
Year
0
50
100
150
10
15
20
25
30
1950
1960
1970
1980
1990
2000
2010
2020
1990
1995
2000
2005
2010
2015
2020
Census 1973 (Census Indirect)
World Fertility Survey 1976 (Other DHS Direct)
National Fertility Survey 1976 (Other DHS Indirect)
Contraceptive Prevalence Survey 1978 (Others Indirect)
Contraceptive Prevalence Survey 1981 (Others Indirect)
Census 1984 (Census Indirect)
National Survey of Fertility and Health 1986 (Others Indirect)
Census 2000 (Census Indirect)
TABLAS DE VIDA NACIONALES 2000 (Others Life Table)
Census 2011 (Census Indirect)
VR Vital Registration (Mexico March 26, Roberto)
VR WHO
VR UNPD Demographic Yearbook Data 2020 version

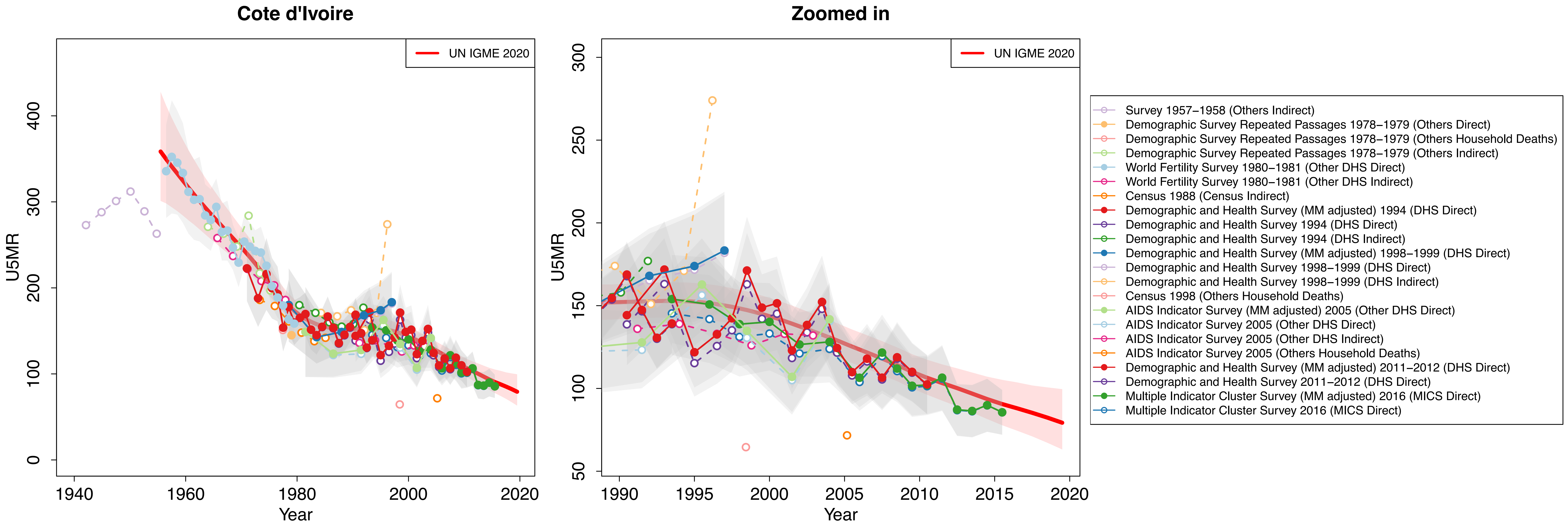

Cote d'Ivoire
Zoomed in
UN IGME 2020
U5MR
Year
Survey 1957–1958 (Others Indirect)
Demographic Survey Repeated Passages 1978–1979 (Others Direct)
Demographic Survey Repeated Passages 1978–1979 (Others Household Deaths)
Demographic Survey Repeated Passages 1978–1979 (Others Indirect)
World Fertility Survey 1980–1981 (Other DHS Direct)
World Fertility Survey 1980–1981 (Other DHS Indirect)
Census 1988 (Census Indirect)
Demographic and Health Survey (MM adjusted) 1994 (DHS Direct)
Demographic and Health Survey 1994 (DHS Direct)
Demographic and Health Survey 1994 (DHS Indirect)
Demographic and Health Survey (MM adjusted) 1998–1999 (DHS Direct)
Demographic and Health Survey 1998–1999 (DHS Direct)
Demographic and Health Survey 1998–1999 (DHS Indirect)
Census 1998 (Others Household Deaths)
AIDS Indicator Survey (MM adjusted) 2005 (Other DHS Direct)
AIDS Indicator Survey 2005 (Other DHS Direct)
AIDS Indicator Survey 2005 (Other DHS Indirect)
AIDS Indicator Survey 2005 (Others Household Deaths)
Demographic and Health Survey (MM adjusted) 2011–2012 (DHS Direct)
Demographic and Health Survey 2011–2012 (DHS Direct)
Multiple Indicator Cluster Survey (MM adjusted) 2016 (MICS Direct)
Multiple Indicator Cluster Survey 2016 (MICS Direct)

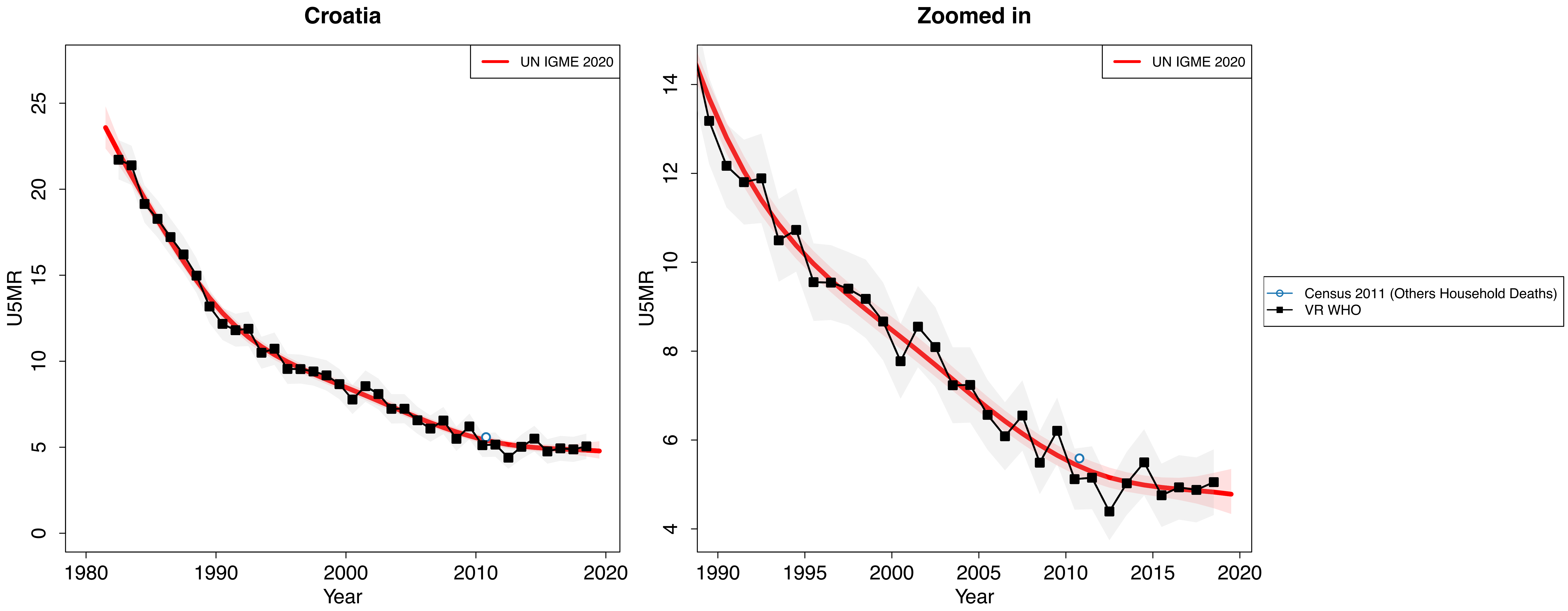

Croatia
UN IGME 2020
U5MR
0
5
10
15
20
25
1980
1990
2000
2010
2020
Year
Zoomed in
UN IGME 2020
U5MR
4
6
8
10
12
14
1990
1995
2000
2005
2010
2015
2020
Year
Census 2011 (Others Household Deaths)
VR WHO

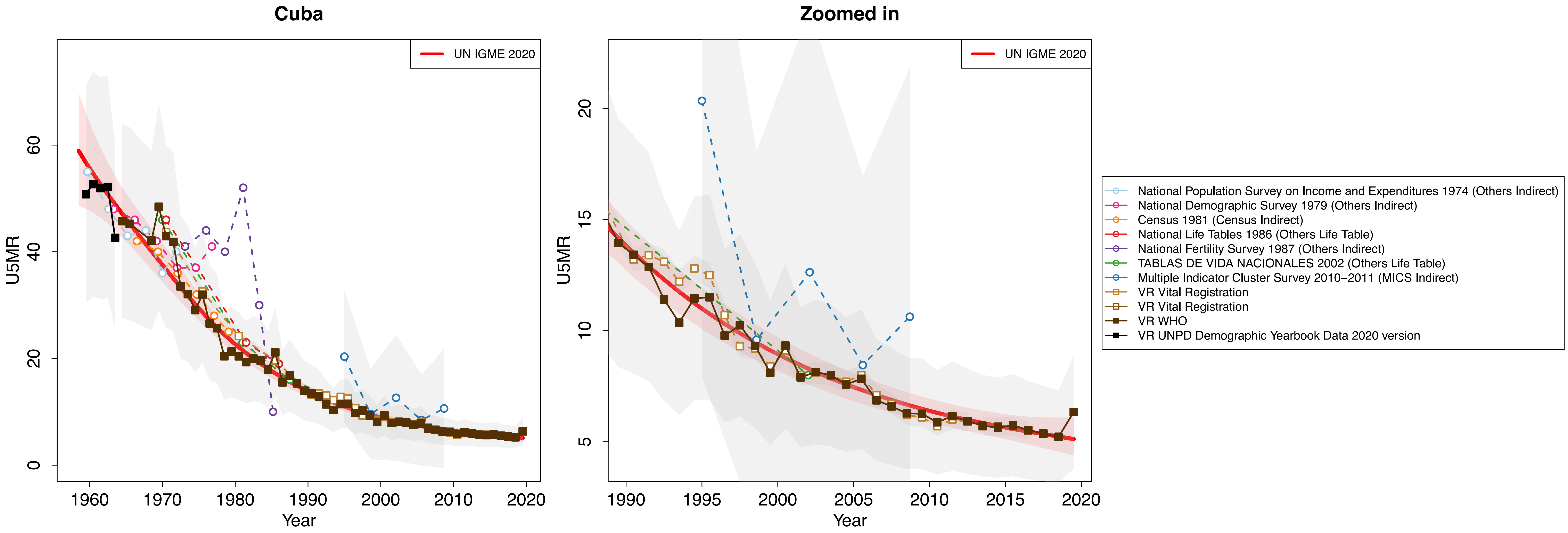

Cuba
Zoomed in
UN IGME 2020
U5MR
Year
National Population Survey on Income and Expenditures 1974 (Others Indirect)
National Demographic Survey 1979 (Others Indirect)
Census 1981 (Census Indirect)
National Life Tables 1986 (Others Life Table)
National Fertility Survey 1987 (Others Indirect)
TABLAS DE VIDA NACIONALES 2002 (Others Life Table)
Multiple Indicator Cluster Survey 2010–2011 (MICS Indirect)
VR Vital Registration
VR Vital Registration
VR WHO
VR UNPD Demographic Yearbook Data 2020 version

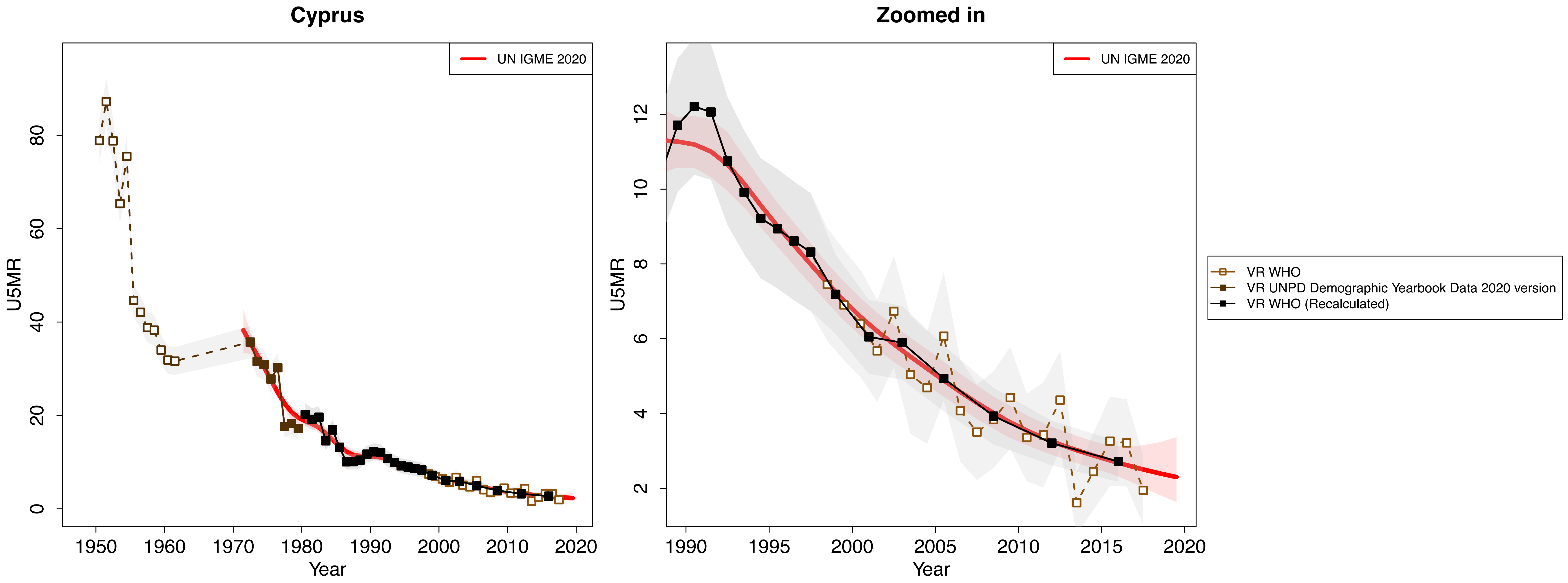

Cyprus
Zoomed in
UN IGME 2020
UN IGME 2020
U5MR
U5MR
Year
Year
0
20
40
60
80
2
4
6
8
10
12
1950
1960
1970
1980
1990
2000
2010
2020
1990
1995
2000
2005
2010
2015
2020
VR WHO
VR UNPD Demographic Yearbook Data 2020 version
VR WHO (Recalculated)

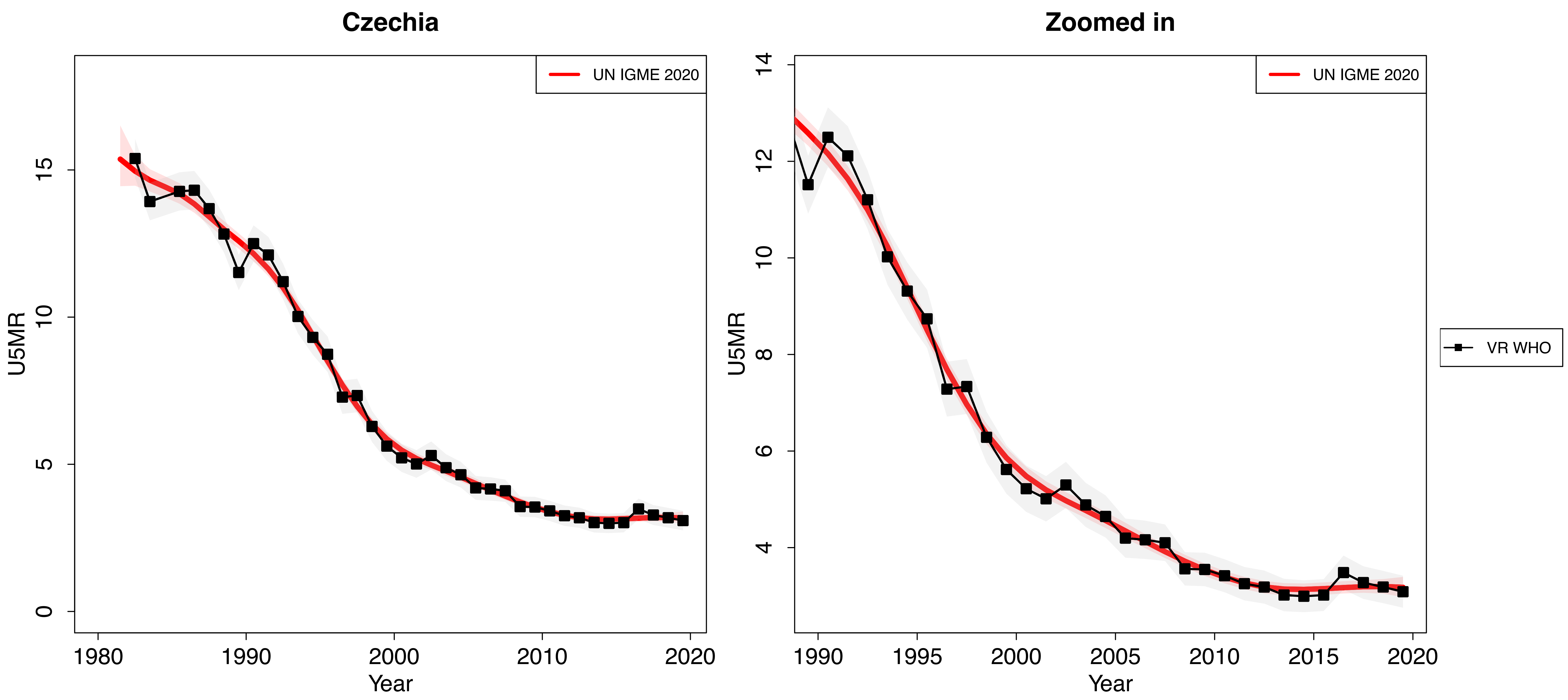
Czechia
Zoomed in
UN IGME 2020
UN IGME 2020
VR WHO
U5MR
U5MR
Year
Year

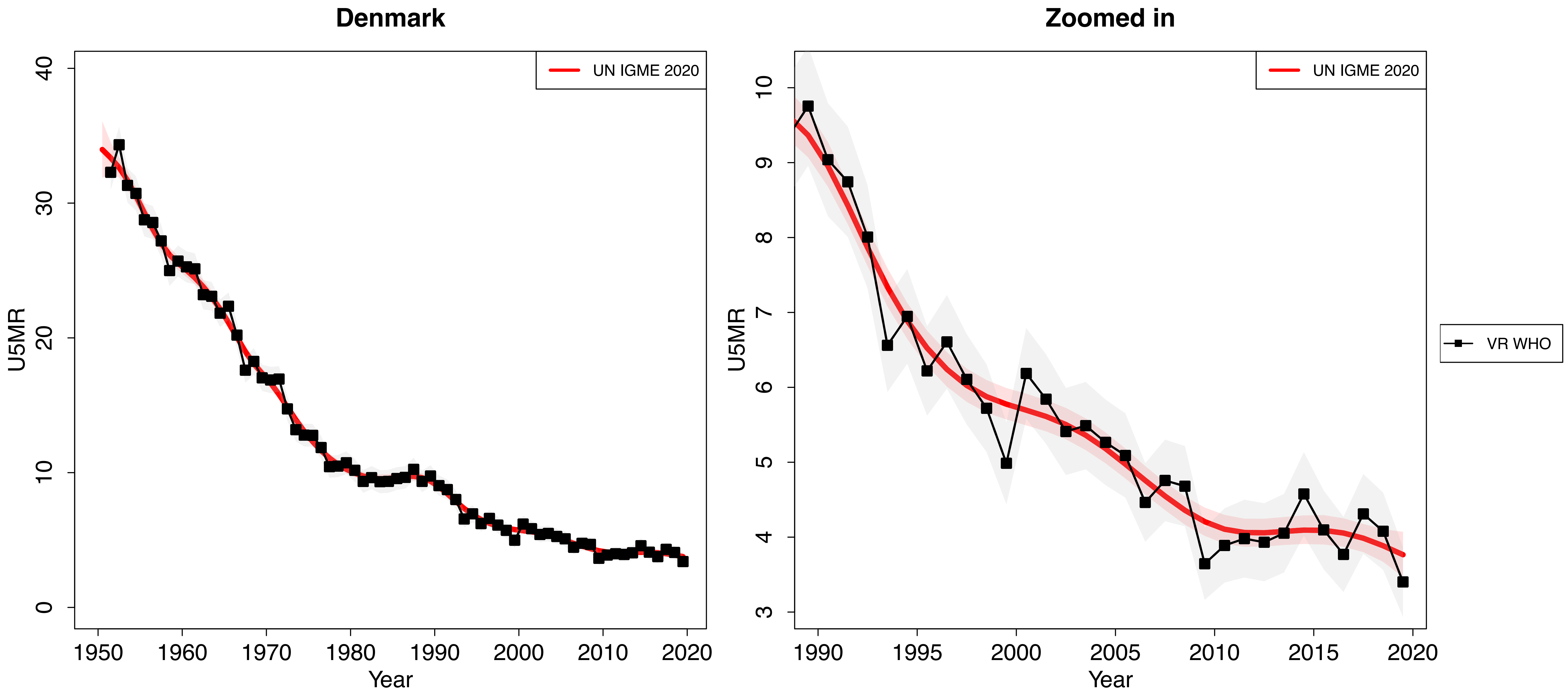
Denmark
Zoomed in
UN IGME 2020
UN IGME 2020
VR WHO
U5MR
U5MR
Year
Year
0
10
20
30
40
1950
1960
1970
1980
1990
2000
2010
2020
3
4
5
6
7
8
9
10
1990
1995
2000
2005
2010
2015
2020

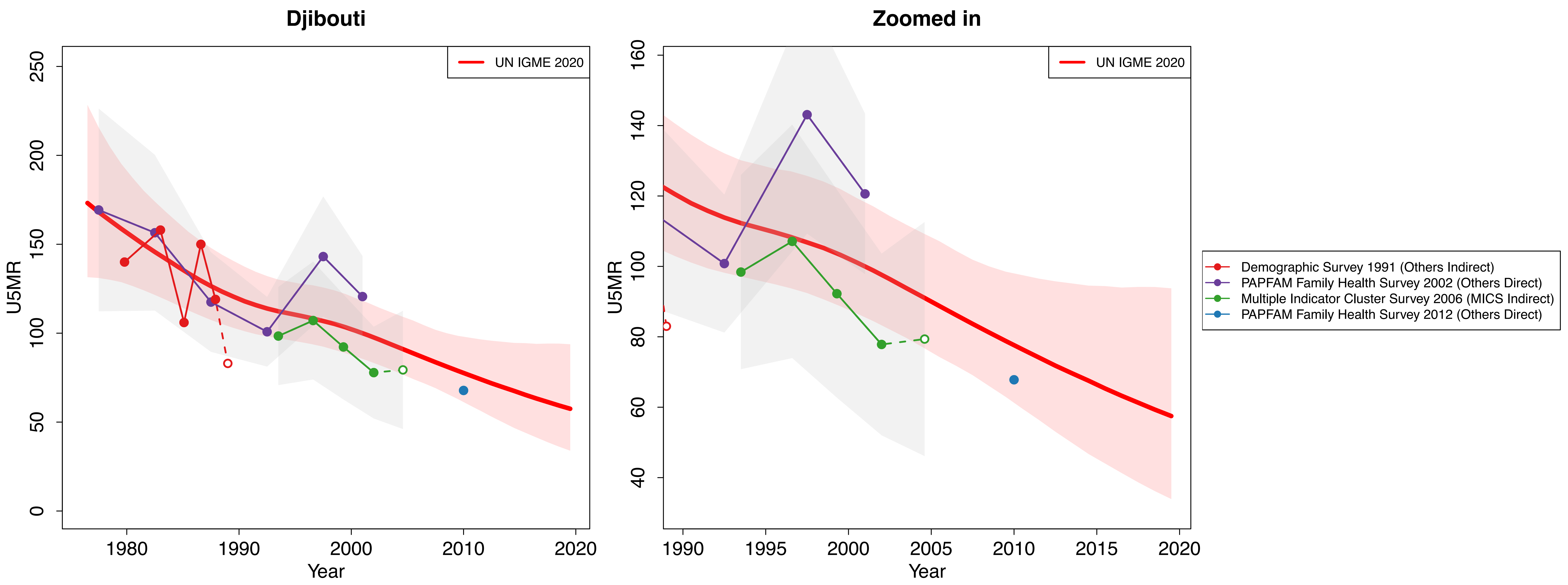

Djibouti
Zoomed in
UN IGME 2020
UN IGME 2020
U5MR
U5MR
Year
Year
0
50
100
150
200
250
1980
1990
2000
2010
2020
40
60
80
100
120
140
160
1990
1995
2000
2005
2010
2015
2020
Demographic Survey 1991 (Others Indirect)
PAPFAM Family Health Survey 2002 (Others Direct)
Multiple Indicator Cluster Survey 2006 (MICS Indirect)
PAPFAM Family Health Survey 2012 (Others Direct)

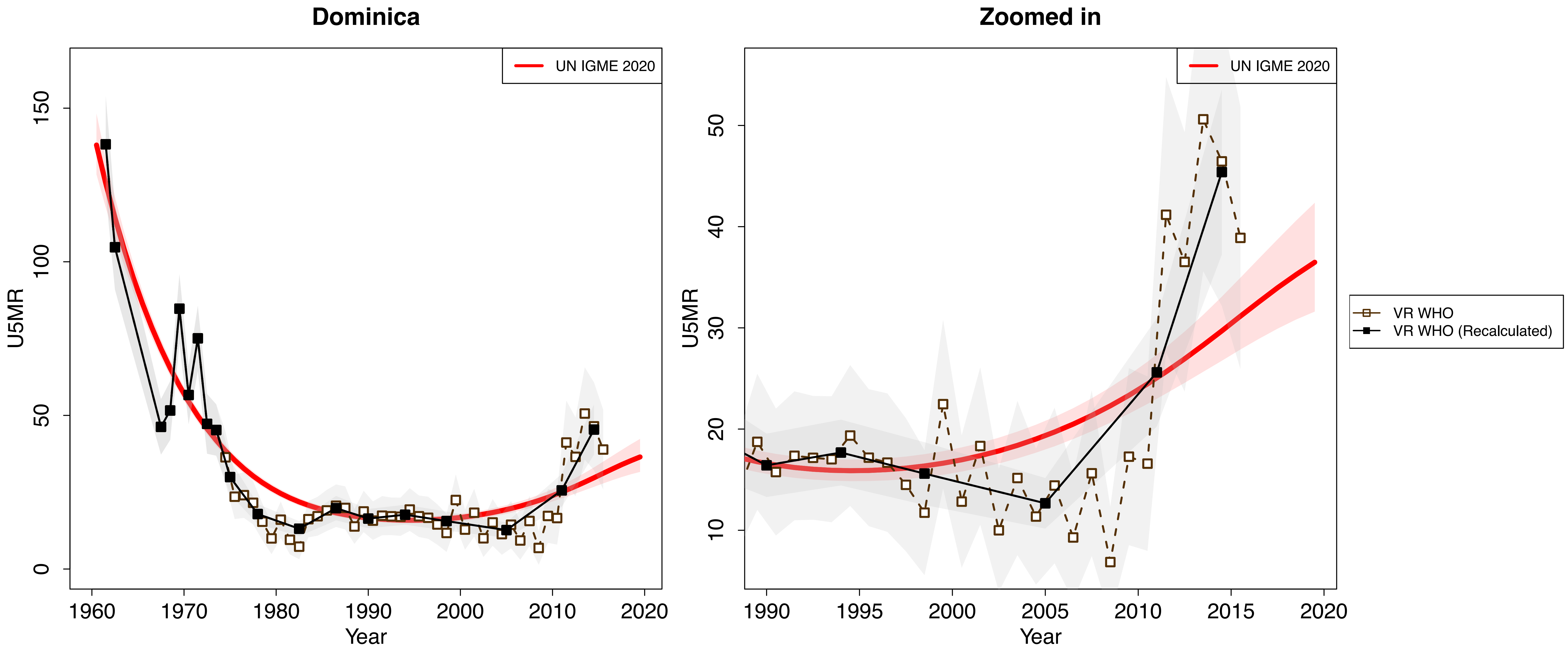

Dominica
Zoomed in
UN IGME 2020
UN IGME 2020
U5MR
U5MR
Year
Year
0
50
100
150
10
20
30
40
50
1960
1970
1980
1990
2000
2010
2020
1990
1995
2000
2005
2010
2015
2020
VR WHO
VR WHO (Recalculated)

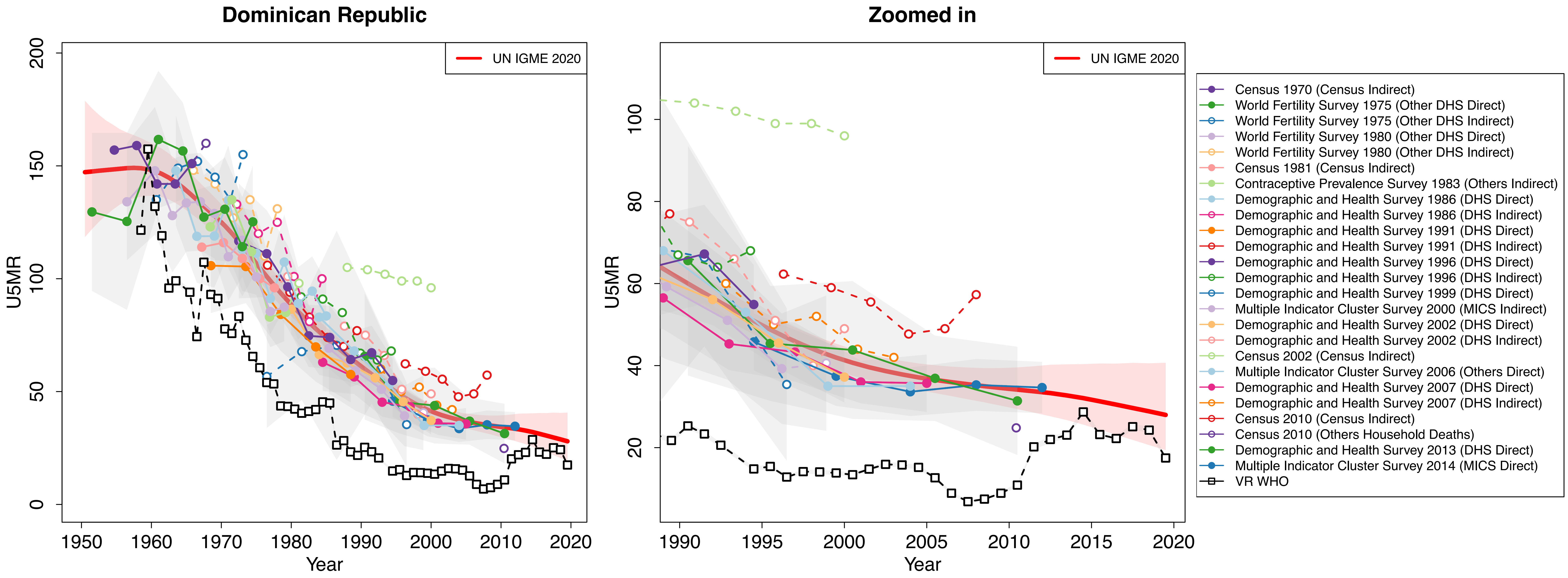

Dominican Republic
Zoomed in
UN IGME 2020
U5MR
Year
0
50
100
150
200
1950
1960
1970
1980
1990
2000
2010
2020
20
40
60
80
100
1990
1995
2000
2005
2010
2015
2020
Census 1970 (Census Indirect)
World Fertility Survey 1975 (Other DHS Direct)
World Fertility Survey 1975 (Other DHS Indirect)
World Fertility Survey 1980 (Other DHS Direct)
World Fertility Survey 1980 (Other DHS Indirect)
Census 1981 (Census Indirect)
Contraceptive Prevalence Survey 1983 (Others Indirect)
Demographic and Health Survey 1986 (DHS Direct)
Demographic and Health Survey 1986 (DHS Indirect)
Demographic and Health Survey 1991 (DHS Direct)
Demographic and Health Survey 1991 (DHS Indirect)
Demographic and Health Survey 1996 (DHS Direct)
Demographic and Health Survey 1996 (DHS Indirect)
Demographic and Health Survey 1999 (DHS Direct)
Multiple Indicator Cluster Survey 2000 (MICS Indirect)
Demographic and Health Survey 2002 (DHS Direct)
Demographic and Health Survey 2002 (DHS Indirect)
Census 2002 (Census Indirect)
Multiple Indicator Cluster Survey 2006 (Others Direct)
Demographic and Health Survey 2007 (DHS Direct)
Demographic and Health Survey 2007 (DHS Indirect)
Census 2010 (Census Indirect)
Census 2010 (Others Household Deaths)
Demographic and Health Survey 2013 (DHS Direct)
Multiple Indicator Cluster Survey 2014 (MICS Direct)
VR WHO

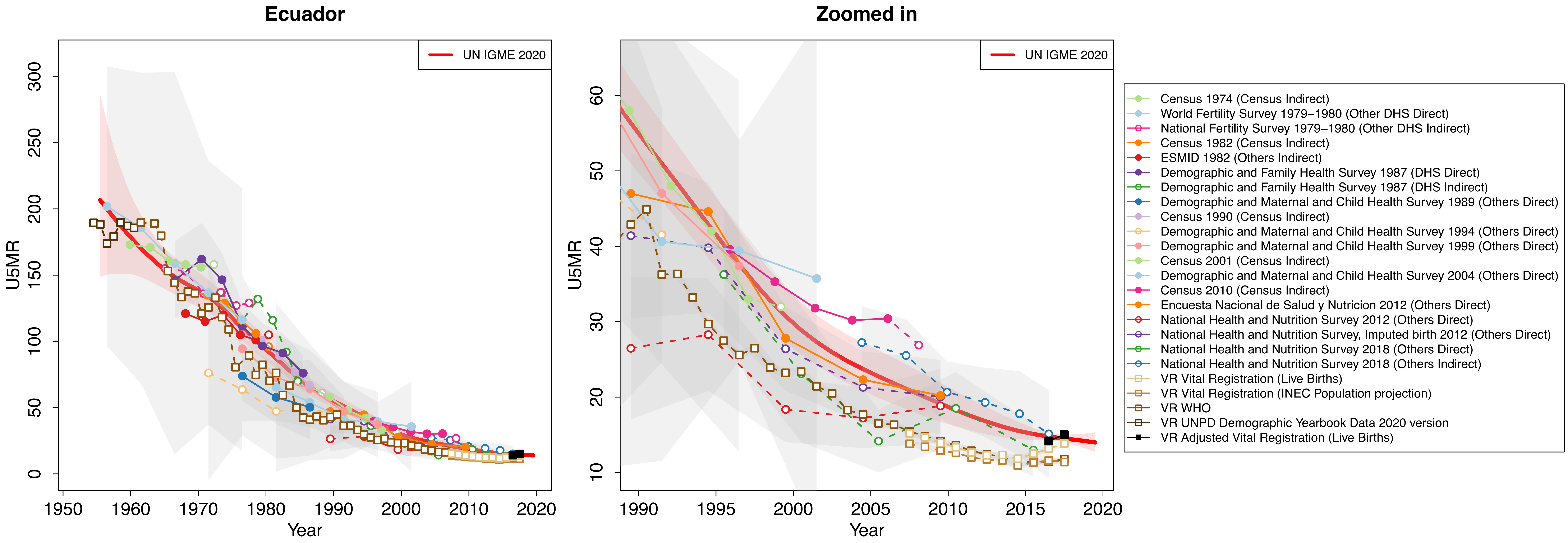

Ecuador
Zoomed in
UN IGME 2020
U5MR
Year
Census 1974 (Census Indirect)
World Fertility Survey 1979–1980 (Other DHS Direct)
National Fertility Survey 1979–1980 (Other DHS Indirect)
Census 1982 (Census Indirect)
ESMID 1982 (Others Indirect)
Demographic and Family Health Survey 1987 (DHS Direct)
Demographic and Family Health Survey 1987 (DHS Indirect)
Demographic and Maternal and Child Health Survey 1989 (Others Direct)
Census 1990 (Census Indirect)
Demographic and Maternal and Child Health Survey 1994 (Others Direct)
Demographic and Maternal and Child Health Survey 1999 (Others Direct)
Census 2001 (Census Indirect)
Demographic and Maternal and Child Health Survey 2004 (Others Direct)
Census 2010 (Census Indirect)
Encuesta Nacional de Salud y Nutricion 2012 (Others Direct)
National Health and Nutrition Survey 2012 (Others Direct)
National Health and Nutrition Survey, Imputed birth 2012 (Others Direct)
National Health and Nutrition Survey 2018 (Others Direct)
National Health and Nutrition Survey 2018 (Others Indirect)
VR Vital Registration (Live Births)
VR Vital Registration (INEC Population projection)
VR WHO
VR UNPD Demographic Yearbook Data 2020 version
VR Adjusted Vital Registration (Live Births)

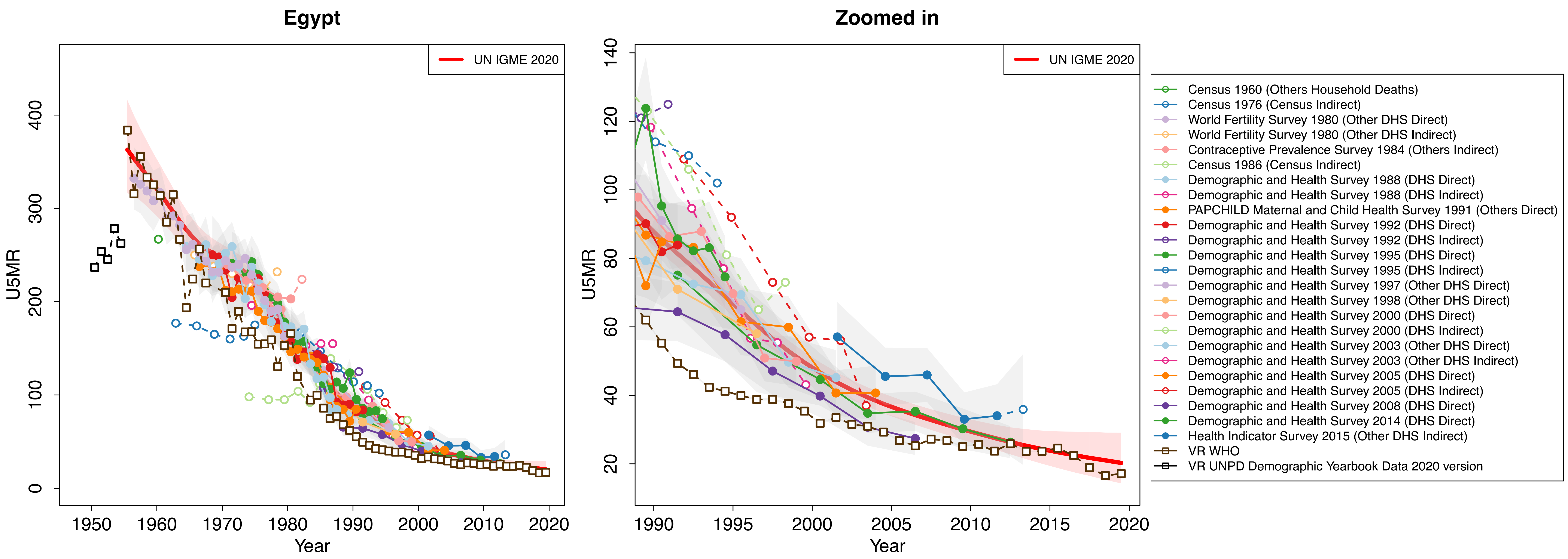

Egypt
Zoomed in
UN IGME 2020
U5MR
Year
Census 1960 (Others Household Deaths)
Census 1976 (Census Indirect)
World Fertility Survey 1980 (Other DHS Direct)
World Fertility Survey 1980 (Other DHS Indirect)
Contraceptive Prevalence Survey 1984 (Others Indirect)
Census 1986 (Census Indirect)
Demographic and Health Survey 1988 (DHS Direct)
Demographic and Health Survey 1988 (DHS Indirect)
PAPCHILD Maternal and Child Health Survey 1991 (Others Direct)
Demographic and Health Survey 1992 (DHS Direct)
Demographic and Health Survey 1992 (DHS Indirect)
Demographic and Health Survey 1995 (DHS Direct)
Demographic and Health Survey 1995 (DHS Indirect)
Demographic and Health Survey 1997 (Other DHS Direct)
Demographic and Health Survey 1998 (Other DHS Direct)
Demographic and Health Survey 2000 (DHS Direct)
Demographic and Health Survey 2000 (DHS Indirect)
Demographic and Health Survey 2003 (Other DHS Direct)
Demographic and Health Survey 2003 (Other DHS Indirect)
Demographic and Health Survey 2005 (DHS Direct)
Demographic and Health Survey 2005 (DHS Indirect)
Demographic and Health Survey 2008 (DHS Direct)
Demographic and Health Survey 2014 (DHS Direct)
Health Indicator Survey 2015 (Other DHS Indirect)
VR WHO
VR UNPD Demographic Yearbook Data 2020 version

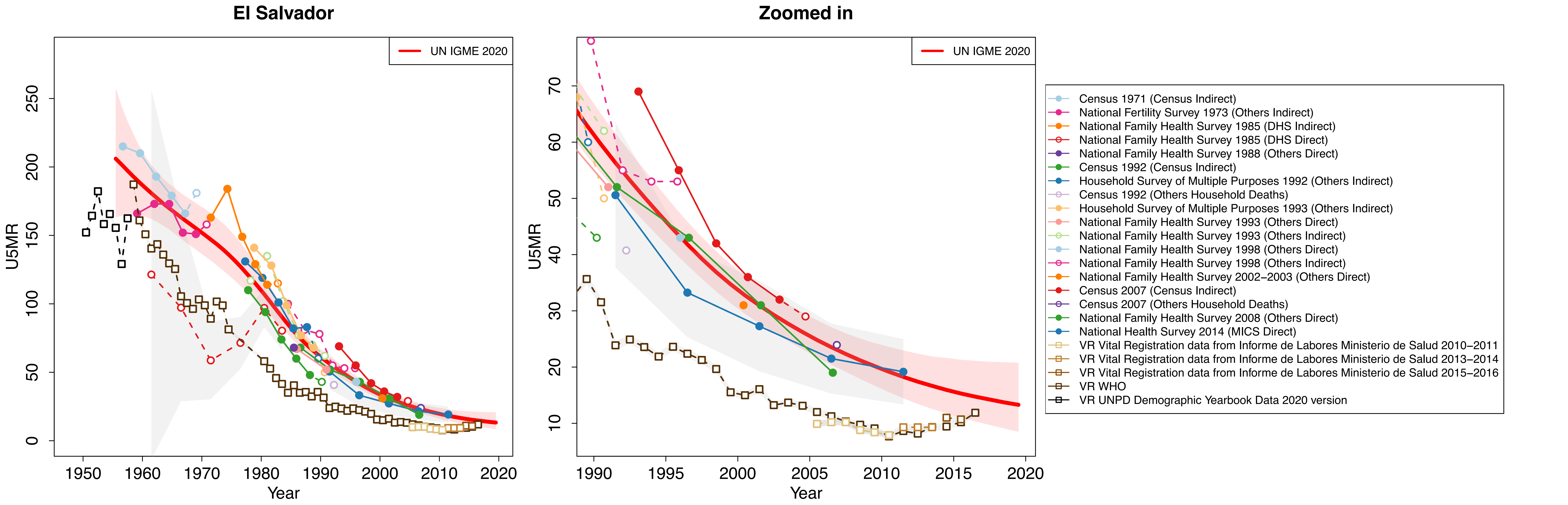
El Salvador
Zoomed in
UN IGME 2020
U5MR
Year
Census 1971 (Census Indirect)
National Fertility Survey 1973 (Others Indirect)
National Family Health Survey 1985 (DHS Indirect)
National Family Health Survey 1985 (DHS Direct)
National Family Health Survey 1988 (Others Direct)
Census 1992 (Census Indirect)
Household Survey of Multiple Purposes 1992 (Others Indirect)
Census 1992 (Others Household Deaths)
Household Survey of Multiple Purposes 1993 (Others Indirect)
National Family Health Survey 1993 (Others Direct)
National Family Health Survey 1993 (Others Indirect)
National Family Health Survey 1998 (Others Direct)
National Family Health Survey 1998 (Others Indirect)
National Family Health Survey 2002–2003 (Others Direct)
Census 2007 (Census Indirect)
Census 2007 (Others Household Deaths)
National Family Health Survey 2008 (Others Direct)
National Health Survey 2014 (MICS Direct)
VR Vital Registration data from Informe de Labores Ministerio de Salud 2010–2011
VR Vital Registration data from Informe de Labores Ministerio de Salud 2013–2014
VR Vital Registration data from Informe de Labores Ministerio de Salud 2015–2016
VR WHO
VR UNPD Demographic Yearbook Data 2020 version

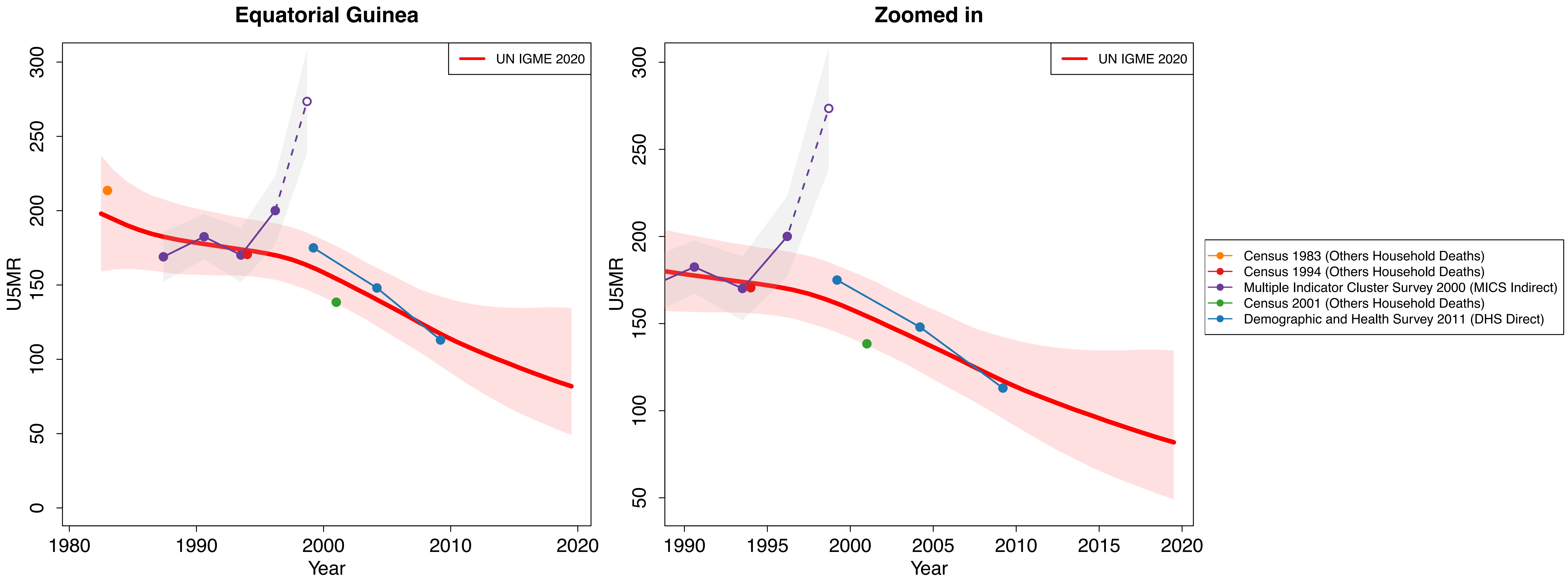

Equatorial Guinea
Zoomed in
UN IGME 2020
UN IGME 2020
U5MR
U5MR
Year
Year
0
50
100
150
200
250
300
1980
1990
2000
2010
2020
1995
2005
2015
Census 1983 (Others Household Deaths)
Census 1994 (Others Household Deaths)
Multiple Indicator Cluster Survey 2000 (MICS Indirect)
Census 2001 (Others Household Deaths)
Demographic and Health Survey 2011 (DHS Direct)

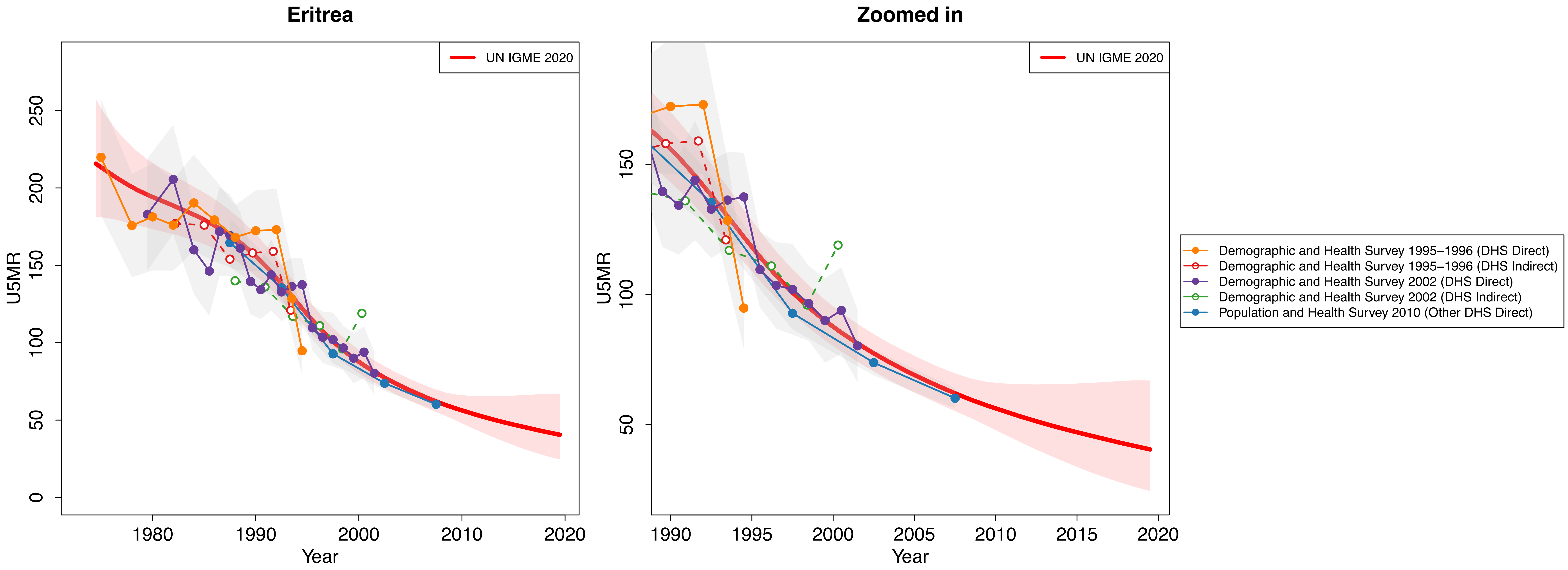

Eritrea
Zoomed in
UN IGME 2020
U5MR
Year
Demographic and Health Survey 1995–1996 (DHS Direct)
Demographic and Health Survey 1995–1996 (DHS Indirect)
Demographic and Health Survey 2002 (DHS Direct)
Demographic and Health Survey 2002 (DHS Indirect)
Population and Health Survey 2010 (Other DHS Direct)

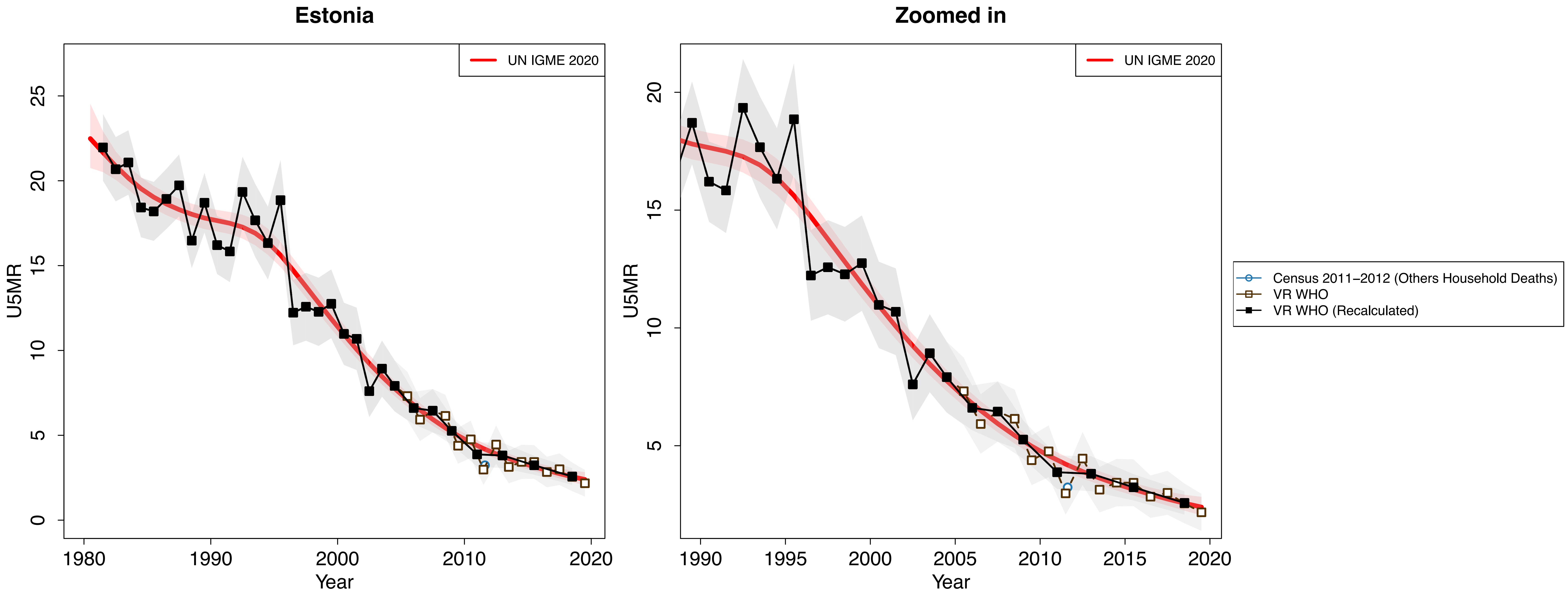

Estonia
Zoomed in
UN IGME 2020
U5MR
Year
Census 2011–2012 (Others Household Deaths)
VR WHO
VR WHO (Recalculated)

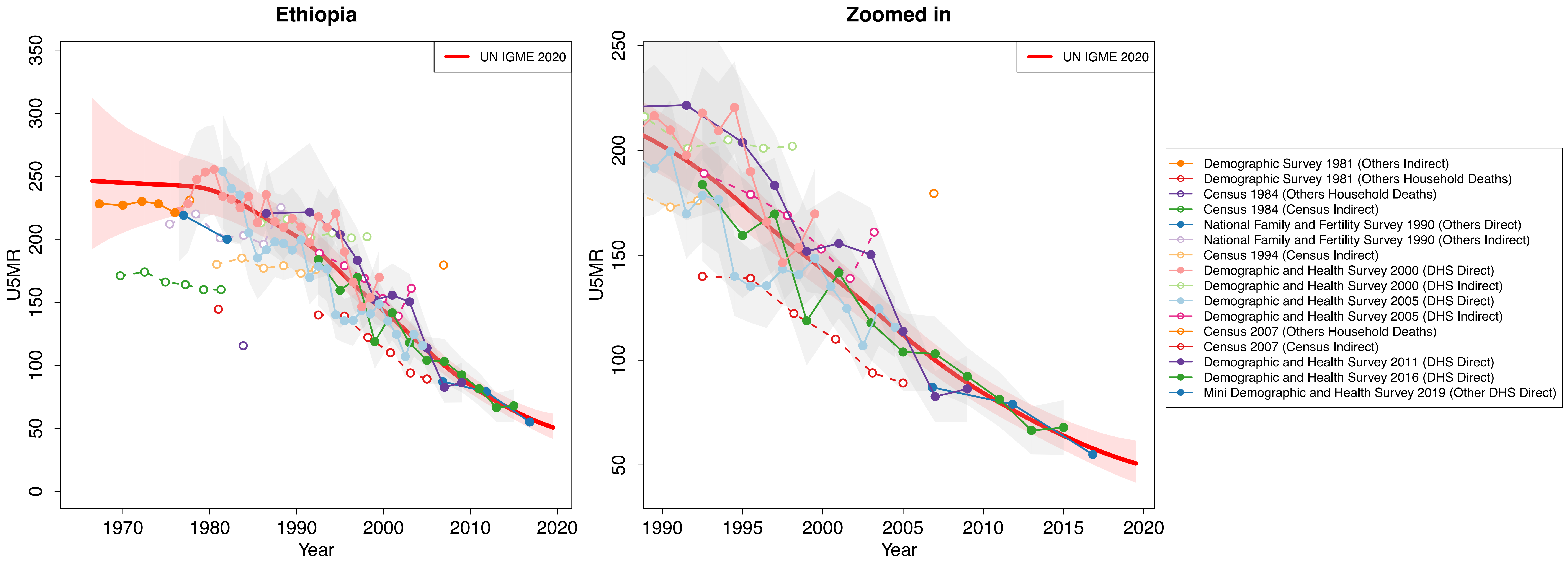
Ethiopia
Zoomed in
UN IGME 2020
U5MR
Year
Demographic Survey 1981 (Others Indirect)
Demographic Survey 1981 (Others Household Deaths)
Census 1984 (Others Household Deaths)
Census 1984 (Census Indirect)
National Family and Fertility Survey 1990 (Others Direct)
National Family and Fertility Survey 1990 (Others Indirect)
Census 1994 (Census Indirect)
Demographic and Health Survey 2000 (DHS Direct)
Demographic and Health Survey 2000 (DHS Indirect)
Demographic and Health Survey 2005 (DHS Direct)
Demographic and Health Survey 2005 (DHS Indirect)
Census 2007 (Others Household Deaths)
Census 2007 (Census Indirect)
Demographic and Health Survey 2011 (DHS Direct)
Demographic and Health Survey 2016 (DHS Direct)
Mini Demographic and Health Survey 2019 (Other DHS Direct)

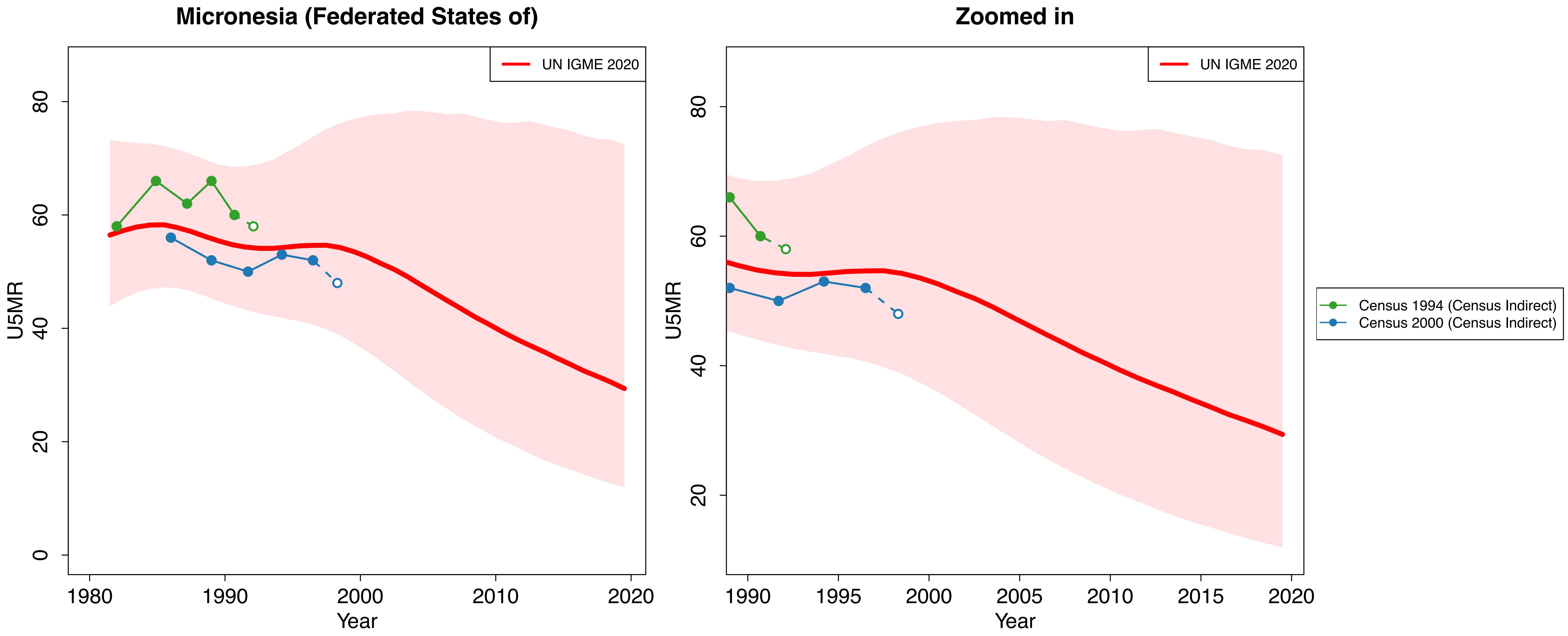
Micronesia (Federated States of)
Zoomed in
UN IGME 2020
UN IGME 2020
U5MR
U5MR
Year
Year
0
20
40
60
80
1980
1990
2000
2010
2020
20
40
60
80
1990
1995
2000
2005
2010
2015
2020
Census 1994 (Census Indirect)
Census 2000 (Census Indirect)

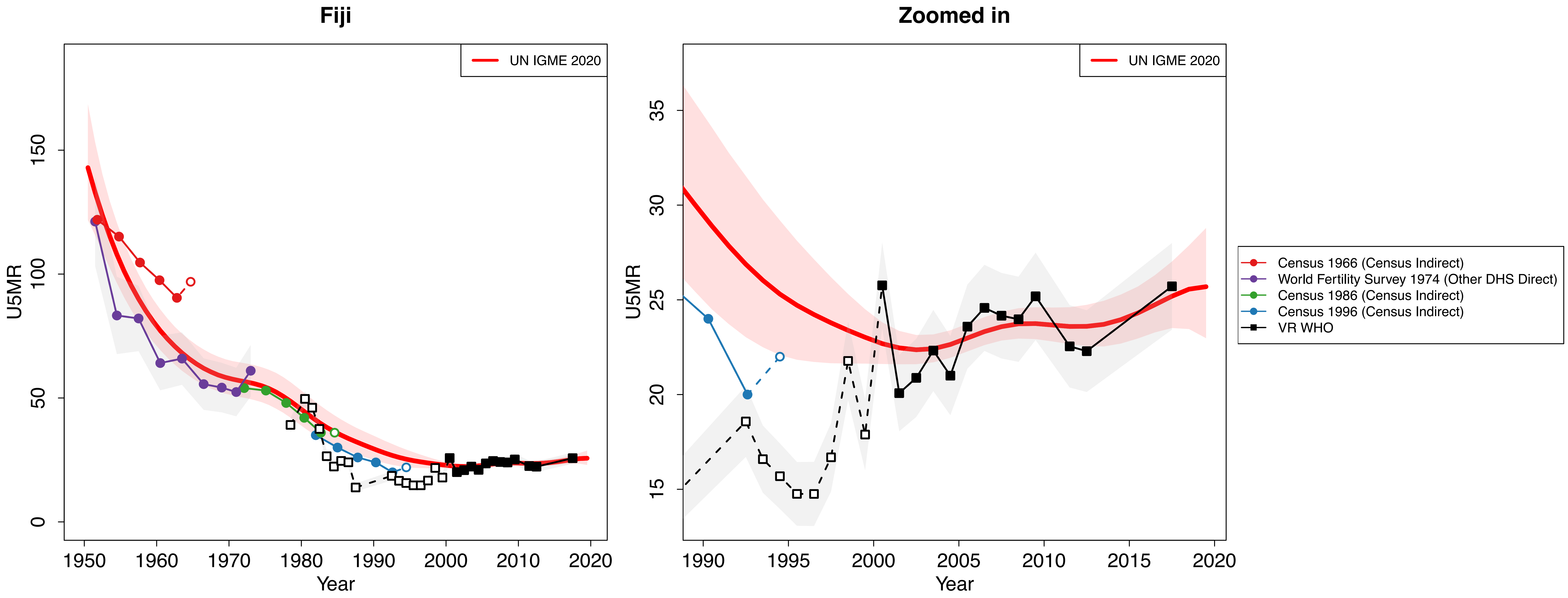
Fiji
Zoomed in
UN IGME 2020
UN IGME 2020
U5MR
U5MR
Year
Year
0
50
100
150
15
20
25
30
35
1950
1960
1970
1980
1990
2000
2010
2020
1990
1995
2000
2005
2010
2015
2020
Census 1966 (Census Indirect)
World Fertility Survey 1974 (Other DHS Direct)
Census 1986 (Census Indirect)
Census 1996 (Census Indirect)
VR WHO

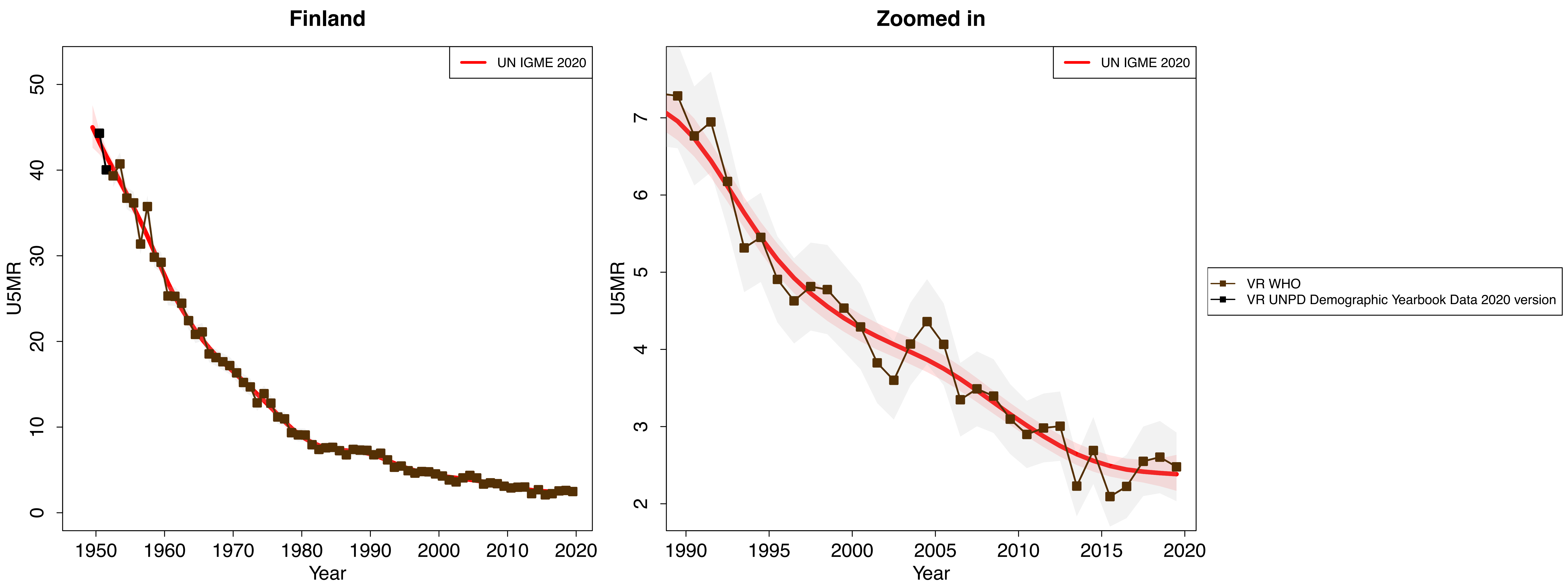
Finland
Zoomed in
UN IGME 2020
UN IGME 2020
U5MR
U5MR
Year
Year
0
10
20
30
40
50
1950
1960
1970
1980
1990
2000
2010
2020
2
3
4
5
6
7
1990
1995
2000
2005
2010
2015
2020
VR WHO
VR UNPD Demographic Yearbook Data 2020 version

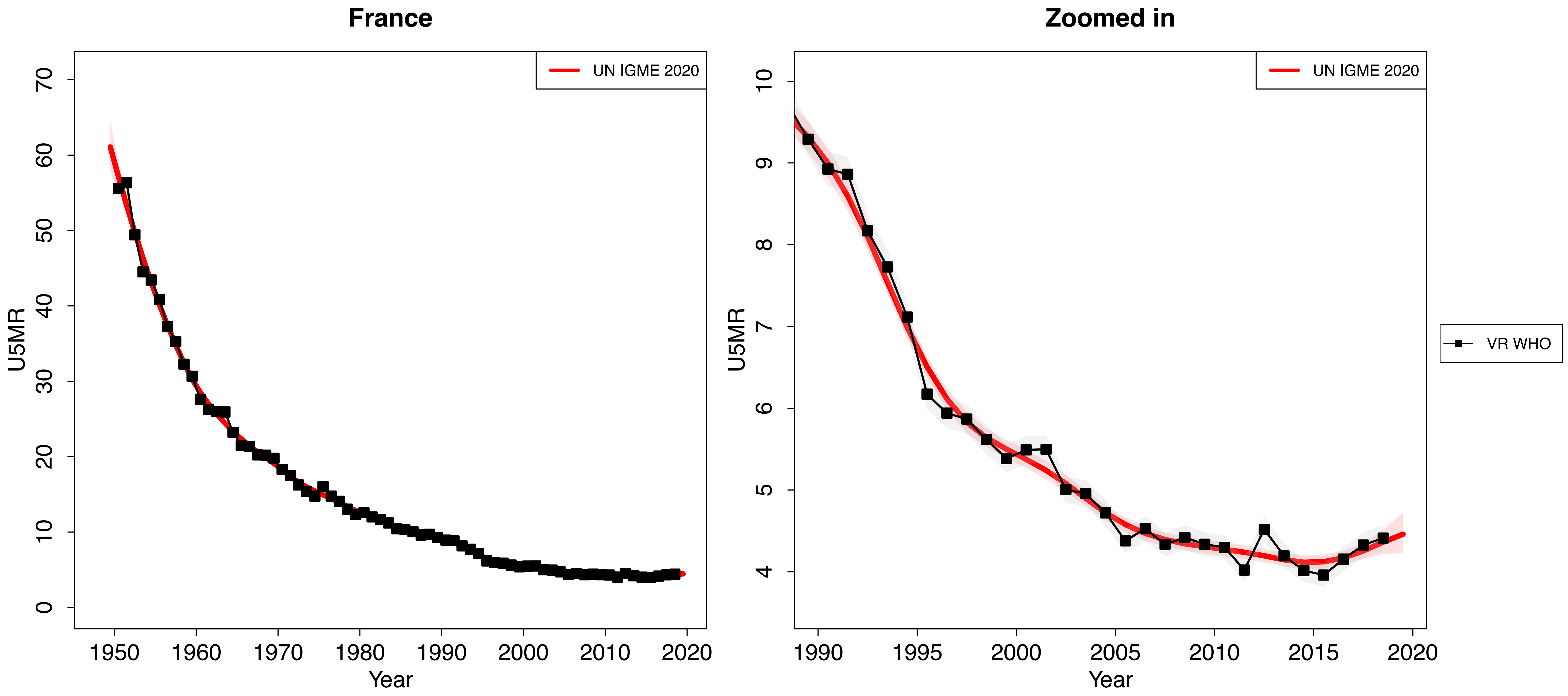
France
Zoomed in
UN IGME 2020
UN IGME 2020
VR WHO
U5MR
U5MR
Year
Year
0
10
20
30
40
50
60
70
1950
1960
1970
1980
1990
2000
2010
2020
4
5
6
7
8
9
10
1990
1995
2000
2005
2010
2015
2020

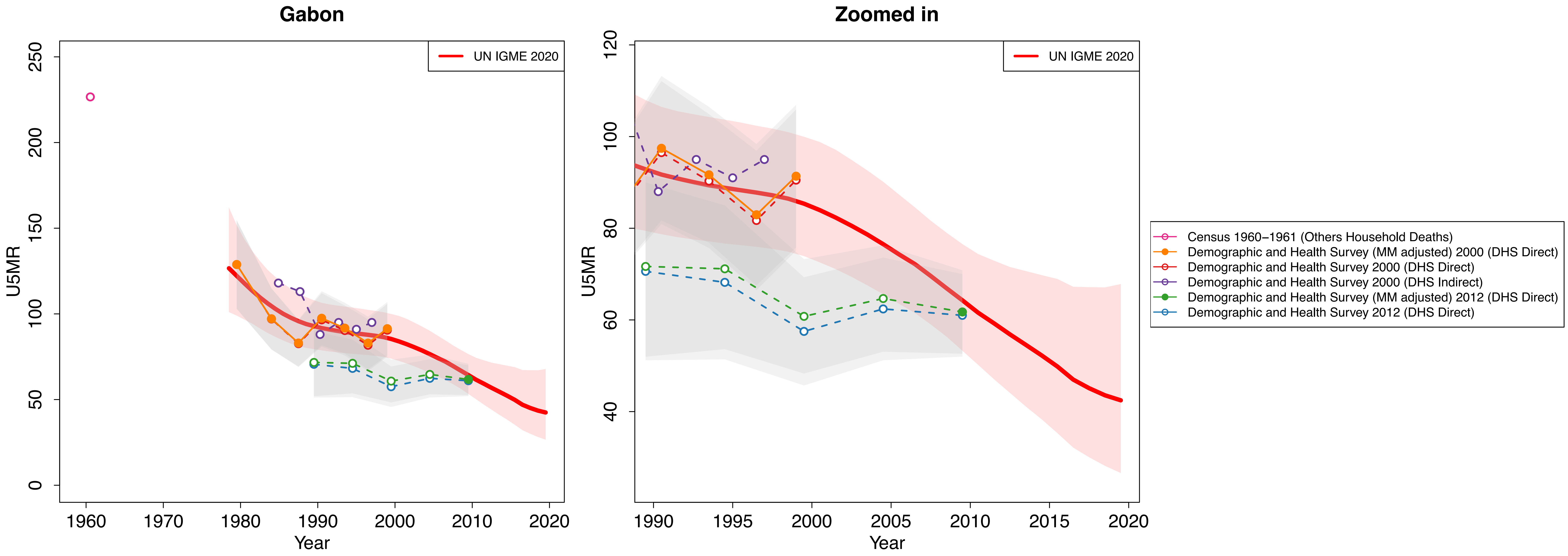
Gabon
Zoomed in
UN IGME 2020
UN IGME 2020
U5MR
Year
Census 1960–1961 (Others Household Deaths)
Demographic and Health Survey (MM adjusted) 2000 (DHS Direct)
Demographic and Health Survey 2000 (DHS Direct)
Demographic and Health Survey 2000 (DHS Indirect)
Demographic and Health Survey (MM adjusted) 2012 (DHS Direct)
Demographic and Health Survey 2012 (DHS Direct)

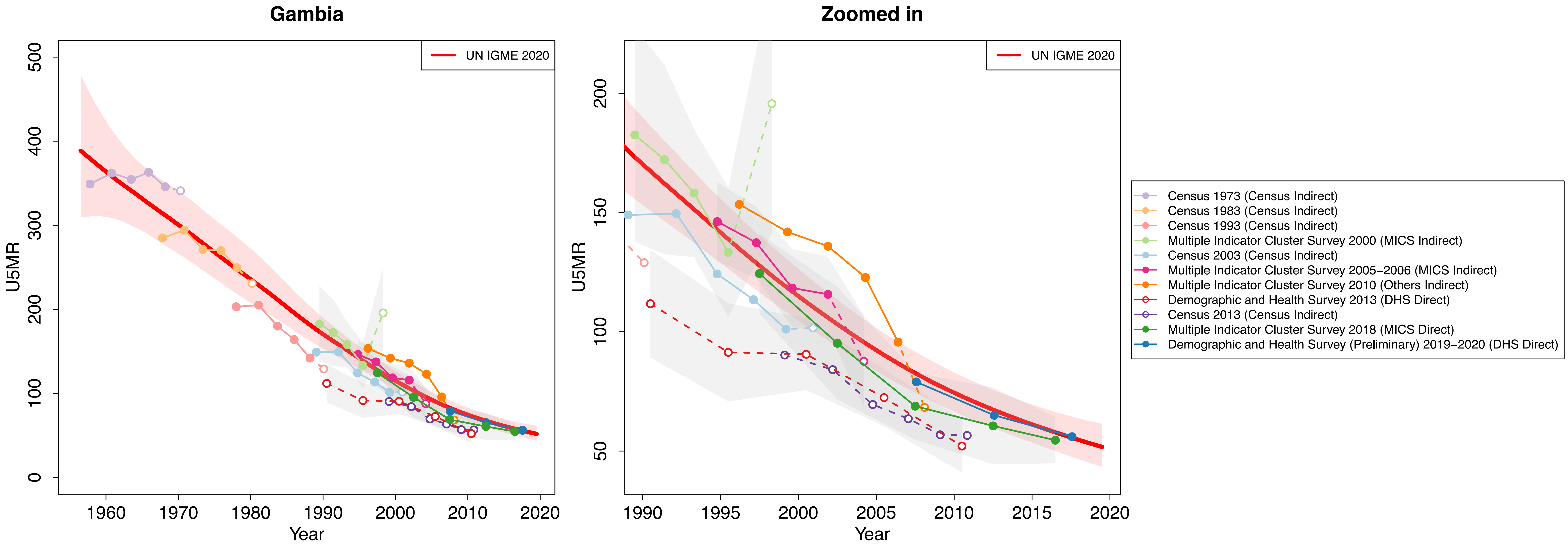
Gambia
Zoomed in
UN IGME 2020
U5MR
Year
Census 1973 (Census Indirect)
Census 1983 (Census Indirect)
Census 1993 (Census Indirect)
Multiple Indicator Cluster Survey 2000 (MICS Indirect)
Census 2003 (Census Indirect)
Multiple Indicator Cluster Survey 2005–2006 (MICS Indirect)
Multiple Indicator Cluster Survey 2010 (Others Indirect)
Demographic and Health Survey 2013 (DHS Direct)
Census 2013 (Census Indirect)
Multiple Indicator Cluster Survey 2018 (MICS Direct)
Demographic and Health Survey (Preliminary) 2019–2020 (DHS Direct)

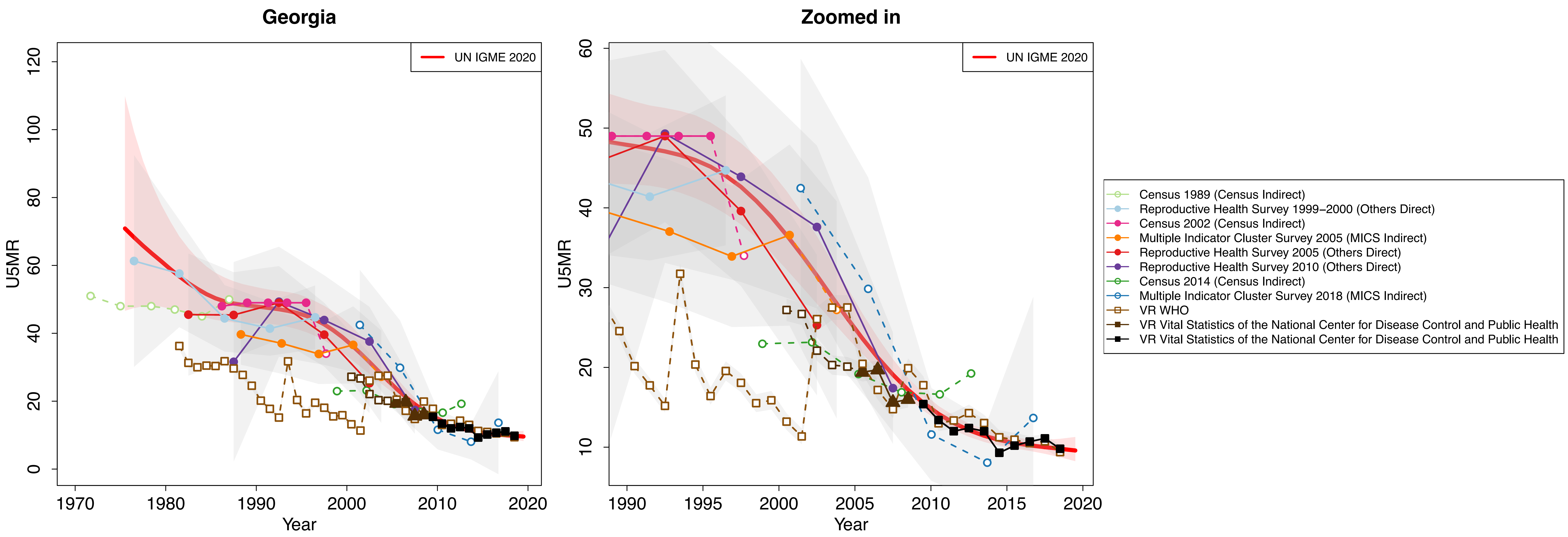

Georgia
Zoomed in
UN IGME 2020
U5MR
Year
Census 1989 (Census Indirect)
Reproductive Health Survey 1999–2000 (Others Direct)
Census 2002 (Census Indirect)
Multiple Indicator Cluster Survey 2005 (MICS Indirect)
Reproductive Health Survey 2005 (Others Direct)
Reproductive Health Survey 2010 (Others Direct)
Census 2014 (Census Indirect)
Multiple Indicator Cluster Survey 2018 (MICS Indirect)
VR WHO
VR Vital Statistics of the National Center for Disease Control and Public Health
VR Vital Statistics of the National Center for Disease Control and Public Health

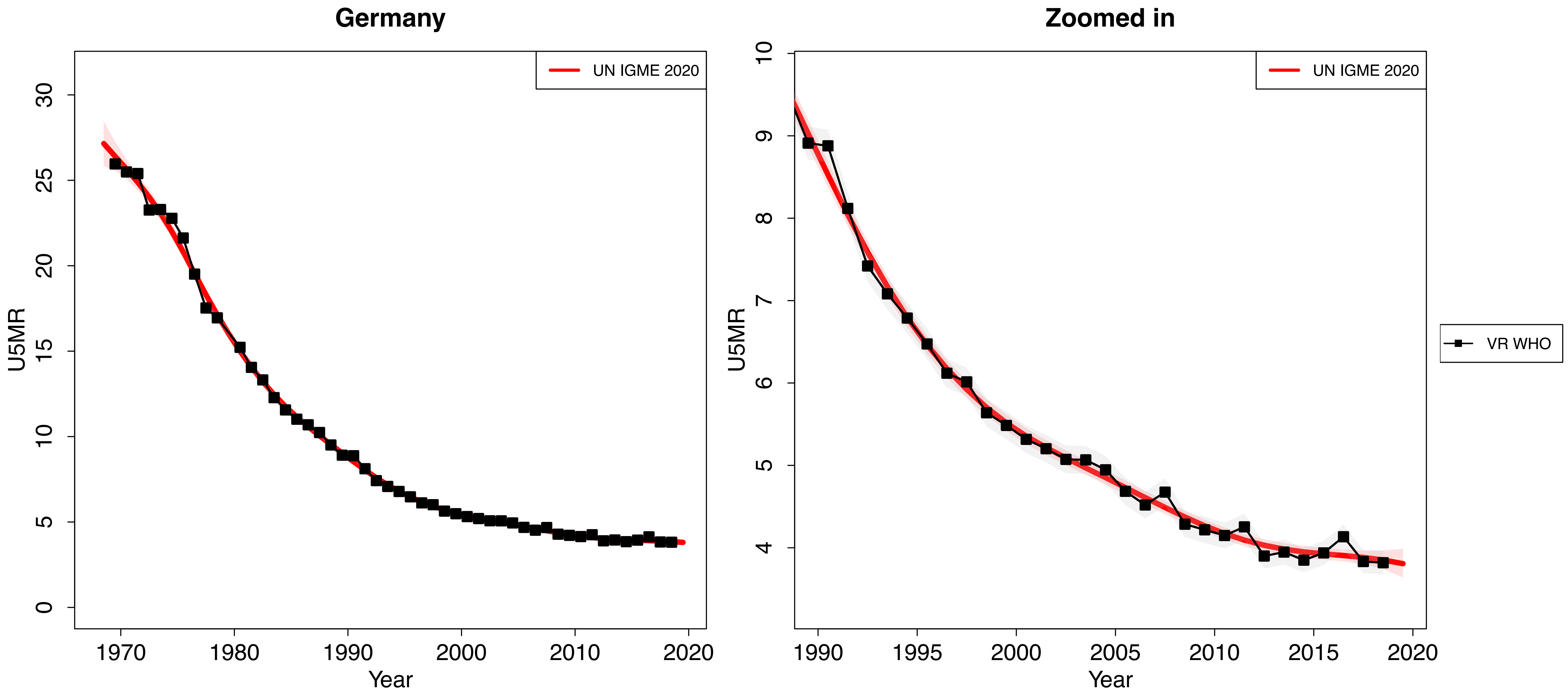

Germany
Zoomed in
UN IGME 2020
UN IGME 2020
VR WHO
U5MR
U5MR
Year
Year
0
5
10
15
20
25
30
4
5
6
7
8
9
10
1970
1980
1990
2000
2010
2020
1990
1995
2000
2005
2010
2015
2020

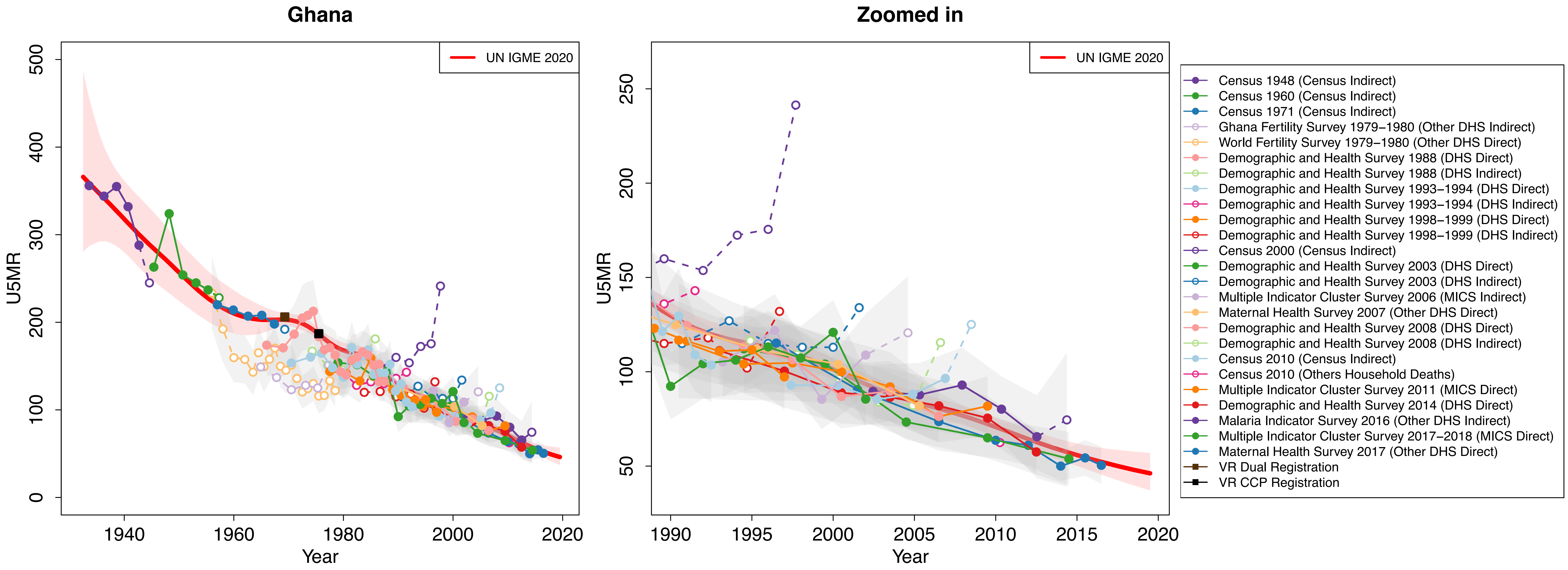

Ghana
Zoomed in
UN IGME 2020
U5MR
Year
Census 1948 (Census Indirect)
Census 1960 (Census Indirect)
Census 1971 (Census Indirect)
Ghana Fertility Survey 1979–1980 (Other DHS Indirect)
World Fertility Survey 1979–1980 (Other DHS Direct)
Demographic and Health Survey 1988 (DHS Direct)
Demographic and Health Survey 1988 (DHS Indirect)
Demographic and Health Survey 1993–1994 (DHS Direct)
Demographic and Health Survey 1993–1994 (DHS Indirect)
Demographic and Health Survey 1998–1999 (DHS Direct)
Demographic and Health Survey 1998–1999 (DHS Indirect)
Census 2000 (Census Indirect)
Demographic and Health Survey 2003 (DHS Direct)
Demographic and Health Survey 2003 (DHS Indirect)
Multiple Indicator Cluster Survey 2006 (MICS Indirect)
Maternal Health Survey 2007 (Other DHS Direct)
Demographic and Health Survey 2008 (DHS Direct)
Demographic and Health Survey 2008 (DHS Indirect)
Census 2010 (Census Indirect)
Census 2010 (Others Household Deaths)
Multiple Indicator Cluster Survey 2011 (MICS Direct)
Demographic and Health Survey 2014 (DHS Direct)
Malaria Indicator Survey 2016 (Other DHS Indirect)
Multiple Indicator Cluster Survey 2017–2018 (MICS Direct)
Maternal Health Survey 2017 (Other DHS Direct)
VR Dual Registration
VR CCP Registration

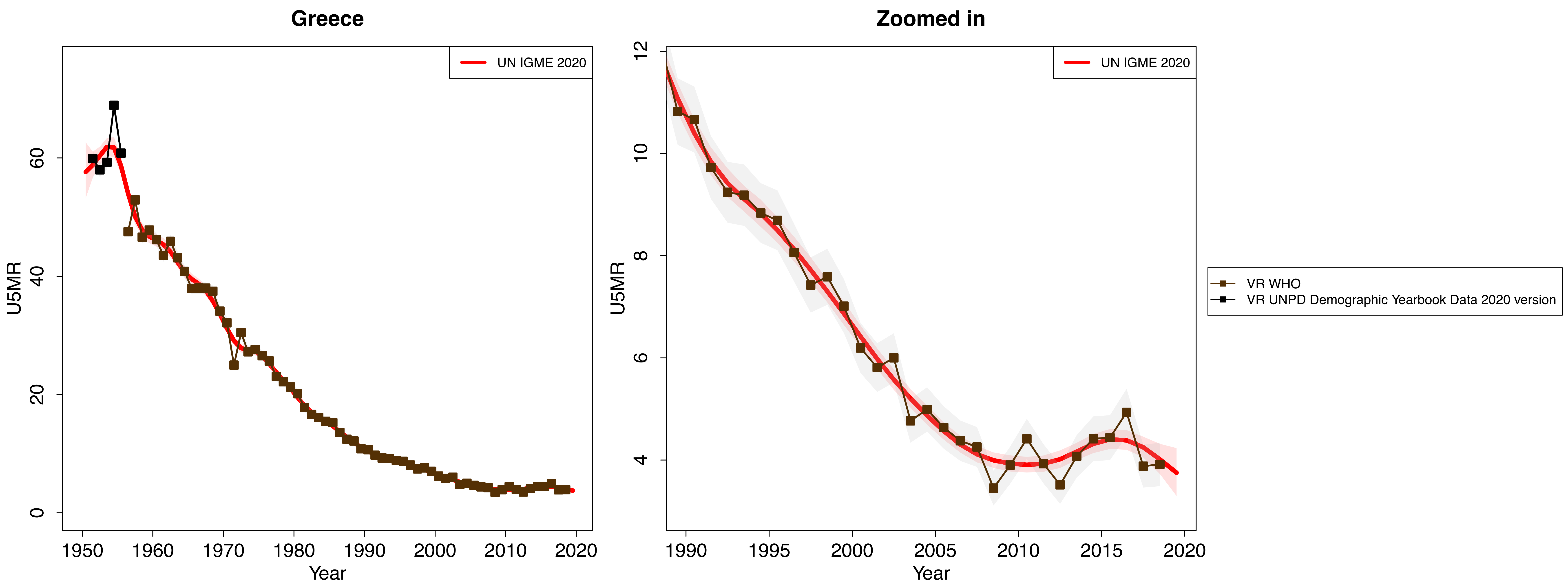

Greece
Zoomed in
UN IGME 2020
U5MR
Year
VR WHO
VR UNPD Demographic Yearbook Data 2020 version

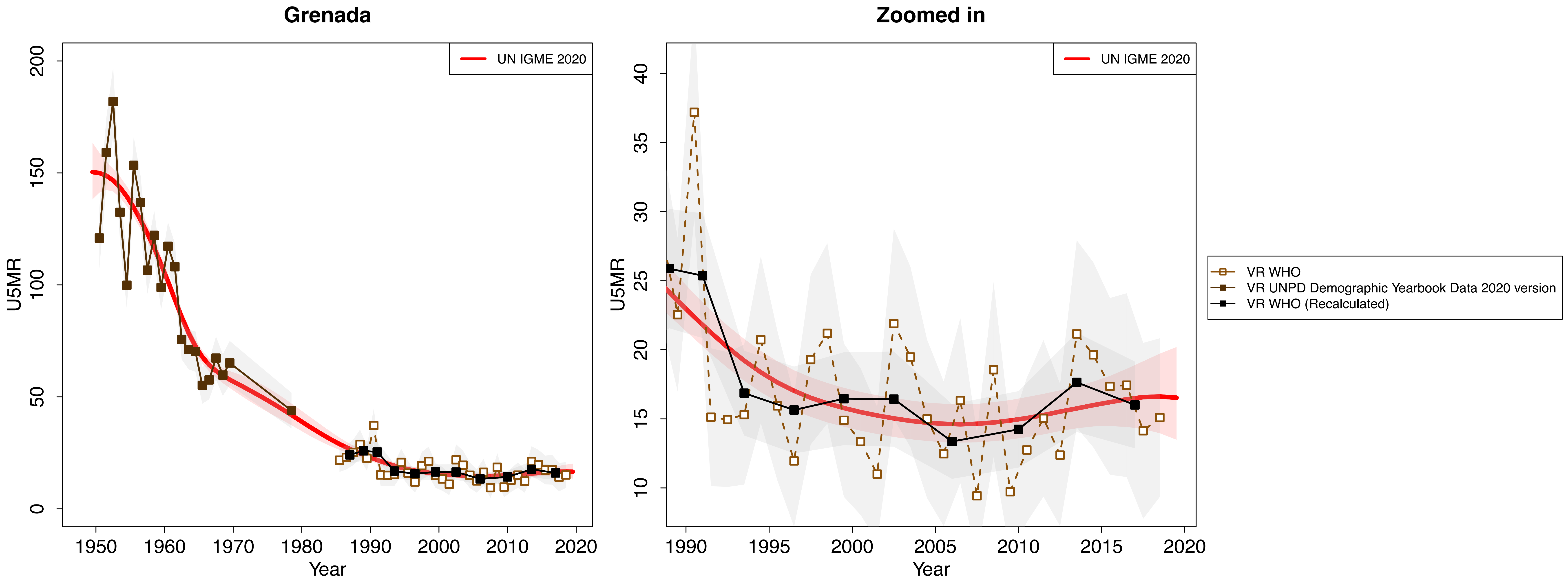
Grenada
Zoomed in
UN IGME 2020
UN IGME 2020
U5MR
Year
VR WHO
VR UNPD Demographic Yearbook Data 2020 version
VR WHO (Recalculated)
0
50
100
150
200
1950
1960
1970
1980
1990
2000
2010
2020
10
15
20
25
30
35
40
1990
1995
2000
2005
2010
2015
2020

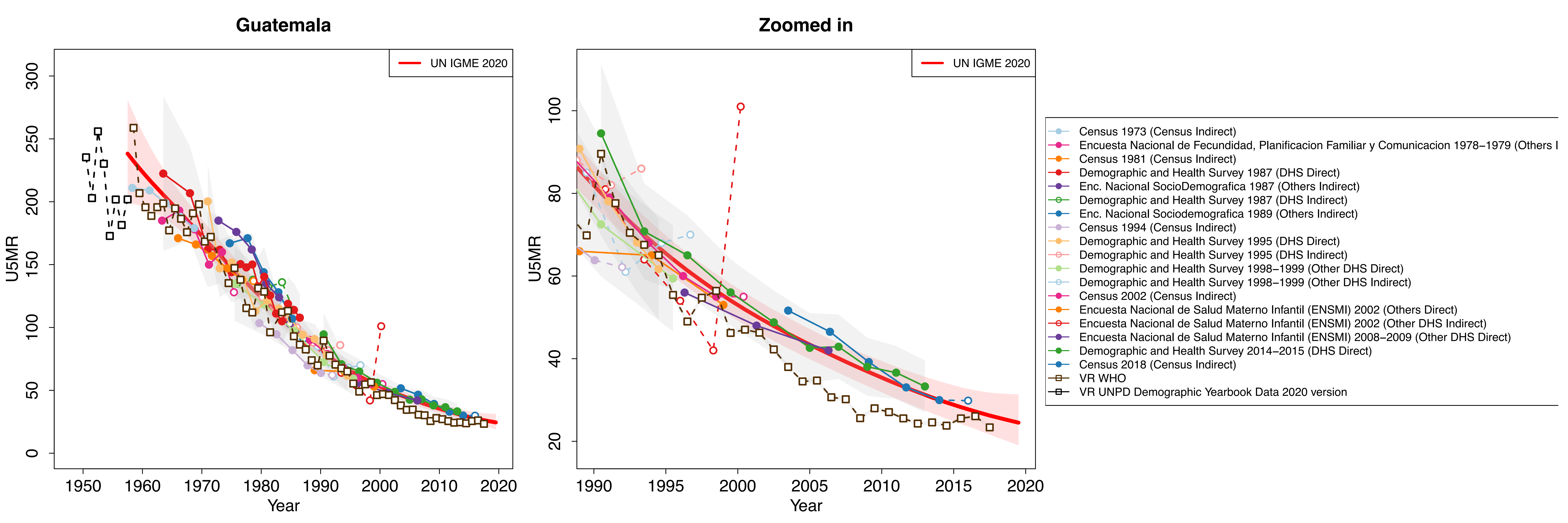

Guatemala
Zoomed in
UN IGME 2020
U5MR
Year
0
50
100
150
200
250
300
1950
1960
1970
1980
1990
2000
2010
2020
20
40
60
80
100
1995
2005
2015
Census 1973 (Census Indirect)
Encuesta Nacional de Fecundidad, Planificacion Familiar y Comunicacion 1978–1979 (Others I
Census 1981 (Census Indirect)
Demographic and Health Survey 1987 (DHS Direct)
Enc. Nacional SocioDemografica 1987 (Others Indirect)
Demographic and Health Survey 1987 (DHS Indirect)
Enc. Nacional Sociodemografica 1989 (Others Indirect)
Census 1994 (Census Indirect)
Demographic and Health Survey 1995 (DHS Direct)
Demographic and Health Survey 1995 (DHS Indirect)
Demographic and Health Survey 1998–1999 (Other DHS Direct)
Demographic and Health Survey 1998–1999 (Other DHS Indirect)
Census 2002 (Census Indirect)
Encuesta Nacional de Salud Materno Infantil (ENSMI) 2002 (Others Direct)
Encuesta Nacional de Salud Materno Infantil (ENSMI) 2002 (Other DHS Indirect)
Encuesta Nacional de Salud Materno Infantil (ENSMI) 2008–2009 (Other DHS Direct)
Demographic and Health Survey 2014–2015 (DHS Direct)
Census 2018 (Census Indirect)
VR WHO
VR UNPD Demographic Yearbook Data 2020 version

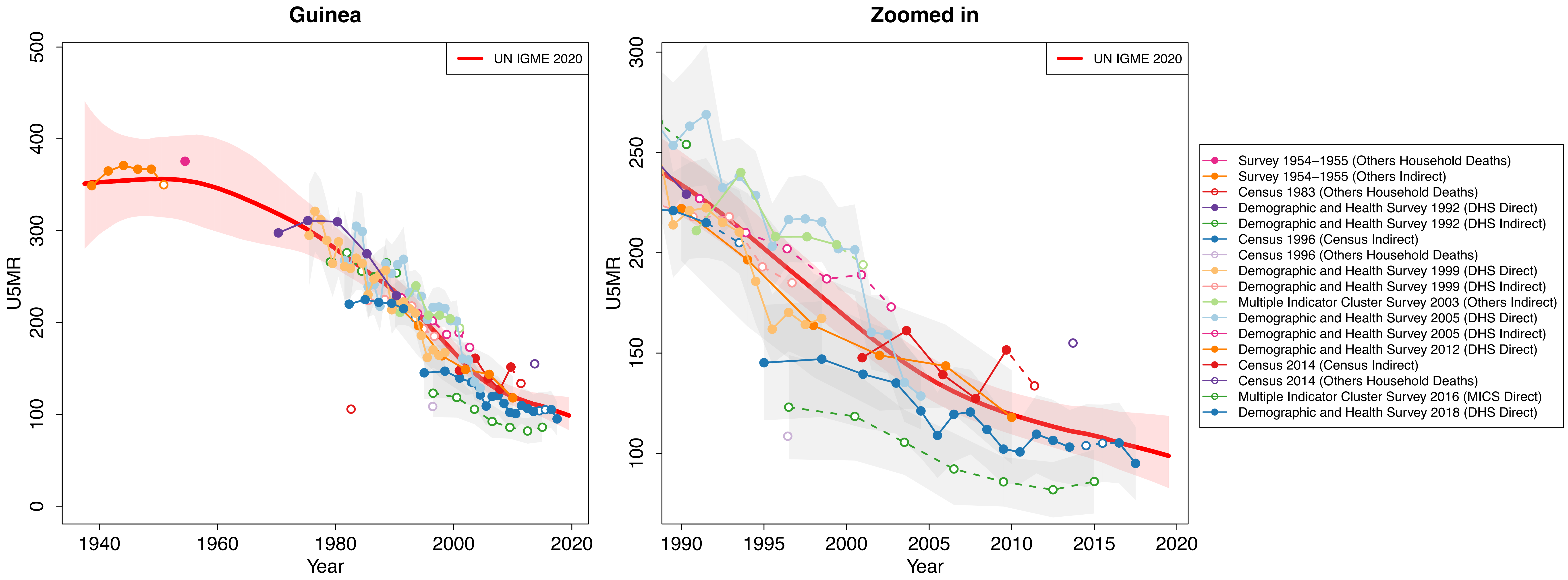

Guinea
Zoomed in
UN IGME 2020
U5MR
Year
Survey 1954–1955 (Others Household Deaths)
Survey 1954–1955 (Others Indirect)
Census 1983 (Others Household Deaths)
Demographic and Health Survey 1992 (DHS Direct)
Demographic and Health Survey 1992 (DHS Indirect)
Census 1996 (Census Indirect)
Census 1996 (Others Household Deaths)
Demographic and Health Survey 1999 (DHS Direct)
Demographic and Health Survey 1999 (DHS Indirect)
Multiple Indicator Cluster Survey 2003 (Others Indirect)
Demographic and Health Survey 2005 (DHS Direct)
Demographic and Health Survey 2005 (DHS Indirect)
Demographic and Health Survey 2012 (DHS Direct)
Census 2014 (Census Indirect)
Census 2014 (Others Household Deaths)
Multiple Indicator Cluster Survey 2016 (MICS Direct)
Demographic and Health Survey 2018 (DHS Direct)

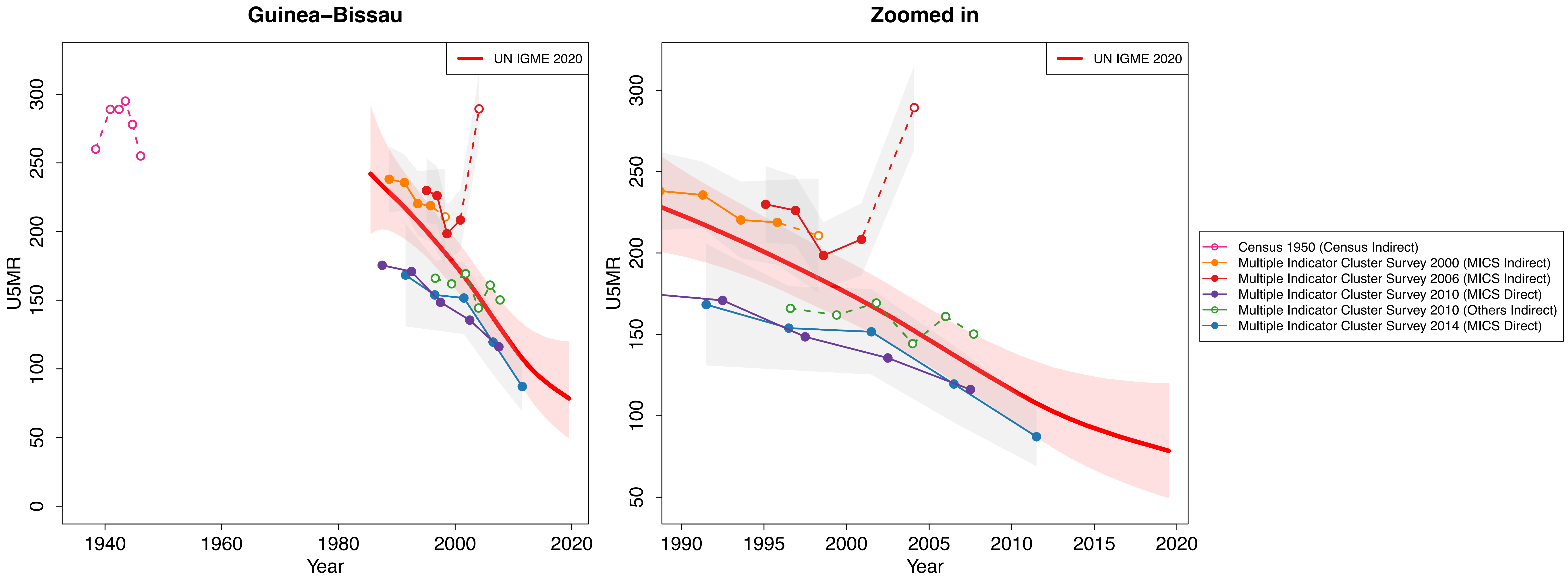

Guinea−Bissau
Zoomed in
UN IGME 2020
U5MR
Year
Census 1950 (Census Indirect)
Multiple Indicator Cluster Survey 2000 (MICS Indirect)
Multiple Indicator Cluster Survey 2006 (MICS Indirect)
Multiple Indicator Cluster Survey 2010 (MICS Direct)
Multiple Indicator Cluster Survey 2010 (Others Indirect)
Multiple Indicator Cluster Survey 2014 (MICS Direct)

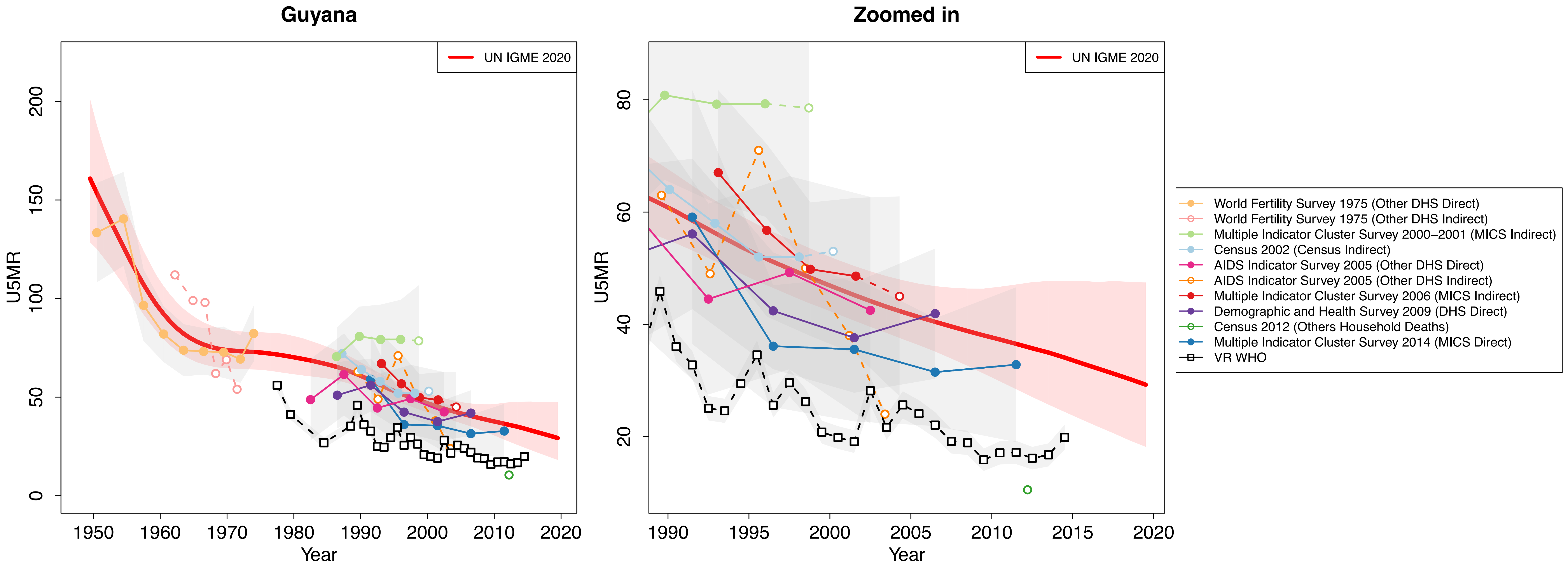
Guyana
Zoomed in
UN IGME 2020
U5MR
Year
World Fertility Survey 1975 (Other DHS Direct)
World Fertility Survey 1975 (Other DHS Indirect)
Multiple Indicator Cluster Survey 2000–2001 (MICS Indirect)
Census 2002 (Census Indirect)
AIDS Indicator Survey 2005 (Other DHS Direct)
AIDS Indicator Survey 2005 (Other DHS Indirect)
Multiple Indicator Cluster Survey 2006 (MICS Indirect)
Demographic and Health Survey 2009 (DHS Direct)
Census 2012 (Others Household Deaths)
Multiple Indicator Cluster Survey 2014 (MICS Direct)
VR WHO

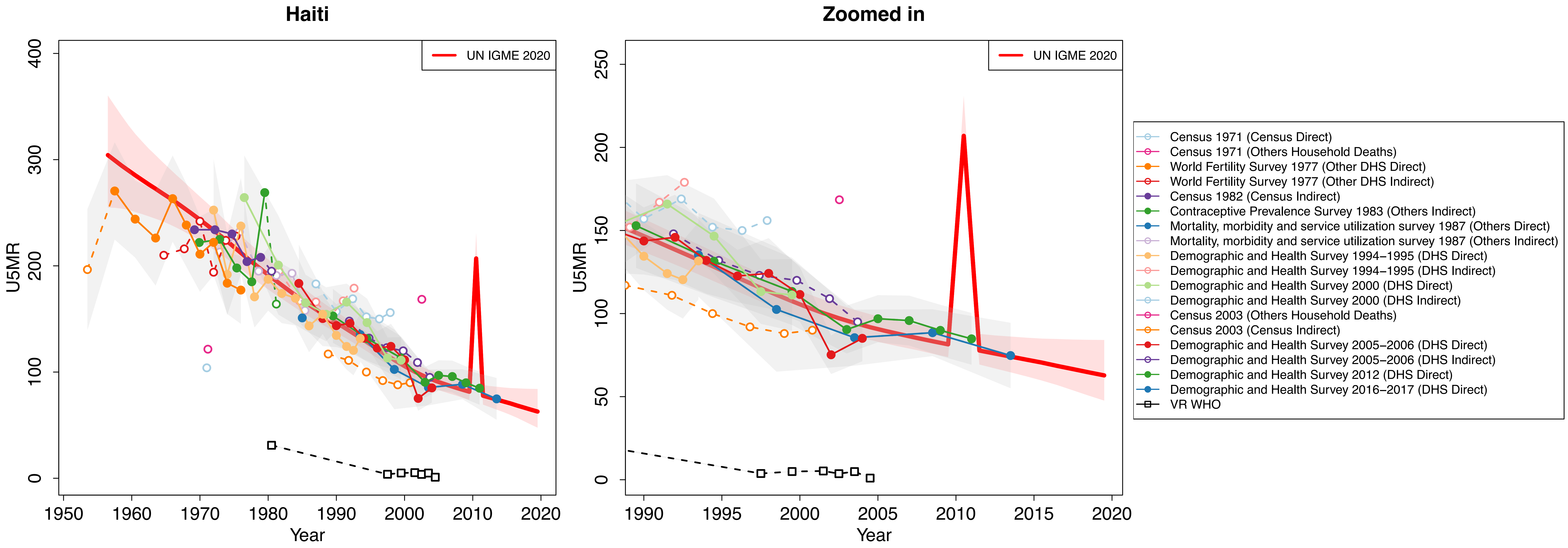

Haiti
UN IGME 2020
U5MR
Year
Zoomed in
UN IGME 2020
U5MR
Year
Census 1971 (Census Direct)
Census 1971 (Others Household Deaths)
World Fertility Survey 1977 (Other DHS Direct)
World Fertility Survey 1977 (Other DHS Indirect)
Census 1982 (Census Indirect)
Contraceptive Prevalence Survey 1983 (Others Indirect)
Mortality, morbidity and service utilization survey 1987 (Others Direct)
Mortality, morbidity and service utilization survey 1987 (Others Indirect)
Demographic and Health Survey 1994–1995 (DHS Direct)
Demographic and Health Survey 1994–1995 (DHS Indirect)
Demographic and Health Survey 2000 (DHS Direct)
Demographic and Health Survey 2000 (DHS Indirect)
Census 2003 (Others Household Deaths)
Census 2003 (Census Indirect)
Demographic and Health Survey 2005–2006 (DHS Direct)
Demographic and Health Survey 2005–2006 (DHS Indirect)
Demographic and Health Survey 2012 (DHS Direct)
Demographic and Health Survey 2016–2017 (DHS Direct)
VR WHO

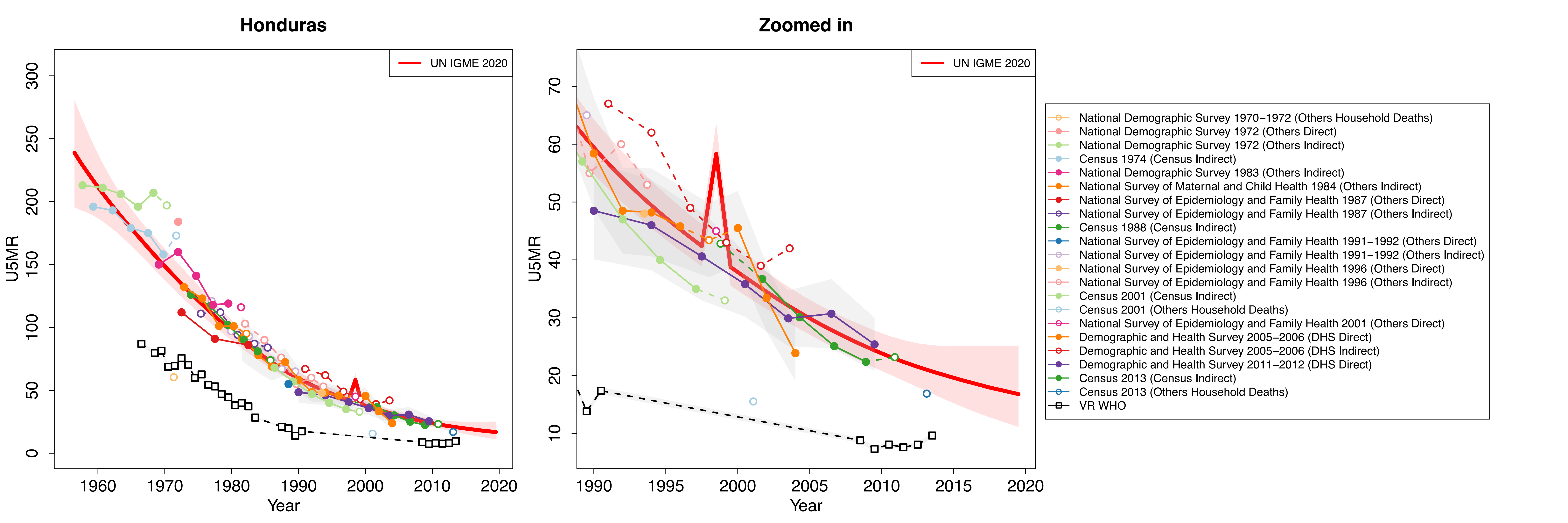
Honduras
Zoomed in
UN IGME 2020
U5MR
Year
National Demographic Survey 1970−1972 (Others Household Deaths)
National Demographic Survey 1972 (Others Direct)
National Demographic Survey 1972 (Others Indirect)
Census 1974 (Census Indirect)
National Demographic Survey 1983 (Others Indirect)
National Survey of Maternal and Child Health 1984 (Others Indirect)
National Survey of Epidemiology and Family Health 1987 (Others Direct)
National Survey of Epidemiology and Family Health 1987 (Others Indirect)
Census 1988 (Census Indirect)
National Survey of Epidemiology and Family Health 1991−1992 (Others Direct)
National Survey of Epidemiology and Family Health 1991−1992 (Others Indirect)
National Survey of Epidemiology and Family Health 1996 (Others Direct)
National Survey of Epidemiology and Family Health 1996 (Others Indirect)
Census 2001 (Census Indirect)
Census 2001 (Others Household Deaths)
National Survey of Epidemiology and Family Health 2001 (Others Direct)
Demographic and Health Survey 2005−2006 (DHS Direct)
Demographic and Health Survey 2005−2006 (DHS Indirect)
Demographic and Health Survey 2011−2012 (DHS Direct)
Census 2013 (Census Indirect)
Census 2013 (Others Household Deaths)
VR WHO

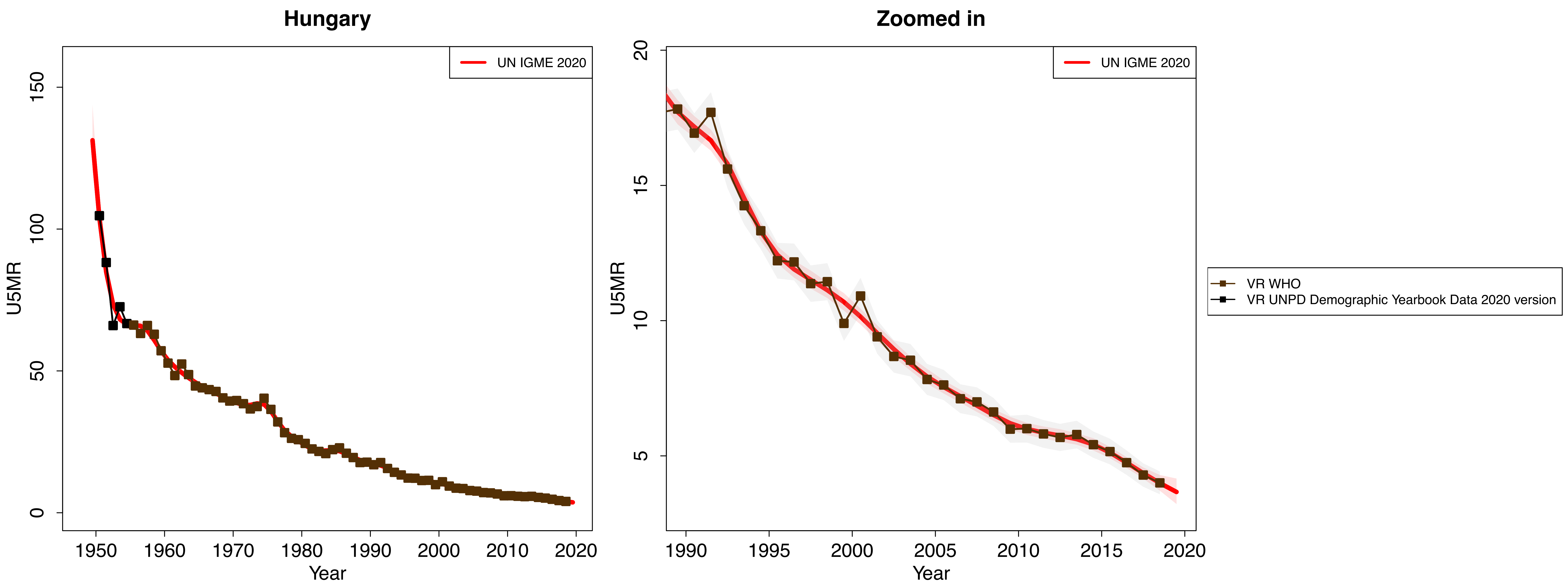

Hungary
Zoomed in
UN IGME 2020
UN IGME 2020
U5MR
U5MR
Year
Year
VR WHO
VR UNPD Demographic Yearbook Data 2020 version

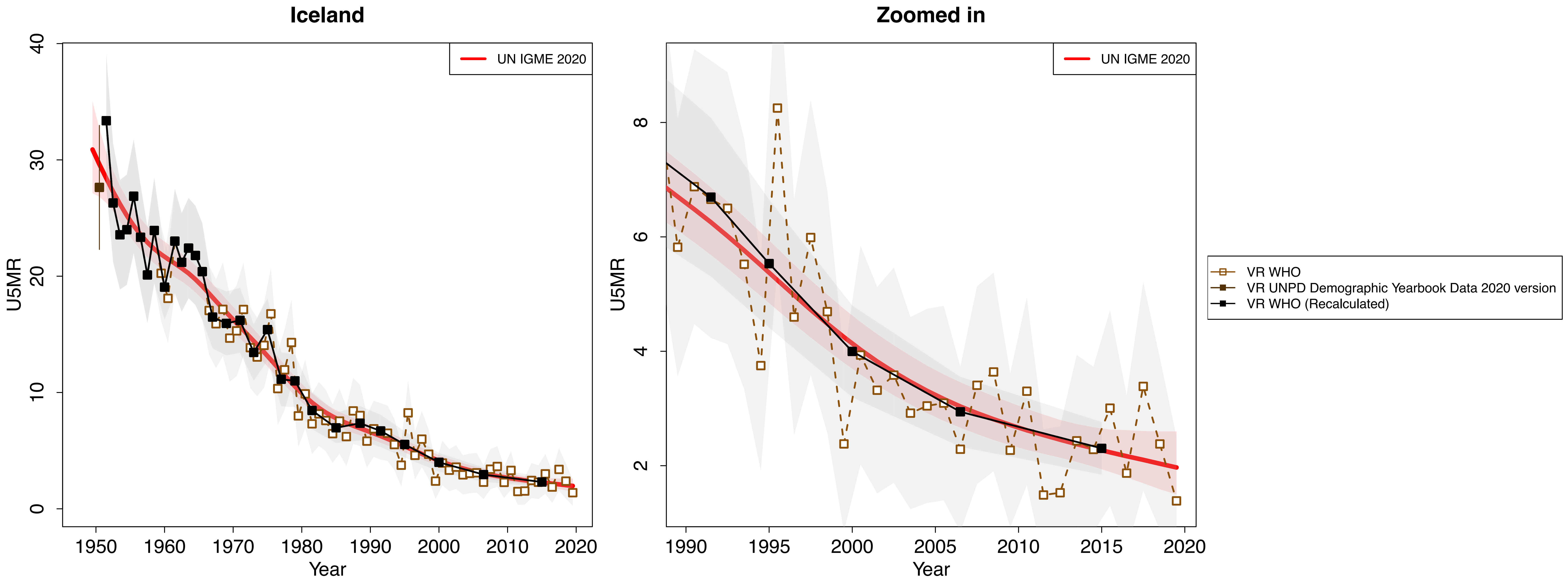

Iceland
Zoomed in
UN IGME 2020
U5MR
Year
VR WHO
VR UNPD Demographic Yearbook Data 2020 version
VR WHO (Recalculated)

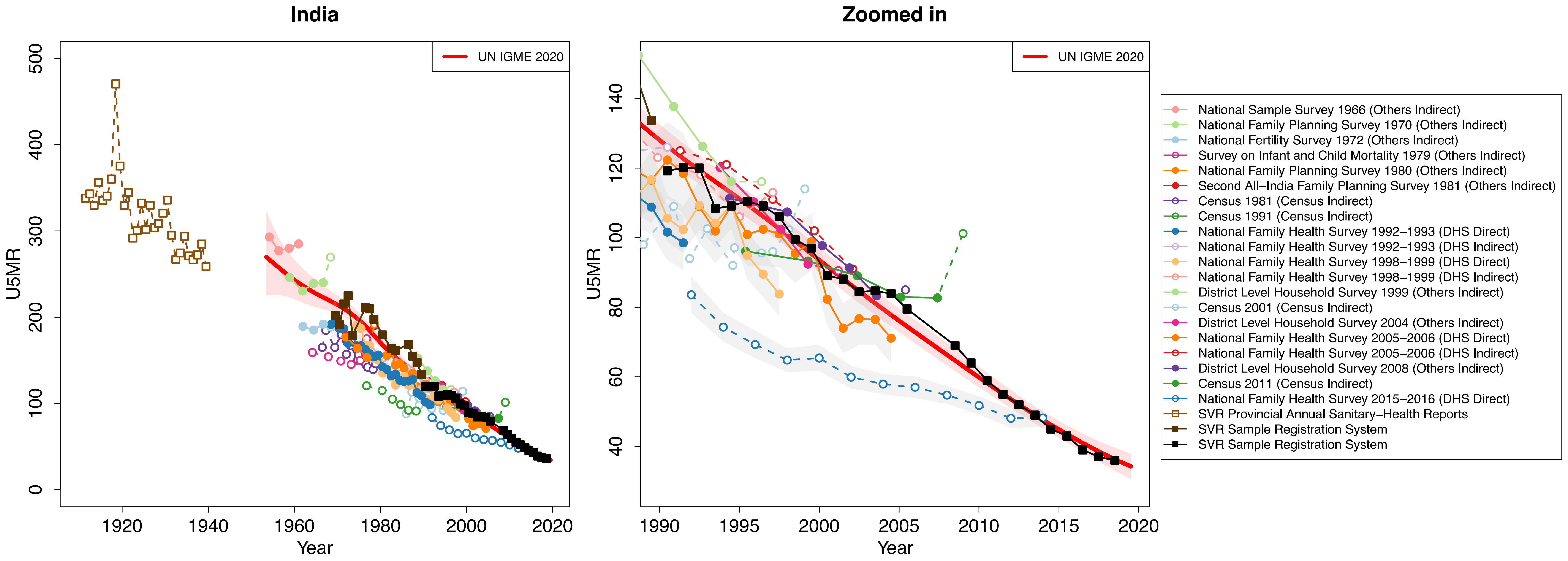

India
Zoomed in
UN IGME 2020
U5MR
Year
National Sample Survey 1966 (Others Indirect)
National Family Planning Survey 1970 (Others Indirect)
National Fertility Survey 1972 (Others Indirect)
Survey on Infant and Child Mortality 1979 (Others Indirect)
National Family Planning Survey 1980 (Others Indirect)
Second All–India Family Planning Survey 1981 (Others Indirect)
Census 1981 (Census Indirect)
Census 1991 (Census Indirect)
National Family Health Survey 1992–1993 (DHS Direct)
National Family Health Survey 1992–1993 (DHS Indirect)
National Family Health Survey 1998–1999 (DHS Direct)
National Family Health Survey 1998–1999 (DHS Indirect)
District Level Household Survey 1999 (Others Indirect)
Census 2001 (Census Indirect)
District Level Household Survey 2004 (Others Indirect)
National Family Health Survey 2005–2006 (DHS Direct)
National Family Health Survey 2005–2006 (DHS Indirect)
District Level Household Survey 2008 (Others Indirect)
Census 2011 (Census Indirect)
National Family Health Survey 2015–2016 (DHS Direct)
SVR Provincial Annual Sanitary–Health Reports
SVR Sample Registration System
SVR Sample Registration System

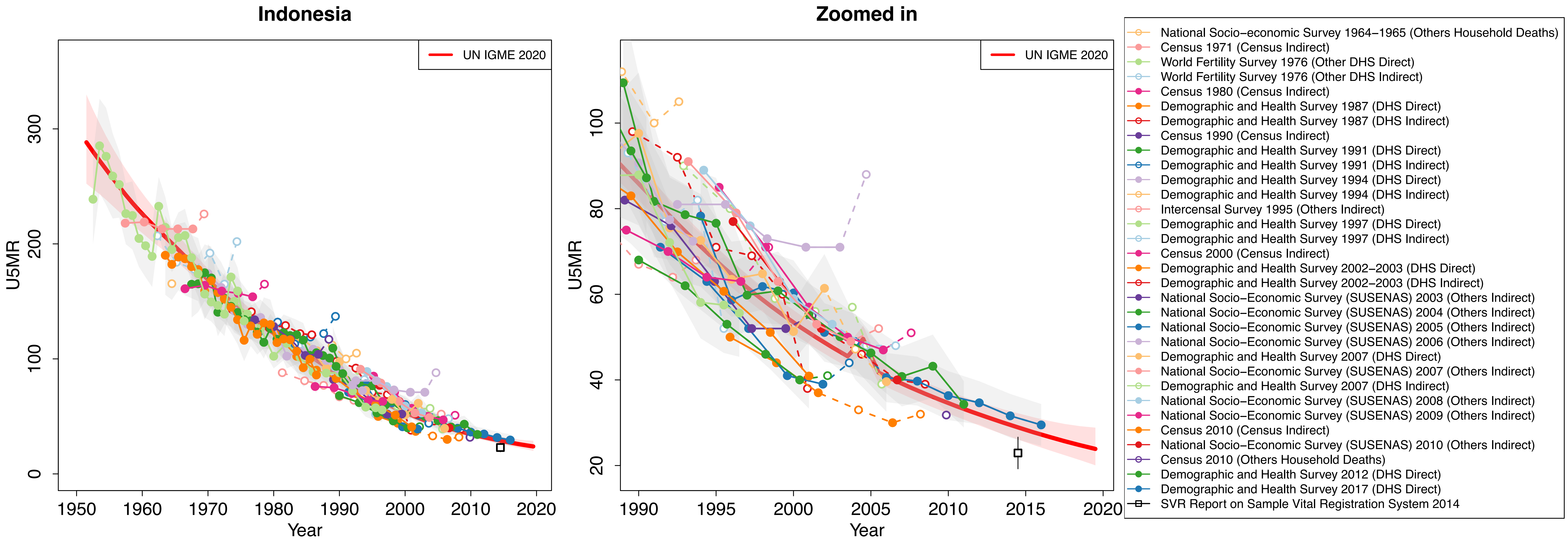

Indonesia
Zoomed in
UN IGME 2020
U5MR
Year
National Socio–economic Survey 1964–1965 (Others Household Deaths)
Census 1971 (Census Indirect)
World Fertility Survey 1976 (Other DHS Direct)
World Fertility Survey 1976 (Other DHS Indirect)
Census 1980 (Census Indirect)
Demographic and Health Survey 1987 (DHS Direct)
Demographic and Health Survey 1987 (DHS Indirect)
Census 1990 (Census Indirect)
Demographic and Health Survey 1991 (DHS Direct)
Demographic and Health Survey 1991 (DHS Indirect)
Demographic and Health Survey 1994 (DHS Direct)
Demographic and Health Survey 1994 (DHS Indirect)
Intercensal Survey 1995 (Others Indirect)
Demographic and Health Survey 1997 (DHS Direct)
Demographic and Health Survey 1997 (DHS Indirect)
Census 2000 (Census Indirect)
Demographic and Health Survey 2002–2003 (DHS Direct)
Demographic and Health Survey 2002–2003 (DHS Indirect)
National Socio–Economic Survey (SUSENAS) 2003 (Others Indirect)
National Socio–Economic Survey (SUSENAS) 2004 (Others Indirect)
National Socio–Economic Survey (SUSENAS) 2005 (Others Indirect)
National Socio–Economic Survey (SUSENAS) 2006 (Others Indirect)
Demographic and Health Survey 2007 (DHS Direct)
National Socio–Economic Survey (SUSENAS) 2007 (Others Indirect)
Demographic and Health Survey 2007 (DHS Indirect)
National Socio–Economic Survey (SUSENAS) 2008 (Others Indirect)
National Socio–Economic Survey (SUSENAS) 2009 (Others Indirect)
Census 2010 (Census Indirect)
National Socio–Economic Survey (SUSENAS) 2010 (Others Indirect)
Census 2010 (Others Household Deaths)
Demographic and Health Survey 2012 (DHS Direct)
Demographic and Health Survey 2017 (DHS Direct)
SVR Report on Sample Vital Registration System 2014

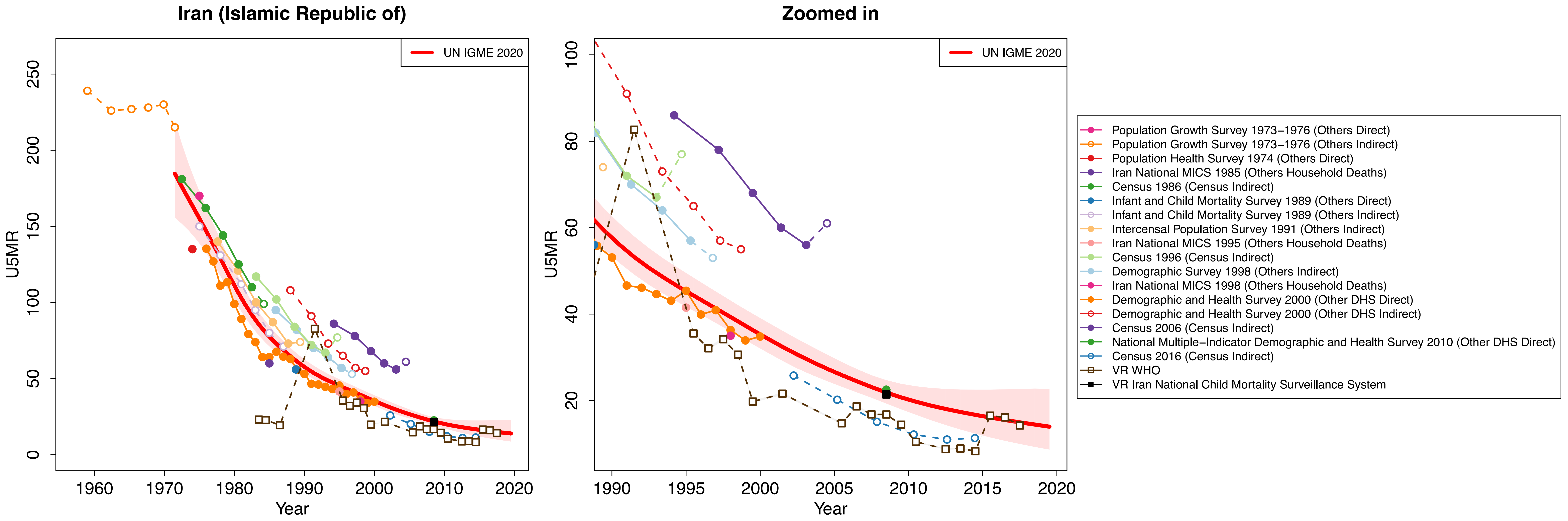

Iran (Islamic Republic of)
Zoomed in
UN IGME 2020
U5MR
Year
Population Growth Survey 1973−1976 (Others Direct)
Population Growth Survey 1973−1976 (Others Indirect)
Population Health Survey 1974 (Others Direct)
Iran National MICS 1985 (Others Household Deaths)
Census 1986 (Census Indirect)
Infant and Child Mortality Survey 1989 (Others Direct)
Infant and Child Mortality Survey 1989 (Others Indirect)
Intercensal Population Survey 1991 (Others Indirect)
Iran National MICS 1995 (Others Household Deaths)
Census 1996 (Census Indirect)
Demographic Survey 1998 (Others Indirect)
Iran National MICS 1998 (Others Household Deaths)
Demographic and Health Survey 2000 (Other DHS Direct)
Demographic and Health Survey 2000 (Other DHS Indirect)
Census 2006 (Census Indirect)
National Multiple−Indicator Demographic and Health Survey 2010 (Other DHS Direct)
Census 2016 (Census Indirect)
VR WHO
VR Iran National Child Mortality Surveillance System

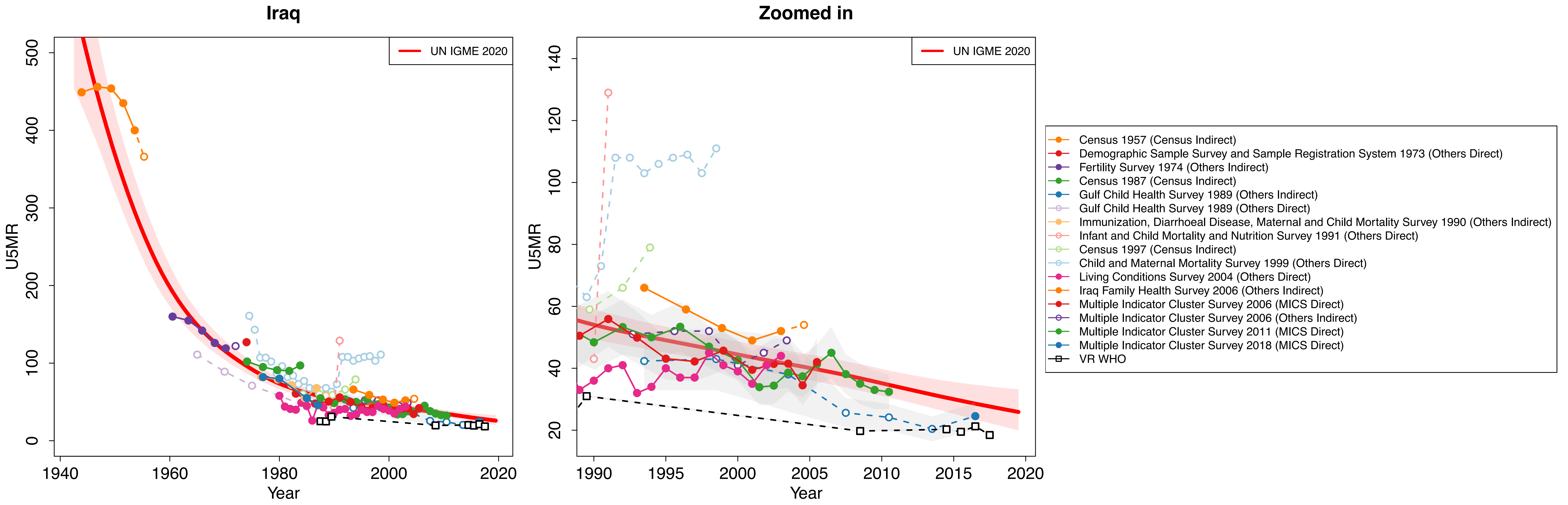

Iraq
Zoomed in
UN IGME 2020
UN IGME 2020
U5MR
Year
U5MR
Year
Census 1957 (Census Indirect)
Demographic Sample Survey and Sample Registration System 1973 (Others Direct)
Fertility Survey 1974 (Others Indirect)
Census 1987 (Census Indirect)
Gulf Child Health Survey 1989 (Others Indirect)
Gulf Child Health Survey 1989 (Others Direct)
Immunization, Diarrhoeal Disease, Maternal and Child Mortality Survey 1990 (Others Indirect)
Infant and Child Mortality and Nutrition Survey 1991 (Others Direct)
Census 1997 (Census Indirect)
Child and Maternal Mortality Survey 1999 (Others Direct)
Living Conditions Survey 2004 (Others Direct)
Iraq Family Health Survey 2006 (Others Indirect)
Multiple Indicator Cluster Survey 2006 (MICS Direct)
Multiple Indicator Cluster Survey 2006 (Others Indirect)
Multiple Indicator Cluster Survey 2011 (MICS Direct)
Multiple Indicator Cluster Survey 2018 (MICS Direct)
VR WHO

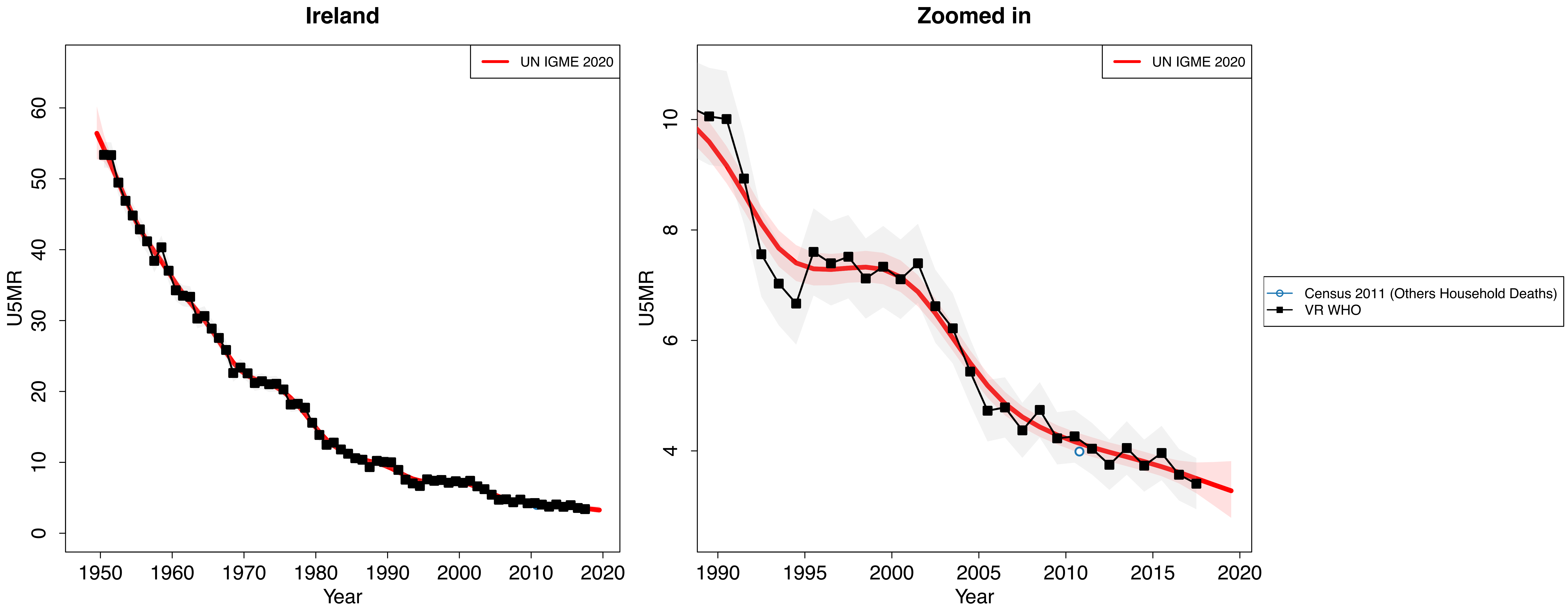

Ireland
UN IGME 2020
U5MR
Year
Zoomed in
UN IGME 2020
Census 2011 (Others Household Deaths)
VR WHO

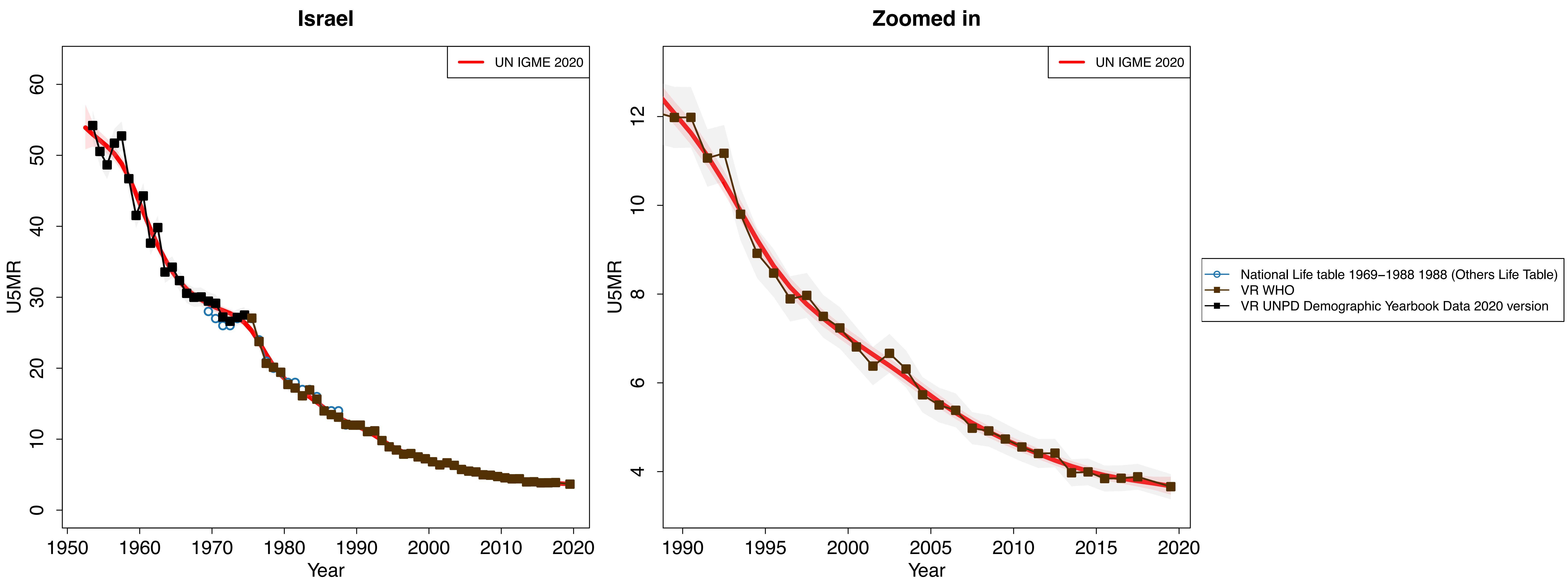
Israel
Zoomed in
UN IGME 2020
UN IGME 2020
U5MR
U5MR
Year
Year
National Life table 1969–1988 1988 (Others Life Table)
VR WHO
VR UNPD Demographic Yearbook Data 2020 version

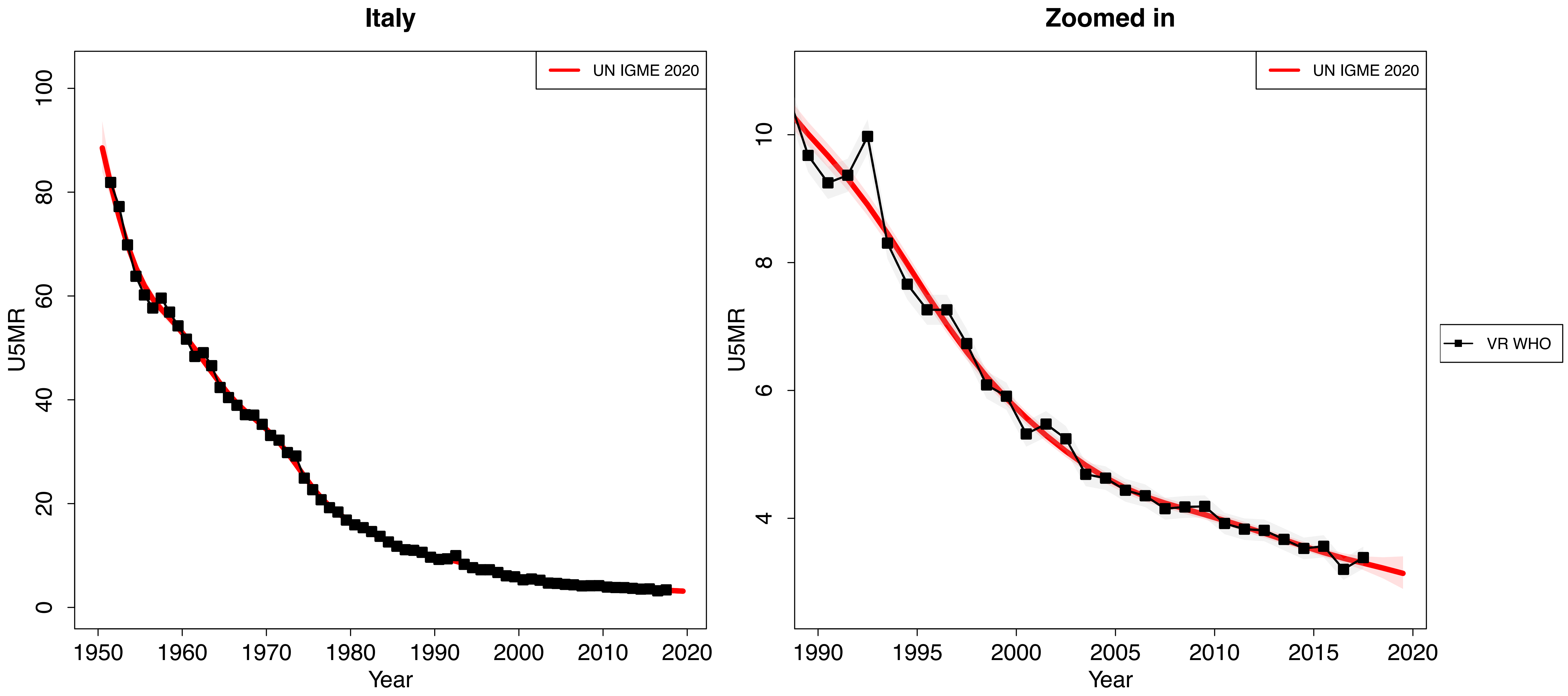
Italy
Zoomed in
UN IGME 2020
UN IGME 2020
VR WHO
U5MR
U5MR
Year
Year
0
20
40
60
80
100
4
6
8
10
1950
1960
1970
1980
1990
2000
2010
2020
1990
1995
2000
2005
2010
2015
2020

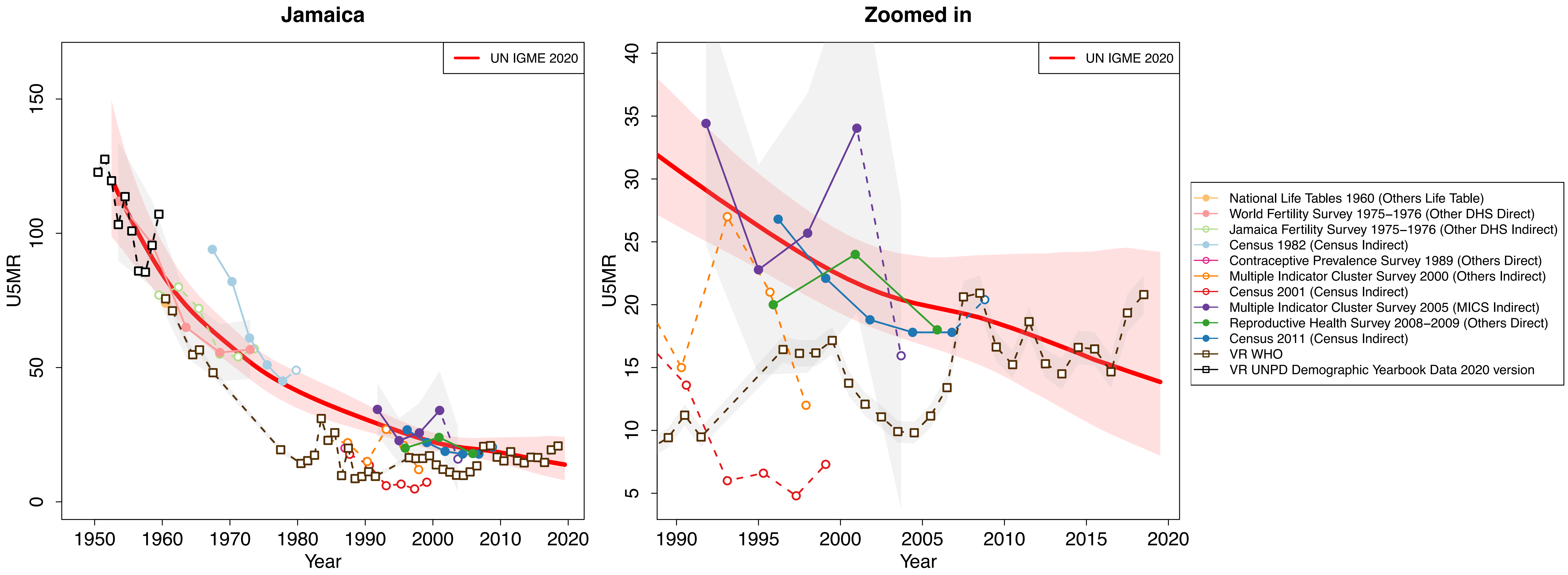

Jamaica
Zoomed in
UN IGME 2020
U5MR
Year
National Life Tables 1960 (Others Life Table)
World Fertility Survey 1975–1976 (Other DHS Direct)
Jamaica Fertility Survey 1975–1976 (Other DHS Indirect)
Census 1982 (Census Indirect)
Contraceptive Prevalence Survey 1989 (Others Direct)
Multiple Indicator Cluster Survey 2000 (Others Indirect)
Census 2001 (Census Indirect)
Multiple Indicator Cluster Survey 2005 (MICS Indirect)
Reproductive Health Survey 2008–2009 (Others Direct)
Census 2011 (Census Indirect)
VR WHO
VR UNPD Demographic Yearbook Data 2020 version

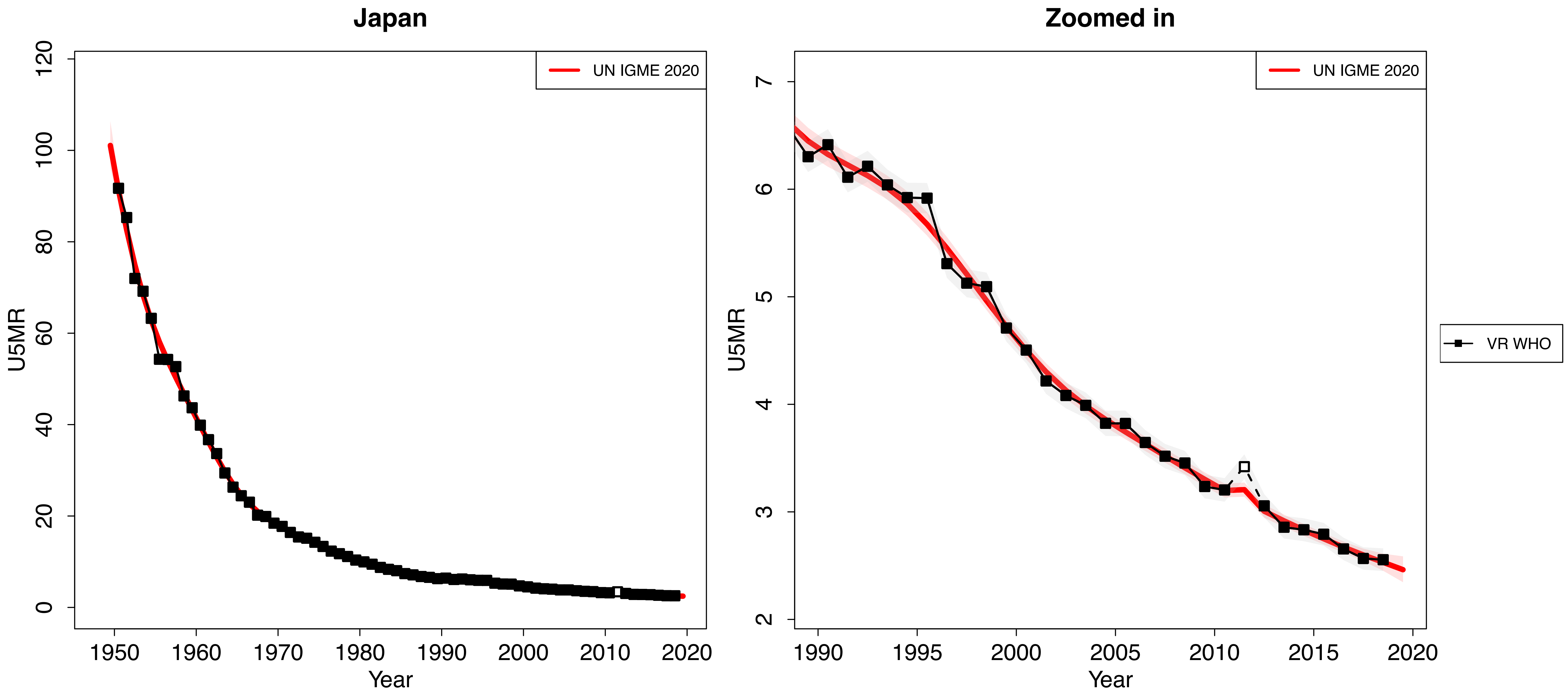

Japan
UN IGME 2020
U5MR
Year
0
20
40
60
80
100
120
1950
1960
1970
1980
1990
2000
2010
2020
Zoomed in
UN IGME 2020
U5MR
Year
2
3
4
5
6
7
1990
1995
2000
2005
2010
2015
2020
VR WHO

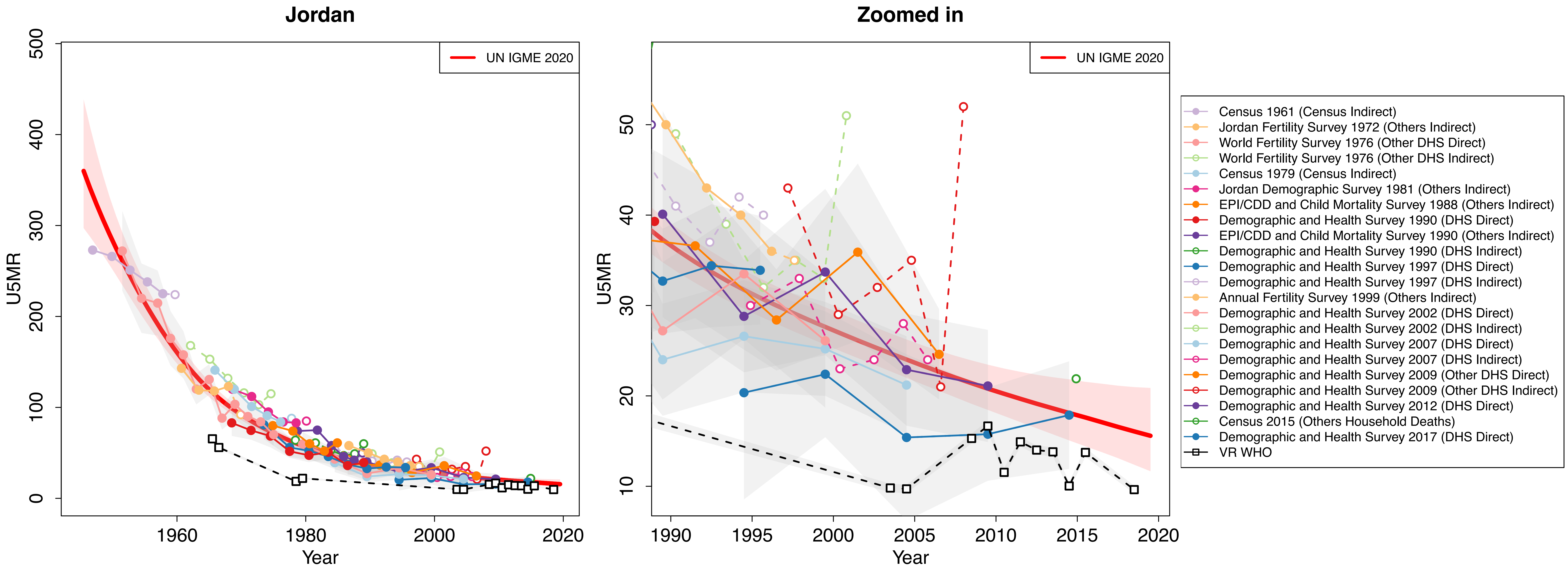

Jordan
Zoomed in
UN IGME 2020
U5MR
Year
0
100
200
300
400
500
1960
1980
2000
2020
10
20
30
40
50
1990
1995
2000
2005
2010
2015
2020
Census 1961 (Census Indirect)
Jordan Fertility Survey 1972 (Others Indirect)
World Fertility Survey 1976 (Other DHS Direct)
World Fertility Survey 1976 (Other DHS Indirect)
Census 1979 (Census Indirect)
Jordan Demographic Survey 1981 (Others Indirect)
EPI/CDD and Child Mortality Survey 1988 (Others Indirect)
Demographic and Health Survey 1990 (DHS Direct)
EPI/CDD and Child Mortality Survey 1990 (Others Indirect)
Demographic and Health Survey 1990 (DHS Indirect)
Demographic and Health Survey 1997 (DHS Direct)
Demographic and Health Survey 1997 (DHS Indirect)
Annual Fertility Survey 1999 (Others Indirect)
Demographic and Health Survey 2002 (DHS Direct)
Demographic and Health Survey 2002 (DHS Indirect)
Demographic and Health Survey 2007 (DHS Direct)
Demographic and Health Survey 2007 (DHS Indirect)
Demographic and Health Survey 2009 (Other DHS Direct)
Demographic and Health Survey 2009 (Other DHS Indirect)
Demographic and Health Survey 2012 (DHS Direct)
Census 2015 (Others Household Deaths)
Demographic and Health Survey 2017 (DHS Direct)
VR WHO

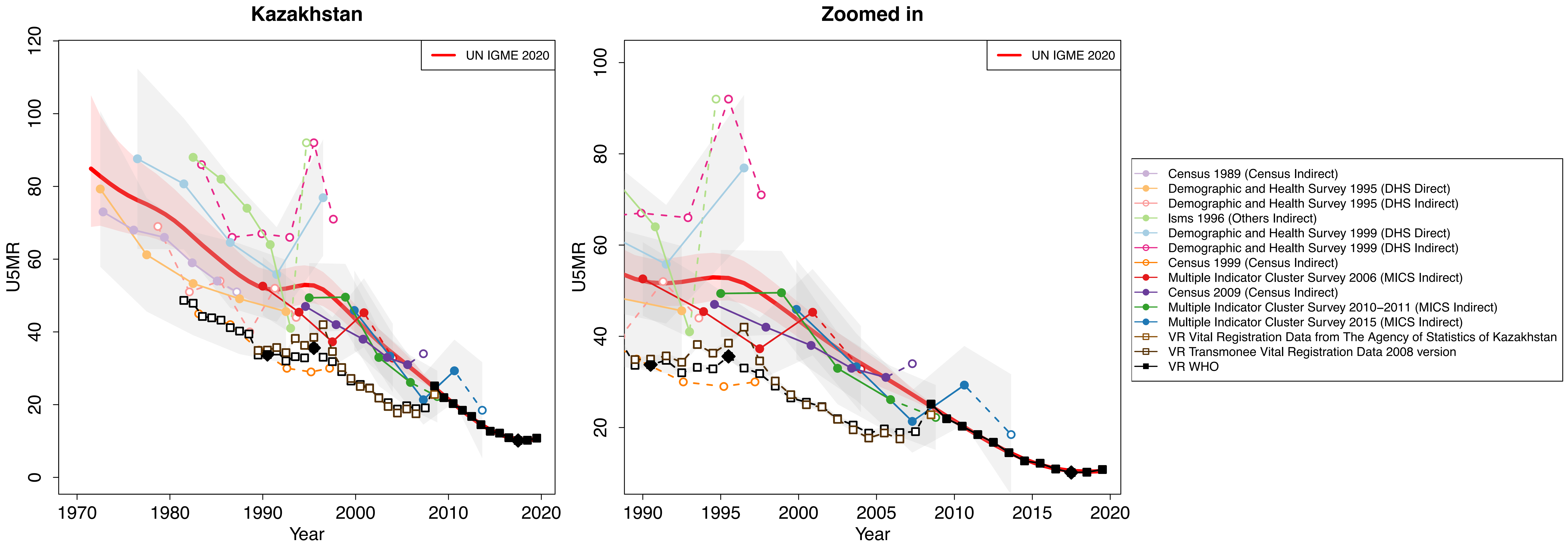

Kazakhstan
Zoomed in
UN IGME 2020
U5MR
Year
Census 1989 (Census Indirect)
Demographic and Health Survey 1995 (DHS Direct)
Demographic and Health Survey 1995 (DHS Indirect)
Isms 1996 (Others Indirect)
Demographic and Health Survey 1999 (DHS Direct)
Demographic and Health Survey 1999 (DHS Indirect)
Census 1999 (Census Indirect)
Multiple Indicator Cluster Survey 2006 (MICS Indirect)
Census 2009 (Census Indirect)
Multiple Indicator Cluster Survey 2010–2011 (MICS Indirect)
Multiple Indicator Cluster Survey 2015 (MICS Indirect)
VR Vital Registration Data from The Agency of Statistics of Kazakhstan
VR Transmonee Vital Registration Data 2008 version
VR WHO

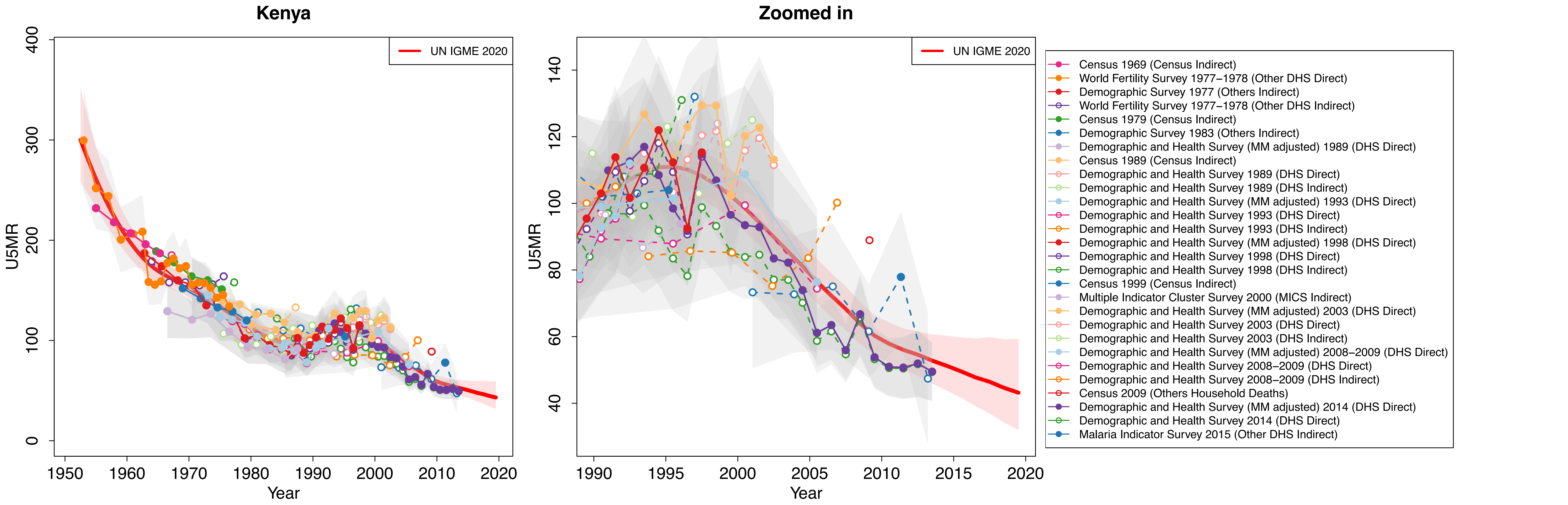

Kenya
Zoomed in
UN IGME 2020
U5MR
Year
Census 1969 (Census Indirect)
World Fertility Survey 1977–1978 (Other DHS Direct)
Demographic Survey 1977 (Others Indirect)
World Fertility Survey 1977–1978 (Other DHS Indirect)
Census 1979 (Census Indirect)
Demographic Survey 1983 (Others Indirect)
Demographic and Health Survey (MM adjusted) 1989 (DHS Direct)
Census 1989 (Census Indirect)
Demographic and Health Survey 1989 (DHS Direct)
Demographic and Health Survey 1989 (DHS Indirect)
Demographic and Health Survey (MM adjusted) 1993 (DHS Direct)
Demographic and Health Survey 1993 (DHS Direct)
Demographic and Health Survey 1993 (DHS Indirect)
Demographic and Health Survey (MM adjusted) 1998 (DHS Direct)
Demographic and Health Survey 1998 (DHS Direct)
Demographic and Health Survey 1998 (DHS Indirect)
Census 1999 (Census Indirect)
Multiple Indicator Cluster Survey 2000 (MICS Indirect)
Demographic and Health Survey (MM adjusted) 2003 (DHS Direct)
Demographic and Health Survey 2003 (DHS Direct)
Demographic and Health Survey 2003 (DHS Indirect)
Demographic and Health Survey (MM adjusted) 2008–2009 (DHS Direct)
Demographic and Health Survey 2008–2009 (DHS Direct)
Demographic and Health Survey 2008–2009 (DHS Indirect)
Census 2009 (Others Household Deaths)
Demographic and Health Survey (MM adjusted) 2014 (DHS Direct)
Demographic and Health Survey 2014 (DHS Direct)
Malaria Indicator Survey 2015 (Other DHS Indirect)

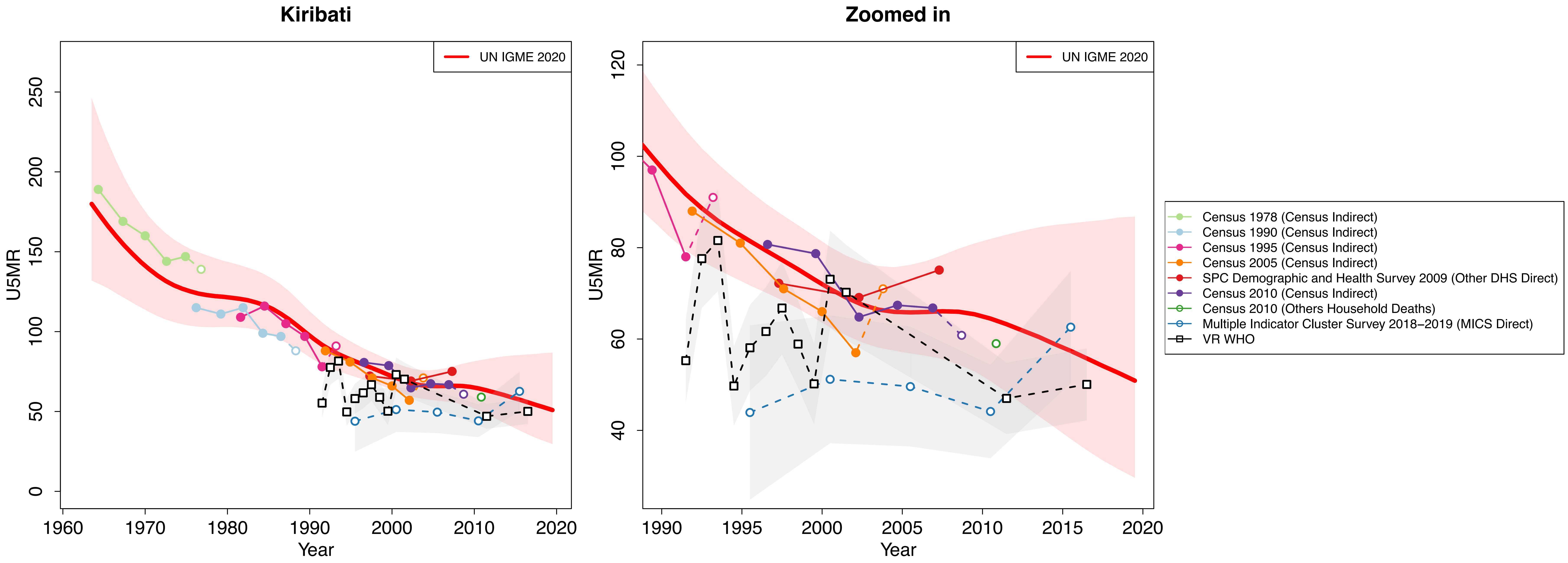

Kiribati
Zoomed in
UN IGME 2020
U5MR
Year
Census 1978 (Census Indirect)
Census 1990 (Census Indirect)
Census 1995 (Census Indirect)
Census 2005 (Census Indirect)
SPC Demographic and Health Survey 2009 (Other DHS Direct)
Census 2010 (Census Indirect)
Census 2010 (Others Household Deaths)
Multiple Indicator Cluster Survey 2018–2019 (MICS Direct)
VR WHO

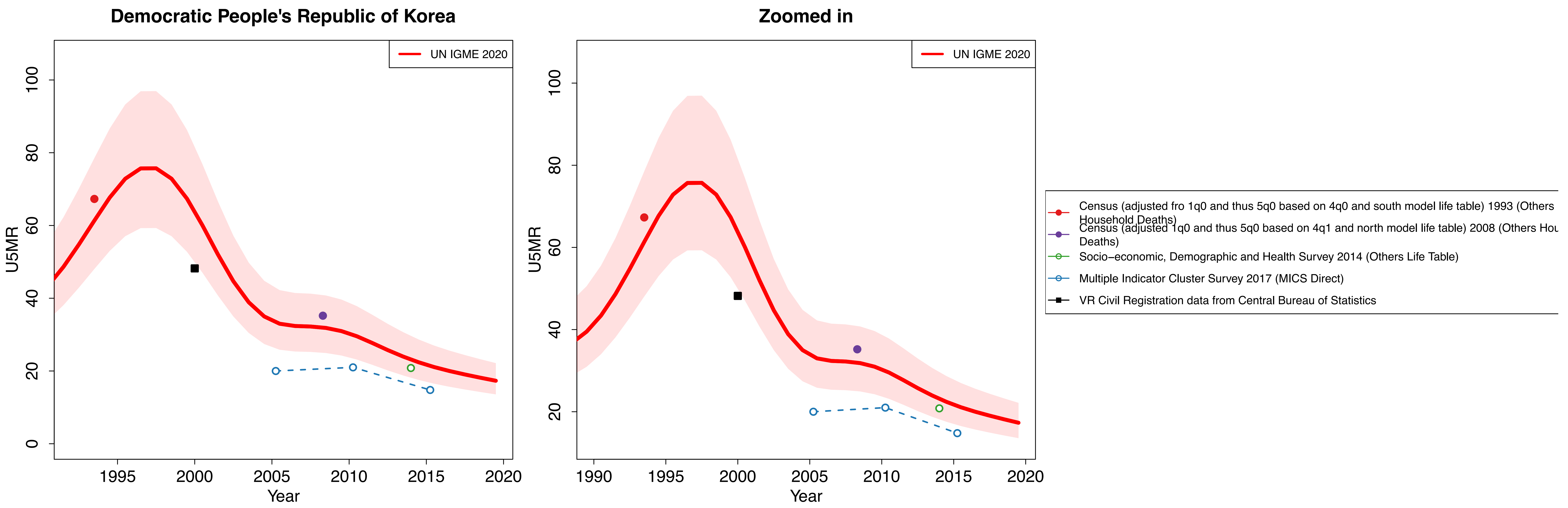

Democratic People's Republic of Korea
Zoomed in
UN IGME 2020
U5MR
Year
Census (adjusted fro 1q0 and thus 5q0 based on 4q0 and south model life table) 1993 (Others Household Deaths)
Census (adjusted 1q0 and thus 5q0 based on 4q1 and north model life table) 2008 (Others Hou Deaths)
Socio−economic, Demographic and Health Survey 2014 (Others Life Table)
Multiple Indicator Cluster Survey 2017 (MICS Direct)
VR Civil Registration data from Central Bureau of Statistics

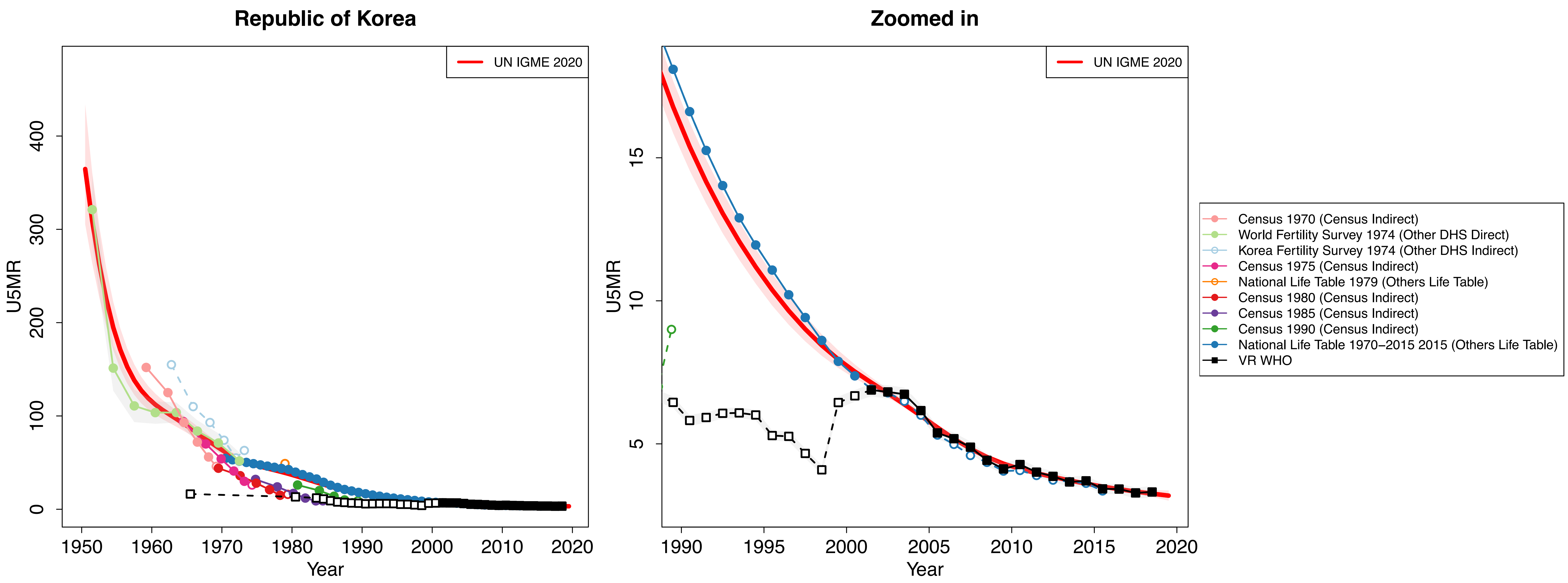
Republic of Korea
Zoomed in
UN IGME 2020
U5MR
Year
Census 1970 (Census Indirect)
World Fertility Survey 1974 (Other DHS Direct)
Korea Fertility Survey 1974 (Other DHS Indirect)
Census 1975 (Census Indirect)
National Life Table 1979 (Others Life Table)
Census 1980 (Census Indirect)
Census 1985 (Census Indirect)
Census 1990 (Census Indirect)
National Life Table 1970–2015 2015 (Others Life Table)
VR WHO

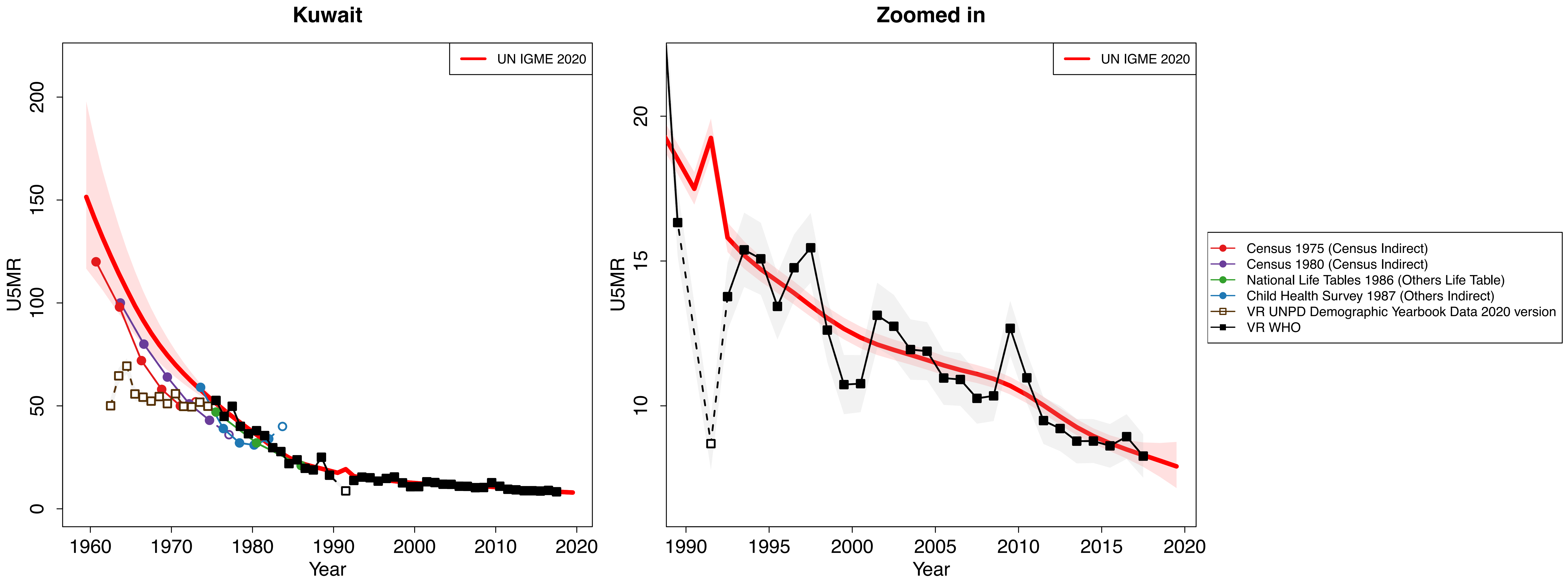

Kuwait
Zoomed in
UN IGME 2020
UN IGME 2020
U5MR
U5MR
Year
Year
Census 1975 (Census Indirect)
Census 1980 (Census Indirect)
National Life Tables 1986 (Others Life Table)
Child Health Survey 1987 (Others Indirect)
VR UNPD Demographic Yearbook Data 2020 version
VR WHO

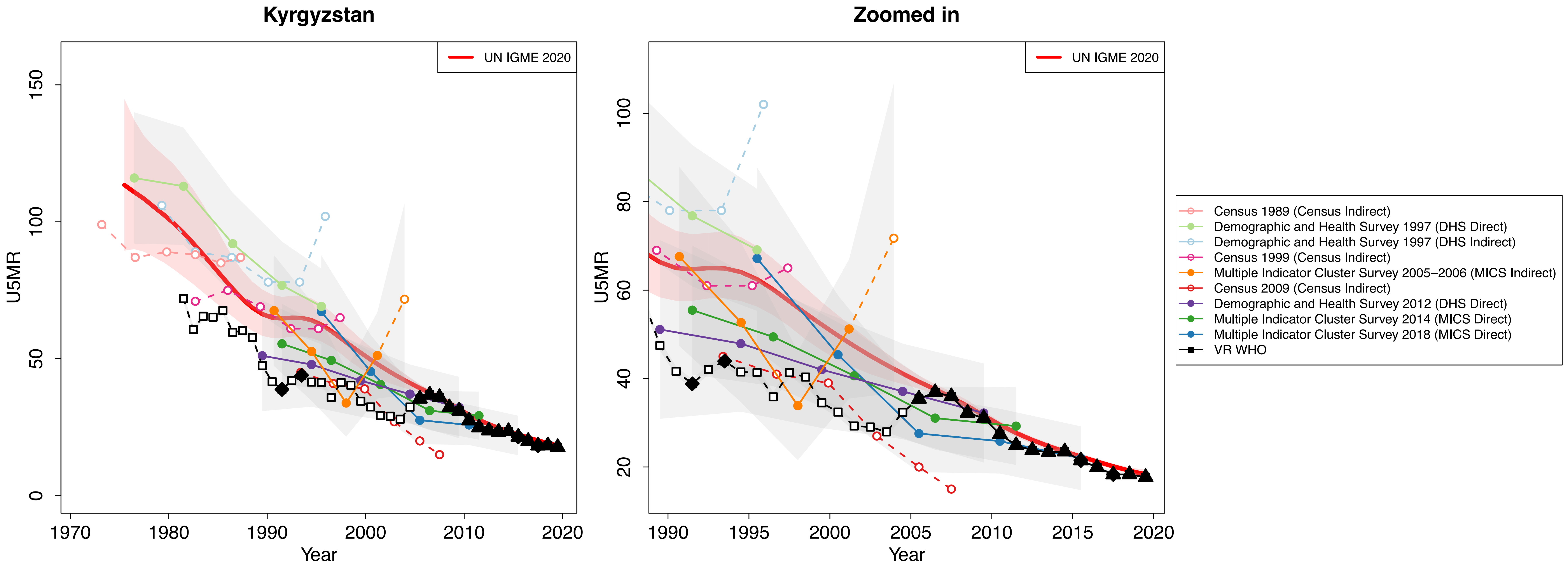

Kyrgyzstan
Zoomed in
UN IGME 2020
U5MR
Year
Census 1989 (Census Indirect)
Demographic and Health Survey 1997 (DHS Direct)
Demographic and Health Survey 1997 (DHS Indirect)
Census 1999 (Census Indirect)
Multiple Indicator Cluster Survey 2005–2006 (MICS Indirect)
Census 2009 (Census Indirect)
Demographic and Health Survey 2012 (DHS Direct)
Multiple Indicator Cluster Survey 2014 (MICS Direct)
Multiple Indicator Cluster Survey 2018 (MICS Direct)
VR WHO

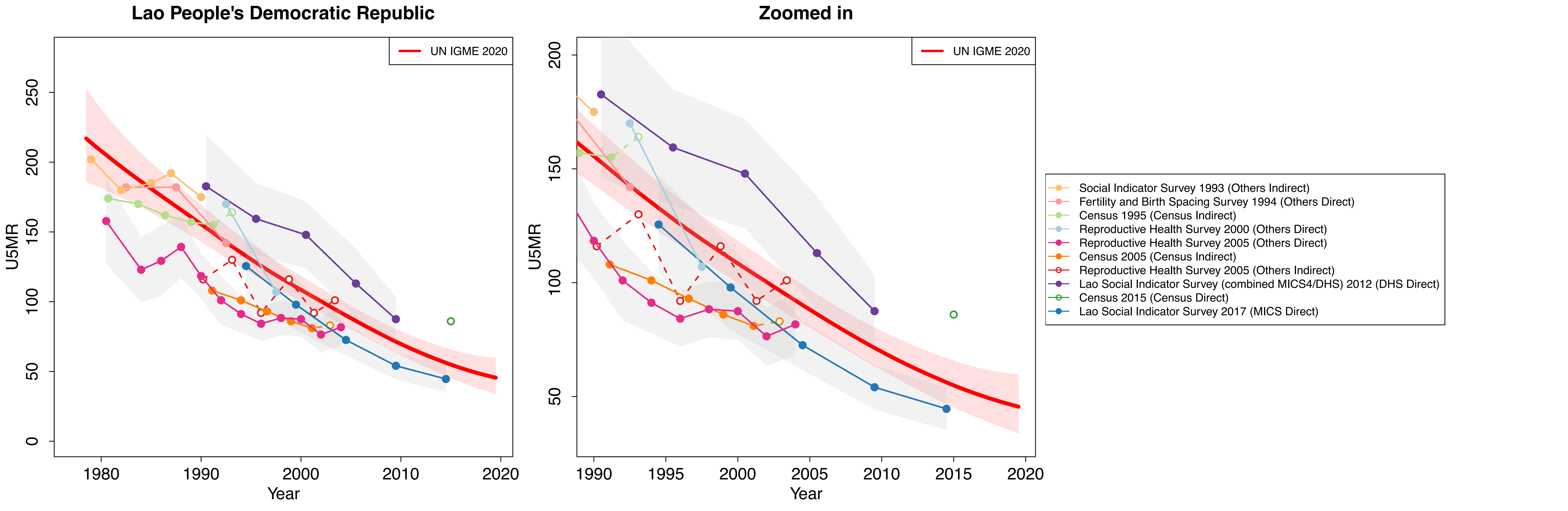

Lao People's Democratic Republic
Zoomed in
UN IGME 2020
U5MR
Year
Social Indicator Survey 1993 (Others Indirect)
Fertility and Birth Spacing Survey 1994 (Others Direct)
Census 1995 (Census Indirect)
Reproductive Health Survey 2000 (Others Direct)
Reproductive Health Survey 2005 (Others Direct)
Census 2005 (Census Indirect)
Reproductive Health Survey 2005 (Others Indirect)
Lao Social Indicator Survey (combined MICS4/DHS) 2012 (DHS Direct)
Census 2015 (Census Direct)
Lao Social Indicator Survey 2017 (MICS Direct)

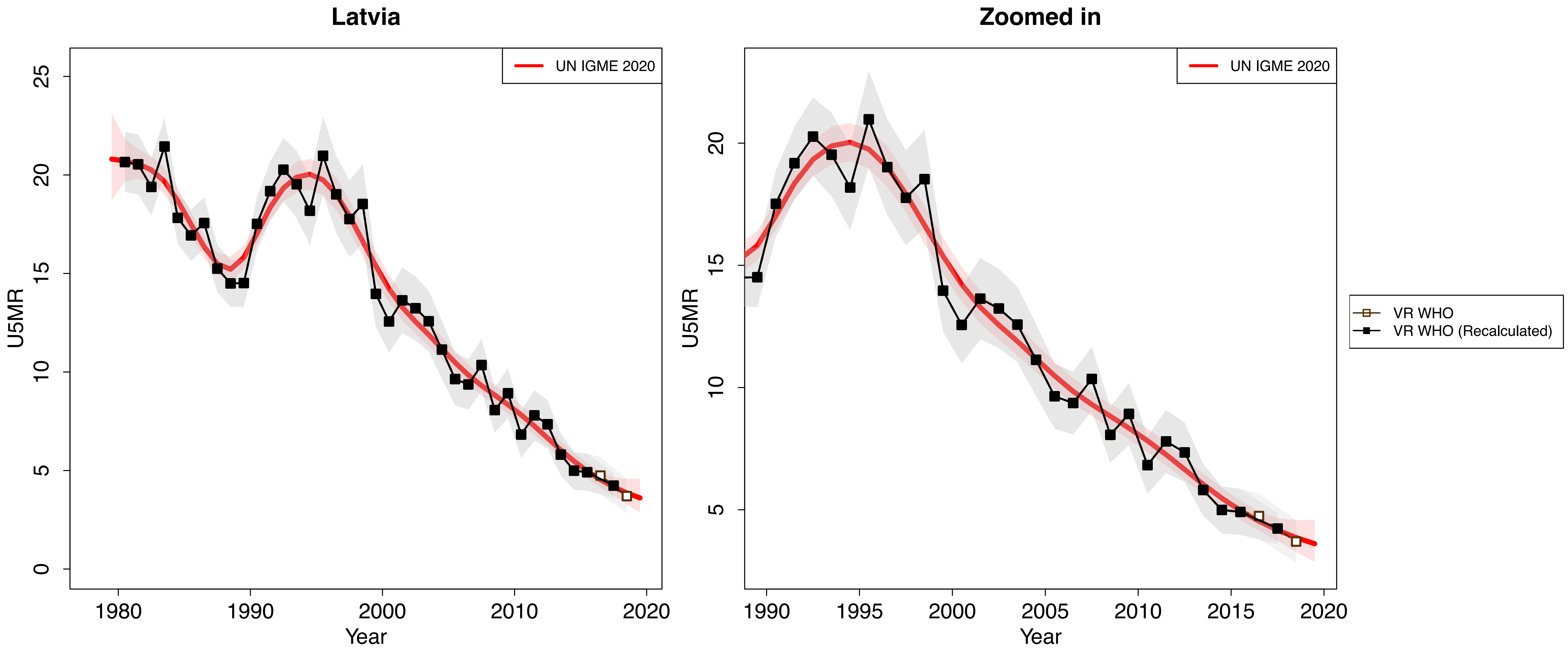
Latvia
Zoomed in
UN IGME 2020
U5MR
Year
VR WHO
VR WHO (Recalculated)
0
5
10
15
20
25
1980
1990
2000
2010
2020
1995
2005
2015

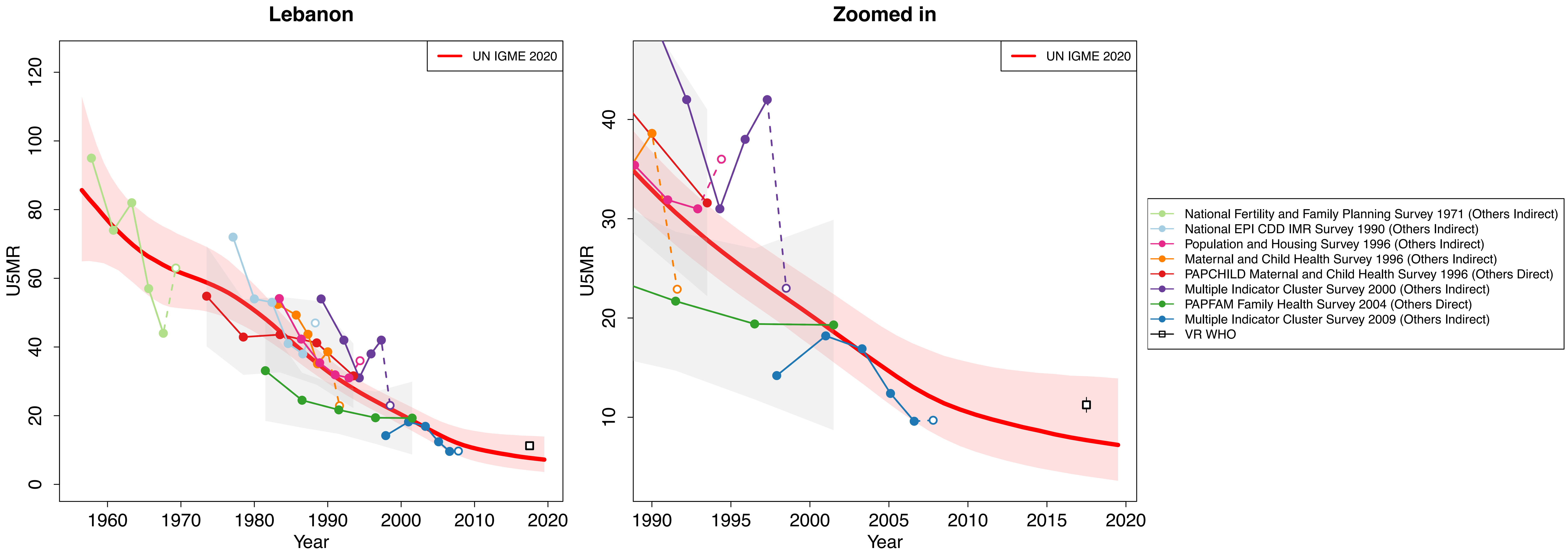

Lebanon
Zoomed in
UN IGME 2020
U5MR
Year
National Fertility and Family Planning Survey 1971 (Others Indirect)
National EPI CDD IMR Survey 1990 (Others Indirect)
Population and Housing Survey 1996 (Others Indirect)
Maternal and Child Health Survey 1996 (Others Indirect)
PAPCHILD Maternal and Child Health Survey 1996 (Others Direct)
Multiple Indicator Cluster Survey 2000 (Others Indirect)
PAPFAM Family Health Survey 2004 (Others Direct)
Multiple Indicator Cluster Survey 2009 (Others Indirect)
VR WHO

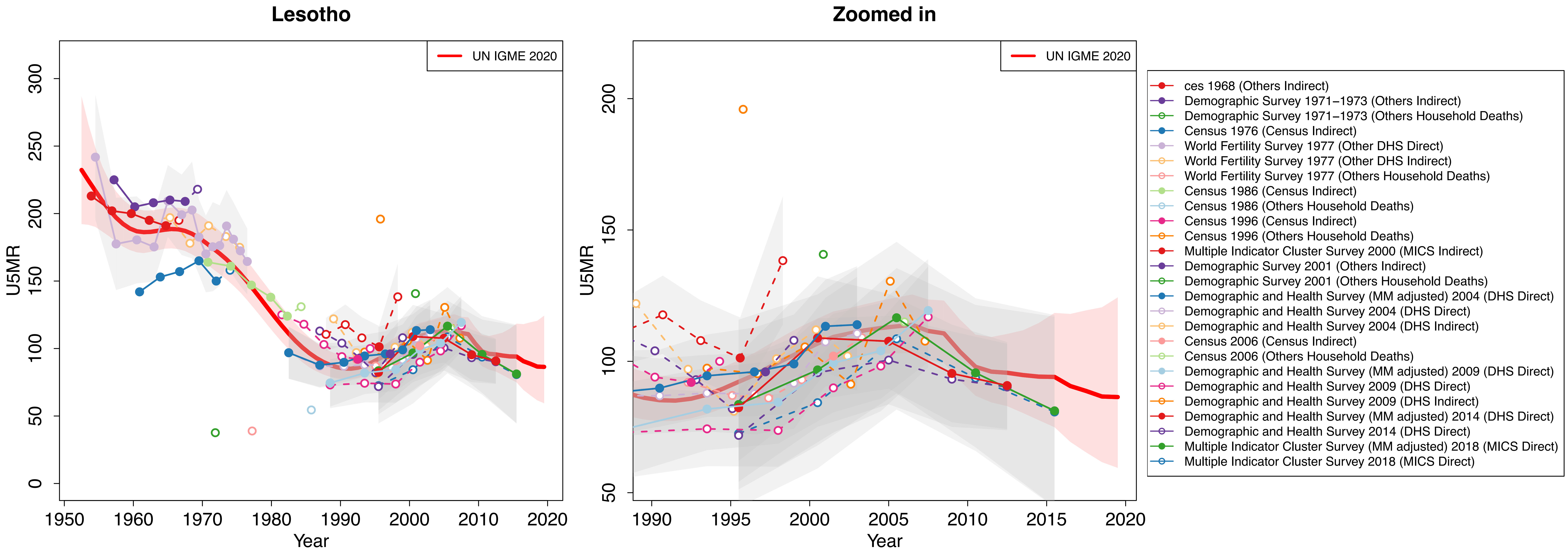

Lesotho
Zoomed in
UN IGME 2020
U5MR
Year
ces 1968 (Others Indirect)
Demographic Survey 1971–1973 (Others Indirect)
Demographic Survey 1971–1973 (Others Household Deaths)
Census 1976 (Census Indirect)
World Fertility Survey 1977 (Other DHS Direct)
World Fertility Survey 1977 (Other DHS Indirect)
World Fertility Survey 1977 (Others Household Deaths)
Census 1986 (Census Indirect)
Census 1986 (Others Household Deaths)
Census 1996 (Census Indirect)
Census 1996 (Others Household Deaths)
Multiple Indicator Cluster Survey 2000 (MICS Indirect)
Demographic Survey 2001 (Others Indirect)
Demographic Survey 2001 (Others Household Deaths)
Demographic and Health Survey (MM adjusted) 2004 (DHS Direct)
Demographic and Health Survey 2004 (DHS Direct)
Demographic and Health Survey 2004 (DHS Indirect)
Census 2006 (Census Indirect)
Census 2006 (Others Household Deaths)
Demographic and Health Survey (MM adjusted) 2009 (DHS Direct)
Demographic and Health Survey 2009 (DHS Direct)
Demographic and Health Survey 2009 (DHS Indirect)
Demographic and Health Survey (MM adjusted) 2014 (DHS Direct)
Demographic and Health Survey 2014 (DHS Direct)
Multiple Indicator Cluster Survey (MM adjusted) 2018 (MICS Direct)
Multiple Indicator Cluster Survey 2018 (MICS Direct)

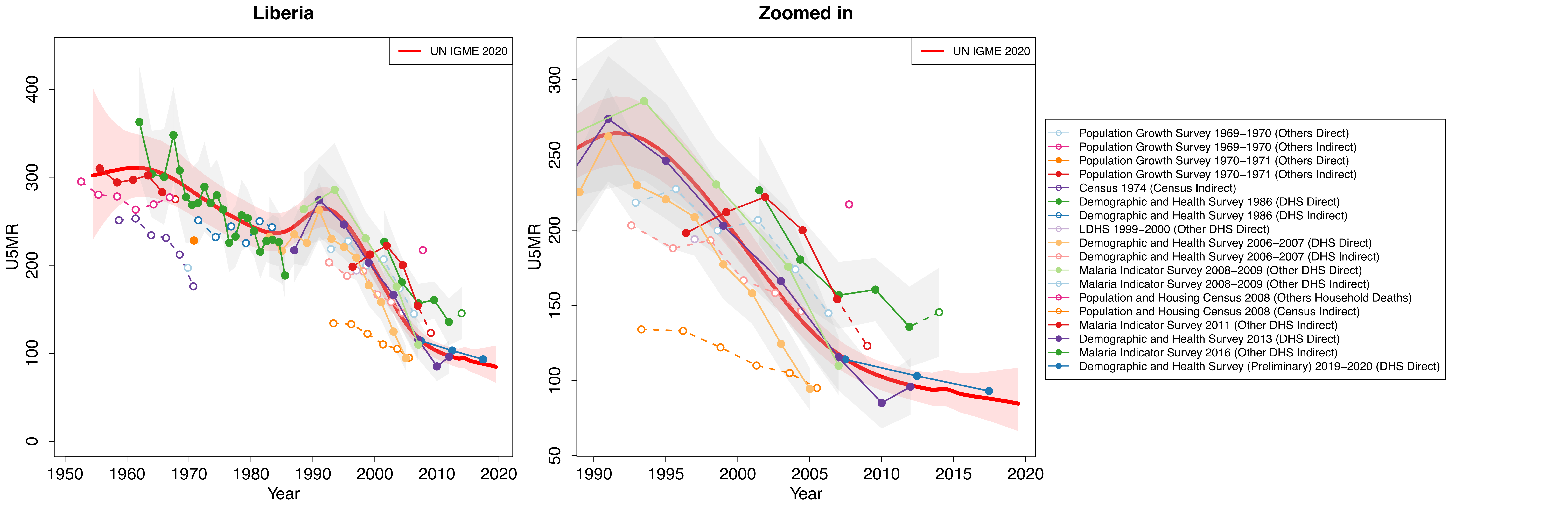

Liberia
Zoomed in
UN IGME 2020
U5MR
Year
Population Growth Survey 1969–1970 (Others Direct)
Population Growth Survey 1969–1970 (Others Indirect)
Population Growth Survey 1970–1971 (Others Direct)
Population Growth Survey 1970–1971 (Others Indirect)
Census 1974 (Census Indirect)
Demographic and Health Survey 1986 (DHS Direct)
Demographic and Health Survey 1986 (DHS Indirect)
LDHS 1999–2000 (Other DHS Direct)
Demographic and Health Survey 2006–2007 (DHS Direct)
Demographic and Health Survey 2006–2007 (DHS Indirect)
Malaria Indicator Survey 2008–2009 (Other DHS Direct)
Malaria Indicator Survey 2008–2009 (Other DHS Indirect)
Population and Housing Census 2008 (Others Household Deaths)
Population and Housing Census 2008 (Census Indirect)
Malaria Indicator Survey 2011 (Other DHS Indirect)
Demographic and Health Survey 2013 (DHS Direct)
Malaria Indicator Survey 2016 (Other DHS Indirect)
Demographic and Health Survey (Preliminary) 2019–2020 (DHS Direct)

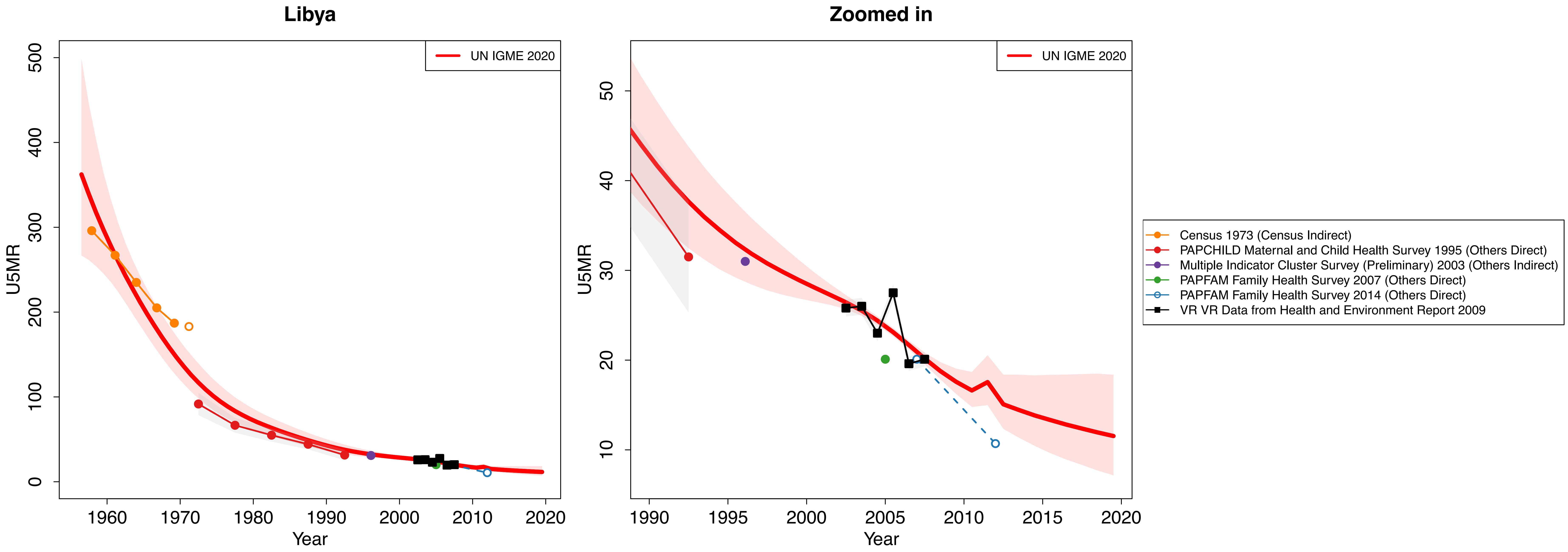

Libya
Zoomed in
UN IGME 2020
U5MR
Year
Census 1973 (Census Indirect)
PAPCHILD Maternal and Child Health Survey 1995 (Others Direct)
Multiple Indicator Cluster Survey (Preliminary) 2003 (Others Indirect)
PAPFAM Family Health Survey 2007 (Others Direct)
PAPFAM Family Health Survey 2014 (Others Direct)
VR VR Data from Health and Environment Report 2009

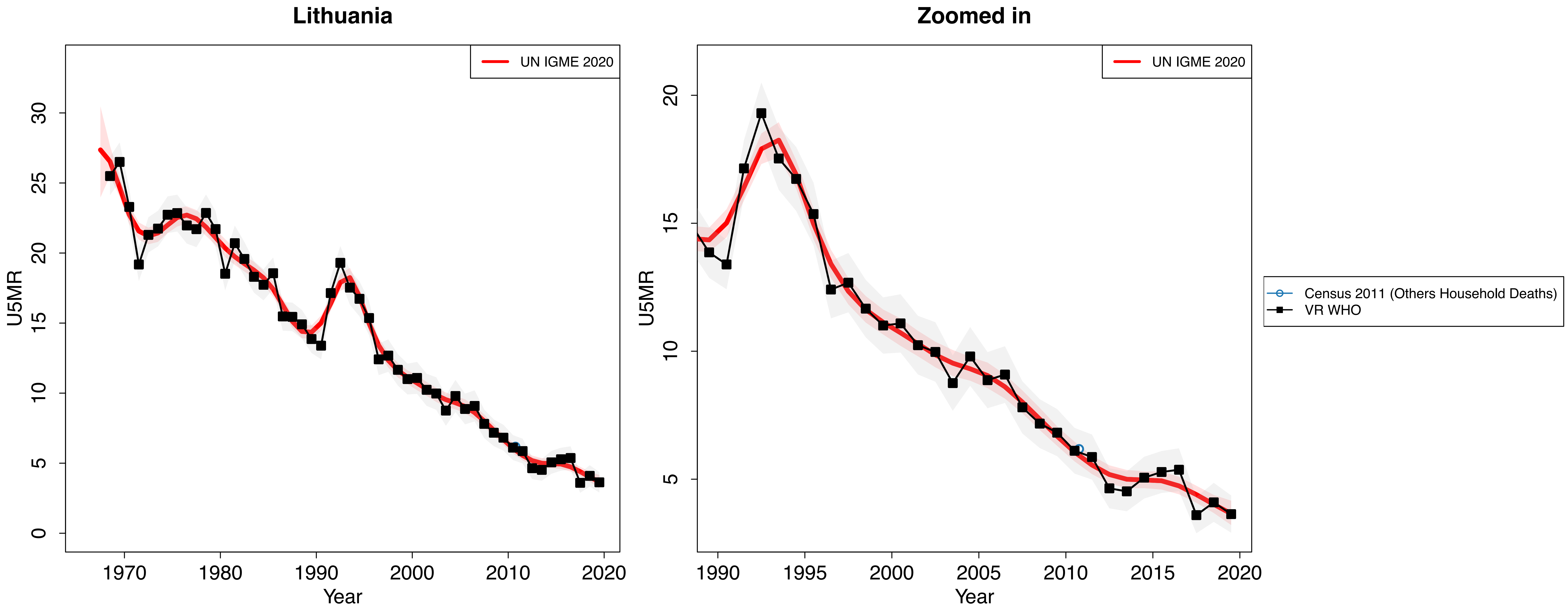

Lithuania
Zoomed in
UN IGME 2020
UN IGME 2020
U5MR
U5MR
Year
Year
0
5
10
15
20
25
30
1970
1980
1990
2000
2010
2020
5
10
15
20
1990
1995
2000
2005
2010
2015
2020
Census 2011 (Others Household Deaths)
VR WHO

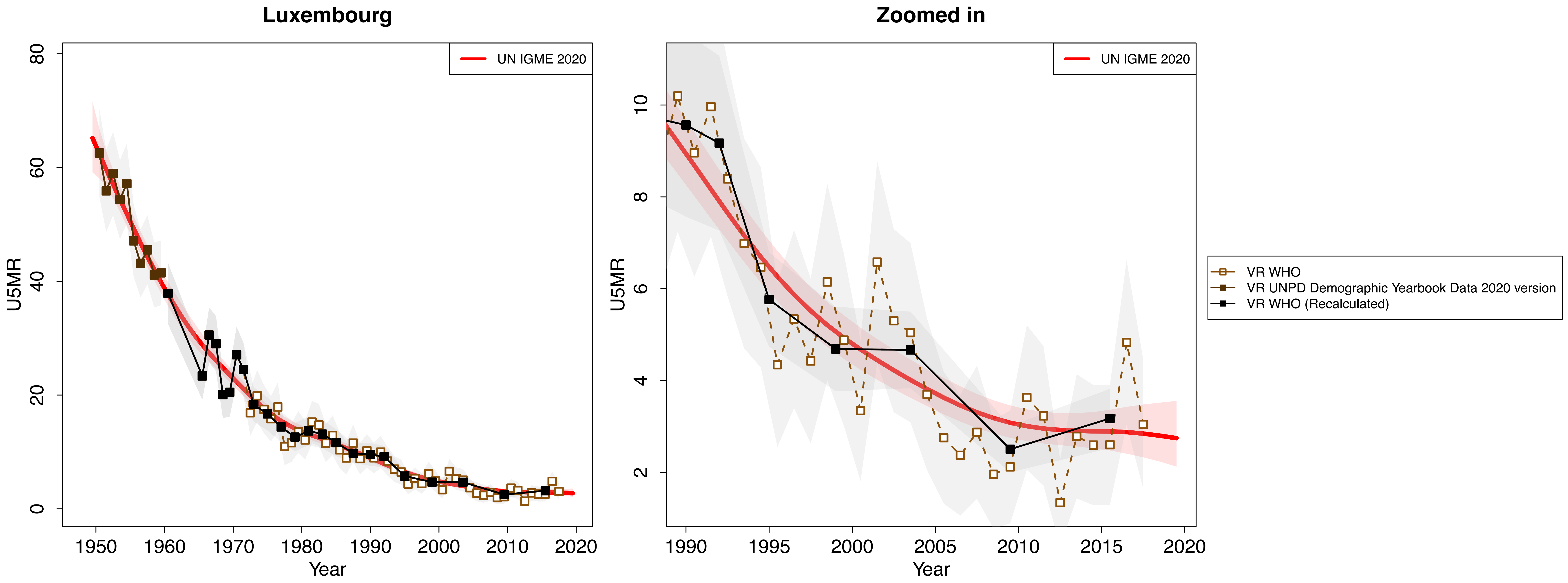

Luxembourg
Zoomed in
UN IGME 2020
UN IGME 2020
U5MR
U5MR
Year
Year
0
20
40
60
80
1950
1960
1970
1980
1990
2000
2010
2020
2
4
6
8
10
1990
1995
2000
2005
2010
2015
2020
VR WHO
VR UNPD Demographic Yearbook Data 2020 version
VR WHO (Recalculated)

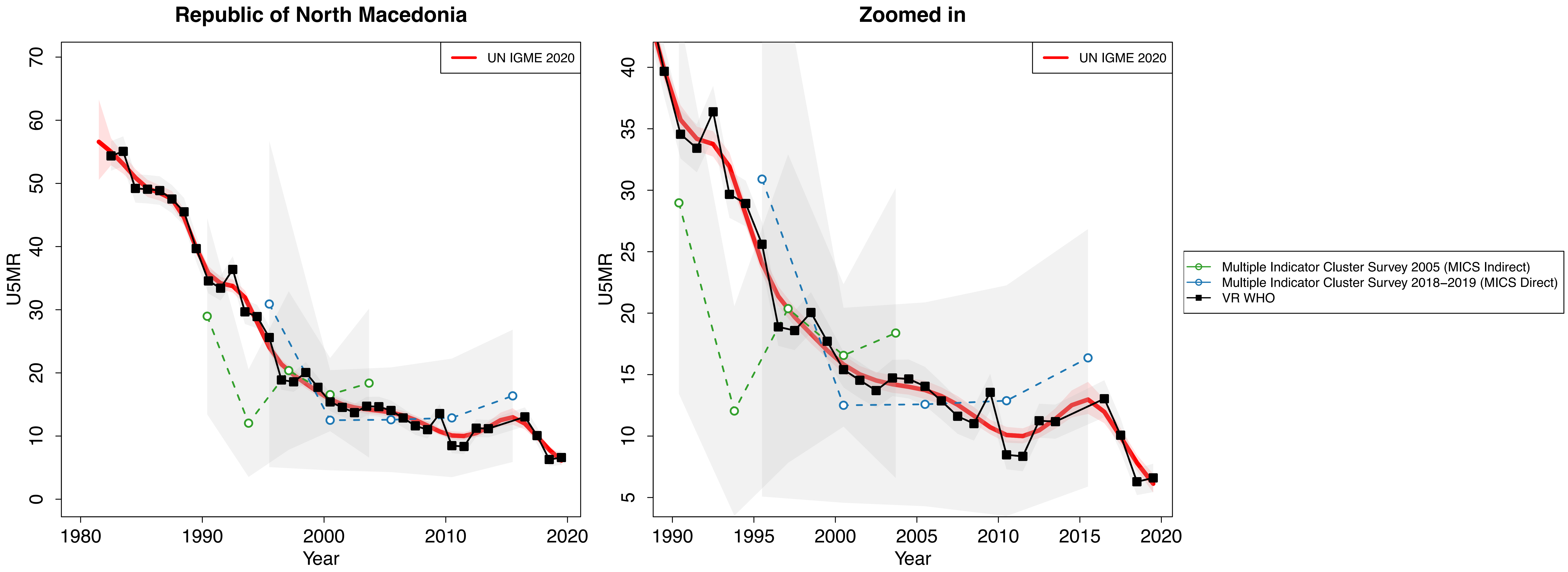

Republic of North Macedonia
Zoomed in
UN IGME 2020
U5MR
Year
Multiple Indicator Cluster Survey 2005 (MICS Indirect)
Multiple Indicator Cluster Survey 2018–2019 (MICS Direct)
VR WHO

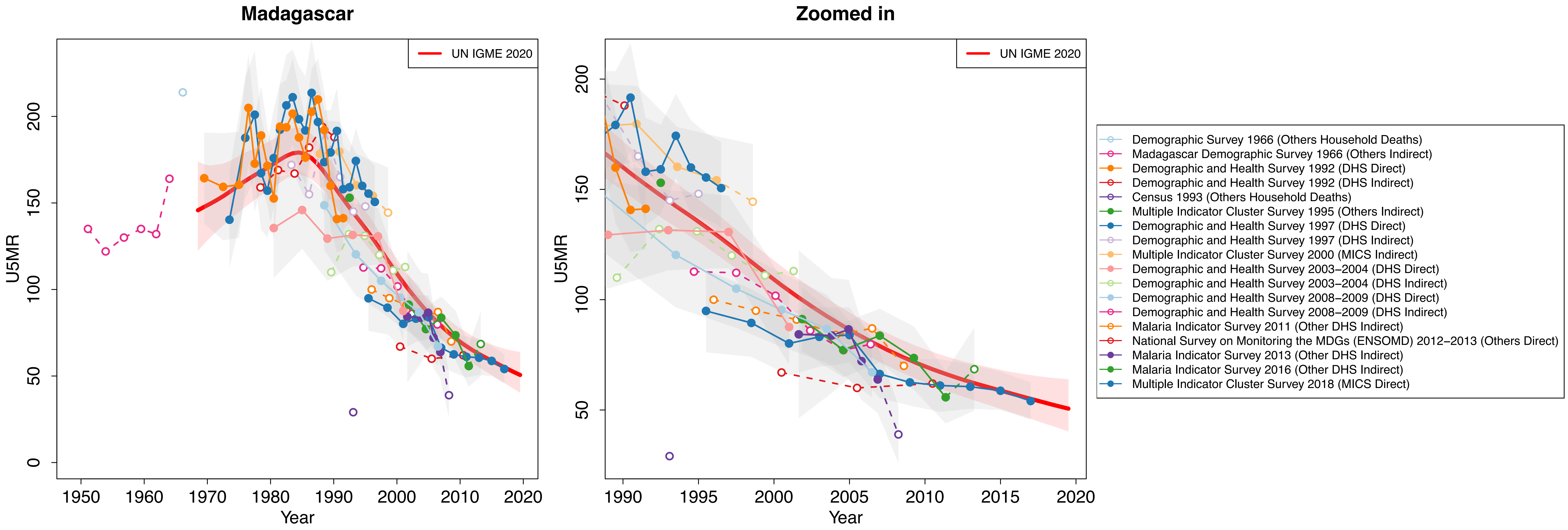

Madagascar
Zoomed in
UN IGME 2020
U5MR
Year
Demographic Survey 1966 (Others Household Deaths)
Madagascar Demographic Survey 1966 (Others Indirect)
Demographic and Health Survey 1992 (DHS Direct)
Demographic and Health Survey 1992 (DHS Indirect)
Census 1993 (Others Household Deaths)
Multiple Indicator Cluster Survey 1995 (Others Indirect)
Demographic and Health Survey 1997 (DHS Direct)
Demographic and Health Survey 1997 (DHS Indirect)
Multiple Indicator Cluster Survey 2000 (MICS Indirect)
Demographic and Health Survey 2003–2004 (DHS Direct)
Demographic and Health Survey 2003–2004 (DHS Indirect)
Demographic and Health Survey 2008–2009 (DHS Direct)
Demographic and Health Survey 2008–2009 (DHS Indirect)
Malaria Indicator Survey 2011 (Other DHS Indirect)
National Survey on Monitoring the MDGs (ENSOMD) 2012–2013 (Others Direct)
Malaria Indicator Survey 2013 (Other DHS Indirect)
Malaria Indicator Survey 2016 (Other DHS Indirect)
Multiple Indicator Cluster Survey 2018 (MICS Direct)

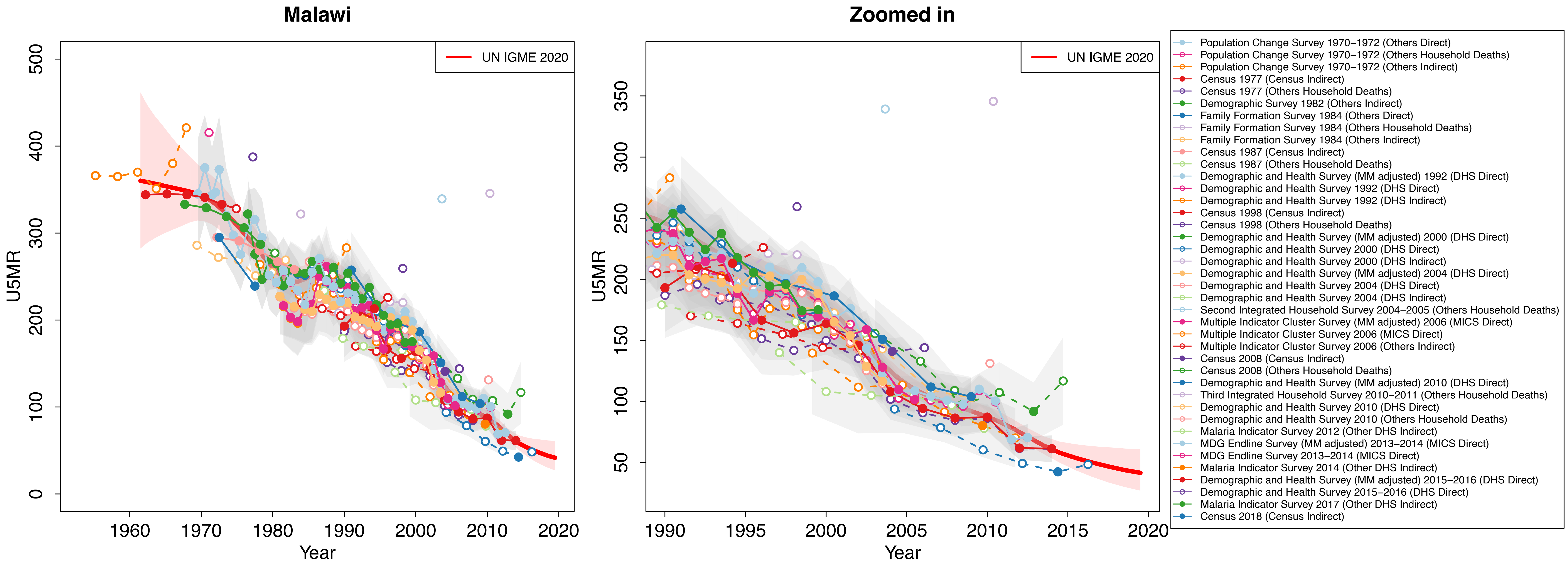
Malawi
Zoomed in
U5MR
Year
UN IGME 2020
Population Change Survey 1970–1972 (Others Direct)
Population Change Survey 1970–1972 (Others Household Deaths)
Population Change Survey 1970–1972 (Others Indirect)
Census 1977 (Census Indirect)
Census 1977 (Others Household Deaths)
Demographic Survey 1982 (Others Indirect)
Family Formation Survey 1984 (Others Direct)
Family Formation Survey 1984 (Others Household Deaths)
Family Formation Survey 1984 (Others Indirect)
Census 1987 (Census Indirect)
Census 1987 (Others Household Deaths)
Demographic and Health Survey (MM adjusted) 1992 (DHS Direct)
Demographic and Health Survey 1992 (DHS Direct)
Demographic and Health Survey 1992 (DHS Indirect)
Census 1998 (Census Indirect)
Census 1998 (Others Household Deaths)
Demographic and Health Survey (MM adjusted) 2000 (DHS Direct)
Demographic and Health Survey 2000 (DHS Direct)
Demographic and Health Survey 2000 (DHS Indirect)
Demographic and Health Survey (MM adjusted) 2004 (DHS Direct)
Demographic and Health Survey 2004 (DHS Direct)
Demographic and Health Survey 2004 (DHS Indirect)
Second Integrated Household Survey 2004–2005 (Others Household Deaths)
Multiple Indicator Cluster Survey (MM adjusted) 2006 (MICS Direct)
Multiple Indicator Cluster Survey 2006 (MICS Direct)
Multiple Indicator Cluster Survey 2006 (Others Indirect)
Census 2008 (Census Indirect)
Census 2008 (Others Household Deaths)
Demographic and Health Survey (MM adjusted) 2010 (DHS Direct)
Third Integrated Household Survey 2010–2011 (Others Household Deaths)
Demographic and Health Survey 2010 (DHS Direct)
Demographic and Health Survey 2010 (Others Household Deaths)
Malaria Indicator Survey 2012 (Other DHS Indirect)
MDG Endline Survey (MM adjusted) 2013–2014 (MICS Direct)
MDG Endline Survey 2013–2014 (MICS Direct)
Malaria Indicator Survey 2014 (Other DHS Indirect)
Demographic and Health Survey (MM adjusted) 2015–2016 (DHS Direct)
Demographic and Health Survey 2015–2016 (DHS Direct)
Malaria Indicator Survey 2017 (Other DHS Indirect)
Census 2018 (Census Indirect)

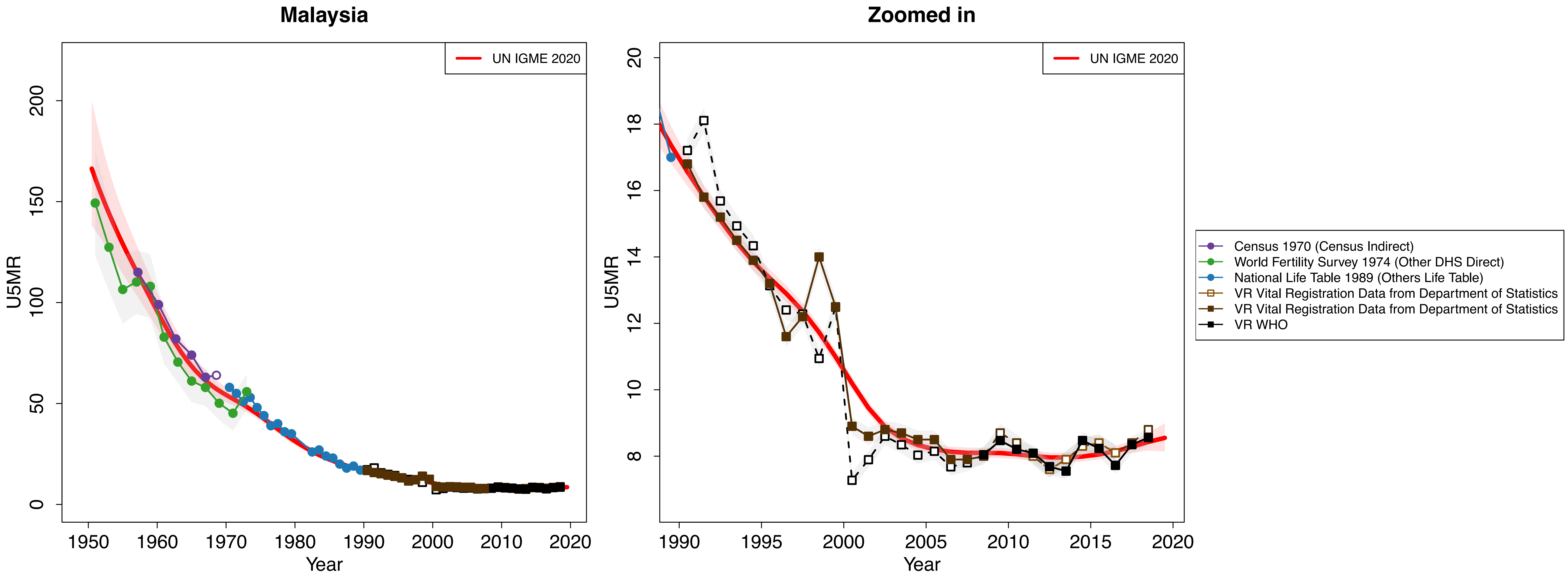

Malaysia
Zoomed in
UN IGME 2020
U5MR
Year
Census 1970 (Census Indirect)
World Fertility Survey 1974 (Other DHS Direct)
National Life Table 1989 (Others Life Table)
VR Vital Registration Data from Department of Statistics
VR Vital Registration Data from Department of Statistics
VR WHO

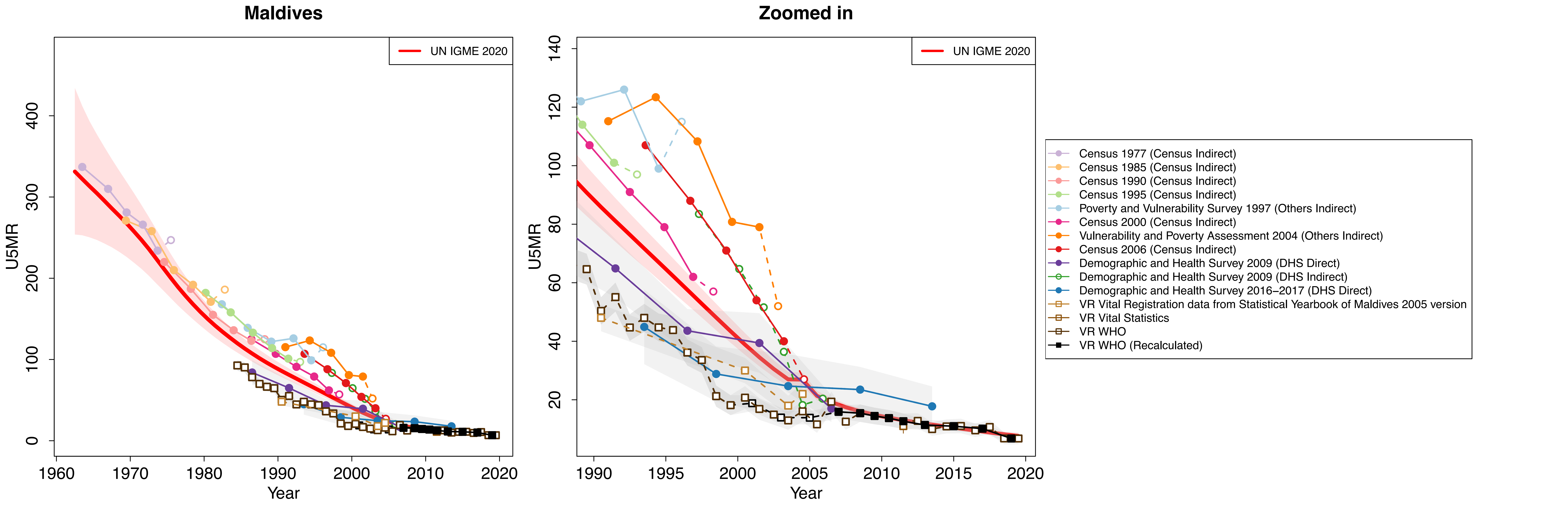

Maldives
Zoomed in
UN IGME 2020
U5MR
Year
Census 1977 (Census Indirect)
Census 1985 (Census Indirect)
Census 1990 (Census Indirect)
Census 1995 (Census Indirect)
Poverty and Vulnerability Survey 1997 (Others Indirect)
Census 2000 (Census Indirect)
Vulnerability and Poverty Assessment 2004 (Others Indirect)
Census 2006 (Census Indirect)
Demographic and Health Survey 2009 (DHS Direct)
Demographic and Health Survey 2009 (DHS Indirect)
Demographic and Health Survey 2016–2017 (DHS Direct)
VR Vital Registration data from Statistical Yearbook of Maldives 2005 version
VR Vital Statistics
VR WHO
VR WHO (Recalculated)

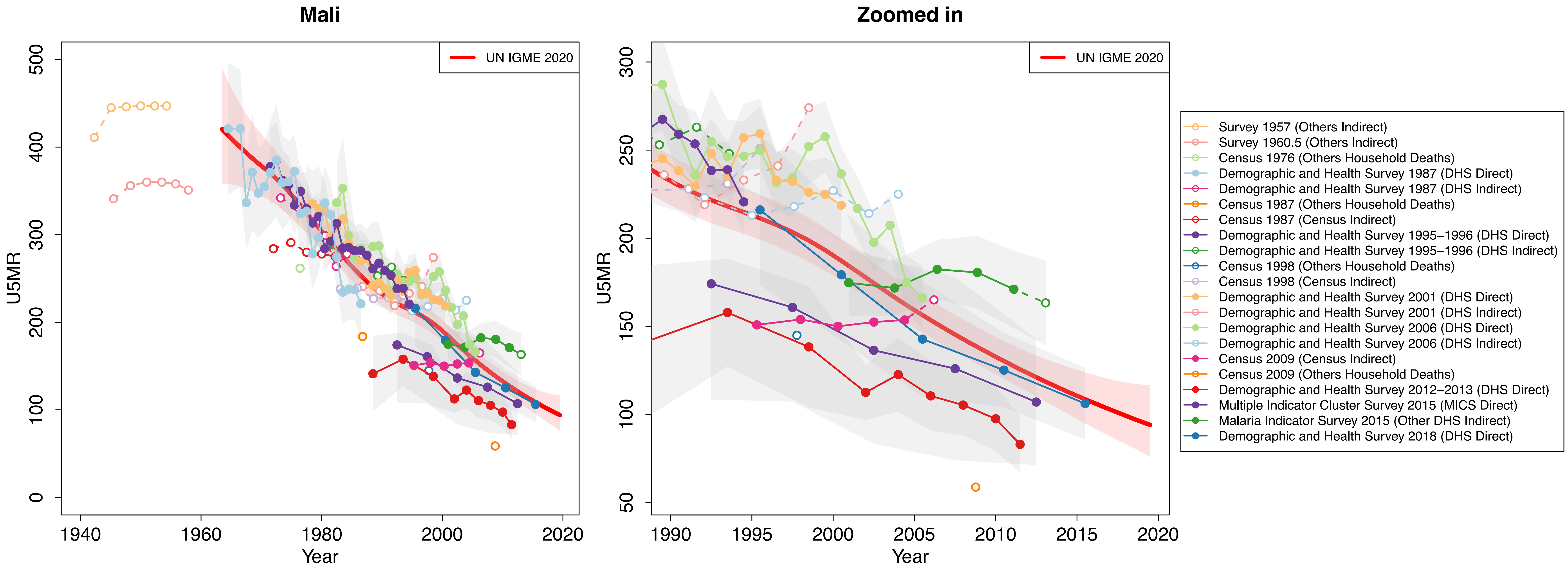

Mali
Zoomed in
UN IGME 2020
U5MR
Year
Survey 1957 (Others Indirect)
Survey 1960.5 (Others Indirect)
Census 1976 (Others Household Deaths)
Demographic and Health Survey 1987 (DHS Direct)
Demographic and Health Survey 1987 (DHS Indirect)
Census 1987 (Others Household Deaths)
Census 1987 (Census Indirect)
Demographic and Health Survey 1995–1996 (DHS Direct)
Demographic and Health Survey 1995–1996 (DHS Indirect)
Census 1998 (Others Household Deaths)
Census 1998 (Census Indirect)
Demographic and Health Survey 2001 (DHS Direct)
Demographic and Health Survey 2001 (DHS Indirect)
Demographic and Health Survey 2006 (DHS Direct)
Demographic and Health Survey 2006 (DHS Indirect)
Census 2009 (Census Indirect)
Census 2009 (Others Household Deaths)
Demographic and Health Survey 2012–2013 (DHS Direct)
Multiple Indicator Cluster Survey 2015 (MICS Direct)
Malaria Indicator Survey 2015 (Other DHS Indirect)
Demographic and Health Survey 2018 (DHS Direct)

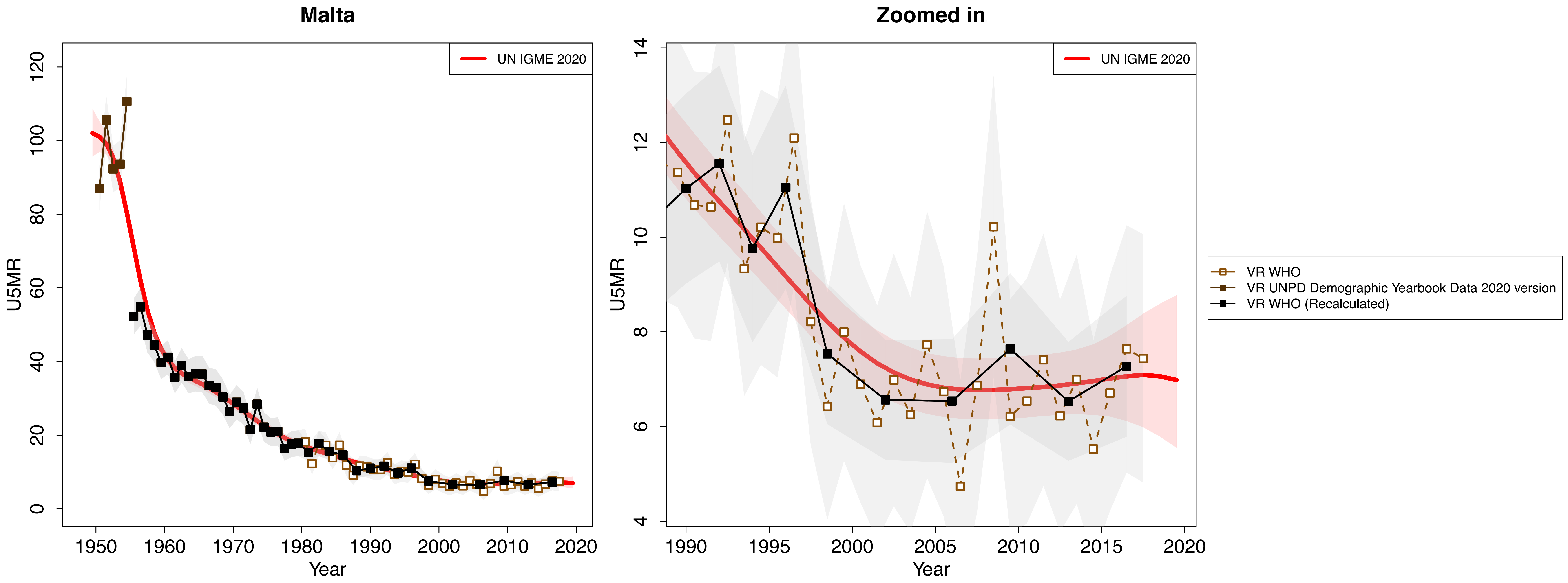
Malta
UN IGME 2020
U5MR
0
20
40
60
80
100
120
1950
1960
1970
1980
1990
2000
2010
2020
Year
Zoomed in
UN IGME 2020
U5MR
4
6
8
10
12
14
1990
1995
2000
2005
2010
2015
2020
Year
VR WHO
VR UNPD Demographic Yearbook Data 2020 version
VR WHO (Recalculated)

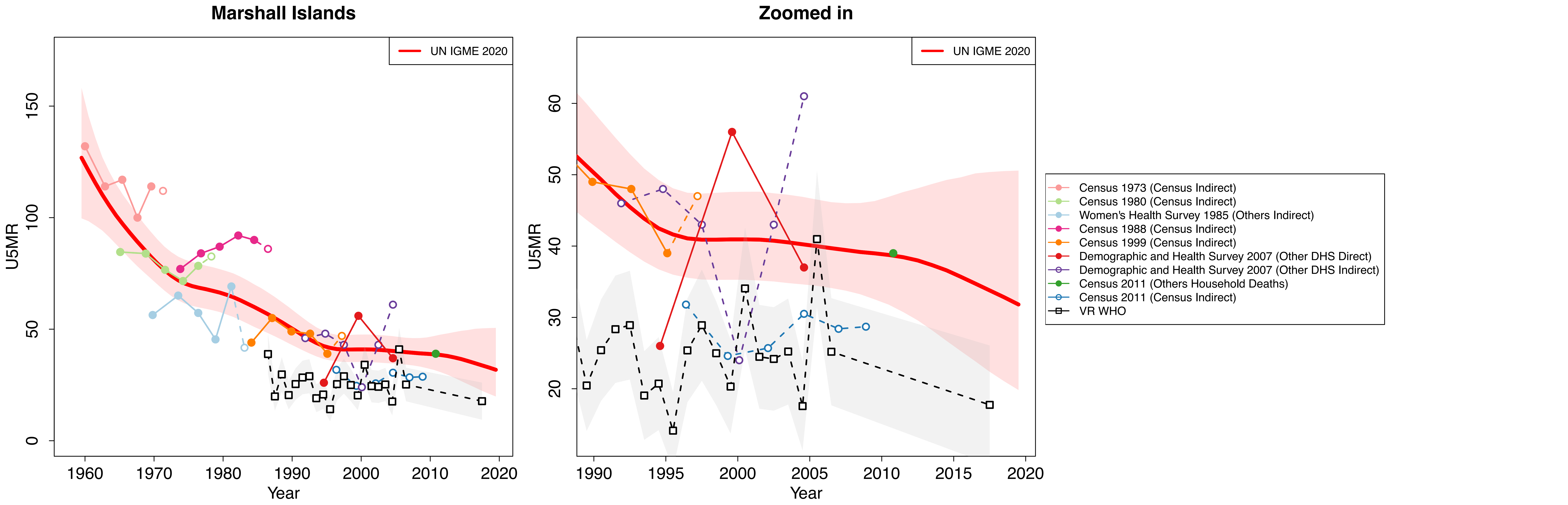

Marshall Islands
Zoomed in
UN IGME 2020
U5MR
Year
Census 1973 (Census Indirect)
Census 1980 (Census Indirect)
Women's Health Survey 1985 (Others Indirect)
Census 1988 (Census Indirect)
Census 1999 (Census Indirect)
Demographic and Health Survey 2007 (Other DHS Direct)
Demographic and Health Survey 2007 (Other DHS Indirect)
Census 2011 (Others Household Deaths)
Census 2011 (Census Indirect)
VR WHO

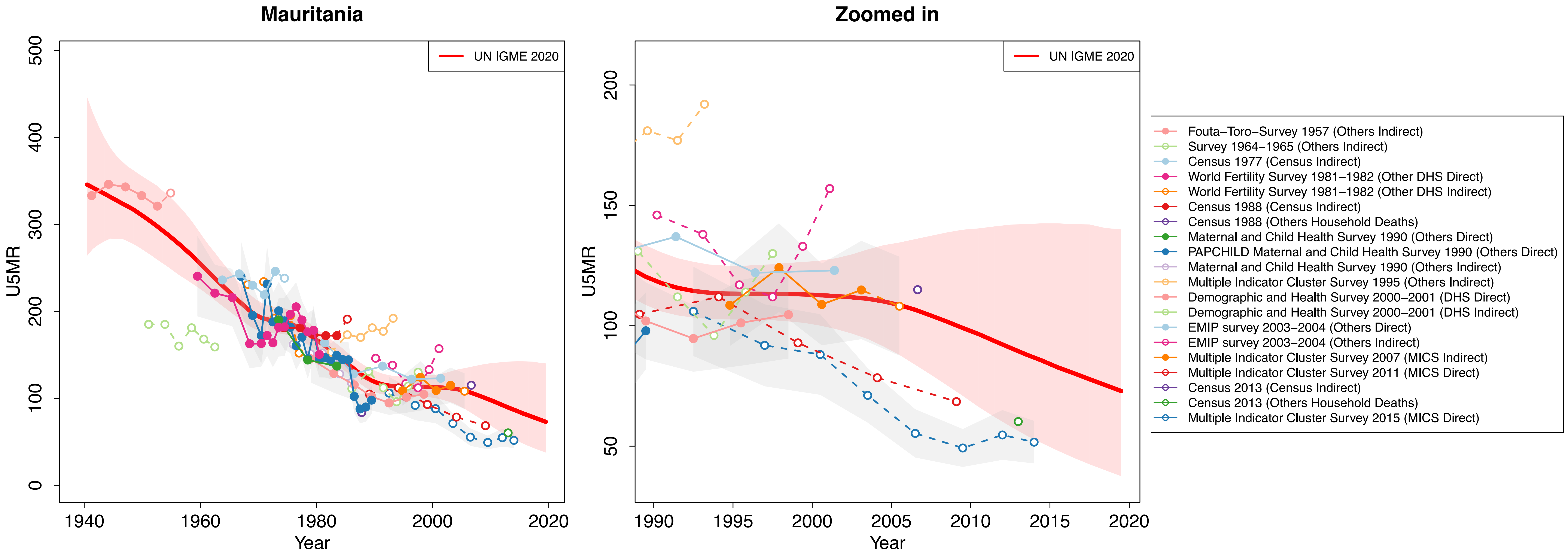

Mauritania
Zoomed in
UN IGME 2020
U5MR
Year
Fouta–Toro–Survey 1957 (Others Indirect)
Survey 1964–1965 (Others Indirect)
Census 1977 (Census Indirect)
World Fertility Survey 1981–1982 (Other DHS Direct)
World Fertility Survey 1981–1982 (Other DHS Indirect)
Census 1988 (Census Indirect)
Census 1988 (Others Household Deaths)
Maternal and Child Health Survey 1990 (Others Direct)
PAPCHILD Maternal and Child Health Survey 1990 (Others Direct)
Maternal and Child Health Survey 1990 (Others Indirect)
Multiple Indicator Cluster Survey 1995 (Others Indirect)
Demographic and Health Survey 2000–2001 (DHS Direct)
Demographic and Health Survey 2000–2001 (DHS Indirect)
EMIP survey 2003–2004 (Others Direct)
EMIP survey 2003–2004 (Others Indirect)
Multiple Indicator Cluster Survey 2007 (MICS Indirect)
Multiple Indicator Cluster Survey 2011 (MICS Direct)
Census 2013 (Census Indirect)
Census 2013 (Others Household Deaths)
Multiple Indicator Cluster Survey 2015 (MICS Direct)

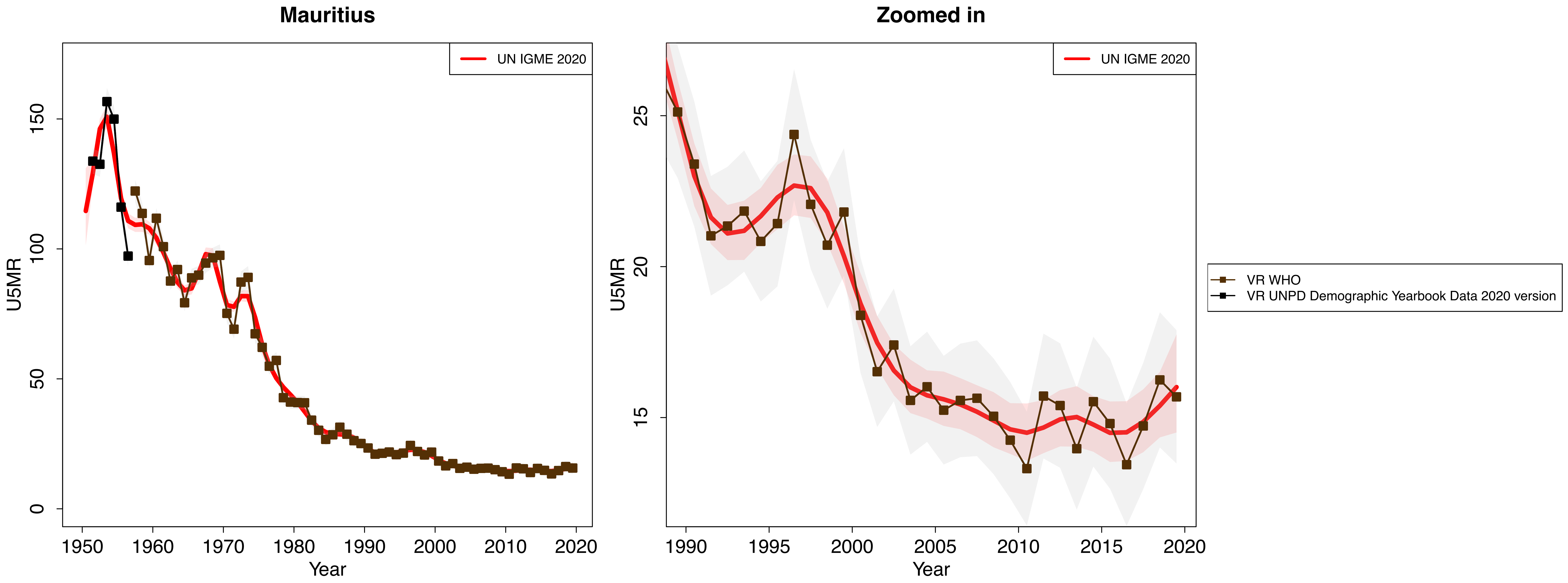

Mauritius
Zoomed in
UN IGME 2020
U5MR
Year
VR WHO
VR UNPD Demographic Yearbook Data 2020 version

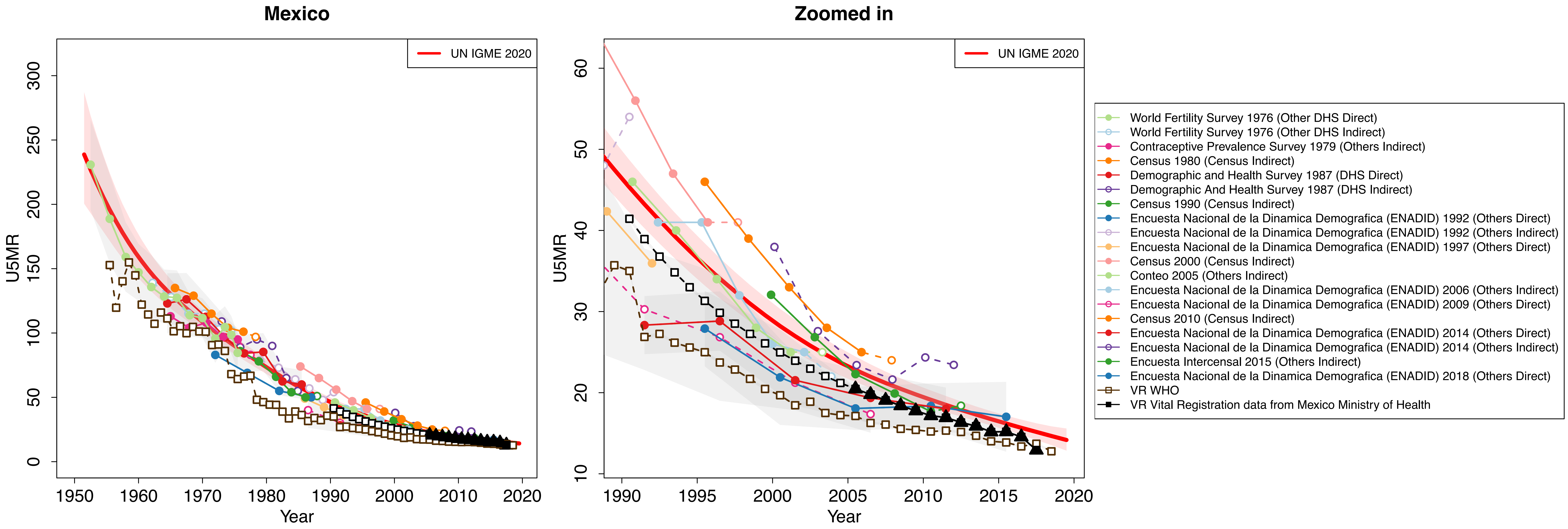

Mexico
Zoomed in
UN IGME 2020
U5MR
Year
World Fertility Survey 1976 (Other DHS Direct)
World Fertility Survey 1976 (Other DHS Indirect)
Contraceptive Prevalence Survey 1979 (Others Indirect)
Census 1980 (Census Indirect)
Demographic and Health Survey 1987 (DHS Direct)
Demographic And Health Survey 1987 (DHS Indirect)
Census 1990 (Census Indirect)
Encuesta Nacional de la Dinamica Demografica (ENADID) 1992 (Others Direct)
Encuesta Nacional de la Dinamica Demografica (ENADID) 1992 (Others Indirect)
Encuesta Nacional de la Dinamica Demografica (ENADID) 1997 (Others Direct)
Census 2000 (Census Indirect)
Conteo 2005 (Others Indirect)
Encuesta Nacional de la Dinamica Demografica (ENADID) 2006 (Others Indirect)
Encuesta Nacional de la Dinamica Demografica (ENADID) 2009 (Others Direct)
Census 2010 (Census Indirect)
Encuesta Nacional de la Dinamica Demografica (ENADID) 2014 (Others Direct)
Encuesta Nacional de la Dinamica Demografica (ENADID) 2014 (Others Indirect)
Encuesta Intercensal 2015 (Others Indirect)
Encuesta Nacional de la Dinamica Demografica (ENADID) 2018 (Others Direct)
VR WHO
VR Vital Registration data from Mexico Ministry of Health

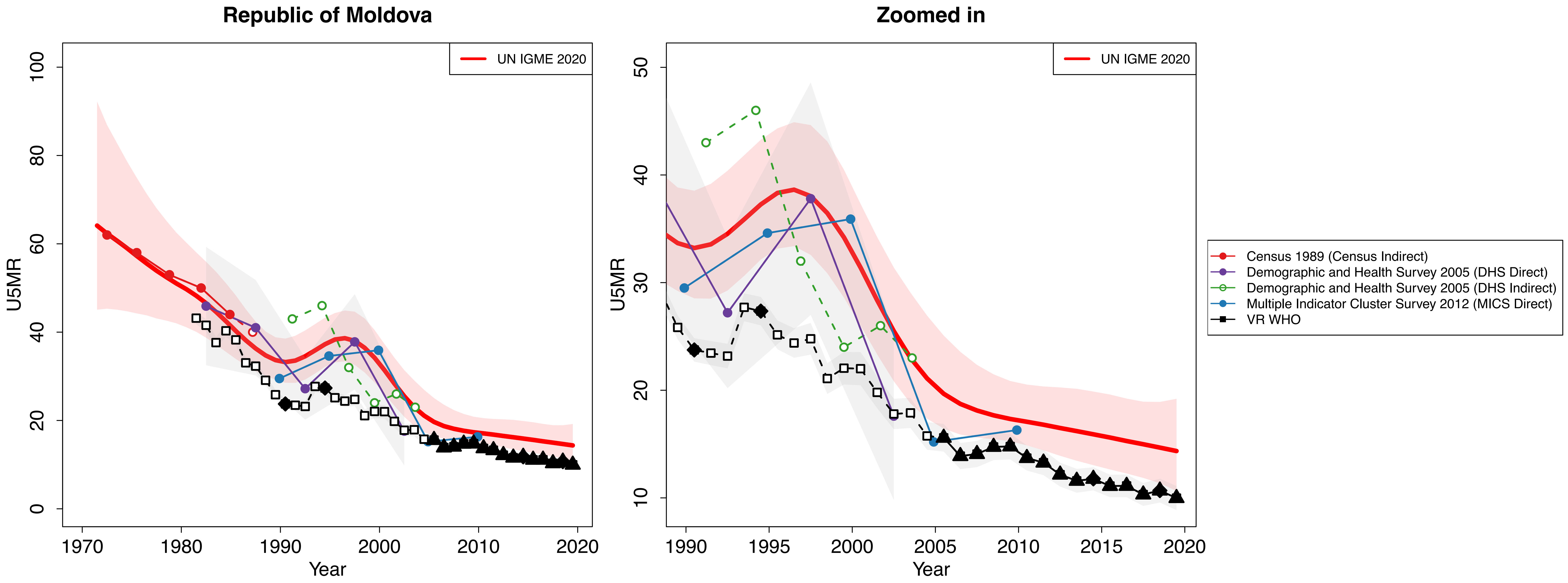

Republic of Moldova
Zoomed in
UN IGME 2020
U5MR
Year
Census 1989 (Census Indirect)
Demographic and Health Survey 2005 (DHS Direct)
Demographic and Health Survey 2005 (DHS Indirect)
Multiple Indicator Cluster Survey 2012 (MICS Direct)
VR WHO

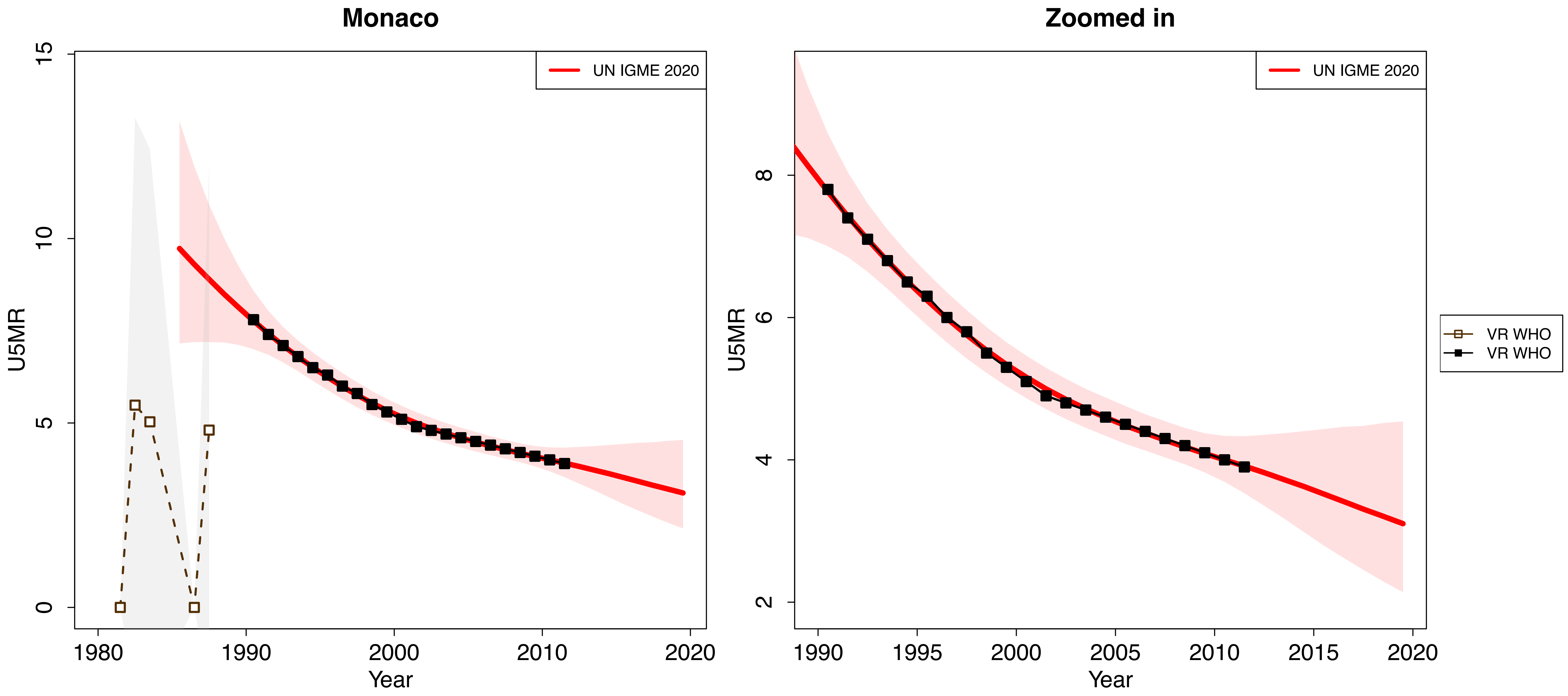

Monaco
Zoomed in
UN IGME 2020
UN IGME 2020
U5MR
U5MR
Year
Year
VR WHO
VR WHO

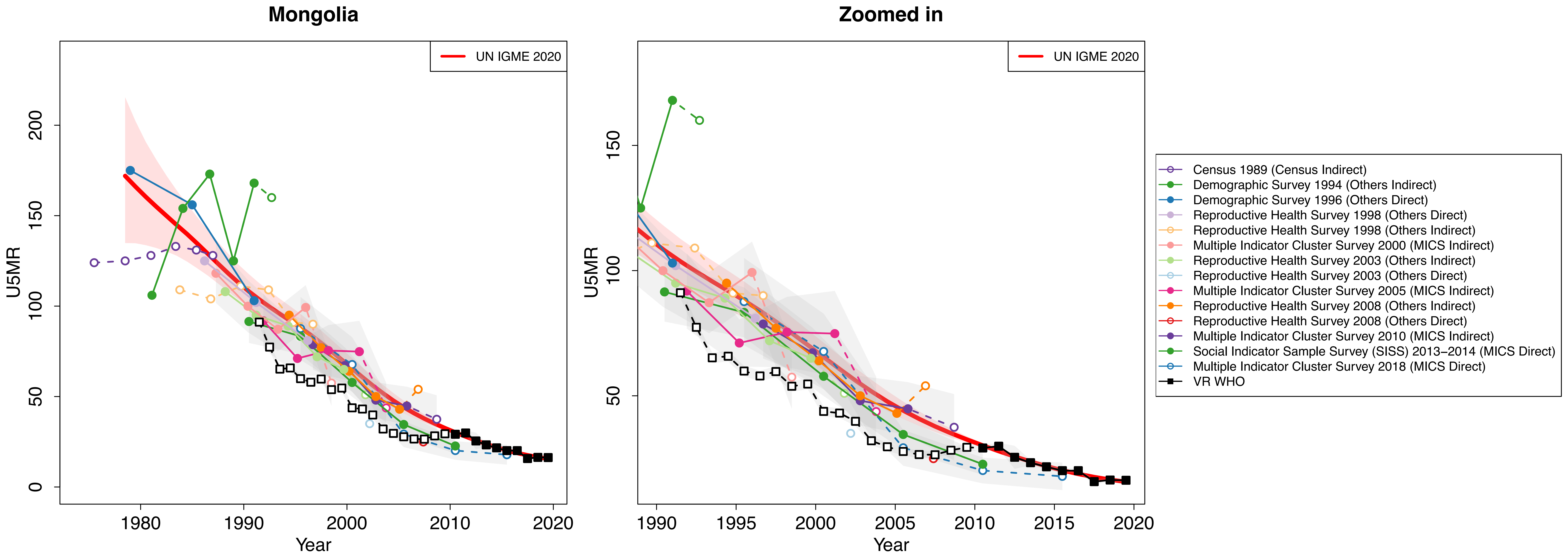

Mongolia
Zoomed in
UN IGME 2020
U5MR
Year
0
50
100
150
200
1980
1990
2000
2010
2020
1995
2005
2015
Census 1989 (Census Indirect)
Demographic Survey 1994 (Others Indirect)
Demographic Survey 1996 (Others Direct)
Reproductive Health Survey 1998 (Others Direct)
Reproductive Health Survey 1998 (Others Indirect)
Multiple Indicator Cluster Survey 2000 (MICS Indirect)
Reproductive Health Survey 2003 (Others Indirect)
Reproductive Health Survey 2003 (Others Direct)
Multiple Indicator Cluster Survey 2005 (MICS Indirect)
Reproductive Health Survey 2008 (Others Indirect)
Reproductive Health Survey 2008 (Others Direct)
Multiple Indicator Cluster Survey 2010 (MICS Indirect)
Social Indicator Sample Survey (SISS) 2013–2014 (MICS Direct)
Multiple Indicator Cluster Survey 2018 (MICS Direct)
VR WHO

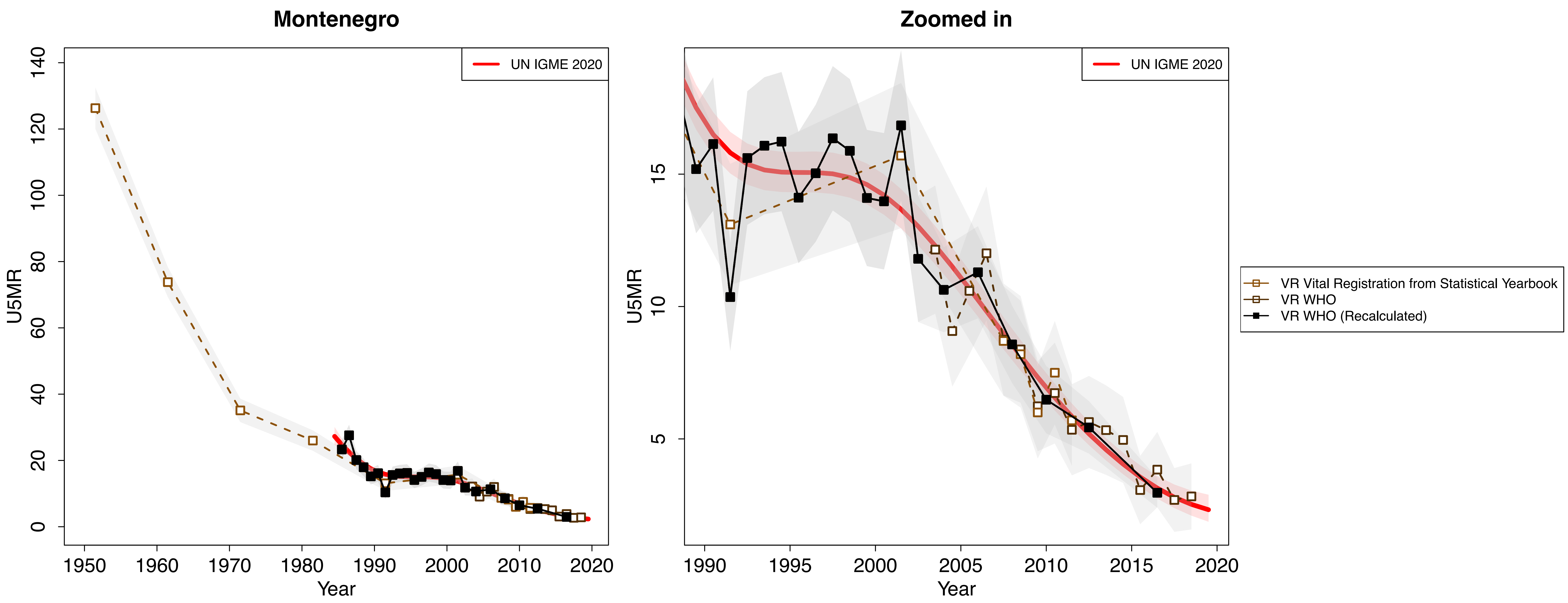

Montenegro
Zoomed in
UN IGME 2020
U5MR
Year
VR Vital Registration from Statistical Yearbook
VR WHO
VR WHO (Recalculated)

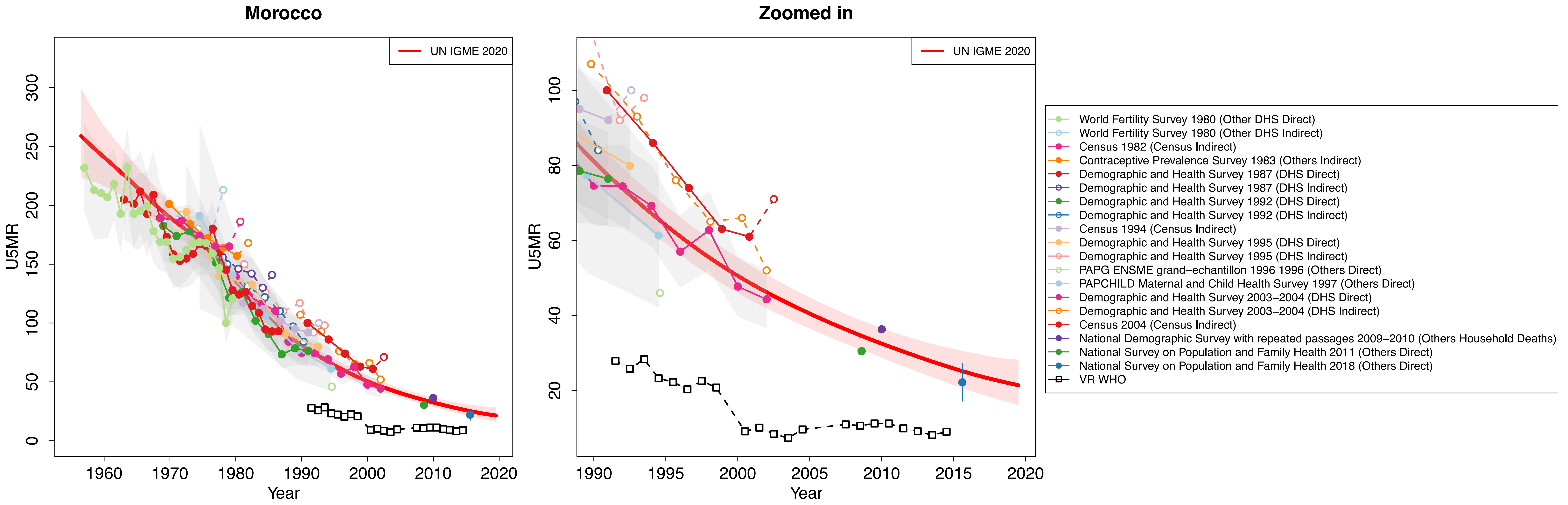

Morocco
Zoomed in
UN IGME 2020
U5MR
Year
World Fertility Survey 1980 (Other DHS Direct)
World Fertility Survey 1980 (Other DHS Indirect)
Census 1982 (Census Indirect)
Contraceptive Prevalence Survey 1983 (Others Indirect)
Demographic and Health Survey 1987 (DHS Direct)
Demographic and Health Survey 1987 (DHS Indirect)
Demographic and Health Survey 1992 (DHS Direct)
Demographic and Health Survey 1992 (DHS Indirect)
Census 1994 (Census Indirect)
Demographic and Health Survey 1995 (DHS Direct)
Demographic and Health Survey 1995 (DHS Indirect)
PAPG ENSME grand−echantillon 1996 1996 (Others Direct)
PAPCHILD Maternal and Child Health Survey 1997 (Others Direct)
Demographic and Health Survey 2003−2004 (DHS Direct)
Demographic and Health Survey 2003−2004 (DHS Indirect)
Census 2004 (Census Indirect)
National Demographic Survey with repeated passages 2009−2010 (Others Household Deaths)
National Survey on Population and Family Health 2011 (Others Direct)
National Survey on Population and Family Health 2018 (Others Direct)
VR WHO

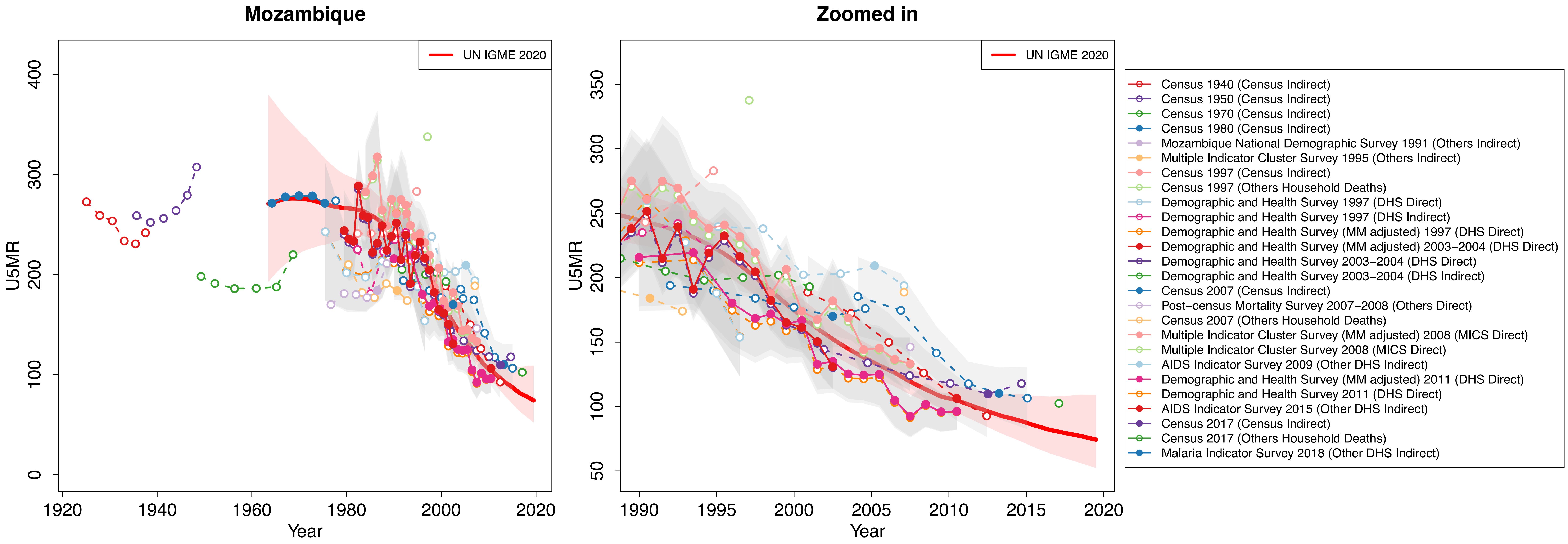

Mozambique
Zoomed in
UN IGME 2020
U5MR
Year
Census 1940 (Census Indirect)
Census 1950 (Census Indirect)
Census 1970 (Census Indirect)
Census 1980 (Census Indirect)
Mozambique National Demographic Survey 1991 (Others Indirect)
Multiple Indicator Cluster Survey 1995 (Others Indirect)
Census 1997 (Census Indirect)
Census 1997 (Others Household Deaths)
Demographic and Health Survey 1997 (DHS Direct)
Demographic and Health Survey 1997 (DHS Indirect)
Demographic and Health Survey (MM adjusted) 1997 (DHS Direct)
Demographic and Health Survey (MM adjusted) 2003–2004 (DHS Direct)
Demographic and Health Survey 2003–2004 (DHS Direct)
Demographic and Health Survey 2003–2004 (DHS Indirect)
Census 2007 (Census Indirect)
Post–census Mortality Survey 2007–2008 (Others Direct)
Census 2007 (Others Household Deaths)
Multiple Indicator Cluster Survey (MM adjusted) 2008 (MICS Direct)
Multiple Indicator Cluster Survey 2008 (MICS Direct)
AIDS Indicator Survey 2009 (Other DHS Indirect)
Demographic and Health Survey (MM adjusted) 2011 (DHS Direct)
Demographic and Health Survey 2011 (DHS Direct)
AIDS Indicator Survey 2015 (Other DHS Indirect)
Census 2017 (Census Indirect)
Census 2017 (Others Household Deaths)
Malaria Indicator Survey 2018 (Other DHS Indirect)

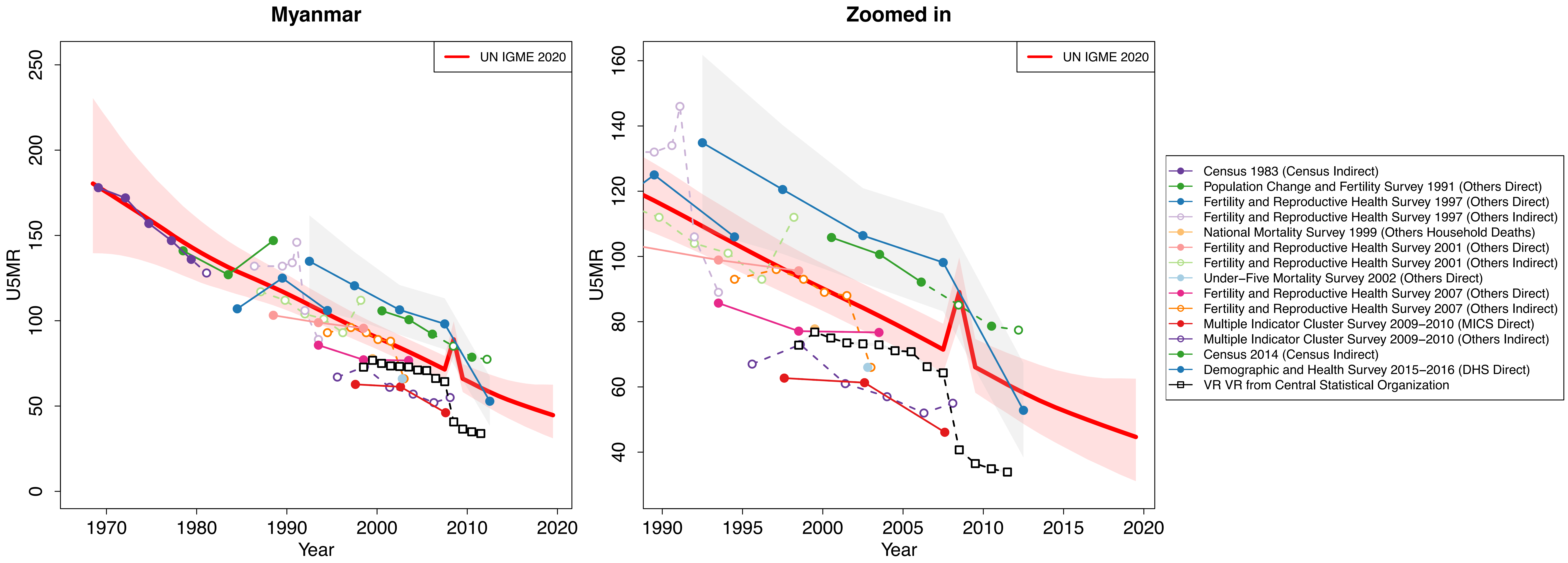

Myanmar
Zoomed in
UN IGME 2020
U5MR
Year
Census 1983 (Census Indirect)
Population Change and Fertility Survey 1991 (Others Direct)
Fertility and Reproductive Health Survey 1997 (Others Direct)
Fertility and Reproductive Health Survey 1997 (Others Indirect)
National Mortality Survey 1999 (Others Household Deaths)
Fertility and Reproductive Health Survey 2001 (Others Direct)
Fertility and Reproductive Health Survey 2001 (Others Indirect)
Under−Five Mortality Survey 2002 (Others Direct)
Fertility and Reproductive Health Survey 2007 (Others Direct)
Fertility and Reproductive Health Survey 2007 (Others Indirect)
Multiple Indicator Cluster Survey 2009−2010 (MICS Direct)
Multiple Indicator Cluster Survey 2009−2010 (Others Indirect)
Census 2014 (Census Indirect)
Demographic and Health Survey 2015−2016 (DHS Direct)
VR VR from Central Statistical Organization

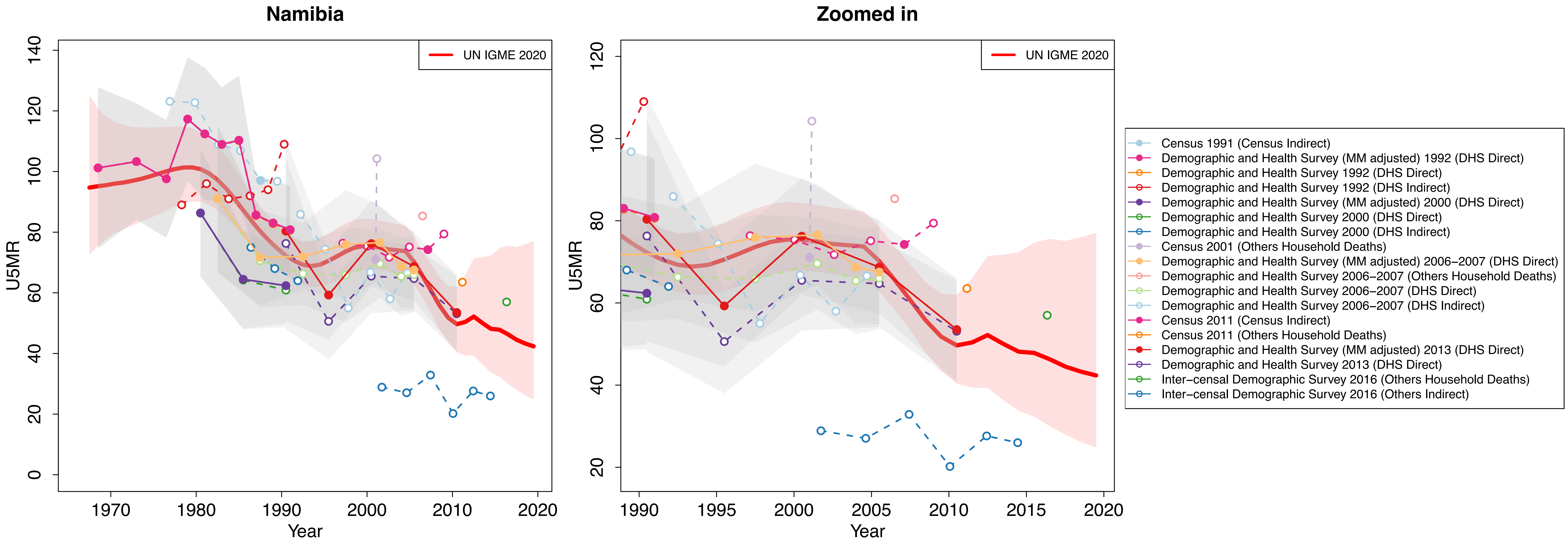
Namibia
Zoomed in
UN IGME 2020
U5MR
Year
0
20
40
60
80
100
120
140
1970
1980
1990
2000
2010
2020
1995
2005
2015
Census 1991 (Census Indirect)
Demographic and Health Survey (MM adjusted) 1992 (DHS Direct)
Demographic and Health Survey 1992 (DHS Direct)
Demographic and Health Survey 1992 (DHS Indirect)
Demographic and Health Survey (MM adjusted) 2000 (DHS Direct)
Demographic and Health Survey 2000 (DHS Direct)
Demographic and Health Survey 2000 (DHS Indirect)
Census 2001 (Others Household Deaths)
Demographic and Health Survey (MM adjusted) 2006−2007 (DHS Direct)
Demographic and Health Survey 2006−2007 (Others Household Deaths)
Demographic and Health Survey 2006−2007 (DHS Direct)
Demographic and Health Survey 2006−2007 (DHS Indirect)
Census 2011 (Census Indirect)
Census 2011 (Others Household Deaths)
Demographic and Health Survey (MM adjusted) 2013 (DHS Direct)
Demographic and Health Survey 2013 (DHS Direct)
Inter−censal Demographic Survey 2016 (Others Household Deaths)
Inter−censal Demographic Survey 2016 (Others Indirect)

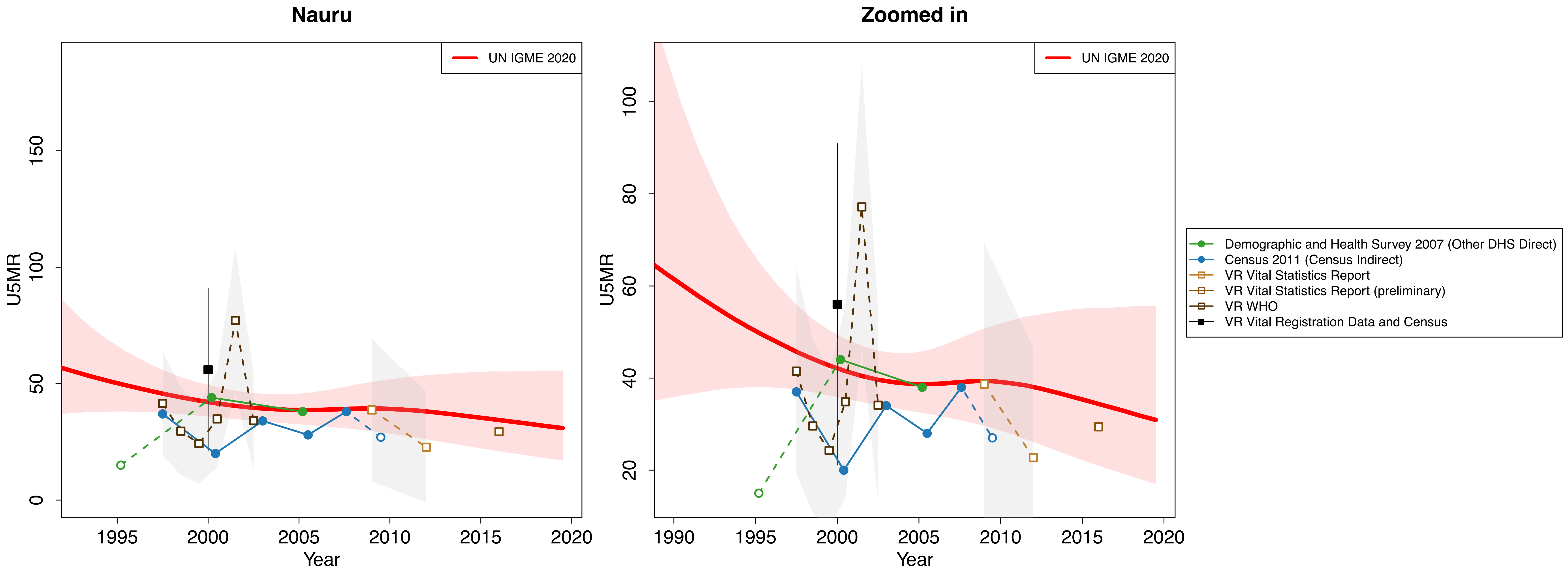

Nauru
Zoomed in
UN IGME 2020
U5MR
Year
Demographic and Health Survey 2007 (Other DHS Direct)
Census 2011 (Census Indirect)
VR Vital Statistics Report
VR Vital Statistics Report (preliminary)
VR WHO
VR Vital Registration Data and Census

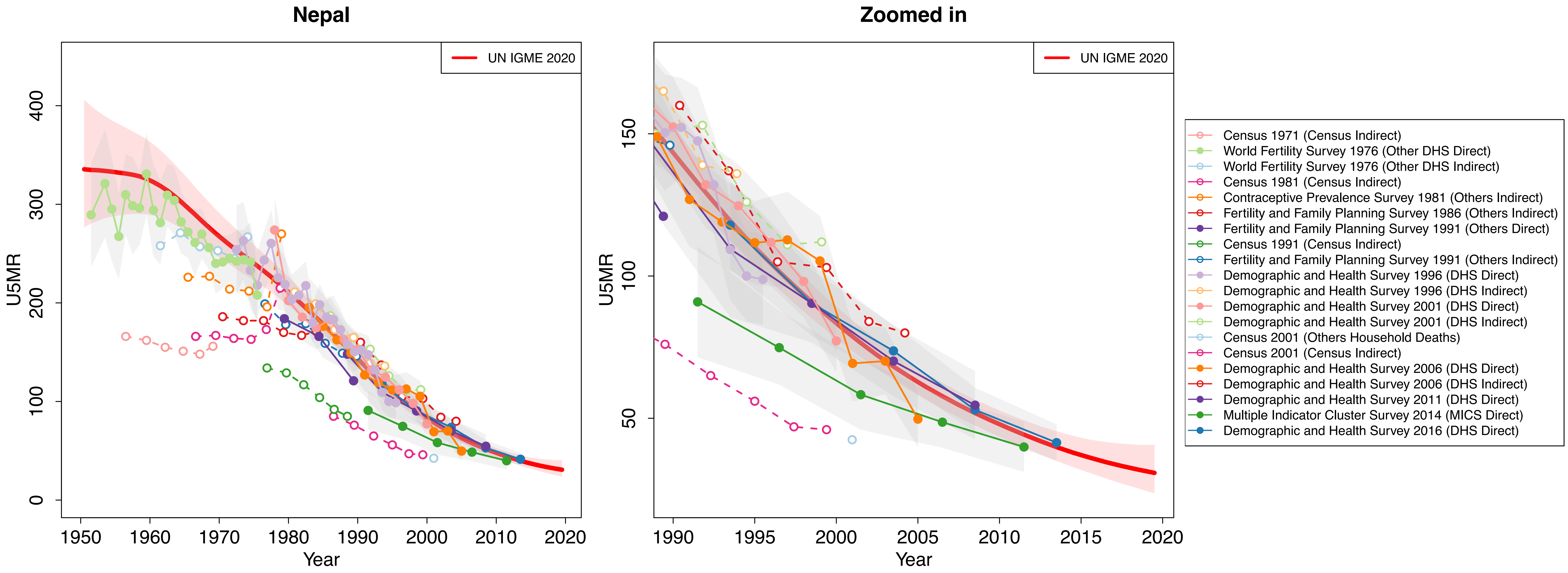

Nepal
Zoomed in
UN IGME 2020
U5MR
Year
Census 1971 (Census Indirect)
World Fertility Survey 1976 (Other DHS Direct)
World Fertility Survey 1976 (Other DHS Indirect)
Census 1981 (Census Indirect)
Contraceptive Prevalence Survey 1981 (Others Indirect)
Fertility and Family Planning Survey 1986 (Others Indirect)
Fertility and Family Planning Survey 1991 (Others Direct)
Census 1991 (Census Indirect)
Fertility and Family Planning Survey 1991 (Others Indirect)
Demographic and Health Survey 1996 (DHS Direct)
Demographic and Health Survey 1996 (DHS Indirect)
Demographic and Health Survey 2001 (DHS Direct)
Demographic and Health Survey 2001 (DHS Indirect)
Census 2001 (Others Household Deaths)
Census 2001 (Census Indirect)
Demographic and Health Survey 2006 (DHS Direct)
Demographic and Health Survey 2006 (DHS Indirect)
Demographic and Health Survey 2011 (DHS Direct)
Multiple Indicator Cluster Survey 2014 (MICS Direct)
Demographic and Health Survey 2016 (DHS Direct)

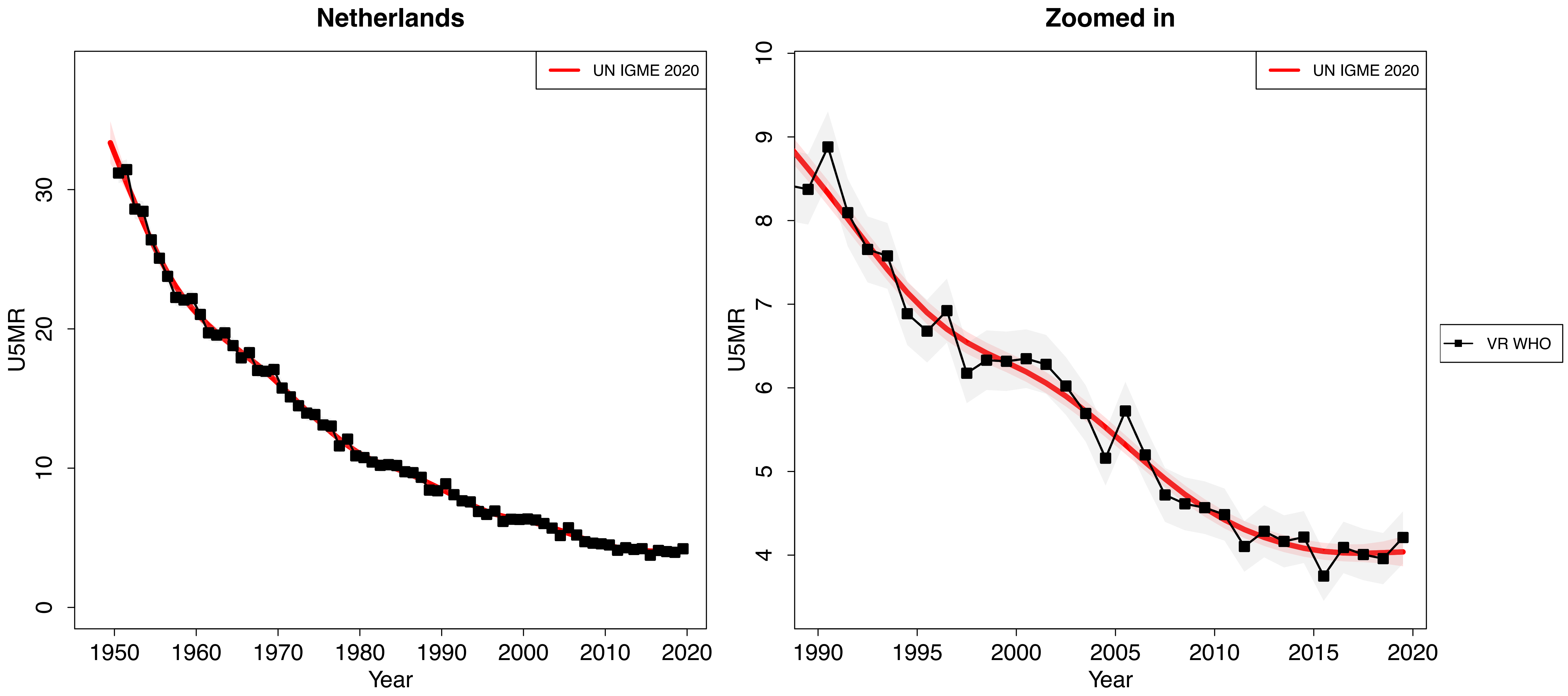

Netherlands
UN IGME 2020
U5MR
0
10
20
30
1950
1960
1970
1980
1990
2000
2010
2020
Year
Zoomed in
UN IGME 2020
U5MR
4
5
6
7
8
9
10
1990
1995
2000
2005
2010
2015
2020
Year
VR WHO

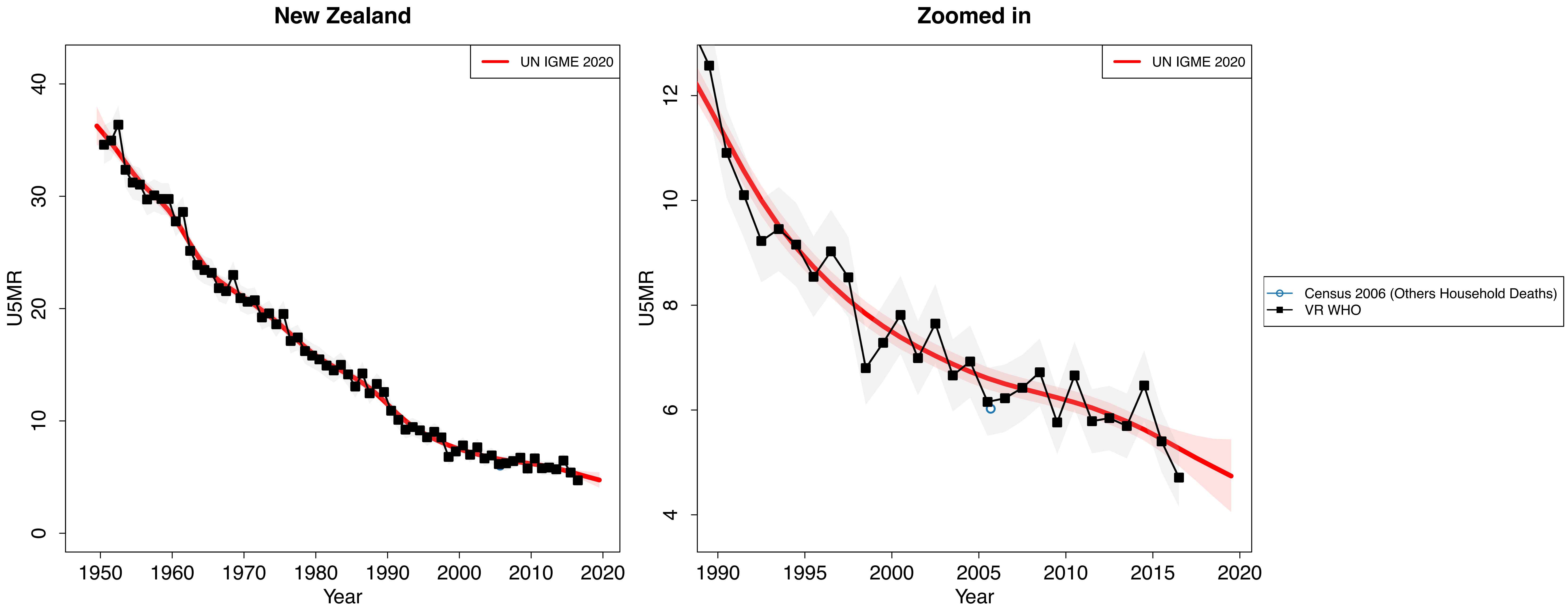

New Zealand
Zoomed in
UN IGME 2020
UN IGME 2020
U5MR
Year
Census 2006 (Others Household Deaths)
VR WHO
0
10
20
30
40
1950
1960
1970
1980
1990
2000
2010
2020
4
6
8
10
12
1990
1995
2000
2005
2010
2015
2020

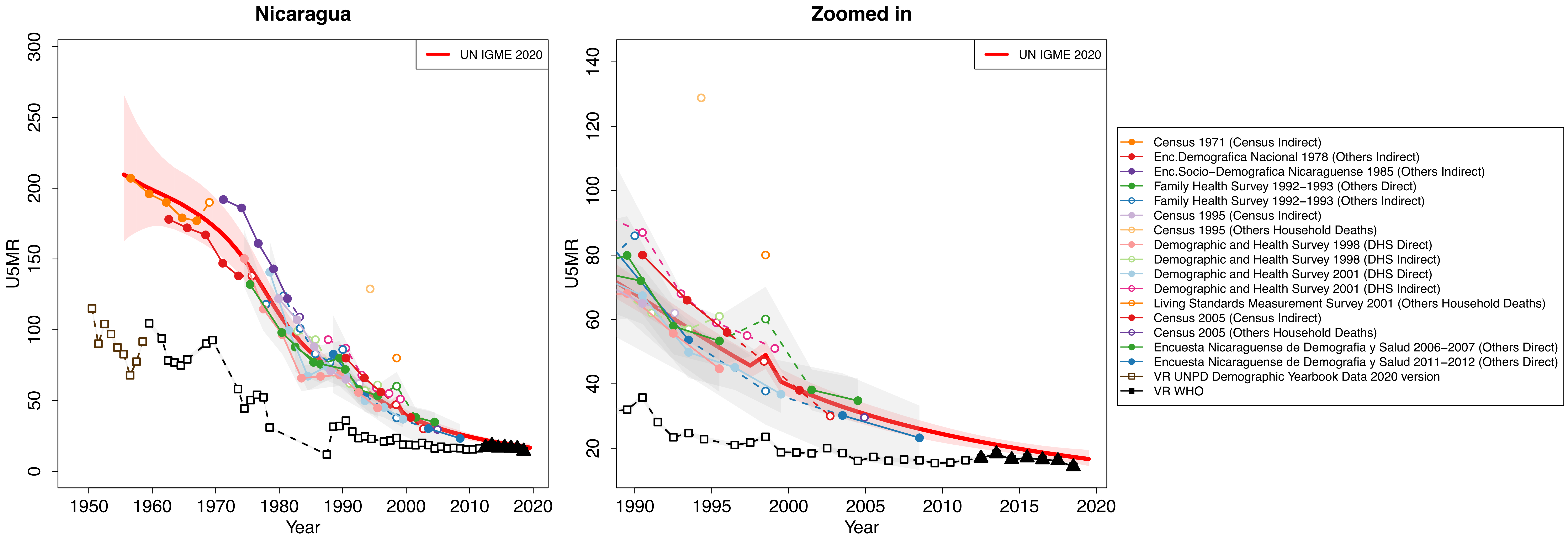

Nicaragua
Zoomed in
UN IGME 2020
U5MR
Year
Census 1971 (Census Indirect)
Enc.Demografica Nacional 1978 (Others Indirect)
Enc.Socio−Demografica Nicaraguense 1985 (Others Indirect)
Family Health Survey 1992−1993 (Others Direct)
Family Health Survey 1992−1993 (Others Indirect)
Census 1995 (Census Indirect)
Census 1995 (Others Household Deaths)
Demographic and Health Survey 1998 (DHS Direct)
Demographic and Health Survey 1998 (DHS Indirect)
Demographic and Health Survey 2001 (DHS Direct)
Demographic and Health Survey 2001 (DHS Indirect)
Living Standards Measurement Survey 2001 (Others Household Deaths)
Census 2005 (Census Indirect)
Census 2005 (Others Household Deaths)
Encuesta Nicaraguense de Demografia y Salud 2006−2007 (Others Direct)
Encuesta Nicaraguense de Demografia y Salud 2011−2012 (Others Direct)
VR UNPD Demographic Yearbook Data 2020 version
VR WHO

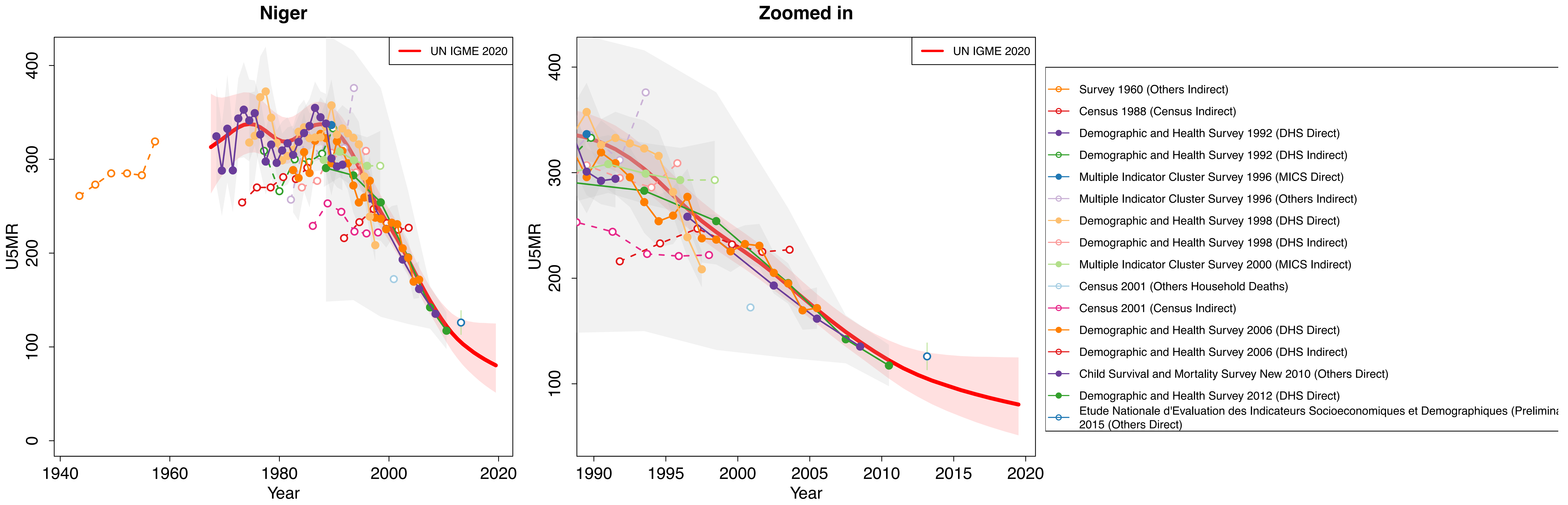

Niger
Zoomed in
UN IGME 2020
UN IGME 2020
U5MR
U5MR
Year
Year
0
100
200
300
400
1940
1960
1980
2000
2020
1990
1995
2000
2005
2010
2015
2020
Survey 1960 (Others Indirect)
Census 1988 (Census Indirect)
Demographic and Health Survey 1992 (DHS Direct)
Demographic and Health Survey 1992 (DHS Indirect)
Multiple Indicator Cluster Survey 1996 (MICS Direct)
Multiple Indicator Cluster Survey 1996 (Others Indirect)
Demographic and Health Survey 1998 (DHS Direct)
Demographic and Health Survey 1998 (DHS Indirect)
Multiple Indicator Cluster Survey 2000 (MICS Indirect)
Census 2001 (Others Household Deaths)
Census 2001 (Census Indirect)
Demographic and Health Survey 2006 (DHS Direct)
Demographic and Health Survey 2006 (DHS Indirect)
Child Survival and Mortality Survey New 2010 (Others Direct)
Demographic and Health Survey 2012 (DHS Direct)
Etude Nationale d'Evaluation des Indicateurs Socioeconomiques et Demographiques (Prelimina
2015 (Others Direct)

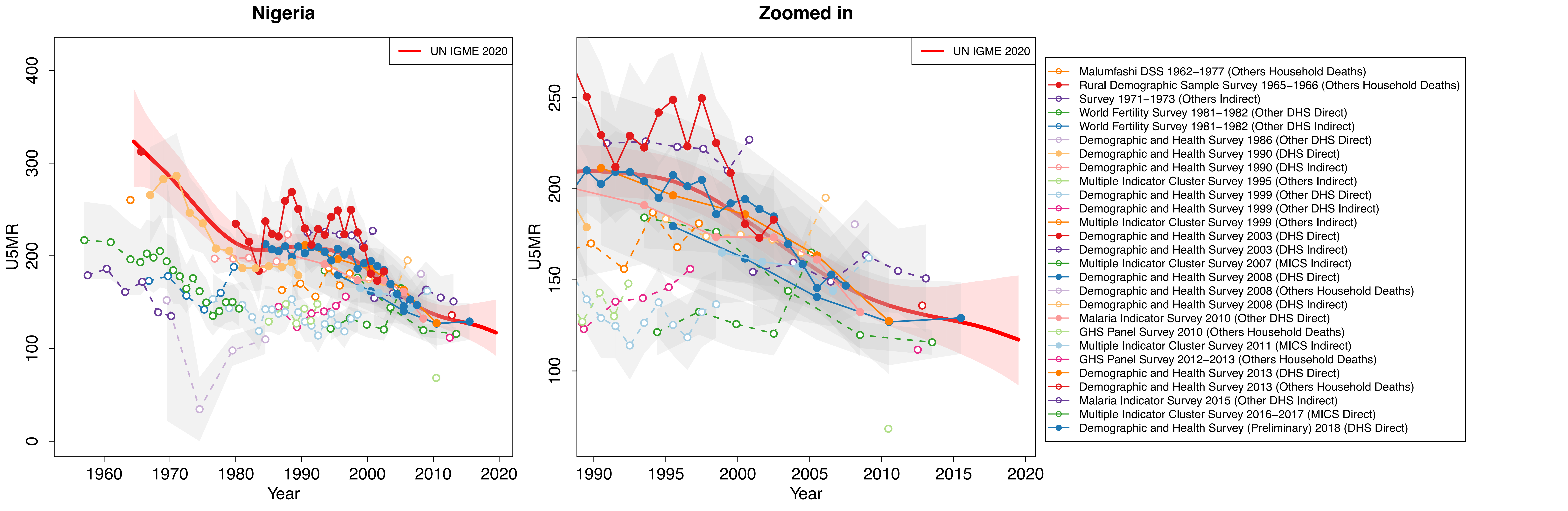
Nigeria
Zoomed in
UN IGME 2020
U5MR
Year
Malumfashi DSS 1962–1977 (Others Household Deaths)
Rural Demographic Sample Survey 1965–1966 (Others Household Deaths)
Survey 1971–1973 (Others Indirect)
World Fertility Survey 1981–1982 (Other DHS Direct)
World Fertility Survey 1981–1982 (Other DHS Indirect)
Demographic and Health Survey 1986 (Other DHS Direct)
Demographic and Health Survey 1990 (DHS Direct)
Demographic and Health Survey 1990 (DHS Indirect)
Multiple Indicator Cluster Survey 1995 (Others Indirect)
Demographic and Health Survey 1999 (Other DHS Direct)
Demographic and Health Survey 1999 (Other DHS Indirect)
Multiple Indicator Cluster Survey 1999 (Others Indirect)
Demographic and Health Survey 2003 (DHS Direct)
Demographic and Health Survey 2003 (DHS Indirect)
Multiple Indicator Cluster Survey 2007 (MICS Indirect)
Demographic and Health Survey 2008 (DHS Direct)
Demographic and Health Survey 2008 (Others Household Deaths)
Demographic and Health Survey 2008 (DHS Indirect)
Malaria Indicator Survey 2010 (Other DHS Direct)
GHS Panel Survey 2010 (Others Household Deaths)
Multiple Indicator Cluster Survey 2011 (MICS Indirect)
GHS Panel Survey 2012–2013 (Others Household Deaths)
Demographic and Health Survey 2013 (DHS Direct)
Demographic and Health Survey 2013 (Others Household Deaths)
Malaria Indicator Survey 2015 (Other DHS Indirect)
Multiple Indicator Cluster Survey 2016–2017 (MICS Direct)
Demographic and Health Survey (Preliminary) 2018 (DHS Direct)

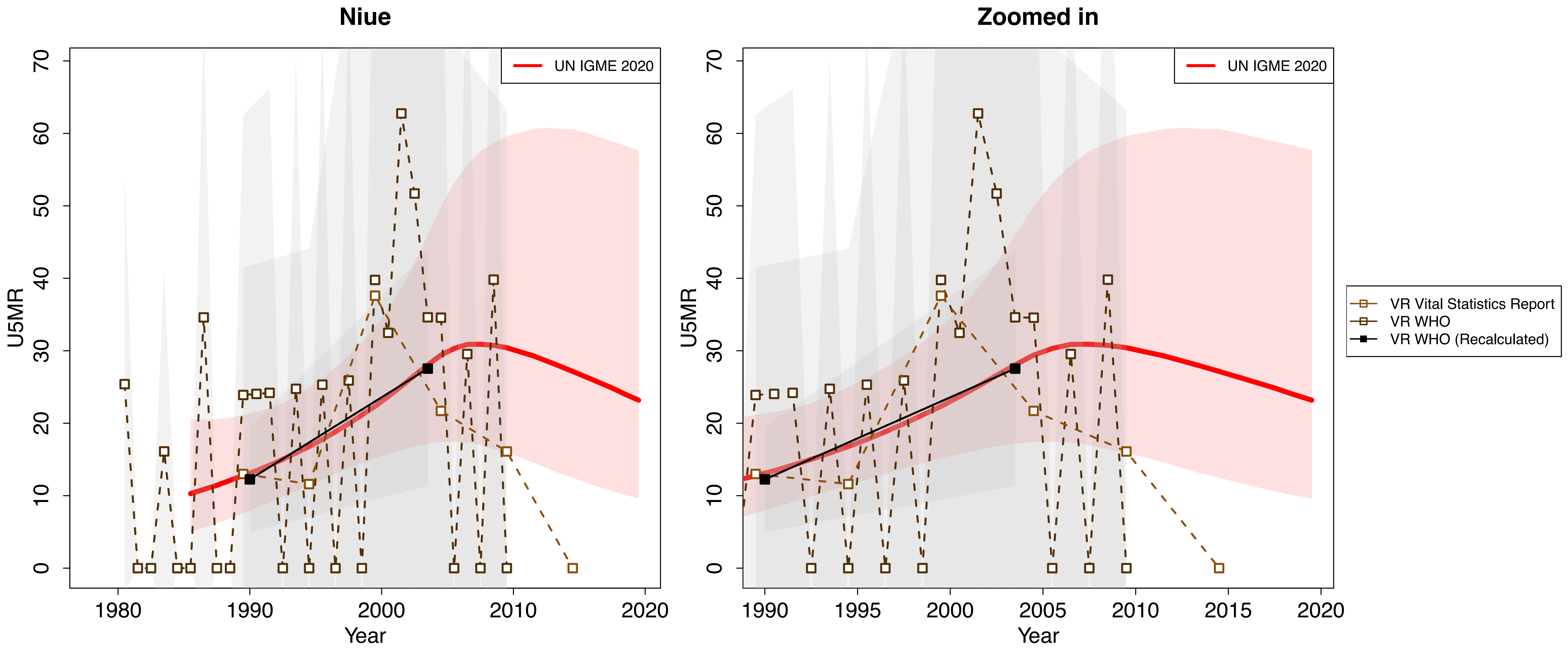

Niue
Zoomed in
UN IGME 2020
U5MR
Year
VR Vital Statistics Report
VR WHO
VR WHO (Recalculated)
0
10
20
30
40
50
60
70
1980
1990
2000
2010
2020
1995
2005
2015

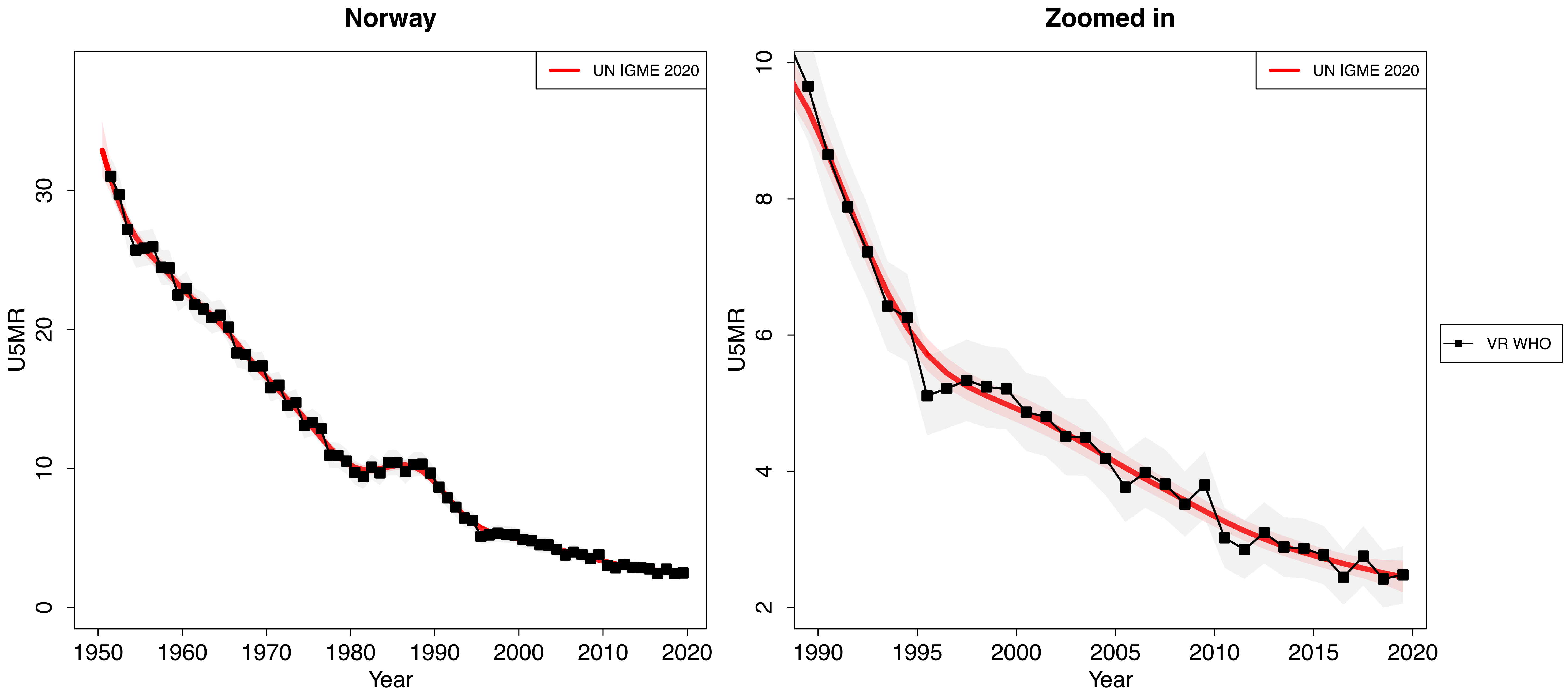

Norway
UN IGME 2020
U5MR
0
10
20
30
1950
1960
1970
1980
1990
2000
2010
2020
Year
Zoomed in
UN IGME 2020
U5MR
2
4
6
8
10
1990
1995
2000
2005
2010
2015
2020
Year
VR WHO

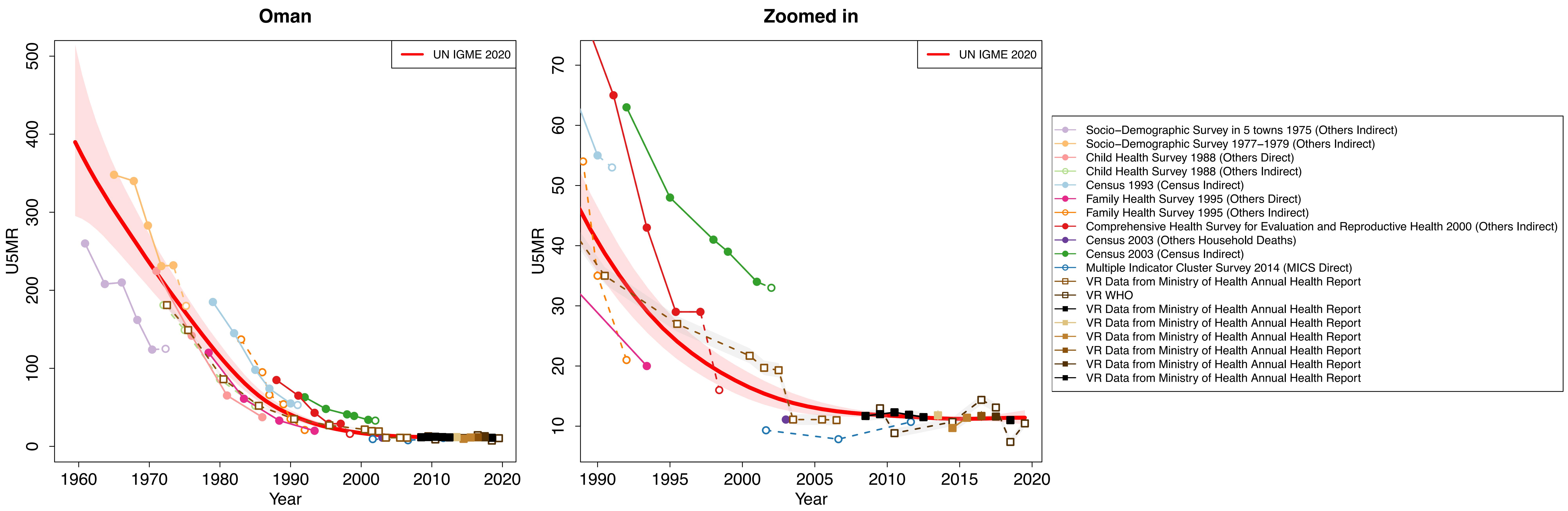

Oman
Zoomed in
UN IGME 2020
U5MR
Year
Socio−Demographic Survey in 5 towns 1975 (Others Indirect)
Socio−Demographic Survey 1977−1979 (Others Indirect)
Child Health Survey 1988 (Others Direct)
Child Health Survey 1988 (Others Indirect)
Census 1993 (Census Indirect)
Family Health Survey 1995 (Others Direct)
Family Health Survey 1995 (Others Indirect)
Comprehensive Health Survey for Evaluation and Reproductive Health 2000 (Others Indirect)
Census 2003 (Others Household Deaths)
Census 2003 (Census Indirect)
Multiple Indicator Cluster Survey 2014 (MICS Direct)
VR Data from Ministry of Health Annual Health Report
VR WHO
VR Data from Ministry of Health Annual Health Report
VR Data from Ministry of Health Annual Health Report
VR Data from Ministry of Health Annual Health Report
VR Data from Ministry of Health Annual Health Report
VR Data from Ministry of Health Annual Health Report
VR Data from Ministry of Health Annual Health Report

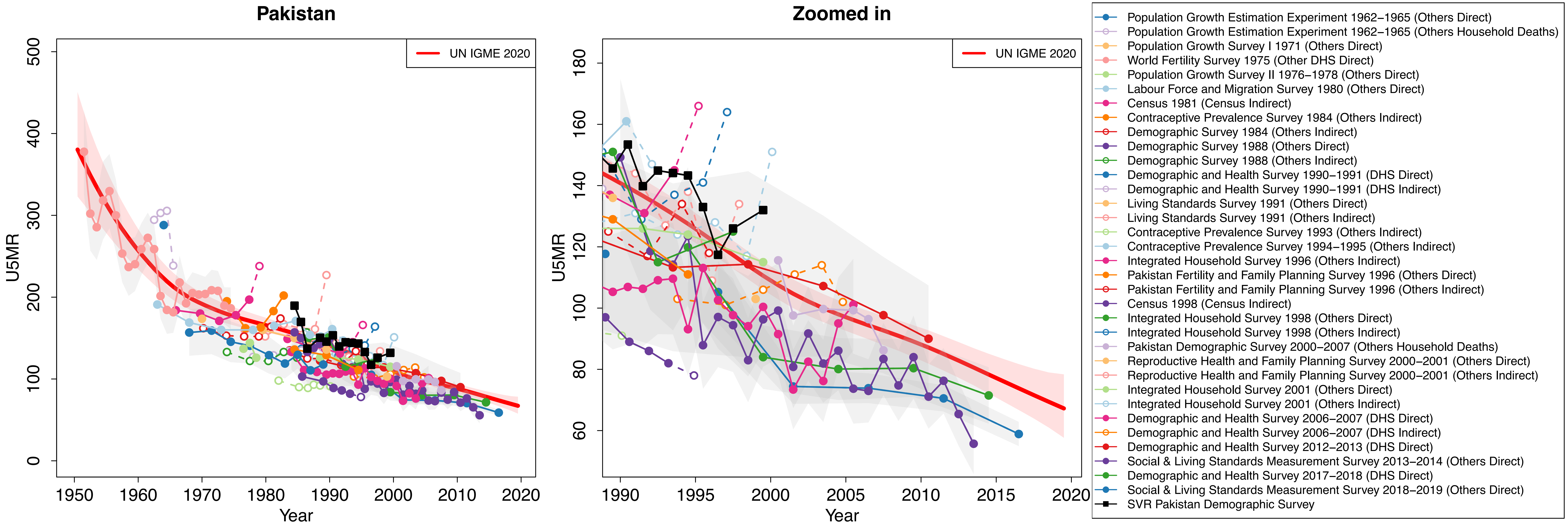

Pakistan
Zoomed in
UN IGME 2020
UN IGME 2020
U5MR
U5MR
Year
Year
0
100
200
300
400
500
60
80
100
120
140
160
180
1950
1960
1970
1980
1990
2000
2010
2020
1990
1995
2000
2005
2010
2015
2020
Population Growth Estimation Experiment 1962–1965 (Others Direct)
Population Growth Estimation Experiment 1962–1965 (Others Household Deaths)
Population Growth Survey I 1971 (Others Direct)
World Fertility Survey 1975 (Other DHS Direct)
Population Growth Survey II 1976–1978 (Others Direct)
Labour Force and Migration Survey 1980 (Others Direct)
Census 1981 (Census Indirect)
Contraceptive Prevalence Survey 1984 (Others Indirect)
Demographic Survey 1984 (Others Indirect)
Demographic Survey 1988 (Others Direct)
Demographic Survey 1988 (Others Indirect)
Demographic and Health Survey 1990–1991 (DHS Direct)
Demographic and Health Survey 1990–1991 (DHS Indirect)
Living Standards Survey 1991 (Others Direct)
Living Standards Survey 1991 (Others Indirect)
Contraceptive Prevalence Survey 1993 (Others Indirect)
Contraceptive Prevalence Survey 1994–1995 (Others Indirect)
Integrated Household Survey 1996 (Others Indirect)
Pakistan Fertility and Family Planning Survey 1996 (Others Direct)
Pakistan Fertility and Family Planning Survey 1996 (Others Indirect)
Census 1998 (Census Indirect)
Integrated Household Survey 1998 (Others Direct)
Integrated Household Survey 1998 (Others Indirect)
Pakistan Demographic Survey 2000–2007 (Others Household Deaths)
Reproductive Health and Family Planning Survey 2000–2001 (Others Direct)
Reproductive Health and Family Planning Survey 2000–2001 (Others Indirect)
Integrated Household Survey 2001 (Others Direct)
Integrated Household Survey 2001 (Others Indirect)
Demographic and Health Survey 2006–2007 (DHS Direct)
Demographic and Health Survey 2006–2007 (DHS Indirect)
Demographic and Health Survey 2012–2013 (DHS Direct)
Social & Living Standards Measurement Survey 2013–2014 (Others Direct)
Demographic and Health Survey 2017–2018 (DHS Direct)
Social & Living Standards Measurement Survey 2018–2019 (Others Direct)
SVR Pakistan Demographic Survey

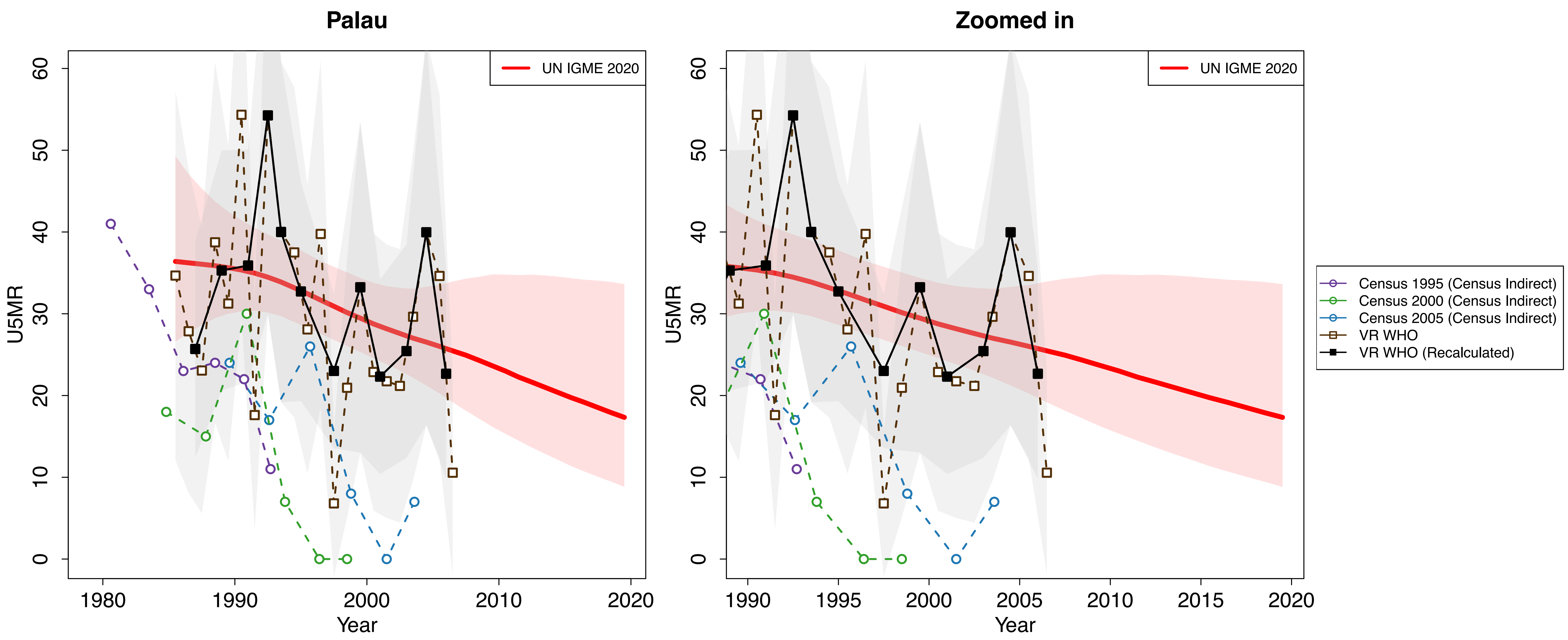

Palau
Zoomed in
UN IGME 2020
U5MR
Year
0
10
20
30
40
50
60
1980
1990
2000
2010
2020
1995
2005
2015
Census 1995 (Census Indirect)
Census 2000 (Census Indirect)
Census 2005 (Census Indirect)
VR WHO
VR WHO (Recalculated)

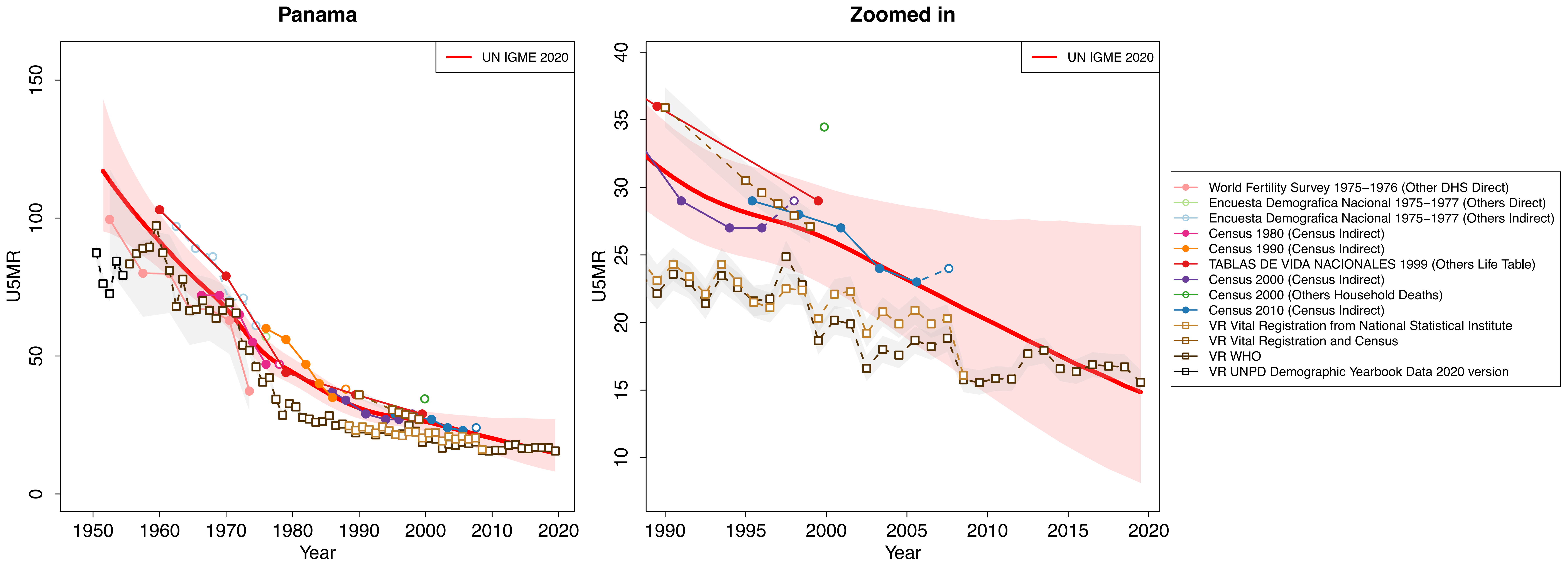

Panama
Zoomed in
UN IGME 2020
U5MR
Year
0
50
100
150
1950
1960
1970
1980
1990
2000
2010
2020
10
15
20
25
30
35
40
1995
2005
2015
World Fertility Survey 1975−1976 (Other DHS Direct)
Encuesta Demografica Nacional 1975−1977 (Others Direct)
Encuesta Demografica Nacional 1975−1977 (Others Indirect)
Census 1980 (Census Indirect)
Census 1990 (Census Indirect)
TABLAS DE VIDA NACIONALES 1999 (Others Life Table)
Census 2000 (Census Indirect)
Census 2000 (Others Household Deaths)
Census 2010 (Census Indirect)
VR Vital Registration from National Statistical Institute
VR Vital Registration and Census
VR WHO
VR UNPD Demographic Yearbook Data 2020 version

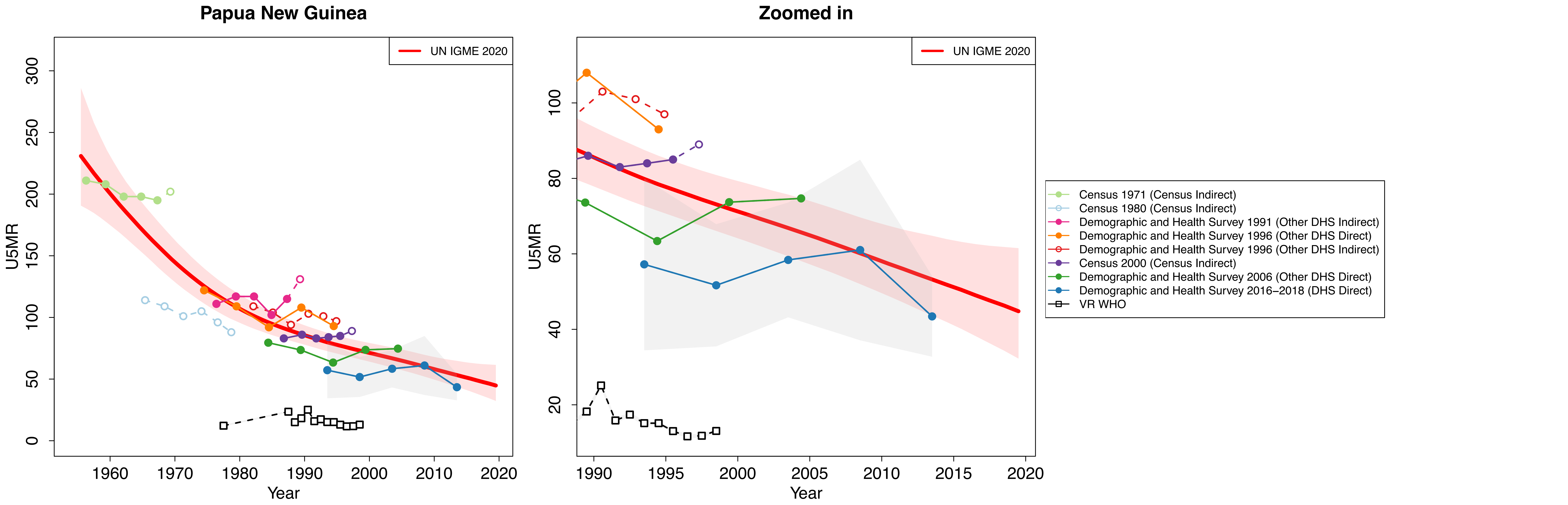

Papua New Guinea
Zoomed in
UN IGME 2020
U5MR
Year
Census 1971 (Census Indirect)
Census 1980 (Census Indirect)
Demographic and Health Survey 1991 (Other DHS Indirect)
Demographic and Health Survey 1996 (Other DHS Direct)
Demographic and Health Survey 1996 (Other DHS Indirect)
Census 2000 (Census Indirect)
Demographic and Health Survey 2006 (Other DHS Direct)
Demographic and Health Survey 2016–2018 (DHS Direct)
VR WHO

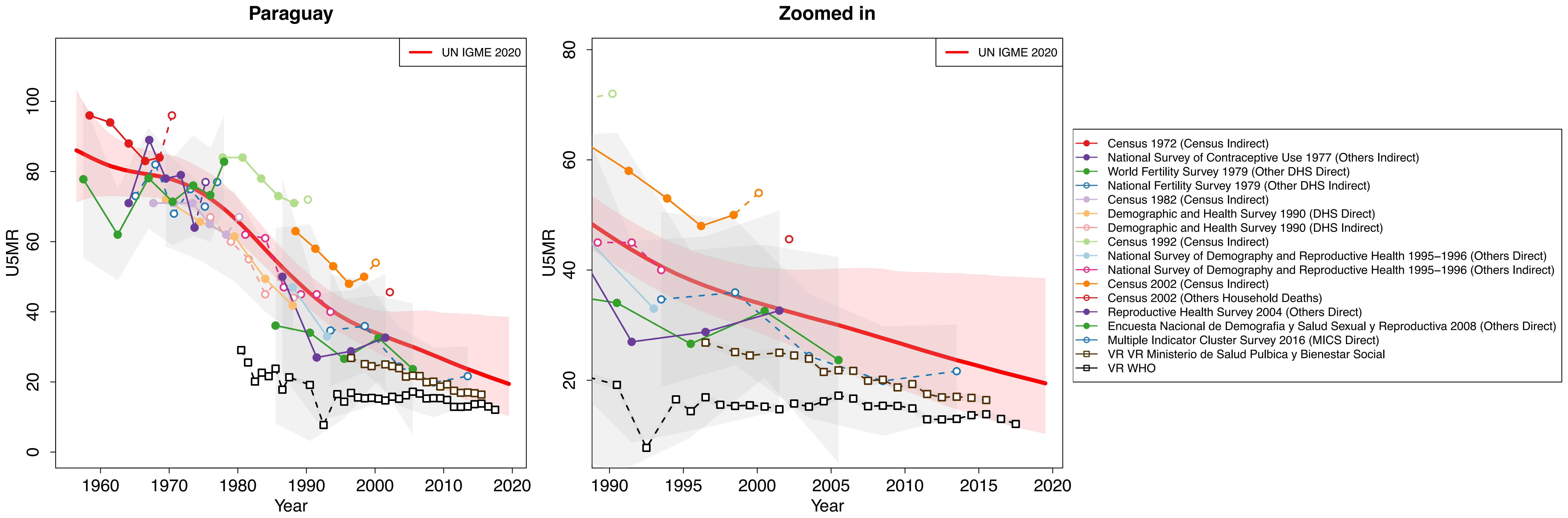
Paraguay
Zoomed in
UN IGME 2020
U5MR
Year
Census 1972 (Census Indirect)
National Survey of Contraceptive Use 1977 (Others Indirect)
World Fertility Survey 1979 (Other DHS Direct)
National Fertility Survey 1979 (Other DHS Indirect)
Census 1982 (Census Indirect)
Demographic and Health Survey 1990 (DHS Direct)
Demographic and Health Survey 1990 (DHS Indirect)
Census 1992 (Census Indirect)
National Survey of Demography and Reproductive Health 1995–1996 (Others Direct)
National Survey of Demography and Reproductive Health 1995–1996 (Others Indirect)
Census 2002 (Census Indirect)
Census 2002 (Others Household Deaths)
Reproductive Health Survey 2004 (Others Direct)
Encuesta Nacional de Demografia y Salud Sexual y Reproductiva 2008 (Others Direct)
Multiple Indicator Cluster Survey 2016 (MICS Direct)
VR VR Ministerio de Salud Pulbica y Bienestar Social
VR WHO

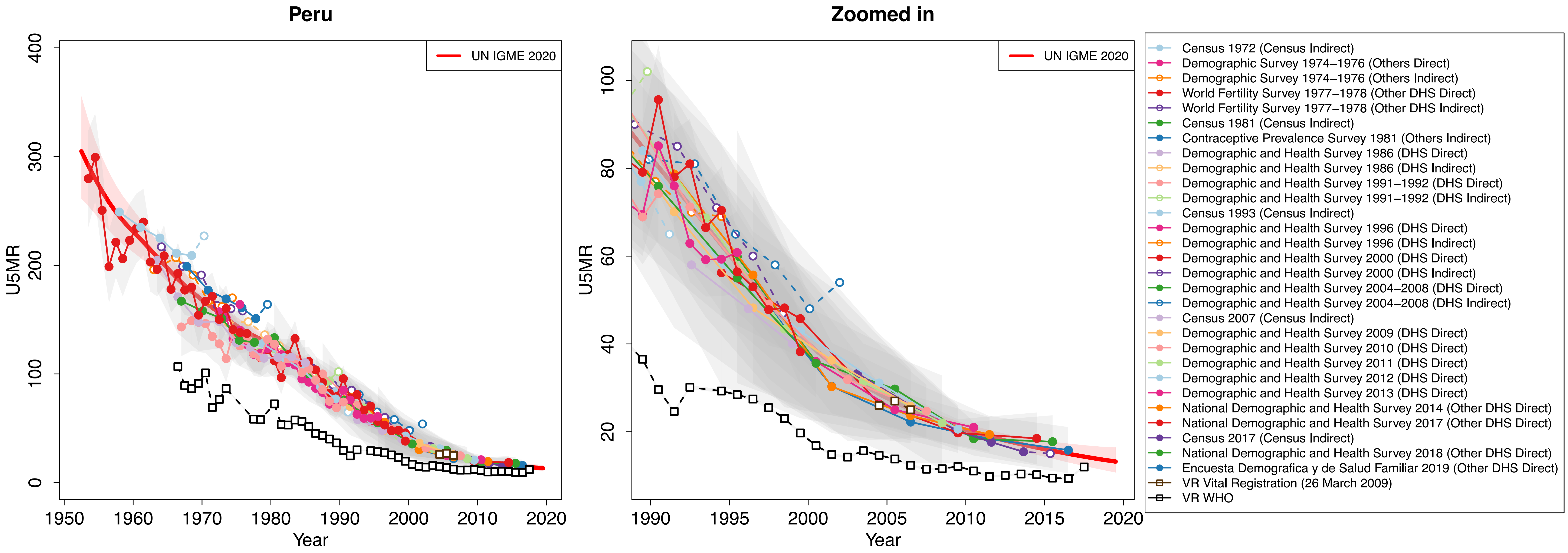

Peru
Zoomed in
UN IGME 2020
U5MR
Year
Census 1972 (Census Indirect)
Demographic Survey 1974–1976 (Others Direct)
Demographic Survey 1974–1976 (Others Indirect)
World Fertility Survey 1977–1978 (Other DHS Direct)
World Fertility Survey 1977–1978 (Other DHS Indirect)
Census 1981 (Census Indirect)
Contraceptive Prevalence Survey 1981 (Others Indirect)
Demographic and Health Survey 1986 (DHS Direct)
Demographic and Health Survey 1986 (DHS Indirect)
Demographic and Health Survey 1991–1992 (DHS Direct)
Demographic and Health Survey 1991–1992 (DHS Indirect)
Census 1993 (Census Indirect)
Demographic and Health Survey 1996 (DHS Direct)
Demographic and Health Survey 1996 (DHS Indirect)
Demographic and Health Survey 2000 (DHS Direct)
Demographic and Health Survey 2000 (DHS Indirect)
Demographic and Health Survey 2004–2008 (DHS Direct)
Demographic and Health Survey 2004–2008 (DHS Indirect)
Census 2007 (Census Indirect)
Demographic and Health Survey 2009 (DHS Direct)
Demographic and Health Survey 2010 (DHS Direct)
Demographic and Health Survey 2011 (DHS Direct)
Demographic and Health Survey 2012 (DHS Direct)
Demographic and Health Survey 2013 (DHS Direct)
National Demographic and Health Survey 2014 (Other DHS Direct)
National Demographic and Health Survey 2017 (Other DHS Direct)
Census 2017 (Census Indirect)
National Demographic and Health Survey 2018 (Other DHS Direct)
Encuesta Demografica y de Salud Familiar 2019 (Other DHS Direct)
VR Vital Registration (26 March 2009)
VR WHO

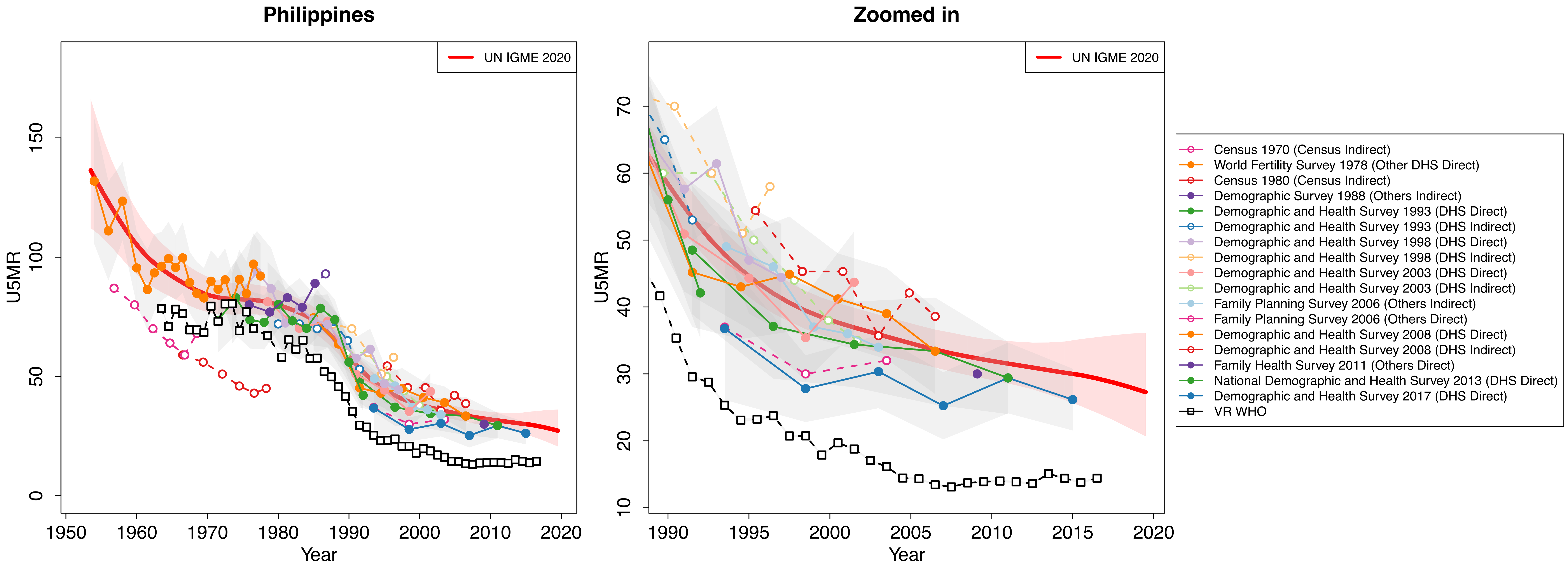

Philippines
Zoomed in
UN IGME 2020
U5MR
Year
Census 1970 (Census Indirect)
World Fertility Survey 1978 (Other DHS Direct)
Census 1980 (Census Indirect)
Demographic Survey 1988 (Others Indirect)
Demographic and Health Survey 1993 (DHS Direct)
Demographic and Health Survey 1993 (DHS Indirect)
Demographic and Health Survey 1998 (DHS Direct)
Demographic and Health Survey 1998 (DHS Indirect)
Demographic and Health Survey 2003 (DHS Direct)
Demographic and Health Survey 2003 (DHS Indirect)
Family Planning Survey 2006 (Others Indirect)
Family Planning Survey 2006 (Others Direct)
Demographic and Health Survey 2008 (DHS Direct)
Demographic and Health Survey 2008 (DHS Indirect)
Family Health Survey 2011 (Others Direct)
National Demographic and Health Survey 2013 (DHS Direct)
Demographic and Health Survey 2017 (DHS Direct)
VR WHO

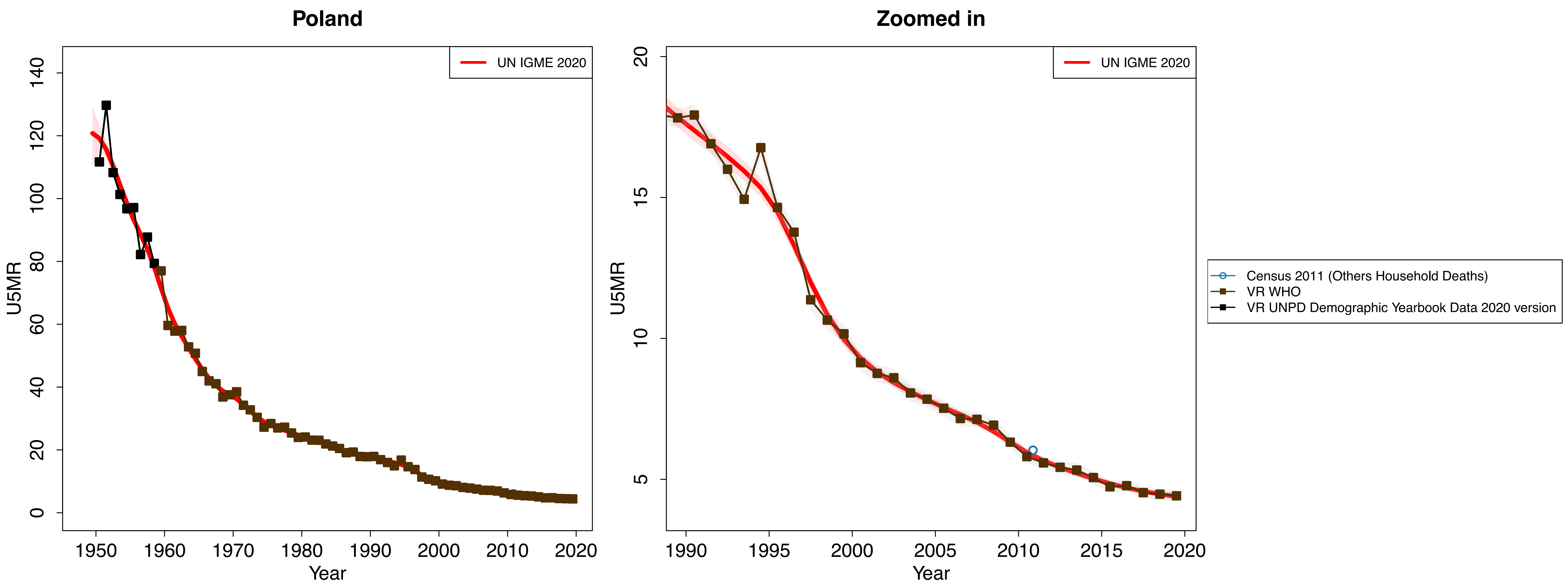

Poland
Zoomed in
UN IGME 2020
U5MR
Year
Census 2011 (Others Household Deaths)
VR WHO
VR UNPD Demographic Yearbook Data 2020 version

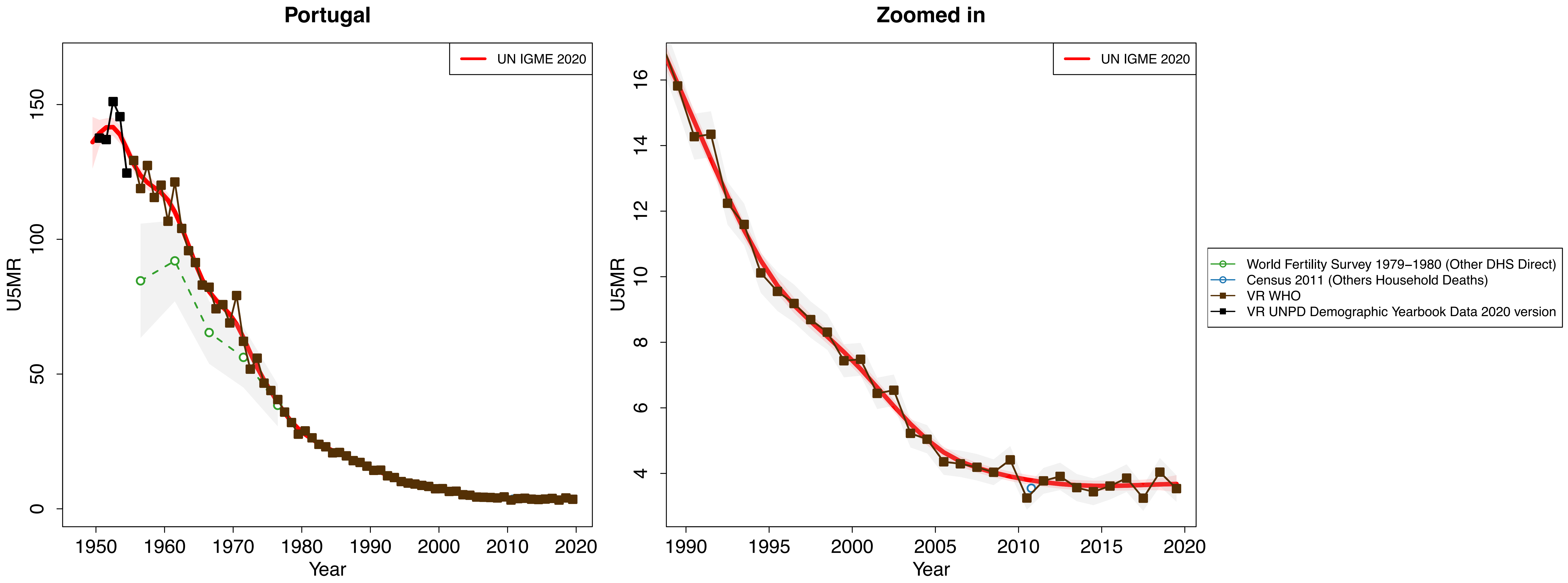

Portugal
UN IGME 2020
U5MR
Year
Zoomed in
UN IGME 2020
U5MR
Year
World Fertility Survey 1979–1980 (Other DHS Direct)
Census 2011 (Others Household Deaths)
VR WHO
VR UNPD Demographic Yearbook Data 2020 version

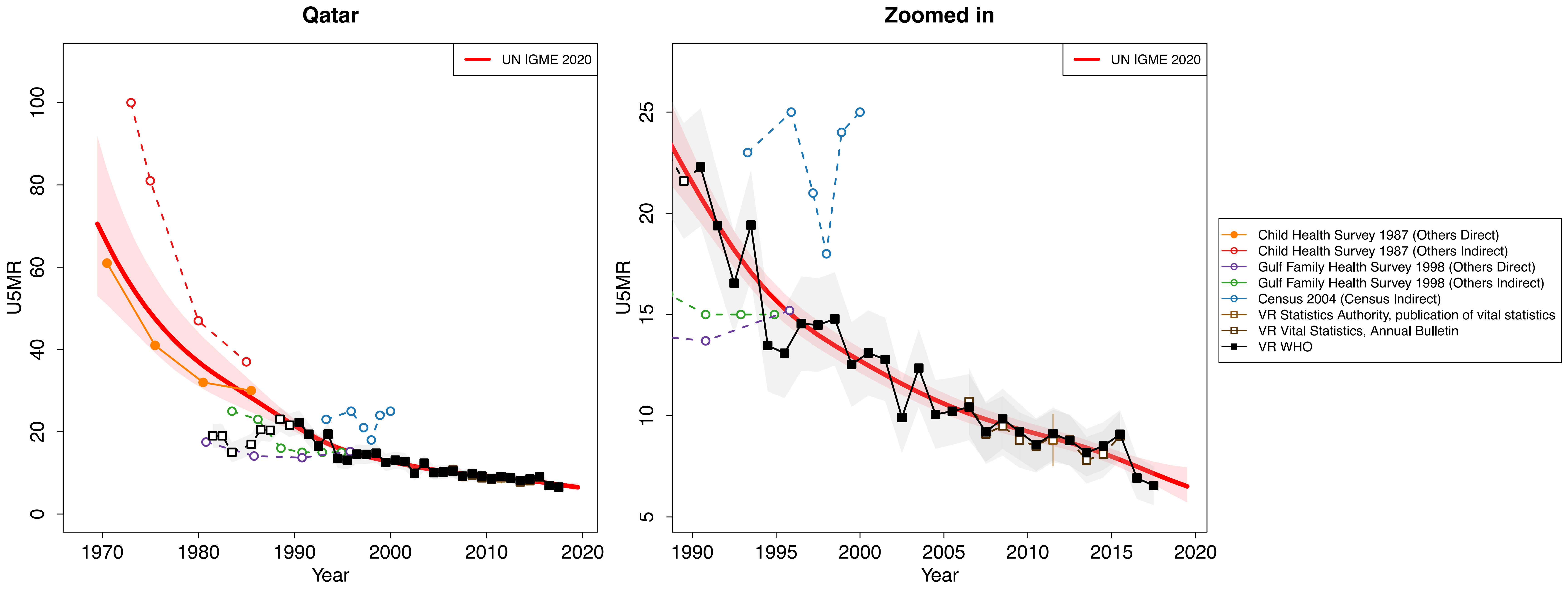

Qatar
Zoomed in
UN IGME 2020
U5MR
Year
Child Health Survey 1987 (Others Direct)
Child Health Survey 1987 (Others Indirect)
Gulf Family Health Survey 1998 (Others Direct)
Gulf Family Health Survey 1998 (Others Indirect)
Census 2004 (Census Indirect)
VR Statistics Authority, publication of vital statistics
VR Vital Statistics, Annual Bulletin
VR WHO

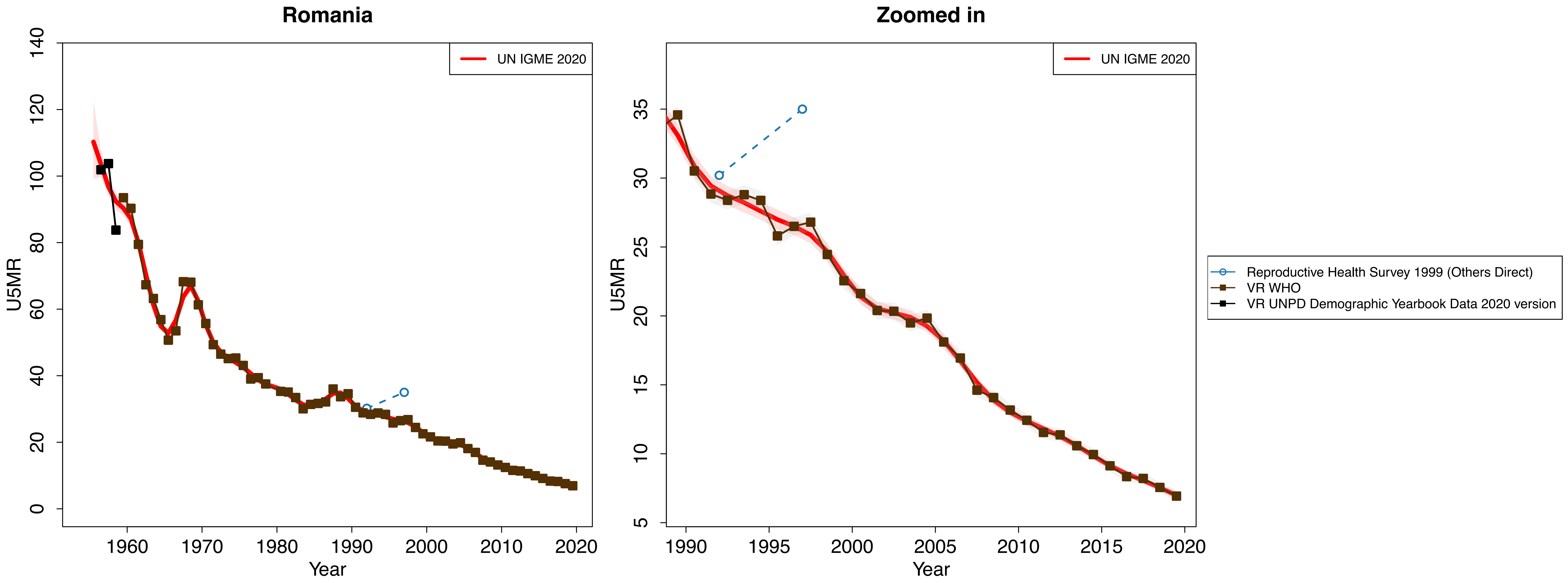

Romania
UN IGME 2020
U5MR
0
20
40
60
80
100
120
140
1960
1970
1980
1990
2000
2010
2020
Year
Zoomed in
UN IGME 2020
U5MR
5
10
15
20
25
30
35
1990
1995
2000
2005
2010
2015
2020
Year
Reproductive Health Survey 1999 (Others Direct)
VR WHO
VR UNPD Demographic Yearbook Data 2020 version

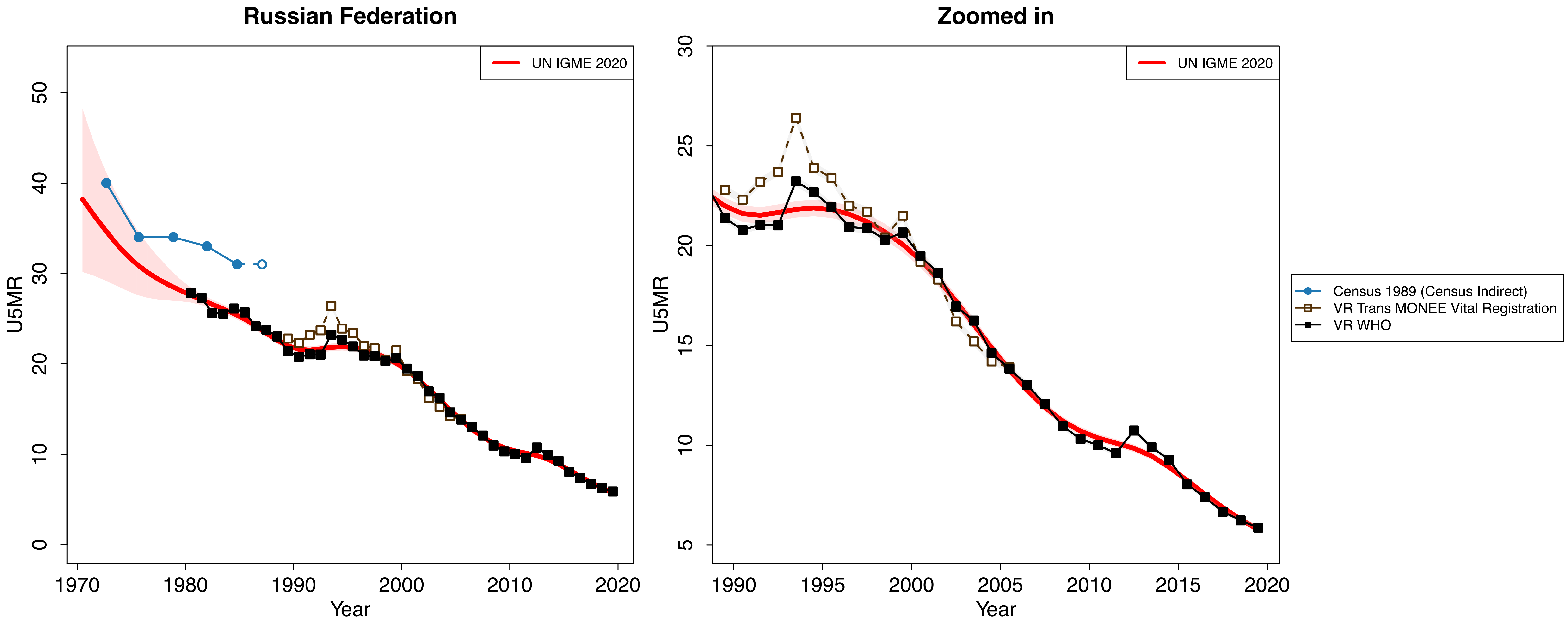

Russian Federation
Zoomed in
UN IGME 2020
UN IGME 2020
U5MR
Year
Census 1989 (Census Indirect)
VR Trans MONEE Vital Registration
VR WHO

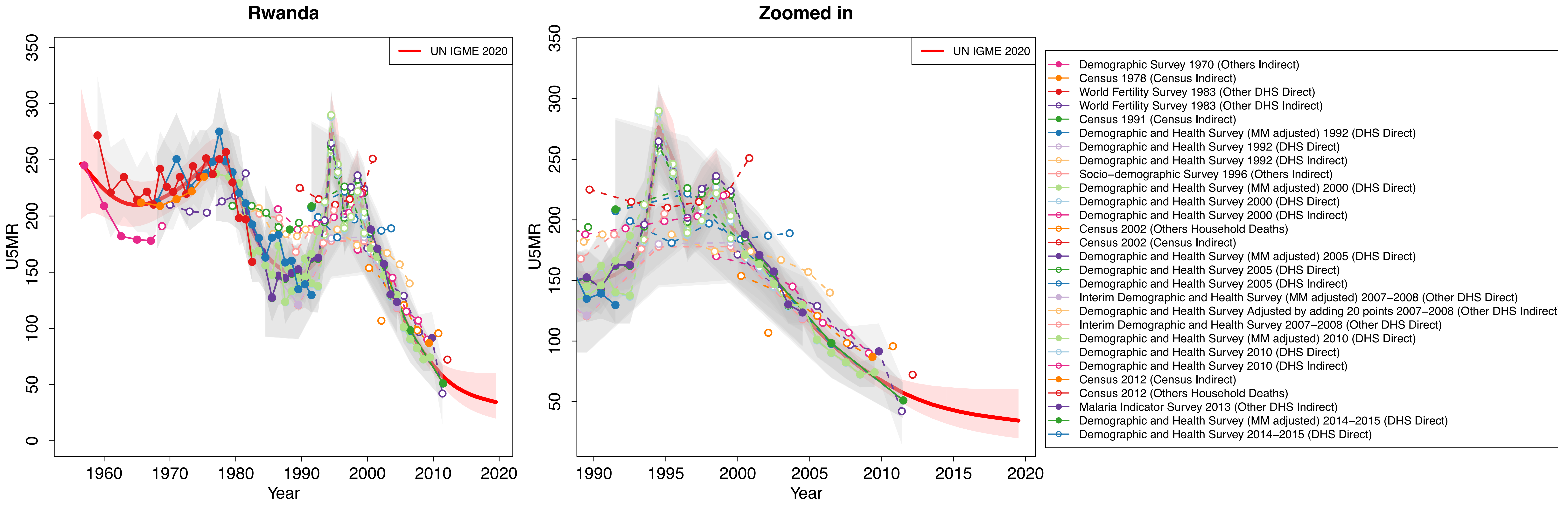

Rwanda
Zoomed in
UN IGME 2020
U5MR
Year
0
50
100
150
200
250
300
350
1960
1970
1980
1990
2000
2010
2020
1995
2005
2015
Demographic Survey 1970 (Others Indirect)
Census 1978 (Census Indirect)
World Fertility Survey 1983 (Other DHS Direct)
World Fertility Survey 1983 (Other DHS Indirect)
Census 1991 (Census Indirect)
Demographic and Health Survey (MM adjusted) 1992 (DHS Direct)
Demographic and Health Survey 1992 (DHS Direct)
Demographic and Health Survey 1992 (DHS Indirect)
Socio−demographic Survey 1996 (Others Indirect)
Demographic and Health Survey (MM adjusted) 2000 (DHS Direct)
Demographic and Health Survey 2000 (DHS Direct)
Demographic and Health Survey 2000 (DHS Indirect)
Census 2002 (Others Household Deaths)
Census 2002 (Census Indirect)
Demographic and Health Survey (MM adjusted) 2005 (DHS Direct)
Demographic and Health Survey 2005 (DHS Direct)
Demographic and Health Survey 2005 (DHS Indirect)
Interim Demographic and Health Survey (MM adjusted) 2007−2008 (Other DHS Direct)
Demographic and Health Survey Adjusted by adding 20 points 2007−2008 (Other DHS Indirect
Interim Demographic and Health Survey 2007−2008 (Other DHS Direct)
Demographic and Health Survey (MM adjusted) 2010 (DHS Direct)
Demographic and Health Survey 2010 (DHS Direct)
Demographic and Health Survey 2010 (DHS Indirect)
Census 2012 (Census Indirect)
Census 2012 (Others Household Deaths)
Malaria Indicator Survey 2013 (Other DHS Indirect)
Demographic and Health Survey (MM adjusted) 2014−2015 (DHS Direct)
Demographic and Health Survey 2014−2015 (DHS Direct)

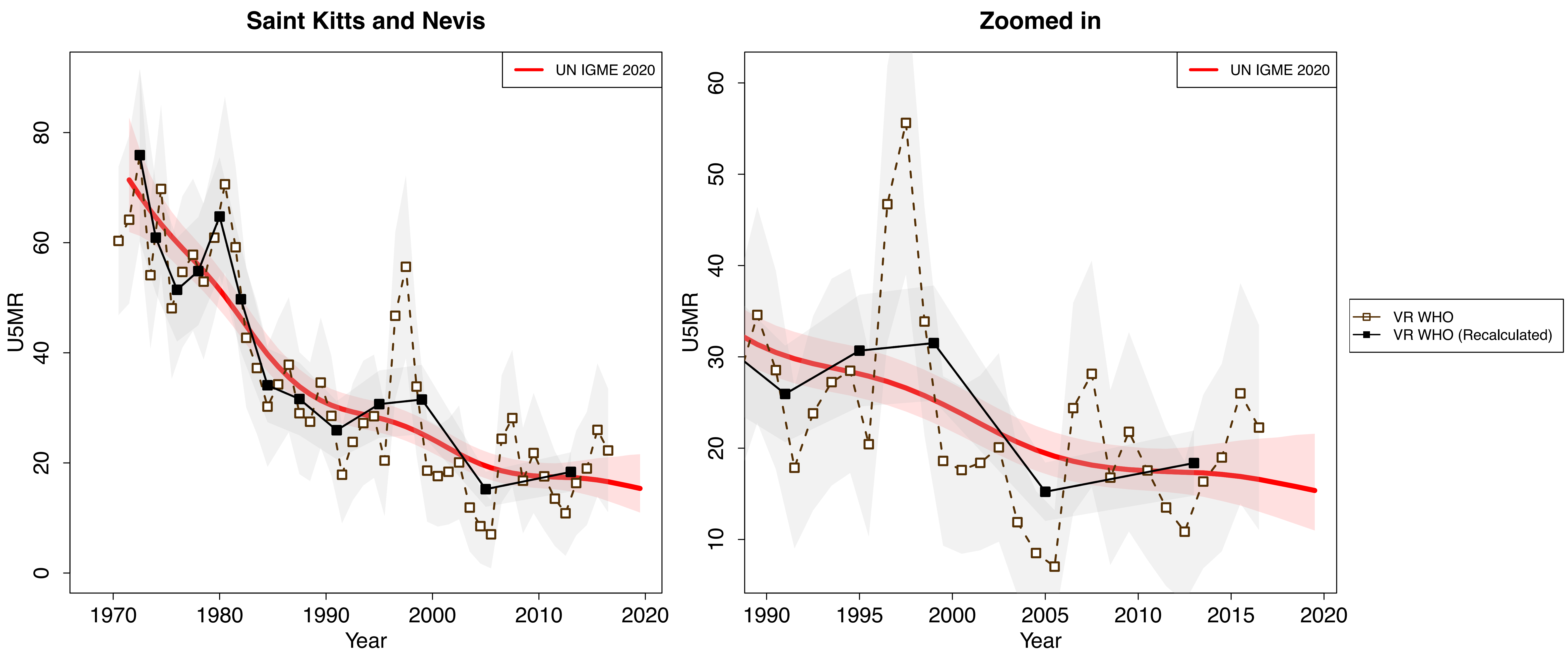

Saint Kitts and Nevis
Zoomed in
UN IGME 2020
UN IGME 2020
U5MR
Year
VR WHO
VR WHO (Recalculated)

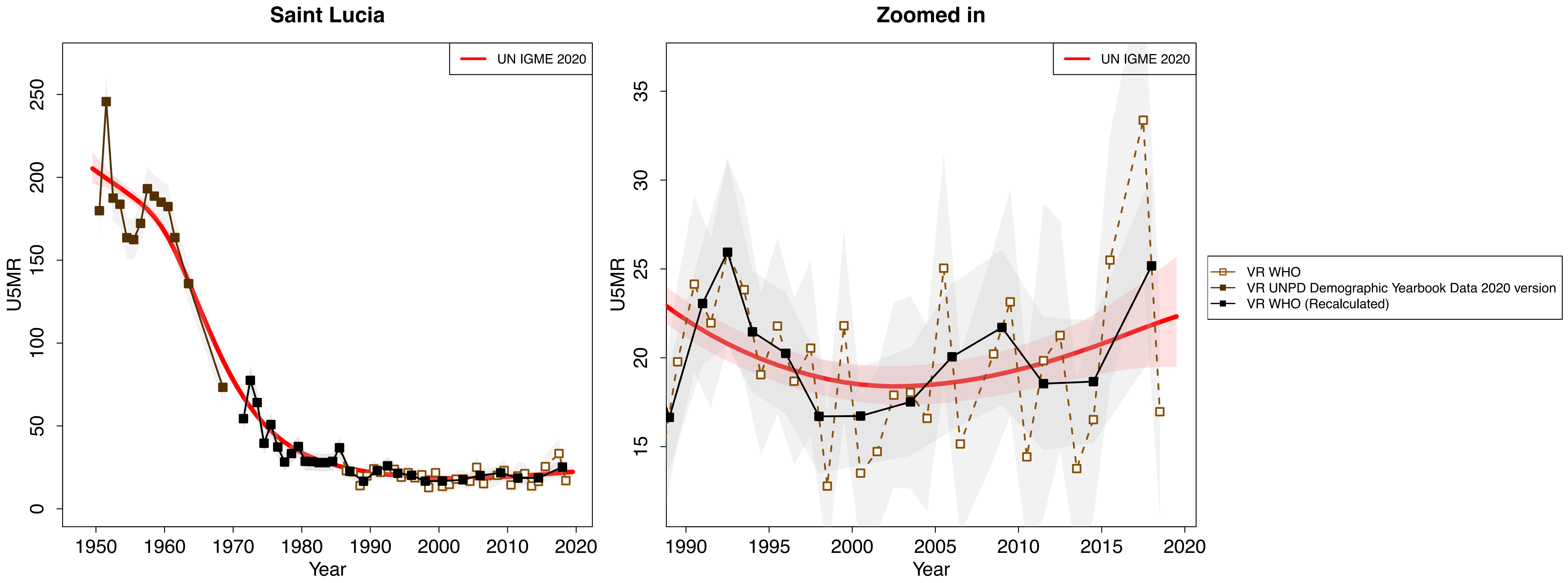

Saint Lucia
Zoomed in
UN IGME 2020
U5MR
Year
VR WHO
VR UNPD Demographic Yearbook Data 2020 version
VR WHO (Recalculated)

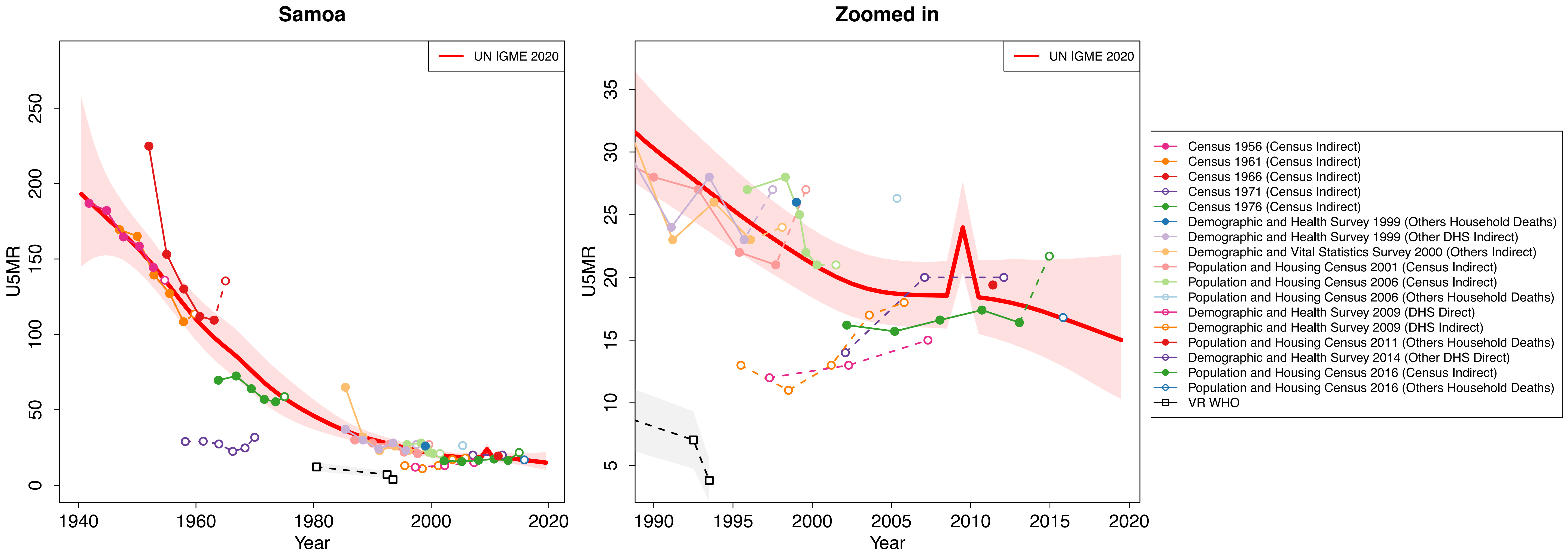

Samoa
Zoomed in
UN IGME 2020
U5MR
Year
Census 1956 (Census Indirect)
Census 1961 (Census Indirect)
Census 1966 (Census Indirect)
Census 1971 (Census Indirect)
Census 1976 (Census Indirect)
Demographic and Health Survey 1999 (Others Household Deaths)
Demographic and Health Survey 1999 (Other DHS Indirect)
Demographic and Vital Statistics Survey 2000 (Others Indirect)
Population and Housing Census 2001 (Census Indirect)
Population and Housing Census 2006 (Census Indirect)
Population and Housing Census 2006 (Others Household Deaths)
Demographic and Health Survey 2009 (DHS Direct)
Demographic and Health Survey 2009 (DHS Indirect)
Population and Housing Census 2011 (Others Household Deaths)
Demographic and Health Survey 2014 (Other DHS Direct)
Population and Housing Census 2016 (Census Indirect)
Population and Housing Census 2016 (Others Household Deaths)
VR WHO

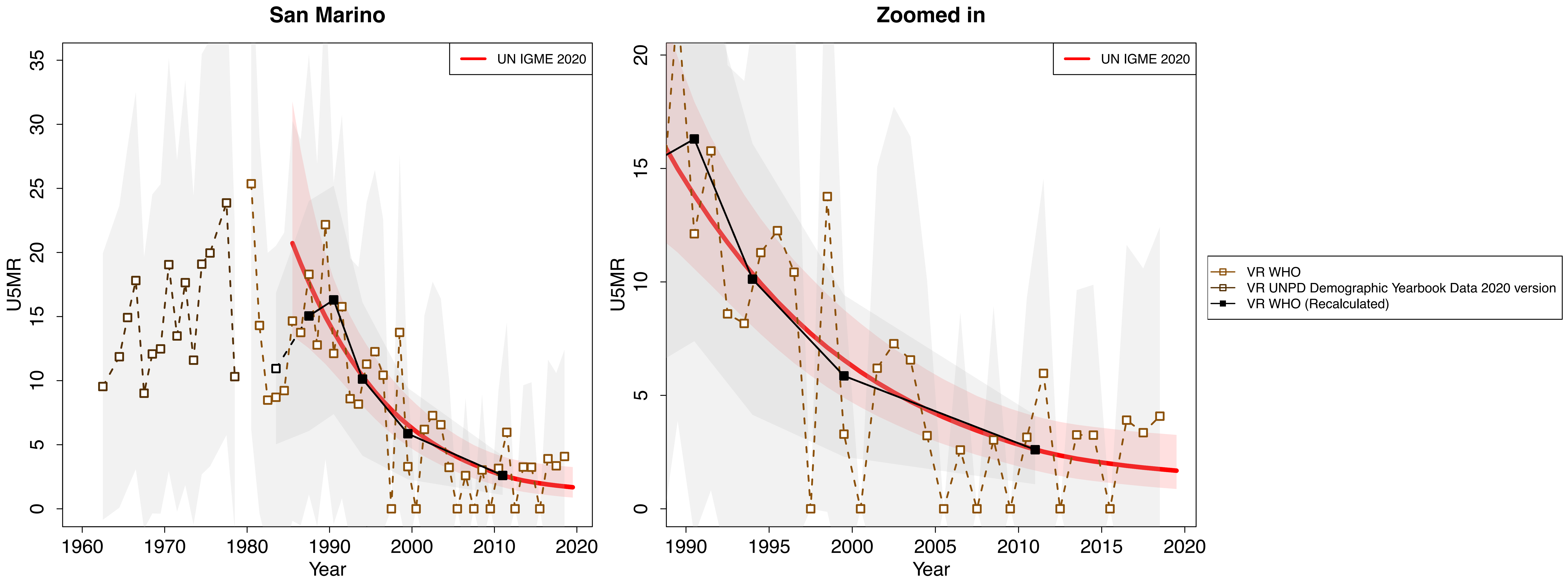

San Marino
Zoomed in
UN IGME 2020
U5MR
Year
VR WHO
VR UNPD Demographic Yearbook Data 2020 version
VR WHO (Recalculated)

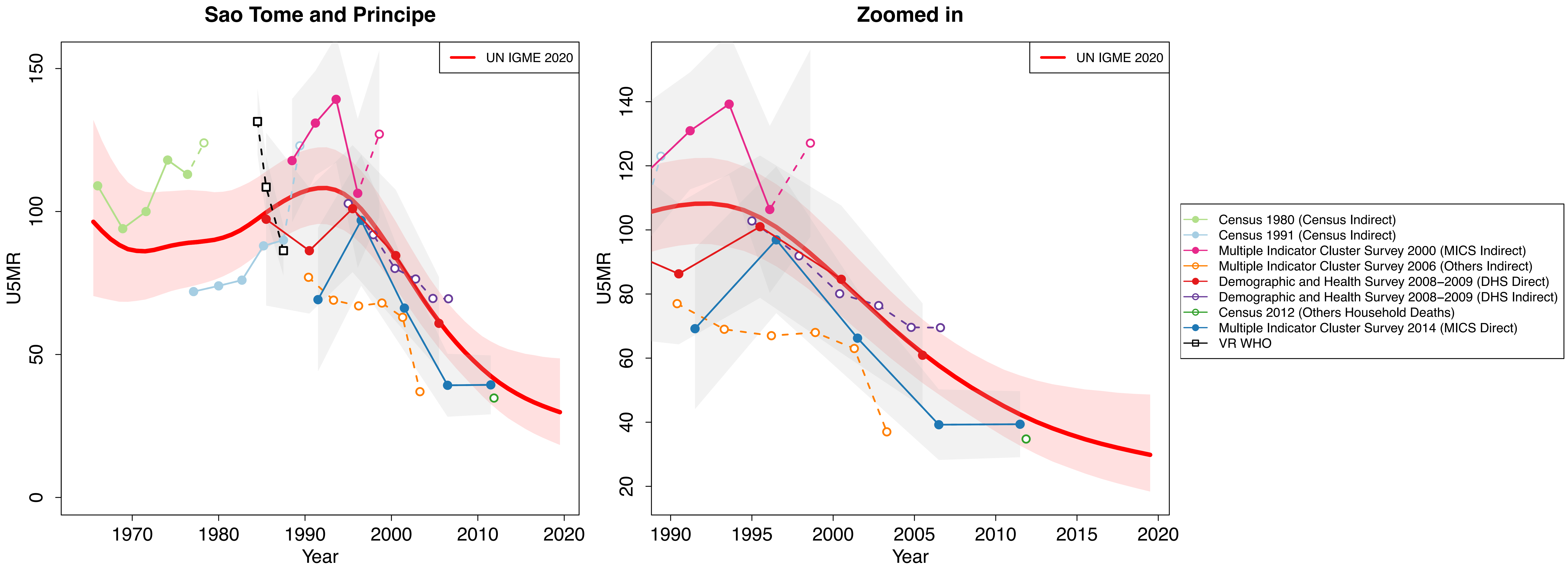

Sao Tome and Principe
Zoomed in
UN IGME 2020
U5MR
Year
Census 1980 (Census Indirect)
Census 1991 (Census Indirect)
Multiple Indicator Cluster Survey 2000 (MICS Indirect)
Multiple Indicator Cluster Survey 2006 (Others Indirect)
Demographic and Health Survey 2008–2009 (DHS Direct)
Demographic and Health Survey 2008–2009 (DHS Indirect)
Census 2012 (Others Household Deaths)
Multiple Indicator Cluster Survey 2014 (MICS Direct)
VR WHO

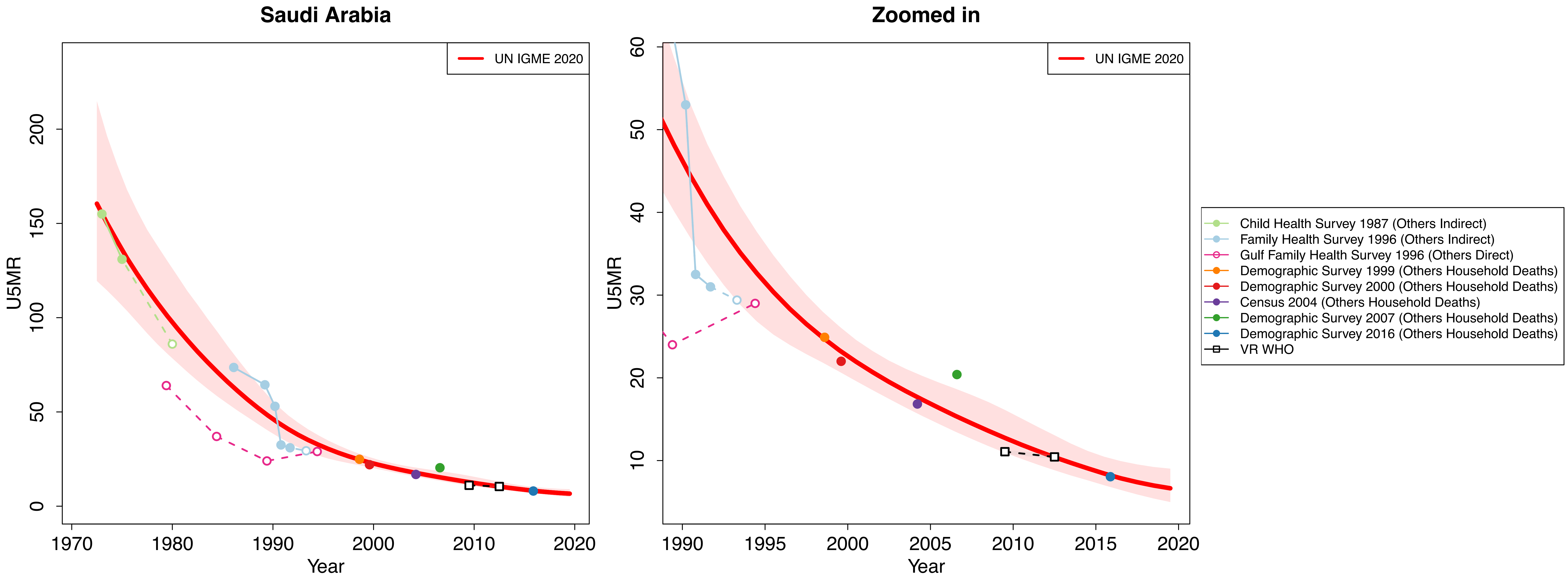
Saudi Arabia
Zoomed in
UN IGME 2020
U5MR
Year
Child Health Survey 1987 (Others Indirect)
Family Health Survey 1996 (Others Indirect)
Gulf Family Health Survey 1996 (Others Direct)
Demographic Survey 1999 (Others Household Deaths)
Demographic Survey 2000 (Others Household Deaths)
Census 2004 (Others Household Deaths)
Demographic Survey 2007 (Others Household Deaths)
Demographic Survey 2016 (Others Household Deaths)
VR WHO

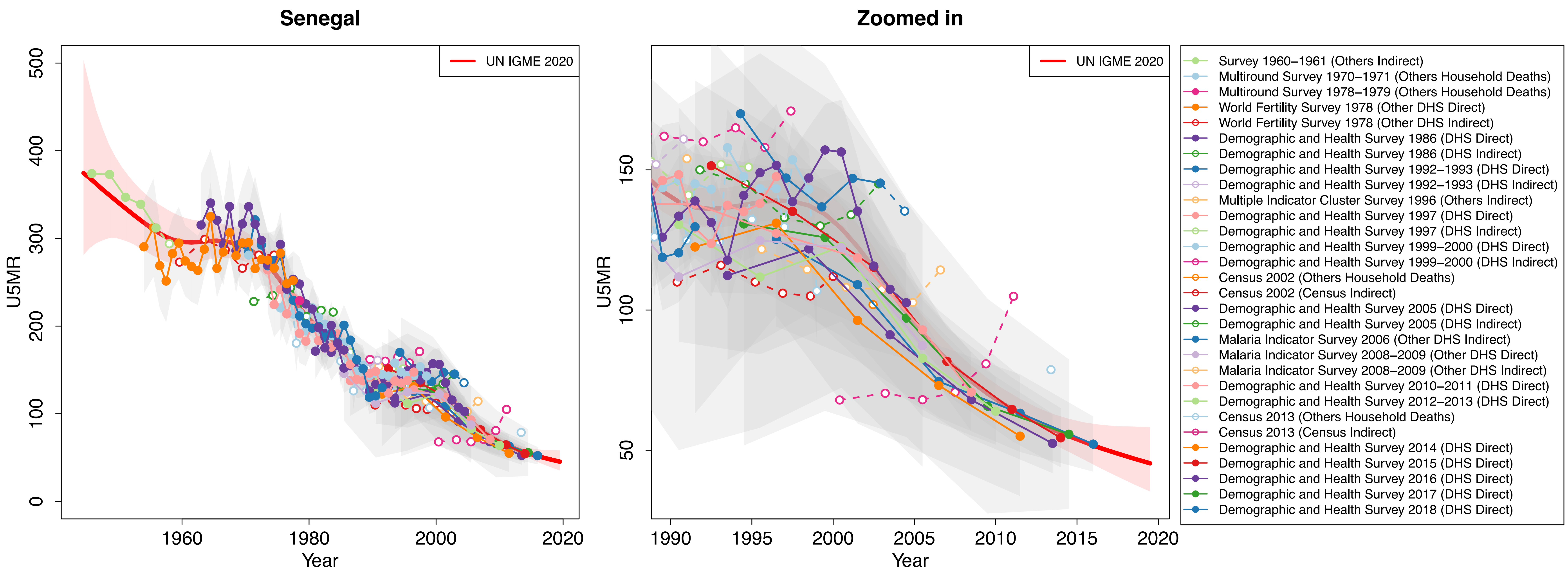

Senegal
Zoomed in
U5MR
Year
UN IGME 2020
0
100
200
300
400
500
1960
1980
2000
2020
50
100
150
1990
1995
2000
2005
2010
2015
2020
Survey 1960–1961 (Others Indirect)
Multiround Survey 1970–1971 (Others Household Deaths)
Multiround Survey 1978–1979 (Others Household Deaths)
World Fertility Survey 1978 (Other DHS Direct)
World Fertility Survey 1978 (Other DHS Indirect)
Demographic and Health Survey 1986 (DHS Direct)
Demographic and Health Survey 1986 (DHS Indirect)
Demographic and Health Survey 1992–1993 (DHS Direct)
Demographic and Health Survey 1992–1993 (DHS Indirect)
Multiple Indicator Cluster Survey 1996 (Others Indirect)
Demographic and Health Survey 1997 (DHS Direct)
Demographic and Health Survey 1997 (DHS Indirect)
Demographic and Health Survey 1999–2000 (DHS Direct)
Demographic and Health Survey 1999–2000 (DHS Indirect)
Census 2002 (Others Household Deaths)
Census 2002 (Census Indirect)
Demographic and Health Survey 2005 (DHS Direct)
Demographic and Health Survey 2005 (DHS Indirect)
Malaria Indicator Survey 2006 (Other DHS Indirect)
Malaria Indicator Survey 2008–2009 (Other DHS Direct)
Malaria Indicator Survey 2008–2009 (Other DHS Indirect)
Demographic and Health Survey 2010–2011 (DHS Direct)
Demographic and Health Survey 2012–2013 (DHS Direct)
Census 2013 (Others Household Deaths)
Census 2013 (Census Indirect)
Demographic and Health Survey 2014 (DHS Direct)
Demographic and Health Survey 2015 (DHS Direct)
Demographic and Health Survey 2016 (DHS Direct)
Demographic and Health Survey 2017 (DHS Direct)
Demographic and Health Survey 2018 (DHS Direct)

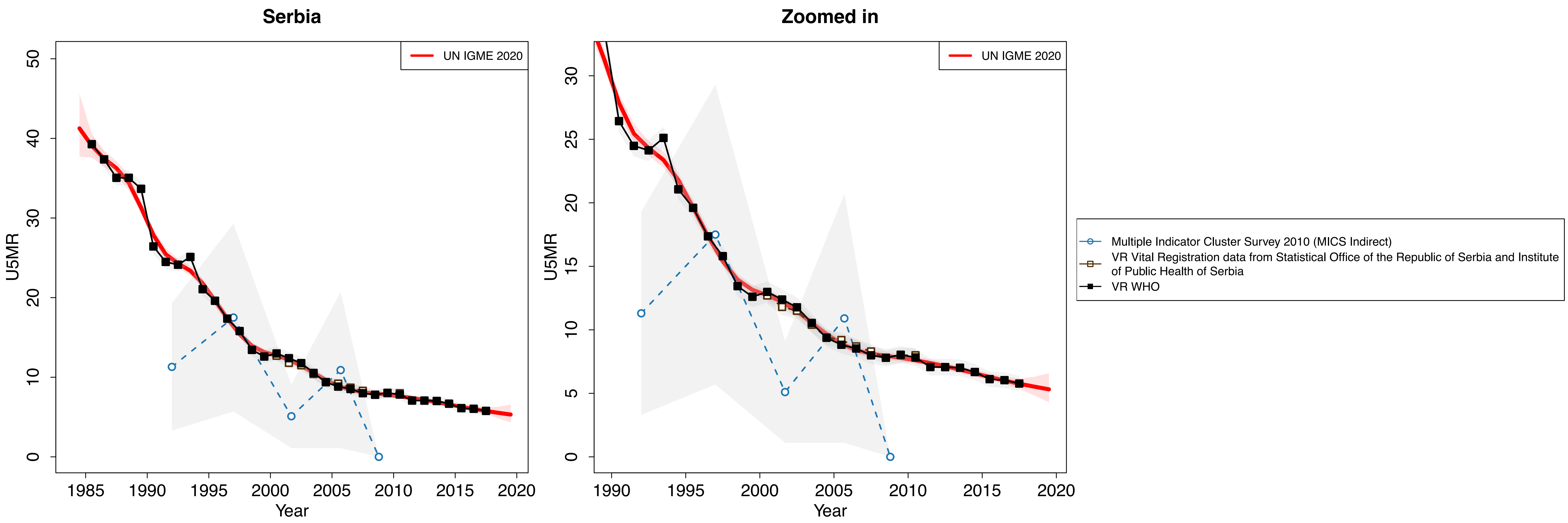

Serbia
Zoomed in
UN IGME 2020
U5MR
Year
Multiple Indicator Cluster Survey 2010 (MICS Indirect)
VR Vital Registration data from Statistical Office of the Republic of Serbia and Institute of Public Health of Serbia
VR WHO

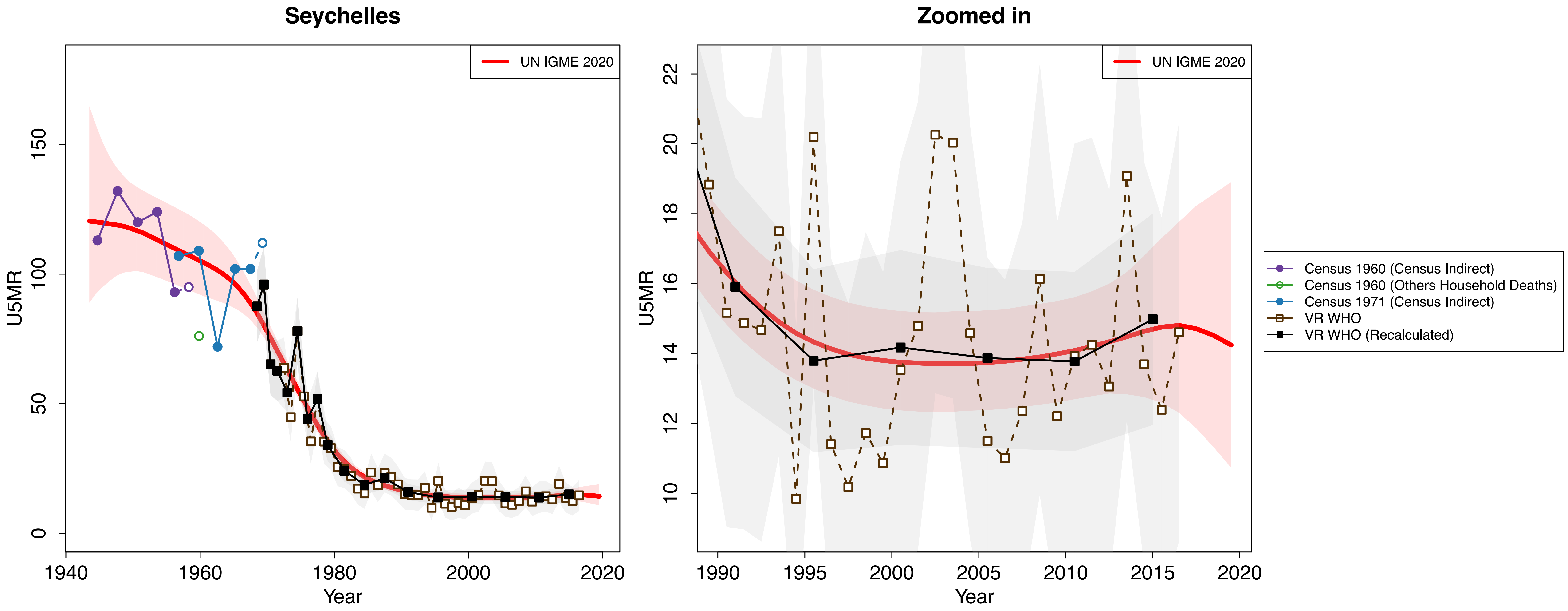
Seychelles
Zoomed in
UN IGME 2020
U5MR
Year
Census 1960 (Census Indirect)
Census 1960 (Others Household Deaths)
Census 1971 (Census Indirect)
VR WHO
VR WHO (Recalculated)

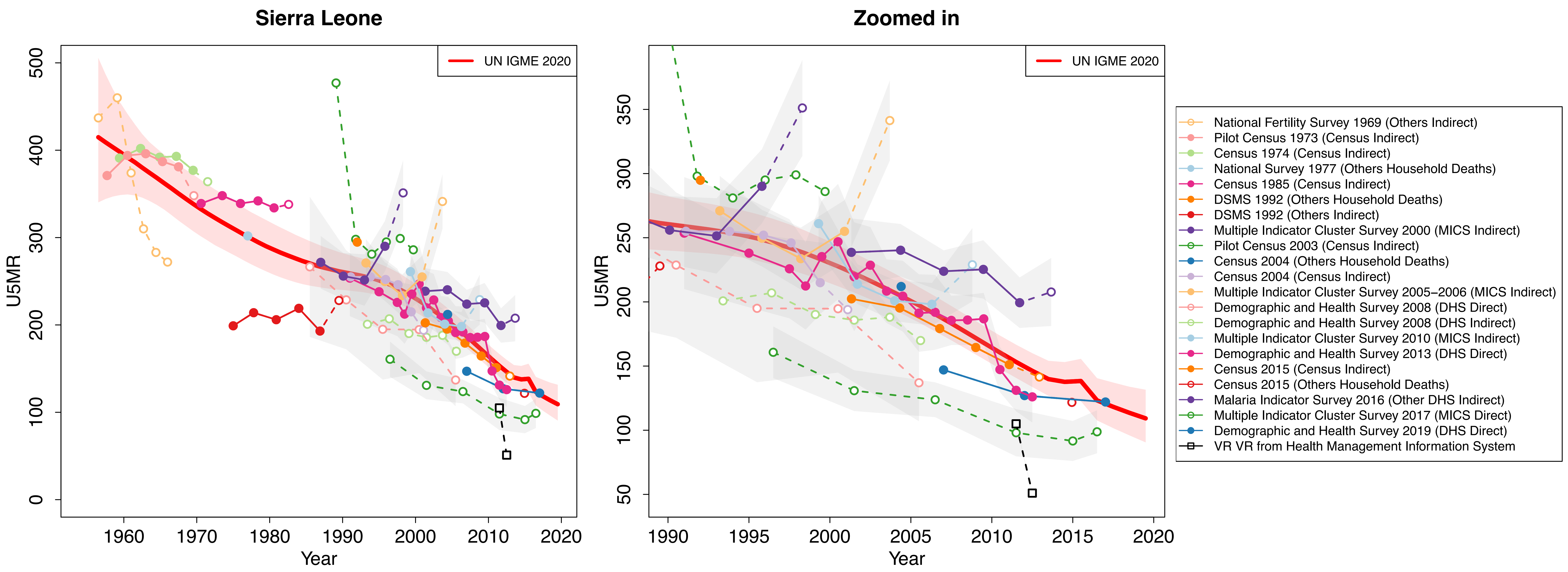
Sierra Leone
Zoomed in
UN IGME 2020
U5MR
Year
0
100
200
300
400
500
50
150
250
350
1960
1970
1980
1990
1995
2000
2005
2010
2015
2020
National Fertility Survey 1969 (Others Indirect)
Pilot Census 1973 (Census Indirect)
Census 1974 (Census Indirect)
National Survey 1977 (Others Household Deaths)
Census 1985 (Census Indirect)
DSMS 1992 (Others Household Deaths)
DSMS 1992 (Others Indirect)
Multiple Indicator Cluster Survey 2000 (MICS Indirect)
Pilot Census 2003 (Census Indirect)
Census 2004 (Others Household Deaths)
Census 2004 (Census Indirect)
Multiple Indicator Cluster Survey 2005–2006 (MICS Indirect)
Demographic and Health Survey 2008 (DHS Direct)
Demographic and Health Survey 2008 (DHS Indirect)
Multiple Indicator Cluster Survey 2010 (MICS Indirect)
Demographic and Health Survey 2013 (DHS Direct)
Census 2015 (Census Indirect)
Census 2015 (Others Household Deaths)
Malaria Indicator Survey 2016 (Other DHS Indirect)
Multiple Indicator Cluster Survey 2017 (MICS Direct)
Demographic and Health Survey 2019 (DHS Direct)
VR VR from Health Management Information System

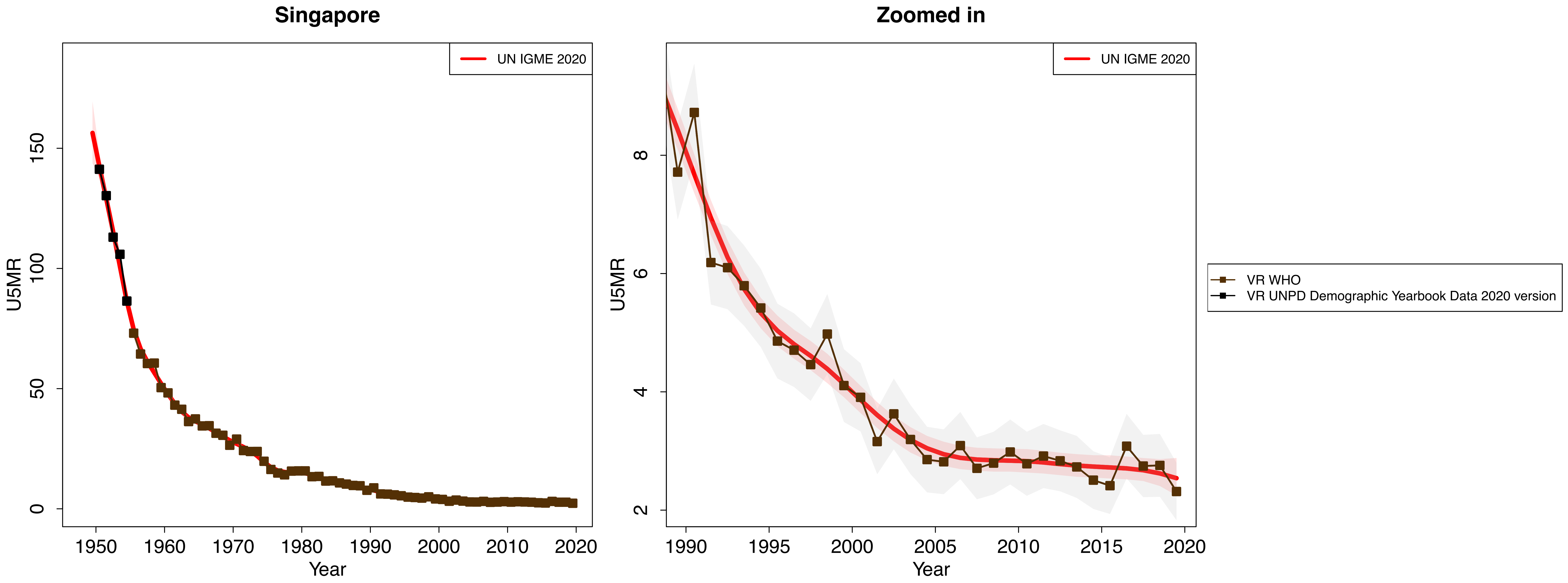

Singapore
UN IGME 2020
U5MR
Year
Zoomed in
VR WHO
VR UNPD Demographic Yearbook Data 2020 version

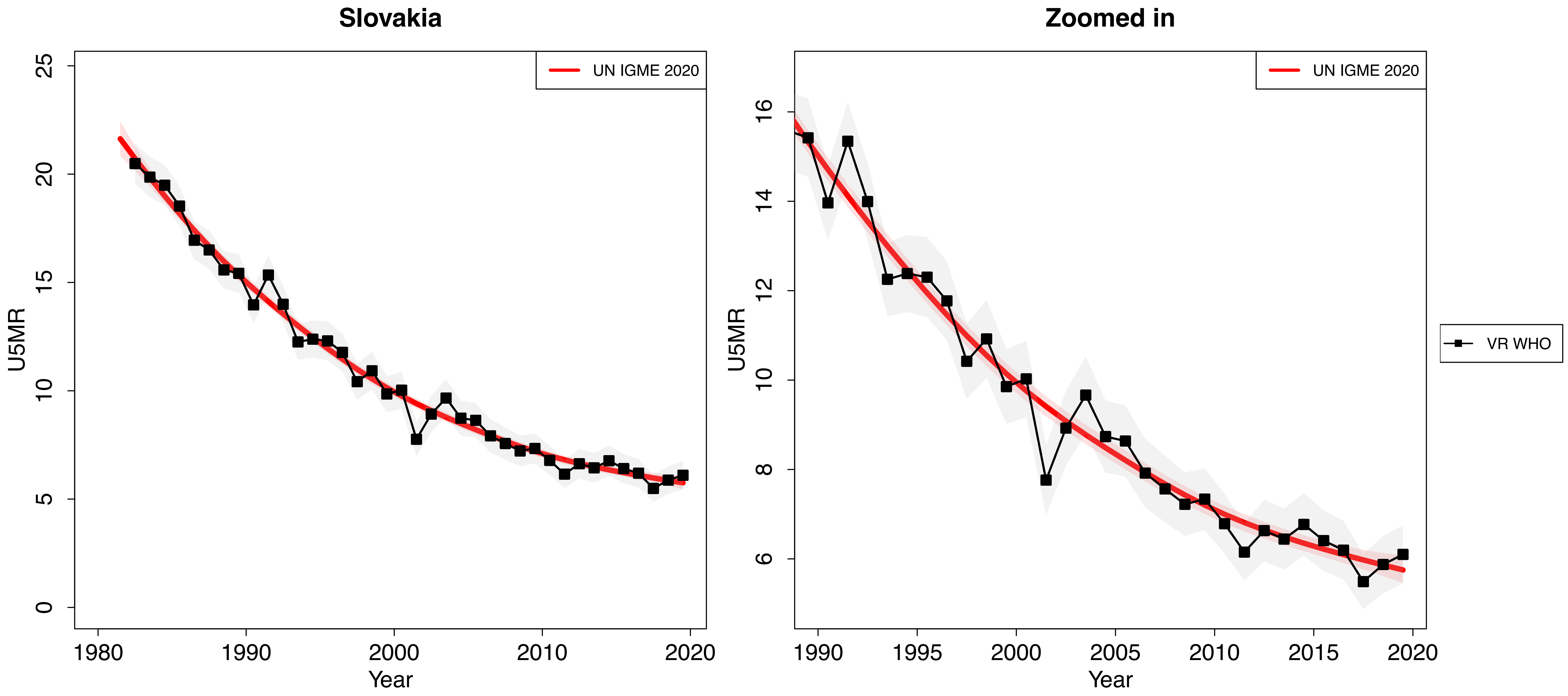
Slovakia
Zoomed in
UN IGME 2020
UN IGME 2020
VR WHO
U5MR
U5MR
Year
Year
0
5
10
15
20
25
6
8
10
12
14
16
1980
1990
2000
2010
2020
1990
1995
2000
2005
2010
2015
2020

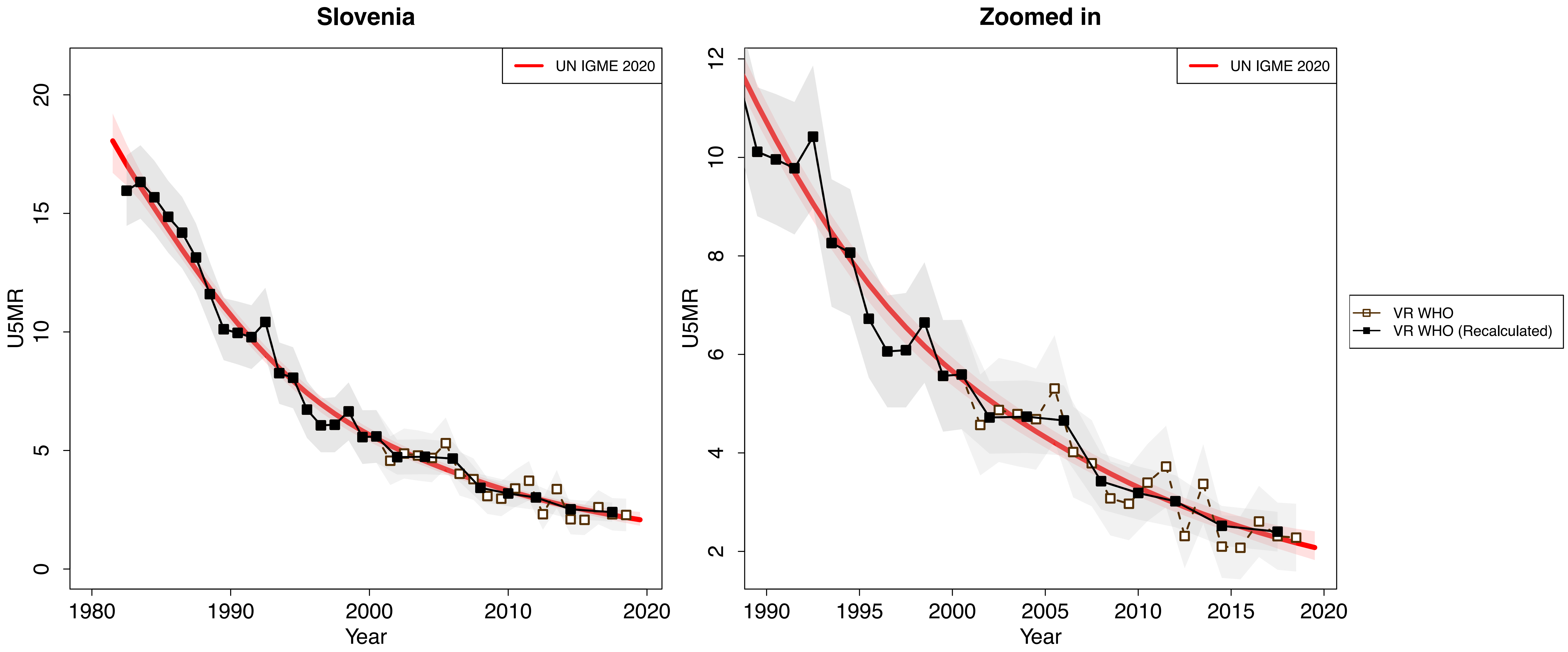

Slovenia
Zoomed in
UN IGME 2020
UN IGME 2020
U5MR
U5MR
Year
Year
VR WHO
VR WHO (Recalculated)

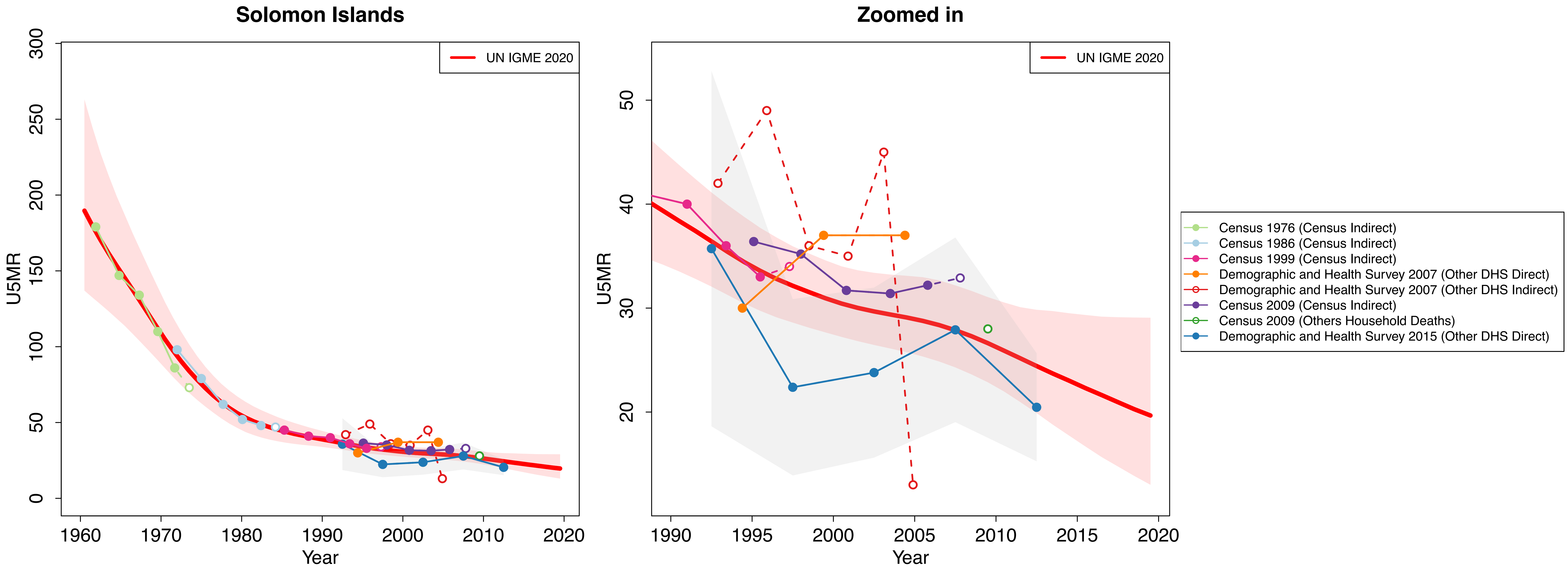

Solomon Islands
Zoomed in
UN IGME 2020
U5MR
Year
Census 1976 (Census Indirect)
Census 1986 (Census Indirect)
Census 1999 (Census Indirect)
Demographic and Health Survey 2007 (Other DHS Direct)
Demographic and Health Survey 2007 (Other DHS Indirect)
Census 2009 (Census Indirect)
Census 2009 (Others Household Deaths)
Demographic and Health Survey 2015 (Other DHS Direct)

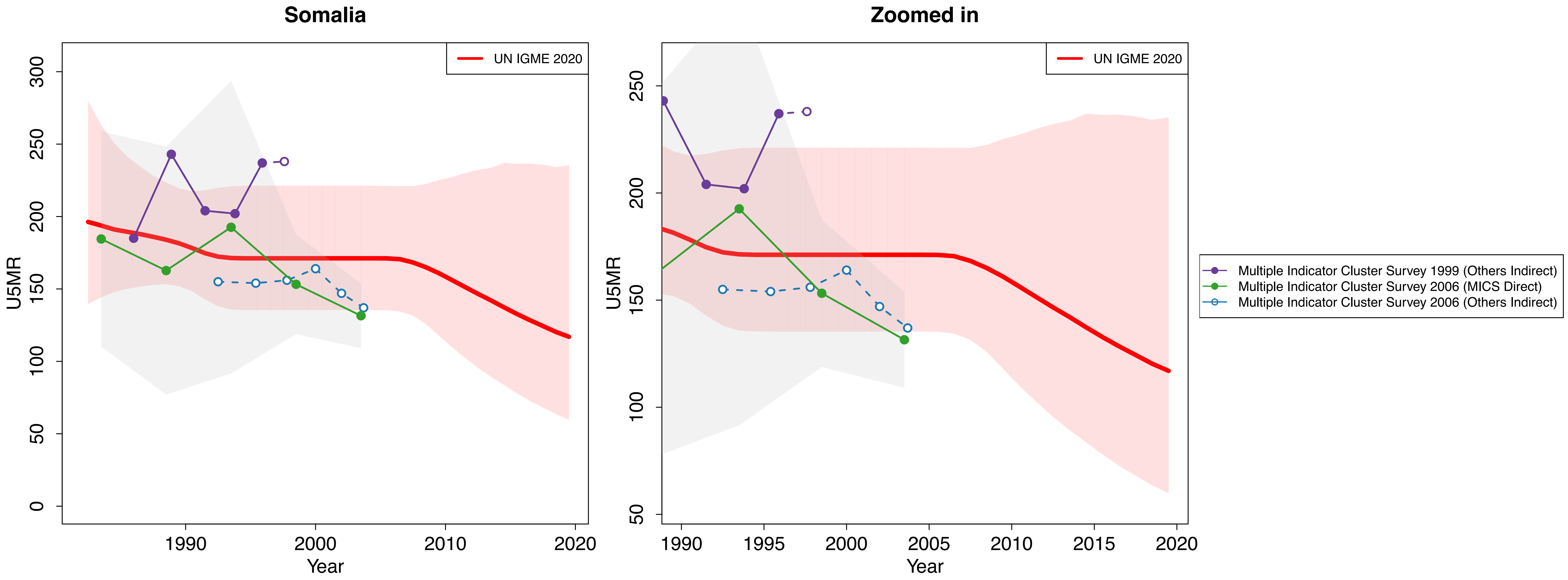

Somalia
Zoomed in
UN IGME 2020
U5MR
Year
Multiple Indicator Cluster Survey 1999 (Others Indirect)
Multiple Indicator Cluster Survey 2006 (MICS Direct)
Multiple Indicator Cluster Survey 2006 (Others Indirect)

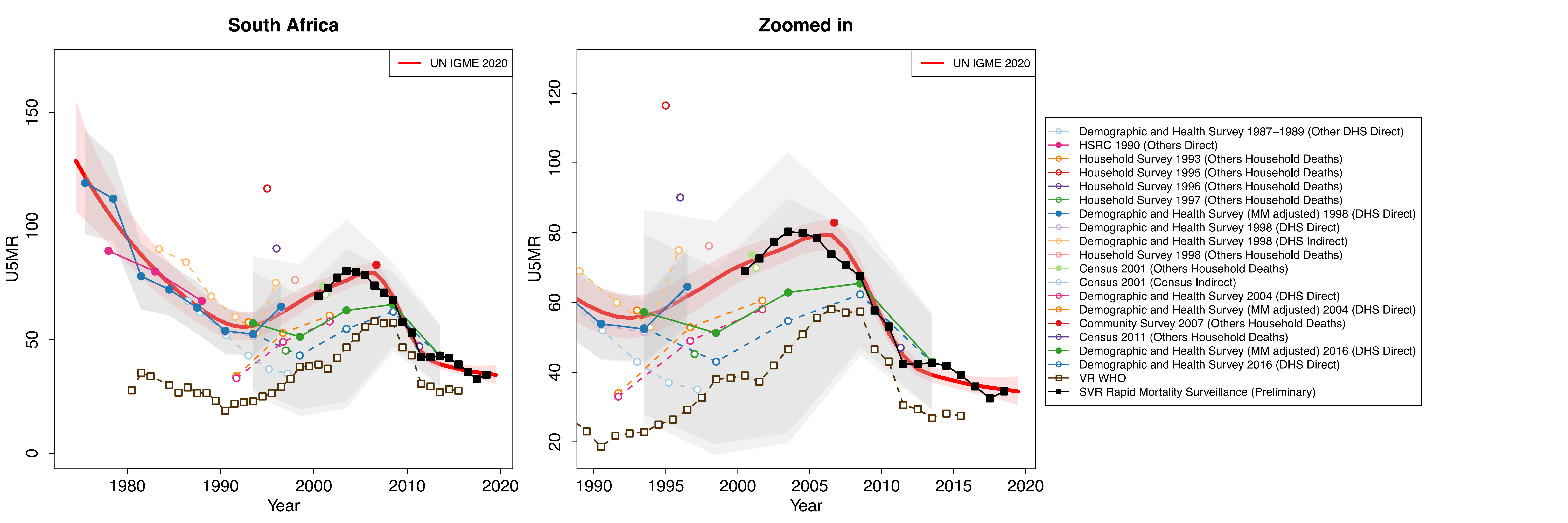

South Africa
Zoomed in
UN IGME 2020
U5MR
Year
0
50
100
150
20
40
60
80
100
120
1980
1990
2000
2010
2020
1995
2005
2015
Demographic and Health Survey 1987–1989 (Other DHS Direct)
HSRC 1990 (Others Direct)
Household Survey 1993 (Others Household Deaths)
Household Survey 1995 (Others Household Deaths)
Household Survey 1996 (Others Household Deaths)
Household Survey 1997 (Others Household Deaths)
Demographic and Health Survey (MM adjusted) 1998 (DHS Direct)
Demographic and Health Survey 1998 (DHS Direct)
Demographic and Health Survey 1998 (DHS Indirect)
Household Survey 1998 (Others Household Deaths)
Census 2001 (Others Household Deaths)
Census 2001 (Census Indirect)
Demographic and Health Survey 2004 (DHS Direct)
Demographic and Health Survey (MM adjusted) 2004 (DHS Direct)
Community Survey 2007 (Others Household Deaths)
Census 2011 (Others Household Deaths)
Demographic and Health Survey (MM adjusted) 2016 (DHS Direct)
Demographic and Health Survey 2016 (DHS Direct)
VR WHO
SVR Rapid Mortality Surveillance (Preliminary)

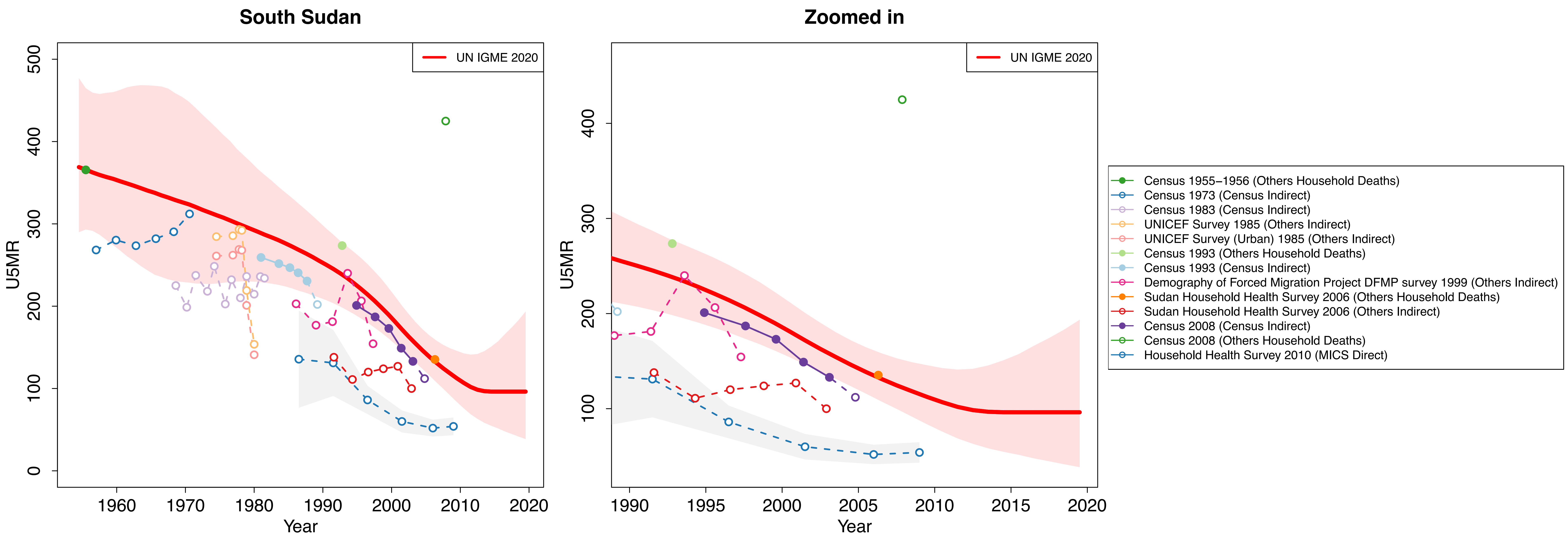
South Sudan
Zoomed in
UN IGME 2020
U5MR
Year
Census 1955–1956 (Others Household Deaths)
Census 1973 (Census Indirect)
Census 1983 (Census Indirect)
UNICEF Survey 1985 (Others Indirect)
UNICEF Survey (Urban) 1985 (Others Indirect)
Census 1993 (Others Household Deaths)
Census 1993 (Census Indirect)
Demography of Forced Migration Project DFMP survey 1999 (Others Indirect)
Sudan Household Health Survey 2006 (Others Household Deaths)
Sudan Household Health Survey 2006 (Others Indirect)
Census 2008 (Census Indirect)
Census 2008 (Others Household Deaths)
Household Health Survey 2010 (MICS Direct)

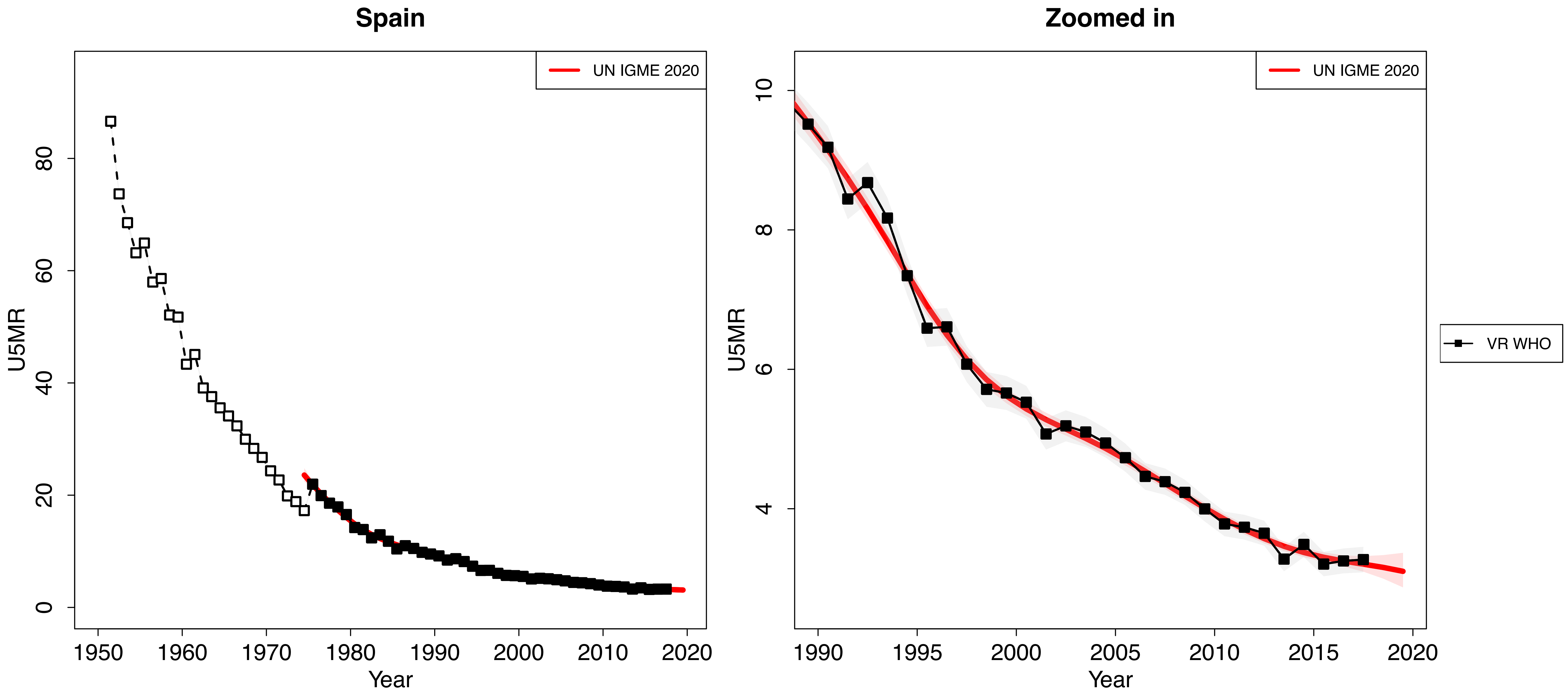

Spain
Zoomed in
UN IGME 2020
UN IGME 2020
VR WHO
U5MR
U5MR
Year
Year
0
20
40
60
80
4
6
8
10
1950
1960
1970
1980
1990
2000
2010
2020
1990
1995
2000
2005
2010
2015
2020

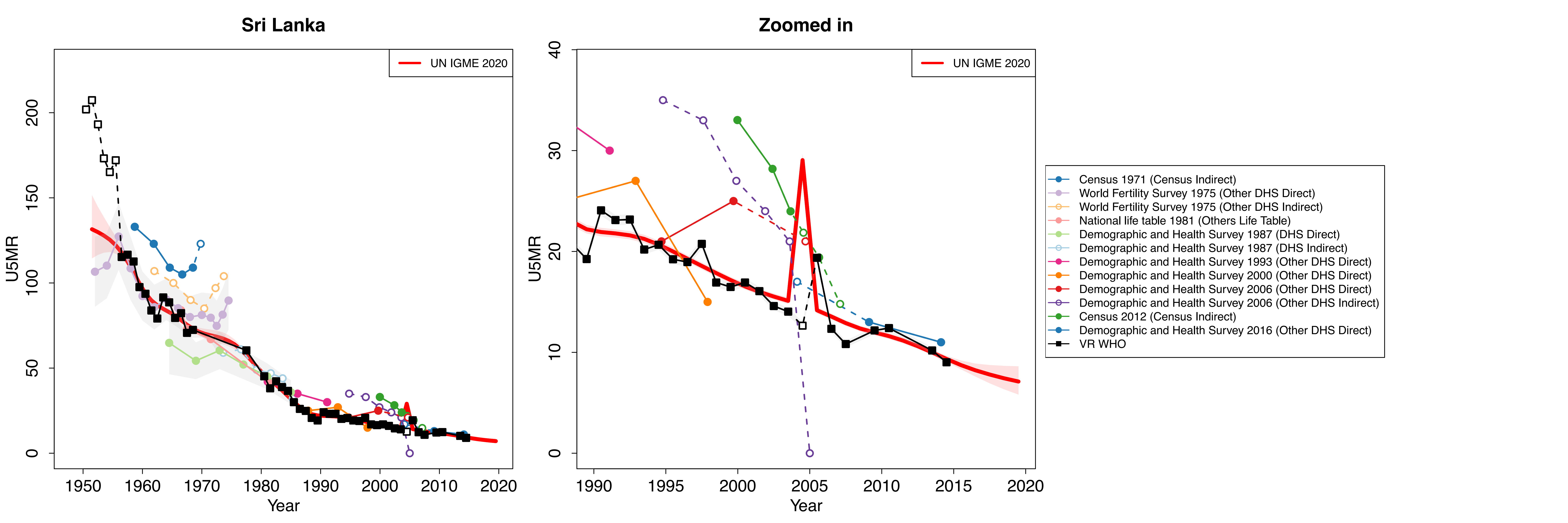
Sri Lanka
Zoomed in
UN IGME 2020
U5MR
Year
Census 1971 (Census Indirect)
World Fertility Survey 1975 (Other DHS Direct)
World Fertility Survey 1975 (Other DHS Indirect)
National life table 1981 (Others Life Table)
Demographic and Health Survey 1987 (DHS Direct)
Demographic and Health Survey 1987 (DHS Indirect)
Demographic and Health Survey 1993 (Other DHS Direct)
Demographic and Health Survey 2000 (Other DHS Direct)
Demographic and Health Survey 2006 (Other DHS Direct)
Demographic and Health Survey 2006 (Other DHS Indirect)
Census 2012 (Census Indirect)
Demographic and Health Survey 2016 (Other DHS Direct)
VR WHO

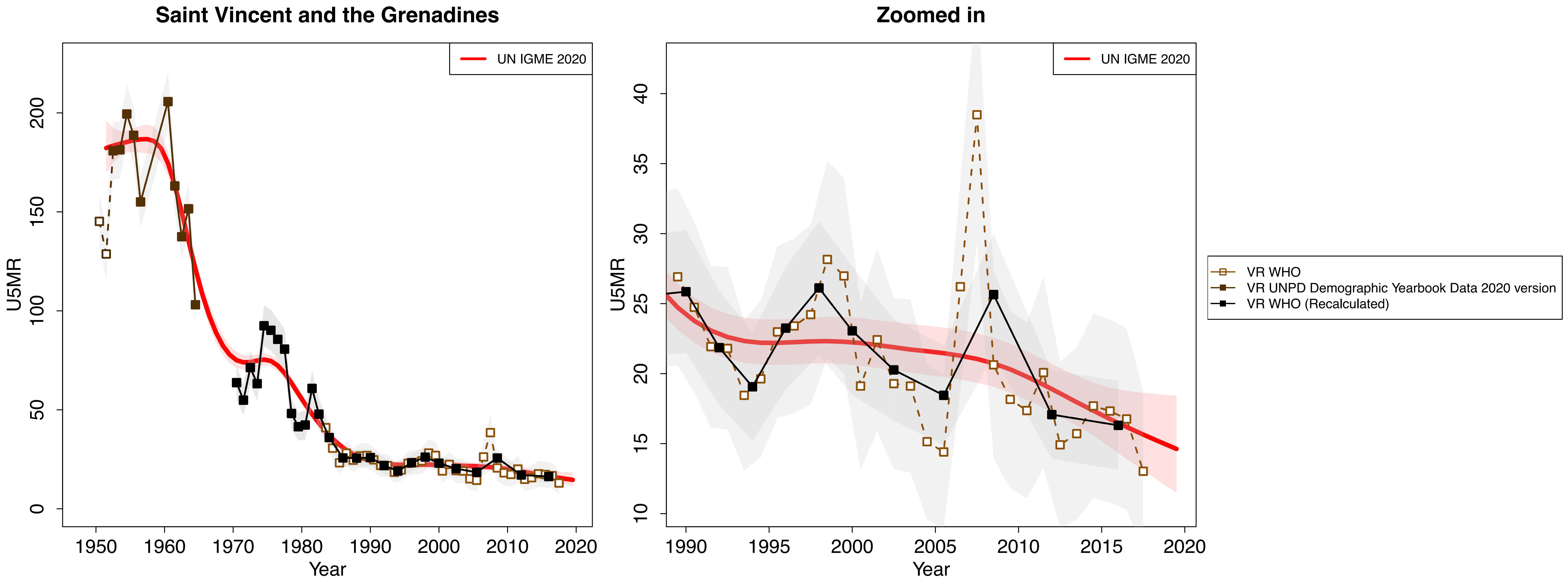

Saint Vincent and the Grenadines
Zoomed in
UN IGME 2020
U5MR
Year
VR WHO
VR UNPD Demographic Yearbook Data 2020 version
VR WHO (Recalculated)

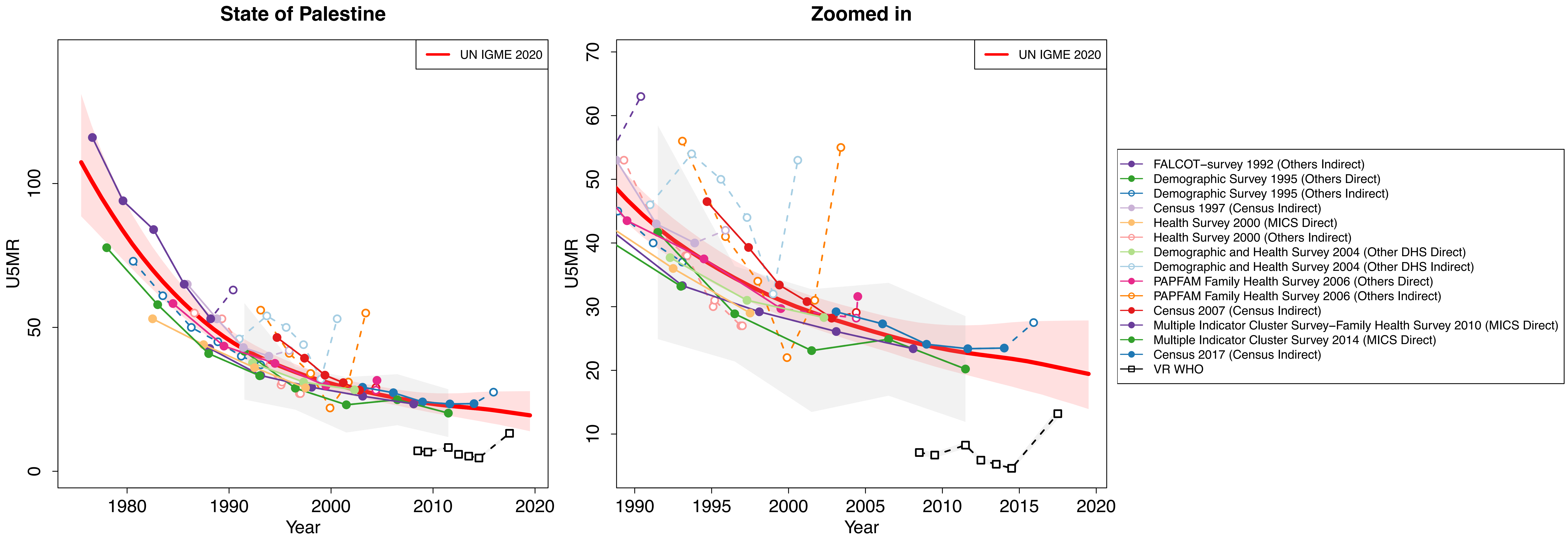

State of Palestine
Zoomed in
UN IGME 2020
U5MR
Year
0
50
100
10
20
30
40
50
60
70
1980
1990
2000
2010
2020
1995
2005
2015
FALCOT−survey 1992 (Others Indirect)
Demographic Survey 1995 (Others Direct)
Demographic Survey 1995 (Others Indirect)
Census 1997 (Census Indirect)
Health Survey 2000 (MICS Direct)
Health Survey 2000 (Others Indirect)
Demographic and Health Survey 2004 (Other DHS Direct)
Demographic and Health Survey 2004 (Other DHS Indirect)
PAPFAM Family Health Survey 2006 (Others Direct)
PAPFAM Family Health Survey 2006 (Others Indirect)
Census 2007 (Census Indirect)
Multiple Indicator Cluster Survey−Family Health Survey 2010 (MICS Direct)
Multiple Indicator Cluster Survey 2014 (MICS Direct)
Census 2017 (Census Indirect)
VR WHO

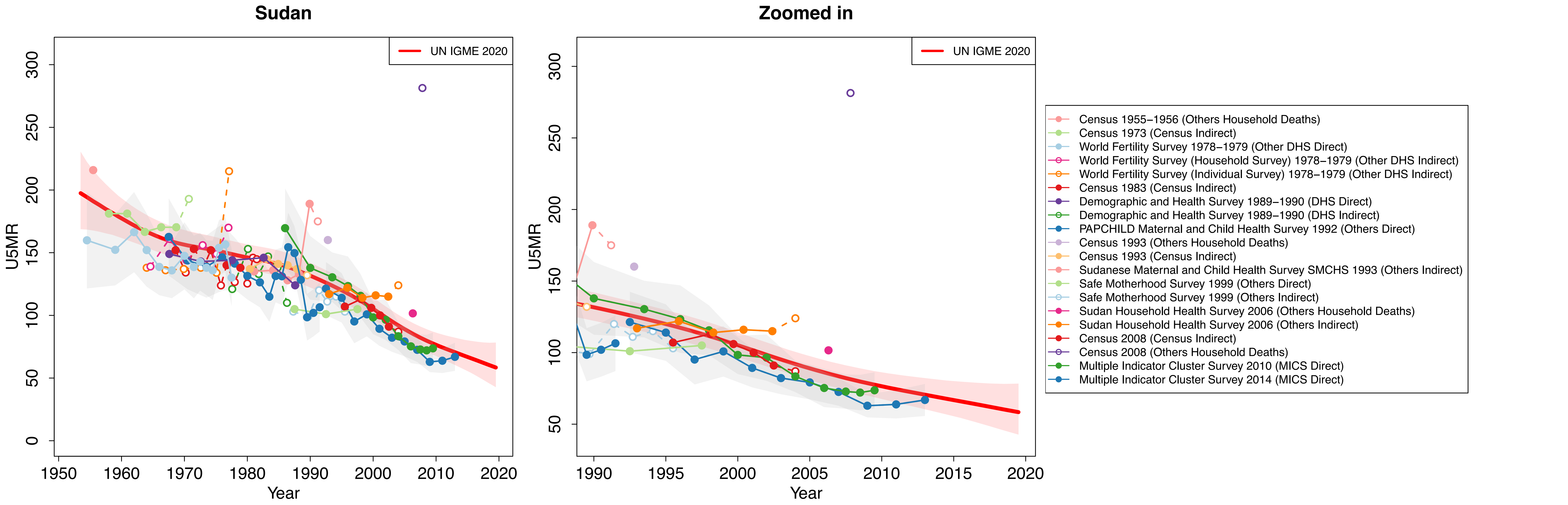

Sudan
Zoomed in
UN IGME 2020
U5MR
Year
Census 1955–1956 (Others Household Deaths)
Census 1973 (Census Indirect)
World Fertility Survey 1978–1979 (Other DHS Direct)
World Fertility Survey (Household Survey) 1978–1979 (Other DHS Indirect)
World Fertility Survey (Individual Survey) 1978–1979 (Other DHS Indirect)
Census 1983 (Census Indirect)
Demographic and Health Survey 1989–1990 (DHS Direct)
Demographic and Health Survey 1989–1990 (DHS Indirect)
PAPCHILD Maternal and Child Health Survey 1992 (Others Direct)
Census 1993 (Others Household Deaths)
Census 1993 (Census Indirect)
Sudanese Maternal and Child Health Survey SMCHS 1993 (Others Indirect)
Safe Motherhood Survey 1999 (Others Direct)
Safe Motherhood Survey 1999 (Others Indirect)
Sudan Household Health Survey 2006 (Others Household Deaths)
Sudan Household Health Survey 2006 (Others Indirect)
Census 2008 (Census Indirect)
Census 2008 (Others Household Deaths)
Multiple Indicator Cluster Survey 2010 (MICS Direct)
Multiple Indicator Cluster Survey 2014 (MICS Direct)

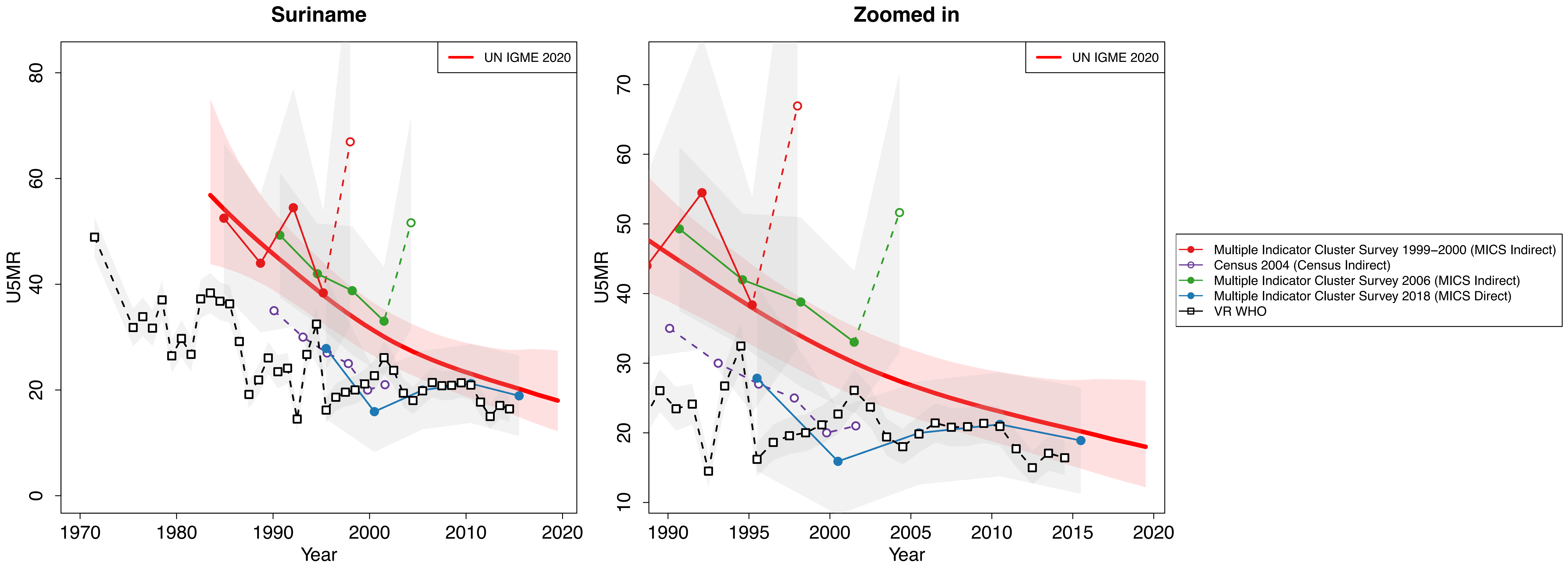

Suriname
Zoomed in
UN IGME 2020
U5MR
Year
Multiple Indicator Cluster Survey 1999–2000 (MICS Indirect)
Census 2004 (Census Indirect)
Multiple Indicator Cluster Survey 2006 (MICS Indirect)
Multiple Indicator Cluster Survey 2018 (MICS Direct)
VR WHO

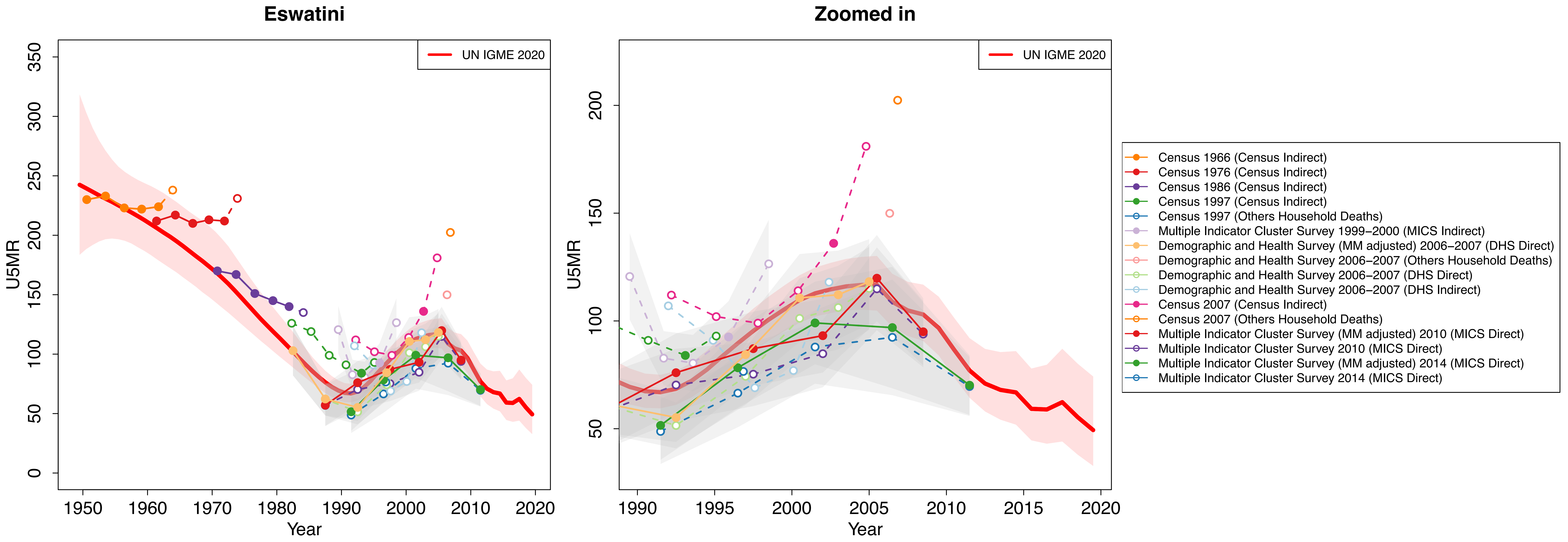

Eswatini
Zoomed in
UN IGME 2020
U5MR
Year
Census 1966 (Census Indirect)
Census 1976 (Census Indirect)
Census 1986 (Census Indirect)
Census 1997 (Census Indirect)
Census 1997 (Others Household Deaths)
Multiple Indicator Cluster Survey 1999–2000 (MICS Indirect)
Demographic and Health Survey (MM adjusted) 2006–2007 (DHS Direct)
Demographic and Health Survey 2006–2007 (Others Household Deaths)
Demographic and Health Survey 2006–2007 (DHS Direct)
Demographic and Health Survey 2006–2007 (DHS Indirect)
Census 2007 (Census Indirect)
Census 2007 (Others Household Deaths)
Multiple Indicator Cluster Survey (MM adjusted) 2010 (MICS Direct)
Multiple Indicator Cluster Survey 2010 (MICS Direct)
Multiple Indicator Cluster Survey (MM adjusted) 2014 (MICS Direct)
Multiple Indicator Cluster Survey 2014 (MICS Direct)

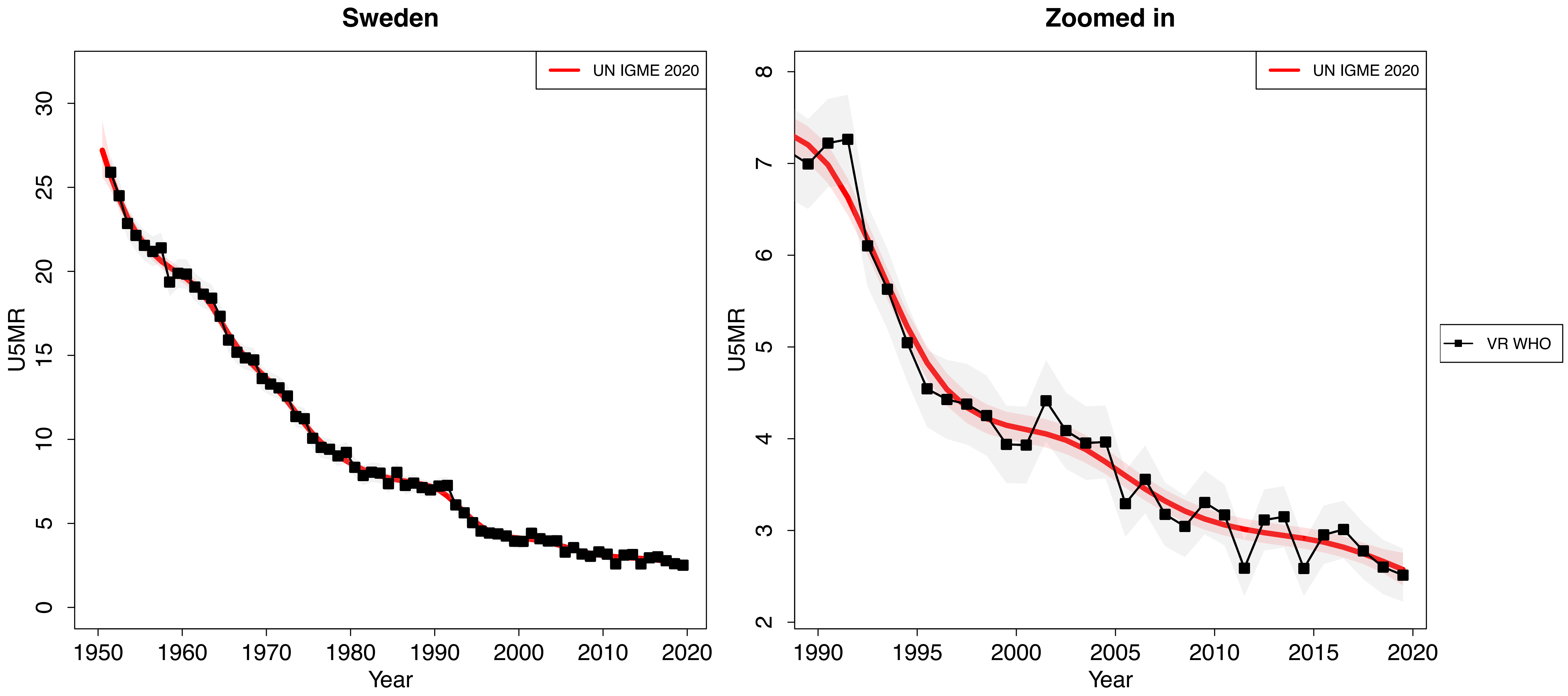
Sweden
Zoomed in
UN IGME 2020
UN IGME 2020
VR WHO
U5MR
U5MR
Year
Year
0
5
10
15
20
25
30
1950
1960
1970
1980
1990
2000
2010
2020
2
3
4
5
6
7
8
1990
1995
2000
2005
2010
2015
2020

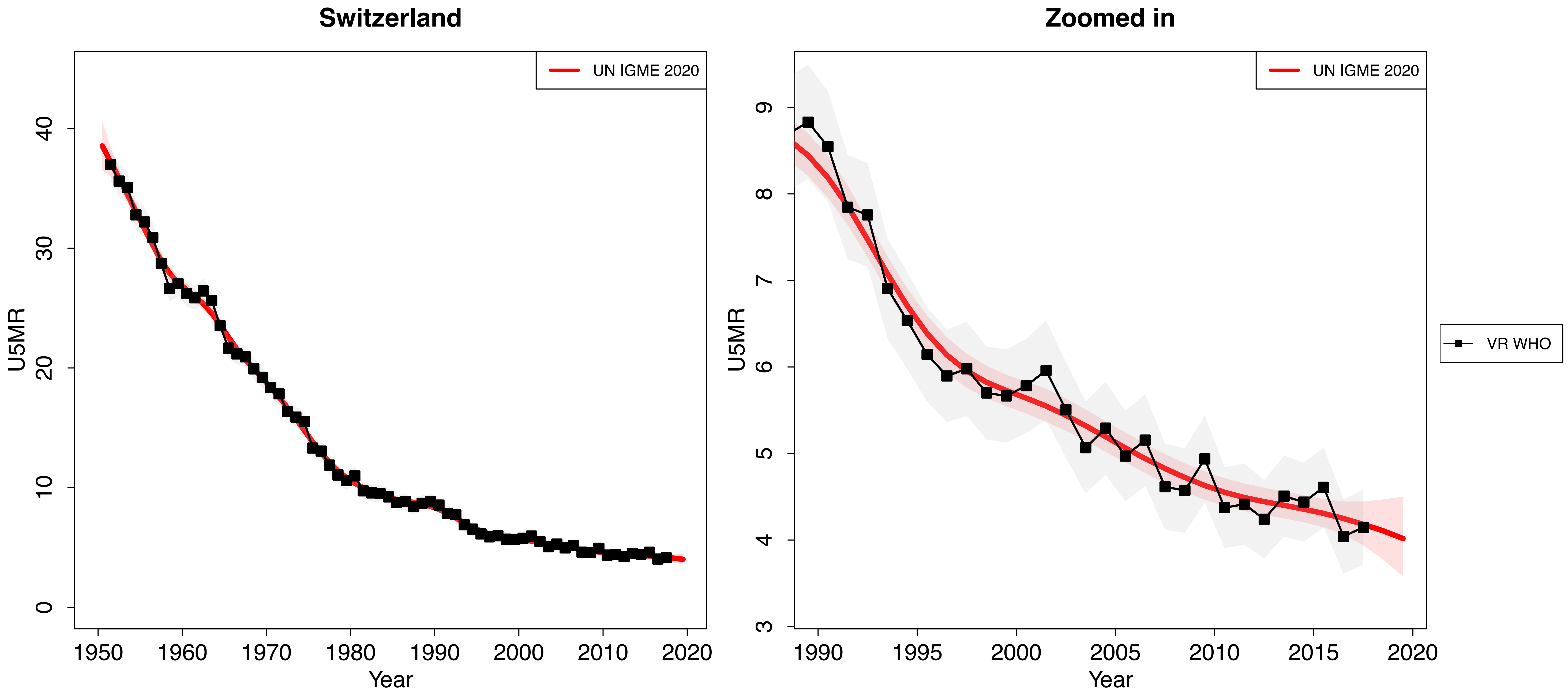

Switzerland
Zoomed in
UN IGME 2020
UN IGME 2020
VR WHO
U5MR
U5MR
Year
Year
0
10
20
30
40
3
4
5
6
7
8
9
1950
1960
1970
1980
1990
2000
2010
2020
1990
1995
2000
2005
2010
2015
2020

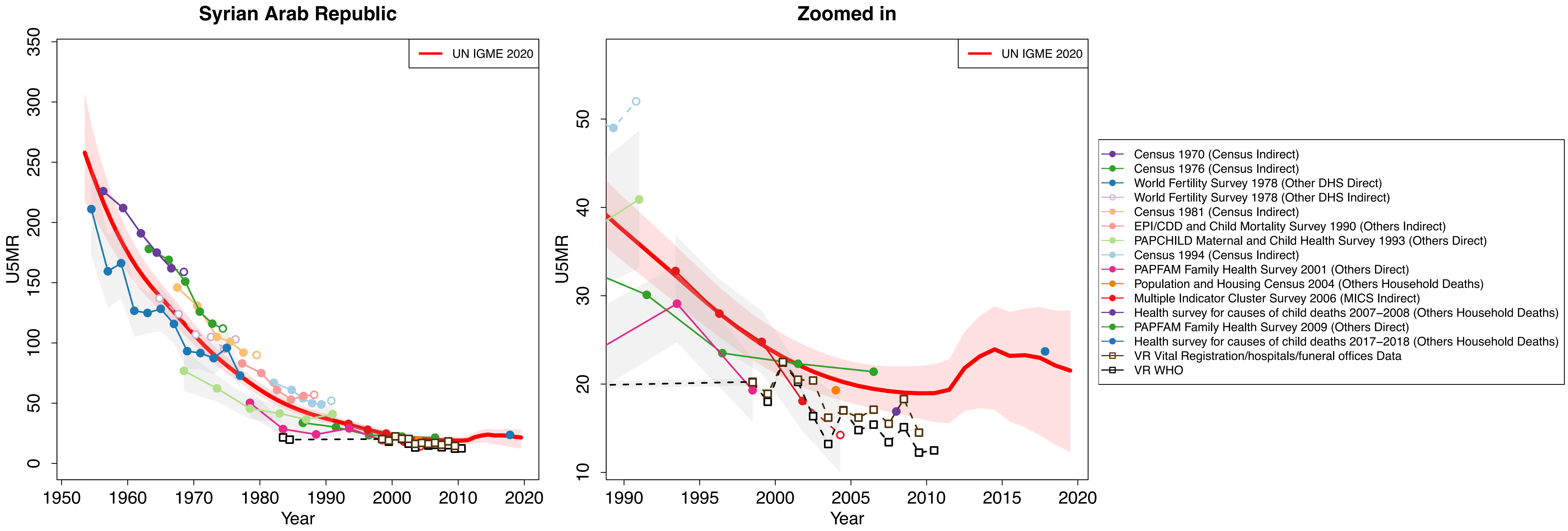

Syrian Arab Republic
Zoomed in
UN IGME 2020
U5MR
Year
Census 1970 (Census Indirect)
Census 1976 (Census Indirect)
World Fertility Survey 1978 (Other DHS Direct)
World Fertility Survey 1978 (Other DHS Indirect)
Census 1981 (Census Indirect)
EPI/CDD and Child Mortality Survey 1990 (Others Indirect)
PAPCHILD Maternal and Child Health Survey 1993 (Others Direct)
Census 1994 (Census Indirect)
PAPFAM Family Health Survey 2001 (Others Direct)
Population and Housing Census 2004 (Others Household Deaths)
Multiple Indicator Cluster Survey 2006 (MICS Indirect)
Health survey for causes of child deaths 2007–2008 (Others Household Deaths)
PAPFAM Family Health Survey 2009 (Others Direct)
Health survey for causes of child deaths 2017–2018 (Others Household Deaths)
VR Vital Registration/hospitals/funeral offices Data
VR WHO

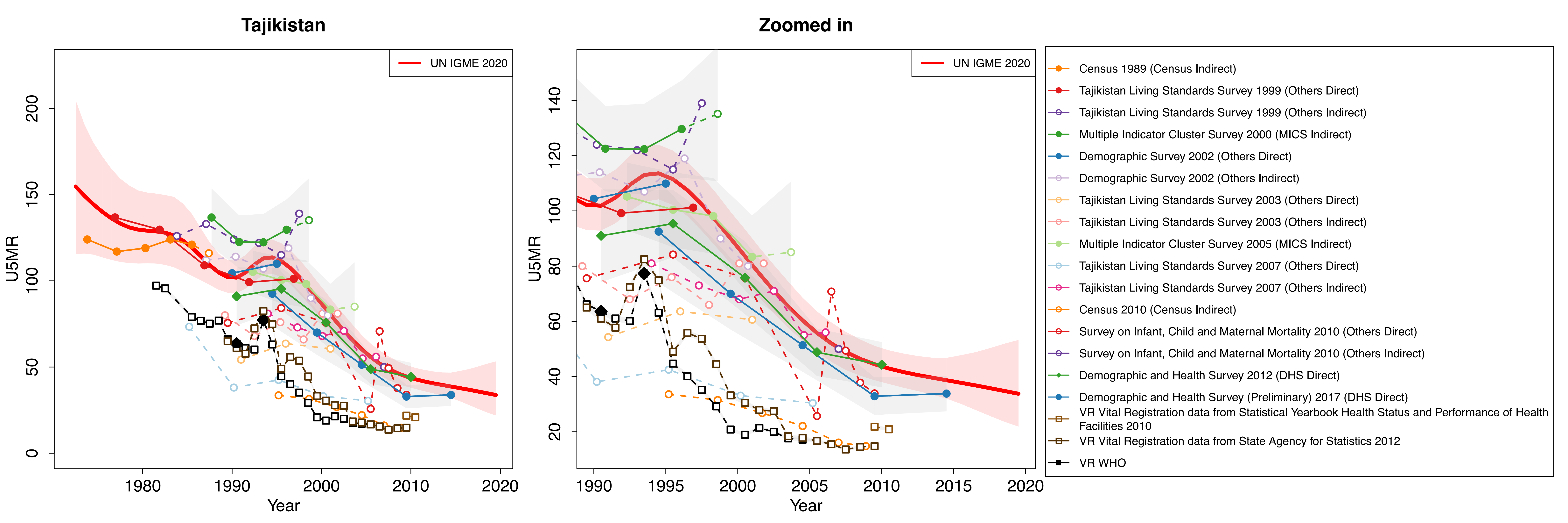

Tajikistan
Zoomed in
UN IGME 2020
U5MR
Year
Census 1989 (Census Indirect)
Tajikistan Living Standards Survey 1999 (Others Direct)
Tajikistan Living Standards Survey 1999 (Others Indirect)
Multiple Indicator Cluster Survey 2000 (MICS Indirect)
Demographic Survey 2002 (Others Direct)
Demographic Survey 2002 (Others Indirect)
Tajikistan Living Standards Survey 2003 (Others Direct)
Tajikistan Living Standards Survey 2003 (Others Indirect)
Multiple Indicator Cluster Survey 2005 (MICS Indirect)
Tajikistan Living Standards Survey 2007 (Others Direct)
Tajikistan Living Standards Survey 2007 (Others Indirect)
Census 2010 (Census Indirect)
Survey on Infant, Child and Maternal Mortality 2010 (Others Direct)
Survey on Infant, Child and Maternal Mortality 2010 (Others Indirect)
Demographic and Health Survey 2012 (DHS Direct)
Demographic and Health Survey (Preliminary) 2017 (DHS Direct)
VR Vital Registration data from Statistical Yearbook Health Status and Performance of Health Facilities 2010
VR Vital Registration data from State Agency for Statistics 2012
VR WHO

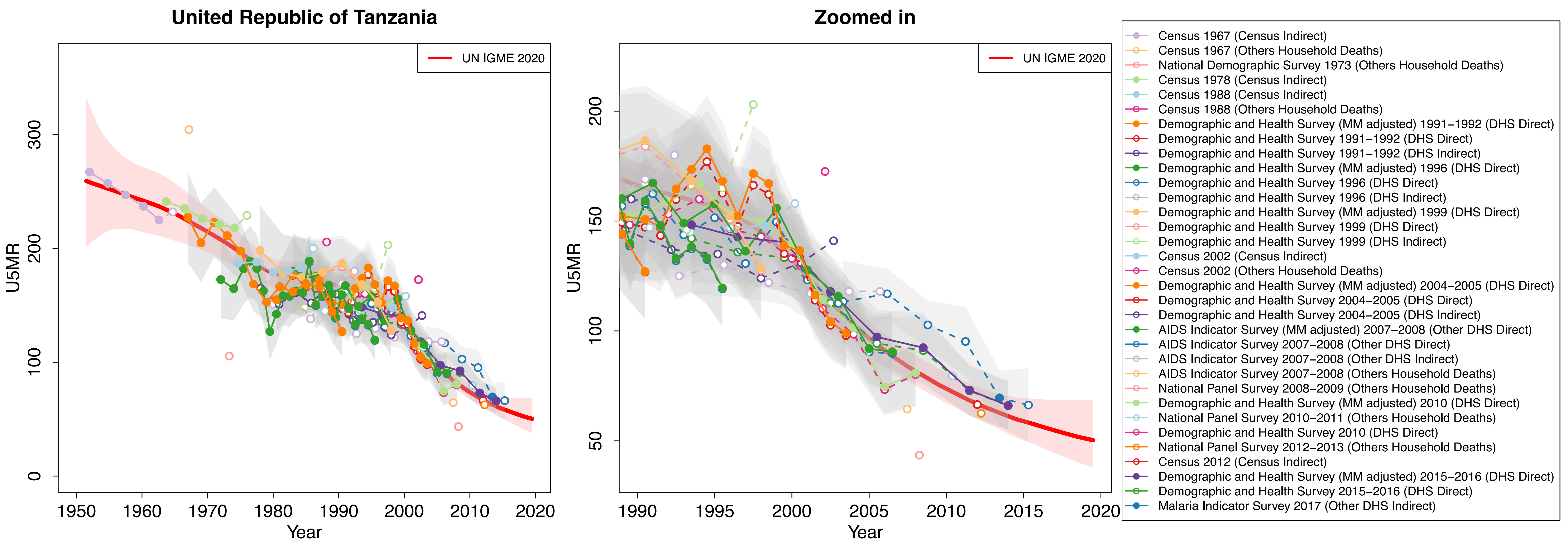

United Republic of Tanzania
Zoomed in
UN IGME 2020
U5MR
Year
Census 1967 (Census Indirect)
Census 1967 (Others Household Deaths)
National Demographic Survey 1973 (Others Household Deaths)
Census 1978 (Census Indirect)
Census 1988 (Census Indirect)
Census 1988 (Others Household Deaths)
Demographic and Health Survey (MM adjusted) 1991–1992 (DHS Direct)
Demographic and Health Survey 1991–1992 (DHS Direct)
Demographic and Health Survey 1991–1992 (DHS Indirect)
Demographic and Health Survey (MM adjusted) 1996 (DHS Direct)
Demographic and Health Survey 1996 (DHS Direct)
Demographic and Health Survey 1996 (DHS Indirect)
Demographic and Health Survey (MM adjusted) 1999 (DHS Direct)
Demographic and Health Survey 1999 (DHS Direct)
Demographic and Health Survey 1999 (DHS Indirect)
Census 2002 (Census Indirect)
Census 2002 (Others Household Deaths)
Demographic and Health Survey (MM adjusted) 2004–2005 (DHS Direct)
Demographic and Health Survey 2004–2005 (DHS Direct)
Demographic and Health Survey 2004–2005 (DHS Indirect)
AIDS Indicator Survey (MM adjusted) 2007–2008 (Other DHS Direct)
AIDS Indicator Survey 2007–2008 (Other DHS Direct)
AIDS Indicator Survey 2007–2008 (Other DHS Indirect)
AIDS Indicator Survey 2007–2008 (Others Household Deaths)
National Panel Survey 2008–2009 (Others Household Deaths)
Demographic and Health Survey (MM adjusted) 2010 (DHS Direct)
National Panel Survey 2010–2011 (Others Household Deaths)
Demographic and Health Survey 2010 (DHS Direct)
National Panel Survey 2012–2013 (Others Household Deaths)
Census 2012 (Census Indirect)
Demographic and Health Survey (MM adjusted) 2015–2016 (DHS Direct)
Demographic and Health Survey 2015–2016 (DHS Direct)
Malaria Indicator Survey 2017 (Other DHS Indirect)

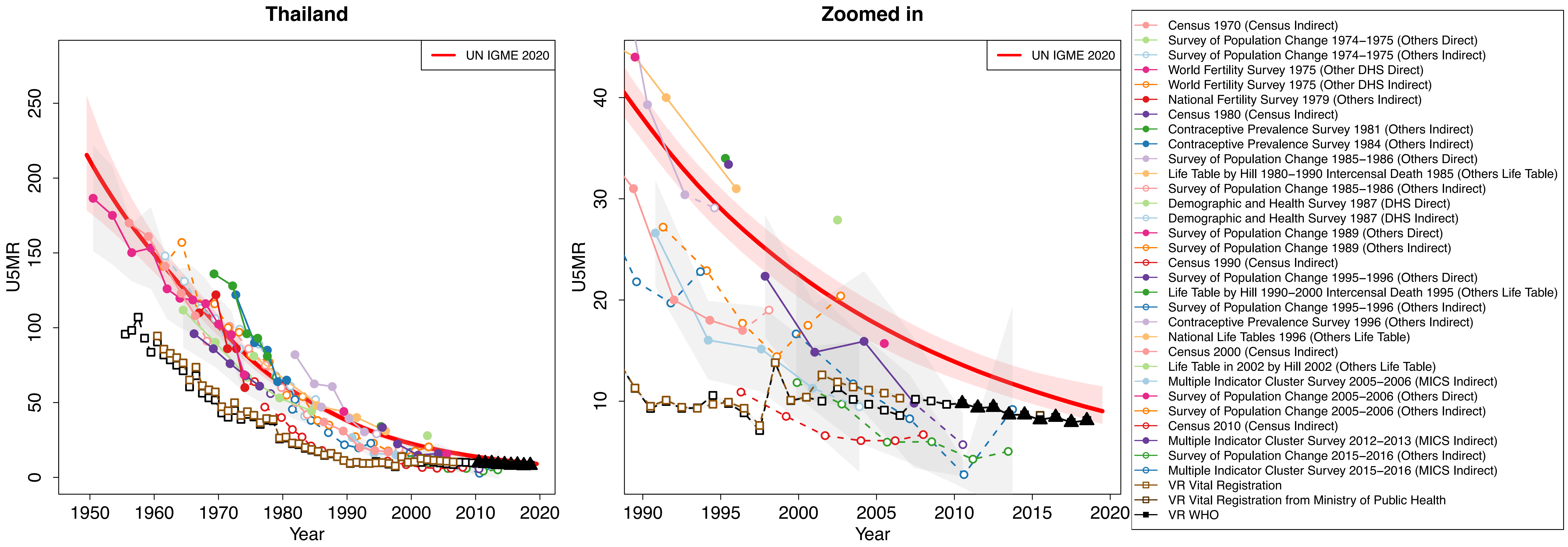
Thailand
Zoomed in
UN IGME 2020
U5MR
Year
0
50
100
150
200
250
1950
1960
1970
1980
1990
2000
2010
2020
10
20
30
40
1995
2005
2015
2020
Census 1970 (Census Indirect)
Survey of Population Change 1974–1975 (Others Direct)
Survey of Population Change 1974–1975 (Others Indirect)
World Fertility Survey 1975 (Other DHS Direct)
World Fertility Survey 1975 (Other DHS Indirect)
National Fertility Survey 1979 (Others Indirect)
Census 1980 (Census Indirect)
Contraceptive Prevalence Survey 1981 (Others Indirect)
Contraceptive Prevalence Survey 1984 (Others Indirect)
Survey of Population Change 1985–1986 (Others Direct)
Life Table by Hill 1980–1990 Intercensal Death 1985 (Others Life Table)
Survey of Population Change 1985–1986 (Others Indirect)
Demographic and Health Survey 1987 (DHS Direct)
Demographic and Health Survey 1987 (DHS Indirect)
Survey of Population Change 1989 (Others Direct)
Survey of Population Change 1989 (Others Indirect)
Census 1990 (Census Indirect)
Survey of Population Change 1995–1996 (Others Direct)
Life Table by Hill 1990–2000 Intercensal Death 1995 (Others Life Table)
Survey of Population Change 1995–1996 (Others Indirect)
Contraceptive Prevalence Survey 1996 (Others Indirect)
National Life Tables 1996 (Others Life Table)
Census 2000 (Census Indirect)
Life Table in 2002 by Hill 2002 (Others Life Table)
Multiple Indicator Cluster Survey 2005–2006 (MICS Indirect)
Survey of Population Change 2005–2006 (Others Direct)
Survey of Population Change 2005–2006 (Others Indirect)
Census 2010 (Census Indirect)
Multiple Indicator Cluster Survey 2012–2013 (MICS Indirect)
Survey of Population Change 2015–2016 (Others Indirect)
Multiple Indicator Cluster Survey 2015–2016 (MICS Indirect)
VR Vital Registration
VR Vital Registration from Ministry of Public Health
VR WHO

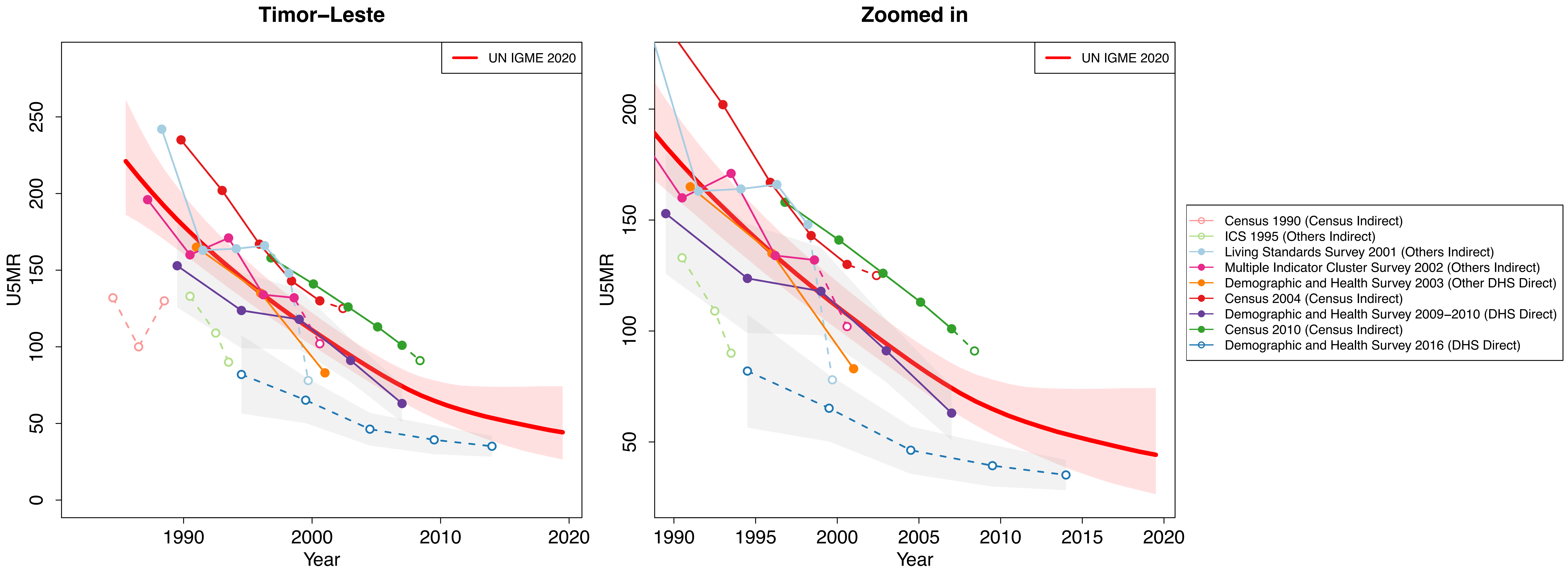

Timor−Leste
Zoomed in
UN IGME 2020
U5MR
Year
Census 1990 (Census Indirect)
ICS 1995 (Others Indirect)
Living Standards Survey 2001 (Others Indirect)
Multiple Indicator Cluster Survey 2002 (Others Indirect)
Demographic and Health Survey 2003 (Other DHS Direct)
Census 2004 (Census Indirect)
Demographic and Health Survey 2009−2010 (DHS Direct)
Census 2010 (Census Indirect)
Demographic and Health Survey 2016 (DHS Direct)

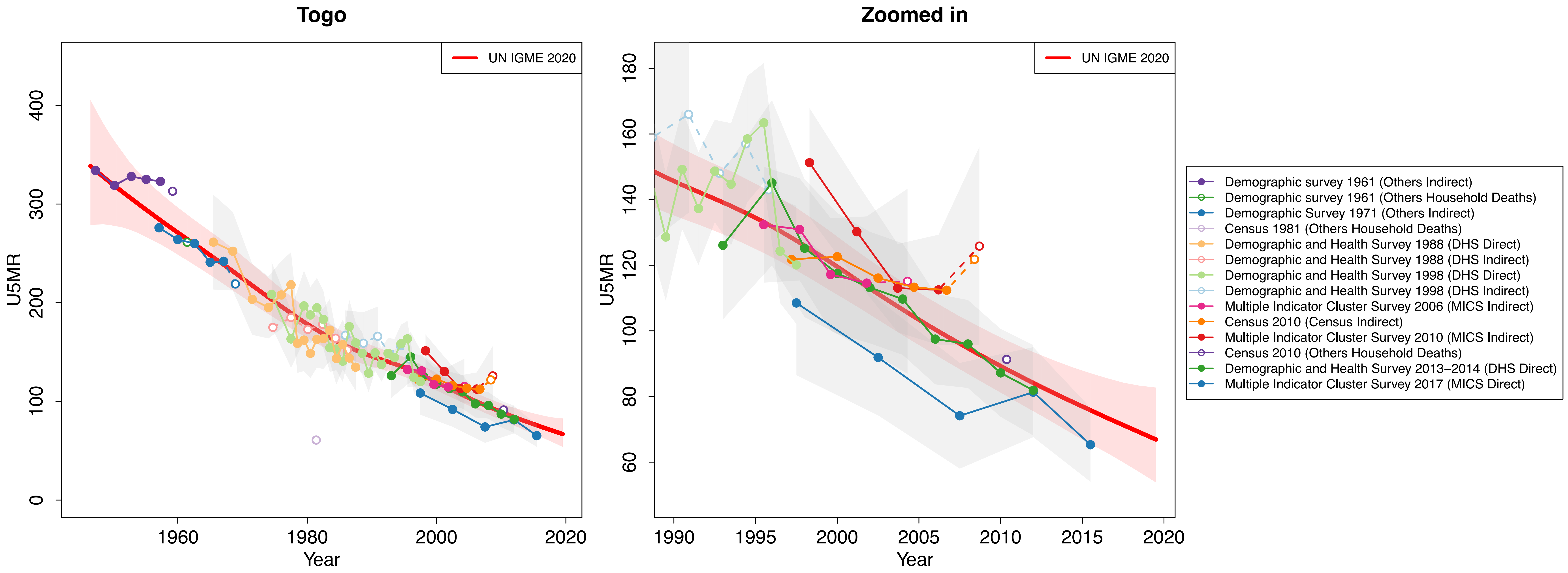
Togo
Zoomed in
UN IGME 2020
U5MR
Year
Demographic survey 1961 (Others Indirect)
Demographic survey 1961 (Others Household Deaths)
Demographic Survey 1971 (Others Indirect)
Census 1981 (Others Household Deaths)
Demographic and Health Survey 1988 (DHS Direct)
Demographic and Health Survey 1988 (DHS Indirect)
Demographic and Health Survey 1998 (DHS Direct)
Demographic and Health Survey 1998 (DHS Indirect)
Multiple Indicator Cluster Survey 2006 (MICS Indirect)
Census 2010 (Census Indirect)
Multiple Indicator Cluster Survey 2010 (MICS Indirect)
Census 2010 (Others Household Deaths)
Demographic and Health Survey 2013–2014 (DHS Direct)
Multiple Indicator Cluster Survey 2017 (MICS Direct)

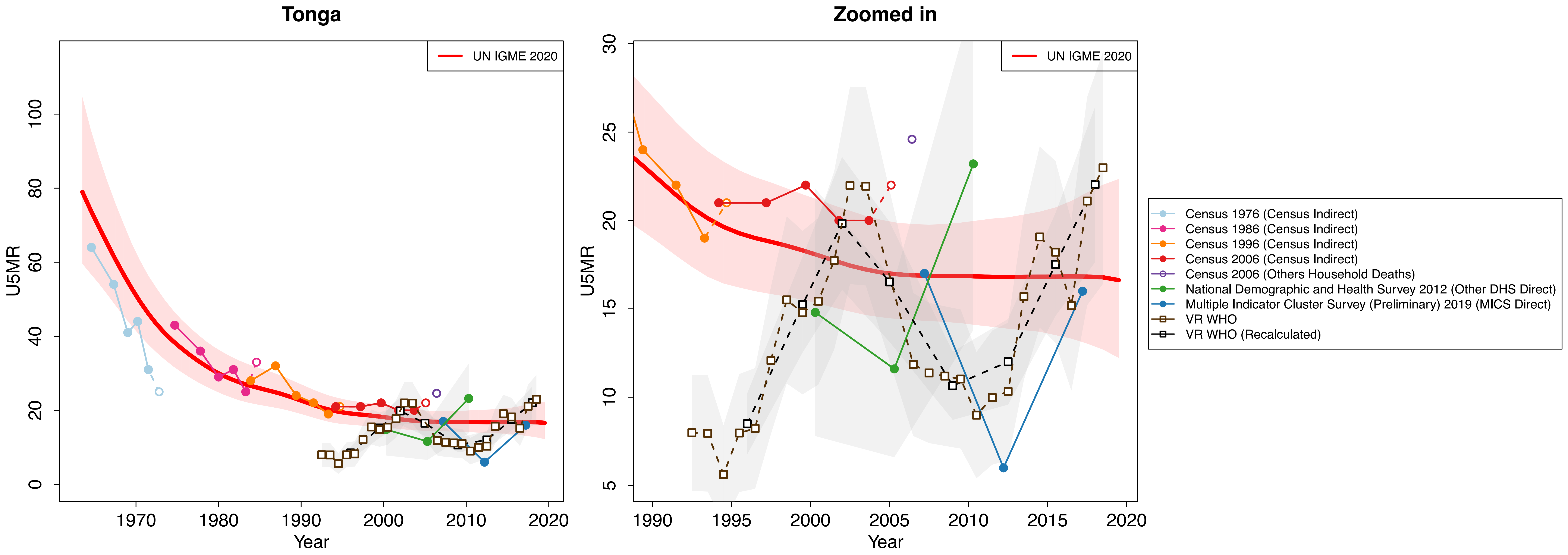

Tonga
Zoomed in
UN IGME 2020
UN IGME 2020
U5MR
Year
Census 1976 (Census Indirect)
Census 1986 (Census Indirect)
Census 1996 (Census Indirect)
Census 2006 (Census Indirect)
Census 2006 (Others Household Deaths)
National Demographic and Health Survey 2012 (Other DHS Direct)
Multiple Indicator Cluster Survey (Preliminary) 2019 (MICS Direct)
VR WHO
VR WHO (Recalculated)

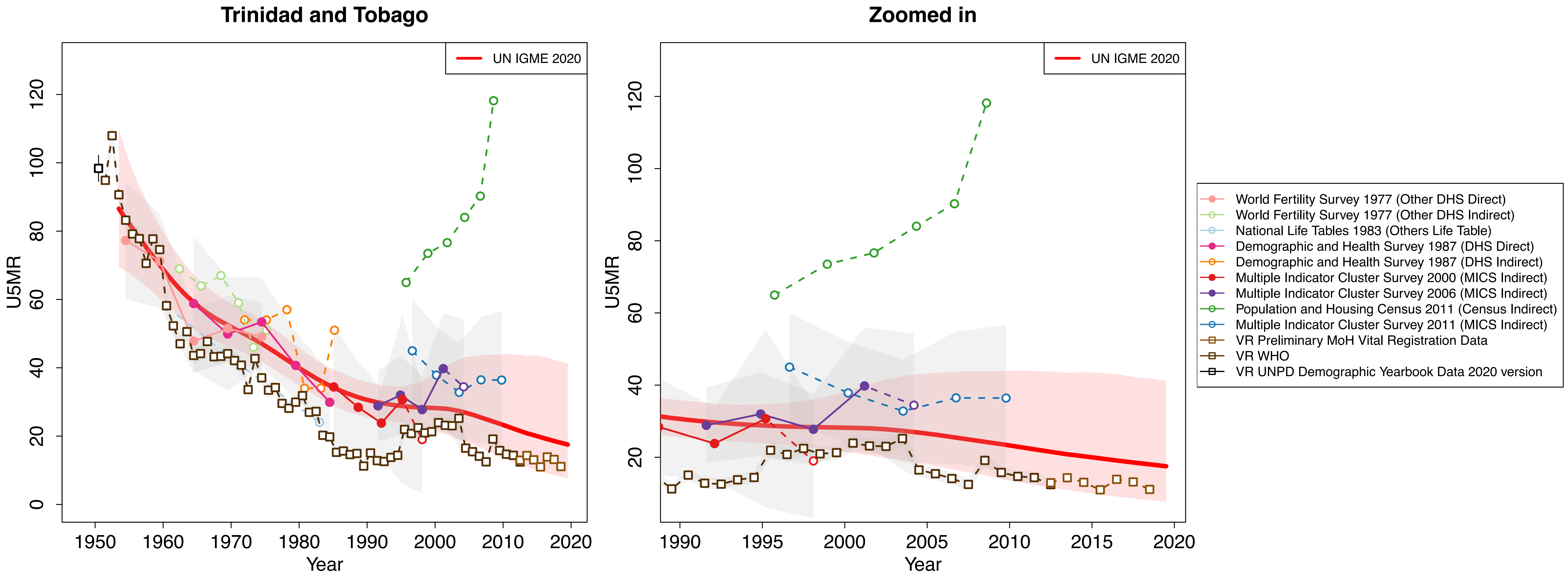
Trinidad and Tobago
Zoomed in
UN IGME 2020
U5MR
Year
World Fertility Survey 1977 (Other DHS Direct)
World Fertility Survey 1977 (Other DHS Indirect)
National Life Tables 1983 (Others Life Table)
Demographic and Health Survey 1987 (DHS Direct)
Demographic and Health Survey 1987 (DHS Indirect)
Multiple Indicator Cluster Survey 2000 (MICS Indirect)
Multiple Indicator Cluster Survey 2006 (MICS Indirect)
Population and Housing Census 2011 (Census Indirect)
Multiple Indicator Cluster Survey 2011 (MICS Indirect)
VR Preliminary MoH Vital Registration Data
VR WHO
VR UNPD Demographic Yearbook Data 2020 version

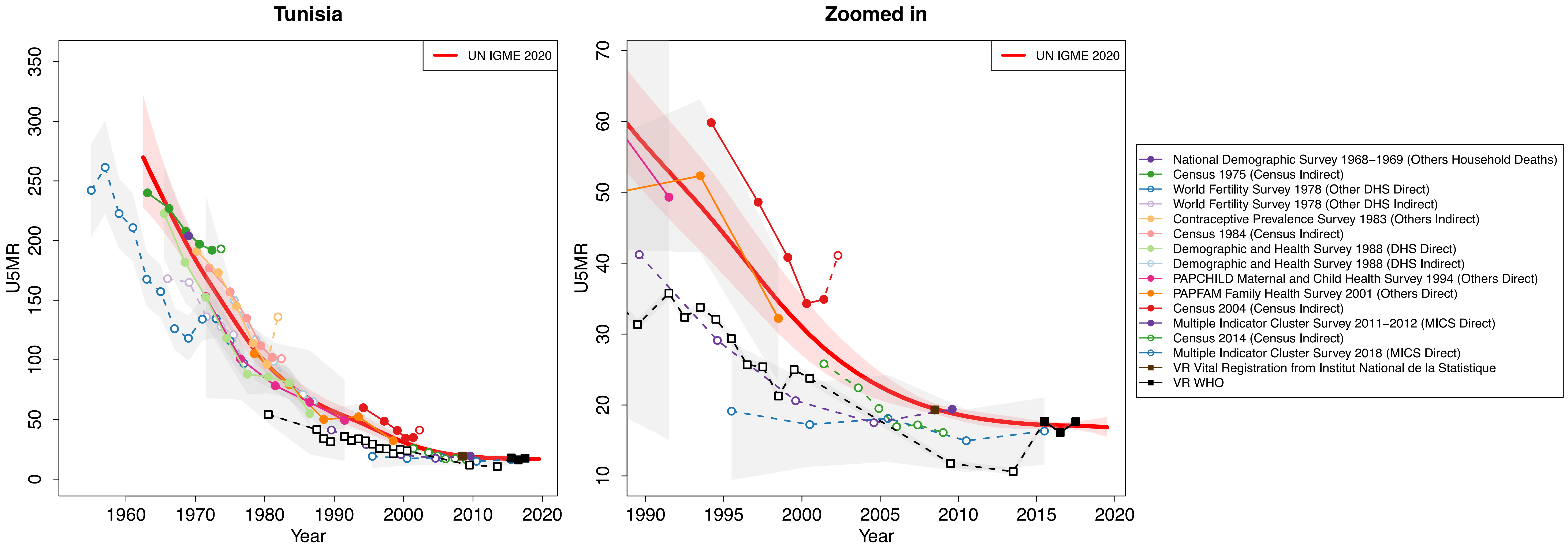

Tunisia
Zoomed in
UN IGME 2020
U5MR
Year
National Demographic Survey 1968–1969 (Others Household Deaths)
Census 1975 (Census Indirect)
World Fertility Survey 1978 (Other DHS Direct)
World Fertility Survey 1978 (Other DHS Indirect)
Contraceptive Prevalence Survey 1983 (Others Indirect)
Census 1984 (Census Indirect)
Demographic and Health Survey 1988 (DHS Direct)
Demographic and Health Survey 1988 (DHS Indirect)
PAPCHILD Maternal and Child Health Survey 1994 (Others Direct)
PAPFAM Family Health Survey 2001 (Others Direct)
Census 2004 (Census Indirect)
Multiple Indicator Cluster Survey 2011–2012 (MICS Direct)
Census 2014 (Census Indirect)
Multiple Indicator Cluster Survey 2018 (MICS Direct)
VR Vital Registration from Institut National de la Statistique
VR WHO

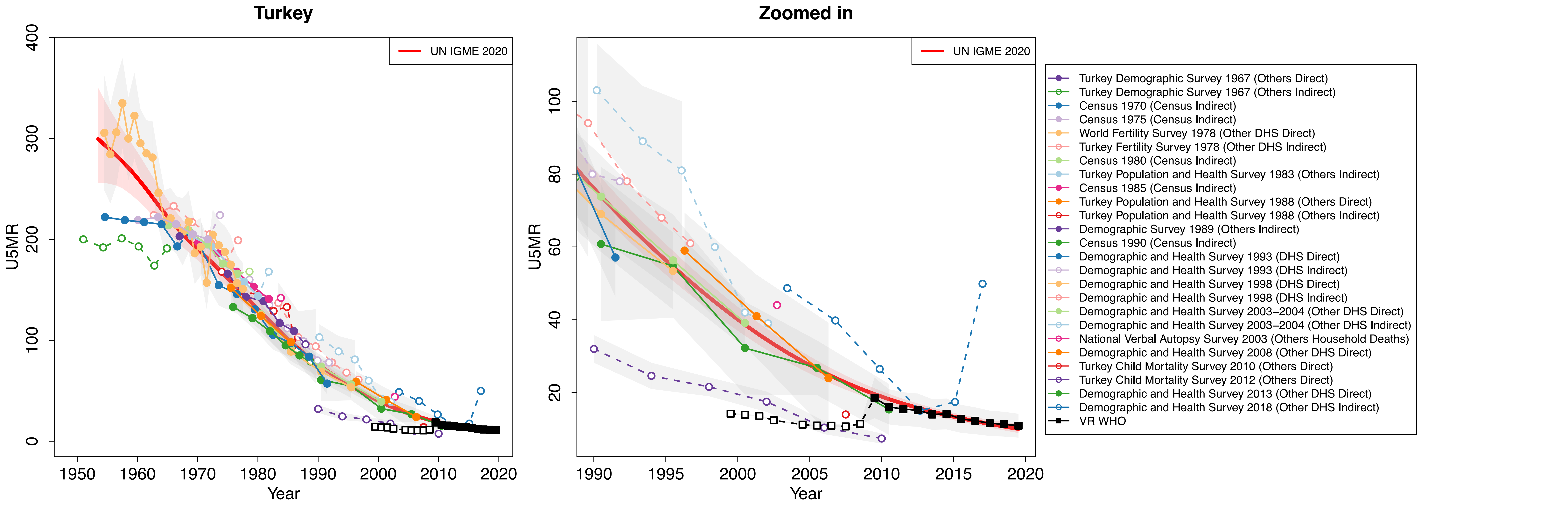
Turkey
Zoomed in
U5MR
Year
UN IGME 2020
Turkey Demographic Survey 1967 (Others Direct)
Turkey Demographic Survey 1967 (Others Indirect)
Census 1970 (Census Indirect)
Census 1975 (Census Indirect)
World Fertility Survey 1978 (Other DHS Direct)
Turkey Fertility Survey 1978 (Other DHS Indirect)
Census 1980 (Census Indirect)
Turkey Population and Health Survey 1983 (Others Indirect)
Census 1985 (Census Indirect)
Turkey Population and Health Survey 1988 (Others Direct)
Turkey Population and Health Survey 1988 (Others Indirect)
Demographic Survey 1989 (Others Indirect)
Census 1990 (Census Indirect)
Demographic and Health Survey 1993 (DHS Direct)
Demographic and Health Survey 1993 (DHS Indirect)
Demographic and Health Survey 1998 (DHS Direct)
Demographic and Health Survey 1998 (DHS Indirect)
Demographic and Health Survey 2003–2004 (Other DHS Direct)
Demographic and Health Survey 2003–2004 (Other DHS Indirect)
National Verbal Autopsy Survey 2003 (Others Household Deaths)
Demographic and Health Survey 2008 (Other DHS Direct)
Turkey Child Mortality Survey 2010 (Others Direct)
Turkey Child Mortality Survey 2012 (Others Direct)
Demographic and Health Survey 2013 (Other DHS Direct)
Demographic and Health Survey 2018 (Other DHS Indirect)
VR WHO

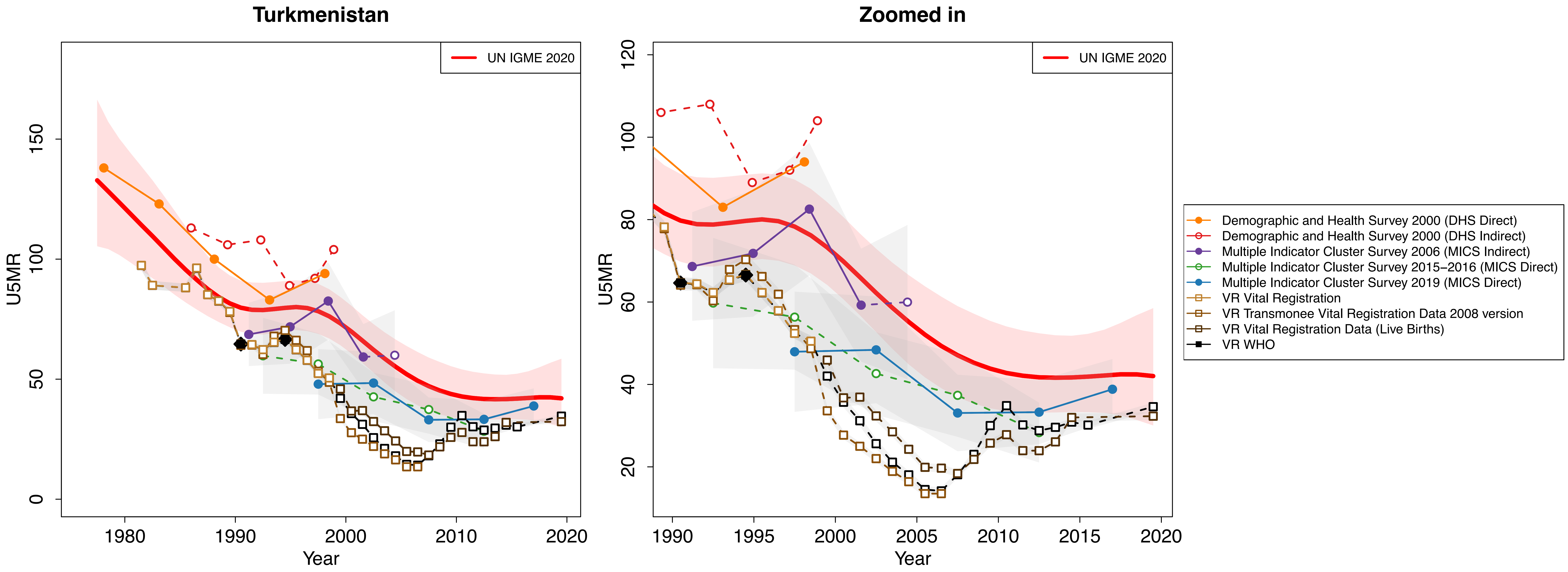

Turkmenistan
Zoomed in
UN IGME 2020
U5MR
Year
0
50
100
150
1980
1990
2000
2010
2020
20
40
60
80
100
120
1995
2005
2015
Demographic and Health Survey 2000 (DHS Direct)
Demographic and Health Survey 2000 (DHS Indirect)
Multiple Indicator Cluster Survey 2006 (MICS Indirect)
Multiple Indicator Cluster Survey 2015–2016 (MICS Direct)
Multiple Indicator Cluster Survey 2019 (MICS Direct)
VR Vital Registration
VR Transmonee Vital Registration Data 2008 version
VR Vital Registration Data (Live Births)
VR WHO

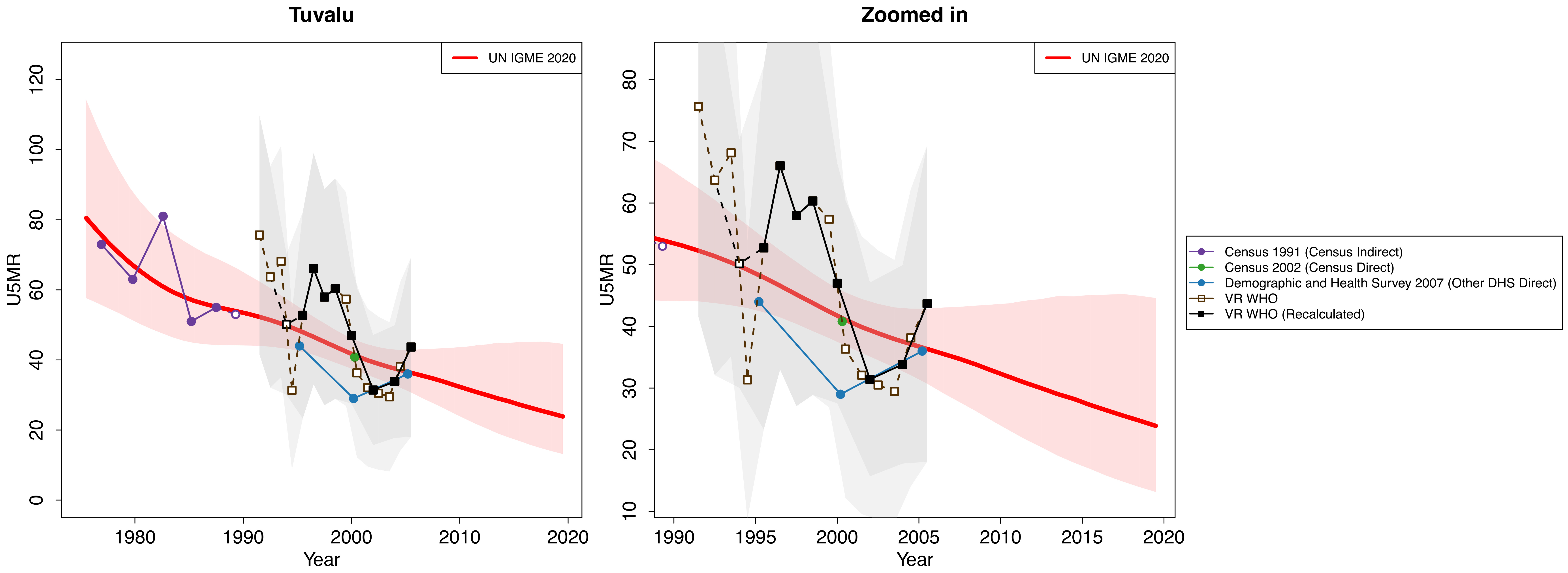

Tuvalu
Zoomed in
UN IGME 2020
U5MR
Year
Census 1991 (Census Indirect)
Census 2002 (Census Direct)
Demographic and Health Survey 2007 (Other DHS Direct)
VR WHO
VR WHO (Recalculated)

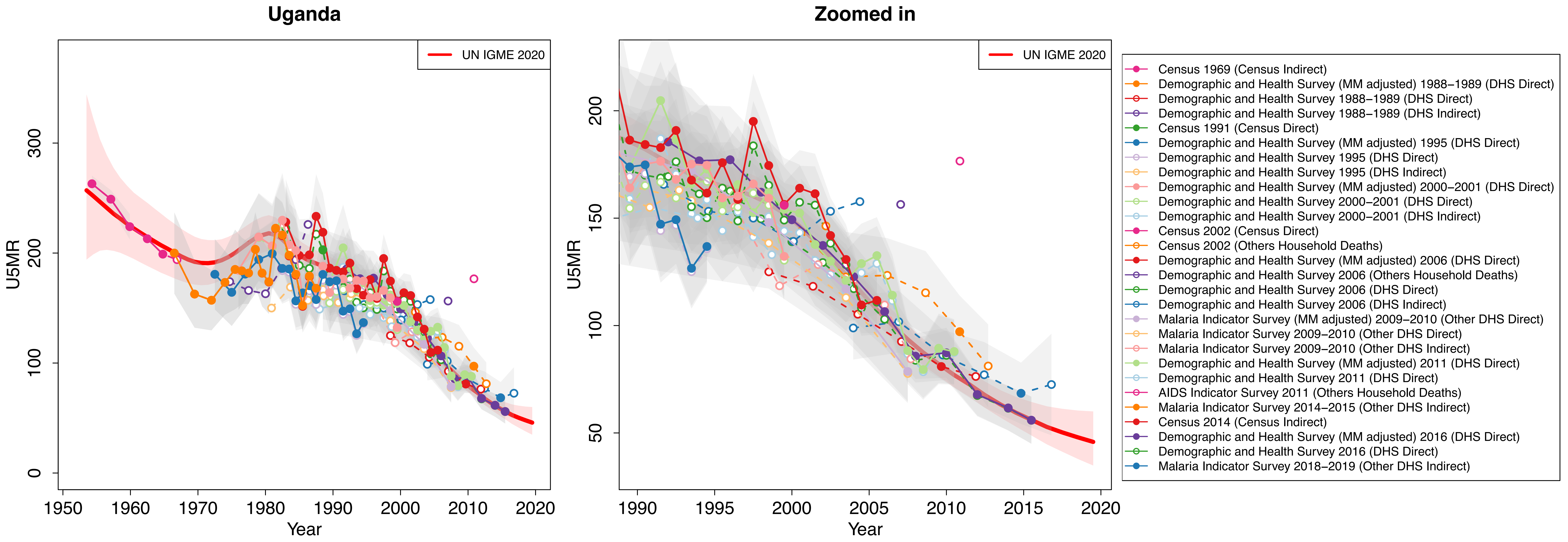

Uganda
Zoomed in
UN IGME 2020
U5MR
Year
Census 1969 (Census Indirect)
Demographic and Health Survey (MM adjusted) 1988–1989 (DHS Direct)
Demographic and Health Survey 1988–1989 (DHS Direct)
Demographic and Health Survey 1988–1989 (DHS Indirect)
Census 1991 (Census Direct)
Demographic and Health Survey (MM adjusted) 1995 (DHS Direct)
Demographic and Health Survey 1995 (DHS Direct)
Demographic and Health Survey 1995 (DHS Indirect)
Demographic and Health Survey (MM adjusted) 2000–2001 (DHS Direct)
Demographic and Health Survey 2000–2001 (DHS Direct)
Demographic and Health Survey 2000–2001 (DHS Indirect)
Census 2002 (Census Direct)
Census 2002 (Others Household Deaths)
Demographic and Health Survey (MM adjusted) 2006 (DHS Direct)
Demographic and Health Survey 2006 (Others Household Deaths)
Demographic and Health Survey 2006 (DHS Direct)
Demographic and Health Survey 2006 (DHS Indirect)
Malaria Indicator Survey (MM adjusted) 2009–2010 (Other DHS Direct)
Malaria Indicator Survey 2009–2010 (Other DHS Direct)
Malaria Indicator Survey 2009–2010 (Other DHS Indirect)
Demographic and Health Survey (MM adjusted) 2011 (DHS Direct)
Demographic and Health Survey 2011 (DHS Direct)
AIDS Indicator Survey 2011 (Others Household Deaths)
Malaria Indicator Survey 2014–2015 (Other DHS Indirect)
Census 2014 (Census Indirect)
Demographic and Health Survey (MM adjusted) 2016 (DHS Direct)
Demographic and Health Survey 2016 (DHS Direct)
Malaria Indicator Survey 2018–2019 (Other DHS Indirect)

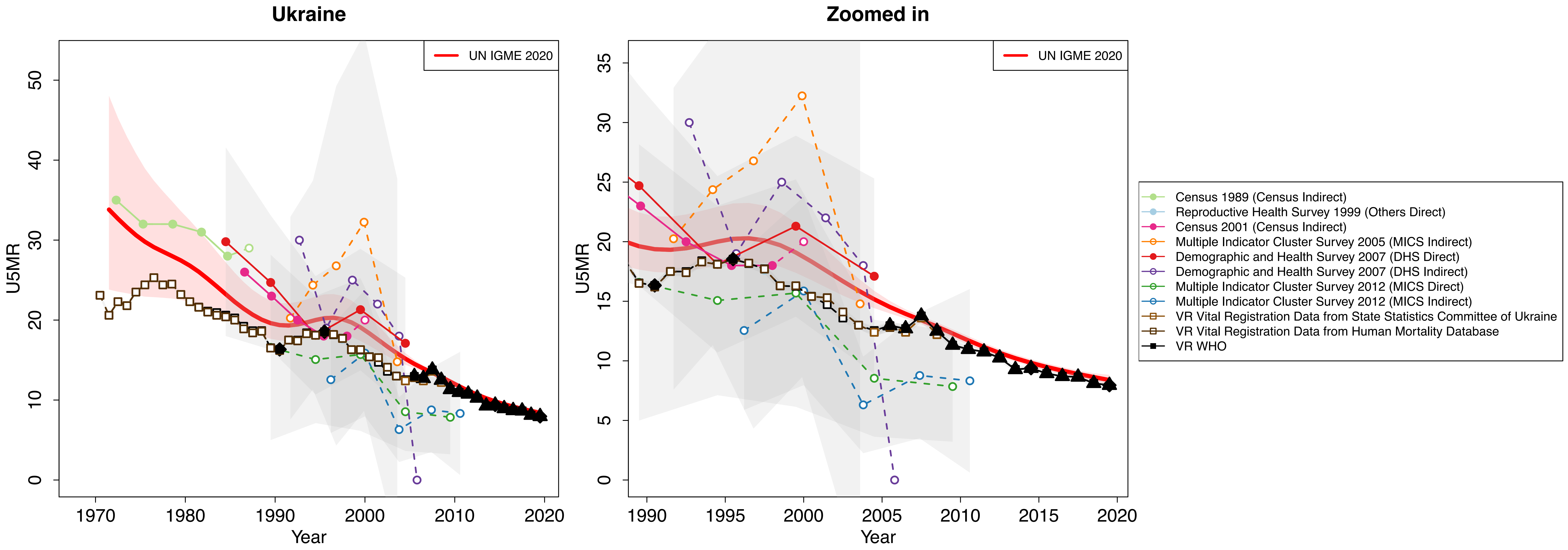

Ukraine
Zoomed in
UN IGME 2020
U5MR
Year
Census 1989 (Census Indirect)
Reproductive Health Survey 1999 (Others Direct)
Census 2001 (Census Indirect)
Multiple Indicator Cluster Survey 2005 (MICS Indirect)
Demographic and Health Survey 2007 (DHS Direct)
Demographic and Health Survey 2007 (DHS Indirect)
Multiple Indicator Cluster Survey 2012 (MICS Direct)
Multiple Indicator Cluster Survey 2012 (MICS Indirect)
VR Vital Registration Data from State Statistics Committee of Ukraine
VR Vital Registration Data from Human Mortality Database
VR WHO

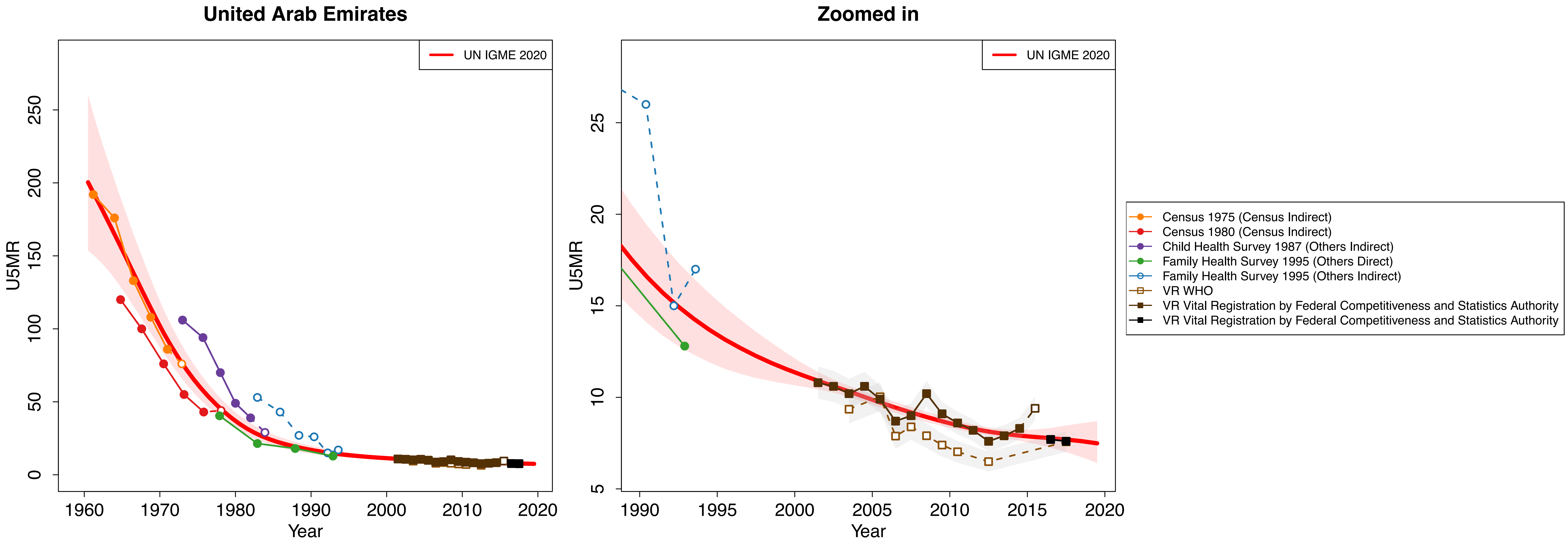
United Arab Emirates
Zoomed in
UN IGME 2020
U5MR
Year
0
50
100
150
200
250
1960
1970
1980
1990
2000
2010
2020
5
10
15
20
25
1995
2005
2015
Census 1975 (Census Indirect)
Census 1980 (Census Indirect)
Child Health Survey 1987 (Others Indirect)
Family Health Survey 1995 (Others Direct)
Family Health Survey 1995 (Others Indirect)
VR WHO
VR Vital Registration by Federal Competitiveness and Statistics Authority
VR Vital Registration by Federal Competitiveness and Statistics Authority

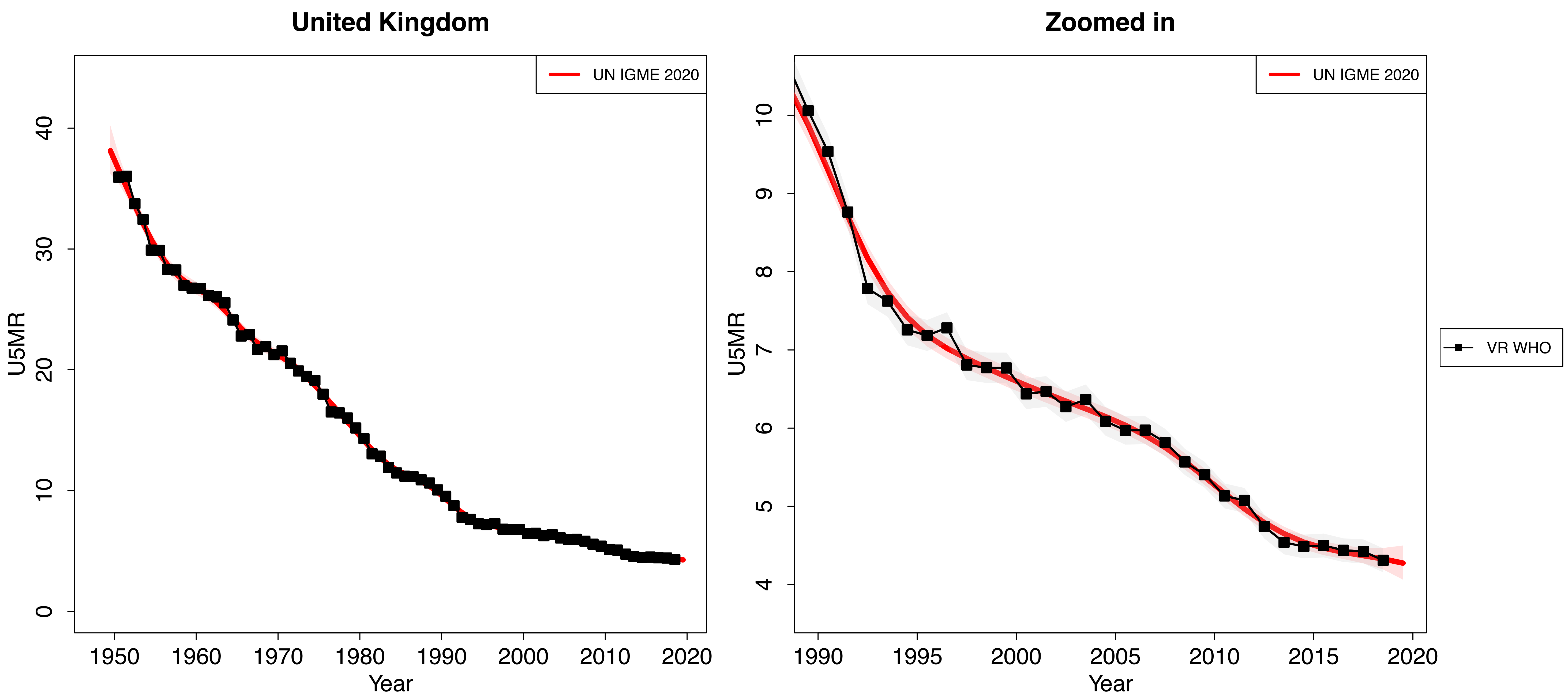

United Kingdom
UN IGME 2020
U5MR
0
10
20
30
40
1950
1960
1970
1980
1990
2000
2010
2020
Year
Zoomed in
UN IGME 2020
U5MR
4
5
6
7
8
9
10
1990
1995
2000
2005
2010
2015
2020
Year
VR WHO

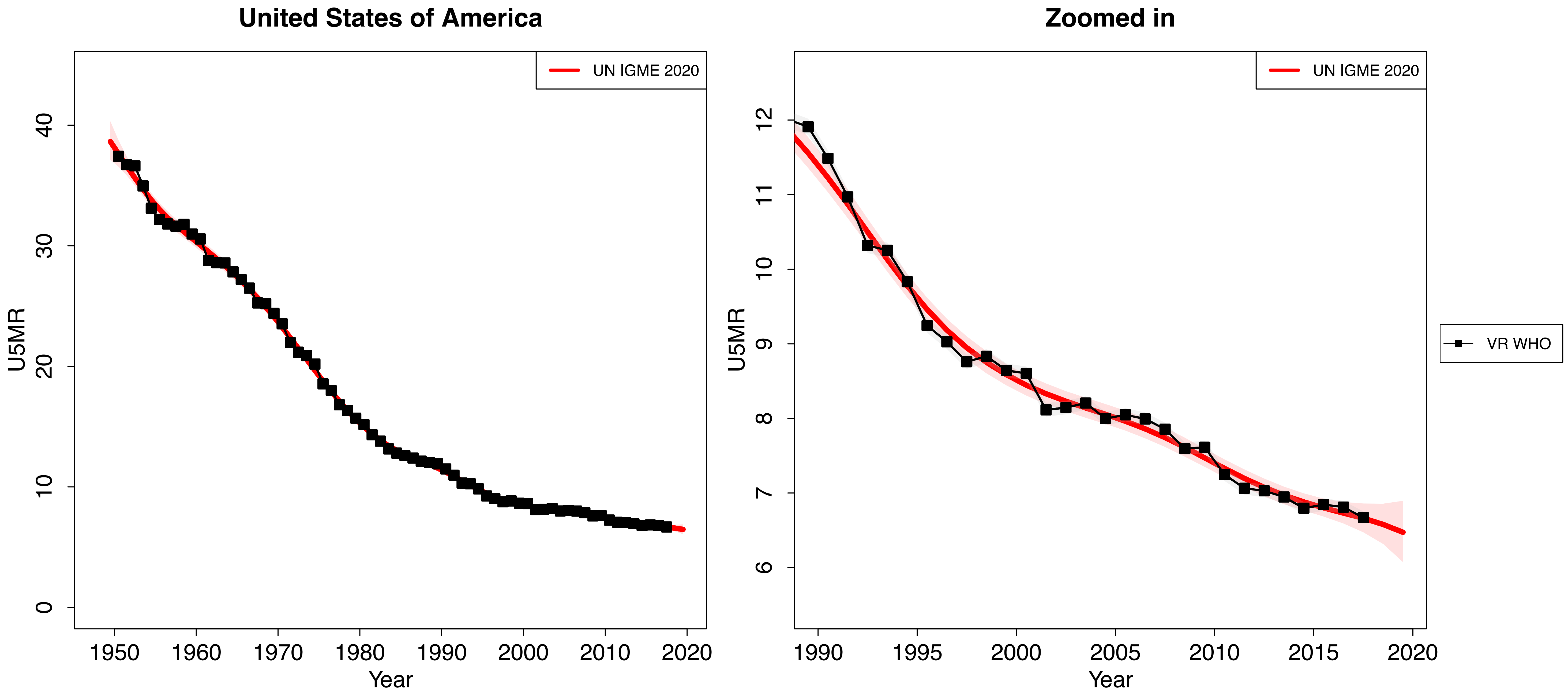
United States of America
Zoomed in
UN IGME 2020
UN IGME 2020
VR WHO
U5MR
U5MR
Year
Year
0
10
20
30
40
6
7
8
9
10
11
12
1950
1960
1970
1980
1990
2000
2010
2020
1990
1995
2000
2005
2010
2015
2020

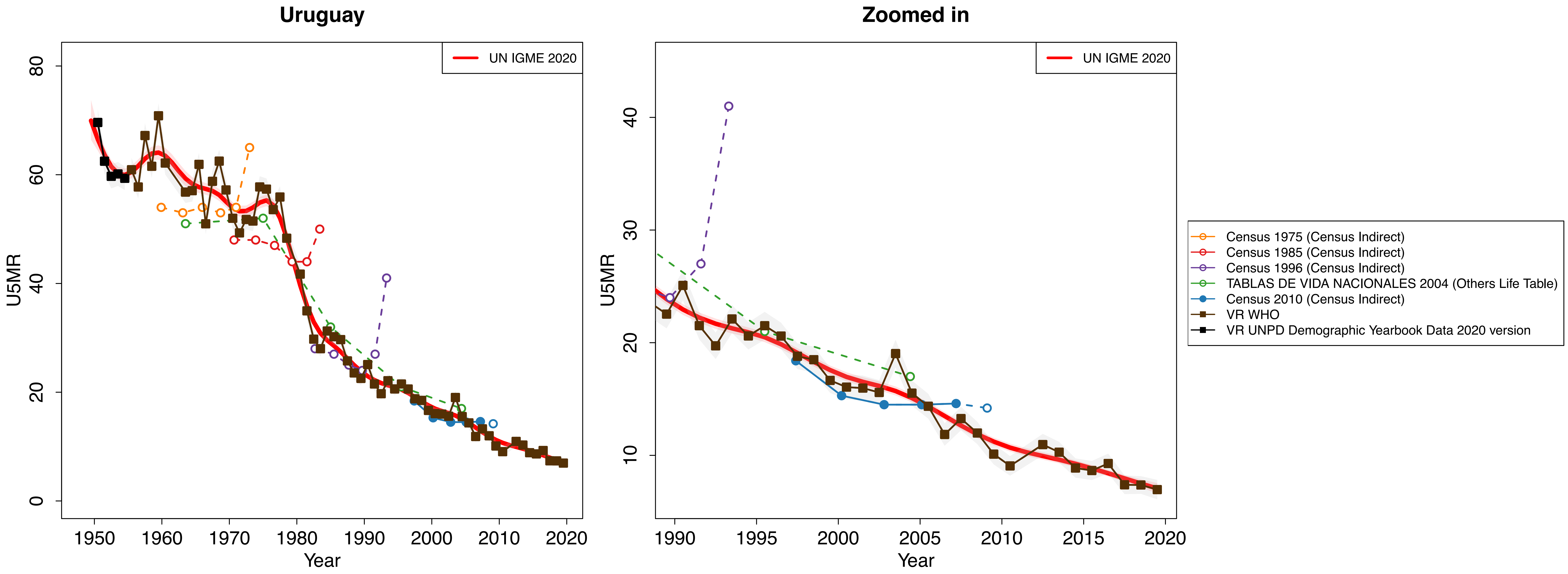

Uruguay
Zoomed in
UN IGME 2020
U5MR
Year
Census 1975 (Census Indirect)
Census 1985 (Census Indirect)
Census 1996 (Census Indirect)
TABLAS DE VIDA NACIONALES 2004 (Others Life Table)
Census 2010 (Census Indirect)
VR WHO
VR UNPD Demographic Yearbook Data 2020 version

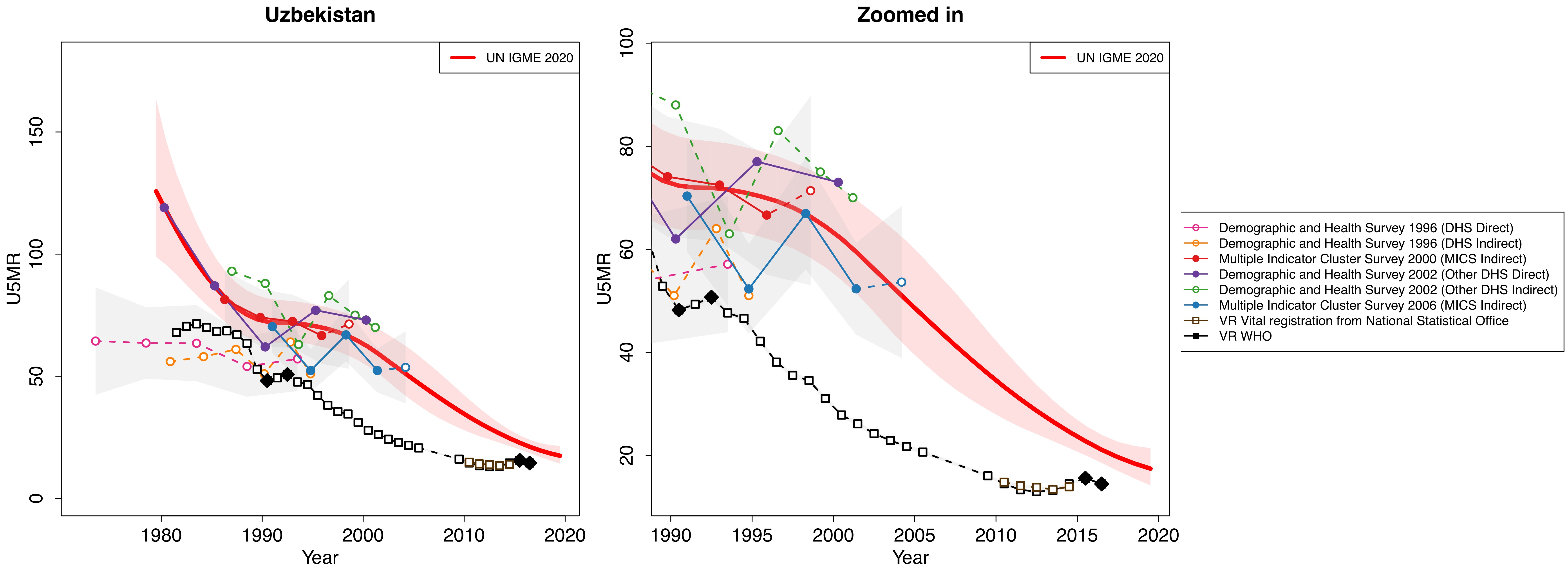

Uzbekistan
Zoomed in
UN IGME 2020
U5MR
Year
Demographic and Health Survey 1996 (DHS Direct)
Demographic and Health Survey 1996 (DHS Indirect)
Multiple Indicator Cluster Survey 2000 (MICS Indirect)
Demographic and Health Survey 2002 (Other DHS Direct)
Demographic and Health Survey 2002 (Other DHS Indirect)
Multiple Indicator Cluster Survey 2006 (MICS Indirect)
VR Vital registration from National Statistical Office
VR WHO

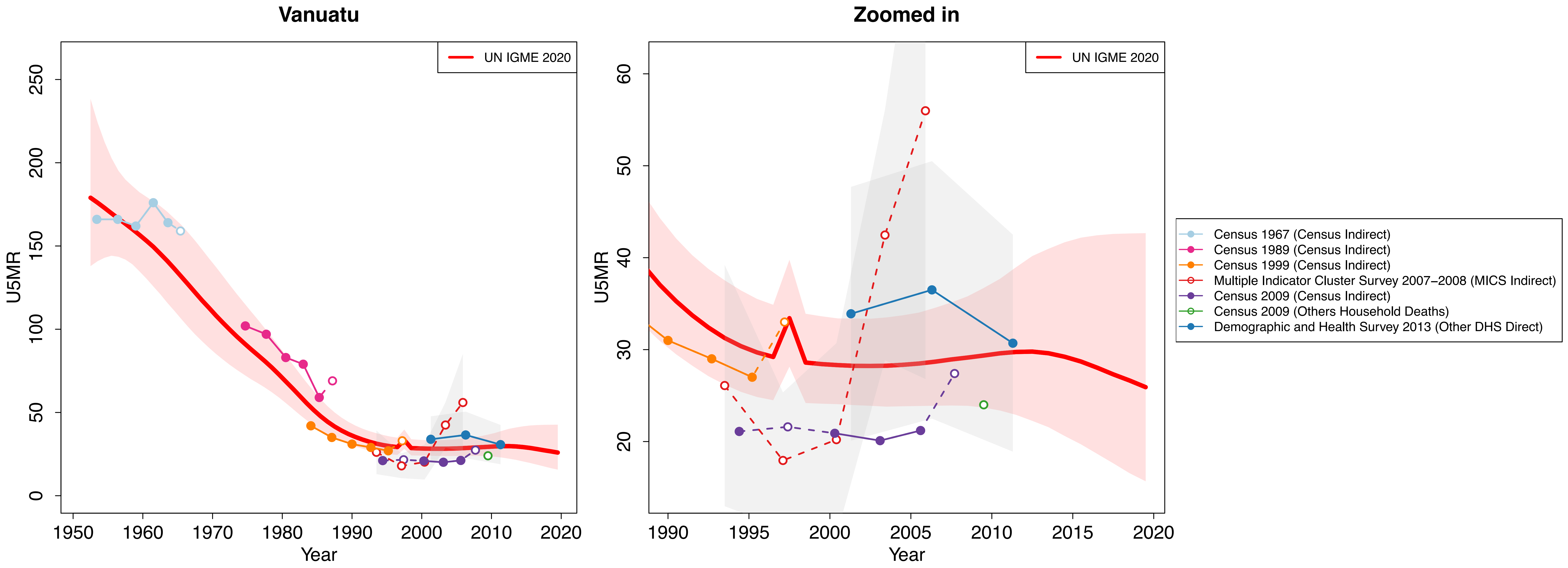

Vanuatu
Zoomed in
UN IGME 2020
U5MR
Year
Census 1967 (Census Indirect)
Census 1989 (Census Indirect)
Census 1999 (Census Indirect)
Multiple Indicator Cluster Survey 2007–2008 (MICS Indirect)
Census 2009 (Census Indirect)
Census 2009 (Others Household Deaths)
Demographic and Health Survey 2013 (Other DHS Direct)

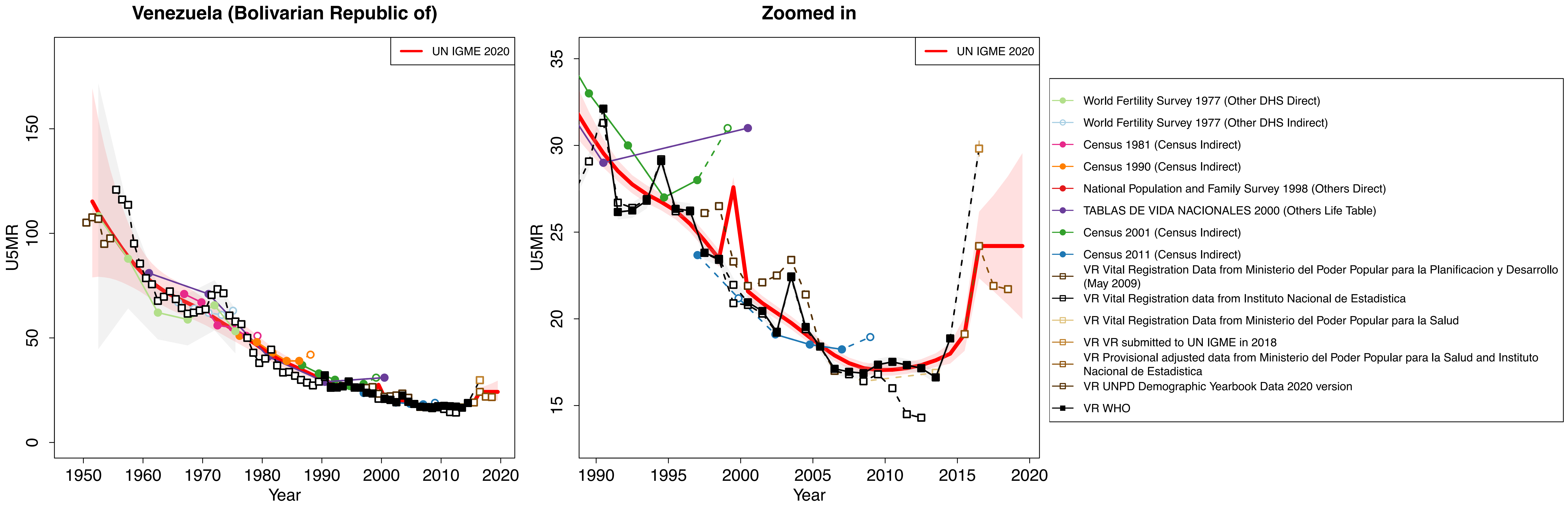

Venezuela (Bolivarian Republic of)
Zoomed in
UN IGME 2020
U5MR
Year
World Fertility Survey 1977 (Other DHS Direct)
World Fertility Survey 1977 (Other DHS Indirect)
Census 1981 (Census Indirect)
Census 1990 (Census Indirect)
National Population and Family Survey 1998 (Others Direct)
TABLAS DE VIDA NACIONALES 2000 (Others Life Table)
Census 2001 (Census Indirect)
Census 2011 (Census Indirect)
VR Vital Registration Data from Ministerio del Poder Popular para la Planificacion y Desarrollo (May 2009)
VR Vital Registration data from Instituto Nacional de Estadistica
VR Vital Registration Data from Ministerio del Poder Popular para la Salud
VR VR submitted to UN IGME in 2018
VR Provisional adjusted data from Ministerio del Poder Popular para la Salud and Instituto Nacional de Estadistica
VR UNPD Demographic Yearbook Data 2020 version
VR WHO

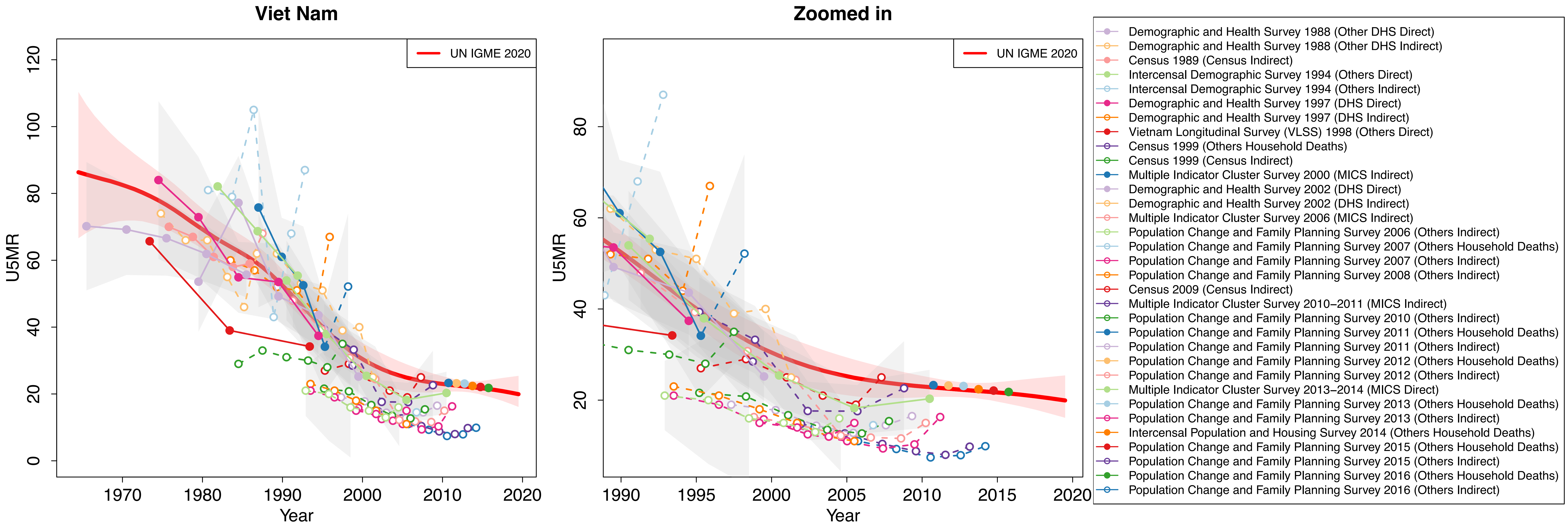

Viet Nam
Zoomed in
U5MR
Year
UN IGME 2020
0
20
40
60
80
100
120
1970
1980
1990
2000
2010
2020
1995
2005
2015
Demographic and Health Survey 1988 (Other DHS Direct)
Demographic and Health Survey 1988 (Other DHS Indirect)
Census 1989 (Census Indirect)
Intercensal Demographic Survey 1994 (Others Direct)
Intercensal Demographic Survey 1994 (Others Indirect)
Demographic and Health Survey 1997 (DHS Direct)
Demographic and Health Survey 1997 (DHS Indirect)
Vietnam Longitudinal Survey (VLSS) 1998 (Others Direct)
Census 1999 (Others Household Deaths)
Census 1999 (Census Indirect)
Multiple Indicator Cluster Survey 2000 (MICS Indirect)
Demographic and Health Survey 2002 (DHS Direct)
Demographic and Health Survey 2002 (DHS Indirect)
Multiple Indicator Cluster Survey 2006 (MICS Indirect)
Population Change and Family Planning Survey 2006 (Others Indirect)
Population Change and Family Planning Survey 2007 (Others Household Deaths)
Population Change and Family Planning Survey 2007 (Others Indirect)
Population Change and Family Planning Survey 2008 (Others Indirect)
Census 2009 (Census Indirect)
Multiple Indicator Cluster Survey 2010–2011 (MICS Indirect)
Population Change and Family Planning Survey 2010 (Others Indirect)
Population Change and Family Planning Survey 2011 (Others Household Deaths)
Population Change and Family Planning Survey 2011 (Others Indirect)
Population Change and Family Planning Survey 2012 (Others Household Deaths)
Population Change and Family Planning Survey 2012 (Others Indirect)
Multiple Indicator Cluster Survey 2013–2014 (MICS Direct)
Population Change and Family Planning Survey 2013 (Others Household Deaths)
Population Change and Family Planning Survey 2013 (Others Indirect)
Intercensal Population and Housing Survey 2014 (Others Household Deaths)
Population Change and Family Planning Survey 2015 (Others Household Deaths)
Population Change and Family Planning Survey 2015 (Others Indirect)
Population Change and Family Planning Survey 2016 (Others Household Deaths)
Population Change and Family Planning Survey 2016 (Others Indirect)

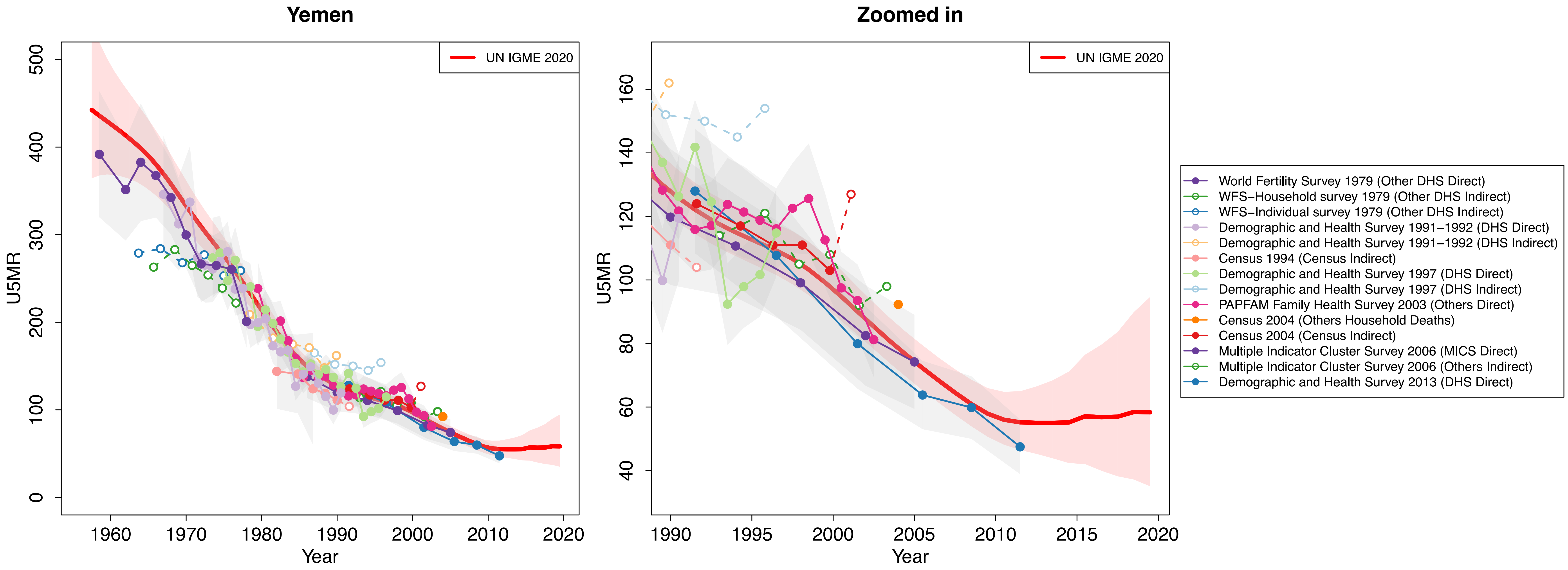

Yemen
Zoomed in
UN IGME 2020
U5MR
Year
World Fertility Survey 1979 (Other DHS Direct)
WFS−Household survey 1979 (Other DHS Indirect)
WFS−Individual survey 1979 (Other DHS Indirect)
Demographic and Health Survey 1991−1992 (DHS Direct)
Demographic and Health Survey 1991−1992 (DHS Indirect)
Census 1994 (Census Indirect)
Demographic and Health Survey 1997 (DHS Direct)
Demographic and Health Survey 1997 (DHS Indirect)
PAPFAM Family Health Survey 2003 (Others Direct)
Census 2004 (Others Household Deaths)
Census 2004 (Census Indirect)
Multiple Indicator Cluster Survey 2006 (MICS Direct)
Multiple Indicator Cluster Survey 2006 (Others Indirect)
Demographic and Health Survey 2013 (DHS Direct)

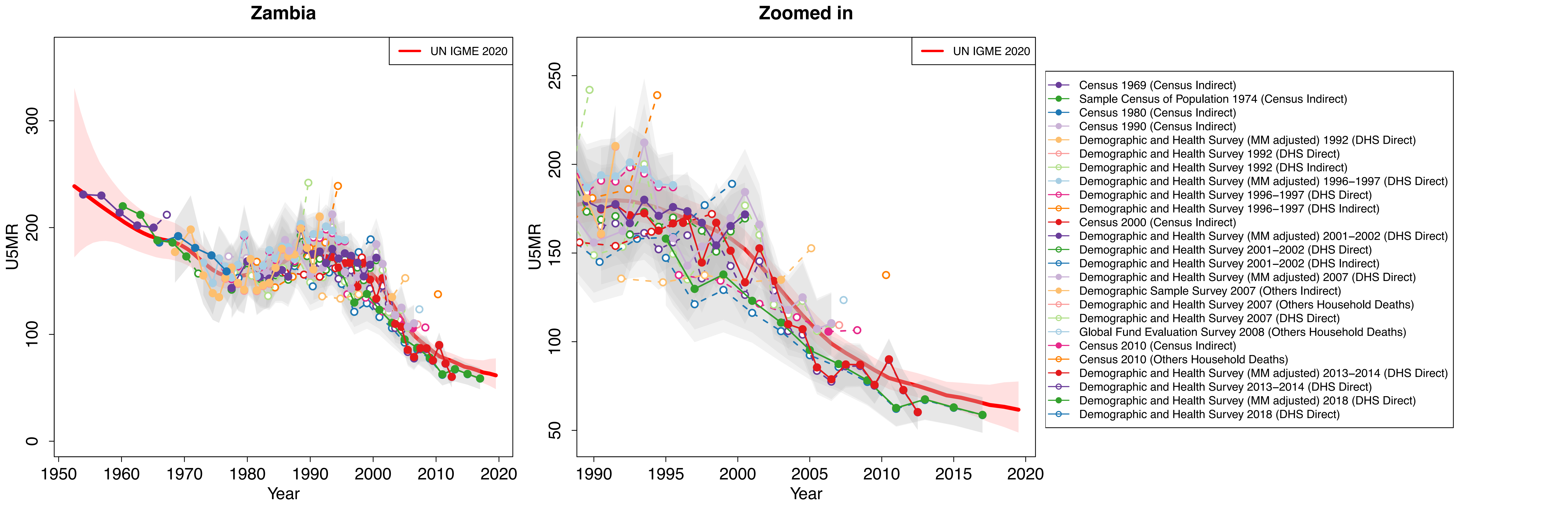

Zambia
Zoomed in
UN IGME 2020
U5MR
Year
Census 1969 (Census Indirect)
Sample Census of Population 1974 (Census Indirect)
Census 1980 (Census Indirect)
Census 1990 (Census Indirect)
Demographic and Health Survey (MM adjusted) 1992 (DHS Direct)
Demographic and Health Survey 1992 (DHS Direct)
Demographic and Health Survey 1992 (DHS Indirect)
Demographic and Health Survey (MM adjusted) 1996–1997 (DHS Direct)
Demographic and Health Survey 1996–1997 (DHS Direct)
Demographic and Health Survey 1996–1997 (DHS Indirect)
Census 2000 (Census Indirect)
Demographic and Health Survey (MM adjusted) 2001–2002 (DHS Direct)
Demographic and Health Survey 2001–2002 (DHS Direct)
Demographic and Health Survey 2001–2002 (DHS Indirect)
Demographic and Health Survey (MM adjusted) 2007 (DHS Direct)
Demographic Sample Survey 2007 (Others Indirect)
Demographic and Health Survey 2007 (Others Household Deaths)
Demographic and Health Survey 2007 (DHS Direct)
Global Fund Evaluation Survey 2008 (Others Household Deaths)
Census 2010 (Census Indirect)
Census 2010 (Others Household Deaths)
Demographic and Health Survey (MM adjusted) 2013–2014 (DHS Direct)
Demographic and Health Survey 2013–2014 (DHS Direct)
Demographic and Health Survey (MM adjusted) 2018 (DHS Direct)
Demographic and Health Survey 2018 (DHS Direct)

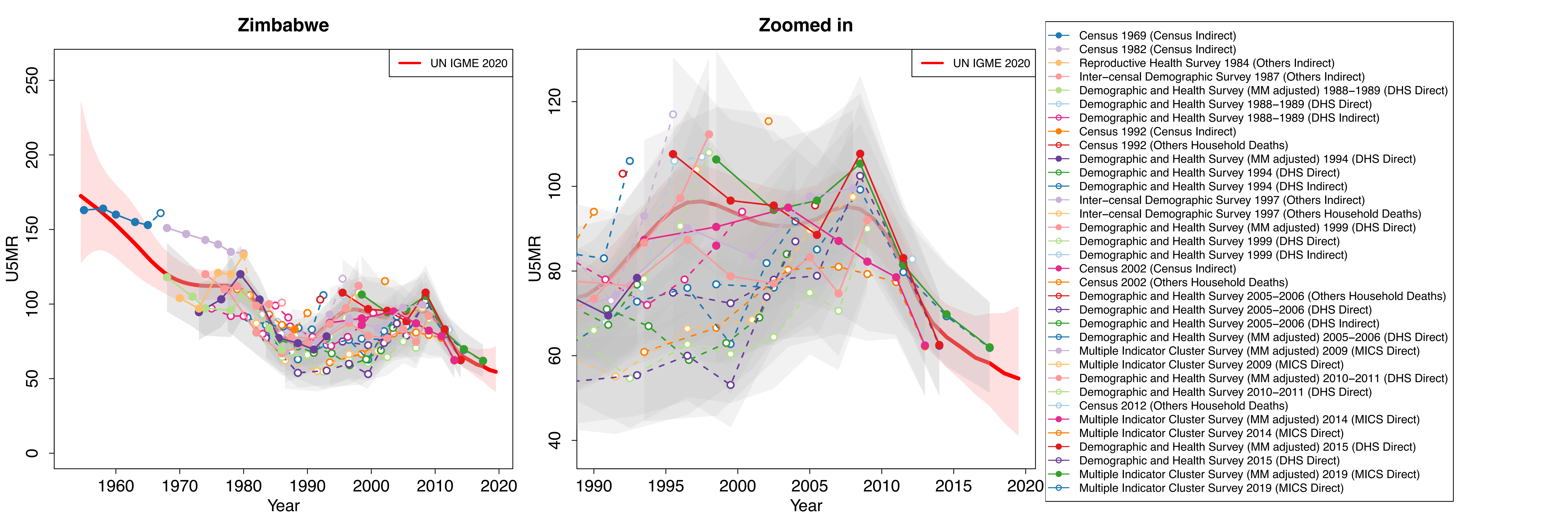
Zimbabwe
Zoomed in
UN IGME 2020
UN IGME 2020
U5MR
U5MR
Year
Year
Census 1969 (Census Indirect)
Census 1982 (Census Indirect)
Reproductive Health Survey 1984 (Others Indirect)
Inter−censal Demographic Survey 1987 (Others Indirect)
Demographic and Health Survey (MM adjusted) 1988−1989 (DHS Direct)
Demographic and Health Survey 1988−1989 (DHS Direct)
Demographic and Health Survey 1988−1989 (DHS Indirect)
Census 1992 (Census Indirect)
Census 1992 (Others Household Deaths)
Demographic and Health Survey (MM adjusted) 1994 (DHS Direct)
Demographic and Health Survey 1994 (DHS Direct)
Demographic and Health Survey 1994 (DHS Indirect)
Inter−censal Demographic Survey 1997 (Others Indirect)
Inter−censal Demographic Survey 1997 (Others Household Deaths)
Demographic and Health Survey (MM adjusted) 1999 (DHS Direct)
Demographic and Health Survey 1999 (DHS Direct)
Demographic and Health Survey 1999 (DHS Indirect)
Census 2002 (Census Indirect)
Census 2002 (Others Household Deaths)
Demographic and Health Survey 2005−2006 (Others Household Deaths)
Demographic and Health Survey 2005−2006 (DHS Direct)
Demographic and Health Survey 2005−2006 (DHS Indirect)
Demographic and Health Survey (MM adjusted) 2005−2006 (DHS Direct)
Multiple Indicator Cluster Survey (MM adjusted) 2009 (MICS Direct)
Multiple Indicator Cluster Survey 2009 (MICS Direct)
Demographic and Health Survey (MM adjusted) 2010−2011 (DHS Direct)
Demographic and Health Survey 2010−2011 (DHS Direct)
Census 2012 (Others Household Deaths)
Multiple Indicator Cluster Survey (MM adjusted) 2014 (MICS Direct)
Multiple Indicator Cluster Survey 2014 (MICS Direct)
Demographic and Health Survey (MM adjusted) 2015 (DHS Direct)
Demographic and Health Survey 2015 (DHS Direct)
Multiple Indicator Cluster Survey (MM adjusted) 2019 (MICS Direct)
Multiple Indicator Cluster Survey 2019 (MICS Direct)

4.5. Figure A.5 Neonatal mortality estimates with underlying data, by country

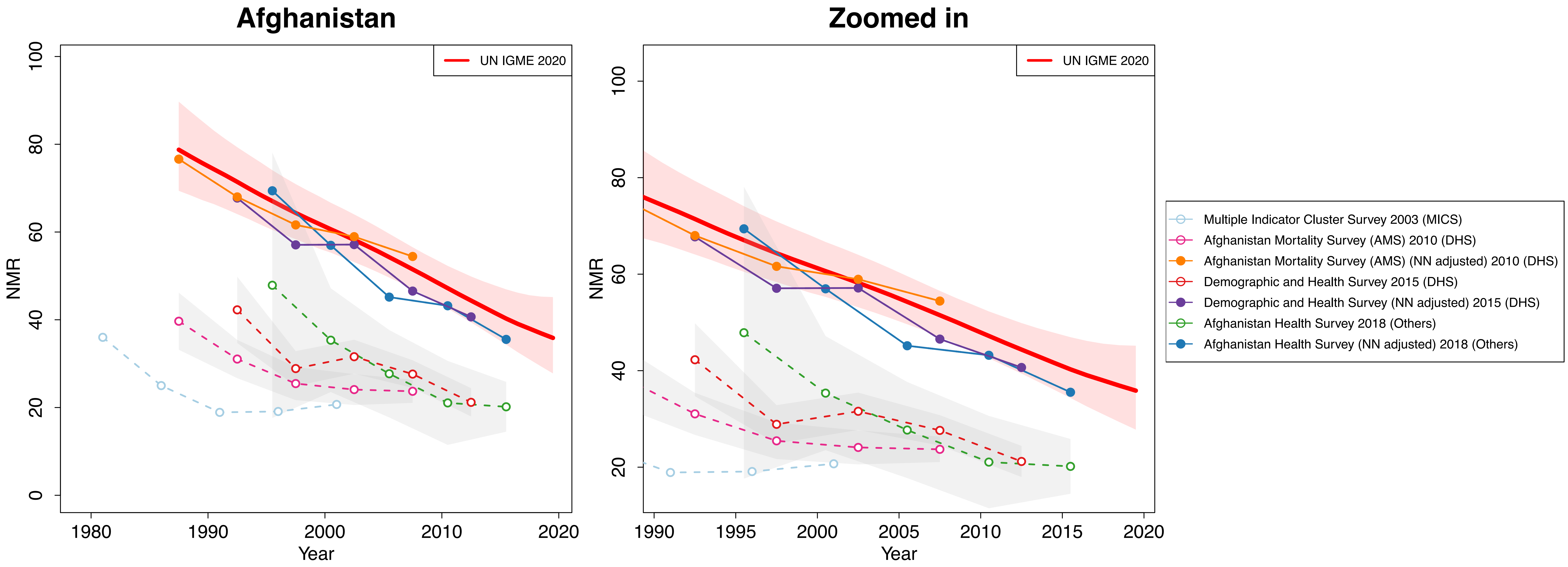

Afghanistan
Zoomed in
UN IGME 2020
NMR
Year
Multiple Indicator Cluster Survey 2003 (MICS)
Afghanistan Mortality Survey (AMS) 2010 (DHS)
Afghanistan Mortality Survey (AMS) (NN adjusted) 2010 (DHS)
Demographic and Health Survey 2015 (DHS)
Demographic and Health Survey (NN adjusted) 2015 (DHS)
Afghanistan Health Survey 2018 (Others)
Afghanistan Health Survey (NN adjusted) 2018 (Others)

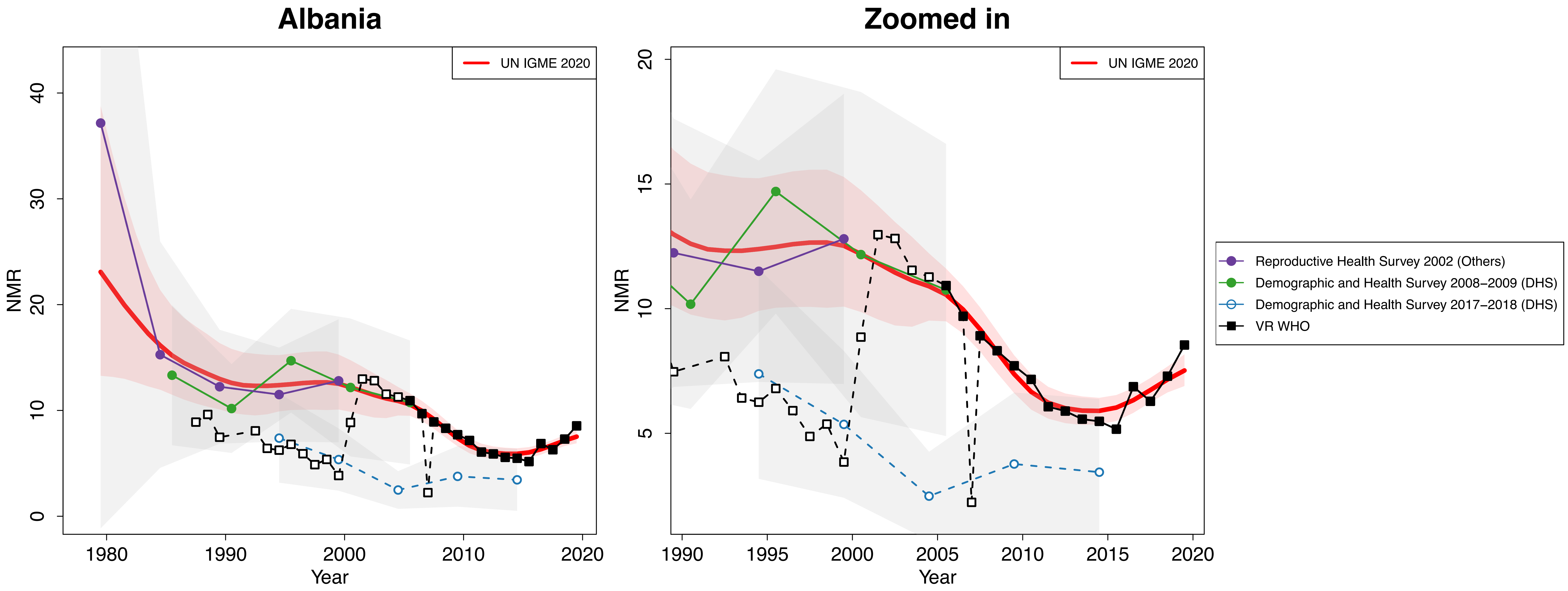
Albania
Zoomed in
UN IGME 2020
NMR
Year
Reproductive Health Survey 2002 (Others)
Demographic and Health Survey 2008–2009 (DHS)
Demographic and Health Survey 2017–2018 (DHS)
VR WHO

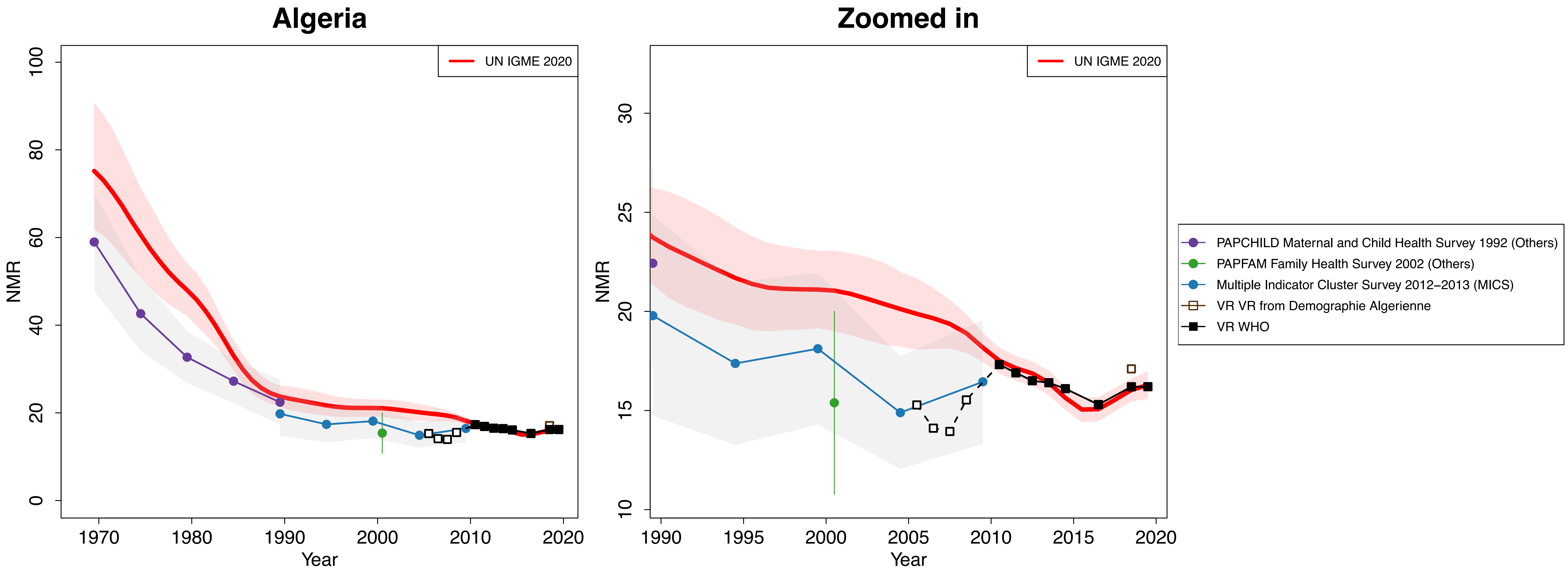

Algeria
Zoomed in
UN IGME 2020
UN IGME 2020
NMR
NMR
Year
Year
0
20
40
60
80
100
10
15
20
25
30
1970
1980
1990
2000
2010
2020
1990
1995
2000
2005
2010
2015
2020
PAPCHILD Maternal and Child Health Survey 1992 (Others)
PAPFAM Family Health Survey 2002 (Others)
Multiple Indicator Cluster Survey 2012–2013 (MICS)
VR VR from Demographie Algerienne
VR WHO

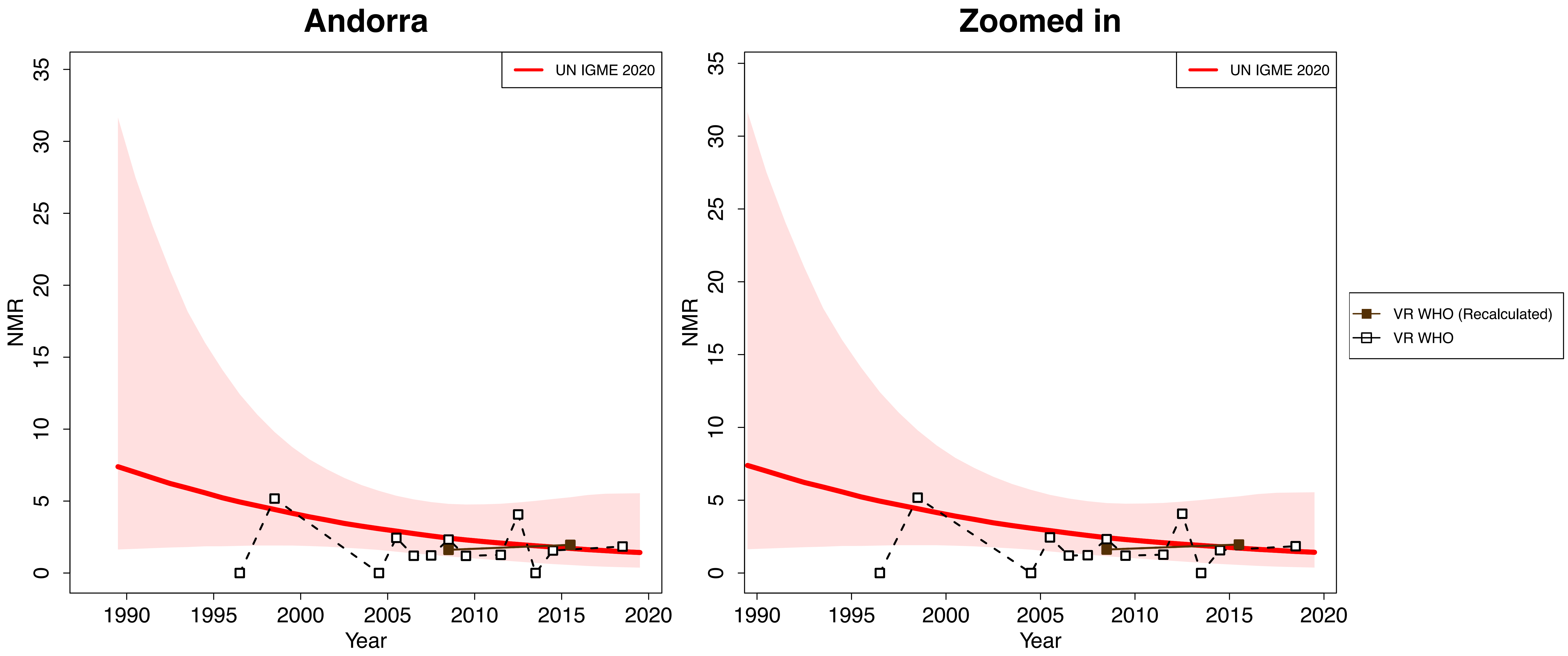

Andorra
Zoomed in
UN IGME 2020
NMR
Year
0
5
10
15
20
25
30
35
1990
1995
2000
2005
2010
2015
2020
VR WHO (Recalculated)
VR WHO

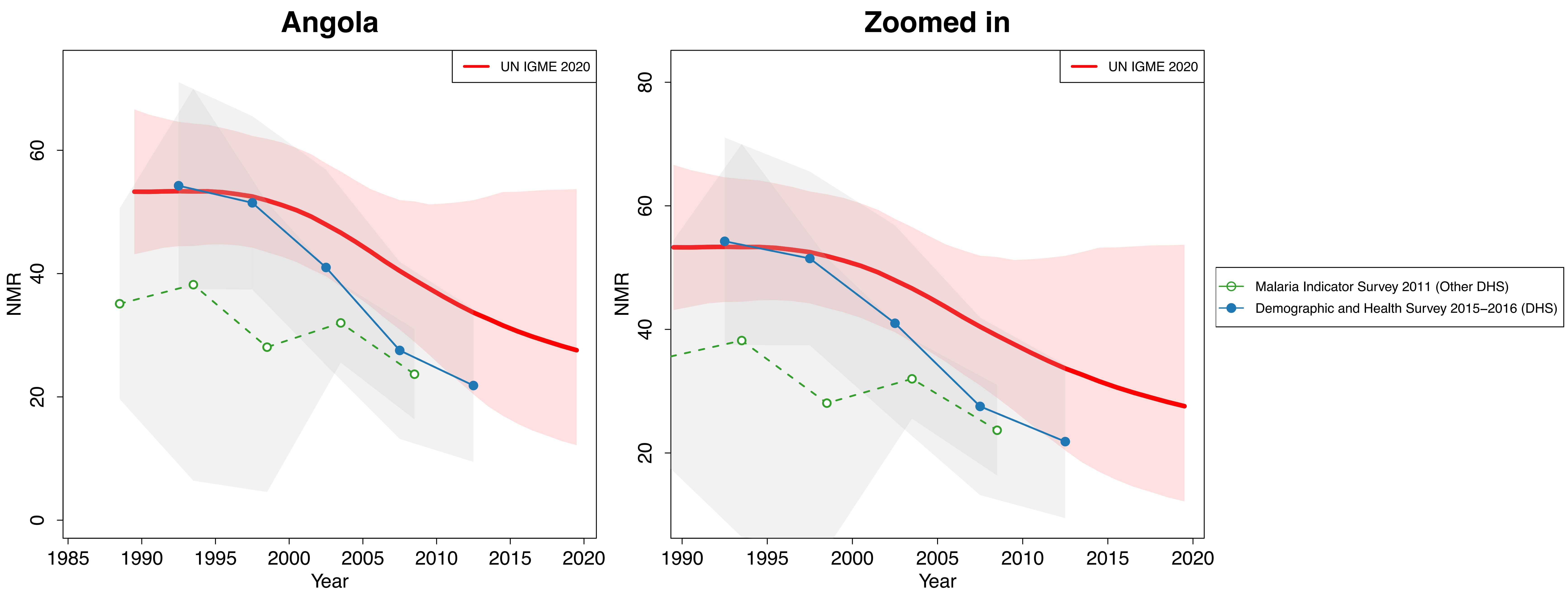

Angola
Zoomed in
UN IGME 2020
UN IGME 2020
NMR
NMR
Year
Year
0
20
40
60
80
1985
1990
1995
2000
2005
2010
2015
2020
Malaria Indicator Survey 2011 (Other DHS)
Demographic and Health Survey 2015–2016 (DHS)

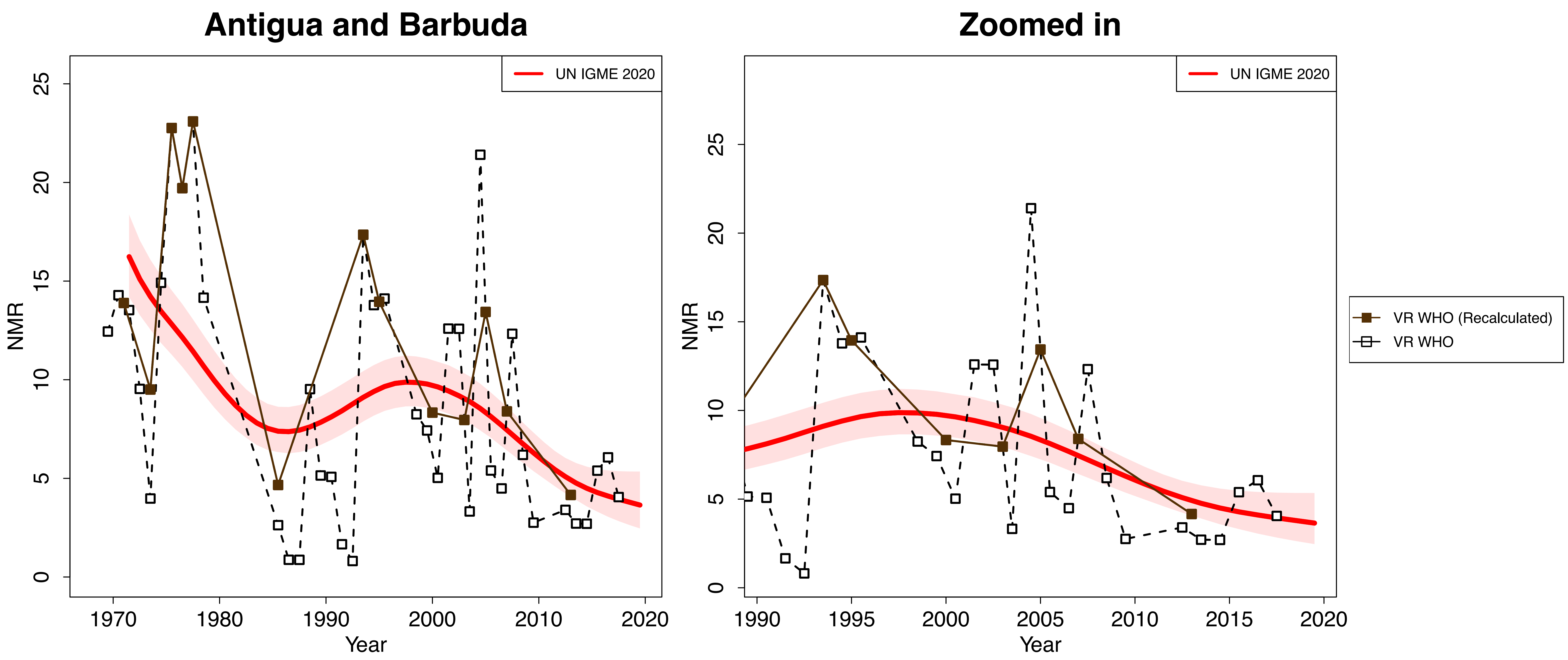

Antigua and Barbuda
Zoomed in
UN IGME 2020
NMR
Year
VR WHO (Recalculated)
VR WHO

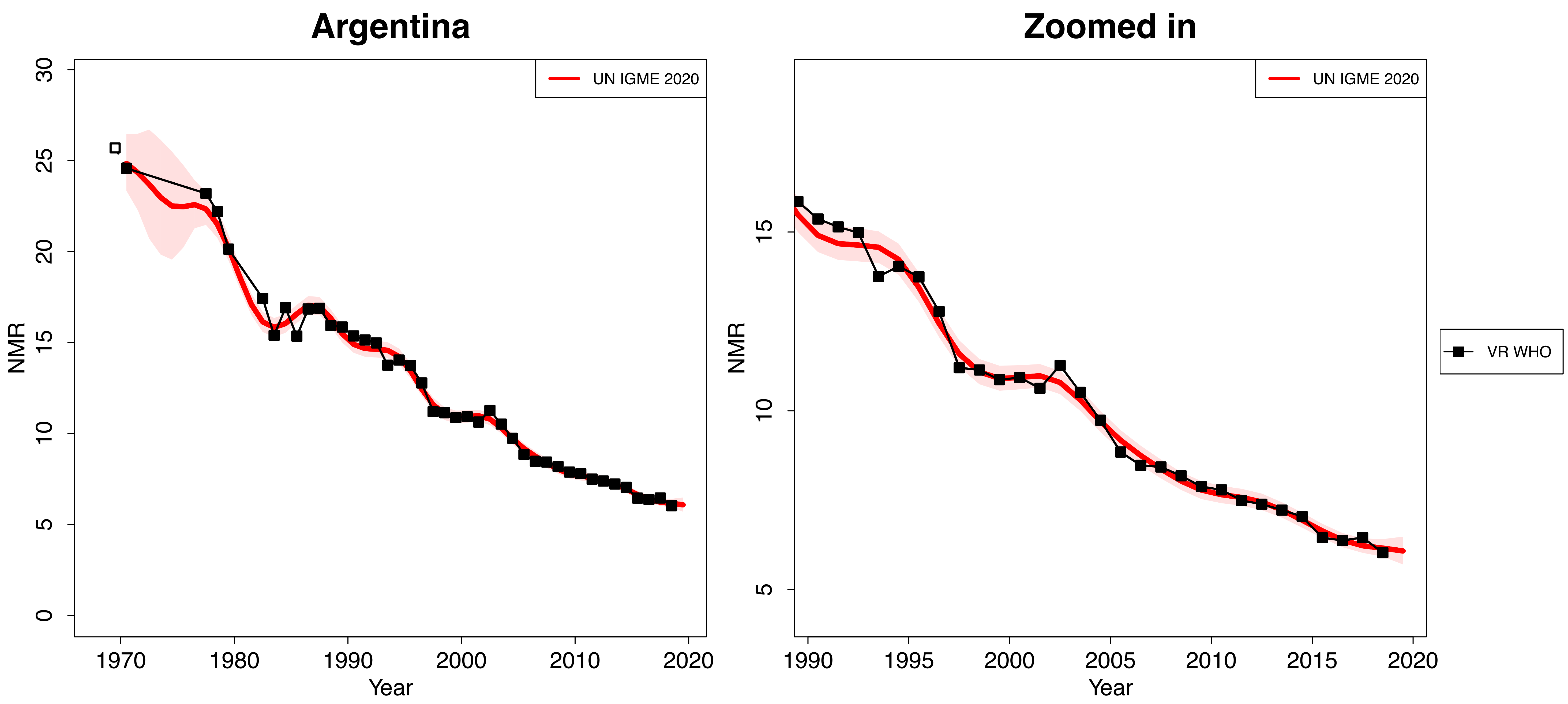

Argentina
Zoomed in
UN IGME 2020
VR WHO
NMR
Year

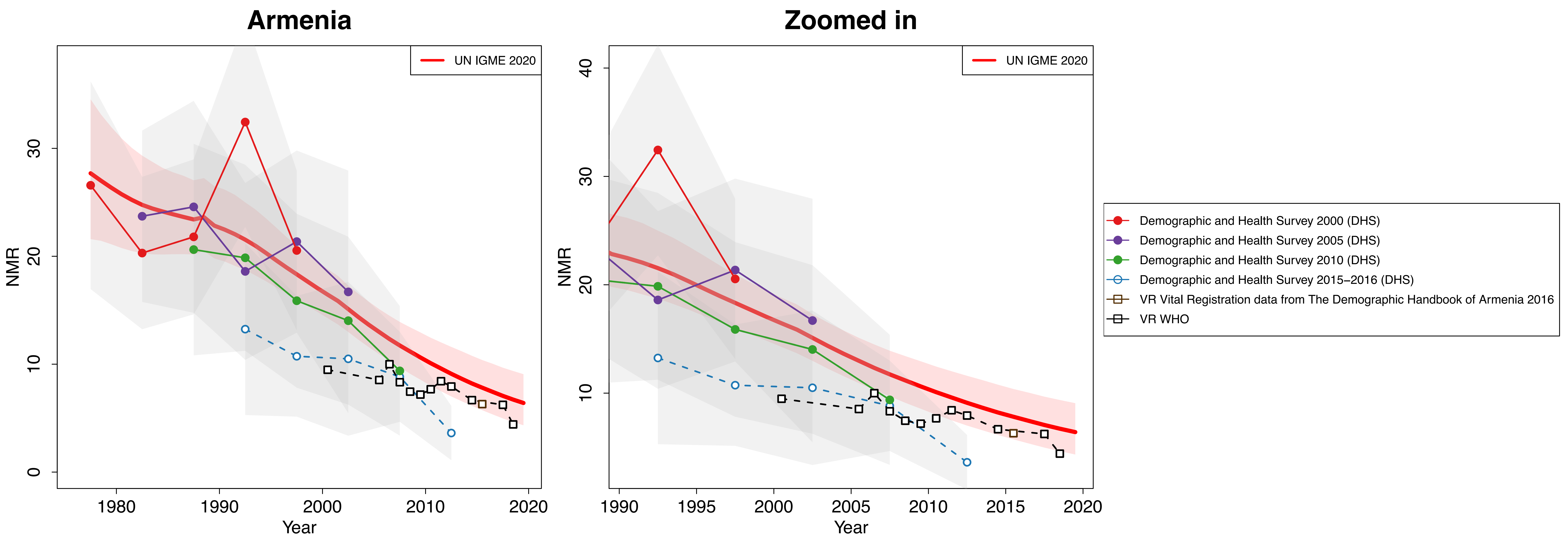
Armenia
Zoomed in
UN IGME 2020
NMR
Year
Demographic and Health Survey 2000 (DHS)
Demographic and Health Survey 2005 (DHS)
Demographic and Health Survey 2010 (DHS)
Demographic and Health Survey 2015–2016 (DHS)
VR Vital Registration data from The Demographic Handbook of Armenia 2016
VR WHO

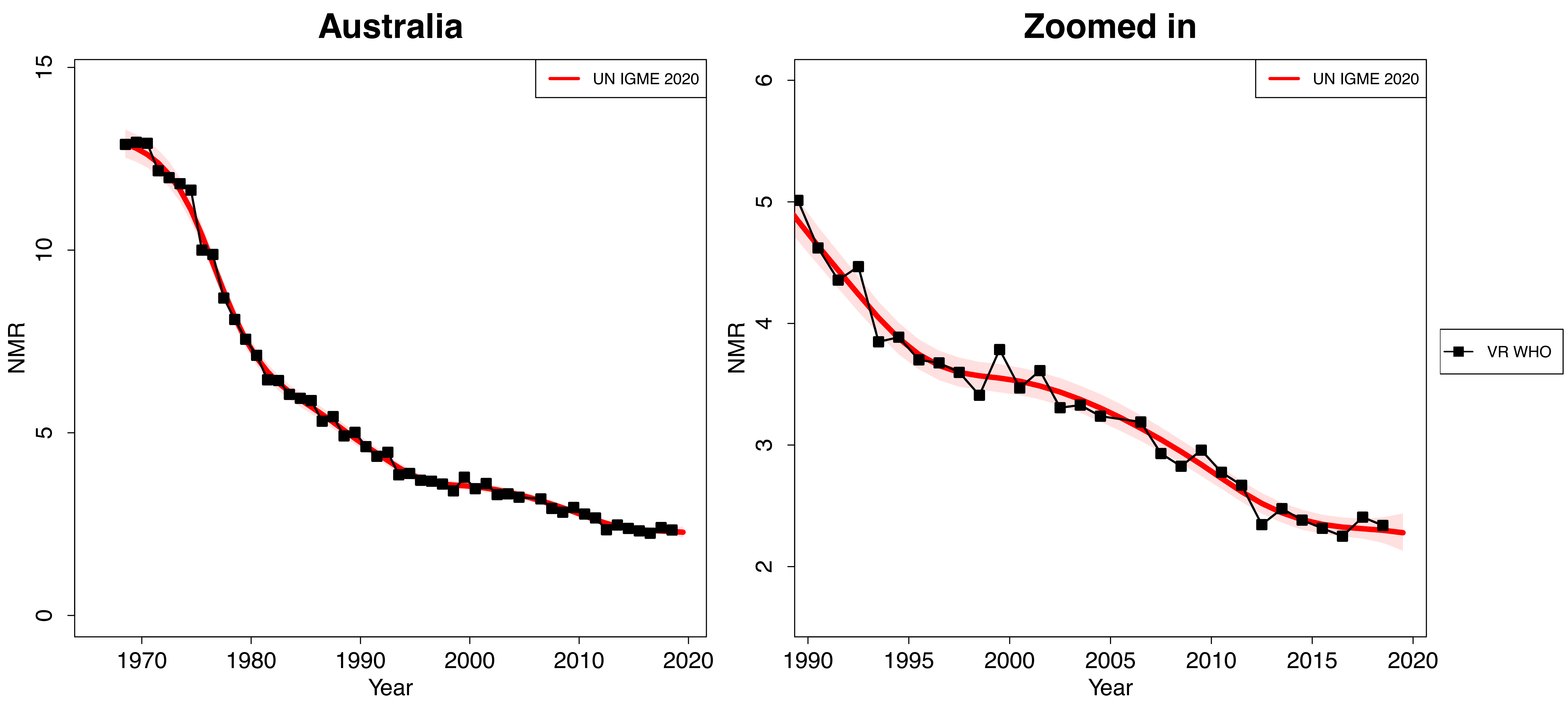

Australia
UN IGME 2020
NMR
Year
0
5
10
15
1970
1980
1990
2000
2010
2020
Zoomed in
UN IGME 2020
NMR
Year
2
3
4
5
6
1990
1995
2000
2005
2010
2015
2020
VR WHO

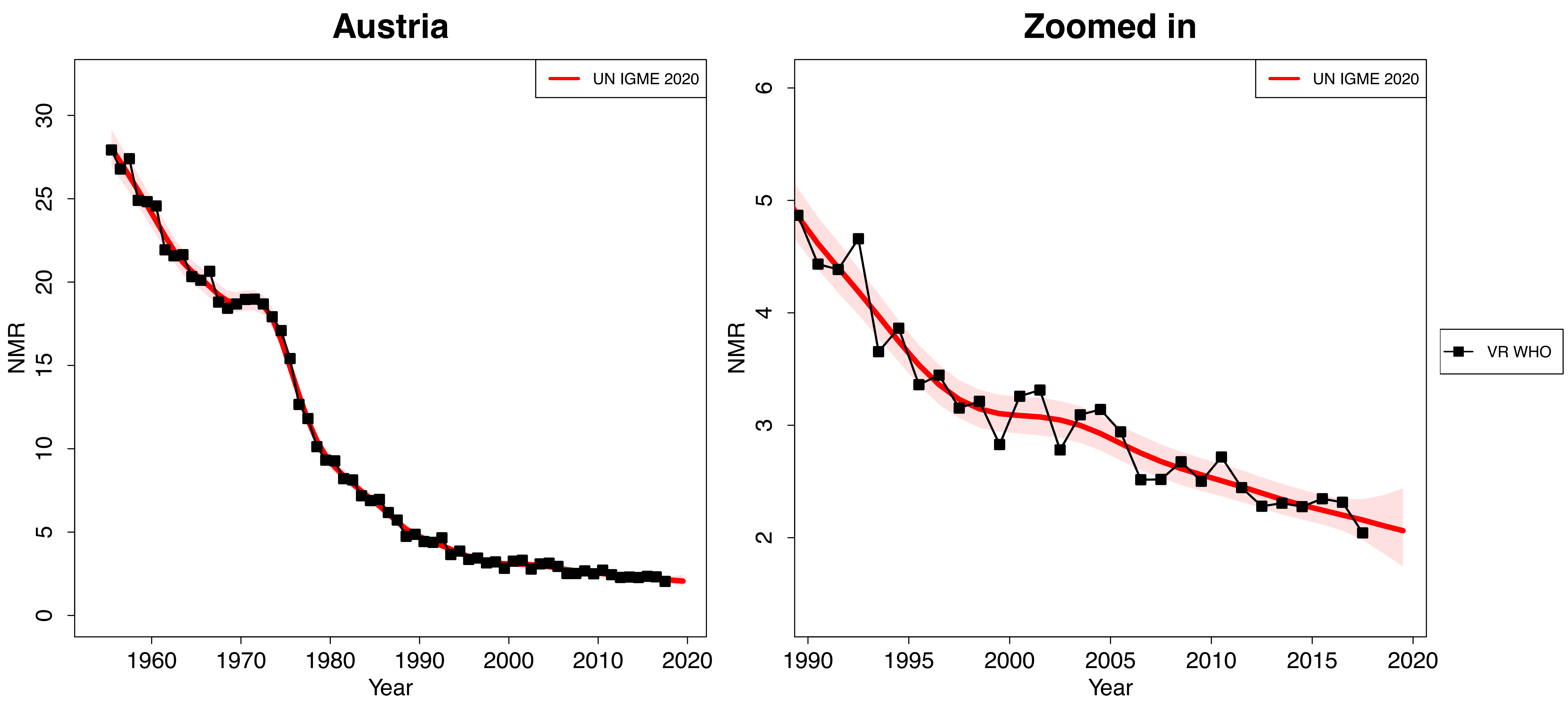

Austria
Zoomed in
UN IGME 2020
UN IGME 2020
VR WHO
NMR
NMR
Year
Year
0
5
10
15
20
25
30
1960
1970
1980
1990
2000
2010
2020
2
3
4
5
6
1990
1995
2000
2005
2010
2015
2020

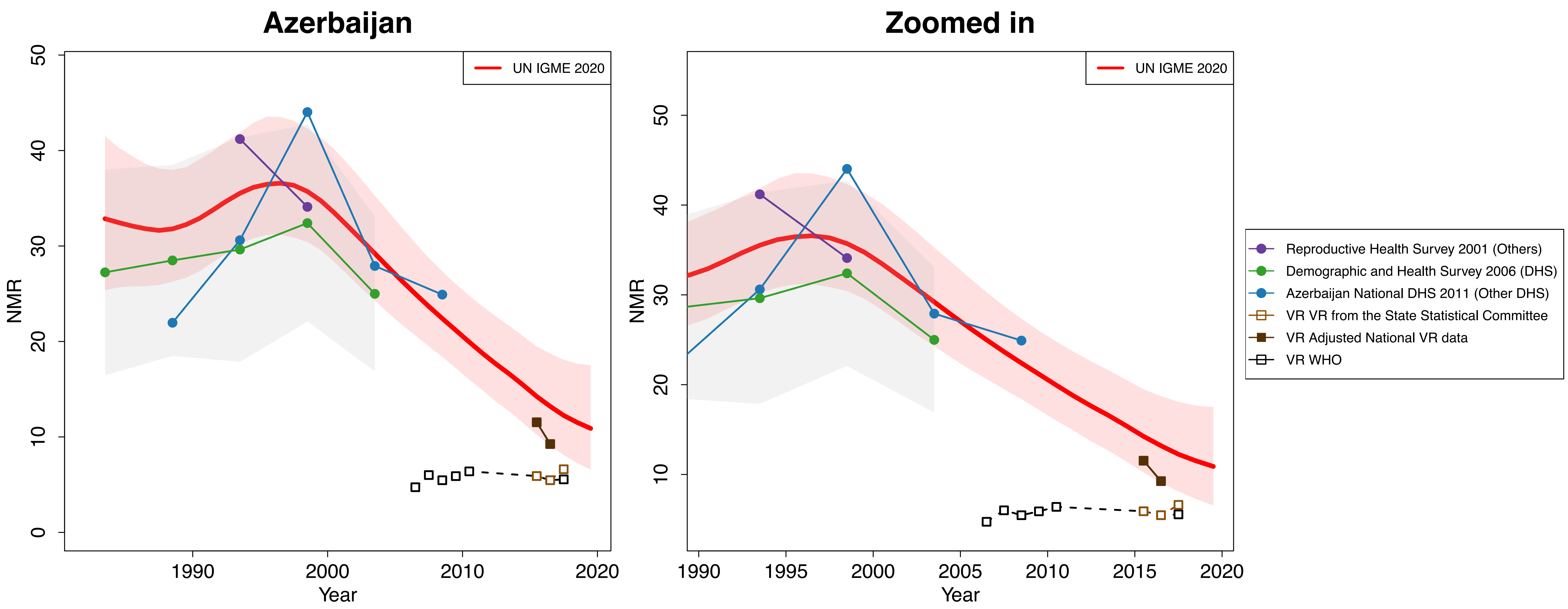
Azerbaijan
Zoomed in
UN IGME 2020
NMR
Year
Reproductive Health Survey 2001 (Others)
Demographic and Health Survey 2006 (DHS)
Azerbaijan National DHS 2011 (Other DHS)
VR VR from the State Statistical Committee
VR Adjusted National VR data
VR WHO

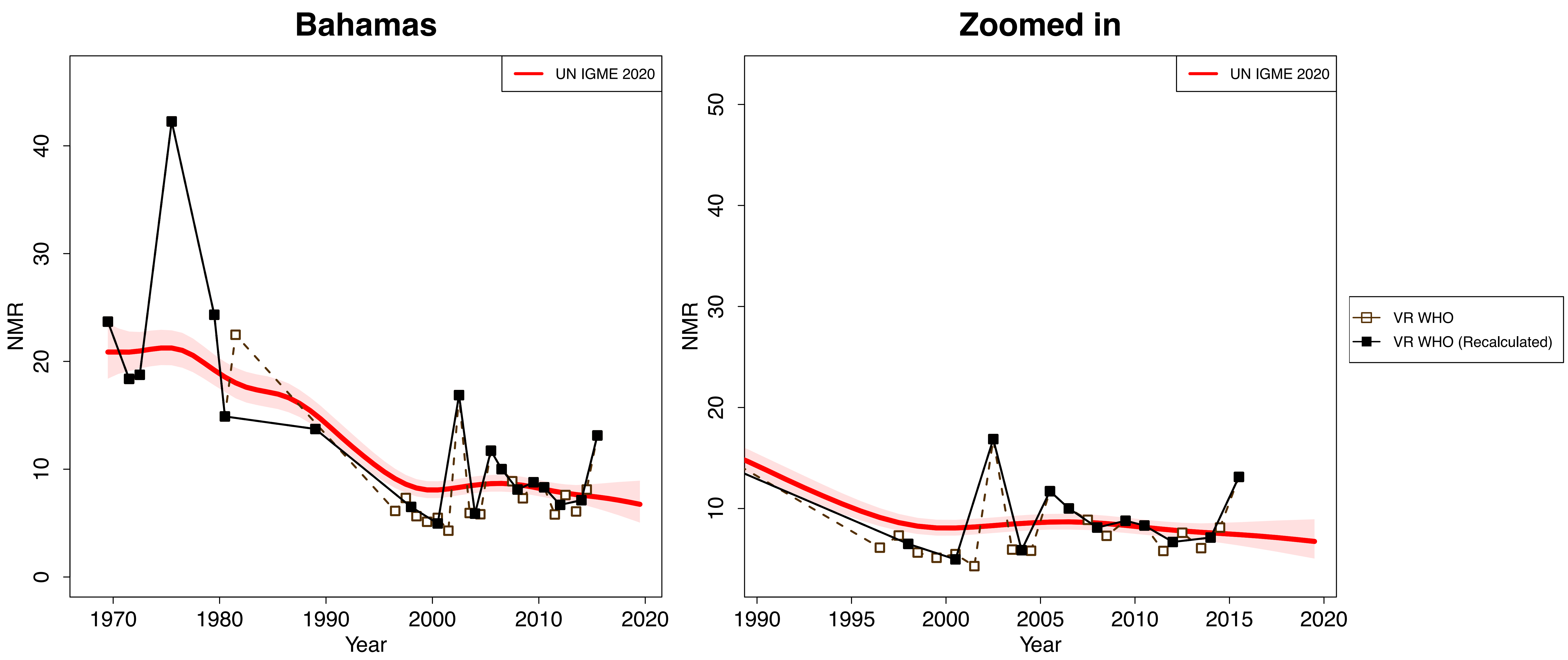

Bahamas
Zoomed in
UN IGME 2020
NMR
Year
0
10
20
30
40
50
1970
1980
1990
1995
2000
2005
2010
2015
2020
VR WHO
VR WHO (Recalculated)

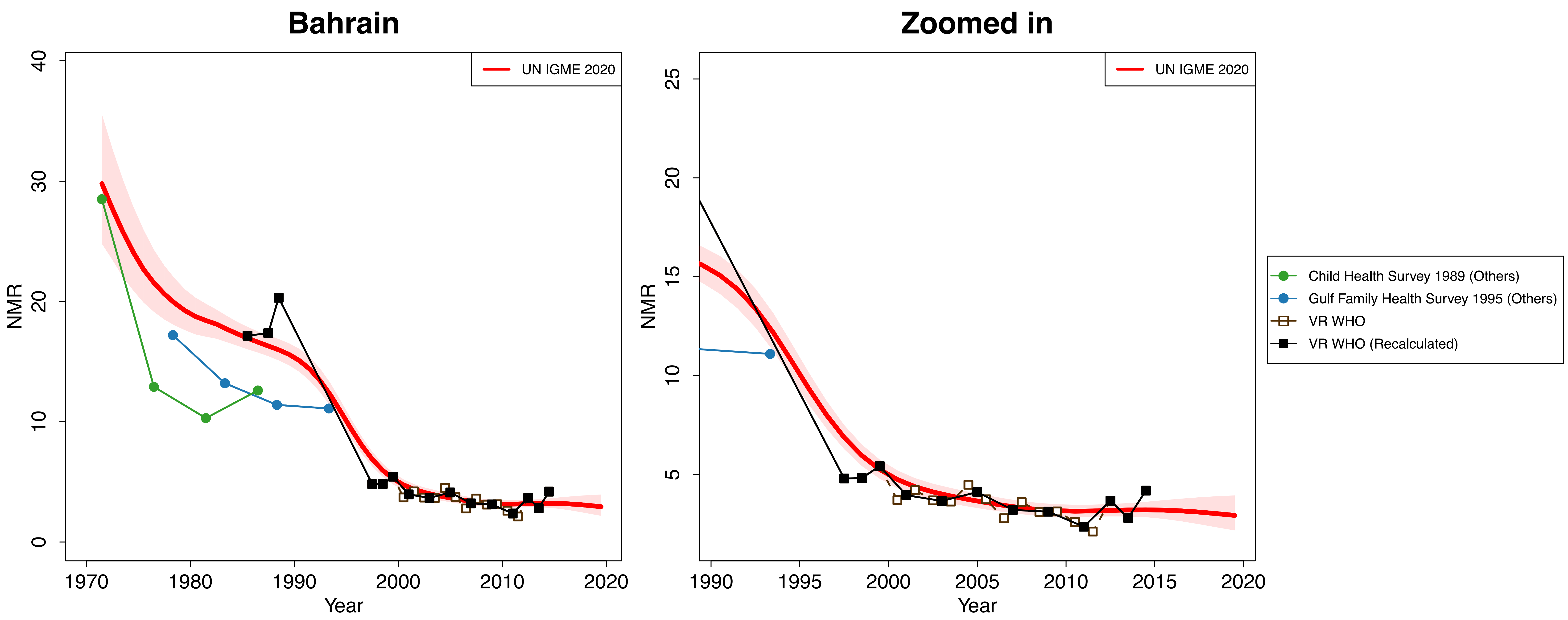

Bahrain
Zoomed in
UN IGME 2020
UN IGME 2020
NMR
NMR
Year
Year
Child Health Survey 1989 (Others)
Gulf Family Health Survey 1995 (Others)
VR WHO
VR WHO (Recalculated)

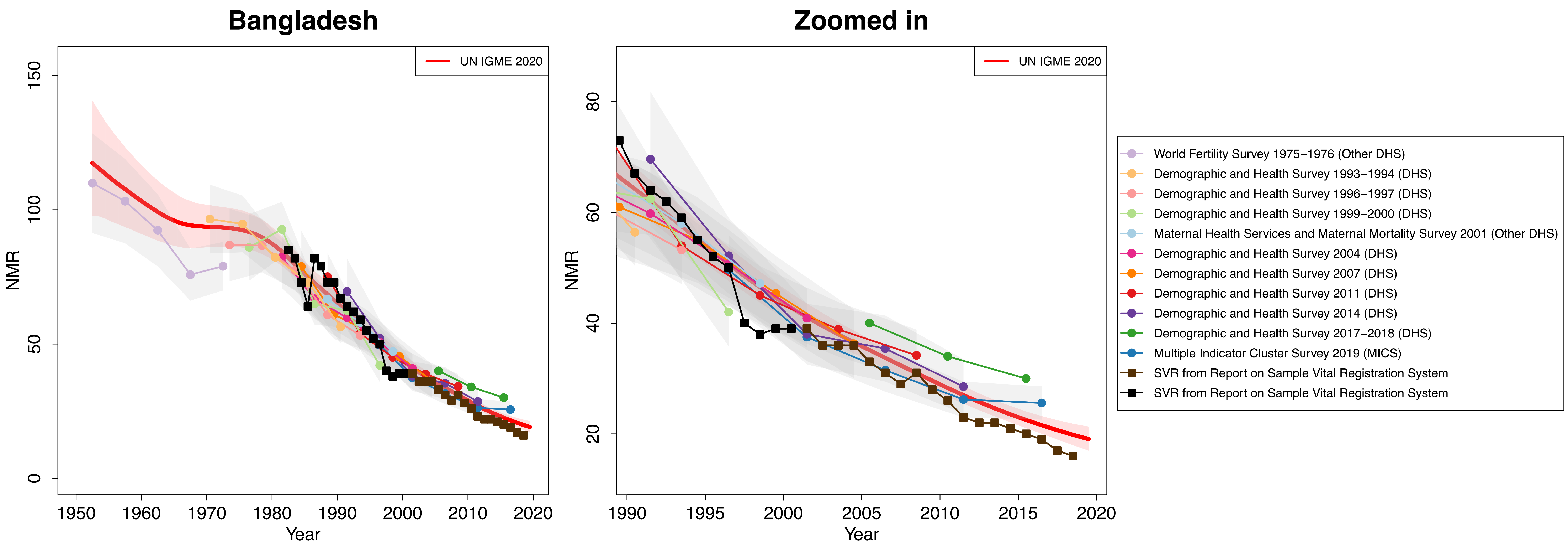

Bangladesh
Zoomed in
UN IGME 2020
NMR
Year
World Fertility Survey 1975–1976 (Other DHS)
Demographic and Health Survey 1993–1994 (DHS)
Demographic and Health Survey 1996–1997 (DHS)
Demographic and Health Survey 1999–2000 (DHS)
Maternal Health Services and Maternal Mortality Survey 2001 (Other DHS)
Demographic and Health Survey 2004 (DHS)
Demographic and Health Survey 2007 (DHS)
Demographic and Health Survey 2011 (DHS)
Demographic and Health Survey 2014 (DHS)
Demographic and Health Survey 2017–2018 (DHS)
Multiple Indicator Cluster Survey 2019 (MICS)
SVR from Report on Sample Vital Registration System
SVR from Report on Sample Vital Registration System

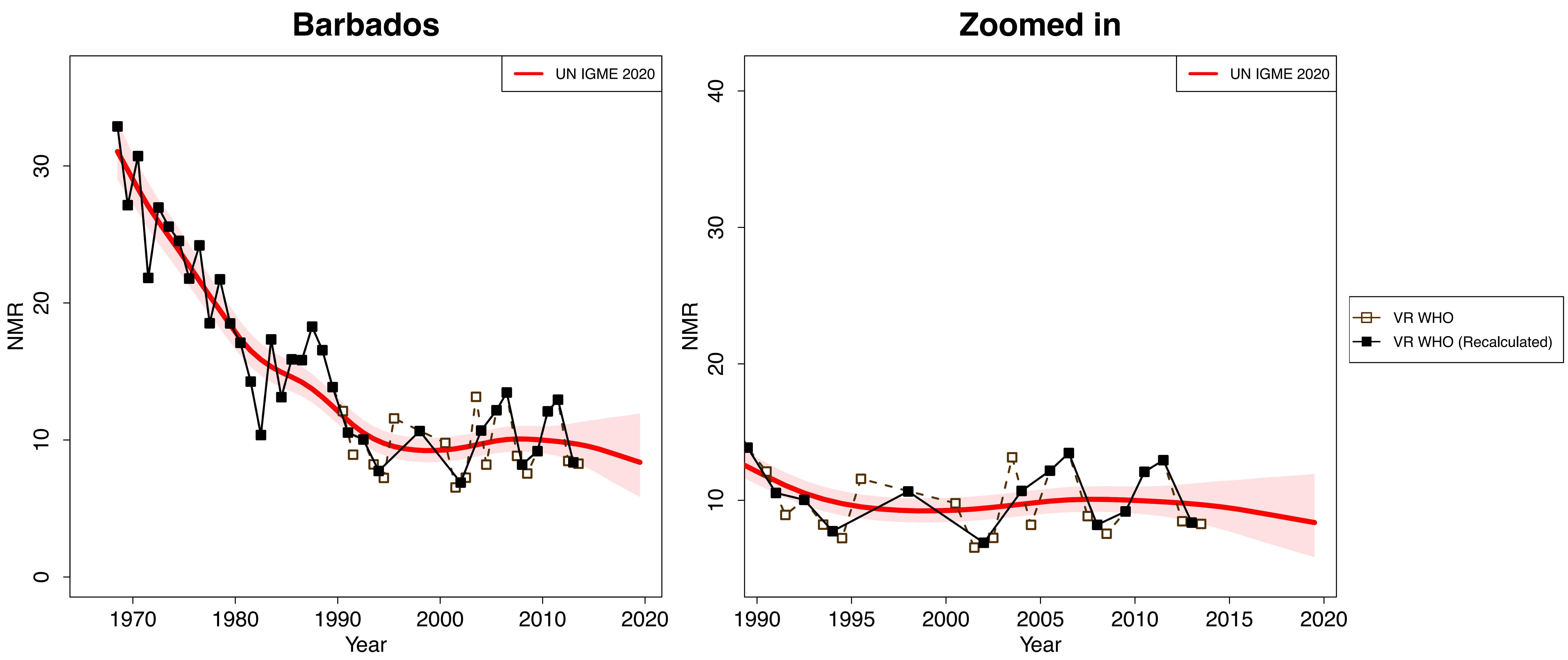

Barbados
Zoomed in
UN IGME 2020
UN IGME 2020
VR WHO
VR WHO (Recalculated)
NMR
NMR
Year
Year
0
10
20
30
10
20
30
40
1970
1980
1990
2000
2010
2020
1990
1995
2000
2005
2010
2015
2020

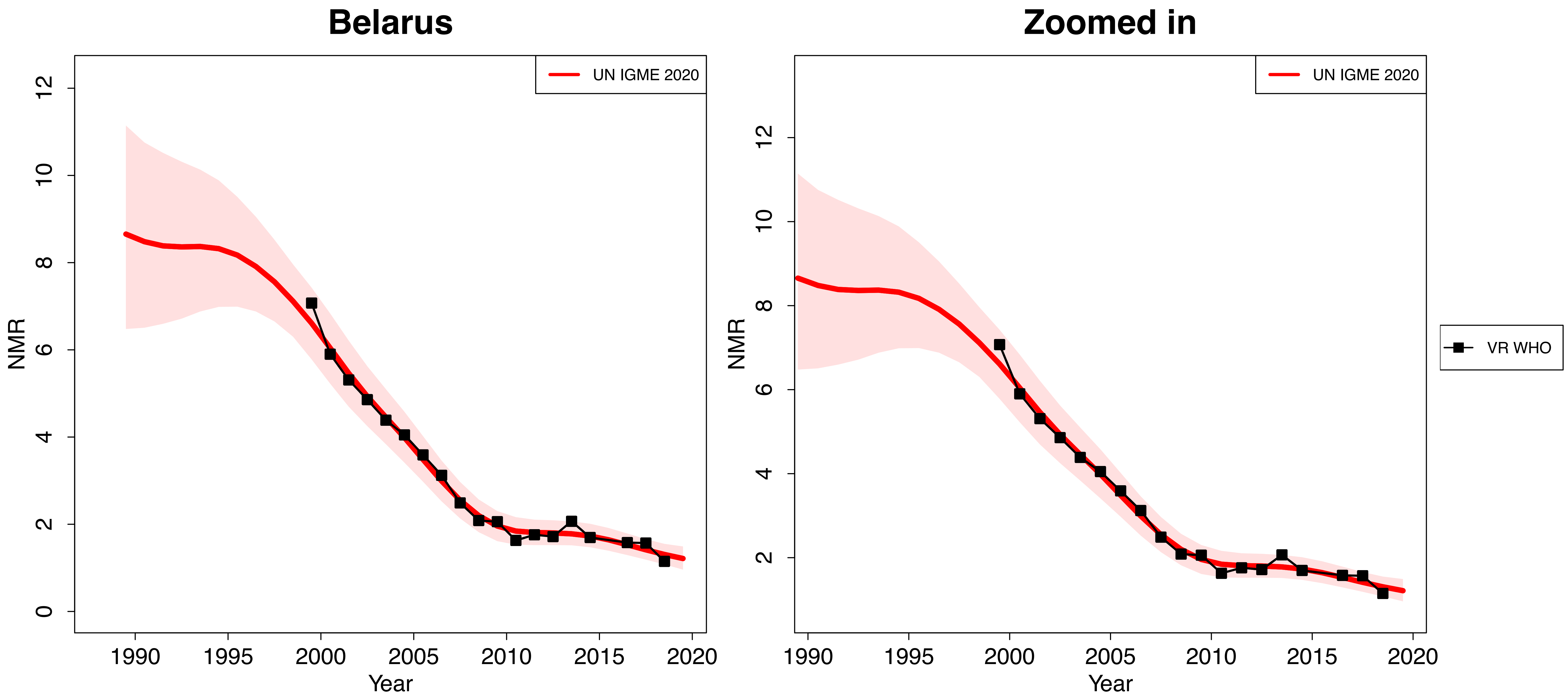

Belarus
Zoomed in
UN IGME 2020
VR WHO
NMR
Year
1990
1995
2000
2005
2010
2015
2020
0
2
4
6
8
10
12

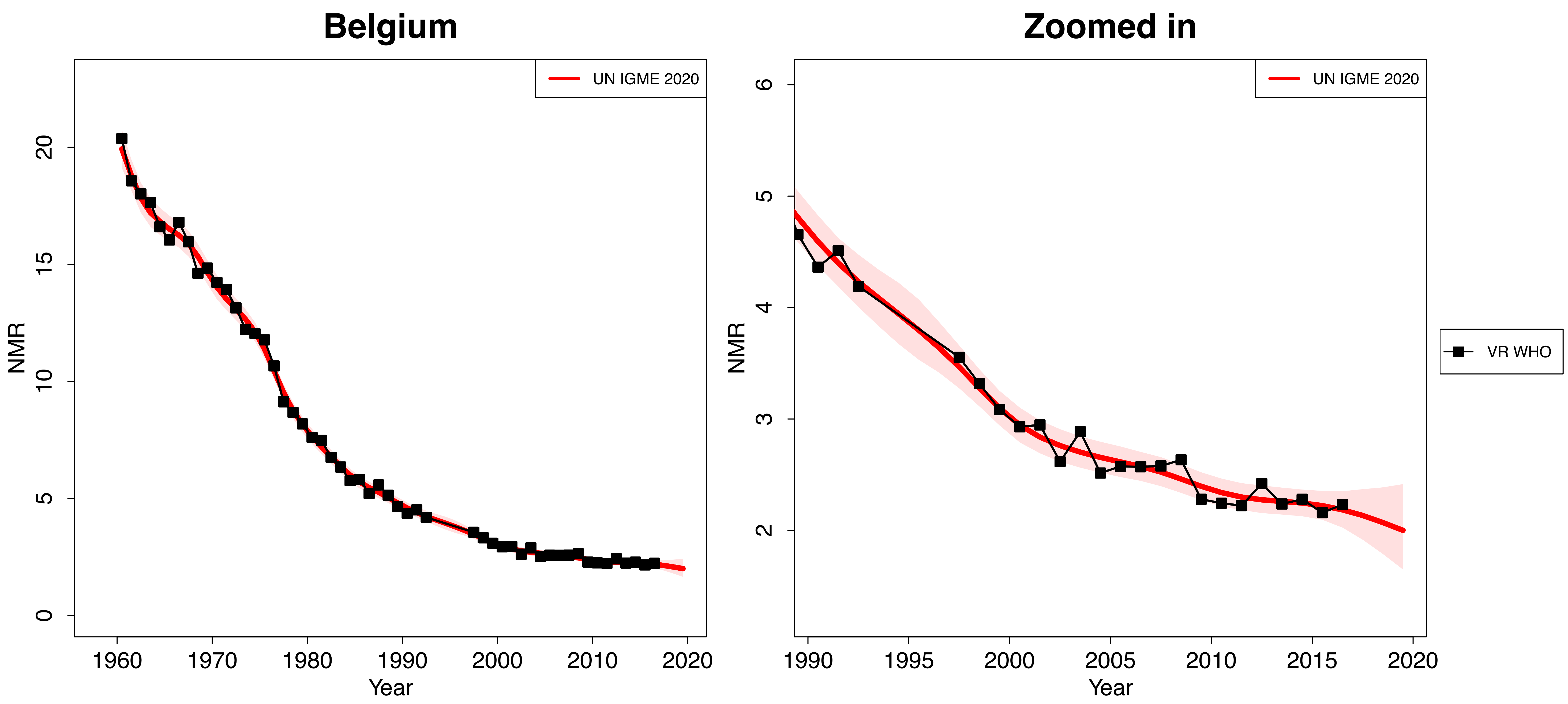

Belgium
Zoomed in
UN IGME 2020
UN IGME 2020
VR WHO
NMR
NMR
Year
Year
0
5
10
15
20
2
3
4
5
6
1960
1970
1980
1990
2000
2010
2020
1990
1995
2000
2005
2010
2015
2020

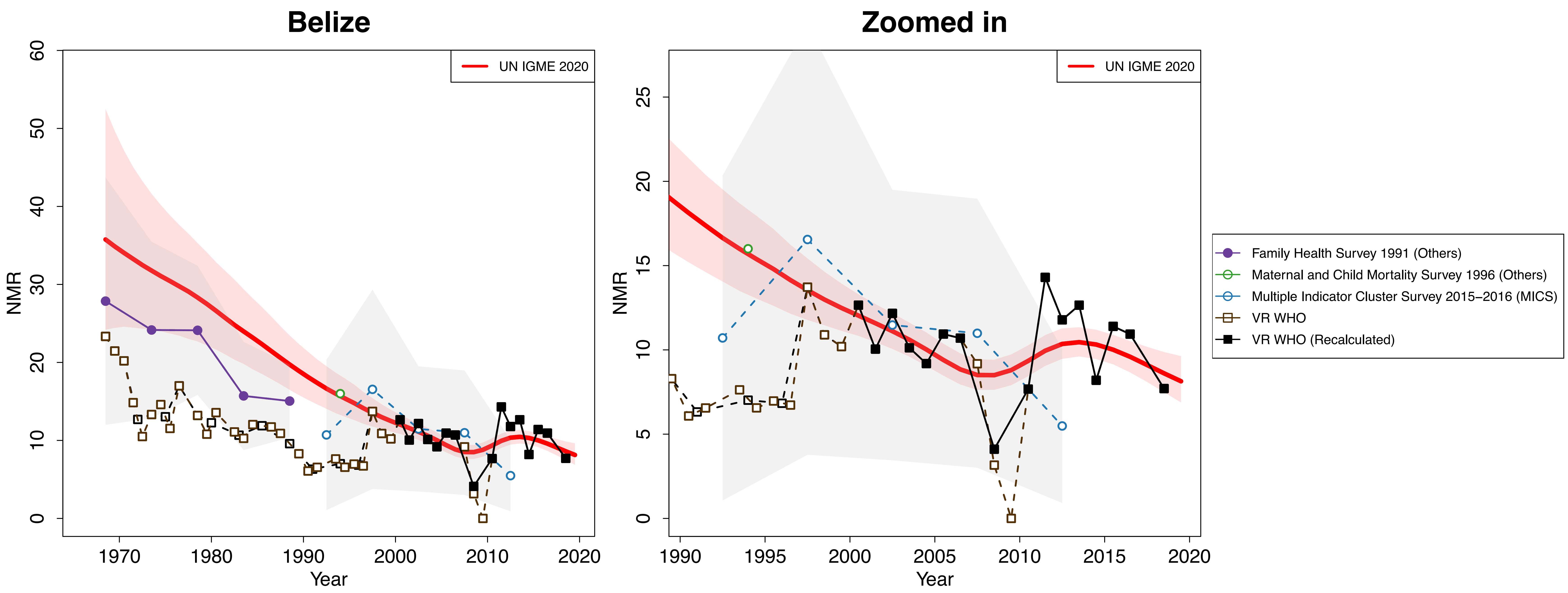

Belize
Zoomed in
UN IGME 2020
NMR
Year
Family Health Survey 1991 (Others)
Maternal and Child Mortality Survey 1996 (Others)
Multiple Indicator Cluster Survey 2015–2016 (MICS)
VR WHO
VR WHO (Recalculated)

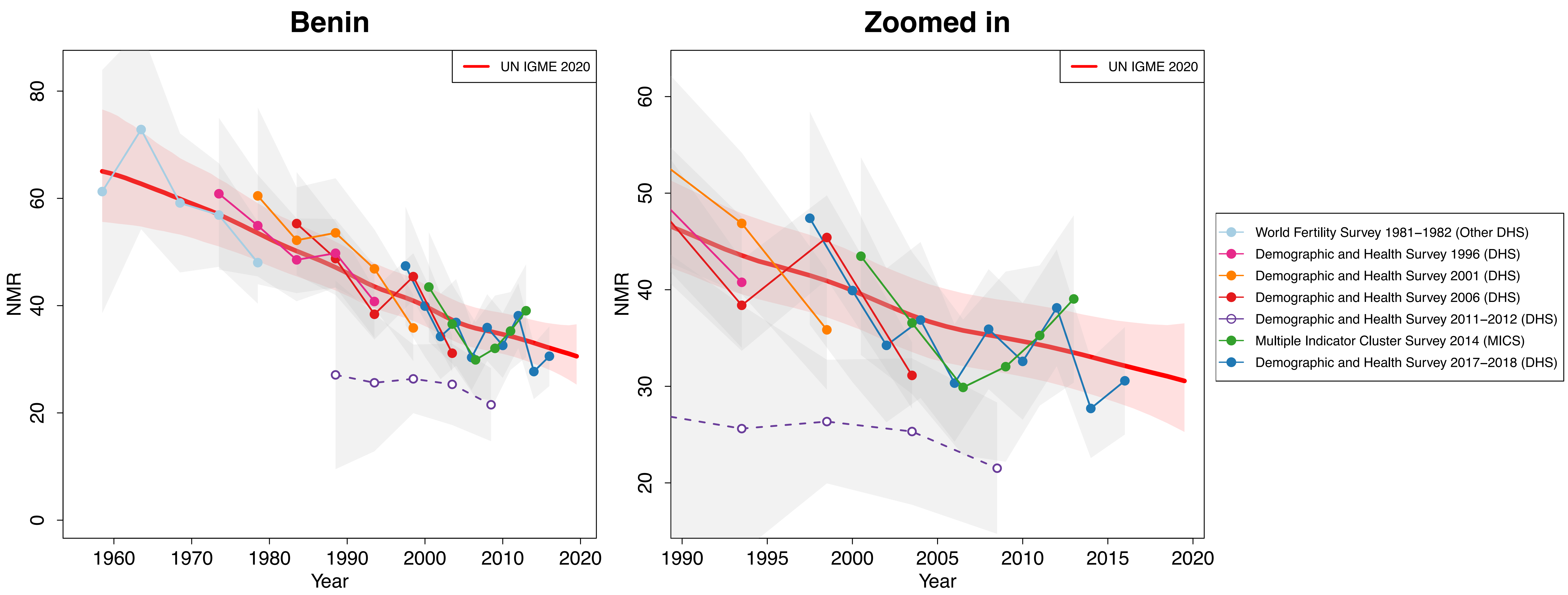

Benin
Zoomed in
UN IGME 2020
NMR
Year
World Fertility Survey 1981–1982 (Other DHS)
Demographic and Health Survey 1996 (DHS)
Demographic and Health Survey 2001 (DHS)
Demographic and Health Survey 2006 (DHS)
Demographic and Health Survey 2011–2012 (DHS)
Multiple Indicator Cluster Survey 2014 (MICS)
Demographic and Health Survey 2017–2018 (DHS)

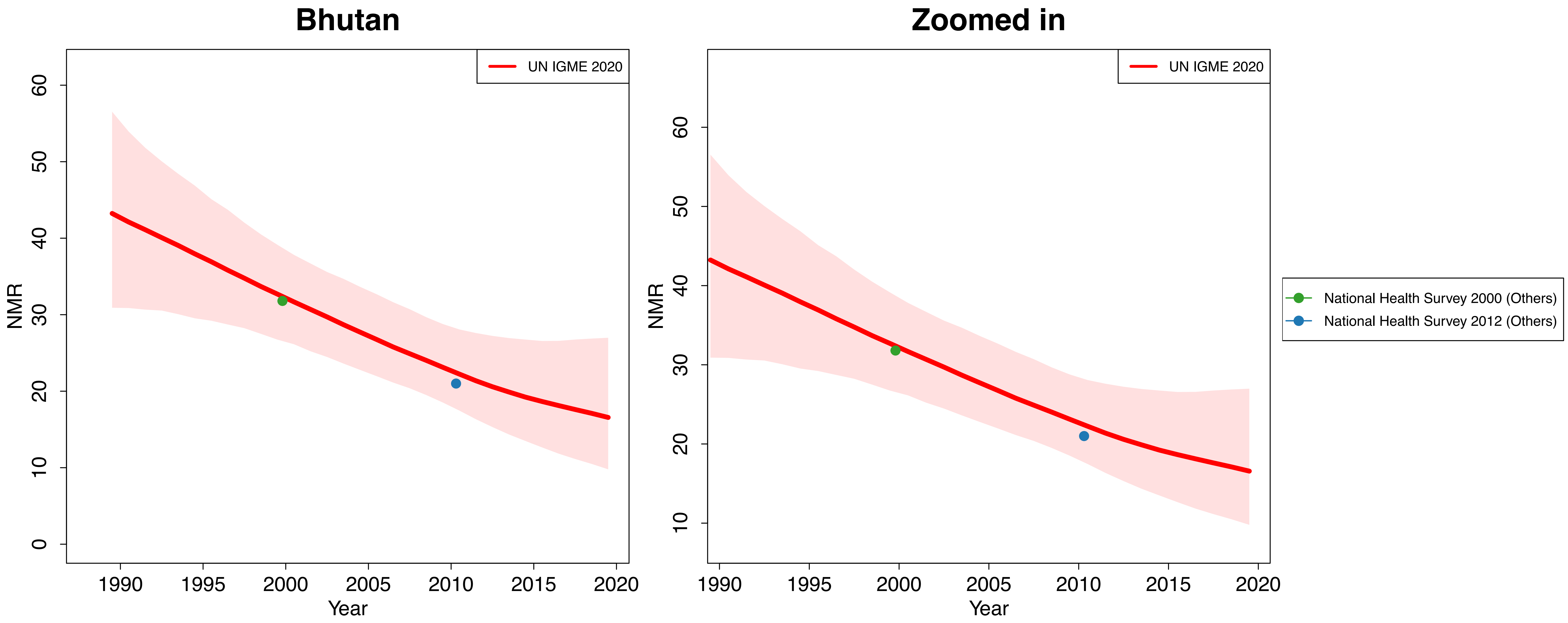

Bhutan
Zoomed in
UN IGME 2020
UN IGME 2020
National Health Survey 2000 (Others)
National Health Survey 2012 (Others)
NMR
NMR
Year
Year
0
10
20
30
40
50
60
1990
1995
2000
2005
2010
2015
2020

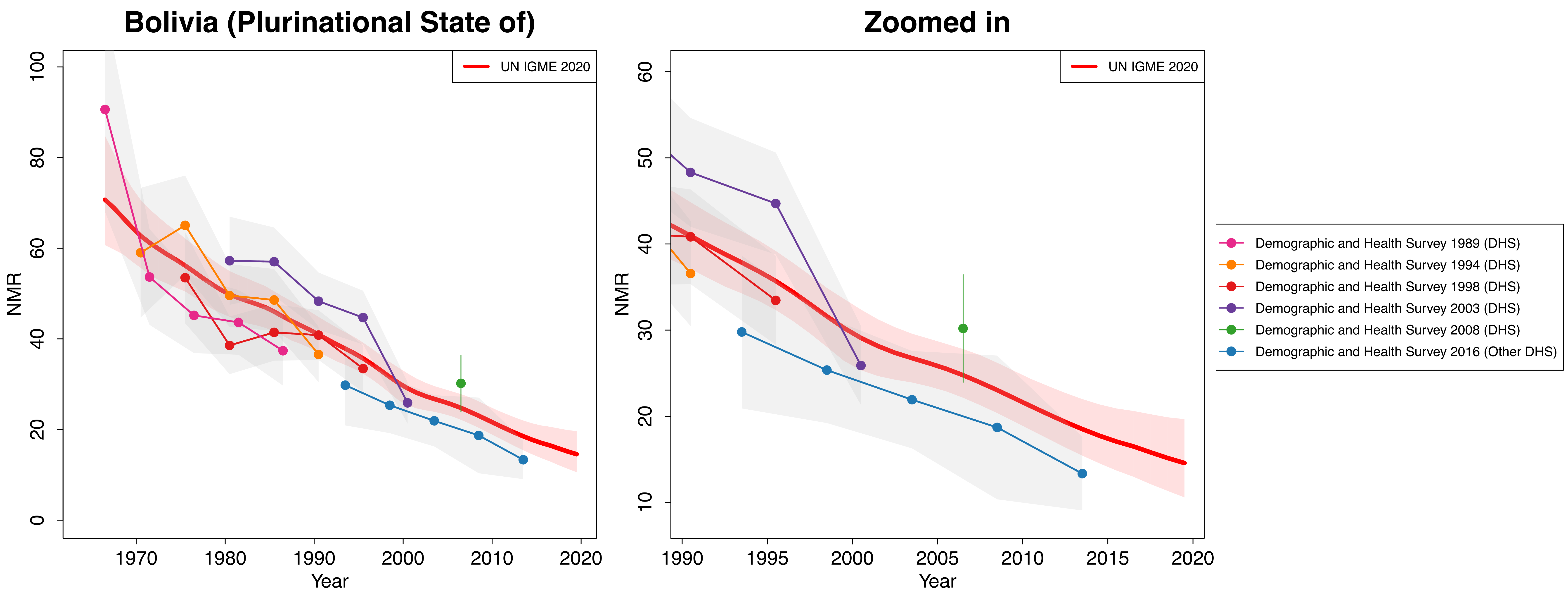

Bolivia (Plurinational State of)
Zoomed in
UN IGME 2020
NMR
Year
0
20
40
60
80
100
1970
1980
1990
2000
2010
2020
10
20
30
40
50
60
1990
1995
2000
2005
2010
2015
2020
Demographic and Health Survey 1989 (DHS)
Demographic and Health Survey 1994 (DHS)
Demographic and Health Survey 1998 (DHS)
Demographic and Health Survey 2003 (DHS)
Demographic and Health Survey 2008 (DHS)
Demographic and Health Survey 2016 (Other DHS)

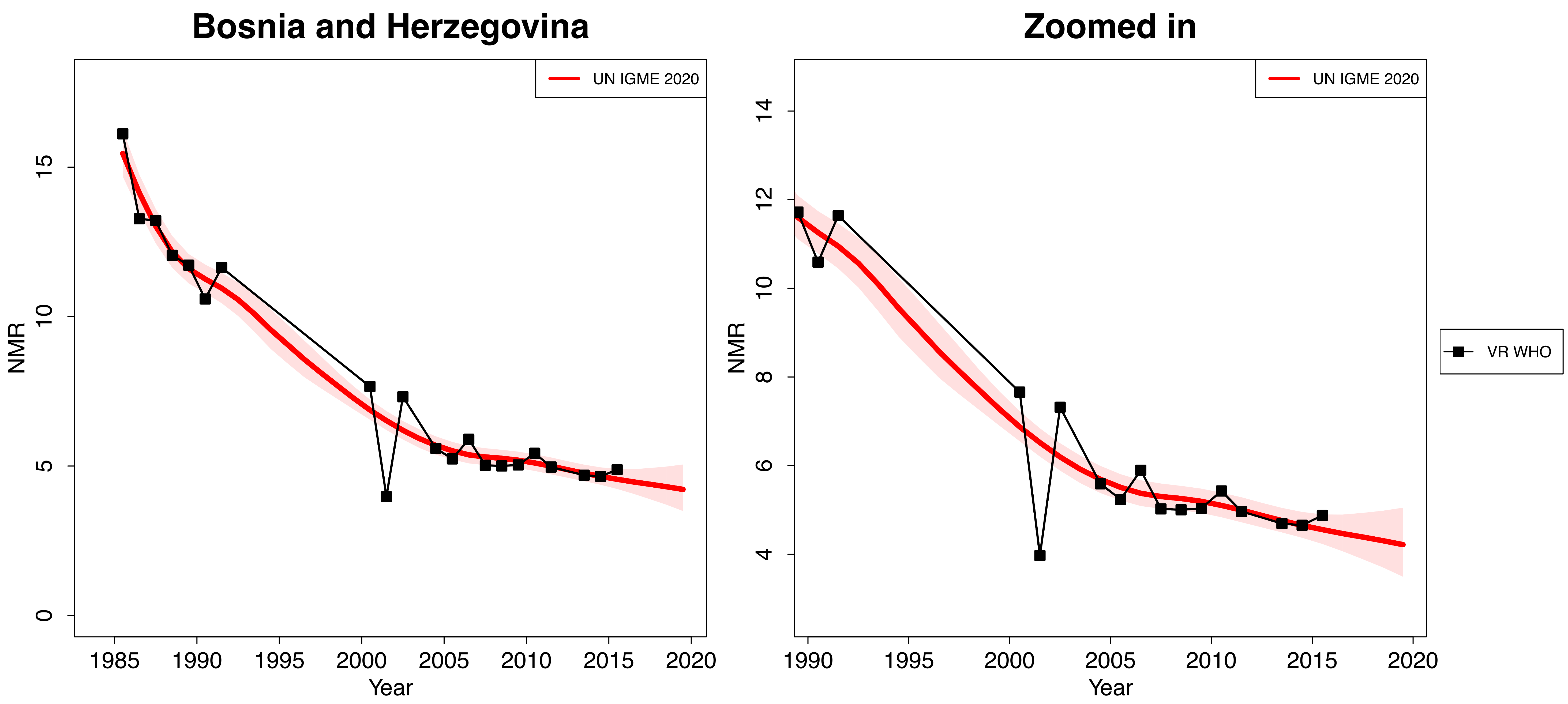

Bosnia and Herzegovina
Zoomed in
UN IGME 2020
VR WHO
NMR
Year

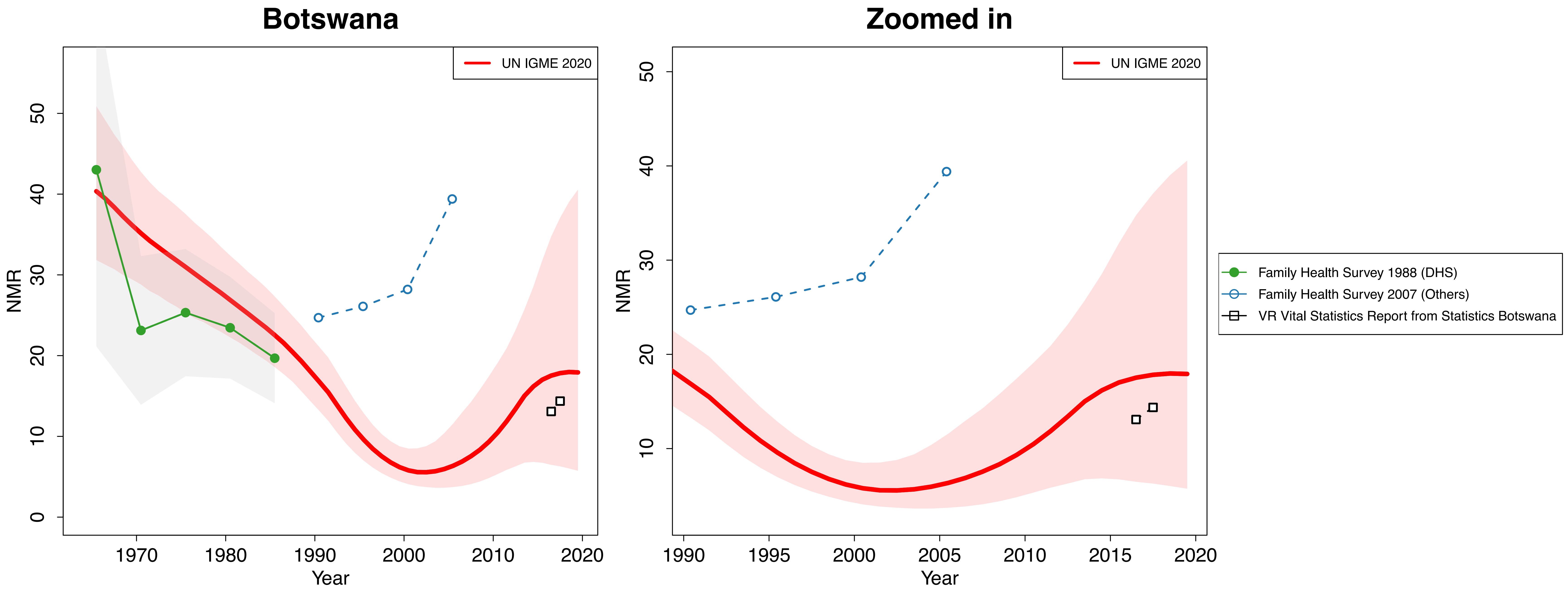

Botswana
Zoomed in
UN IGME 2020
NMR
Year
Family Health Survey 1988 (DHS)
Family Health Survey 2007 (Others)
VR Vital Statistics Report from Statistics Botswana

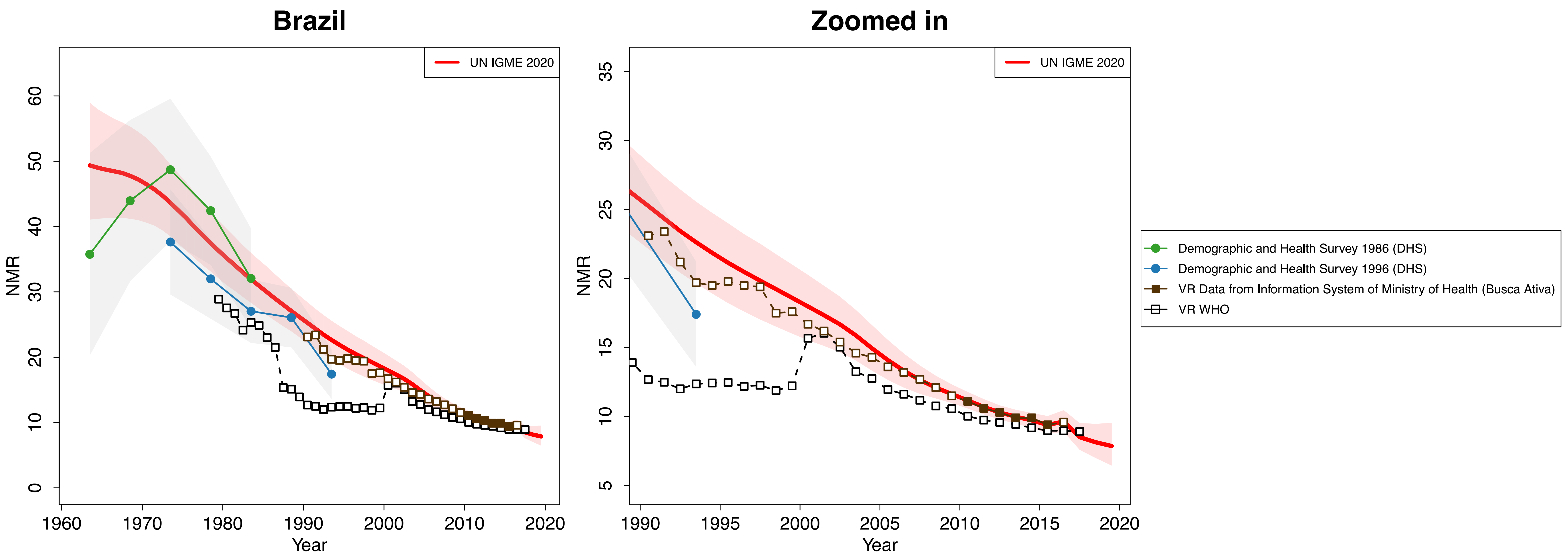

Brazil
Zoomed in
UN IGME 2020
NMR
Year
Demographic and Health Survey 1986 (DHS)
Demographic and Health Survey 1996 (DHS)
VR Data from Information System of Ministry of Health (Busca Ativa)
VR WHO

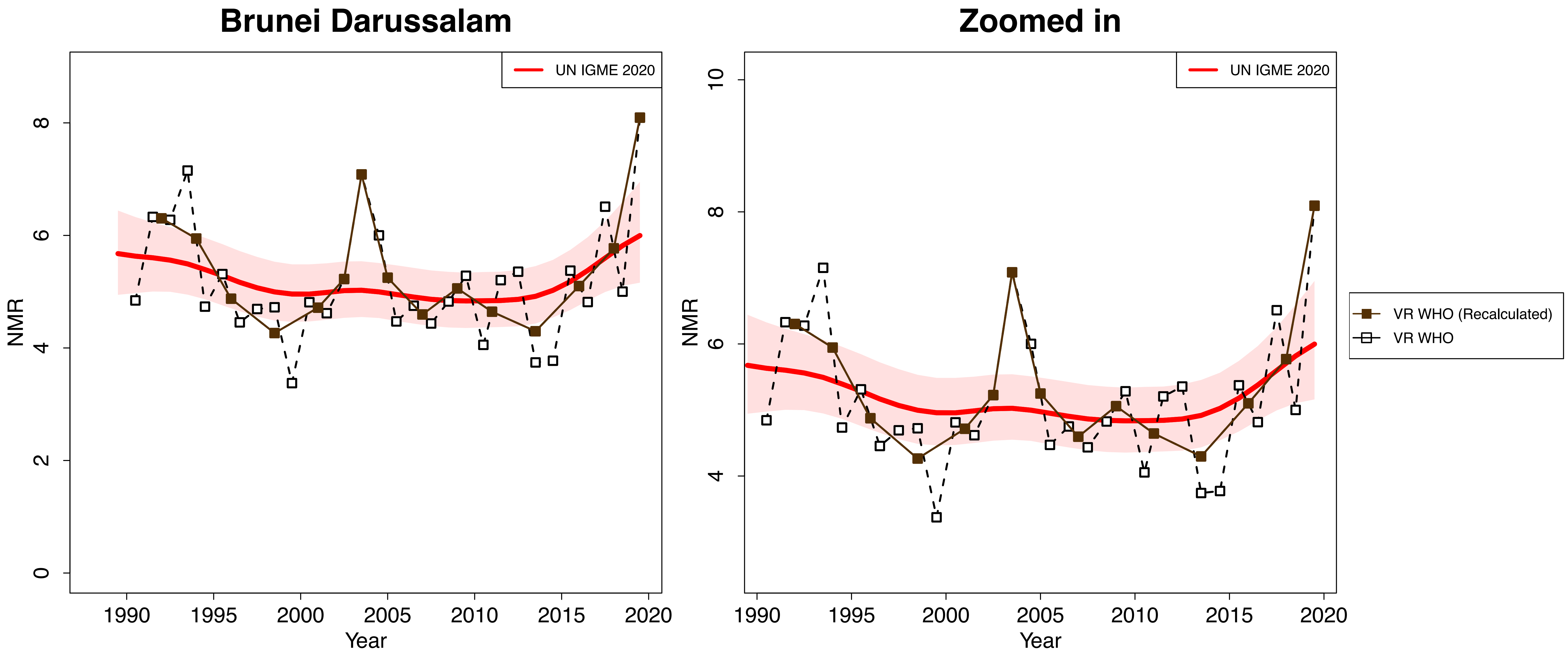
Brunei Darussalam
Zoomed in
UN IGME 2020
NMR
Year
VR WHO (Recalculated)
VR WHO

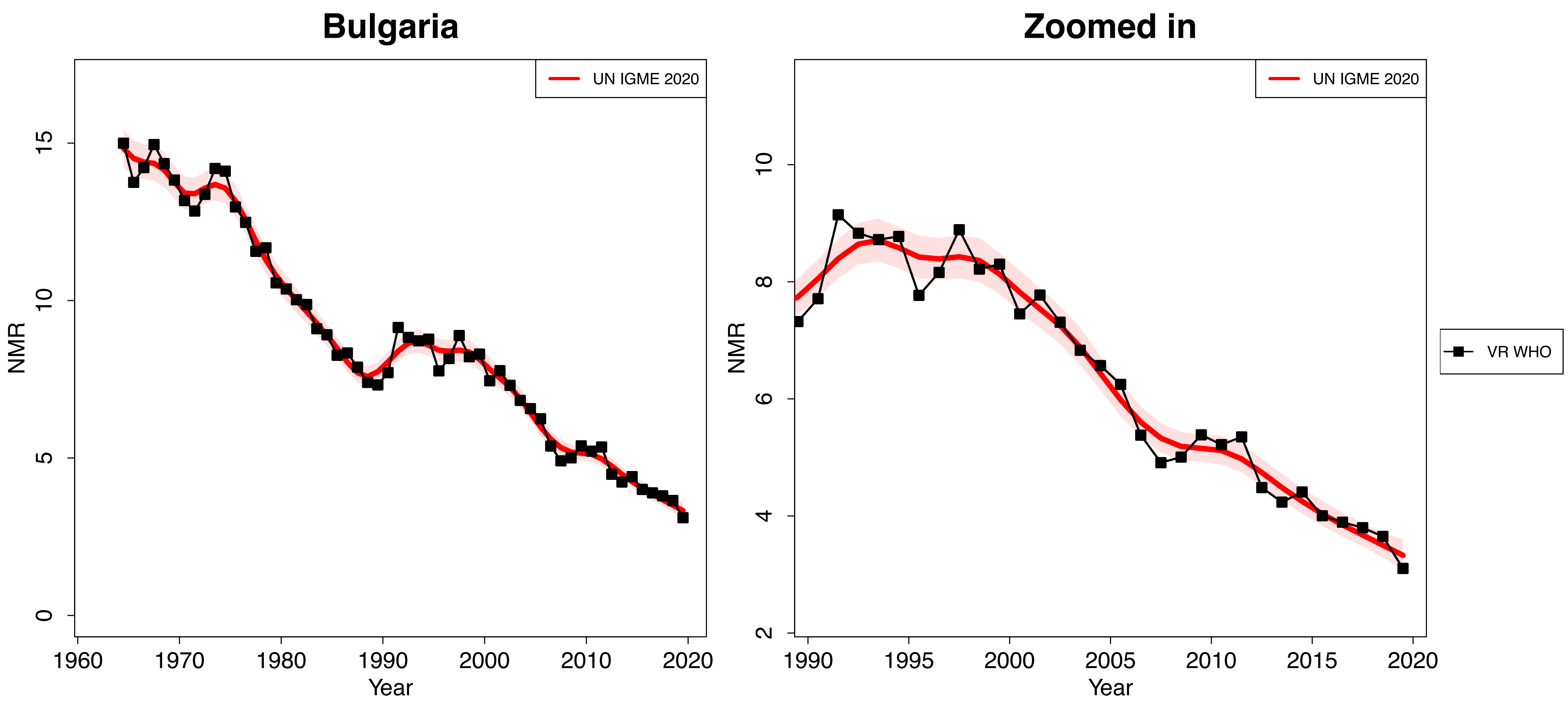

Bulgaria
Zoomed in
UN IGME 2020
UN IGME 2020
VR WHO
NMR
NMR
Year
Year

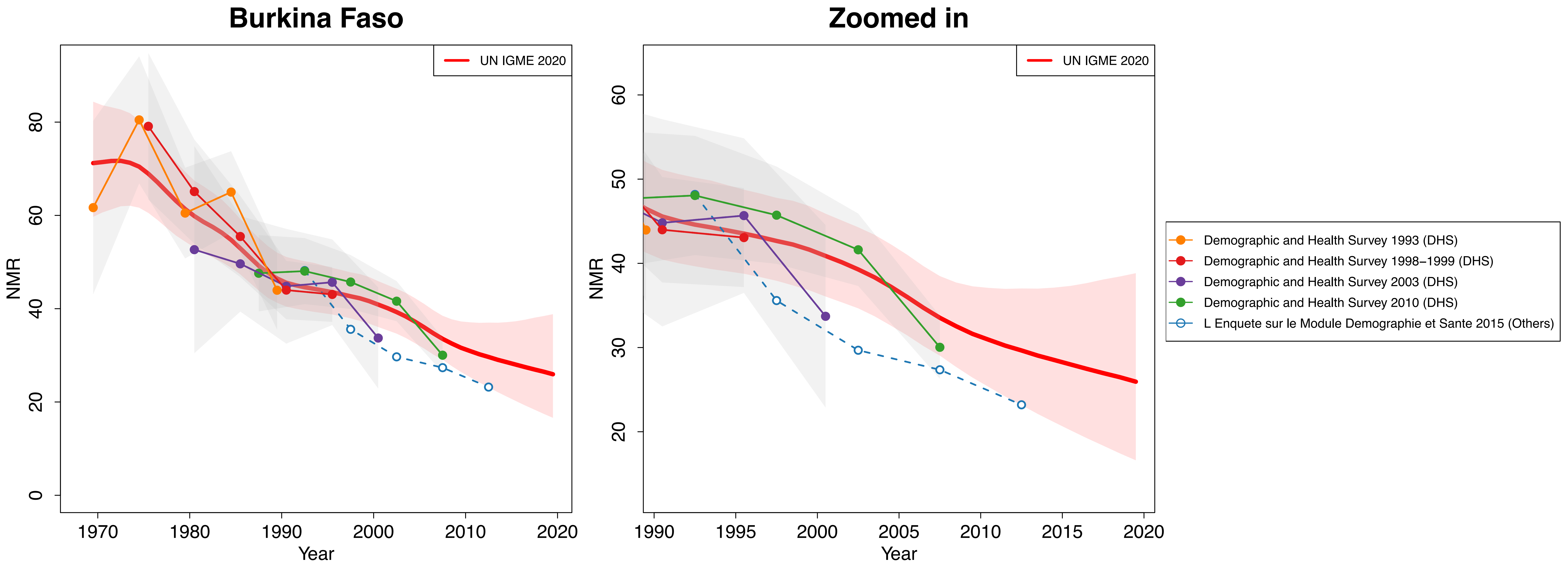

Burkina Faso
Zoomed in
UN IGME 2020
NMR
Year
0
20
40
60
80
1970
1980
1990
2000
2010
2020
20
30
40
50
60
1990
1995
2000
2005
2010
2015
2020
Demographic and Health Survey 1993 (DHS)
Demographic and Health Survey 1998–1999 (DHS)
Demographic and Health Survey 2003 (DHS)
Demographic and Health Survey 2010 (DHS)
L Enquete sur le Module Demographie et Sante 2015 (Others)

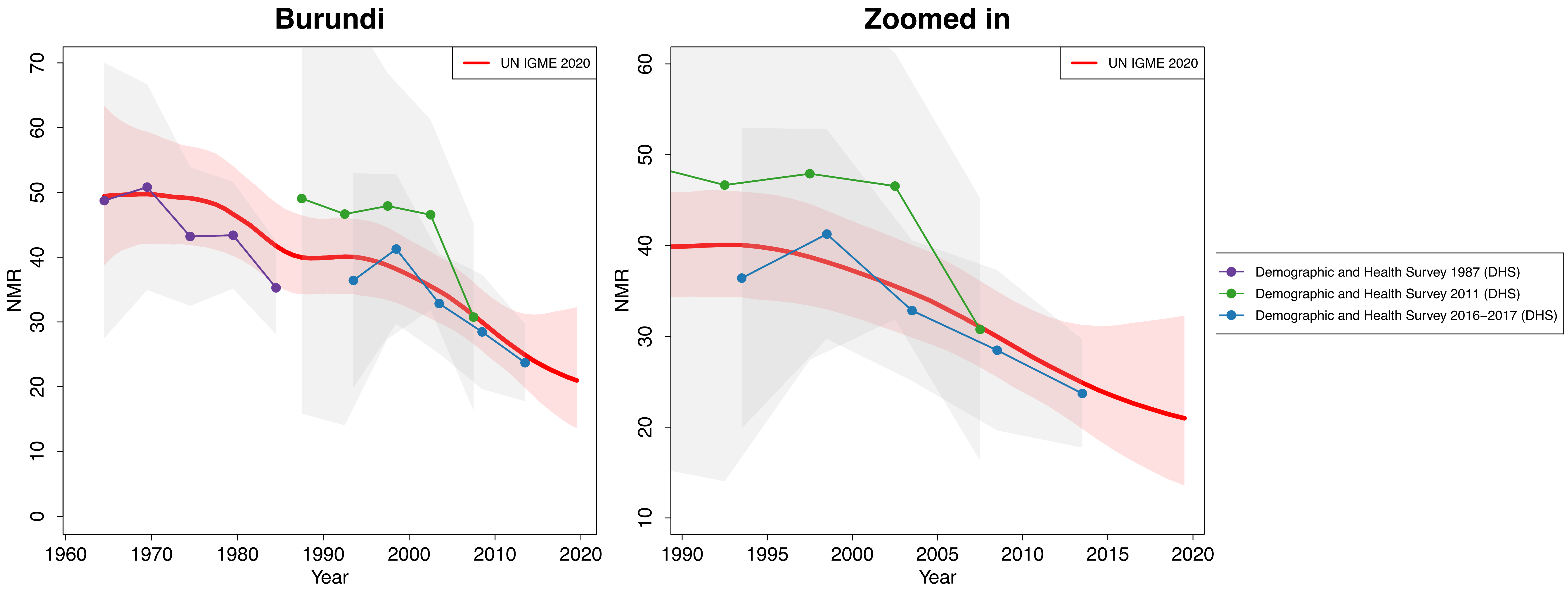

Burundi
Zoomed in
UN IGME 2020
NMR
Year
Demographic and Health Survey 1987 (DHS)
Demographic and Health Survey 2011 (DHS)
Demographic and Health Survey 2016–2017 (DHS)

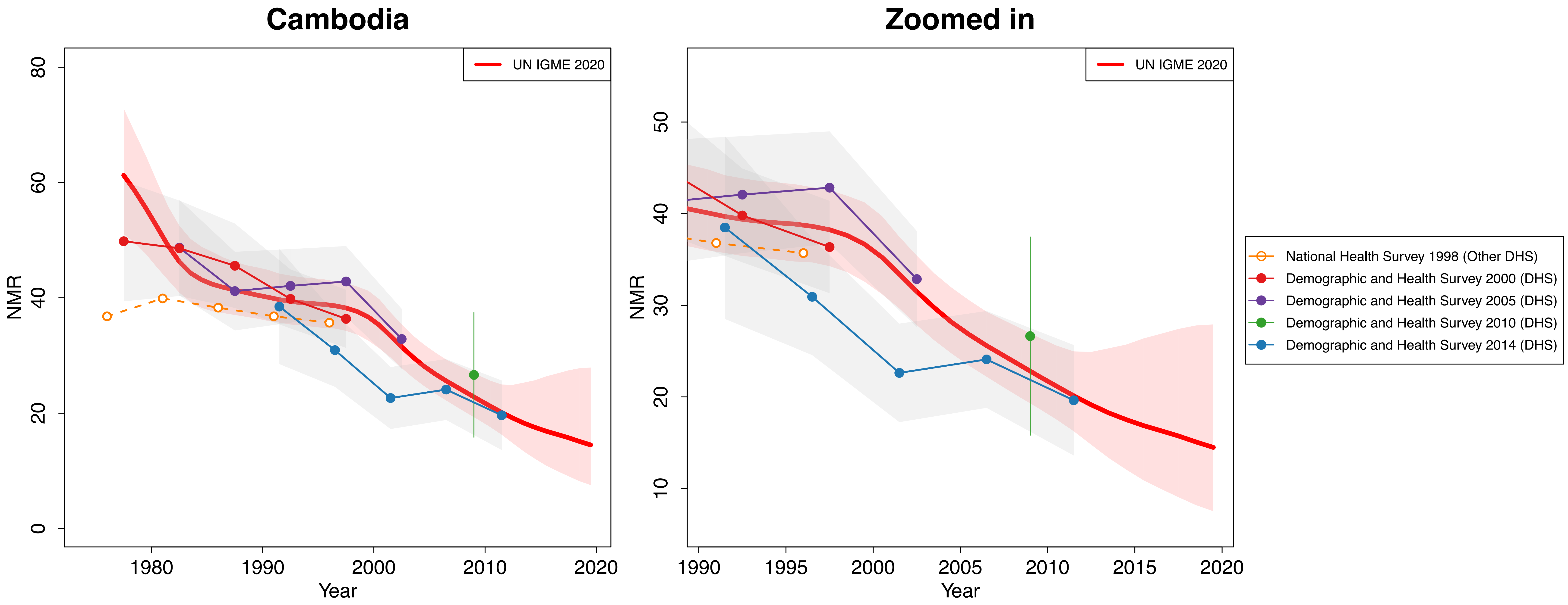

Cambodia
Zoomed in
UN IGME 2020
NMR
Year
National Health Survey 1998 (Other DHS)
Demographic and Health Survey 2000 (DHS)
Demographic and Health Survey 2005 (DHS)
Demographic and Health Survey 2010 (DHS)
Demographic and Health Survey 2014 (DHS)

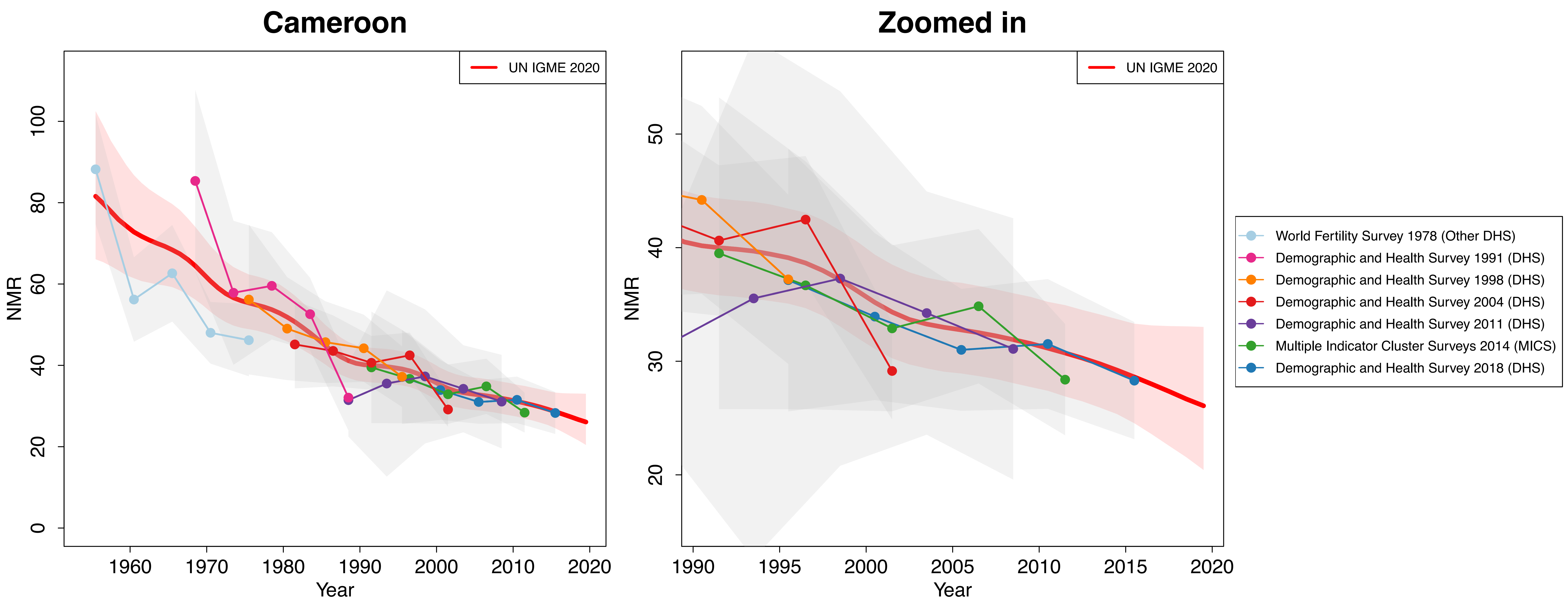

Cameroon
Zoomed in
UN IGME 2020
NMR
Year
World Fertility Survey 1978 (Other DHS)
Demographic and Health Survey 1991 (DHS)
Demographic and Health Survey 1998 (DHS)
Demographic and Health Survey 2004 (DHS)
Demographic and Health Survey 2011 (DHS)
Multiple Indicator Cluster Surveys 2014 (MICS)
Demographic and Health Survey 2018 (DHS)

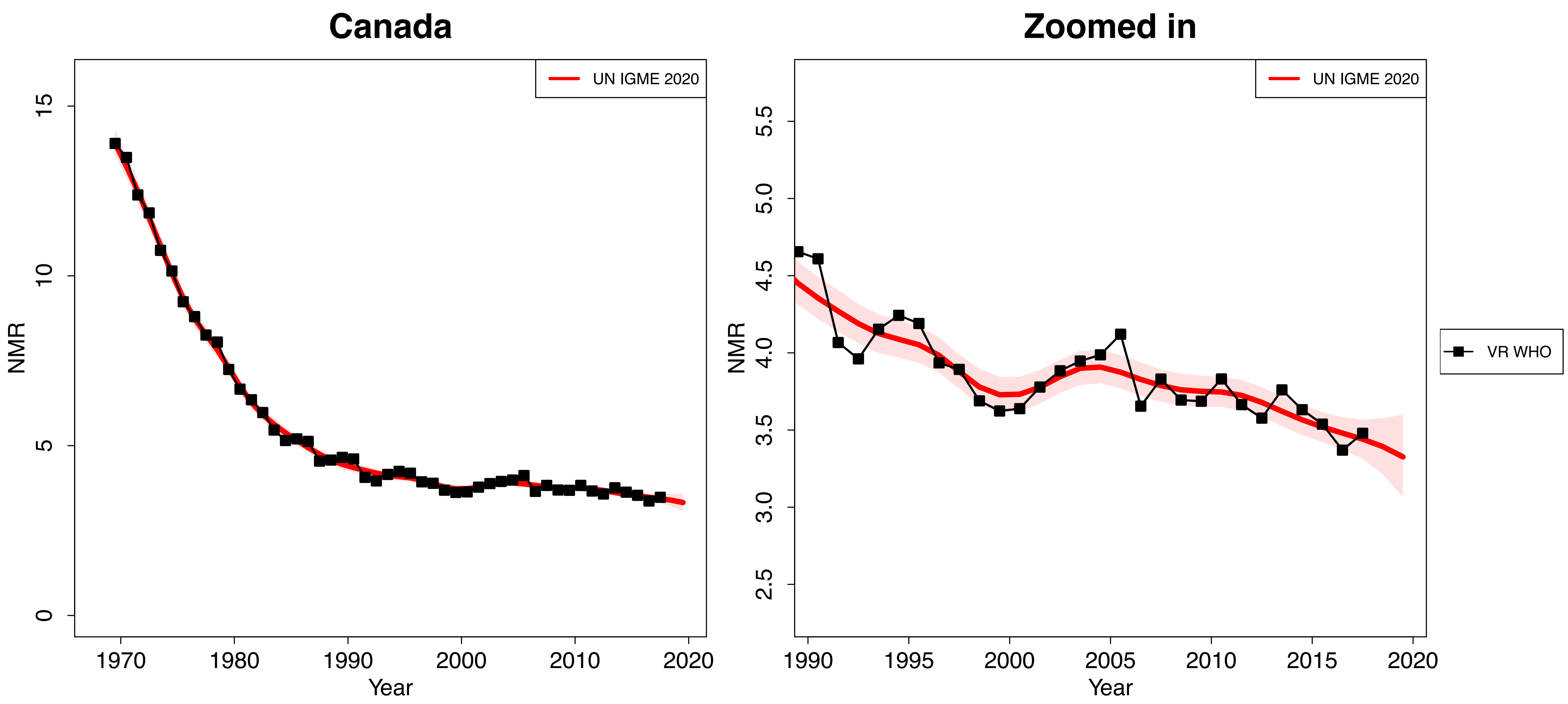

Canada
UN IGME 2020
NMR
0
5
10
15
1970
1980
1990
2000
2010
2020
Year
Zoomed in
UN IGME 2020
NMR
2.5
3.0
3.5
4.0
4.5
5.0
5.5
1990
1995
2000
2005
2010
2015
2020
Year
VR WHO

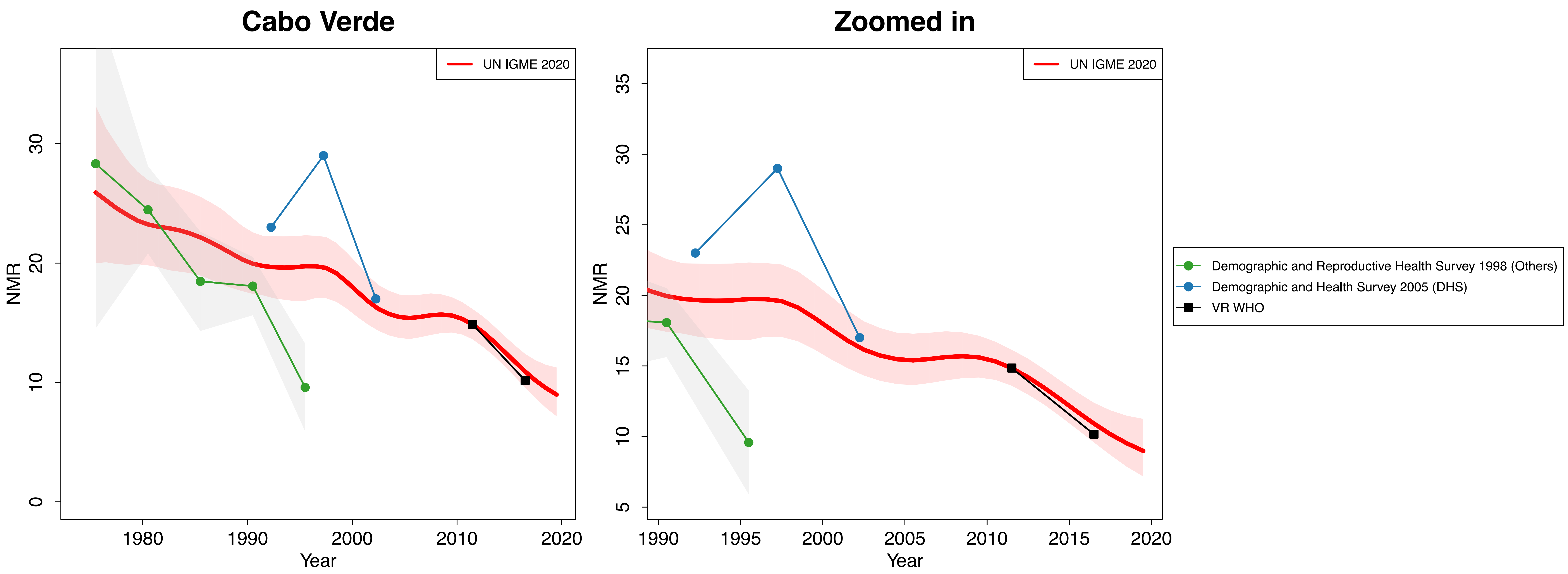

Cabo Verde
Zoomed in
UN IGME 2020
UN IGME 2020
NMR
Year
1980
1990
2000
2010
2020
0
10
20
30
1995
2005
2015
5
15
25
35
Demographic and Reproductive Health Survey 1998 (Others)
Demographic and Health Survey 2005 (DHS)
VR WHO

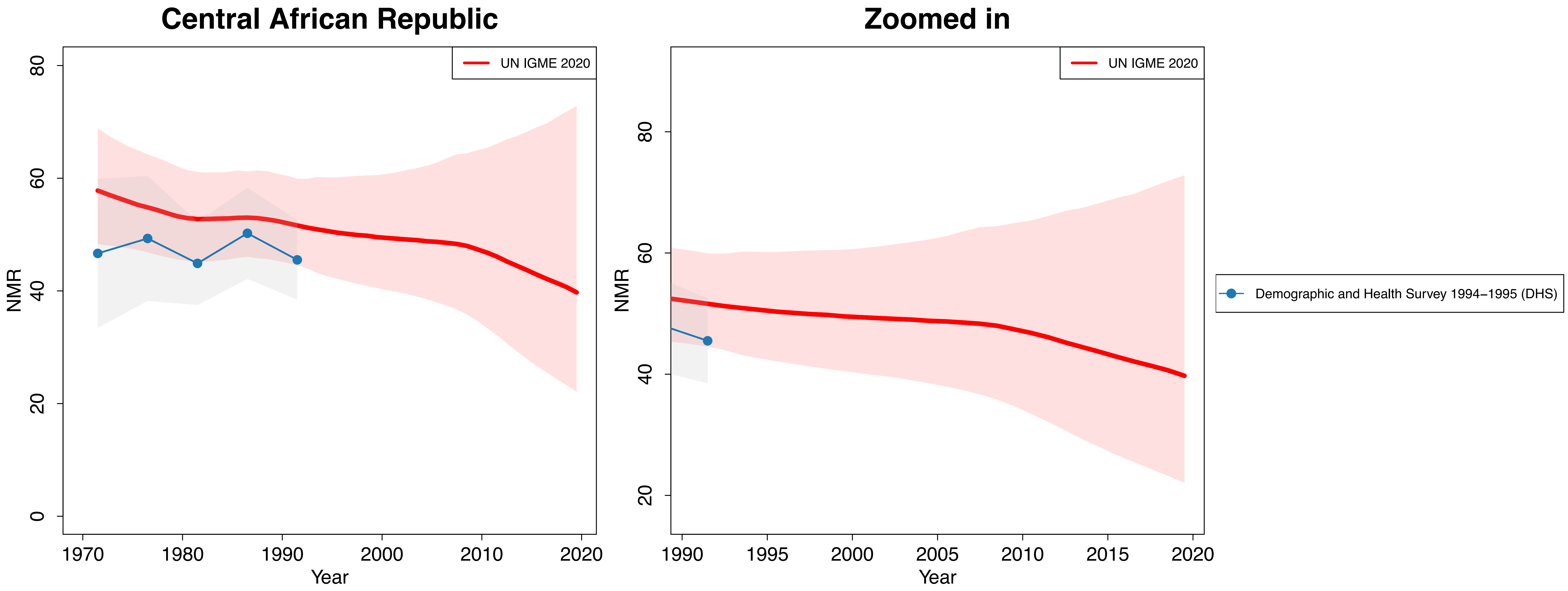
Central African Republic
Zoomed in
UN IGME 2020
UN IGME 2020
NMR
NMR
Year
Year
0
20
40
60
80
20
40
60
80
1970
1980
1990
2000
2010
2020
1990
1995
2000
2005
2010
2015
2020
Demographic and Health Survey 1994–1995 (DHS)

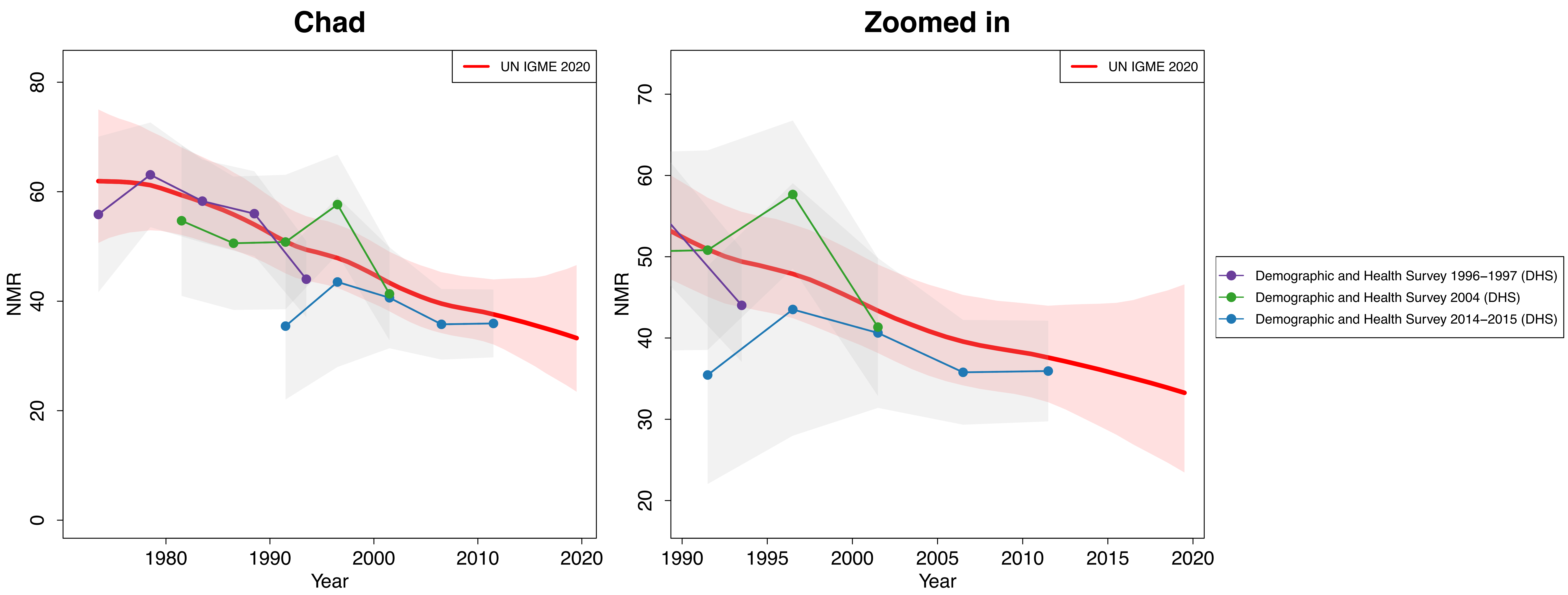

Chad
Zoomed in
UN IGME 2020
UN IGME 2020
NMR
Year
Demographic and Health Survey 1996–1997 (DHS)
Demographic and Health Survey 2004 (DHS)
Demographic and Health Survey 2014–2015 (DHS)

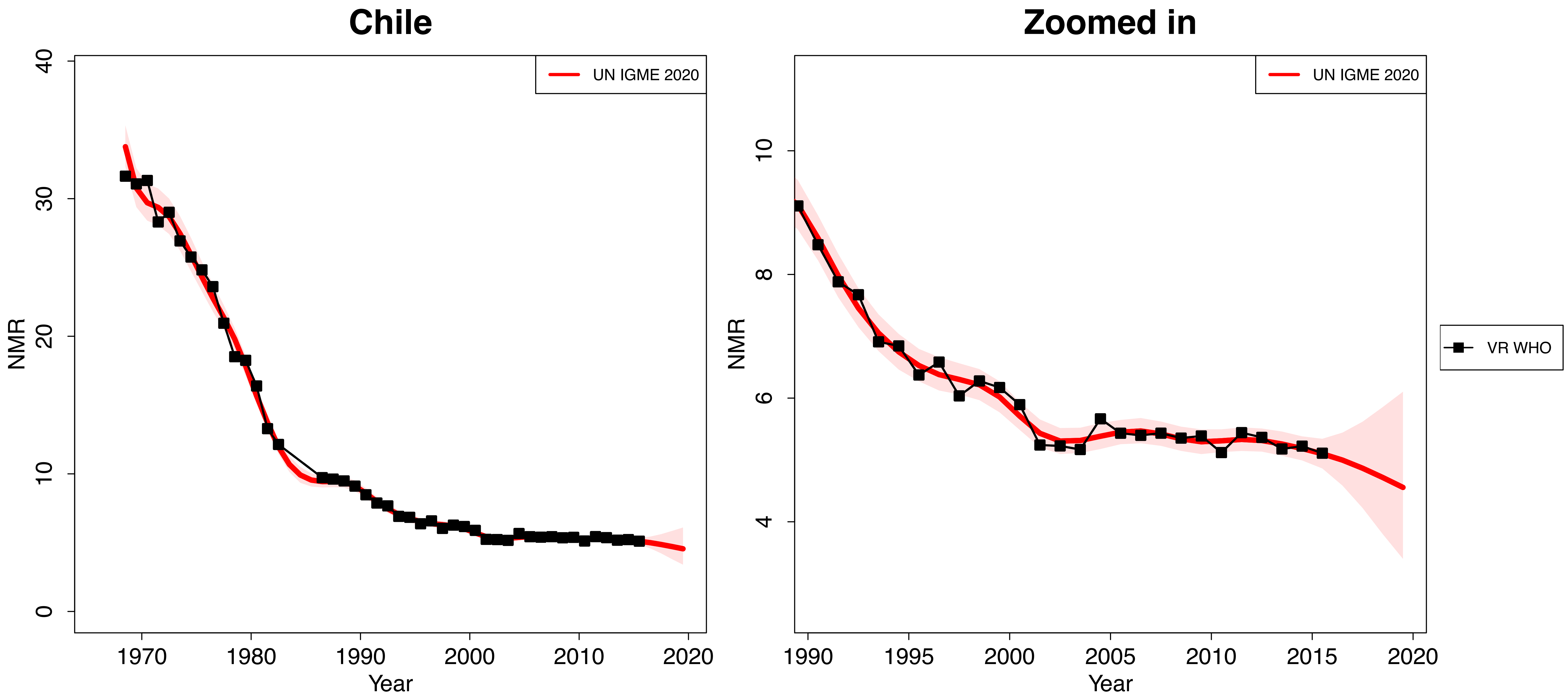

Chile
UN IGME 2020
NMR
Year
0
10
20
30
40
1970
1980
1990
2000
2010
2020
Zoomed in
UN IGME 2020
NMR
Year
4
6
8
10
1990
1995
2000
2005
2010
2015
2020
VR WHO

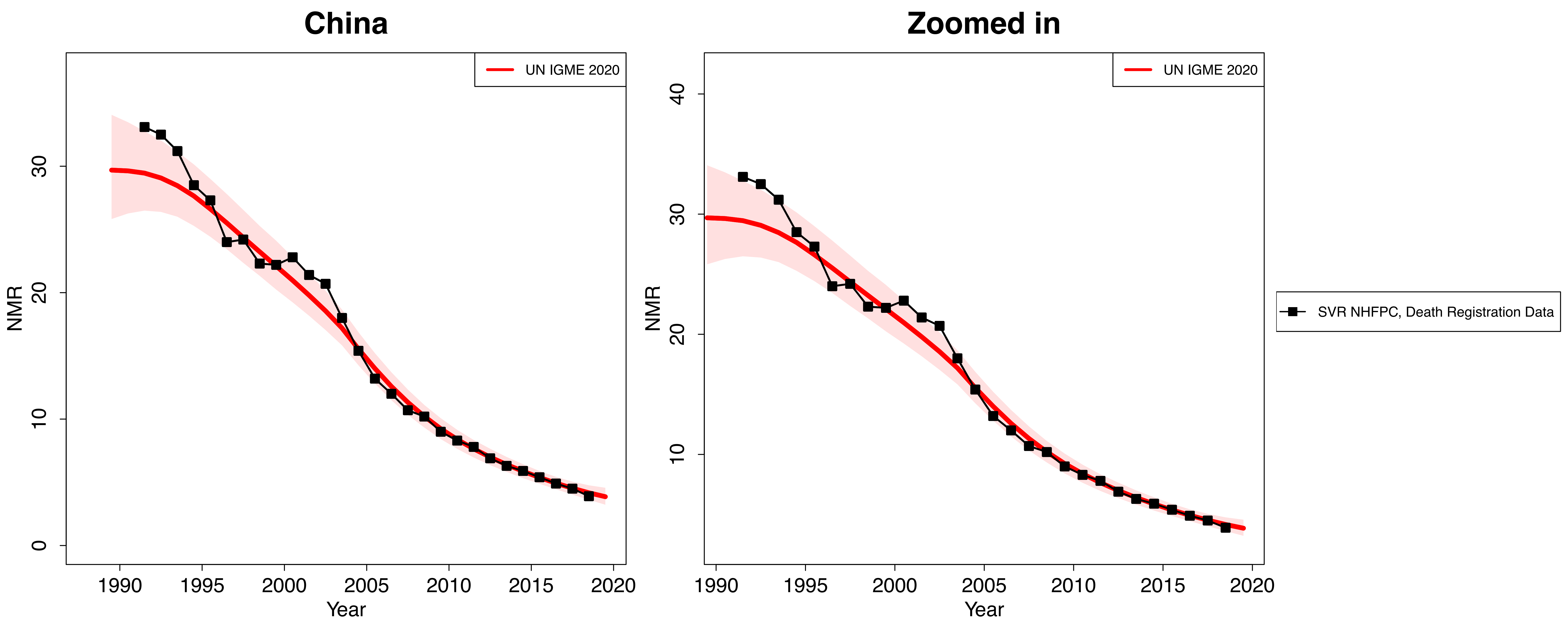
China
Zoomed in
UN IGME 2020
UN IGME 2020
SVR NHFPC, Death Registration Data
NMR
NMR
Year
Year
0
10
20
30
40
1990
1995
2000
2005
2010
2015
2020

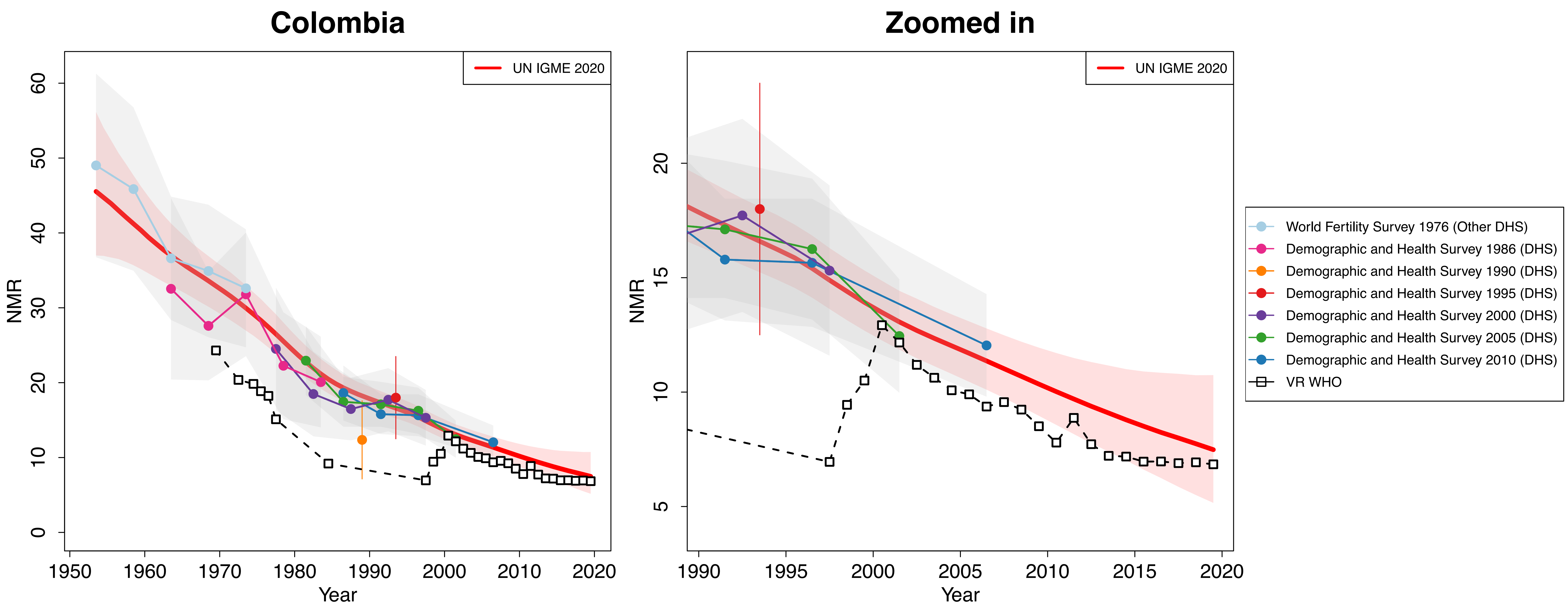

Colombia
Zoomed in
UN IGME 2020
NMR
Year
1950
1960
1970
1980
1990
2000
2010
2020
0
10
20
30
40
50
60
1995
2005
2015
5
15
World Fertility Survey 1976 (Other DHS)
Demographic and Health Survey 1986 (DHS)
Demographic and Health Survey 1990 (DHS)
Demographic and Health Survey 1995 (DHS)
Demographic and Health Survey 2000 (DHS)
Demographic and Health Survey 2005 (DHS)
Demographic and Health Survey 2010 (DHS)
VR WHO

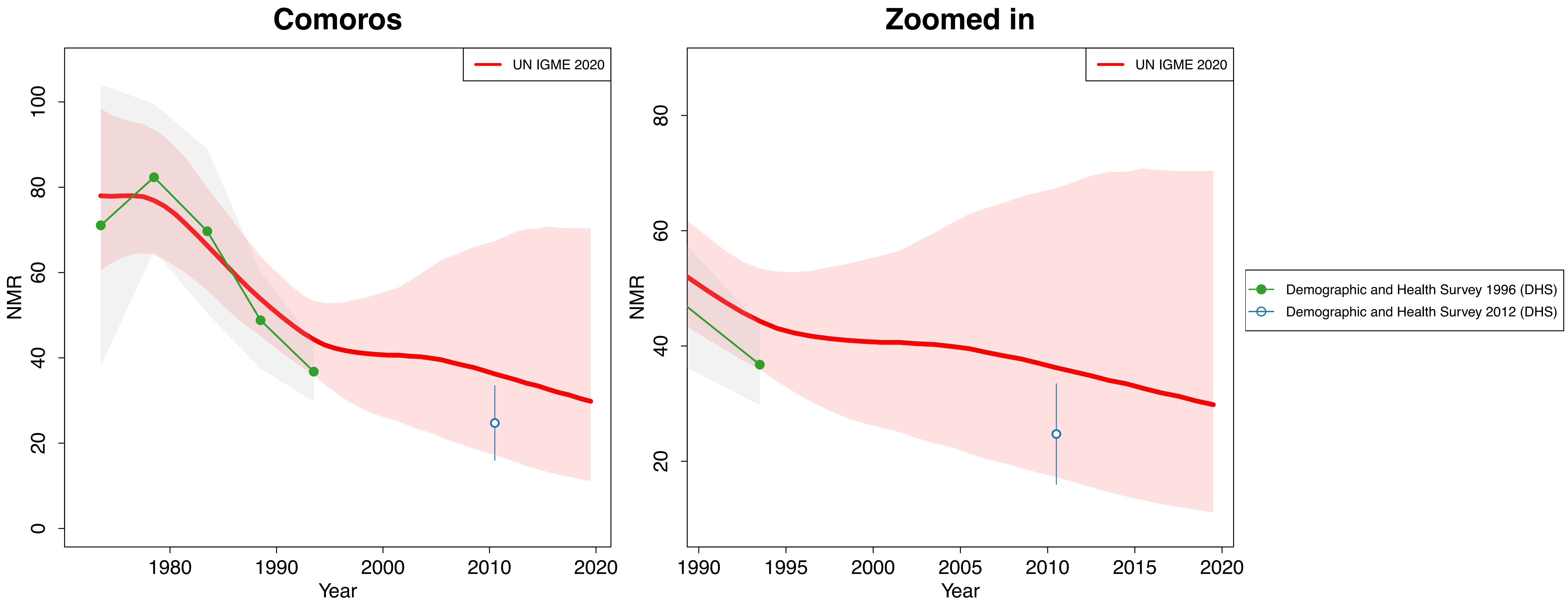

Comoros
Zoomed in
UN IGME 2020
UN IGME 2020
NMR
NMR
Year
Year
0
20
40
60
80
100
1980
1990
2000
2010
2020
20
40
60
80
1990
1995
2000
2005
2010
2015
2020
Demographic and Health Survey 1996 (DHS)
Demographic and Health Survey 2012 (DHS)

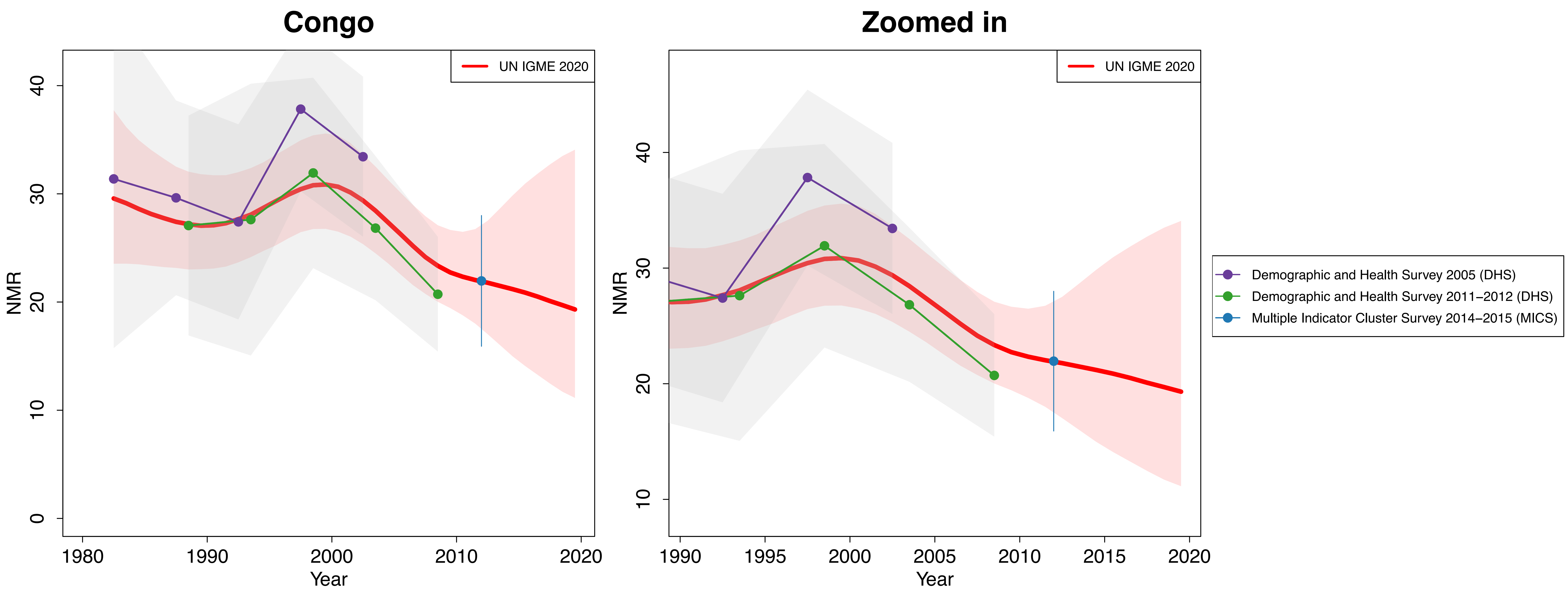

Congo
Zoomed in
UN IGME 2020
NMR
Year
0
10
20
30
40
1980
1990
2000
2010
2020
1995
2005
2015
Demographic and Health Survey 2005 (DHS)
Demographic and Health Survey 2011–2012 (DHS)
Multiple Indicator Cluster Survey 2014–2015 (MICS)

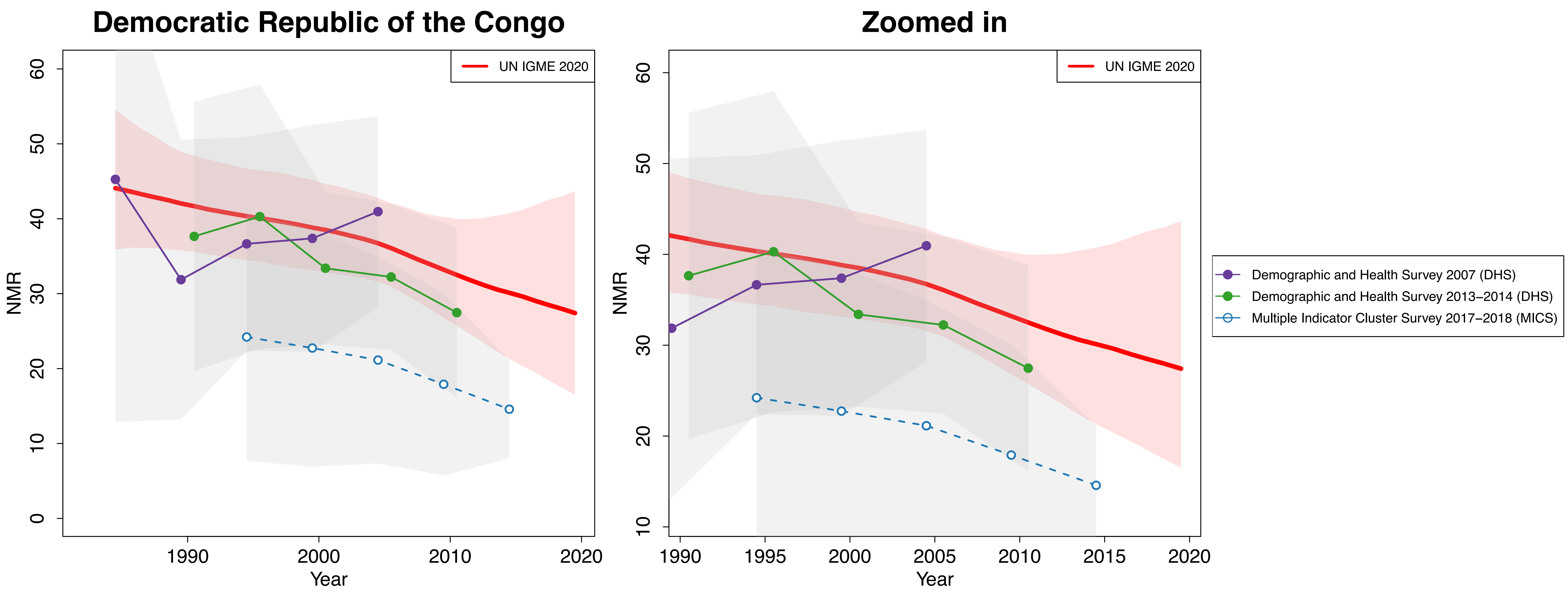

Democratic Republic of the Congo
Zoomed in
UN IGME 2020
NMR
Year
Demographic and Health Survey 2007 (DHS)
Demographic and Health Survey 2013–2014 (DHS)
Multiple Indicator Cluster Survey 2017–2018 (MICS)

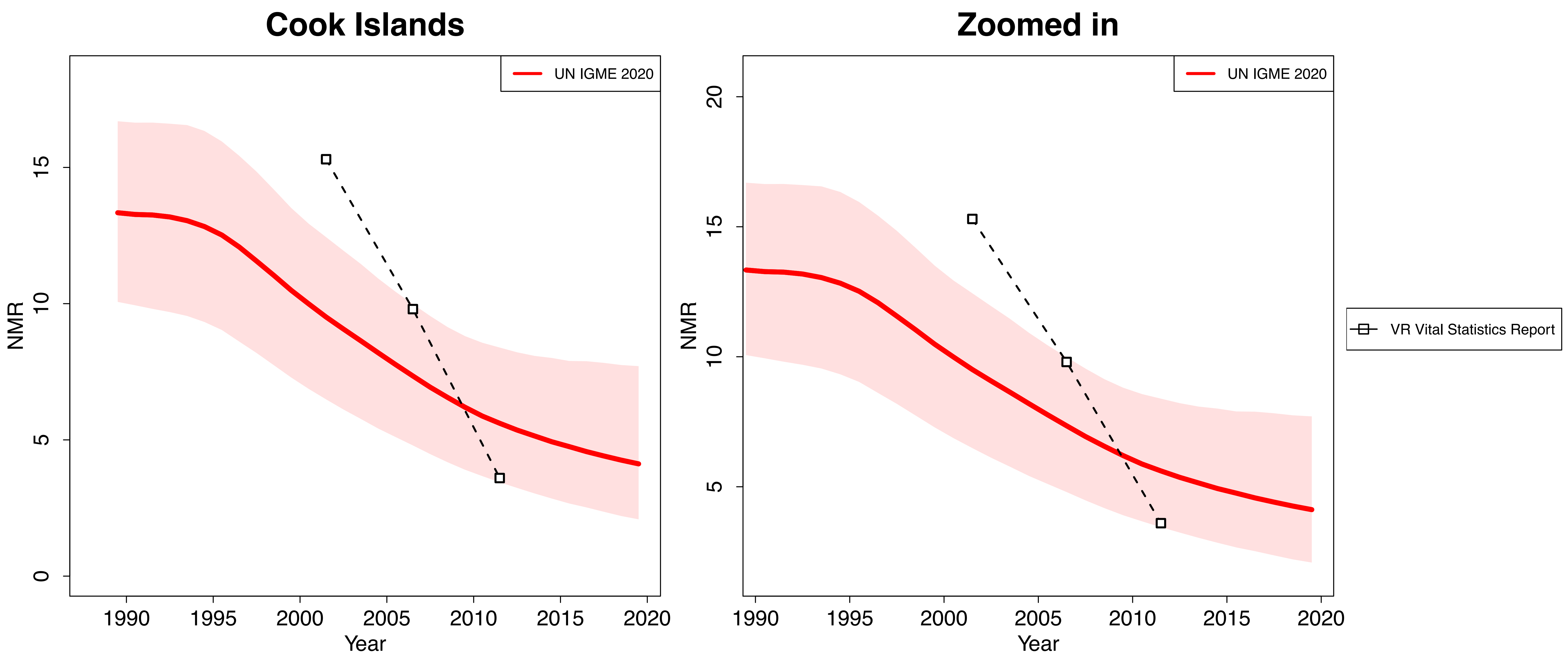

Cook Islands
Zoomed in
UN IGME 2020
VR Vital Statistics Report
NMR
Year
0
5
10
15
20
1990
1995
2000
2005
2010
2015
2020

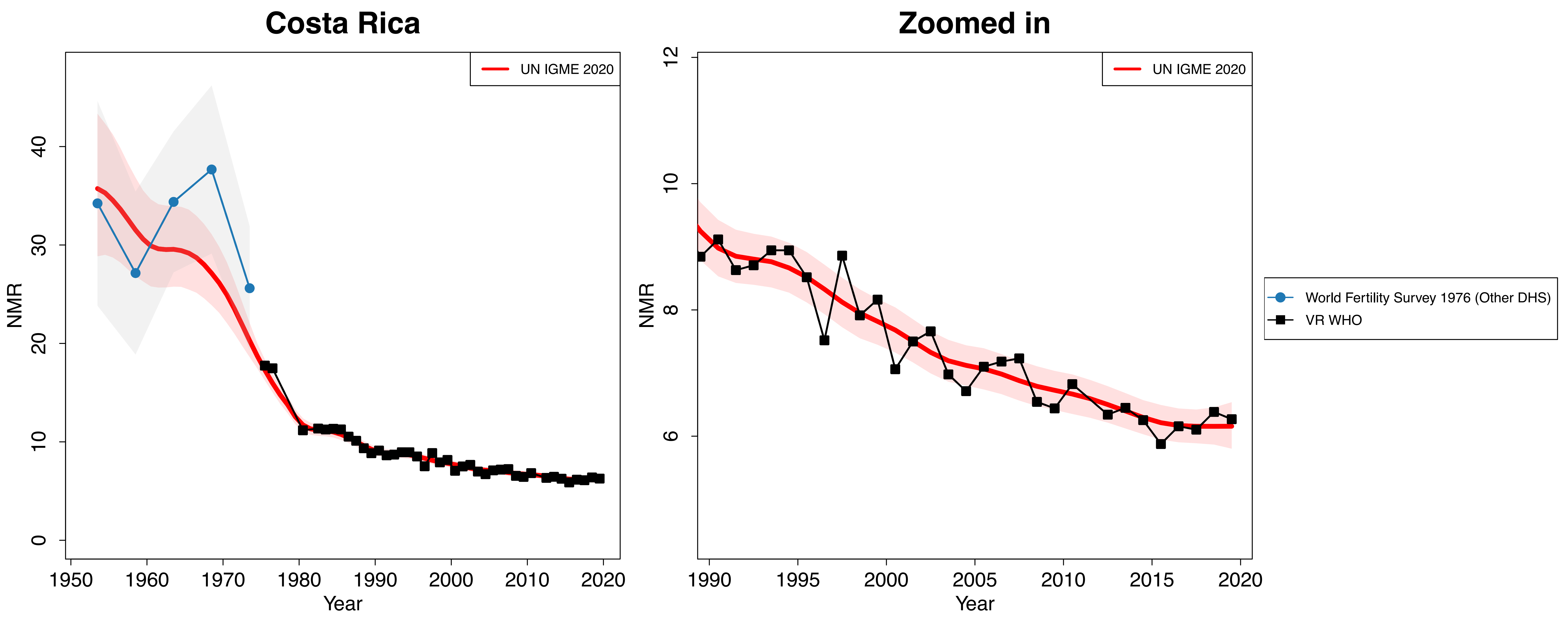

Costa Rica
Zoomed in
UN IGME 2020
NMR
Year
World Fertility Survey 1976 (Other DHS)
VR WHO

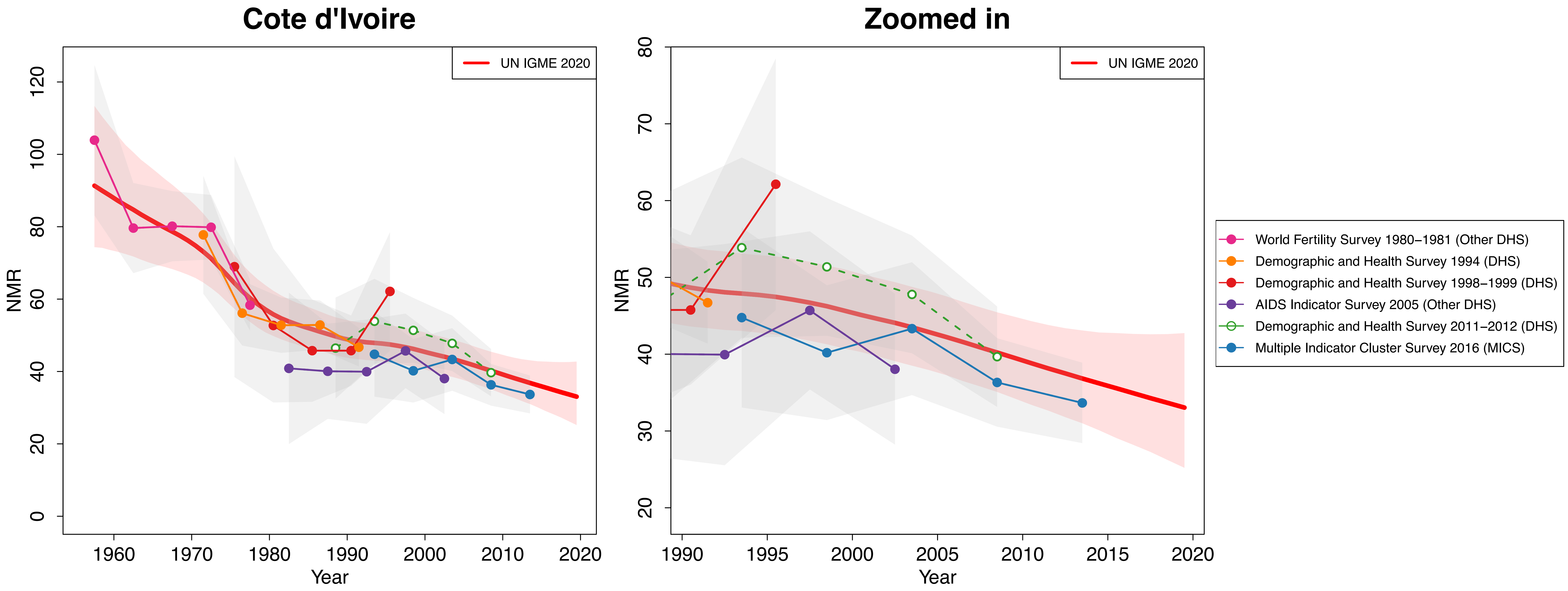

Cote d'Ivoire
Zoomed in
UN IGME 2020
NMR
Year
World Fertility Survey 1980–1981 (Other DHS)
Demographic and Health Survey 1994 (DHS)
Demographic and Health Survey 1998–1999 (DHS)
AIDS Indicator Survey 2005 (Other DHS)
Demographic and Health Survey 2011–2012 (DHS)
Multiple Indicator Cluster Survey 2016 (MICS)

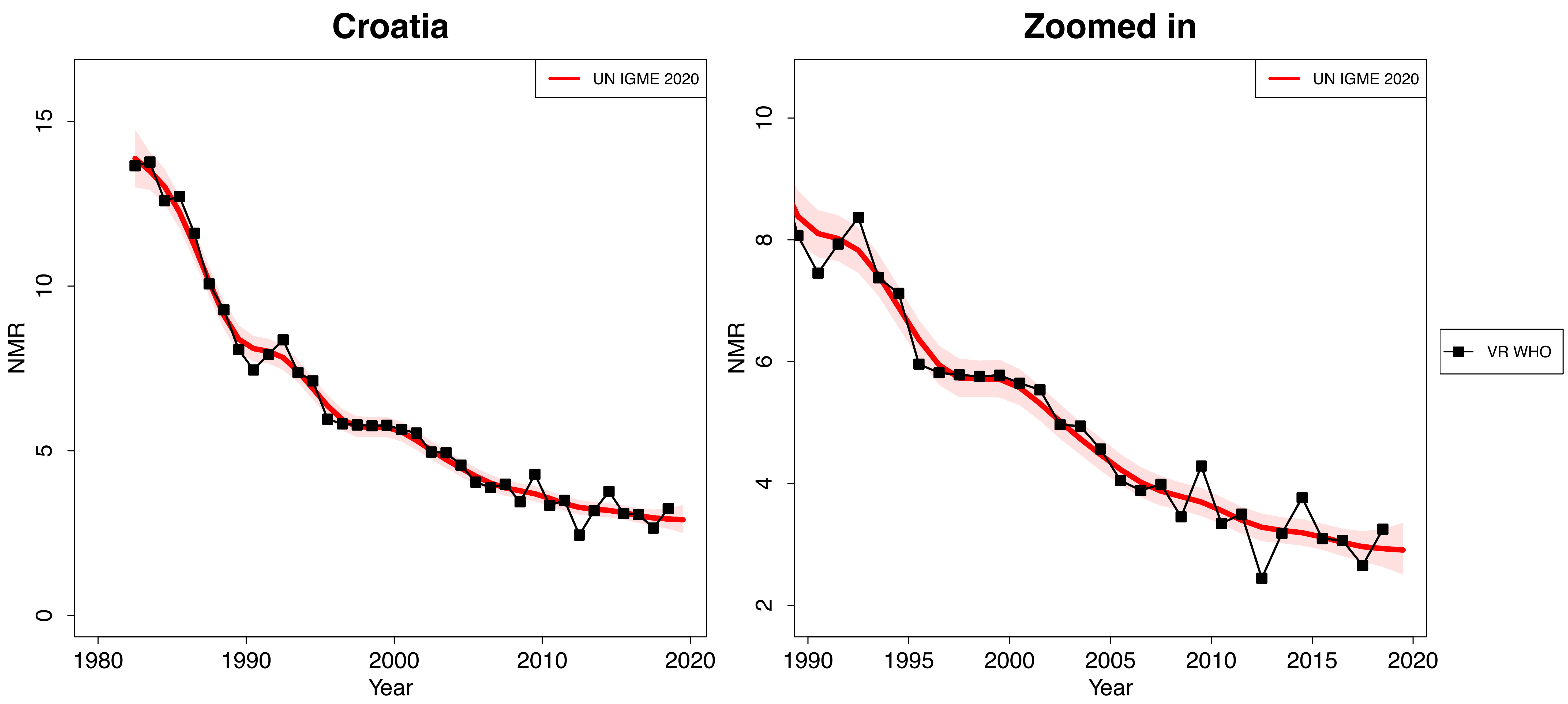

Croatia
Zoomed in
UN IGME 2020
UN IGME 2020
VR WHO
NMR
NMR
Year
Year
0
5
10
15
2
4
6
8
10
1980
1990
2000
2010
2020
1990
1995
2000
2005
2010
2015
2020

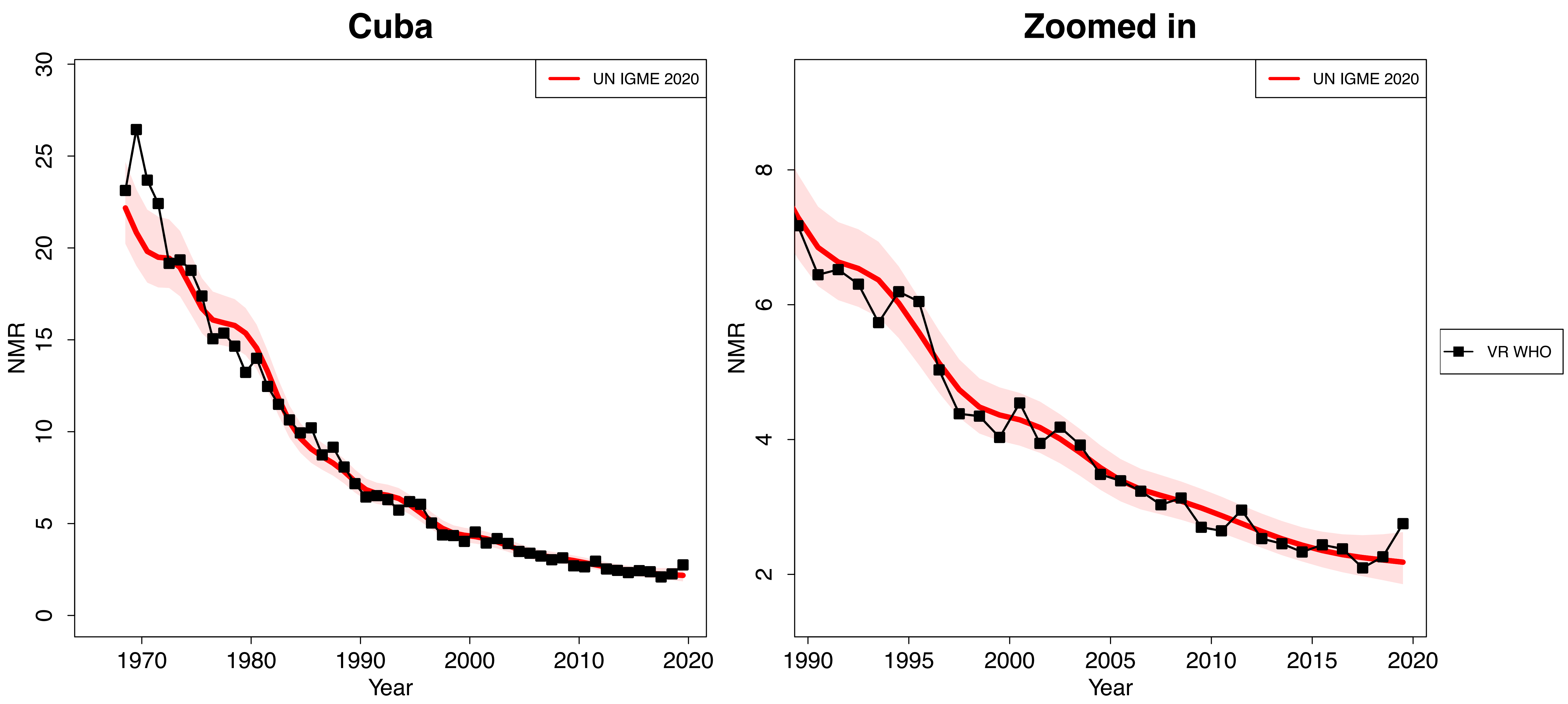

Cuba
Zoomed in
UN IGME 2020
UN IGME 2020
VR WHO
NMR
NMR
Year
Year
0
5
10
15
20
25
30
1970
1980
1990
2000
2010
2020
2
4
6
8
1990
1995
2000
2005
2010
2015
2020

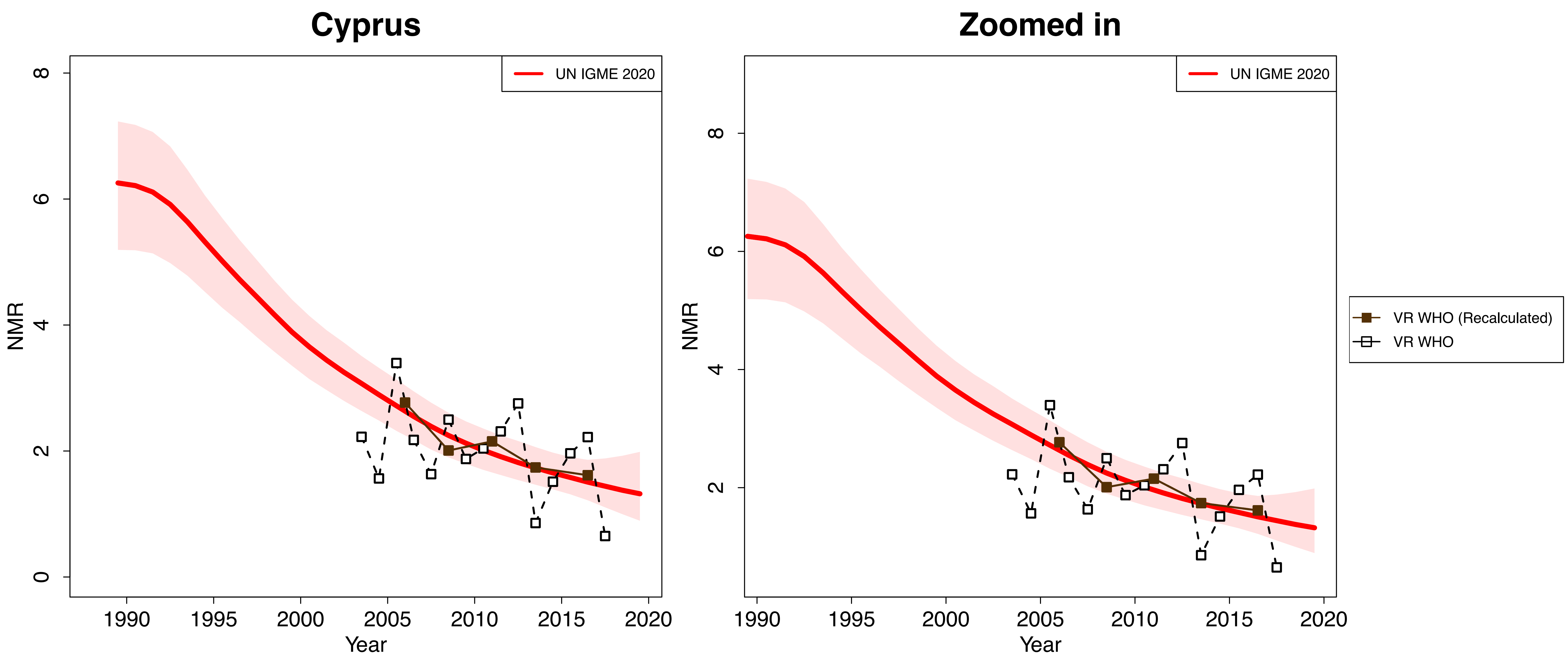

Cyprus
Zoomed in
UN IGME 2020
UN IGME 2020
VR WHO (Recalculated)
VR WHO
NMR
NMR
Year
Year
0
2
4
6
8
2
4
6
8
1990
1995
2000
2005
2010
2015
2020
1990
1995
2000
2005
2010
2015
2020

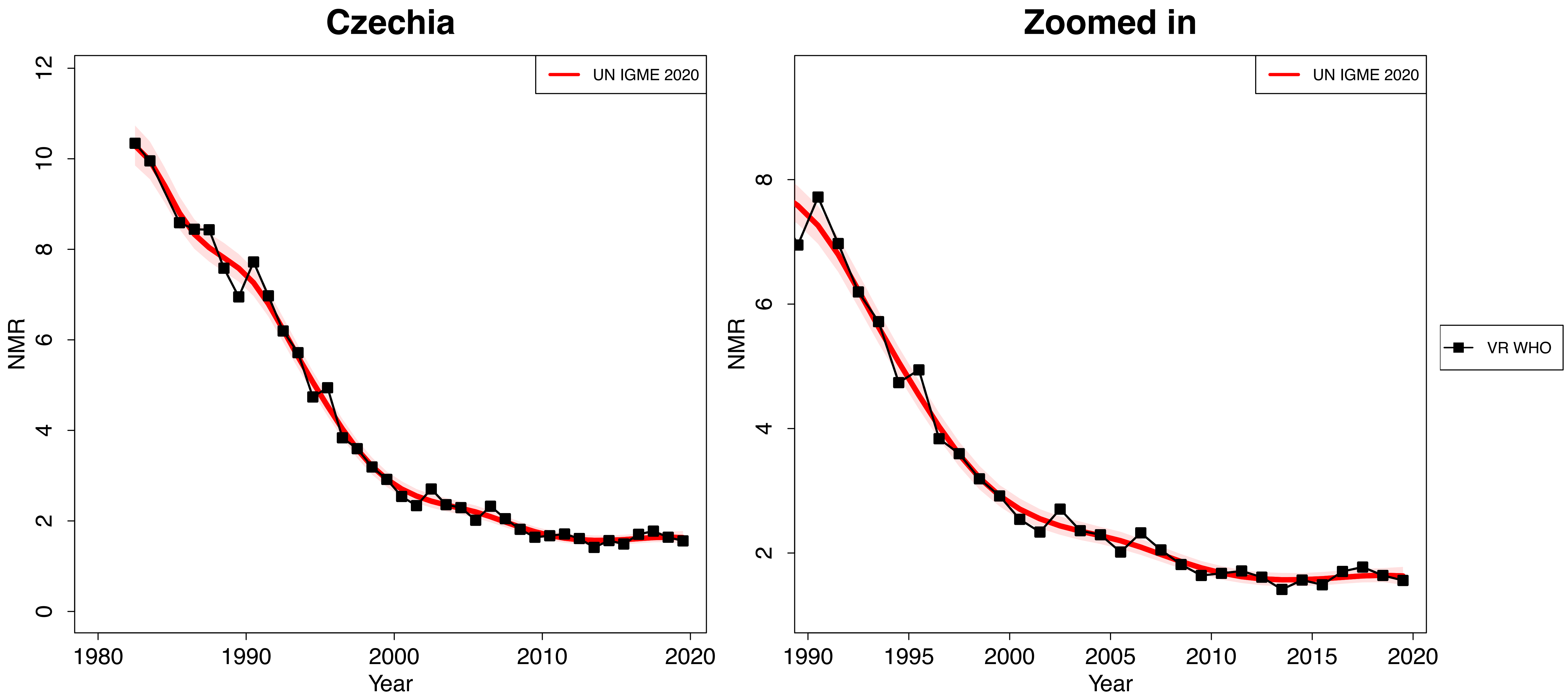

Czechia
Zoomed in
UN IGME 2020
UN IGME 2020
VR WHO
NMR
NMR
Year
Year
0
2
4
6
8
10
12
2
4
6
8
1980
1990
2000
2010
2020
1990
1995
2000
2005
2010
2015
2020

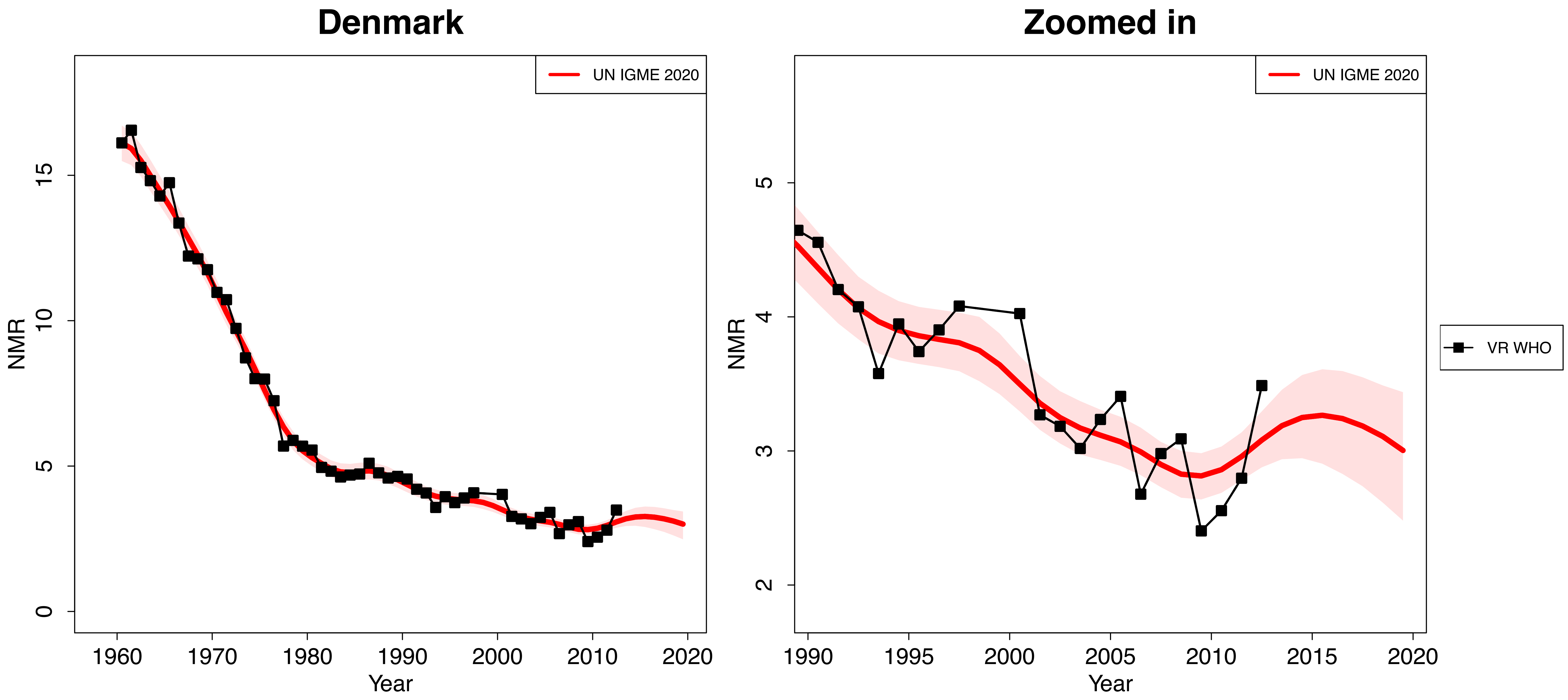
Denmark
Zoomed in
UN IGME 2020
UN IGME 2020
VR WHO
NMR
NMR
Year
Year
0
5
10
15
2
3
4
5
1960
1970
1980
1990
2000
2010
2020
1990
1995
2000
2005
2010
2015
2020

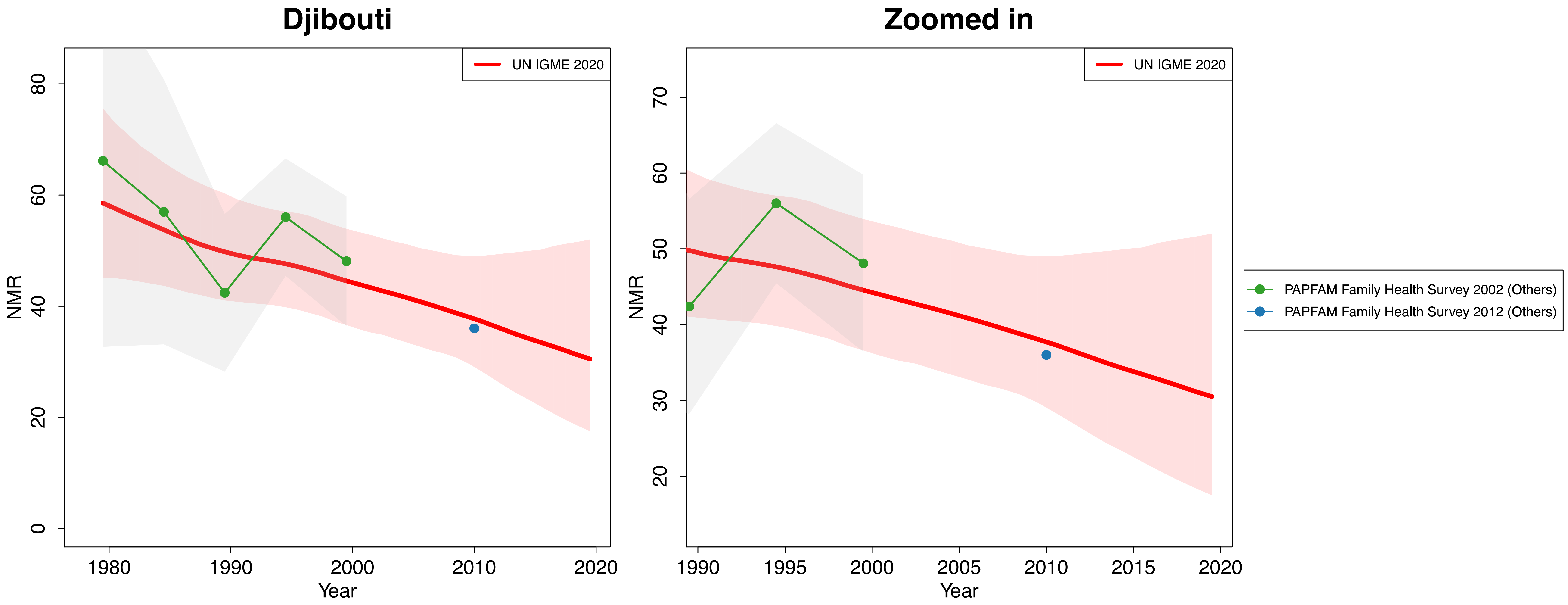

Djibouti
Zoomed in
UN IGME 2020
UN IGME 2020
NMR
Year
0
20
40
60
80
1980
1990
2000
2010
2020
20
30
40
50
60
70
1990
1995
2000
2005
2010
2015
2020
PAPFAM Family Health Survey 2002 (Others)
PAPFAM Family Health Survey 2012 (Others)

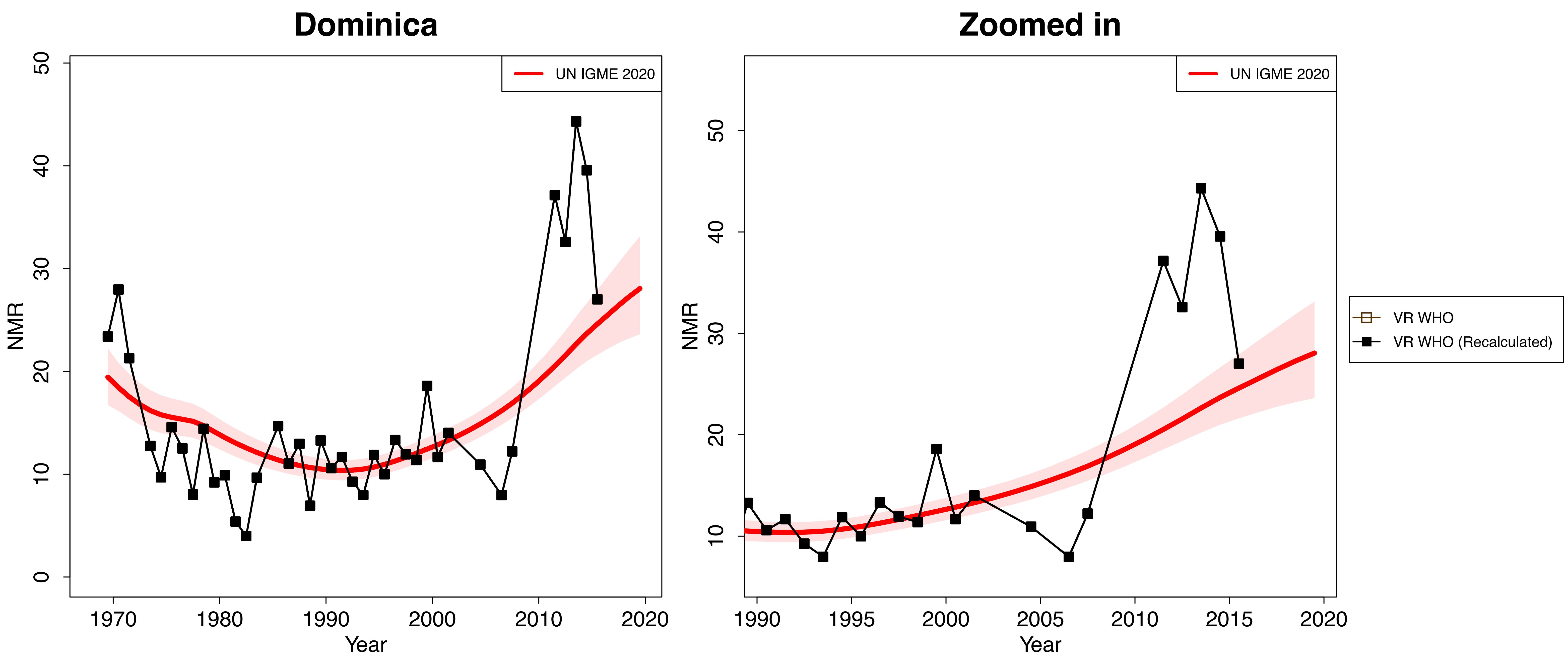

Dominica
Zoomed in
UN IGME 2020
UN IGME 2020
NMR
NMR
Year
Year
0
10
20
30
40
50
1970
1980
1990
2000
2010
2020
1990
1995
2000
2005
2010
2015
2020
VR WHO
VR WHO (Recalculated)

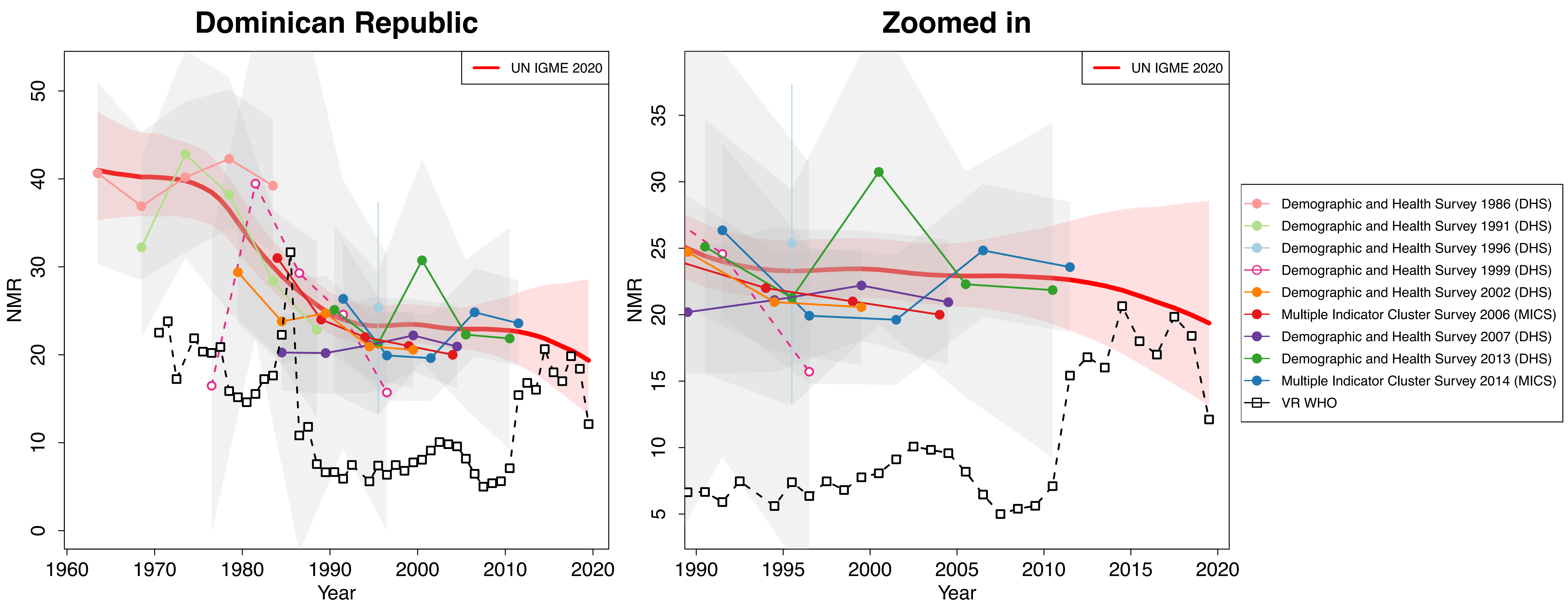

Dominican Republic
Zoomed in
UN IGME 2020
NMR
Year
Demographic and Health Survey 1986 (DHS)
Demographic and Health Survey 1991 (DHS)
Demographic and Health Survey 1996 (DHS)
Demographic and Health Survey 1999 (DHS)
Demographic and Health Survey 2002 (DHS)
Multiple Indicator Cluster Survey 2006 (MICS)
Demographic and Health Survey 2007 (DHS)
Demographic and Health Survey 2013 (DHS)
Multiple Indicator Cluster Survey 2014 (MICS)
VR WHO

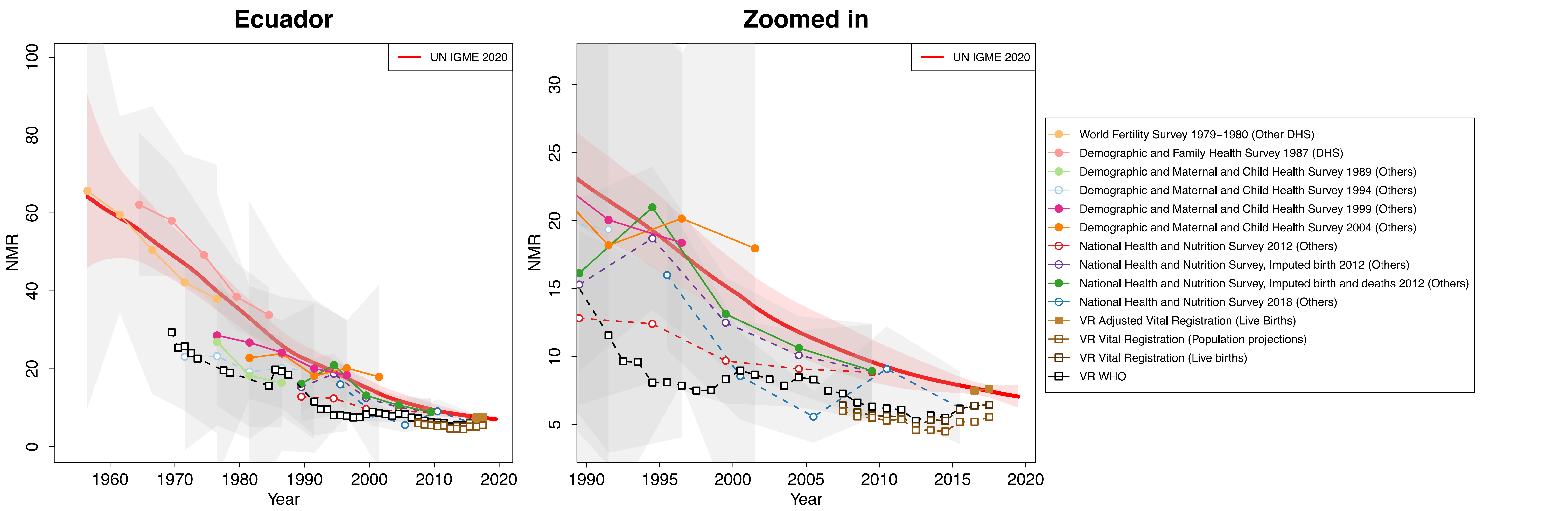

Ecuador
Zoomed in
UN IGME 2020
NMR
Year
World Fertility Survey 1979–1980 (Other DHS)
Demographic and Family Health Survey 1987 (DHS)
Demographic and Maternal and Child Health Survey 1989 (Others)
Demographic and Maternal and Child Health Survey 1994 (Others)
Demographic and Maternal and Child Health Survey 1999 (Others)
Demographic and Maternal and Child Health Survey 2004 (Others)
National Health and Nutrition Survey 2012 (Others)
National Health and Nutrition Survey, Imputed birth 2012 (Others)
National Health and Nutrition Survey, Imputed birth and deaths 2012 (Others)
National Health and Nutrition Survey 2018 (Others)
VR Adjusted Vital Registration (Live Births)
VR Vital Registration (Population projections)
VR Vital Registration (Live births)
VR WHO

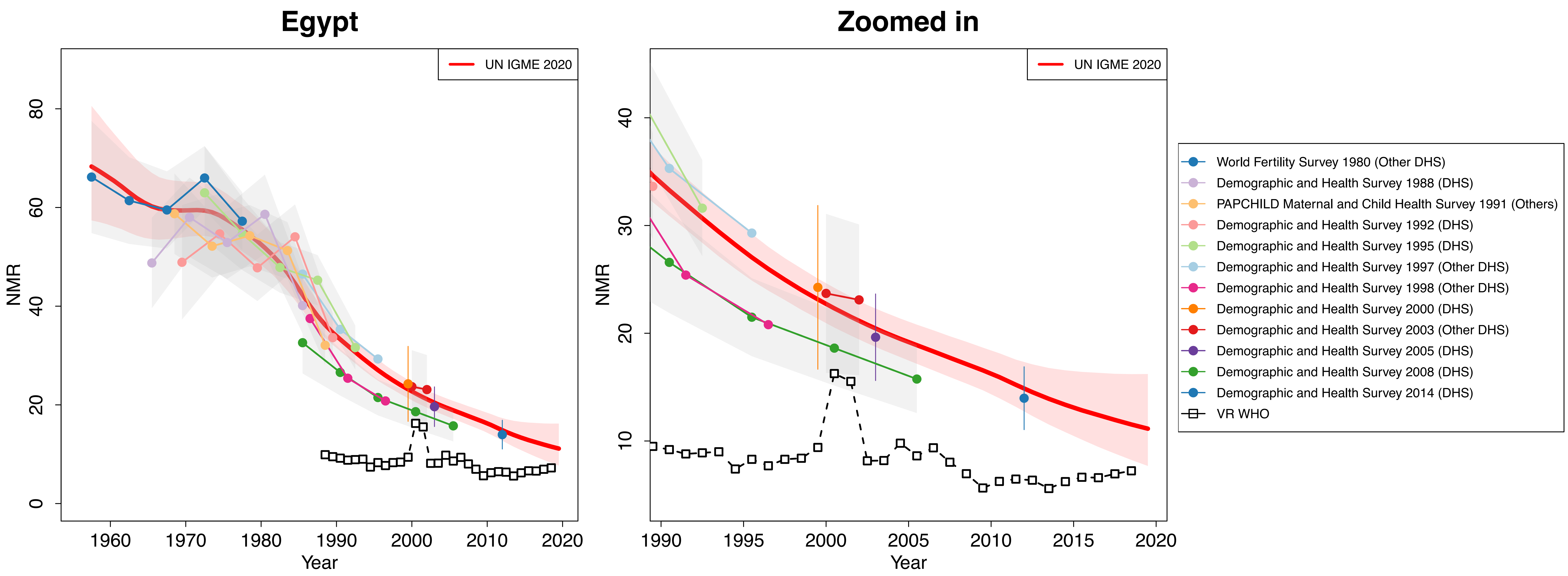

Egypt
Zoomed in
UN IGME 2020
NMR
Year
World Fertility Survey 1980 (Other DHS)
Demographic and Health Survey 1988 (DHS)
PAPCHILD Maternal and Child Health Survey 1991 (Others)
Demographic and Health Survey 1992 (DHS)
Demographic and Health Survey 1995 (DHS)
Demographic and Health Survey 1997 (Other DHS)
Demographic and Health Survey 1998 (Other DHS)
Demographic and Health Survey 2000 (DHS)
Demographic and Health Survey 2003 (Other DHS)
Demographic and Health Survey 2005 (DHS)
Demographic and Health Survey 2008 (DHS)
Demographic and Health Survey 2014 (DHS)
VR WHO

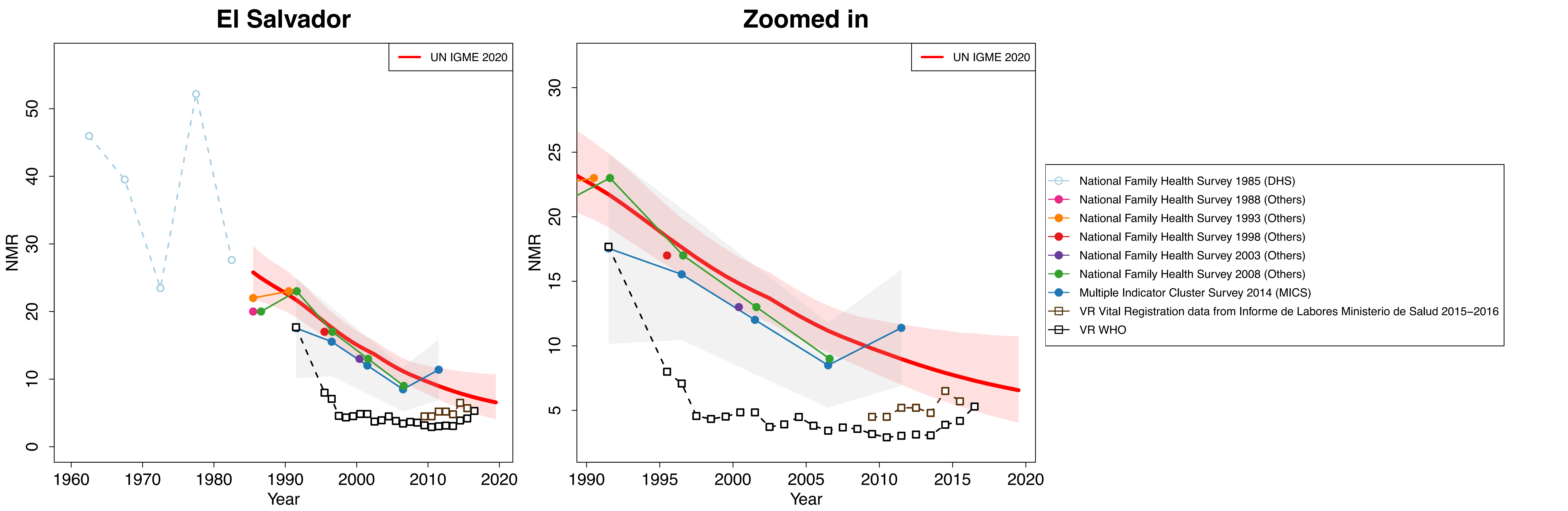

El Salvador
Zoomed in
UN IGME 2020
NMR
Year
National Family Health Survey 1985 (DHS)
National Family Health Survey 1988 (Others)
National Family Health Survey 1993 (Others)
National Family Health Survey 1998 (Others)
National Family Health Survey 2003 (Others)
National Family Health Survey 2008 (Others)
Multiple Indicator Cluster Survey 2014 (MICS)
VR Vital Registration data from Informe de Labores Ministerio de Salud 2015–2016
VR WHO

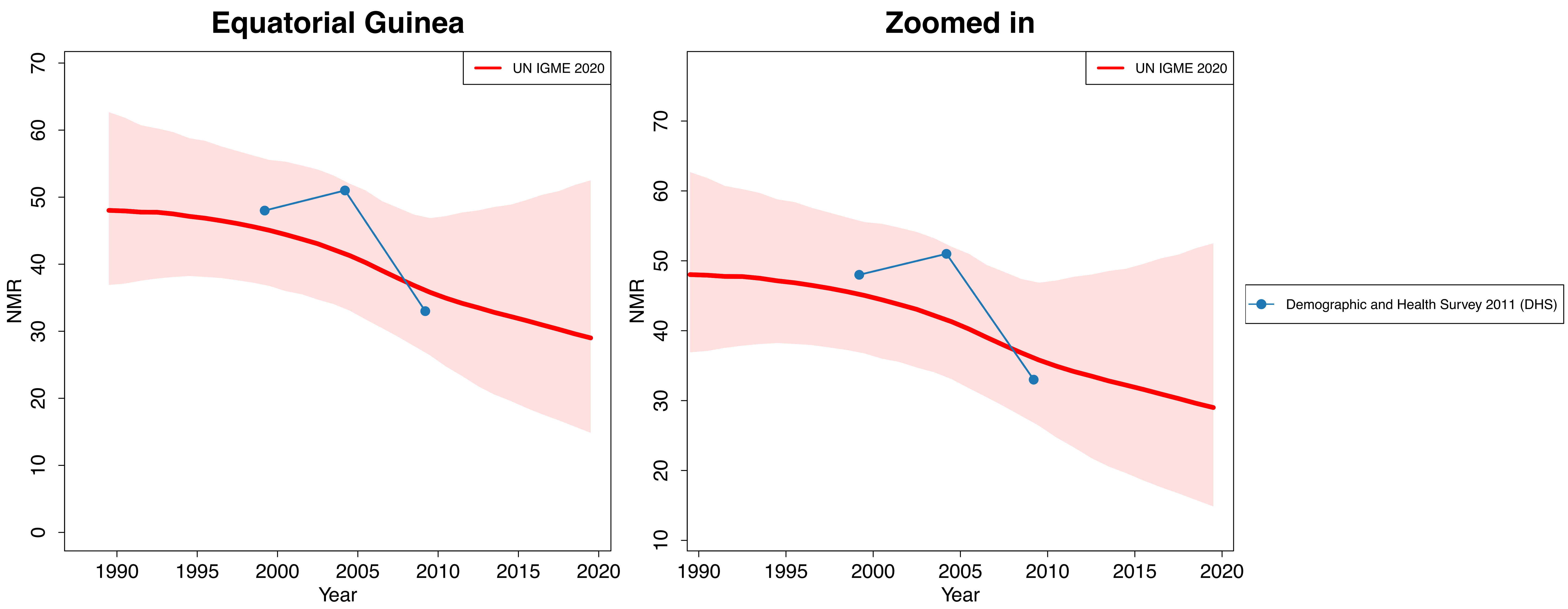

Equatorial Guinea
Zoomed in
UN IGME 2020
Demographic and Health Survey 2011 (DHS)
NMR
Year
0
10
20
30
40
50
60
70
1990
1995
2000
2005
2010
2015
2020

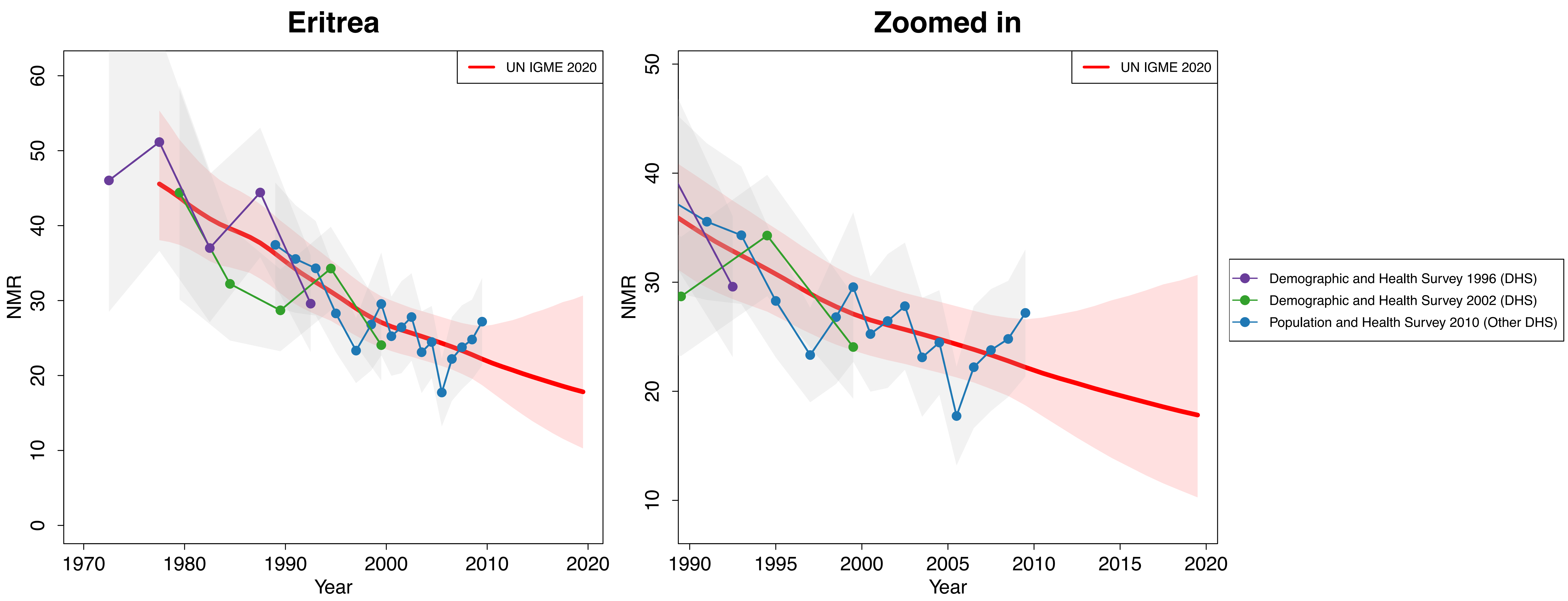

Eritrea
Zoomed in
UN IGME 2020
UN IGME 2020
NMR
NMR
Year
Year
0
10
20
30
40
50
60
10
20
30
40
50
1970
1980
1990
2000
2010
2020
1990
1995
2000
2005
2010
2015
2020
Demographic and Health Survey 1996 (DHS)
Demographic and Health Survey 2002 (DHS)
Population and Health Survey 2010 (Other DHS)

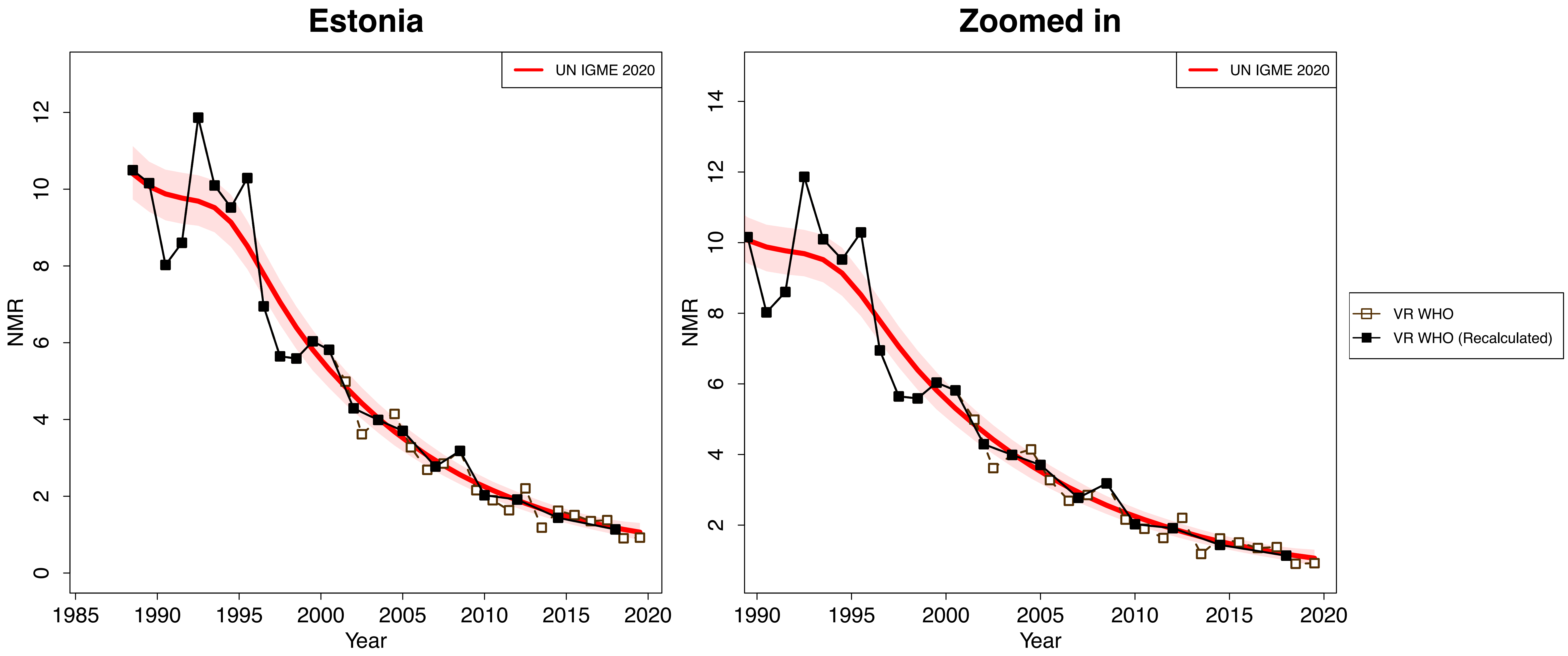
Estonia
Zoomed in
UN IGME 2020
UN IGME 2020
NMR
NMR
Year
Year
VR WHO
VR WHO (Recalculated)
0
2
4
6
8
10
12
14
1985
1990
1995
2000
2005
2010
2015
2020

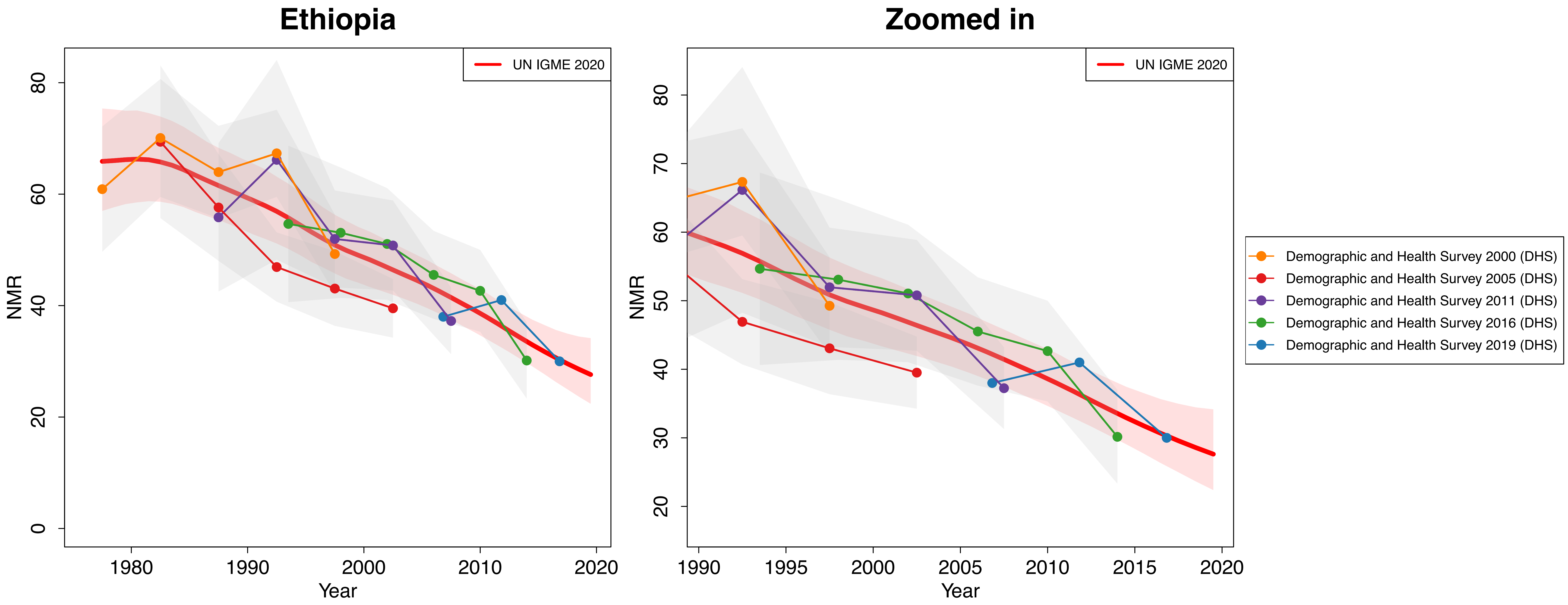

Ethiopia
Zoomed in
UN IGME 2020
NMR
Year
Demographic and Health Survey 2000 (DHS)
Demographic and Health Survey 2005 (DHS)
Demographic and Health Survey 2011 (DHS)
Demographic and Health Survey 2016 (DHS)
Demographic and Health Survey 2019 (DHS)

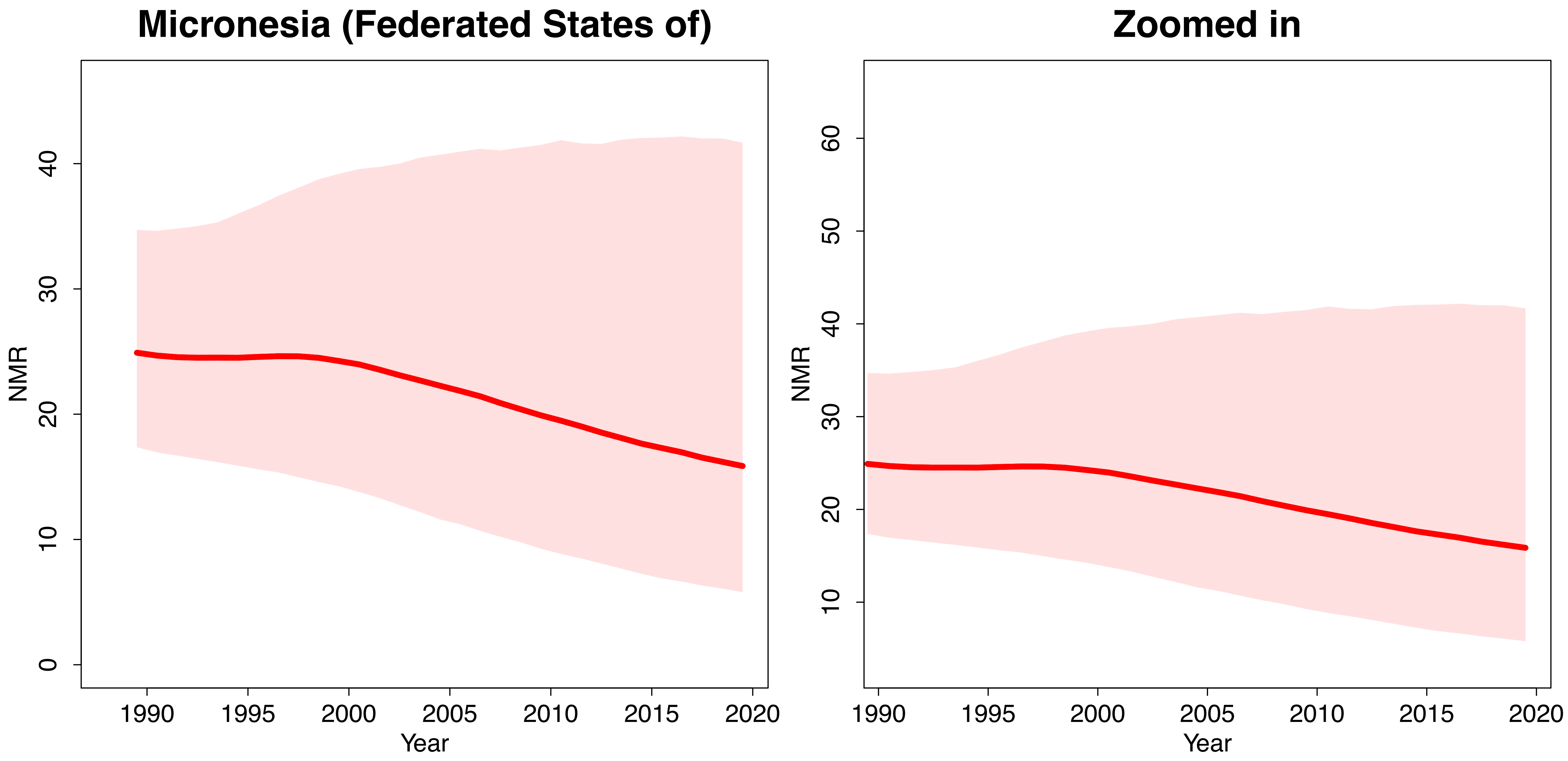

Micronesia (Federated States of)
Zoomed in
NMR
Year
0
10
20
30
40
50
60
1990
1995
2000
2005
2010
2015
2020

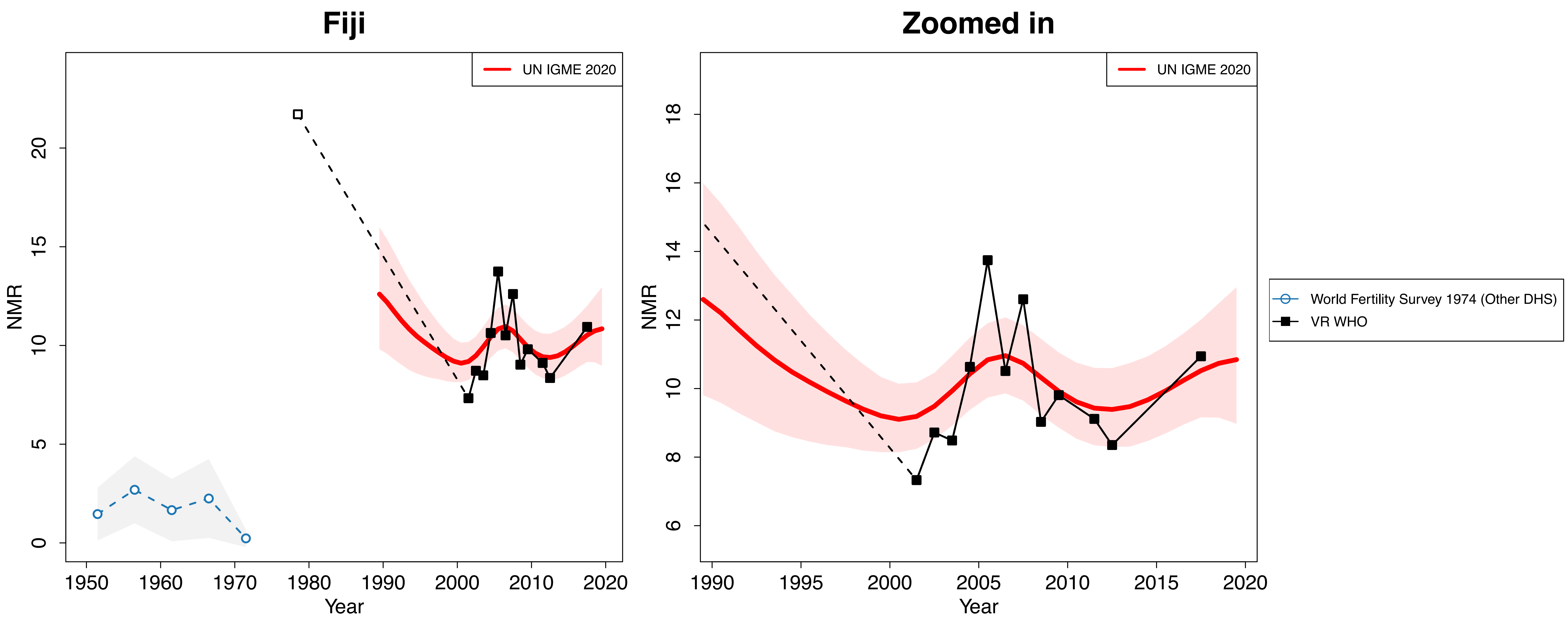

Fiji
Zoomed in
UN IGME 2020
UN IGME 2020
World Fertility Survey 1974 (Other DHS)
VR WHO
NMR
Year

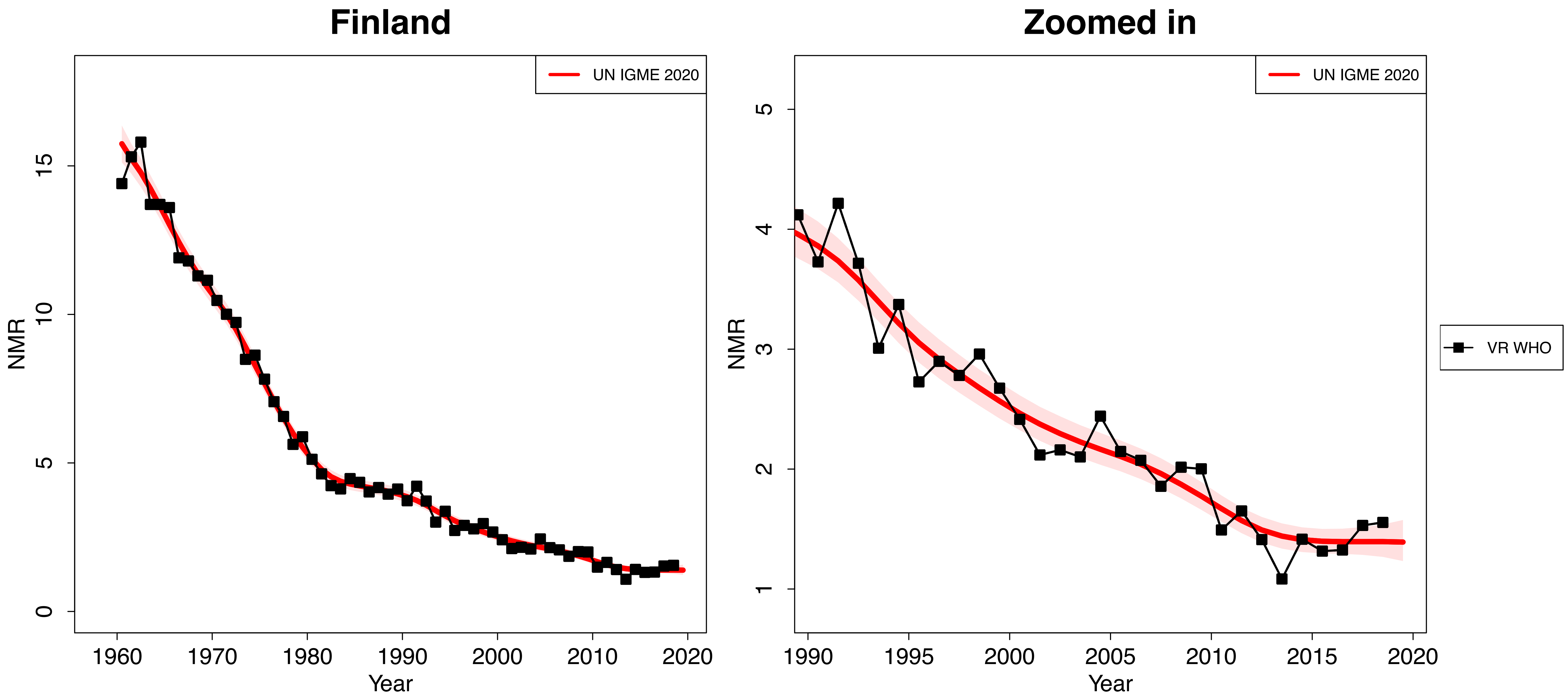

Finland
Zoomed in
UN IGME 2020
UN IGME 2020
VR WHO
NMR
NMR
Year
Year
0
5
10
15
1
2
3
4
5
1960
1970
1980
1990
2000
2010
2020
1990
1995
2000
2005
2010
2015
2020

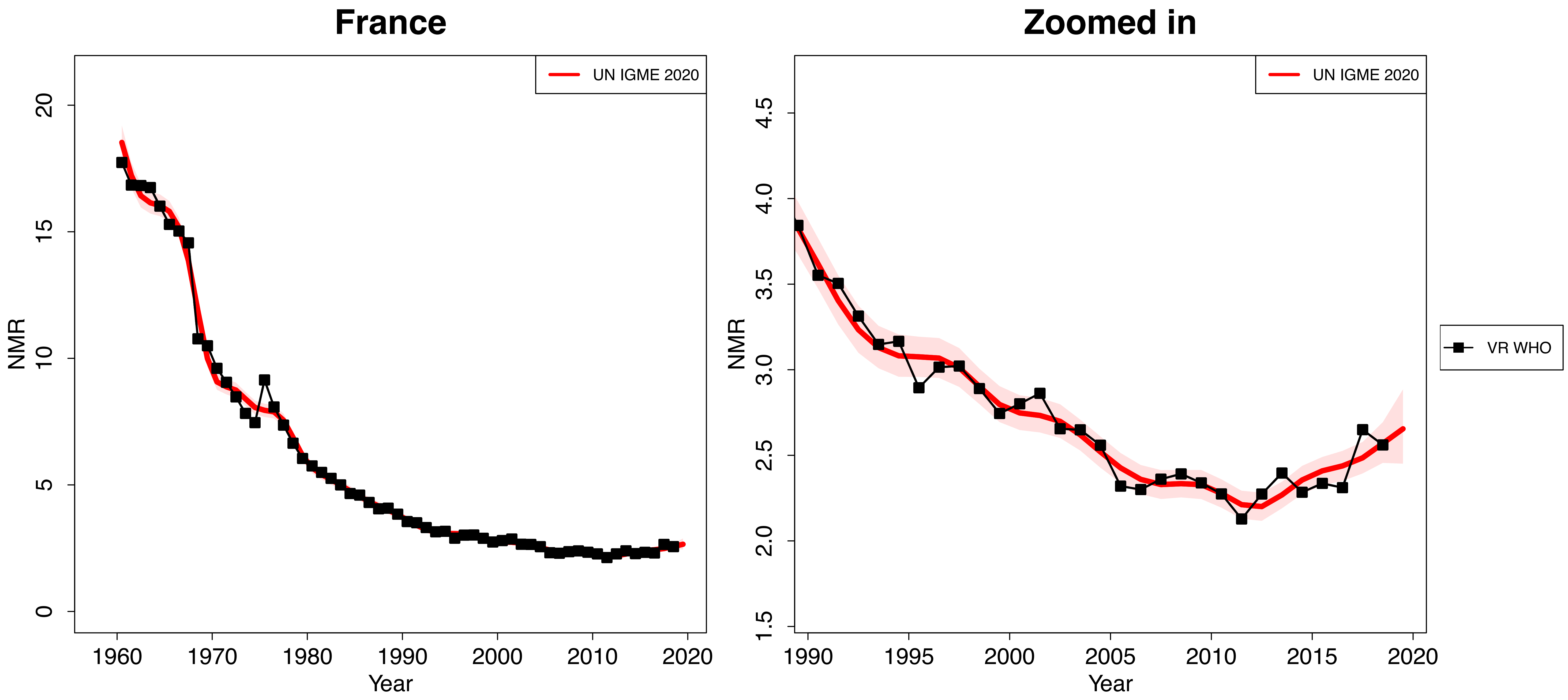

France
Zoomed in
UN IGME 2020
UN IGME 2020
VR WHO
NMR
NMR
Year
Year
0
5
10
15
20
1.5
2.0
2.5
3.0
3.5
4.0
4.5
1960
1970
1980
1990
2000
2010
2020
1990
1995
2000
2005
2010
2015
2020

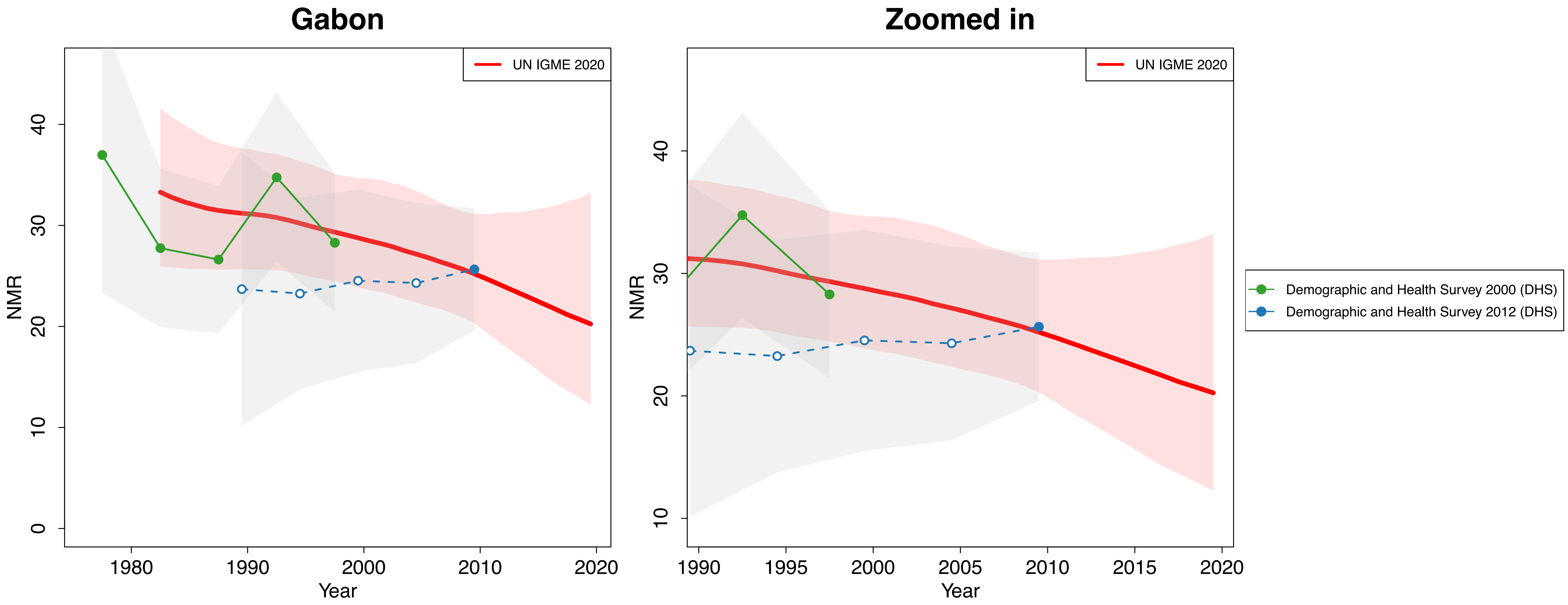

Gabon
Zoomed in
UN IGME 2020
NMR
Year
0
10
20
30
40
1980
1990
2000
2010
2020
1995
2005
2015
Demographic and Health Survey 2000 (DHS)
Demographic and Health Survey 2012 (DHS)

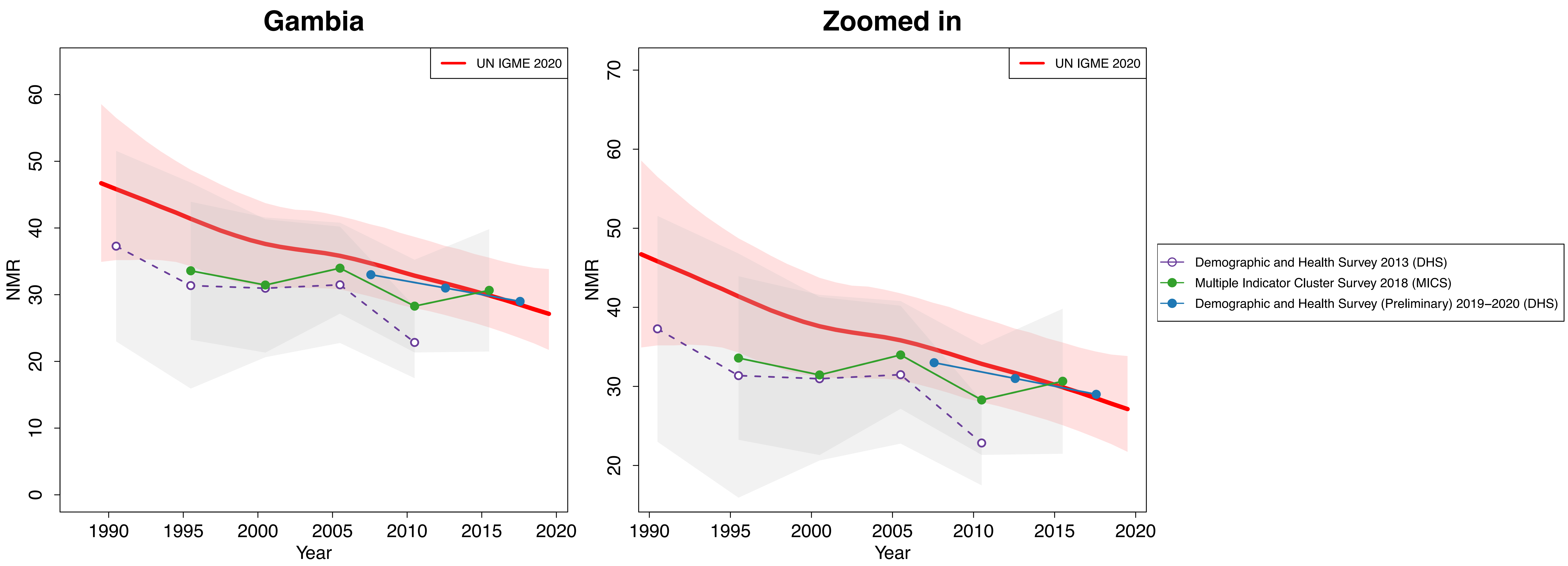

Gambia
Zoomed in
UN IGME 2020
UN IGME 2020
NMR
NMR
Year
Year
0
10
20
30
40
50
60
70
1990
1995
2000
2005
2010
2015
2020
Demographic and Health Survey 2013 (DHS)
Multiple Indicator Cluster Survey 2018 (MICS)
Demographic and Health Survey (Preliminary) 2019–2020 (DHS)

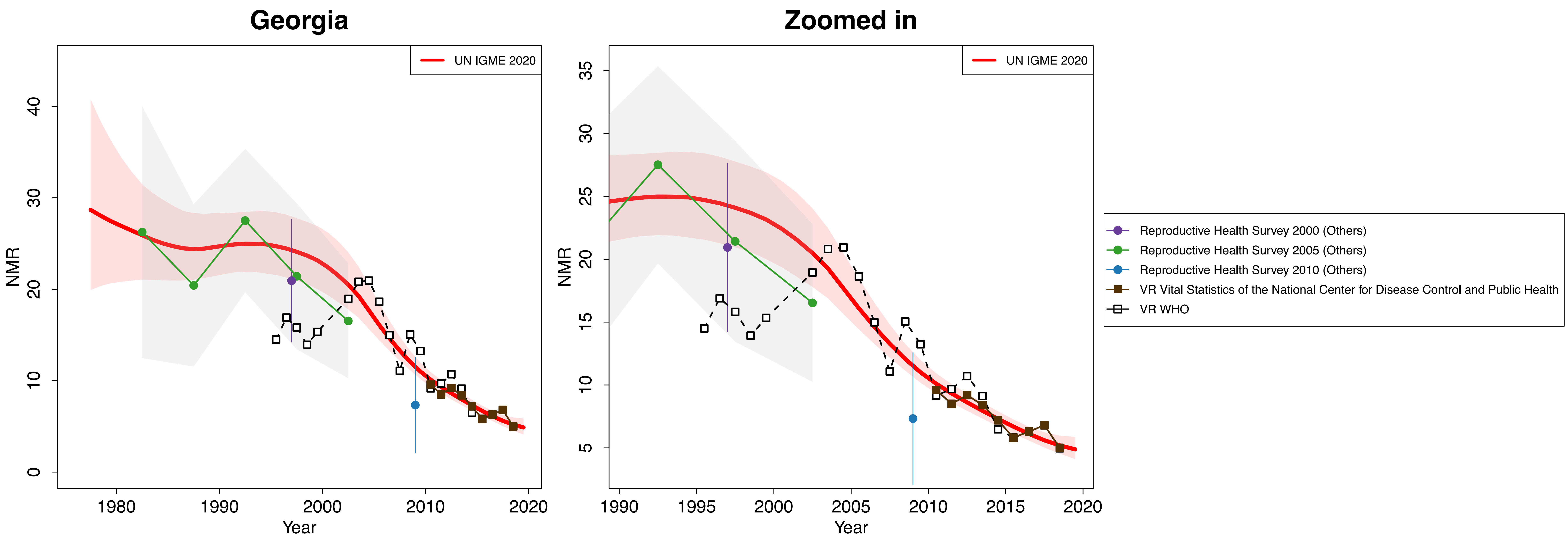
Georgia
Zoomed in
UN IGME 2020
NMR
Year
Reproductive Health Survey 2000 (Others)
Reproductive Health Survey 2005 (Others)
Reproductive Health Survey 2010 (Others)
VR Vital Statistics of the National Center for Disease Control and Public Health
VR WHO

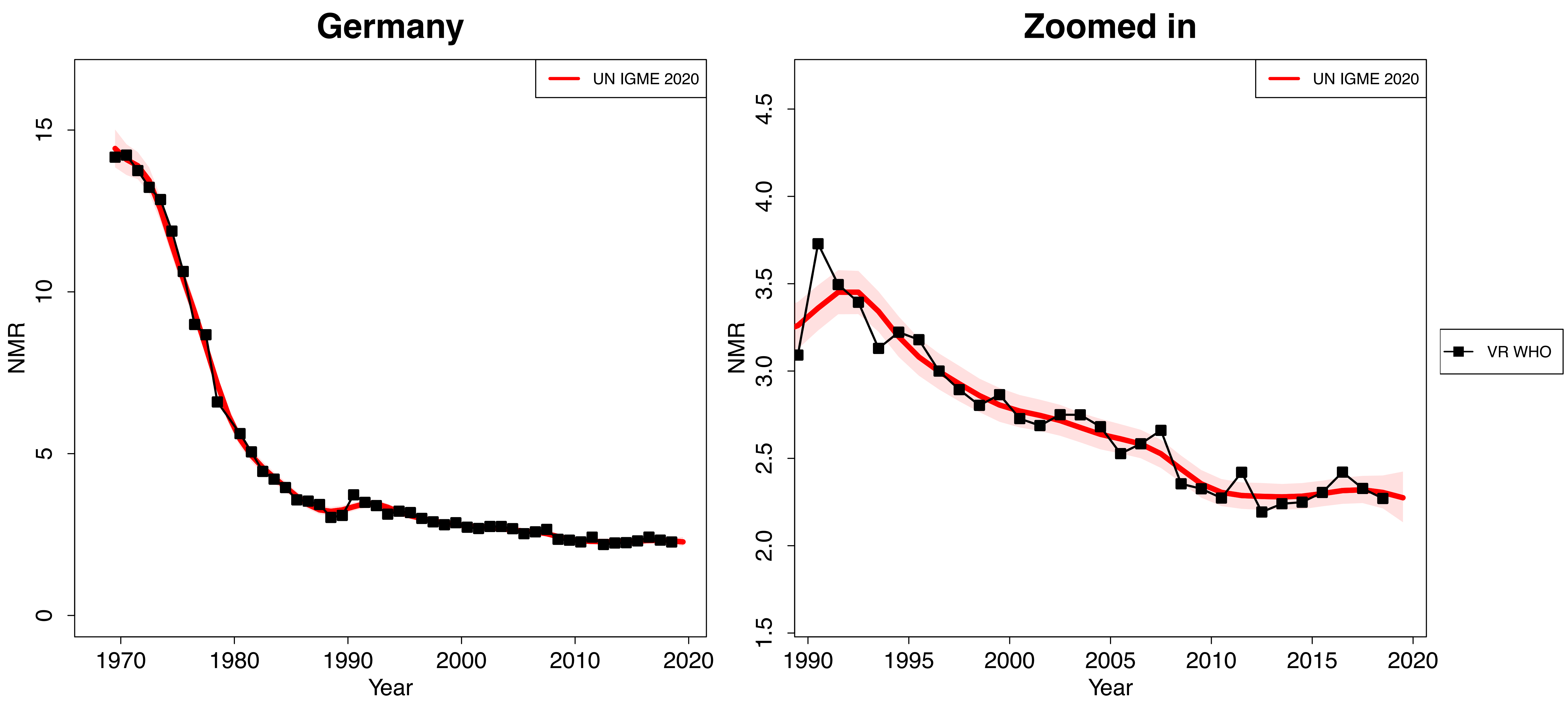

Germany
Zoomed in
UN IGME 2020
VR WHO
NMR
Year

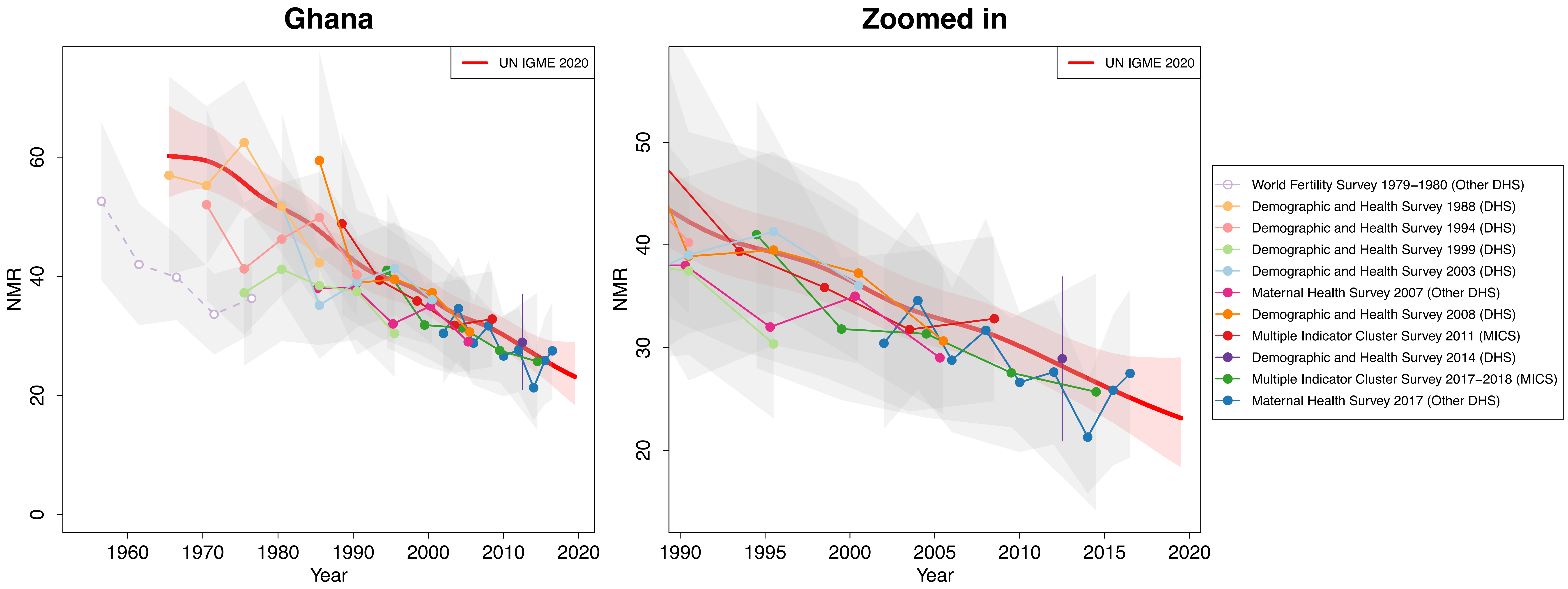

Ghana
Zoomed in
UN IGME 2020
NMR
Year
World Fertility Survey 1979–1980 (Other DHS)
Demographic and Health Survey 1988 (DHS)
Demographic and Health Survey 1994 (DHS)
Demographic and Health Survey 1999 (DHS)
Demographic and Health Survey 2003 (DHS)
Maternal Health Survey 2007 (Other DHS)
Demographic and Health Survey 2008 (DHS)
Multiple Indicator Cluster Survey 2011 (MICS)
Demographic and Health Survey 2014 (DHS)
Multiple Indicator Cluster Survey 2017–2018 (MICS)
Maternal Health Survey 2017 (Other DHS)

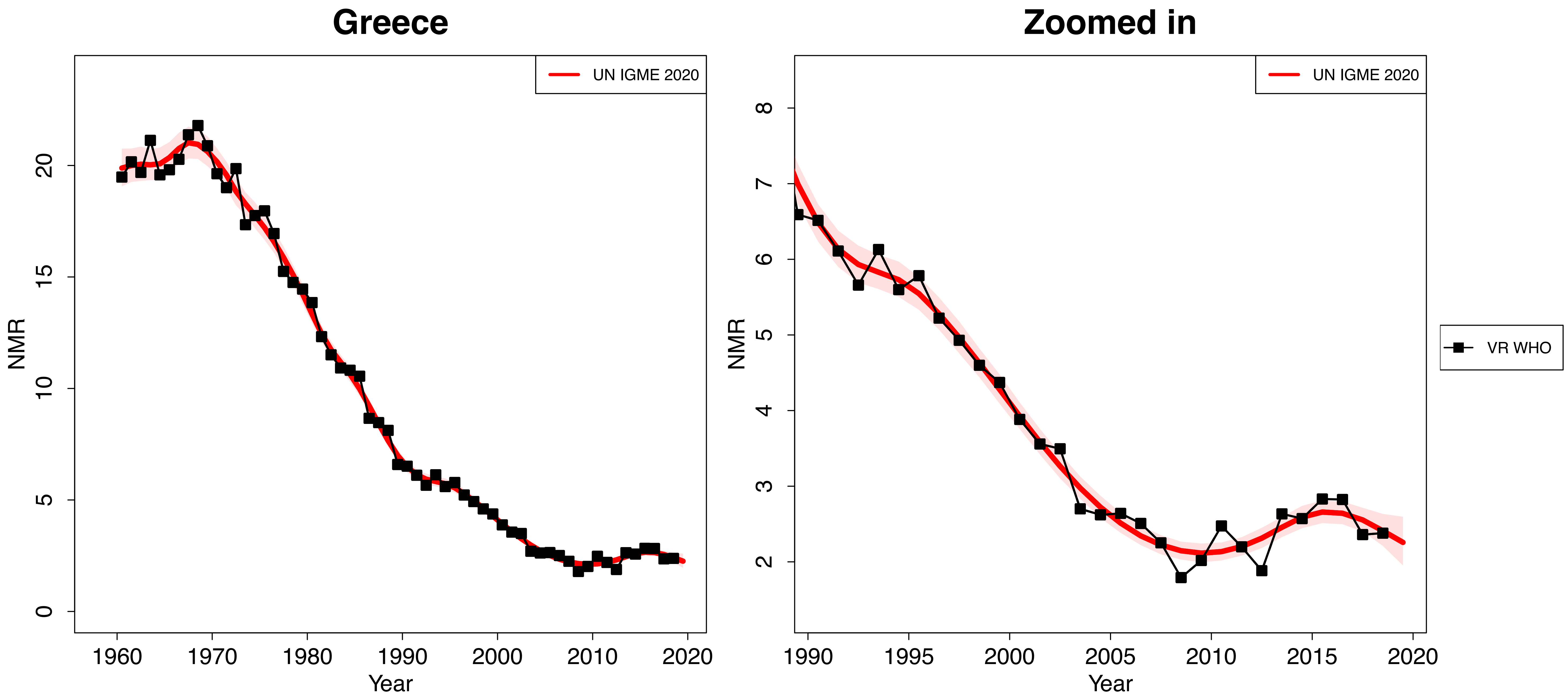
Greece
Zoomed in
UN IGME 2020
UN IGME 2020
VR WHO
NMR
NMR
Year
Year
0
5
10
15
20
2
3
4
5
6
7
8
1960
1970
1980
1990
2000
2010
2020
1990
1995
2000
2005
2010
2015
2020

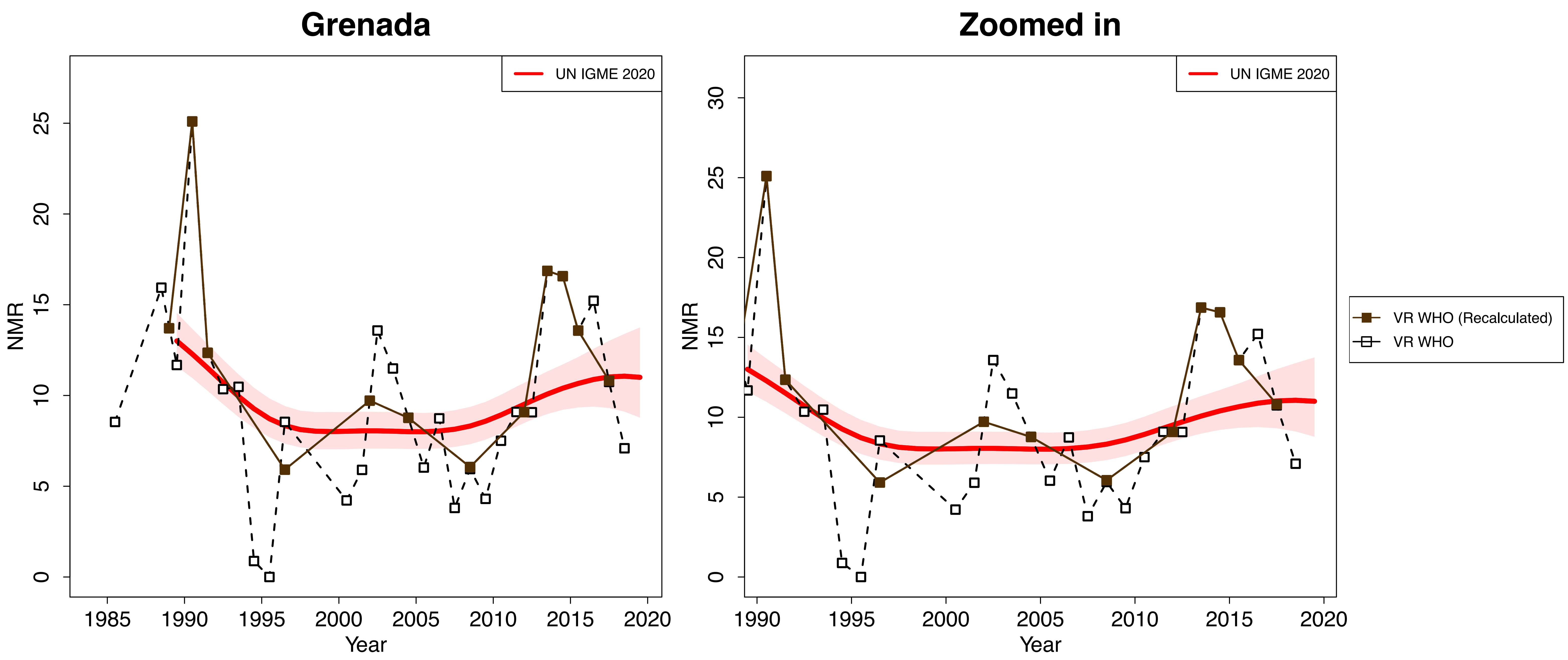

Grenada
Zoomed in
UN IGME 2020
VR WHO (Recalculated)
VR WHO
NMR
Year

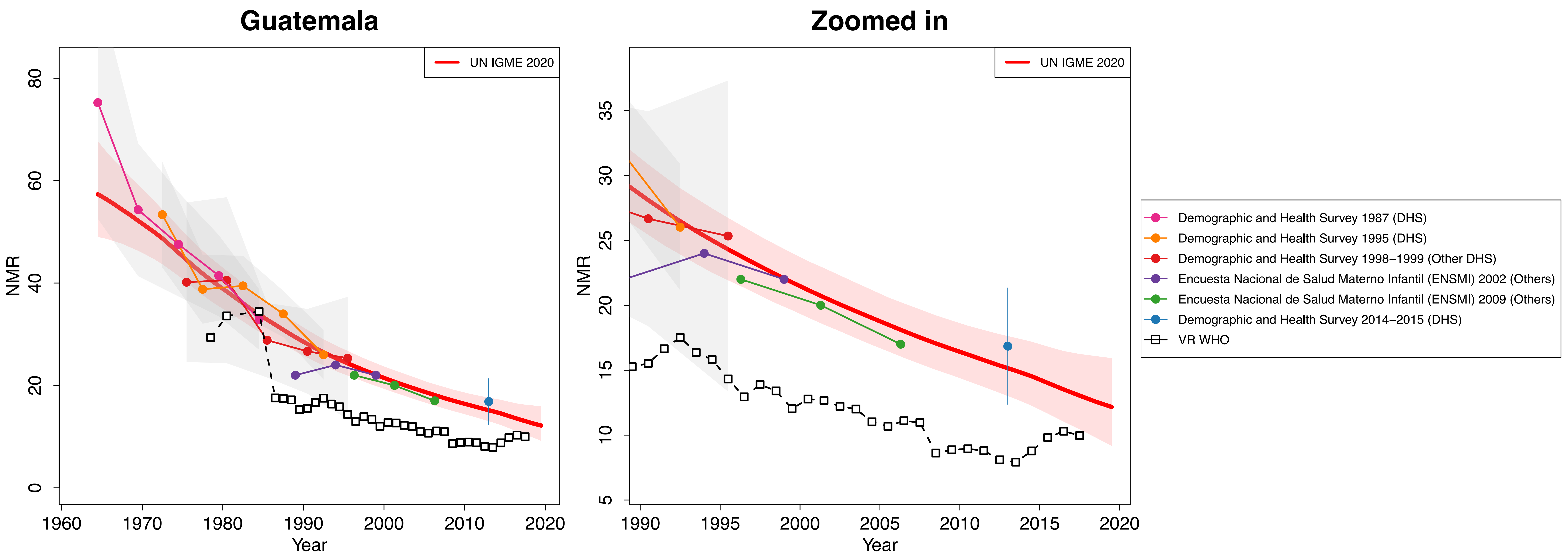
Guatemala
Zoomed in
UN IGME 2020
UN IGME 2020
NMR
NMR
Year
Year
0
20
40
60
80
1960
1970
1980
1990
2000
2010
2020
5
10
15
20
25
30
35
1990
1995
2000
2005
2010
2015
2020
Demographic and Health Survey 1987 (DHS)
Demographic and Health Survey 1995 (DHS)
Demographic and Health Survey 1998–1999 (Other DHS)
Encuesta Nacional de Salud Materno Infantil (ENSMI) 2002 (Others)
Encuesta Nacional de Salud Materno Infantil (ENSMI) 2009 (Others)
Demographic and Health Survey 2014–2015 (DHS)
VR WHO

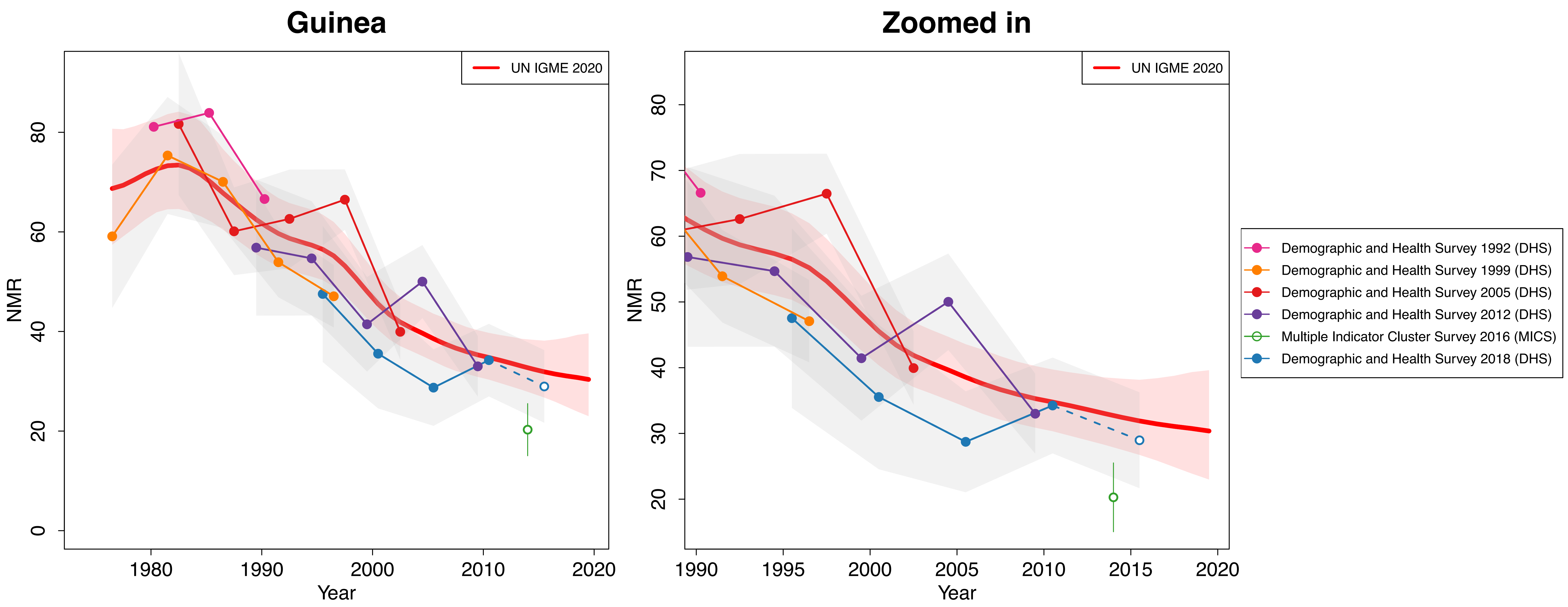

Guinea
Zoomed in
UN IGME 2020
NMR
Year
Demographic and Health Survey 1992 (DHS)
Demographic and Health Survey 1999 (DHS)
Demographic and Health Survey 2005 (DHS)
Demographic and Health Survey 2012 (DHS)
Multiple Indicator Cluster Survey 2016 (MICS)
Demographic and Health Survey 2018 (DHS)

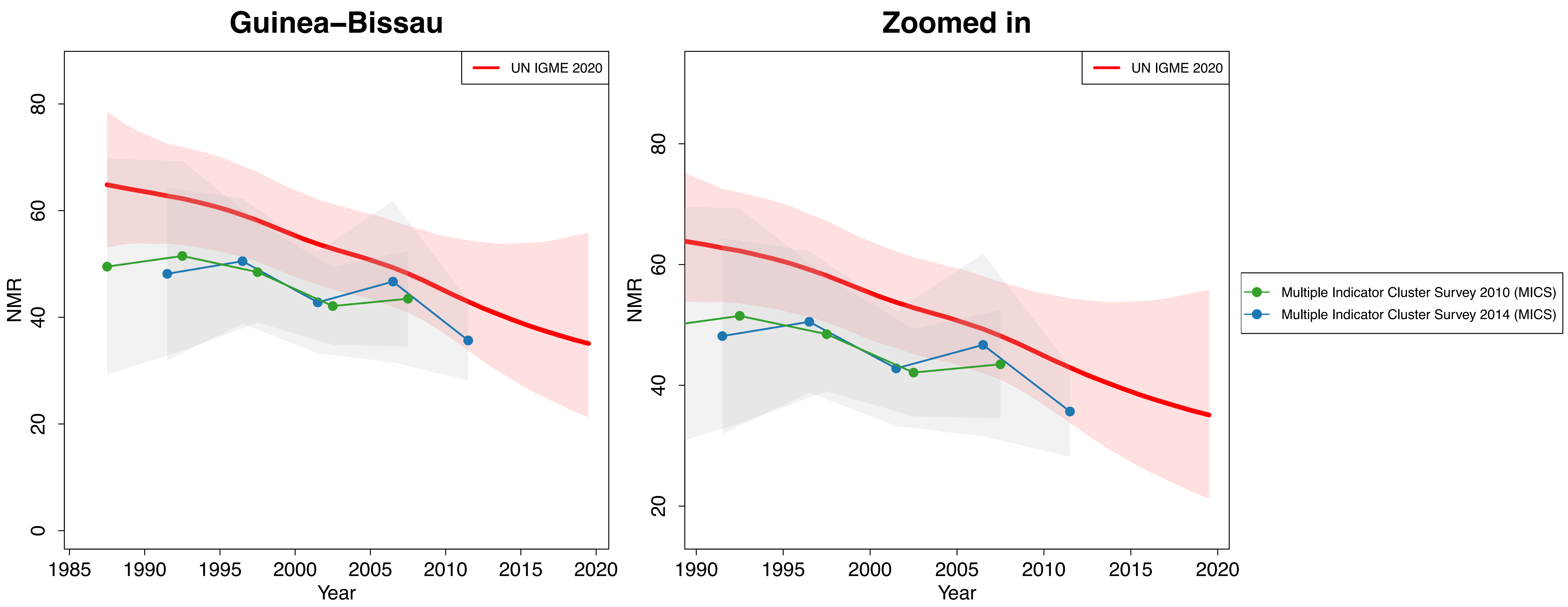

Guinea–Bissau
Zoomed in
UN IGME 2020
NMR
Year
Multiple Indicator Cluster Survey 2010 (MICS)
Multiple Indicator Cluster Survey 2014 (MICS)

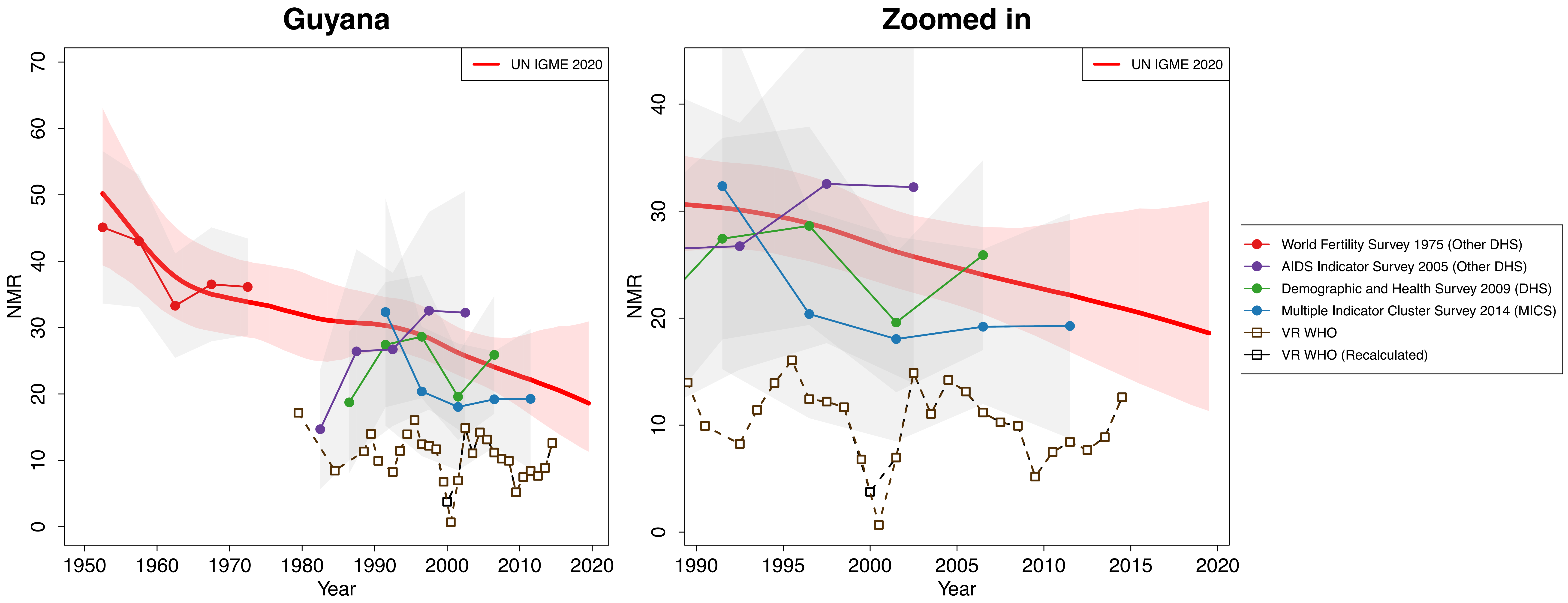

Guyana
Zoomed in
UN IGME 2020
UN IGME 2020
NMR
NMR
Year
Year
0
10
20
30
40
50
60
70
1950
1960
1970
1980
1990
2000
2010
2020
0
10
20
30
40
1990
1995
2000
2005
2010
2015
2020
World Fertility Survey 1975 (Other DHS)
AIDS Indicator Survey 2005 (Other DHS)
Demographic and Health Survey 2009 (DHS)
Multiple Indicator Cluster Survey 2014 (MICS)
VR WHO
VR WHO (Recalculated)

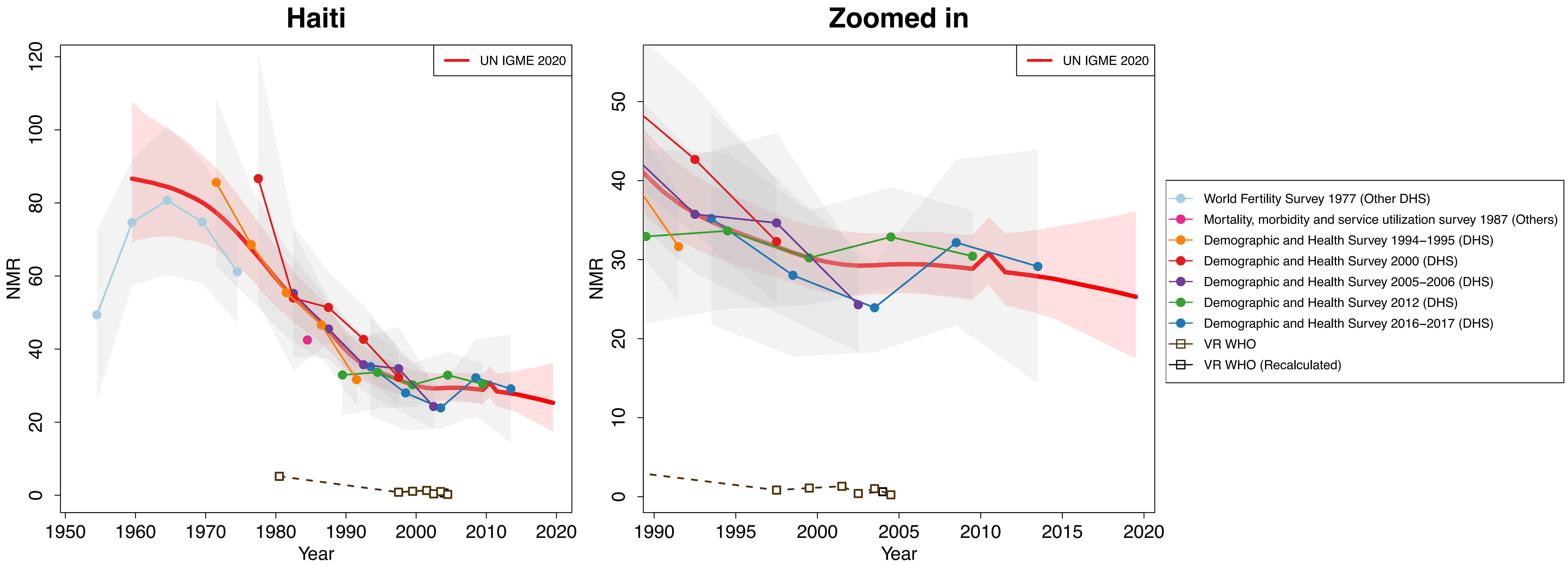

Haiti
Zoomed in
UN IGME 2020
NMR
Year
World Fertility Survey 1977 (Other DHS)
Mortality, morbidity and service utilization survey 1987 (Others)
Demographic and Health Survey 1994–1995 (DHS)
Demographic and Health Survey 2000 (DHS)
Demographic and Health Survey 2005–2006 (DHS)
Demographic and Health Survey 2012 (DHS)
Demographic and Health Survey 2016–2017 (DHS)
VR WHO
VR WHO (Recalculated)

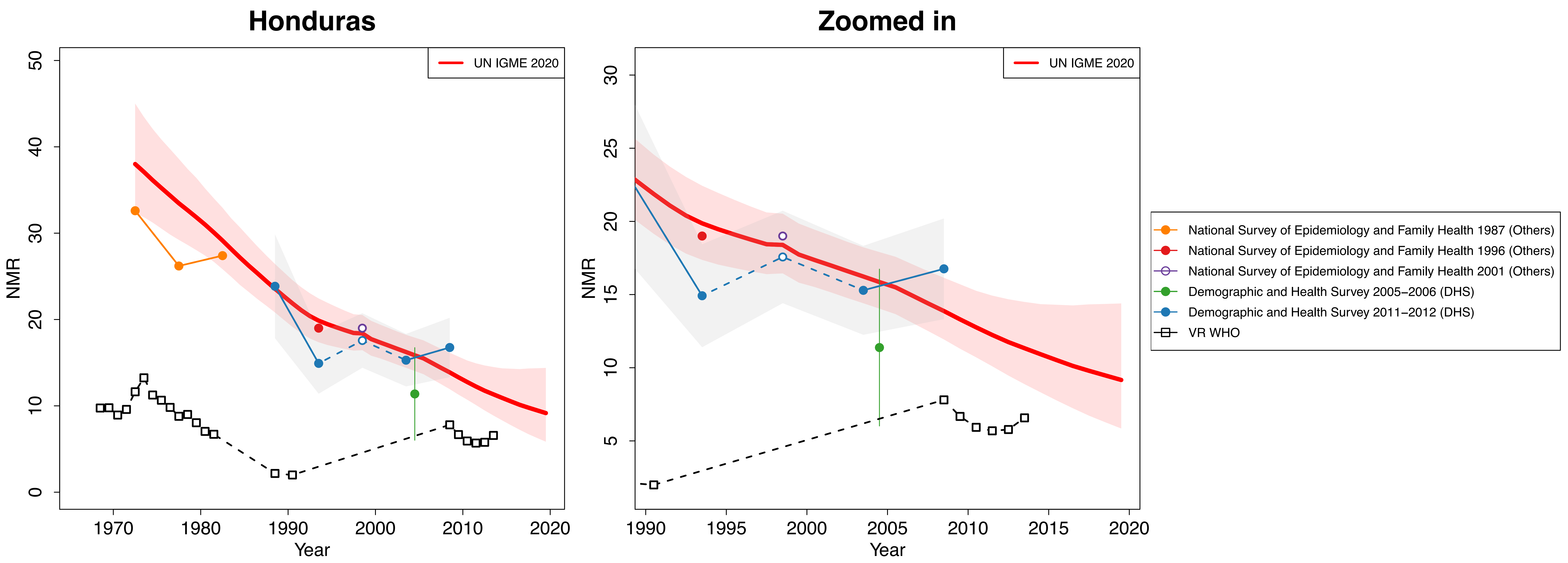

Honduras
Zoomed in
UN IGME 2020
NMR
Year
National Survey of Epidemiology and Family Health 1987 (Others)
National Survey of Epidemiology and Family Health 1996 (Others)
National Survey of Epidemiology and Family Health 2001 (Others)
Demographic and Health Survey 2005–2006 (DHS)
Demographic and Health Survey 2011–2012 (DHS)
VR WHO

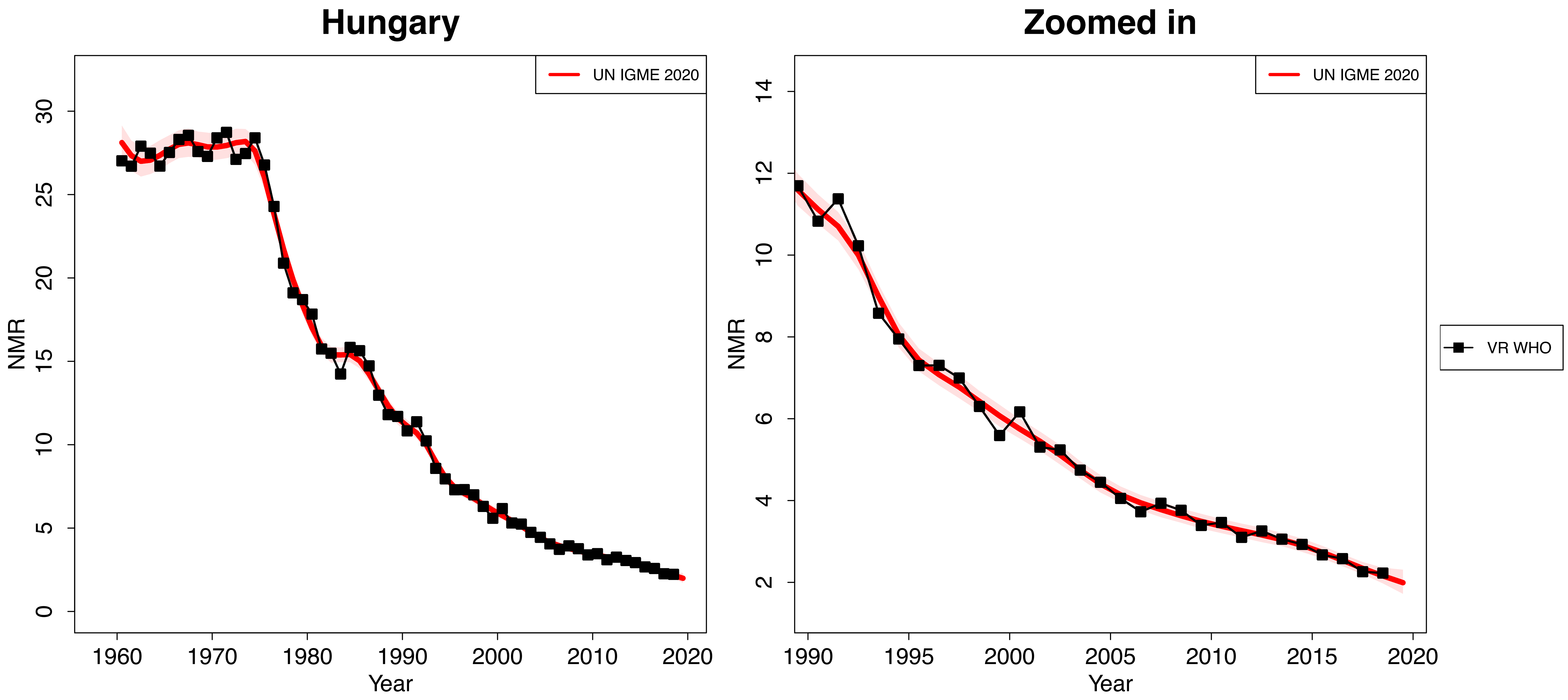

Hungary
Zoomed in
UN IGME 2020
UN IGME 2020
VR WHO
NMR
NMR
Year
Year

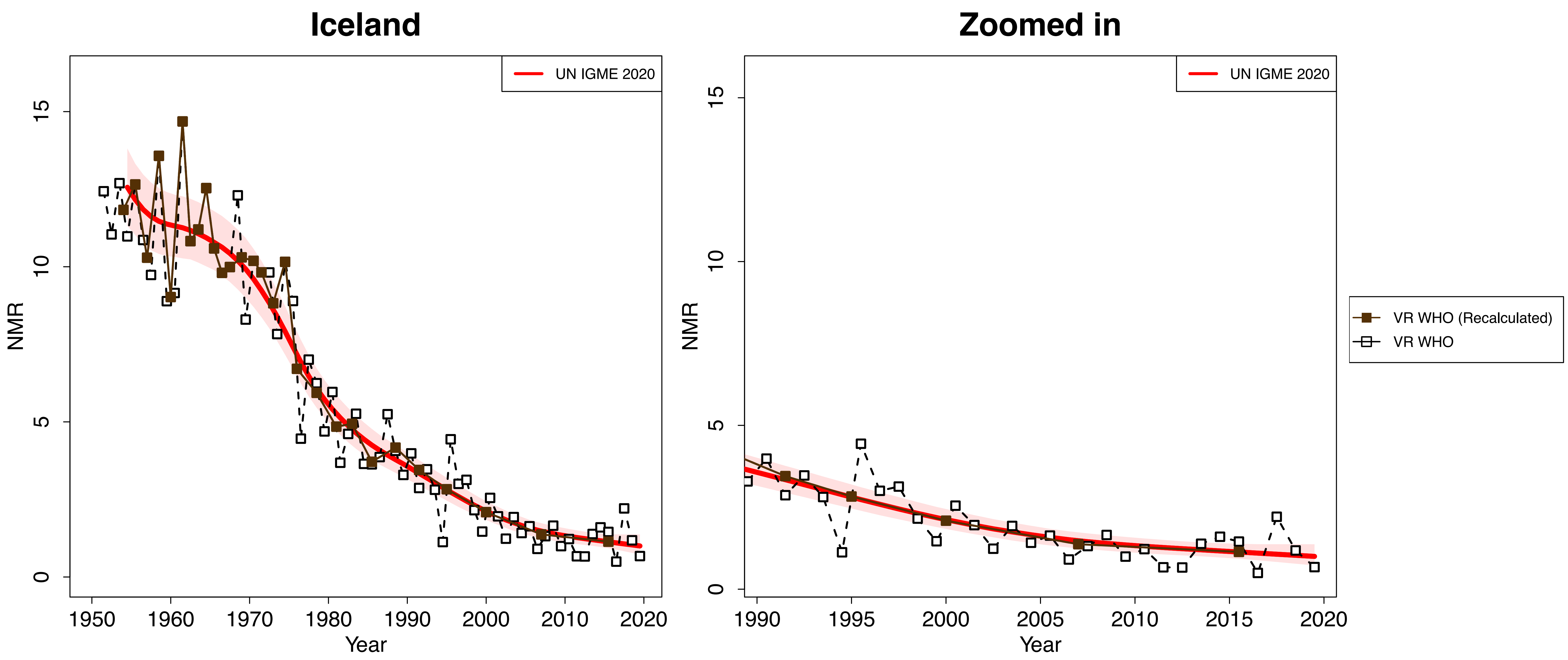
Iceland
Zoomed in
UN IGME 2020
NMR
Year
0
5
10
15
1950
1960
1970
1980
1990
2000
2010
2020
1995
2005
2015
VR WHO (Recalculated)
VR WHO

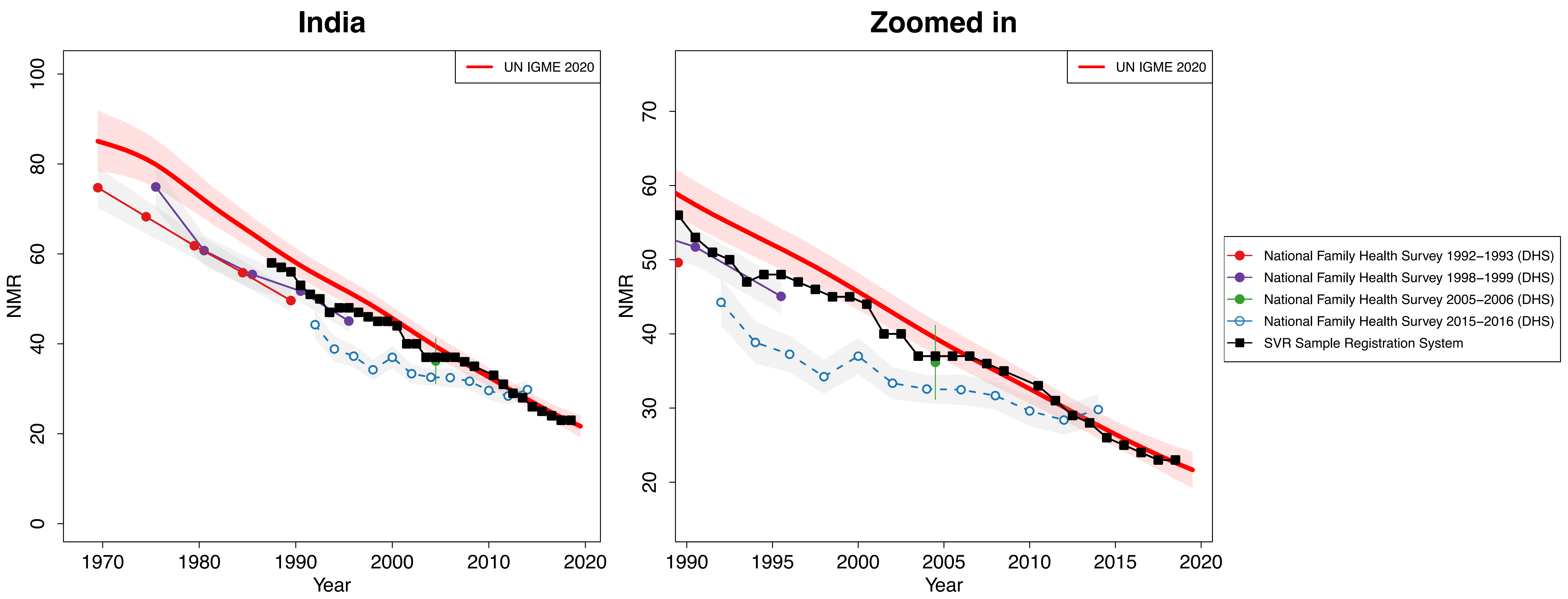
India
Zoomed in
UN IGME 2020
NMR
Year
National Family Health Survey 1992–1993 (DHS)
National Family Health Survey 1998–1999 (DHS)
National Family Health Survey 2005–2006 (DHS)
National Family Health Survey 2015–2016 (DHS)
SVR Sample Registration System

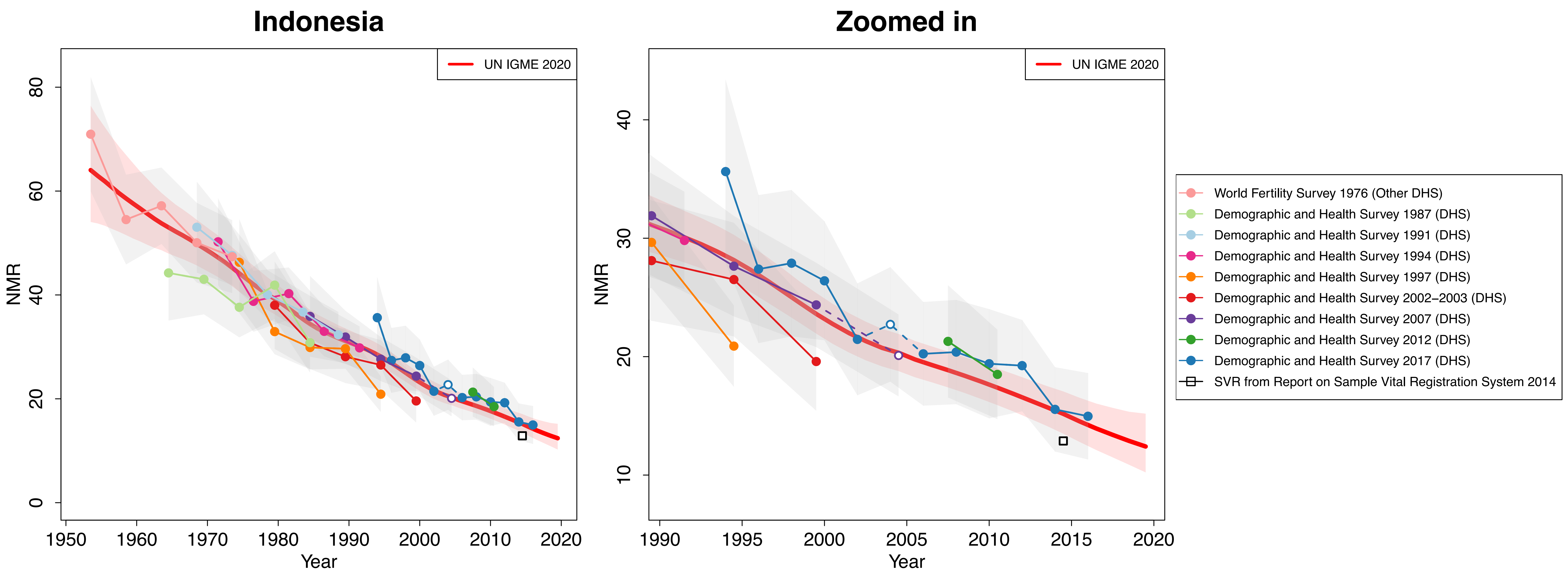
Indonesia
Zoomed in
UN IGME 2020
NMR
Year
World Fertility Survey 1976 (Other DHS)
Demographic and Health Survey 1987 (DHS)
Demographic and Health Survey 1991 (DHS)
Demographic and Health Survey 1994 (DHS)
Demographic and Health Survey 1997 (DHS)
Demographic and Health Survey 2002–2003 (DHS)
Demographic and Health Survey 2007 (DHS)
Demographic and Health Survey 2012 (DHS)
Demographic and Health Survey 2017 (DHS)
SVR from Report on Sample Vital Registration System 2014

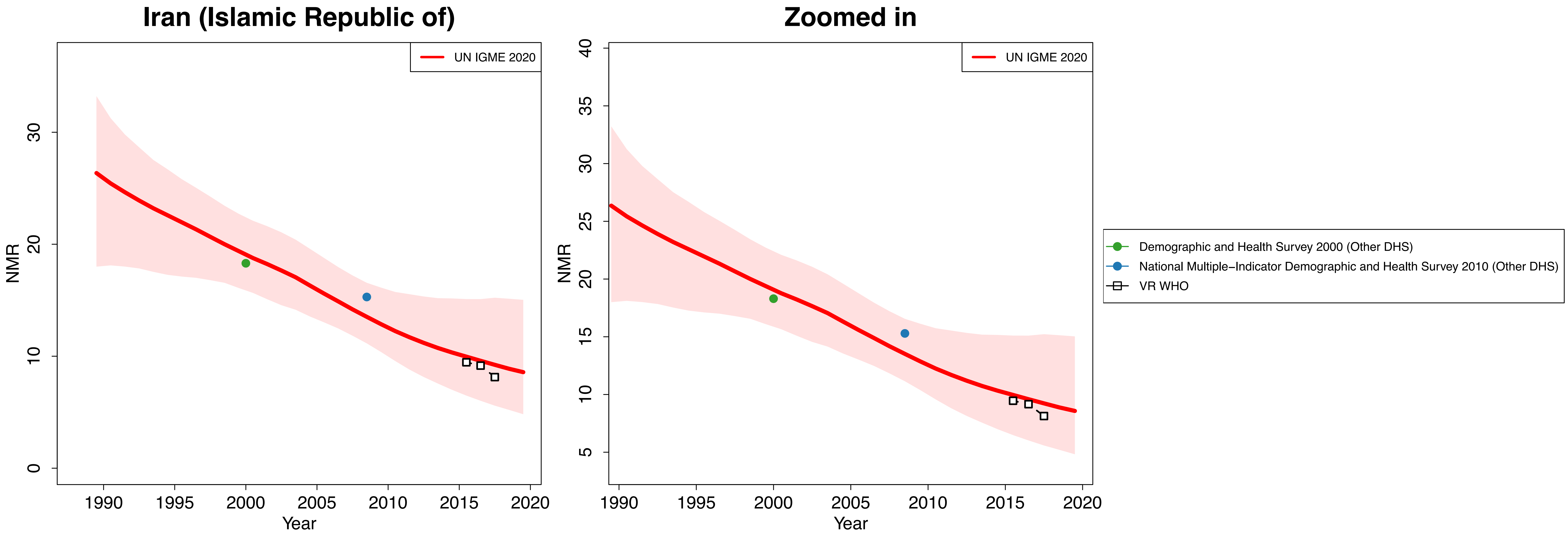

Iran (Islamic Republic of)
Zoomed in
UN IGME 2020
NMR
Year
Demographic and Health Survey 2000 (Other DHS)
National Multiple−Indicator Demographic and Health Survey 2010 (Other DHS)
VR WHO

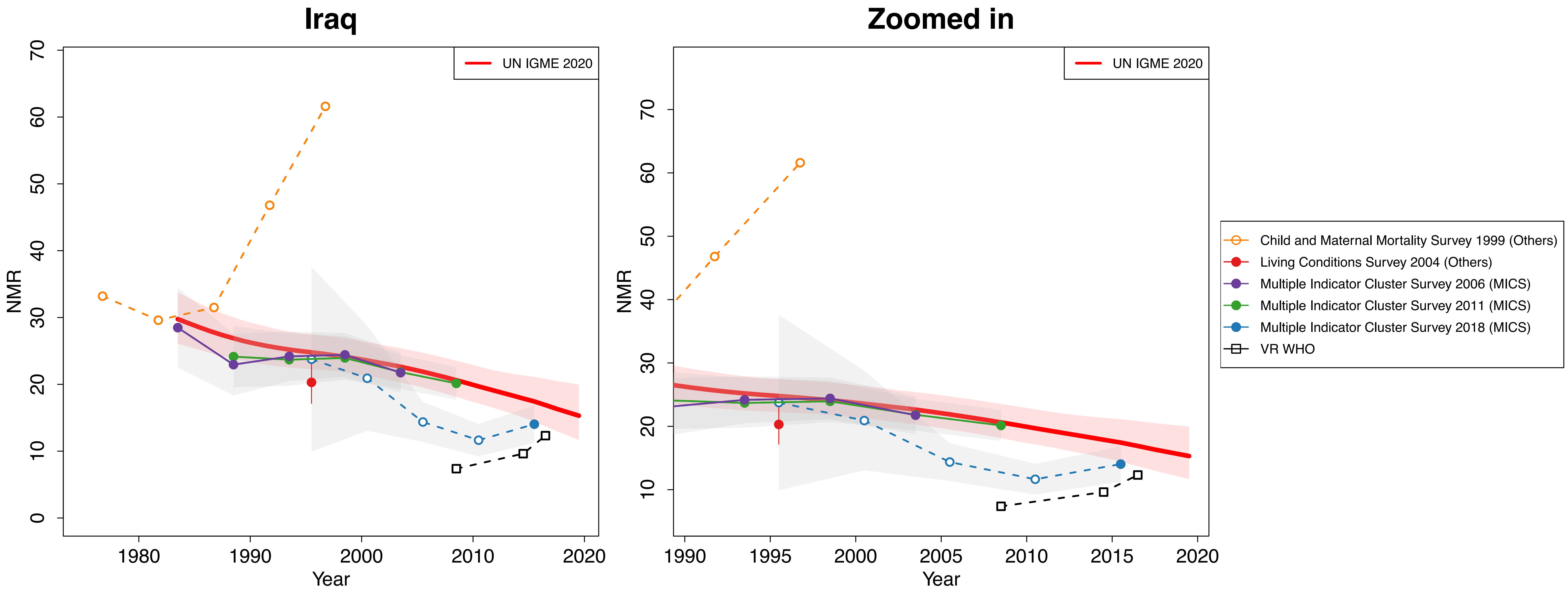
Iraq
Zoomed in
UN IGME 2020
UN IGME 2020
NMR
NMR
Year
Year
0
10
20
30
40
50
60
70
1980
1990
2000
2010
2020
1990
1995
2000
2005
2010
2015
2020
Child and Maternal Mortality Survey 1999 (Others)
Living Conditions Survey 2004 (Others)
Multiple Indicator Cluster Survey 2006 (MICS)
Multiple Indicator Cluster Survey 2011 (MICS)
Multiple Indicator Cluster Survey 2018 (MICS)
VR WHO

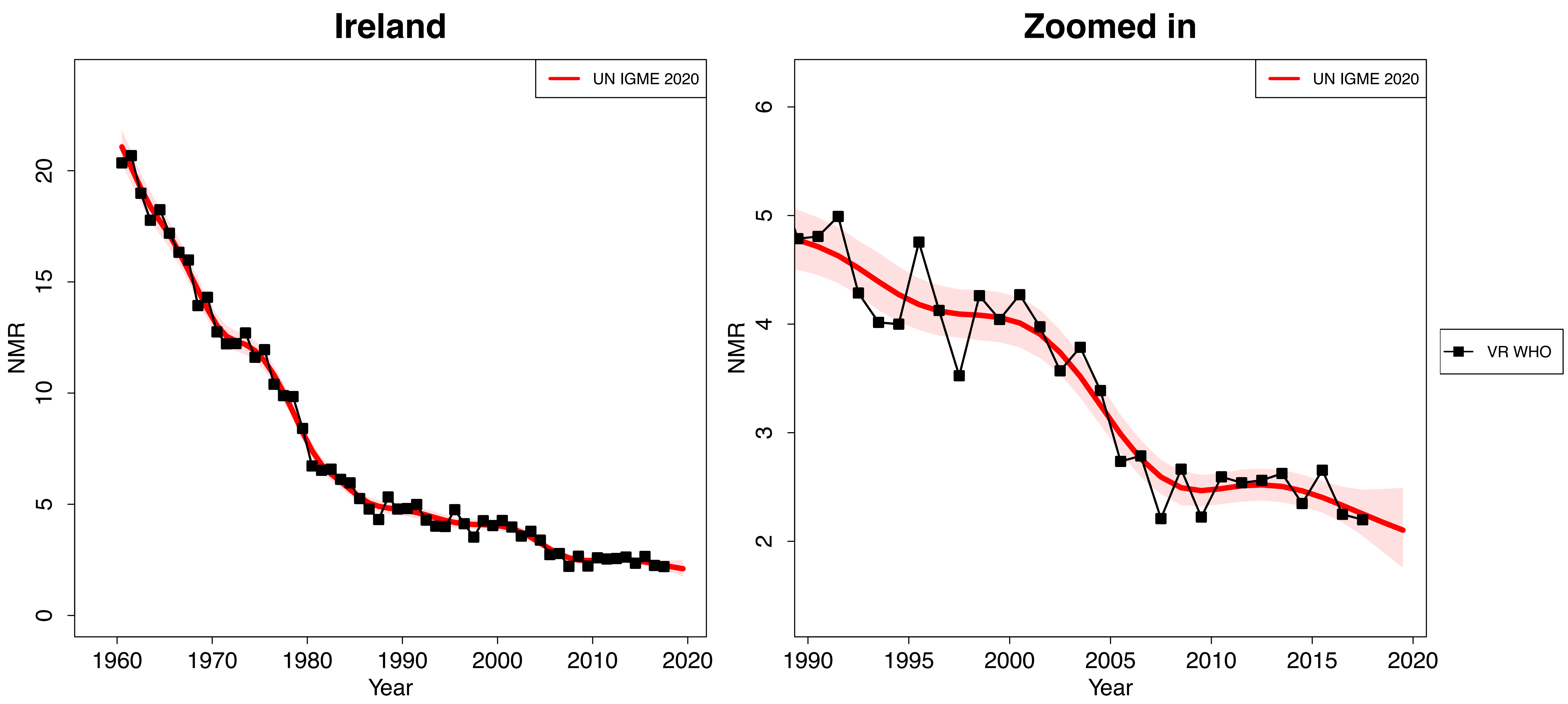

Ireland
UN IGME 2020
NMR
Year
Zoomed in
UN IGME 2020
NMR
Year
VR WHO

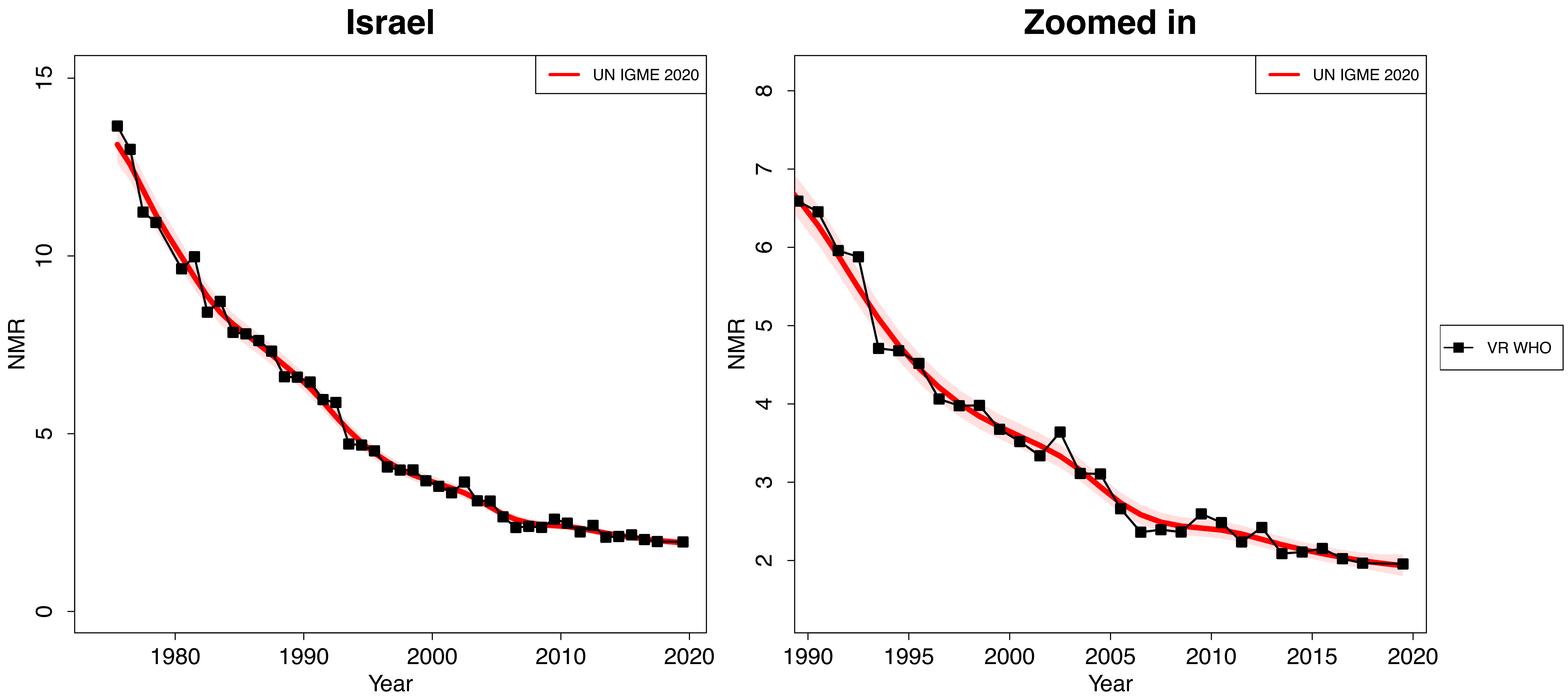

Israel
Zoomed in
UN IGME 2020
UN IGME 2020
VR WHO
NMR
NMR
Year
Year
0
5
10
15
2
3
4
5
6
7
8
1980
1990
2000
2010
2020
1990
1995
2000
2005
2010
2015
2020

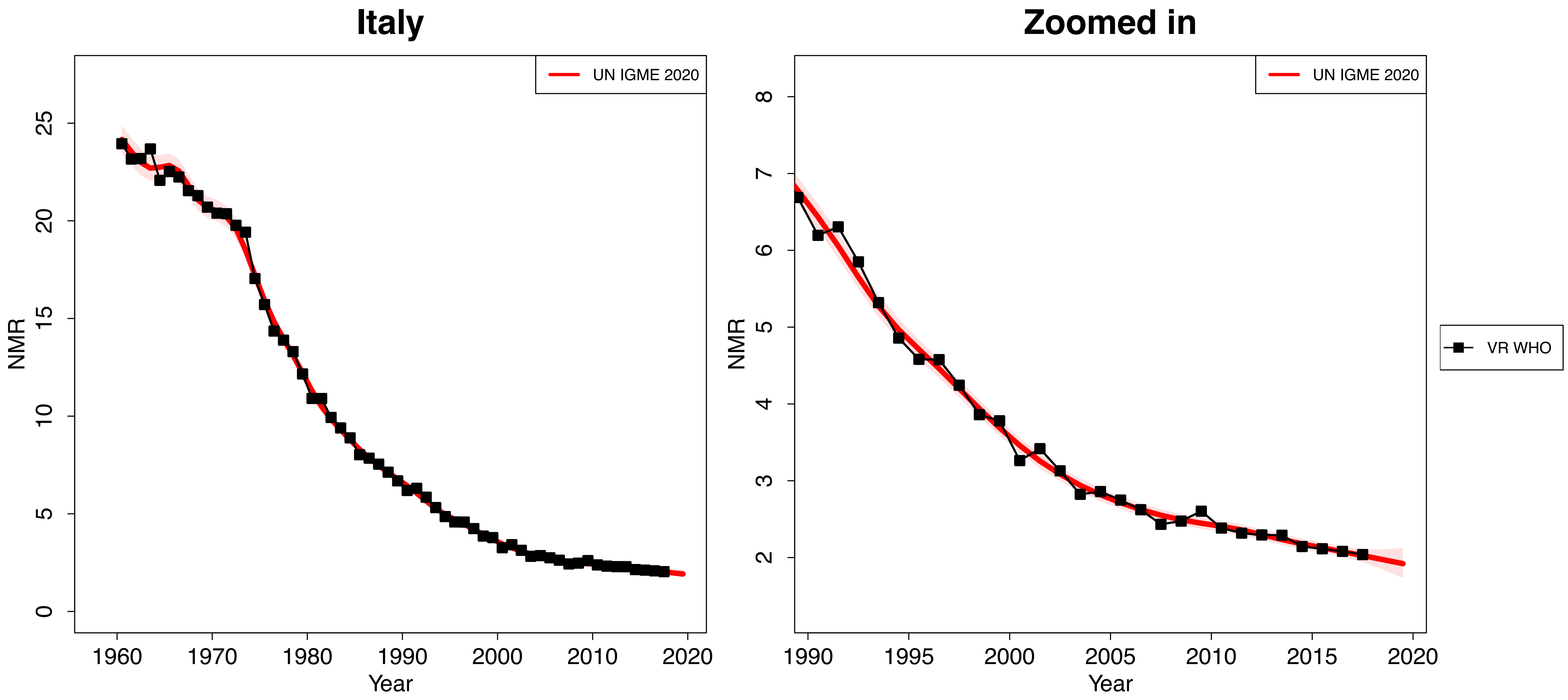

Italy
Zoomed in
UN IGME 2020
VR WHO
NMR
Year

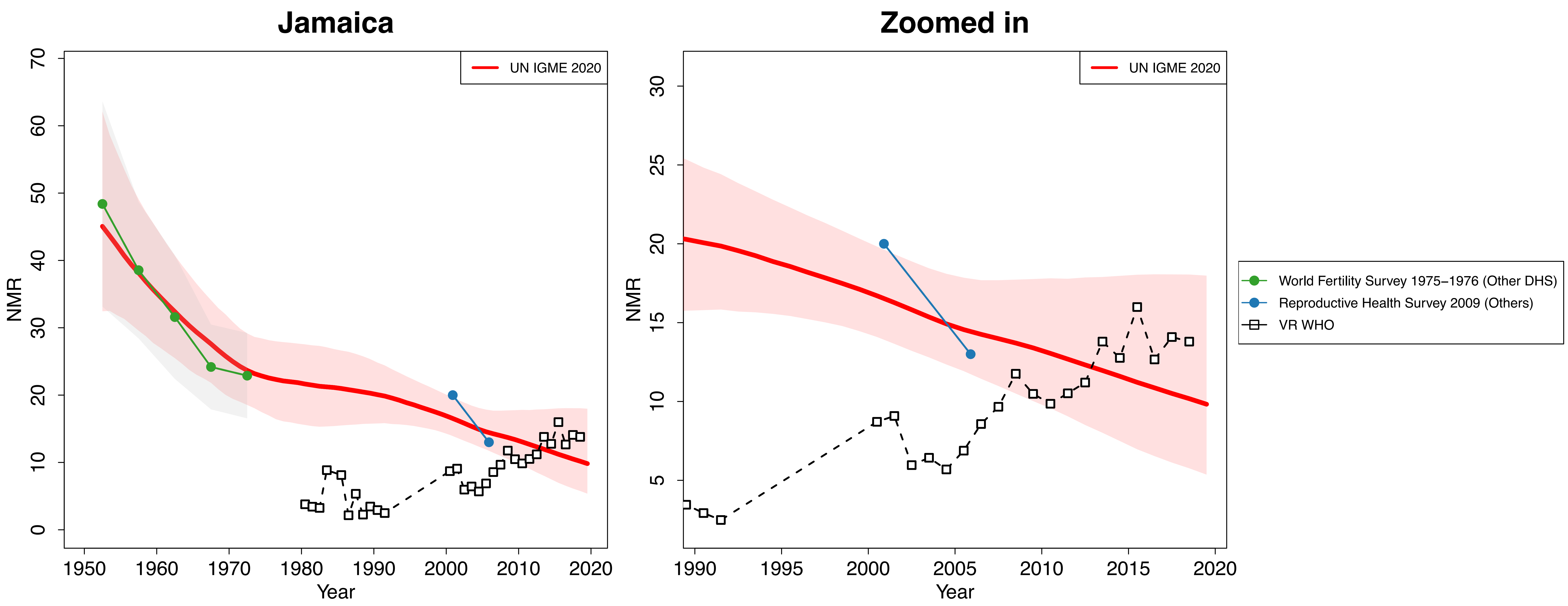

Jamaica
Zoomed in
UN IGME 2020
NMR
Year
World Fertility Survey 1975–1976 (Other DHS)
Reproductive Health Survey 2009 (Others)
VR WHO

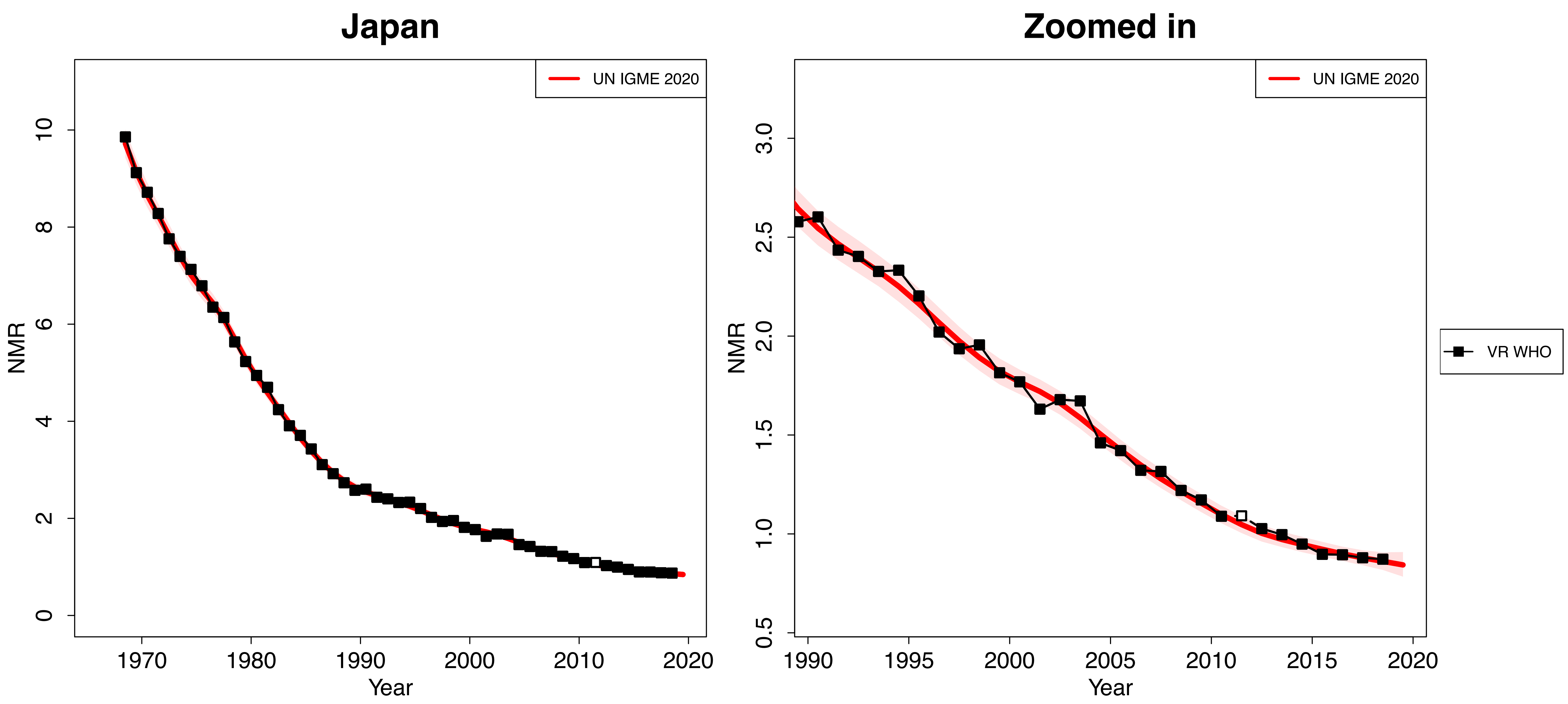

Japan
Zoomed in
UN IGME 2020
VR WHO
NMR
Year

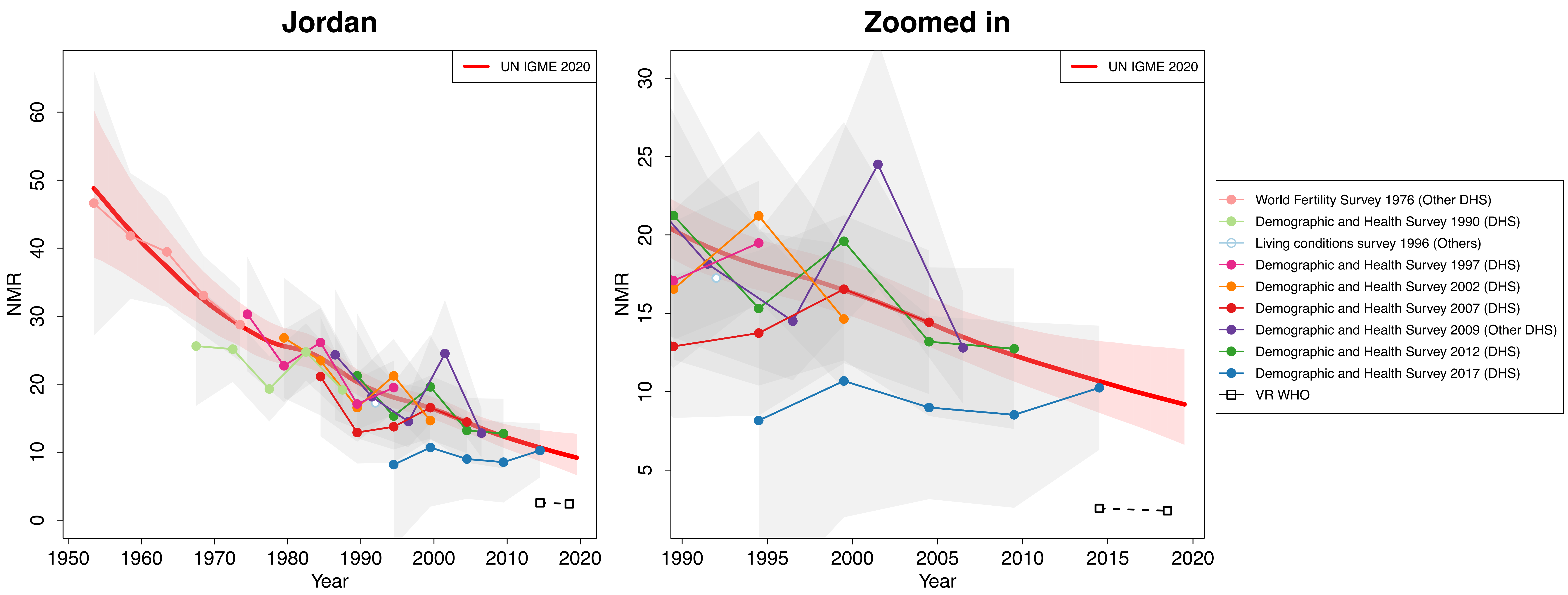

Jordan
Zoomed in
UN IGME 2020
NMR
Year
0
10
20
30
40
50
60
1950
1960
1970
1980
1990
2000
2010
2020
5
10
15
20
25
30
1995
2005
2015
World Fertility Survey 1976 (Other DHS)
Demographic and Health Survey 1990 (DHS)
Living conditions survey 1996 (Others)
Demographic and Health Survey 1997 (DHS)
Demographic and Health Survey 2002 (DHS)
Demographic and Health Survey 2007 (DHS)
Demographic and Health Survey 2009 (Other DHS)
Demographic and Health Survey 2012 (DHS)
Demographic and Health Survey 2017 (DHS)
VR WHO

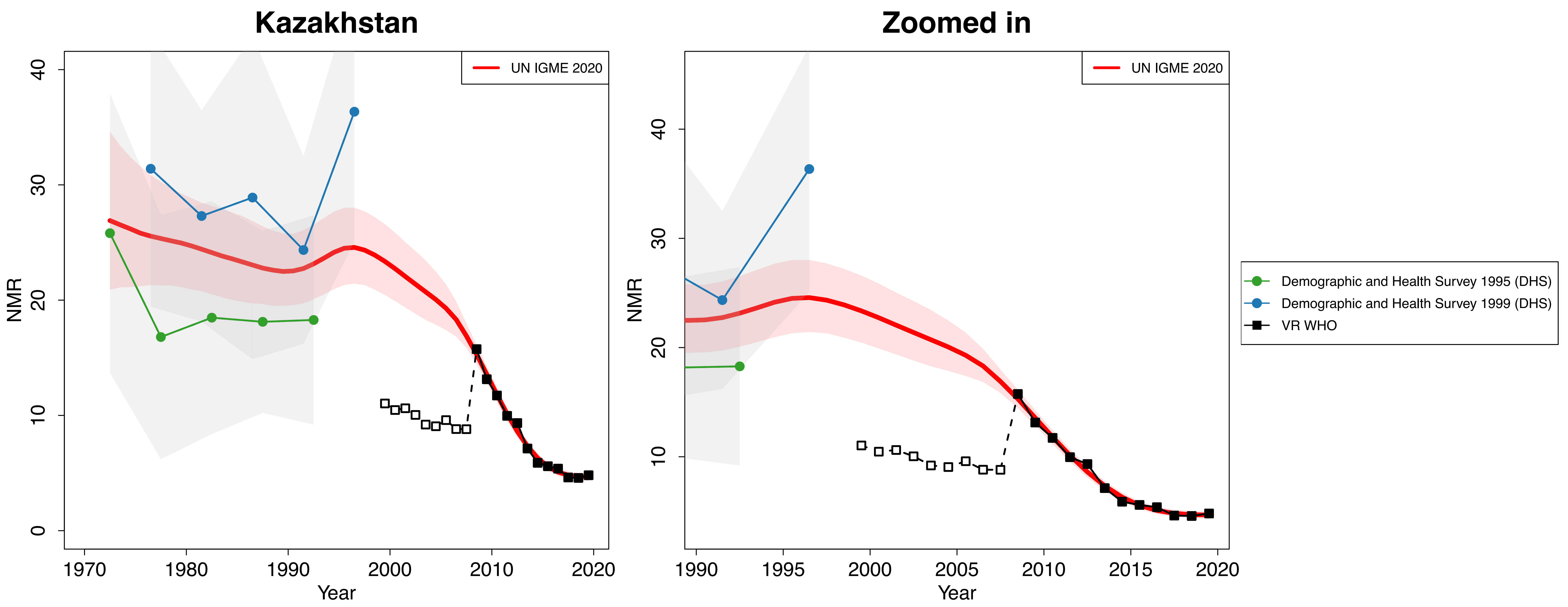
Kazakhstan
Zoomed in
UN IGME 2020
NMR
Year
Demographic and Health Survey 1995 (DHS)
Demographic and Health Survey 1999 (DHS)
VR WHO
0
10
20
30
40
1970
1980
1990
2000
2010
2020
1995
2005
2015

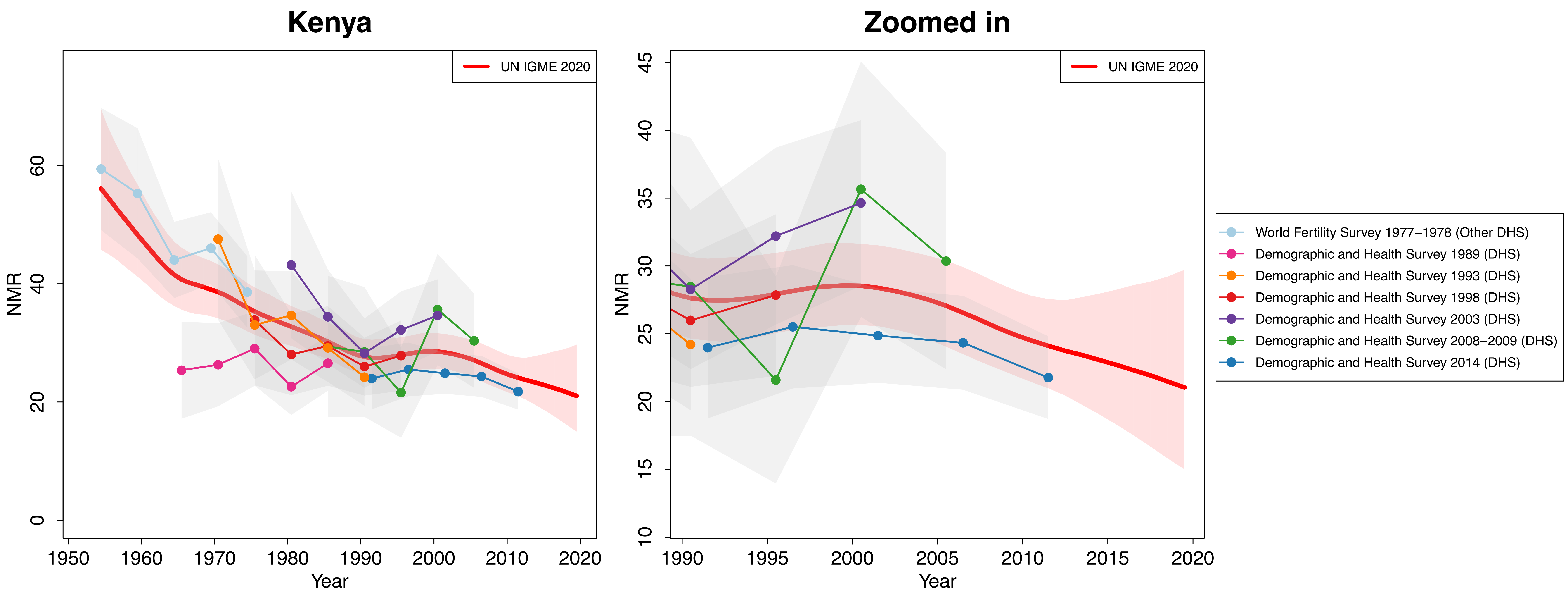
Kenya
Zoomed in
UN IGME 2020
NMR
Year
0
20
40
60
1950
1960
1970
1980
1990
2000
2010
2020
10
15
20
25
30
35
40
45
1995
2005
2015
World Fertility Survey 1977–1978 (Other DHS)
Demographic and Health Survey 1989 (DHS)
Demographic and Health Survey 1993 (DHS)
Demographic and Health Survey 1998 (DHS)
Demographic and Health Survey 2003 (DHS)
Demographic and Health Survey 2008–2009 (DHS)
Demographic and Health Survey 2014 (DHS)

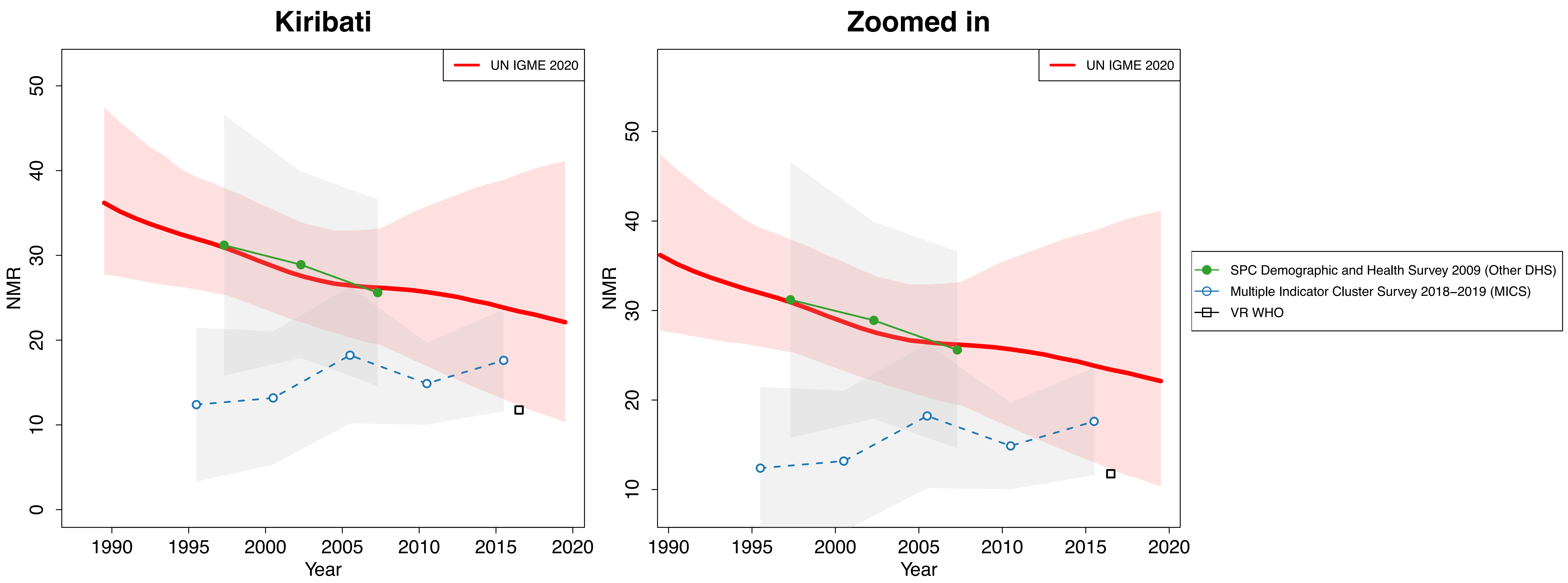

Kiribati
Zoomed in
UN IGME 2020
UN IGME 2020
NMR
NMR
Year
Year
0
10
20
30
40
50
10
20
30
40
50
1990
1995
2000
2005
2010
2015
2020
1990
1995
2000
2005
2010
2015
2020
SPC Demographic and Health Survey 2009 (Other DHS)
Multiple Indicator Cluster Survey 2018–2019 (MICS)
VR WHO

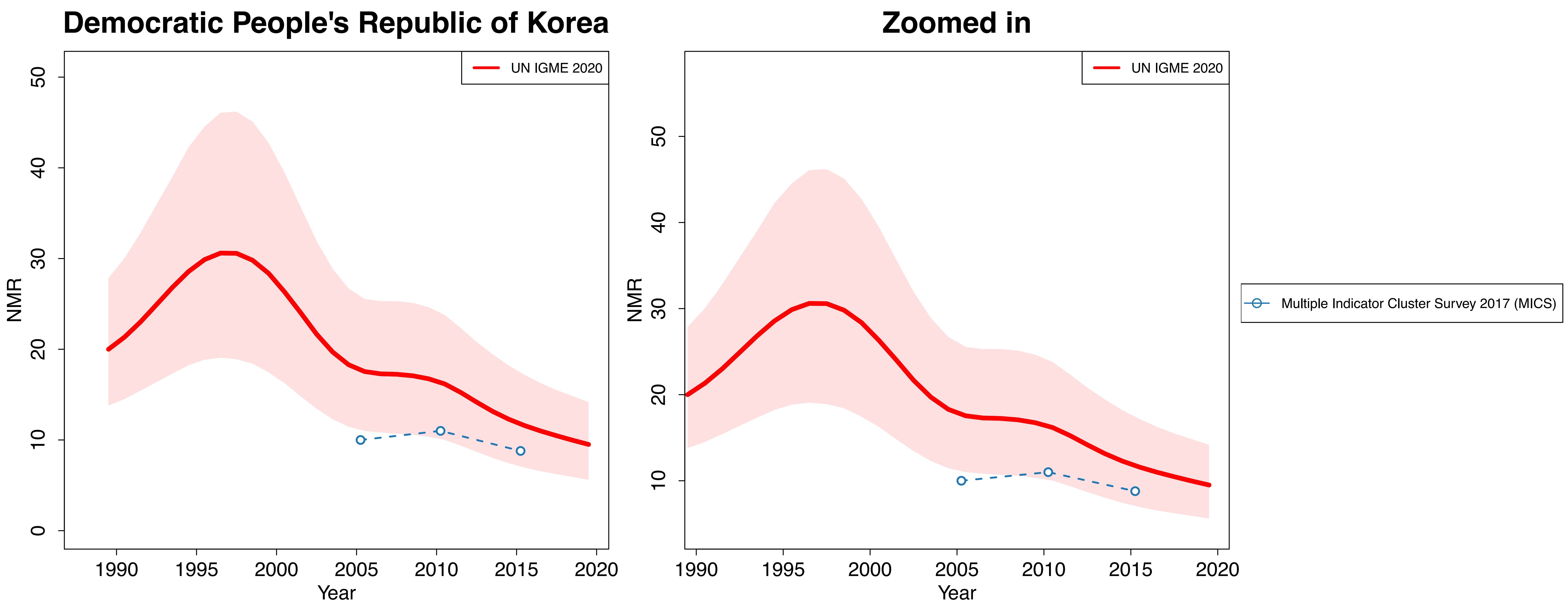
Democratic People's Republic of Korea
Zoomed in
UN IGME 2020
UN IGME 2020
Multiple Indicator Cluster Survey 2017 (MICS)
NMR
NMR
Year
Year
0
10
20
30
40
50
1990
1995
2000
2005
2010
2015
2020

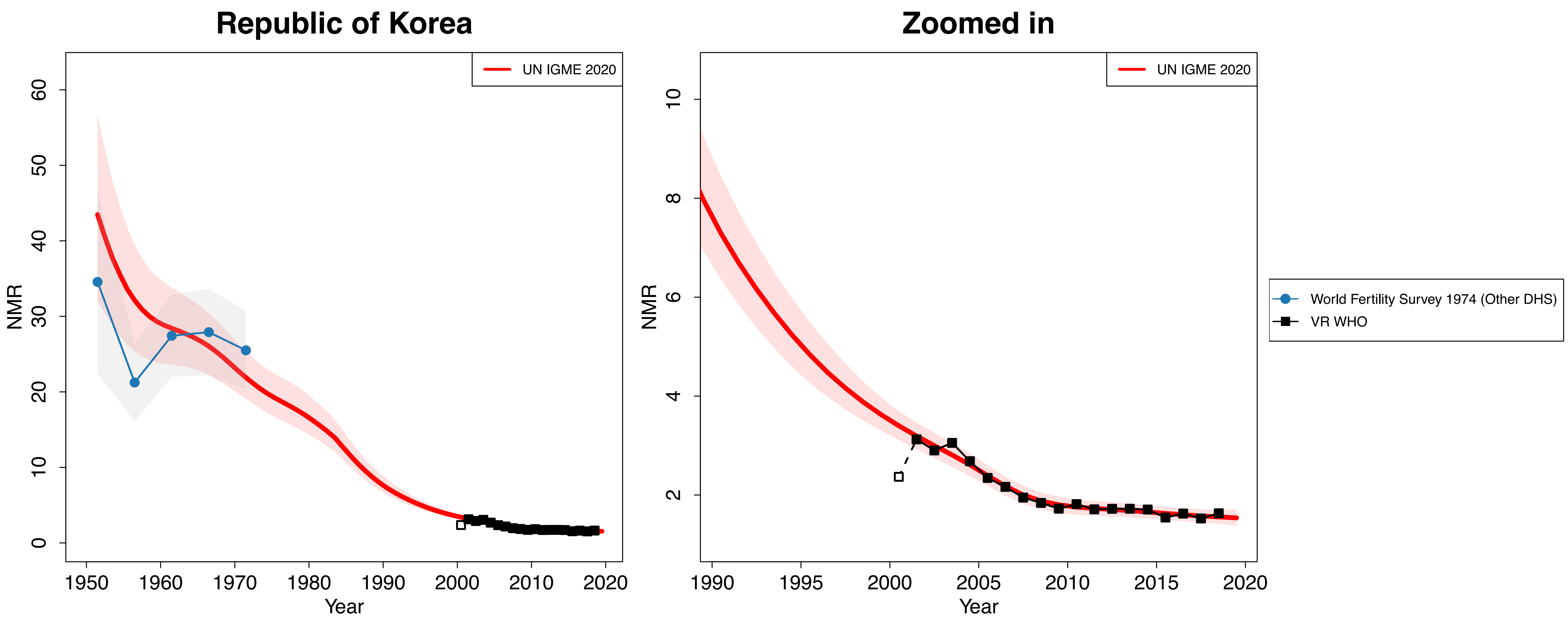

Republic of Korea
Zoomed in
UN IGME 2020
World Fertility Survey 1974 (Other DHS)
VR WHO
NMR
Year

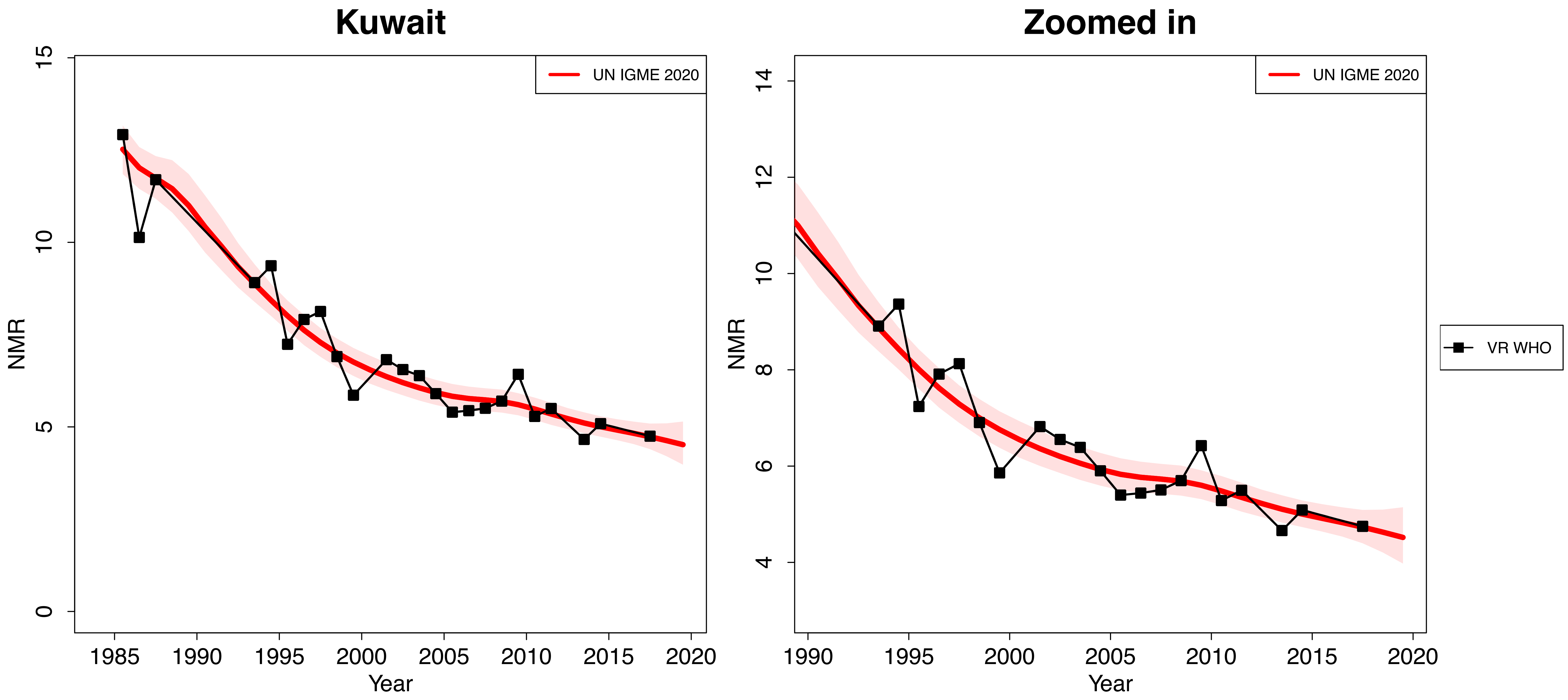

Kuwait
Zoomed in
UN IGME 2020
UN IGME 2020
VR WHO
NMR
NMR
Year
Year
0
5
10
15
4
6
8
10
12
14
1985
1990
1995
2000
2005
2010
2015
2020
1990
1995
2000
2005
2010
2015
2020

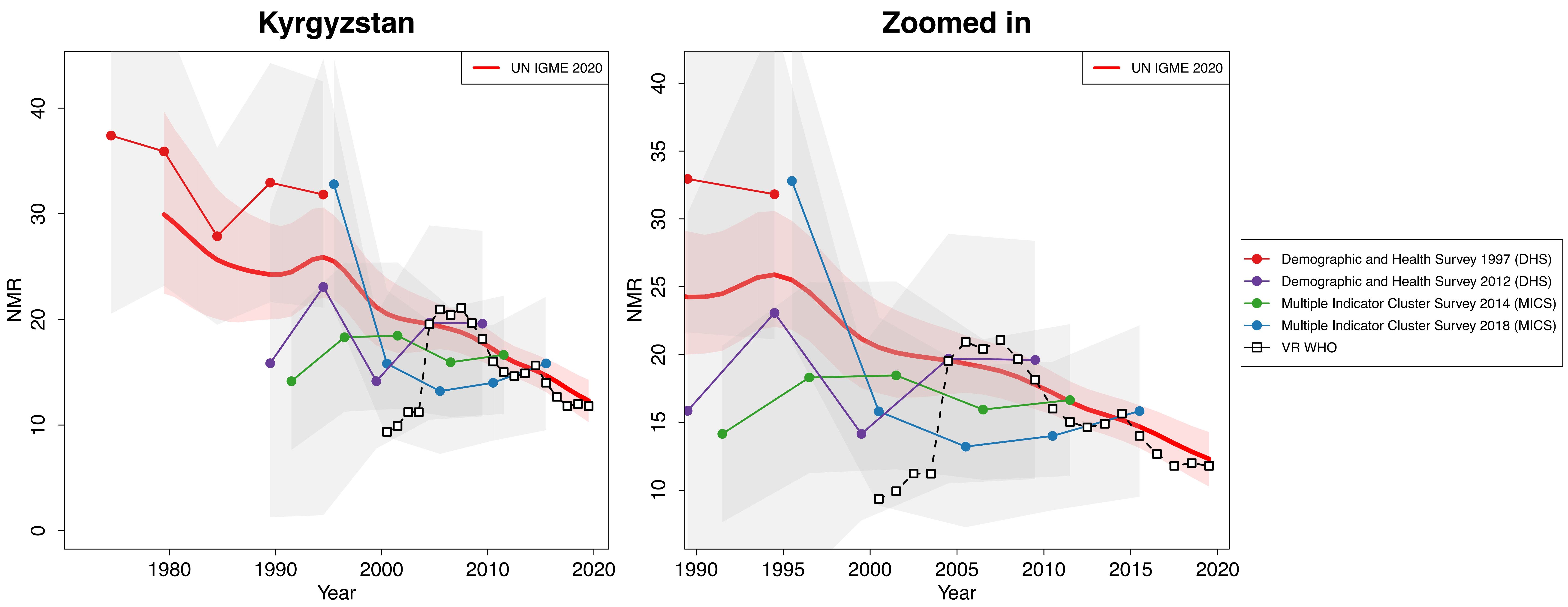

Kyrgyzstan
Zoomed in
UN IGME 2020
NMR
Year
Demographic and Health Survey 1997 (DHS)
Demographic and Health Survey 2012 (DHS)
Multiple Indicator Cluster Survey 2014 (MICS)
Multiple Indicator Cluster Survey 2018 (MICS)
VR WHO

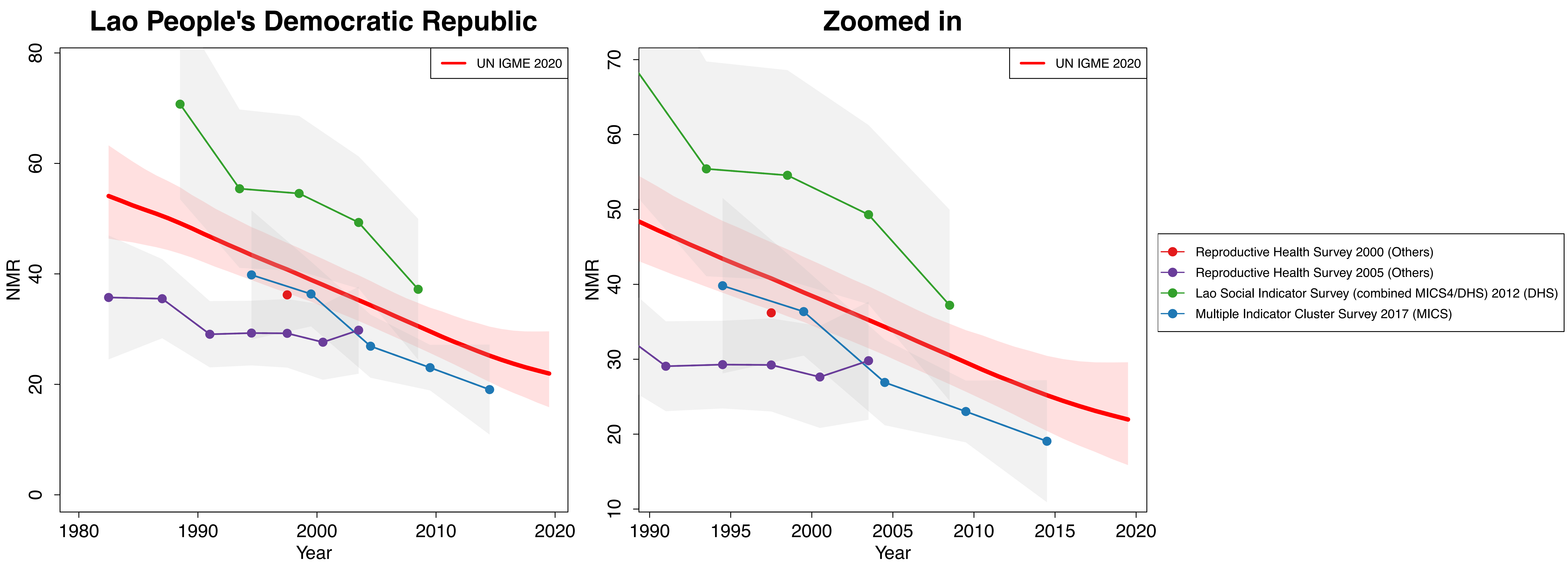

Lao People's Democratic Republic
Zoomed in
UN IGME 2020
NMR
Year
Reproductive Health Survey 2000 (Others)
Reproductive Health Survey 2005 (Others)
Lao Social Indicator Survey (combined MICS4/DHS) 2012 (DHS)
Multiple Indicator Cluster Survey 2017 (MICS)

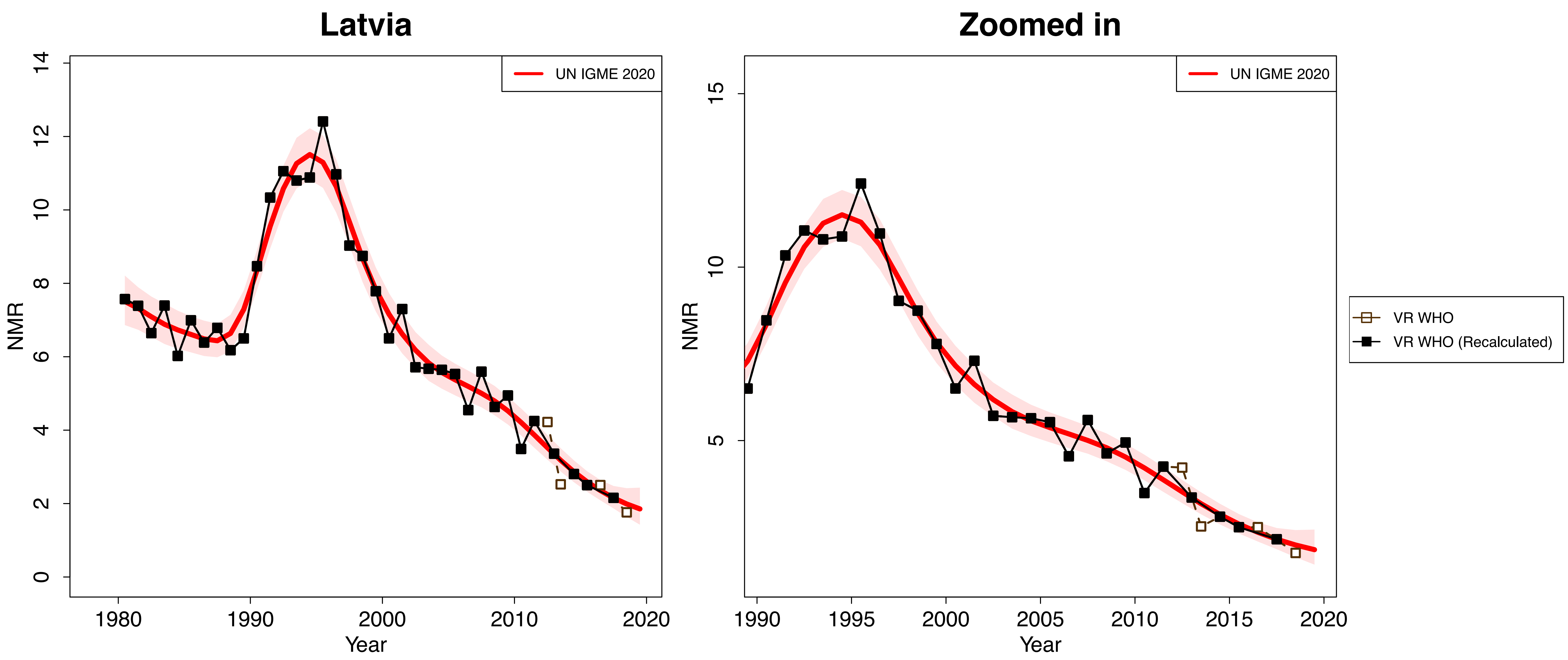

Latvia
Zoomed in
UN IGME 2020
NMR
Year
VR WHO
VR WHO (Recalculated)

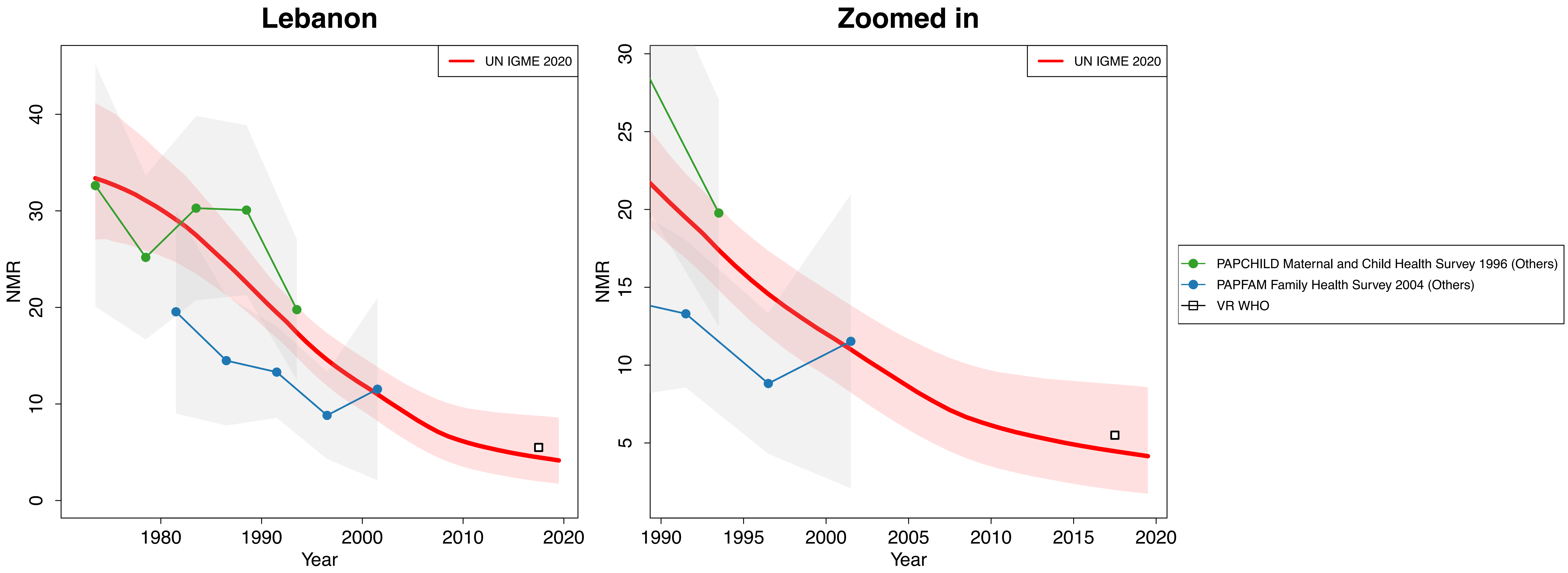
Lebanon
Zoomed in
UN IGME 2020
UN IGME 2020
PAPCHILD Maternal and Child Health Survey 1996 (Others)
PAPFAM Family Health Survey 2004 (Others)
VR WHO
NMR
NMR
Year
Year
0
10
20
30
40
1980
1990
2000
2010
2020
5
10
15
20
25
30
1990
1995
2000
2005
2010
2015
2020

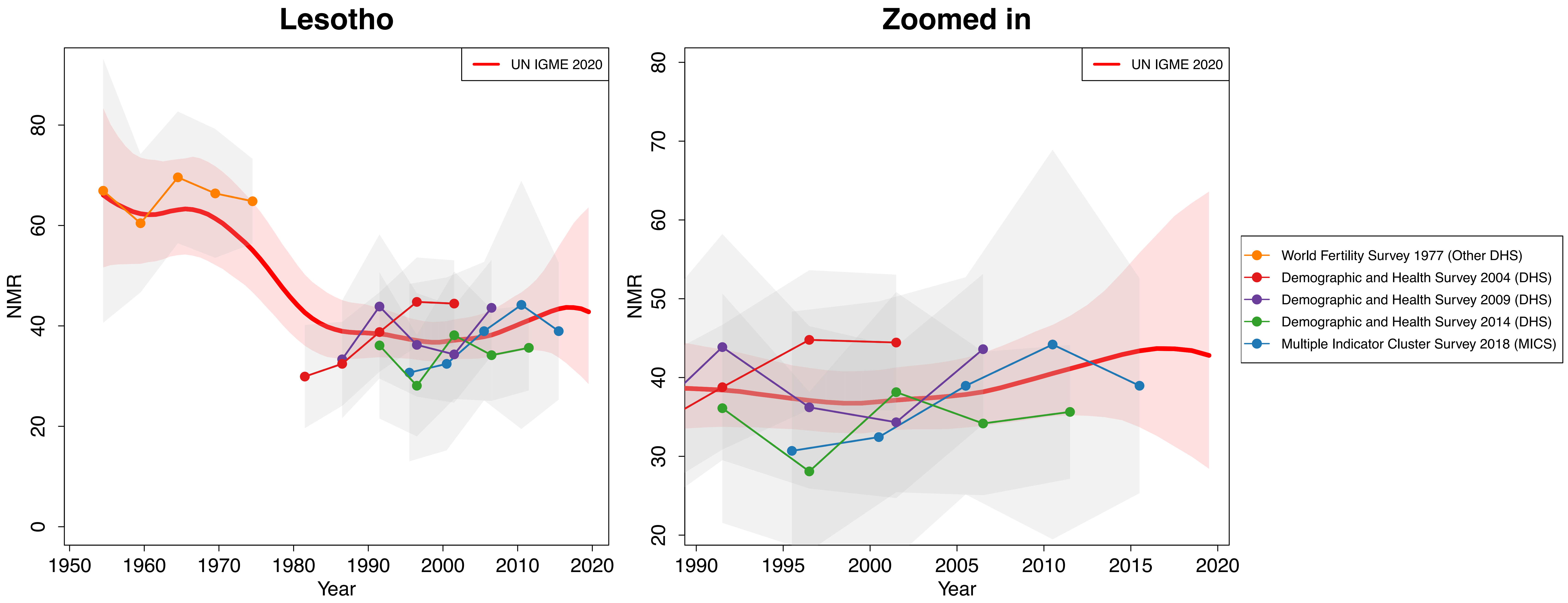

Lesotho
Zoomed in
UN IGME 2020
NMR
Year
World Fertility Survey 1977 (Other DHS)
Demographic and Health Survey 2004 (DHS)
Demographic and Health Survey 2009 (DHS)
Demographic and Health Survey 2014 (DHS)
Multiple Indicator Cluster Survey 2018 (MICS)

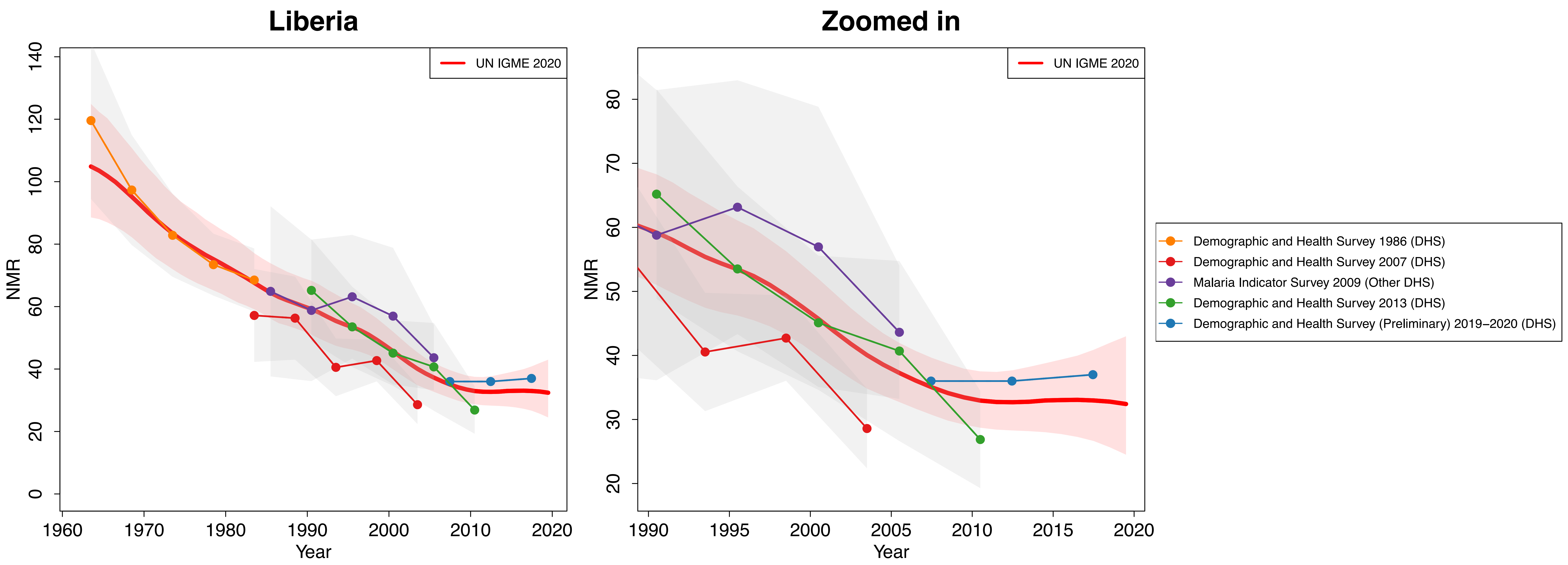
Liberia
Zoomed in
UN IGME 2020
NMR
Year
Demographic and Health Survey 1986 (DHS)
Demographic and Health Survey 2007 (DHS)
Malaria Indicator Survey 2009 (Other DHS)
Demographic and Health Survey 2013 (DHS)
Demographic and Health Survey (Preliminary) 2019–2020 (DHS)

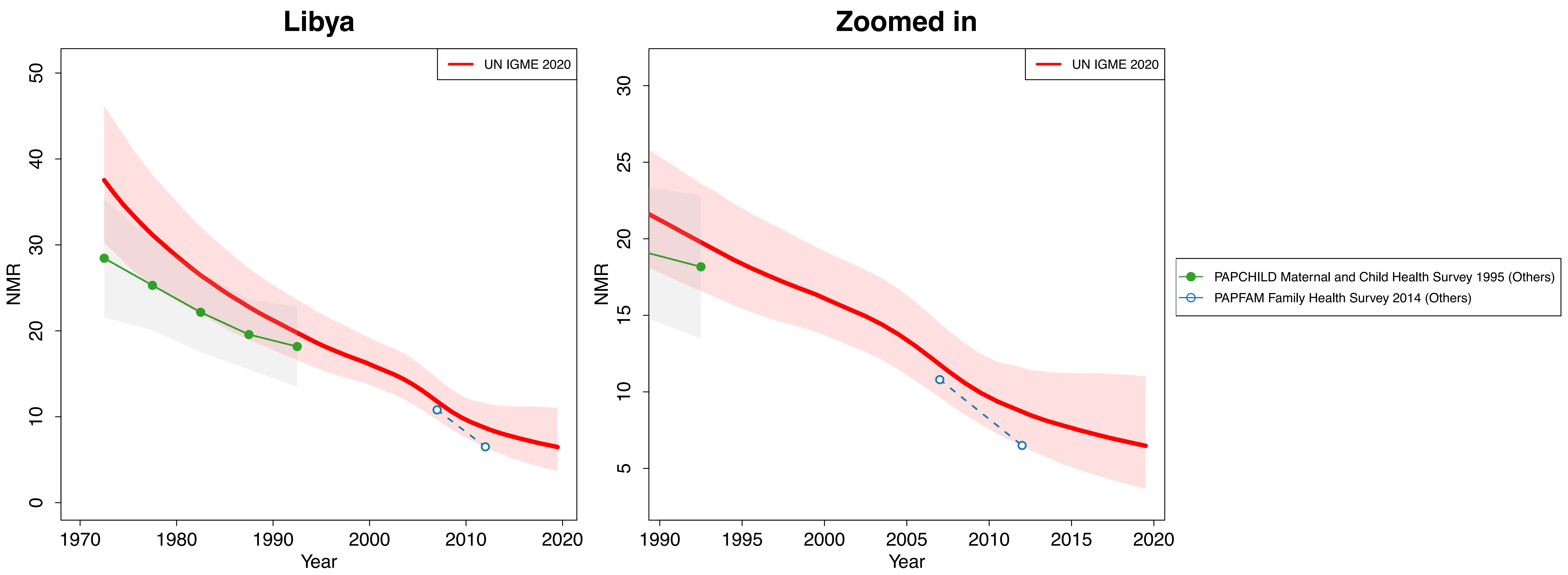
Libya
Zoomed in
UN IGME 2020
NMR
Year
PAPCHILD Maternal and Child Health Survey 1995 (Others)
PAPFAM Family Health Survey 2014 (Others)

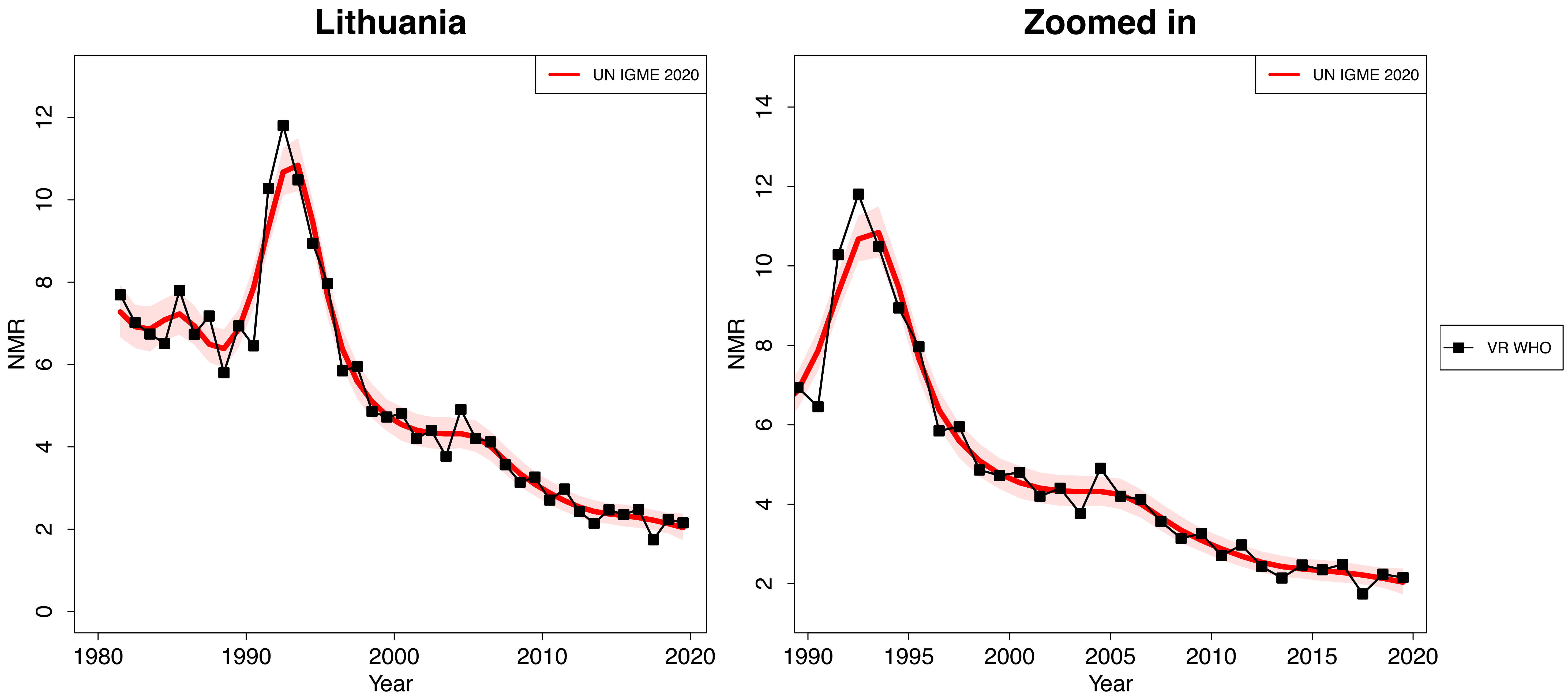

Lithuania
UN IGME 2020
NMR
Year
Zoomed in
UN IGME 2020
NMR
Year
VR WHO

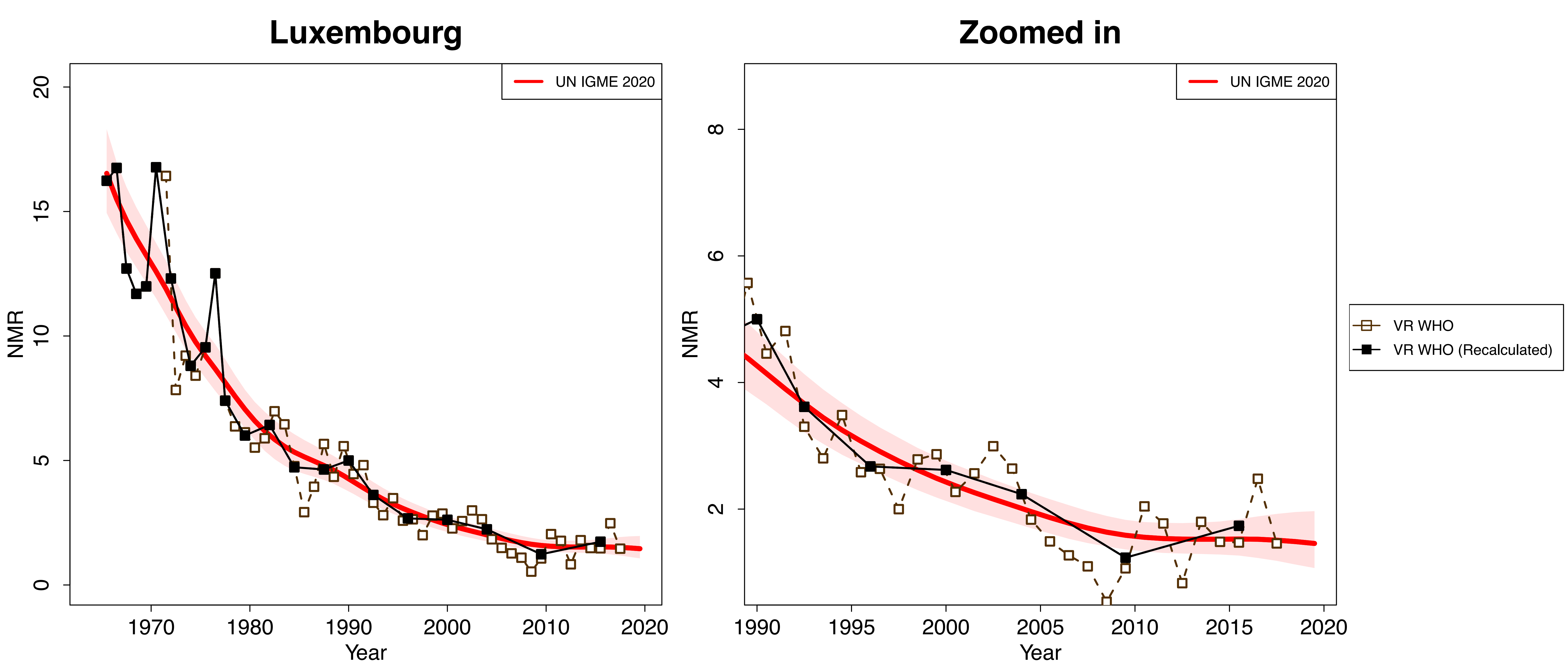

Luxembourg
Zoomed in
UN IGME 2020
NMR
Year
VR WHO
VR WHO (Recalculated)

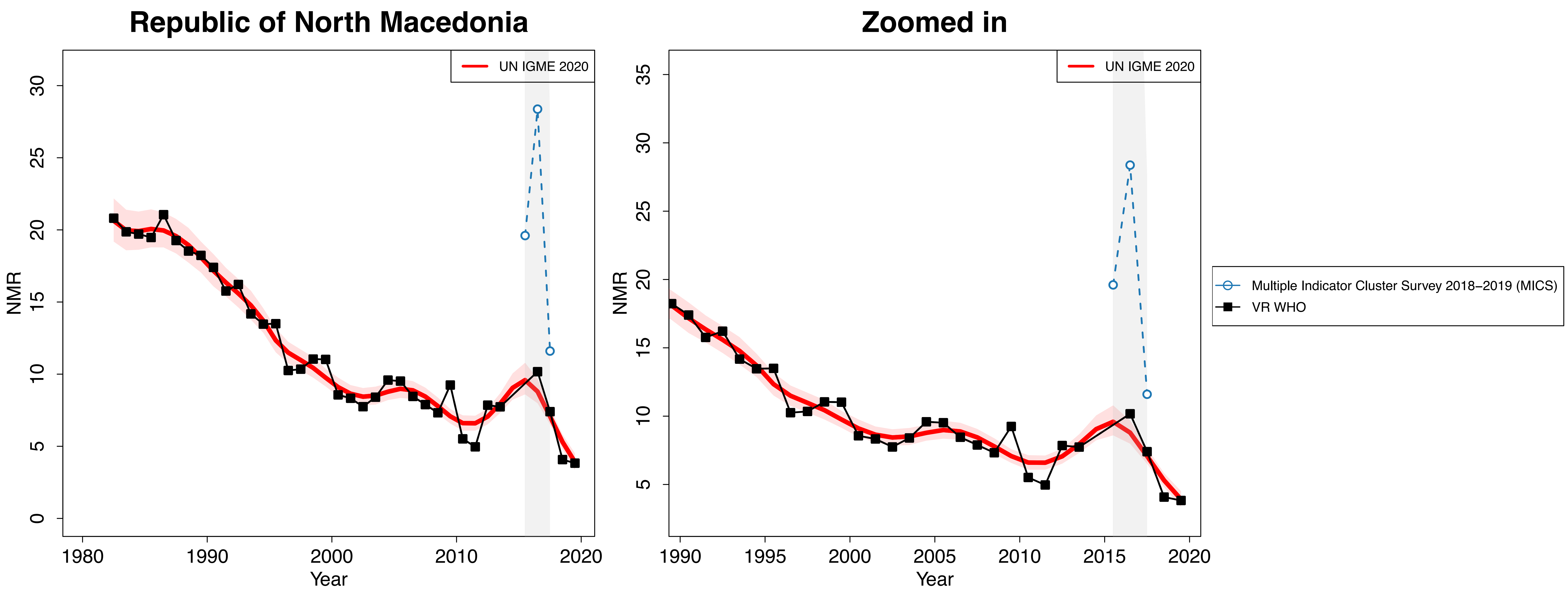

Republic of North Macedonia
Zoomed in
UN IGME 2020
NMR
Year
Multiple Indicator Cluster Survey 2018–2019 (MICS)
VR WHO

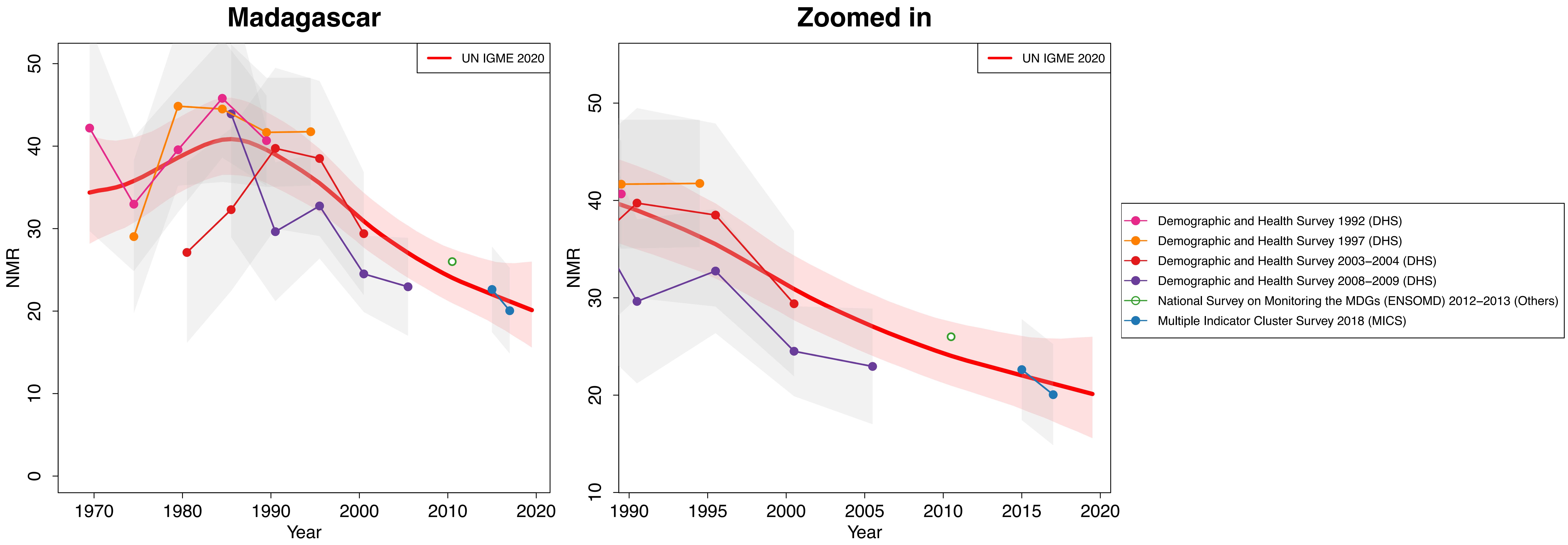

Madagascar
Zoomed in
UN IGME 2020
NMR
Year
Demographic and Health Survey 1992 (DHS)
Demographic and Health Survey 1997 (DHS)
Demographic and Health Survey 2003–2004 (DHS)
Demographic and Health Survey 2008–2009 (DHS)
National Survey on Monitoring the MDGs (ENSOMD) 2012–2013 (Others)
Multiple Indicator Cluster Survey 2018 (MICS)

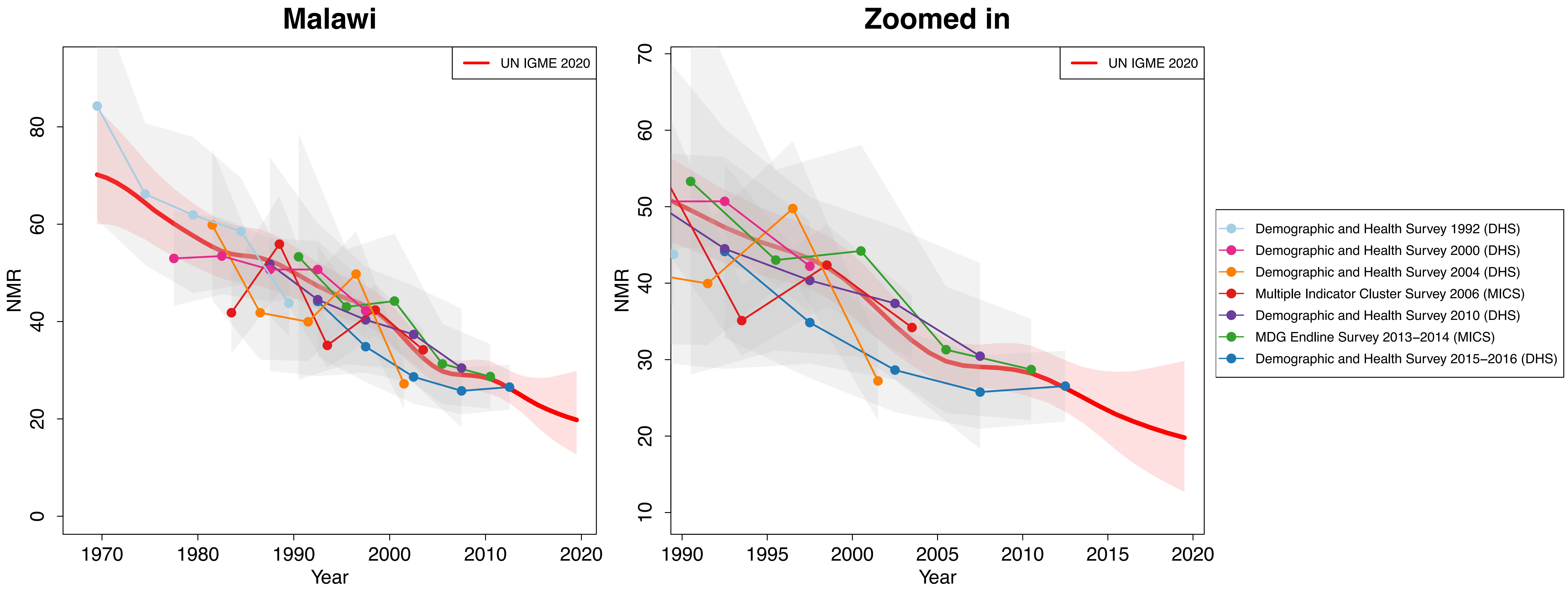
Malawi
Zoomed in
UN IGME 2020
NMR
Year
1970
1980
1990
2000
2010
2020
0
20
40
60
80
1995
2005
2015
10
30
50
70
Demographic and Health Survey 1992 (DHS)
Demographic and Health Survey 2000 (DHS)
Demographic and Health Survey 2004 (DHS)
Multiple Indicator Cluster Survey 2006 (MICS)
Demographic and Health Survey 2010 (DHS)
MDG Endline Survey 2013–2014 (MICS)
Demographic and Health Survey 2015–2016 (DHS)

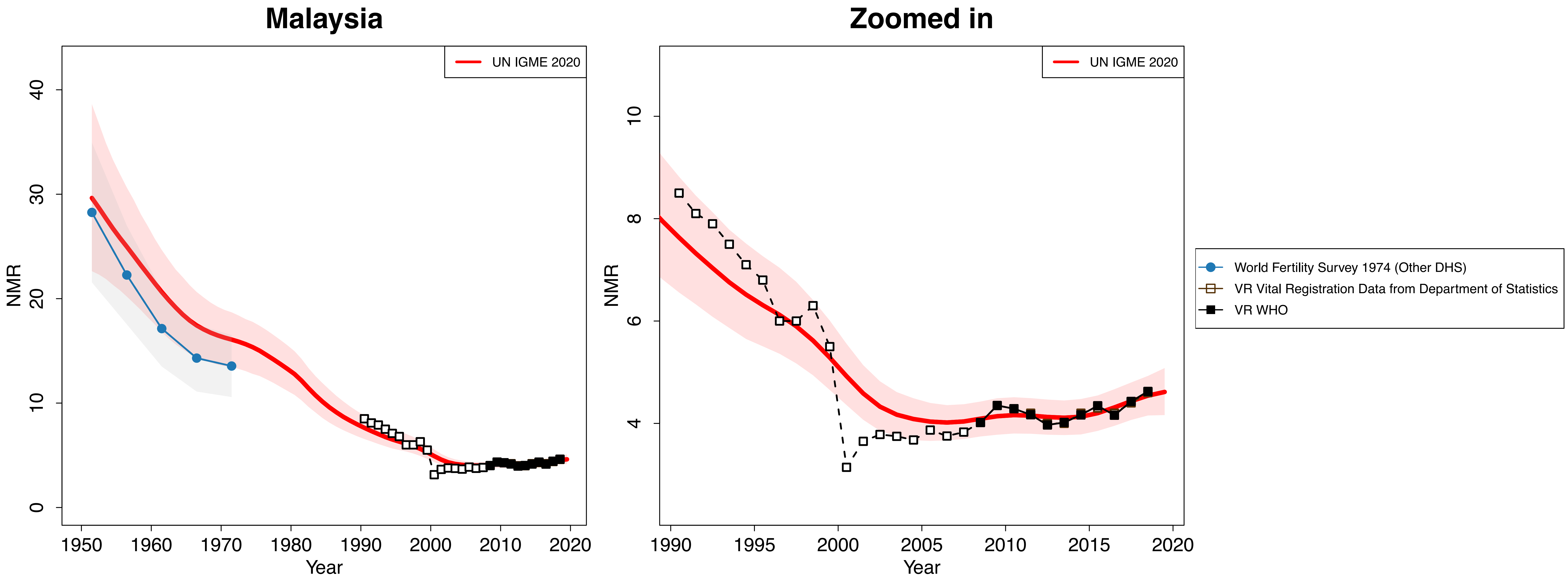

Malaysia
Zoomed in
UN IGME 2020
NMR
Year
World Fertility Survey 1974 (Other DHS)
VR Vital Registration Data from Department of Statistics
VR WHO

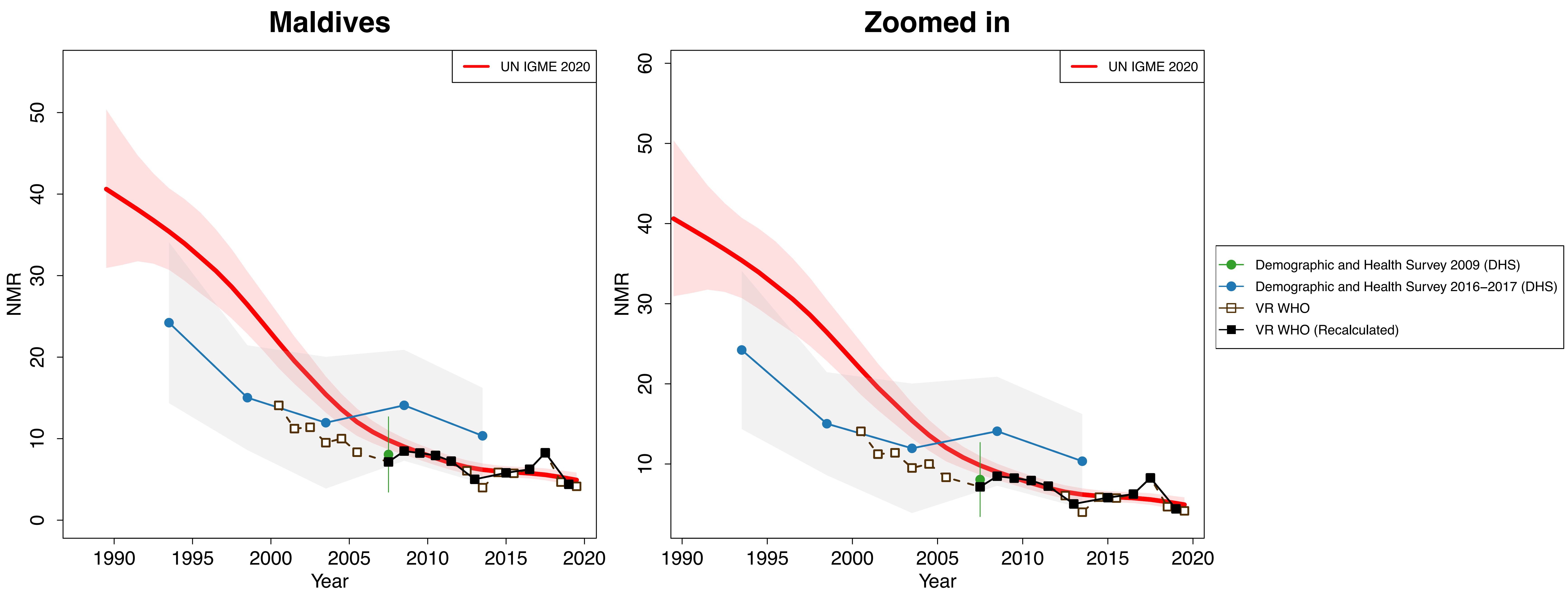

Maldives
Zoomed in
UN IGME 2020
NMR
Year
Demographic and Health Survey 2009 (DHS)
Demographic and Health Survey 2016–2017 (DHS)
VR WHO
VR WHO (Recalculated)

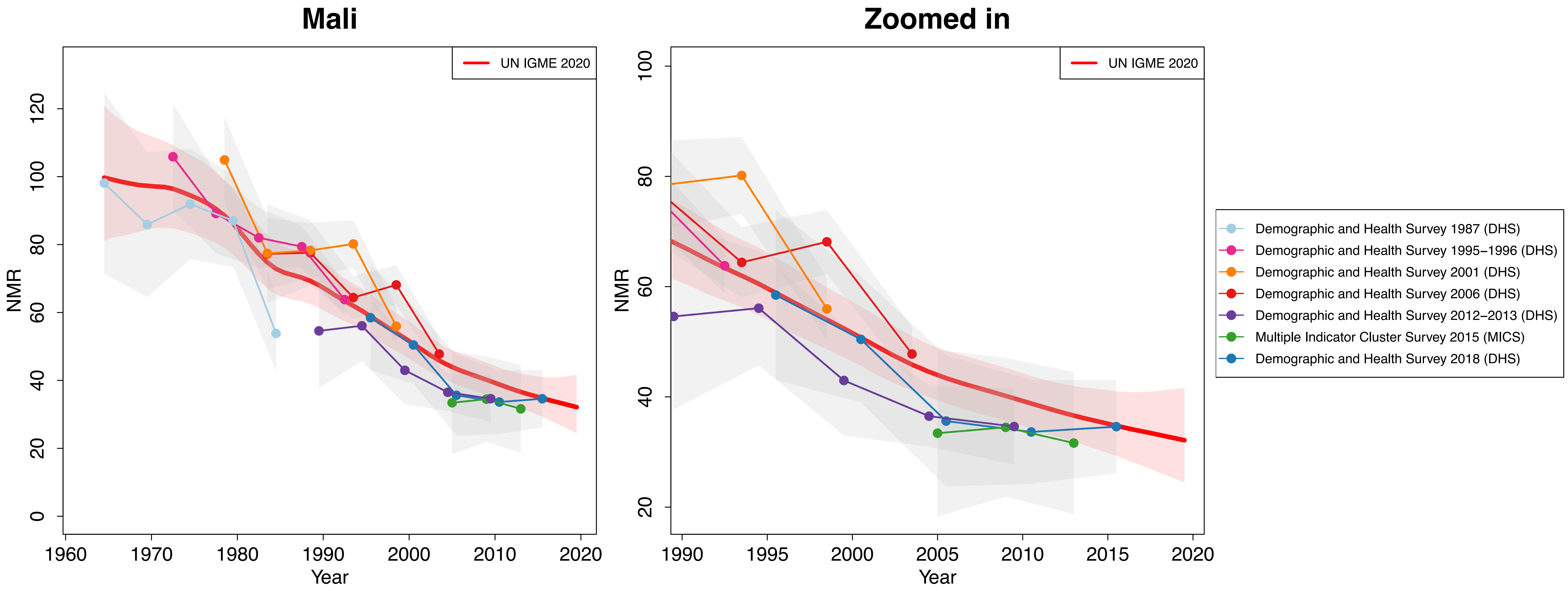
Mali
Zoomed in
UN IGME 2020
NMR
Year
0
20
40
60
80
100
120
1960
1970
1980
1990
2000
2010
2020
1995
2005
2015
Demographic and Health Survey 1987 (DHS)
Demographic and Health Survey 1995–1996 (DHS)
Demographic and Health Survey 2001 (DHS)
Demographic and Health Survey 2006 (DHS)
Demographic and Health Survey 2012–2013 (DHS)
Multiple Indicator Cluster Survey 2015 (MICS)
Demographic and Health Survey 2018 (DHS)

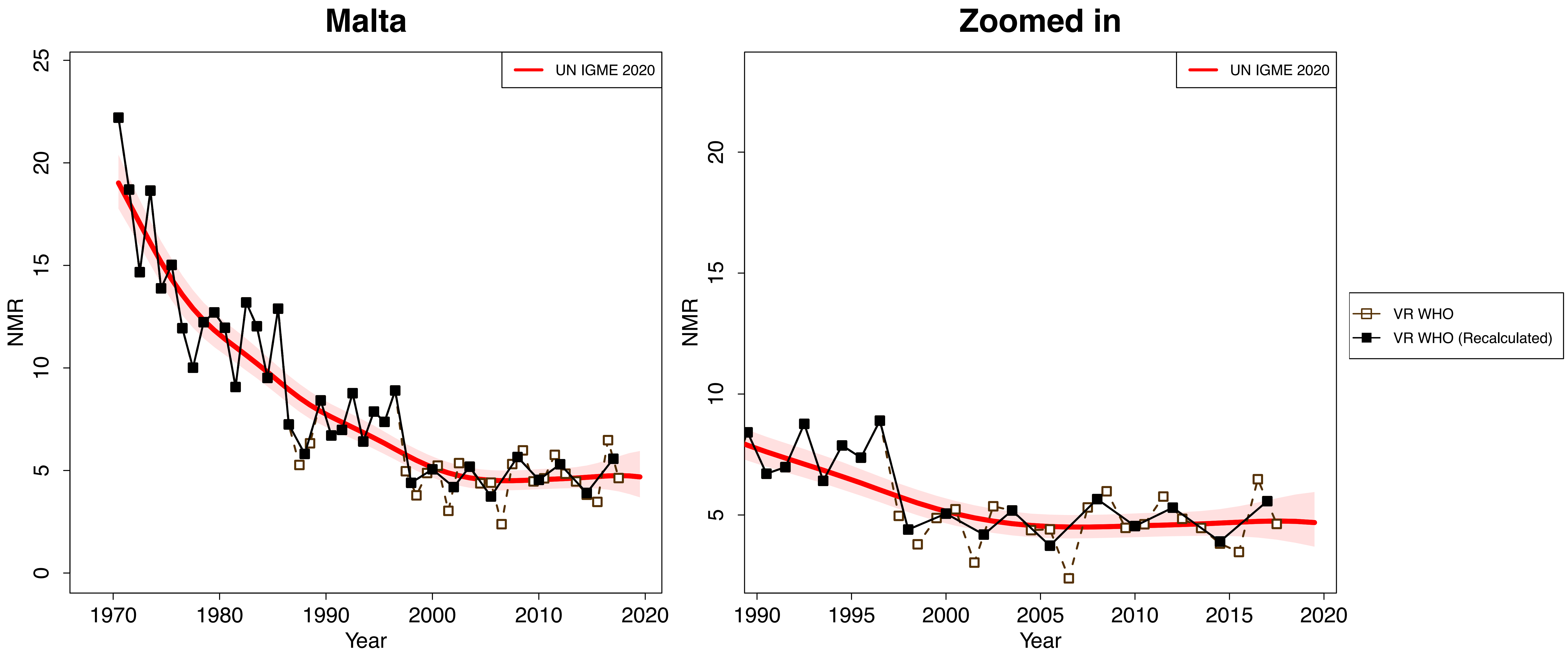
Malta
Zoomed in
UN IGME 2020
UN IGME 2020
NMR
NMR
Year
Year
0
5
10
15
20
25
1970
1980
1990
2000
2010
2020
5
10
15
20
1990
1995
2000
2005
2010
2015
2020
VR WHO
VR WHO (Recalculated)

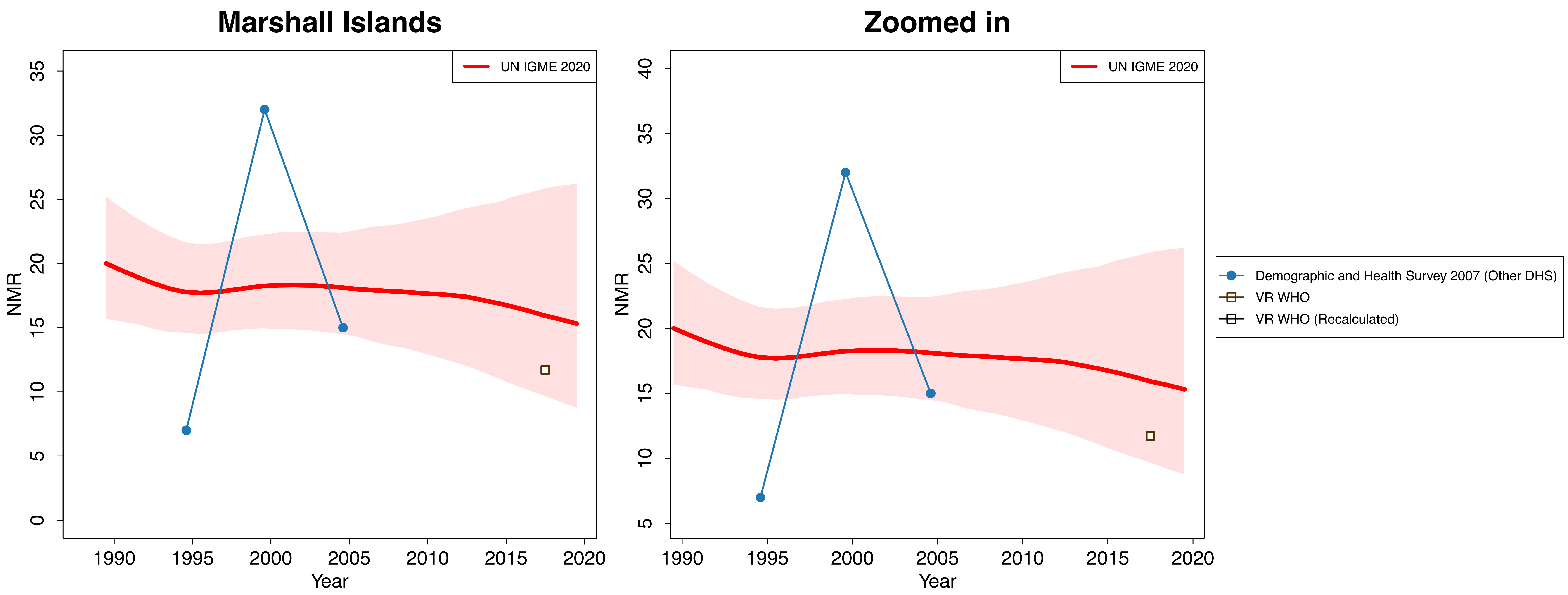

Marshall Islands
Zoomed in
UN IGME 2020
UN IGME 2020
NMR
Year
1990
1995
2000
2005
2010
2015
2020
Demographic and Health Survey 2007 (Other DHS)
VR WHO
VR WHO (Recalculated)

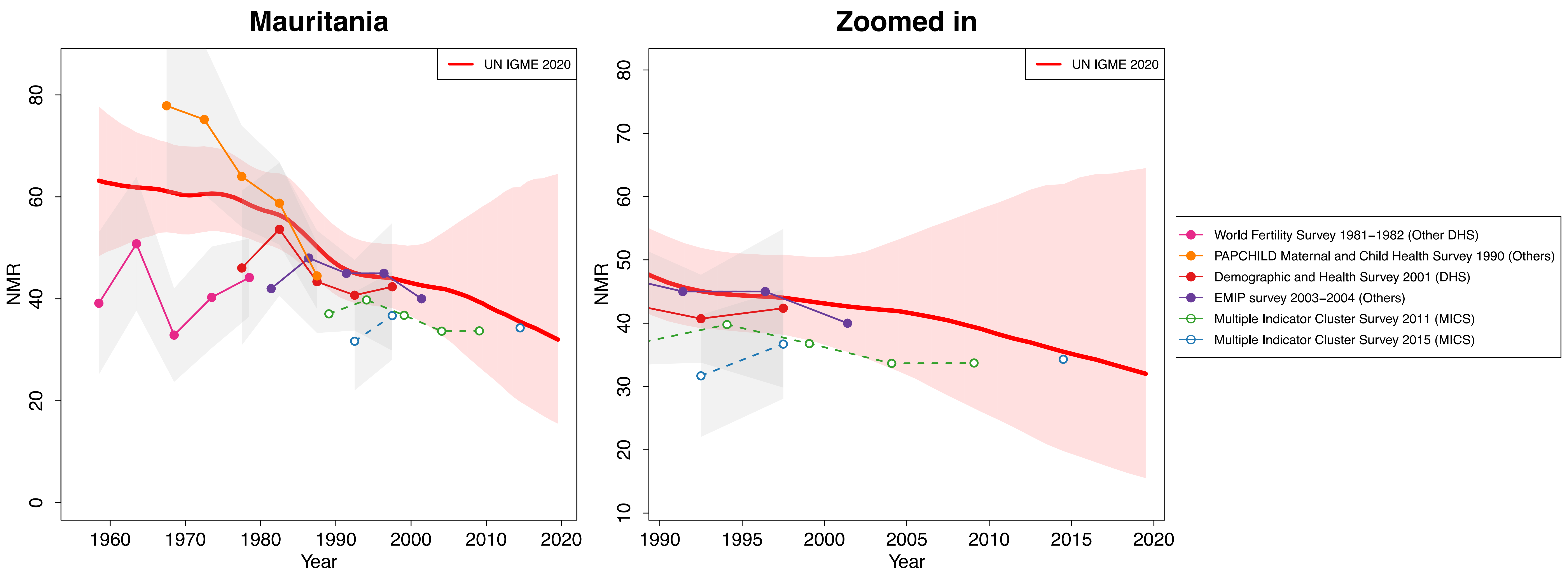

Mauritania
Zoomed in
UN IGME 2020
NMR
Year
World Fertility Survey 1981–1982 (Other DHS)
PAPCHILD Maternal and Child Health Survey 1990 (Others)
Demographic and Health Survey 2001 (DHS)
EMIP survey 2003–2004 (Others)
Multiple Indicator Cluster Survey 2011 (MICS)
Multiple Indicator Cluster Survey 2015 (MICS)

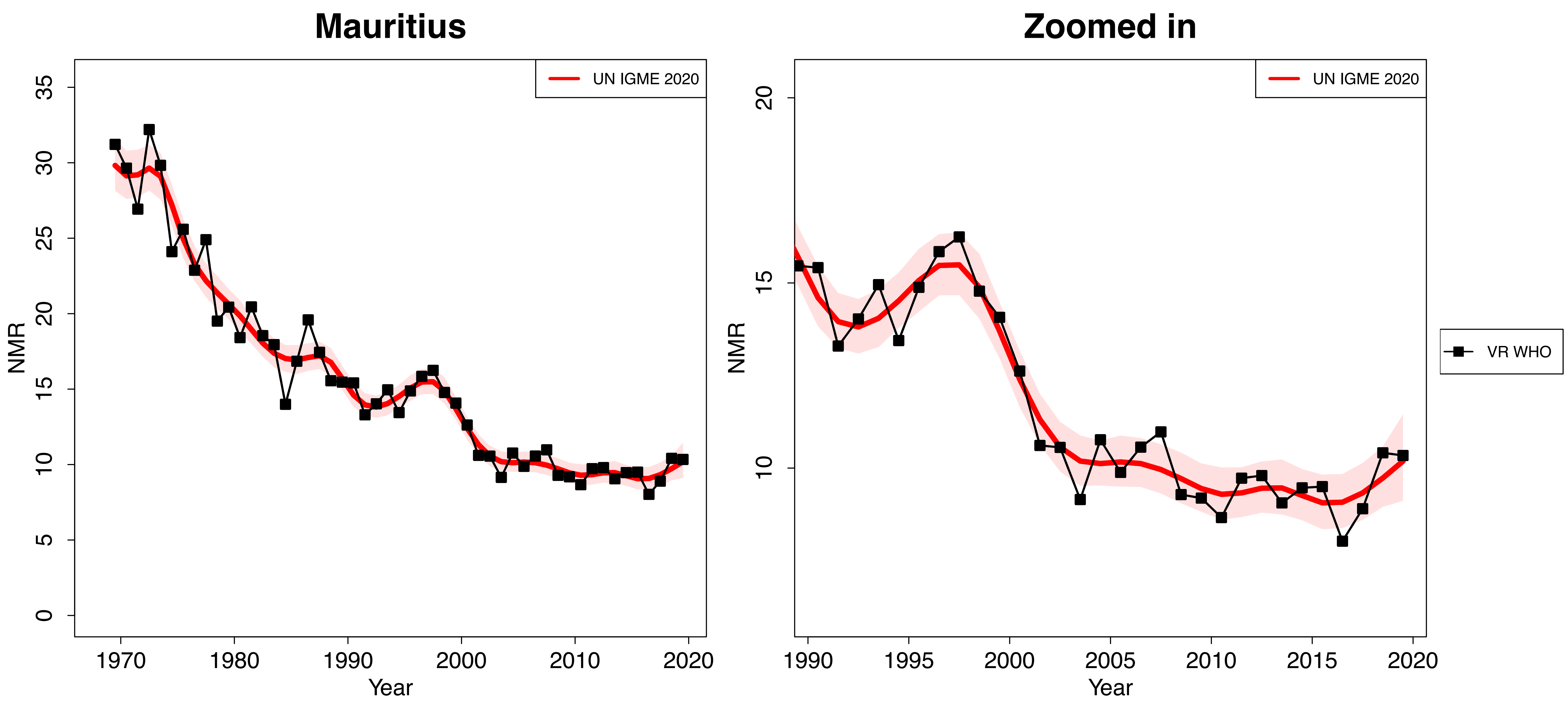

Mauritius
Zoomed in
UN IGME 2020
VR WHO
NMR
Year

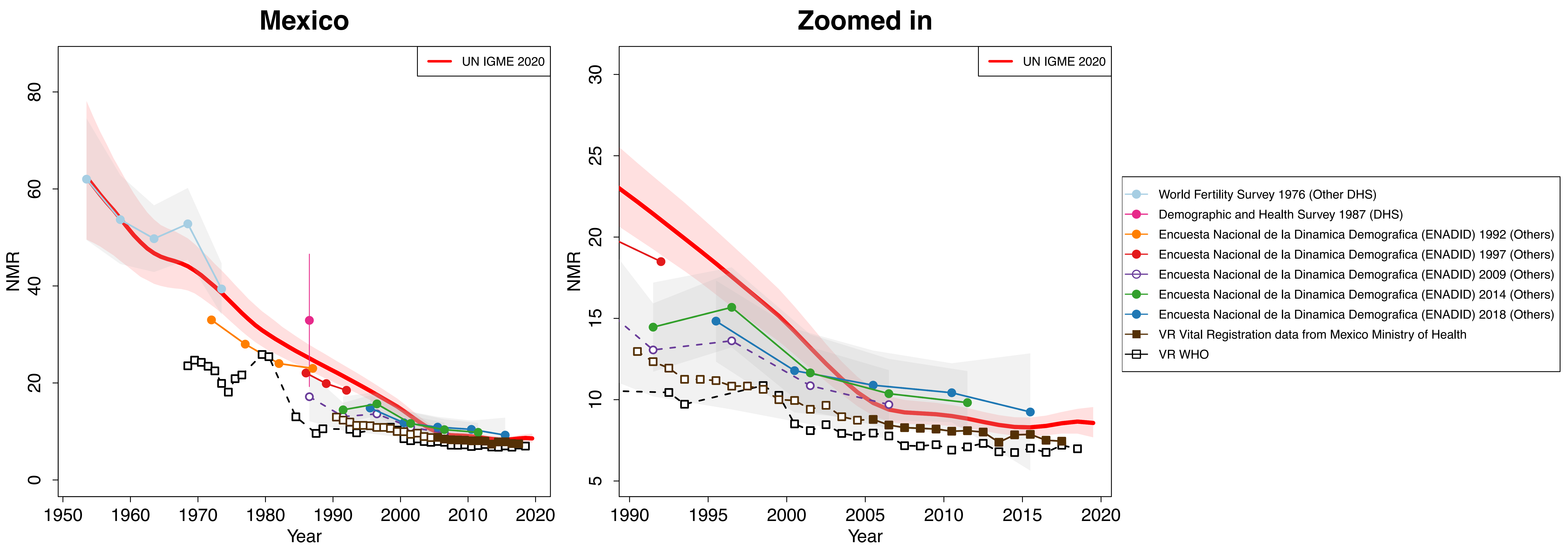

Mexico
Zoomed in
UN IGME 2020
NMR
Year
World Fertility Survey 1976 (Other DHS)
Demographic and Health Survey 1987 (DHS)
Encuesta Nacional de la Dinamica Demografica (ENADID) 1992 (Others)
Encuesta Nacional de la Dinamica Demografica (ENADID) 1997 (Others)
Encuesta Nacional de la Dinamica Demografica (ENADID) 2009 (Others)
Encuesta Nacional de la Dinamica Demografica (ENADID) 2014 (Others)
Encuesta Nacional de la Dinamica Demografica (ENADID) 2018 (Others)
VR Vital Registration data from Mexico Ministry of Health
VR WHO

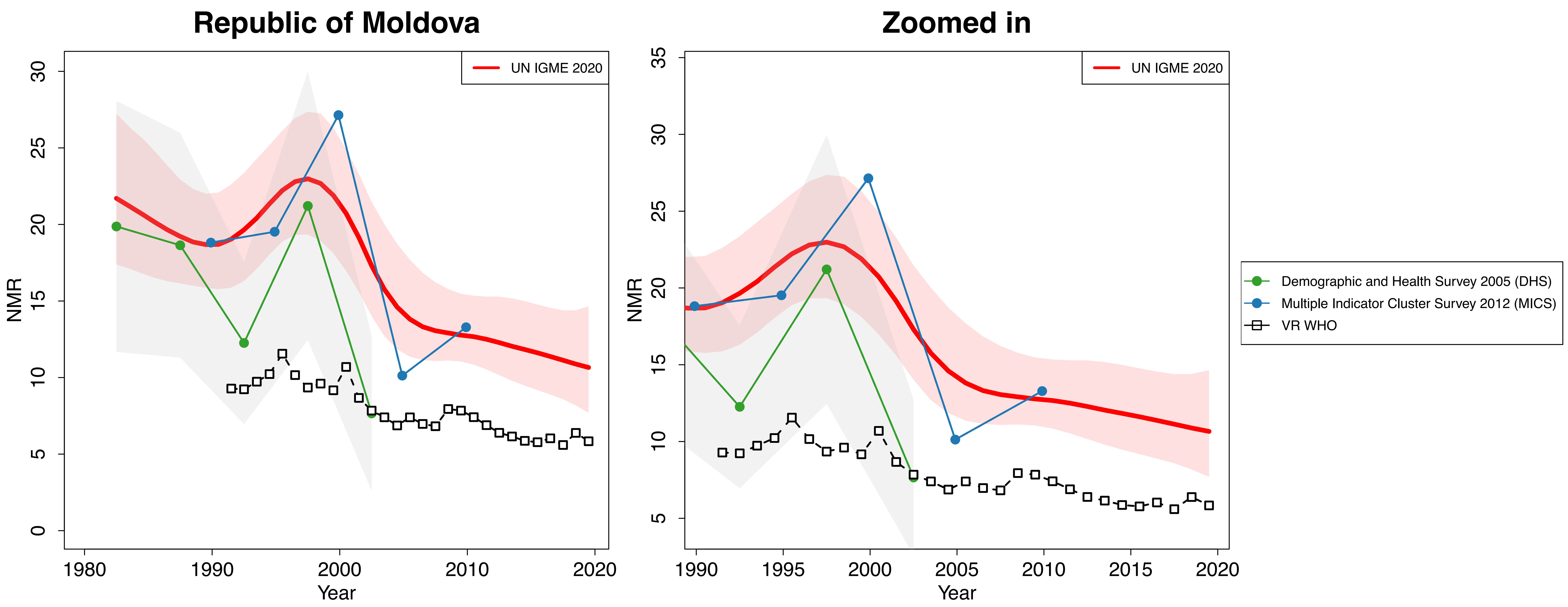

Republic of Moldova
Zoomed in
UN IGME 2020
NMR
Year
Demographic and Health Survey 2005 (DHS)
Multiple Indicator Cluster Survey 2012 (MICS)
VR WHO

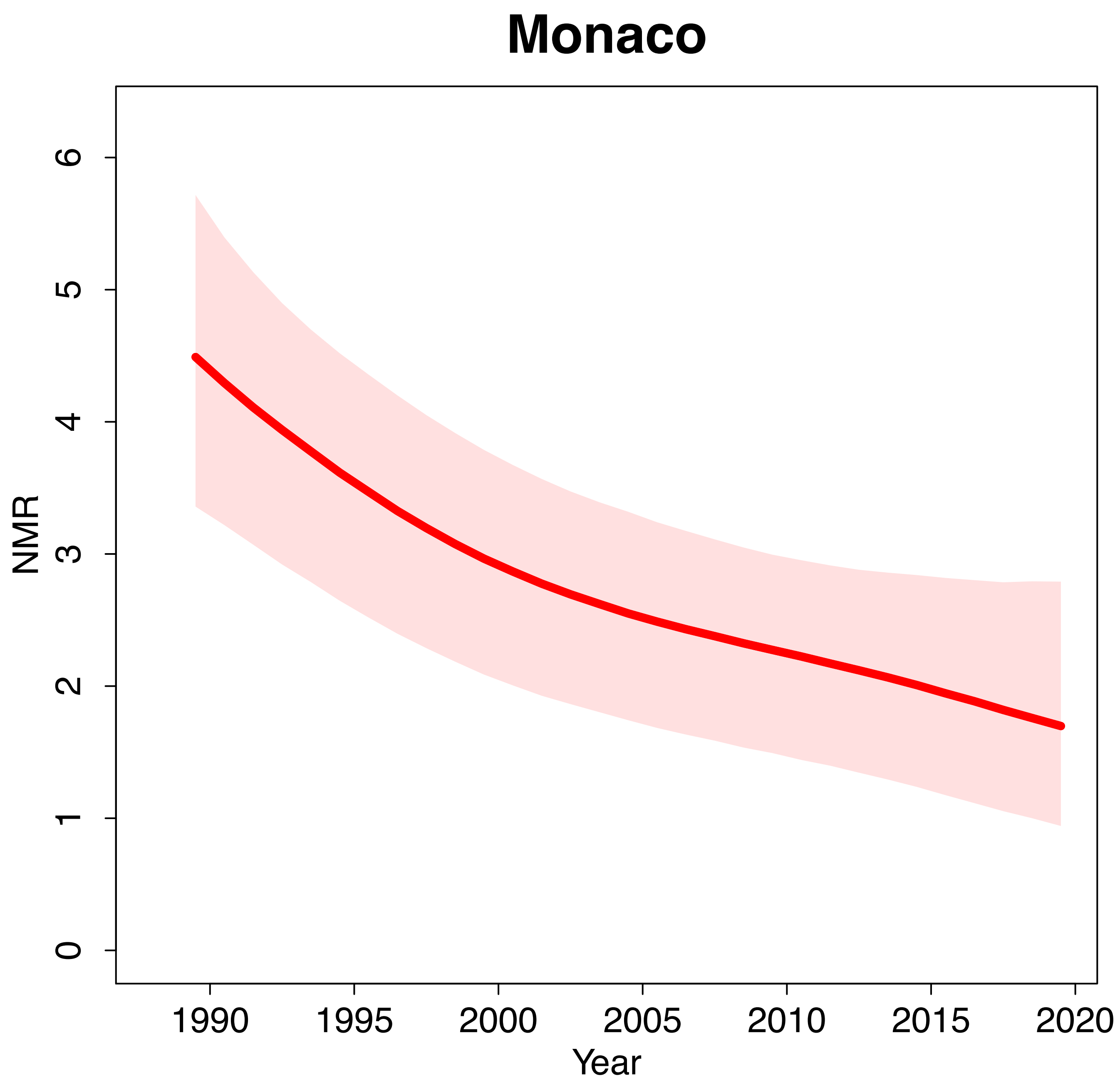

Monaco
NMR
0
1
2
3
4
5
6
1990
1995
2000
2005
2010
2015
2020
Year

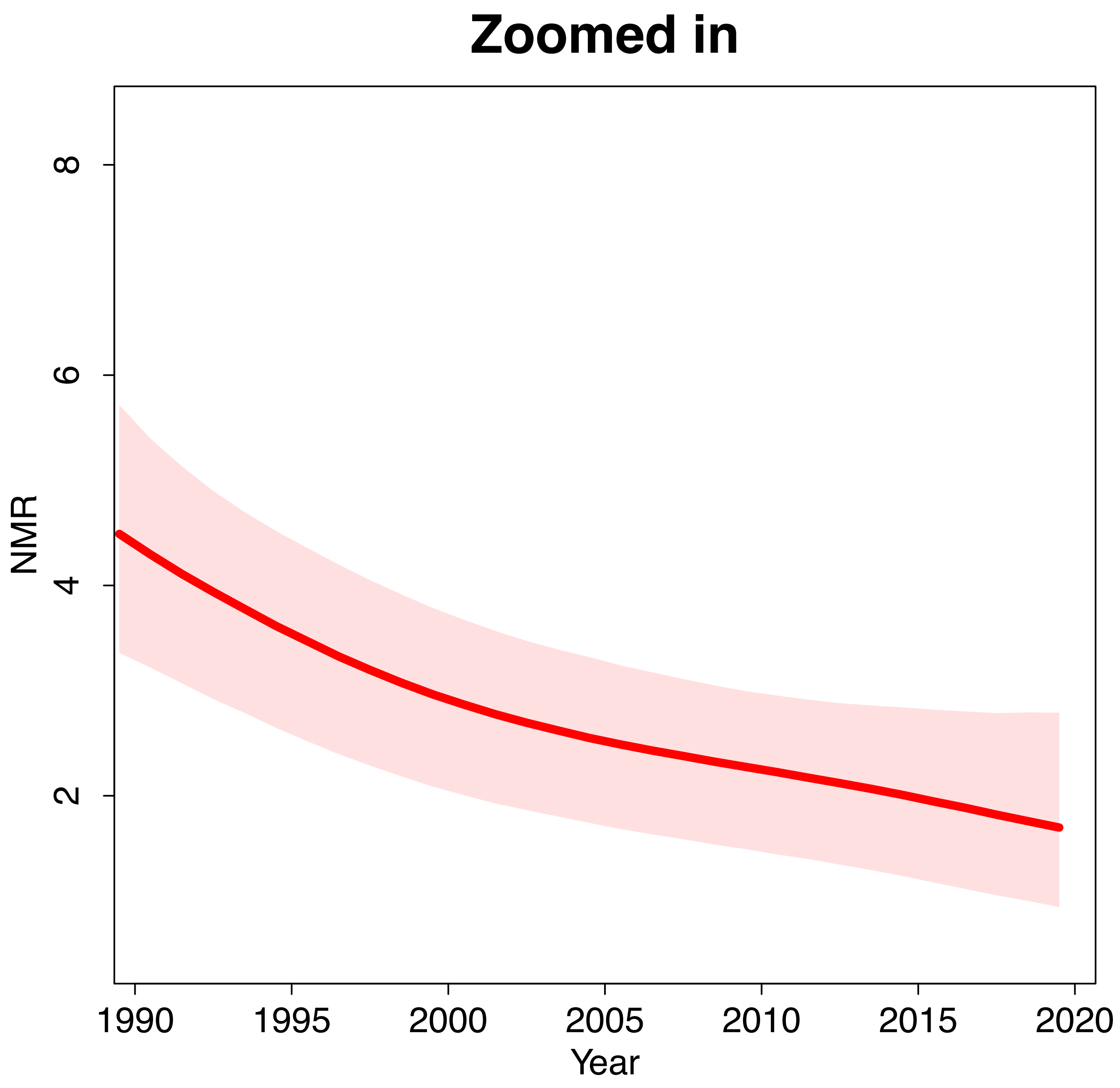

Zoomed in
NMR
2
4
6
8
1990
1995
2000
2005
2010
2015
2020
Year

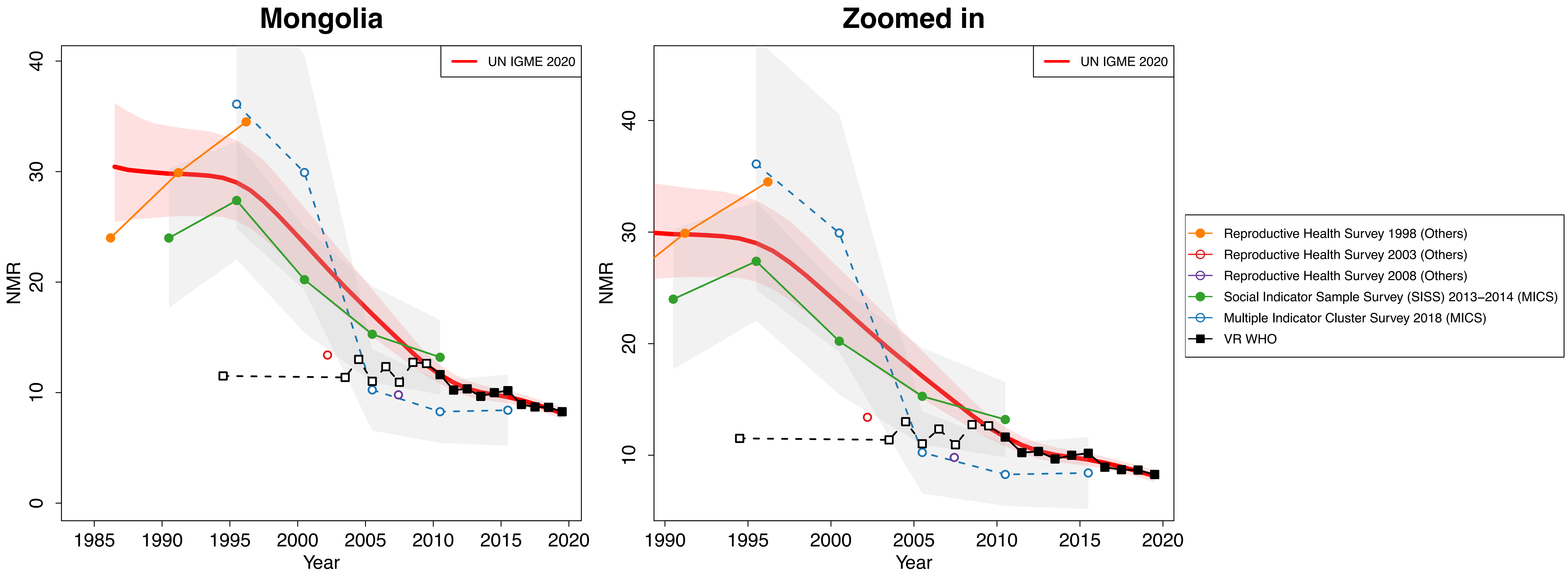

Mongolia
Zoomed in
UN IGME 2020
NMR
Year
Reproductive Health Survey 1998 (Others)
Reproductive Health Survey 2003 (Others)
Reproductive Health Survey 2008 (Others)
Social Indicator Sample Survey (SISS) 2013–2014 (MICS)
Multiple Indicator Cluster Survey 2018 (MICS)
VR WHO

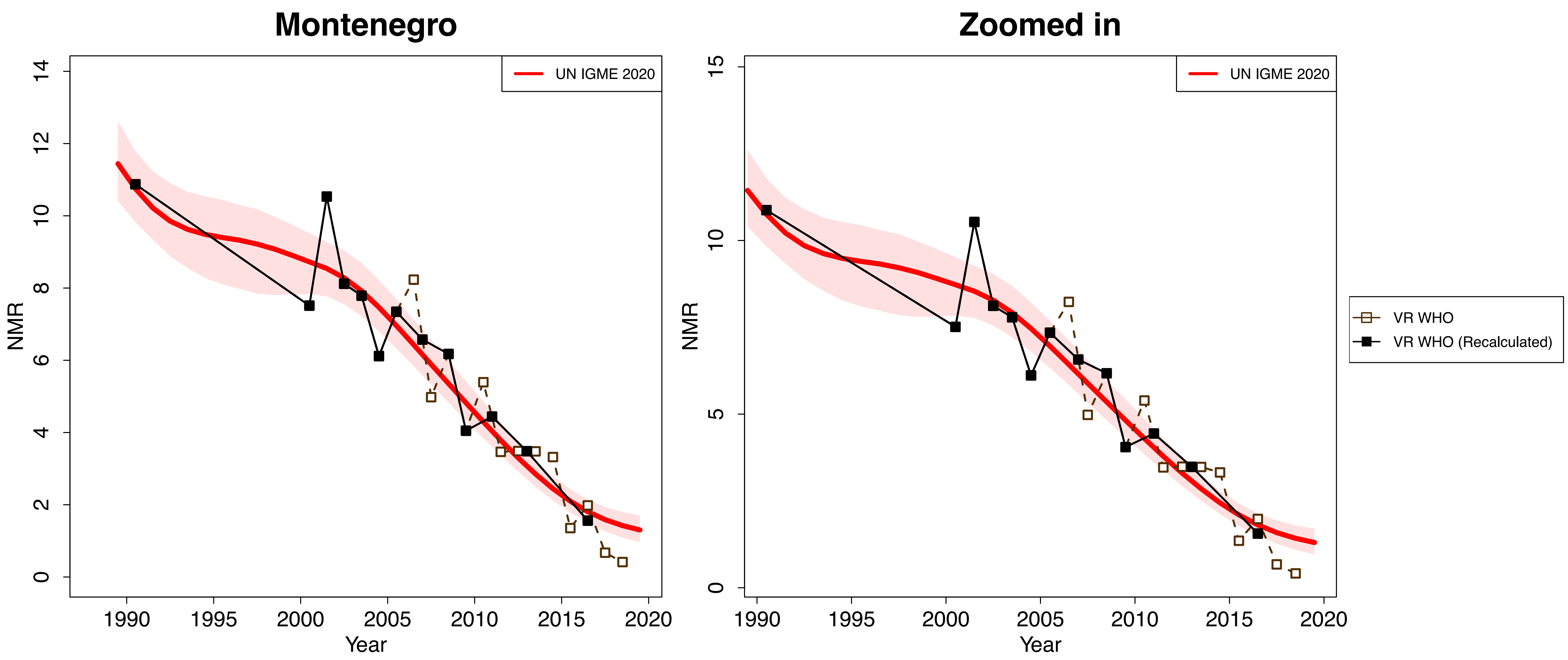
Montenegro
Zoomed in
UN IGME 2020
VR WHO
VR WHO (Recalculated)
NMR
Year

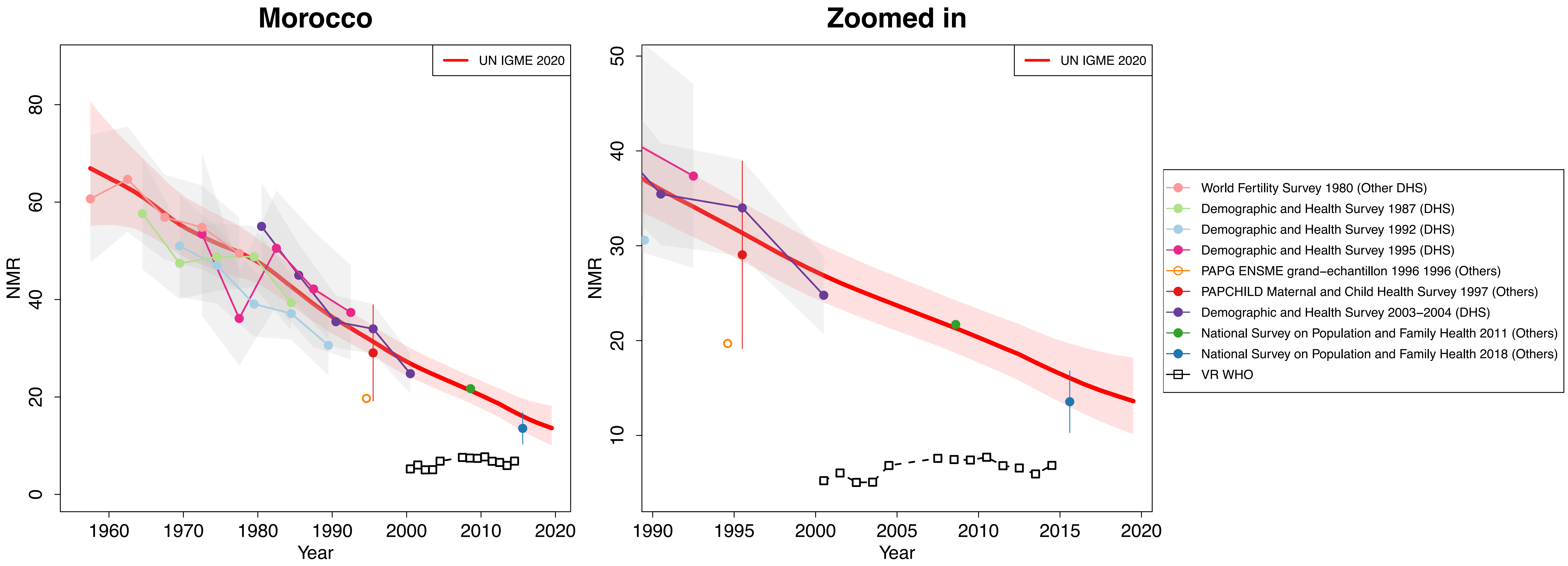

Morocco
Zoomed in
UN IGME 2020
NMR
Year
World Fertility Survey 1980 (Other DHS)
Demographic and Health Survey 1987 (DHS)
Demographic and Health Survey 1992 (DHS)
Demographic and Health Survey 1995 (DHS)
PAPG ENSME grand−echantillon 1996 1996 (Others)
PAPCHILD Maternal and Child Health Survey 1997 (Others)
Demographic and Health Survey 2003−2004 (DHS)
National Survey on Population and Family Health 2011 (Others)
National Survey on Population and Family Health 2018 (Others)
VR WHO

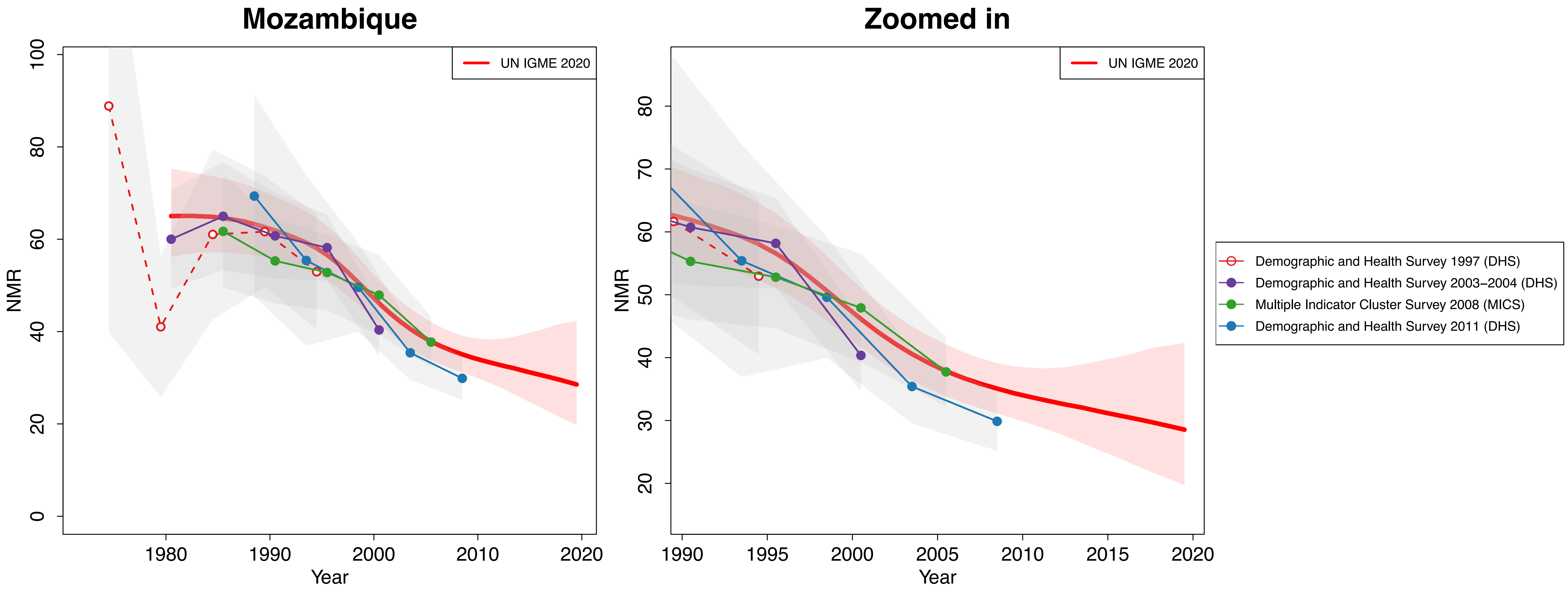

Mozambique
Zoomed in
UN IGME 2020
UN IGME 2020
NMR
NMR
Year
Year
Demographic and Health Survey 1997 (DHS)
Demographic and Health Survey 2003–2004 (DHS)
Multiple Indicator Cluster Survey 2008 (MICS)
Demographic and Health Survey 2011 (DHS)

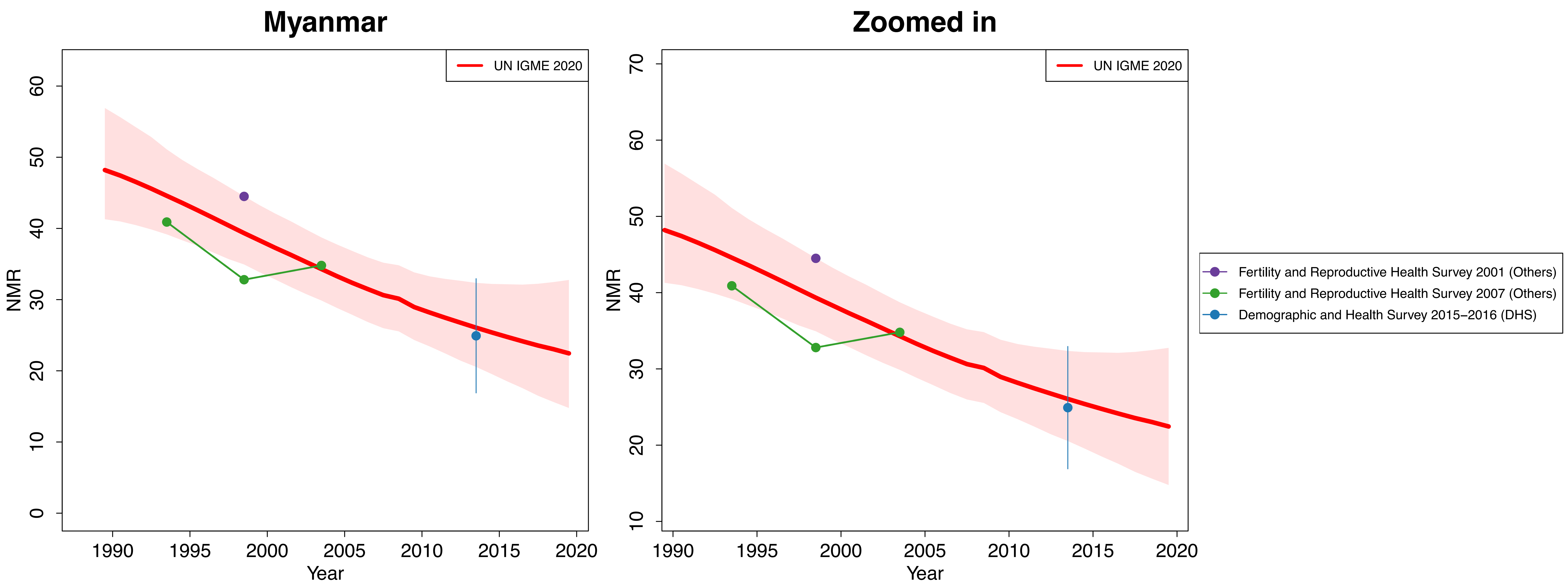

Myanmar
UN IGME 2020
NMR
Year
Zoomed in
UN IGME 2020
Fertility and Reproductive Health Survey 2001 (Others)
Fertility and Reproductive Health Survey 2007 (Others)
Demographic and Health Survey 2015–2016 (DHS)

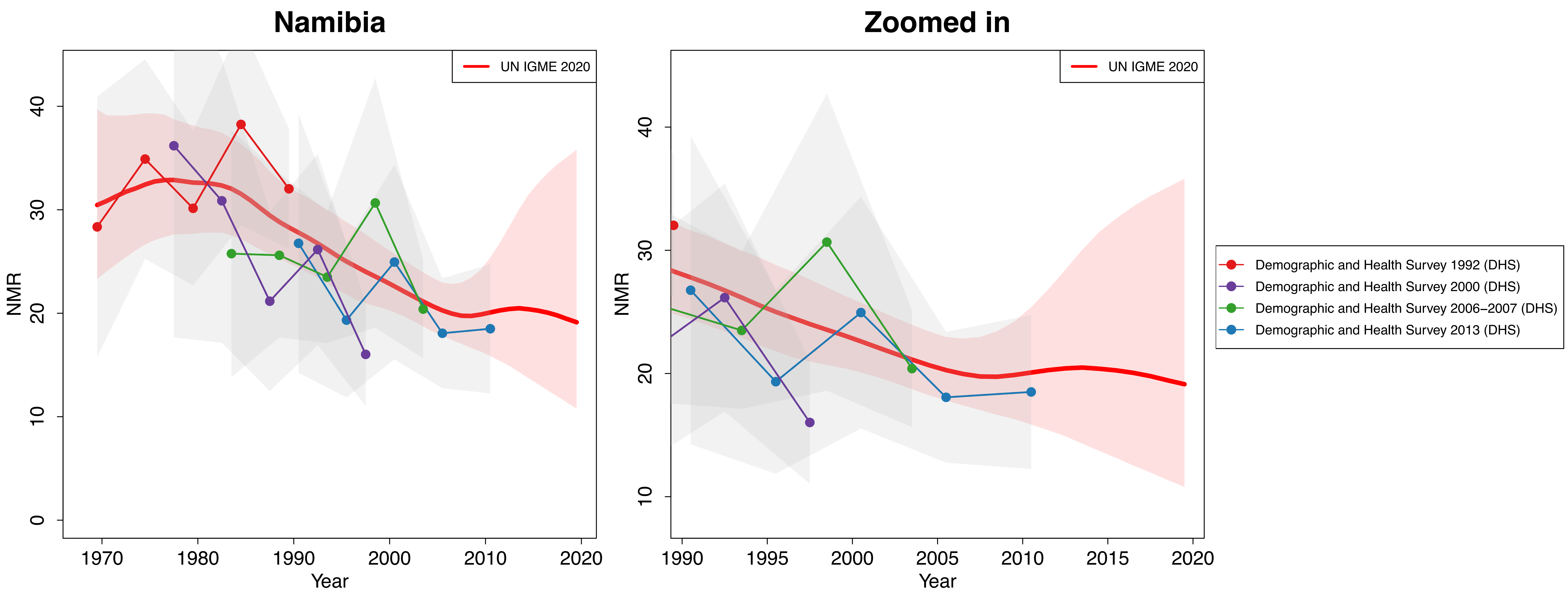
Namibia
Zoomed in
UN IGME 2020
UN IGME 2020
NMR
NMR
Year
Year
0
10
20
30
40
1970
1980
1990
2000
2010
2020
1990
1995
2000
2005
2010
2015
2020
Demographic and Health Survey 1992 (DHS)
Demographic and Health Survey 2000 (DHS)
Demographic and Health Survey 2006–2007 (DHS)
Demographic and Health Survey 2013 (DHS)

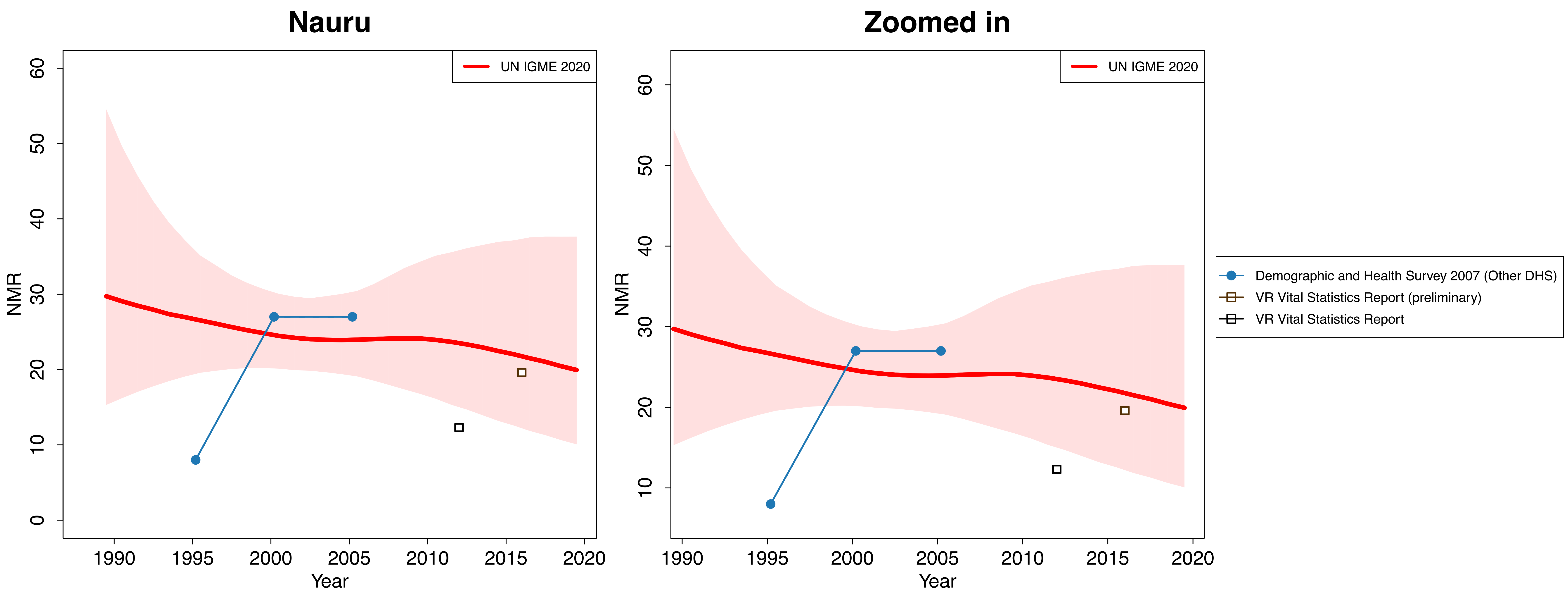

Nauru
Zoomed in
UN IGME 2020
NMR
Year
Demographic and Health Survey 2007 (Other DHS)
VR Vital Statistics Report (preliminary)
VR Vital Statistics Report

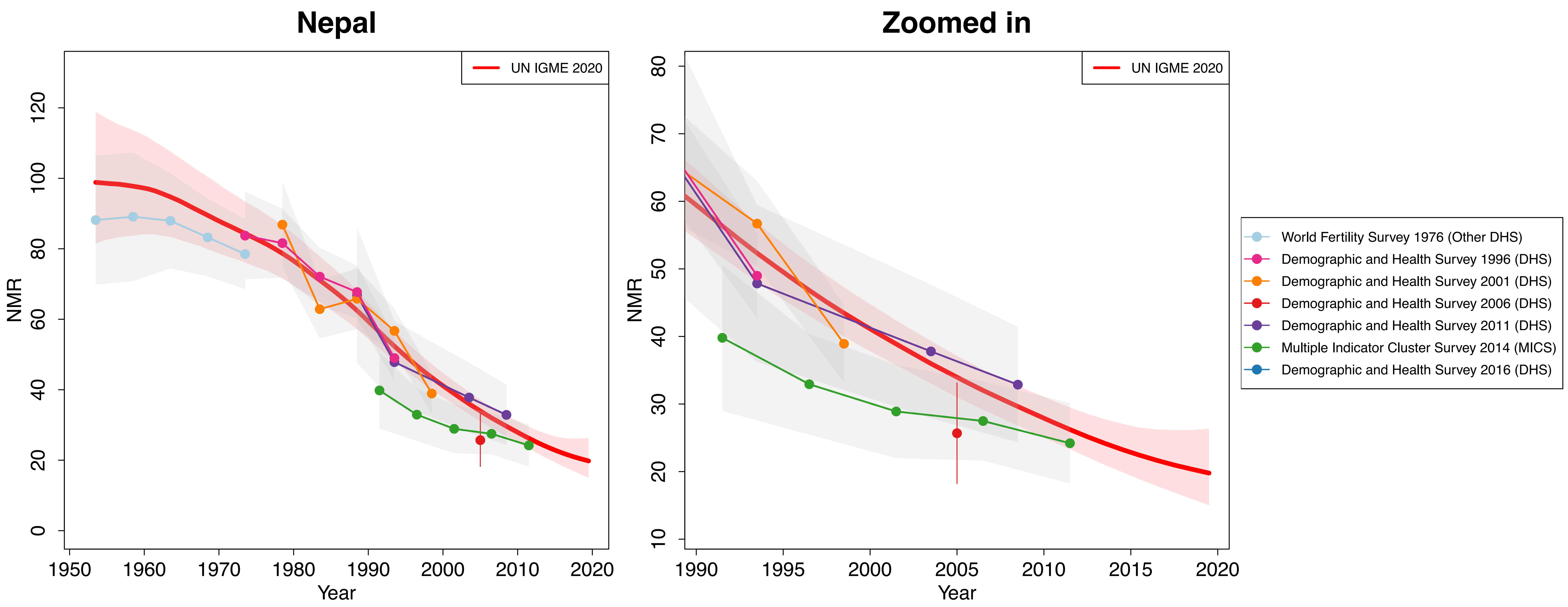
Nepal
Zoomed in
UN IGME 2020
NMR
Year
World Fertility Survey 1976 (Other DHS)
Demographic and Health Survey 1996 (DHS)
Demographic and Health Survey 2001 (DHS)
Demographic and Health Survey 2006 (DHS)
Demographic and Health Survey 2011 (DHS)
Multiple Indicator Cluster Survey 2014 (MICS)
Demographic and Health Survey 2016 (DHS)

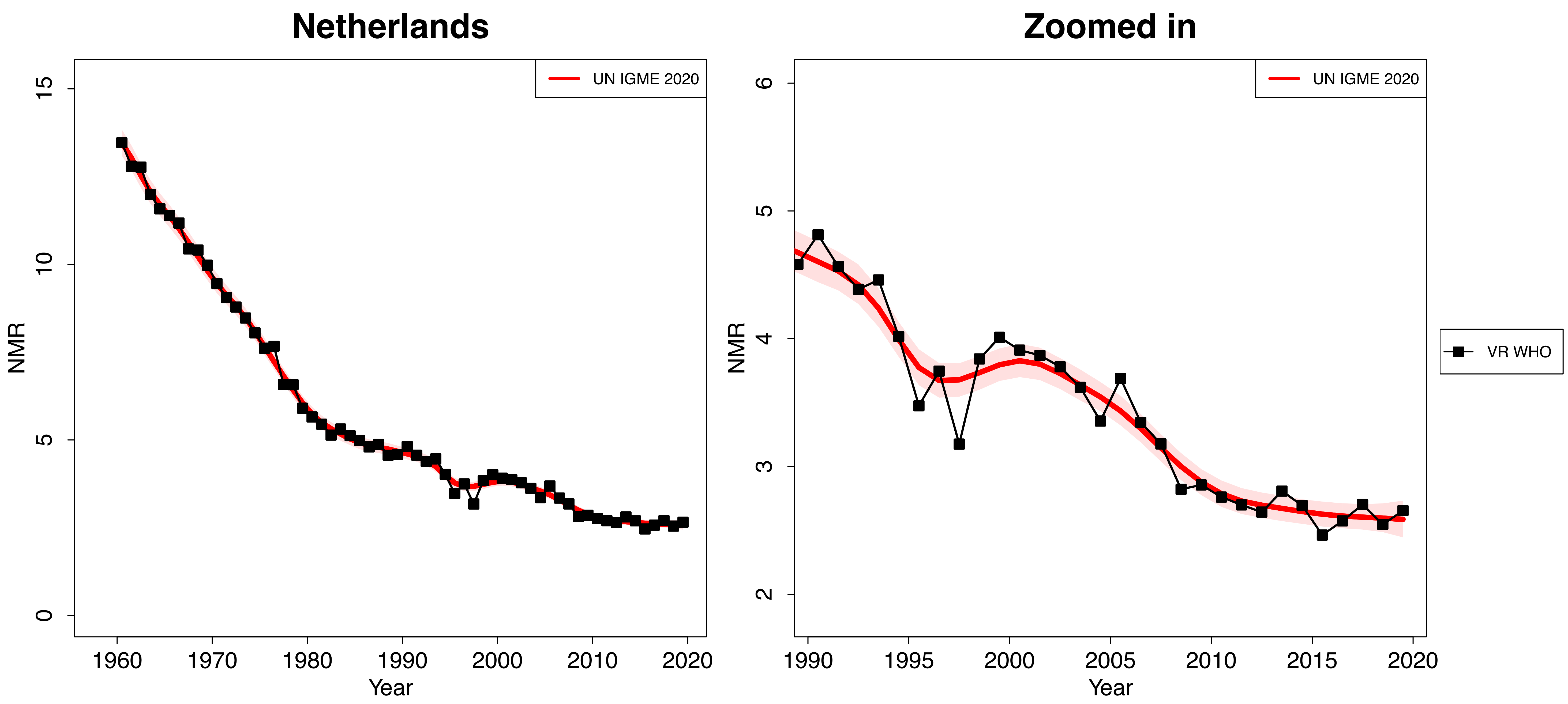

Netherlands
UN IGME 2020
NMR
Year
Zoomed in
UN IGME 2020
NMR
Year
VR WHO

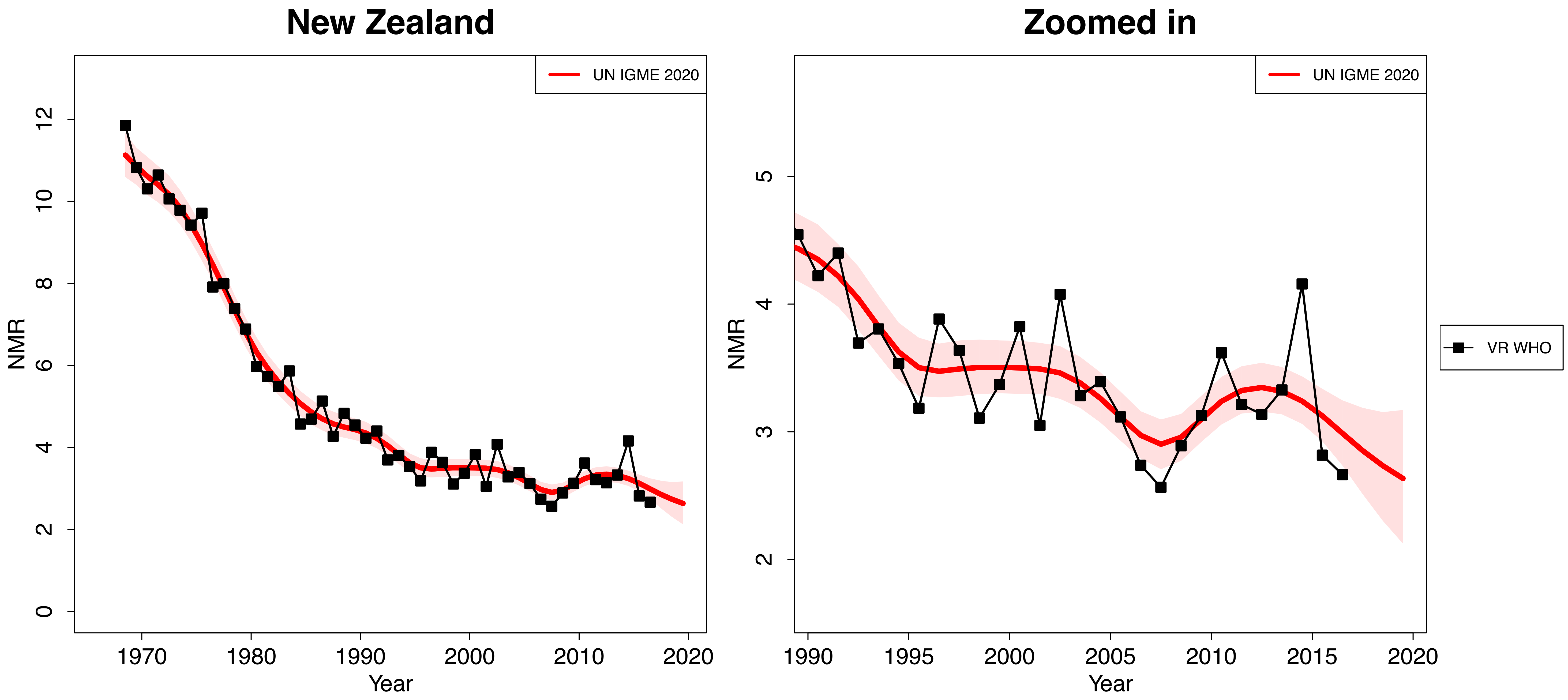

New Zealand
Zoomed in
UN IGME 2020
VR WHO
NMR
Year

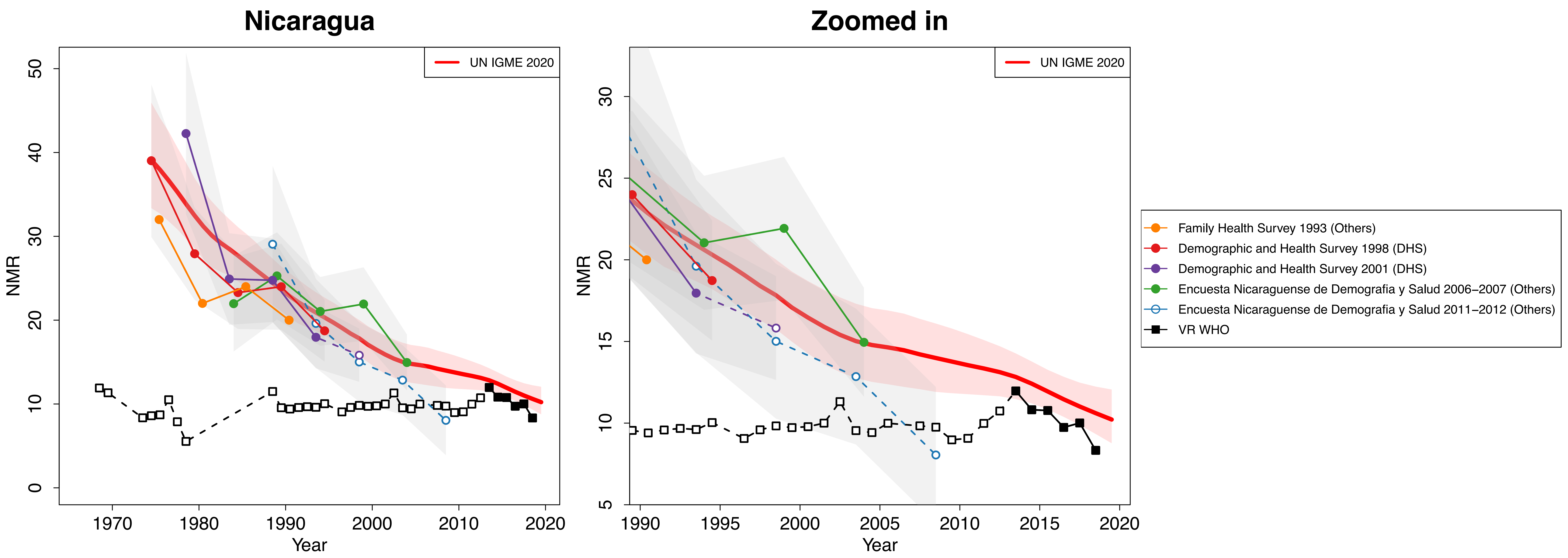
Nicaragua
Zoomed in
UN IGME 2020
NMR
Year
Family Health Survey 1993 (Others)
Demographic and Health Survey 1998 (DHS)
Demographic and Health Survey 2001 (DHS)
Encuesta Nicaraguense de Demografia y Salud 2006–2007 (Others)
Encuesta Nicaraguense de Demografia y Salud 2011–2012 (Others)
VR WHO

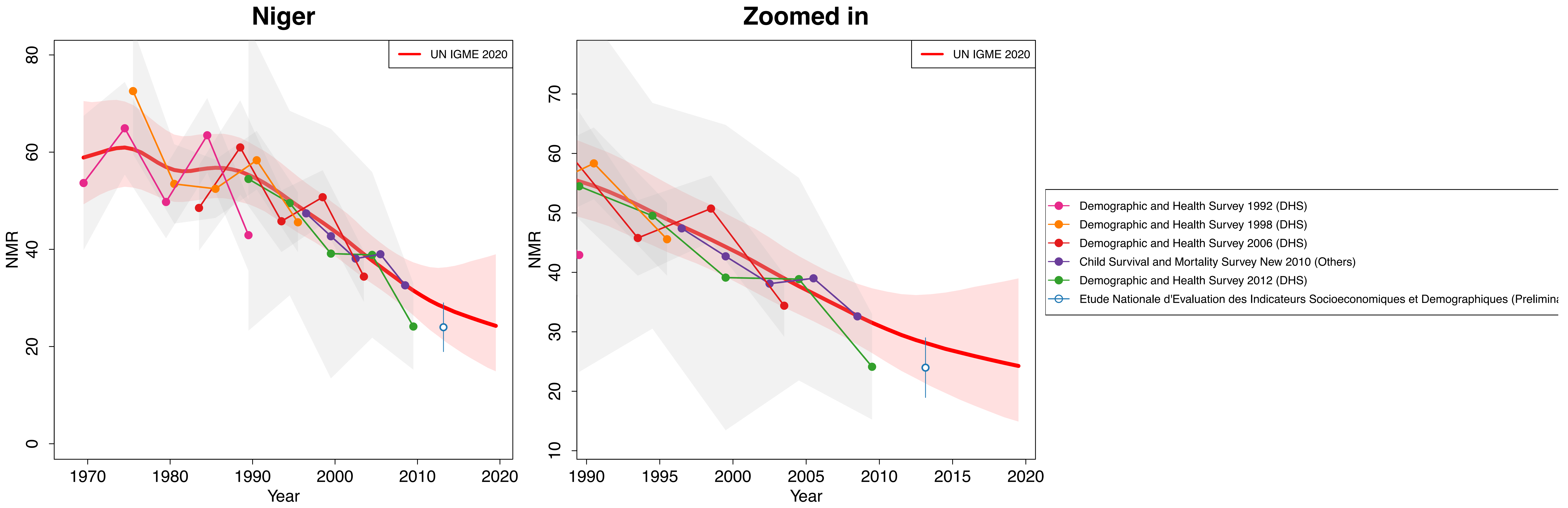

Niger
Zoomed in
UN IGME 2020
NMR
Year
Demographic and Health Survey 1992 (DHS)
Demographic and Health Survey 1998 (DHS)
Demographic and Health Survey 2006 (DHS)
Child Survival and Mortality Survey New 2010 (Others)
Demographic and Health Survey 2012 (DHS)
Etude Nationale d'Evaluation des Indicateurs Socioeconomiques et Demographiques (Prelimina

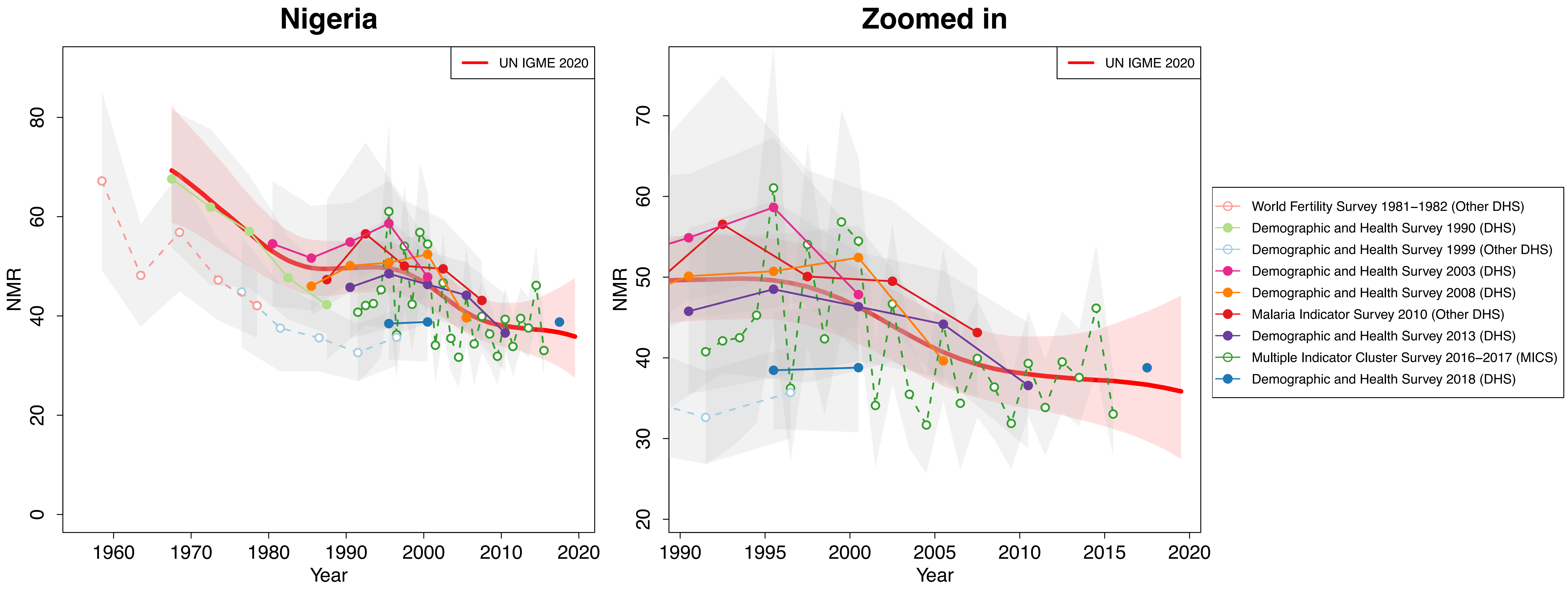
Nigeria
Zoomed in
UN IGME 2020
NMR
Year
World Fertility Survey 1981–1982 (Other DHS)
Demographic and Health Survey 1990 (DHS)
Demographic and Health Survey 1999 (Other DHS)
Demographic and Health Survey 2003 (DHS)
Demographic and Health Survey 2008 (DHS)
Malaria Indicator Survey 2010 (Other DHS)
Demographic and Health Survey 2013 (DHS)
Multiple Indicator Cluster Survey 2016–2017 (MICS)
Demographic and Health Survey 2018 (DHS)

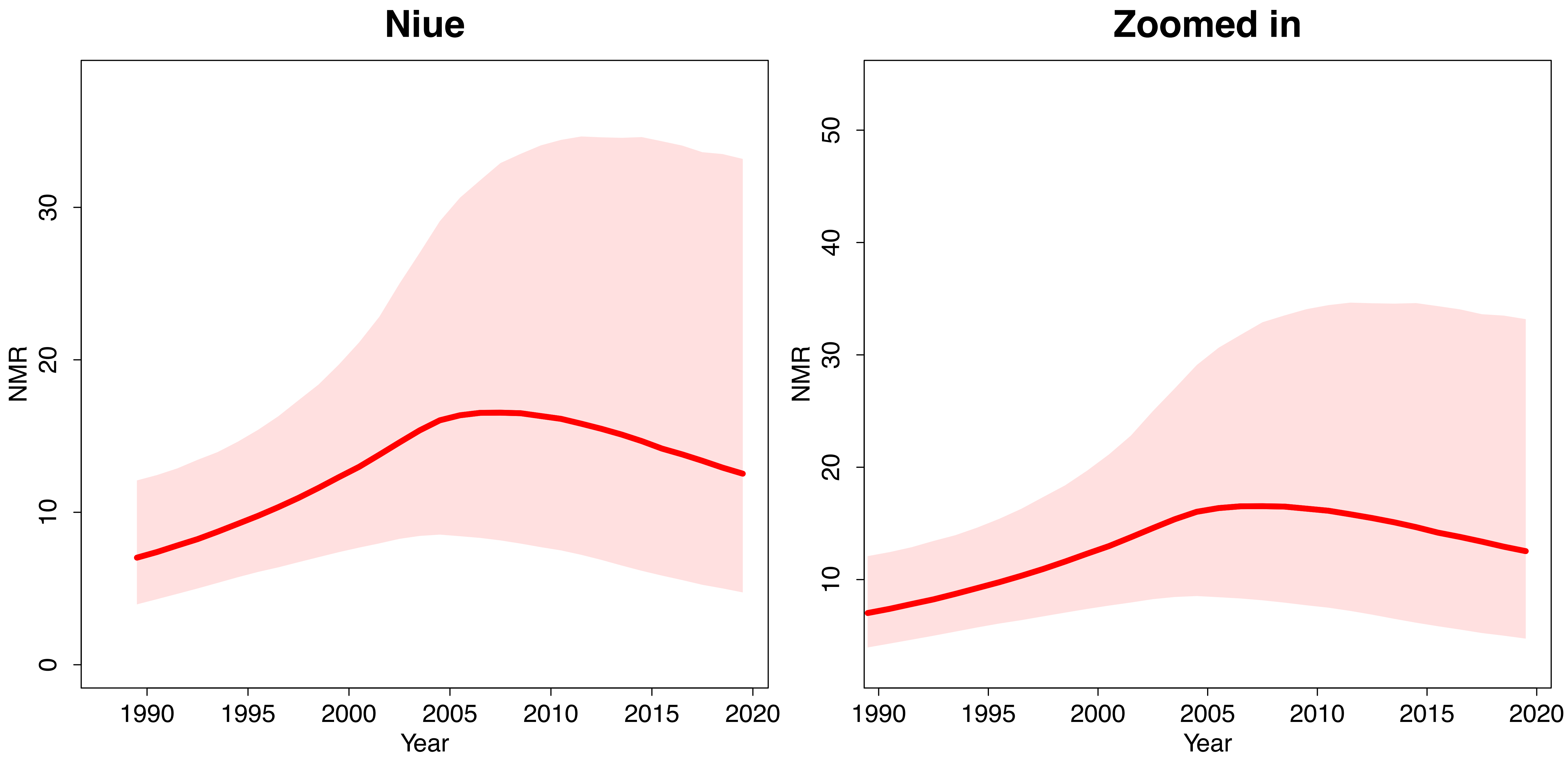

Niue
NMR
0
10
20
30
1990
1995
2000
2005
2010
2015
2020
Year
Zoomed in
NMR
10
20
30
40
50
1990
1995
2000
2005
2010
2015
2020
Year

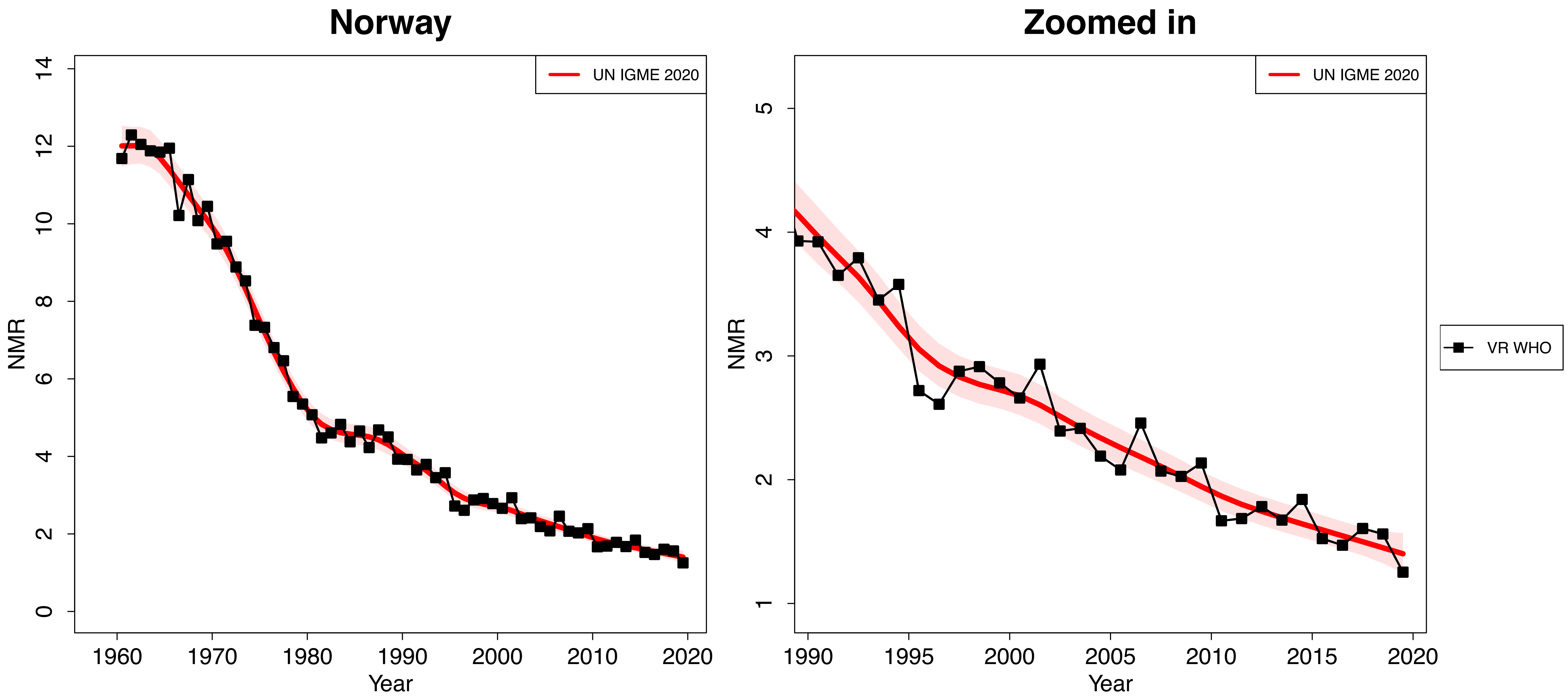
Norway
Zoomed in
UN IGME 2020
UN IGME 2020
VR WHO
NMR
NMR
Year
Year
0
2
4
6
8
10
12
14
1
2
3
4
5
1960
1970
1980
1990
2000
2010
2020
1990
1995
2000
2005
2010
2015
2020

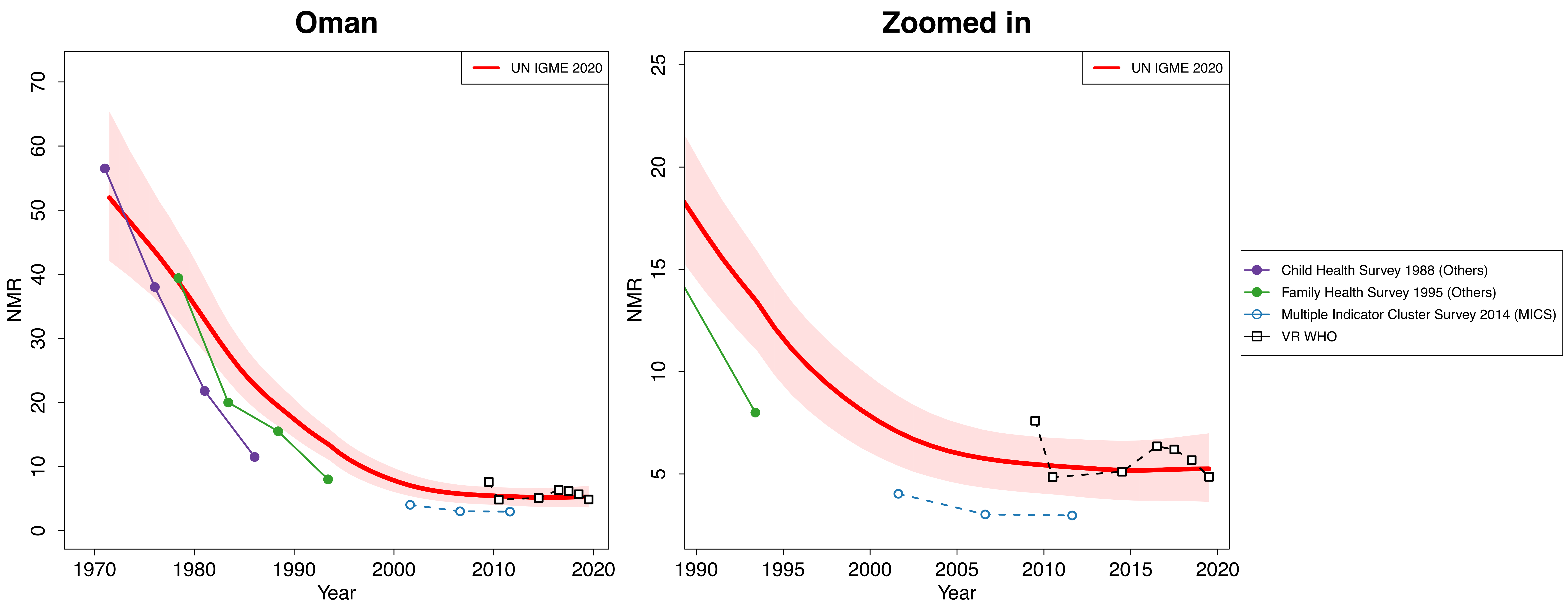

Oman
Zoomed in
UN IGME 2020
UN IGME 2020
NMR
NMR
Year
Year
Child Health Survey 1988 (Others)
Family Health Survey 1995 (Others)
Multiple Indicator Cluster Survey 2014 (MICS)
VR WHO

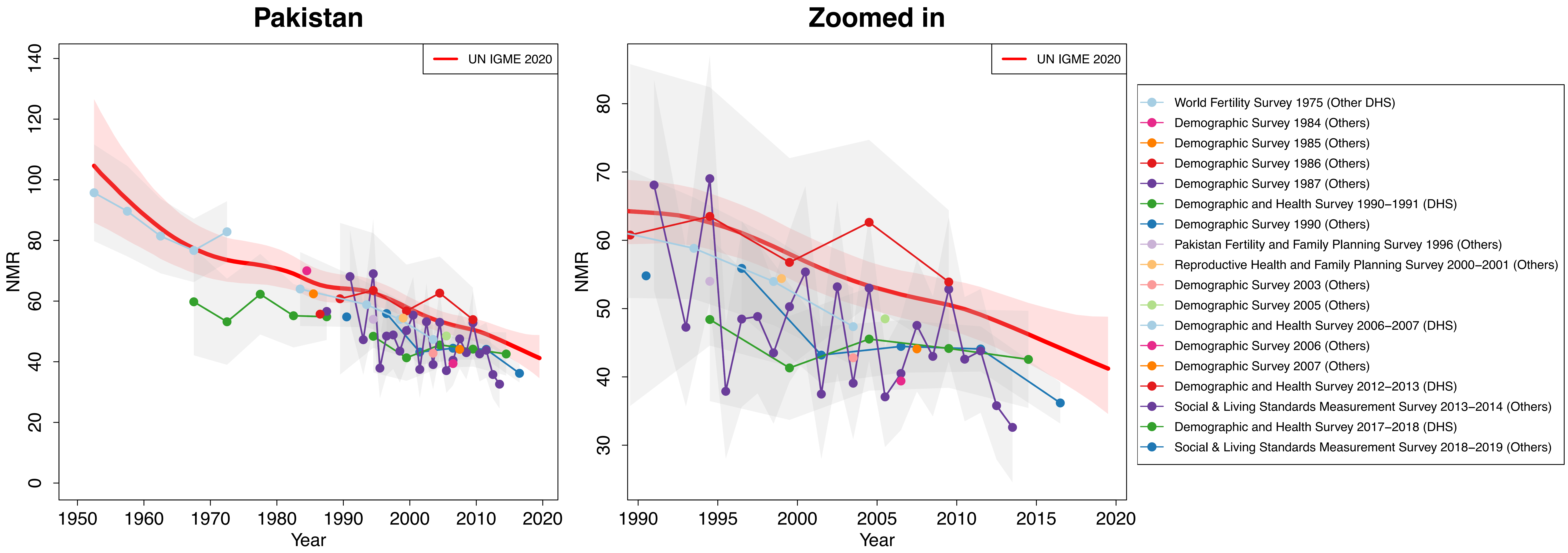

Pakistan
Zoomed in
UN IGME 2020
NMR
Year
World Fertility Survey 1975 (Other DHS)
Demographic Survey 1984 (Others)
Demographic Survey 1985 (Others)
Demographic Survey 1986 (Others)
Demographic Survey 1987 (Others)
Demographic and Health Survey 1990–1991 (DHS)
Demographic Survey 1990 (Others)
Pakistan Fertility and Family Planning Survey 1996 (Others)
Reproductive Health and Family Planning Survey 2000–2001 (Others)
Demographic Survey 2003 (Others)
Demographic Survey 2005 (Others)
Demographic and Health Survey 2006–2007 (DHS)
Demographic Survey 2006 (Others)
Demographic Survey 2007 (Others)
Demographic and Health Survey 2012–2013 (DHS)
Social & Living Standards Measurement Survey 2013–2014 (Others)
Demographic and Health Survey 2017–2018 (DHS)
Social & Living Standards Measurement Survey 2018–2019 (Others)

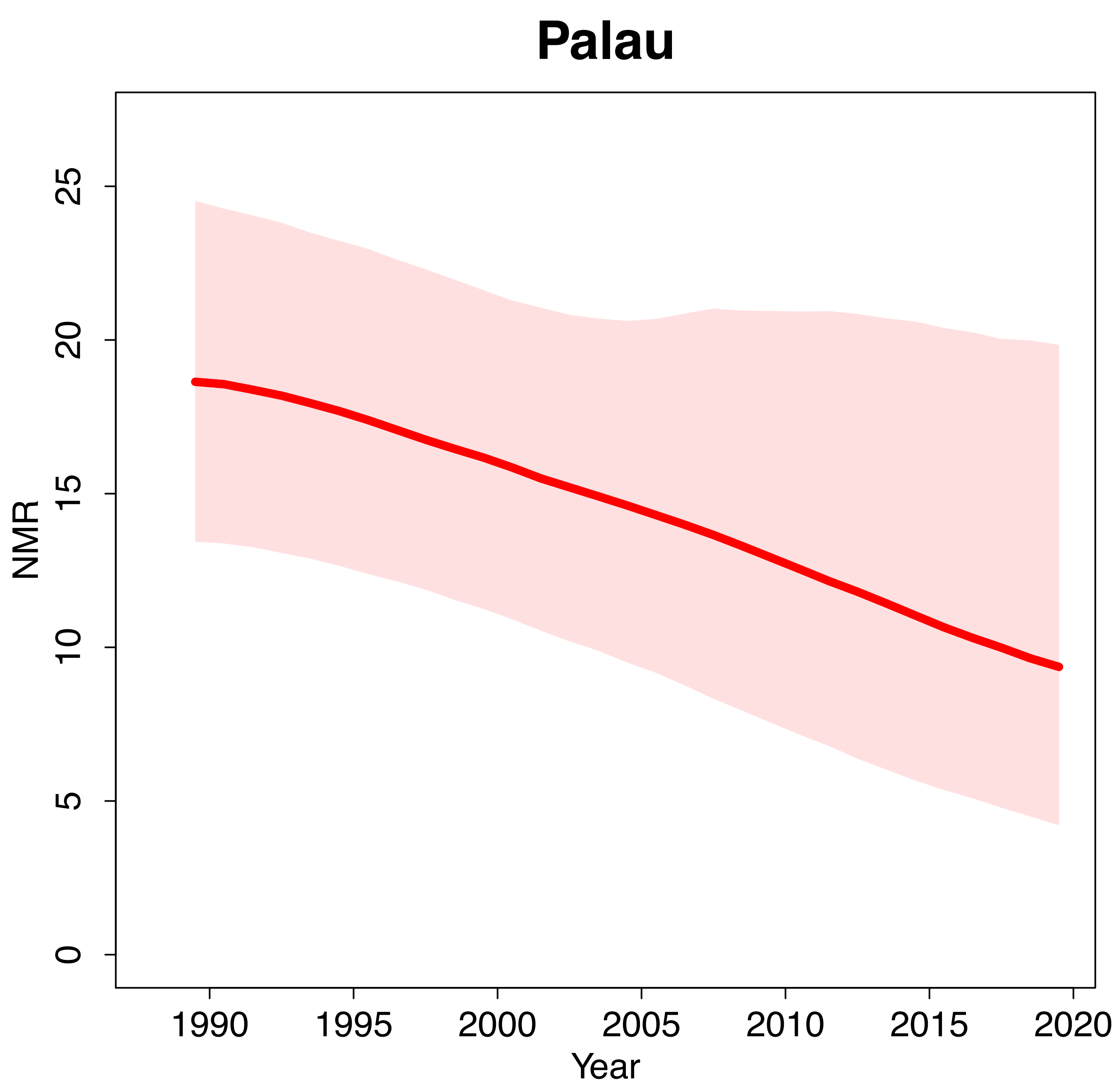

Palau
NMR
Year

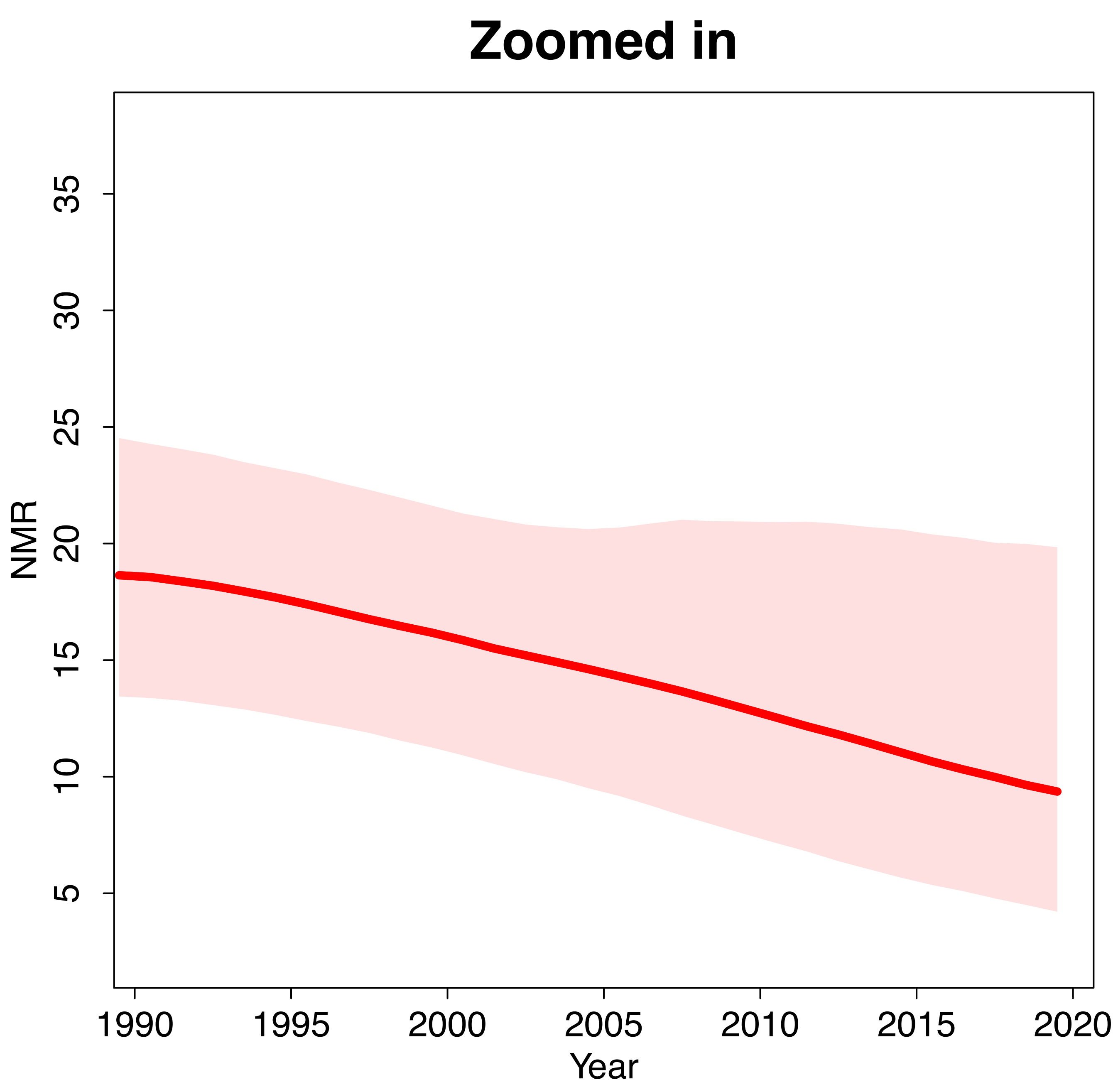

Zoomed in
NMR
Year

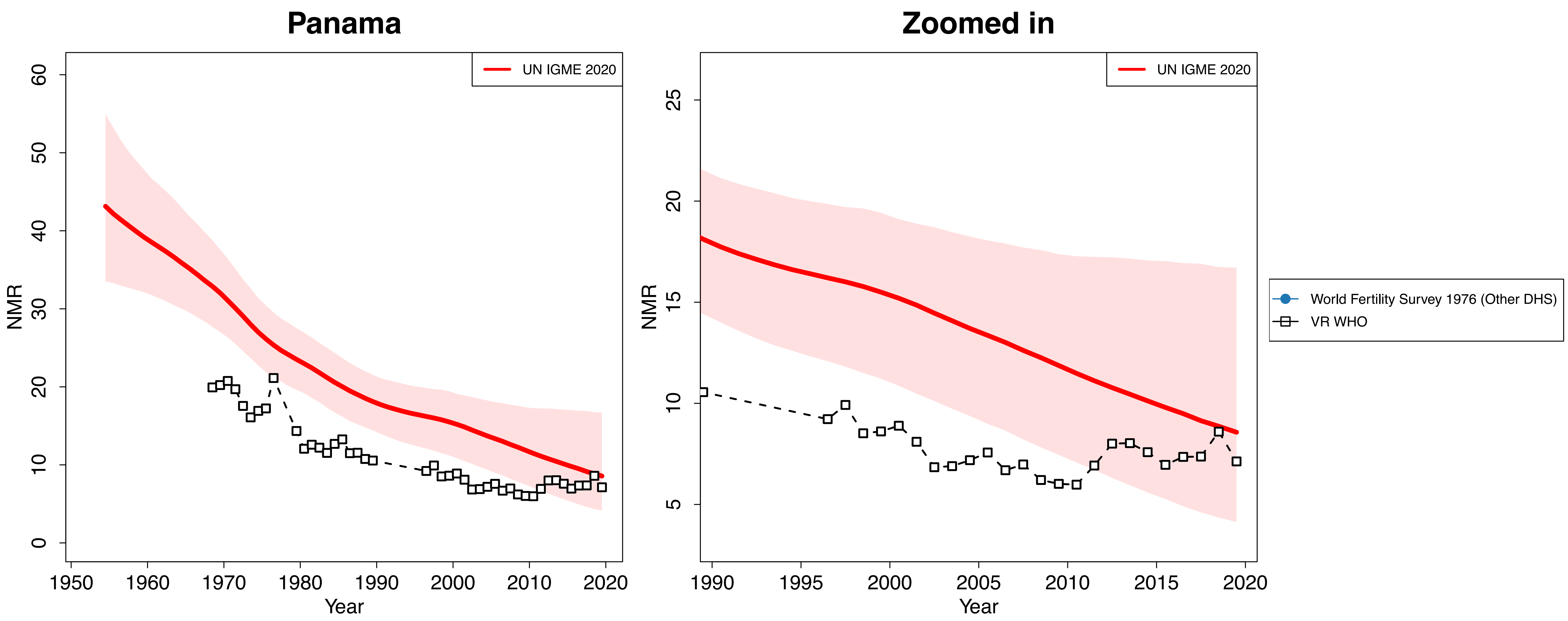

Panama
UN IGME 2020
NMR
Year
0
10
20
30
40
50
60
1950
1960
1970
1980
1990
2000
2010
2020
Zoomed in
UN IGME 2020
NMR
Year
5
10
15
20
25
1990
1995
2000
2005
2010
2015
2020
World Fertility Survey 1976 (Other DHS)
VR WHO

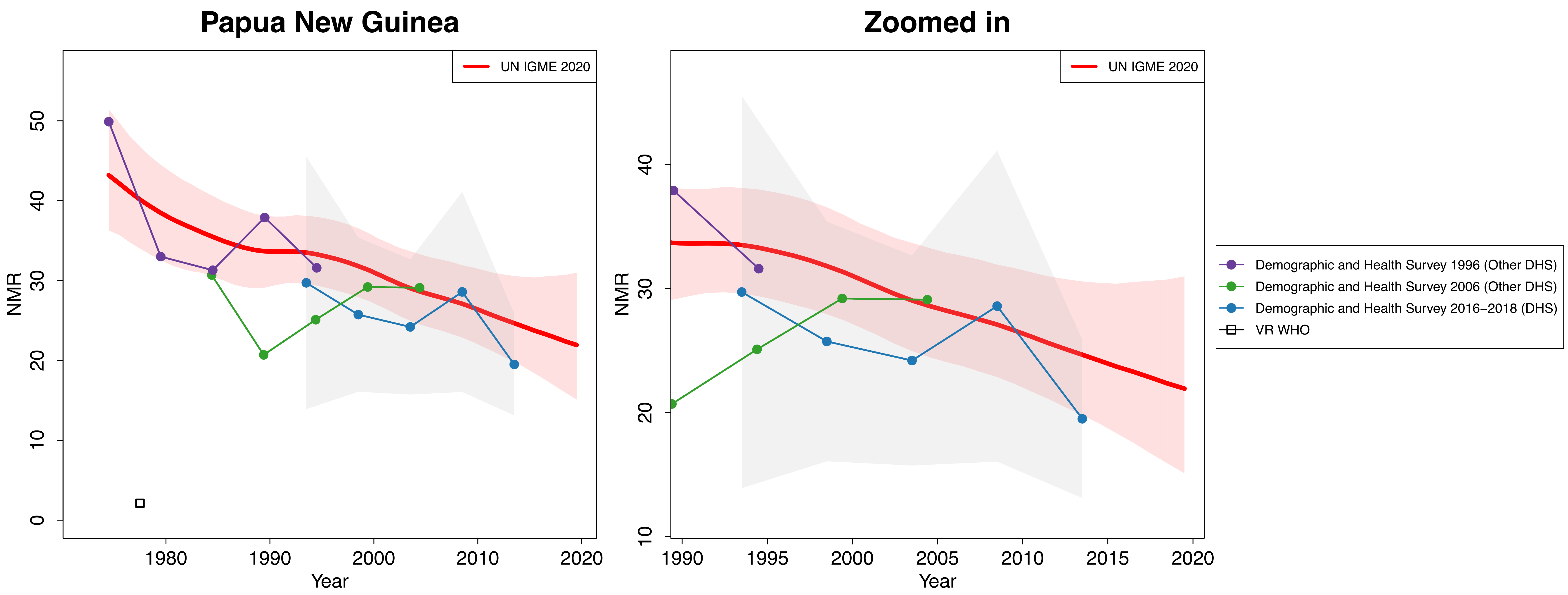

Papua New Guinea
Zoomed in
UN IGME 2020
NMR
Year
Demographic and Health Survey 1996 (Other DHS)
Demographic and Health Survey 2006 (Other DHS)
Demographic and Health Survey 2016–2018 (DHS)
VR WHO

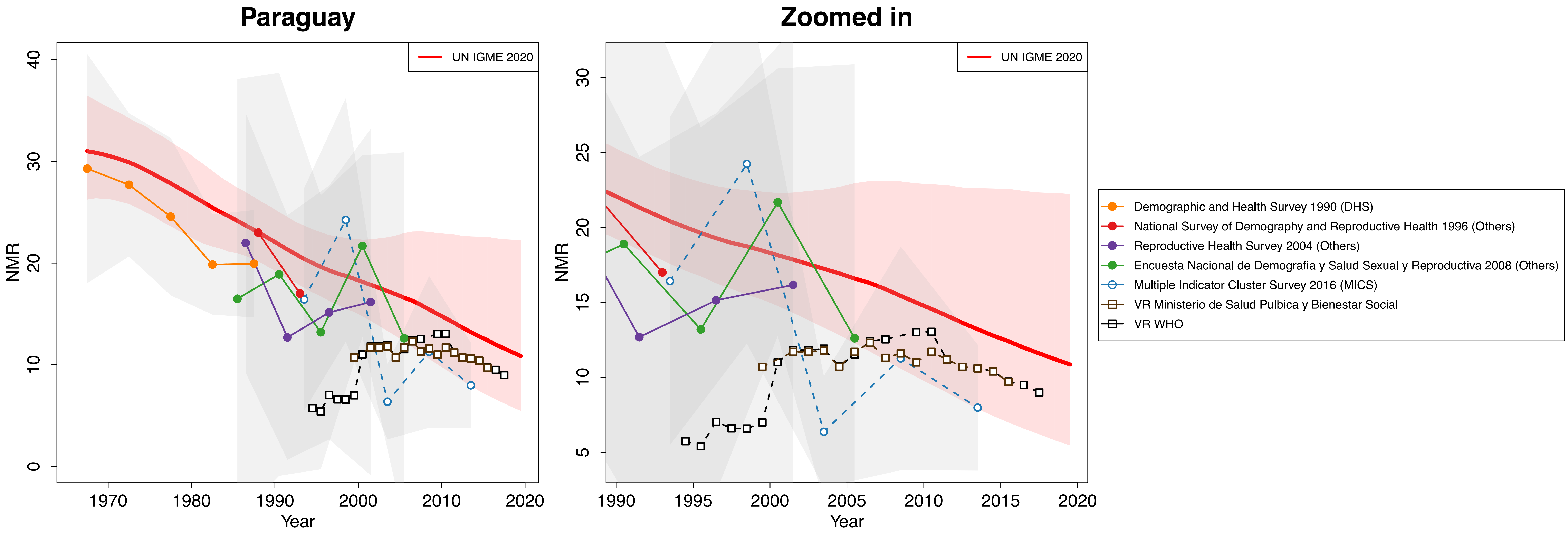
Paraguay
Zoomed in
UN IGME 2020
NMR
Year
Demographic and Health Survey 1990 (DHS)
National Survey of Demography and Reproductive Health 1996 (Others)
Reproductive Health Survey 2004 (Others)
Encuesta Nacional de Demografia y Salud Sexual y Reproductiva 2008 (Others)
Multiple Indicator Cluster Survey 2016 (MICS)
VR Ministerio de Salud Pulbica y Bienestar Social
VR WHO

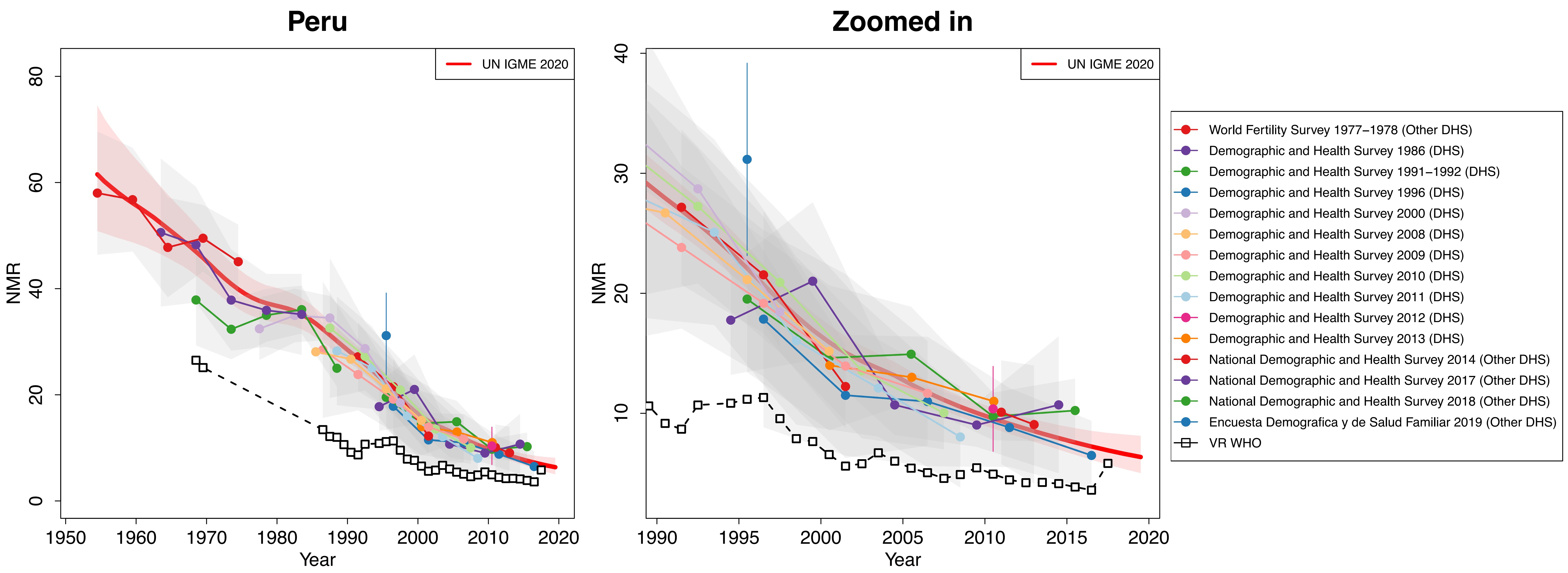

Peru
Zoomed in
UN IGME 2020
NMR
Year
World Fertility Survey 1977–1978 (Other DHS)
Demographic and Health Survey 1986 (DHS)
Demographic and Health Survey 1991–1992 (DHS)
Demographic and Health Survey 1996 (DHS)
Demographic and Health Survey 2000 (DHS)
Demographic and Health Survey 2008 (DHS)
Demographic and Health Survey 2009 (DHS)
Demographic and Health Survey 2010 (DHS)
Demographic and Health Survey 2011 (DHS)
Demographic and Health Survey 2012 (DHS)
Demographic and Health Survey 2013 (DHS)
National Demographic and Health Survey 2014 (Other DHS)
National Demographic and Health Survey 2017 (Other DHS)
National Demographic and Health Survey 2018 (Other DHS)
Encuesta Demografica y de Salud Familiar 2019 (Other DHS)
VR WHO

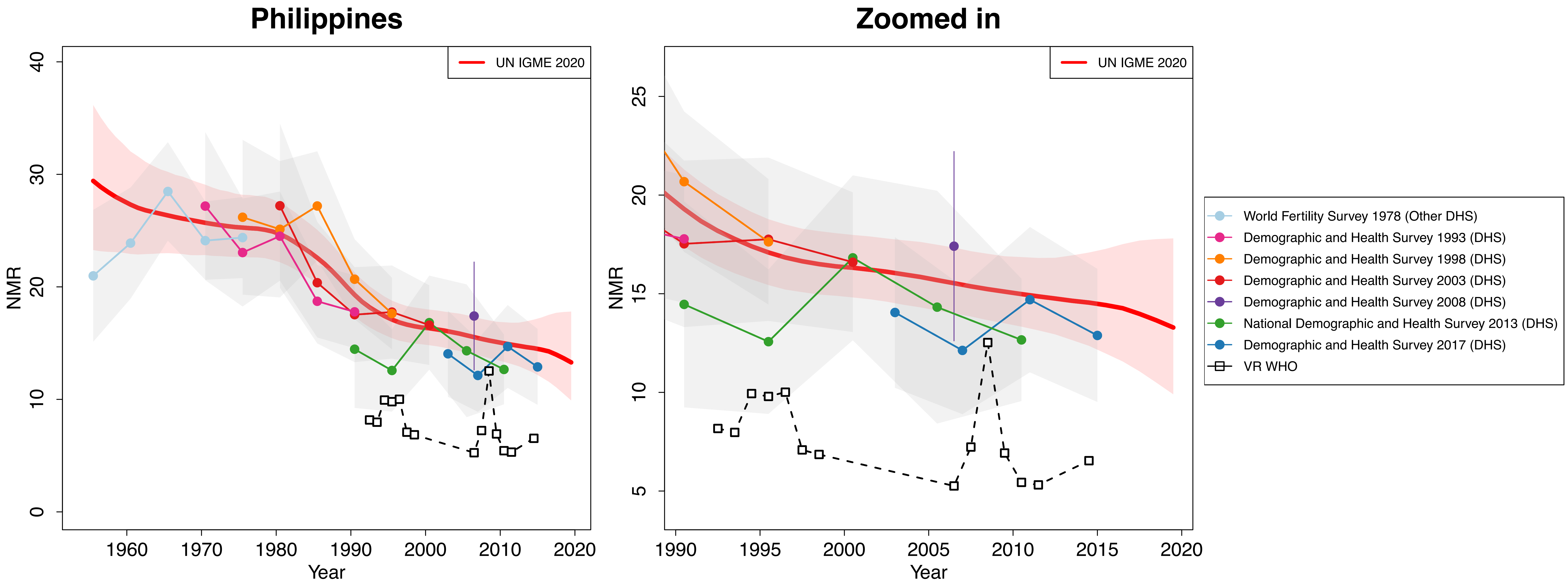

Philippines
Zoomed in
UN IGME 2020
NMR
Year
World Fertility Survey 1978 (Other DHS)
Demographic and Health Survey 1993 (DHS)
Demographic and Health Survey 1998 (DHS)
Demographic and Health Survey 2003 (DHS)
Demographic and Health Survey 2008 (DHS)
National Demographic and Health Survey 2013 (DHS)
Demographic and Health Survey 2017 (DHS)
VR WHO

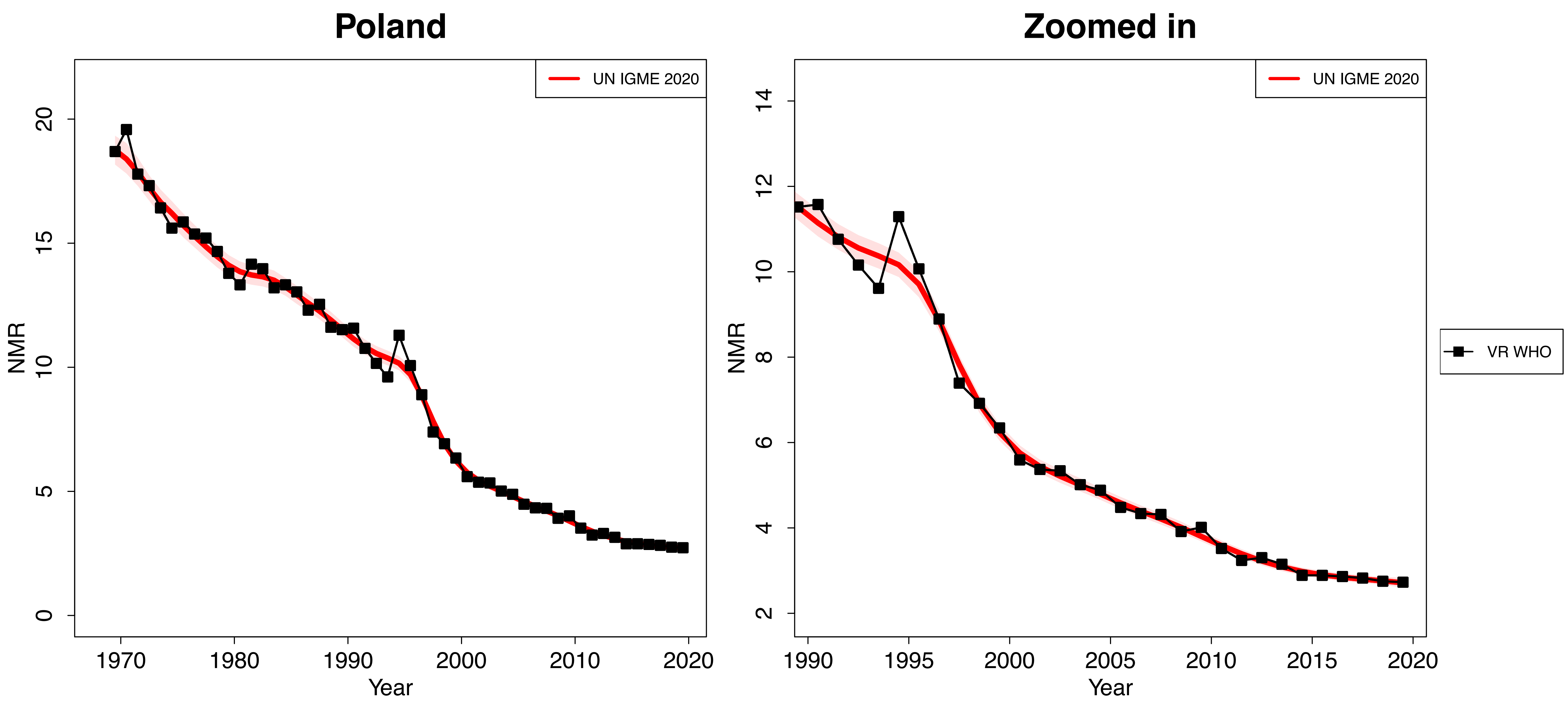

Poland
Zoomed in
UN IGME 2020
UN IGME 2020
VR WHO
NMR
NMR
Year
Year

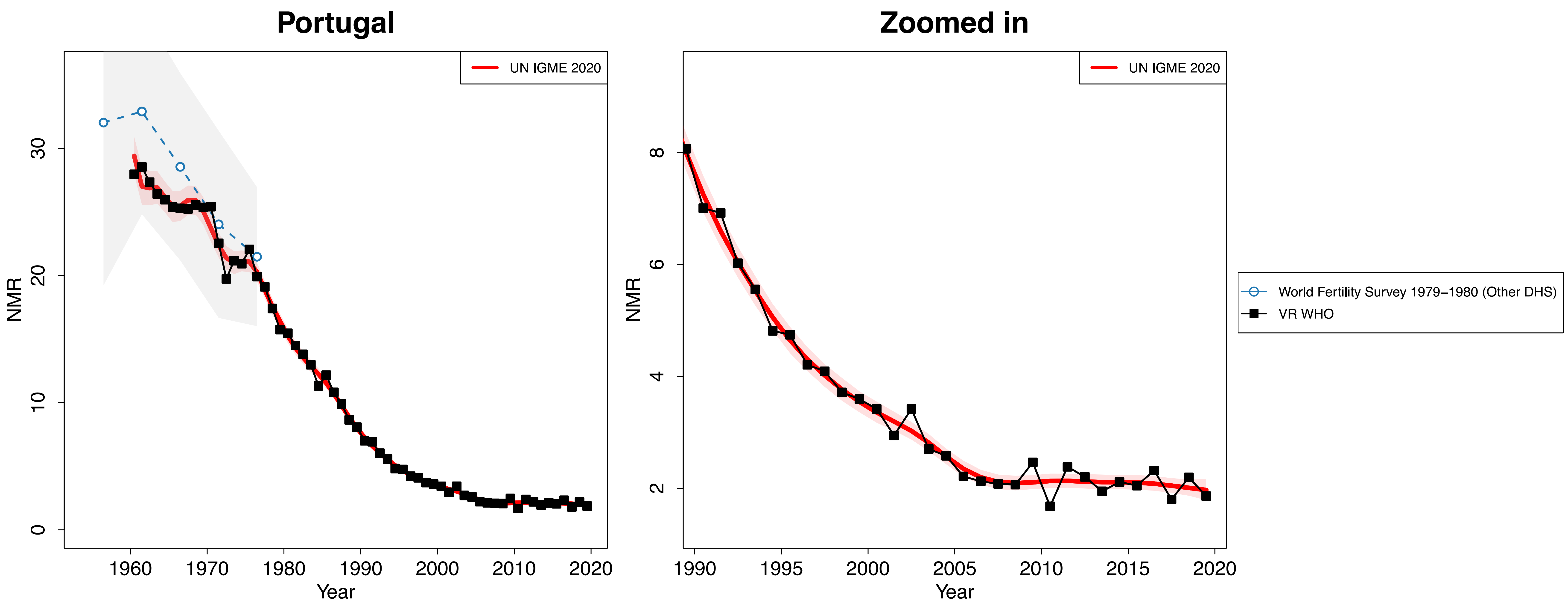

Portugal
Zoomed in
UN IGME 2020
UN IGME 2020
NMR
NMR
Year
Year
World Fertility Survey 1979–1980 (Other DHS)
VR WHO

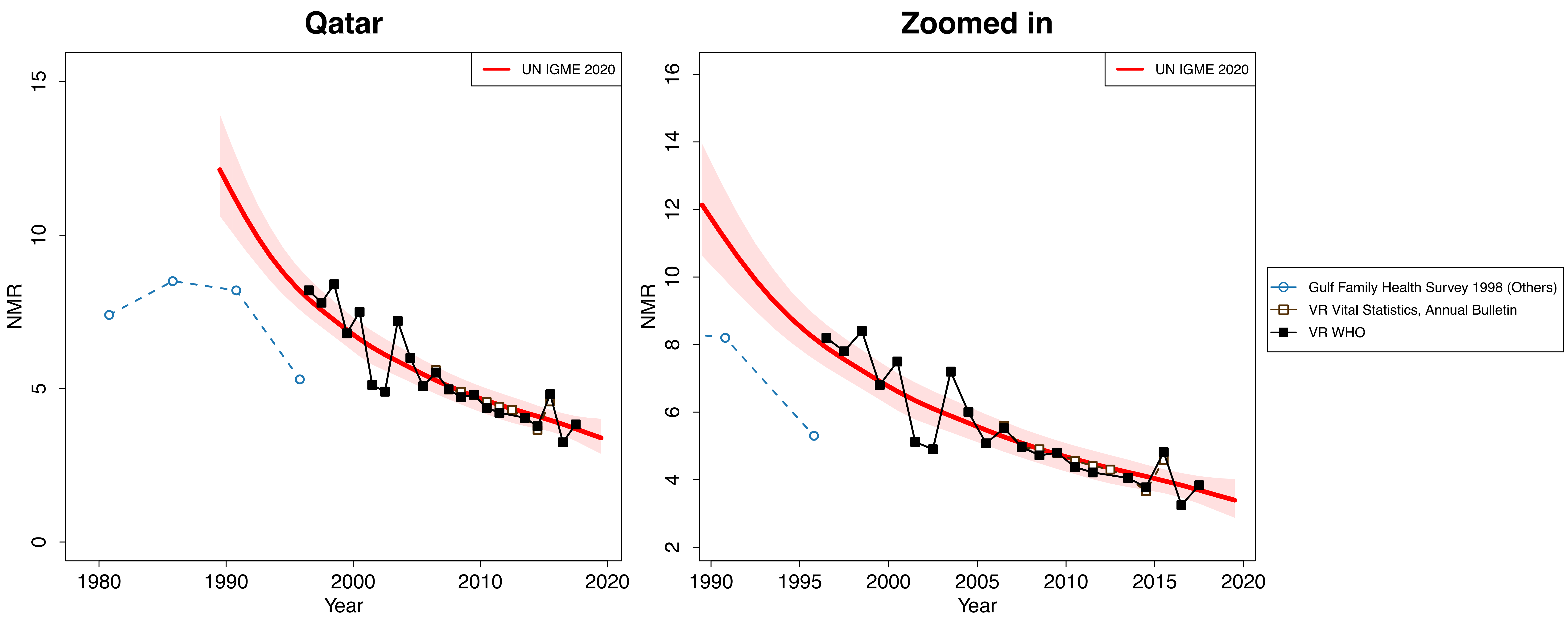

Qatar
Zoomed in
UN IGME 2020
NMR
Year
Gulf Family Health Survey 1998 (Others)
VR Vital Statistics, Annual Bulletin
VR WHO

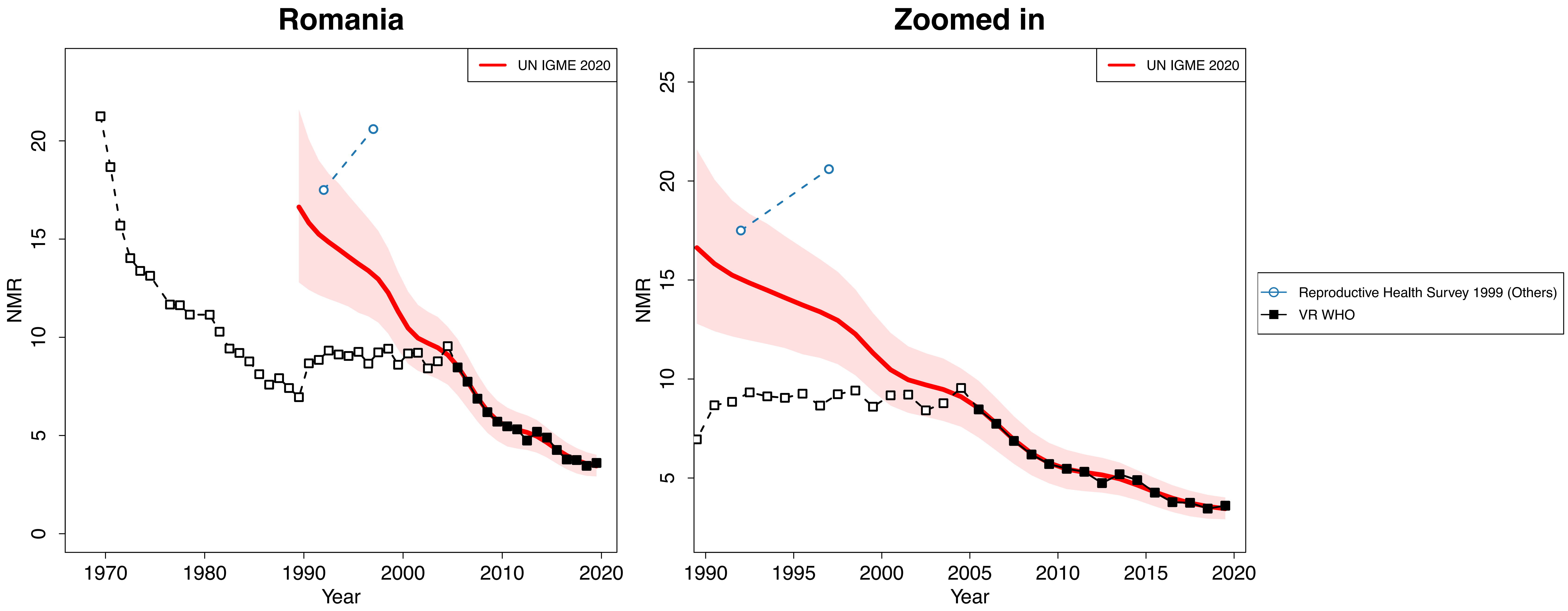
Romania
Zoomed in
UN IGME 2020
UN IGME 2020
NMR
NMR
Year
Year
Reproductive Health Survey 1999 (Others)
VR WHO
0
5
10
15
20
25
1970
1980
1990
2000
2010
2020
1995
2005
2015

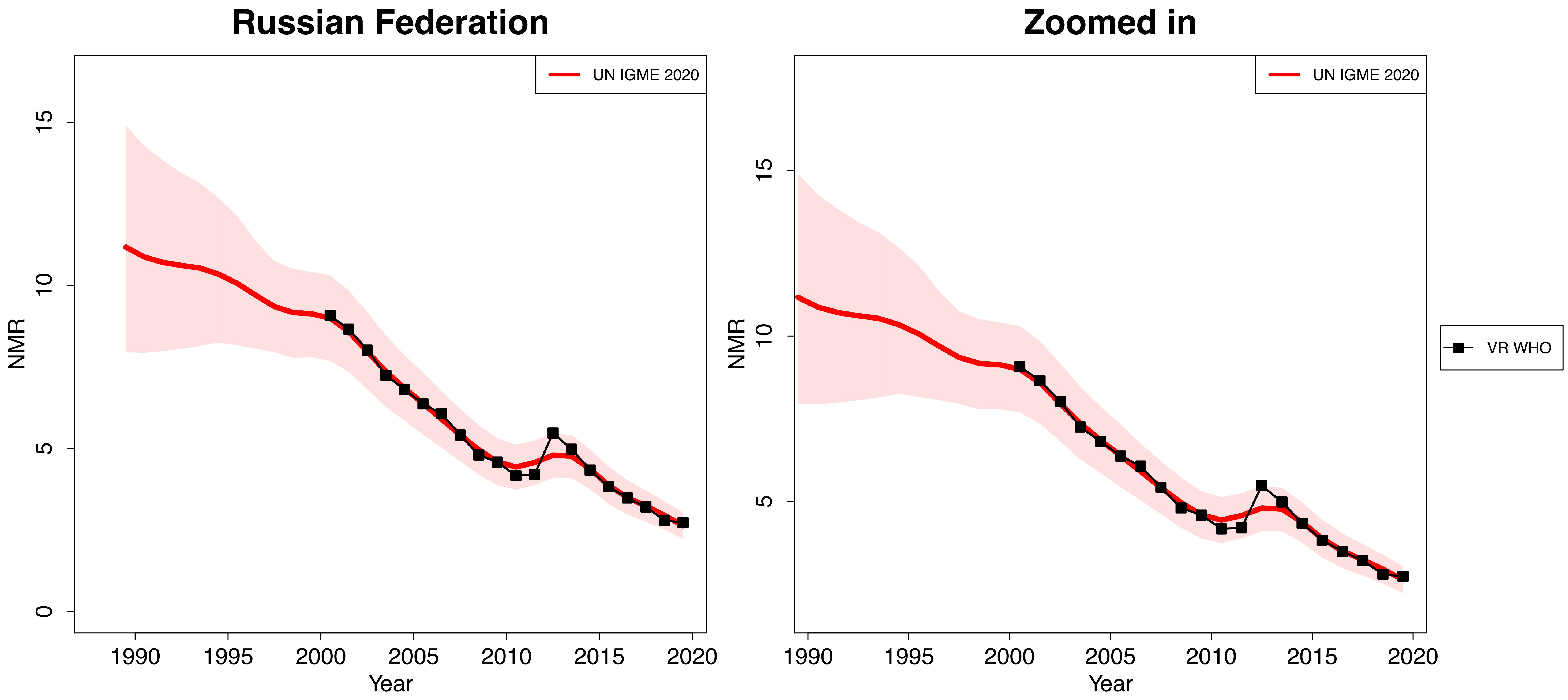
Russian Federation
Zoomed in
UN IGME 2020
UN IGME 2020
VR WHO
NMR
NMR
Year
Year
0
5
10
15
5
10
15
1990
1995
2000
2005
2010
2015
2020
1990
1995
2000
2005
2010
2015
2020

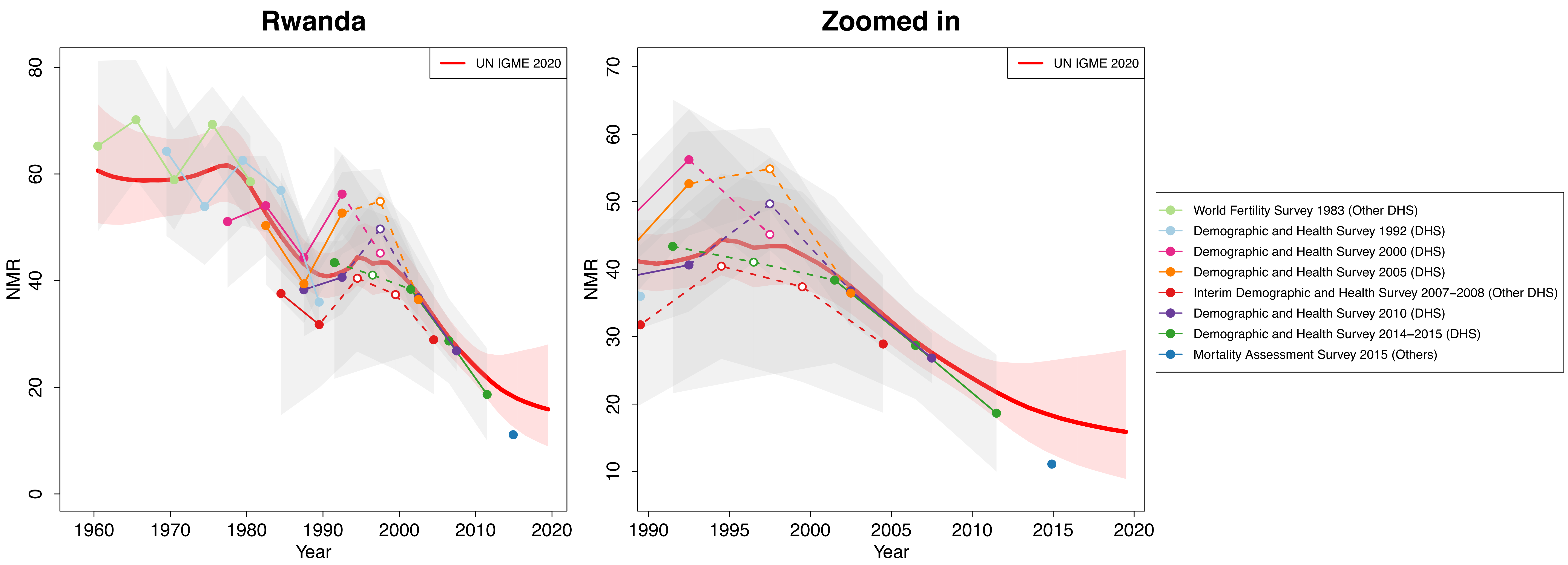

Rwanda
Zoomed in
UN IGME 2020
NMR
Year
World Fertility Survey 1983 (Other DHS)
Demographic and Health Survey 1992 (DHS)
Demographic and Health Survey 2000 (DHS)
Demographic and Health Survey 2005 (DHS)
Interim Demographic and Health Survey 2007–2008 (Other DHS)
Demographic and Health Survey 2010 (DHS)
Demographic and Health Survey 2014–2015 (DHS)
Mortality Assessment Survey 2015 (Others)

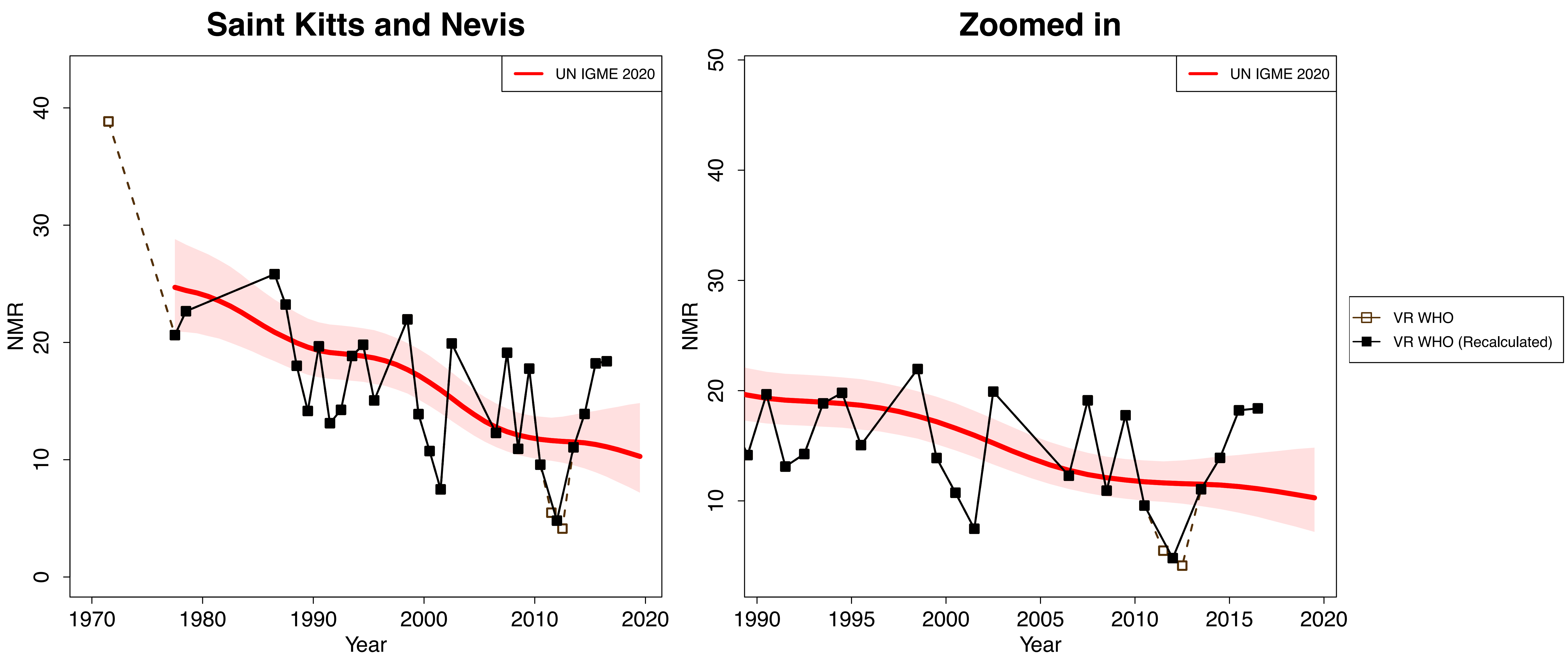

Saint Kitts and Nevis
Zoomed in
UN IGME 2020
VR WHO
VR WHO (Recalculated)
NMR
Year

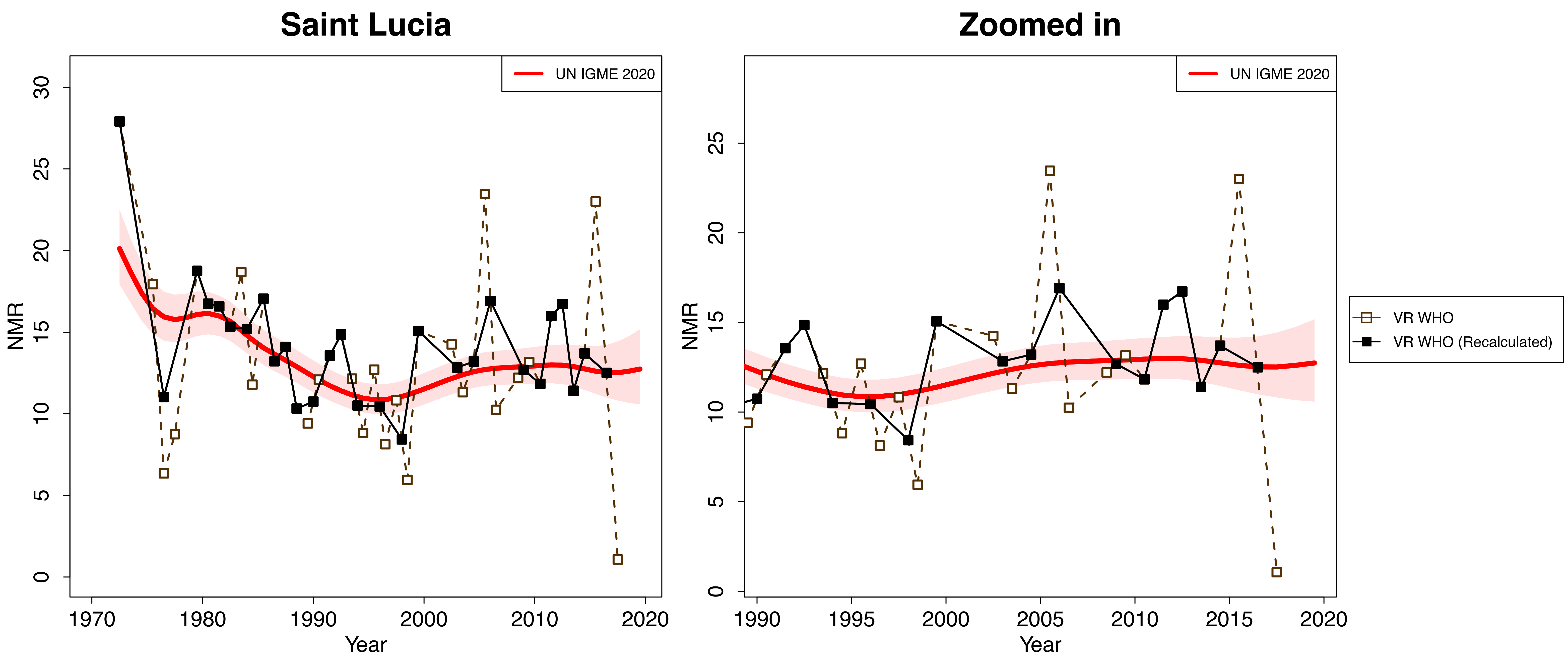

Saint Lucia
Zoomed in
UN IGME 2020
NMR
Year
VR WHO
VR WHO (Recalculated)

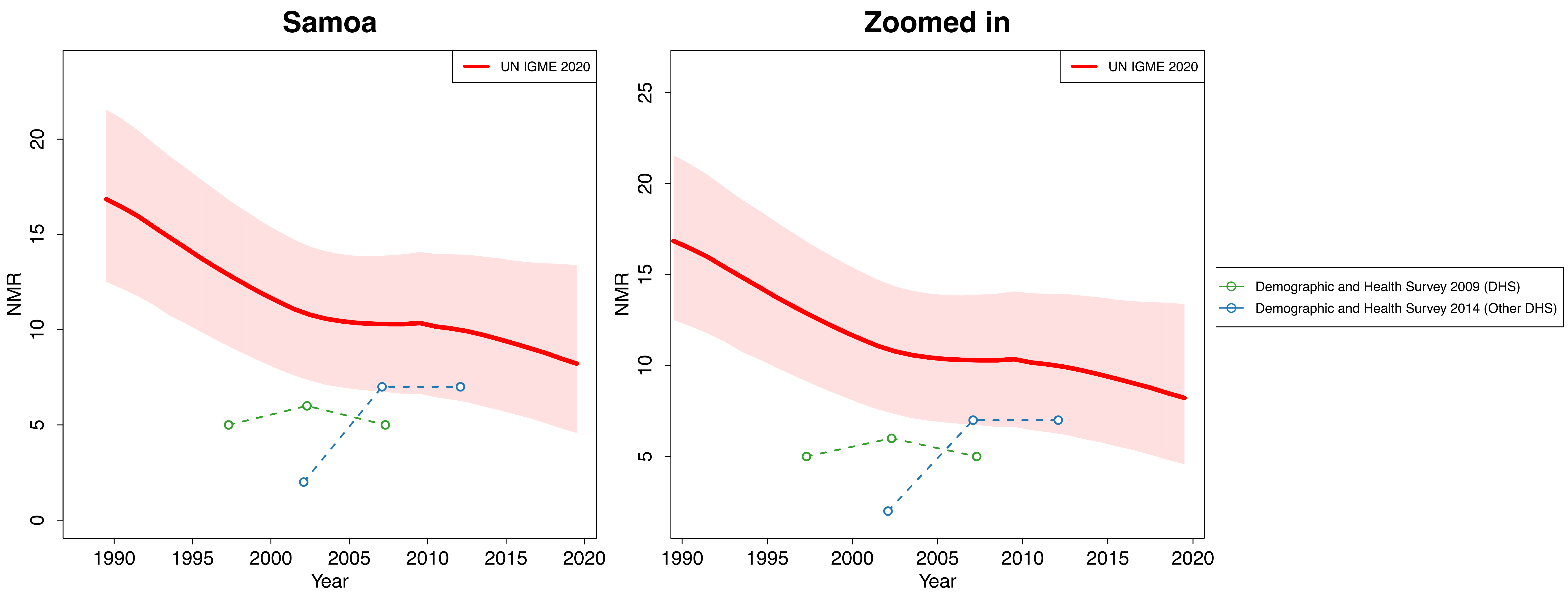

Samoa
Zoomed in
UN IGME 2020
NMR
Year
Demographic and Health Survey 2009 (DHS)
Demographic and Health Survey 2014 (Other DHS)

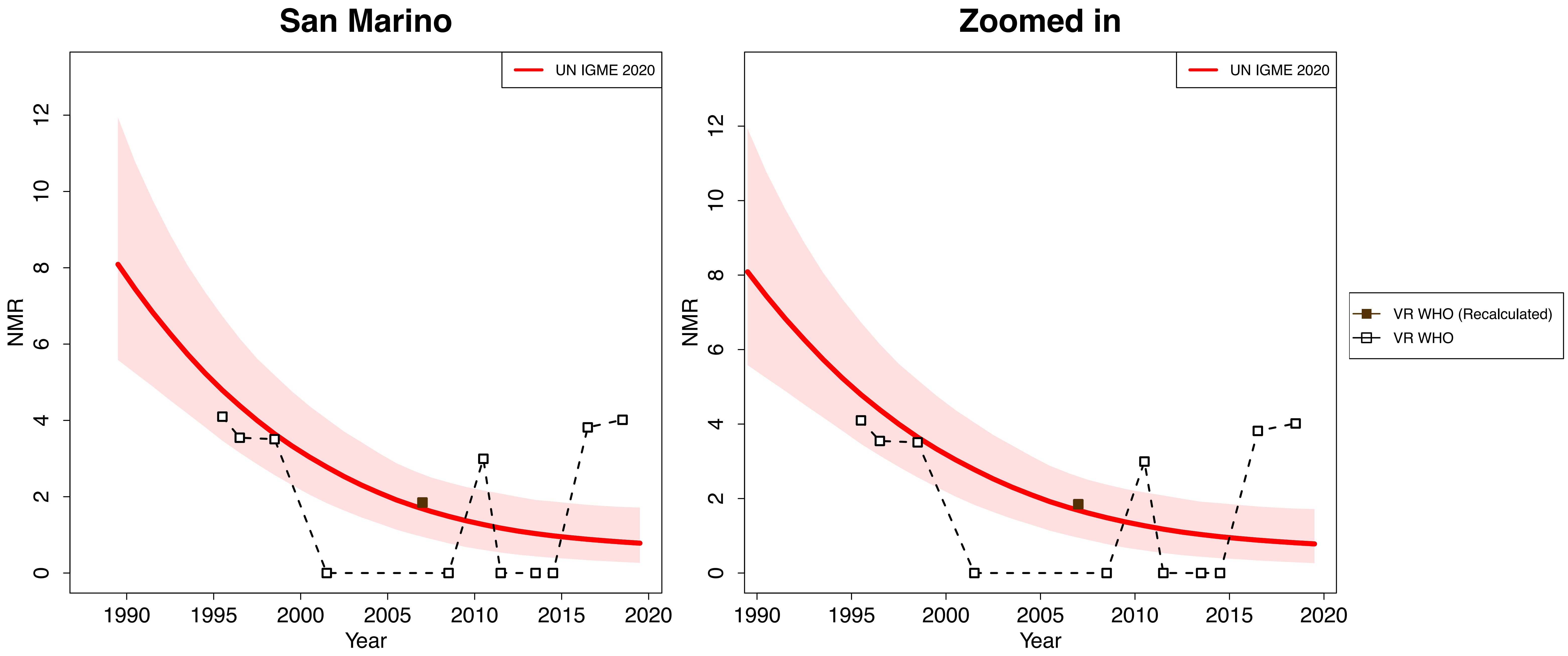

San Marino
UN IGME 2020
NMR
Year
0
2
4
6
8
10
12
1990
1995
2000
2005
2010
2015
2020
Zoomed in
UN IGME 2020
VR WHO (Recalculated)
VR WHO

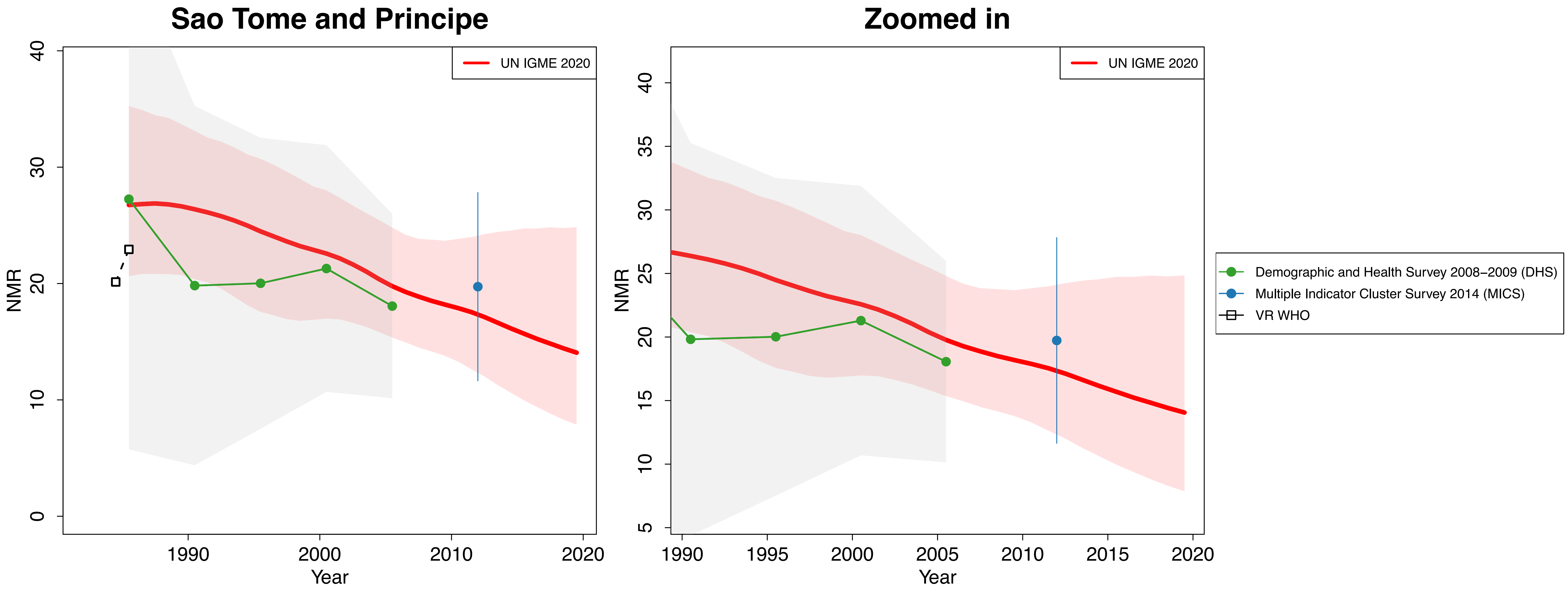
Sao Tome and Principe
Zoomed in
UN IGME 2020
UN IGME 2020
NMR
NMR
Year
Year
0
10
20
30
40
5
10
15
20
25
30
35
40
1990
2000
2010
2020
1990
1995
2000
2005
2010
2015
2020
Demographic and Health Survey 2008–2009 (DHS)
Multiple Indicator Cluster Survey 2014 (MICS)
VR WHO

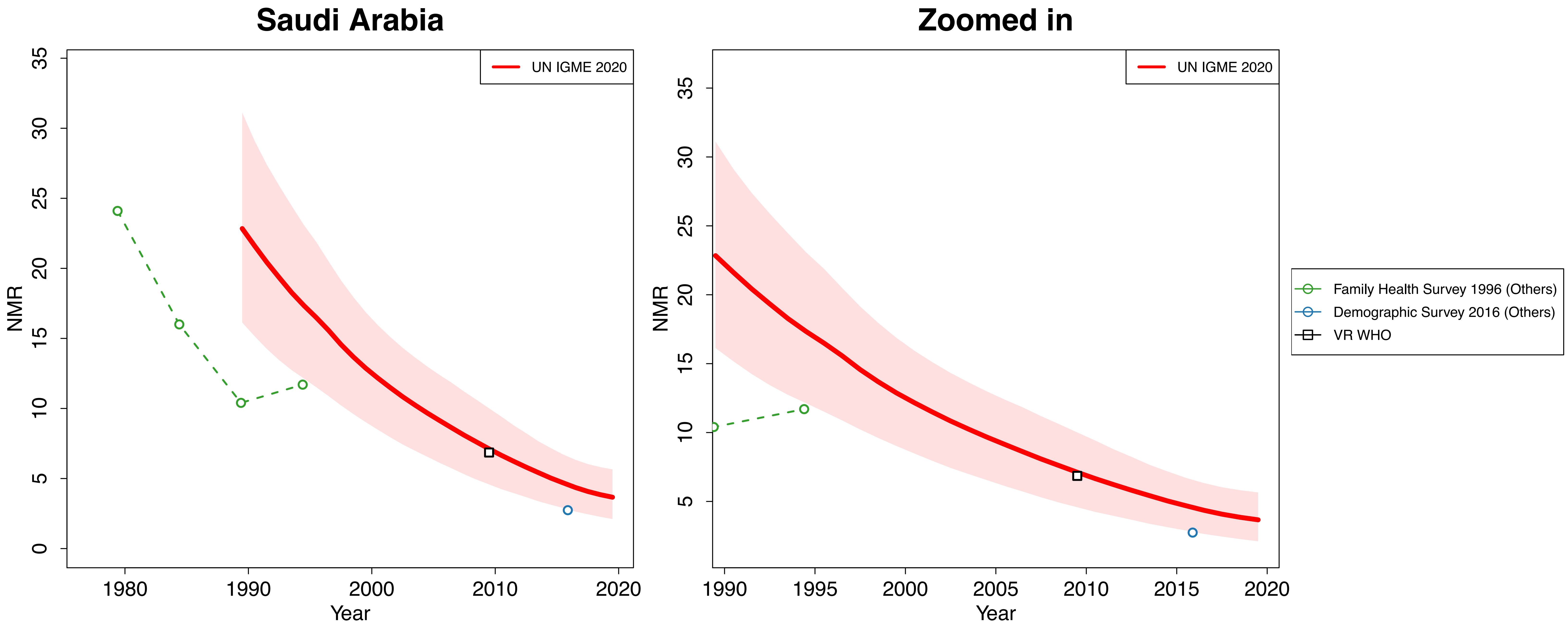
Saudi Arabia
Zoomed in
UN IGME 2020
UN IGME 2020
NMR
NMR
Year
Year
Family Health Survey 1996 (Others)
Demographic Survey 2016 (Others)
VR WHO
0
5
10
15
20
25
30
35
1980
1990
2000
2010
2020
1995
2005
2015

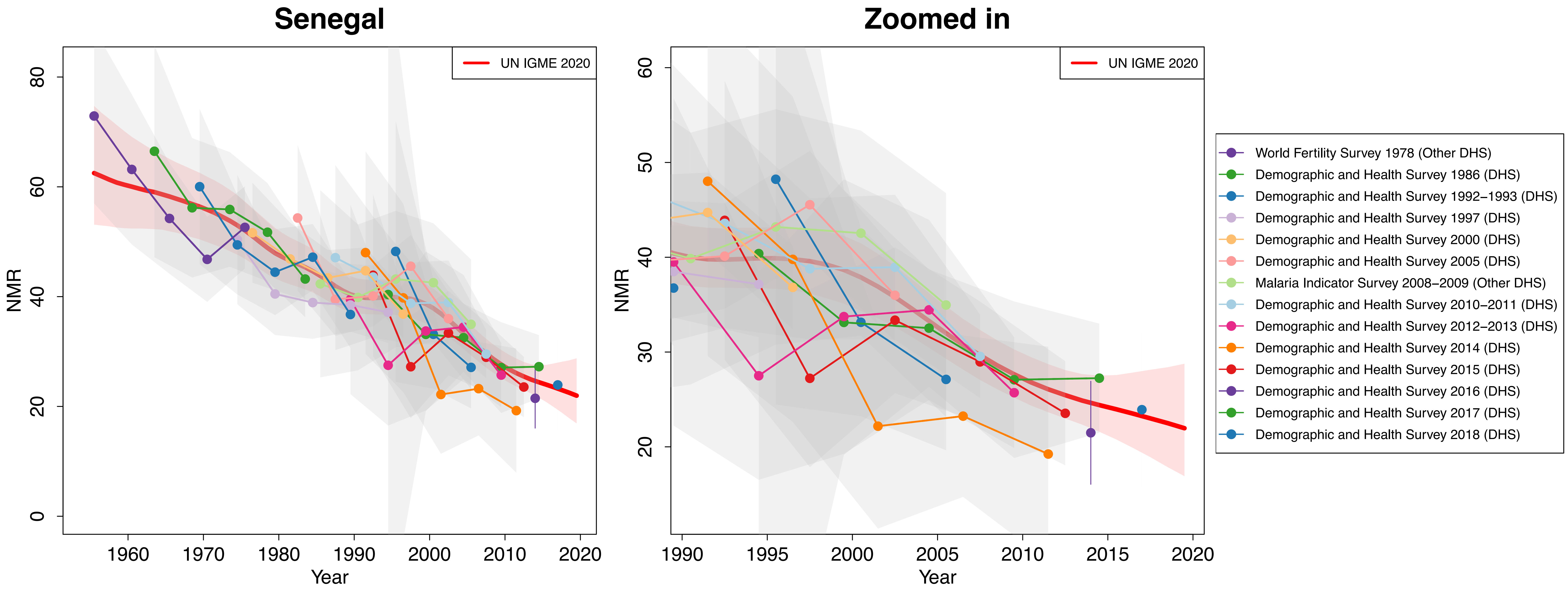
Senegal
Zoomed in
UN IGME 2020
NMR
Year
0
20
40
60
80
1960
1970
1980
1990
2000
2010
2020
30
50
1995
2005
2015
World Fertility Survey 1978 (Other DHS)
Demographic and Health Survey 1986 (DHS)
Demographic and Health Survey 1992–1993 (DHS)
Demographic and Health Survey 1997 (DHS)
Demographic and Health Survey 2000 (DHS)
Demographic and Health Survey 2005 (DHS)
Malaria Indicator Survey 2008–2009 (Other DHS)
Demographic and Health Survey 2010–2011 (DHS)
Demographic and Health Survey 2012–2013 (DHS)
Demographic and Health Survey 2014 (DHS)
Demographic and Health Survey 2015 (DHS)
Demographic and Health Survey 2016 (DHS)
Demographic and Health Survey 2017 (DHS)
Demographic and Health Survey 2018 (DHS)

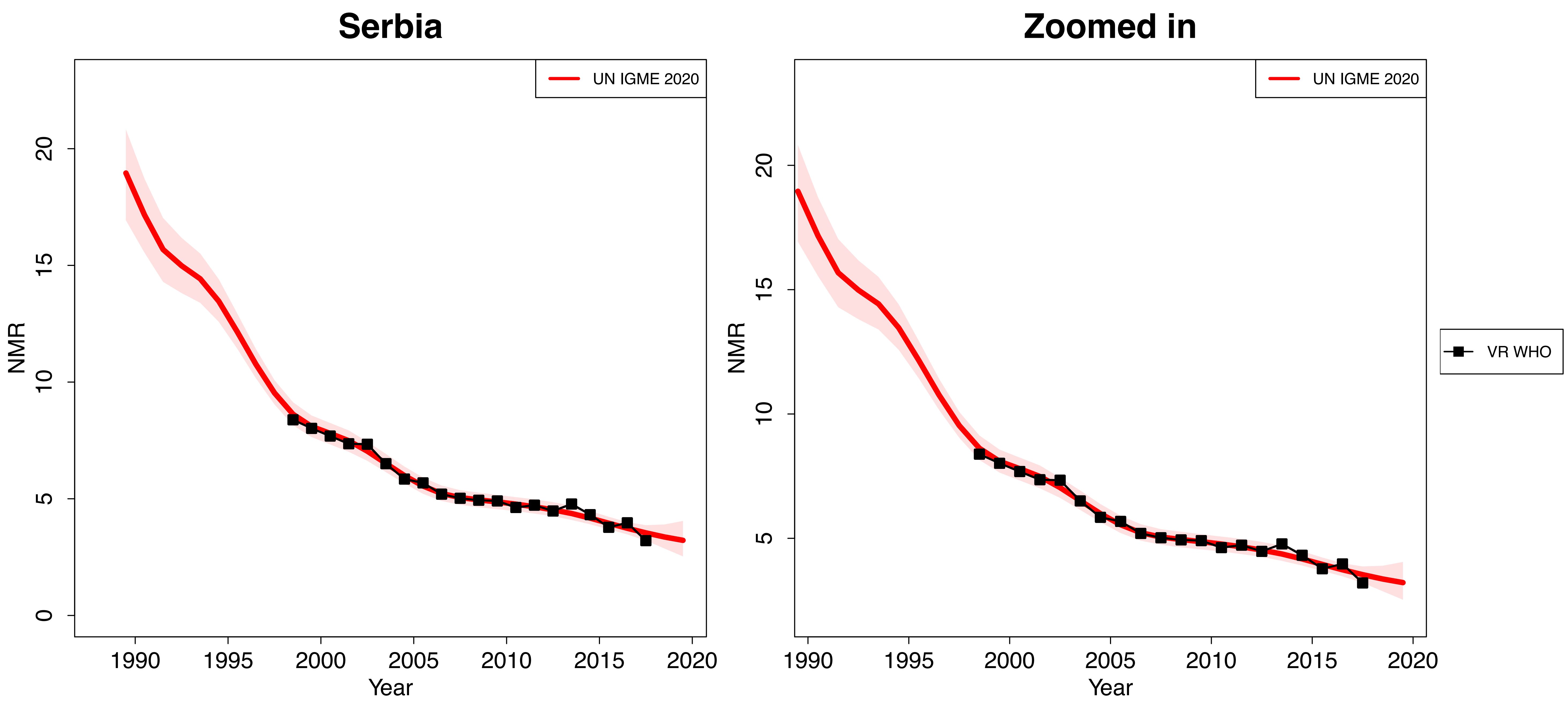
Serbia
Zoomed in
UN IGME 2020
UN IGME 2020
VR WHO
NMR
NMR
Year
Year
0
5
10
15
20
1990
1995
2000
2005
2010
2015
2020

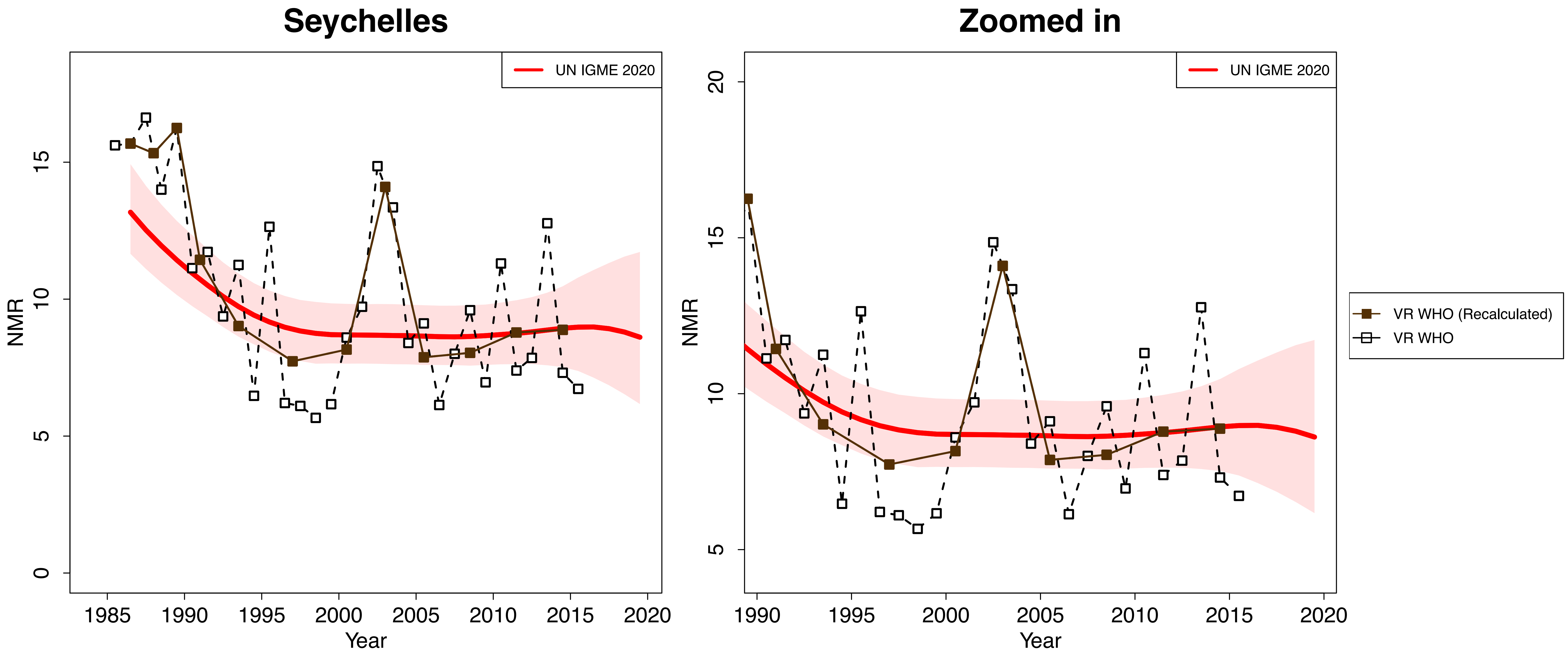

Seychelles
Zoomed in
UN IGME 2020
UN IGME 2020
VR WHO (Recalculated)
VR WHO
NMR
NMR
Year
Year
0
5
10
15
5
10
15
20
1985
1990
1995
2000
2005
2010
2015
2020
1990
1995
2000
2005
2010
2015
2020

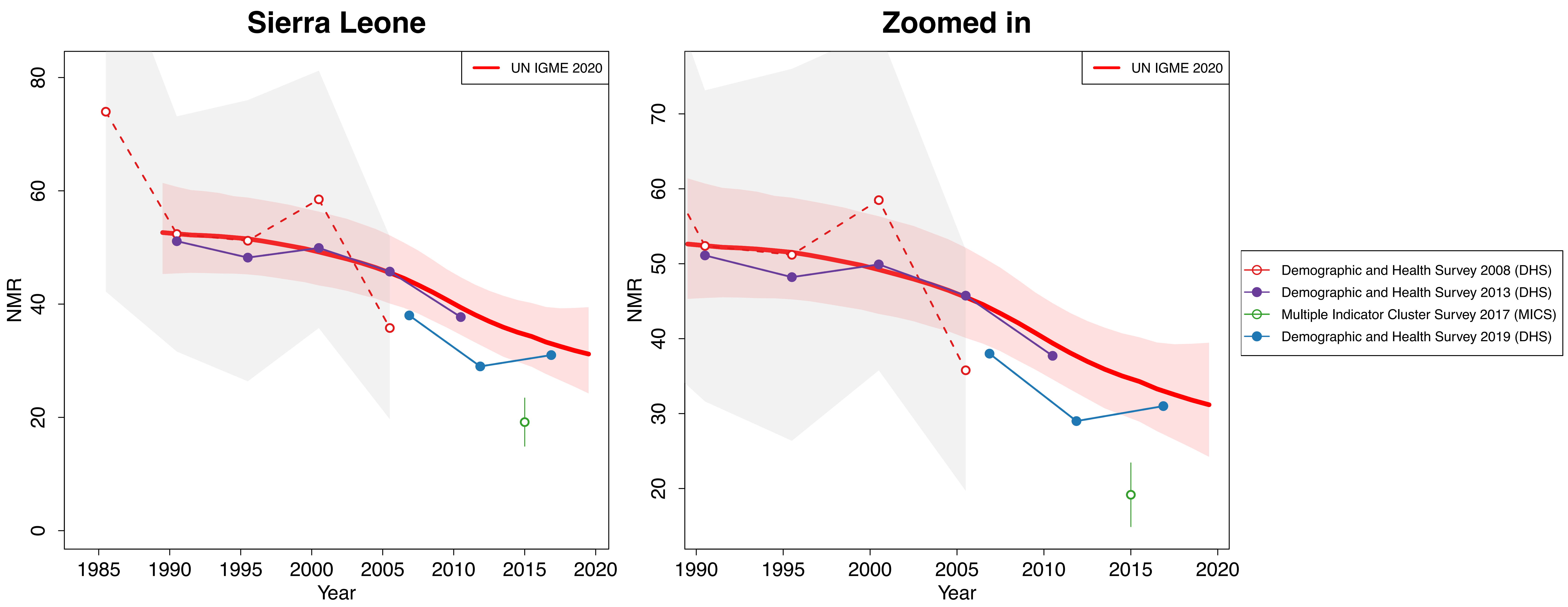

Sierra Leone
Zoomed in
UN IGME 2020
NMR
Year
Demographic and Health Survey 2008 (DHS)
Demographic and Health Survey 2013 (DHS)
Multiple Indicator Cluster Survey 2017 (MICS)
Demographic and Health Survey 2019 (DHS)

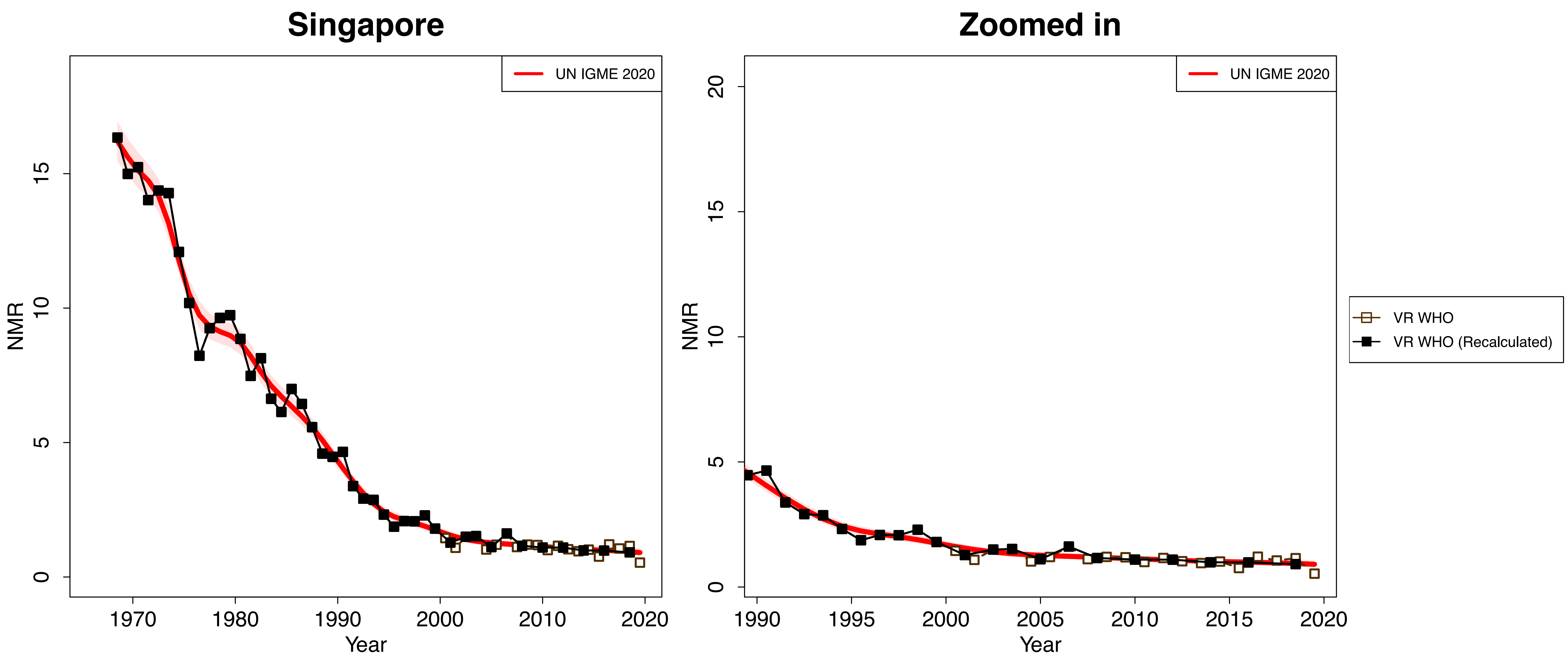
Singapore
Zoomed in
UN IGME 2020
NMR
Year
VR WHO
VR WHO (Recalculated)

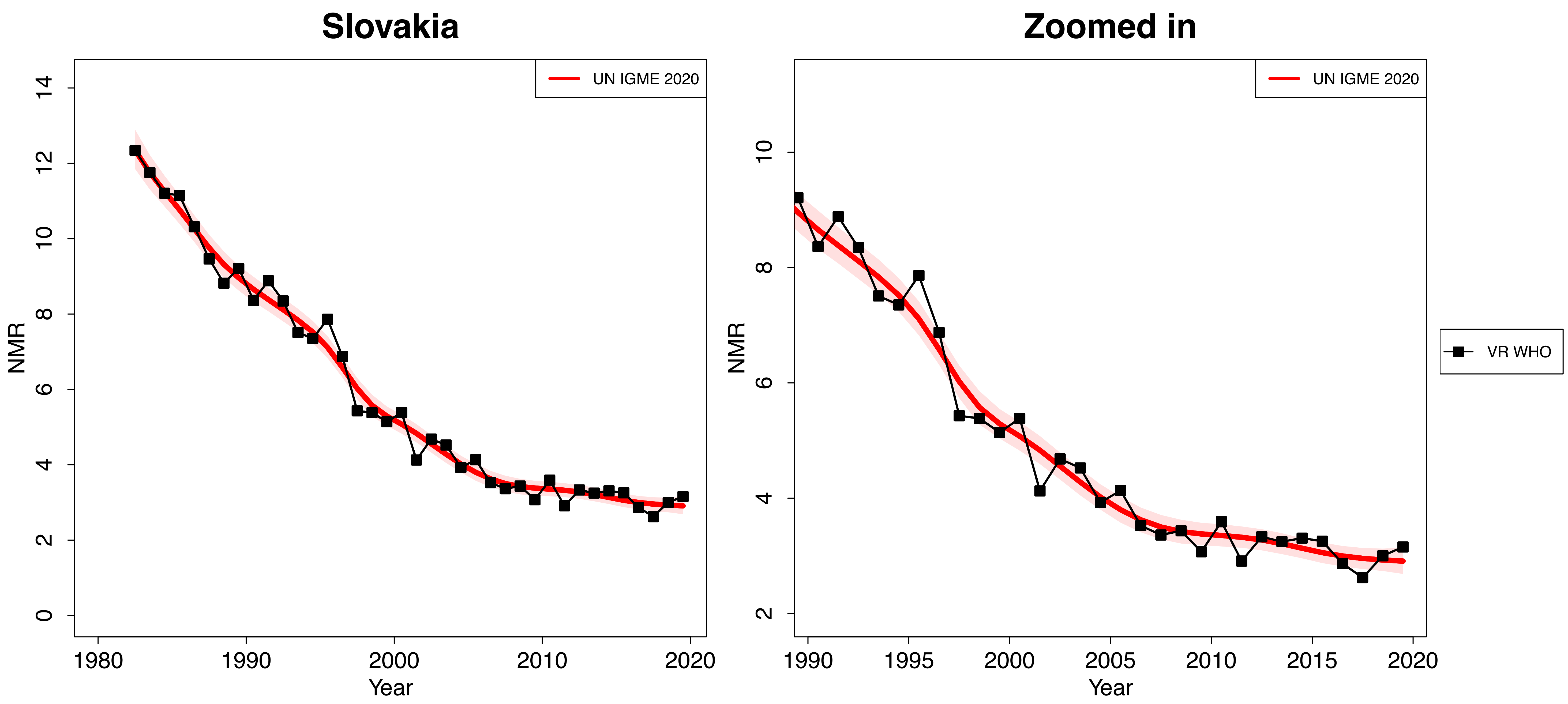

Slovakia
UN IGME 2020
NMR
Year
0
2
4
6
8
10
12
14
1980
1990
2000
2010
2020
Zoomed in
UN IGME 2020
NMR
Year
2
4
6
8
10
1990
1995
2000
2005
2010
2015
2020
VR WHO

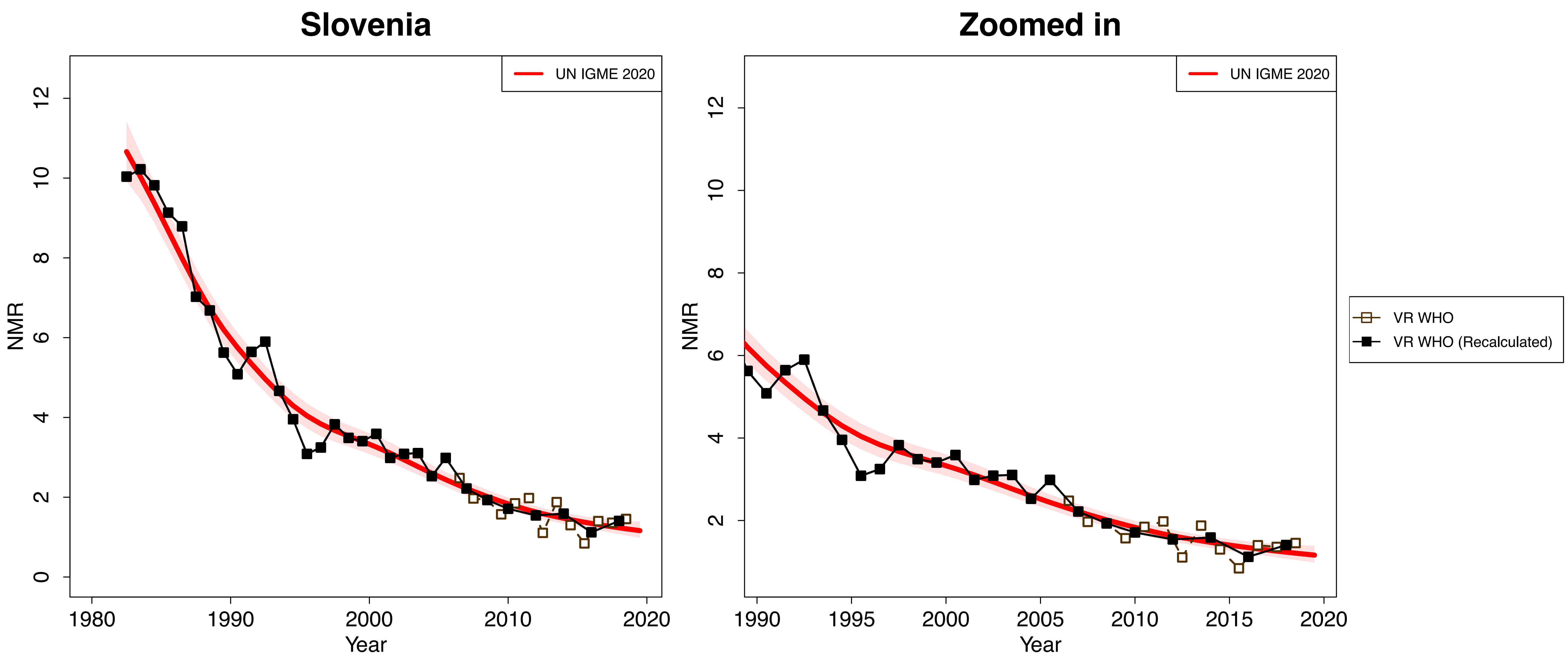

Slovenia
Zoomed in
UN IGME 2020
NMR
Year
VR WHO
VR WHO (Recalculated)

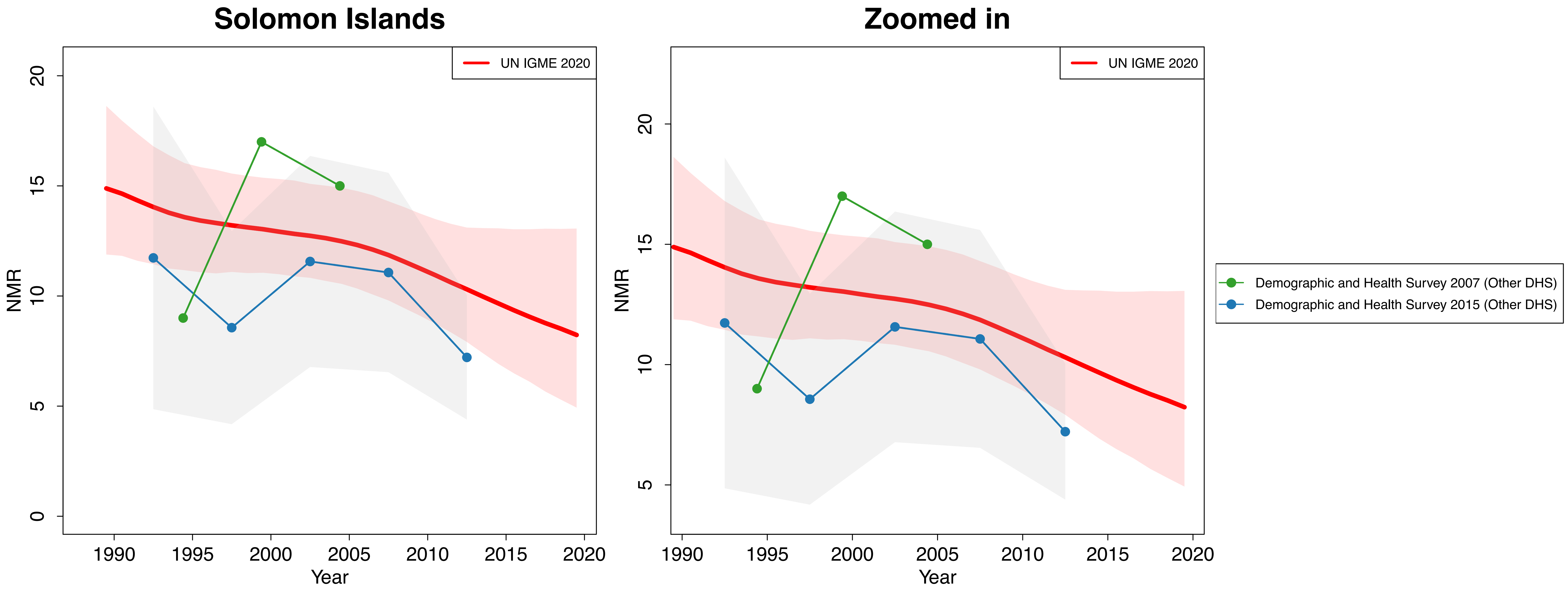

Solomon Islands
Zoomed in
UN IGME 2020
NMR
Year
Demographic and Health Survey 2007 (Other DHS)
Demographic and Health Survey 2015 (Other DHS)

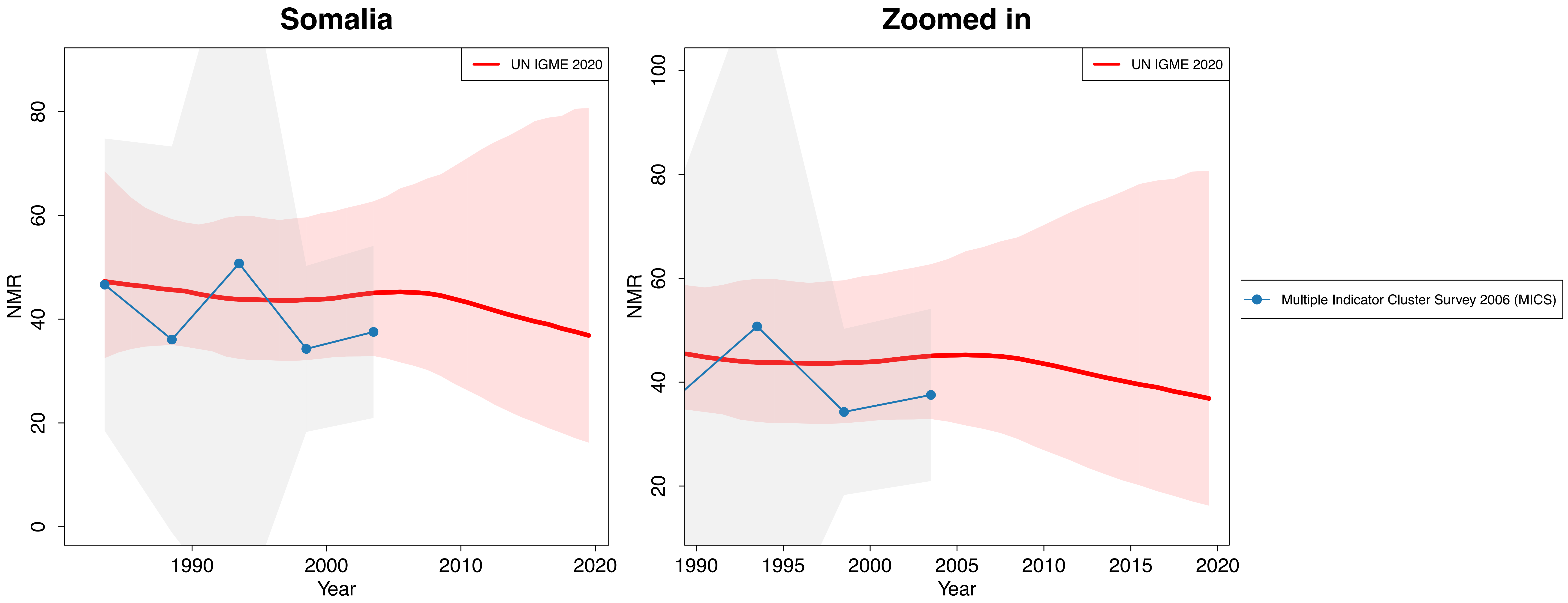

Somalia
Zoomed in
UN IGME 2020
UN IGME 2020
Multiple Indicator Cluster Survey 2006 (MICS)
NMR
Year
0
20
40
60
80
100
1990
1995
2000
2005
2010
2015
2020

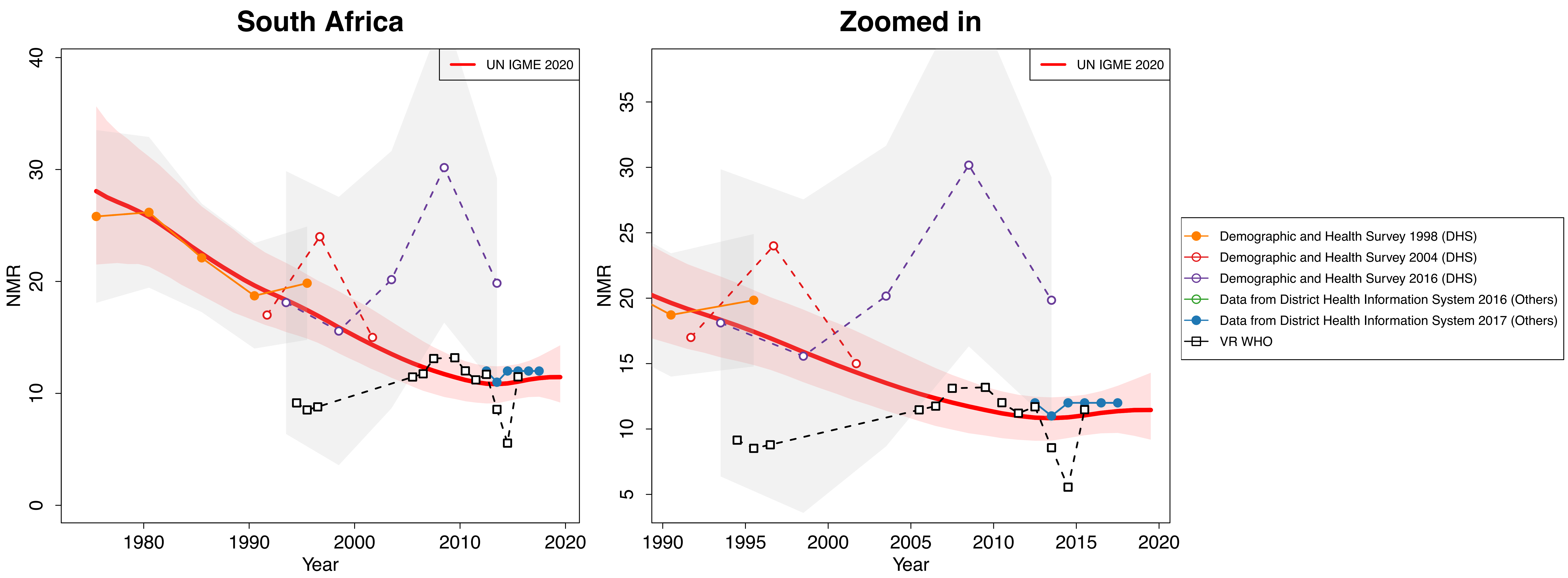

South Africa
Zoomed in
UN IGME 2020
NMR
Year
Demographic and Health Survey 1998 (DHS)
Demographic and Health Survey 2004 (DHS)
Demographic and Health Survey 2016 (DHS)
Data from District Health Information System 2016 (Others)
Data from District Health Information System 2017 (Others)
VR WHO

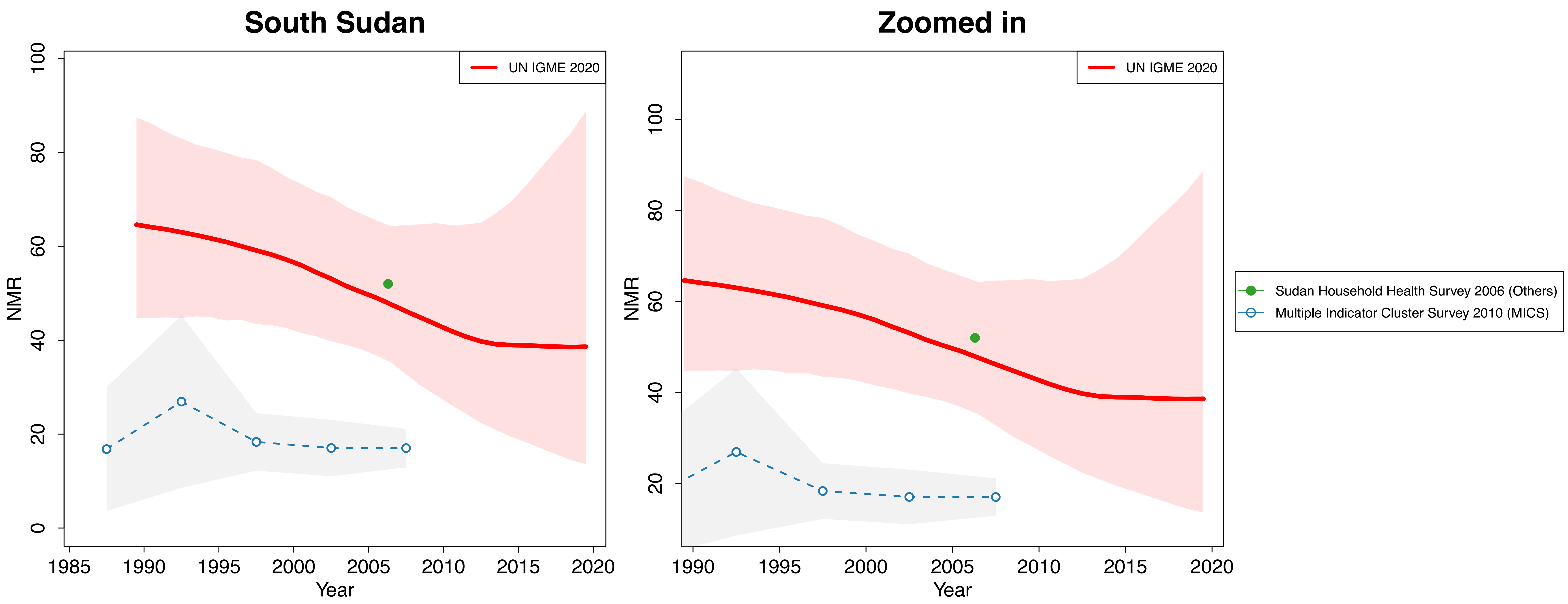

South Sudan
Zoomed in
UN IGME 2020
UN IGME 2020
Sudan Household Health Survey 2006 (Others)
Multiple Indicator Cluster Survey 2010 (MICS)
NMR
NMR
Year
Year
0
20
40
60
80
100
1985
1990
1995
2000
2005
2010
2015
2020

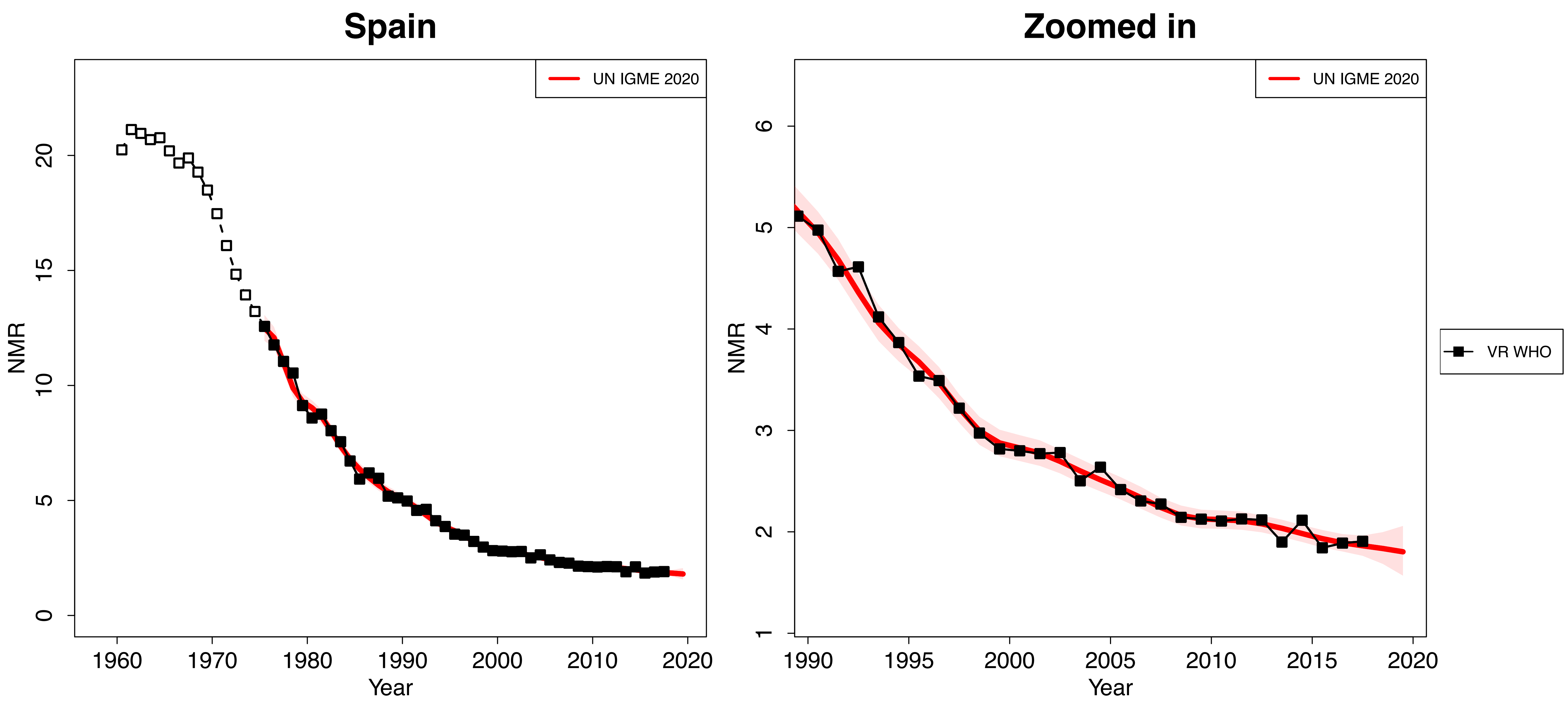

Spain
UN IGME 2020
NMR
0
5
10
15
20
1960
1970
1980
1990
2000
2010
2020
Year
Zoomed in
UN IGME 2020
NMR
1
2
3
4
5
6
1990
1995
2000
2005
2010
2015
2020
Year
VR WHO

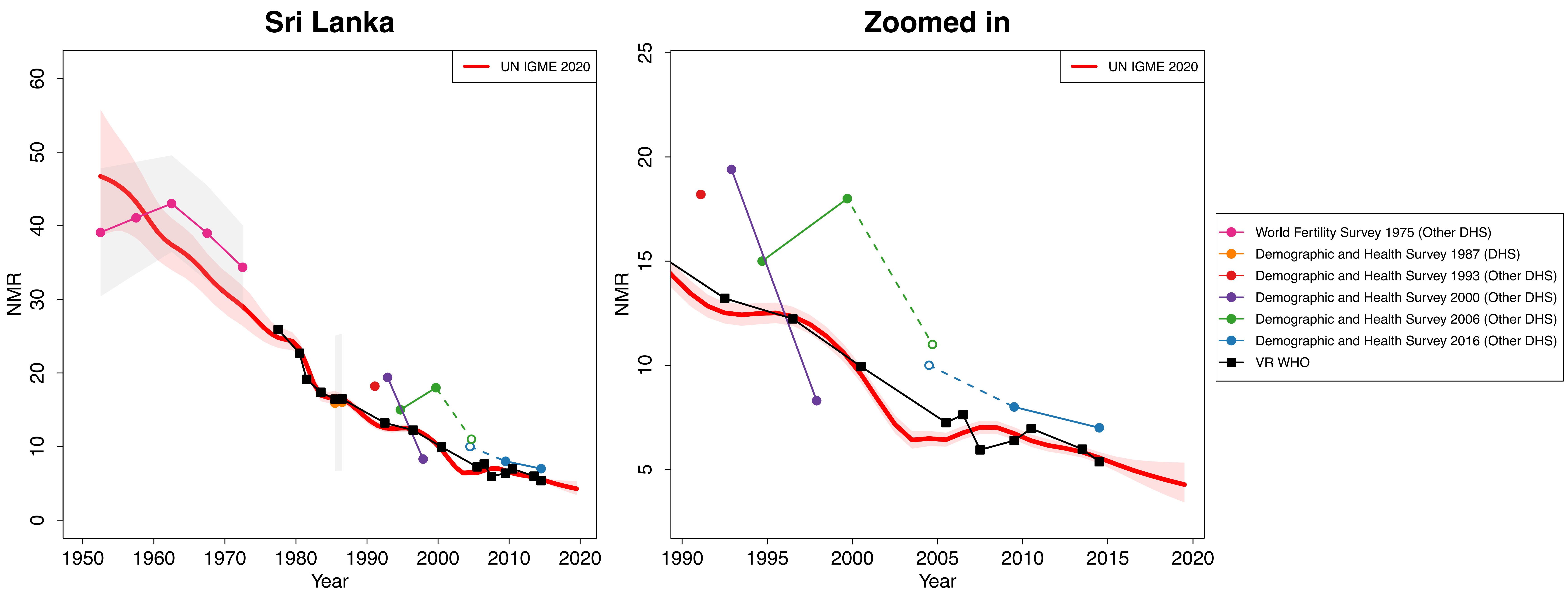
Sri Lanka
Zoomed in
UN IGME 2020
NMR
Year
World Fertility Survey 1975 (Other DHS)
Demographic and Health Survey 1987 (DHS)
Demographic and Health Survey 1993 (Other DHS)
Demographic and Health Survey 2000 (Other DHS)
Demographic and Health Survey 2006 (Other DHS)
Demographic and Health Survey 2016 (Other DHS)
VR WHO

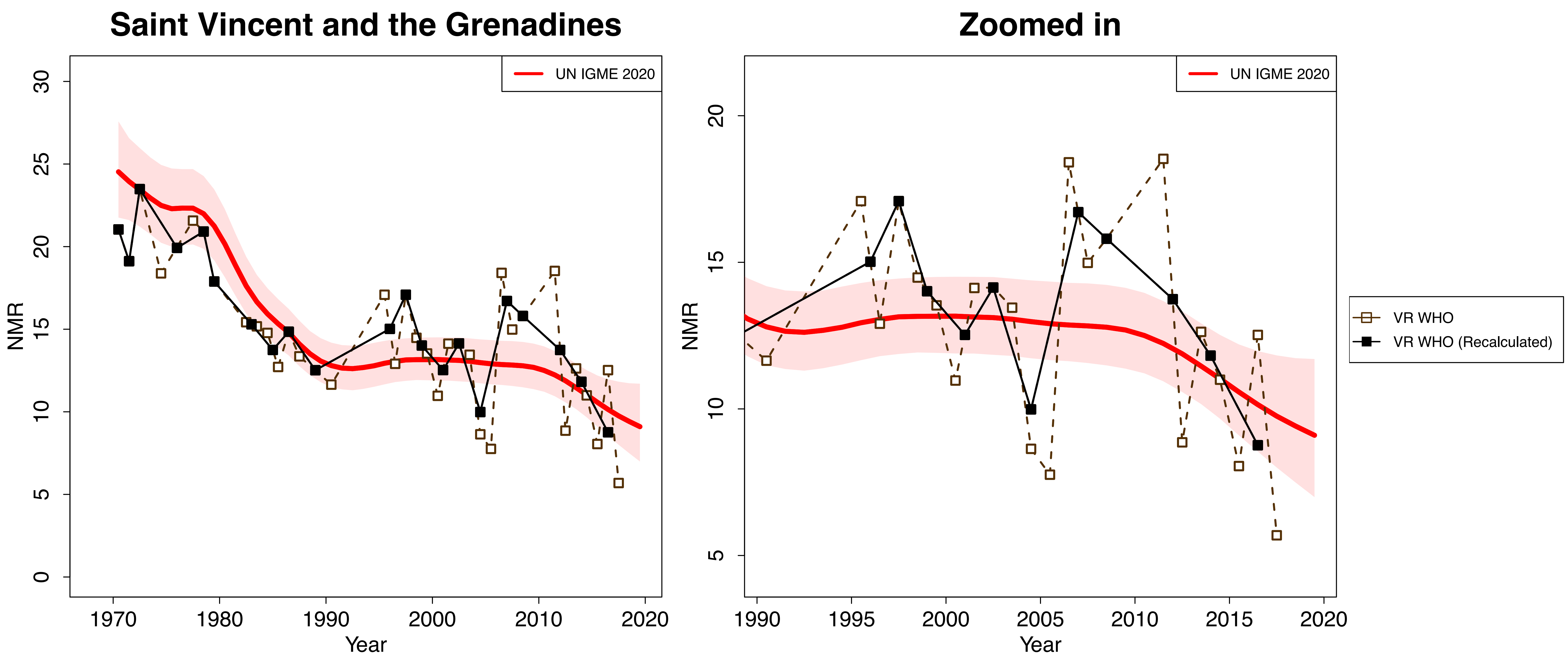

Saint Vincent and the Grenadines
Zoomed in
UN IGME 2020
NMR
Year
VR WHO
VR WHO (Recalculated)

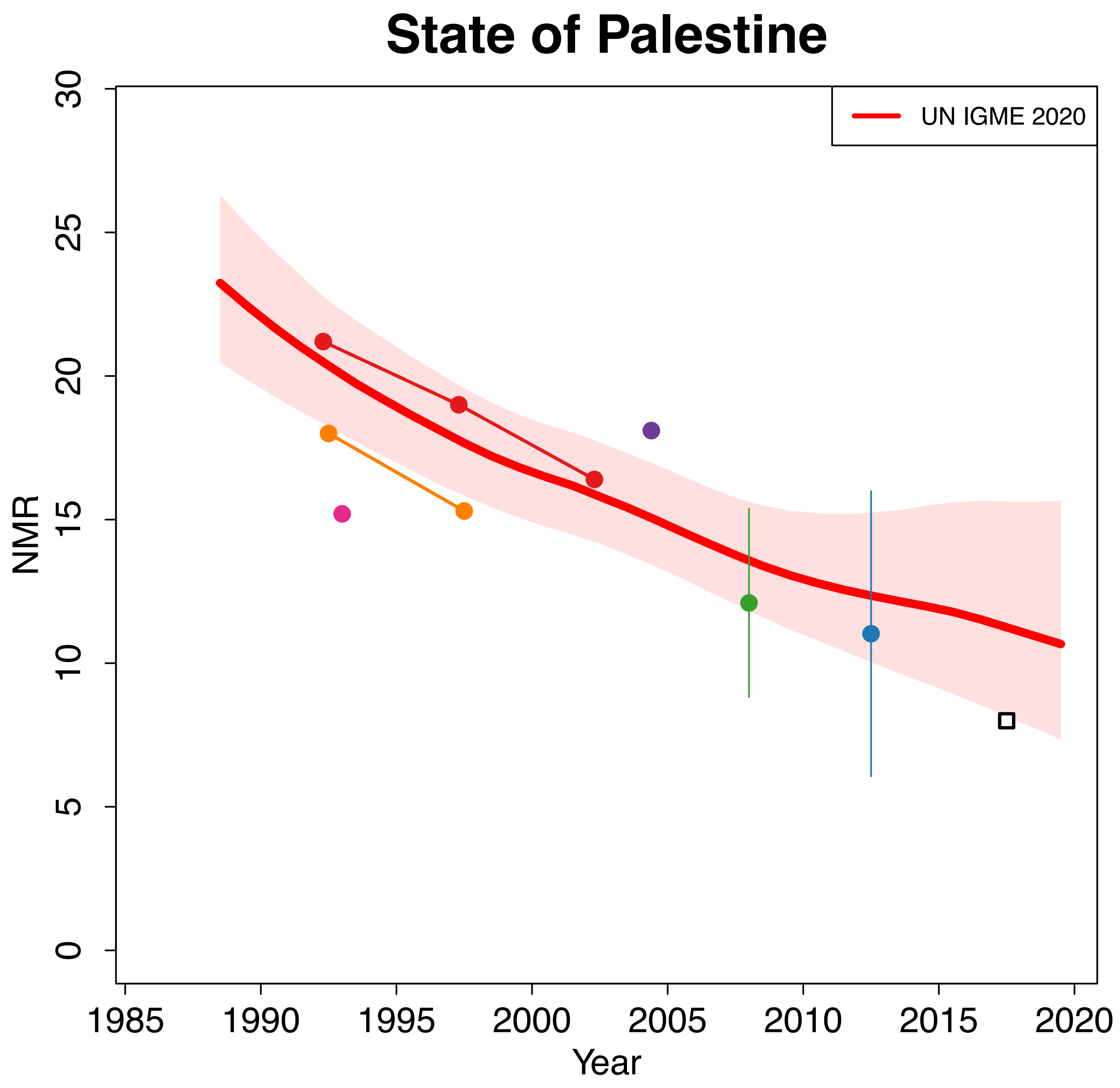
State of Palestine
UN IGME 2020
NMR
Year
0
5
10
15
20
25
30
1985
1990
1995
2000
2005
2010
2015
2020

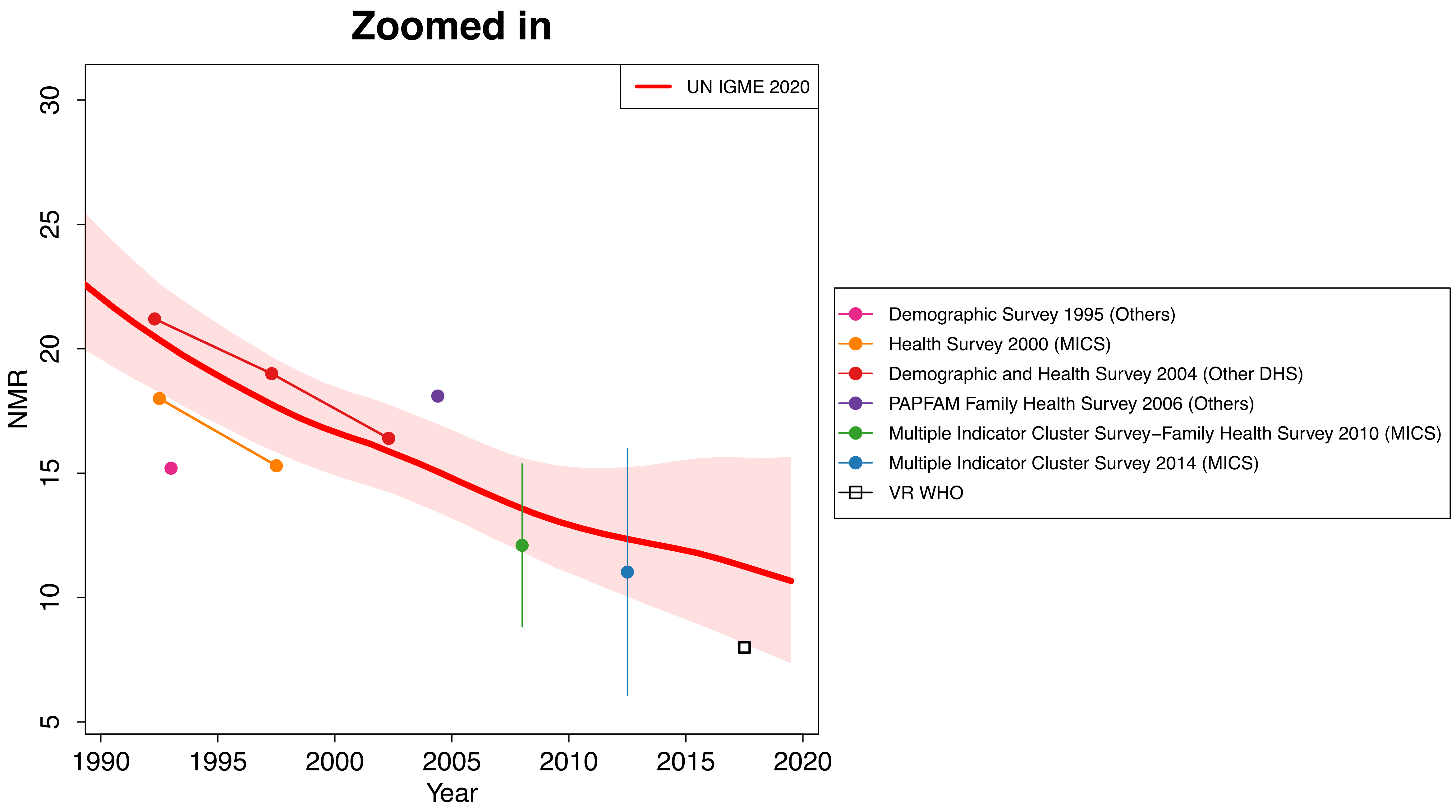
Zoomed in
UN IGME 2020
NMR
Year
5
10
15
20
25
30
1990
1995
2000
2005
2010
2015
2020
Demographic Survey 1995 (Others)
Health Survey 2000 (MICS)
Demographic and Health Survey 2004 (Other DHS)
PAPFAM Family Health Survey 2006 (Others)
Multiple Indicator Cluster Survey–Family Health Survey 2010 (MICS)
Multiple Indicator Cluster Survey 2014 (MICS)
VR WHO

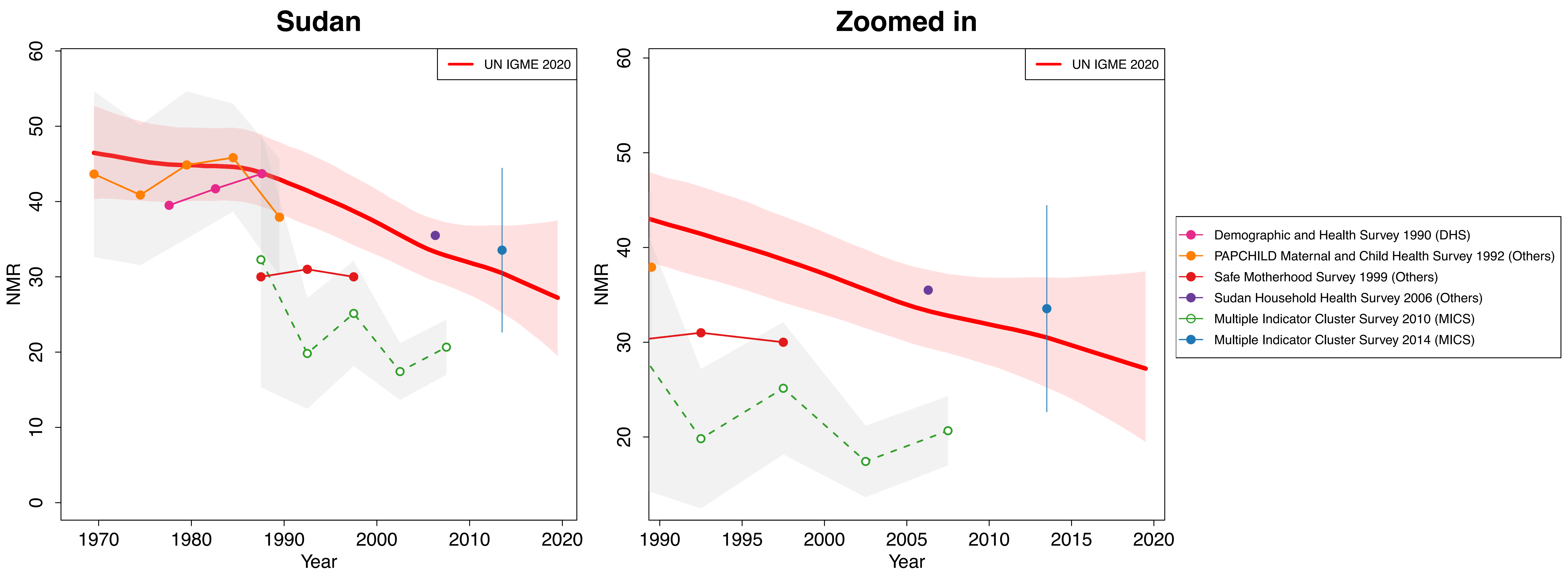

Sudan
Zoomed in
UN IGME 2020
NMR
Year
Demographic and Health Survey 1990 (DHS)
PAPCHILD Maternal and Child Health Survey 1992 (Others)
Safe Motherhood Survey 1999 (Others)
Sudan Household Health Survey 2006 (Others)
Multiple Indicator Cluster Survey 2010 (MICS)
Multiple Indicator Cluster Survey 2014 (MICS)

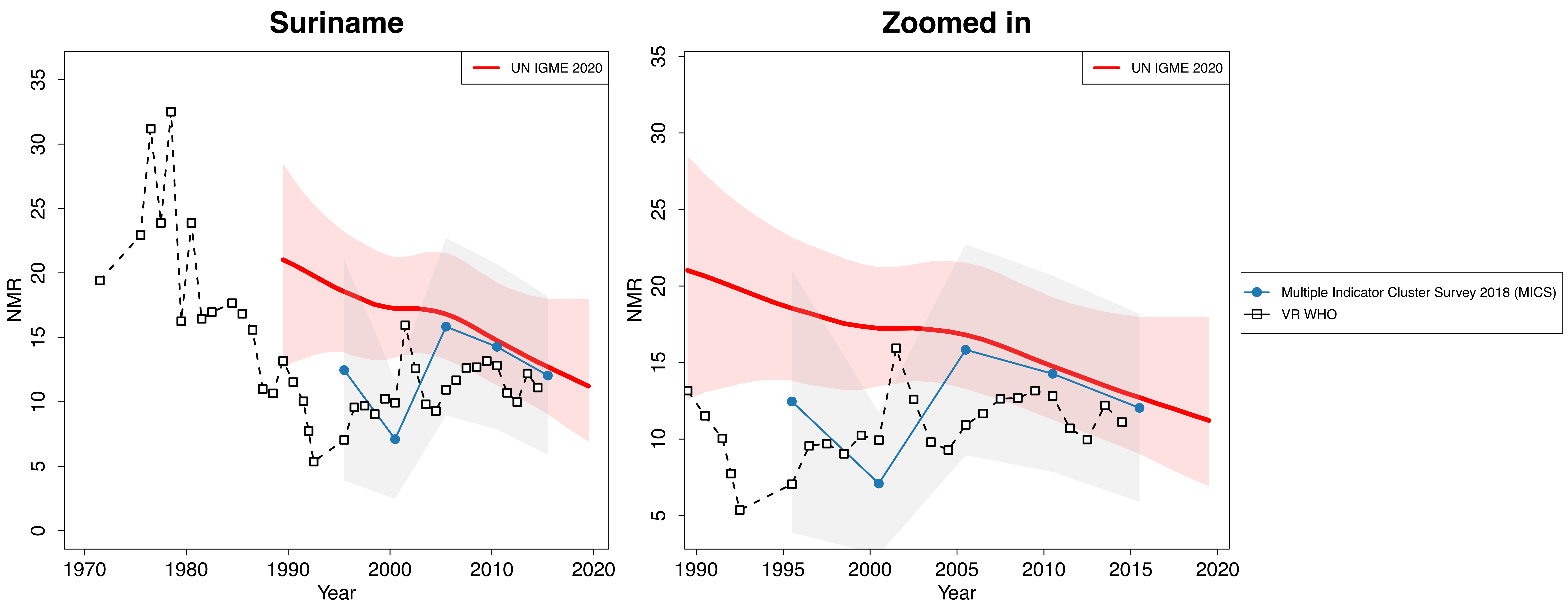

Suriname
Zoomed in
UN IGME 2020
NMR
Year
Multiple Indicator Cluster Survey 2018 (MICS)
VR WHO

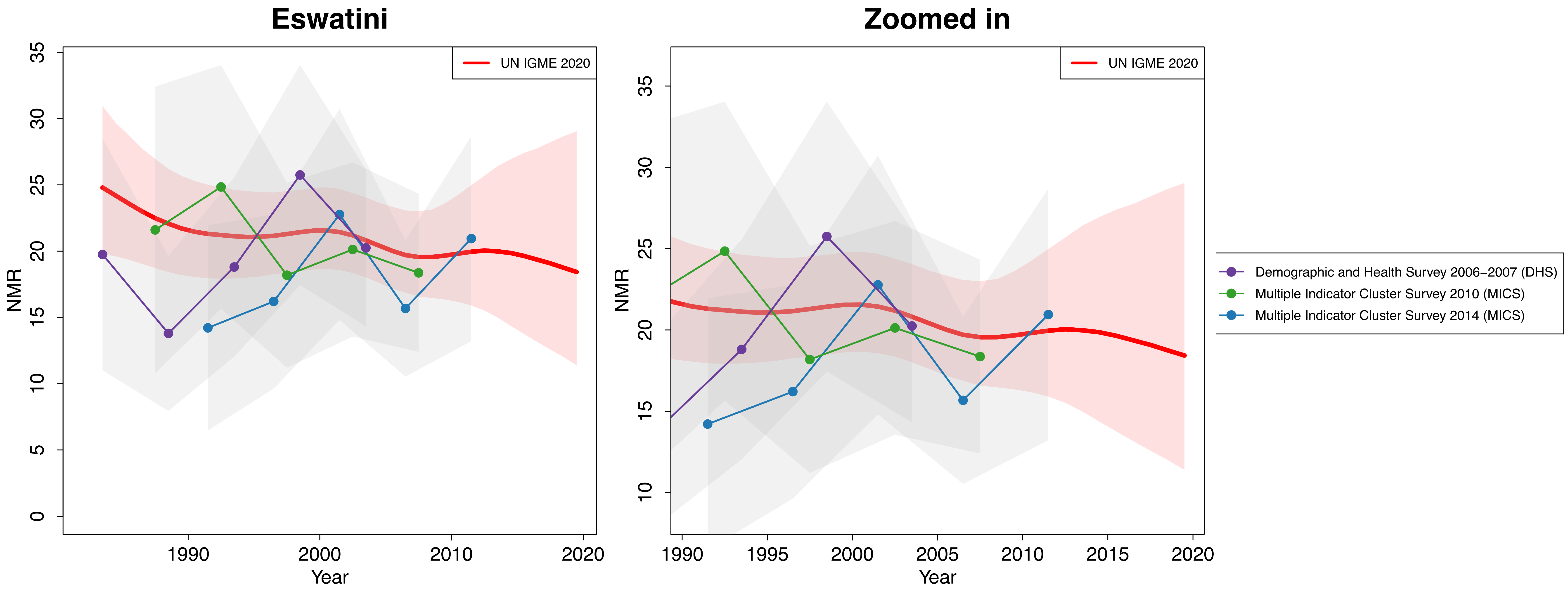

Eswatini
Zoomed in
UN IGME 2020
NMR
Year
Demographic and Health Survey 2006–2007 (DHS)
Multiple Indicator Cluster Survey 2010 (MICS)
Multiple Indicator Cluster Survey 2014 (MICS)

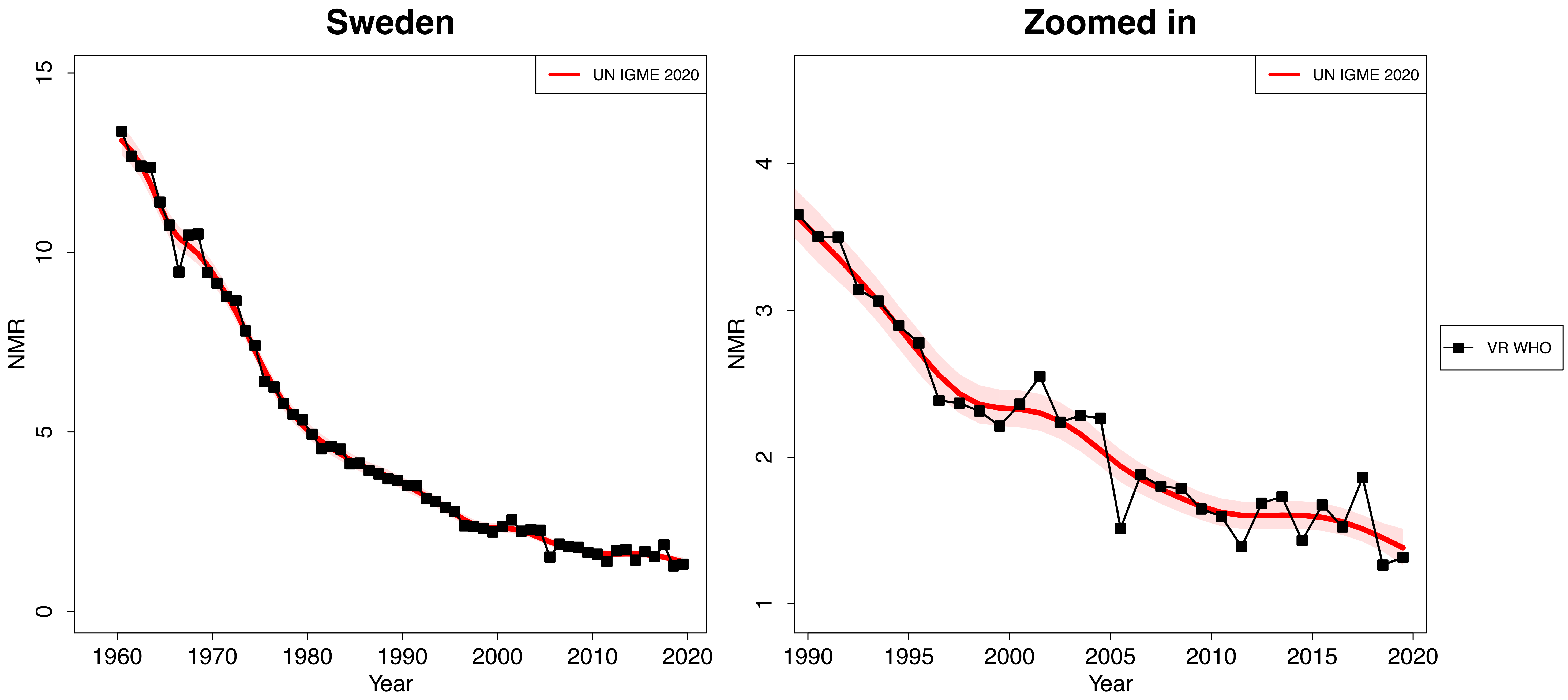
Sweden
Zoomed in
UN IGME 2020
UN IGME 2020
VR WHO
NMR
NMR
Year
Year

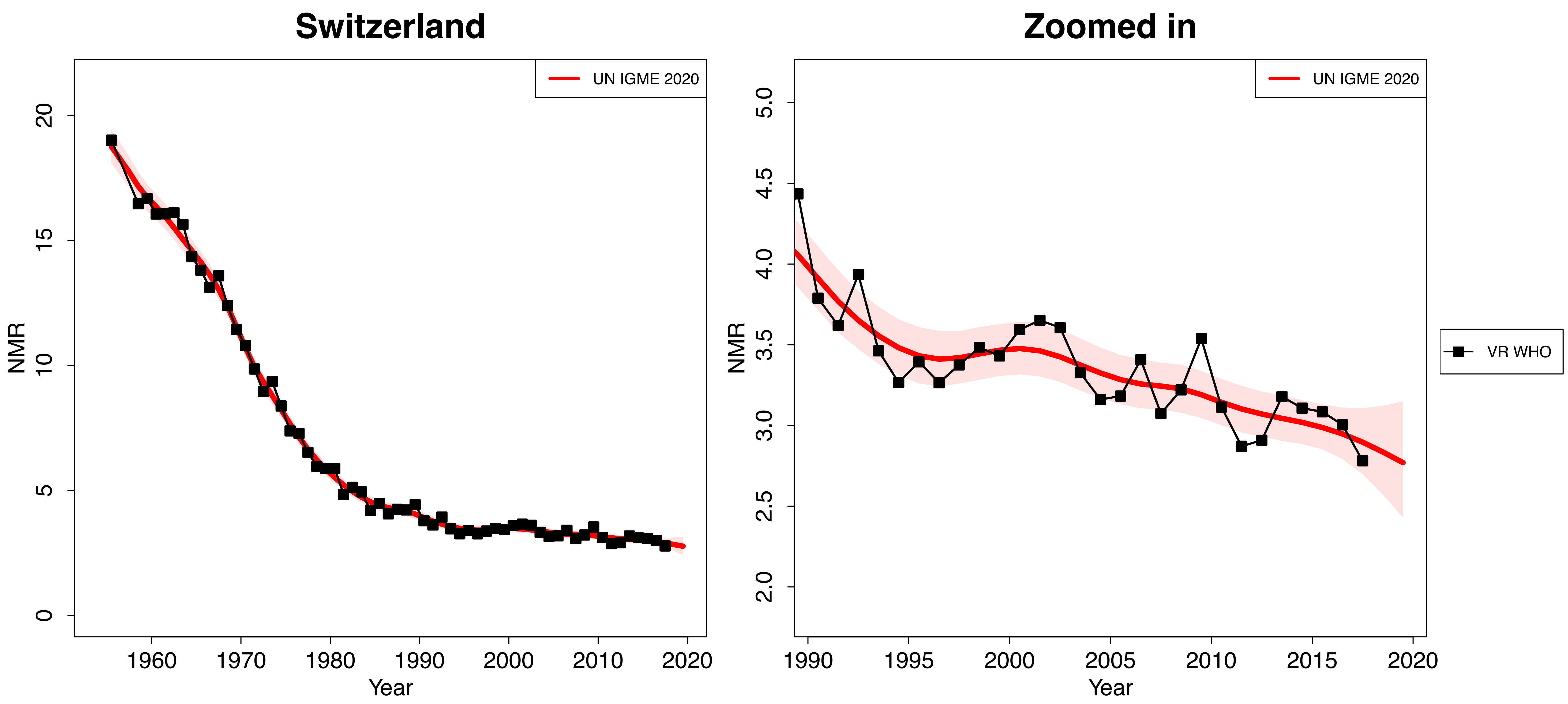

Switzerland
UN IGME 2020
NMR
Year
Zoomed in
UN IGME 2020
NMR
Year
VR WHO

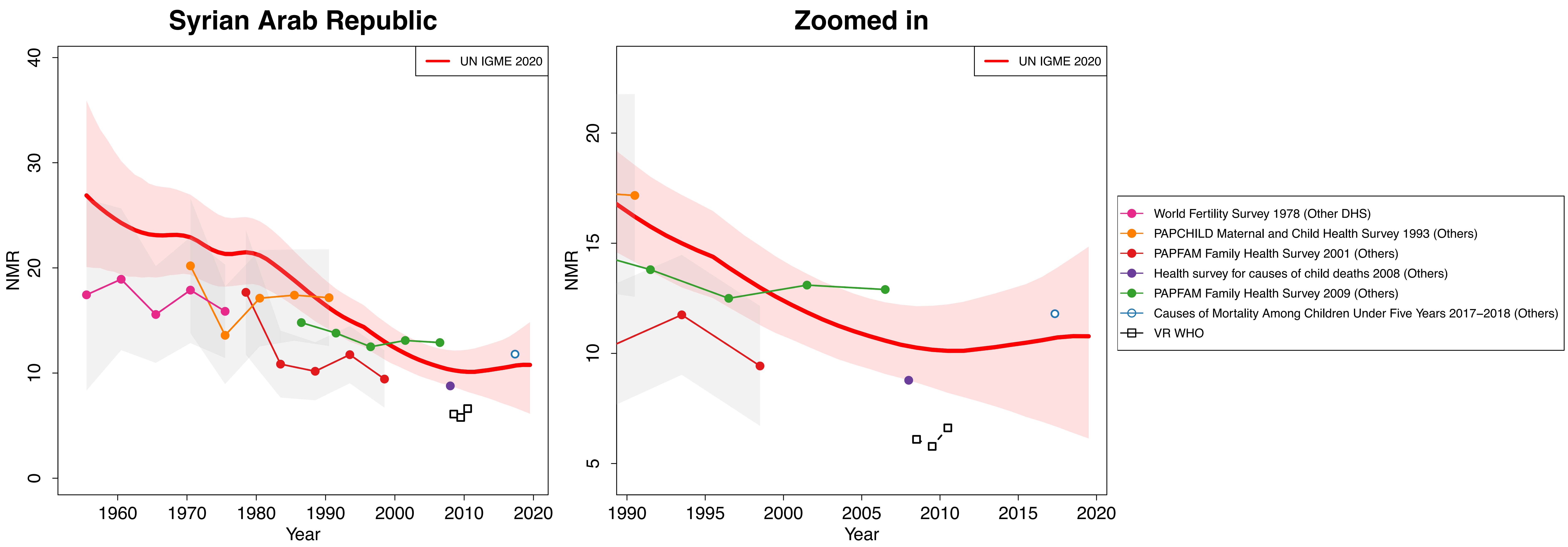

Syrian Arab Republic
Zoomed in
UN IGME 2020
NMR
Year
World Fertility Survey 1978 (Other DHS)
PAPCHILD Maternal and Child Health Survey 1993 (Others)
PAPFAM Family Health Survey 2001 (Others)
Health survey for causes of child deaths 2008 (Others)
PAPFAM Family Health Survey 2009 (Others)
Causes of Mortality Among Children Under Five Years 2017–2018 (Others)
VR WHO

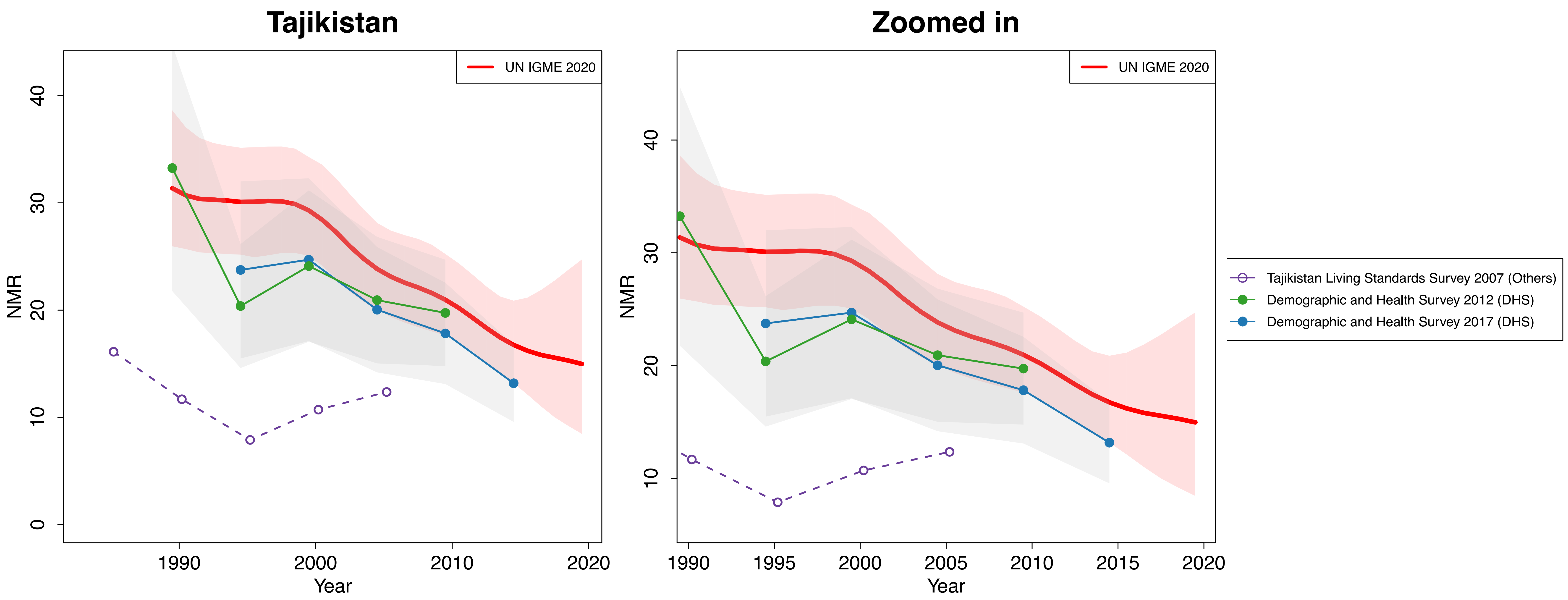

Tajikistan
Zoomed in
UN IGME 2020
NMR
Year
Tajikistan Living Standards Survey 2007 (Others)
Demographic and Health Survey 2012 (DHS)
Demographic and Health Survey 2017 (DHS)

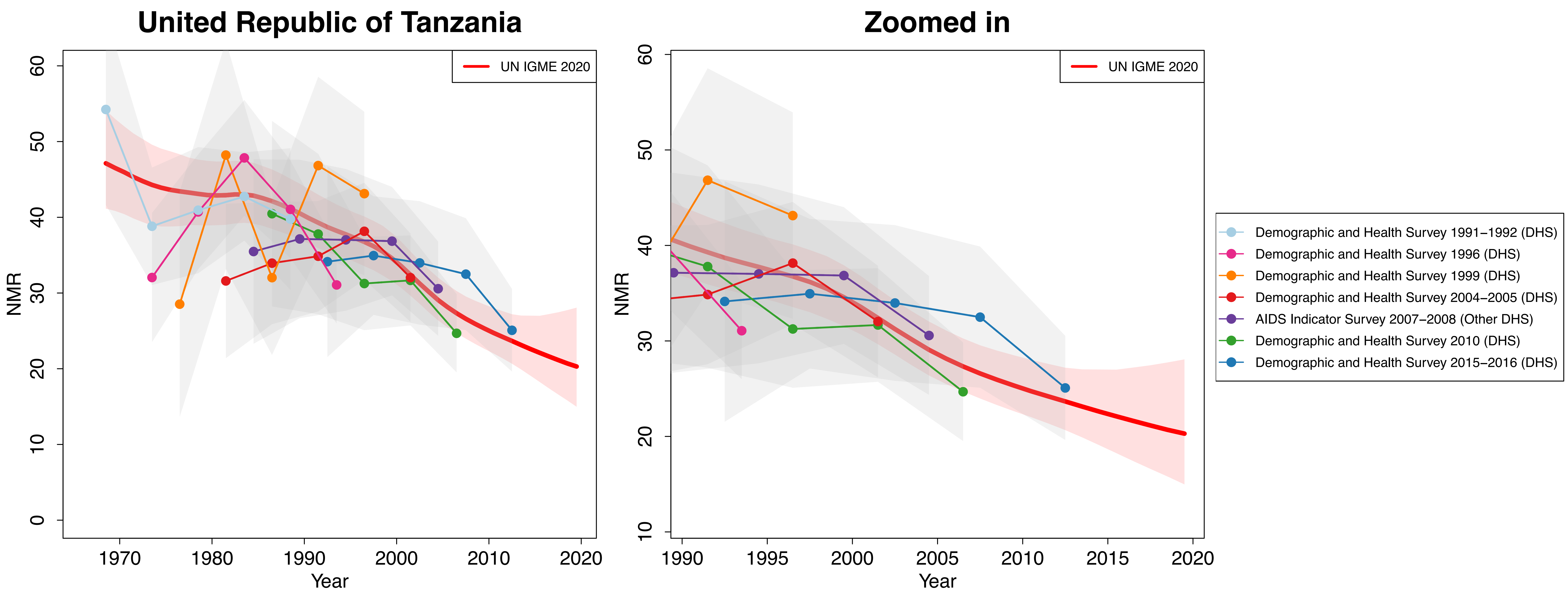

United Republic of Tanzania
Zoomed in
UN IGME 2020
UN IGME 2020
NMR
NMR
Year
Year
0
10
20
30
40
50
60
1970
1980
1990
2000
2010
2020
10
20
30
40
50
60
1990
1995
2000
2005
2010
2015
2020
Demographic and Health Survey 1991–1992 (DHS)
Demographic and Health Survey 1996 (DHS)
Demographic and Health Survey 1999 (DHS)
Demographic and Health Survey 2004–2005 (DHS)
AIDS Indicator Survey 2007–2008 (Other DHS)
Demographic and Health Survey 2010 (DHS)
Demographic and Health Survey 2015–2016 (DHS)

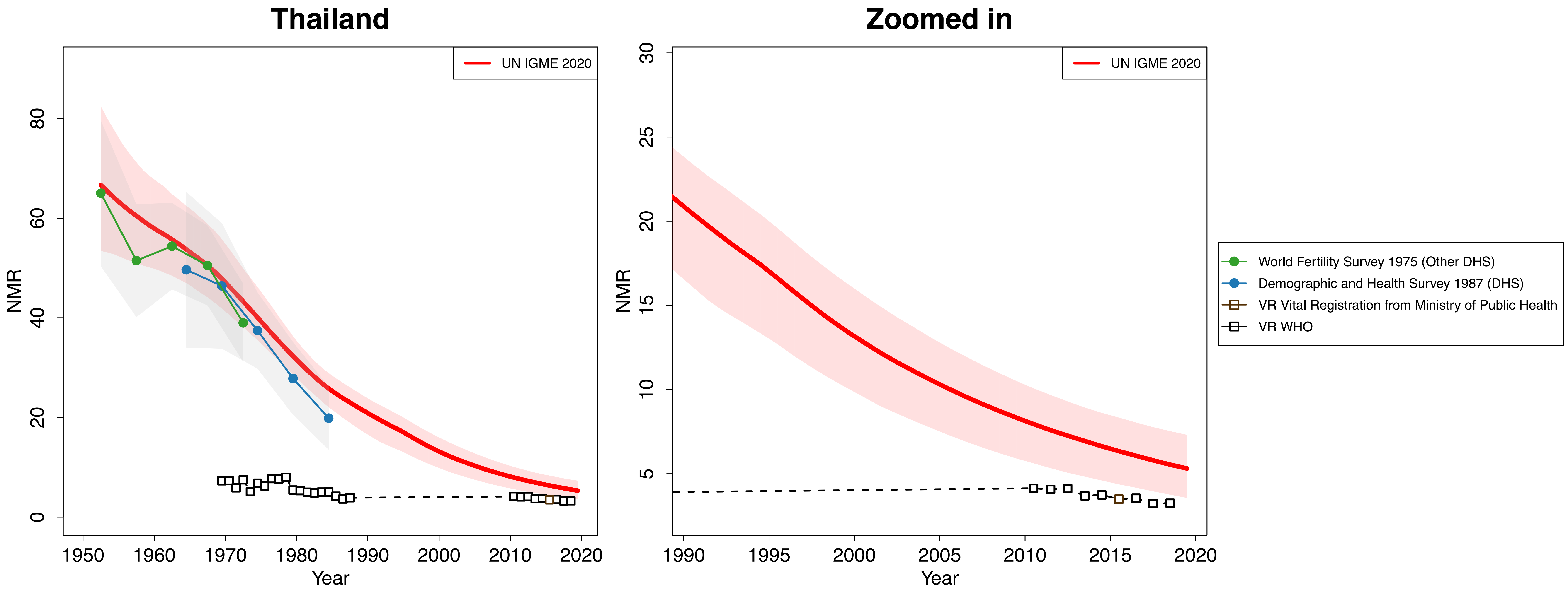

Thailand
UN IGME 2020
NMR
Year
Zoomed in
UN IGME 2020
World Fertility Survey 1975 (Other DHS)
Demographic and Health Survey 1987 (DHS)
VR Vital Registration from Ministry of Public Health
VR WHO

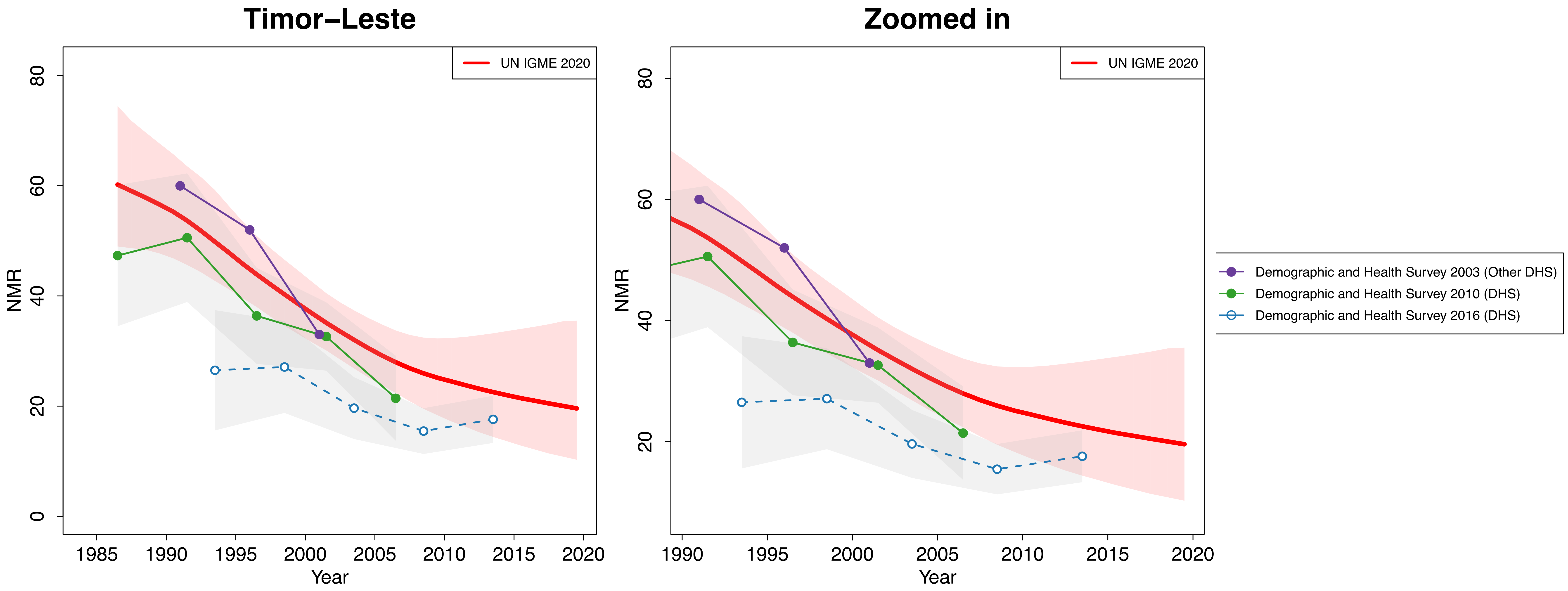
Timor–Leste
Zoomed in
UN IGME 2020
UN IGME 2020
NMR
NMR
Year
Year
0
20
40
60
80
20
40
60
80
1985
1990
1995
2000
2005
2010
2015
2020
1990
1995
2000
2005
2010
2015
2020
Demographic and Health Survey 2003 (Other DHS)
Demographic and Health Survey 2010 (DHS)
Demographic and Health Survey 2016 (DHS)

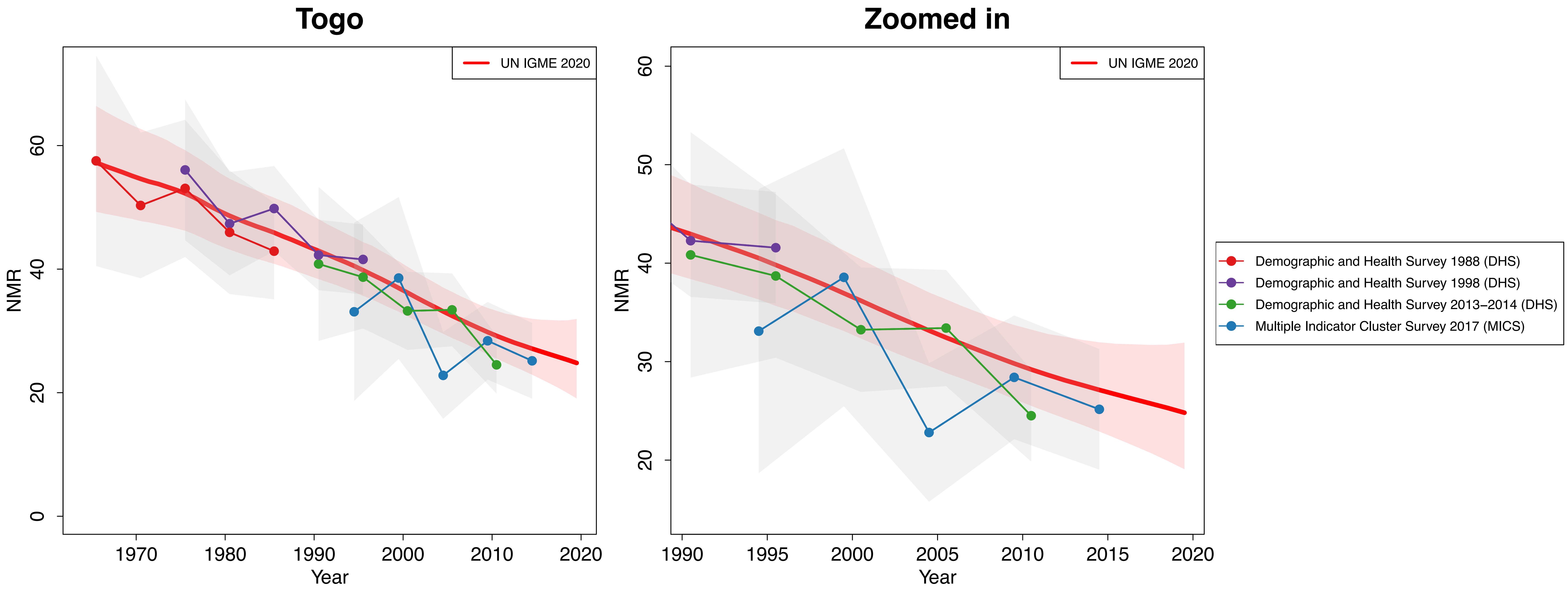

Togo
Zoomed in
UN IGME 2020
NMR
Year
Demographic and Health Survey 1988 (DHS)
Demographic and Health Survey 1998 (DHS)
Demographic and Health Survey 2013–2014 (DHS)
Multiple Indicator Cluster Survey 2017 (MICS)

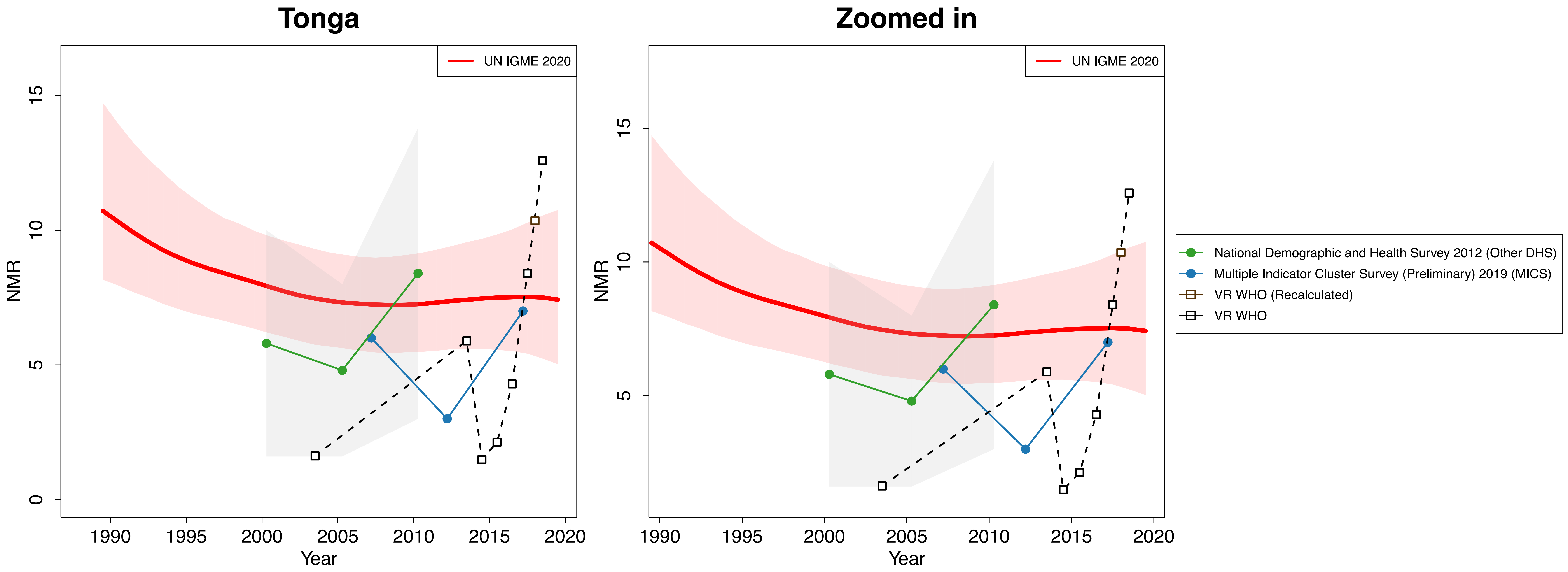
Tonga
Zoomed in
UN IGME 2020
NMR
Year
National Demographic and Health Survey 2012 (Other DHS)
Multiple Indicator Cluster Survey (Preliminary) 2019 (MICS)
VR WHO (Recalculated)
VR WHO
1990
1995
2000
2005
2010
2015
2020
0
5
10
15

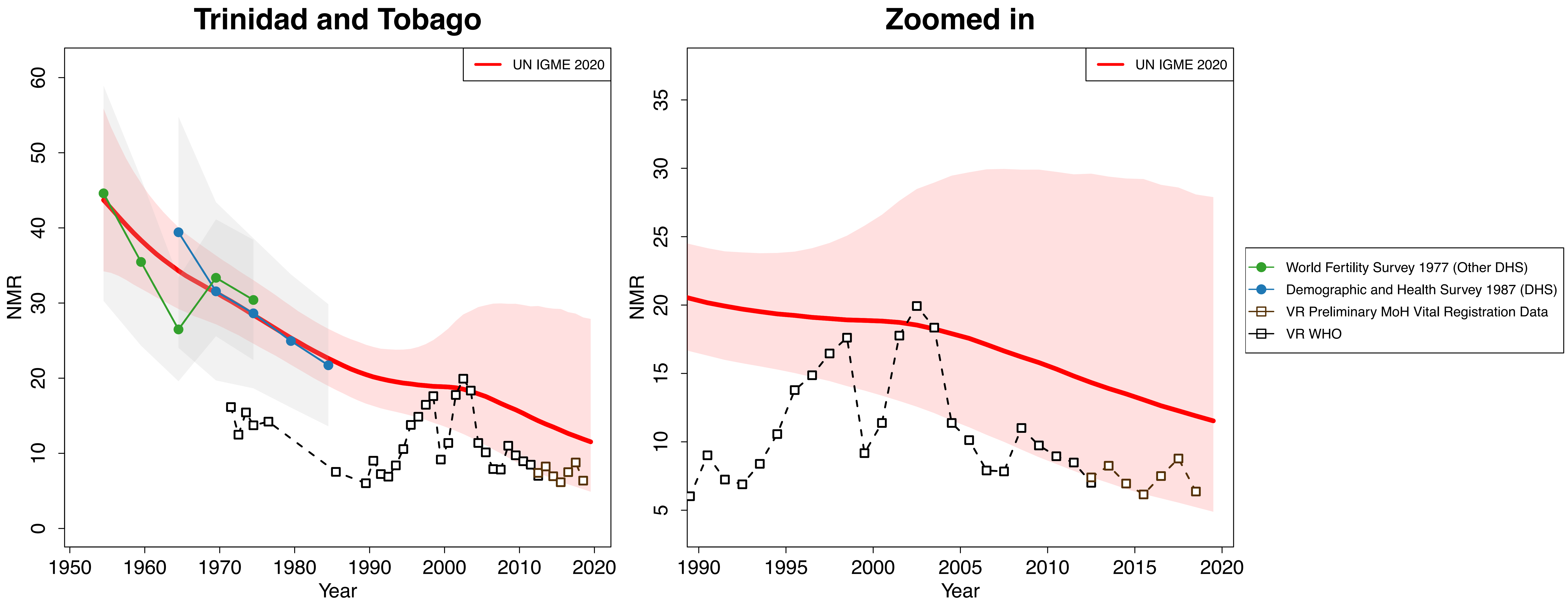

Trinidad and Tobago
Zoomed in
UN IGME 2020
NMR
Year
World Fertility Survey 1977 (Other DHS)
Demographic and Health Survey 1987 (DHS)
VR Preliminary MoH Vital Registration Data
VR WHO

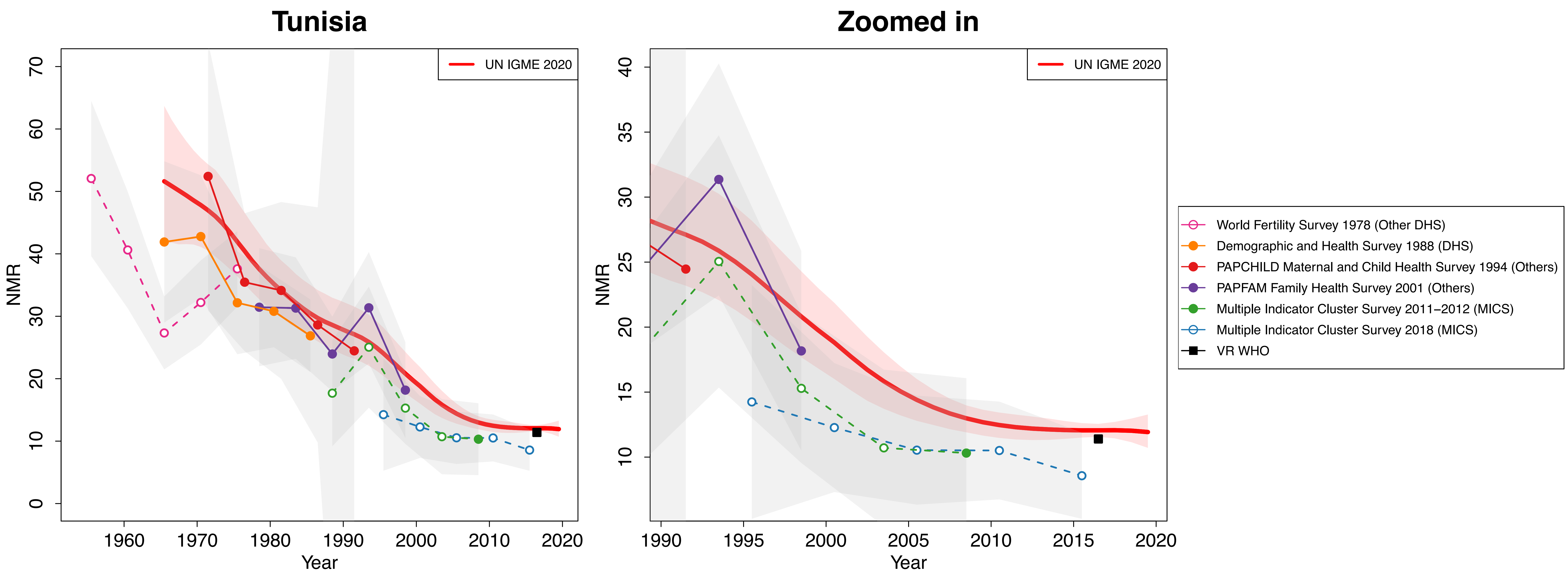
Tunisia
Zoomed in
UN IGME 2020
NMR
Year
World Fertility Survey 1978 (Other DHS)
Demographic and Health Survey 1988 (DHS)
PAPCHILD Maternal and Child Health Survey 1994 (Others)
PAPFAM Family Health Survey 2001 (Others)
Multiple Indicator Cluster Survey 2011–2012 (MICS)
Multiple Indicator Cluster Survey 2018 (MICS)
VR WHO

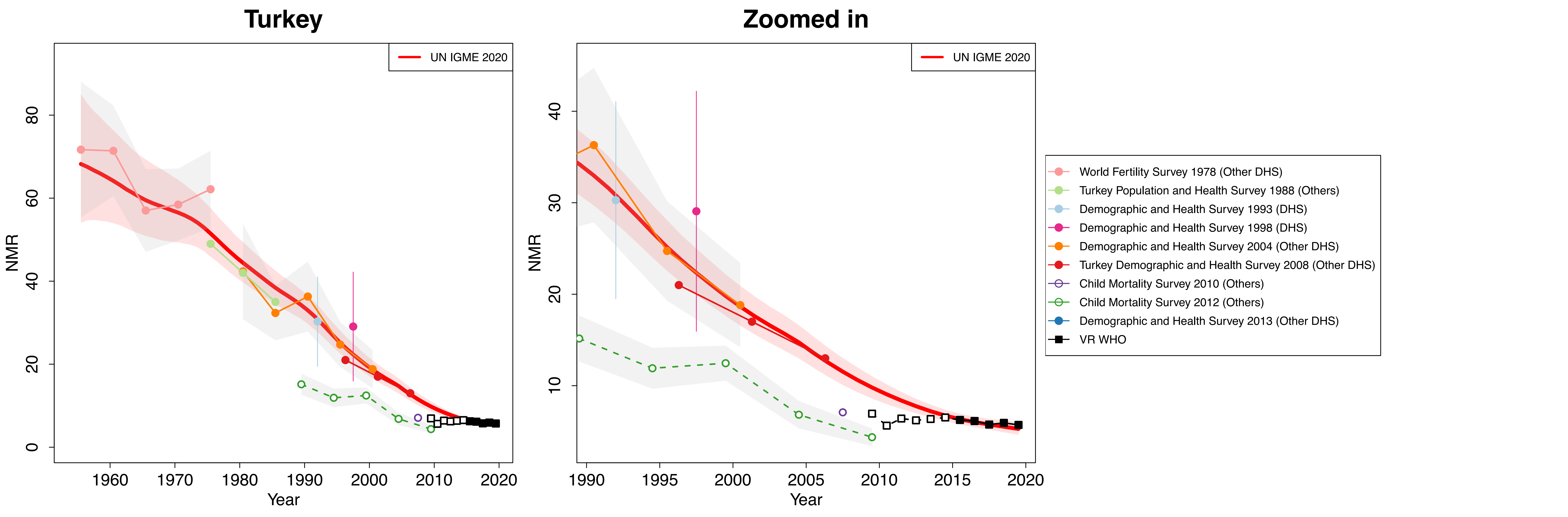

Turkey
Zoomed in
UN IGME 2020
NMR
Year
World Fertility Survey 1978 (Other DHS)
Turkey Population and Health Survey 1988 (Others)
Demographic and Health Survey 1993 (DHS)
Demographic and Health Survey 1998 (DHS)
Demographic and Health Survey 2004 (Other DHS)
Turkey Demographic and Health Survey 2008 (Other DHS)
Child Mortality Survey 2010 (Others)
Child Mortality Survey 2012 (Others)
Demographic and Health Survey 2013 (Other DHS)
VR WHO

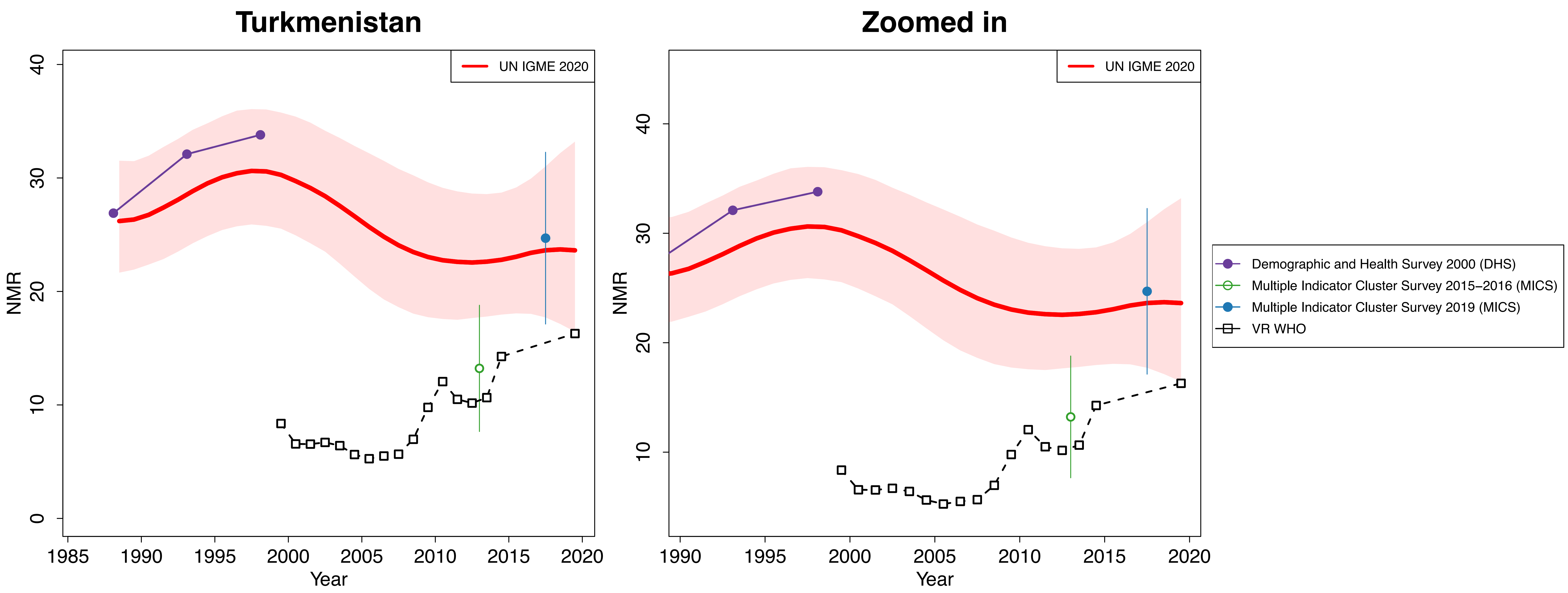

Turkmenistan
Zoomed in
UN IGME 2020
UN IGME 2020
NMR
NMR
Year
Year
0
10
20
30
40
1985
1990
1995
2000
2005
2010
2015
2020
Demographic and Health Survey 2000 (DHS)
Multiple Indicator Cluster Survey 2015–2016 (MICS)
Multiple Indicator Cluster Survey 2019 (MICS)
VR WHO

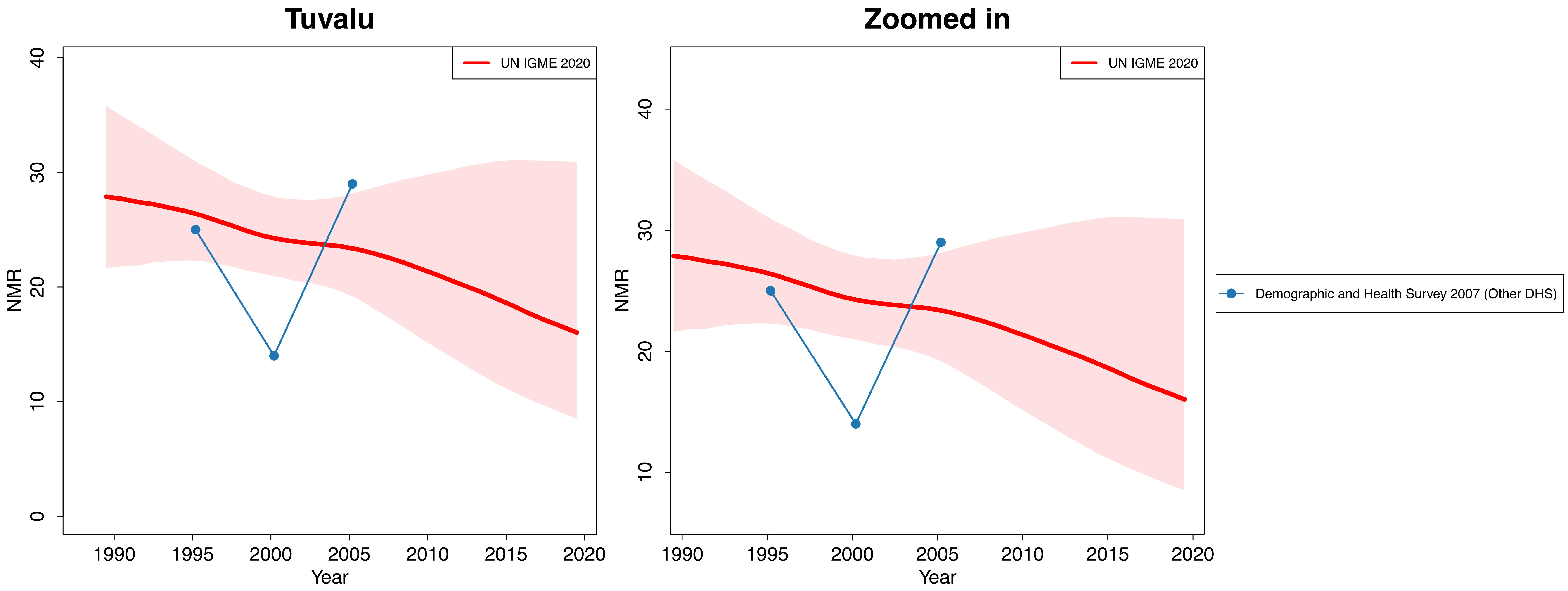
Tuvalu
Zoomed in
UN IGME 2020
UN IGME 2020
Demographic and Health Survey 2007 (Other DHS)
NMR
NMR
Year
Year
0
10
20
30
40
10
20
30
40
1990
1995
2000
2005
2010
2015
2020
1990
1995
2000
2005
2010
2015
2020

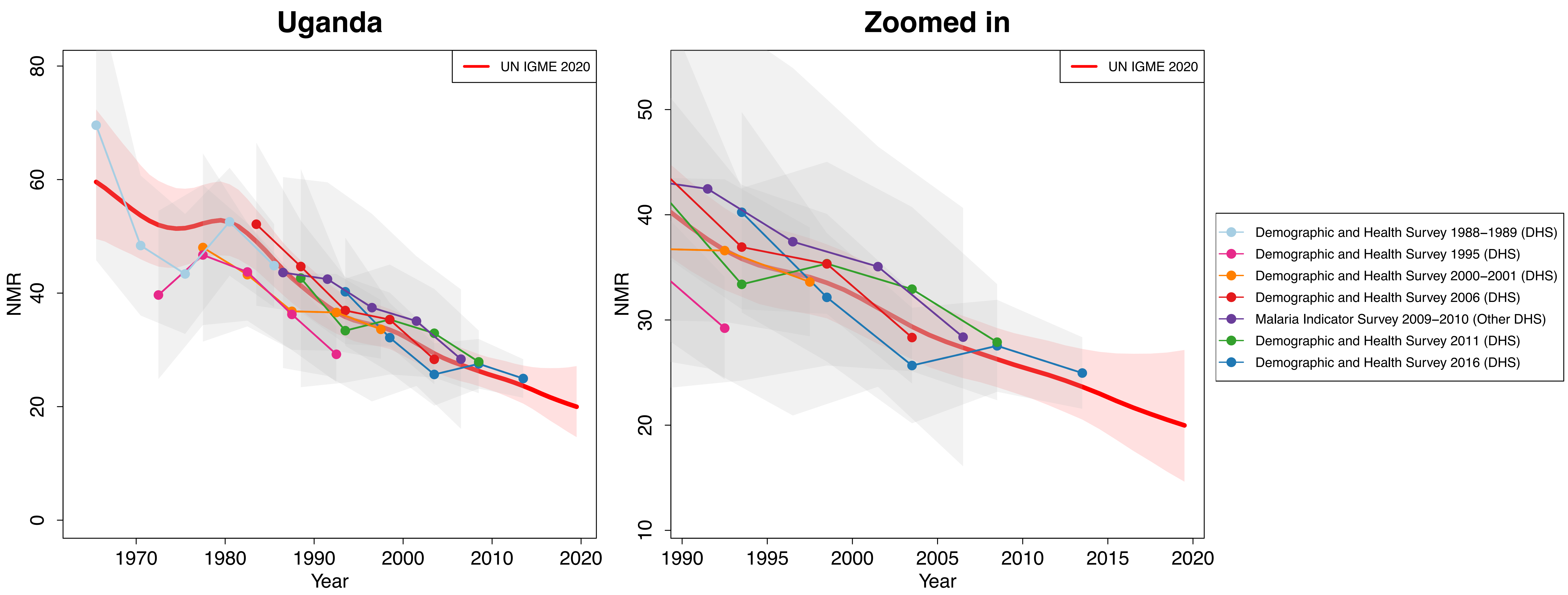

Uganda
Zoomed in
UN IGME 2020
NMR
Year
0
20
40
60
80
1970
1980
1990
2000
2010
2020
10
20
30
40
50
1990
1995
2000
2005
2010
2015
2020
Demographic and Health Survey 1988–1989 (DHS)
Demographic and Health Survey 1995 (DHS)
Demographic and Health Survey 2000–2001 (DHS)
Demographic and Health Survey 2006 (DHS)
Malaria Indicator Survey 2009–2010 (Other DHS)
Demographic and Health Survey 2011 (DHS)
Demographic and Health Survey 2016 (DHS)

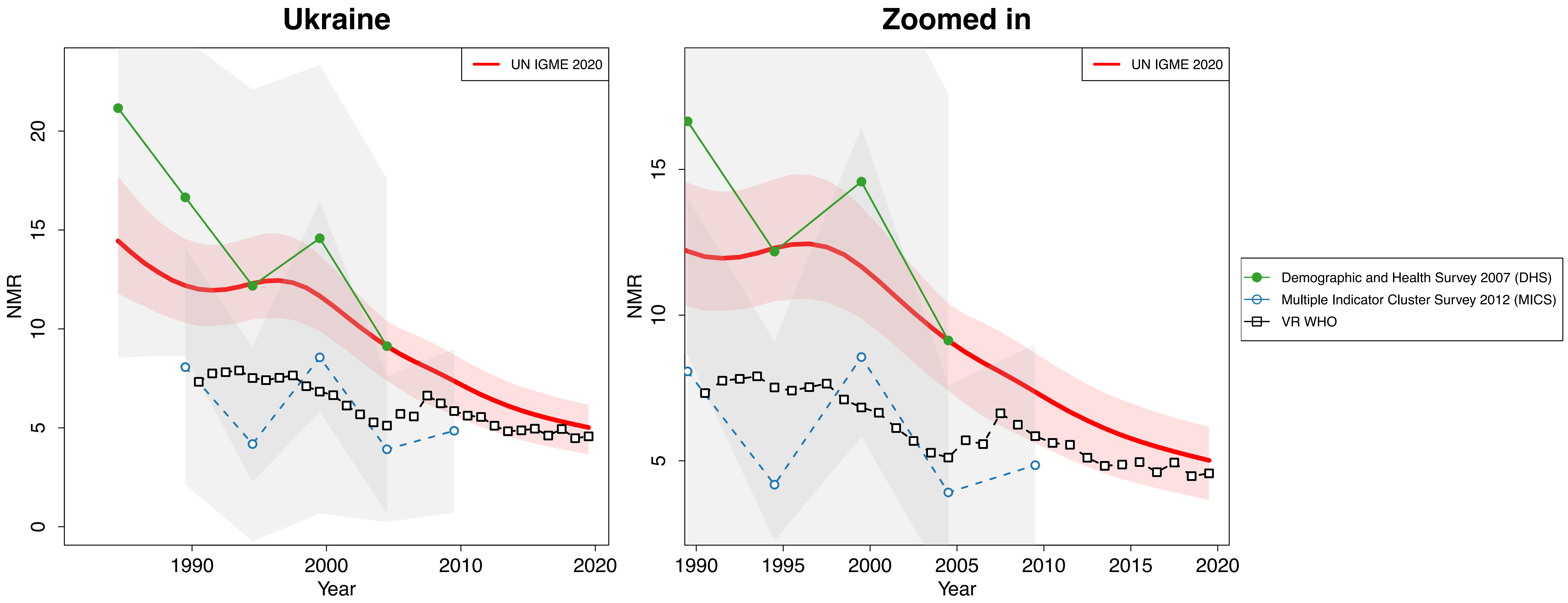

Ukraine
Zoomed in
UN IGME 2020
UN IGME 2020
NMR
NMR
Year
Year
0
5
10
15
20
1990
2000
2010
2020
5
10
15
1990
1995
2000
2005
2010
2015
2020
Demographic and Health Survey 2007 (DHS)
Multiple Indicator Cluster Survey 2012 (MICS)
VR WHO

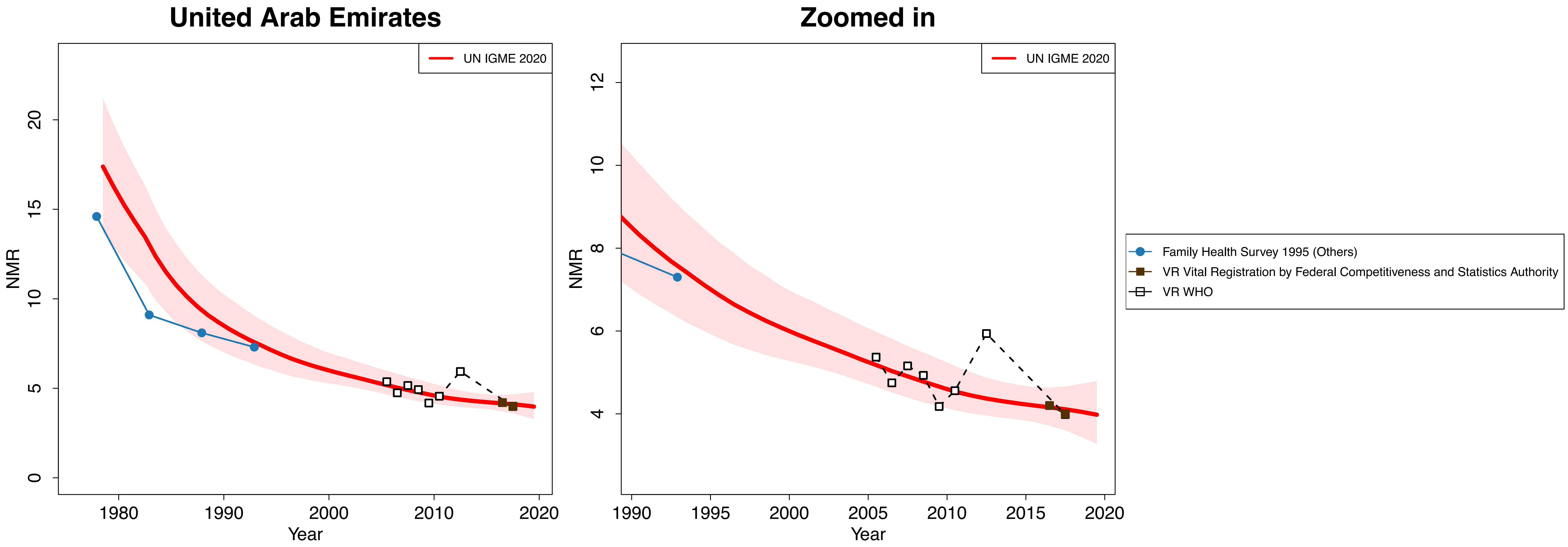

United Arab Emirates
UN IGME 2020
NMR
Year
0
5
10
15
20
1980
1990
2000
2010
2020
Zoomed in
UN IGME 2020
NMR
Year
4
6
8
10
12
1990
1995
2000
2005
2010
2015
2020
Family Health Survey 1995 (Others)
VR Vital Registration by Federal Competitiveness and Statistics Authority
VR WHO

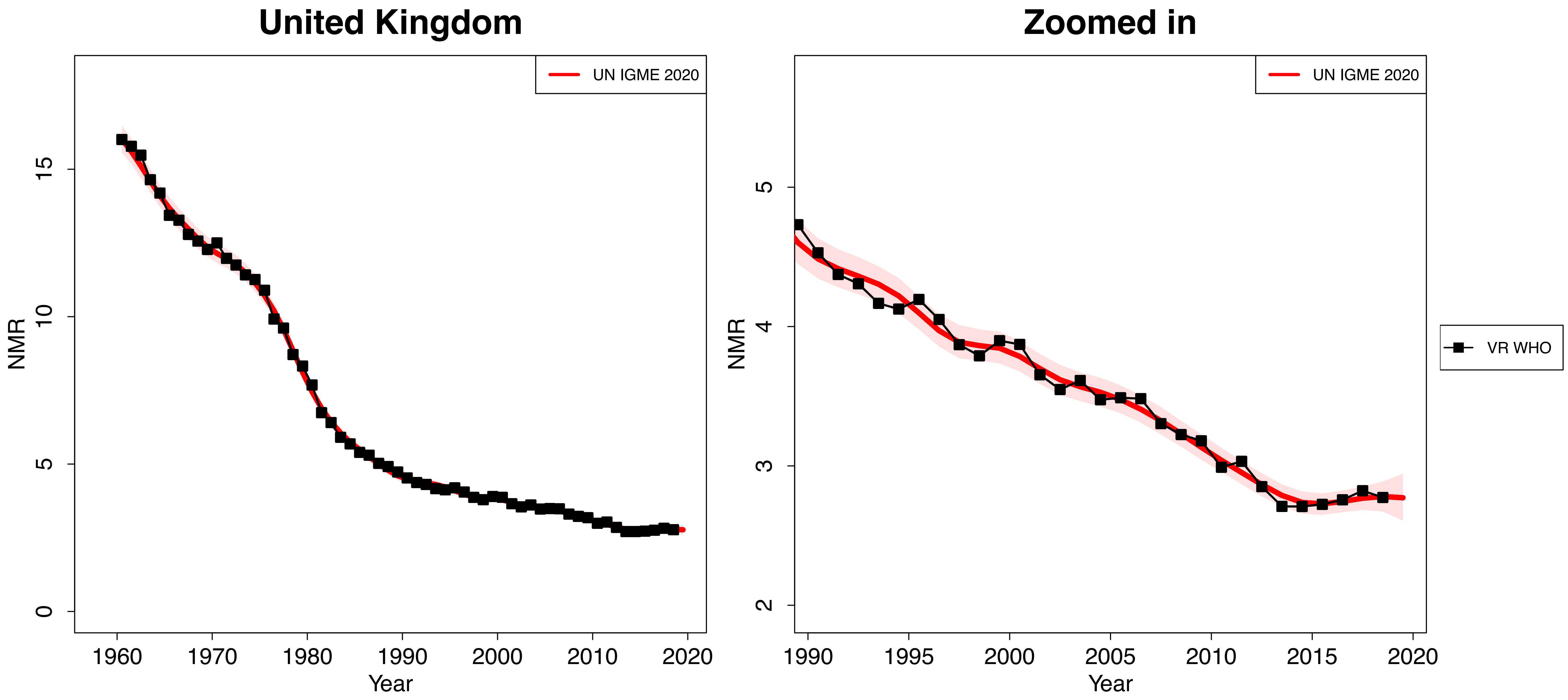

United Kingdom
Zoomed in
UN IGME 2020
UN IGME 2020
VR WHO
NMR
NMR
Year
Year
0
5
10
15
2
3
4
5
1960
1970
1980
1990
2000
2010
2020
1990
1995
2000
2005
2010
2015
2020

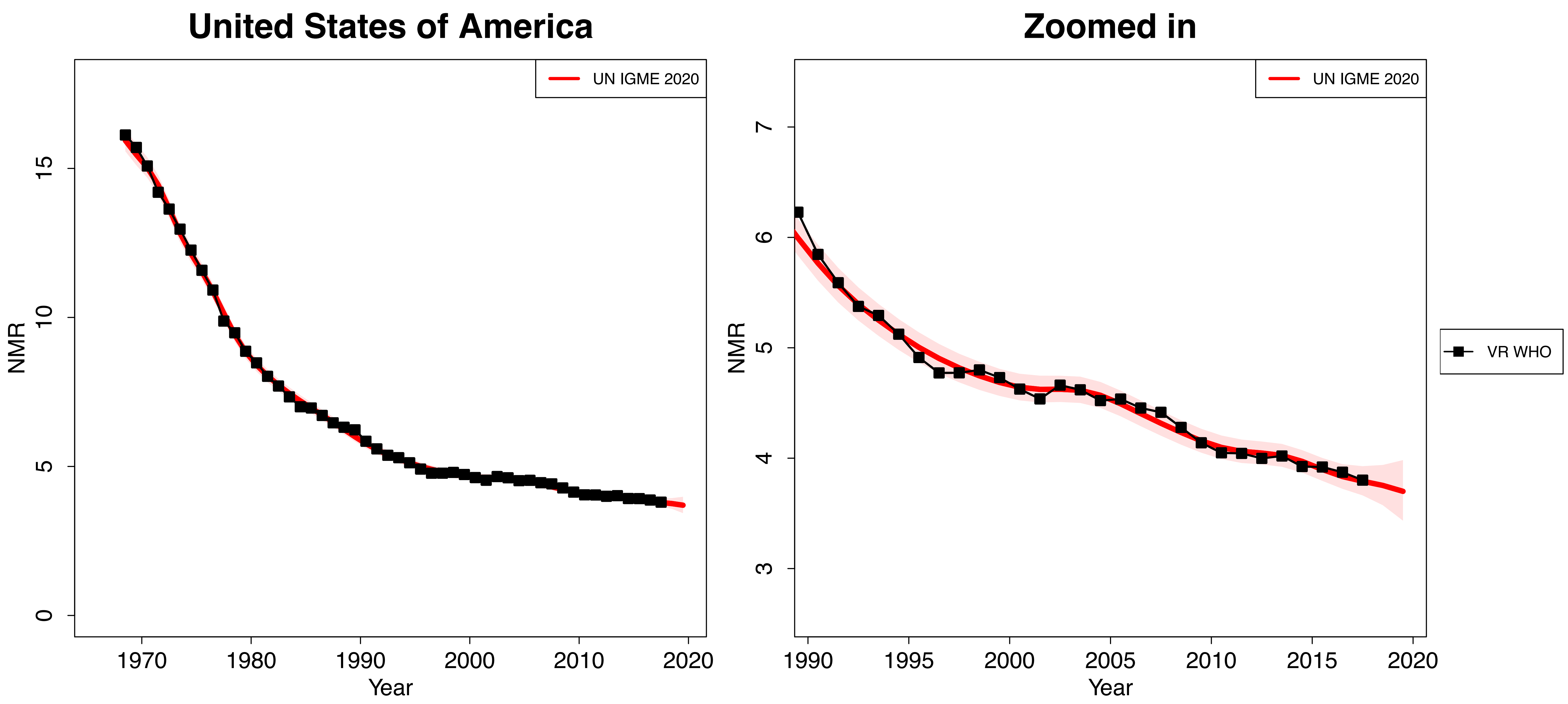

United States of America
Zoomed in
UN IGME 2020
VR WHO
NMR
Year

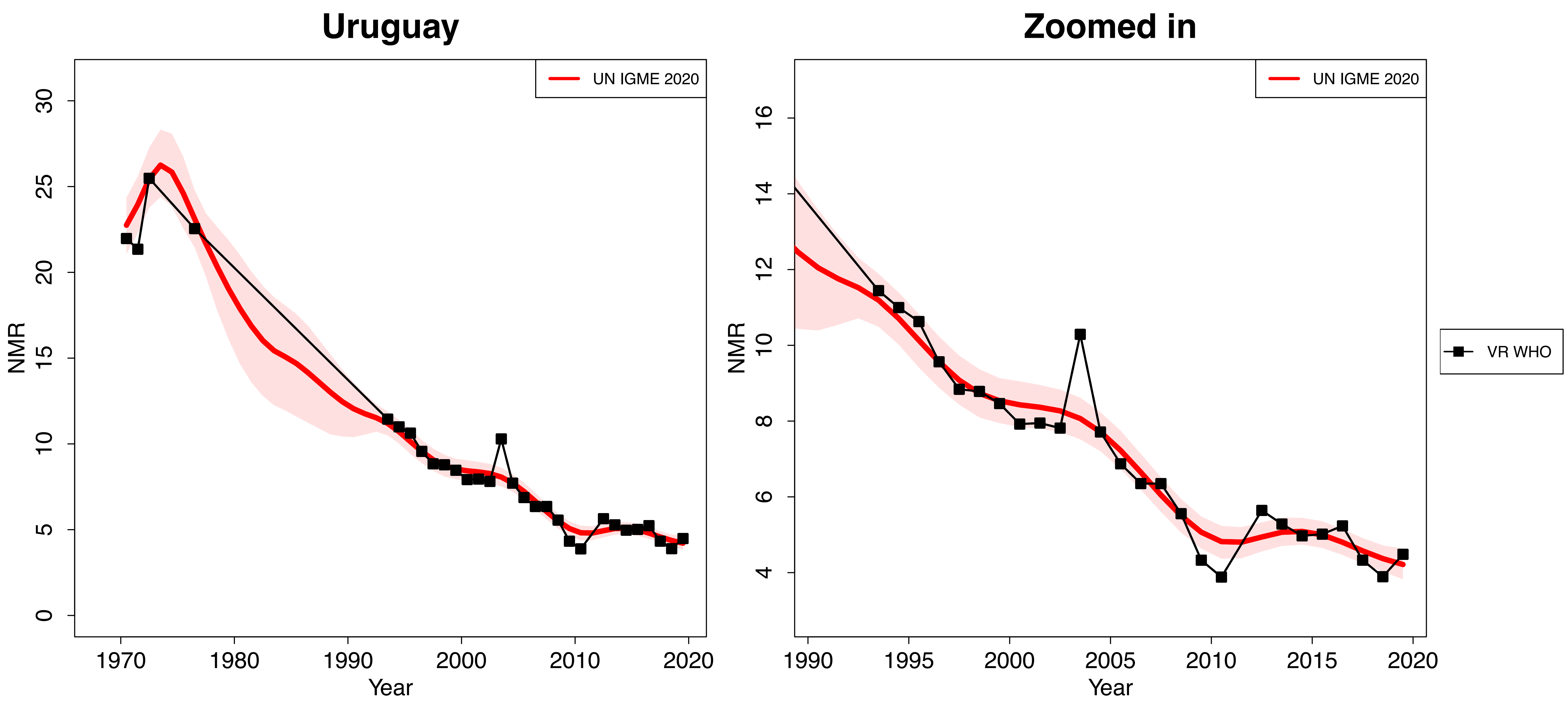
Uruguay
Zoomed in
UN IGME 2020
UN IGME 2020
VR WHO
NMR
NMR
Year
Year
0
5
10
15
20
25
30
1970
1980
1990
2000
2010
2020
4
6
8
10
12
14
16
1990
1995
2000
2005
2010
2015
2020

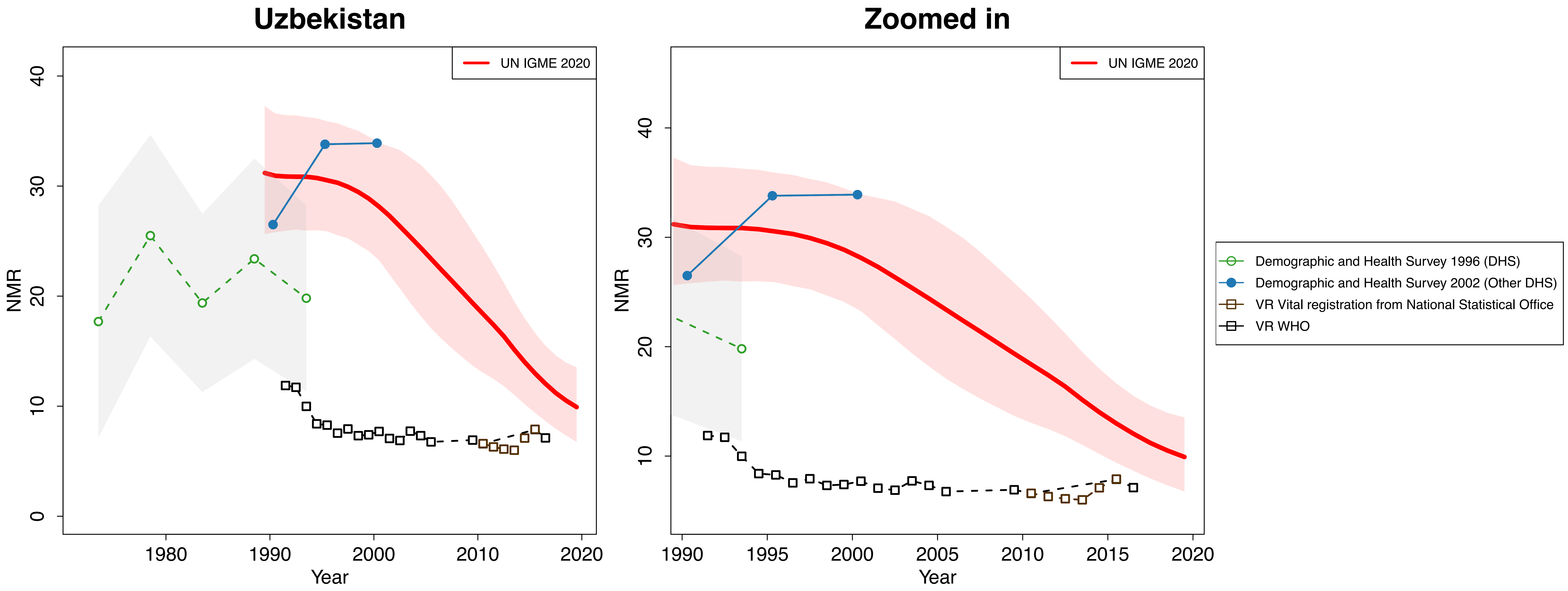
Uzbekistan
Zoomed in
UN IGME 2020
UN IGME 2020
NMR
NMR
Year
Year
0
10
20
30
40
1980
1990
2000
2010
2020
1990
1995
2000
2005
2010
2015
2020
Demographic and Health Survey 1996 (DHS)
Demographic and Health Survey 2002 (Other DHS)
VR Vital registration from National Statistical Office
VR WHO

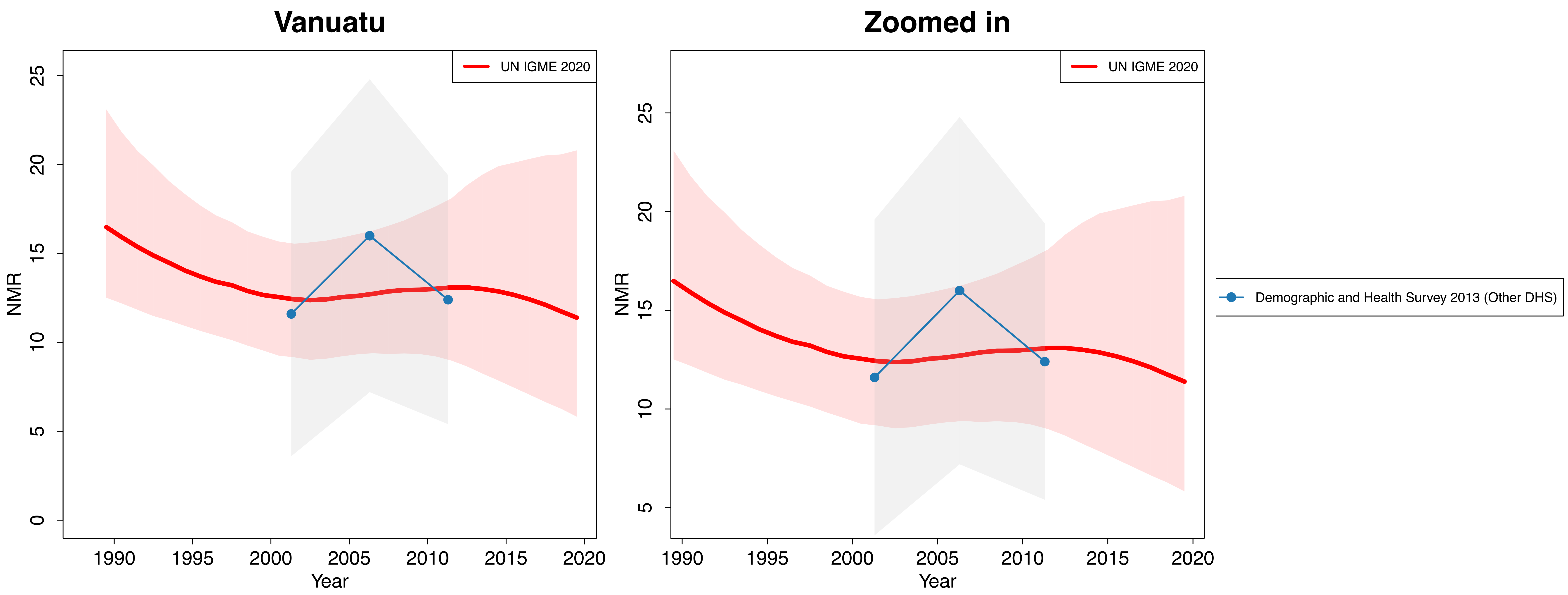

Vanuatu
Zoomed in
UN IGME 2020
UN IGME 2020
NMR
NMR
Year
Year
0
5
10
15
20
25
1990
1995
2000
2005
2010
2015
2020
Demographic and Health Survey 2013 (Other DHS)

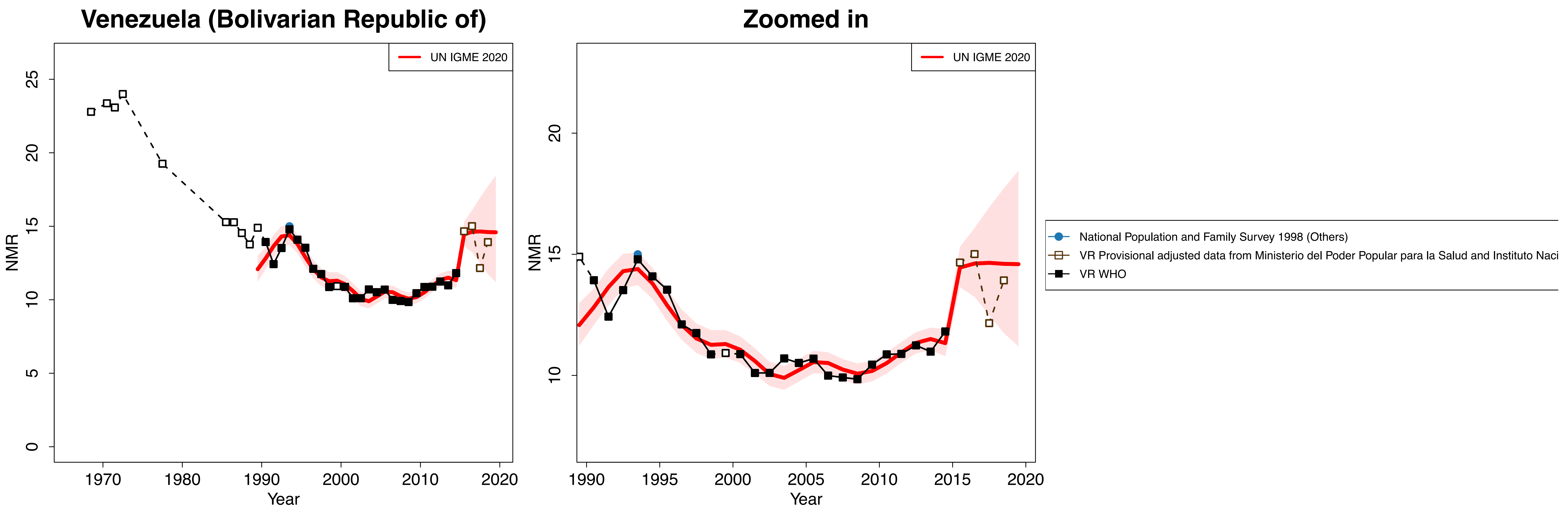

Venezuela (Bolivarian Republic of)
UN IGME 2020
NMR
Year
Zoomed in
UN IGME 2020
NMR
Year
National Population and Family Survey 1998 (Others)
VR Provisional adjusted data from Ministerio del Poder Popular para la Salud and Instituto Naci
VR WHO

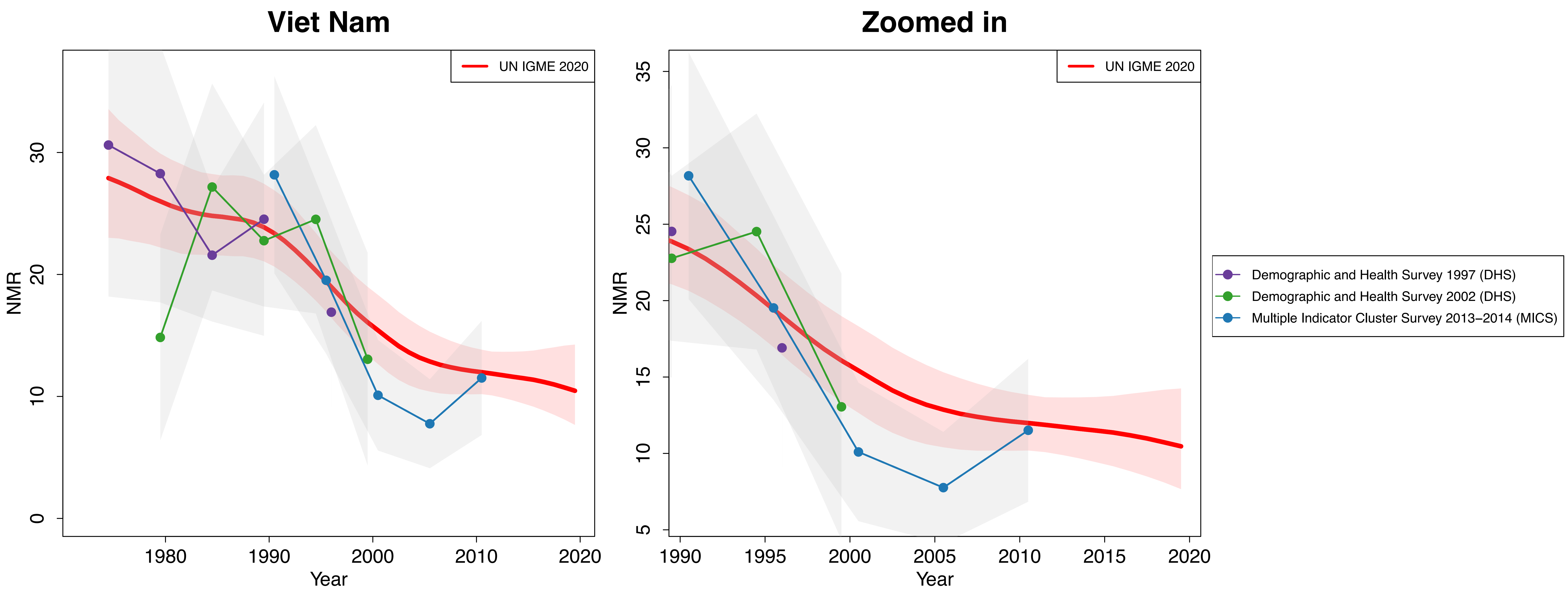
Viet Nam
Zoomed in
UN IGME 2020
NMR
Year
Demographic and Health Survey 1997 (DHS)
Demographic and Health Survey 2002 (DHS)
Multiple Indicator Cluster Survey 2013–2014 (MICS)

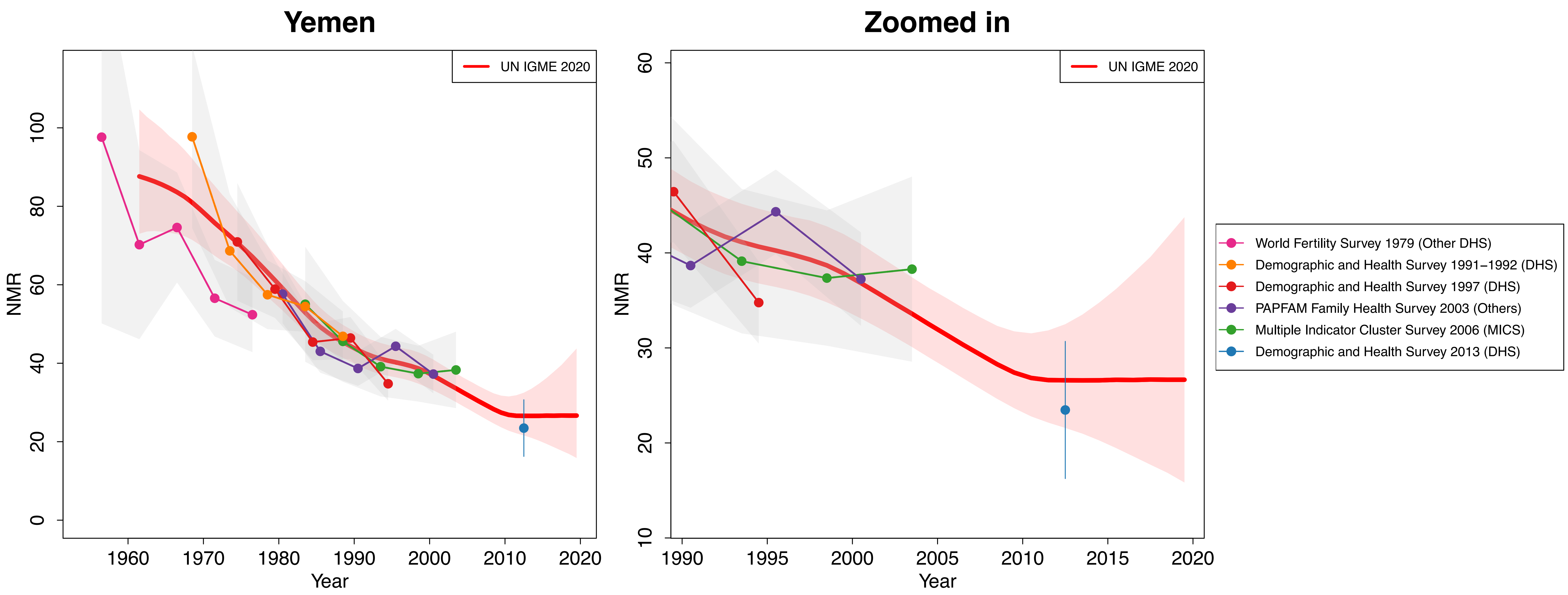

Yemen
Zoomed in
UN IGME 2020
NMR
Year
World Fertility Survey 1979 (Other DHS)
Demographic and Health Survey 1991–1992 (DHS)
Demographic and Health Survey 1997 (DHS)
PAPFAM Family Health Survey 2003 (Others)
Multiple Indicator Cluster Survey 2006 (MICS)
Demographic and Health Survey 2013 (DHS)

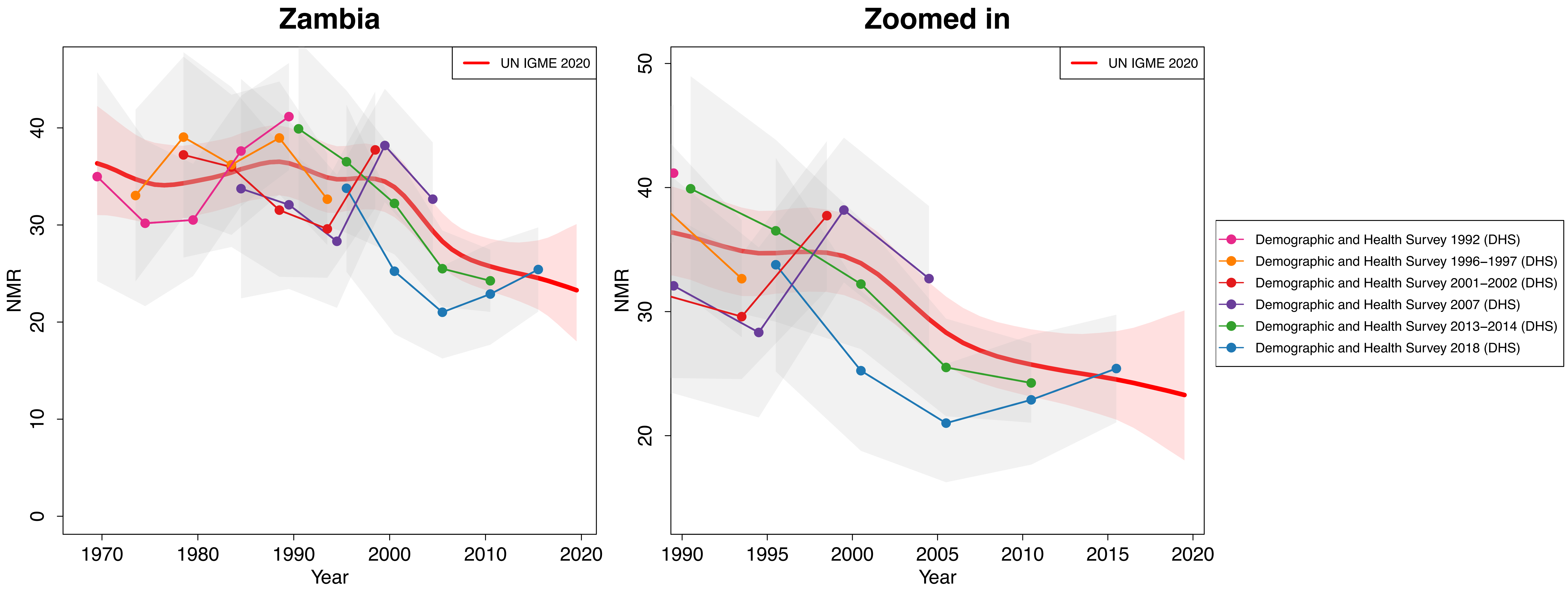

Zambia
Zoomed in
UN IGME 2020
NMR
Year
0
10
20
30
40
50
1970
1980
1990
1995
2000
2005
2010
2015
2020
Demographic and Health Survey 1992 (DHS)
Demographic and Health Survey 1996–1997 (DHS)
Demographic and Health Survey 2001–2002 (DHS)
Demographic and Health Survey 2007 (DHS)
Demographic and Health Survey 2013–2014 (DHS)
Demographic and Health Survey 2018 (DHS)

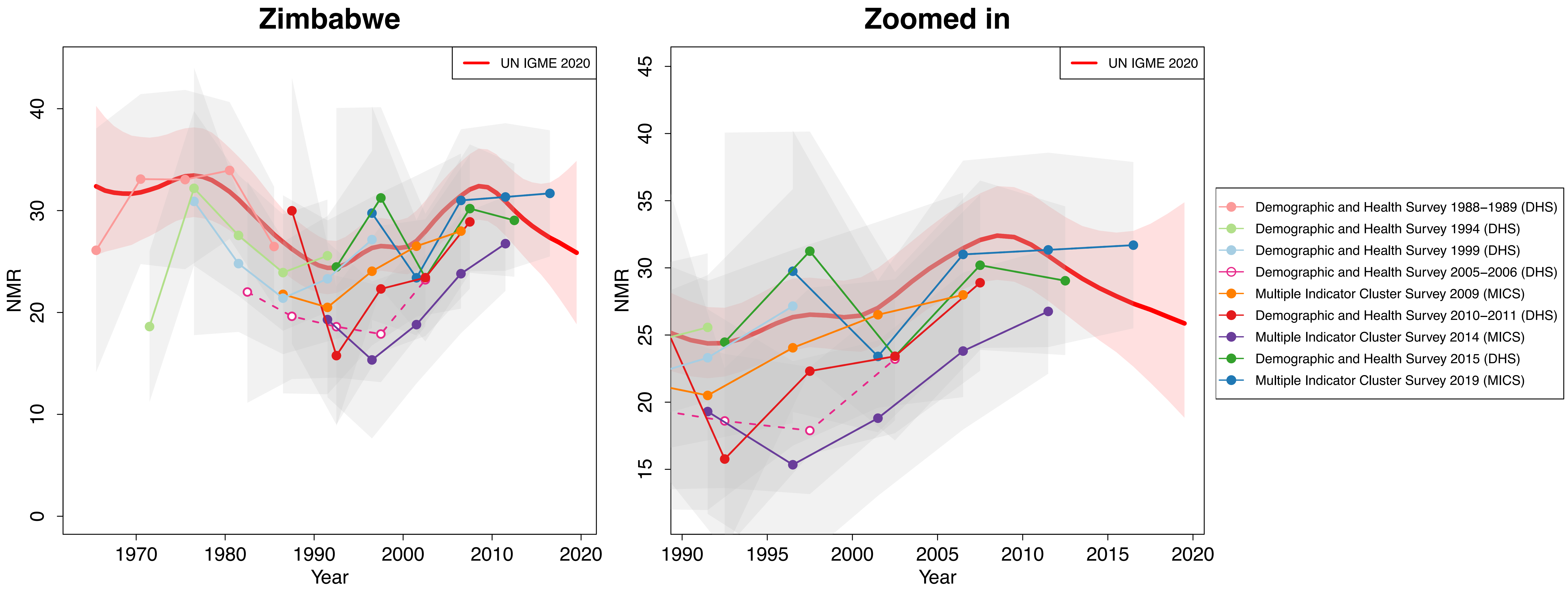
Zimbabwe
Zoomed in
UN IGME 2020
NMR
Year
0
10
20
30
40
1970
1980
1990
2000
2010
2020
15
25
35
45
1995
2005
2015
Demographic and Health Survey 1988–1989 (DHS)
Demographic and Health Survey 1994 (DHS)
Demographic and Health Survey 1999 (DHS)
Demographic and Health Survey 2005–2006 (DHS)
Multiple Indicator Cluster Survey 2009 (MICS)
Demographic and Health Survey 2010–2011 (DHS)
Multiple Indicator Cluster Survey 2014 (MICS)
Demographic and Health Survey 2015 (DHS)
Multiple Indicator Cluster Survey 2019 (MICS)
